# Mining umami peptides in lager and multidimensional sensory evaluation of the beer body integrating computational biology with modern sensomics

**DOI:** 10.1016/j.fochx.2025.103132

**Published:** 2025-10-06

**Authors:** Yashuai Wu, Ruiyang Yin, Zhiyuan Gao, Liyun Guo, Yumei Song, Dongrui Zhao, Jinyuan Sun, Mingquan Huang, Baoguo Sun

**Affiliations:** a School of Food Science and Engineering, South China University of Technology, Guangzhou 510640, China; b Technology Center of Beijing Yanjing Beer Co., Ltd., Beijing, 101300, China; c China Food Flavor and Nutrition Health Innovation Center, Beijing Technology and Business University, Beijing 100048, China; d Key Laboratory of Brewing Molecular Engineering of China Light Industry, Beijing Technology and Business University, Beijing 100048, China; e Beijing Laboratory of Food Quality and Safety, Beijing Technology and Business University, Beijing 100048, China

**Keywords:** Lager beer, umami peptides, T1R1/T1R3, molecular docking, MD simulation, modern sensomics

## Abstract

A systematic study identified umami peptides in lager beer, probed T1R1/T1R3 mechanisms, and quantified multidimensional sensory effects. RPLC–Q-TOF-MS with database search and de novo sequencing identified 1178 peptides; UMPred-FRL and TastePeptides-Meta yielded 142 candidates, of which 128 docked to modeled T1R1/T1R3. Four peptides—EESY, IEVVD, EIVDV, IGVND—were advanced to MD/MM-GBSA and sensory tests. Complexes showed favorable gas-phase interactions offset by polar solvation; binding free energies (kcal/Mol, mean ± SD) were EESY −70.91 ± 7.37, EIVDV −61.26 ± 3.42, IEVVD −60.93 ± 2.33, IGVND −55.24 ± 3.69, indicating EESY had the highest affinity/stability. Single-addition tests increased “umami” by +41.5 % (EESY), +34.0 % (IGVND), +14.9 % (IEVVD), and + 1.1 % (EIVDV). Effects concentrated in taste: EESY and IGVND reduced bitterness and enhanced overall balance; EIVDV maximized balance (+18.11 %) with slight bitterness rise; IEVVD increased bitterness (+17.65 %) yet improved balance. This established an integrated workflow for formulation optimization of lager beer

## Highlights

• Developed an integrated workflow to identify umami peptides in lager beer.
• Screened 128 potential umami peptides.
• Validated four novel umami peptides.
• Proposed a polar-anchoring/hydrophobic-registration mechanism for umami peptides.
• Sensory tests showed umami peptides enhanced umami and overall balance.

## Graphical abstract

**Figure:**
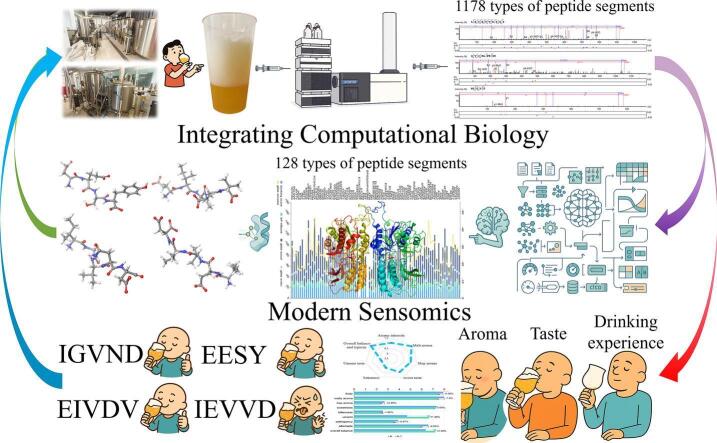

## 1 Introduction

As a key pillar of the global beverage industry, global beer consumption in 2023 was about 187.9 million kiloliters. By region, Asia had ranked first for 16 consecutive years (32.0 %), and China had remained the largest single-country consumer for 21 consecutive years. These structural facts indicated that, in the post-pandemic period, demand recovery had continued steadily in the global beer market with a concurrent concentration of momentum (Kirin《Global Beer Consumption Report 2023》，2024-12-19) (https://www.kirinholdings.com/en/newsroom/release/2024/1219_01.html; https://www.imarcgroup.com/prefeasibility-report-lager-manufacturing-plant). Among beer styles, lager—with its crisp and smooth mouthfeel, balanced bitterness, and rich nutrients, including amino acids, minerals, vitamins, and polyphenols—was favored by consumers worldwide. Within the many segments, lager products had long dominated because of stable flavor and strong industrial adaptability. Although estimates by independent institutions varied, all pointed to its absolute global advantage. For example, Mordor Intelligence estimated that lager's share in 2024 was about 86.46 %, while IWSR reported that lager accounted for more than nine-tenths of global beer (92 %) (https://www.mordorintelligence.com/industry-reports/beer-market). Together these findings confirmed its role as the industry's “baseline—main track.” Against this background, with steady improvement in the global economy and rising disposable income, the growth paradigm of the beer industry had shifted from extensive scale to quality connotation. On the demand side, preferences for streamlined formulations, health-related attributes, and sensory stability had strengthened. The focus of competition had moved from capacity and price to quality dimensions centered on flavor consistency, delicate mouthfeel, and nutritional value. Consequently, the quality advancement of lager beer had become a consensus topic at the industry level.

Within this shift, Lager—defined as lager products produced by “small and independent” brewing entities using low-temperature fermentation and lagering, and emphasizing ingredient character and process profiles—gradually became a vehicle for flavor-driven innovation. According to the Brewers Association (BA), “craft/handmade” beer was characterized by small scale and independence(Carabetta et al., 2024; Martin-Lobera et al., 2025). Meanwhile, the U.S. craft segment experienced a structural slowdown in 2024–2025 amid saturated-market competition and a down-alcohol consumption trend, which underscored the need to advance craft-focused research on the lager track described as “crisp, clean, and repeatable.”

Recent advances in sensory science indicated that beer flavor was highly complex, with formation mechanisms comparable to those of fermented foods such as cheese(Gomez-Ruiz et al., 2007; Yuxiang et al., 2024) and soy sauce(Cai et al., 2024; Ju et al., 2023; Wang et al., 2024; Zhao et al., 2021). In terms of evaluation, beer “flavor” was an integrated phenotype of aroma, mouthfeel, and taste. In addition to sweet, sour, salty, and bitter, umami was an important taste axis that affected roundness, fullness, and persistence of the beer body. The chemical basis of umami mainly arose from free amino acids (e.g., glutamate and aspartate) and 5′-nucleotides formed during fermentation, and produced marked taste-enhancing effects through the cooperative (allosteric) mechanism of the T1R1/T1R3 receptor(Hong et al., 2025; Huang et al., 2025). Studies showed that extended yeast contact or specific aging conditions increased free glutamate levels and improved fullness and aftertaste. These findings provided solid sensory and molecular targets for systematic research on umami in Lager systems(Langdon et al., 2019; Wu et al., 2022; Zhou et al., 2021).

However, with respect to Lager, the open literature remained scarce, and the research focus had long centered on the formation and control of volatile backbones (higher alcohols, esters, sulfides) and hop-derived compounds (polyfunctional thiols). Systematic elucidation of the taste dimension in Lager, especially umami and its molecular basis, remained inadequate. This imbalance—favoring aroma while neglecting taste—was also observed in recent reviews and empirical studies. Many works concentrated on the predictability of complex beer flavor and on hop/yeast biotransformation (e.g., thiol release), whereas studies that targeted Lager and placed umami within the core evaluation and control framework were scarce(Schubert et al., 2022; Vrzal et al., 2023; Yang, Zhang, et al., 2024). Notably, umami peptides opened a new molecular-level window for beer taste research. Studies on beer and other fermented beverages showed that proteolysis generated oligopeptides enriched in acidic residues; these short peptides might directly participate in T1R1/T1R3 recognition and amplification. By 2025, research identified hundreds to thousands of peptides in lager beer using high-resolution mass spectrometry with de novo sequencing, screened dozens of potential umami peptides by machine learning, and then built a sequence–receptor–phenotype line of evidence through molecular docking and sensory verification(Wu et al., 2025). Although this body of work was nascent, it suggested that the peptide–umami–beer-body linkage held within the Lager research context.

It should be noted that the highly complex matrix of Lager beer markedly increased the difficulty of extracting, fractionating, and identifying umami peptides. Traditional identification workflows centered on gel filtration chromatography (GFC) involved cumbersome steps, high time and economic costs, and limited throughput, which in turn impeded progress. To alleviate these bottlenecks, a computer-aided paradigm was introduced by combining machine learning with in vitro/in vivo bioinformatics to enhance screening efficiency and discrimination accuracy for candidate peptides(Cui et al., 2023; Mei et al., 2025; Qi et al., 2022). UMPred-FRL, Umami-MRNN, and TastePeptides-Meta were used for early candidate selection and estimation of umami thresholds, and they demonstrated high effectiveness and stability. For example, the MLP-RNN model constructed by Qi et al.(Qi et al., 2023). was trained on multidimensional sequence features from 499 samples and achieved about 90.5 % accuracy for umami prediction on an independent test set. Likewise, the “taste peptide docking machine” (TPDM) developed by Cui et al.(Cui et al., 2025). integrated residue-interaction data from docking, physicochemical descriptors, and molecular fingerprints, and employed an ensemble classifier to rapidly and accurately distinguish umami peptides from bitter peptides. The resulting “pre-optimization rapid screening” route reflected an overall efficiency advantage of computer-aided analysis over chromatography-centered workflows and provided a more accurate basis for subsequent experimental validation.

Based on the above, the research focused on mining umami peptides in Lager beer and evaluating their multidimensional sensory effects on the beer body. Peptides in Lager beer were identified by reversed-phase liquid chromatography–quadrupole time-of-flight mass spectrometry (RPLC–Q-TOF-MS). Computational screening of umami peptides was then performed with UMPred-FRL, Umami-MRNN, and TPDM/TastePeptides-Meta, and modeled complexes were docked to T1R1/T1R3 to assess binding affinity and key interactions. Sensory-guided analysis quantified thresholds of target peptides. Finally, validation experiments were conducted with a single-addition variable approach, and the multidimensional effects of key umami peptides on the beer body were assessed. The research provided translatable mechanistic evidence for the integrated optimization goal of Lager toward “flavor stability, delicate mouthfeel, and streamlined composition.”

## 2 Materials and methods

### 2.1 Samples and reagents

The experimental sample was a Lager beer (wort concentration 8°P; raw materials: water, malt, wheat, rice, hops). After collection, samples were stored at −4 °C, and sampling time was recorded in 24-h intervals (Craft Beer Workshop Laboratory, Room 108, Building 9, East Campus, Fucheng Road Campus, Beijing Technology and Business University, the detailed information on the craft beer laboratory can be found in Supplementary 6). The main reagents included acetonitrile (ACN) (≥99 %, HPLC grade; Fisher, USA), formic acid (FA) (≥99 %, HPLC grade; Sigma, USA), and ultrapure water. Four umami peptides were used (EESY, IEVVD, EIVDV, IGVND; purity ≥90 % each; Nanjing Taopu Biotechnology Co., Ltd., Nanjing, China).

### 2.2 Experimental methods

#### 2.2.1 Beer sample cleanup and RPLC–QTOF–MS analysis

Four hundred microliters of beer sample were transferred to a 1.5 mL microcentrifuge tube and vortex-mixed for 30 s. The mixture was centrifuged at 17,000*g* for 15 min at 20 °C, and the supernatant was transferred to a new microcentrifuge tube. The supernatant was evaporated to complete dryness in a vacuum centrifuge at 35 °C. The residue was reconstituted in 100 μL of 75 % methanol–water, vortexed for 60 s to dissolve, and then sonicated for 10 min. The mixture was centrifuged again at 17,000*g* for 10 min at 20 °C. After centrifugation, the final supernatant was passed through a 0.22 μm low-protein-binding syringe filter and transferred to an autosampler vial. The extracted samples were subjected to RPLC–QTOF–MS analysis on a UHPLC system (Waters Corporation, Milford, MA, USA) coupled to a high-resolution 5600 QTOF mass spectrometer (SCIEX, Framingham, MA, USA). Ancillary supplies used during sample handling and cleanup included a Milli-Q ultrapure water system (Merck Millipore, Burlington, MA, USA); a 1000 μL adjustable pipette and Class A 10 mL and 100 mL volumetric flasks (Sinopharm Chemical Reagent Co., Ltd., Beijing, China); 2 mL autosampler vials for LC (12 × 32 mm, 9–425 thread; Beijing InnoChem Science & Technology Co., Ltd., Beijing, China); a VM-500S vortex mixer (JOANLAB Equipment, Huzhou, China); a SHB-III water-circulating vacuum pump (Zhengzhou Great Wall Scientific Industrial and Trade Co., Ltd., Zhengzhou, China); and a refrigerated Fresco™ 17 microcentrifuge (Thermo Fisher Scientific, Waltham, MA, USA).

#### 2.2.2 RPLC–Q-TOF-MS analytical conditions

Chromatographic conditions: Peptides were separated on an ACQUITY UPLC HSS T3 column (1.8 μm, 2.1 × 100 mm). Mobile phase A was 0.1 % formic acid in water, and mobile phase B was 0.1 % formic acid in acetonitrile. The injection volume was 4 μL. Separation was performed with a gradient at a constant flow rate of 200 μL/min for 60 min. At 0 min the composition was 95 % A/5 % B and was held for 3 min. From 3 to 5 min, B was increased to 10 %. B was then ramped to 20 % by 34 min and to 40 % by 50 min. From 50 to 56 min, B was increased to 95 % and held for 0.1 min. From 56.1 min to the end, the mobile phase returned to the initial composition (95 % A/5 % B) and was held for 3.9 min.

Mass spectrometric conditions: An AB 5600 Triple TOF mass spectrometer under Analyst TF 1.7 control acquired MS and MS/MS data in information-dependent acquisition (IDA) mode. In each cycle, precursor ions with intensities above 100 cps were selected for MS/MS. The MS^1^ scan range was *m*/*z* 100–1500. MS^2^ used a fixed collision energy of 30 eV, with up to 10 product ion spectra per cycle. ESI source parameters were: GS^1^ 60 psi, GS^2^ 60 psi, CUR 35 psi, temperature 550 °C, and ion spray voltage 5500 V (positive mode).

#### 2.2.3 Qualitative identification of peptides in beer

PEAKS Studio was used for protein identification. The search strategy was as follows. The MS data were searched against a protein sequence database integrating *Triticum aestivum*, Komagataella phaffii, and *Oryza sativa*. The PEAKS de novo sequencing function was enabled to enhance identification. In view of the sample characteristics, the enzyme setting was None, i.e., a nonspecific digestion search. Strict mass tolerances were applied: precursor mass tolerance 10 ppm and fragment mass tolerance 0.03 Da, consistent with high-resolution MS. Dynamic modifications considered included methionine oxidation, protein N-terminal acetylation, pyro-glutamate formation from glutamate or glutamine, deamidation of asparagine/glutamine, and reduction of cystine disulfide bonds(Toda et al., 2013; Wang et al., 2022; Wu et al., 2025).

#### 2.2.4 Efficient screening of potential umami peptides by machine learning

First, UMPred-FRL (http://pmlabstack.pythonanywhere.com/UMPred-FRL) and TastePeptides-Meta (http://tastepeptides-meta.com/TPDM) were prioritized to classify the umami activity of candidate peptide sequences, and the model-predicted umami probabilities were recorded. Next, Umami-MRNN (https://umami-mrnn.herokuapp.com/) was used to estimate the threshold range of umami peptides. Finally, thresholds were verified and determined through standardized sensory experiments and were used as the basis for subsequent single-factor addition experiments.

#### 2.2.5 Molecular docking

Starting from homology modeling, the reference sequence of the metabotropic glutamate receptor mGluR1 (GRM1) was obtained from UniProtKB. The crystal structure of its ligand-binding domain (RCSB PDB: 1EWK) was selected as the template, the amino acid sequences of T1R1 and T1R3 were aligned to it, and three-dimensional homology models were generated on the SWISS-MODEL platform. After model construction, SAVES v6 was used to generate Ramachandran plots to check the consistency of residue φ/ψ dihedrals and stereochemical geometry. Upon passing validation, the docking workflow proceeded. Before docking, the receptor was preprocessed in PyMOL 2.6.0 to remove solvent, inorganic ions, and noncovalently bound small molecules, and a search grid covering the protein surface was defined. Peptide ligands were built in BIOVIA Discovery Studio 2019 with CHARMm force-field parameters, energy-minimized with Smart Minimizer (maximum 2000 steps; RMS gradient threshold 0.01), and potential binding cavities were identified on the same platform. After specifying the binding site, the semi-flexible CDOCKER protocol embedded candidate umami peptides into the T1R1/T1R3 complex; other settings were kept as default, and only the pose with the highest CDOCKER Energy score was retained. Finally, Schrödinger molecular docking software was used to interpret the docking results(Zhang et al., 2025; Zhang, Zhang, et al., 2023).

#### 2.2.6 Determination of umami peptide taste thresholds

All sensory work adhered to international standards. Assessor selection and training followed ISO 8586:2023; sessions were conducted in individual booths designed according to ISO 8589; and general methodology followed ISO 6658. Recognition-threshold procedures used a 3-alternative forced-choice ascending concentration series (method of limits) as specified in ASTM E679, and taste-sensitivity control followed ISO 3972.

To define recognition thresholds for target umami peptides, a taste dilution analysis (TDA) with geometric serial dilutions was used. A stock solution (8 % *v*/v ethanol, pH 6.5) containing each peptide at 1 mg/mL was prepared, then diluted stepwise 1:1 with deionized water to generate a concentration series. Samples, arranged from low to high concentration, were presented to a trained sensory panel (*n* = 20; the same members throughout). At each level, a three-alternative forced-choice (3-AFC) procedure was used; each presentation consisted of one sample and two blanks. The sample differing from the blanks was identified, and the lowest correctly recognized concentration at that level was recorded. The same level was then repeated for verification; agreement in two consecutive trials was taken as the threshold(Lee et al., 2025; Toontom et al., 2024).

#### 2.2.7 Molecular dynamics (MD) and MM/GBSA binding free energy calculations

Docking-derived peptide–receptor complexes served as starting conformations. All-atom MD was performed in AMBER 22. The ff14SB force field was applied to both protein and peptides. Hydrogen atoms were added in LEaP. Systems were solvated in a truncated octahedral TIP3P water box with a minimum solute–boundary distance of 10 Å. Na^+^/Cl^−^ ions were added to neutralize the system, and topology and coordinate files were generated. Energy minimization used 2500 steps of steepest descent followed by 2500 steps of conjugate gradient. Heating was carried out for 200 ps under an NVT ensemble from 0 K to 298.15 K. After temperature stabilization, a further 500 ps NVT equilibration was performed, followed by 500 ps under NPT conditions for pre-equilibration. Production runs of 100 ns were then conducted under periodic boundary conditions. The cutoff for short-range nonbonded interactions was 10 Å. Long-range electrostatics were treated with Particle–Mesh Ewald. All bonds involving hydrogen were constrained with SHAKE. Temperature was controlled by Langevin dynamics (γ = 2 ps^−1^). Pressure was maintained at 1 atm. The time step was 2 fs. Trajectories were saved every 10 ps for subsequent MM/GBSA analysis. Binding free energies were computed within the MM/GBSA framework(Wang et al., 2021; Yang et al., 2021).

To reduce time correlation and conformational drift, frames from the 90–100 ns window were used for statistics. The total free energy was decomposed as follows:

(1) $$\Delta G_{\mathrm{bind}}={\mathrm{\Delta G}}_{\text{complex}}-\left({\mathrm{\Delta G}}_{\text{receptor}}+\cdot {\mathrm{\Delta G}}_{\text{ligand}}\right)={\mathrm{\Delta E}}_{\text{internal}}+{\mathrm{\Delta E}}_{\mathrm{vDW}}+{\mathrm{\Delta E}}_{\text{elec}}+{\mathrm{\Delta G}}_{\mathrm{GB}}+{\mathrm{\Delta G}}_{\mathrm{SA}}$$

In Eq. (1), ΔE_internal_ denoted the internal energy term, and ΔE_vdw_ and ΔE_elec_ corresponded to van der Waals and Coulomb interactions, respectively; the internal term comprised bond (E_bond_), angle (E_angle_), and dihedral (E_torsion_) energies. The solvation free energy consisted of polar and nonpolar components, denoted ΔG_GB_ and ΔG_SA_. The polar term ΔG_GB_ was calculated using the generalized Born model in AMBER (igb = 2). The nonpolar term ΔG_SA_ was obtained as the product of the surface tension coefficient γ and the solvent-accessible surface area SA $\Delta G_{\mathrm{SA}}=\cdot 0.0072\cdot \times \cdot \text{ΔSASA}$ (2). Given the high computational cost and limited accuracy of entropy estimation by normal-mode or quasi-harmonic approaches, −TΔS was not included in the total energy(Chen et al., 2016; Chen et al., 2020).

#### 2.2.8 Sensory evaluation and single-factor addition design

Each sample had a fixed volume of 20 mL. The unmodified base sample was labeled A. After adding a single umami peptide (EESY, IEVVD, EIVDV, or IGVND) to the base, the resulting samples were labeled A-1, A-2, A-3, and A-4, respectively. For each peptide, 500 μL of its taste-threshold solution was added. Sample A was first subjected to sensory evaluation. Table 1 listed nine core sensory dimensions for lager beer and the 0–9 scoring criteria for standardized characterization of aroma, taste, and mouthfeel. Using the same evaluation dimensions and scoring system as for sample A, A-1 to A-4 were then evaluated and compared with the base to determine changes in the beer body after single-peptide addition.

**Table 1:** Sensory dimensions and scoring criteria.

**sensory dimension**	**description**	**score**
fruity	was defined as a fruit-like impression mainly from fermentation esters (e.g., apple/pear/banana), clean and free of solvent-like harshness.	0 = none; 9 = extremely strong
malty aroma	was defined as grain/bread-crust/light-caramel malt notes, clean, without burnt or grassy off-notes.	0 = none; 9 = extremely strong
hop aroma	was defined as herbal, floral, or citrus/resin hop notes, fresh and without oxidative off-odors.	0 = none; 9 = extremely strong
sweetness	was defined as sweetness from residual malt sugars and related components, with a clean finish, not cloying.	0 = none; 9 = extremely sweet
bitterness	was defined as bitterness from hops/isomerized α-acids, required to be clean and non-astringent.	0 = none; 9 = extremely bitter
umami	was defined as a savory, mouth-filling sensation induced by amino acids/peptides, without an MSG-like abruptness.	0 = none; 9 = extremely strong
astringency	was defined as oral dryness/astringency (primarily due to polyphenol–protein interactions), not throat-irritating.	0 = not astringent; 9 = extremely astringent
aftertaste	was defined as the persistence and pleasantness of aroma and taste after swallowing, avoiding sharp or metallic residues.	0 = none; 9 = persistent and rich
overall balance	was defined as the overall balance and harmony among malt, hops, umami, and carbonation.	0 = unbalanced; 9 = highly harmonized

#### 2.2.9 Statistical analysis

Radar and fingerprint plots were generated in OriginPro 2021 (OriginLab Corporation, Northampton, MA, USA). Heat maps were produced with TBtools (developed by Chen et al.; South China Agricultural University, Guangzhou, China). One-way ANOVA and correlation analyses were performed in IBM SPSS Statistics 24.0 (IBM Corp., Armonk, NY, USA). Cluster analysis was conducted in R (R Foundation for Statistical Computing, Vienna, Austria), and results were extracted and visualized within the R environment. All tests were two-tailed with α = 0.05. Normality and homoscedasticity were checked prior to ANOVA; when omnibus F tests were significant, Tukey's HSD was applied for post-hoc comparisons.

## 3 Results and analysis

### 3.1 Qualitative identification of peptides in lager beer and analysis of their potential contribution to umami

In this study, a confidence threshold of −10logP ≥ 15 was set for database-searched peptides; for de novo results, only sequences with ALC ≥ 90 % (Average Local Confidence, ALC denoted the average local confidence across residues in the de novo peptide sequence) were retained to ensure reliability. Under this strategy, 1178 peptides were obtained. UMPred-FRL and TastePeptides-Meta were then used to predict umami properties. The qualitative indices of peptides in the lager sample and the prediction results were shown in Table 2 (due to space limitations, only peptides predicted as potential umami peptides were retained; other peptides were listed in Appendix Table 1).

**Table 2:** Identification of peptides in lager beer and prediction of their umami activity.

**Number**	**Peptide**	**-10LgP**	**ALC (%)**	**Mass**	**m/z**	**RT**	**Class**	**UMPred-FRL-Probability**	**ProUmami**
1	DMVV	16.27		462.21	463.21	14.95	Umami	0.99	0.18
2	EGS	15.77		291.11	292.12	3.53	Umami	0.99	1.00
3	EVSQ	17.14		461.21	462.20	2.19	Umami	0.99	1.00
4	RSF	17.50		408.21	409.22	9.17	Umami	0.99	0.00
5	NDT	19.64		348.13	349.14	7.03	Umami	0.98	1.00
6	EPH	16.21		363.15	364.16	2.31	Umami	0.98	1.00
7	VFST	19.60		452.23	453.23	22.49	Umami	0.98	1.00
8	IEKYSGA	20.92		766.39	767.39	7.09	Umami	0.98	0.77
9	AAVLEY	15.60		664.34	665.38	22.86	Umami	0.98	1.00
10	EVVQ	17.07		473.25	474.25	9.22	Umami	0.97	1.00
11	YTGGNST	15.60		698.29	699.29	4.79	Umami	0.97	1.00
12	AVGF	17.44		392.21	393.21	15.98	Umami	0.97	0.00
13	GNLMTCK	16.21		765.35	765.87	23.58	Umami	0.97	1.00
14	LSAISPL	16.82		699.42	700.41	26.75	Umami	0.97	0.00
15	EKVVNQ	19.63		715.39	716.39	8.78	Umami	0.97	1.00
16	HTY	16.01		419.18	420.19	13.76	Umami	0.97	0.01
17	LEMAGY	16.51		682.30	683.31	3.00	Umami	0.97	0.83
18	EESY	22.97		526.19	527.20	5.35	Umami	0.97	1.00
19	ASGVE	20.96		461.21	462.22	4.50	Umami	0.97	1.00
20	ISVP	19.74		414.25	415.32	17.50	Umami	0.97	0.41
21	PSVP	21.16		398.22	399.22	9.14	Umami	0.97	0.98
22	EV	22.06		246.12	247.07	3.40	Umami	0.97	0.67
23	EYCPQ	15.58		638.24	639.24	3.00	Umami	0.97	1.00
24	VVNSP	23.94		514.28	515.28	9.29	Umami	0.97	0.85
25	ATTSIA	16.07		562.30	563.30	8.27	Umami	0.97	1.00
26	TYAT	16.43		454.21	455.16	6.22	Umami	0.97	1.00
27	TKC	16.14		350.16	351.17	4.93	Umami	0.96	0.66
28	ATAQDIQT	22.46		847.39	848.37	25.42	Umami	0.96	1.00
29	EIVDV	19.73		573.30	574.31	13.67	Umami	0.96	1.00
30	EM	19.13		278.09	279.10	3.44	Umami	0.96	0.95
31	KCT	16.48		350.16	351.17	4.96	Umami	0.96	0.32
32	TMPV	17.70		446.22	447.23	15.43	Umami	0.96	0.00
33	VTGV	20.36		374.22	375.22	4.87	Umami	0.96	1.00
34	TPRHT	20.25		610.32	611.32	19.58	Umami	0.96	1.00
35	VDVIA	19.24		515.30	516.30	15.59	Umami	0.96	0.90
36	KCA	19.71		320.15	321.16	15.28	Umami	0.96	0.57
37	ASQLVE	17.70		687.34	688.35	19.97	Umami	0.96	1.00
38	AGVT	21.65		346.19	347.19	2.64	Umami	0.96	1.00
39	SAGIVNS	22.66		646.33	647.33	8.54	Umami	0.96	1.00
40	VSGEAGNAAAAEERPV	15.19		1526.73	764.36	37.20	Umami	0.96	1.00
41	EVQ	16.23		374.18	375.19	2.89	Umami	0.96	1.00
42	ETLISE	22.28		690.34	691.34	17.28	Umami	0.95	1.00
43	LNFNNRP	18.97		874.43	438.22	15.06	Umami	0.95	1.00
44	GVVT	19.24		374.22	375.22	5.91	Umami	0.95	1.00
45	SAE	20.39		305.12	306.13	8.24	Umami	0.95	1.00
46	TATSLA	17.99		562.30	563.30	8.34	Umami	0.95	1.00
47	IEVVD	18.14		573.30	574.30	13.83	Umami	0.95	1.00
48	GITCT	15.02		493.22	494.23	16.05	Umami	0.95	1.00
49	PTTTY	15.70		581.27	582.28	15.44	Umami	0.95	1.00
50	ATSTLA	19.03		562.30	563.30	8.25	Umami	0.95	1.00
51	VSGID	19.10		489.24	490.25	9.58	Umami	0.95	1.00
52	AQLPSMCRVEPQQCSIF	30.22		1933.88	967.94	40.26	Umami	0.95	1.00
53	SFTEIAKWTSLNT	17.42		1496.75	749.38	37.23	Umami	0.95	1.00
54	TVSGF	18.39		509.25	510.25	13.94	Umami	0.95	1.00
55	PVLSVP	19.43		610.37	611.37	27.21	Umami	0.94	1.00
56	RIC	16.16		390.20	391.18	3.31	Umami	0.94	0.00
57	EVMTSIA	16.15		749.36	750.37	19.29	Umami	0.94	0.97
58	CAFK	17.09		467.22	468.23	22.80	Umami	0.94	0.00
59	DVYVNA	16.99		679.32	680.32	14.65	Umami	0.94	1.00
60	MSSGF	18.06		543.20	544.21	9.81	Umami	0.94	1.00
61	AMY	15.75		383.15	384.15	17.73	Umami	0.94	0.00
62	ARQYAAQLPSMCRVEPQQCSIFAAGQY	20.26		3014.38	1005.81	40.40	Umami	0.94	1.00
63	ESN	17.14		348.13	349.14	5.39	Umami	0.94	1.00
64	FEIST	18.64		595.29	596.29	17.04	Umami	0.94	1.00
65	FSTEY	15.98		645.26	646.27	13.80	Umami	0.94	1.00
66	KST	15.47		334.19	335.19	54.73	Umami	0.94	0.79
67	TVDST	15.00		521.23	522.25	23.55	Umami	0.94	1.00
68	AIDTRGV	16.83		730.40	731.39	8.91	Umami	0.94	1.00
69	AQLPSMCRVEPQQCSIFA	26.58		2004.92	1003.46	40.04	Umami	0.94	1.00
70	SVGI	18.49		374.22	375.22	14.61	Umami	0.94	0.16
71	AAQLPSMCRVEPQQCSIF	18.60		2004.92	1003.46	40.48	Umami	0.94	1.00
72	ESF	19.93		381.15	382.15	8.47	Umami	0.94	1.00
73	RSEQ	15.23		518.24	519.25	9.48	Umami	0.94	1.00
74	RSTT	18.45		463.24	464.25	17.05	Umami	0.93	1.00
75	TSN	17.54		320.13	321.14	8.11	Umami	0.93	1.00
76	KCQGLGNVCF	15.38		1066.48	534.24	9.44	Umami	0.93	1.00
77	EAVT	17.24		418.21	419.21	7.36	Umami	0.93	1.00
78	VDVIANA	24.33		700.38	701.38	16.79	Umami	0.93	1.00
79	AVIE	17.01		430.24	431.25	9.58	Umami	0.93	0.00
80	IHAD	17.73		454.22	455.22	17.29	Umami	0.93	0.03
81	QQCCQQ	33.43		845.28	846.28	7.86	Umami	0.93	1.00
82	RCGP	18.50		431.20	432.20	4.15	Umami	0.93	0.00
83	SSD	20.10		307.10	308.11	10.67	Umami	0.93	1.00
84	GWAVE	17.08		560.26	561.28	9.25	Umami	0.92	1.00
85	IAMM	18.58		464.21	465.22	16.49	Umami	0.92	0.00
86	AFSGCK	15.13		611.27	612.23	6.45	Umami	0.92	0.89
87	IGVND	22.39		516.25	517.26	5.18	Umami	0.92	1.00
88	MSTT	15.97		438.18	439.19	30.41	Umami	0.92	1.00
89	QPVSVPALPQGY	18.17		1255.64	628.83	30.27	Umami	0.92	1.00
90	NTS	21.44		320.13	321.14	6.16	Umami	0.92	1.00
91	RHDGP	15.01		580.27	581.31	19.27	Umami	0.92	1.00
92	QCCQQ	27.84		717.22	718.23	8.07	Umami	0.92	1.00
93	ISVGI	20.08		487.30	488.30	21.28	Umami	0.92	0.63
94	ALGSIVG	16.57		615.36	616.36	13.66	Umami	0.91	0.00
95	AQLPSMCRVEPQQCSIFAA	16.49		2075.96	1039.48	40.43	Umami	0.91	1.00
96	MTNI	17.05		477.23	478.23	11.48	Umami	0.91	0.11
97	VSEHVE	15.10		698.32	699.33	15.99	Umami	0.91	1.00
98	DTRVG	15.17		546.28	547.28	8.24	Umami	0.91	1.00
99	DVYGG	21.94		509.21	510.22	5.75	Umami	0.91	1.00
100	IAEV	19.27		430.24	431.25	13.56	Umami	0.91	0.00
101	AQLPSMCRVEPQQCSIFAAGQY	24.58		2424.10	1213.04	40.98	Umami	0.91	1.00
102	GSVI	20.47		374.22	375.22	10.65	Umami	0.91	0.07
103	SSSQQP	22.63		632.28	634.29	9.60	Umami	0.91	1.00
104	IAGSP	18.55		443.24	444.28	5.61	Umami	0.91	0.01
105	TCR	17.37		378.17	379.18	12.47	Umami	0.91	1.00
106	AAQLPSMCRVEPQQCSIFAAGQY	29.18		2495.14	833.41	41.04	Umami	0.91	1.00
107	AGSPI	15.16		443.24	444.21	10.49	Umami	0.90	0.00
108	TVES	16.03		434.20	435.23	2.84	Umami	0.90	1.00
109	VKLDVLQTL	35.97		1027.63	1028.62	42.64	Umami	0.90	1.00
110	TATGGTSKTAV	16.32		992.51	497.26	8.66	Umami	0.90	1.00
111	RCI	20.60		390.20	391.21	8.08	Umami	0.90	0.00
112	IIVD	18.11		458.27	459.28	14.70	Umami	0.90	0.00
113	PVVP	25.03		410.25	411.26	13.91	Umami	0.90	0.00
114	AEGR		97.70	473.22	474.23	2.20	Umami	0.90	1.00
115	ETSKTQKVVT	15.53		1120.60	561.30	20.12	Umami	0.90	1.00
116	ADNAYY	16.33		715.28	716.28	11.09	Umami	0.90	1.00
117	AAVIQ	17.35		500.30	501.30	11.63	Umami	0.89	0.01
118	AVVG	18.26		344.21	345.21	5.51	Umami	0.89	0.00
119	ALEGATVN	18.41		773.39	774.39	10.07	Umami	0.89	1.00
120	AGE	17.50		275.11	276.12	9.52	Umami	0.89	1.00
121	HISTA	17.56		527.27	528.26	21.94	Umami	0.89	0.01
122	VSSSLVS	22.57		677.36	678.36	14.16	Umami	0.89	1.00
123	AGAIE	15.33		459.23	460.24	4.72	Umami	0.89	0.98
124	KKFGNSRF	15.49		982.53	492.28	24.96	Umami	0.89	0.07
125	FVVP	23.15		460.27	461.27	28.36	Umami	0.89	0.00
126	PRHT	15.57		509.27	510.28	15.61	Umami	0.89	0.02
127	TAY	19.30		353.16	354.16	12.50	Umami	0.89	1.00
128	VVLPSTE	27.55		743.41	744.41	16.83	Umami	0.89	1.00
129	AVAEQAGP	21.26		741.37	742.37	5.23	Umami	0.89	1.00
130	AAGQY	32.48	98.40	508.23	509.23	4.42	Umami	0.89	1.00
131	AIGISVGL	18.33		728.44	729.47	33.38	Umami	0.89	0.99
132	VVGI	26.80		386.25	387.25	17.54	Umami	0.89	0.00
133	ECSEEE	15.88		705.20	706.21	11.24	Umami	0.89	1.00
134	DTAEGAI	15.91		675.31	676.31	9.61	Umami	0.89	1.00
135	IDGI	22.09		416.23	417.23	13.94	Umami	0.88	0.28
136	PDPI	15.18		440.23	441.23	16.91	Umami	0.88	0.40
137	AEVE	16.37		446.20	447.21	4.90	Umami	0.88	1.00
138	FVTP	16.21		462.25	463.25	16.04	Umami	0.88	0.00
139	HVFT	20.60		502.25	504.24	15.02	Umami	0.88	0.01
140	IPVA	16.56		398.25	399.25	16.13	Umami	0.88	0.00
141	IQEQPQ	18.24		741.37	742.37	5.18	Umami	0.88	1.00
142	KGY	19.27		366.19	367.15	6.64	Umami	0.88	0.00
143	VPGD	16.88		386.18	387.19	3.41	Umami	0.88	0.01
144	AISGMH	18.60		614.28	616.36	23.57	Umami	0.88	0.01
145	QNGFVTSAQ	15.11		950.45	951.44	10.32	Umami	0.88	1.00
146	SSKSS	21.18		494.23	495.24	12.41	Umami	0.88	1.00
147	AEP	15.46		315.14	316.15	2.86	Umami	0.88	0.95
148	KCHI	19.04		499.26	500.26	26.46	Umami	0.88	0.00
149	DPGV	16.24		386.18	387.17	11.18	Umami	0.88	0.18
150	GAPNYAHP	24.12		825.38	826.36	14.25	Umami	0.88	1.00
151	ITSTSP	15.83		604.31	605.30	9.27	Umami	0.88	1.00
152	RPDTVSVVD	19.49		1028.51	1029.54	30.37	Umami	0.87	1.00
153	DVYNVA	22.80		679.32	680.32	14.57	Umami	0.87	1.00
154	SEVH	17.17		470.21	471.22	1.22	Umami	0.87	0.14
155	TTCGP	15.42		477.19	478.20	3.07	Umami	0.87	1.00
156	IAR	20.74		358.23	359.24	2.08	Umami	0.87	0.00
157	HCQSIGA	15.01		714.31	715.31	9.61	Umami	0.87	0.55
158	EAG	16.10		275.11	276.12	9.56	Umami	0.86	1.00
159	TSKET	15.34		564.28	565.15	15.68	Umami	0.86	1.00
160	IVATPLL	25.95		725.47	726.47	39.66	Umami	0.86	0.00
161	AAQGCL	15.92		561.26	562.26	13.54	Umami	0.86	1.00
162	SLSSAATG	18.50		692.33	693.29	29.71	Umami	0.86	1.00
163	EPQQQVPVEVMR		94.20	1420.71	711.36	28.23	Umami	0.86	1.00
164	TTR	21.26		418.22	419.22	16.15	Umami	0.86	1.00
165	ALEGATVNF	20.38		920.46	921.46	31.33	Umami	0.86	1.00
166	GGGCCCQ	18.24		626.16	627.16	5.31	Umami	0.86	1.00
167	SSSSG	25.38		423.16	424.17	9.71	Umami	0.86	1.00
168	VVDR		90.20	487.28	488.28	5.63	Umami	0.86	1.00
169	ELSESEWT		95.40	961.40	481.71	16.62	Umami	0.85	1.00
170	KYT	21.18		410.22	411.20	12.56	Umami	0.85	0.29
171	SSITT	15.84		507.25	508.23	14.49	Umami	0.85	1.00
172	ATGDIT	16.34		576.28	577.28	11.26	Umami	0.85	1.00
173	AVGV	16.66		344.21	345.21	5.47	Umami	0.85	0.06
174	GSFKT	15.40		538.28	539.28	16.18	Umami	0.85	1.00
175	AQLPSMCRVEPQQCS	21.60		1673.73	837.87	25.66	Umami	0.85	1.00
176	GGNVACN	17.82		632.25	633.30	11.09	Umami	0.84	1.00
177	EIVQQ	18.59		615.32	616.32	8.49	Umami	0.84	1.00
178	TVTA	17.32		390.21	391.21	7.97	Umami	0.84	1.00
179	EGRASFG	15.07		704.32	705.41	49.49	Umami	0.84	1.00
180	EAIQ	15.76		459.23	460.24	4.37	Umami	0.84	1.00
181	SVTVSLSSTSGP	16.97		1120.56	561.29	27.59	Umami	0.84	1.00
182	VSIS	20.57		404.23	405.20	7.98	Umami	0.84	0.01
183	DYNIQ	20.46		651.29	652.29	10.80	Umami	0.84	1.00
184	SIVP	18.32		414.25	415.25	17.02	Umami	0.84	0.04
185	YDNIQ	16.69		651.29	652.29	10.84	Umami	0.83	1.00
186	DAANYY	15.30		715.28	716.29	11.14	Umami	0.83	1.00
187	GADMI	17.78		505.22	506.22	13.68	Umami	0.83	0.54
188	EAVSI	15.22		517.27	518.25	15.61	Umami	0.83	0.02
189	EF	22.86		294.12	295.13	9.46	Umami	0.83	0.00
190	SPAARNH	19.71		751.37	752.37	15.24	Umami	0.83	1.00
191	RRAEGRAIRM	15.38		1214.68	1215.68	44.24	Umami	0.82	0.55
192	QCCQQLPQIPEQ	18.96		1522.65	762.33	36.03	Umami	0.82	1.00
193	QQCCQQLPQIPEQ	22.27		1650.71	826.36	34.84	Umami	0.82	1.00
194	GGTIVNS	19.53		646.33	647.33	8.59	Umami	0.82	1.00
195	VVDMPGLGSGDIKVQ	23.16		1513.78	757.89	35.46	Umami	0.82	0.79
196	AYSV	15.26		438.21	439.22	7.85	Umami	0.82	1.00
197	ELSESEMR	29.79		961.42	962.42	16.65	Umami	0.81	1.00
198	AIDT	16.70		418.21	419.21	4.85	Umami	0.81	1.00
199	KDVM	15.67		491.24	492.25	9.60	Umami	0.81	1.00
200	VSNSVAGGAQLT	18.35		1102.56	552.28	12.88	Umami	0.81	1.00
201	TVVI	19.26		430.28	431.28	27.78	Umami	0.81	0.93
202	AIVMQQ	24.38		688.36	689.36	12.17	Umami	0.81	0.02
203	LPAGVAHW	17.57		849.45	425.73	27.36	Umami	0.81	0.00
204	FRC	17.70		424.19	425.20	10.09	Umami	0.80	0.00
205	SVADR	17.30		546.28	547.28	8.97	Umami	0.80	1.00
206	EWGYT	16.36		654.27	655.27	20.62	Umami	0.80	1.00
207	EGVP	19.01		400.20	401.20	8.19	Umami	0.80	0.96
208	QCCQQLPQIPE	17.95		1394.60	698.30	38.98	Umami	0.80	1.00
209	DAIP	23.06		414.21	415.16	12.85	Umami	0.80	0.70
210	ADEF	16.65		480.19	481.19	10.67	Umami	0.80	1.00
211	NFSP	19.33		463.21	464.21	8.57	Umami	0.80	0.00
212	GMGELHLD	15.54		886.39	887.39	19.94	Umami	0.80	0.93
213	EFAT	18.36		466.21	467.21	11.21	Umami	0.80	1.00
214	TTFTT	23.99		569.27	570.28	3.50	Umami	0.79	1.00
215	AGAAVGGQVVEK	30.31		1084.59	543.30	9.31	Umami	0.79	1.00
216	AGEQAFH	29.93		800.35	801.35	11.93	Umami	0.79	0.13
217	QCCQQLP	16.72		927.36	928.35	26.77	Umami	0.79	1.00
218	RTRGGQ		90.30	715.37	716.38	8.73	Umami	0.79	1.00
219	LNIYSTNETEIDDHR	15.21		1818.84	910.42	33.97	Umami	0.79	1.00
220	DLTRGV	20.27		659.36	660.36	8.19	Umami	0.79	1.00
221	ISPM	19.59		446.22	447.22	15.59	Umami	0.79	0.00
222	GRCFG	17.99		538.23	539.24	4.95	Umami	0.79	0.00
223	VSVVD	21.80		517.27	518.28	9.99	Umami	0.79	1.00
224	CRPP	15.32		471.23	472.23	7.26	Umami	0.78	0.00
225	AATLQ		91.30	544.29	545.29	11.22	Umami	0.78	1.00
226	SIGQAGE	22.97		660.31	661.29	12.25	Umami	0.78	1.00
227	IDEI	16.03		488.25	489.25	15.61	Umami	0.78	1.00
228	HICR	15.53		527.26	528.26	8.19	Umami	0.78	0.00
229	VTVI	21.25		430.28	431.28	27.75	Umami	0.77	0.96
230	VAVAVSAPTR	22.71		969.56	485.78	11.82	Umami	0.77	1.00
231	VGADI	15.44		473.25	474.25	13.56	Umami	0.77	1.00
232	TFT	15.92		367.17	368.18	8.54	Umami	0.77	1.00
233	DIVT	16.75		446.24	447.24	23.24	Umami	0.77	1.00
234	FGTT	17.32		424.20	425.20	9.22	Umami	0.77	1.00
235	QDATNVGDEGGFAPNIQ	21.46		1714.74	858.38	32.12	Umami	0.76	1.00
236	IVTGGDSGIGRA	21.82		1101.58	551.79	12.24	Umami	0.76	1.00
237	PDYT	17.57		494.20	495.21	9.82	Umami	0.76	1.00
238	EPEY	15.35		536.21	537.22	4.38	Umami	0.76	0.69
239	SQNDQRGEIIHV	16.73		1394.69	698.35	16.83	Umami	0.76	1.00
240	ADI	17.27		317.16	318.16	3.06	Umami	0.76	0.00
241	QAVE	15.80		445.22	446.23	2.45	Umami	0.76	1.00
242	EFET	18.59		524.21	525.27	6.82	Umami	0.75	1.00
243	VIGS	24.20		374.22	375.22	5.01	Umami	0.75	0.00
244	VPTVDVS	37.21		715.38	716.38	15.28	Umami	0.75	1.00
245	RSSS	18.80		435.21	436.21	11.39	Umami	0.75	1.00
246	SYQDVYN	25.11		887.37	888.37	11.59	Umami	0.75	1.00
247	VDVSVVD	36.35		731.37	732.37	17.66	Umami	0.75	1.00
248	GVRNPEEIPWGEAGAD	16.45		1695.79	848.89	30.66	Umami	0.75	1.00
249	MGV	16.17		305.14	306.14	9.15	Umami	0.75	0.00
250	MAMKCMPMPLE		90.50	1311.53	656.77	38.19	Umami	0.75	1.00
251	ELSESEM	20.49		805.32	806.32	22.78	Umami	0.75	1.00
252	VGGI	24.87		344.21	345.21	10.71	Umami	0.75	0.00
253	HHY	19.96		455.19	456.20	6.88	Umami	0.74	0.00
254	VTTI	20.12		432.26	433.26	11.15	Umami	0.74	1.00
255	YHH	24.30		455.19	456.20	6.94	Umami	0.74	0.00
256	RQHSP	15.54		623.31	624.30	19.18	Umami	0.74	1.00
257	EAGVGAG	15.34		541.25	542.24	5.17	Umami	0.74	1.00
258	STIA	17.83		390.21	391.22	6.00	Umami	0.74	1.00
259	SKVH	16.42		469.26	470.27	1.00	Umami	0.74	0.36
260	VAGNEGNLA	20.75		843.41	844.41	9.44	Umami	0.73	1.00
261	ERFQPM	19.15		806.37	807.38	14.05	Umami	0.73	0.33
262	PMVATN	15.91		632.28	633.30	12.02	Umami	0.73	0.90
263	FKAS	16.80		451.24	453.22	15.51	Umami	0.73	0.00
264	RDR	18.68		445.24	447.24	10.62	Umami	0.73	1.00
265	VRNPEEIPWGEAGAD	34.95		1638.76	820.38	29.87	Umami	0.73	1.00
266	KFHT	23.81		531.28	532.29	16.61	Umami	0.73	0.06
267	LQEQLQ	21.01		757.40	758.40	10.38	Umami	0.73	1.00
268	LDVEPQQ	18.28		869.41	870.42	5.61	Umami	0.72	1.00
269	RSHG	15.23		455.22	456.28	20.28	Umami	0.72	1.00
270	TSIA	24.80		390.21	391.21	6.15	Umami	0.72	1.00
271	SYEESMPMPLE	45.45		1311.54	656.77	38.15	Umami	0.72	1.00
272	ASNVGPD	15.15		658.29	659.29	5.28	Umami	0.71	1.00
273	ACISCAEELERK	15.30		1348.61	450.54	14.01	Umami	0.71	1.00
274	IDGGI	18.31		473.25	474.25	12.60	Umami	0.71	0.06
275	TTNDSFTF	17.62		931.39	932.40	22.26	Umami	0.71	1.00
276	QPQQPQ	28.36		707.32	708.33	6.57	Umami	0.71	1.00
277	AIAGSIGIG	23.09		757.43	758.43	27.00	Umami	0.70	0.55
278	DVGIT	15.38		503.26	504.27	10.80	Umami	0.70	1.00
279	IDQLGGVE	15.06		829.42	830.42	14.94	Umami	0.70	1.00
280	THH	21.55		393.18	394.18	2.11	Umami	0.70	0.97
281	ELRGGSWVVVDP	16.45		1312.68	657.34	30.19	Umami	0.70	1.00
282	STPSPTS	15.60		675.31	676.28	13.20	Umami	0.70	1.00
283	LVADMP		90.00	644.32	645.33	19.89	Umami	0.70	0.99
284	HGAV	16.13		382.20	383.14	3.02	Umami	0.70	0.49
285	ISVGP	18.81		471.27	472.28	8.89	Umami	0.70	0.70
286	ATAAGVGV	16.25		644.35	645.35	14.83	Umami	0.70	1.00
287	LVLPGELAK	27.57		938.58	939.58	31.08	Umami	0.70	0.83
288	ATTI	15.18		446.24	447.24	16.15	Umami	0.69	1.00
289	DAVT	16.27		404.19	405.20	8.21	Umami	0.69	1.00
290	AFR	19.10		392.22	393.22	6.33	Umami	0.69	0.00
291	MAPNYTA	16.39		766.33	767.34	25.08	Umami	0.69	1.00
292	ESVPE	15.49		559.25	560.25	11.39	Umami	0.69	1.00
293	EPHQ		90.10	491.21	492.22	2.44	Umami	0.69	1.00
294	CRF	16.11		424.19	425.19	10.12	Umami	0.69	0.00
295	HSSEESLSS	17.89		961.40	481.71	16.66	Umami	0.68	1.00
296	EPKP	17.69		469.25	470.26	15.45	Umami	0.68	0.87
297	MSLSDPE	15.33		777.32	778.34	20.15	Umami	0.68	1.00
298	FHH	18.20		439.20	440.21	9.68	Umami	0.67	0.00
299	PACR	16.06		445.21	446.22	4.98	Umami	0.67	0.00
300	LASGALGLGGLSSL		91.00	1214.69	1215.68	44.29	Umami	0.67	1.00
301	NFPAGGS	17.80		648.29	649.29	9.13	Umami	0.67	1.00
302	EIA	21.09		331.17	332.18	6.91	Umami	0.67	0.00
303	RTPTVG	15.34		629.35	630.35	4.95	Umami	0.67	1.00
304	AAQ	16.83		288.14	289.15	5.89	Umami	0.66	0.93
305	DIRH	16.08		539.28	540.29	24.89	Umami	0.66	0.01
306	EQAQLP	15.36		684.34	685.35	11.03	Umami	0.66	1.00
307	HHVQ	16.38		519.26	520.27	14.97	Umami	0.66	0.90
308	VGSQP	23.82		486.24	487.24	4.32	Umami	0.66	0.97
309	QPLQPQP	16.59		789.40	790.40	19.30	Umami	0.66	0.04
310	GRSH	19.96		455.22	456.19	3.29	Umami	0.66	1.00
311	DTTY	17.77		498.20	499.21	3.23	Umami	0.66	1.00
312	EVCCAGS	22.83		648.21	649.22	1.78	Umami	0.65	1.00
313	PNDQPP	23.54		666.30	667.30	12.87	Umami	0.65	1.00
314	AHF	18.57		415.19	416.19	13.63	Umami	0.65	0.00
315	VHLHNQ	15.29		746.38	747.39	15.58	Umami	0.64	0.93
316	VDPSGSY	22.91		723.31	724.32	8.53	Umami	0.64	1.00
317	EGDVIVAPAGSIMHL	24.74		1507.77	755.38	42.29	Umami	0.64	0.98
318	HHAP	15.82		502.23	503.23	2.39	Umami	0.64	0.00
319	VVDI	19.91		444.26	445.26	17.21	Umami	0.64	0.22
320	IRV	18.90		386.26	387.27	27.77	Umami	0.64	0.00
321	HVW	16.29		440.22	441.22	17.39	Umami	0.63	0.00
322	DAKDAN	24.27		632.28	633.28	27.62	Umami	0.63	1.00
323	VVDMPGLGSGDIK	34.23		1286.65	644.79	30.24	Umami	0.63	0.98
324	CGKHA	24.37		514.23	515.24	12.62	Umami	0.63	1.00
325	IVAP	22.50		398.25	399.23	9.61	Umami	0.63	0.00
326	PEF	20.63		391.17	392.18	4.06	Umami	0.63	0.00
327	VSSI	20.48		404.23	405.23	8.06	Umami	0.63	0.07
328	PEAQ	16.90		443.20	444.21	9.50	Umami	0.62	0.95
329	INQVD	15.40		587.29	588.27	9.96	Umami	0.62	1.00
330	YAHPH	17.64		623.28	624.29	16.17	Umami	0.62	0.03
331	VGGP	23.36		328.17	329.18	5.39	Umami	0.62	0.00
332	QPQQPQQPQ	21.83		1079.49	1080.03	31.89	Umami	0.62	1.00
333	QAIP	21.78		427.24	428.25	11.68	Umami	0.62	0.00
334	PMKR	18.74		530.30	531.31	9.17	Umami	0.62	0.00
335	YQVQ	16.86		536.26	537.27	7.48	Umami	0.62	1.00
336	IDLLGGA	25.17		657.37	658.37	26.93	Umami	0.61	0.00
337	SVDP	15.45		416.19	417.20	4.88	Umami	0.61	0.22
338	DVTPVPTDSTR	15.90		1186.58	594.29	10.79	Umami	0.61	1.00
339	MVHGQPQ	16.71		795.37	796.38	32.82	Umami	0.61	0.86
340	VRALVY	18.49		719.43	720.43	16.53	Umami	0.61	0.02
341	LVKNST	17.53		660.38	661.38	35.31	Umami	0.61	1.00
342	QPQQP	22.54		579.27	580.27	8.21	Umami	0.60	0.89
343	ATQQP	19.21		543.27	544.27	6.86	Umami	0.60	1.00
344	VISLQELC	21.07		902.47	903.47	16.16	Umami	0.60	1.00
345	ICIM	16.12		478.23	479.24	29.67	Umami	0.60	0.00
346	IDKER	18.14		659.36	660.36	8.26	Umami	0.60	1.00
347	AVGDLFE	15.40		749.36	750.36	29.94	Umami	0.60	1.00
348	QQPPQQ	20.62		724.35	725.35	3.38	Umami	0.60	1.00
349	WPDICT	15.29		733.31	735.36	17.27	Umami	0.59	1.00
350	KLEAVG	15.11		615.36	616.36	9.47	Umami	0.59	0.98
351	LCSLYSSQF	19.92		1046.47	1047.49	35.35	Umami	0.59	1.00
352	YMR	15.84		468.22	469.22	8.89	Umami	0.59	0.01
353	AIVTAFVP	21.21		816.47	817.48	40.63	Umami	0.59	0.98
354	PSVM	15.29		432.20	433.23	17.69	Umami	0.59	0.35
355	YCP	17.81		381.14	382.20	6.74	Umami	0.59	0.00
356	ESPF	17.80		478.21	479.22	13.89	Umami	0.58	0.00
357	CCCAGS	15.44		542.13	543.13	1.35	Umami	0.58	1.00
358	HVSH	16.22		478.23	479.23	5.30	Umami	0.58	0.94
359	SHKDWR	22.06		827.40	828.41	14.96	Umami	0.58	1.00
360	EKAP	17.00		443.24	444.21	12.34	Umami	0.58	1.00
361	VVDT	16.67		432.22	433.22	10.78	Umami	0.58	1.00
362	HPHH	18.95		526.24	527.24	2.70	Umami	0.58	0.00
363	IHDA	16.92		454.22	455.22	19.48	Umami	0.57	0.03
364	DVLRCLPVD	20.34		1028.53	515.27	30.46	Umami	0.57	1.00
365	YDAF	24.25		514.21	515.21	17.23	Umami	0.57	1.00
366	ERFQP	17.30		675.33	676.34	9.74	Umami	0.57	0.03
367	QAGHAIQ	16.62		706.34	707.34	8.20	Umami	0.56	0.00
368	AHGV	18.25		382.20	383.20	2.46	Umami	0.56	0.36
369	YVVD	15.28		494.24	495.25	10.21	Umami	0.56	1.00
370	QIQQLQI	18.57		870.48	871.48	30.72	Umami	0.56	1.00
371	SIDT	19.03		434.20	435.21	15.10	Umami	0.56	1.00
372	TV	16.54		218.13	219.11	6.29	Umami	0.56	0.96
373	QPQPQQR	20.45		863.43	864.42	11.07	Umami	0.56	1.00
374	NGV	15.37		288.14	289.15	5.84	Umami	0.56	0.57
375	QQQEQAQAQDQYQQVQ	48.13		1929.85	965.92	12.78	Umami	0.56	1.00
376	IGGE	17.74		374.18	375.22	2.96	Umami	0.56	0.91
377	ETW	26.20		434.18	435.19	12.44	Umami	0.56	0.00
378	PQPQLHPQPQQP	15.48		1394.69	698.35	38.66	Umami	0.55	1.00
379	DVFG	16.34		436.20	437.22	8.67	Umami	0.55	1.00
380	QPQQPQQP	30.12		951.43	952.44	22.99	Umami	0.55	1.00
381	ASVS	16.88		362.18	363.18	3.31	Umami	0.55	1.00
382	SYEESMPMPLEQG	21.83		1496.62	749.30	36.36	Umami	0.55	1.00
383	AITP	18.91		400.23	401.24	9.38	Umami	0.55	0.00
384	IVATP	15.93		499.30	500.30	10.75	Umami	0.55	0.00
385	ADPF	15.69		448.20	449.20	14.94	Umami	0.55	0.17
386	QKRQEQ	16.29		815.43	816.43	16.77	Umami	0.55	1.00
387	QPQQQAQG	15.78		884.40	885.39	28.65	Umami	0.54	1.00
388	NHI	15.79		382.20	383.20	19.29	Umami	0.54	0.00
389	ESW	17.43		420.16	421.17	11.21	Umami	0.54	0.90
390	FSIGS	16.56		509.25	510.24	10.83	Umami	0.54	0.00
391	WAN	21.03		389.17	390.17	10.31	Umami	0.54	0.00
392	ETSW	17.06		521.21	522.22	13.40	Umami	0.54	1.00
393	QAIE	17.59		459.23	460.24	4.85	Umami	0.53	1.00
394	SITP	19.55		416.23	417.23	10.26	Umami	0.53	0.00
395	VTATAVLP	15.96		770.45	771.46	23.64	Umami	0.53	1.00
396	IFVVG	15.20		533.32	534.33	18.59	Umami	0.53	0.00
397	KATPVFL		96.80	816.47	817.47	42.15	Umami	0.53	1.00
398	ANDSNPG	18.26		673.27	675.36	5.72	Umami	0.53	1.00
399	ARTP	15.59		443.25	444.25	2.47	Umami	0.53	0.69
400	FAR	20.16		392.22	393.22	7.37	Umami	0.53	0.00
401	VAATVP	16.77		556.32	557.32	11.42	Umami	0.53	1.00
402	DVVF	16.74		478.24	479.25	30.82	Umami	0.53	0.11
403	NSAGVPH	19.04		680.32	681.32	20.07	Umami	0.52	0.86
404	AAVI	19.13		372.24	373.24	12.89	Umami	0.52	0.00
405	AAYH	19.53		460.21	231.11	13.04	Umami	0.52	0.31
406	KADNASSTVA	15.83		962.47	482.23	8.70	Umami	0.52	1.00
407	TSG	21.44		263.11	264.12	3.53	Umami	0.52	1.00
408	ERH	16.79		440.21	441.21	19.15	Umami	0.52	0.96
409	FPVG	25.77		418.22	419.22	16.08	Umami	0.52	0.00
410	SLQVGGP	19.05		656.35	657.35	5.90	Umami	0.52	0.02
411	SPT	23.16		303.14	304.18	7.14	Umami	0.52	1.00
412	AVVA	16.90		358.22	359.23	6.95	Umami	0.51	0.00
413	PQPQQQQPQPQ	17.92		1304.60	653.31	21.83	Umami	0.51	1.00
414	AAVE	20.66		388.20	389.20	2.65	Umami	0.51	1.00
415	ITGCI	16.93		505.26	506.26	18.46	Umami	0.51	0.00
416	GFDTP	19.32		535.23	536.23	13.65	Umami	0.51	1.00

Notes: −10logP denoted the identification confidence for the peptide based on the spectral match; ALC (%) denoted the average local confidence for the peptide; Mass denoted the molecular mass; m/z denoted the mass-to-charge ratio; RT denoted the retention time in minutes; Class indicated whether it was a potential umami peptide; UMPred-FRL probability and ProUmami indicated the predicted probability of umami.

Among all identified peptides, lengths were dominated by short peptides: the median was 5 aa, with ≤7 aa accounting for 79.0 %. The amino acid composition showed P/Q enrichment (residue frequencies P 12.27 %, Q 9.87 %; ∼22 % combined), followed by G, A, I, V, and S. By property, hydrophobic residues accounted for ∼37.5 %, charged residues ∼18.4 %, and polar uncharged residues ∼44.1 %. At the sequence level, ∼44.7 % contained P and 23.3 % contained Q, and the proportion carrying the dipeptide motif “QP” was 9.3 %. The overall distribution of peptides in this sample was consistent with the general features of major beer source proteins (barley hordein/prolamin), which were rich in Pro/Gln and contained repeat sequences.

Compared with the overall set and the non-umami group, potential umami peptides showed a stronger acidic/hydrophilic bias and a shorter length distribution. The median remained 5 aa, but the proportion ≤ 7 aa reached 82.5 % (non-umami peptides 77.2 %). At the residue level, the proportion of sequences containing acidic residues (E/D) was 48.8 % (non-umami peptides 31.9 %); S/T occurrences were also slightly higher (54.3 % and 51.2 %, respectively). Correspondingly, the enrichment of P and Q decreased markedly (residue frequencies P 7.50 %, Q 8.12 %, both lower than in non-umami peptides). In addition, the proportion containing Cys was 12.5 % in this sample (non-umami peptides 1.7 %), possibly related to repeated parent fragments rich in Cys (e.g., AQLPSMCRVEPQQCSIF and variants). Overall, the higher fraction of charged residues was consistent with the known selectivity and mechanism of T1R1/T1R3, which recognized short peptides bearing acidic and hydrophilic side chains through electrostatic–hydrogen-bond networks.

### 3.2 Screening of potential umami peptides and analysis of their interaction mechanism with the T1R1/T1R3 receptor

**Fig. 1:**
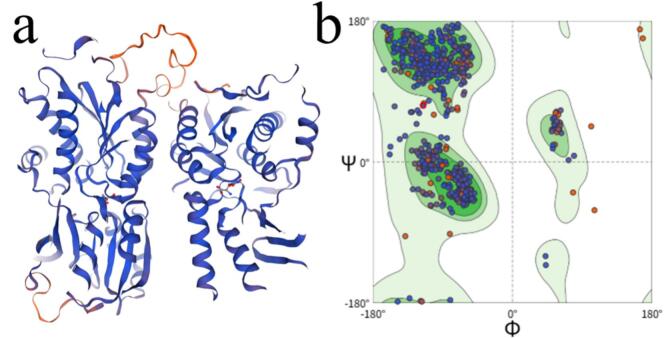
Homology model of the umami receptor T1R1/T1R3 and model evaluation: (a) structural model of the T1R1/T1R3 heterodimer; (b) Ramachandran plot of the model.

Candidate sequences with probability values >0.80 from both UMPred-FRL and ProUmami were selected, yielding 142 short peptides, which were used for molecular docking to the human umami receptor T1R1/T1R3 for subsequent screening. T1R1/T1R3 was a heterodimer of the class I taste receptor family, and its extracellular Venus fly-trap (VFT)–like domain performed the primary functions of ligand capture and fixation. In this study, the dimer was first split into two subunits, homology models were built for each, and stereochemical geometry was examined with Ramachandran plots. As shown in Fig. 1a, T1R1 adopted a closed conformation, whereas T1R3 was relatively open, forming a cavity that accommodated long-chain peptides. Reports indicated that ligand recognition by T1R1/T1R3 involved multiple sites within the VFT(Yan et al., 2025; Zhang, Pu, et al., 2023). Other studies suggested that opening of T1R3 favored peptide binding, and site-directed mutagenesis and computational work showed that T1R1 recognized amino acids and di/tri-peptides; thus, both subunits were indispensable in taste recognition. Homology modeling generally yielded reliable folds when target–template sequence identity was ≥30 %. In this study, sequence identities for T1R1 and T1R3 were 34.34 % and 33.55 %, respectively, meeting this empirical threshold. The subsequent Ramachandran statistics (Fig. 1b) showed that 97.7 % of residues fell within allowed regions, with 87.7 % in the most favored region, 10.0 % in additionally allowed regions, ∼1.8 % in generously allowed regions, and < 0.5 % in disallowed regions. Taken together, the geometric metrics met the requirements and supported docking and mechanism-level analyses.

Docking was performed using the semi-flexible protocol in Schrödinger; other parameters were kept at default, and only the pose with the lowest docking energy was retained. In total, 128 peptides docked successfully (Table 3), including dipeptides, tripeptides, tetrapeptides, pentapeptides, hexapeptides, heptapeptides, octapeptides, nonapeptides, decapeptides, undecapeptides, and dodecapeptides, with counts of 1, 16, 23, 34, 22, 15, 8, 4, 1, 1, and 3, respectively.

**Table 3:** Docking binding energies of potential umami peptides with the T1R1/T1R3 receptor.

Number	Peptide	Docking Score	Hbond (kcal/mol)	Glide Ecoul (kcal/mol)	Glide Evdw (kcal/mol)	Glide Emodel (kcal/mol)
1	AAGQY	−5.31	−2.57	−16.92	−46.81	−84.60
2	AAQGCL	−8.03	−3.24	−17.90	−50.36	−94.53
3	AAQLPSMCRVEPQQCSIF	–	–	–	–	–
4	AAQLPSMCRVEPQQCSIFAAGQY	–	–	–	–	–
5	AAVLEY	−6.96	−2.53	−21.29	−53.29	−90.14
6	ADNAYY	−5.00	−1.69	−22.48	−58.80	−119.07
7	AEGR	−3.92	−4.39	−21.54	−29.49	−66.45
8	AEP	−3.38	−1.76	−12.62	−23.24	−44.99
9	AEVE	−5.15	−1.87	−23.12	−37.17	−65.13
10	AFSGCK	−7.71	−3.35	−29.14	−46.98	−94.13
11	AGAIE	−5.87	−3.82	−19.54	−34.29	−68.45
12	AGE	−3.75	−2.28	−13.95	−25.04	−43.77
13	AGVT	−6.21	−3.14	−22.48	−19.71	−52.24
14	AIDT	−5.24	−2.32	−15.19	−29.00	−52.89
15	AIDTRGV	−7.97	−3.02	−25.56	−52.30	−105.69
16	AIGISVGL	−6.40	−1.60	−19.82	−60.75	−108.63
17	ALEGATVN	−6.51	−2.15	−18.61	−64.21	−104.49
18	ALEGATVNF	−6.57	−2.64	−19.82	−79.62	−145.21
19	AQLPSMCRVEPQQCS	–	–	–	–	–
20	AQLPSMCRVEPQQCSIF	–	–	–	–	–
21	AQLPSMCRVEPQQCSIFA	–	–	–	–	–
22	AQLPSMCRVEPQQCSIFAA	–	–	–	–	–
23	AQLPSMCRVEPQQCSIFAAGQY	–	–	–	–	–
24	ARQYAAQLPSMCRVEPQQCSIFAAGQY	–	–	–	–	–
25	ASGVE	−5.31	−3.26	−21.00	−31.90	−76.02
26	ASQLVE	−7.37	−3.41	−24.34	−52.02	−97.86
27	ATAQDIQT	−4.69	−3.78	−16.00	−68.02	−112.64
28	ATGDIT	−4.27	−3.57	−20.03	−46.82	−83.96
29	ATSTLA	−6.11	−2.71	−16.41	−47.20	−81.42
30	ATTSIA	−7.33	−2.59	−13.49	−49.43	−74.15
31	AVAEQAGP	−8.29	−3.79	−18.23	−57.57	−108.38
32	AYSV	−6.19	−1.86	−9.84	−39.51	−61.30
33	DAANYY	−7.37	−1.93	−16.77	−65.96	−99.63
34	DTAEGAI	−3.57	−1.60	−11.89	−53.80	−79.76
35	DTRVG	−6.56	−3.48	−23.49	−34.03	−70.88
36	DVYGG	−5.77	−1.43	−20.41	−39.02	−75.63
37	DVYNVA	−5.39	−0.89	−9.49	−57.12	−81.90
38	DVYVNA	−7.15	−2.99	−19.61	−49.47	−94.45
39	DYNIQ	−7.50	−3.06	−25.47	−45.42	−103.57
40	EAG	−3.18	−1.95	−17.47	−20.57	−39.58
41	EAIQ	−5.16	−1.98	−12.63	−41.83	−66.12
42	EAVT	−5.95	−2.26	−16.54	−30.78	−56.72
43	ECSEEE	−6.85	−4.29	−28.70	−47.22	−88.42
44	EESY	−5.06	−2.34	−13.99	−33.96	−61.71
45	EGRASFG	−9.46	−5.01	−25.76	−52.28	−106.48
46	EGS	−3.66	−2.13	−14.51	−23.48	−39.24
47	EIVDV	−0.47	−0.67	−13.25	−42.24	−65.63
48	EIVQQ	−5.93	−1.55	−20.51	−56.89	−89.11
49	EKVVNQ	−7.94	−3.95	−26.70	−59.89	−118.37
50	ELSESEMR	−3.01	−3.31	−20.00	−69.77	−95.12
51	ELSESEWT	−7.11	−3.94	−21.60	−73.15	−13.00
52	EM	−3.02	−1.47	−12.15	−24.30	−34.88
53	EPH	−4.05	−1.63	−12.40	−32.71	−52.69
54	EPQQQVPVEVMR	–	–	–	–	–
55	ESF	−5.16	−1.90	−17.08	−29.20	−54.56
56	ESN	−3.83	−3.19	−20.75	−25.60	−54.53
57	ETLISE	−6.67	−1.73	−19.01	−51.13	−89.01
58	ETSKTQKVVT	–	–	–	–	–
59	EVMTSIA	−8.45	−4.60	−28.10	−44.07	−92.12
60	EVQ	−4.41	−1.89	−12.72	−33.55	−56.87
61	EVSQ	−6.81	−3.34	−23.50	−36.52	−79.84
62	EVVQ	−5.55	−2.29	−18.60	−38.21	−68.46
63	EWGYT	−4.77	−1.75	−27.68	−44.35	−107.58
64	EYCPQ	−7.87	−2.78	−18.74	−55.42	−102.62
65	FEIST	−7.54	−2.76	−14.90	−51.63	−76.47
66	FSTEY	−8.47	−3.41	−21.09	−52.36	−112.34
67	GAPNYAHP	−9.73	−5.30	−31.01	−65.49	−151.89
68	GGGCCCQ	−9.15	−3.35	−23.61	−58.00	−113.17
69	GGNVACN	−7.26	−2.71	−20.38	−55.95	−100.55
70	GGTIVNS	−9.49	−4.37	−26.60	−54.77	−105.21
71	GITCT	−7.37	−3.99	−21.32	−32.75	−57.50
72	GNLMTCK	−9.04	−2.46	−26.55	−65.09	−117.52
73	GSFKT	−7.55	−4.71	−23.77	−39.73	−79.35
74	GVVT	−4.44	−3.28	−13.68	−25.24	−45.96
75	GWAVE	−6.58	−2.31	−14.66	−51.34	−86.23
76	IEVVD	−7.42	−2.99	−24.67	−43.28	−81.29
77	IGVND	−7.94	−3.82	−28.99	−33.55	−81.11
78	IQEQPQ	−6.10	−3.81	−26.58	−53.78	−106.56
79	ITSTSP	−6.22	−2.85	−16.77	−49.59	−80.93
80	KCQGLGNVCF	−10.63	−3.91	−31.91	−68.12	−151.00
81	KDVM	−5.79	−2.91	−18.57	−36.10	−71.38
82	LEMAGY	−7.22	−2.38	−18.30	−58.77	−109.35
83	LNFNNRP	−7.81	−3.10	−34.09	−64.49	−131.19
84	MSSGF	−7.08	−2.05	−16.31	−50.39	−89.08
85	MSTT	−4.95	−1.96	−9.94	−39.91	−57.93
86	NDT	−6.03	−4.03	−23.88	−22.48	−59.25
87	NTS	−6.39	−3.25	−20.66	−22.27	−52.68
88	PSVP	−5.30	−3.18	−18.49	−27.26	−54.97
89	PTTTY	−7.24	−2.80	−17.22	−49.47	−85.69
90	PVLSVP	−5.92	−1.91	−13.36	−47.22	−79.14
91	QCCQQ	−6.35	−3.74	−23.02	−44.86	−97.52
92	QCCQQLPQIPEQ	–	–	–	–	–
93	QNGFVTSAQ	−7.83	−2.40	−16.52	−59.43	−6.84
94	QPVSVPALPQGY	−0.58	−1.56	−13.08	−87.65	−9.31
95	QQCCQQ	−9.86	−4.36	−30.10	−62.65	−131.36
96	QQCCQQLPQIPEQ	–	–	–	–	–
97	RHDGP	−6.51	−2.80	−22.87	−38.37	−78.16
98	RPDTVSVVD	−6.51	−3.02	−12.68	−63.90	−78.00
99	RSEQ	−8.89	−4.57	−22.74	−30.25	−74.18
100	RSTT	−8.01	−3.11	−19.53	−30.10	−60.18
101	SAE	−6.38	−2.98	−21.55	−26.69	−59.53
102	SAGIVNS	−10.09	−4.82	−20.18	−54.30	−107.59
103	SFTEIAKWTSLNT	–	–	–	–	–
104	SLSSAATG	−10.23	−4.85	−27.80	−65.38	−128.37
105	SPAARNH	−10.25	−4.43	−29.63	−65.66	−140.01
106	SSD	−6.15	−3.92	−13.65	−23.45	−51.75
107	SSITT	−5.65	−2.74	−22.36	−42.87	−73.23
108	SSKSS	−8.95	−4.98	−21.91	−39.92	−76.67
109	SSSQQP	−8.84	−3.78	−24.88	−49.20	−98.04
110	SSSSG	−5.69	−3.53	−31.38	−23.28	−63.17
111	SVADR	−8.72	−5.01	−26.12	−40.53	−81.73
112	SVTVSLSSTSGP	−3.94	−3.31	−21.63	−71.28	138.88
113	TATGGTSKTAV	−9.16	−5.02	−26.45	−68.59	−47.82
114	TATSLA	−9.34	−4.72	−24.25	−43.17	−97.23
115	TAY	−5.67	−2.89	−17.36	−28.58	−55.38
116	TCR	−6.51	−4.77	−19.02	−17.63	−46.85
117	TPRHT	−8.26	−5.55	−24.46	−40.19	−82.23
118	TSKET	−5.25	−3.89	−24.70	−38.48	−71.14
119	TSN	−5.44	−2.87	−20.45	−24.92	−52.57
120	TTCGP	−5.96	−2.60	−16.60	−39.68	−68.44
121	TTR	−6.57	−4.38	−13.75	−25.01	−47.75
122	TVDST	−6.13	−2.81	−14.32	−39.62	−68.88
123	TVES	−6.79	−4.27	−20.70	−27.05	−57.50
124	TVSGF	−4.94	−2.21	−20.09	−38.32	−73.66
125	TVTA	−3.58	−1.46	−9.92	−31.79	−48.73
126	TVVI	−4.39	−2.08	−14.29	−35.17	−65.75
127	TYAT	−5.13	−2.45	−13.74	−38.91	−76.47
128	VDVIA	0.39	−3.18	−20.77	−33.52	−68.64
129	VDVIANA	−8.90	−1.68	−17.90	−60.00	−96.38
130	VFST	−5.14	−1.37	−14.49	−35.45	−61.09
131	VKLDVLQTL	−10.61	−3.35	−19.98	−78.79	−74.07
132	VSEHVE	−5.27	−1.76	−16.46	−45.90	−78.30
133	VSGEAGNAAAAEERPV	–	–	–	–	–
134	VSGID	−6.57	−3.24	−18.32	−34.08	−73.97
135	VSNSVAGGAQLT	−7.23	−4.16	−21.44	−86.46	−68.45
136	VSSSLVS	−7.68	−4.35	−24.92	−50.19	−108.17
137	VTGV	−2.72	−1.84	−17.21	−32.98	−59.49
138	VVDR	−5.04	−3.00	−24.09	−28.18	−65.88
139	VVLPSTE	−6.30	−2.29	−5.75	−62.60	−95.39
140	VVNSP	−5.01	−2.47	−18.99	−30.72	−64.19
141	YDNIQ	−8.11	−3.72	−27.14	−47.20	−97.26
142	YTGGNST	−10.32	−5.48	−21.58	−52.87	−105.65

Notes: Docking score: the primary metric for ligand ranking and screening; smaller values indicated better scores (same below). Hbond: the hydrogen-bond contribution to the total score. Glide Ecoul: the receptor–ligand Coulomb (electrostatic) interaction energy. Glide Evdw: the receptor–ligand van der Waals interaction energy (from the force-field vdW term). Glide Emodel: used for pose selection and stability comparison (intra-ligand ranking), mainly for choosing among poses of the same ligand.

Potential umami peptides that docked successfully showed a highly consistent “polar–hydrophobic mosaic” pattern at the sequence level. Peptide length was dominated by short peptides (mean 5.52 aa; ≤7 aa accounted for 86.7 %). Acidic residues (E/D) and hydrophilic hydroxy residues (S/T) covered 56.3 % and 66.4 %, respectively, while medium- and short-chain hydrophobic residues (V, A, I, L, G) alternated across many sequences (82.8 % of sequences contained at least one class of hydrophobic residue). N termini were dominated by A and E (∼36.7 % combined), whereas C termini favored polar endings (T/Q/P/E/Y enriched). In addition, ∼10 % of sequences contained Cys (e.g., KCQGLGNVCF, GGGCCCQ, QCCQQ/QQCCQQ), and Pro occurred at a moderate frequency (16.4 %). These features suggested that limited backbone rigidity and kinks facilitated multipoint coordination and spatial complementarity in docking poses. In sum, the amino acid sequence features pointed to a structure–activity logic in which acidic/polar side chains provided anchoring, while hydrophobic residues promoted burial and close fit, matching the geometric and chemical requirements of the extracellular VFT pocket of class I GPCR taste receptors.

The peptides that failed docking generally exhibited greater length (mostly 12–24 aa) and contained multiple cysteine and proline residues. These features introduced conformational heterogeneity and uncertainty in charge/oxidation states (e.g., disulfide formation, thiol oxidation, or multiple microstates under ionization), which reduced success rates when a static receptor with a rigid pocket was assumed. In parallel, the VFT pocket of T1R1/T1R3 had a size/polarity window for ligand volume and distribution of charged groups. Reports indicated that known umami peptides were mainly di- to hexapeptides; when size limits were exceeded or hydrophobic/polar mosaics were mismatched, stable occupancy and favorable scores were seldom obtained. In the absence of synergistic co-ligands (e.g., IMP/GMP), the VFT conformation and hydrogen-bond network could also deviate from the actual binding state. These factors together explained the non-convergent or low-score outcomes observed for sequences such as AAQLPSMCRVEPQQCSIF.

Extensive studies indicated that ligand recognition by the VFT domain of the T1R1/T1R3 heterodimer occurred through open–closed conformational transitions(Tian et al., 2019; Toda et al., 2021). A glutamate-like anionic head group was preferentially anchored to positively charged/polar sites near the hinge region of T1R1-VFT, after which hydrogen-bond and electrostatic networks stabilized the closing tendency. The relative openness of T1R3 provided secondary space that admitted longer or more polar peptides. Mutagenesis and simulation studies showed that the glutamate-binding site resided in the VFT of T1R1 and could act allosterically with 5′-nucleotides(Guo et al., 2024; Wang et al., 2022; Xu et al., 2004). Cross-species chimeras and point mutations further revealed charge and geometric determinants for amino-acid/short-peptide recognition. These mechanisms aligned with the sequence features emphasized in this study—acidic/polar enrichment, short chain length, and limited hydrophobic insertion—namely, side chains such as E/D and S/T supplied dense donor–acceptor sites for directional hydrogen bonds and salt bridges, whereas hydrophobic segments V/I/L/A/G promoted van der Waals burial and conformational restraint of peptides within hydrophobic grooves of the VFT, thereby improved the energetic and geometric stability of docking poses.

EESY, IEVVD, EIVDV, and IGVND were prioritized for subsequent investigations because their primary sequences and side-chain chemistries spanned the acidic, polar, hydrophobic, and aromatic/π categories essential for umami peptides, and because a “same composition, different sequence” control was enabled to test site-specific effects. EESY featured a double glutamate (E–E) as an acidic anchor. Serine (S) could build hydrogen-bond networks, and tyrosine (Y) offered a π surface. This conformed to the empirical enrichment of Asp/Glu in umami peptides and provided the chemical basis for potential hydrogen-bond/electrostatic and cation–π interactions with receptor cationic sites. IEVVD and EIVDV shared the same amino-acid composition but differed in order, enabling a systematic test of the hypothesis that terminal and adjacent residues determined binding orientation and strength. Multiple studies indicated that the binding of short peptides to T1R1/T1R3 was highly sensitive to residues at the N/C termini; different orders could alter hydrogen-bond networks and occupancy of key sites (e.g., Arg151, Asp108), leading to differences in intensity and time–intensity profiles and even to synergistic enhancement with salty taste. IGVND represented an amphipathic pentapeptide model of a “hydrophobic scaffold + flexible spacer + polar/acidic pair.” I/V provided a hydrophobic contact surface, G conferred local flexibility to facilitate induced fit, and N/D formed an amide–carboxylate pair to build dense hydrogen-bond/salt-bridge networks. Such small peptides centered on D/E and N/Q with a moderate proportion of hydrophobic residues had been repeatedly shown to favor higher receptor affinity and robust umami perception. Importantly, the lengths of all four peptides fell within the effective range for umami short peptides. They complemented one another for structure–activity analysis and supported an integrated validation pipeline from molecular docking/MD to sensory measurements. These judgments were supported by multiple sources. Reports that umami peptides were enriched in Asp/Glu and preferred acidic/polar groups agreed with cross-species extraction studies. Effects of terminal residues and sequence order on receptor binding and sensory intensity, together with simulation-derived correlations between binding free energy and umami strength, suggested that the “sequence isomers” IEVVD and EIVDV would yield measurable differences. In addition, aromatic residues could mediate cation–π interactions, and an appropriate hydrophobic fraction helped distinguish umami from bitter peptides, supporting the inclusion of Y in EESY and I/V in IGVND.

Based on the above and the docking binding energies in Table 3, four novel umami peptides—EESY, IEVVD, EIVDV, and IGVND—were selected for subsequent peptide synthesis, taste-threshold determination, MD simulation, and single-addition variable experiments. According to species-database matching, these peptides originated from *Triticum turgidum*, *Saccharomyces cerevisiae*, and barley, consistent with raw materials used in lager brewing. Notably, the four potential umami peptides were newly identified and had no matches in the sensory peptides and amino acids database (https://www.uwm.edu.pl/biochemia/index.php/pl/biopep). Basic information, taste descriptions, and thresholds for the selected peptides were provided in Appendix Table 2. Subsequently, the interaction mechanism with taste receptors was further investigated.

### 3.3 Analysis of binding modes of four umami peptides with the receptor proteins

**Fig. 2:**
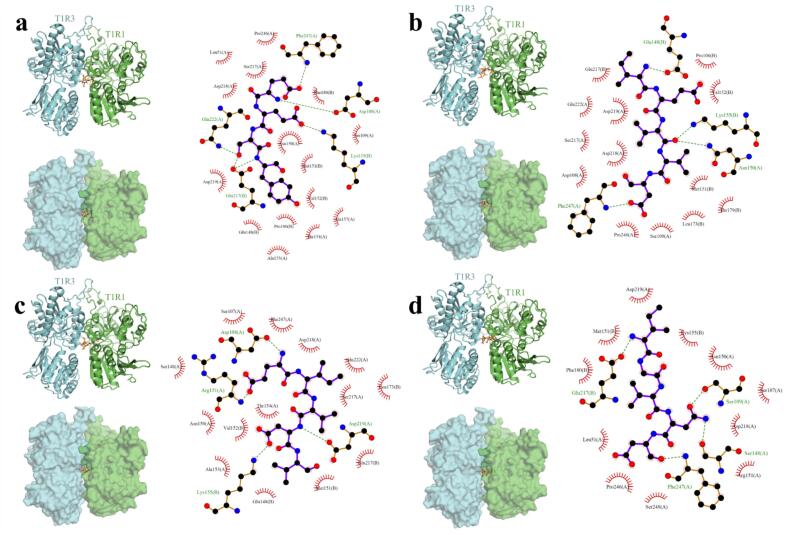
Molecular docking schematic. The left panel showed the overall view; orange sticks denoted the small molecule, green denoted T1R1, and cyan denoted T1R3. The right panel showed the 2D interaction view; dashed lines indicated hydrogen bonds; chain A was T1R1 and chain B was T1R3. (a) Binding mode of T1R1–T1R3/EESY obtained by docking. (b) Binding mode of T1R1–T1R3/IEVVD obtained by docking. (c) Binding mode of T1R1–T1R3/EIVDV obtained by docking. (d) Binding mode of T1R1–T1R3/IGVND obtained by docking.

As shown in Fig. 2a, EESY (orange sticks) was positioned in the narrow cleft formed by the T1R1 (green) and T1R3 (cyan) subunits, spanning the interchain interface (same below). The 2D interaction map indicated that multiple directional hydrogen bonds “locked” the ligand in place, mainly involving Gln222(A), Asp219(A), Glu217(B), Asp108(A), Phe247(A), and Lys155(B); these hydrogen bonds stabilized the ligand backbone in the core of the pocket. A ring of hydrophobic/weakly polar residues around the cavity (e.g., Met151(B), Val152(B), Phe180(B), Glu148(B), Thr154(A), Ala153(A), Ala157(A), Pro106(B), Ser109(A), Ser217(A), Asp218(A), Leu51(A)) formed close contacts with the peptide's hydrophobic side chains, thereby providing van der Waals support beyond the hydrogen-bond network. Overall, EESY attained a stable pose through an “interface bridging + H-bond–hydrophobic cooperation” mode, consistent with the general preference of the T1R1/T1R3-VFT pocket for polar–hydrophobic mosaic motifs.

As shown in Fig. 2b, the peptide was first oriented in the binding cavity by several key hydrogen bonds. Residues such as Glu148(B), Lys155(B), Asn150(A), and Phe247(A) provided multipoint receptor-side coordination and markedly reduced ligand freedom at the active site. On this basis, a hydrophobic “shell” at the pocket periphery—typically Pro106(B), Val152(B), Met151(B), Leu173(B), Thr179(B), and nearby sites including Ser109(A), Pro246(A), Asp218(A), Asp219(A), and Gln222(A)—complemented the ligand's hydrophobic groups to form a continuous vdW support belt. This two-tier stabilization process, “H-bond orientation plus hydrophobic burial,” was consistent with mutagenesis–chimera studies and multi-source computational results on T1R1/T1R3.

As shown in Fig. 2c, the peptide was fixed in the site through multiple anchors. Residues such as Asp108(A), Arg151(A), Asp219(A), and Lys155(B) provided hydrogen-bond/electrostatic pairing, constraining both the ligand backbone and key side chains. A hydrophobic, weakly polar belt formed by Phe247(A), Val152(B), Met151(B), Thr154(A), Ala153(A), Glu148(B), Ser107(A), Ser148(A), Ser217(A), Asp218(A), and Leu173(B) afforded inner-wall complementarity to the ligand side chains. This combination of “multipoint H-bond anchoring plus inner-wall fitting” provided sufficient conformational stabilization without sacrificing flexibility and matched the VFT mechanism for short-peptide recognition, where polar anchors dominated and hydrophobic registration assisted.

As shown in Fig. 2d, IGVND (orange sticks) was inserted into the narrow groove at the T1R1/T1R3 interface, achieving cross-interface insertion. Its hydrogen-bond framework was provided by Glu217(B), Ser109(A), Ser148(A), and Phe247(A), ensuring precise positioning of the ligand in the pocket. Surrounding residues—Met151(B), Phe180(B), Lys155(B), Asp218(A), Asp219(A), Leu51(A), Pro246(A), Ser248(A), Ser107(A), and Arg151(A)—further formed an outer-ring reinforcement through hydrophobic and electrostatic interactions. This mode reflected cooperative recognition in the VFT domain of the T1R1/T1R3 heterodimer, dominated by hydrogen bonding with hydrophobic contributions, and was consistent with prior structure–function studies on short-peptide and amino-acid ligands.

Based on the docking results in Fig. 2, the molecular mechanism of the umami peptide–T1R1/T1R3 complex was summarized as “VFT conformational stabilization driven by polar anchoring and hydrophobic registration.” Specifically, short peptides first formed multipoint directional hydrogen bonds and electrostatic pairs at the interface of the clam-shell VFT cleft through acidic/polar groups (e.g., with positively charged or strongly polar residues near the hinge), which rapidly reduced the ligand's translational and rotational freedom in the site. Subsequently, medium-sized hydrophobic side chains (A/V/I/L/F) achieved van der Waals burial along the pocket groove and complemented hydrophobic patches on the receptor inner wall. These interactions coupled to yield a stable “inner anchoring–outer reinforcement” framework. The multipoint network increased geometric complementarity and energetic advantage at the subunit interface and biased the VFT open/closed equilibrium toward a more closed, activation-favored state of the extracellular domain. This established the structural precondition for downstream signal transduction via the CRD to the seven-transmembrane region. The process was consistent with the class C GPCR paradigm of “VFT recognition–conformational coupling–receptor activation” and agreed with mutagenesis, homology-modeling, and simulation observations on T1R1/T1R3 regarding ligand-driven closure and H-bond–hydrophobic cooperativity. Molecular dynamics simulations were then used to further examine the binding stability of umami peptides with T1R1/T1R3.

### 3.4 Molecular dynamics simulation analysis

#### 3.4.1 Stability analysis

**Fig. 3:**
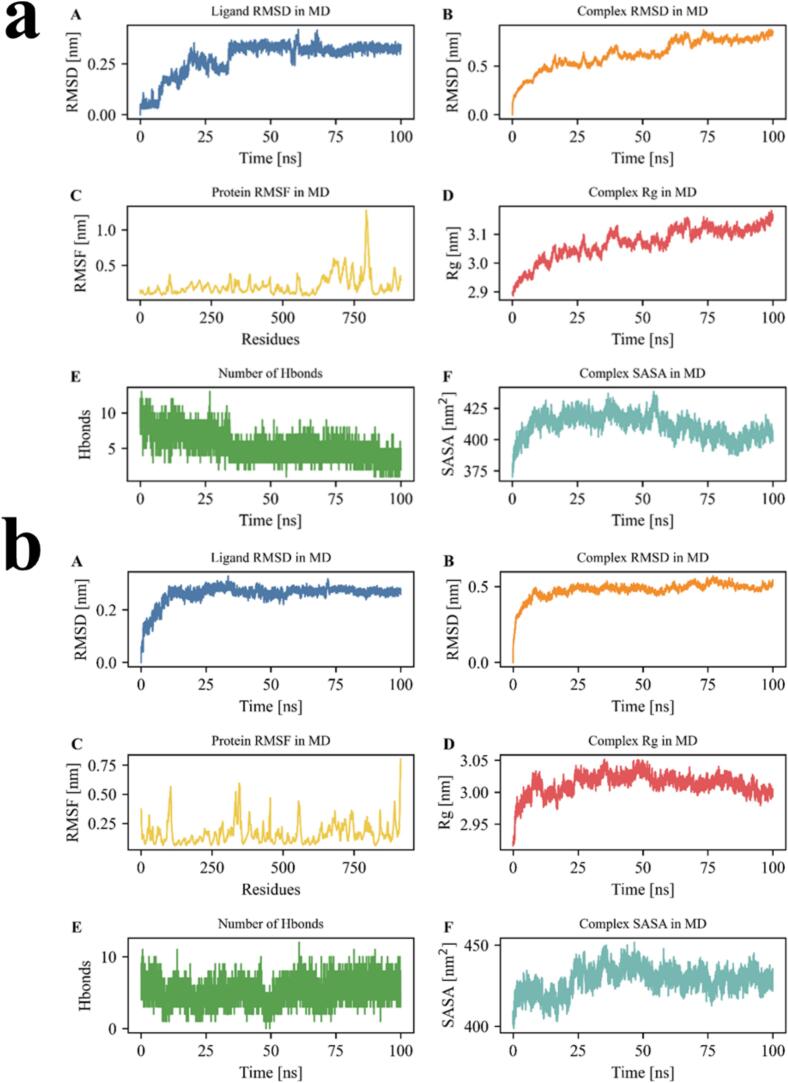

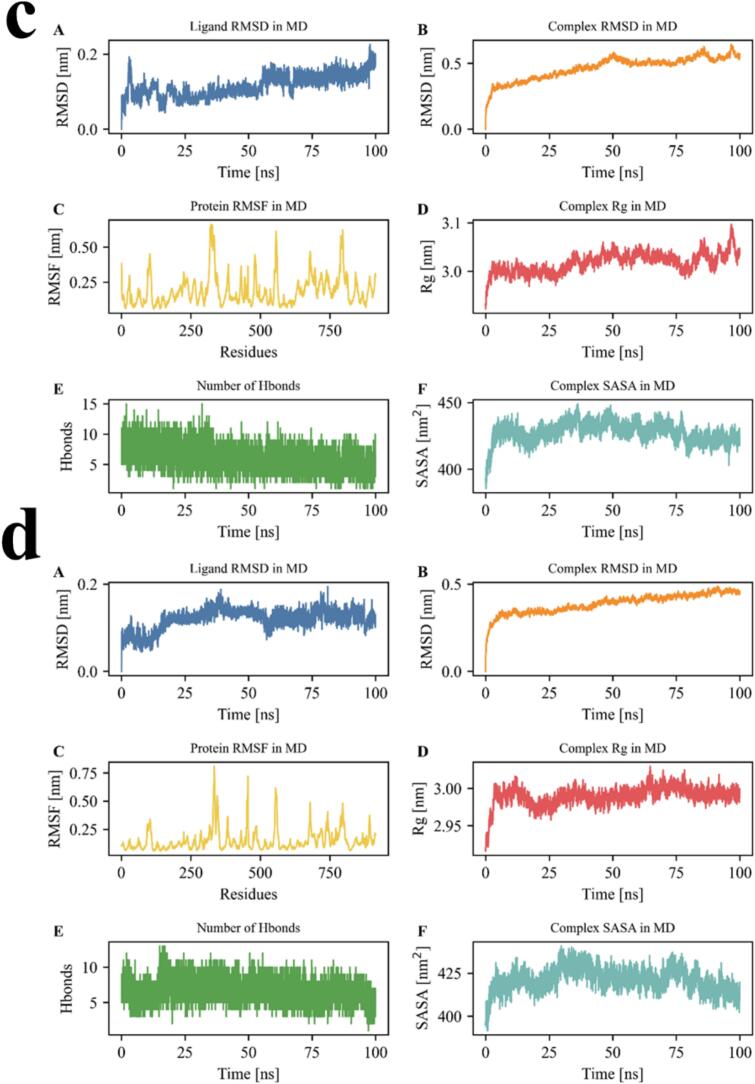
Assessment of binding stability of umami peptides EESY (a), IEVVD (b), EIVDV (c), and IGVND (d) on the T1R1–T1R3 receptor Note: (A) ligand RMSD (Ligand RMSD in MD); (B) complex RMSD (Complex RMSD in MD); (C) protein RMSF (Protein RMSF in MD); (D) complex radius of gyration Rg (Complex Rg in MD); (E) number of H-bonds; (F) complex solvent-accessible surface area SASA (Complex SASA in MD). All plots were time-evolution curves of MD trajectories. RMSD/RMSF were used to characterize structural deviations and residue flexibility; Rg reflected overall compactness; SASA represented solvent exposure; H-bond counts quantified instantaneous ligand–receptor interaction stability. Common units: RMSD, RMSF, and Rg in Å; SASA in Å^2^; H-bonds in counts.

As shown in Fig. 3a, the RMSD of EESY rose rapidly to ∼0.25 nm within the first 20 ns and then stabilized at 0.25–0.30 nm, indicating an initial conformational adaptation followed by a stable binding state with consistent spatial positioning. Although the RMSD value was slightly higher than that of some short peptides, the long-term fluctuation was small, suggesting good conformational stability in the binding pocket. The complex RMSD increased quickly from the start to ∼30 ns, then rose slowly to ∼0.6 nm and reached a plateau, indicating completion of initial conformational adjustments and attainment of a near-equilibrated state. The final plateau showed minor fluctuations without sharp oscillations, indicating overall complex stability and no pronounced protein perturbation upon EESY binding. Protein RMSF values for most residues remained within 0.1–0.3 nm, reflecting good rigidity. A peak appeared only at the C-terminus (∼residue 750), approaching 1.0 nm. This flexible region was likely a non-structured segment or terminal loop and typically did not affect core binding stability. Fluctuations around the binding region were modest, further supporting complex stability. The Rg increased from ∼2.91 nm to 3.13 nm, with a faster rise during 0–50 ns, implying moderate expansion or relaxation after EESY binding. Rg then remained stable, indicating convergence after structural adjustment and completion of binding-induced conformational changes. The number of hydrogen bonds reached 12–13 at the start but showed an overall decline, stabilizing at ∼4–7 after 50 ns. This trend indicated a tight initial interface, followed by partial breakage or rearrangement of hydrogen bonds over time; newly formed hydrogen bonds continued to maintain stable ligand–protein binding. The hydrogen-bond network weakened later but did not disappear, and binding remained effective. SASA increased rapidly from ∼385 nm^2^ to 420–430 nm^2^ and then stayed at a higher level, indicating greater solvent exposure during binding. This trend was consistent with the rise in Rg, suggesting a looser protein structure with more surface area exposed, which could favor water bridges and hydrophobic surfaces in stabilizing the complex. Overall, EESY formed an initially tight complex, underwent adaptive conformational adjustment, and then maintained a relatively stable bound state. Despite increases in Rg and SASA and a reduction in hydrogen bonds, the overall RMSD was steady, the ligand conformation remained stable, and the protein backbone showed no obvious disturbance, supporting EESY as a candidate peptide with good binding capability and adaptive flexibility(Liu et al., 2023; Mei et al., 2025; Yang, Xu, & Xiang, 2024).

The RMSD of IEVVD rose rapidly to ∼0.25 nm within the first 10 ns and then fluctuated within a narrow range of 0.23–0.27 nm, showing an overall stable trend. These results indicated that, after initial conformational adaptation, the binding mode was stable with no obvious drift, demonstrating good conformational stability in the target site. The complex RMSD increased quickly to ∼0.48 nm within the first 20 ns and then remained stable at 0.48–0.52 nm throughout the simulation. This indicated that, after initial adjustment, the protein–peptide system reached a stable state without major later perturbations, reflecting good dynamic stability of the complex. Overall residue fluctuations were low, with most RMSF values at 0.1–0.3 nm. Peaks appeared only at the N/C termini and several loop regions (around residues ∼50, 250, 500, and 750), with a maximum of ∼0.75 nm. These fluctuations occurred in non-core regions and did not affect structural stability at the binding site, indicating a relatively rigid interface that was not markedly perturbed by IEVVD binding. The Rg increased from ∼2.96 nm to ∼3.03 nm and then stabilized with small fluctuations in the mid-to-late stage. This suggested slight expansion after IEVVD binding while overall compactness was maintained; the binding process did not cause unfolding or abnormal opening and reflected a reasonable binding-induced adjustment. The number of hydrogen bonds fluctuated between ∼5 and 10 during the simulation, indicating a persistent hydrogen-bond network. A slight increase around 50 ns likely reflected formation of new hydrogen bonds during fine-tuning of the interface, which further stabilized the complex. The overall hydrogen-bond distribution was balanced and favored stable binding. SASA increased from ∼405 nm^2^ to ∼440 nm^2^ and then approached a plateau, indicating modest expansion of the protein surface and greater solvent exposure during binding. This change was typically associated with local pocket opening and surface adaptation, further supporting reasonable rearrangement and enhanced stability after complex formation. In sum, IEVVD exhibited good binding adaptability and structural stability in the pocket. Ligand RMSD was steady, the complex showed no large perturbations, backbone flexibility remained reasonable, and a persistent hydrogen-bond network was maintained at the interface. The slight increases in Rg and SASA indicated moderate conformational adjustment that favored formation of a robust binding interface(Cui et al., 2025; Liu et al., 2023; Mei et al., 2025).

The RMSD of EIVDV rose rapidly to ∼0.15 nm at the start of the simulation and then remained relatively stable at ∼0.12–0.17 nm during 10–70 ns. A late increase appeared after ∼90 ns, approaching 0.20 nm, indicating a slight decline in conformational stability that might have been related to loosening or fine adjustments of the binding mode. Overall fluctuations were small, with no drift or signs of dissociation, indicating that EIVDV maintained good positioning in the pocket. The complex RMSD increased quickly to ∼0.45 nm within the first 25 ns, then rose slowly to ∼0.52 nm and reached a plateau. The system therefore underwent early conformational adjustment and then entered a stable phase, showing good global stability without protein disorder during binding. Protein RMSF values in most regions stayed within 0.15–0.30 nm, indicating strong rigidity and structural stability. Peaks near residues ∼250, 450, and 750 reached ∼0.50–0.60 nm and were mainly located in loop or terminal regions, which were within normal fluctuation ranges. Fluctuations around the binding region were small, indicating that the ligand did not markedly perturb the core binding conformation. The Rg increased from ∼3.00 nm to ∼3.07 nm with small fluctuations and a steady trend, indicating no pronounced collapse or expansion after EIVDV binding. A slight late increase might have been related to local adaptation or modest pocket opening and remained within a stable range. The number of hydrogen bonds remained at ∼10–13 in the early stage, then decreased slightly but fluctuated overall within ∼8–12, indicating a stable ligand–protein hydrogen-bond network. Maintenance of this network was critical for the binding stability of EIVDV, as multipoint interactions helped fix the ligand conformation and enhance affinity. SASA rose from ∼400 nm^2^ to ∼430–440 nm^2^ and then approached a plateau, indicating slight expansion or exposure of the protein surface during binding. This change was usually associated with local pocket opening and surface adaptation, which supported reasonable rearrangement and enhanced stability after complex formation. Overall, EIVDV showed good binding stability and dynamic adaptability during the simulation. Ligand RMSD was stable, the hydrogen-bond network persisted, and the complex structure remained stable without major perturbations. Although ligand RMSD and SASA increased slightly at the end, these changes were within an acceptable adjustment range, indicating that EIVDV was a promising protein-binding peptide(Cui et al., 2025; Liu et al., 2023; Mei et al., 2025).

The RMSD of IGVND rose rapidly to ∼0.15 nm within the first 20 ns and then fluctuated at 0.15–0.20 nm during the mid-to-late stage with small amplitude, indicating high conformational stability without drift or dissociation. The complex RMSD increased to ∼0.45 nm within the first 20 ns and then fluctuated slightly at 0.45–0.48 nm, showing that the system entered a dynamic equilibrium after initial adaptation and that binding did not markedly perturb the protein. Most residue RMSF values were < 0.30 nm, indicating good rigidity. Distinct peaks appeared near residues ∼275, 450, 600, and 800, approaching ∼0.75 nm; these regions were likely loop or terminal segments without direct coupling to the core structure. Binding of IGVND did not appreciably increase flexibility in these regions, indicating a stable local environment around the site. The Rg increased slowly from ∼2.95 nm to ∼3.01 nm with minor fluctuations, showing neither unfolding nor over-compression. The steady rise reflected moderate binding-induced adaptation toward a stable global fold. The number of hydrogen bonds remained within 6–11 throughout the simulation with a relatively high mean, indicating a persistent network that was key to maintaining the bound conformation and enhancing binding affinity and thermal stability. SASA rose from ∼400 nm^2^ to ∼430 nm^2^ during 0–25 ns and then remained stably elevated, suggesting moderate opening around the site and increased exposure of polar residues. This favored water-mediated interactions and was consistent with a dynamically stable complex. Overall, IGVND exhibited high conformational stability and binding adaptability: ligand RMSD remained stable, the complex showed no major perturbations, the interfacial hydrogen-bond network persisted, and the protein retained a compact fold. Moderate increases in SASA and Rg reflected reasonable binding-induced remodeling that facilitated deep pocket insertion and stable positioning(Cui et al., 2025; Liu et al., 2023; Mei et al., 2025).

#### 3.4.2 MM-GBSA binding energy results

Based on molecular dynamics trajectories, the MM-GBSA endpoint method was used to evaluate the binding free energies of ligand–receptor complexes. This method combined molecular mechanics gas-phase energy (E_vdw_ + E_elec_) with continuum solvation terms (polar ΔG_GB_ and nonpolar ΔG_SA_) to approximate the binding driving force. It was commonly used for relative ranking of multiple ligands and assessment of pose robustness; more negative values generally indicated more favorable interactions (unit: kcal/mol). As shown in Appendix Table 3, ΔG_bind_ for T1R1–T1R3/EESY, T1R1–T1R3/EIVDV, T1R1–T1R3/IEVVD, and T1R1–T1R3/IGVND were − 70.91 ± 7.37, −61.26 ± 3.42, −60.93 ± 2.33, and − 55.24 ± 3.69 kcal/mol (mean ± SD), respectively. All values were negative, indicating effective affinity of these peptides for the receptor complex. Among them, EESY was most favorable; EIVDV and IEVVD were comparable; IGVND was slightly weaker.

**Fig. 4:**
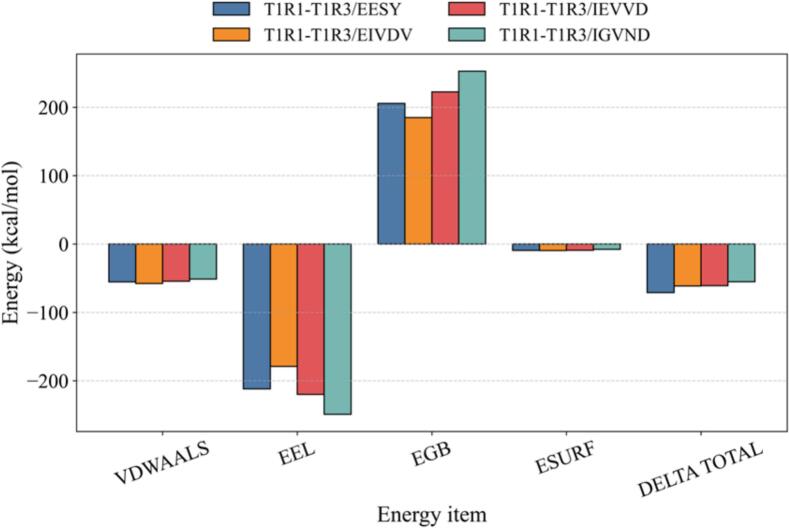
MM-GBSA binding energies and energy decomposition. Note: VDWAALS (van der Waals)—molecular-mechanics van der Waals interaction energy from the force-field Lennard–Jones term; EEL (electrostatic)—molecular-mechanics electrostatic interaction energy (point-charge Coulomb term); EGB (polar solvation)—polar solvation free energy calculated by the generalized Born (GB) model; ESURF (nonpolar solvation / surface area)—nonpolar solvation free energy from an empirical surface-area model; DELTA TOTAL / ΔG_bind_—end-state approximation of binding free energy.

**Fig. 5:**
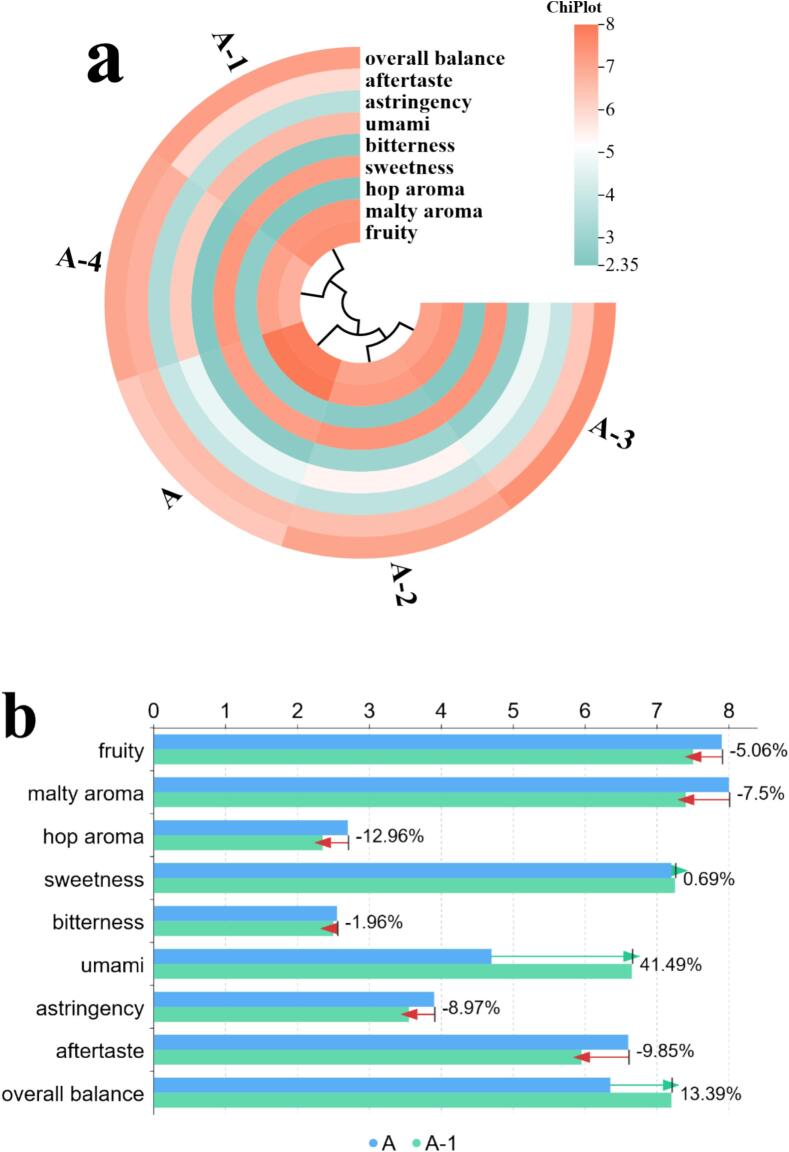

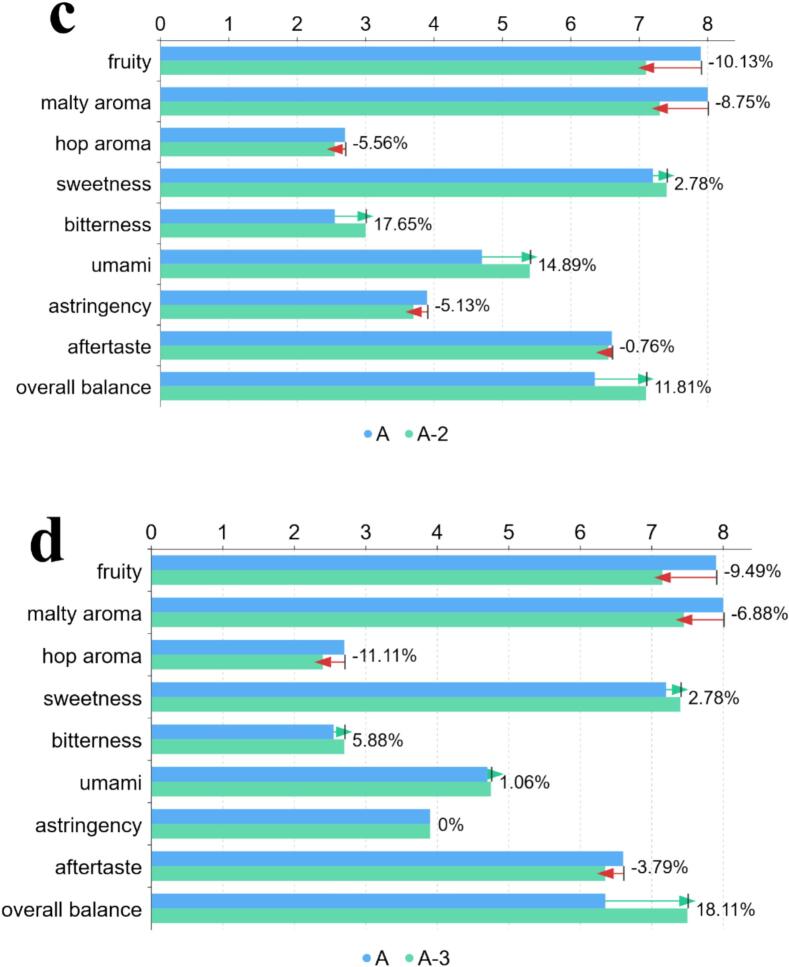

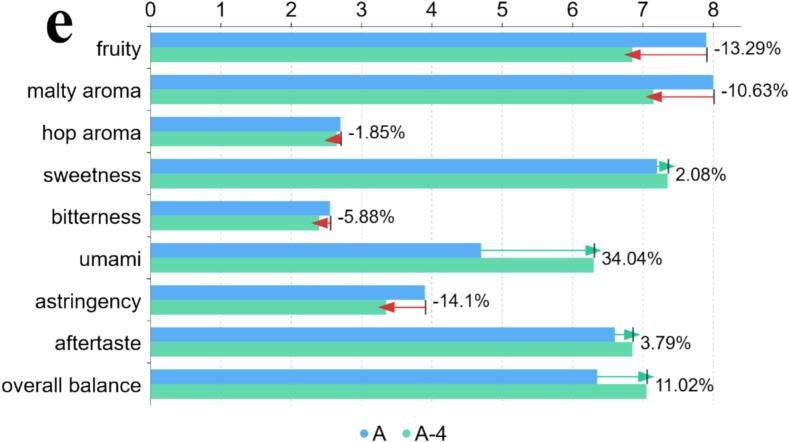
Multidimensional sensory effects of the single-addition variable experiment on the beer body. (a) overall sensory scores of beer samples; (b) comparison of beer-body sensory attributes for single addition of EESY vs the base sample; (c) comparison for single addition of IEVVD vs the base sample; (d) comparison for single addition of EIVDV vs the base sample; (e) comparison for single addition of IGVND vs the base sample.

As shown in Fig. 4, MM-GBSA energy decomposition indicated a typical pattern of “favorable gas-phase interactions offset by polar solvation” for all four complexes. The van der Waals term (VDWAALS) and Coulombic term (EEL) were markedly negative (about −51 to −58 and − 179 to −249 kcal/mol, respectively). The generalized Born polar solvation (EGB) was positive and strongly compensated the gas-phase electrostatics, while the nonpolar solvation (ESURF) was slightly negative (about −8 to −9 kcal/mol). The net binding free energies (ΔG_bind_; DELTA TOTAL) were EESY (−70.91 ± 7.37) < EIVDV (−61.26 ± 3.42) ≈ IEVVD (−60.93 ± 2.33) < IGVND (−55.24 ± 3.69) kcal/mol, indicating effective affinity for all four, with EESY most favorable and IGVND relatively weaker. Notably, IGVND showed the strongest EEL (−248.94) but also the largest EGB penalty (+252.73), which reduced the net favorability. In contrast, EESY showed a smaller degree of EEL (−211.78)–EGB (+205.62) cancellation, yielding a more favorable total energy. This electrostatic–desolvation cancellation was a common feature of the MM-GBSA endpoint framework. ΔG_bind_ consisted of gas-phase molecular mechanics energy (E_vdW_ + E_elec_) and solvation terms (G_GB_ + G_SA_). Enhancement of electrostatics was often accompanied by a higher polar desolvation cost, and the relative ranking was determined by the net balance rather than any single extreme component. The van der Waals term varied little across the set and mainly provided baseline burial stabilization. Subsequently, modern sensomics was used to validate the multidimensional effects of these four umami peptides on the beer body. (See Fig. 5.)

### 3.5 Single-addition variable verification experiments

#### 3.5.1 Sensory enhancement by umami peptides and structure–function analysis

Using base sample, A (4.70) as reference, “umami” scores after single-peptide addition were: A-1 (EESY) 6.65, +41.5 %; A-4 (IGVND) 6.30, +34.0 %; A-2 (IEVVD) 5.40, +14.9 %; A-3 (EIVDV) 4.75, +1.1 %. The overall order was EESY > IGVND > IEVVD ≫ EIVDV. EESY and IGVND produced significant increases (*p* < 0.01). IEVVD showed a moderate enhancement (*p* < 0.05). EIVDV was near baseline (*p* > 0.05).

From a molecular-recognition view, the extracellular VFT of T1R1/T1R3 tended to stabilize short-peptide poses by multipoint H-bond/electrostatic anchoring from acidic/polar side chains, assisted by van der Waals burial of hydrophobic fragments, thereby biasing the VFT open/closed balance. EESY (two Glu and containing S/Y) more readily formed a dense polar network and thus performed best. IGVND achieved the next-best effect via hydrophobic fitting from I/V/G. Sequence-order differences between EIVDV and IEVVD altered the geometric matching of anchors and hydrophobic groups in the pocket, as well as the desolvation balance, leading to divergent gains. This “polar anchoring–hydrophobic registration” mechanism agreed with the class C GPCR recognition paradigm and the classic model of umami synergy with nucleotides (IMP/GMP), providing a structure–energetics explanation for the sensory differences.

#### 3.5.2 Multidimensional sensory effects of single-peptide umami additions on the beer body

In the aroma dimension, fruity and malty aroma decreased significantly (p < 0.05) across all four samples: A-1 (EESY) −5.06 %/−7.50 %, A-2 (IEVVD) −10.13 %/−8.75 %, A-3 (EIVDV) −9.49 %/−6.87 %, A-4 (IGVND) −13.29 %/−10.62 %. Hop aroma decreased significantly in A-1 (−12.96 %) and A-3 (−11.11 %), while changes in A-2 (−5.56 %) and A-4 (−1.85 %) were not significant. In taste and mouthfeel–related dimensions, sweetness rose slightly (A-1 + 0.69 %; A-2/A-3 + 2.78 %; A-4 + 2.08 %). Bitterness diverged: A-2 + 17.65 %, A-3 + 5.88 %, whereas A-1 − 1.96 % and A-4 − 5.88 %. Astringency decreased in A-1/A-2/A-4 by −8.97 %/−5.13 %/−14.10 %; A-3 was unchanged. For aftertaste, A-1 − 9.85 %, A-2 − 0.76 %, A-3 − 3.79 %, whereas A-4 + 3.79 %. Overall balance increased in all groups: A-1 + 13.39 %, A-2 + 11.81 %, A-3 + 18.11 %, A-4 + 11.02 %. The overall pattern showed that EESY and IGVND tended to reduce bitterness and astringency and improve overall balance; EIVDV produced the largest gain in overall balance with a slight rise in bitterness; IEVVD showed a marked increase in bitterness while overall balance still improved.

These results suggested stronger modulation of taste–mouthfeel dimensions than of the aroma dimension. EESY and IGVND consistently reduced bitterness/astringency and enhanced overall balance, reflecting masking and balancing effects of umami. IEVVD and EIVDV both slightly increased sweetness, but their effects on bitterness diverged, indicating sensitivity to sequence order. Related findings indicated that umami substances could suppress bitter-receptor pathways or reduce bitterness saliency at integrative levels and showed synergy for saltiness/overall savory perception, thereby improving mouthfeel balance and the roundness of aftertaste. These patterns were consistent with the observed increases in overall balance and multidimensional decreases in bitterness and astringency.

## 4 Conclusion

Using real lager samples, this work closed the loop from sequence to sensory. At the proteomics level, a beer-matrix–oriented identification and high-confidence filtering paradigm was established. At the receptor level, homology modeling, docking, and MD/MM-GBSA supported a unified mechanism of “VFT stabilization via polar anchoring and hydrophobic registration,” with representative peptides (e.g., EESY) showing energetic plausibility and conformational robustness. At the phenotype level, single-factor additions increased umami and improved balance, while shifting bitterness and astringency profiles. These findings were directly translatable to practice: breweries could modulate proteolysis and fermentation to tune short-peptide release, pair umami peptides with 5′-nucleotides to leverage synergy, and target bitterness/astringency management in formulation and process control. The approach provided an actionable framework that connected molecular recognition to sensory outcomes in lager, advancing mechanism-based flavor design.

Several limitations remained. Beer was a complex matrix in which nucleotides, organic acids, amino acids, and polyphenols could modulate taste and mouthfeel, complicating attribution to peptides alone. RPLC–Q-TOF-MS/de novo sequencing introduced confidence and mass-coincidence challenges, and homology models of T1R1/T1R3 carried structural uncertainty relative to experimental receptors. Sensory outcomes also depended on panel methods and temporal profiling, which introduced variability across batches and processes. Future work should quantify key co-factors, strengthen identification with orthogonal targets, refine receptor models, and expand cross-batch validation under industrial conditions.

## CRediT authorship contribution statement

**Yashuai Wu:** Writing – review & editing, Writing – original draft, Supervision, Software, Resources, Methodology, Investigation, Formal analysis, Data curation, Conceptualization. **Ruiyang Yin:** Validation, Resources, Methodology, Investigation, Formal analysis, Conceptualization. **Zhiyuan Gao:** Validation, Software, Project administration, Investigation, Formal analysis, Data curation, Conceptualization. **Liyun Guo:** Supervision, Software, Resources, Investigation, Data curation. **Yumei Song:** Supervision, Resources, Investigation, Formal analysis, Conceptualization. **Dongrui Zhao:** Writing – review & editing, Writing – original draft, Supervision, Software, Methodology, Investigation, Funding acquisition, Formal analysis, Data curation, Conceptualization. **Jinyuan Sun:** Validation, Resources, Methodology, Formal analysis, Data curation, Conceptualization. **Mingquan Huang:** Validation, Software, Resources, Project administration, Investigation, Conceptualization. **Baoguo Sun:** Supervision, Software, Project administration, Funding acquisition, Formal analysis, Data curation, Conceptualization.

## Institutional review board statement

In accordance with the ethical principles outlined in the Declaration of Helsinki, all participants provided informed consent before participating in the study. The anonymity and confidentiality of the participants were guaranteed, and participation was completely voluntary. The participants' informed consent form was provided in Appendix 4.

## Funding

This research was supported by the Beijing Elite Scientist Sponsorship Program of BAST (No. BYESA.2023055), the National Key Research and Development Program of China (No. 2022YFD2101205), and the National Natural Science Foundation of China (No. 32322068).

## Declaration of competing interest

The authors declare that they have no known competing financial interests or personal relationships that could have appeared to influence the work reported in this paper.

## Figures and Tables

**Supplementary Fig. S1:**
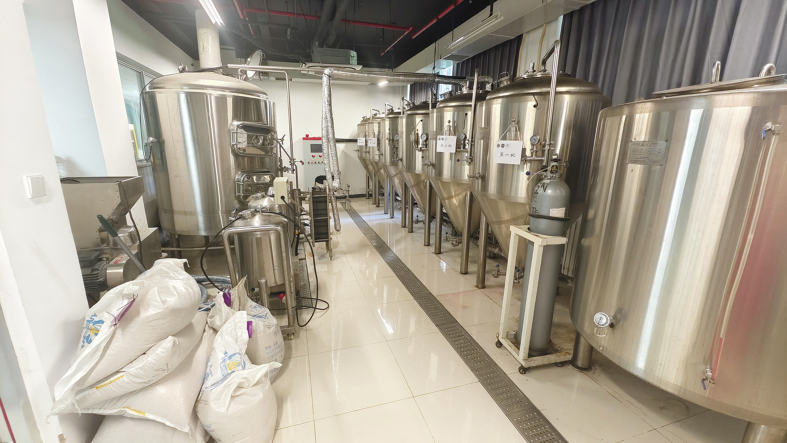


## Data Availability

Data will be made available on request.

## Appendix A Supplementary data

Supplementary material 1 Identification of Peptides and Prediction of Umami Activity in Lager Beer. Supplementary material 1

Supplementary material 2 Basic information, taste description, and threshold of selected umami peptides Supplementary material 2

Supplementary material 3 Binding free energies and energy components predicted by MM GBSA Supplementary material 3

Supplementary material 4 Subject informed consent form Supplementary material 4

Supplementary material 5 Map of the craft beer workshop of Beijing Technology and Business University Supplementary material 5

Supplementary material 6 Detailed results of peptide retrieval in beer Supplementary material 6
