# Supplementary material for: Mining umami peptides in lager and multidimensional sensory evaluation of the beer body integrating computational biology with modern sensomics
Source: Food Chem X. 2025 Oct 6;31:103132. doi: 10.1016/j.fochx.2025.103132 (PMC12538414; doi:10.1016/j.fochx.2025.103132)
Supplement: Supplementary material 4 — Subject informed consent form [file mmc4.pdf]

受试者知情同意书

尊敬的先生/女士：

我们将邀请您参加一项"Mining Umami Peptides in Lager and Multidimensional Sensory Evaluation of the Beer Body Integrating Computational Biology with Modern Sensomics）"的科学研究。

在您决定是否参加这项研究之前，请仔细阅读以下内容，它可以帮助您了解该项研究内容、为何要进行这项研究以及本研究可能给您带来的益处、风险和不适等。

以下是本项研究的介绍：

一、研究背景

拉格啤酒的风味品质与其所含的滋味肽密切相关，这类小分子物质通过调控苦味、鲜味及口感等感官属性，直接影响消费者对产品的接受度。尽管滋味肽的形成机制已有部分研究，但其在复杂啤酒体系中对整体感官特征的多维作用（如协同或抑制效应）仍缺乏系统性分析。现有技术手段难以完全模拟人类感官对风味的综合感知，因此需通过受试者的感官评价实验获取真实反馈。

二、研究目的

本研究旨在明确拉格啤酒中滋味肽的感官贡献，揭示其与酒体特性的关联规律，为优化生产工艺提供依据。实验将遵循伦理规范，确保参与者知情权及数据隐私。

三、具体程序和流程

测试内容：品尝拉格啤酒样品及处理后的拉格啤酒样品（每份 20 mL），分别对应不同滋味肽含量或工艺条件；根据标准化感官评价表，对样品的香气、滋味、口感顺滑度、余味持久性及整体接受度等进行评分（采用 10 分量表，0=极不喜欢，9=极喜欢等类似感官量表）。

测试流程：准备阶段（5 分钟）：参与者签署知情同意书，确认无酒精过敏或健康禁忌；实验员讲解评分规则，提供清水及无味饼干用于清洁口腔；样品测试阶段（15 分钟）：样品按随机顺序提供，每次品尝后需用清水漱口，间隔 1 分钟以减少感官残留；独立隔间内完成评价，避免受他人或环境干扰；结束阶段（5 分钟）：回收问卷，确认数据完整性。

四、如果参加研究，您需要做什么？

样品品评：在约定时间到达指定实验室，按照研究人员指引，依次品尝不同拉格啤酒样品（每份 20 mL，编号随机）。每次品尝后需用清水漱口，间隔 1 分钟，再进行下一轮品评。

填写问卷：品评后立即通过平板电脑或纸质表单完成标准化感官评分问卷，内容包括对啤酒的香气、滋味、口感顺滑度、余味持久性及整体接受度等的评分（0-9 分）。

参加研究前：请在测试前 1 小时内避免食用辛辣、高糖或重口味食物（如咖啡、巧克力、大蒜等），且勿吸烟或饮酒，以确保感官灵敏度。建议穿着舒适衣物，避免使用香水或气味强烈的护肤品。

参加研究中：请严格按编号顺序独立品尝样品，勿与他人讨论感受或观察他人反应。所有评价需在独立隔间内完成，以排除环境干扰。

参加研究后：完成问卷并确认无误后，可领取小礼品并自由离开。若在测试后 24 小时内出现不适（如头晕等），请及时联系研究人员。

五、参加此项研究可能给您带来的受益

参与本研究可使您深入了解拉格啤酒的酿造工艺及风味科学原理，直观感受不同滋味肽对啤酒感官特征的调控作用，提升对啤酒品质的鉴赏能力。您的评价数据将直接贡献于食品风味研究领域，为优化生产工艺、提升产品适口性提供科学依据，推动行业技术创新。此外，您对样品的反馈可能被纳入后续产品改进参考，潜在影响未来市场啤酒风味设计。

六、参加此项研究可能给您带来的不良反应、风险及风险防范措施

本研究涉及少量酒精摄入（每份样品约 20 mL，实际摄入 1-3mL 即可，酒精度≤6%），可能对酒精敏感者引发轻微头晕或口腔黏膜刺激，但风险极低。感官测试中频繁品尝可能导致短暂味觉疲劳或口感麻木。为防控风险，实验前将严格筛选参与者健康状况（如酒精过敏史、妊娠等），并提供清水漱口及无味饼干以清除残留。测试全程单次样品量经安全评估。若出现不适（如恶心、眩晕等），可立即暂停或退出实验，并联系医护人员。所有数据匿名处理，隐私受法律保护。研究者已制定应急预案，确保测试环境安全可控。

七、费用情况说明

参与本次研究无需您支付任何费用。所有用于试喝的产品以及相关问卷材料均由研究团队提供。

八、参加此项研究的补偿，包括损伤赔偿

您将获得 10 元的报酬以感谢您的参与。产品经过严格安全检测，但如因参与本次研究导致身体损伤，我们将承担相应的医疗费用，并根据实际情况给予合理赔偿。在研究过程中

若发生任何意外，请立即联系研究负责人。

九、您个人信息的保密

您的个人信息（姓名、联系方式）将严格保密，仅用于研究记录。感官评价数据将以匿名形式处理，不涉及个人身份信息。

十、您必须参加此项研究吗？

参与本次研究完全基于您的自愿。您有权随时拒绝参加或在研究过程中随时退出，且不会因此遭受任何不利影响。您的决定将得到充分尊重。

十一、是否中途可以退出此试验？

您的参与完全自愿，拒绝参与不会影响您的任何权益。您可在测试过程中随时退出，无需说明理由。

十二、伦理委员会

如果您对本研究有疑惑，可咨询本研究负责人，电话见下文。

如果您在研究中有不满或建议，请联系北京工商大学食品学院科学研究伦理委员会。

联系电话：15210909542

请您保留这份资料。

同意声明

1. 我已经阅读了本知情同意书，项目相关责任人已经将此次试验的目的、内容、风险和受益情况向我作了详细的解释说明。
2. 我已经讨论并询问了有关本研究的相关问题，这些问题的解答令我满意。
3. 我有充足的时间作出决定。
4. 我是自愿同意参加本文说介绍的科学研究，并同意将我的研究数据用于本研究的发表。
5. 我同意伦理委员会或课题资助部门代表查阅我的研究资料。
6. 我将获得一份经过签名并注明日期的知情同意书副本。

最后，我决定同意参加本项试验研究。

受试者签名：苗子健　　日期：2025 年 8 月 24 日

受试者联系电话：18511951847

受试者签名：陈乙源　　日期：2025 年 8 月 24 日
受试者联系电话：19500267648

受试者签名：谢江　　日期：2015 年 8 月 24 日
受试者联系电话：13426343695

受试者签名：袁欣　　日期：2025 年 8 月 24 日
受试者联系电话：13141367267

受试者签名：薛松保　　日期：2015 年 8 月 24 日
受试者联系电话：13760792637

受试者签名：武亚惠　　日期：2025 年 8 月 24 日
受试者联系电话：15226537124

受试者签名：邹明钟　　日期：2025 年 8 月 24 日
受试者联系电话：18288739231

受试者签名：洪嘉欣　　日期：2025 年 8 月 24 日
受试者联系电话：18400704529

受试者签名：武王帅　　日期：2025 年 8 月 24 日
受试者联系电话：15110107515

受试者签名：武亚茹 日期：2025 年 8 月 24 日

受试者联系电话：18468274172

受试者签名：陈锡 日期：2025 年 8 月 24 日

受试者联系电话：13717881073

受试者签名：张明霞 日期：2025 年 8 月 24 日

受试者联系电话：14701576548

受试者签名：陈德奇 日期：2025 年 8 月 24 日

受试者联系电话：13439189028

受试者签名：赵保龙 日期：2025 年 8 月 24 日

受试者联系电话：13241465845

受试者签名：[illegible] 日期：2025 年 8 月 24 日

受试者联系电话：15210909542

我确认已向受试者解释了本研究的详细情况，包括其权利以及可能的受益和风险，并给其一份签署过的知情同意书副本。

研究者签名：[illegible] 日期：2025 年 8 月 24 日

研究者联系方式：15210909542
