# Supplementary material for: Mining umami peptides in lager and multidimensional sensory evaluation of the beer body integrating computational biology with modern sensomics
Source: Food Chem X. 2025 Oct 6;31:103132. doi: 10.1016/j.fochx.2025.103132 (PMC12538414; doi:10.1016/j.fochx.2025.103132)
Supplement: Supplementary material 5 — Map of the craft beer workshop of Beijing Technology and Business University [file mmc5.zip › db.proteins.html]

proteins


Protein List

  

| Protein Group | Protein ID | Accession | -10lgP | Coverage (%) | Coverage (%) POS\_R15-1 | Area POS\_R15-1 | #Peptides | #Unique | #Spec POS\_R15-1 | PTM | Avg. Mass | Description |
| --- | --- | --- | --- | --- | --- | --- | --- | --- | --- | --- | --- | --- |
| 1 | 1 | C4QWD1|C4QWD1\_KOMPG | 119.11 | 6 | 6 | 3.37e3 | 85 | 1 | 174 | Pyro-glu from E, Pyro-glu from Q | 559559.4375 | Midasin OS=Komagataella phaffii (strain GS115 / ATCC 20864) OX=644223 GN=PAS\_chr1-1\_0187 PE=3 SV=1 |
| 4 | 2 | C4QYT3|C4QYT3\_KOMPG | 107.24 | 11 | 11 | 1.75e3 | 47 | 2 | 104 | Pyro-glu from E, Pyro-glu from Q | 147367.4688 | Phosphoribosylformylglycinamidine synthase OS=Komagataella phaffii (strain GS115 / ATCC 20864) OX=644223 GN=PAS\_chr1-4\_0551 PE=3 SV=1 |
| 15 | 3 | C4QVT9|C4QVT9\_KOMPG | 99.47 | 8 | 8 | 0 | 39 | 1 | 82 |  | 164798.1719 | Lysophospholipase NTE1 OS=Komagataella phaffii (strain GS115 / ATCC 20864) OX=644223 GN=PAS\_chr1-1\_0009 PE=3 SV=1 |
| 16 | 4 | C4R5H2|C4R5H2\_KOMPG | 100.32 | 9 | 9 | 1.07e3 | 38 | 1 | 79 | Pyro-glu from E, Pyro-glu from Q | 147900.6094 | GDP/GTP exchange factor required for mitotic exit at low temperatures OS=Komagataella phaffii (strain GS115 / ATCC 20864) OX=644223 GN=PAS\_chr3\_0758 PE=4 SV=1 |
| 20 | 5 | C4QXA9|C4QXA9\_KOMPG | 98.59 | 6 | 6 | 4.18e3 | 37 | 2 | 73 | Pyro-glu from E, Pyro-glu from Q | 245627.9219 | RNA-dependent ATPase RNA helicase (DEIH box) OS=Komagataella phaffii (strain GS115 / ATCC 20864) OX=644223 GN=PAS\_chr1-4\_0051 PE=4 SV=1 |
| 24 | 6 | C4R7W9|C4R7W9\_KOMPG | 95.63 | 9 | 9 | 0 | 34 | 1 | 75 | Pyro-glu from E, Pyro-glu from Q | 124250.0547 | Lon protease homolog, mitochondrial OS=Komagataella phaffii (strain GS115 / ATCC 20864) OX=644223 GN=PIM1 PE=3 SV=1 |
| 31 | 7 | C4R6X0|C4R6X0\_KOMPG | 93.6 | 5 | 5 | 1.14e3 | 33 | 2 | 45 | Pyro-glu from Q | 292292.3438 | Protein involved in cell morphogenesis and proliferation, associated with protein kinase Cbk1p OS=Komagataella phaffii (strain GS115 / ATCC 20864) OX=644223 GN=PAS\_chr4\_0119 PE=4 SV=1 |
| 37 | 8 | C4R8N7|C4R8N7\_KOMPG | 95.12 | 11 | 11 | 3.52e2 | 32 | 1 | 76 | Pyro-glu from E, Deamidation (NQ), Pyro-glu from Q | 98292.1016 | Phosphoinositide phospholipase C OS=Komagataella phaffii (strain GS115 / ATCC 20864) OX=644223 GN=PAS\_chr4\_0700 PE=4 SV=1 |
| 38 | 9 | C4QVT8|C4QVT8\_KOMPG | 93.04 | 6 | 6 | 0 | 32 | 2 | 51 | Oxidation (M), Pyro-glu from Q | 229741.6094 | Fatty acid synthase subunit beta OS=Komagataella phaffii (strain GS115 / ATCC 20864) OX=644223 GN=PAS\_chr1-1\_0008 PE=1 SV=1 |
| 40 | 10 | C4QZ69|C4QZ69\_KOMPG | 93.99 | 6 | 6 | 0 | 32 | 1 | 76 | Pyro-glu from E | 177551.5625 | Myosin-2 OS=Komagataella phaffii (strain GS115 / ATCC 20864) OX=644223 GN=PAS\_FragB\_0012 PE=3 SV=1 |
| 40 | 11 | C4R8M1|C4R8M1\_KOMPG | 20.62 | 2 | 2 | 0 | 1 | 1 | 1 |  | 28337.2637 | Uncharacterized protein OS=Komagataella phaffii (strain GS115 / ATCC 20864) OX=644223 GN=PAS\_chr4\_0687 PE=4 SV=1 |
| 41 | 12 | C4R2A4|C4R2A4\_KOMPG | 98.44 | 12 | 12 | 3.25e3 | 32 | 1 | 100 | Acetylation (Protein N-term) | 81764.5703 | Transcriptional regulator involved in glucose repression of Gal4p-regulated genes OS=Komagataella phaffii (strain GS115 / ATCC 20864) OX=644223 GN=PAS\_chr2-2\_0284 PE=1 SV=1 |
| 43 | 13 | C4R0P1|C4R0P1\_KOMPG | 139.98 | 25 | 25 | 5.65e3 | 32 | 6 | 86 |  | 35603.5234 | Glyceraldehyde-3-phosphate dehydrogenase OS=Komagataella phaffii (strain GS115 / ATCC 20864) OX=644223 GN=PAS\_chr2-1\_0437 PE=3 SV=1 |
| 44 | 14 | C4R8Y4|C4R8Y4\_KOMPG | 94.04 | 10 | 10 | 0 | 31 | 1 | 67 | Pyro-glu from E | 124925.4219 | Alpha-mannosidase OS=Komagataella phaffii (strain GS115 / ATCC 20864) OX=644223 GN=PAS\_chr4\_0790 PE=3 SV=1 |
| 55 | 15 | C4R564|C4R564\_KOMPG | 91.19 | 7 | 7 | 0 | 30 | 1 | 44 | Pyro-glu from Q | 167051.7031 | Type I transmembrane sorting receptor for multiple vacuolar hydrolases OS=Komagataella phaffii (strain GS115 / ATCC 20864) OX=644223 GN=PAS\_chr3\_0653 PE=4 SV=1 |
| 74 | 16 | C4R723|C4R723\_KOMPG | 92.14 | 6 | 6 | 1.26e3 | 27 | 2 | 37 |  | 201150.4688 | Urea amidolyase OS=Komagataella phaffii (strain GS115 / ATCC 20864) OX=644223 GN=PAS\_chr4\_0173 PE=4 SV=1 |
| 76 | 17 | C4QZB6|C4QZB6\_KOMPG | 91.5 | 5 | 5 | 3.12e2 | 27 | 1 | 41 |  | 216815.5 | Formin OS=Komagataella phaffii (strain GS115 / ATCC 20864) OX=644223 GN=PAS\_FragB\_0058 PE=3 SV=1 |
| 78 | 18 | C4QWB0|C4QWB0\_KOMPG | 95.47 | 7 | 7 | 0 | 27 | 1 | 49 |  | 143896.7812 | Vacuolar membrane protein that transits through the biosynthetic vacuolar protein sorting pathway OS=Komagataella phaffii (strain GS115 / ATCC 20864) OX=644223 GN=PAS\_chr1-1\_0168 PE=3 SV=1 |
| 97 | 19 | C4QWW6|C4QWW6\_KOMPG | 91.63 | 6 | 6 | 4.15e1 | 26 | 1 | 43 | Pyro-glu from E | 186969.6094 | HECT-type E3 ubiquitin transferase OS=Komagataella phaffii (strain GS115 / ATCC 20864) OX=644223 GN=PAS\_chr1-1\_0363 PE=3 SV=1 |
| 103 | 20 | C4R6B2|C4R6B2\_KOMPG | 90.2 | 8 | 8 | 4.48e3 | 25 | 1 | 63 | Pyro-glu from E | 120142.4453 | Component of the Paf1p complex OS=Komagataella phaffii (strain GS115 / ATCC 20864) OX=644223 GN=PAS\_chr3\_1035 PE=1 SV=1 |
| 110 | 21 | C4R567|C4R567\_KOMPG | 85.73 | 9 | 9 | 0 | 25 | 1 | 43 | Pyro-glu from E | 115926.2734 | GTPase activating factor for Rsr1p/Bud1p required for both axial and bipolar budding patterns OS=Komagataella phaffii (strain GS115 / ATCC 20864) OX=644223 GN=PAS\_chr3\_0656 PE=4 SV=1 |
| 115 | 22 | C4R692|C4R692\_KOMPG | 91.63 | 12 | 12 | 7.33e3 | 25 | 1 | 66 |  | 66626.6094 | Arrestin C-terminal-like domain-containing protein OS=Komagataella phaffii (strain GS115 / ATCC 20864) OX=644223 GN=PAS\_chr3\_1017 PE=4 SV=1 |
| 121 | 23 | C4R6C5|C4R6C5\_KOMPG | 85.44 | 8 | 8 | 1.47e3 | 24 | 1 | 42 | Pyro-glu from E, Pyro-glu from Q | 122829.7969 | Uncharacterized protein OS=Komagataella phaffii (strain GS115 / ATCC 20864) OX=644223 GN=PAS\_chr3\_1049 PE=4 SV=1 |
| 126 | 24 | C4R0N9|C4R0N9\_KOMPG | 91.92 | 10 | 10 | 3.04e3 | 24 | 1 | 46 | Pyro-glu from E, Pyro-glu from Q | 90327.2812 | Mitochondrial intermediate peptidase OS=Komagataella phaffii (strain GS115 / ATCC 20864) OX=644223 GN=PAS\_chr2-1\_0435 PE=3 SV=1 |
| 139 | 25 | C4R3C5|C4R3C5\_KOMPG | 86.67 | 6 | 6 | 5.67e2 | 23 | 1 | 38 |  | 160216.8594 | Uncharacterized protein OS=Komagataella phaffii (strain GS115 / ATCC 20864) OX=644223 GN=PAS\_chr3\_0032 PE=4 SV=1 |
| 143 | 26 | C4QZ71|C4QZ71\_KOMPG | 86.75 | 10 | 10 | 0 | 23 | 1 | 43 |  | 84188.1641 | Leucine-rich repeat-containing protein OS=Komagataella phaffii (strain GS115 / ATCC 20864) OX=644223 GN=PAS\_FragB\_0013 PE=4 SV=1 |
| 155 | 27 | C4QYQ0|C4QYQ0\_KOMPG | 88.81 | 12 | 12 | 1.42e4 | 23 | 1 | 62 | Pyro-glu from E, Pyro-glu from Q | 59575.4453 | Cysteine protease OS=Komagataella phaffii (strain GS115 / ATCC 20864) OX=644223 GN=PAS\_chr1-4\_0522 PE=3 SV=1 |
| 157 | 28 | C4QXT4|C4QXT4\_KOMPG | 91.32 | 14 | 14 | 2.74e2 | 23 | 1 | 53 |  | 54181.5352 | RNA binding protein that negatively regulates growth rate OS=Komagataella phaffii (strain GS115 / ATCC 20864) OX=644223 GN=PAS\_chr1-4\_0223 PE=4 SV=1 |
| 160 | 29 | C4R5D6|C4R5D6\_KOMPG | 87.56 | 13 | 13 | 2.59e2 | 23 | 1 | 63 |  | 51558.4141 | PXA domain-containing protein OS=Komagataella phaffii (strain GS115 / ATCC 20864) OX=644223 GN=PAS\_chr3\_0723 PE=4 SV=1 |
| 166 | 30 | C4R6N4|C4R6N4\_KOMPG | 82.03 | 9 | 9 | 1.02e3 | 22 | 2 | 40 | Pyro-glu from E, Oxidation (M), Pyro-glu from Q | 90634.6719 | Ribosome-releasing factor 2, mitochondrial OS=Komagataella phaffii (strain GS115 / ATCC 20864) OX=644223 GN=MEF2 PE=3 SV=1 |
| 173 | 31 | C4R2U0|C4R2U0\_KOMPG | 80.47 | 6 | 6 | 0 | 22 | 1 | 35 | Pyro-glu from Q | 155793.3281 | Xanthine dehydrogenase OS=Komagataella phaffii (strain GS115 / ATCC 20864) OX=644223 GN=PAS\_chr2-2\_0112 PE=3 SV=1 |
| 181 | 32 | C4R6D7|C4R6D7\_KOMPG | 83.98 | 9 | 9 | 0 | 21 | 1 | 52 | Pyro-glu from E | 88660.6797 | Protein kinase of the Mitotic Exit Network OS=Komagataella phaffii (strain GS115 / ATCC 20864) OX=644223 GN=PAS\_chr3\_1061 PE=4 SV=1 |
| 187 | 33 | C4R051|C4R051\_KOMPG | 85.47 | 5 | 5 | 2.88e3 | 21 | 1 | 29 | Pyro-glu from Q | 201490.7969 | 1,3-beta-glucan synthase OS=Komagataella phaffii (strain GS115 / ATCC 20864) OX=644223 GN=PAS\_chr2-1\_0263 PE=3 SV=1 |
| 195 | 34 | C4QZN7|C4QZN7\_KOMPG | 85.04 | 10 | 10 | 0 | 20 | 1 | 39 | Acetylation (Protein N-term) | 85701.0938 | Uncharacterized protein OS=Komagataella phaffii (strain GS115 / ATCC 20864) OX=644223 GN=PAS\_chr2-1\_0108 PE=4 SV=1 |
| 197 | 35 | C4QXW1|C4QXW1\_KOMPG | 79.74 | 4 | 4 | 2.73e2 | 21 | 2 | 33 | Pyro-glu from E, Pyro-glu from Q, Deamidation (NQ) | 247976.1562 | Acetyl-CoA carboxylase, biotin containing enzyme OS=Komagataella phaffii (strain GS115 / ATCC 20864) OX=644223 GN=PAS\_chr1-4\_0249 PE=4 SV=1 |
| 199 | 36 | C4QYF1|C4QYF1\_KOMPG | 91.08 | 8 | 8 | 1.27e4 | 21 | 2 | 51 | Pyro-glu from E, Half of a disulfide bridge, Pyro-glu from Q | 109311.4531 | Rho GTPase activating protein (RhoGAP) involved in control of the cytoskeleton organization OS=Komagataella phaffii (strain GS115 / ATCC 20864) OX=644223 GN=PAS\_chr1-4\_0424 PE=4 SV=1 |
| 210 | 37 | C4QW41|C4QW41\_KOMPG | 80.66 | 12 | 12 | 0 | 20 | 1 | 51 | Pyro-glu from E | 66685.5703 | Histone methyltransferase with a role in transcriptional elongation OS=Komagataella phaffii (strain GS115 / ATCC 20864) OX=644223 GN=PAS\_chr1-1\_0102 PE=4 SV=1 |
| 220 | 38 | C4QVN9|C4QVN9\_KOMPG | 83.95 | 25 | 25 | 9.8e2 | 20 | 1 | 42 | Pyro-glu from Q | 27218.7031 | Ribophorin II C-terminal domain-containing protein OS=Komagataella phaffii (strain GS115 / ATCC 20864) OX=644223 GN=PAS\_chr1-3\_0248 PE=4 SV=1 |
| 221 | 39 | C4R8H1|C4R8H1\_KOMPG | 76.07 | 5 | 5 | 1.67e3 | 20 | 3 | 34 | Deamidation (NQ) | 204317.7344 | Formin, nucleates the formation of linear actin filaments OS=Komagataella phaffii (strain GS115 / ATCC 20864) OX=644223 GN=PAS\_chr4\_0636 PE=3 SV=1 |
| 222 | 40 | C4R155|C4R155\_KOMPG | 93.11 | 4 | 4 | 2.18e2 | 20 | 1 | 37 |  | 241437.6406 | Presumed helicase required for RNA polymerase II transcription termination and processing of RNAs OS=Komagataella phaffii (strain GS115 / ATCC 20864) OX=644223 GN=PAS\_chr2-1\_0592 PE=3 SV=1 |
| 232 | 41 | C4QWS1|C4QWS1\_KOMPG | 78.68 | 7 | 7 | 2.15e2 | 19 | 2 | 28 | Pyro-glu from Q, Half of a disulfide bridge | 135088.4531 | Helicase C-terminal domain-containing protein OS=Komagataella phaffii (strain GS115 / ATCC 20864) OX=644223 GN=PAS\_chr1-1\_0318 PE=4 SV=1 |
| 240 | 42 | C4R6R3|C4R6R3\_KOMPG | 83.84 | 6 | 6 | 4.75e3 | 19 | 1 | 39 | Pyro-glu from E | 123198.0469 | Vacuolar membrane protein OS=Komagataella phaffii (strain GS115 / ATCC 20864) OX=644223 GN=PAS\_chr4\_0062 PE=4 SV=1 |
| 252 | 43 | C4R8B9|C4R8B9\_KOMPG | 81.03 | 11 | 11 | 1.87e3 | 19 | 1 | 47 |  | 64666.2266 | RING-type domain-containing protein OS=Komagataella phaffii (strain GS115 / ATCC 20864) OX=644223 GN=PAS\_chr4\_0979 PE=4 SV=1 |
| 266 | 44 | C4R1C2|C4R1C2\_KOMPG | 81.27 | 5 | 5 | 2.06e2 | 18 | 1 | 30 | Pyro-glu from E, Pyro-glu from Q | 155325.7812 | Key endocytic protein involved in a network of interactions with other endocytic proteins OS=Komagataella phaffii (strain GS115 / ATCC 20864) OX=644223 GN=PAS\_chr2-1\_0651 PE=4 SV=1 |
| 274 | 45 | C4QWP7|C4QWP7\_KOMPG | 76.07 | 5 | 5 | 1.55e3 | 18 | 2 | 32 |  | 136735.5938 | Coatomer subunit alpha OS=Komagataella phaffii (strain GS115 / ATCC 20864) OX=644223 GN=PAS\_chr1-1\_0294 PE=4 SV=1 |
| 279 | 46 | C4R3B8|C4R3B8\_KOMPG | 81.71 | 9 | 9 | 7.05e1 | 18 | 1 | 36 |  | 75308.8438 | Plasma membrane transporter for both urea and polyamines, expression is highly sensitive to nitrogen OS=Komagataella phaffii (strain GS115 / ATCC 20864) OX=644223 GN=PAS\_chr3\_0024 PE=3 SV=1 |
| 290 | 47 | C4R6W3|C4R6W3\_KOMPG | 79.02 | 7 | 7 | 5.82e2 | 18 | 1 | 34 |  | 109923.5391 | Aminopeptidase OS=Komagataella phaffii (strain GS115 / ATCC 20864) OX=644223 GN=PAS\_chr4\_0113 PE=3 SV=1 |
| 294 | 48 | C4R0S7|C4R0S7\_KOMPG | 80.13 | 9 | 9 | 9.35e2 | 18 | 1 | 39 | Pyro-glu from E | 82468.6172 | Chloride channel protein OS=Komagataella phaffii (strain GS115 / ATCC 20864) OX=644223 GN=PAS\_chr2-1\_0471 PE=3 SV=1 |
| 310 | 49 | C4QZT4|C4QZT4\_KOMPG | 81.81 | 9 | 9 | 6.74e2 | 18 | 3 | 32 | Pyro-glu from Q | 69977.3984 | WH2 domain-containing protein OS=Komagataella phaffii (strain GS115 / ATCC 20864) OX=644223 GN=PAS\_chr2-1\_0151 PE=4 SV=1 |
| 320 | 50 | C4QV05|C4QV05\_KOMPG | 73.16 | 11 | 11 | 2.34e2 | 17 | 1 | 30 | Pyro-glu from E | 72015.7891 | Allantoinase, converts allantoin to allantoate in the first step of allantoin degradation OS=Komagataella phaffii (strain GS115 / ATCC 20864) OX=644223 GN=PAS\_chr1-3\_0023 PE=4 SV=1 |
| 326 | 51 | C4R581|C4R581\_KOMPG | 71.97 | 6 | 6 | 4.29e2 | 17 | 1 | 27 | Pyro-glu from Q, Deamidation (NQ) | 111344.9766 | Zn(2)-C6 fungal-type domain-containing protein OS=Komagataella phaffii (strain GS115 / ATCC 20864) OX=644223 GN=PAS\_chr3\_0669 PE=4 SV=1 |
| 332 | 52 | C4QWL7|C4QWL7\_KOMPG | 77.65 | 3 | 3 | 0 | 17 | 1 | 29 | Pyro-glu from Q | 219862.0156 | RNA helicase related to Ski2p, involved in translation inhibition of non-poly(A) mRNAs OS=Komagataella phaffii (strain GS115 / ATCC 20864) OX=644223 GN=PAS\_chr1-1\_0269 PE=3 SV=1 |
| 348 | 53 | C4R0G3|C4R0G3\_KOMPG | 85.79 | 6 | 6 | 7.16e1 | 17 | 1 | 30 |  | 128012.6094 | Actin cytoskeleton-regulatory complex protein SLA1 OS=Komagataella phaffii (strain GS115 / ATCC 20864) OX=644223 GN=PAS\_chr2-1\_0366 PE=3 SV=1 |
| 377 | 54 | C4QV87|C4QV87\_KOMPG | 84.65 | 3 | 3 | 5.07e1 | 16 | 1 | 27 |  | 208883.7812 | 1-phosphatidylinositol 4-kinase OS=Komagataella phaffii (strain GS115 / ATCC 20864) OX=644223 GN=PAS\_chr1-3\_0100 PE=3 SV=1 |
| 382 | 55 | C4R9D9|C4R9D9\_KOMPG | 72.98 | 5 | 5 | 1.05e3 | 16 | 1 | 23 |  | 141953.0312 | Multidrug resistance protein OS=Komagataella phaffii (strain GS115 / ATCC 20864) OX=644223 GN=PAS\_c034\_0016 PE=3 SV=1 |
| 385 | 56 | C4R3M1|C4R3M1\_KOMPG | 76.38 | 9 | 9 | 1.01e2 | 16 | 1 | 30 |  | 80574.8203 | non-specific serine/threonine protein kinase OS=Komagataella phaffii (strain GS115 / ATCC 20864) OX=644223 GN=PAS\_chr3\_0127 PE=3 SV=1 |
| 395 | 57 | C4R3F4|C4R3F4\_KOMPG | 74.66 | 7 | 7 | 5.71e2 | 16 | 1 | 24 |  | 88067.3281 | RING-type E3 ubiquitin transferase OS=Komagataella phaffii (strain GS115 / ATCC 20864) OX=644223 GN=PAS\_chr3\_0061 PE=4 SV=1 |
| 401 | 58 | C4R6A0|C4R6A0\_KOMPG | 77.28 | 3 | 3 | 0 | 16 | 1 | 28 |  | 236864.625 | Glutamate synthase [NADH] OS=Komagataella phaffii (strain GS115 / ATCC 20864) OX=644223 GN=PAS\_chr3\_1024 PE=3 SV=1 |
| 404 | 59 | C4QY05|C4QY05\_KOMPG | 68.47 | 13 | 13 | 1.08e3 | 16 | 1 | 24 | Pyro-glu from Q | 39873.5664 | Zn(2)-C6 fungal-type domain-containing protein OS=Komagataella phaffii (strain GS115 / ATCC 20864) OX=644223 GN=PAS\_chr1-4\_0290 PE=4 SV=1 |
| 410 | 60 | C4R6N6|C4R6N6\_KOMPG | 75.85 | 4 | 4 | 0 | 16 | 1 | 21 | Oxidation (M) | 177158.625 | Helicase OS=Komagataella phaffii (strain GS115 / ATCC 20864) OX=644223 GN=PAS\_chr4\_0035 PE=4 SV=1 |
| 453 | 61 | C4QZJ2|C4QZJ2\_KOMPG | 76.78 | 5 | 5 | 3.06e3 | 15 | 1 | 28 | Pyro-glu from Q | 134341.5312 | chitin synthase OS=Komagataella phaffii (strain GS115 / ATCC 20864) OX=644223 GN=PAS\_chr2-1\_0065 PE=4 SV=1 |
| 491 | 62 | C4QV93|C4QV93\_KOMPG | 72.47 | 8 | 8 | 0 | 15 | 1 | 23 | Pyro-glu from E | 77868.3516 | non-specific serine/threonine protein kinase OS=Komagataella phaffii (strain GS115 / ATCC 20864) OX=644223 GN=PAS\_chr1-3\_0106 PE=4 SV=1 |
| 499 | 63 | C4R875|C4R875\_KOMPG | 74.17 | 6 | 6 | 0 | 15 | 1 | 24 |  | 101030.0625 | Zinc cluster transcriptional activator OS=Komagataella phaffii (strain GS115 / ATCC 20864) OX=644223 GN=PAS\_chr4\_0540 PE=4 SV=1 |
| 501 | 64 | C4QXL2|C4QXL2\_KOMPG | 75.58 | 6 | 6 | 1.45e2 | 15 | 2 | 24 | Pyro-glu from E, Pyro-glu from Q | 114705.25 | Mediator of RNA polymerase II transcription subunit 15 OS=Komagataella phaffii (strain GS115 / ATCC 20864) OX=644223 GN=PAS\_chr1-4\_0155 PE=3 SV=1 |
| 502 | 65 | C4QVD8|C4QVD8\_KOMPG | 71.61 | 6 | 6 | 4.86e1 | 15 | 1 | 21 |  | 122772.2656 | Nucleolar protein required for normal metabolism of the rRNA primary transcript OS=Komagataella phaffii (strain GS115 / ATCC 20864) OX=644223 GN=PAS\_chr1-3\_0151 PE=4 SV=1 |
| 504 | 66 | C4QV24|C4QV24\_KOMPG | 78.69 | 5 | 5 | 1.18e3 | 15 | 1 | 25 |  | 110924.8516 | Pheromone-regulated membrane protein 10 OS=Komagataella phaffii (strain GS115 / ATCC 20864) OX=644223 GN=PAS\_chr1-3\_0044 PE=3 SV=1 |
| 524 | 67 | C4QZS3|C4QZS3\_KOMPG | 73.9 | 8 | 8 | 7.3e3 | 14 | 2 | 34 |  | 74213.7188 | Endoplasmic reticulum chaperone BIP OS=Komagataella phaffii (strain GS115 / ATCC 20864) OX=644223 GN=PAS\_chr2-1\_0140 PE=3 SV=1 |
| 527 | 68 | C4R6W9|C4R6W9\_KOMPG | 82.85 | 5 | 5 | 1.91e2 | 14 | 2 | 27 |  | 114288.9453 | MMS19 nucleotide excision repair protein OS=Komagataella phaffii (strain GS115 / ATCC 20864) OX=644223 GN=PAS\_chr4\_0118 PE=3 SV=1 |
| 531 | 69 | C4QW18|C4QW18\_KOMPG | 76.69 | 6 | 6 | 1.66e3 | 14 | 1 | 27 | Deamidation (NQ) | 98187.7109 | Alpha/beta hydrolase fold-3 domain-containing protein OS=Komagataella phaffii (strain GS115 / ATCC 20864) OX=644223 GN=PAS\_chr1-1\_0471 PE=4 SV=1 |
| 532 | 70 | C4R4X3|C4R4X3\_KOMPG | 72.41 | 10 | 10 | 3.14e4 | 14 | 2 | 24 |  | 69323.4844 | Glutathione hydrolase OS=Komagataella phaffii (strain GS115 / ATCC 20864) OX=644223 GN=PAS\_chr3\_0561 PE=3 SV=1 |
| 543 | 71 | C4R5C0|C4R5C0\_KOMPG | 70.12 | 6 | 6 | 0 | 14 | 1 | 22 |  | 94920.5703 | Uncharacterized protein OS=Komagataella phaffii (strain GS115 / ATCC 20864) OX=644223 GN=PAS\_chr3\_0705 PE=4 SV=1 |
| 553 | 72 | C4R048|C4R048\_KOMPG | 76.32 | 5 | 5 | 0 | 14 | 1 | 20 |  | 122466.875 | Altered inheritance of mitochondria protein 3 OS=Komagataella phaffii (strain GS115 / ATCC 20864) OX=644223 GN=PAS\_chr2-1\_0260 PE=4 SV=1 |
| 556 | 73 | C4R0H5|C4R0H5\_KOMPG | 64.17 | 6 | 6 | 9.03e2 | 14 | 1 | 23 |  | 94220.4375 | Spindle pole body component OS=Komagataella phaffii (strain GS115 / ATCC 20864) OX=644223 GN=PAS\_chr2-1\_0377 PE=3 SV=1 |
| 557 | 74 | C4R922|C4R922\_KOMPG | 76.79 | 8 | 8 | 0 | 14 | 1 | 23 |  | 70651.9922 | Myo-inositol transporter with strong similarity to the minor myo-inositol transporter Itr2p OS=Komagataella phaffii (strain GS115 / ATCC 20864) OX=644223 GN=PAS\_chr4\_0828 PE=3 SV=1 |
| 567 | 75 | C4QY80|C4QY80\_KOMPG | 72.44 | 6 | 6 | 1.97e3 | 14 | 1 | 25 | Acetylation (Protein N-term) | 103653.5156 | DNA replication licensing factor MCM6 OS=Komagataella phaffii (strain GS115 / ATCC 20864) OX=644223 GN=PAS\_chr1-4\_0360 PE=3 SV=1 |
| 568 | 76 | C4R417|C4R417\_KOMPG | 71.78 | 5 | 5 | 1.55e2 | 14 | 1 | 30 | Pyro-glu from E | 127541.7266 | Condensin complex subunit 1 OS=Komagataella phaffii (strain GS115 / ATCC 20864) OX=644223 GN=PAS\_chr3\_0265 PE=3 SV=1 |
| 577 | 77 | C4R2Z6|C4R2Z6\_KOMPG | 67.49 | 13 | 13 | 5.25e2 | 14 | 1 | 60 | Pyro-glu from E | 32206.5 | NADPH-dependent 1-acyl dihydroxyacetone phosphate reductase OS=Komagataella phaffii (strain GS115 / ATCC 20864) OX=644223 GN=PAS\_chr2-2\_0063 PE=3 SV=1 |
| 587 | 78 | C4QZJ3|C4QZJ3\_KOMPG | 72.89 | 4 | 4 | 1.02e2 | 14 | 1 | 20 |  | 176684.0156 | Peripheral membrane protein with a role in endocytosis and vacuole integrity, interacts with Arl1p a OS=Komagataella phaffii (strain GS115 / ATCC 20864) OX=644223 GN=PAS\_chr2-1\_0066 PE=4 SV=1 |
| 591 | 79 | C4QVG3|C4QVG3\_KOMPG | 66.13 | 7 | 7 | 0 | 14 | 1 | 21 | Pyro-glu from E, Pyro-glu from Q | 91053.2891 | Ketopantoate reductase C-terminal domain-containing protein OS=Komagataella phaffii (strain GS115 / ATCC 20864) OX=644223 GN=PAS\_chr1-3\_0173 PE=4 SV=1 |
| 592 | 80 | C4R8U9|C4R8U9\_KOMPG | 67.22 | 7 | 7 | 0 | 14 | 1 | 31 |  | 66585.2578 | Protein required for transcription of rDNA by RNA polymerase I OS=Komagataella phaffii (strain GS115 / ATCC 20864) OX=644223 GN=PAS\_chr4\_0760 PE=3 SV=1 |
| 595 | 81 | C4R1Z6|C4R1Z6\_KOMPG | 71.74 | 5 | 5 | 2.42e2 | 14 | 1 | 19 | Pyro-glu from Q | 138172.5 | Essential nucleoporin OS=Komagataella phaffii (strain GS115 / ATCC 20864) OX=644223 GN=PAS\_chr2-2\_0385 PE=3 SV=1 |
| 636 | 82 | C4R001|C4R001\_KOMPG | 65.54 | 4 | 4 | 4.34e1 | 13 | 1 | 19 |  | 156049.1406 | DNA binding protein with similarity to the S. pombe Snt2 protein OS=Komagataella phaffii (strain GS115 / ATCC 20864) OX=644223 GN=PAS\_chr2-1\_0215 PE=4 SV=1 |
| 645 | 83 | C4R1R9|C4R1R9\_KOMPG | 67.98 | 9 | 9 | 1.75e3 | 13 | 2 | 27 | Deamidation (NQ) | 78333.9609 | Long chain fatty acyl-CoA synthetase with a preference for C12:0-C16:0 fatty acids OS=Komagataella phaffii (strain GS115 / ATCC 20864) OX=644223 GN=PAS\_chr2-1\_0785 PE=3 SV=1 |
| 651 | 84 | C4QYQ4|C4QYQ4\_KOMPG | 65.98 | 10 | 10 | 1.16e4 | 13 | 5 | 22 | Deamidation (NQ), Pyro-glu from Q | 50515.5664 | Regulatory protein MIG1 OS=Komagataella phaffii (strain GS115 / ATCC 20864) OX=644223 GN=PAS\_chr1-4\_0526 PE=3 SV=1 |
| 656 | 85 | C4R5R1|C4R5R1\_KOMPG | 70.28 | 5 | 5 | 3.83e3 | 13 | 1 | 18 | Pyro-glu from E | 114044.125 | 5'-3' exoribonuclease OS=Komagataella phaffii (strain GS115 / ATCC 20864) OX=644223 GN=PAS\_chr3\_0846 PE=1 SV=1 |
| 659 | 86 | C4R317|C4R317\_KOMPG | 73.51 | 8 | 8 | 3.73e2 | 13 | 1 | 30 | Pyro-glu from Q | 74718.2188 | Homeobox transcription factor OS=Komagataella phaffii (strain GS115 / ATCC 20864) OX=644223 GN=PAS\_chr2-2\_0043 PE=4 SV=1 |
| 663 | 87 | C4R360|PSD2\_KOMPG | 72.13 | 5 | 5 | 1.51e4 | 13 | 2 | 26 | Oxidation (M) | 116241.1875 | Phosphatidylserine decarboxylase proenzyme 2 OS=Komagataella phaffii (strain GS115 / ATCC 20864) OX=644223 GN=PSD2 PE=3 SV=1 |
| 665 | 88 | C4R123|C4R123\_KOMPG | 67.93 | 5 | 5 | 2.6e2 | 13 | 1 | 24 | Pyro-glu from E, Deamidation (NQ), Pyro-glu from Q | 116106.3828 | Protein phosphatase with specificity for serine, threonine, and tyrosine residues OS=Komagataella phaffii (strain GS115 / ATCC 20864) OX=644223 GN=PAS\_chr2-1\_0562 PE=4 SV=1 |
| 666 | 89 | C4R558|C4R558\_KOMPG | 76.01 | 3 | 3 | 3.85e3 | 13 | 1 | 28 |  | 196212.4531 | Phospholipase OS=Komagataella phaffii (strain GS115 / ATCC 20864) OX=644223 GN=PAS\_chr3\_0646 PE=3 SV=1 |
| 675 | 90 | C4R5L7|C4R5L7\_KOMPG | 75.66 | 5 | 5 | 1.66e4 | 13 | 2 | 20 |  | 125937.4531 | Carbamoyl phosphate synthase arginine-specific large chain OS=Komagataella phaffii (strain GS115 / ATCC 20864) OX=644223 GN=PAS\_chr3\_0799 PE=3 SV=1 |
| 676 | 91 | C4R2V7|C4R2V7\_KOMPG | 72.47 | 7 | 7 | 0 | 13 | 1 | 22 |  | 91653.3594 | Mitochondrial escape protein 2 OS=Komagataella phaffii (strain GS115 / ATCC 20864) OX=644223 GN=PAS\_chr2-2\_0096 PE=3 SV=1 |
| 681 | 92 | C4R9E3|C4R9E3\_KOMPG | 71.41 | 9 | 9 | 2.32e3 | 13 | 1 | 24 |  | 63758.1445 | Major facilitator superfamily OS=Komagataella phaffii (strain GS115 / ATCC 20864) OX=644223 GN=PAS\_c034\_0021 PE=3 SV=1 |
| 687 | 93 | C4R0L6|C4R0L6\_KOMPG | 77.31 | 5 | 5 | 2.48e2 | 13 | 1 | 25 |  | 116875.0781 | U2-snRNP associated splicing factor OS=Komagataella phaffii (strain GS115 / ATCC 20864) OX=644223 GN=PAS\_chr2-1\_0418 PE=3 SV=1 |
| 692 | 94 | C4R5N1|C4R5N1\_KOMPG | 69.06 | 7 | 7 | 1.17e3 | 13 | 1 | 27 | Pyro-glu from Q | 69517.6484 | Chromatin assembly factor 1 subunit A dimerization domain-containing protein OS=Komagataella phaffii (strain GS115 / ATCC 20864) OX=644223 GN=PAS\_chr3\_0814 PE=4 SV=1 |
| 709 | 95 | C4R5C9|C4R5C9\_KOMPG | 72.68 | 4 | 4 | 4.89e2 | 13 | 1 | 24 |  | 147300.1406 | Nuclear pore membrane glycoprotein OS=Komagataella phaffii (strain GS115 / ATCC 20864) OX=644223 GN=PAS\_chr3\_0715 PE=4 SV=1 |
| 718 | 96 | C4R7W6|C4R7W6\_KOMPG | 69.25 | 7 | 7 | 8.42e3 | 12 | 1 | 20 |  | 75212.8047 | Plasma membrane transporter for both urea and polyamines OS=Komagataella phaffii (strain GS115 / ATCC 20864) OX=644223 GN=PAS\_chr4\_0439 PE=3 SV=1 |
| 719 | 97 | C4R8H7|C4R8H7\_KOMPG | 71.59 | 7 | 7 | 8.39e2 | 12 | 1 | 24 |  | 63899.3828 | Cellulase OS=Komagataella phaffii (strain GS115 / ATCC 20864) OX=644223 GN=PAS\_chr4\_0643 PE=3 SV=1 |
| 721 | 98 | C4R5H3|C4R5H3\_KOMPG | 62.89 | 5 | 5 | 0 | 12 | 1 | 23 |  | 94248.3281 | Zinc-finger transcription factor OS=Komagataella phaffii (strain GS115 / ATCC 20864) OX=644223 GN=PAS\_chr3\_0759 PE=4 SV=1 |
| 741 | 99 | C4R075|C4R075\_KOMPG | 70.91 | 6 | 6 | 1.9e3 | 12 | 1 | 25 | Pyro-glu from E | 91327.1172 | Mitochondrial integral membrane protein involved in mitochondrial fusion and maintenanceof the mitoc OS=Komagataella phaffii (strain GS115 / ATCC 20864) OX=644223 GN=PAS\_chr2-1\_0285 PE=4 SV=1 |
| 747 | 100 | C4R3K9|C4R3K9\_KOMPG | 63.1 | 6 | 6 | 1.8e3 | 12 | 1 | 21 | Pyro-glu from Q | 82887.6484 | Transcriptional repressor and activator OS=Komagataella phaffii (strain GS115 / ATCC 20864) OX=644223 GN=PAS\_chr3\_0115 PE=3 SV=1 |
| 769 | 101 | C4QX93|C4QX93\_KOMPG | 67.08 | 6 | 6 | 2.47e2 | 12 | 1 | 26 |  | 95009.9219 | Uncharacterized protein OS=Komagataella phaffii (strain GS115 / ATCC 20864) OX=644223 GN=PAS\_chr1-4\_0035 PE=4 SV=1 |
| 781 | 102 | C4QYT0|C4QYT0\_KOMPG | 71.22 | 9 | 9 | 4.58e2 | 12 | 1 | 23 |  | 51680.0781 | Vacuolar proteinase B (YscB), a serine protease of the subtilisin family OS=Komagataella phaffii (strain GS115 / ATCC 20864) OX=644223 GN=PAS\_chr1-4\_0548 PE=3 SV=1 |
| 795 | 103 | C4QXV6|C4QXV6\_KOMPG | 75.85 | 8 | 8 | 9.7e2 | 12 | 4 | 20 | Pyro-glu from E, Deamidation (NQ), Pyro-glu from Q | 56958.1484 | Epsin-like protein involved in endocytosis and actin patch assembly OS=Komagataella phaffii (strain GS115 / ATCC 20864) OX=644223 GN=PAS\_chr1-4\_0244 PE=3 SV=1 |
| 801 | 104 | C4R3D3|C4R3D3\_KOMPG | 71.77 | 3 | 3 | 3.04e3 | 12 | 1 | 30 | Pyro-glu from E | 151887.8906 | non-specific serine/threonine protein kinase OS=Komagataella phaffii (strain GS115 / ATCC 20864) OX=644223 GN=PAS\_chr3\_0042 PE=4 SV=1 |
| 808 | 105 | C4R2E9|C4R2E9\_KOMPG | 73.97 | 8 | 8 | 2.69e2 | 12 | 1 | 21 |  | 60527.4375 | Uncharacterized protein OS=Komagataella phaffii (strain GS115 / ATCC 20864) OX=644223 GN=PAS\_chr2-2\_0479 PE=4 SV=1 |
| 812 | 106 | C4R675|C4R675\_KOMPG | 69.86 | 8 | 8 | 3.91e2 | 12 | 1 | 18 |  | 66393.4844 | Alpha-1,3-glucosyltransferase OS=Komagataella phaffii (strain GS115 / ATCC 20864) OX=644223 GN=PAS\_chr3\_0999 PE=3 SV=1 |
| 827 | 107 | C4R153|C4R153\_KOMPG | 59.35 | 7 | 7 | 4.7e1 | 11 | 1 | 22 | Pyro-glu from E | 68872.2656 | RING-type domain-containing protein OS=Komagataella phaffii (strain GS115 / ATCC 20864) OX=644223 GN=PAS\_chr2-1\_0590 PE=4 SV=1 |
| 843 | 108 | C4R7I8|C4R7I8\_KOMPG | 65.4 | 5 | 5 | 8.86e1 | 11 | 1 | 16 |  | 92752.6875 | Uncharacterized protein OS=Komagataella phaffii (strain GS115 / ATCC 20864) OX=644223 GN=PAS\_chr4\_0320 PE=4 SV=1 |
| 849 | 109 | C4QZ46|C4QZ46\_KOMPG | 67.82 | 3 | 3 | 1.58e2 | 11 | 1 | 18 |  | 176138.375 | E3 ubiquitin-protein ligase listerin OS=Komagataella phaffii (strain GS115 / ATCC 20864) OX=644223 GN=PAS\_c121\_0007 PE=3 SV=1 |
| 874 | 110 | C4R4T4|C4R4T4\_KOMPG | 63.11 | 10 | 10 | 1.16e3 | 11 | 1 | 20 | Pyro-glu from Q | 39174.9648 | RING-type E3 ubiquitin transferase OS=Komagataella phaffii (strain GS115 / ATCC 20864) OX=644223 GN=PAS\_chr3\_0521 PE=3 SV=1 |
| 882 | 111 | C4QZI1|C4QZI1\_KOMPG | 76.2 | 8 | 8 | 1.2e3 | 11 | 1 | 34 | Pyro-glu from E | 58691.9609 | Hexose transporter, expressed at low levels and expression is repressed by glucose OS=Komagataella phaffii (strain GS115 / ATCC 20864) OX=644223 GN=PAS\_chr2-1\_0054 PE=3 SV=1 |
| 885 | 112 | C4QW82|C4QW82\_KOMPG | 60.65 | 9 | 9 | 0 | 11 | 1 | 20 | Pyro-glu from E | 45670.6406 | Uncharacterized protein OS=Komagataella phaffii (strain GS115 / ATCC 20864) OX=644223 GN=PAS\_chr1-1\_0141 PE=4 SV=1 |
| 893 | 113 | C4R150|C4R150\_KOMPG | 71.05 | 6 | 6 | 2.18e4 | 11 | 2 | 23 | Deamidation (NQ), Pyro-glu from Q | 66621.3828 | Transcription initiation factor TFIID subunit 12 OS=Komagataella phaffii (strain GS115 / ATCC 20864) OX=644223 GN=PAS\_chr2-1\_0588 PE=1 SV=1 |
| 902 | 114 | C4R701|C4R701\_KOMPG | 75.27 | 9 | 9 | 2.96e3 | 11 | 1 | 18 |  | 43486.2148 | Uncharacterized protein OS=Komagataella phaffii (strain GS115 / ATCC 20864) OX=644223 GN=PAS\_chr4\_0151 PE=4 SV=1 |
| 922 | 115 | C4R603|C4R603\_KOMPG | 69.74 | 3 | 3 | 2.7e2 | 11 | 1 | 14 |  | 166024.6719 | Protein required for beta-1,6 glucan biosynthesis OS=Komagataella phaffii (strain GS115 / ATCC 20864) OX=644223 GN=PAS\_chr3\_0929 PE=4 SV=1 |
| 938 | 116 | C4QX36|C4QX36\_KOMPG | 68.02 | 10 | 10 | 2.89e2 | 11 | 1 | 21 |  | 44310.1094 | Ketol-acid reductoisomerase, mitochondrial OS=Komagataella phaffii (strain GS115 / ATCC 20864) OX=644223 GN=PAS\_chr1-1\_0432 PE=3 SV=1 |
| 939 | 117 | C4QVD7|C4QVD7\_KOMPG | 63.79 | 2 | 2 | 2.89e1 | 11 | 1 | 16 |  | 207981.9375 | E3 ubiquitin-protein ligase OS=Komagataella phaffii (strain GS115 / ATCC 20864) OX=644223 GN=PAS\_chr1-3\_0150 PE=3 SV=1 |
| 942 | 118 | C4R5W4|C4R5W4\_KOMPG | 68.09 | 9 | 9 | 1.02e3 | 11 | 1 | 18 | Deamidation (NQ), Pyro-glu from Q | 58140.2578 | Transcription factor OS=Komagataella phaffii (strain GS115 / ATCC 20864) OX=644223 GN=PAS\_chr3\_1235 PE=4 SV=1 |
| 955 | 119 | C4R8P7|C4R8P7\_KOMPG | 62.69 | 6 | 6 | 8.18e3 | 11 | 3 | 18 | Pyro-glu from Q, Deamidation (NQ) | 88860.5781 | Uncharacterized protein OS=Komagataella phaffii (strain GS115 / ATCC 20864) OX=644223 GN=PAS\_chr4\_0711 PE=4 SV=1 |
| 963 | 120 | C4R7K8|C4R7K8\_KOMPG | 69.09 | 4 | 4 | 0 | 11 | 2 | 15 |  | 106199.9219 | Zn(2)-C6 fungal-type domain-containing protein OS=Komagataella phaffii (strain GS115 / ATCC 20864) OX=644223 GN=PAS\_chr4\_0340 PE=4 SV=1 |
| 975 | 121 | C4R3M7|C4R3M7\_KOMPG | 69.3 | 6 | 6 | 2.75e2 | 11 | 1 | 16 |  | 81611.3281 | Carbohydrate kinase PfkB domain-containing protein OS=Komagataella phaffii (strain GS115 / ATCC 20864) OX=644223 GN=PAS\_chr3\_0134 PE=3 SV=1 |
| 982 | 122 | C4QVW8|C4QVW8\_KOMPG | 66.17 | 11 | 11 | 2.72e3 | 11 | 1 | 23 | Deamidation (NQ) | 43423.7031 | Uncharacterized protein OS=Komagataella phaffii (strain GS115 / ATCC 20864) OX=644223 GN=PAS\_chr1-1\_0033 PE=4 SV=1 |
| 984 | 123 | C4QXS3|C4QXS3\_KOMPG | 62.42 | 7 | 7 | 1.74e3 | 11 | 1 | 23 |  | 71421.6484 | Signal recognition particle subunit SRP72 OS=Komagataella phaffii (strain GS115 / ATCC 20864) OX=644223 GN=PAS\_chr1-4\_0210 PE=3 SV=1 |
| 989 | 124 | C4QZ37|C4QZ37\_KOMPG | 79.66 | 4 | 4 | 9.87e2 | 10 | 1 | 19 |  | 94275.4453 | Mitochondrial respiratory chain complexes assembly protein RCA1 OS=Komagataella phaffii (strain GS115 / ATCC 20864) OX=644223 GN=PAS\_chr1-4\_0649 PE=3 SV=1 |
| 1017 | 125 | C4R2L2|C4R2L2\_KOMPG | 61.82 | 5 | 5 | 2.4e4 | 10 | 1 | 18 | Pyro-glu from Q, Deamidation (NQ) | 126094.875 | ARID domain-containing protein OS=Komagataella phaffii (strain GS115 / ATCC 20864) OX=644223 GN=PAS\_chr2-2\_0471 PE=4 SV=1 |
| 1041 | 126 | C4QXQ4|C4QXQ4\_KOMPG | 67.8 | 7 | 7 | 1.31e2 | 10 | 1 | 15 | Deamidation (NQ) | 63729.1914 | Polyamine transporter that recognizes spermine, putrescine, and spermidine OS=Komagataella phaffii (strain GS115 / ATCC 20864) OX=644223 GN=PAS\_chr1-4\_0194 PE=4 SV=1 |
| 1126 | 127 | C4QXW2|C4QXW2\_KOMPG | 58.41 | 9 | 9 | 3.92e0 | 10 | 1 | 20 | Pyro-glu from E | 47928.6914 | Peptidase S8/S53 domain-containing protein OS=Komagataella phaffii (strain GS115 / ATCC 20864) OX=644223 GN=PAS\_chr1-4\_0251 PE=3 SV=1 |
| 1131 | 128 | C4R0Y9|C4R0Y9\_KOMPG | 57.9 | 10 | 10 | 5.42e3 | 10 | 1 | 22 |  | 38332.5977 | Ornithine transporter of the mitochondrial inner membrane OS=Komagataella phaffii (strain GS115 / ATCC 20864) OX=644223 GN=PAS\_chr2-1\_0530 PE=3 SV=1 |
| 1133 | 129 | C4R192|VPS10\_KOMPG | 62.16 | 3 | 3 | 0 | 10 | 1 | 17 | Deamidation (NQ), Half of a disulfide bridge | 173835.0312 | Vacuolar protein sorting/targeting protein 10 OS=Komagataella phaffii (strain GS115 / ATCC 20864) OX=644223 GN=VPS10 PE=3 SV=1 |
| 1134 | 130 | C4R460|C4R460\_KOMPG | 59.49 | 7 | 7 | 2.9e2 | 10 | 2 | 16 |  | 67625.7969 | 1,3-beta-glucanosyltransferase OS=Komagataella phaffii (strain GS115 / ATCC 20864) OX=644223 GN=PAS\_chr3\_0306 PE=3 SV=1 |
| 1137 | 131 | C4QZH8|C4QZH8\_KOMPG | 61.53 | 10 | 10 | 4.91e1 | 10 | 1 | 14 |  | 41020.1875 | Inositol oxygenase OS=Komagataella phaffii (strain GS115 / ATCC 20864) OX=644223 GN=PAS\_chr2-1\_0051 PE=3 SV=1 |
| 1138 | 132 | C4R043|C4R043\_KOMPG | 67.95 | 5 | 5 | 0 | 10 | 1 | 14 | Pyro-glu from Q | 79626.4375 | LDB19 N-terminal domain-containing protein OS=Komagataella phaffii (strain GS115 / ATCC 20864) OX=644223 GN=PAS\_chr2-1\_0255 PE=4 SV=1 |
| 1139 | 133 | C4QZF0|C4QZF0\_KOMPG | 70.13 | 8 | 8 | 0 | 10 | 1 | 30 |  | 51187.3047 | Mitochondrial metal transporter OS=Komagataella phaffii (strain GS115 / ATCC 20864) OX=644223 GN=PAS\_chr2-1\_0806 PE=3 SV=1 |
| 1179 | 134 | C4QYA1|C4QYA1\_KOMPG | 59.73 | 8 | 8 | 3.9e2 | 9 | 1 | 18 |  | 44302.4453 | Penta-EF-hand protein OS=Komagataella phaffii (strain GS115 / ATCC 20864) OX=644223 GN=PAS\_chr1-4\_0379 PE=4 SV=1 |
| 1198 | 135 | C4R609|C4R609\_KOMPG | 64.04 | 9 | 9 | 5.48e3 | 9 | 3 | 15 | Acetylation (Protein N-term) | 62840.2422 | Rhomboid-type serine protease OS=Komagataella phaffii (strain GS115 / ATCC 20864) OX=644223 GN=PAS\_chr3\_0934 PE=3 SV=1 |
| 1205 | 136 | C4QYI7|C4QYI7\_KOMPG | 59.5 | 6 | 6 | 0 | 9 | 1 | 11 |  | 65925.5391 | dihydroxy-acid dehydratase OS=Komagataella phaffii (strain GS115 / ATCC 20864) OX=644223 GN=PAS\_chr1-4\_0458 PE=3 SV=1 |
| 1218 | 137 | C4QXF3|C4QXF3\_KOMPG | 71.34 | 5 | 5 | 0 | 9 | 1 | 19 |  | 86222.2188 | Plasma membrane Mg(2+) transporter, expression and turnover are regulated by Mg(2+) concentration OS=Komagataella phaffii (strain GS115 / ATCC 20864) OX=644223 GN=PAS\_chr1-4\_0096 PE=3 SV=1 |
| 1239 | 138 | C4QWZ3|C4QWZ3\_KOMPG | 62.41 | 8 | 8 | 0 | 9 | 2 | 17 |  | 52330.3828 | argininosuccinate lyase OS=Komagataella phaffii (strain GS115 / ATCC 20864) OX=644223 GN=PAS\_chr1-1\_0389 PE=3 SV=1 |
| 1260 | 139 | C4QWP4|C4QWP4\_KOMPG | 49.08 | 8 | 8 | 5.4e2 | 9 | 1 | 15 | Pyro-glu from E, Pyro-glu from Q | 46693.7773 | Protein required for ribosomal large subunit maturation, functionally redundant with Ssf1p OS=Komagataella phaffii (strain GS115 / ATCC 20864) OX=644223 GN=PAS\_chr1-1\_0291 PE=4 SV=1 |
| 1271 | 140 | C4QWH8|C4QWH8\_KOMPG | 69.36 | 4 | 4 | 2e3 | 9 | 1 | 13 | Oxidation (M) | 104136.25 | Subunit of the nuclear pore complex (NPC) that is localized to both sides of the pore OS=Komagataella phaffii (strain GS115 / ATCC 20864) OX=644223 GN=PAS\_chr1-1\_0232 PE=3 SV=1 |
| 1299 | 141 | C4R218|C4R218\_KOMPG | 55.5 | 4 | 4 | 1.42e3 | 9 | 1 | 15 |  | 109351.8906 | DNA-binding protein of the mitochondria involved in repair of mitochondrial DNA OS=Komagataella phaffii (strain GS115 / ATCC 20864) OX=644223 GN=PAS\_chr2-2\_0364 PE=3 SV=1 |
| 1347 | 142 | C4QVY8|C4QVY8\_KOMPG | 58.16 | 3 | 3 | 0 | 9 | 1 | 12 |  | 121427.0078 | Translation initiation factor eIF4G, subunit of the mRNA cap-binding protein complex (eIF4F) OS=Komagataella phaffii (strain GS115 / ATCC 20864) OX=644223 GN=PAS\_chr1-1\_0053 PE=3 SV=1 |
| 1353 | 143 | C4R0K4|C4R0K4\_KOMPG | 56.45 | 6 | 6 | 1.35e3 | 9 | 1 | 18 | Pyro-glu from E, Pyro-glu from Q | 60779.582 | Serine/threonine-protein phosphatase OS=Komagataella phaffii (strain GS115 / ATCC 20864) OX=644223 GN=PAS\_chr2-1\_0407 PE=3 SV=1 |
| 1353 | 144 | C4R8V1|C4R8V1\_KOMPG | 20.46 | 2 | 2 | 1.35e3 | 1 | 1 | 2 |  | 34817.8477 | Ubiquitin OS=Komagataella phaffii (strain GS115 / ATCC 20864) OX=644223 GN=PAS\_chr4\_0762 PE=3 SV=1 |
| 1353 | 145 | C4R0U2|C4R0U2\_KOMPG | 20.46 | 4 | 4 | 1.35e3 | 1 | 1 | 2 |  | 14568.0273 | Fusion protein, identical to Rpl40Bp OS=Komagataella phaffii (strain GS115 / ATCC 20864) OX=644223 GN=PAS\_chr2-1\_0486 PE=3 SV=1 |
| 1353 | 146 | C4R5D4|C4R5D4\_KOMPG | 20.46 | 3 | 3 | 1.35e3 | 1 | 1 | 2 |  | 17185 | Fusion protein that is cleaved to yield a ribosomal protein of the small (40S) subunit and ubiquitin OS=Komagataella phaffii (strain GS115 / ATCC 20864) OX=644223 GN=PAS\_chr3\_0722 PE=3 SV=1 |
| 1359 | 147 | C4QXJ4|C4QXJ4\_KOMPG | 63.64 | 9 | 9 | 1.22e3 | 9 | 1 | 19 |  | 44461.4375 | Widely conserved NADPH oxidoreductase containing flavin mononucleotide (FMN) OS=Komagataella phaffii (strain GS115 / ATCC 20864) OX=644223 GN=PAS\_chr1-4\_0139 PE=4 SV=1 |
| 1372 | 148 | C4R610|C4R610\_KOMPG | 65.33 | 4 | 4 | 5.51e2 | 9 | 1 | 20 |  | 109586.4453 | ATP-dependent permease of the ABC transporter family of proteins OS=Komagataella phaffii (strain GS115 / ATCC 20864) OX=644223 GN=PAS\_chr3\_0935 PE=4 SV=1 |
| 1413 | 149 | C4QZK5|C4QZK5\_KOMPG | 54.49 | 6 | 6 | 1.02e4 | 8 | 1 | 16 | Deamidation (NQ) | 78309.4453 | Plasma membrane G protein coupled receptor (GPCR) that interacts with the heterotrimeric G protein a OS=Komagataella phaffii (strain GS115 / ATCC 20864) OX=644223 GN=PAS\_chr2-1\_0078 PE=4 SV=1 |
| 1417 | 150 | C4QXJ6|C4QXJ6\_KOMPG | 60.99 | 6 | 6 | 3.46e2 | 8 | 1 | 11 |  | 61644.9375 | Protein with similarity to hydroxymethylpyrimidine phosphate kinases OS=Komagataella phaffii (strain GS115 / ATCC 20864) OX=644223 GN=PAS\_chr1-4\_0141 PE=4 SV=1 |
| 1444 | 151 | C4R0U1|C4R0U1\_KOMPG | 59.7 | 9 | 9 | 1.06e3 | 8 | 1 | 19 | Pyro-glu from E | 42831.8789 | FIST domain-containing protein OS=Komagataella phaffii (strain GS115 / ATCC 20864) OX=644223 GN=PAS\_chr2-1\_0485 PE=4 SV=1 |
| 1469 | 152 | C4R8H2|C4R8H2\_KOMPG | 60.42 | 7 | 7 | 5.8e1 | 8 | 1 | 11 |  | 49111.9023 | RING-type domain-containing protein OS=Komagataella phaffii (strain GS115 / ATCC 20864) OX=644223 GN=PAS\_chr4\_0637 PE=4 SV=1 |
| 1473 | 153 | C4QXR9|C4QXR9\_KOMPG | 59.36 | 4 | 4 | 0 | 8 | 1 | 10 | Deamidation (NQ) | 109961.4062 | Pre-mRNA-splicing factor ATP-dependent RNA helicase PRP16 OS=Komagataella phaffii (strain GS115 / ATCC 20864) OX=644223 GN=PAS\_chr1-4\_0661 PE=3 SV=1 |
| 1501 | 154 | C4R9E1|C4R9E1\_KOMPG | 58.65 | 5 | 5 | 2.36e3 | 8 | 1 | 18 |  | 63015.7852 | Basic amino-acid permease OS=Komagataella phaffii (strain GS115 / ATCC 20864) OX=644223 GN=PAS\_c034\_0019 PE=3 SV=1 |
| 1521 | 155 | C4QXD7|C4QXD7\_KOMPG | 58.33 | 6 | 6 | 8.53e2 | 8 | 2 | 14 | Deamidation (NQ) | 63364.8906 | Tyrosine-protein phosphatase CDC14 OS=Komagataella phaffii (strain GS115 / ATCC 20864) OX=644223 GN=PAS\_chr1-4\_0081 PE=3 SV=1 |
| 1575 | 156 | C4QYA0|C4QYA0\_KOMPG | 63.24 | 5 | 5 | 0 | 8 | 1 | 15 |  | 73961.8828 | Uncharacterized protein OS=Komagataella phaffii (strain GS115 / ATCC 20864) OX=644223 GN=PAS\_chr1-4\_0378 PE=4 SV=1 |
| 1581 | 157 | C4QW10|C4QW10\_KOMPG | 60.08 | 5 | 5 | 3.04e3 | 8 | 1 | 20 | Deamidation (NQ), Pyro-glu from Q | 70020.9062 | RNA polymerase II degradation factor 1 OS=Komagataella phaffii (strain GS115 / ATCC 20864) OX=644223 GN=PAS\_chr1-1\_0470 PE=3 SV=1 |
| 1596 | 158 | C4R6L5|C4R6L5\_KOMPG | 53.57 | 6 | 6 | 2.79e3 | 8 | 1 | 14 |  | 60728.7773 | Maltose permease, high-affinity maltose transporter (Alpha-glucoside transporter) OS=Komagataella phaffii (strain GS115 / ATCC 20864) OX=644223 GN=PAS\_chr4\_0011 PE=3 SV=1 |
| 1618 | 159 | C4R301|C4R301\_KOMPG | 54.77 | 5 | 5 | 0 | 8 | 1 | 9 |  | 68142.7578 | Eukaryotic translation initiation factor 2A OS=Komagataella phaffii (strain GS115 / ATCC 20864) OX=644223 GN=PAS\_chr2-2\_0058 PE=3 SV=1 |
| 1626 | 160 | C4R953|C4R953\_KOMPG | 55.03 | 5 | 5 | 4.77e2 | 8 | 1 | 12 | Deamidation (NQ) | 74606.2734 | Mitochondrial inner membrane half-type ATP-binding cassette (ABC) transporter OS=Komagataella phaffii (strain GS115 / ATCC 20864) OX=644223 GN=PAS\_chr4\_0859 PE=4 SV=1 |
| 1636 | 161 | C4R3L9|C4R3L9\_KOMPG | 63.3 | 6 | 6 | 9.26e2 | 8 | 1 | 14 |  | 56193.8711 | Major facilitator superfamily (MFS) profile domain-containing protein OS=Komagataella phaffii (strain GS115 / ATCC 20864) OX=644223 GN=PAS\_chr3\_0125 PE=4 SV=1 |
| 1661 | 162 | C4R4Q0|C4R4Q0\_KOMPG | 58.79 | 3 | 3 | 3.96e3 | 8 | 1 | 16 | Pyro-glu from E | 129302.3281 | Bud-specific protein with a potential role in membrane trafficking OS=Komagataella phaffii (strain GS115 / ATCC 20864) OX=644223 GN=PAS\_chr3\_0488 PE=4 SV=1 |
| 1723 | 163 | C4R6V5|C4R6V5\_KOMPG | 60.43 | 7 | 7 | 1.54e3 | 7 | 1 | 14 |  | 39327.5781 | C3H1-type domain-containing protein OS=Komagataella phaffii (strain GS115 / ATCC 20864) OX=644223 GN=PAS\_chr4\_0104 PE=4 SV=1 |
| 1734 | 164 | C4QXN6|C4QXN6\_KOMPG | 49.64 | 4 | 4 | 0 | 7 | 1 | 11 |  | 79359.2266 | DNA 3'-5' helicase OS=Komagataella phaffii (strain GS115 / ATCC 20864) OX=644223 GN=PAS\_chr1-4\_0175 PE=3 SV=1 |
| 1746 | 165 | C4R899|C4R899\_KOMPG | 50.36 | 9 | 9 | 7.62e1 | 7 | 1 | 10 | Oxidation (M) | 32246.3242 | Subunit of the exosome, which is an essential complex present in both nucleus and cytoplasm OS=Komagataella phaffii (strain GS115 / ATCC 20864) OX=644223 GN=PAS\_chr4\_0563 PE=4 SV=1 |
| 1749 | 166 | C4R614|C4R614\_KOMPG | 61.18 | 5 | 5 | 2.49e3 | 7 | 1 | 17 |  | 72370.5391 | Ser-Thr protein kinase, member (With Ark1p and Prk1p) of the Ark kinase family OS=Komagataella phaffii (strain GS115 / ATCC 20864) OX=644223 GN=PAS\_chr3\_0940 PE=4 SV=1 |
| 1798 | 167 | C4QVI1|C4QVI1\_KOMPG | 60.05 | 6 | 6 | 0 | 7 | 1 | 14 |  | 57008.332 | GMP synthase [glutamine-hydrolyzing] OS=Komagataella phaffii (strain GS115 / ATCC 20864) OX=644223 GN=PAS\_chr1-3\_0191 PE=3 SV=1 |
| 1806 | 168 | C4QX22|C4QX22\_KOMPG | 56.84 | 11 | 11 | 2.35e3 | 7 | 1 | 9 |  | 30092.5156 | Transmembrane protein involved in export of ammonia OS=Komagataella phaffii (strain GS115 / ATCC 20864) OX=644223 GN=PAS\_chr1-1\_0417 PE=3 SV=1 |
| 1816 | 169 | C4R6X4|C4R6X4\_KOMPG | 60.48 | 5 | 5 | 0 | 7 | 1 | 9 |  | 54157.9883 | Probable transporter, member of the Ca2+-binding subfamily of the mitochondrial carrier family OS=Komagataella phaffii (strain GS115 / ATCC 20864) OX=644223 GN=PAS\_chr4\_0122 PE=3 SV=1 |
| 1834 | 170 | C4R0F2|C4R0F2\_KOMPG | 60.58 | 8 | 8 | 3.85e3 | 7 | 1 | 10 | Pyro-glu from Q | 38077.8789 | Chromatin modification-related protein EAF3 OS=Komagataella phaffii (strain GS115 / ATCC 20864) OX=644223 GN=PAS\_chr2-1\_0356 PE=3 SV=1 |
| 1923 | 171 | C4R1V8|C4R1V8\_KOMPG | 60.64 | 8 | 8 | 4.62e2 | 7 | 1 | 11 |  | 45185.6484 | GPI transamidase subunit OS=Komagataella phaffii (strain GS115 / ATCC 20864) OX=644223 GN=PAS\_chr2-2\_0427 PE=3 SV=1 |
| 1966 | 172 | C4QVB3|C4QVB3\_KOMPG | 64.17 | 5 | 5 | 1.42e3 | 7 | 1 | 14 | Pyro-glu from Q | 62617.2539 | Transcriptional activator of genes regulated by nitrogen catabolite repression (NCR) OS=Komagataella phaffii (strain GS115 / ATCC 20864) OX=644223 GN=PAS\_chr1-3\_0126 PE=4 SV=1 |
| 1997 | 173 | C4QZX1|C4QZX1\_KOMPG | 53.41 | 8 | 8 | 0 | 6 | 1 | 6 | Pyro-glu from E | 27577.623 | Uncharacterized protein OS=Komagataella phaffii (strain GS115 / ATCC 20864) OX=644223 GN=PAS\_chr2-1\_0187 PE=4 SV=1 |
| 2002 | 174 | C4R9D6|C4R9D6\_KOMPG | 50.95 | 12 | 12 | 1.35e3 | 6 | 1 | 8 |  | 21387.666 | superoxide dismutase OS=Komagataella phaffii (strain GS115 / ATCC 20864) OX=644223 GN=PAS\_c034\_0013 PE=3 SV=1 |
| 2009 | 175 | C4R8A6|C4R8A6\_KOMPG | 48 | 5 | 5 | 1.83e2 | 6 | 1 | 10 |  | 59290.2422 | Glycerol proton symporter of the plasma membrane, subject to glucose-induced inactivation OS=Komagataella phaffii (strain GS115 / ATCC 20864) OX=644223 GN=PAS\_chr4\_0571 PE=3 SV=1 |
| 2026 | 176 | C4QWG1|C4QWG1\_KOMPG | 52.21 | 7 | 7 | 1.32e3 | 6 | 1 | 9 | Pyro-glu from Q | 29709.5488 | Uncharacterized protein OS=Komagataella phaffii (strain GS115 / ATCC 20864) OX=644223 GN=PAS\_chr1-1\_0485 PE=4 SV=1 |
| 2028 | 177 | C4R4D4|C4R4D4\_KOMPG | 54.21 | 4 | 4 | 1.68e3 | 6 | 1 | 13 |  | 74175.6406 | glycine--tRNA ligase OS=Komagataella phaffii (strain GS115 / ATCC 20864) OX=644223 GN=PAS\_chr3\_0376 PE=3 SV=1 |
| 2033 | 178 | C4R1H4|C4R1H4\_KOMPG | 58.33 | 3 | 3 | 1.55e4 | 6 | 1 | 17 |  | 82512.1562 | Integral plasma membrane protein required for axial budding in haploid cells OS=Komagataella phaffii (strain GS115 / ATCC 20864) OX=644223 GN=PAS\_chr2-1\_0700 PE=4 SV=1 |
| 2041 | 179 | C4R8S1|C4R8S1\_KOMPG | 53.09 | 4 | 4 | 4.44e2 | 6 | 1 | 15 | Pyro-glu from E | 70232.2109 | Succinate dehydrogenase [ubiquinone] flavoprotein subunit, mitochondrial OS=Komagataella phaffii (strain GS115 / ATCC 20864) OX=644223 GN=PAS\_chr4\_0733 PE=3 SV=1 |
| 2043 | 180 | C4QXH1|C4QXH1\_KOMPG | 56.01 | 9 | 9 | 1.15e2 | 6 | 1 | 16 | Half of a disulfide bridge | 32661.5195 | 18S rRNA aminocarboxypropyltransferase OS=Komagataella phaffii (strain GS115 / ATCC 20864) OX=644223 GN=TSR3 PE=3 SV=1 |
| 2048 | 181 | C4QVP8|C4QVP8\_KOMPG | 50.54 | 10 | 10 | 4.72e3 | 6 | 1 | 13 | Pyro-glu from E, Half of a disulfide bridge | 31721.0703 | 3-methyl-adenine DNA glycosylase involved in protecting DNA against alkylating agents OS=Komagataella phaffii (strain GS115 / ATCC 20864) OX=644223 GN=PAS\_chr1-3\_0257 PE=4 SV=1 |
| 2084 | 182 | C4QYF7|C4QYF7\_KOMPG | 45.25 | 5 | 5 | 1.57e2 | 6 | 1 | 7 |  | 45090.3867 | Metacaspase-1 OS=Komagataella phaffii (strain GS115 / ATCC 20864) OX=644223 GN=PAS\_chr1-4\_0430 PE=3 SV=1 |
| 2142 | 183 | C4QWA7|C4QWA7\_KOMPG | 57.18 | 6 | 6 | 3.76e3 | 6 | 1 | 17 |  | 45915.7539 | BZIP domain-containing protein OS=Komagataella phaffii (strain GS115 / ATCC 20864) OX=644223 GN=PAS\_chr1-1\_0165 PE=3 SV=1 |
| 2171 | 184 | C4R801|C4R801\_KOMPG | 47.92 | 11 | 11 | 0 | 6 | 1 | 6 |  | 25443.3691 | Thioester reductase (TE) domain-containing protein OS=Komagataella phaffii (strain GS115 / ATCC 20864) OX=644223 GN=PAS\_chr4\_0965 PE=4 SV=1 |
| 2182 | 185 | C4R783|C4R783\_KOMPG | 50.3 | 4 | 4 | 2.47e2 | 6 | 1 | 8 |  | 73033.7031 | Acetolactate synthase OS=Komagataella phaffii (strain GS115 / ATCC 20864) OX=644223 GN=PAS\_chr4\_0228 PE=3 SV=1 |
| 2193 | 186 | C4R782|C4R782\_KOMPG | 49.82 | 3 | 3 | 1.26e3 | 6 | 1 | 10 |  | 85353.3672 | Nucleolar complex protein 2 OS=Komagataella phaffii (strain GS115 / ATCC 20864) OX=644223 GN=PAS\_chr4\_0930 PE=3 SV=1 |
| 2232 | 187 | C4QWV6|C4QWV6\_KOMPG | 59.22 | 4 | 4 | 3.93e2 | 6 | 1 | 15 | Oxidation (M) | 58612.375 | Autophagy-related protein OS=Komagataella phaffii (strain GS115 / ATCC 20864) OX=644223 GN=PAS\_chr1-1\_0353 PE=3 SV=1 |
| 2264 | 188 | C4QYR1|C4QYR1\_KOMPG | 54.66 | 5 | 5 | 1.37e3 | 6 | 1 | 7 |  | 51755.1172 | Phosphatidylinositol transfer protein OS=Komagataella phaffii (strain GS115 / ATCC 20864) OX=644223 GN=PAS\_chr1-4\_0532 PE=4 SV=1 |
| 2272 | 189 | C4R1W6|C4R1W6\_KOMPG | 51.37 | 4 | 4 | 9.75e3 | 6 | 2 | 9 |  | 58301.3906 | Phosphatidyl synthase OS=Komagataella phaffii (strain GS115 / ATCC 20864) OX=644223 GN=PAS\_chr2-2\_0419 PE=4 SV=1 |
| 2392 | 190 | C4R7T5|C4R7T5\_KOMPG | 49.04 | 4 | 4 | 2.46e2 | 5 | 1 | 6 |  | 54798.3633 | Protein SIP5 OS=Komagataella phaffii (strain GS115 / ATCC 20864) OX=644223 GN=PAS\_chr4\_0411 PE=3 SV=1 |
| 2450 | 191 | C4R6F1|C4R6F1\_KOMPG | 51.74 | 16 | 16 | 1.72e3 | 5 | 1 | 9 | Acetylation (Protein N-term) | 13001.9307 | Extracellular membrane protein CFEM domain-containing protein OS=Komagataella phaffii (strain GS115 / ATCC 20864) OX=644223 GN=PAS\_chr3\_1074 PE=4 SV=1 |
| 2512 | 192 | C4QZ32|C4QZ32\_KOMPG | 50.82 | 6 | 6 | 0 | 5 | 1 | 9 |  | 39381.1094 | Uncharacterized protein OS=Komagataella phaffii (strain GS115 / ATCC 20864) OX=644223 GN=PAS\_chr1-4\_0701 PE=4 SV=1 |
| 2552 | 193 | C4R7H9|C4R7H9\_KOMPG | 48.98 | 3 | 3 | 0 | 5 | 1 | 20 |  | 84862.3125 | non-specific serine/threonine protein kinase OS=Komagataella phaffii (strain GS115 / ATCC 20864) OX=644223 GN=PAS\_chr4\_0313 PE=3 SV=1 |
| 2557 | 194 | C4R0F7|C4R0F7\_KOMPG | 49.95 | 8 | 8 | 0 | 5 | 1 | 13 |  | 18070.625 | Cytochrome c oxidase subunit 4, mitochondrial OS=Komagataella phaffii (strain GS115 / ATCC 20864) OX=644223 GN=PAS\_chr2-1\_0361 PE=3 SV=1 |
| 2582 | 195 | C4R4R0|C4R4R0\_KOMPG | 52.44 | 6 | 6 | 2.96e2 | 5 | 1 | 7 |  | 42833.3984 | Actin-associated protein OS=Komagataella phaffii (strain GS115 / ATCC 20864) OX=644223 GN=PAS\_chr3\_0498 PE=4 SV=1 |
| 2587 | 196 | C4QW22|C4QW22\_KOMPG | 47.88 | 10 | 10 | 3.76e3 | 5 | 1 | 8 |  | 23650.209 | MARVEL domain-containing protein OS=Komagataella phaffii (strain GS115 / ATCC 20864) OX=644223 GN=PAS\_chr1-1\_0085 PE=4 SV=1 |
| 2598 | 197 | C4R1F5|C4R1F5\_KOMPG | 56.66 | 2 | 2 | 1.66e4 | 5 | 1 | 14 |  | 86141.4141 | TATA element modulatory factor 1 TATA binding domain-containing protein OS=Komagataella phaffii (strain GS115 / ATCC 20864) OX=644223 GN=PAS\_chr2-1\_0682 PE=4 SV=1 |
| 2610 | 198 | C4R646|C4R646\_KOMPG | 50.52 | 7 | 7 | 3.3e2 | 5 | 1 | 6 |  | 28483.6543 | Transcription factor involved in cell-type-specific transcription and pheromone response OS=Komagataella phaffii (strain GS115 / ATCC 20864) OX=644223 GN=PAS\_chr3\_0968 PE=4 SV=1 |
| 2617 | 199 | C4R3X1|C4R3X1\_KOMPG | 52.48 | 3 | 3 | 0 | 5 | 1 | 7 |  | 94184.125 | DNA-dependent ATPase OS=Komagataella phaffii (strain GS115 / ATCC 20864) OX=644223 GN=PAS\_chr3\_0224 PE=3 SV=1 |
| 2655 | 200 | C4R2R4|C4R2R4\_KOMPG | 53.48 | 5 | 5 | 6.06e2 | 5 | 1 | 11 |  | 54920.707 | Cystathionine beta-synthase OS=Komagataella phaffii (strain GS115 / ATCC 20864) OX=644223 GN=PAS\_chr2-2\_0137 PE=3 SV=1 |
| 2673 | 201 | C4QZV7|KEX1\_KOMPG | 41.48 | 4 | 4 | 1.4e3 | 5 | 1 | 8 |  | 70017.9297 | Pheromone-processing carboxypeptidase KEX1 OS=Komagataella phaffii (strain GS115 / ATCC 20864) OX=644223 GN=KEX1 PE=3 SV=1 |
| 2716 | 202 | C4R586|C4R586\_KOMPG | 48.15 | 4 | 4 | 3.33e3 | 5 | 1 | 9 | Half of a disulfide bridge | 59544.5977 | D-lactate ferricytochrome C oxidoreductase OS=Komagataella phaffii (strain GS115 / ATCC 20864) OX=644223 GN=PAS\_chr3\_0674 PE=3 SV=1 |
| 2731 | 203 | C4QYP2|C4QYP2\_KOMPG | 40.32 | 2 | 2 | 0 | 5 | 1 | 6 | Pyro-glu from Q | 87620.7109 | cysteine--tRNA ligase OS=Komagataella phaffii (strain GS115 / ATCC 20864) OX=644223 GN=PAS\_chr1-4\_0514 PE=3 SV=1 |
| 2736 | 204 | C4QXM2|C4QXM2\_KOMPG | 47.33 | 6 | 6 | 0 | 5 | 1 | 6 |  | 31451.7734 | SCP domain-containing protein OS=Komagataella phaffii (strain GS115 / ATCC 20864) OX=644223 GN=PAS\_chr1-4\_0164 PE=4 SV=1 |
| 2762 | 205 | C4R643|C4R643\_KOMPG | 43.97 | 6 | 6 | 0 | 5 | 1 | 6 |  | 45752.3047 | Component of the septin ring of the mother-bud neck that is required for cytokinesis OS=Komagataella phaffii (strain GS115 / ATCC 20864) OX=644223 GN=PAS\_chr3\_0967 PE=3 SV=1 |
| 2808 | 206 | C4QX04|C4QX04\_KOMPG | 54.78 | 3 | 3 | 7.58e2 | 5 | 1 | 15 |  | 77866.125 | SUN domain-containing protein OS=Komagataella phaffii (strain GS115 / ATCC 20864) OX=644223 GN=PAS\_chr1-1\_0400 PE=4 SV=1 |
| 2837 | 207 | C4QY77|C4QY77\_KOMPG | 50.64 | 3 | 3 | 2.52e2 | 4 | 1 | 7 | Pyro-glu from Q | 50850.6328 | cAMP-dependent protein kinase OS=Komagataella phaffii (strain GS115 / ATCC 20864) OX=644223 GN=PAS\_chr1-4\_0357 PE=3 SV=1 |
| 2861 | 208 | C4QXE6|C4QXE6\_KOMPG | 51.67 | 3 | 3 | 0 | 4 | 1 | 7 | Half of a disulfide bridge | 69564.0469 | Polyamine transport protein specific for spermine OS=Komagataella phaffii (strain GS115 / ATCC 20864) OX=644223 GN=PAS\_chr1-4\_0090 PE=4 SV=1 |
| 2878 | 209 | C4R6M9|C4R6M9\_KOMPG | 39.99 | 7 | 7 | 0 | 4 | 1 | 5 | Acetylation (Protein N-term) | 22206.2012 | DNA replication complex GINS protein PSF1 OS=Komagataella phaffii (strain GS115 / ATCC 20864) OX=644223 GN=PAS\_chr4\_0028 PE=3 SV=1 |
| 2903 | 210 | C4R7B2|C4R7B2\_KOMPG | 39.96 | 5 | 5 | 2.33e2 | 4 | 1 | 5 |  | 39007.293 | Essential protein required for the accumulation of box C/D snoRNA OS=Komagataella phaffii (strain GS115 / ATCC 20864) OX=644223 GN=PAS\_chr4\_0256 PE=3 SV=1 |
| 2935 | 211 | C4R883|C4R883\_KOMPG | 34.01 | 3 | 3 | 0 | 4 | 1 | 6 | Pyro-glu from E | 54546.2266 | Component of the septin ring of the mother-bud neck that is required for cytokinesis OS=Komagataella phaffii (strain GS115 / ATCC 20864) OX=644223 GN=PAS\_chr4\_0548 PE=3 SV=1 |
| 2991 | 212 | C4R3X3|C4R3X3\_KOMPG | 48.59 | 3 | 3 | 7.97e2 | 4 | 1 | 7 |  | 56100.6055 | Vacuolar transporter OS=Komagataella phaffii (strain GS115 / ATCC 20864) OX=644223 GN=PAS\_chr3\_0226 PE=3 SV=1 |
| 3021 | 213 | C4R359|C4R359\_KOMPG | 43.4 | 3 | 3 | 0 | 4 | 1 | 5 |  | 57064.3555 | DUF7082 domain-containing protein OS=Komagataella phaffii (strain GS115 / ATCC 20864) OX=644223 GN=PAS\_chr2-2\_0003 PE=4 SV=1 |
| 3045 | 214 | C4R3K7|C4R3K7\_KOMPG | 48.78 | 7 | 7 | 2.6e3 | 4 | 1 | 5 |  | 28809.457 | Component of the inositol phosphorylceramide synthase OS=Komagataella phaffii (strain GS115 / ATCC 20864) OX=644223 GN=PAS\_chr3\_0113 PE=4 SV=1 |
| 3148 | 215 | C4QX41|C4QX41\_KOMPG | 37.5 | 7 | 7 | 5.14e3 | 4 | 1 | 4 |  | 27998.6992 | Ubiquitin-conjugating enzyme E2 6 OS=Komagataella phaffii (strain GS115 / ATCC 20864) OX=644223 GN=PAS\_chr1-1\_0437 PE=4 SV=1 |
| 3165 | 216 | C4R2I3|C4R2I3\_KOMPG | 35.12 | 17 | 17 | 4.44e3 | 4 | 1 | 8 |  | 8964.4814 | Protein transport protein Sec61 subunit beta OS=Komagataella phaffii (strain GS115 / ATCC 20864) OX=644223 GN=PAS\_chr2-2\_0210 PE=3 SV=1 |
| 3185 | 217 | C4R6L3|C4R6L3\_KOMPG | 38.9 | 5 | 5 | 1.92e2 | 4 | 1 | 6 |  | 32044.6836 | Uncharacterized protein OS=Komagataella phaffii (strain GS115 / ATCC 20864) OX=644223 GN=PAS\_chr4\_0009 PE=4 SV=1 |
| 3235 | 218 | C4R7J0|C4R7J0\_KOMPG | 45.28 | 4 | 4 | 6e1 | 4 | 1 | 5 | Deamidation (NQ) | 56953.2227 | Signal recognition particle 54 kDa protein OS=Komagataella phaffii (strain GS115 / ATCC 20864) OX=644223 GN=PAS\_chr4\_0322 PE=3 SV=1 |
| 3242 | 219 | C4R632|C4R632\_KOMPG | 38.21 | 3 | 3 | 2.22e2 | 4 | 1 | 10 | Pyro-glu from Q | 58860.6055 | Lanosterol 14-alpha demethylase OS=Komagataella phaffii (strain GS115 / ATCC 20864) OX=644223 GN=PAS\_chr3\_0957 PE=3 SV=1 |
| 3282 | 220 | C4R2Z0|C4R2Z0\_KOMPG | 33.84 | 2 | 2 | 3.68e1 | 3 | 1 | 4 |  | 84103.3281 | Nuclear actin-related protein involved in chromatin remodeling OS=Komagataella phaffii (strain GS115 / ATCC 20864) OX=644223 GN=PAS\_chr2-2\_0068 PE=3 SV=1 |
| 3285 | 221 | C4R7A9|C4R7A9\_KOMPG | 40.24 | 6 | 6 | 4.27e2 | 3 | 1 | 4 |  | 27958.4648 | Ribosome-recycling factor, mitochondrial OS=Komagataella phaffii (strain GS115 / ATCC 20864) OX=644223 GN=PAS\_chr4\_0253 PE=3 SV=1 |
| 3316 | 222 | C4R6R0|C4R6R0\_KOMPG | 37.61 | 12 | 12 | 4.39e3 | 3 | 1 | 5 | Deamidation (NQ) | 17741.6387 | Glucosamine 6-phosphate N-acetyltransferase OS=Komagataella phaffii (strain GS115 / ATCC 20864) OX=644223 GN=PAS\_chr4\_0060 PE=3 SV=1 |
| 3338 | 223 | C4R8L4|C4R8L4\_KOMPG | 31.83 | 4 | 4 | 2.92e2 | 3 | 1 | 4 |  | 41079.0234 | Chromatin modification-related protein OS=Komagataella phaffii (strain GS115 / ATCC 20864) OX=644223 GN=PAS\_chr4\_0679 PE=3 SV=1 |
| 3384 | 224 | C4QZK0|C4QZK0\_KOMPG | 33.87 | 2 | 2 | 4.24e2 | 3 | 1 | 5 |  | 56927.207 | pH-response regulator protein palH/RIM21 OS=Komagataella phaffii (strain GS115 / ATCC 20864) OX=644223 GN=PAS\_chr2-1\_0073 PE=3 SV=1 |
| 3393 | 225 | C4QZ09|C4QZ09\_KOMPG | 34.68 | 2 | 2 | 0 | 3 | 1 | 3 | Pyro-glu from E | 73105.7812 | Actin assembly factor, activates the Arp2/3 protein complex that nucleates branched actin filaments OS=Komagataella phaffii (strain GS115 / ATCC 20864) OX=644223 GN=PAS\_chr1-4\_0623 PE=4 SV=1 |
| 3406 | 226 | C4R4X4|C4R4X4\_KOMPG | 39.55 | 3 | 3 | 9.2e2 | 3 | 1 | 6 |  | 46817.3047 | Elongation factor Tu OS=Komagataella phaffii (strain GS115 / ATCC 20864) OX=644223 GN=PAS\_chr3\_0562 PE=3 SV=1 |
| 3411 | 227 | C4R740|C4R740\_KOMPG | 30.25 | 4 | 4 | 7.13e1 | 3 | 1 | 5 | Oxidation (M) | 35176.6289 | Uncharacterized protein OS=Komagataella phaffii (strain GS115 / ATCC 20864) OX=644223 GN=PAS\_chr4\_0190 PE=4 SV=1 |
| 3425 | 228 | C4QYD9|C4QYD9\_KOMPG | 34.81 | 5 | 5 | 1.85e3 | 3 | 1 | 5 |  | 43199.8555 | Transcription initiation factor IIF subunit beta OS=Komagataella phaffii (strain GS115 / ATCC 20864) OX=644223 GN=PAS\_chr1-4\_0679 PE=3 SV=1 |
| 3445 | 229 | C4QWJ1|C4QWJ1\_KOMPG | 33.28 | 2 | 2 | 1.54e2 | 3 | 1 | 5 |  | 90159.9844 | SWR1-complex protein 3 OS=Komagataella phaffii (strain GS115 / ATCC 20864) OX=644223 GN=PAS\_chr1-1\_0244 PE=4 SV=1 |
| 3450 | 230 | C4R0M8|C4R0M8\_KOMPG | 39.53 | 11 | 11 | 1.28e3 | 3 | 1 | 5 |  | 13896.0244 | Histone H2A OS=Komagataella phaffii (strain GS115 / ATCC 20864) OX=644223 GN=PAS\_chr2-1\_0429 PE=3 SV=1 |
| 3481 | 231 | C4R836|C4R836\_KOMPG | 35.36 | 4 | 4 | 5.62e3 | 3 | 1 | 4 |  | 29204.1641 | Prohibitin OS=Komagataella phaffii (strain GS115 / ATCC 20864) OX=644223 GN=PAS\_chr4\_0505 PE=3 SV=1 |
| 3481 | 232 | C4R5I1|C4R5I1\_KOMPG | 19.85 | 3 | 3 | 5.62e3 | 1 | 1 | 1 |  | 22204.582 | Transcriptional activator HAP2 OS=Komagataella phaffii (strain GS115 / ATCC 20864) OX=644223 GN=PAS\_chr3\_0766 PE=3 SV=1 |
| 3533 | 233 | C4QVH8|C4QVH8\_KOMPG | 34.63 | 3 | 3 | 2.41e3 | 3 | 1 | 3 |  | 49572.5352 | Ribosome biogenesis protein YTM1 OS=Komagataella phaffii (strain GS115 / ATCC 20864) OX=644223 GN=YTM1 PE=3 SV=1 |
| 3542 | 234 | C4R406|C4R406\_KOMPG | 38.61 | 4 | 4 | 0 | 3 | 1 | 3 |  | 32748.916 | Mitochondrial GTP/GDP transporter OS=Komagataella phaffii (strain GS115 / ATCC 20864) OX=644223 GN=PAS\_chr3\_0255 PE=3 SV=1 |
| 3594 | 235 | C4R550|C4R550\_KOMPG | 31.35 | 4 | 4 | 1.18e2 | 3 | 1 | 3 |  | 45293.6523 | Membrane anchor Opy2 N-terminal domain-containing protein OS=Komagataella phaffii (strain GS115 / ATCC 20864) OX=644223 GN=PAS\_chr3\_0637 PE=4 SV=1 |
| 3715 | 236 | C4R1M3|C4R1M3\_KOMPG | 32.83 | 16 | 16 | 1.39e3 | 2 | 1 | 3 |  | 7050.2905 | Cytochrome c oxidase subunit 7 OS=Komagataella phaffii (strain GS115 / ATCC 20864) OX=644223 GN=PAS\_chr2-1\_0746 PE=4 SV=1 |
| 3720 | 237 | C4R777|C4R777\_KOMPG | 28.27 | 3 | 3 | 0 | 2 | 1 | 3 |  | 38873.8711 | Evolutionarily conserved subunit of the CCR4-NOT complex involved in controlling mRNA initiation OS=Komagataella phaffii (strain GS115 / ATCC 20864) OX=644223 GN=PAS\_chr4\_0224 PE=3 SV=1 |
| 3732 | 238 | C4QZX2|C4QZX2\_KOMPG | 34.28 | 4 | 4 | 7.31e3 | 2 | 1 | 3 |  | 27344.8594 | Proteasome subunit alpha type OS=Komagataella phaffii (strain GS115 / ATCC 20864) OX=644223 GN=PAS\_chr2-1\_0188 PE=3 SV=1 |
| 3750 | 239 | C4R4Y6|C4R4Y6\_KOMPG | 34.96 | 3 | 3 | 9.1e3 | 2 | 1 | 2 |  | 39338.0508 | DNA-directed RNA polymerase III subunit RPC4 OS=Komagataella phaffii (strain GS115 / ATCC 20864) OX=644223 GN=PAS\_chr3\_0574 PE=4 SV=1 |
| 3762 | 240 | C4QYZ9|C4QYZ9\_KOMPG | 29.4 | 13 | 13 | 0 | 2 | 1 | 4 |  | 11160.8223 | Small nuclear ribonucleoprotein Sm D3 OS=Komagataella phaffii (strain GS115 / ATCC 20864) OX=644223 GN=PAS\_chr1-4\_0614 PE=3 SV=1 |
| 3781 | 241 | C4QYV1|C4QYV1\_KOMPG | 32.6 | 4 | 4 | 0 | 2 | 1 | 6 |  | 35248.2812 | Primary component of eisosomes OS=Komagataella phaffii (strain GS115 / ATCC 20864) OX=644223 GN=PAS\_chr1-4\_0569 PE=4 SV=1 |
| 3825 | 242 | C4R937|C4R937\_KOMPG | 19.32 | 4 | 4 | 0 | 1 | 1 | 1 |  | 14237.0234 | Peptide-methionine (R)-S-oxide reductase OS=Komagataella phaffii (strain GS115 / ATCC 20864) OX=644223 GN=PAS\_chr4\_0843 PE=3 SV=1 |
| total 242 proteins |
| --- |

  

C4QWB0|C4QWB0\_KOMPG

back to list

  

| Protein Coverage
| Supporting Peptides
| Best Unique PSM
|

Protein Coverage:

Supporting Peptides:

| Peptide | Uniq | -10lgP | Mass | Length | ppm | m/z | z | RT | Scan | Area POS\_R15-1 | #Feature | #Feature POS\_R15-1 | Start | End | PTM | AScore | Found By |
| --- | --- | --- | --- | --- | --- | --- | --- | --- | --- | --- | --- | --- | --- | --- | --- | --- | --- |
| G.PSIL.L | N | 29.95 | 428.2635 | 4 | -0.56 | 429.2694 | 1 | 21.28 | 7884 | 0 | 0 | 0 | 708 | 711 |  |  | DB Search |
| P.FYR.T | N | 26.82 | 484.2434 | 3 | 1.64 | 485.2503 | 1 | 17.34 | 6854 | 0 | 0 | 0 | 401 | 403 |  |  | DB Search |
| L.IGGGA.K | N | 25.05 | 373.1961 | 5 | 1.54 | 374.203 | 1 | 3.00 | 1292 | 0 | 0 | 0 | 175 | 179 |  |  | DB Search |
| L.AIFG.T | N | 24.47 | 406.2216 | 4 | 1.3 | 407.2284 | 1 | 22.63 | 8312 | 0 | 0 | 0 | 830 | 833 |  |  | DB Search |
| L.GVGP.P | N | 24.4 | 328.1746 | 4 | -1.91 | 329.1805 | 1 | 5.92 | 2228 | 4.71e4 | 1 | 1 | 874 | 877 |  |  | DB Search |
| I.ILLP.L | N | 24.07 | 454.3155 | 4 | -2.4 | 455.3206 | 1 | 34.52 | 11565 | 2.64e3 | 1 | 1 | 3 | 6 |  |  | DB Search |
| I.VGVT.V | N | 24.06 | 374.2165 | 4 | -2.35 | 375.222 | 1 | 5.91 | 2327 | 7.9e3 | 1 | 1 | 1213 | 1216 |  |  | DB Search |
| F.ASAL.I | N | 22.98 | 360.2009 | 4 | -6.45 | 361.2049 | 1 | 8.68 | 3069 | 1.41e4 | 1 | 1 | 1113 | 1116 |  |  | DB Search |
| G.LSGI.I | N | 22.6 | 388.2322 | 4 | -3.81 | 389.237 | 1 | 9.87 | 3804 | 6.08e3 | 1 | 1 | 632 | 635 |  |  | DB Search |
| G.VTVL.A | N | 21.25 | 430.2791 | 4 | -6.35 | 431.2826 | 1 | 27.75 | 9755 | 1.56e3 | 1 | 1 | 1215 | 1218 |  |  | DB Search |
| L.LSVS.S | N | 20.49 | 404.2271 | 4 | -7.08 | 405.2305 | 1 | 8.17 | 3035 | 7.47e3 | 1 | 1 | 640 | 643 |  |  | DB Search |
| Y.KCA.C | N | 19.71 | 320.1518 | 3 | 2.66 | 321.1591 | 1 | 15.28 | 6053 | 3.59e2 | 1 | 1 | 234 | 236 |  |  | DB Search |
| E.QFF.N | N | 19.56 | 440.206 | 3 | 3.92 | 441.2139 | 1 | 19.15 | 7313 | 0 | 0 | 0 | 315 | 317 |  |  | DB Search |
| I.IAEV.I | N | 19.27 | 430.2427 | 4 | 1.69 | 431.2497 | 1 | 13.56 | 5318 | 0 | 0 | 0 | 660 | 663 |  |  | DB Search |
| C.STSP.K | N | 19.15 | 390.1751 | 4 | -0.08 | 391.1813 | 1 | 5.92 | 2360 | 3.31e2 | 1 | 1 | 346 | 349 |  |  | DB Search |
| T.SLDT.T | N | 19.03 | 434.2013 | 4 | -3.95 | 435.2057 | 1 | 15.10 | 5999 | 0 | 0 | 0 | 1178 | 1181 |  |  | DB Search |
| R.LQEQ.T | N | 18.84 | 516.2544 | 4 | -0.21 | 517.2603 | 1 | 2.57 | 1095 | 1.3e3 | 1 | 1 | 1278 | 1281 |  |  | DB Search |
| F.GTFT.I | N | 18.77 | 424.1958 | 4 | -4.93 | 425.1999 | 1 | 10.09 | 3929 | 0 | 0 | 0 | 833 | 836 |  |  | DB Search |
| A.TVVS.M | N | 18.11 | 404.2271 | 4 | -7.08 | 405.2305 | 1 | 8.17 | 3091 | 7.47e3 | 1 | 1 | 725 | 728 |  |  | DB Search |
| L.DTTY.A | N | 17.77 | 498.1962 | 4 | 5.72 | 499.2051 | 1 | 3.23 | 1391 | 0 | 0 | 0 | 1180 | 1183 |  |  | DB Search |
| A.PYSSSV.F | Y | 17.48 | 638.2911 | 6 | 6.46 | 639.301 | 1 | 14.48 | 5685 | 0 | 0 | 0 | 1015 | 1020 |  |  | DB Search |
| G.RGY.I | N | 17.46 | 394.1965 | 3 | 3.18 | 395.204 | 1 | 18.17 | 7080 | 0 | 0 | 0 | 1259 | 1261 |  |  | DB Search |
| S.FMR.F | N | 17.07 | 452.2206 | 3 | -0.76 | 453.2264 | 1 | 15.57 | 6149 | 2.05e2 | 1 | 1 | 366 | 368 |  |  | DB Search |
| L.IDYF.D | N | 16.91 | 556.2533 | 4 | -2.81 | 557.2576 | 1 | 23.71 | 8633 | 4.27e1 | 1 | 1 | 863 | 866 |  |  | DB Search |
| K.APY.S | N | 16.84 | 349.1638 | 3 | -4.81 | 350.1685 | 1 | 16.32 | 6384 | 1.41e3 | 1 | 1 | 1014 | 1016 |  |  | DB Search |
| C.PACR.S | N | 16.06 | 445.2107 | 4 | 4.57 | 446.2189 | 1 | 4.98 | 1993 | 0 | 0 | 0 | 102 | 105 |  |  | DB Search |
| G.TFT.I | N | 15.92 | 367.1743 | 3 | -7.47 | 368.1779 | 1 | 8.54 | 3263 | 0 | 0 | 0 | 834 | 836 |  |  | DB Search |
| total 27 peptides |
| --- |

Best Unique PSM (Scan POS\_R15-1.wiff:5685, m/z=639.2788, z=1, RT=14.48, ppm=3.98):


C4R7B2|C4R7B2\_KOMPG

back to list

  

| Protein Coverage
| Supporting Peptides
| Best Unique PSM
|

Protein Coverage:

Supporting Peptides:

| Peptide | Uniq | -10lgP | Mass | Length | ppm | m/z | z | RT | Scan | Area POS\_R15-1 | #Feature | #Feature POS\_R15-1 | Start | End | PTM | AScore | Found By |
| --- | --- | --- | --- | --- | --- | --- | --- | --- | --- | --- | --- | --- | --- | --- | --- | --- | --- |
| L.GTLF.P | N | 20.4 | 436.2322 | 4 | -4.1 | 437.2366 | 1 | 23.18 | 8454 | 1.09e3 | 1 | 1 | 187 | 190 |  |  | DB Search |
| C.SGII.D | N | 19.42 | 388.2322 | 4 | -5.15 | 389.2365 | 1 | 17.34 | 6766 | 1.43e3 | 1 | 1 | 43 | 46 |  |  | DB Search |
| R.MLPP.G | N | 18.04 | 456.2406 | 4 | 3.4 | 457.2483 | 1 | 13.75 | 5423 | 0 | 0 | 0 | 134 | 137 |  |  | DB Search |
| P.TIYASH.F | Y | 15.32 | 690.3337 | 6 | -5.85 | 691.3352 | 1 | 25.91 | 9212 | 2.33e2 | 1 | 1 | 244 | 249 |  |  | DB Search |
| total 4 peptides |
| --- |

Best Unique PSM (Scan POS\_R15-1.wiff:9212, m/z=691.3352, z=1, RT=25.91, ppm=-8.33):


C4QYI7|C4QYI7\_KOMPG

back to list

  

| Protein Coverage
| Supporting Peptides
| Best Unique PSM
|

Protein Coverage:

Supporting Peptides:

| Peptide | Uniq | -10lgP | Mass | Length | ppm | m/z | z | RT | Scan | Area POS\_R15-1 | #Feature | #Feature POS\_R15-1 | Start | End | PTM | AScore | Found By |
| --- | --- | --- | --- | --- | --- | --- | --- | --- | --- | --- | --- | --- | --- | --- | --- | --- | --- |
| N.TIDI.R | N | 22.27 | 460.2533 | 4 | -8.03 | 461.2557 | 1 | 9.41 | 3625 | 2.97e2 | 1 | 1 | 558 | 561 |  |  | DB Search |
| L.VENGDVI.T | Y | 22.24 | 744.3654 | 7 | -6.72 | 745.3658 | 1 | 15.15 | 6014 | 0 | 0 | 0 | 544 | 550 |  |  | DB Search |
| G.SLAP.G | N | 20.83 | 386.2165 | 4 | -4.65 | 387.221 | 1 | 8.35 | 3276 | 4.13e3 | 1 | 1 | 416 | 419 |  |  | DB Search |
| H.TVGV.E | N | 20.58 | 374.2165 | 4 | -4.06 | 375.2213 | 1 | 5.20 | 1970 | 6.27e3 | 1 | 1 | 315 | 318 |  |  | DB Search |
| I.ALVE.N | N | 20.45 | 430.2427 | 4 | -5.9 | 431.2464 | 1 | 12.49 | 4901 | 0 | 0 | 0 | 542 | 545 |  |  | DB Search |
| A.WSD.G | N | 19.99 | 406.1488 | 3 | -8.45 | 407.1517 | 1 | 8.75 | 3390 | 0 | 0 | 0 | 521 | 523 |  |  | DB Search |
| N.TIGV.S | N | 18.91 | 388.2322 | 4 | -5.15 | 389.2365 | 1 | 17.34 | 6852 | 1.43e3 | 1 | 1 | 102 | 105 |  |  | DB Search |
| A.LLTD.G | N | 17.52 | 460.2533 | 4 | -9.09 | 461.2552 | 1 | 10.16 | 3965 | 9.46e2 | 1 | 1 | 498 | 501 |  |  | DB Search |
| I.EALE.R | N | 15.32 | 460.2169 | 4 | -0.24 | 461.2229 | 1 | 13.77 | 5437 | 0 | 0 | 0 | 449 | 452 |  |  | DB Search |
| total 9 peptides |
| --- |

Best Unique PSM (Scan POS\_R15-1.wiff:6014, m/z=745.8396, z=2, RT=15.18, ppm=-9.2):


C4R359|C4R359\_KOMPG

back to list

  

| Protein Coverage
| Supporting Peptides
| Best Unique PSM
|

Protein Coverage:

Supporting Peptides:

| Peptide | Uniq | -10lgP | Mass | Length | ppm | m/z | z | RT | Scan | Area POS\_R15-1 | #Feature | #Feature POS\_R15-1 | Start | End | PTM | AScore | Found By |
| --- | --- | --- | --- | --- | --- | --- | --- | --- | --- | --- | --- | --- | --- | --- | --- | --- | --- |
| K.VLGS.M | N | 24.2 | 374.2165 | 4 | -4.06 | 375.2213 | 1 | 5.20 | 2012 | 6.27e3 | 1 | 1 | 224 | 227 |  |  | DB Search |
| N.SNG.I | N | 19.07 | 276.107 | 3 | 1.61 | 277.114 | 1 | 16.82 | 6604 | 0 | 0 | 0 | 33 | 35 |  |  | DB Search |
| G.RSF.G | N | 17.5 | 408.2121 | 3 | -1.76 | 409.2177 | 1 | 9.17 | 3526 | 0 | 0 | 0 | 241 | 243 |  |  | DB Search |
| N.CRPP.K | Y | 15.32 | 471.2264 | 4 | 0.77 | 472.2328 | 1 | 7.26 | 2757 | 0 | 0 | 0 | 248 | 251 |  |  | DB Search |
| total 4 peptides |
| --- |

Best Unique PSM (Scan POS\_R15-1.wiff:2757, m/z=472.2461, z=1, RT=7.31, ppm=-1.71):


C4R6D7|C4R6D7\_KOMPG

back to list

  

| Protein Coverage
| Supporting Peptides
| Best Unique PSM
|

Protein Coverage:

Supporting Peptides:

| Peptide | Uniq | -10lgP | Mass | Length | ppm | m/z | z | RT | Scan | Area POS\_R15-1 | #Feature | #Feature POS\_R15-1 | Start | End | PTM | AScore | Found By |
| --- | --- | --- | --- | --- | --- | --- | --- | --- | --- | --- | --- | --- | --- | --- | --- | --- | --- |
| L.NGL.V | N | 28.25 | 302.159 | 3 | -3.76 | 303.1644 | 1 | 6.06 | 2374 | 3.14e3 | 1 | 1 | 244 | 246 |  |  | DB Search |
| T.LSLE.A | N | 25.68 | 460.2533 | 4 | -2.19 | 461.2584 | 1 | 13.54 | 5303 | 1.35e4 | 1 | 1 | 370 | 373 |  |  | DB Search |
| S.LGATTLEL.L | Y | 24.2 | 816.4593 | 8 | -2.06 | 817.4628 | 1 | 42.05 | 13313 | 0 | 0 | 0 | 332 | 339 |  |  | DB Search |
| E.HFT.M | N | 23.64 | 403.1856 | 3 | -0.83 | 404.1915 | 1 | 13.94 | 5543 | 0 | 0 | 0 | 717 | 719 |  |  | DB Search |
| D.ELF.H | N | 21.7 | 407.2056 | 3 | -8.99 | 408.2082 | 1 | 29.23 | 10157 | 9.42e2 | 1 | 1 | 18 | 20 |  |  | DB Search |
| L.ITVA.F | N | 19.62 | 402.2478 | 4 | -4.79 | 403.2522 | 1 | 10.65 | 4156 | 4.9e3 | 1 | 1 | 740 | 743 |  |  | DB Search |
| V.SLTP.A | N | 19.55 | 416.2271 | 4 | 2 | 417.2342 | 1 | 10.25 | 4029 | 3.69e3 | 1 | 1 | 112 | 115 |  |  | DB Search |
| Q.NLTI.W | N | 19.19 | 459.2693 | 4 | -0.54 | 460.2752 | 1 | 17.07 | 6712 | 2.02e3 | 1 | 1 | 667 | 670 |  |  | DB Search |
| S.SNG.I | N | 19.07 | 276.107 | 3 | 1.61 | 277.114 | 1 | 16.82 | 6604 | 0 | 0 | 0 | 677 | 679 |  |  | DB Search |
| Y.HGY.I | N | 18.63 | 375.1543 | 3 | -9 | 376.1572 | 1 | 4.83 | 1959 | 3.43e3 | 1 | 1 | 197 | 199 |  |  | DB Search |
| L.EIVQQ.L | N | 18.59 | 615.3228 | 5 | -8.79 | 616.3231 | 1 | 8.49 | 3240 | 2.96e3 | 1 | 1 | 654 | 658 |  |  | DB Search |
| S.LTP.A | N | 17.99 | 329.1951 | 3 | 1.36 | 330.202 | 1 | 7.49 | 2815 | 1.47e3 | 1 | 1 | 113 | 115 |  |  | DB Search |
| Q.LNFD.R | N | 17.97 | 507.2329 | 4 | -4.86 | 508.2365 | 1 | 16.93 | 6638 | 1.73e3 | 1 | 1 | 436 | 439 |  |  | DB Search |
| T.ESN.L | N | 17.14 | 348.1281 | 3 | 8.51 | 349.1375 | 1 | 5.67 | 2161 | 1.07e4 | 1 | 1 | 203 | 205 |  |  | DB Search |
| L.SPK.K | N | 16.89 | 330.1903 | 3 | -8.46 | 331.194 | 1 | 5.91 | 2235 | 5.17e3 | 1 | 1 | 36 | 38 |  |  | DB Search |
| S.E(-18.01)PR.V | N | 16.87 | 382.1965 | 3 | -0.37 | 383.2026 | 1 | 2.61 | 1082 | 1.62e4 | 1 | 1 | 93 | 95 | Pyro-glu from E | E1:Pyro-glu from E:1000 | DB Search |
| P.E(-18.01)PL.V | N | 16.1 | 339.1794 | 3 | -5.62 | 340.1839 | 1 | 16.05 | 6351 | 1.63e4 | 1 | 1 | 232 | 234 | Pyro-glu from E | E1:Pyro-glu from E:1000 | DB Search |
| E.TLNG.L | N | 15.96 | 403.2067 | 4 | -9.11 | 404.2093 | 1 | 5.03 | 2025 | 0 | 0 | 0 | 242 | 245 |  |  | DB Search |
| K.LKTP.T | N | 15.64 | 457.29 | 4 | -5.17 | 458.2938 | 1 | 15.66 | 6234 | 0 | 0 | 0 | 505 | 508 |  |  | DB Search |
| K.QLVA.I | N | 15.44 | 429.2587 | 4 | -4.77 | 430.2629 | 1 | 10.63 | 4142 | 1.75e3 | 1 | 1 | 160 | 163 |  |  | DB Search |
| K.YHG.Y | N | 15.26 | 375.1543 | 3 | -7.53 | 376.1578 | 1 | 4.73 | 1840 | 0 | 0 | 0 | 196 | 198 |  |  | DB Search |
| total 21 peptides |
| --- |

Best Unique PSM (Scan POS\_R15-1.wiff:13313, m/z=817.4705, z=1, RT=42.08, ppm=-4.54):


C4QXR9|C4QXR9\_KOMPG

back to list

  

| Protein Coverage
| Supporting Peptides
| Best Unique PSM
|

Protein Coverage:

Supporting Peptides:

| Peptide | Uniq | -10lgP | Mass | Length | ppm | m/z | z | RT | Scan | Area POS\_R15-1 | #Feature | #Feature POS\_R15-1 | Start | End | PTM | AScore | Found By |
| --- | --- | --- | --- | --- | --- | --- | --- | --- | --- | --- | --- | --- | --- | --- | --- | --- | --- |
| V.FYR.P | N | 26.82 | 484.2434 | 3 | 1.64 | 485.2503 | 1 | 17.34 | 6854 | 0 | 0 | 0 | 749 | 751 |  |  | DB Search |
| R.RCL.P | N | 20.6 | 390.2049 | 3 | -4.41 | 391.2095 | 1 | 8.08 | 3003 | 0 | 0 | 0 | 293 | 295 |  |  | DB Search |
| M.LSVP.N | N | 19.74 | 414.2478 | 4 | -0.09 | 415.254 | 1 | 17.14 | 6914 | 3.72e3 | 1 | 1 | 743 | 746 |  |  | DB Search |
| T.SLDT.F | N | 19.03 | 434.2013 | 4 | -3.95 | 435.2057 | 1 | 15.10 | 5999 | 0 | 0 | 0 | 167 | 170 |  |  | DB Search |
| T.VDGI.V | N | 18.71 | 402.2114 | 4 | 1.69 | 403.2184 | 1 | 10.06 | 3921 | 2.85e3 | 1 | 1 | 579 | 582 |  |  | DB Search |
| P.IYSS.L | N | 17.99 | 468.222 | 4 | 0.32 | 469.2283 | 1 | 16.06 | 6398 | 1.94e3 | 1 | 1 | 545 | 548 |  |  | DB Search |
| M.TSFP.M | N | 15.56 | 450.2114 | 4 | -3.74 | 451.2159 | 1 | 14.02 | 5557 | 2.84e3 | 1 | 1 | 712 | 715 |  |  | DB Search |
| L.ETSKTQ(+0.98)KVVT.N | Y | 15.53 | 1120.5976 | 10 | -2.35 | 561.3033 | 2 | 20.12 | 7563 | 0 | 0 | 0 | 942 | 951 | Deamidation (NQ) | Q6:Deamidation (NQ):1000 | DB Search |
| total 8 peptides |
| --- |

Best Unique PSM (Scan POS\_R15-1.wiff:7563, m/z=561.3033, z=2, RT=20.12, ppm=-4.83):


C4R922|C4R922\_KOMPG

back to list

  

| Protein Coverage
| Supporting Peptides
| Best Unique PSM
|

Protein Coverage:

Supporting Peptides:

| Peptide | Uniq | -10lgP | Mass | Length | ppm | m/z | z | RT | Scan | Area POS\_R15-1 | #Feature | #Feature POS\_R15-1 | Start | End | PTM | AScore | Found By |
| --- | --- | --- | --- | --- | --- | --- | --- | --- | --- | --- | --- | --- | --- | --- | --- | --- | --- |
| I.GGGGL.A | N | 28.62 | 359.1805 | 5 | -3.01 | 360.1858 | 1 | 6.13 | 2409 | 0 | 0 | 0 | 271 | 275 |  |  | DB Search |
| G.GGGL.A | N | 27.46 | 302.159 | 4 | -3.72 | 303.1644 | 1 | 6.06 | 2387 | 3.14e3 | 1 | 1 | 272 | 275 |  |  | DB Search |
| F.TIDL.A | N | 22.27 | 460.2533 | 4 | -8.03 | 461.2557 | 1 | 9.41 | 3625 | 2.97e2 | 1 | 1 | 424 | 427 |  |  | DB Search |
| S.SSIF.V | N | 21.63 | 452.2271 | 4 | -3.7 | 453.2316 | 1 | 22.49 | 8262 | 1.33e3 | 1 | 1 | 390 | 393 |  |  | DB Search |
| L.ITFPL.M | N | 21.27 | 589.3475 | 5 | -4.36 | 590.3508 | 1 | 43.54 | 13581 | 0 | 0 | 0 | 436 | 440 |  |  | DB Search |
| S.MGR.N | N | 20.83 | 362.1736 | 3 | 6.82 | 363.1825 | 1 | 3.30 | 1420 | 1.5e3 | 1 | 1 | 50 | 52 |  |  | DB Search |
| A.EAY.E | N | 19.24 | 381.1536 | 3 | -5.14 | 382.158 | 1 | 5.09 | 2057 | 2.63e3 | 1 | 1 | 313 | 315 |  |  | DB Search |
| A.IYSS.G | N | 17.99 | 468.222 | 4 | 0.32 | 469.2283 | 1 | 16.06 | 6398 | 1.94e3 | 1 | 1 | 476 | 479 |  |  | DB Search |
| G.GGLAWRL.M | Y | 17.38 | 771.4391 | 7 | -1.94 | 772.443 | 1 | 35.58 | 11827 | 0 | 0 | 0 | 273 | 279 |  |  | DB Search |
| S.APY.I | N | 16.84 | 349.1638 | 3 | -4.81 | 350.1685 | 1 | 16.32 | 6384 | 1.41e3 | 1 | 1 | 157 | 159 |  |  | DB Search |
| K.ERH.A | N | 16.79 | 440.2132 | 3 | -0.12 | 441.2193 | 1 | 19.25 | 7313 | 6.39e2 | 1 | 1 | 309 | 311 |  |  | DB Search |
| F.ISAI.T | N | 16.63 | 402.2478 | 4 | -8.97 | 403.2505 | 1 | 9.42 | 3655 | 1.65e3 | 1 | 1 | 189 | 192 |  |  | DB Search |
| M.EFLSQ.E | N | 16.28 | 622.2962 | 5 | -5.13 | 623.2988 | 1 | 16.19 | 6365 | 4.92e2 | 1 | 1 | 82 | 86 |  |  | DB Search |
| F.DVPT.W | N | 15.45 | 430.2063 | 4 | 7.02 | 431.2156 | 1 | 13.00 | 5140 | 9.87e2 | 1 | 1 | 567 | 570 |  |  | DB Search |
| total 14 peptides |
| --- |

Best Unique PSM (Scan POS\_R15-1.wiff:11827, m/z=772.4515, z=1, RT=35.75, ppm=-4.42):


C4R4Y6|C4R4Y6\_KOMPG

back to list

  

| Protein Coverage
| Supporting Peptides
| Best Unique PSM
|

Protein Coverage:

Supporting Peptides:

| Peptide | Uniq | -10lgP | Mass | Length | ppm | m/z | z | RT | Scan | Area POS\_R15-1 | #Feature | #Feature POS\_R15-1 | Start | End | PTM | AScore | Found By |
| --- | --- | --- | --- | --- | --- | --- | --- | --- | --- | --- | --- | --- | --- | --- | --- | --- | --- |
| N.TLPT.F | N | 26.11 | 430.2427 | 4 | -4.36 | 431.2471 | 1 | 9.16 | 3520 | 0 | 0 | 0 | 282 | 285 |  |  | DB Search |
| R.RTTPVG.N | Y | 17.69 | 629.3497 | 6 | -7.22 | 630.3508 | 1 | 5.01 | 1976 | 9.1e3 | 1 | 1 | 16 | 21 |  |  | DB Search |
| total 2 peptides |
| --- |

Best Unique PSM (Scan POS\_R15-1.wiff:1976, m/z=630.3508, z=1, RT=5.01, ppm=-9.7):


C4R3F4|C4R3F4\_KOMPG

back to list

  

| Protein Coverage
| Supporting Peptides
| Best Unique PSM
|

Protein Coverage:

Supporting Peptides:

| Peptide | Uniq | -10lgP | Mass | Length | ppm | m/z | z | RT | Scan | Area POS\_R15-1 | #Feature | #Feature POS\_R15-1 | Start | End | PTM | AScore | Found By |
| --- | --- | --- | --- | --- | --- | --- | --- | --- | --- | --- | --- | --- | --- | --- | --- | --- | --- |
| R.IGSL.D | N | 25.26 | 388.2322 | 4 | -3.81 | 389.237 | 1 | 9.87 | 3785 | 6.08e3 | 1 | 1 | 199 | 202 |  |  | DB Search |
| F.YHH.Y | N | 24.3 | 455.1917 | 3 | -4.98 | 456.1956 | 1 | 6.94 | 2666 | 0 | 0 | 0 | 621 | 623 |  |  | DB Search |
| I.LSGI.V | N | 22.6 | 388.2322 | 4 | -3.81 | 389.237 | 1 | 9.87 | 3804 | 6.08e3 | 1 | 1 | 384 | 387 |  |  | DB Search |
| A.LDGL.N | N | 22.09 | 416.2271 | 4 | -1.79 | 417.2326 | 1 | 13.94 | 5544 | 2.42e3 | 1 | 1 | 75 | 78 |  |  | DB Search |
| V.TSG.L | N | 21.44 | 263.1117 | 3 | 2.31 | 264.119 | 1 | 3.71 | 1537 | 5.72e3 | 1 | 1 | 349 | 351 |  |  | DB Search |
| Q.NSG.N | N | 20.42 | 276.107 | 3 | 6.48 | 277.1154 | 1 | 16.74 | 6592 | 0 | 0 | 0 | 244 | 246 |  |  | DB Search |
| Y.HHY.D | N | 19.96 | 455.1917 | 3 | -1.96 | 456.197 | 1 | 6.88 | 2643 | 0 | 0 | 0 | 622 | 624 |  |  | DB Search |
| T.SGLL.Y | N | 19.42 | 388.2322 | 4 | -5.15 | 389.2365 | 1 | 17.34 | 6766 | 1.43e3 | 1 | 1 | 350 | 353 |  |  | DB Search |
| S.INFD.E | N | 17.97 | 507.2329 | 4 | -4.86 | 508.2365 | 1 | 16.93 | 6638 | 1.73e3 | 1 | 1 | 362 | 365 |  |  | DB Search |
| F.STLA.I | N | 17.83 | 390.2114 | 4 | -5.32 | 391.2157 | 1 | 6.25 | 2360 | 1.07e4 | 1 | 1 | 416 | 419 |  |  | DB Search |
| L.ESW.M | N | 17.43 | 420.1645 | 3 | -0.16 | 421.1707 | 1 | 11.21 | 4460 | 0 | 0 | 0 | 752 | 754 |  |  | DB Search |
| R.VLPV.G | N | 17 | 426.2842 | 4 | -1.74 | 427.2897 | 1 | 17.76 | 6936 | 3.8e3 | 1 | 1 | 606 | 609 |  |  | DB Search |
| L.WPR.K | N | 16.44 | 457.2437 | 3 | 4.14 | 458.2518 | 1 | 16.95 | 6653 | 0 | 0 | 0 | 557 | 559 |  |  | DB Search |
| D.DPTP.T | N | 16.05 | 428.1907 | 4 | 7.39 | 429.2001 | 1 | 11.23 | 4471 | 0 | 0 | 0 | 171 | 174 |  |  | DB Search |
| Y.HPVLT.E | Y | 15.96 | 565.3224 | 5 | -2.92 | 566.3266 | 1 | 14.68 | 5793 | 5.71e2 | 1 | 1 | 667 | 671 |  |  | DB Search |
| I.SELA.M | N | 15.6 | 418.2063 | 4 | -9.49 | 419.2086 | 1 | 4.93 | 1962 | 0 | 0 | 0 | 102 | 105 |  |  | DB Search |
| total 16 peptides |
| --- |

Best Unique PSM (Scan POS\_R15-1.wiff:5793, m/z=566.2762, z=2, RT=14.69, ppm=-5.41):


C4QVI1|C4QVI1\_KOMPG

back to list

  

| Protein Coverage
| Supporting Peptides
| Best Unique PSM
|

Protein Coverage:

Supporting Peptides:

| Peptide | Uniq | -10lgP | Mass | Length | ppm | m/z | z | RT | Scan | Area POS\_R15-1 | #Feature | #Feature POS\_R15-1 | Start | End | PTM | AScore | Found By |
| --- | --- | --- | --- | --- | --- | --- | --- | --- | --- | --- | --- | --- | --- | --- | --- | --- | --- |
| K.SVGV.M | N | 26.46 | 360.2009 | 4 | -6.45 | 361.2049 | 1 | 8.68 | 3331 | 1.41e4 | 1 | 1 | 444 | 447 |  |  | DB Search |
| N.VGGL.T | N | 24.87 | 344.2059 | 4 | -5.22 | 345.2106 | 1 | 10.74 | 4195 | 1.51e3 | 1 | 1 | 349 | 352 |  |  | DB Search |
| K.THH.N | N | 21.55 | 393.1761 | 3 | -4.54 | 394.1806 | 1 | 2.11 | 908 | 2.12e3 | 1 | 1 | 345 | 347 |  |  | DB Search |
| K.SVGVMGDQRT.Y | Y | 20.74 | 1048.4971 | 10 | 2.17 | 525.2557 | 2 | 28.12 | 9854 | 0 | 0 | 0 | 444 | 453 |  |  | DB Search |
| T.HSVQ.G | N | 17.66 | 469.2285 | 4 | 4.21 | 470.2366 | 1 | 15.47 | 6115 | 1.64e1 | 1 | 1 | 169 | 172 |  |  | DB Search |
| K.AGLY.R | N | 17.2 | 422.2165 | 4 | 3.26 | 423.2241 | 1 | 22.58 | 8294 | 5.26e2 | 1 | 1 | 426 | 429 |  |  | DB Search |
| I.SQAF.A | N | 16.62 | 451.2067 | 4 | -3.62 | 452.2112 | 1 | 10.42 | 4095 | 4.08e2 | 1 | 1 | 433 | 436 |  |  | DB Search |
| total 7 peptides |
| --- |

Best Unique PSM (Scan POS\_R15-1.wiff:9854, m/z=525.2557, z=2, RT=28.12, ppm=-0.31):


C4QYQ4|C4QYQ4\_KOMPG

back to list

  

| Protein Coverage
| Supporting Peptides
| Best Unique PSM
|

Protein Coverage:

Supporting Peptides:

| Peptide | Uniq | -10lgP | Mass | Length | ppm | m/z | z | RT | Scan | Area POS\_R15-1 | #Feature | #Feature POS\_R15-1 | Start | End | PTM | AScore | Found By |
| --- | --- | --- | --- | --- | --- | --- | --- | --- | --- | --- | --- | --- | --- | --- | --- | --- | --- |
| S.DRF.T | N | 24.14 | 436.207 | 3 | -6.05 | 437.2106 | 1 | 17.42 | 6833 | 5.05e2 | 1 | 1 | 435 | 437 |  |  | DB Search |
| N.SPT.V | N | 23.16 | 303.143 | 3 | 6.39 | 304.1515 | 1 | 7.14 | 2731 | 5.4e3 | 2 | 2 | 308 | 310 |  |  | DB Search |
| Q.PQQQ(+0.98)PQ(+0.98)QQPP.L | Y | 19.9 | 1176.5411 | 10 | 4.97 | 589.2793 | 2 | 24.08 | 8743 | 3.75e3 | 1 | 1 | 179 | 188 | Deamidation (NQ), Deamidation (NQ) | Q4:Deamidation (NQ):0 Q6:Deamidation (NQ):30.46 | DB Search |
| P.Q(-17.03)PLQPQ.P | Y | 19.23 | 692.3493 | 6 | 2.51 | 693.3566 | 1 | 13.59 | 5339 | 1.36e3 | 1 | 1 | 192 | 197 | Pyro-glu from Q | Q1:Pyro-glu from Q:1000 | DB Search |
| N.SNG.S | N | 19.07 | 276.107 | 3 | 1.61 | 277.114 | 1 | 16.82 | 6604 | 0 | 0 | 0 | 244 | 246 |  |  | DB Search |
| D.TPLT.T | N | 18.8 | 430.2427 | 4 | -8.61 | 431.2452 | 1 | 9.60 | 3908 | 8.62e3 | 1 | 1 | 326 | 329 |  |  | DB Search |
| P.Q(-17.03)QQPPL.Q | Y | 16.79 | 692.3493 | 6 | -4.89 | 693.3515 | 1 | 17.35 | 6859 | 3.63e3 | 1 | 1 | 184 | 189 | Pyro-glu from Q | Q1:Pyro-glu from Q:1000 | DB Search |
| S.NGST.P | N | 16.67 | 377.1547 | 4 | -5.39 | 378.159 | 1 | 16.22 | 6455 | 6.96e2 | 1 | 1 | 245 | 248 |  |  | DB Search |
| N.LSAL.D | N | 16.63 | 402.2478 | 4 | -8.97 | 403.2505 | 1 | 9.42 | 3655 | 1.65e3 | 1 | 1 | 410 | 413 |  |  | DB Search |
| P.Q(-17.03)PLQPQP.Q | Y | 16.59 | 789.4021 | 7 | -6.34 | 790.4024 | 1 | 19.40 | 7373 | 2.88e3 | 1 | 1 | 192 | 198 | Pyro-glu from Q | Q1:Pyro-glu from Q:1000 | DB Search |
| E.QAVE.L | N | 15.8 | 445.2172 | 4 | 7.06 | 446.2266 | 1 | 2.45 | 1067 | 0 | 0 | 0 | 84 | 87 |  |  | DB Search |
| L.NGV.D | N | 15.37 | 288.1434 | 3 | 8 | 289.1522 | 1 | 5.91 | 2301 | 6.8e3 | 1 | 1 | 154 | 156 |  |  | DB Search |
| P.Q(-17.03)QQPPLQ(+0.98)PQPLQ.P | Y | 15.11 | 1384.6987 | 12 | -8.07 | 693.3493 | 2 | 17.18 | 6762 | 0 | 0 | 0 | 184 | 195 | Pyro-glu from Q, Deamidation (NQ) | Q1:Pyro-glu from Q:1000 Q7:Deamidation (NQ):0 | DB Search |
| total 13 peptides |
| --- |

Best Unique PSM (Scan POS\_R15-1.wiff:8743, m/z=589.2805, z=2, RT=24.23, ppm=2.49):


C4QW10|C4QW10\_KOMPG

back to list

  

| Protein Coverage
| Supporting Peptides
| Best Unique PSM
|

Protein Coverage:

Supporting Peptides:

| Peptide | Uniq | -10lgP | Mass | Length | ppm | m/z | z | RT | Scan | Area POS\_R15-1 | #Feature | #Feature POS\_R15-1 | Start | End | PTM | AScore | Found By |
| --- | --- | --- | --- | --- | --- | --- | --- | --- | --- | --- | --- | --- | --- | --- | --- | --- | --- |
| S.IGGI.S | N | 27.34 | 358.2216 | 4 | -4.39 | 359.2264 | 1 | 15.45 | 6114 | 7.1e3 | 1 | 1 | 318 | 321 |  |  | DB Search |
| Q.AQLP.Q | N | 21.52 | 427.2431 | 4 | -3.04 | 428.248 | 1 | 11.76 | 4668 | 6.31e3 | 1 | 1 | 585 | 588 |  |  | DB Search |
| Q.LYSG.Y | N | 19.57 | 438.2114 | 4 | -3.08 | 439.2163 | 1 | 7.95 | 2941 | 3.53e4 | 1 | 1 | 472 | 475 |  |  | DB Search |
| P.RSTT.S | N | 18.45 | 463.2391 | 4 | -4.23 | 464.2432 | 1 | 17.11 | 6703 | 1.32e2 | 1 | 1 | 125 | 128 |  |  | DB Search |
| L.ERKQ(+0.98)QQQQQ.Q | Y | 18.13 | 1200.5847 | 9 | 1.35 | 601.299 | 2 | 10.82 | 4277 | 3.04e3 | 1 | 1 | 295 | 303 | Deamidation (NQ) | Q4:Deamidation (NQ):9.34 | DB Search |
| Q.EPKP.L | N | 17.69 | 469.2536 | 4 | -5.47 | 470.2572 | 1 | 15.43 | 6115 | 1.58e3 | 1 | 1 | 290 | 293 |  |  | DB Search |
| T.Q(-17.03)PHQ.P | N | 16.34 | 491.2128 | 4 | 0.63 | 492.2192 | 1 | 2.44 | 1064 | 0 | 0 | 0 | 487 | 490 | Pyro-glu from Q | Q1:Pyro-glu from Q:1000 | DB Search |
| T.Q(-17.03)PH.Q | N | 15.48 | 363.1543 | 3 | -0.14 | 364.1606 | 1 | 2.32 | 992 | 3.39e4 | 1 | 1 | 487 | 489 | Pyro-glu from Q | Q1:Pyro-glu from Q:1000 | DB Search |
| total 8 peptides |
| --- |

Best Unique PSM (Scan POS\_R15-1.wiff:4277, m/z=601.2992, z=2, RT=10.83, ppm=-1.14):


C4QV24|C4QV24\_KOMPG

back to list

  

| Protein Coverage
| Supporting Peptides
| Best Unique PSM
|

Protein Coverage:

Supporting Peptides:

| Peptide | Uniq | -10lgP | Mass | Length | ppm | m/z | z | RT | Scan | Area POS\_R15-1 | #Feature | #Feature POS\_R15-1 | Start | End | PTM | AScore | Found By |
| --- | --- | --- | --- | --- | --- | --- | --- | --- | --- | --- | --- | --- | --- | --- | --- | --- | --- |
| G.IGGL.I | N | 27.34 | 358.2216 | 4 | -4.39 | 359.2264 | 1 | 15.45 | 6114 | 7.1e3 | 1 | 1 | 285 | 288 |  |  | DB Search |
| I.YSLF.L | N | 26.31 | 528.2584 | 4 | -2.91 | 529.2628 | 1 | 34.97 | 11598 | 3.74e3 | 1 | 1 | 820 | 823 |  |  | DB Search |
| A.LGSI.H | N | 25.26 | 388.2322 | 4 | -3.81 | 389.237 | 1 | 9.87 | 3785 | 6.08e3 | 1 | 1 | 767 | 770 |  |  | DB Search |
| V.AIGI.S | N | 25.12 | 372.2372 | 4 | -5.1 | 373.2417 | 1 | 21.84 | 8058 | 0 | 0 | 0 | 988 | 991 |  |  | DB Search |
| A.ATLF.V | N | 23.38 | 450.2478 | 4 | -9.17 | 451.2498 | 1 | 24.44 | 8854 | 1.68e3 | 1 | 1 | 998 | 1001 |  |  | DB Search |
| V.NGSS.S | N | 20.12 | 363.139 | 4 | -0.16 | 364.1453 | 1 | 14.82 | 5815 | 1.67e3 | 1 | 1 | 361 | 364 |  |  | DB Search |
| G.ISVGL.F | N | 20.08 | 487.3006 | 5 | -4.27 | 488.3046 | 1 | 21.68 | 7886 | 8.06e3 | 1 | 1 | 991 | 995 |  |  | DB Search |
| N.RSSS.R | N | 18.8 | 435.2078 | 4 | -3.98 | 436.2122 | 1 | 11.38 | 4548 | 7.31e2 | 1 | 1 | 69 | 72 |  |  | DB Search |
| F.SVGL.A | N | 18.49 | 374.2165 | 4 | -4.96 | 375.221 | 1 | 14.62 | 5748 | 1.93e2 | 1 | 1 | 865 | 868 |  |  | DB Search |
| V.AIGISVGL.F | Y | 18.33 | 728.4432 | 8 | -5.29 | 729.4448 | 1 | 33.44 | 11260 | 1.18e3 | 1 | 1 | 988 | 995 |  |  | DB Search |
| N.LYSS.V | N | 17.99 | 468.222 | 4 | 0.32 | 469.2283 | 1 | 16.06 | 6398 | 1.94e3 | 1 | 1 | 747 | 750 |  |  | DB Search |
| G.MAVT.S | N | 17.82 | 420.2042 | 4 | 2.83 | 421.2117 | 1 | 8.16 | 3057 | 1.44e3 | 1 | 1 | 929 | 932 |  |  | DB Search |
| V.QVAI.G | N | 15.07 | 429.2587 | 4 | 5.6 | 430.2673 | 1 | 21.95 | 8124 | 0 | 0 | 0 | 986 | 989 |  |  | DB Search |
| L.YSSV.F | N | 15.06 | 454.2064 | 4 | -3.39 | 455.211 | 1 | 23.68 | 8615 | 0 | 0 | 0 | 748 | 751 |  |  | DB Search |
| T.NNSV.C | N | 15.01 | 432.1969 | 4 | 8.48 | 433.2067 | 1 | 7.60 | 2865 | 3.02e3 | 1 | 1 | 844 | 847 |  |  | DB Search |
| total 15 peptides |
| --- |

Best Unique PSM (Scan POS\_R15-1.wiff:11260, m/z=729.4448, z=1, RT=33.44, ppm=-7.77):


C4QZJ3|C4QZJ3\_KOMPG

back to list

  

| Protein Coverage
| Supporting Peptides
| Best Unique PSM
|

Protein Coverage:

Supporting Peptides:

| Peptide | Uniq | -10lgP | Mass | Length | ppm | m/z | z | RT | Scan | Area POS\_R15-1 | #Feature | #Feature POS\_R15-1 | Start | End | PTM | AScore | Found By |
| --- | --- | --- | --- | --- | --- | --- | --- | --- | --- | --- | --- | --- | --- | --- | --- | --- | --- |
| I.SPLI.L | N | 26.66 | 428.2635 | 4 | -1.63 | 429.269 | 1 | 21.35 | 7905 | 0 | 0 | 0 | 262 | 265 |  |  | DB Search |
| Q.HFT.T | N | 23.64 | 403.1856 | 3 | -0.83 | 404.1915 | 1 | 13.94 | 5543 | 0 | 0 | 0 | 292 | 294 |  |  | DB Search |
| Q.TLDL.N | N | 22.27 | 460.2533 | 4 | -8.03 | 461.2557 | 1 | 9.41 | 3625 | 2.97e2 | 1 | 1 | 902 | 905 |  |  | DB Search |
| Q.ELW.M | N | 21.42 | 446.2165 | 3 | -5.13 | 447.2204 | 1 | 31.30 | 10711 | 1.59e3 | 1 | 1 | 1302 | 1304 |  |  | DB Search |
| L.RCL.N | N | 20.6 | 390.2049 | 3 | -4.41 | 391.2095 | 1 | 8.08 | 3003 | 0 | 0 | 0 | 1445 | 1447 |  |  | DB Search |
| G.LCSLYSSQF.L | Y | 19.92 | 1046.4743 | 9 | -2.99 | 1047.4758 | 1 | 35.32 | 11768 | 1.02e2 | 2 | 2 | 1044 | 1052 |  |  | DB Search |
| G.LTVA.K | N | 19.62 | 402.2478 | 4 | -4.79 | 403.2522 | 1 | 10.65 | 4156 | 4.9e3 | 1 | 1 | 400 | 403 |  |  | DB Search |
| R.SVGL.E | N | 18.49 | 374.2165 | 4 | -4.96 | 375.221 | 1 | 14.62 | 5748 | 1.93e2 | 1 | 1 | 1293 | 1296 |  |  | DB Search |
| F.LYSS.L | N | 17.99 | 468.222 | 4 | 0.32 | 469.2283 | 1 | 16.06 | 6398 | 1.94e3 | 1 | 1 | 479 | 482 |  |  | DB Search |
| F.LITD.S | N | 17.52 | 460.2533 | 4 | -9.09 | 461.2552 | 1 | 10.16 | 3965 | 9.46e2 | 1 | 1 | 746 | 749 |  |  | DB Search |
| A.ESW.S | N | 17.43 | 420.1645 | 3 | -0.16 | 421.1707 | 1 | 11.21 | 4460 | 0 | 0 | 0 | 825 | 827 |  |  | DB Search |
| W.LDYF.L | N | 16.91 | 556.2533 | 4 | -2.81 | 557.2576 | 1 | 23.71 | 8633 | 4.27e1 | 1 | 1 | 607 | 610 |  |  | DB Search |
| L.DAPF.Q | N | 15.82 | 448.1958 | 4 | -3.76 | 449.2003 | 1 | 15.12 | 5980 | 8.13e2 | 1 | 1 | 835 | 838 |  |  | DB Search |
| K.TIQW.L | N | 15.55 | 546.2802 | 4 | 0.72 | 547.2865 | 1 | 22.01 | 8160 | 8.82e1 | 1 | 1 | 603 | 606 |  |  | DB Search |
| total 14 peptides |
| --- |

Best Unique PSM (Scan POS\_R15-1.wiff:11768, m/z=1047.4869, z=1, RT=35.42, ppm=-5.47):


C4R614|C4R614\_KOMPG

back to list

  

| Protein Coverage
| Supporting Peptides
| Best Unique PSM
|

Protein Coverage:

Supporting Peptides:

| Peptide | Uniq | -10lgP | Mass | Length | ppm | m/z | z | RT | Scan | Area POS\_R15-1 | #Feature | #Feature POS\_R15-1 | Start | End | PTM | AScore | Found By |
| --- | --- | --- | --- | --- | --- | --- | --- | --- | --- | --- | --- | --- | --- | --- | --- | --- | --- |
| N.ITGL.S | N | 27.55 | 402.2478 | 4 | -1.76 | 403.2534 | 1 | 15.04 | 5964 | 1.56e4 | 2 | 2 | 381 | 384 |  |  | DB Search |
| W.ALGI.F | N | 25.12 | 372.2372 | 4 | -5.1 | 373.2417 | 1 | 21.84 | 8058 | 0 | 0 | 0 | 228 | 231 |  |  | DB Search |
| K.VTVI.Q | N | 21.25 | 430.2791 | 4 | -6.35 | 431.2826 | 1 | 27.75 | 9755 | 1.56e3 | 1 | 1 | 23 | 26 |  |  | DB Search |
| L.PGSAQSY.E | Y | 19.96 | 708.3079 | 7 | -3.22 | 709.3111 | 1 | 6.91 | 2679 | 2.49e3 | 1 | 1 | 96 | 102 |  |  | DB Search |
| R.VIVP.D | N | 18.9 | 426.2842 | 4 | -1.74 | 427.2897 | 1 | 17.76 | 6878 | 3.8e3 | 1 | 1 | 57 | 60 |  |  | DB Search |
| N.EFLA.L | N | 18.15 | 478.2427 | 4 | -2.38 | 479.2477 | 1 | 20.03 | 7568 | 1.11e3 | 2 | 2 | 456 | 459 |  |  | DB Search |
| L.AVLE.K | N | 15.33 | 430.2427 | 4 | -8.59 | 431.2452 | 1 | 9.60 | 3778 | 8.62e3 | 1 | 1 | 5 | 8 |  |  | DB Search |
| total 7 peptides |
| --- |

Best Unique PSM (Scan POS\_R15-1.wiff:2679, m/z=709.2588, z=1, RT=7.02, ppm=-5.7):


C4R460|C4R460\_KOMPG

back to list

  

| Protein Coverage
| Supporting Peptides
| Best Unique PSM
|

Protein Coverage:

Supporting Peptides:

| Peptide | Uniq | -10lgP | Mass | Length | ppm | m/z | z | RT | Scan | Area POS\_R15-1 | #Feature | #Feature POS\_R15-1 | Start | End | PTM | AScore | Found By |
| --- | --- | --- | --- | --- | --- | --- | --- | --- | --- | --- | --- | --- | --- | --- | --- | --- | --- |
| A.SPT.T | N | 23.16 | 303.143 | 3 | 6.39 | 304.1515 | 1 | 7.14 | 2731 | 5.4e3 | 2 | 2 | 34 | 36 |  |  | DB Search |
| G.YGR.I | N | 21.6 | 394.1965 | 3 | -4.25 | 395.2011 | 1 | 5.35 | 1961 | 1.67e4 | 1 | 1 | 499 | 501 |  |  | DB Search |
| S.QFF.I | N | 19.56 | 440.206 | 3 | 3.92 | 441.2139 | 1 | 19.15 | 7313 | 0 | 0 | 0 | 63 | 65 |  |  | DB Search |
| I.EAY.G | N | 19.24 | 381.1536 | 3 | -5.14 | 382.158 | 1 | 5.09 | 2057 | 2.63e3 | 1 | 1 | 49 | 51 |  |  | DB Search |
| E.LGPV.K | N | 18.01 | 384.2372 | 4 | -2.96 | 385.2424 | 1 | 11.56 | 4504 | 2.76e3 | 1 | 1 | 527 | 530 |  |  | DB Search |
| E.AGIY.V | N | 17.2 | 422.2165 | 4 | 3.26 | 423.2241 | 1 | 22.58 | 8294 | 5.26e2 | 1 | 1 | 153 | 156 |  |  | DB Search |
| M.APY.D | N | 16.84 | 349.1638 | 3 | -4.81 | 350.1685 | 1 | 16.32 | 6384 | 1.41e3 | 1 | 1 | 191 | 193 |  |  | DB Search |
| T.LEMAGY.P | Y | 16.51 | 682.2996 | 6 | 7.86 | 683.3105 | 1 | 3.00 | 1298 | 0 | 0 | 0 | 293 | 298 |  |  | DB Search |
| R.SELA.E | N | 15.6 | 418.2063 | 4 | -9.49 | 419.2086 | 1 | 4.93 | 1962 | 0 | 0 | 0 | 437 | 440 |  |  | DB Search |
| A.WPDICT.R | Y | 15.29 | 733.3105 | 6 | 0.37 | 734.3162 | 1 | 17.34 | 6811 | 2.9e2 | 1 | 1 | 113 | 118 |  |  | DB Search |
| total 10 peptides |
| --- |

Best Unique PSM (Scan POS\_R15-1.wiff:1298, m/z=683.3105, z=1, RT=3.00, ppm=5.38):


C4R5D6|C4R5D6\_KOMPG

back to list

  

| Protein Coverage
| Supporting Peptides
| Best Unique PSM
|

Protein Coverage:

Supporting Peptides:

| Peptide | Uniq | -10lgP | Mass | Length | ppm | m/z | z | RT | Scan | Area POS\_R15-1 | #Feature | #Feature POS\_R15-1 | Start | End | PTM | AScore | Found By |
| --- | --- | --- | --- | --- | --- | --- | --- | --- | --- | --- | --- | --- | --- | --- | --- | --- | --- |
| L.NGI.L | N | 28.25 | 302.159 | 3 | -3.76 | 303.1644 | 1 | 6.06 | 2374 | 3.14e3 | 1 | 1 | 198 | 200 |  |  | DB Search |
| G.NIL.N | N | 28.22 | 358.2216 | 3 | -4.68 | 359.2263 | 1 | 19.18 | 7326 | 4.19e3 | 1 | 1 | 195 | 197 |  |  | DB Search |
| P.LLLP.Y | N | 24.07 | 454.3155 | 4 | -2.4 | 455.3206 | 1 | 34.52 | 11565 | 2.64e3 | 1 | 1 | 177 | 180 |  |  | DB Search |
| D.EW.D | N | 22.33 | 333.1325 | 2 | -3.27 | 334.1378 | 1 | 12.76 | 5036 | 2.08e4 | 1 | 1 | 183 | 184 |  |  | DB Search |
| F.EV.Y | N | 22.06 | 246.1216 | 2 | -3.05 | 247.1275 | 1 | 3.37 | 1479 | 1.39e4 | 2 | 2 | 105 | 106 |  |  | DB Search |
| Q.VGSL.Q | N | 21.91 | 374.2165 | 4 | 0.34 | 375.223 | 1 | 13.82 | 5444 | 2.07e3 | 1 | 1 | 39 | 42 |  |  | DB Search |
| S.EAY.M | N | 19.24 | 381.1536 | 3 | -5.14 | 382.158 | 1 | 5.09 | 2057 | 2.63e3 | 1 | 1 | 211 | 213 |  |  | DB Search |
| Y.EM.L | N | 19.13 | 278.0936 | 2 | 0.1 | 279.1003 | 1 | 3.52 | 1504 | 7.14e3 | 1 | 1 | 108 | 109 |  |  | DB Search |
| I.SNG.I | N | 19.07 | 276.107 | 3 | 1.61 | 277.114 | 1 | 16.82 | 6604 | 0 | 0 | 0 | 337 | 339 |  |  | DB Search |
| P.RT.Q | N | 19.04 | 275.1593 | 2 | 3.6 | 276.1669 | 1 | 5.27 | 2115 | 2.64e3 | 1 | 1 | 161 | 162 |  |  | DB Search |
| D.PR.T | N | 18.98 | 271.1644 | 2 | -8.93 | 272.1686 | 1 | 5.03 | 2023 | 0 | 0 | 0 | 160 | 161 |  |  | DB Search |
| I.ELG.C | N | 18.95 | 317.1587 | 3 | -4.31 | 318.1638 | 1 | 5.49 | 2177 | 4.11e3 | 1 | 1 | 220 | 222 |  |  | DB Search |
| K.IRV.V | N | 18.9 | 386.2641 | 3 | -1.28 | 387.27 | 1 | 27.77 | 9760 | 0 | 0 | 0 | 8 | 10 |  |  | DB Search |
| Q.LP.A | N | 18.63 | 228.1474 | 2 | -2.29 | 229.1536 | 1 | 2.07 | 883 | 8.16e3 | 1 | 1 | 46 | 47 |  |  | DB Search |
| K.IR.V | N | 18.3 | 287.1957 | 2 | 0.04 | 288.2023 | 1 | 2.10 | 904 | 0 | 0 | 0 | 8 | 9 |  |  | DB Search |
| C.ITP.L | N | 17.99 | 329.1951 | 3 | 1.36 | 330.202 | 1 | 7.49 | 2815 | 1.47e3 | 1 | 1 | 174 | 176 |  |  | DB Search |
| W.YP.G | N | 17.92 | 278.1266 | 2 | -4.14 | 279.1321 | 1 | 3.49 | 1522 | 2.89e2 | 1 | 1 | 75 | 76 |  |  | DB Search |
| T.HP.A | N | 17.77 | 252.1222 | 2 | -2.49 | 253.1283 | 1 | 5.54 | 2216 | 1.25e4 | 1 | 1 | 154 | 155 |  |  | DB Search |
| E.IITD.C | N | 17.52 | 460.2533 | 4 | -9.09 | 461.2552 | 1 | 10.16 | 3965 | 9.46e2 | 1 | 1 | 169 | 172 |  |  | DB Search |
| S.TV.A | N | 16.54 | 218.1266 | 2 | -6.32 | 219.132 | 1 | 6.06 | 2461 | 9.87e2 | 1 | 1 | 187 | 188 |  |  | DB Search |
| D.TFT.I | N | 15.92 | 367.1743 | 3 | -7.47 | 368.1779 | 1 | 8.54 | 3263 | 0 | 0 | 0 | 114 | 116 |  |  | DB Search |
| Q.VGSLQDQ.L | Y | 15.54 | 745.3606 | 7 | 4.73 | 746.3696 | 1 | 22.39 | 8253 | 2.59e2 | 1 | 1 | 39 | 45 |  |  | DB Search |
| L.FRG.R | N | 15.51 | 378.2015 | 3 | -9.17 | 379.2044 | 1 | 3.37 | 1492 | 1.94e3 | 1 | 1 | 34 | 36 |  |  | DB Search |
| total 23 peptides |
| --- |

Best Unique PSM (Scan POS\_R15-1.wiff:8253, m/z=746.3686, z=2, RT=22.42, ppm=2.24):


C4QWP7|C4QWP7\_KOMPG

back to list

  

| Protein Coverage
| Supporting Peptides
| Best Unique PSM
|

Protein Coverage:

Supporting Peptides:

| Peptide | Uniq | -10lgP | Mass | Length | ppm | m/z | z | RT | Scan | Area POS\_R15-1 | #Feature | #Feature POS\_R15-1 | Start | End | PTM | AScore | Found By |
| --- | --- | --- | --- | --- | --- | --- | --- | --- | --- | --- | --- | --- | --- | --- | --- | --- | --- |
| D.ITGL.R | N | 27.55 | 402.2478 | 4 | -1.76 | 403.2534 | 1 | 15.04 | 5964 | 1.56e4 | 2 | 2 | 166 | 169 |  |  | DB Search |
| E.SGVL.I | N | 21.93 | 374.2165 | 4 | -4.88 | 375.221 | 1 | 11.77 | 4666 | 3.28e3 | 1 | 1 | 549 | 552 |  |  | DB Search |
| S.YNP.A | N | 21.45 | 392.1696 | 3 | -3.75 | 393.1744 | 1 | 8.45 | 3226 | 1.39e2 | 1 | 1 | 382 | 384 |  |  | DB Search |
| A.KFH.P | N | 20.21 | 430.2328 | 3 | 9.29 | 431.243 | 1 | 12.31 | 5009 | 5.31e3 | 1 | 1 | 144 | 146 |  |  | DB Search |
| P.AHSVHQ.D | Y | 19.46 | 677.3245 | 6 | 4.29 | 678.333 | 1 | 15.22 | 6028 | 1.41e2 | 1 | 1 | 333 | 338 |  |  | DB Search |
| K.KGY.P | N | 19.27 | 366.1903 | 3 | -9.39 | 367.1932 | 1 | 6.66 | 2561 | 3.55e1 | 1 | 1 | 643 | 645 |  |  | DB Search |
| S.KHT.I | N | 18.78 | 384.2121 | 3 | -3.61 | 385.217 | 1 | 6.37 | 2316 | 2.15e3 | 1 | 1 | 519 | 521 |  |  | DB Search |
| A.GTFT.P | N | 18.77 | 424.1958 | 4 | -4.93 | 425.1999 | 1 | 10.09 | 3929 | 0 | 0 | 0 | 1157 | 1160 |  |  | DB Search |
| V.YAGP.G | N | 18.55 | 406.1852 | 4 | -7.11 | 407.1886 | 1 | 10.23 | 4013 | 0 | 0 | 0 | 469 | 472 |  |  | DB Search |
| K.DVVY.A | N | 17.92 | 494.2376 | 4 | -7.07 | 495.2402 | 1 | 12.58 | 4967 | 1.85e3 | 1 | 1 | 466 | 469 |  |  | DB Search |
| Q.QAIE.E | N | 17.59 | 459.2329 | 4 | -5.43 | 460.2365 | 1 | 4.85 | 1909 | 7.42e3 | 2 | 2 | 1013 | 1016 |  |  | DB Search |
| D.TCR.G | N | 17.37 | 378.1685 | 3 | 5.06 | 379.1768 | 1 | 12.47 | 4890 | 0 | 0 | 0 | 252 | 254 |  |  | DB Search |
| D.LSAI.V | N | 16.63 | 402.2478 | 4 | -8.97 | 403.2505 | 1 | 9.42 | 3655 | 1.65e3 | 1 | 1 | 740 | 743 |  |  | DB Search |
| D.HDGP.V | N | 16.44 | 424.1706 | 4 | 3.23 | 425.1782 | 1 | 7.35 | 2793 | 1.25e2 | 1 | 1 | 52 | 55 |  |  | DB Search |
| D.ADNAYY.A | Y | 16.33 | 715.2813 | 6 | -3.33 | 716.2844 | 1 | 11.08 | 4381 | 1.41e3 | 1 | 1 | 396 | 401 |  |  | DB Search |
| E.RPAH.S | N | 16.09 | 479.2604 | 4 | -9.47 | 480.262 | 1 | 12.28 | 4787 | 0 | 0 | 0 | 331 | 334 |  |  | DB Search |
| G.TFT.P | N | 15.92 | 367.1743 | 3 | -7.47 | 368.1779 | 1 | 8.54 | 3263 | 0 | 0 | 0 | 1158 | 1160 |  |  | DB Search |
| L.NNSV.T | N | 15.01 | 432.1969 | 4 | 8.48 | 433.2067 | 1 | 7.60 | 2865 | 3.02e3 | 1 | 1 | 451 | 454 |  |  | DB Search |
| total 18 peptides |
| --- |

Best Unique PSM (Scan POS\_R15-1.wiff:6028, m/z=678.333, z=1, RT=15.22, ppm=1.81):


C4R1V8|C4R1V8\_KOMPG

back to list

  

| Protein Coverage
| Supporting Peptides
| Best Unique PSM
|

Protein Coverage:

Supporting Peptides:

| Peptide | Uniq | -10lgP | Mass | Length | ppm | m/z | z | RT | Scan | Area POS\_R15-1 | #Feature | #Feature POS\_R15-1 | Start | End | PTM | AScore | Found By |
| --- | --- | --- | --- | --- | --- | --- | --- | --- | --- | --- | --- | --- | --- | --- | --- | --- | --- |
| T.SPLL.I | N | 26.66 | 428.2635 | 4 | -1.63 | 429.269 | 1 | 21.35 | 7905 | 0 | 0 | 0 | 57 | 60 |  |  | DB Search |
| Y.AIGL.V | N | 25.12 | 372.2372 | 4 | -5.1 | 373.2417 | 1 | 21.84 | 8058 | 0 | 0 | 0 | 348 | 351 |  |  | DB Search |
| I.MLLTT.Q | N | 22.82 | 577.3145 | 5 | -6.02 | 578.3169 | 1 | 21.75 | 8004 | 0 | 0 | 0 | 196 | 200 |  |  | DB Search |
| S.VVDI.S | N | 19.9 | 444.2584 | 4 | 0.77 | 445.2649 | 1 | 17.32 | 6776 | 2.15e3 | 1 | 1 | 80 | 83 |  |  | DB Search |
| I.TILNLI.N | N | 19.02 | 685.4374 | 6 | 3.49 | 686.4454 | 1 | 22.02 | 8163 | 0 | 0 | 0 | 62 | 67 |  |  | DB Search |
| C.LSAI.F | N | 16.63 | 402.2478 | 4 | -8.97 | 403.2505 | 1 | 9.42 | 3655 | 1.65e3 | 1 | 1 | 145 | 148 |  |  | DB Search |
| T.NSYKQ.L | Y | 15.83 | 638.3024 | 5 | 6.45 | 639.3122 | 1 | 9.41 | 3632 | 4.62e2 | 1 | 1 | 36 | 40 |  |  | DB Search |
| total 7 peptides |
| --- |

Best Unique PSM (Scan POS\_R15-1.wiff:3632, m/z=640.3221, z=2, RT=9.41, ppm=3.96):


C4R1M3|C4R1M3\_KOMPG

back to list

  

| Protein Coverage
| Supporting Peptides
| Best Unique PSM
|

Protein Coverage:

Supporting Peptides:

| Peptide | Uniq | -10lgP | Mass | Length | ppm | m/z | z | RT | Scan | Area POS\_R15-1 | #Feature | #Feature POS\_R15-1 | Start | End | PTM | AScore | Found By |
| --- | --- | --- | --- | --- | --- | --- | --- | --- | --- | --- | --- | --- | --- | --- | --- | --- | --- |
| A.ATLF.Y | N | 23.38 | 450.2478 | 4 | -9.17 | 451.2498 | 1 | 24.44 | 8854 | 1.68e3 | 1 | 1 | 46 | 49 |  |  | DB Search |
| L.LGVSVA.A | Y | 18.89 | 544.322 | 6 | -8.22 | 545.3235 | 1 | 12.67 | 5012 | 1.39e3 | 1 | 1 | 40 | 45 |  |  | DB Search |
| total 2 peptides |
| --- |

Best Unique PSM (Scan POS\_R15-1.wiff:5012, m/z=545.3235, z=1, RT=12.67, ppm=-10.71):


C4R192|VPS10\_KOMPG

back to list

  

| Protein Coverage
| Supporting Peptides
| Best Unique PSM
|

Protein Coverage:

Supporting Peptides:

| Peptide | Uniq | -10lgP | Mass | Length | ppm | m/z | z | RT | Scan | Area POS\_R15-1 | #Feature | #Feature POS\_R15-1 | Start | End | PTM | AScore | Found By |
| --- | --- | --- | --- | --- | --- | --- | --- | --- | --- | --- | --- | --- | --- | --- | --- | --- | --- |
| G.ETW.F | N | 26.2 | 434.1801 | 3 | -4.77 | 435.1843 | 1 | 12.45 | 4871 | 1.27e3 | 1 | 1 | 1176 | 1178 |  |  | DB Search |
| I.DLGF.N | N | 21.22 | 450.2114 | 4 | 2.45 | 451.2187 | 1 | 25.55 | 9169 | 5.5e2 | 1 | 1 | 497 | 500 |  |  | DB Search |
| H.AFR.A | N | 19.1 | 392.2172 | 3 | -7.11 | 393.2207 | 1 | 6.33 | 2475 | 0 | 0 | 0 | 1455 | 1457 |  |  | DB Search |
| T.IDFS.K | N | 18.2 | 480.222 | 4 | -2.08 | 481.2271 | 1 | 16.69 | 6580 | 0 | 0 | 0 | 1223 | 1226 |  |  | DB Search |
| G.VAVG.V | N | 18.17 | 344.2059 | 4 | -3.72 | 345.2111 | 1 | 5.51 | 2206 | 3.05e3 | 1 | 1 | 503 | 506 |  |  | DB Search |
| N.INFD.S | N | 17.97 | 507.2329 | 4 | -4.86 | 508.2365 | 1 | 16.93 | 6638 | 1.73e3 | 1 | 1 | 1537 | 1540 |  |  | DB Search |
| G.FGNYGN(+0.98)IILC(-1.01).C | Y | 16.83 | 1112.5086 | 10 | 0.95 | 557.2607 | 2 | 19.18 | 7328 | 0 | 0 | 0 | 460 | 469 | Deamidation (NQ), Half of a disulfide bridge | N6:Deamidation (NQ):3.29 C10:Half of a disulfide bridge:1000 | DB Search |
| F.SKVH.Q | N | 16.42 | 469.2649 | 4 | -2.56 | 470.2698 | 1 | 1.09 | 269 | 3.02e4 | 1 | 1 | 1226 | 1229 |  |  | DB Search |
| G.TKC.K | N | 16.14 | 350.1624 | 3 | 7.67 | 351.1715 | 1 | 4.93 | 1958 | 0 | 0 | 0 | 662 | 664 |  |  | DB Search |
| V.AVGV.L | N | 15.55 | 344.2059 | 4 | 0.19 | 345.2124 | 1 | 5.48 | 2189 | 3.05e3 | 1 | 1 | 504 | 507 |  |  | DB Search |
| total 10 peptides |
| --- |

Best Unique PSM (Scan POS\_R15-1.wiff:7328, m/z=557.2607, z=2, RT=19.18, ppm=-1.53):


C4R6W9|C4R6W9\_KOMPG

back to list

  

| Protein Coverage
| Supporting Peptides
| Best Unique PSM
|

Protein Coverage:

Supporting Peptides:

| Peptide | Uniq | -10lgP | Mass | Length | ppm | m/z | z | RT | Scan | Area POS\_R15-1 | #Feature | #Feature POS\_R15-1 | Start | End | PTM | AScore | Found By |
| --- | --- | --- | --- | --- | --- | --- | --- | --- | --- | --- | --- | --- | --- | --- | --- | --- | --- |
| A.VKLDVLQTL.N | Y | 35.97 | 1027.6277 | 9 | -9.38 | 1028.6228 | 1 | 42.64 | 13414 | 1.91e2 | 1 | 1 | 261 | 269 |  |  | DB Search |
| A.VKLDVLQT.L | Y | 25.73 | 914.5437 | 8 | -8.3 | 915.5411 | 1 | 31.10 | 10647 | 0 | 0 | 0 | 261 | 268 |  |  | DB Search |
| L.ELVR.N | N | 23.24 | 515.3067 | 4 | -4.7 | 516.3103 | 1 | 11.12 | 4401 | 1.13e4 | 1 | 1 | 531 | 534 |  |  | DB Search |
| E.TIDL.Q | N | 22.27 | 460.2533 | 4 | -8.03 | 461.2557 | 1 | 9.41 | 3625 | 2.97e2 | 1 | 1 | 758 | 761 |  |  | DB Search |
| L.IAEV.S | N | 19.27 | 430.2427 | 4 | 1.69 | 431.2497 | 1 | 13.56 | 5318 | 0 | 0 | 0 | 816 | 819 |  |  | DB Search |
| L.NLTI.E | N | 19.19 | 459.2693 | 4 | -0.54 | 460.2752 | 1 | 17.07 | 6712 | 2.02e3 | 1 | 1 | 915 | 918 |  |  | DB Search |
| T.STSP.A | N | 19.15 | 390.1751 | 4 | -0.08 | 391.1813 | 1 | 5.92 | 2360 | 3.31e2 | 1 | 1 | 256 | 259 |  |  | DB Search |
| S.KHF.E | N | 17.4 | 430.2328 | 3 | 0.78 | 431.2394 | 1 | 20.46 | 7703 | 1.69e2 | 1 | 1 | 720 | 722 |  |  | DB Search |
| S.SSEI.D | N | 17.13 | 434.2013 | 4 | -0.44 | 435.2073 | 1 | 2.87 | 1206 | 7.24e2 | 1 | 1 | 525 | 528 |  |  | DB Search |
| E.VLPV.L | N | 17 | 426.2842 | 4 | -1.74 | 427.2897 | 1 | 17.76 | 6936 | 3.8e3 | 1 | 1 | 387 | 390 |  |  | DB Search |
| K.ESPV.Y | N | 16.59 | 430.2063 | 4 | 5.53 | 431.2149 | 1 | 12.65 | 5009 | 0 | 0 | 0 | 618 | 621 |  |  | DB Search |
| K.LTSTSP.A | N | 15.83 | 604.3068 | 6 | -3.86 | 605.3102 | 1 | 9.28 | 3584 | 7.41e2 | 2 | 2 | 254 | 259 |  |  | DB Search |
| I.VELA.D | N | 15.81 | 430.2427 | 4 | -4.19 | 431.2471 | 1 | 15.22 | 6032 | 0 | 0 | 0 | 305 | 308 |  |  | DB Search |
| K.AVIE.K | N | 15.33 | 430.2427 | 4 | -8.59 | 431.2452 | 1 | 9.60 | 3778 | 8.62e3 | 1 | 1 | 27 | 30 |  |  | DB Search |
| total 14 peptides |
| --- |

Best Unique PSM (Scan POS\_R15-1.wiff:13414, m/z=1028.6228, z=1, RT=42.64, ppm=-11.86):


C4QW41|C4QW41\_KOMPG

back to list

  

| Protein Coverage
| Supporting Peptides
| Best Unique PSM
|

Protein Coverage:

Supporting Peptides:

| Peptide | Uniq | -10lgP | Mass | Length | ppm | m/z | z | RT | Scan | Area POS\_R15-1 | #Feature | #Feature POS\_R15-1 | Start | End | PTM | AScore | Found By |
| --- | --- | --- | --- | --- | --- | --- | --- | --- | --- | --- | --- | --- | --- | --- | --- | --- | --- |
| H.LSLE.Q | N | 25.68 | 460.2533 | 4 | -2.19 | 461.2584 | 1 | 13.54 | 5303 | 1.35e4 | 1 | 1 | 291 | 294 |  |  | DB Search |
| F.LLIP.K | N | 24.07 | 454.3155 | 4 | -2.4 | 455.3206 | 1 | 34.52 | 11565 | 2.64e3 | 1 | 1 | 120 | 123 |  |  | DB Search |
| V.SPT.R | N | 23.16 | 303.143 | 3 | 6.39 | 304.1515 | 1 | 7.14 | 2731 | 5.4e3 | 2 | 2 | 132 | 134 |  |  | DB Search |
| A.EW.A | N | 22.33 | 333.1325 | 2 | -3.27 | 334.1378 | 1 | 12.76 | 5036 | 2.08e4 | 1 | 1 | 155 | 156 |  |  | DB Search |
| A.VLLP.T | N | 22.17 | 440.2998 | 4 | -5.96 | 441.3034 | 1 | 30.99 | 10567 | 1.25e3 | 1 | 1 | 475 | 478 |  |  | DB Search |
| I.SSLF.R | N | 21.63 | 452.2271 | 4 | -3.7 | 453.2316 | 1 | 22.49 | 8262 | 1.33e3 | 1 | 1 | 231 | 234 |  |  | DB Search |
| S.SSKSS.S | N | 21.18 | 494.2336 | 5 | -2.91 | 495.2383 | 1 | 12.41 | 4849 | 0 | 0 | 0 | 530 | 534 |  |  | DB Search |
| L.KYT.G | N | 21.18 | 410.2165 | 3 | -4.93 | 411.2208 | 1 | 12.54 | 4954 | 3.83e2 | 1 | 1 | 503 | 505 |  |  | DB Search |
| E.WAN.L | N | 21.03 | 389.1699 | 3 | 5.94 | 390.1785 | 1 | 10.28 | 4047 | 1.16e3 | 1 | 1 | 156 | 158 |  |  | DB Search |
| K.KGY.G | N | 19.27 | 366.1903 | 3 | -9.39 | 367.1932 | 1 | 6.66 | 2561 | 3.55e1 | 1 | 1 | 247 | 249 |  |  | DB Search |
| L.ELG.I | N | 18.95 | 317.1587 | 3 | -4.31 | 318.1638 | 1 | 5.49 | 2177 | 4.11e3 | 1 | 1 | 227 | 229 |  |  | DB Search |
| L.RSSS.K | N | 18.8 | 435.2078 | 4 | -3.98 | 436.2122 | 1 | 11.38 | 4548 | 7.31e2 | 1 | 1 | 528 | 531 |  |  | DB Search |
| F.SFY.M | N | 18.1 | 415.1743 | 3 | 6.32 | 416.1832 | 1 | 8.32 | 3151 | 0 | 0 | 0 | 12 | 14 |  |  | DB Search |
| I.HSSEESLSS.I | Y | 17.89 | 961.3989 | 9 | 8.75 | 481.7097 | 2 | 16.66 | 6566 | 0 | 0 | 0 | 571 | 579 |  |  | DB Search |
| N.HP.P | N | 17.77 | 252.1222 | 2 | -2.49 | 253.1283 | 1 | 5.54 | 2216 | 1.25e4 | 1 | 1 | 554 | 555 |  |  | DB Search |
| D.E(-18.01)PR.I | N | 16.87 | 382.1965 | 3 | -0.37 | 383.2026 | 1 | 2.61 | 1082 | 1.62e4 | 1 | 1 | 329 | 331 | Pyro-glu from E | E1:Pyro-glu from E:1000 | DB Search |
| F.APY.E | N | 16.84 | 349.1638 | 3 | -4.81 | 350.1685 | 1 | 16.32 | 6384 | 1.41e3 | 1 | 1 | 258 | 260 |  |  | DB Search |
| Q.EGS.Q | N | 15.77 | 291.1066 | 3 | 7.44 | 292.1154 | 1 | 3.53 | 1538 | 0 | 0 | 0 | 397 | 399 |  |  | DB Search |
| P.EALQ.F | N | 15.76 | 459.2329 | 4 | -3.37 | 460.2375 | 1 | 4.47 | 1741 | 3.16e3 | 2 | 2 | 126 | 129 |  |  | DB Search |
| N.AEP.Q | N | 15.46 | 315.143 | 3 | -3.02 | 316.1486 | 1 | 2.92 | 1198 | 1.54e3 | 1 | 1 | 359 | 361 |  |  | DB Search |
| total 20 peptides |
| --- |

Best Unique PSM (Scan POS\_R15-1.wiff:6566, m/z=481.7097, z=2, RT=16.66, ppm=6.26):


C4QYD9|C4QYD9\_KOMPG

back to list

  

| Protein Coverage
| Supporting Peptides
| Best Unique PSM
|

Protein Coverage:

Supporting Peptides:

| Peptide | Uniq | -10lgP | Mass | Length | ppm | m/z | z | RT | Scan | Area POS\_R15-1 | #Feature | #Feature POS\_R15-1 | Start | End | PTM | AScore | Found By |
| --- | --- | --- | --- | --- | --- | --- | --- | --- | --- | --- | --- | --- | --- | --- | --- | --- | --- |
| C.QAIP.V | N | 21.78 | 427.2431 | 4 | -3.04 | 428.248 | 1 | 11.76 | 4638 | 6.31e3 | 1 | 1 | 200 | 203 |  |  | DB Search |
| M.VEIA | N | 15.81 | 430.2427 | 4 | -4.19 | 431.2471 | 1 | 15.22 | 6032 | 0 | 0 | 0 | 369 | 372 |  |  | DB Search |
| D.RRAEGRAIRM.P | Y | 15.38 | 1214.6778 | 10 | -1.67 | 1215.6801 | 1 | 44.24 | 13718 | 1.85e3 | 1 | 1 | 263 | 272 |  |  | DB Search |
| total 3 peptides |
| --- |

Best Unique PSM (Scan POS\_R15-1.wiff:13718, m/z=1215.6802, z=1, RT=44.26, ppm=-4.15):


C4R4X3|C4R4X3\_KOMPG

back to list

  

| Protein Coverage
| Supporting Peptides
| Best Unique PSM
|

Protein Coverage:

Supporting Peptides:

| Peptide | Uniq | -10lgP | Mass | Length | ppm | m/z | z | RT | Scan | Area POS\_R15-1 | #Feature | #Feature POS\_R15-1 | Start | End | PTM | AScore | Found By |
| --- | --- | --- | --- | --- | --- | --- | --- | --- | --- | --- | --- | --- | --- | --- | --- | --- | --- |
| R.VGGL.A | N | 24.87 | 344.2059 | 4 | -5.22 | 345.2106 | 1 | 10.74 | 4195 | 1.51e3 | 1 | 1 | 163 | 166 |  |  | DB Search |
| P.YHH.A | N | 24.3 | 455.1917 | 3 | -4.98 | 456.1956 | 1 | 6.94 | 2666 | 0 | 0 | 0 | 4 | 6 |  |  | DB Search |
| D.LILP.V | N | 24.07 | 454.3155 | 4 | -2.4 | 455.3206 | 1 | 34.52 | 11565 | 2.64e3 | 1 | 1 | 194 | 197 |  |  | DB Search |
| L.GVSI.L | N | 22.74 | 374.2165 | 4 | -2.51 | 375.2219 | 1 | 13.91 | 5529 | 2.07e3 | 1 | 1 | 89 | 92 |  |  | DB Search |
| P.ISGI.I | N | 22.6 | 388.2322 | 4 | -3.81 | 389.237 | 1 | 9.87 | 3804 | 6.08e3 | 1 | 1 | 463 | 466 |  |  | DB Search |
| E.LAEV.G | N | 19.27 | 430.2427 | 4 | 1.69 | 431.2497 | 1 | 13.56 | 5318 | 0 | 0 | 0 | 201 | 204 |  |  | DB Search |
| F.NITL.P | N | 19.19 | 459.2693 | 4 | -0.54 | 460.2752 | 1 | 17.07 | 6712 | 2.02e3 | 1 | 1 | 53 | 56 |  |  | DB Search |
| L.GRPWG.F | Y | 18.67 | 571.2867 | 5 | 7.1 | 572.2966 | 1 | 10.14 | 3897 | 3.14e4 | 1 | 1 | 616 | 620 |  |  | DB Search |
| R.FHH.Q | N | 18.2 | 439.1968 | 3 | 7.99 | 440.2065 | 1 | 9.67 | 3787 | 2e3 | 1 | 1 | 555 | 557 |  |  | DB Search |
| L.LEFP.R | N | 17.54 | 504.2584 | 4 | -6.47 | 505.2611 | 1 | 30.11 | 10334 | 3.53e2 | 2 | 2 | 566 | 569 |  |  | DB Search |
| I.LPVA.E | N | 16.56 | 398.2529 | 4 | -3.75 | 399.2577 | 1 | 16.29 | 6409 | 1.73e3 | 1 | 1 | 196 | 199 |  |  | DB Search |
| P.TFT.P | N | 15.92 | 367.1743 | 3 | -7.47 | 368.1779 | 1 | 8.54 | 3263 | 0 | 0 | 0 | 62 | 64 |  |  | DB Search |
| S.LGVP.H | N | 15.32 | 384.2372 | 4 | -3.68 | 385.2422 | 1 | 16.13 | 6408 | 0 | 0 | 0 | 513 | 516 |  |  | DB Search |
| D.LNIYSTNETEIDDHR.K | Y | 15.21 | 1818.8384 | 15 | 0.31 | 910.4245 | 2 | 33.97 | 11413 | 0 | 0 | 0 | 375 | 389 |  |  | DB Search |
| total 14 peptides |
| --- |

Best Unique PSM (Scan POS\_R15-1.wiff:3897, m/z=572.2966, z=1, RT=10.14, ppm=4.61):


C4QXW2|C4QXW2\_KOMPG

back to list

  

| Protein Coverage
| Supporting Peptides
| Best Unique PSM
|

Protein Coverage:

Supporting Peptides:

| Peptide | Uniq | -10lgP | Mass | Length | ppm | m/z | z | RT | Scan | Area POS\_R15-1 | #Feature | #Feature POS\_R15-1 | Start | End | PTM | AScore | Found By |
| --- | --- | --- | --- | --- | --- | --- | --- | --- | --- | --- | --- | --- | --- | --- | --- | --- | --- |
| H.TLDL.L | N | 22.27 | 460.2533 | 4 | -8.03 | 461.2557 | 1 | 9.41 | 3625 | 2.97e2 | 1 | 1 | 34 | 37 |  |  | DB Search |
| G.PEF.F | N | 20.63 | 391.1743 | 3 | 0.03 | 392.1806 | 1 | 4.06 | 1653 | 5.45e3 | 1 | 1 | 133 | 135 |  |  | DB Search |
| A.VSSL.V | N | 20.48 | 404.2271 | 4 | -7.08 | 405.2305 | 1 | 8.17 | 2993 | 7.47e3 | 1 | 1 | 439 | 442 |  |  | DB Search |
| L.RDR.I | N | 18.68 | 445.2397 | 3 | 8.14 | 446.2495 | 1 | 10.60 | 4131 | 1.44e2 | 1 | 1 | 58 | 60 |  |  | DB Search |
| V.SVGL.L | N | 18.49 | 374.2165 | 4 | -4.96 | 375.221 | 1 | 14.62 | 5748 | 1.93e2 | 1 | 1 | 323 | 326 |  |  | DB Search |
| K.TFGV.A | N | 18.12 | 422.2165 | 4 | -5.92 | 423.2202 | 1 | 22.65 | 8244 | 5.26e2 | 1 | 1 | 195 | 198 |  |  | DB Search |
| L.QAIE.F | N | 17.59 | 459.2329 | 4 | -5.43 | 460.2365 | 1 | 4.85 | 1909 | 7.42e3 | 2 | 2 | 223 | 226 |  |  | DB Search |
| L.NGV.A | N | 15.37 | 288.1434 | 3 | 8 | 289.1522 | 1 | 5.91 | 2301 | 6.8e3 | 1 | 1 | 328 | 330 |  |  | DB Search |
| F.E(-18.01)GRASFG.A | Y | 15.07 | 704.3242 | 7 | 8 | 705.3353 | 1 | 49.47 | 14716 | 3.92e0 | 1 | 1 | 163 | 169 | Pyro-glu from E | E1:Pyro-glu from E:1000 | DB Search |
| D.EFEG.R | N | 15.01 | 480.1856 | 4 | -5.04 | 481.1893 | 1 | 10.64 | 4144 | 0 | 0 | 0 | 161 | 164 |  |  | DB Search |
| total 10 peptides |
| --- |

Best Unique PSM (Scan POS\_R15-1.wiff:14716, m/z=705.408, z=1, RT=49.49, ppm=5.52):


C4QVY8|C4QVY8\_KOMPG

back to list

  

| Protein Coverage
| Supporting Peptides
| Best Unique PSM
|

Protein Coverage:

Supporting Peptides:

| Peptide | Uniq | -10lgP | Mass | Length | ppm | m/z | z | RT | Scan | Area POS\_R15-1 | #Feature | #Feature POS\_R15-1 | Start | End | PTM | AScore | Found By |
| --- | --- | --- | --- | --- | --- | --- | --- | --- | --- | --- | --- | --- | --- | --- | --- | --- | --- |
| S.LSGL.K | N | 22.6 | 388.2322 | 4 | -3.81 | 389.237 | 1 | 9.87 | 3804 | 6.08e3 | 1 | 1 | 496 | 499 |  |  | DB Search |
| N.YNP.H | N | 21.45 | 392.1696 | 3 | -3.75 | 393.1744 | 1 | 8.45 | 3226 | 1.39e2 | 1 | 1 | 34 | 36 |  |  | DB Search |
| A.SIAP.T | N | 20.83 | 386.2165 | 4 | -4.65 | 387.221 | 1 | 8.35 | 3276 | 4.13e3 | 1 | 1 | 172 | 175 |  |  | DB Search |
| E.SLSGL.K | N | 19.58 | 475.2642 | 5 | -6.87 | 476.267 | 1 | 11.14 | 4400 | 2.28e3 | 1 | 1 | 495 | 499 |  |  | DB Search |
| S.LGPV.N | N | 18.01 | 384.2372 | 4 | -2.96 | 385.2424 | 1 | 11.56 | 4504 | 2.76e3 | 1 | 1 | 960 | 963 |  |  | DB Search |
| N.EEEPAEPAVP.A | Y | 17.85 | 1066.4818 | 10 | 5.42 | 534.2498 | 2 | 19.15 | 7315 | 0 | 0 | 0 | 596 | 605 |  |  | DB Search |
| L.EPKP.K | N | 17.69 | 469.2536 | 4 | -5.47 | 470.2572 | 1 | 15.43 | 6115 | 1.58e3 | 1 | 1 | 248 | 251 |  |  | DB Search |
| V.VIPV.N | N | 17 | 426.2842 | 4 | -1.74 | 427.2897 | 1 | 17.76 | 6936 | 3.8e3 | 1 | 1 | 532 | 535 |  |  | DB Search |
| N.TFT.G | N | 15.92 | 367.1743 | 3 | -7.47 | 368.1779 | 1 | 8.54 | 3263 | 0 | 0 | 0 | 727 | 729 |  |  | DB Search |
| total 9 peptides |
| --- |

Best Unique PSM (Scan POS\_R15-1.wiff:7315, m/z=534.2678, z=1, RT=19.19, ppm=2.94):


C4R899|C4R899\_KOMPG

back to list

  

| Protein Coverage
| Supporting Peptides
| Best Unique PSM
|

Protein Coverage:

Supporting Peptides:

| Peptide | Uniq | -10lgP | Mass | Length | ppm | m/z | z | RT | Scan | Area POS\_R15-1 | #Feature | #Feature POS\_R15-1 | Start | End | PTM | AScore | Found By |
| --- | --- | --- | --- | --- | --- | --- | --- | --- | --- | --- | --- | --- | --- | --- | --- | --- | --- |
| I.SLGD.G | N | 20.24 | 390.175 | 4 | -7.72 | 391.1783 | 1 | 3.30 | 1421 | 1.05e3 | 1 | 1 | 244 | 247 |  |  | DB Search |
| R.KCA.K | N | 19.71 | 320.1518 | 3 | 2.66 | 321.1591 | 1 | 15.28 | 6053 | 3.59e2 | 1 | 1 | 295 | 297 |  |  | DB Search |
| D.EAY.L | N | 19.24 | 381.1536 | 3 | -5.14 | 382.158 | 1 | 5.09 | 2057 | 2.63e3 | 1 | 1 | 123 | 125 |  |  | DB Search |
| G.SNG.S | N | 19.07 | 276.107 | 3 | 1.61 | 277.114 | 1 | 16.82 | 6604 | 0 | 0 | 0 | 171 | 173 |  |  | DB Search |
| A.VPGM(+15.99)VLAP.E | Y | 18.97 | 798.4309 | 8 | 6.79 | 799.4417 | 1 | 23.36 | 8506 | 7.62e1 | 1 | 1 | 10 | 17 | Oxidation (M) | M4:Oxidation (M):1000 | DB Search |
| K.GSSL.P | N | 18.21 | 362.1801 | 4 | -9.58 | 363.183 | 1 | 6.30 | 2465 | 1.38e3 | 1 | 1 | 128 | 131 |  |  | DB Search |
| N.AMY.A | N | 15.75 | 383.1515 | 3 | -9.49 | 384.1542 | 1 | 17.73 | 6972 | 0 | 0 | 0 | 274 | 276 |  |  | DB Search |
| total 7 peptides |
| --- |

Best Unique PSM (Scan POS\_R15-1.wiff:8506, m/z=799.4417, z=1, RT=23.36, ppm=4.31):


C4R7K8|C4R7K8\_KOMPG

back to list

  

| Protein Coverage
| Supporting Peptides
| Best Unique PSM
|

Protein Coverage:

Supporting Peptides:

| Peptide | Uniq | -10lgP | Mass | Length | ppm | m/z | z | RT | Scan | Area POS\_R15-1 | #Feature | #Feature POS\_R15-1 | Start | End | PTM | AScore | Found By |
| --- | --- | --- | --- | --- | --- | --- | --- | --- | --- | --- | --- | --- | --- | --- | --- | --- | --- |
| P.ETW.S | N | 26.2 | 434.1801 | 3 | -4.77 | 435.1843 | 1 | 12.45 | 4871 | 1.27e3 | 1 | 1 | 321 | 323 |  |  | DB Search |
| K.FPVG.V | N | 25.77 | 418.2216 | 4 | -7.2 | 419.2248 | 1 | 16.08 | 6373 | 0 | 0 | 0 | 836 | 839 |  |  | DB Search |
| K.LGVND.K | N | 22.39 | 516.2544 | 5 | -3.74 | 517.2584 | 1 | 5.18 | 2080 | 0 | 0 | 0 | 818 | 822 |  |  | DB Search |
| E.RCI.N | N | 20.6 | 390.2049 | 3 | -4.41 | 391.2095 | 1 | 8.08 | 3003 | 0 | 0 | 0 | 607 | 609 |  |  | DB Search |
| T.ALVE.G | N | 20.45 | 430.2427 | 4 | -5.9 | 431.2464 | 1 | 12.49 | 4901 | 0 | 0 | 0 | 620 | 623 |  |  | DB Search |
| M.KFH.L | N | 20.21 | 430.2328 | 3 | 9.29 | 431.243 | 1 | 12.31 | 5009 | 5.31e3 | 1 | 1 | 107 | 109 |  |  | DB Search |
| L.QFF.E | N | 19.56 | 440.206 | 3 | 3.92 | 441.2139 | 1 | 19.15 | 7313 | 0 | 0 | 0 | 349 | 351 |  |  | DB Search |
| R.RSTT.S | N | 18.45 | 463.2391 | 4 | -4.23 | 464.2432 | 1 | 17.11 | 6703 | 1.32e2 | 1 | 1 | 358 | 361 |  |  | DB Search |
| A.LVEG.S | N | 17.38 | 416.2271 | 4 | -5.82 | 417.2309 | 1 | 4.62 | 1798 | 0 | 0 | 0 | 621 | 624 |  |  | DB Search |
| N.SAVGYP.H | Y | 15.35 | 592.2857 | 6 | -6.12 | 593.2878 | 1 | 11.28 | 4507 | 0 | 0 | 0 | 586 | 591 |  |  | DB Search |
| E.GSPLFQD.K | Y | 15.07 | 762.3548 | 7 | -3.3 | 763.3577 | 1 | 20.37 | 7670 | 0 | 0 | 0 | 624 | 630 |  |  | DB Search |
| total 11 peptides |
| --- |

Best Unique PSM (Scan POS\_R15-1.wiff:4507, m/z=593.2878, z=1, RT=11.28, ppm=-8.6):


C4QW82|C4QW82\_KOMPG

back to list

  

| Protein Coverage
| Supporting Peptides
| Best Unique PSM
|

Protein Coverage:

Supporting Peptides:

| Peptide | Uniq | -10lgP | Mass | Length | ppm | m/z | z | RT | Scan | Area POS\_R15-1 | #Feature | #Feature POS\_R15-1 | Start | End | PTM | AScore | Found By |
| --- | --- | --- | --- | --- | --- | --- | --- | --- | --- | --- | --- | --- | --- | --- | --- | --- | --- |
| H.TIDI.V | N | 22.27 | 460.2533 | 4 | -8.03 | 461.2557 | 1 | 9.41 | 3625 | 2.97e2 | 1 | 1 | 290 | 293 |  |  | DB Search |
| S.ELF.S | N | 21.7 | 407.2056 | 3 | -8.99 | 408.2082 | 1 | 29.23 | 10157 | 9.42e2 | 1 | 1 | 101 | 103 |  |  | DB Search |
| E.NSG.N | N | 20.42 | 276.107 | 3 | 6.48 | 277.1154 | 1 | 16.74 | 6592 | 0 | 0 | 0 | 35 | 37 |  |  | DB Search |
| C.ISPM.S | N | 19.59 | 446.2199 | 4 | -1.9 | 447.2252 | 1 | 15.64 | 6201 | 6.79e2 | 1 | 1 | 115 | 118 |  |  | DB Search |
| R.LSLP.E | N | 18.29 | 428.2635 | 4 | -7.33 | 429.2665 | 1 | 24.15 | 8767 | 0 | 0 | 0 | 308 | 311 |  |  | DB Search |
| K.TW.I | N | 18.09 | 305.1375 | 2 | -1.88 | 306.1435 | 1 | 9.22 | 3551 | 5.58e3 | 1 | 1 | 349 | 350 |  |  | DB Search |
| I.DIVT.E | N | 16.75 | 446.2376 | 4 | -4.95 | 447.2416 | 1 | 23.24 | 8484 | 0 | 0 | 0 | 292 | 295 |  |  | DB Search |
| S.ELID.I | N | 16.42 | 488.2482 | 4 | -0.37 | 489.2541 | 1 | 13.94 | 5546 | 0 | 0 | 0 | 7 | 10 |  |  | DB Search |
| E.E(-18.01)PI.V | N | 16.1 | 339.1794 | 3 | -5.62 | 340.1839 | 1 | 16.05 | 6351 | 1.63e4 | 1 | 1 | 52 | 54 | Pyro-glu from E | E1:Pyro-glu from E:1000 | DB Search |
| V.NGV.L | N | 15.37 | 288.1434 | 3 | 8 | 289.1522 | 1 | 5.91 | 2301 | 6.8e3 | 1 | 1 | 129 | 131 |  |  | DB Search |
| T.VGGAP.S | Y | 15.07 | 399.2118 | 5 | -4.57 | 400.2162 | 1 | 3.29 | 1409 | 0 | 0 | 0 | 84 | 88 |  |  | DB Search |
| total 11 peptides |
| --- |

Best Unique PSM (Scan POS\_R15-1.wiff:1409, m/z=400.2162, z=1, RT=3.29, ppm=-7.06):


C4R3D3|C4R3D3\_KOMPG

back to list

  

| Protein Coverage
| Supporting Peptides
| Best Unique PSM
|

Protein Coverage:

Supporting Peptides:

| Peptide | Uniq | -10lgP | Mass | Length | ppm | m/z | z | RT | Scan | Area POS\_R15-1 | #Feature | #Feature POS\_R15-1 | Start | End | PTM | AScore | Found By |
| --- | --- | --- | --- | --- | --- | --- | --- | --- | --- | --- | --- | --- | --- | --- | --- | --- | --- |
| I.ITGI.A | N | 27.55 | 402.2478 | 4 | -1.76 | 403.2534 | 1 | 15.04 | 5964 | 1.56e4 | 2 | 2 | 1314 | 1317 |  |  | DB Search |
| I.TSLA.L | N | 24.8 | 390.2114 | 4 | -5.32 | 391.2157 | 1 | 6.25 | 2416 | 1.07e4 | 1 | 1 | 946 | 949 |  |  | DB Search |
| S.LSGI.V | N | 22.6 | 388.2322 | 4 | -3.81 | 389.237 | 1 | 9.87 | 3804 | 6.08e3 | 1 | 1 | 257 | 260 |  |  | DB Search |
| D.RCL.P | N | 20.6 | 390.2049 | 3 | -4.41 | 391.2095 | 1 | 8.08 | 3003 | 0 | 0 | 0 | 431 | 433 |  |  | DB Search |
| E.GSVL.F | N | 20.47 | 374.2165 | 4 | -5.12 | 375.2209 | 1 | 10.69 | 4154 | 1e3 | 1 | 1 | 236 | 239 |  |  | DB Search |
| A.VTTL.S | N | 20.12 | 432.2584 | 4 | -4.38 | 433.2627 | 1 | 11.15 | 4417 | 0 | 0 | 0 | 1064 | 1067 |  |  | DB Search |
| P.SLSGI.V | N | 19.58 | 475.2642 | 5 | -6.87 | 476.267 | 1 | 11.14 | 4400 | 2.28e3 | 1 | 1 | 256 | 260 |  |  | DB Search |
| I.ITGIAVIQ.K | Y | 19.38 | 813.496 | 8 | -6.1 | 814.4963 | 1 | 28.75 | 10005 | 3.04e3 | 1 | 1 | 1314 | 1321 |  |  | DB Search |
| E.NLTI.Q | N | 19.19 | 459.2693 | 4 | -0.54 | 460.2752 | 1 | 17.07 | 6712 | 2.02e3 | 1 | 1 | 533 | 536 |  |  | DB Search |
| V.E(-18.01)PH.I | N | 16.21 | 363.1543 | 3 | -0.12 | 364.1606 | 1 | 2.32 | 1021 | 3.39e4 | 1 | 1 | 375 | 377 | Pyro-glu from E | E1:Pyro-glu from E:1000 | DB Search |
| S.YMR.M | N | 15.84 | 468.2155 | 3 | 0.49 | 469.2218 | 1 | 8.86 | 3455 | 2.39e3 | 1 | 1 | 492 | 494 |  |  | DB Search |
| Q.YHG.D | N | 15.26 | 375.1543 | 3 | -7.53 | 376.1578 | 1 | 4.73 | 1840 | 0 | 0 | 0 | 144 | 146 |  |  | DB Search |
| total 12 peptides |
| --- |

Best Unique PSM (Scan POS\_R15-1.wiff:10005, m/z=814.4963, z=1, RT=28.75, ppm=-8.58):


C4R3C5|C4R3C5\_KOMPG

back to list

  

| Protein Coverage
| Supporting Peptides
| Best Unique PSM
|

Protein Coverage:

Supporting Peptides:

| Peptide | Uniq | -10lgP | Mass | Length | ppm | m/z | z | RT | Scan | Area POS\_R15-1 | #Feature | #Feature POS\_R15-1 | Start | End | PTM | AScore | Found By |
| --- | --- | --- | --- | --- | --- | --- | --- | --- | --- | --- | --- | --- | --- | --- | --- | --- | --- |
| D.PSLI.N | N | 29.95 | 428.2635 | 4 | -0.56 | 429.2694 | 1 | 21.28 | 7884 | 0 | 0 | 0 | 267 | 270 |  |  | DB Search |
| D.IILP.P | N | 24.07 | 454.3155 | 4 | -2.4 | 455.3206 | 1 | 34.52 | 11565 | 2.64e3 | 1 | 1 | 688 | 691 |  |  | DB Search |
| A.TIDI.S | N | 22.27 | 460.2533 | 4 | -8.03 | 461.2557 | 1 | 9.41 | 3625 | 2.97e2 | 1 | 1 | 831 | 834 |  |  | DB Search |
| S.PGE.Y | N | 21.28 | 301.1274 | 3 | 1.88 | 302.1345 | 1 | 2.04 | 869 | 2.57e3 | 1 | 1 | 326 | 328 |  |  | DB Search |
| L.KYT.I | N | 21.18 | 410.2165 | 3 | -4.93 | 411.2208 | 1 | 12.54 | 4954 | 3.83e2 | 1 | 1 | 855 | 857 |  |  | DB Search |
| A.ITDI.K | N | 20.88 | 460.2533 | 4 | -0.2 | 461.2593 | 1 | 14.80 | 5851 | 7.36e2 | 1 | 1 | 203 | 206 |  |  | DB Search |
| P.SIAP.T | N | 20.83 | 386.2165 | 4 | -4.65 | 387.221 | 1 | 8.35 | 3276 | 4.13e3 | 1 | 1 | 1193 | 1196 |  |  | DB Search |
| R.TAPQ.K | N | 20.69 | 415.2067 | 4 | 3.43 | 416.2144 | 1 | 8.30 | 3151 | 1.09e3 | 1 | 1 | 352 | 355 |  |  | DB Search |
| F.AAVE.A | N | 20.66 | 388.1958 | 4 | -4.49 | 389.2003 | 1 | 2.65 | 1124 | 0 | 0 | 0 | 15 | 18 |  |  | DB Search |
| T.VPVE.N | N | 20.6 | 442.2427 | 4 | -5.67 | 443.2464 | 1 | 7.79 | 2913 | 1.07e3 | 1 | 1 | 1402 | 1405 |  |  | DB Search |
| V.GSVI.I | N | 20.47 | 374.2165 | 4 | -5.12 | 375.2209 | 1 | 10.69 | 4154 | 1e3 | 1 | 1 | 385 | 388 |  |  | DB Search |
| I.AGAA.D | N | 20.37 | 288.1433 | 4 | 8.03 | 289.1522 | 1 | 5.91 | 2203 | 6.8e3 | 1 | 1 | 810 | 813 |  |  | DB Search |
| W.VVDI.P | N | 19.9 | 444.2584 | 4 | 0.77 | 445.2649 | 1 | 17.32 | 6776 | 2.15e3 | 1 | 1 | 368 | 371 |  |  | DB Search |
| S.AFR.S | N | 19.1 | 392.2172 | 3 | -7.11 | 393.2207 | 1 | 6.33 | 2475 | 0 | 0 | 0 | 927 | 929 |  |  | DB Search |
| E.TPIT.V | N | 18.8 | 430.2427 | 4 | -8.61 | 431.2452 | 1 | 9.60 | 3908 | 8.62e3 | 1 | 1 | 1262 | 1265 |  |  | DB Search |
| G.APY.P | N | 16.84 | 349.1638 | 3 | -4.81 | 350.1685 | 1 | 16.32 | 6384 | 1.41e3 | 1 | 1 | 374 | 376 |  |  | DB Search |
| A.EVQ.C | N | 16.23 | 374.1801 | 3 | 0.92 | 375.1868 | 1 | 2.99 | 1221 | 7.34e3 | 1 | 1 | 897 | 899 |  |  | DB Search |
| N.VLNF.T | N | 16.08 | 491.2744 | 4 | -1.92 | 492.2795 | 1 | 24.95 | 8989 | 2.33e2 | 1 | 1 | 291 | 294 |  |  | DB Search |
| G.LGGV.S | N | 15.94 | 344.2059 | 4 | 0.19 | 345.2124 | 1 | 5.48 | 2143 | 3.05e3 | 1 | 1 | 442 | 445 |  |  | DB Search |
| A.KST.T | N | 15.47 | 334.1852 | 3 | 0.84 | 335.192 | 1 | 54.68 | 16215 | 3.66e2 | 1 | 1 | 1374 | 1376 |  |  | DB Search |
| T.IDPTGQP.T | Y | 15.4 | 726.3548 | 7 | -6.11 | 727.3558 | 1 | 19.06 | 7365 | 5.67e2 | 1 | 1 | 1177 | 1183 |  |  | DB Search |
| D.PVE.E | N | 15.19 | 343.1743 | 3 | -3.43 | 344.1796 | 1 | 12.76 | 5037 | 6.34e2 | 1 | 1 | 981 | 983 |  |  | DB Search |
| V.QVAL.P | N | 15.07 | 429.2587 | 4 | 5.6 | 430.2673 | 1 | 21.95 | 8124 | 0 | 0 | 0 | 246 | 249 |  |  | DB Search |
| total 23 peptides |
| --- |

Best Unique PSM (Scan POS\_R15-1.wiff:7365, m/z=727.3062, z=2, RT=19.28, ppm=-8.59):


C4R3B8|C4R3B8\_KOMPG

back to list

  

| Protein Coverage
| Supporting Peptides
| Best Unique PSM
|

Protein Coverage:

Supporting Peptides:

| Peptide | Uniq | -10lgP | Mass | Length | ppm | m/z | z | RT | Scan | Area POS\_R15-1 | #Feature | #Feature POS\_R15-1 | Start | End | PTM | AScore | Found By |
| --- | --- | --- | --- | --- | --- | --- | --- | --- | --- | --- | --- | --- | --- | --- | --- | --- | --- |
| A.SPII.G | N | 26.66 | 428.2635 | 4 | -1.63 | 429.269 | 1 | 21.35 | 7905 | 0 | 0 | 0 | 463 | 466 |  |  | DB Search |
| A.SVGV.W | N | 26.46 | 360.2009 | 4 | -6.45 | 361.2049 | 1 | 8.68 | 3331 | 1.41e4 | 1 | 1 | 471 | 474 |  |  | DB Search |
| K.IGSI.D | N | 25.26 | 388.2322 | 4 | -3.81 | 389.237 | 1 | 9.87 | 3785 | 6.08e3 | 1 | 1 | 221 | 224 |  |  | DB Search |
| S.LLIP.F | N | 24.07 | 454.3155 | 4 | -2.4 | 455.3206 | 1 | 34.52 | 11565 | 2.64e3 | 1 | 1 | 177 | 180 |  |  | DB Search |
| S.SPT.E | N | 23.16 | 303.143 | 3 | 6.39 | 304.1515 | 1 | 7.14 | 2731 | 5.4e3 | 2 | 2 | 558 | 560 |  |  | DB Search |
| I.VASSL.L | N | 20.92 | 475.2642 | 5 | -5.07 | 476.2679 | 1 | 17.73 | 6973 | 0 | 0 | 0 | 173 | 177 |  |  | DB Search |
| M.TFR.S | N | 20.53 | 422.2278 | 3 | -9.2 | 423.2301 | 1 | 22.38 | 8244 | 2.98e1 | 1 | 1 | 250 | 252 |  |  | DB Search |
| M.ISVS.S | N | 20.49 | 404.2271 | 4 | -7.08 | 405.2305 | 1 | 8.17 | 3035 | 7.47e3 | 1 | 1 | 375 | 378 |  |  | DB Search |
| S.VSSI.L | N | 20.48 | 404.2271 | 4 | -7.08 | 405.2305 | 1 | 8.17 | 2993 | 7.47e3 | 1 | 1 | 377 | 380 |  |  | DB Search |
| G.TAY.H | N | 19.3 | 353.1587 | 3 | -1.32 | 354.1646 | 1 | 12.51 | 4913 | 1.39e3 | 1 | 1 | 478 | 480 |  |  | DB Search |
| V.DVVY.E | N | 17.92 | 494.2376 | 4 | -7.07 | 495.2402 | 1 | 12.58 | 4967 | 1.85e3 | 1 | 1 | 548 | 551 |  |  | DB Search |
| G.SVSSPT.E | Y | 17.85 | 576.2755 | 6 | 2.49 | 577.2828 | 1 | 16.26 | 6482 | 7.05e1 | 1 | 1 | 555 | 560 |  |  | DB Search |
| F.RGY.T | N | 17.46 | 394.1965 | 3 | 3.18 | 395.204 | 1 | 18.17 | 7080 | 0 | 0 | 0 | 635 | 637 |  |  | DB Search |
| P.SFPTY.P | N | 17.28 | 613.2748 | 5 | -3.69 | 614.2783 | 1 | 23.52 | 8598 | 2.27e2 | 1 | 1 | 323 | 327 |  |  | DB Search |
| E.TTVH.L | N | 16.14 | 456.2332 | 4 | 1.87 | 457.2402 | 1 | 17.22 | 6793 | 1.04e2 | 1 | 1 | 6 | 9 |  |  | DB Search |
| M.IGGV.C | N | 15.94 | 344.2059 | 4 | 0.19 | 345.2124 | 1 | 5.48 | 2143 | 3.05e3 | 1 | 1 | 295 | 298 |  |  | DB Search |
| A.FSANL.K | N | 15.79 | 550.2751 | 5 | -7.88 | 551.2767 | 1 | 19.15 | 7316 | 0 | 0 | 0 | 282 | 286 |  |  | DB Search |
| F.FRG.Y | N | 15.51 | 378.2015 | 3 | -9.17 | 379.2044 | 1 | 3.37 | 1492 | 1.94e3 | 1 | 1 | 634 | 636 |  |  | DB Search |
| total 18 peptides |
| --- |

Best Unique PSM (Scan POS\_R15-1.wiff:6482, m/z=578.2772, z=2, RT=16.28, ppm=0):


C4QZI1|C4QZI1\_KOMPG

back to list

  

| Protein Coverage
| Supporting Peptides
| Best Unique PSM
|

Protein Coverage:

Supporting Peptides:

| Peptide | Uniq | -10lgP | Mass | Length | ppm | m/z | z | RT | Scan | Area POS\_R15-1 | #Feature | #Feature POS\_R15-1 | Start | End | PTM | AScore | Found By |
| --- | --- | --- | --- | --- | --- | --- | --- | --- | --- | --- | --- | --- | --- | --- | --- | --- | --- |
| Q.LTGI.N | N | 27.55 | 402.2478 | 4 | -1.76 | 403.2534 | 1 | 15.04 | 5964 | 1.56e4 | 2 | 2 | 299 | 302 |  |  | DB Search |
| A.LGGL.L | N | 27.34 | 358.2216 | 4 | -4.39 | 359.2264 | 1 | 15.45 | 6114 | 7.1e3 | 1 | 1 | 88 | 91 |  |  | DB Search |
| L.SPLI.I | N | 26.66 | 428.2635 | 4 | -1.63 | 429.269 | 1 | 21.35 | 7905 | 0 | 0 | 0 | 440 | 443 |  |  | DB Search |
| D.IGSI.G | N | 25.26 | 388.2322 | 4 | -3.81 | 389.237 | 1 | 9.87 | 3785 | 6.08e3 | 1 | 1 | 43 | 46 |  |  | DB Search |
| G.LLLP.R | N | 24.07 | 454.3155 | 4 | -2.4 | 455.3206 | 1 | 34.52 | 11565 | 2.64e3 | 1 | 1 | 91 | 94 |  |  | DB Search |
| L.SPT.D | N | 23.16 | 303.143 | 3 | 6.39 | 304.1515 | 1 | 7.14 | 2731 | 5.4e3 | 2 | 2 | 242 | 244 |  |  | DB Search |
| V.VGSI.M | N | 21.91 | 374.2165 | 4 | 0.34 | 375.223 | 1 | 13.82 | 5444 | 2.07e3 | 1 | 1 | 382 | 385 |  |  | DB Search |
| V.AIVTAFVP.E | Y | 21.21 | 816.4745 | 8 | -4.06 | 817.4764 | 1 | 40.62 | 13039 | 1.2e3 | 1 | 1 | 209 | 216 |  |  | DB Search |
| S.SNG.A | N | 19.07 | 276.107 | 3 | 1.61 | 277.114 | 1 | 16.82 | 6604 | 0 | 0 | 0 | 377 | 379 |  |  | DB Search |
| G.E(-18.01)PR.I | N | 16.87 | 382.1965 | 3 | -0.37 | 383.2026 | 1 | 2.61 | 1082 | 1.62e4 | 1 | 1 | 280 | 282 | Pyro-glu from E | E1:Pyro-glu from E:1000 | DB Search |
| P.ESPV.Y | N | 16.59 | 430.2063 | 4 | 5.53 | 431.2149 | 1 | 12.65 | 5009 | 0 | 0 | 0 | 217 | 220 |  |  | DB Search |
| total 11 peptides |
| --- |

Best Unique PSM (Scan POS\_R15-1.wiff:13039, m/z=817.4759, z=1, RT=40.63, ppm=-6.55):


C4QXN6|C4QXN6\_KOMPG

back to list

  

| Protein Coverage
| Supporting Peptides
| Best Unique PSM
|

Protein Coverage:

Supporting Peptides:

| Peptide | Uniq | -10lgP | Mass | Length | ppm | m/z | z | RT | Scan | Area POS\_R15-1 | #Feature | #Feature POS\_R15-1 | Start | End | PTM | AScore | Found By |
| --- | --- | --- | --- | --- | --- | --- | --- | --- | --- | --- | --- | --- | --- | --- | --- | --- | --- |
| A.TFR.M | N | 20.53 | 422.2278 | 3 | -9.2 | 423.2301 | 1 | 22.38 | 8244 | 2.98e1 | 1 | 1 | 273 | 275 |  |  | DB Search |
| H.LTVA.G | N | 19.62 | 402.2478 | 4 | -4.79 | 403.2522 | 1 | 10.65 | 4156 | 4.9e3 | 1 | 1 | 230 | 233 |  |  | DB Search |
| L.VNVP.F | N | 18.75 | 427.2431 | 4 | 2.54 | 428.2504 | 1 | 12.03 | 4834 | 6.48e3 | 1 | 1 | 128 | 131 |  |  | DB Search |
| T.PVESPAP.N | Y | 18.34 | 695.349 | 7 | -3.48 | 696.3521 | 1 | 12.52 | 4929 | 0 | 0 | 0 | 296 | 302 |  |  | DB Search |
| G.LEFP.V | N | 17.54 | 504.2584 | 4 | -6.47 | 505.2611 | 1 | 30.11 | 10334 | 3.53e2 | 2 | 2 | 550 | 553 |  |  | DB Search |
| N.DLIP.N | N | 16.51 | 456.2584 | 4 | -3.13 | 457.2631 | 1 | 23.84 | 8659 | 1.35e3 | 1 | 1 | 107 | 110 |  |  | DB Search |
| V.QLVA.V | N | 15.44 | 429.2587 | 4 | -4.77 | 430.2629 | 1 | 10.63 | 4142 | 1.75e3 | 1 | 1 | 219 | 222 |  |  | DB Search |
| total 7 peptides |
| --- |

Best Unique PSM (Scan POS\_R15-1.wiff:4929, m/z=696.3521, z=1, RT=12.52, ppm=-5.96):


C4R8S1|C4R8S1\_KOMPG

back to list

  

| Protein Coverage
| Supporting Peptides
| Best Unique PSM
|

Protein Coverage:

Supporting Peptides:

| Peptide | Uniq | -10lgP | Mass | Length | ppm | m/z | z | RT | Scan | Area POS\_R15-1 | #Feature | #Feature POS\_R15-1 | Start | End | PTM | AScore | Found By |
| --- | --- | --- | --- | --- | --- | --- | --- | --- | --- | --- | --- | --- | --- | --- | --- | --- | --- |
| R.GVGP.L | N | 24.4 | 328.1746 | 4 | -1.91 | 329.1805 | 1 | 5.92 | 2228 | 4.71e4 | 1 | 1 | 345 | 348 |  |  | DB Search |
| G.YGR.A | N | 21.6 | 394.1965 | 3 | -4.25 | 395.2011 | 1 | 5.35 | 1961 | 1.67e4 | 1 | 1 | 248 | 250 |  |  | DB Search |
| A.DIGF.E | N | 21.22 | 450.2114 | 4 | 2.45 | 451.2187 | 1 | 25.55 | 9169 | 5.5e2 | 1 | 1 | 477 | 480 |  |  | DB Search |
| K.E(-18.01)SRGAHA.R | Y | 19.52 | 708.3303 | 7 | 8.42 | 709.3418 | 1 | 2.95 | 1189 | 4.44e2 | 1 | 1 | 577 | 583 | Pyro-glu from E | E1:Pyro-glu from E:1000 | DB Search |
| N.AALG.N | N | 16.78 | 330.1903 | 4 | -8.43 | 331.194 | 1 | 5.91 | 2160 | 5.17e3 | 1 | 1 | 98 | 101 |  |  | DB Search |
| V.ITET.L | N | 15.47 | 462.2326 | 4 | 1.41 | 463.2393 | 1 | 15.37 | 6065 | 2.41e2 | 1 | 1 | 618 | 621 |  |  | DB Search |
| total 6 peptides |
| --- |

Best Unique PSM (Scan POS\_R15-1.wiff:1189, m/z=709.3113, z=1, RT=2.96, ppm=5.94):


C4R0M8|C4R0M8\_KOMPG

back to list

  

| Protein Coverage
| Supporting Peptides
| Best Unique PSM
|

Protein Coverage:

Supporting Peptides:

| Peptide | Uniq | -10lgP | Mass | Length | ppm | m/z | z | RT | Scan | Area POS\_R15-1 | #Feature | #Feature POS\_R15-1 | Start | End | PTM | AScore | Found By |
| --- | --- | --- | --- | --- | --- | --- | --- | --- | --- | --- | --- | --- | --- | --- | --- | --- | --- |
| T.FPVG.R | N | 25.77 | 418.2216 | 4 | -7.2 | 419.2248 | 1 | 16.08 | 6373 | 0 | 0 | 0 | 26 | 29 |  |  | DB Search |
| G.SGAPVY.L | Y | 17.29 | 592.2857 | 6 | -6.84 | 593.2874 | 1 | 11.24 | 4496 | 1.28e3 | 1 | 1 | 46 | 51 |  |  | DB Search |
| T.AVLE.Y | N | 15.33 | 430.2427 | 4 | -8.59 | 431.2452 | 1 | 9.60 | 3778 | 8.62e3 | 1 | 1 | 54 | 57 |  |  | DB Search |
| total 3 peptides |
| --- |

Best Unique PSM (Scan POS\_R15-1.wiff:4496, m/z=593.2869, z=1, RT=11.27, ppm=-9.32):


C4QYF7|C4QYF7\_KOMPG

back to list

  

| Protein Coverage
| Supporting Peptides
| Best Unique PSM
|

Protein Coverage:

Supporting Peptides:

| Peptide | Uniq | -10lgP | Mass | Length | ppm | m/z | z | RT | Scan | Area POS\_R15-1 | #Feature | #Feature POS\_R15-1 | Start | End | PTM | AScore | Found By |
| --- | --- | --- | --- | --- | --- | --- | --- | --- | --- | --- | --- | --- | --- | --- | --- | --- | --- |
| H.NSG.Y | N | 20.42 | 276.107 | 3 | 6.48 | 277.1154 | 1 | 16.74 | 6592 | 0 | 0 | 0 | 43 | 45 |  |  | DB Search |
| Y.HGY.K | N | 18.63 | 375.1543 | 3 | -9 | 376.1572 | 1 | 4.83 | 1959 | 3.43e3 | 1 | 1 | 145 | 147 |  |  | DB Search |
| V.ILTD.D | N | 17.52 | 460.2533 | 4 | -9.09 | 461.2552 | 1 | 10.16 | 3965 | 9.46e2 | 1 | 1 | 154 | 157 |  |  | DB Search |
| E.KFSP.A | N | 16.37 | 477.2587 | 4 | -1.04 | 478.2643 | 1 | 8.51 | 3251 | 0 | 0 | 0 | 324 | 327 |  |  | DB Search |
| N.YHG.Y | N | 15.26 | 375.1543 | 3 | -7.53 | 376.1578 | 1 | 4.73 | 1840 | 0 | 0 | 0 | 144 | 146 |  |  | DB Search |
| I.AFSGCK.D | Y | 15.13 | 611.2737 | 6 | 4.9 | 612.2825 | 1 | 6.42 | 2510 | 1.57e2 | 1 | 1 | 332 | 337 |  |  | DB Search |
| total 6 peptides |
| --- |

Best Unique PSM (Scan POS\_R15-1.wiff:2510, m/z=612.2554, z=1, RT=6.52, ppm=2.41):


C4QWW6|C4QWW6\_KOMPG

back to list

  

| Protein Coverage
| Supporting Peptides
| Best Unique PSM
|

Protein Coverage:

Supporting Peptides:

| Peptide | Uniq | -10lgP | Mass | Length | ppm | m/z | z | RT | Scan | Area POS\_R15-1 | #Feature | #Feature POS\_R15-1 | Start | End | PTM | AScore | Found By |
| --- | --- | --- | --- | --- | --- | --- | --- | --- | --- | --- | --- | --- | --- | --- | --- | --- | --- |
| V.PSIL.E | N | 29.95 | 428.2635 | 4 | -0.56 | 429.2694 | 1 | 21.28 | 7884 | 0 | 0 | 0 | 1303 | 1306 |  |  | DB Search |
| A.ISIE.A | N | 25.68 | 460.2533 | 4 | -2.19 | 461.2584 | 1 | 13.54 | 5303 | 1.35e4 | 1 | 1 | 497 | 500 |  |  | DB Search |
| K.ALISL.C | N | 24.46 | 515.3319 | 5 | -6.84 | 516.3344 | 1 | 37.01 | 12185 | 8.65e2 | 1 | 1 | 836 | 840 |  |  | DB Search |
| V.HFT.R | N | 23.64 | 403.1856 | 3 | -0.83 | 404.1915 | 1 | 13.94 | 5543 | 0 | 0 | 0 | 1222 | 1224 |  |  | DB Search |
| L.IDGL.S | N | 22.09 | 416.2271 | 4 | -1.79 | 417.2326 | 1 | 13.94 | 5544 | 2.42e3 | 1 | 1 | 184 | 187 |  |  | DB Search |
| V.TSG.V | N | 21.44 | 263.1117 | 3 | 2.31 | 264.119 | 1 | 3.71 | 1537 | 5.72e3 | 1 | 1 | 977 | 979 |  |  | DB Search |
| M.MGR.G | N | 20.83 | 362.1736 | 3 | 6.82 | 363.1825 | 1 | 3.30 | 1420 | 1.5e3 | 1 | 1 | 214 | 216 |  |  | DB Search |
| N.PEF.V | N | 20.63 | 391.1743 | 3 | 0.03 | 392.1806 | 1 | 4.06 | 1653 | 5.45e3 | 1 | 1 | 258 | 260 |  |  | DB Search |
| C.RCL.Y | N | 20.6 | 390.2049 | 3 | -4.41 | 391.2095 | 1 | 8.08 | 3003 | 0 | 0 | 0 | 248 | 250 |  |  | DB Search |
| K.TFR.T | N | 20.53 | 422.2278 | 3 | -9.2 | 423.2301 | 1 | 22.38 | 8244 | 2.98e1 | 1 | 1 | 452 | 454 |  |  | DB Search |
| N.NSG.F | N | 20.42 | 276.107 | 3 | 6.48 | 277.1154 | 1 | 16.74 | 6592 | 0 | 0 | 0 | 1055 | 1057 |  |  | DB Search |
| L.VTTL.L | N | 20.12 | 432.2584 | 4 | -4.38 | 433.2627 | 1 | 11.15 | 4417 | 0 | 0 | 0 | 510 | 513 |  |  | DB Search |
| V.WSD.L | N | 19.99 | 406.1488 | 3 | -8.45 | 407.1517 | 1 | 8.75 | 3390 | 0 | 0 | 0 | 891 | 893 |  |  | DB Search |
| A.SNG.S | N | 19.07 | 276.107 | 3 | 1.61 | 277.114 | 1 | 16.82 | 6604 | 0 | 0 | 0 | 566 | 568 |  |  | DB Search |
| L.LQEQ.L | N | 18.84 | 516.2544 | 4 | -0.21 | 517.2603 | 1 | 2.57 | 1095 | 1.3e3 | 1 | 1 | 236 | 239 |  |  | DB Search |
| D.HGY.S | N | 18.63 | 375.1543 | 3 | -9 | 376.1572 | 1 | 4.83 | 1959 | 3.43e3 | 1 | 1 | 1562 | 1564 |  |  | DB Search |
| P.GSSI.N | N | 18.21 | 362.1801 | 4 | -9.58 | 363.183 | 1 | 6.30 | 2465 | 1.38e3 | 1 | 1 | 41 | 44 |  |  | DB Search |
| N.IYSS.T | N | 17.99 | 468.222 | 4 | 0.32 | 469.2283 | 1 | 16.06 | 6398 | 1.94e3 | 1 | 1 | 584 | 587 |  |  | DB Search |
| S.TSN.S | N | 17.54 | 320.1332 | 3 | 3.34 | 321.1407 | 1 | 8.11 | 3024 | 0 | 0 | 0 | 412 | 414 |  |  | DB Search |
| T.RSF.F | N | 17.5 | 408.2121 | 3 | -1.76 | 409.2177 | 1 | 9.17 | 3526 | 0 | 0 | 0 | 1236 | 1238 |  |  | DB Search |
| K.SPK.W | N | 16.89 | 330.1903 | 3 | -8.46 | 331.194 | 1 | 5.91 | 2235 | 5.17e3 | 1 | 1 | 16 | 18 |  |  | DB Search |
| V.E(-18.01)LNP.E | N | 16.86 | 453.2223 | 4 | -6.06 | 454.2257 | 1 | 14.47 | 5683 | 1.68e4 | 1 | 1 | 255 | 258 | Pyro-glu from E | E1:Pyro-glu from E:1000 | DB Search |
| D.DIVT.R | N | 16.75 | 446.2376 | 4 | -4.95 | 447.2416 | 1 | 23.24 | 8484 | 0 | 0 | 0 | 131 | 134 |  |  | DB Search |
| S.FIDGFST.V | Y | 15.95 | 785.3595 | 7 | 4.42 | 786.3683 | 1 | 44.42 | 13749 | 4.15e1 | 1 | 1 | 1517 | 1523 |  |  | DB Search |
| L.QLVA.C | N | 15.44 | 429.2587 | 4 | -4.77 | 430.2629 | 1 | 10.63 | 4142 | 1.75e3 | 1 | 1 | 243 | 246 |  |  | DB Search |
| L.PSVM.T | N | 15.29 | 432.2042 | 4 | 7.34 | 433.2136 | 1 | 17.71 | 6957 | 1.06e2 | 1 | 1 | 1629 | 1632 |  |  | DB Search |
| total 26 peptides |
| --- |

Best Unique PSM (Scan POS\_R15-1.wiff:13749, m/z=787.3831, z=2, RT=44.43, ppm=1.94):


C4R4X4|C4R4X4\_KOMPG

back to list

  

| Protein Coverage
| Supporting Peptides
| Best Unique PSM
|

Protein Coverage:

Supporting Peptides:

| Peptide | Uniq | -10lgP | Mass | Length | ppm | m/z | z | RT | Scan | Area POS\_R15-1 | #Feature | #Feature POS\_R15-1 | Start | End | PTM | AScore | Found By |
| --- | --- | --- | --- | --- | --- | --- | --- | --- | --- | --- | --- | --- | --- | --- | --- | --- | --- |
| Y.IPTP.E | N | 25.97 | 426.2478 | 4 | -3.74 | 427.2524 | 1 | 12.64 | 4915 | 1.93e3 | 1 | 1 | 227 | 230 |  |  | DB Search |
| G.AAQ.M | N | 16.83 | 288.1434 | 3 | 8 | 289.1522 | 1 | 5.91 | 2315 | 6.8e3 | 1 | 1 | 121 | 123 |  |  | DB Search |
| I.VVAAAD.G | Y | 15.49 | 544.2856 | 6 | -8.99 | 545.2867 | 1 | 10.79 | 4177 | 9.2e2 | 1 | 1 | 130 | 135 |  |  | DB Search |
| total 3 peptides |
| --- |

Best Unique PSM (Scan POS\_R15-1.wiff:4177, m/z=545.2867, z=1, RT=10.79, ppm=-11.47):


C4R7H9|C4R7H9\_KOMPG

back to list

  

| Protein Coverage
| Supporting Peptides
| Best Unique PSM
|

Protein Coverage:

Supporting Peptides:

| Peptide | Uniq | -10lgP | Mass | Length | ppm | m/z | z | RT | Scan | Area POS\_R15-1 | #Feature | #Feature POS\_R15-1 | Start | End | PTM | AScore | Found By |
| --- | --- | --- | --- | --- | --- | --- | --- | --- | --- | --- | --- | --- | --- | --- | --- | --- | --- |
| P.PVVP.L | N | 25.03 | 410.2529 | 4 | 0.6 | 411.2594 | 1 | 13.91 | 5530 | 1.28e4 | 1 | 1 | 262 | 265 |  |  | DB Search |
| P.GVSPGGV.S | Y | 21.17 | 571.2965 | 7 | -8.51 | 572.2975 | 1 | 10.15 | 3967 | 0 | 0 | 0 | 365 | 371 |  |  | DB Search |
| R.VSPP.K | N | 17.71 | 398.2165 | 4 | -2.14 | 399.222 | 1 | 8.11 | 3026 | 0 | 0 | 0 | 383 | 386 |  |  | DB Search |
| S.SGLH.L | N | 16.98 | 412.207 | 4 | -3.29 | 413.2119 | 1 | 1.21 | 286 | 3.65e4 | 1 | 1 | 251 | 254 |  |  | DB Search |
| D.VLNF.Y | N | 16.08 | 491.2744 | 4 | -1.92 | 492.2795 | 1 | 24.95 | 8989 | 2.33e2 | 1 | 1 | 216 | 219 |  |  | DB Search |
| total 5 peptides |
| --- |

Best Unique PSM (Scan POS\_R15-1.wiff:3967, m/z=572.2975, z=1, RT=10.15, ppm=-10.99):


C4R5D4|C4R5D4\_KOMPG

back to list

  

| Protein Coverage
| Supporting Peptides
| Best Unique PSM
|

Protein Coverage:

Supporting Peptides:

| Peptide | Uniq | -10lgP | Mass | Length | ppm | m/z | z | RT | Scan | Area POS\_R15-1 | #Feature | #Feature POS\_R15-1 | Start | End | PTM | AScore | Found By |
| --- | --- | --- | --- | --- | --- | --- | --- | --- | --- | --- | --- | --- | --- | --- | --- | --- | --- |
| S.DYNIQ.K | Y | 20.46 | 651.2864 | 5 | -0.94 | 652.2914 | 1 | 10.88 | 4258 | 1.35e3 | 1 | 1 | 58 | 62 |  |  | DB Search |
| total 1 peptides |
| --- |

Best Unique PSM (Scan POS\_R15-1.wiff:4258, m/z=652.2914, z=1, RT=10.88, ppm=-3.43):


C4R740|C4R740\_KOMPG

back to list

  

| Protein Coverage
| Supporting Peptides
| Best Unique PSM
|

Protein Coverage:

Supporting Peptides:

| Peptide | Uniq | -10lgP | Mass | Length | ppm | m/z | z | RT | Scan | Area POS\_R15-1 | #Feature | #Feature POS\_R15-1 | Start | End | PTM | AScore | Found By |
| --- | --- | --- | --- | --- | --- | --- | --- | --- | --- | --- | --- | --- | --- | --- | --- | --- | --- |
| R.AAQ.M | N | 16.83 | 288.1434 | 3 | 8 | 289.1522 | 1 | 5.91 | 2315 | 6.8e3 | 1 | 1 | 82 | 84 |  |  | DB Search |
| D.NGST.T | N | 16.67 | 377.1547 | 4 | -5.39 | 378.159 | 1 | 16.22 | 6455 | 6.96e2 | 1 | 1 | 123 | 126 |  |  | DB Search |
| Q.M(+15.99)PLSHS.T | Y | 15.28 | 686.3058 | 6 | 3.13 | 687.3135 | 1 | 15.26 | 6041 | 7.13e1 | 1 | 1 | 85 | 90 | Oxidation (M) | M1:Oxidation (M):1000 | DB Search |
| total 3 peptides |
| --- |

Best Unique PSM (Scan POS\_R15-1.wiff:6041, m/z=687.3135, z=1, RT=15.26, ppm=0.65):


C4R6R3|C4R6R3\_KOMPG

back to list

  

| Protein Coverage
| Supporting Peptides
| Best Unique PSM
|

Protein Coverage:

Supporting Peptides:

| Peptide | Uniq | -10lgP | Mass | Length | ppm | m/z | z | RT | Scan | Area POS\_R15-1 | #Feature | #Feature POS\_R15-1 | Start | End | PTM | AScore | Found By |
| --- | --- | --- | --- | --- | --- | --- | --- | --- | --- | --- | --- | --- | --- | --- | --- | --- | --- |
| I.IGGI.S | N | 27.34 | 358.2216 | 4 | -4.39 | 359.2264 | 1 | 15.45 | 6114 | 7.1e3 | 1 | 1 | 423 | 426 |  |  | DB Search |
| N.FYR.K | N | 26.82 | 484.2434 | 3 | 1.64 | 485.2503 | 1 | 17.34 | 6854 | 0 | 0 | 0 | 985 | 987 |  |  | DB Search |
| I.IGGISS.I | Y | 26 | 532.2857 | 6 | -3.42 | 533.2898 | 1 | 10.97 | 4322 | 4.75e3 | 1 | 1 | 423 | 428 |  |  | DB Search |
| T.LGVND.Y | N | 22.39 | 516.2544 | 5 | -3.74 | 517.2584 | 1 | 5.18 | 2080 | 0 | 0 | 0 | 485 | 489 |  |  | DB Search |
| A.TIDI.S | N | 22.27 | 460.2533 | 4 | -8.03 | 461.2557 | 1 | 9.41 | 3625 | 2.97e2 | 1 | 1 | 1043 | 1046 |  |  | DB Search |
| V.VGSI.L | N | 21.91 | 374.2165 | 4 | 0.34 | 375.223 | 1 | 13.82 | 5444 | 2.07e3 | 1 | 1 | 245 | 248 |  |  | DB Search |
| S.ELW.D | N | 21.42 | 446.2165 | 3 | -5.13 | 447.2204 | 1 | 31.30 | 10711 | 1.59e3 | 1 | 1 | 892 | 894 |  |  | DB Search |
| E.WAN.F | N | 21.03 | 389.1699 | 3 | 5.94 | 390.1785 | 1 | 10.28 | 4047 | 1.16e3 | 1 | 1 | 593 | 595 |  |  | DB Search |
| R.TFR.A | N | 20.53 | 422.2278 | 3 | -9.2 | 423.2301 | 1 | 22.38 | 8244 | 2.98e1 | 1 | 1 | 216 | 218 |  |  | DB Search |
| N.RPD.I | N | 19.79 | 386.1914 | 3 | -3.61 | 387.1963 | 1 | 2.95 | 1225 | 1.33e2 | 1 | 1 | 350 | 352 |  |  | DB Search |
| L.APVE.D | N | 19.31 | 414.2114 | 4 | -0.87 | 415.2173 | 1 | 3.61 | 1513 | 3.09e3 | 1 | 1 | 440 | 443 |  |  | DB Search |
| A.TLGV.N | N | 18.91 | 388.2322 | 4 | -5.15 | 389.2365 | 1 | 17.34 | 6852 | 1.43e3 | 1 | 1 | 484 | 487 |  |  | DB Search |
| V.HAVI.I | N | 18.78 | 438.259 | 4 | -6.35 | 439.2625 | 1 | 8.70 | 3348 | 6.21e2 | 1 | 1 | 264 | 267 |  |  | DB Search |
| E.AGE.L | N | 17.5 | 275.1117 | 3 | 5.02 | 276.1197 | 1 | 9.57 | 3694 | 3.1e3 | 1 | 1 | 567 | 569 |  |  | DB Search |
| L.E(-18.01)PR.Q | N | 16.87 | 382.1965 | 3 | -0.37 | 383.2026 | 1 | 2.61 | 1082 | 1.62e4 | 1 | 1 | 997 | 999 | Pyro-glu from E | E1:Pyro-glu from E:1000 | DB Search |
| D.HVW.F | N | 16.29 | 440.2172 | 3 | 3.16 | 441.2248 | 1 | 17.52 | 6872 | 4.96e2 | 1 | 1 | 373 | 375 |  |  | DB Search |
| D.SAIEI.C | N | 16.1 | 531.2904 | 5 | -3.87 | 532.2943 | 1 | 13.79 | 5366 | 7.34e2 | 1 | 1 | 840 | 844 |  |  | DB Search |
| Q.NGV.D | N | 15.37 | 288.1434 | 3 | 8 | 289.1522 | 1 | 5.91 | 2301 | 6.8e3 | 1 | 1 | 113 | 115 |  |  | DB Search |
| A.PVE.D | N | 15.19 | 343.1743 | 3 | -3.43 | 344.1796 | 1 | 12.76 | 5037 | 6.34e2 | 1 | 1 | 441 | 443 |  |  | DB Search |
| total 19 peptides |
| --- |

Best Unique PSM (Scan POS\_R15-1.wiff:4322, m/z=533.2898, z=1, RT=10.97, ppm=-5.9):


C4R5I1|C4R5I1\_KOMPG

back to list

  

| Protein Coverage
| Supporting Peptides
| Best Unique PSM
|

Protein Coverage:

Supporting Peptides:

| Peptide | Uniq | -10lgP | Mass | Length | ppm | m/z | z | RT | Scan | Area POS\_R15-1 | #Feature | #Feature POS\_R15-1 | Start | End | PTM | AScore | Found By |
| --- | --- | --- | --- | --- | --- | --- | --- | --- | --- | --- | --- | --- | --- | --- | --- | --- | --- |
| S.LAGSP.Y | Y | 19.85 | 443.238 | 5 | -4.02 | 444.2424 | 1 | 5.55 | 2243 | 5.62e3 | 1 | 1 | 35 | 39 |  |  | DB Search |
| total 1 peptides |
| --- |

Best Unique PSM (Scan POS\_R15-1.wiff:2243, m/z=444.2822, z=1, RT=5.61, ppm=-6.51):


C4QZF0|C4QZF0\_KOMPG

back to list

  

| Protein Coverage
| Supporting Peptides
| Best Unique PSM
|

Protein Coverage:

Supporting Peptides:

| Peptide | Uniq | -10lgP | Mass | Length | ppm | m/z | z | RT | Scan | Area POS\_R15-1 | #Feature | #Feature POS\_R15-1 | Start | End | PTM | AScore | Found By |
| --- | --- | --- | --- | --- | --- | --- | --- | --- | --- | --- | --- | --- | --- | --- | --- | --- | --- |
| F.LGGI.Y | N | 27.34 | 358.2216 | 4 | -4.39 | 359.2264 | 1 | 15.45 | 6114 | 7.1e3 | 1 | 1 | 159 | 162 |  |  | DB Search |
| P.VGGL.L | N | 24.87 | 344.2059 | 4 | -5.22 | 345.2106 | 1 | 10.74 | 4195 | 1.51e3 | 1 | 1 | 346 | 349 |  |  | DB Search |
| L.GVGP.S | N | 24.4 | 328.1746 | 4 | -1.91 | 329.1805 | 1 | 5.92 | 2228 | 4.71e4 | 1 | 1 | 421 | 424 |  |  | DB Search |
| V.SPT.D | N | 23.16 | 303.143 | 3 | 6.39 | 304.1515 | 1 | 7.14 | 2731 | 5.4e3 | 2 | 2 | 376 | 378 |  |  | DB Search |
| A.GVSI.G | N | 22.74 | 374.2165 | 4 | -2.51 | 375.2219 | 1 | 13.91 | 5529 | 2.07e3 | 1 | 1 | 224 | 227 |  |  | DB Search |
| L.VSSI.L | N | 20.48 | 404.2271 | 4 | -7.08 | 405.2305 | 1 | 8.17 | 2993 | 7.47e3 | 1 | 1 | 216 | 219 |  |  | DB Search |
| P.NSG.S | N | 20.42 | 276.107 | 3 | 6.48 | 277.1154 | 1 | 16.74 | 6592 | 0 | 0 | 0 | 103 | 105 |  |  | DB Search |
| W.FHH.D | N | 18.2 | 439.1968 | 3 | 7.99 | 440.2065 | 1 | 9.67 | 3787 | 2e3 | 1 | 1 | 34 | 36 |  |  | DB Search |
| V.HAVG.D | N | 15.71 | 382.1964 | 4 | -0.36 | 383.2026 | 1 | 2.61 | 1034 | 1.62e4 | 1 | 1 | 175 | 178 |  |  | DB Search |
| W.LDPVGGL.L | Y | 15.29 | 669.3697 | 7 | -7.59 | 670.3702 | 1 | 27.64 | 9721 | 0 | 0 | 0 | 343 | 349 |  |  | DB Search |
| total 10 peptides |
| --- |

Best Unique PSM (Scan POS\_R15-1.wiff:9721, m/z=670.3702, z=1, RT=27.64, ppm=-10.08):


C4R301|C4R301\_KOMPG

back to list

  

| Protein Coverage
| Supporting Peptides
| Best Unique PSM
|

Protein Coverage:

Supporting Peptides:

| Peptide | Uniq | -10lgP | Mass | Length | ppm | m/z | z | RT | Scan | Area POS\_R15-1 | #Feature | #Feature POS\_R15-1 | Start | End | PTM | AScore | Found By |
| --- | --- | --- | --- | --- | --- | --- | --- | --- | --- | --- | --- | --- | --- | --- | --- | --- | --- |
| D.SSLF.D | N | 21.63 | 452.2271 | 4 | -3.7 | 453.2316 | 1 | 22.49 | 8262 | 1.33e3 | 1 | 1 | 418 | 421 |  |  | DB Search |
| D.SSNK.S | N | 20.83 | 434.2125 | 4 | -0.81 | 435.2184 | 1 | 2.82 | 1206 | 3.52e2 | 1 | 1 | 246 | 249 |  |  | DB Search |
| H.SLAP.S | N | 20.83 | 386.2165 | 4 | -4.65 | 387.221 | 1 | 8.35 | 3276 | 4.13e3 | 1 | 1 | 313 | 316 |  |  | DB Search |
| T.QFF.C | N | 19.56 | 440.206 | 3 | 3.92 | 441.2139 | 1 | 19.15 | 7313 | 0 | 0 | 0 | 5 | 7 |  |  | DB Search |
| Q.PDYT.E | N | 17.57 | 494.2013 | 4 | -2.68 | 495.206 | 1 | 9.85 | 3843 | 4.44e3 | 1 | 1 | 21 | 24 |  |  | DB Search |
| N.VSIW.K | N | 17.54 | 503.2744 | 4 | -8.97 | 504.2759 | 1 | 28.75 | 9938 | 1.84e3 | 1 | 1 | 102 | 105 |  |  | DB Search |
| P.HART.A | Y | 16.72 | 483.2554 | 4 | -6.69 | 484.2582 | 1 | 12.92 | 5108 | 0 | 0 | 0 | 462 | 465 |  |  | DB Search |
| S.AAKN.R | N | 16.54 | 402.2227 | 4 | -0.92 | 403.2286 | 1 | 56.57 | 16912 | 1.95e3 | 1 | 1 | 515 | 518 |  |  | DB Search |
| total 8 peptides |
| --- |

Best Unique PSM (Scan POS\_R15-1.wiff:5108, m/z=484.2261, z=1, RT=13.03, ppm=-9.18):


C4QVB3|C4QVB3\_KOMPG

back to list

  

| Protein Coverage
| Supporting Peptides
| Best Unique PSM
|

Protein Coverage:

Supporting Peptides:

| Peptide | Uniq | -10lgP | Mass | Length | ppm | m/z | z | RT | Scan | Area POS\_R15-1 | #Feature | #Feature POS\_R15-1 | Start | End | PTM | AScore | Found By |
| --- | --- | --- | --- | --- | --- | --- | --- | --- | --- | --- | --- | --- | --- | --- | --- | --- | --- |
| Q.Q(-17.03)QQPYP.Y | Y | 28 | 742.3286 | 6 | -0.48 | 743.3337 | 1 | 13.68 | 5404 | 1.42e3 | 1 | 1 | 319 | 324 | Pyro-glu from Q | Q1:Pyro-glu from Q:1000 | DB Search |
| S.SPIL.K | N | 26.66 | 428.2635 | 4 | -1.63 | 429.269 | 1 | 21.35 | 7905 | 0 | 0 | 0 | 489 | 492 |  |  | DB Search |
| N.SLPT.T | N | 22.87 | 416.2271 | 4 | -6.88 | 417.2305 | 1 | 14.64 | 5767 | 5.65e3 | 1 | 1 | 330 | 333 |  |  | DB Search |
| P.GVSI.P | N | 22.74 | 374.2165 | 4 | -2.51 | 375.2219 | 1 | 13.91 | 5529 | 2.07e3 | 1 | 1 | 419 | 422 |  |  | DB Search |
| K.TDVI.R | N | 19.75 | 446.2376 | 4 | -5.56 | 447.2413 | 1 | 13.64 | 5362 | 0 | 0 | 0 | 262 | 265 |  |  | DB Search |
| D.SPSI.W | N | 18.22 | 402.2114 | 4 | -3.42 | 403.2163 | 1 | 12.42 | 4855 | 2.18e3 | 1 | 1 | 18 | 21 |  |  | DB Search |
| Q.QPYP.Y | N | 17.73 | 503.238 | 4 | 1.89 | 504.245 | 1 | 15.02 | 5950 | 0 | 0 | 0 | 321 | 324 |  |  | DB Search |
| total 7 peptides |
| --- |

Best Unique PSM (Scan POS\_R15-1.wiff:5404, m/z=742.3677, z=1, RT=13.93, ppm=-2.96):


C4R4R0|C4R4R0\_KOMPG

back to list

  

| Protein Coverage
| Supporting Peptides
| Best Unique PSM
|

Protein Coverage:

Supporting Peptides:

| Peptide | Uniq | -10lgP | Mass | Length | ppm | m/z | z | RT | Scan | Area POS\_R15-1 | #Feature | #Feature POS\_R15-1 | Start | End | PTM | AScore | Found By |
| --- | --- | --- | --- | --- | --- | --- | --- | --- | --- | --- | --- | --- | --- | --- | --- | --- | --- |
| N.PSLI.D | N | 29.95 | 428.2635 | 4 | -0.56 | 429.2694 | 1 | 21.28 | 7884 | 0 | 0 | 0 | 30 | 33 |  |  | DB Search |
| N.FPS.N | N | 19.27 | 349.1638 | 3 | -4.81 | 350.1685 | 1 | 16.32 | 6461 | 1.41e3 | 1 | 1 | 365 | 367 |  |  | DB Search |
| N.LHDA.D | N | 16.92 | 454.2176 | 4 | -1.55 | 455.223 | 1 | 19.46 | 7407 | 2.98e2 | 1 | 1 | 246 | 249 |  |  | DB Search |
| K.GNLMTCK.E | Y | 16.21 | 765.3513 | 7 | 6.52 | 766.3617 | 1 | 23.57 | 8586 | 2.96e2 | 1 | 1 | 168 | 174 |  |  | DB Search |
| Q.EGS.W | N | 15.77 | 291.1066 | 3 | 7.44 | 292.1154 | 1 | 3.53 | 1538 | 0 | 0 | 0 | 350 | 352 |  |  | DB Search |
| total 5 peptides |
| --- |

Best Unique PSM (Scan POS\_R15-1.wiff:8586, m/z=765.3566, z=2, RT=23.60, ppm=4.04):


C4R051|C4R051\_KOMPG

back to list

  

| Protein Coverage
| Supporting Peptides
| Best Unique PSM
|

Protein Coverage:

Supporting Peptides:

| Peptide | Uniq | -10lgP | Mass | Length | ppm | m/z | z | RT | Scan | Area POS\_R15-1 | #Feature | #Feature POS\_R15-1 | Start | End | PTM | AScore | Found By |
| --- | --- | --- | --- | --- | --- | --- | --- | --- | --- | --- | --- | --- | --- | --- | --- | --- | --- |
| K.PSII.N | N | 29.95 | 428.2635 | 4 | -0.56 | 429.2694 | 1 | 21.28 | 7884 | 0 | 0 | 0 | 1436 | 1439 |  |  | DB Search |
| Y.IGGI.N | N | 27.34 | 358.2216 | 4 | -4.39 | 359.2264 | 1 | 15.45 | 6114 | 7.1e3 | 1 | 1 | 126 | 129 |  |  | DB Search |
| G.ALFG.S | N | 24.47 | 406.2216 | 4 | 1.3 | 407.2284 | 1 | 22.63 | 8312 | 0 | 0 | 0 | 476 | 479 |  |  | DB Search |
| S.VLIP.H | N | 22.17 | 440.2998 | 4 | -5.96 | 441.3034 | 1 | 30.99 | 10567 | 1.25e3 | 1 | 1 | 736 | 739 |  |  | DB Search |
| R.DLGF.G | N | 21.22 | 450.2114 | 4 | 2.45 | 451.2187 | 1 | 25.55 | 9169 | 5.5e2 | 1 | 1 | 1130 | 1133 |  |  | DB Search |
| I.ITDI.S | N | 20.88 | 460.2533 | 4 | -0.2 | 461.2593 | 1 | 14.80 | 5851 | 7.36e2 | 1 | 1 | 1739 | 1742 |  |  | DB Search |
| L.LLQP.L | N | 19.13 | 469.29 | 4 | -8.42 | 470.2922 | 1 | 12.16 | 4756 | 6.43e3 | 1 | 1 | 1497 | 1500 |  |  | DB Search |
| R.TVSGF.M | N | 18.39 | 509.2485 | 5 | -4.28 | 510.2524 | 1 | 14.07 | 5547 | 8e2 | 1 | 1 | 841 | 845 |  |  | DB Search |
| S.LHAD.Y | N | 17.73 | 454.2176 | 4 | 2.41 | 455.2248 | 1 | 17.29 | 6827 | 0 | 0 | 0 | 121 | 124 |  |  | DB Search |
| S.IITD.I | N | 17.52 | 460.2533 | 4 | -9.09 | 461.2552 | 1 | 10.16 | 3965 | 9.46e2 | 1 | 1 | 1738 | 1741 |  |  | DB Search |
| Q.ESW.I | N | 17.43 | 420.1645 | 3 | -0.16 | 421.1707 | 1 | 11.21 | 4460 | 0 | 0 | 0 | 1401 | 1403 |  |  | DB Search |
| R.Q(-17.03)PLI.L | N | 17.35 | 452.2635 | 4 | -6.01 | 453.2669 | 1 | 32.30 | 10953 | 1.74e3 | 1 | 1 | 562 | 565 | Pyro-glu from Q | Q1:Pyro-glu from Q:1000 | DB Search |
| D.LPYYTVGFK.T | Y | 16.8 | 1086.575 | 9 | 2 | 544.2945 | 2 | 10.78 | 4210 | 2.88e3 | 1 | 1 | 809 | 817 |  |  | DB Search |
| D.NGST.S | N | 16.67 | 377.1547 | 4 | -5.39 | 378.159 | 1 | 16.22 | 6455 | 6.96e2 | 1 | 1 | 628 | 631 |  |  | DB Search |
| S.VLNF.N | N | 16.08 | 491.2744 | 4 | -1.92 | 492.2795 | 1 | 24.95 | 8989 | 2.33e2 | 1 | 1 | 338 | 341 |  |  | DB Search |
| L.FSANL.A | N | 15.79 | 550.2751 | 5 | -7.88 | 551.2767 | 1 | 19.15 | 7316 | 0 | 0 | 0 | 1195 | 1199 |  |  | DB Search |
| N.IDLG.Y | N | 15.65 | 416.2271 | 4 | -5.75 | 417.2309 | 1 | 13.92 | 5536 | 5.65e3 | 1 | 1 | 1428 | 1431 |  |  | DB Search |
| A.LKTP.F | N | 15.64 | 457.29 | 4 | -5.17 | 458.2938 | 1 | 15.66 | 6234 | 0 | 0 | 0 | 5 | 8 |  |  | DB Search |
| K.TLQW.L | N | 15.55 | 546.2802 | 4 | 0.72 | 547.2865 | 1 | 22.01 | 8160 | 8.82e1 | 1 | 1 | 1387 | 1390 |  |  | DB Search |
| S.KSIP.S | N | 15.42 | 443.2744 | 4 | -3.64 | 444.2789 | 1 | 17.16 | 6753 | 0 | 0 | 0 | 231 | 234 |  |  | DB Search |
| L.QVAL.I | N | 15.07 | 429.2587 | 4 | 5.6 | 430.2673 | 1 | 21.95 | 8124 | 0 | 0 | 0 | 1690 | 1693 |  |  | DB Search |
| total 21 peptides |
| --- |

Best Unique PSM (Scan POS\_R15-1.wiff:4210, m/z=544.2945, z=2, RT=10.78, ppm=-0.48):


C4QZX1|C4QZX1\_KOMPG

back to list

  

| Protein Coverage
| Supporting Peptides
| Best Unique PSM
|

Protein Coverage:

Supporting Peptides:

| Peptide | Uniq | -10lgP | Mass | Length | ppm | m/z | z | RT | Scan | Area POS\_R15-1 | #Feature | #Feature POS\_R15-1 | Start | End | PTM | AScore | Found By |
| --- | --- | --- | --- | --- | --- | --- | --- | --- | --- | --- | --- | --- | --- | --- | --- | --- | --- |
| A.FYR.R | N | 26.82 | 484.2434 | 3 | 1.64 | 485.2503 | 1 | 17.34 | 6854 | 0 | 0 | 0 | 13 | 15 |  |  | DB Search |
| K.E(-18.01)ISESE.R | Y | 19.46 | 674.2759 | 6 | -6.05 | 675.2774 | 1 | 11.31 | 4525 | 0 | 0 | 0 | 113 | 118 | Pyro-glu from E | E1:Pyro-glu from E:1000 | DB Search |
| D.STSP.Q | N | 19.15 | 390.1751 | 4 | -0.08 | 391.1813 | 1 | 5.92 | 2360 | 3.31e2 | 1 | 1 | 94 | 97 |  |  | DB Search |
| V.RSF.E | N | 17.5 | 408.2121 | 3 | -1.76 | 409.2177 | 1 | 9.17 | 3526 | 0 | 0 | 0 | 190 | 192 |  |  | DB Search |
| A.TCR.K | N | 17.37 | 378.1685 | 3 | 5.06 | 379.1768 | 1 | 12.47 | 4890 | 0 | 0 | 0 | 196 | 198 |  |  | DB Search |
| K.E(-18.01)ISES.E | N | 15.77 | 545.2333 | 5 | -9.55 | 546.234 | 1 | 10.09 | 3935 | 1.92e3 | 1 | 1 | 113 | 117 | Pyro-glu from E | E1:Pyro-glu from E:1000 | DB Search |
| total 6 peptides |
| --- |

Best Unique PSM (Scan POS\_R15-1.wiff:4525, m/z=675.2774, z=1, RT=11.31, ppm=-8.54):


C4R3M1|C4R3M1\_KOMPG

back to list

  

| Protein Coverage
| Supporting Peptides
| Best Unique PSM
|

Protein Coverage:

Supporting Peptides:

| Peptide | Uniq | -10lgP | Mass | Length | ppm | m/z | z | RT | Scan | Area POS\_R15-1 | #Feature | #Feature POS\_R15-1 | Start | End | PTM | AScore | Found By |
| --- | --- | --- | --- | --- | --- | --- | --- | --- | --- | --- | --- | --- | --- | --- | --- | --- | --- |
| L.ITGL.L | N | 27.55 | 402.2478 | 4 | -1.76 | 403.2534 | 1 | 15.04 | 5964 | 1.56e4 | 2 | 2 | 623 | 626 |  |  | DB Search |
| S.SPT.R | N | 23.16 | 303.143 | 3 | 6.39 | 304.1515 | 1 | 7.14 | 2731 | 5.4e3 | 2 | 2 | 24 | 26 |  |  | DB Search |
| S.VLIP.E | N | 22.17 | 440.2998 | 4 | -5.96 | 441.3034 | 1 | 30.99 | 10567 | 1.25e3 | 1 | 1 | 298 | 301 |  |  | DB Search |
| S.ERPLDSVVD.D | Y | 22.01 | 1028.5138 | 9 | -3.3 | 1029.5151 | 1 | 30.42 | 10438 | 1.01e2 | 1 | 1 | 686 | 694 |  |  | DB Search |
| S.THH.F | N | 21.55 | 393.1761 | 3 | -4.54 | 394.1806 | 1 | 2.11 | 908 | 2.12e3 | 1 | 1 | 13 | 15 |  |  | DB Search |
| V.LSVS.K | N | 20.49 | 404.2271 | 4 | -7.08 | 405.2305 | 1 | 8.17 | 3035 | 7.47e3 | 1 | 1 | 46 | 49 |  |  | DB Search |
| K.LPIP.L | N | 20.33 | 438.2842 | 4 | -7.82 | 439.287 | 1 | 27.42 | 9635 | 1.51e3 | 1 | 1 | 162 | 165 |  |  | DB Search |
| L.ISVP.S | N | 19.74 | 414.2478 | 4 | -0.09 | 415.254 | 1 | 17.14 | 6914 | 3.72e3 | 1 | 1 | 231 | 234 |  |  | DB Search |
| H.QFF.A | N | 19.56 | 440.206 | 3 | 3.92 | 441.2139 | 1 | 19.15 | 7313 | 0 | 0 | 0 | 647 | 649 |  |  | DB Search |
| D.TSN.F | N | 17.54 | 320.1332 | 3 | 3.34 | 321.1407 | 1 | 8.11 | 3024 | 0 | 0 | 0 | 676 | 678 |  |  | DB Search |
| N.SPK.F | N | 16.89 | 330.1903 | 3 | -8.46 | 331.194 | 1 | 5.91 | 2235 | 5.17e3 | 1 | 1 | 269 | 271 |  |  | DB Search |
| L.LSAI.E | N | 16.63 | 402.2478 | 4 | -8.97 | 403.2505 | 1 | 9.42 | 3655 | 1.65e3 | 1 | 1 | 502 | 505 |  |  | DB Search |
| T.TFDV.S | N | 16.44 | 480.222 | 4 | -1.51 | 481.2274 | 1 | 11.18 | 4442 | 1.56e3 | 1 | 1 | 277 | 280 |  |  | DB Search |
| Y.DPTP.V | N | 16.05 | 428.1907 | 4 | 7.39 | 429.2001 | 1 | 11.23 | 4471 | 0 | 0 | 0 | 238 | 241 |  |  | DB Search |
| N.KST.D | N | 15.47 | 334.1852 | 3 | 0.84 | 335.192 | 1 | 54.68 | 16215 | 3.66e2 | 1 | 1 | 341 | 343 |  |  | DB Search |
| T.KSIP.A | N | 15.42 | 443.2744 | 4 | -3.64 | 444.2789 | 1 | 17.16 | 6753 | 0 | 0 | 0 | 225 | 228 |  |  | DB Search |
| total 16 peptides |
| --- |

Best Unique PSM (Scan POS\_R15-1.wiff:10438, m/z=1029.5151, z=1, RT=30.42, ppm=-5.78):


C4R3L9|C4R3L9\_KOMPG

back to list

  

| Protein Coverage
| Supporting Peptides
| Best Unique PSM
|

Protein Coverage:

Supporting Peptides:

| Peptide | Uniq | -10lgP | Mass | Length | ppm | m/z | z | RT | Scan | Area POS\_R15-1 | #Feature | #Feature POS\_R15-1 | Start | End | PTM | AScore | Found By |
| --- | --- | --- | --- | --- | --- | --- | --- | --- | --- | --- | --- | --- | --- | --- | --- | --- | --- |
| R.SPLL.T | N | 26.66 | 428.2635 | 4 | -1.63 | 429.269 | 1 | 21.35 | 7905 | 0 | 0 | 0 | 325 | 328 |  |  | DB Search |
| V.VGGI.A | N | 24.87 | 344.2059 | 4 | -5.22 | 345.2106 | 1 | 10.74 | 4195 | 1.51e3 | 1 | 1 | 169 | 172 |  |  | DB Search |
| I.VGSL.L | N | 21.91 | 374.2165 | 4 | 0.34 | 375.223 | 1 | 13.82 | 5444 | 2.07e3 | 1 | 1 | 261 | 264 |  |  | DB Search |
| K.IPRP.T | N | 21.04 | 481.3012 | 4 | -9.61 | 482.3027 | 1 | 17.02 | 6682 | 0 | 0 | 0 | 4 | 7 |  |  | DB Search |
| T.TAY.I | N | 19.3 | 353.1587 | 3 | -1.32 | 354.1646 | 1 | 12.51 | 4913 | 1.39e3 | 1 | 1 | 330 | 332 |  |  | DB Search |
| S.SVGL.A | N | 18.49 | 374.2165 | 4 | -4.96 | 375.221 | 1 | 14.62 | 5748 | 1.93e2 | 1 | 1 | 68 | 71 |  |  | DB Search |
| V.VAVG.T | N | 18.17 | 344.2059 | 4 | -3.72 | 345.2111 | 1 | 5.51 | 2206 | 3.05e3 | 1 | 1 | 127 | 130 |  |  | DB Search |
| C.FAAGAP.L | Y | 16.81 | 532.2645 | 6 | 3.39 | 533.2723 | 1 | 9.60 | 3727 | 9.26e2 | 1 | 1 | 155 | 160 |  |  | DB Search |
| total 8 peptides |
| --- |

Best Unique PSM (Scan POS\_R15-1.wiff:3727, m/z=533.2723, z=1, RT=9.60, ppm=0.91):


C4QW22|C4QW22\_KOMPG

back to list

  

| Protein Coverage
| Supporting Peptides
| Best Unique PSM
|

Protein Coverage:

Supporting Peptides:

| Peptide | Uniq | -10lgP | Mass | Length | ppm | m/z | z | RT | Scan | Area POS\_R15-1 | #Feature | #Feature POS\_R15-1 | Start | End | PTM | AScore | Found By |
| --- | --- | --- | --- | --- | --- | --- | --- | --- | --- | --- | --- | --- | --- | --- | --- | --- | --- |
| P.VAAP.P | N | 23.43 | 356.2059 | 4 | -1.11 | 357.2119 | 1 | 7.38 | 2801 | 2.39e3 | 1 | 1 | 211 | 214 |  |  | DB Search |
| T.LDQSPATV.A | Y | 22.38 | 829.4181 | 8 | 0.14 | 830.4235 | 1 | 15.30 | 6034 | 3.76e3 | 1 | 1 | 173 | 180 |  |  | DB Search |
| M.ADLW.G | N | 17.86 | 503.238 | 4 | -1.06 | 504.2435 | 1 | 28.73 | 10039 | 1.84e3 | 1 | 1 | 97 | 100 |  |  | DB Search |
| R.LSAI.I | N | 16.63 | 402.2478 | 4 | -8.97 | 403.2505 | 1 | 9.42 | 3655 | 1.65e3 | 1 | 1 | 11 | 14 |  |  | DB Search |
| A.TVAP.T | N | 15.74 | 386.2165 | 4 | -8.68 | 387.2195 | 1 | 10.63 | 4140 | 0 | 0 | 0 | 179 | 182 |  |  | DB Search |
| total 5 peptides |
| --- |

Best Unique PSM (Scan POS\_R15-1.wiff:6034, m/z=830.4235, z=1, RT=15.30, ppm=-2.34):


C4R9E1|C4R9E1\_KOMPG

back to list

  

| Protein Coverage
| Supporting Peptides
| Best Unique PSM
|

Protein Coverage:

Supporting Peptides:

| Peptide | Uniq | -10lgP | Mass | Length | ppm | m/z | z | RT | Scan | Area POS\_R15-1 | #Feature | #Feature POS\_R15-1 | Start | End | PTM | AScore | Found By |
| --- | --- | --- | --- | --- | --- | --- | --- | --- | --- | --- | --- | --- | --- | --- | --- | --- | --- |
| E.LTGI.A | N | 27.55 | 402.2478 | 4 | -1.76 | 403.2534 | 1 | 15.04 | 5964 | 1.56e4 | 2 | 2 | 276 | 279 |  |  | DB Search |
| F.TAY.V | N | 19.3 | 353.1587 | 3 | -1.32 | 354.1646 | 1 | 12.51 | 4913 | 1.39e3 | 1 | 1 | 502 | 504 |  |  | DB Search |
| T.DAVP.I | N | 18.95 | 400.1958 | 4 | -2.37 | 401.2011 | 1 | 8.27 | 3131 | 4.2e3 | 1 | 1 | 171 | 174 |  |  | DB Search |
| A.AVAG.L | N | 17.64 | 316.1746 | 4 | -4.4 | 317.1797 | 1 | 5.33 | 2131 | 0 | 0 | 0 | 431 | 434 |  |  | DB Search |
| K.AVGF.A | N | 17.44 | 392.2059 | 4 | -3.97 | 393.2107 | 1 | 15.98 | 6324 | 2.62e3 | 1 | 1 | 138 | 141 |  |  | DB Search |
| A.WYSA.I | Y | 17.02 | 525.2223 | 4 | -5.77 | 526.2253 | 1 | 15.71 | 6227 | 2.36e3 | 1 | 1 | 474 | 477 |  |  | DB Search |
| W.HKF.W | N | 16.83 | 430.2328 | 3 | 9.29 | 431.243 | 1 | 12.31 | 4825 | 5.31e3 | 1 | 1 | 556 | 558 |  |  | DB Search |
| A.TFT.P | N | 15.92 | 367.1743 | 3 | -7.47 | 368.1779 | 1 | 8.54 | 3263 | 0 | 0 | 0 | 119 | 121 |  |  | DB Search |
| total 8 peptides |
| --- |

Best Unique PSM (Scan POS\_R15-1.wiff:6227, m/z=526.2253, z=1, RT=15.71, ppm=-8.25):


C4R0P1|C4R0P1\_KOMPG

back to list

  

| Protein Coverage
| Supporting Peptides
| Best Unique PSM
|

Protein Coverage:

Supporting Peptides:

| Peptide | Uniq | -10lgP | Mass | Length | ppm | m/z | z | RT | Scan | Area POS\_R15-1 | #Feature | #Feature POS\_R15-1 | Start | End | PTM | AScore | Found By |
| --- | --- | --- | --- | --- | --- | --- | --- | --- | --- | --- | --- | --- | --- | --- | --- | --- | --- |
| R.VPTVDVSVVD.L | Y | 60.49 | 1028.539 | 10 | -6.03 | 1029.5375 | 1 | 30.36 | 10434 | 0 | 0 | 0 | 234 | 243 |  |  | DB Search |
| R.VPTVDVS.V | Y | 37.21 | 715.3752 | 7 | -2.34 | 716.379 | 1 | 15.28 | 6054 | 2.47e3 | 2 | 2 | 234 | 240 |  |  | DB Search |
| T.VDVSVVD.L | Y | 36.35 | 731.3701 | 7 | -3.95 | 732.3727 | 1 | 17.66 | 6952 | 2.47e3 | 1 | 1 | 237 | 243 |  |  | DB Search |
| G.NII.P | N | 28.22 | 358.2216 | 3 | -4.68 | 359.2263 | 1 | 19.18 | 7326 | 4.19e3 | 1 | 1 | 204 | 206 |  |  | DB Search |
| K.LTGL.A | N | 27.55 | 402.2478 | 4 | -1.76 | 403.2534 | 1 | 15.04 | 5964 | 1.56e4 | 2 | 2 | 227 | 230 |  |  | DB Search |
| A.SAGIQ.L | N | 23.04 | 474.2438 | 5 | -3.9 | 475.248 | 1 | 3.16 | 1372 | 1.15e3 | 1 | 1 | 297 | 301 |  |  | DB Search |
| R.VPTVD.V | Y | 22.67 | 529.2748 | 5 | -8.92 | 530.276 | 1 | 9.43 | 3636 | 0 | 0 | 0 | 234 | 238 |  |  | DB Search |
| N.EI.T | N | 22.54 | 260.1372 | 2 | -4.37 | 261.1427 | 1 | 8.17 | 3066 | 4.03e4 | 1 | 1 | 72 | 73 |  |  | DB Search |
| P.SHKDWR.G | Y | 22.06 | 827.4038 | 6 | 9.61 | 828.417 | 1 | 14.99 | 5914 | 7.04e2 | 1 | 1 | 191 | 196 |  |  | DB Search |
| G.EV.S | N | 22.06 | 246.1216 | 2 | -3.05 | 247.1275 | 1 | 3.37 | 1479 | 1.39e4 | 2 | 2 | 58 | 59 |  |  | DB Search |
| D.VSVVD.L | N | 21.8 | 517.2748 | 5 | -5.76 | 518.2778 | 1 | 9.99 | 3883 | 0 | 0 | 0 | 239 | 243 |  |  | DB Search |
| R.SSIF.D | N | 21.63 | 452.2271 | 4 | -3.7 | 453.2316 | 1 | 22.49 | 8262 | 1.33e3 | 1 | 1 | 291 | 294 |  |  | DB Search |
| E.KYT.S | N | 21.18 | 410.2165 | 3 | -4.93 | 411.2208 | 1 | 12.54 | 4954 | 3.83e2 | 1 | 1 | 138 | 140 |  |  | DB Search |
| V.SSD.F | N | 20.1 | 307.1016 | 3 | 8.02 | 308.1105 | 1 | 10.67 | 4164 | 0 | 0 | 0 | 282 | 284 |  |  | DB Search |
| S.VVDL.T | N | 19.9 | 444.2584 | 4 | 0.77 | 445.2649 | 1 | 17.32 | 6776 | 2.15e3 | 1 | 1 | 241 | 244 |  |  | DB Search |
| V.NDT.F | N | 19.64 | 348.1281 | 3 | 7.11 | 349.137 | 1 | 7.31 | 2704 | 6.3e4 | 1 | 1 | 164 | 166 |  |  | DB Search |
| L.AFR.V | N | 19.1 | 392.2172 | 3 | -7.11 | 393.2207 | 1 | 6.33 | 2475 | 0 | 0 | 0 | 231 | 233 |  |  | DB Search |
| G.RT.A | N | 19.04 | 275.1593 | 2 | 3.6 | 276.1669 | 1 | 5.27 | 2115 | 2.64e3 | 1 | 1 | 199 | 200 |  |  | DB Search |
| V.LRV.A | N | 18.9 | 386.2641 | 3 | -1.28 | 387.27 | 1 | 27.77 | 9760 | 0 | 0 | 0 | 18 | 20 |  |  | DB Search |
| A.DI.K | N | 18.87 | 246.1216 | 2 | -3.79 | 247.1273 | 1 | 6.12 | 2386 | 4.36e3 | 2 | 2 | 26 | 27 |  |  | DB Search |
| V.DVSV.V | N | 18.79 | 418.2063 | 4 | -2.48 | 419.2115 | 1 | 4.65 | 1733 | 3.85e3 | 1 | 1 | 238 | 241 |  |  | DB Search |
| N.IP.W | N | 18.63 | 228.1474 | 2 | -2.29 | 229.1536 | 1 | 2.07 | 883 | 8.16e3 | 1 | 1 | 84 | 85 |  |  | DB Search |
| V.LR.V | N | 18.3 | 287.1957 | 2 | 0.04 | 288.2023 | 1 | 2.10 | 904 | 0 | 0 | 0 | 18 | 19 |  |  | DB Search |
| E.SGLM.T | N | 18.1 | 406.1886 | 4 | -5.81 | 407.1925 | 1 | 11.12 | 4398 | 1.56e3 | 1 | 1 | 171 | 174 |  |  | DB Search |
| Q.LTP.S | N | 17.99 | 329.1951 | 3 | 1.36 | 330.202 | 1 | 7.49 | 2815 | 1.47e3 | 1 | 1 | 302 | 304 |  |  | DB Search |
| A.FRVPTVDVSVVDL.T | Y | 17.65 | 1444.7925 | 13 | 2.09 | 723.4033 | 2 | 44.03 | 13671 | 0 | 0 | 0 | 232 | 244 |  |  | DB Search |
| G.FGRI.G | N | 17.51 | 491.2856 | 4 | -7.95 | 492.2878 | 1 | 15.01 | 5965 | 9.54e2 | 1 | 1 | 10 | 13 |  |  | DB Search |
| R.ADI.K | N | 17.27 | 317.1587 | 3 | -4.79 | 318.1636 | 1 | 3.30 | 1336 | 3.11e3 | 1 | 1 | 25 | 27 |  |  | DB Search |
| G.INGF.G | N | 16.9 | 449.2274 | 4 | 0.62 | 450.2339 | 1 | 15.55 | 6174 | 0 | 0 | 0 | 7 | 10 |  |  | DB Search |
| I.TV.G | N | 16.54 | 218.1266 | 2 | -6.32 | 219.132 | 1 | 6.06 | 2461 | 9.87e2 | 1 | 1 | 4 | 5 |  |  | DB Search |
| M.FVVG.V | N | 16.5 | 420.2372 | 4 | -7.94 | 421.2401 | 1 | 13.63 | 5446 | 6.2e2 | 1 | 1 | 130 | 133 |  |  | DB Search |
| M.TTVH.S | N | 16.14 | 456.2332 | 4 | 1.87 | 457.2402 | 1 | 17.22 | 6793 | 1.04e2 | 1 | 1 | 175 | 178 |  |  | DB Search |
| total 32 peptides |
| --- |

Best Unique PSM (Scan POS\_R15-1.wiff:10434, m/z=1029.5151, z=1, RT=30.42, ppm=-8.52):


C4R7T5|C4R7T5\_KOMPG

back to list

  

| Protein Coverage
| Supporting Peptides
| Best Unique PSM
|

Protein Coverage:

Supporting Peptides:

| Peptide | Uniq | -10lgP | Mass | Length | ppm | m/z | z | RT | Scan | Area POS\_R15-1 | #Feature | #Feature POS\_R15-1 | Start | End | PTM | AScore | Found By |
| --- | --- | --- | --- | --- | --- | --- | --- | --- | --- | --- | --- | --- | --- | --- | --- | --- | --- |
| T.ASAI.H | N | 22.98 | 360.2009 | 4 | -6.45 | 361.2049 | 1 | 8.68 | 3069 | 1.41e4 | 1 | 1 | 414 | 417 |  |  | DB Search |
| P.ETLISE.P | Y | 22.28 | 690.3436 | 6 | -5.58 | 691.3453 | 1 | 17.12 | 6821 | 2.46e2 | 1 | 1 | 282 | 287 |  |  | DB Search |
| D.WSD.E | N | 19.99 | 406.1488 | 3 | -8.45 | 407.1517 | 1 | 8.75 | 3390 | 0 | 0 | 0 | 122 | 124 |  |  | DB Search |
| P.QFF.N | N | 19.56 | 440.206 | 3 | 3.92 | 441.2139 | 1 | 19.15 | 7313 | 0 | 0 | 0 | 238 | 240 |  |  | DB Search |
| L.APY.G | N | 16.84 | 349.1638 | 3 | -4.81 | 350.1685 | 1 | 16.32 | 6384 | 1.41e3 | 1 | 1 | 83 | 85 |  |  | DB Search |
| total 5 peptides |
| --- |

Best Unique PSM (Scan POS\_R15-1.wiff:6821, m/z=691.3414, z=1, RT=17.28, ppm=-8.07):


C4QXJ4|C4QXJ4\_KOMPG

back to list

  

| Protein Coverage
| Supporting Peptides
| Best Unique PSM
|

Protein Coverage:

Supporting Peptides:

| Peptide | Uniq | -10lgP | Mass | Length | ppm | m/z | z | RT | Scan | Area POS\_R15-1 | #Feature | #Feature POS\_R15-1 | Start | End | PTM | AScore | Found By |
| --- | --- | --- | --- | --- | --- | --- | --- | --- | --- | --- | --- | --- | --- | --- | --- | --- | --- |
| S.VGGI.Y | N | 24.87 | 344.2059 | 4 | -5.22 | 345.2106 | 1 | 10.74 | 4195 | 1.51e3 | 1 | 1 | 339 | 342 |  |  | DB Search |
| L.VLLGS.R | N | 23.87 | 487.3006 | 5 | 1.5 | 488.3074 | 1 | 15.69 | 6182 | 1.57e3 | 1 | 1 | 360 | 364 |  |  | DB Search |
| E.PNPGPTH.S | Y | 23.38 | 718.3398 | 7 | 1.31 | 719.3463 | 1 | 19.08 | 7299 | 1.22e3 | 1 | 1 | 309 | 315 |  |  | DB Search |
| K.LVAP.S | N | 22.5 | 398.2529 | 4 | -6.51 | 399.2566 | 1 | 9.50 | 3744 | 1.49e3 | 1 | 1 | 163 | 166 |  |  | DB Search |
| G.IYSG.T | N | 19.57 | 438.2114 | 4 | -3.08 | 439.2163 | 1 | 7.95 | 2941 | 3.53e4 | 1 | 1 | 342 | 345 |  |  | DB Search |
| C.SNG.G | N | 19.07 | 276.107 | 3 | 1.61 | 277.114 | 1 | 16.82 | 6604 | 0 | 0 | 0 | 301 | 303 |  |  | DB Search |
| V.ESW.K | N | 17.43 | 420.1645 | 3 | -0.16 | 421.1707 | 1 | 11.21 | 4460 | 0 | 0 | 0 | 276 | 278 |  |  | DB Search |
| R.NGST.L | N | 16.67 | 377.1547 | 4 | -5.39 | 378.159 | 1 | 16.22 | 6455 | 6.96e2 | 1 | 1 | 138 | 141 |  |  | DB Search |
| E.STAI.N | N | 15.79 | 390.2114 | 4 | -5.32 | 391.2157 | 1 | 6.25 | 2317 | 1.07e4 | 1 | 1 | 89 | 92 |  |  | DB Search |
| total 9 peptides |
| --- |

Best Unique PSM (Scan POS\_R15-1.wiff:7299, m/z=719.3491, z=2, RT=19.12, ppm=-1.17):


C4R836|C4R836\_KOMPG

back to list

  

| Protein Coverage
| Supporting Peptides
| Best Unique PSM
|

Protein Coverage:

Supporting Peptides:

| Peptide | Uniq | -10lgP | Mass | Length | ppm | m/z | z | RT | Scan | Area POS\_R15-1 | #Feature | #Feature POS\_R15-1 | Start | End | PTM | AScore | Found By |
| --- | --- | --- | --- | --- | --- | --- | --- | --- | --- | --- | --- | --- | --- | --- | --- | --- | --- |
| T.LAGSP.N | Y | 19.85 | 443.238 | 5 | -4.02 | 444.2424 | 1 | 5.55 | 2243 | 5.62e3 | 1 | 1 | 235 | 239 |  |  | DB Search |
| H.RPD.V | N | 19.79 | 386.1914 | 3 | -3.61 | 387.1963 | 1 | 2.95 | 1225 | 1.33e2 | 1 | 1 | 96 | 98 |  |  | DB Search |
| S.AAQ.Y | N | 16.83 | 288.1434 | 3 | 8 | 289.1522 | 1 | 5.91 | 2315 | 6.8e3 | 1 | 1 | 21 | 23 |  |  | DB Search |
| total 3 peptides |
| --- |

Best Unique PSM (Scan POS\_R15-1.wiff:2243, m/z=444.2822, z=1, RT=5.61, ppm=-6.51):


C4R0F7|C4R0F7\_KOMPG

back to list

  

| Protein Coverage
| Supporting Peptides
| Best Unique PSM
|

Protein Coverage:

Supporting Peptides:

| Peptide | Uniq | -10lgP | Mass | Length | ppm | m/z | z | RT | Scan | Area POS\_R15-1 | #Feature | #Feature POS\_R15-1 | Start | End | PTM | AScore | Found By |
| --- | --- | --- | --- | --- | --- | --- | --- | --- | --- | --- | --- | --- | --- | --- | --- | --- | --- |
| A.TSIA.E | N | 24.8 | 390.2114 | 4 | -5.32 | 391.2157 | 1 | 6.25 | 2416 | 1.07e4 | 1 | 1 | 42 | 45 |  |  | DB Search |
| K.TATSIA.E | Y | 22.4 | 562.2962 | 6 | -9.2 | 563.2969 | 1 | 8.27 | 3134 | 0 | 0 | 0 | 40 | 45 |  |  | DB Search |
| S.IAEV.E | N | 19.27 | 430.2427 | 4 | 1.69 | 431.2497 | 1 | 13.56 | 5318 | 0 | 0 | 0 | 44 | 47 |  |  | DB Search |
| Q.ATGL.E | N | 17.01 | 360.2009 | 4 | -0.18 | 361.2072 | 1 | 8.07 | 3002 | 1.41e4 | 1 | 1 | 70 | 73 |  |  | DB Search |
| I.AEVE.G | N | 16.37 | 446.2013 | 4 | -1.36 | 447.2068 | 1 | 4.90 | 1940 | 0 | 0 | 0 | 45 | 48 |  |  | DB Search |
| total 5 peptides |
| --- |

Best Unique PSM (Scan POS\_R15-1.wiff:3134, m/z=563.2959, z=1, RT=8.30, ppm=-11.68):


C4R1Z6|C4R1Z6\_KOMPG

back to list

  

| Protein Coverage
| Supporting Peptides
| Best Unique PSM
|

Protein Coverage:

Supporting Peptides:

| Peptide | Uniq | -10lgP | Mass | Length | ppm | m/z | z | RT | Scan | Area POS\_R15-1 | #Feature | #Feature POS\_R15-1 | Start | End | PTM | AScore | Found By |
| --- | --- | --- | --- | --- | --- | --- | --- | --- | --- | --- | --- | --- | --- | --- | --- | --- | --- |
| S.GGGL.F | N | 27.46 | 302.159 | 4 | -3.72 | 303.1644 | 1 | 6.06 | 2387 | 3.14e3 | 1 | 1 | 91 | 94 |  |  | DB Search |
| L.VLGS.L | N | 24.2 | 374.2165 | 4 | -4.06 | 375.2213 | 1 | 5.20 | 2012 | 6.27e3 | 1 | 1 | 173 | 176 |  |  | DB Search |
| T.KFH.P | N | 20.21 | 430.2328 | 3 | 9.29 | 431.243 | 1 | 12.31 | 5009 | 5.31e3 | 1 | 1 | 543 | 545 |  |  | DB Search |
| N.QFF.G | N | 19.56 | 440.206 | 3 | 3.92 | 441.2139 | 1 | 19.15 | 7313 | 0 | 0 | 0 | 538 | 540 |  |  | DB Search |
| W.RSSS.W | N | 18.8 | 435.2078 | 4 | -3.98 | 436.2122 | 1 | 11.38 | 4548 | 7.31e2 | 1 | 1 | 875 | 878 |  |  | DB Search |
| P.SVGL.F | N | 18.49 | 374.2165 | 4 | -4.96 | 375.221 | 1 | 14.62 | 5748 | 1.93e2 | 1 | 1 | 106 | 109 |  |  | DB Search |
| S.SPSI.E | N | 18.22 | 402.2114 | 4 | -3.42 | 403.2163 | 1 | 12.42 | 4855 | 2.18e3 | 1 | 1 | 357 | 360 |  |  | DB Search |
| L.FGDP.E | N | 17.87 | 434.1801 | 4 | -4.75 | 435.1843 | 1 | 12.45 | 4977 | 1.27e3 | 1 | 1 | 659 | 662 |  |  | DB Search |
| H.IGGE.V | N | 17.74 | 374.1801 | 4 | 0.95 | 375.1868 | 1 | 2.99 | 1264 | 7.34e3 | 1 | 1 | 1152 | 1155 |  |  | DB Search |
| F.TTNDSFTF.A | Y | 17.62 | 931.3923 | 8 | 0.52 | 932.3978 | 1 | 22.31 | 8216 | 2.42e2 | 1 | 1 | 745 | 752 |  |  | DB Search |
| P.Q(-17.03)PIL.K | N | 17.35 | 452.2635 | 4 | -6.01 | 453.2669 | 1 | 32.30 | 10953 | 1.74e3 | 1 | 1 | 884 | 887 | Pyro-glu from Q | Q1:Pyro-glu from Q:1000 | DB Search |
| Y.Q(-17.03)PH.S | N | 15.48 | 363.1543 | 3 | -0.14 | 364.1606 | 1 | 2.32 | 992 | 3.39e4 | 1 | 1 | 209 | 211 | Pyro-glu from Q | Q1:Pyro-glu from Q:1000 | DB Search |
| P.APVT.S | N | 15.47 | 386.2165 | 4 | -4.26 | 387.2212 | 1 | 3.33 | 1445 | 2.99e3 | 1 | 1 | 44 | 47 |  |  | DB Search |
| S.TFGGP.S | N | 15.12 | 477.2223 | 5 | 3.53 | 478.2301 | 1 | 12.91 | 5098 | 0 | 0 | 0 | 27 | 31 |  |  | DB Search |
| total 14 peptides |
| --- |

Best Unique PSM (Scan POS\_R15-1.wiff:8216, m/z=932.3978, z=1, RT=22.31, ppm=-1.96):


C4R1W6|C4R1W6\_KOMPG

back to list

  

| Protein Coverage
| Supporting Peptides
| Best Unique PSM
|

Protein Coverage:

Supporting Peptides:

| Peptide | Uniq | -10lgP | Mass | Length | ppm | m/z | z | RT | Scan | Area POS\_R15-1 | #Feature | #Feature POS\_R15-1 | Start | End | PTM | AScore | Found By |
| --- | --- | --- | --- | --- | --- | --- | --- | --- | --- | --- | --- | --- | --- | --- | --- | --- | --- |
| E.LTPMT.R | Y | 23.09 | 561.2832 | 5 | -6.07 | 562.2857 | 1 | 20.23 | 7638 | 4.29e3 | 1 | 1 | 26 | 30 |  |  | DB Search |
| L.TLDL.P | N | 22.27 | 460.2533 | 4 | -8.03 | 461.2557 | 1 | 9.41 | 3625 | 2.97e2 | 1 | 1 | 414 | 417 |  |  | DB Search |
| R.VSSL.S | N | 20.48 | 404.2271 | 4 | -7.08 | 405.2305 | 1 | 8.17 | 2993 | 7.47e3 | 1 | 1 | 35 | 38 |  |  | DB Search |
| D.ESW.Y | N | 17.43 | 420.1645 | 3 | -0.16 | 421.1707 | 1 | 11.21 | 4460 | 0 | 0 | 0 | 442 | 444 |  |  | DB Search |
| T.PMTRR.K | Y | 16.48 | 659.3537 | 5 | -2.37 | 660.3578 | 1 | 8.25 | 3083 | 5.46e3 | 1 | 1 | 28 | 32 |  |  | DB Search |
| V.IGGV.G | N | 15.94 | 344.2059 | 4 | 0.19 | 345.2124 | 1 | 5.48 | 2143 | 3.05e3 | 1 | 1 | 182 | 185 |  |  | DB Search |
| total 6 peptides |
| --- |

Best Unique PSM (Scan POS\_R15-1.wiff:7638, m/z=562.286, z=2, RT=20.28, ppm=-8.55):


C4R701|C4R701\_KOMPG

back to list

  

| Protein Coverage
| Supporting Peptides
| Best Unique PSM
|

Protein Coverage:

Supporting Peptides:

| Peptide | Uniq | -10lgP | Mass | Length | ppm | m/z | z | RT | Scan | Area POS\_R15-1 | #Feature | #Feature POS\_R15-1 | Start | End | PTM | AScore | Found By |
| --- | --- | --- | --- | --- | --- | --- | --- | --- | --- | --- | --- | --- | --- | --- | --- | --- | --- |
| V.IPPP.T | N | 27.73 | 422.2529 | 4 | -1.45 | 423.2585 | 1 | 9.57 | 3721 | 1.63e3 | 1 | 1 | 356 | 359 |  |  | DB Search |
| N.LGGL.T | N | 27.34 | 358.2216 | 4 | -4.39 | 359.2264 | 1 | 15.45 | 6114 | 7.1e3 | 1 | 1 | 393 | 396 |  |  | DB Search |
| V.TIPT.I | N | 26.11 | 430.2427 | 4 | -4.36 | 431.2471 | 1 | 9.16 | 3520 | 0 | 0 | 0 | 377 | 380 |  |  | DB Search |
| S.ALGL.G | N | 25.12 | 372.2372 | 4 | -5.1 | 373.2417 | 1 | 21.84 | 8058 | 0 | 0 | 0 | 77 | 80 |  |  | DB Search |
| L.TVGVAGI.A | Y | 23.87 | 615.3591 | 7 | -0.94 | 616.3643 | 1 | 23.76 | 8599 | 2.96e3 | 1 | 1 | 397 | 403 |  |  | DB Search |
| I.VPVE.P | N | 20.6 | 442.2427 | 4 | -5.67 | 443.2464 | 1 | 7.79 | 2913 | 1.07e3 | 1 | 1 | 46 | 49 |  |  | DB Search |
| L.TVGV.A | N | 20.58 | 374.2165 | 4 | -4.06 | 375.2213 | 1 | 5.20 | 1970 | 6.27e3 | 1 | 1 | 397 | 400 |  |  | DB Search |
| V.TAY.I | N | 19.3 | 353.1587 | 3 | -1.32 | 354.1646 | 1 | 12.51 | 4913 | 1.39e3 | 1 | 1 | 107 | 109 |  |  | DB Search |
| T.VTAT.K | N | 19.15 | 390.2114 | 4 | -9 | 391.2142 | 1 | 7.51 | 2842 | 9.19e2 | 1 | 1 | 252 | 255 |  |  | DB Search |
| E.TVTA.T | N | 17.32 | 390.2114 | 4 | -8.37 | 391.2145 | 1 | 7.97 | 2951 | 0 | 0 | 0 | 251 | 254 |  |  | DB Search |
| E.TKC.Y | N | 16.14 | 350.1624 | 3 | 7.67 | 351.1715 | 1 | 4.93 | 1958 | 0 | 0 | 0 | 272 | 274 |  |  | DB Search |
| total 11 peptides |
| --- |

Best Unique PSM (Scan POS\_R15-1.wiff:8599, m/z=616.3643, z=1, RT=23.76, ppm=-3.42):


C4R317|C4R317\_KOMPG

back to list

  

| Protein Coverage
| Supporting Peptides
| Best Unique PSM
|

Protein Coverage:

Supporting Peptides:

| Peptide | Uniq | -10lgP | Mass | Length | ppm | m/z | z | RT | Scan | Area POS\_R15-1 | #Feature | #Feature POS\_R15-1 | Start | End | PTM | AScore | Found By |
| --- | --- | --- | --- | --- | --- | --- | --- | --- | --- | --- | --- | --- | --- | --- | --- | --- | --- |
| H.LFSP.A | N | 26.07 | 462.2478 | 4 | -0.35 | 463.2538 | 1 | 20.06 | 7534 | 6.05e2 | 1 | 1 | 493 | 496 |  |  | DB Search |
| P.TSLA.G | N | 24.8 | 390.2114 | 4 | -5.32 | 391.2157 | 1 | 6.25 | 2416 | 1.07e4 | 1 | 1 | 260 | 263 |  |  | DB Search |
| A.PVLV.P | N | 23.27 | 426.2842 | 4 | 4.41 | 427.2923 | 1 | 20.36 | 7664 | 0 | 0 | 0 | 122 | 125 |  |  | DB Search |
| K.LSGI.T | N | 22.6 | 388.2322 | 4 | -3.81 | 389.237 | 1 | 9.87 | 3804 | 6.08e3 | 1 | 1 | 227 | 230 |  |  | DB Search |
| L.VGSL.P | N | 21.91 | 374.2165 | 4 | 0.34 | 375.223 | 1 | 13.82 | 5444 | 2.07e3 | 1 | 1 | 446 | 449 |  |  | DB Search |
| H.Q(-17.03)PVTGSRQPP.H | Y | 20.91 | 1048.5301 | 10 | 4.05 | 1049.5391 | 1 | 20.46 | 7708 | 3.73e2 | 1 | 1 | 511 | 520 | Pyro-glu from Q | Q1:Pyro-glu from Q:1000 | DB Search |
| N.VSSL.T | N | 20.48 | 404.2271 | 4 | -7.08 | 405.2305 | 1 | 8.17 | 2993 | 7.47e3 | 1 | 1 | 31 | 34 |  |  | DB Search |
| A.PVLVP.H | N | 20.2 | 523.337 | 5 | -6.37 | 524.3396 | 1 | 27.22 | 9490 | 8.88e3 | 1 | 1 | 122 | 126 |  |  | DB Search |
| A.SLTP.F | N | 19.55 | 416.2271 | 4 | 2 | 417.2342 | 1 | 10.25 | 4029 | 3.69e3 | 1 | 1 | 53 | 56 |  |  | DB Search |
| P.VLVP.H | N | 18.9 | 426.2842 | 4 | -1.74 | 427.2897 | 1 | 17.76 | 6878 | 3.8e3 | 1 | 1 | 123 | 126 |  |  | DB Search |
| R.MTNL.S | N | 17.05 | 477.2257 | 4 | 5.76 | 478.2346 | 1 | 11.53 | 4573 | 7.69e2 | 1 | 1 | 310 | 313 |  |  | DB Search |
| P.VLPV.S | N | 17 | 426.2842 | 4 | -1.74 | 427.2897 | 1 | 17.76 | 6936 | 3.8e3 | 1 | 1 | 272 | 275 |  |  | DB Search |
| N.DLVT.S | N | 16.75 | 446.2376 | 4 | -4.95 | 447.2416 | 1 | 23.24 | 8484 | 0 | 0 | 0 | 646 | 649 |  |  | DB Search |
| total 13 peptides |
| --- |

Best Unique PSM (Scan POS\_R15-1.wiff:7708, m/z=1049.5304, z=1, RT=20.67, ppm=1.57):


C4R632|C4R632\_KOMPG

back to list

  

| Protein Coverage
| Supporting Peptides
| Best Unique PSM
|

Protein Coverage:

Supporting Peptides:

| Peptide | Uniq | -10lgP | Mass | Length | ppm | m/z | z | RT | Scan | Area POS\_R15-1 | #Feature | #Feature POS\_R15-1 | Start | End | PTM | AScore | Found By |
| --- | --- | --- | --- | --- | --- | --- | --- | --- | --- | --- | --- | --- | --- | --- | --- | --- | --- |
| F.GEGVI.Y | N | 20.32 | 473.2485 | 5 | -0.97 | 474.2542 | 1 | 13.69 | 5382 | 4.72e3 | 1 | 1 | 126 | 130 |  |  | DB Search |
| Q.PDYT.S | N | 17.57 | 494.2013 | 4 | -2.68 | 495.206 | 1 | 9.85 | 3843 | 4.44e3 | 1 | 1 | 492 | 495 |  |  | DB Search |
| W.VPWVG.S | Y | 15.7 | 556.3009 | 5 | 7.17 | 557.3108 | 1 | 9.10 | 3506 | 2.22e2 | 1 | 1 | 54 | 58 |  |  | DB Search |
| F.Q(-17.03)PH.R | N | 15.48 | 363.1543 | 3 | -0.14 | 364.1606 | 1 | 2.32 | 992 | 3.39e4 | 1 | 1 | 418 | 420 | Pyro-glu from Q | Q1:Pyro-glu from Q:1000 | DB Search |
| total 4 peptides |
| --- |

Best Unique PSM (Scan POS\_R15-1.wiff:3506, m/z=557.2961, z=1, RT=9.11, ppm=4.68):


C4QYT3|C4QYT3\_KOMPG

back to list

  

| Protein Coverage
| Supporting Peptides
| Best Unique PSM
|

Protein Coverage:

Supporting Peptides:

| Peptide | Uniq | -10lgP | Mass | Length | ppm | m/z | z | RT | Scan | Area POS\_R15-1 | #Feature | #Feature POS\_R15-1 | Start | End | PTM | AScore | Found By |
| --- | --- | --- | --- | --- | --- | --- | --- | --- | --- | --- | --- | --- | --- | --- | --- | --- | --- |
| D.NGL.G | N | 28.25 | 302.159 | 3 | -3.76 | 303.1644 | 1 | 6.06 | 2374 | 3.14e3 | 1 | 1 | 564 | 566 |  |  | DB Search |
| A.NGGL.I | N | 28.18 | 359.1805 | 4 | -1.08 | 360.1865 | 1 | 6.05 | 2378 | 0 | 0 | 0 | 1019 | 1022 |  |  | DB Search |
| A.VVGI.A | N | 26.8 | 386.2529 | 4 | -6 | 387.2569 | 1 | 17.69 | 6924 | 2.17e3 | 1 | 1 | 621 | 624 |  |  | DB Search |
| D.YSLF.Q | N | 26.31 | 528.2584 | 4 | -2.91 | 529.2628 | 1 | 34.97 | 11598 | 3.74e3 | 1 | 1 | 277 | 280 |  |  | DB Search |
| V.LSLE.P | N | 25.68 | 460.2533 | 4 | -2.19 | 461.2584 | 1 | 13.54 | 5303 | 1.35e4 | 1 | 1 | 576 | 579 |  |  | DB Search |
| G.LGSI.R | N | 25.26 | 388.2322 | 4 | -3.81 | 389.237 | 1 | 9.87 | 3785 | 6.08e3 | 1 | 1 | 460 | 463 |  |  | DB Search |
| G.LGGGA.A | N | 25.05 | 373.1961 | 5 | 1.54 | 374.203 | 1 | 3.00 | 1292 | 0 | 0 | 0 | 493 | 497 |  |  | DB Search |
| S.PFLTSE.L | Y | 24.41 | 692.3381 | 6 | -7.52 | 693.3384 | 1 | 20.10 | 7523 | 8.77e2 | 1 | 1 | 670 | 675 |  |  | DB Search |
| A.EW.T | N | 22.33 | 333.1325 | 2 | -3.27 | 334.1378 | 1 | 12.76 | 5036 | 2.08e4 | 1 | 1 | 267 | 268 |  |  | DB Search |
| V.ELF.M | N | 21.7 | 407.2056 | 3 | -8.99 | 408.2082 | 1 | 29.23 | 10157 | 9.42e2 | 1 | 1 | 247 | 249 |  |  | DB Search |
| M.EIW.C | N | 21.42 | 446.2165 | 3 | -5.13 | 447.2204 | 1 | 31.30 | 10711 | 1.59e3 | 1 | 1 | 586 | 588 |  |  | DB Search |
| H.PGE.G | N | 21.28 | 301.1274 | 3 | 1.88 | 302.1345 | 1 | 2.04 | 869 | 2.57e3 | 1 | 1 | 795 | 797 |  |  | DB Search |
| A.VTVL.H | N | 21.25 | 430.2791 | 4 | -6.35 | 431.2826 | 1 | 27.75 | 9755 | 1.56e3 | 1 | 1 | 917 | 920 |  |  | DB Search |
| P.KYT.D | N | 21.18 | 410.2165 | 3 | -4.93 | 411.2208 | 1 | 12.54 | 4954 | 3.83e2 | 1 | 1 | 187 | 189 |  |  | DB Search |
| I.VSIS.A | N | 20.57 | 404.2271 | 4 | -7.08 | 405.2305 | 1 | 8.17 | 2961 | 7.47e3 | 1 | 1 | 1014 | 1017 |  |  | DB Search |
| V.NSG.E | N | 20.42 | 276.107 | 3 | 6.48 | 277.1154 | 1 | 16.74 | 6592 | 0 | 0 | 0 | 502 | 504 |  |  | DB Search |
| Q.SAE.Q | N | 20.39 | 305.1223 | 3 | 3.83 | 306.13 | 1 | 8.24 | 3110 | 9.42e3 | 2 | 2 | 1051 | 1053 |  |  | DB Search |
| T.SLGD.T | N | 20.24 | 390.175 | 4 | -7.72 | 391.1783 | 1 | 3.30 | 1421 | 1.05e3 | 1 | 1 | 731 | 734 |  |  | DB Search |
| G.SSD.I | N | 20.1 | 307.1016 | 3 | 8.02 | 308.1105 | 1 | 10.67 | 4164 | 0 | 0 | 0 | 959 | 961 |  |  | DB Search |
| T.RPD.F | N | 19.79 | 386.1914 | 3 | -3.61 | 387.1963 | 1 | 2.95 | 1225 | 1.33e2 | 1 | 1 | 151 | 153 |  |  | DB Search |
| E.VTAPLSL.V | Y | 19.64 | 699.4167 | 7 | -9.2 | 700.4158 | 1 | 26.75 | 9472 | 8.73e2 | 1 | 1 | 838 | 844 |  |  | DB Search |
| E.PH.M | N | 19.52 | 252.1222 | 2 | -5.45 | 253.1275 | 1 | 5.57 | 2095 | 1.25e4 | 1 | 1 | 580 | 581 |  |  | DB Search |
| S.EAY.L | N | 19.24 | 381.1536 | 3 | -5.14 | 382.158 | 1 | 5.09 | 2057 | 2.63e3 | 1 | 1 | 309 | 311 |  |  | DB Search |
| E.ELG.A | N | 18.95 | 317.1587 | 3 | -4.31 | 318.1638 | 1 | 5.49 | 2177 | 4.11e3 | 1 | 1 | 970 | 972 |  |  | DB Search |
| L.IRV.L | N | 18.9 | 386.2641 | 3 | -1.28 | 387.27 | 1 | 27.77 | 9760 | 0 | 0 | 0 | 108 | 110 |  |  | DB Search |
| S.LDNF.V | N | 18.37 | 507.2329 | 4 | -5.34 | 508.2362 | 1 | 14.74 | 5819 | 0 | 0 | 0 | 1127 | 1130 |  |  | DB Search |
| K.TW.T | N | 18.09 | 305.1375 | 2 | -1.88 | 306.1435 | 1 | 9.22 | 3551 | 5.58e3 | 1 | 1 | 858 | 859 |  |  | DB Search |
| L.IYSS.S | N | 17.99 | 468.222 | 4 | 0.32 | 469.2283 | 1 | 16.06 | 6398 | 1.94e3 | 1 | 1 | 1023 | 1026 |  |  | DB Search |
| R.ITP.G | N | 17.99 | 329.1951 | 3 | 1.36 | 330.202 | 1 | 7.49 | 2815 | 1.47e3 | 1 | 1 | 475 | 477 |  |  | DB Search |
| G.LSGY.T | N | 17.88 | 438.2114 | 4 | -3.08 | 439.2163 | 1 | 7.95 | 2900 | 3.53e4 | 1 | 1 | 374 | 377 |  |  | DB Search |
| N.HP.T | N | 17.77 | 252.1222 | 2 | -2.49 | 253.1283 | 1 | 5.54 | 2216 | 1.25e4 | 1 | 1 | 340 | 341 |  |  | DB Search |
| V.LHAD.L | N | 17.73 | 454.2176 | 4 | 2.41 | 455.2248 | 1 | 17.29 | 6827 | 0 | 0 | 0 | 920 | 923 |  |  | DB Search |
| I.GDR.T | N | 17.49 | 346.1601 | 3 | -0.12 | 347.1664 | 1 | 10.73 | 4173 | 1.09e2 | 1 | 1 | 701 | 703 |  |  | DB Search |
| V.RGY.H | N | 17.46 | 394.1965 | 3 | 3.18 | 395.204 | 1 | 18.17 | 7080 | 0 | 0 | 0 | 448 | 450 |  |  | DB Search |
| S.ADL.D | N | 17.27 | 317.1587 | 3 | -4.79 | 318.1636 | 1 | 3.30 | 1336 | 3.11e3 | 1 | 1 | 508 | 510 |  |  | DB Search |
| L.EAVT.V | N | 17.24 | 418.2063 | 4 | -2.34 | 419.2116 | 1 | 7.30 | 2788 | 4.79e2 | 1 | 1 | 915 | 918 |  |  | DB Search |
| D.YSL.F | N | 17.18 | 381.19 | 3 | 2.91 | 382.1974 | 1 | 6.77 | 2598 | 3.84e2 | 1 | 1 | 277 | 279 |  |  | DB Search |
| E.WT.I | N | 17.04 | 305.1375 | 2 | -1.68 | 306.1436 | 1 | 9.15 | 3517 | 5.58e3 | 1 | 1 | 268 | 269 |  |  | DB Search |
| A.SPK.Y | N | 16.89 | 330.1903 | 3 | -8.46 | 331.194 | 1 | 5.91 | 2235 | 5.17e3 | 1 | 1 | 185 | 187 |  |  | DB Search |
| F.HKF.F | N | 16.83 | 430.2328 | 3 | 9.29 | 431.243 | 1 | 12.31 | 4825 | 5.31e3 | 1 | 1 | 1166 | 1168 |  |  | DB Search |
| A.ELLD.Y | N | 16.42 | 488.2482 | 4 | -0.37 | 489.2541 | 1 | 13.94 | 5546 | 0 | 0 | 0 | 63 | 66 |  |  | DB Search |
| L.E(-18.01)PH.M | N | 16.21 | 363.1543 | 3 | -0.12 | 364.1606 | 1 | 2.32 | 1021 | 3.39e4 | 1 | 1 | 579 | 581 | Pyro-glu from E | E1:Pyro-glu from E:1000 | DB Search |
| D.LSVE.E | N | 16.12 | 446.2376 | 4 | -1.26 | 447.2433 | 1 | 9.47 | 3656 | 0 | 0 | 0 | 73 | 76 |  |  | DB Search |
| G.EGS.A | N | 15.77 | 291.1066 | 3 | 7.44 | 292.1154 | 1 | 3.53 | 1538 | 0 | 0 | 0 | 505 | 507 |  |  | DB Search |
| E.LDIG.K | N | 15.65 | 416.2271 | 4 | -5.75 | 417.2309 | 1 | 13.92 | 5536 | 5.65e3 | 1 | 1 | 393 | 396 |  |  | DB Search |
| D.NGV.F | N | 15.37 | 288.1434 | 3 | 8 | 289.1522 | 1 | 5.91 | 2301 | 6.8e3 | 1 | 1 | 992 | 994 |  |  | DB Search |
| T.Q(-17.03)PL.Q | N | 15.26 | 339.1794 | 3 | -4.38 | 340.1844 | 1 | 15.14 | 5814 | 1.63e4 | 1 | 1 | 664 | 666 | Pyro-glu from Q | Q1:Pyro-glu from Q:1000 | DB Search |
| total 47 peptides |
| --- |

Best Unique PSM (Scan POS\_R15-1.wiff:7523, m/z=693.3384, z=1, RT=20.10, ppm=-10):


C4R0H5|C4R0H5\_KOMPG

back to list

  

| Protein Coverage
| Supporting Peptides
| Best Unique PSM
|

Protein Coverage:

Supporting Peptides:

| Peptide | Uniq | -10lgP | Mass | Length | ppm | m/z | z | RT | Scan | Area POS\_R15-1 | #Feature | #Feature POS\_R15-1 | Start | End | PTM | AScore | Found By |
| --- | --- | --- | --- | --- | --- | --- | --- | --- | --- | --- | --- | --- | --- | --- | --- | --- | --- |
| L.DVTL.K | N | 21.02 | 446.2376 | 4 | -4.47 | 447.2418 | 1 | 16.24 | 6386 | 3.07e3 | 1 | 1 | 130 | 133 |  |  | DB Search |
| L.RCL.P | N | 20.6 | 390.2049 | 3 | -4.41 | 391.2095 | 1 | 8.08 | 3003 | 0 | 0 | 0 | 374 | 376 |  |  | DB Search |
| K.TFR.P | N | 20.53 | 422.2278 | 3 | -9.2 | 423.2301 | 1 | 22.38 | 8244 | 2.98e1 | 1 | 1 | 472 | 474 |  |  | DB Search |
| L.DVLRCLPVD.I | Y | 20.34 | 1028.5324 | 9 | -3.8 | 515.2703 | 2 | 30.44 | 10463 | 9.03e2 | 1 | 1 | 371 | 379 |  |  | DB Search |
| E.NDT.S | N | 19.64 | 348.1281 | 3 | 7.11 | 349.137 | 1 | 7.31 | 2704 | 6.3e4 | 1 | 1 | 383 | 385 |  |  | DB Search |
| M.KGY.K | N | 19.27 | 366.1903 | 3 | -9.39 | 367.1932 | 1 | 6.66 | 2561 | 3.55e1 | 1 | 1 | 419 | 421 |  |  | DB Search |
| F.SNG.V | N | 19.07 | 276.107 | 3 | 1.61 | 277.114 | 1 | 16.82 | 6604 | 0 | 0 | 0 | 437 | 439 |  |  | DB Search |
| R.ISLP.Y | N | 18.29 | 428.2635 | 4 | -7.33 | 429.2665 | 1 | 24.15 | 8767 | 0 | 0 | 0 | 301 | 304 |  |  | DB Search |
| N.IEFP.F | N | 17.54 | 504.2584 | 4 | -6.47 | 505.2611 | 1 | 30.11 | 10334 | 3.53e2 | 2 | 2 | 558 | 561 |  |  | DB Search |
| D.TSN.H | N | 17.54 | 320.1332 | 3 | 3.34 | 321.1407 | 1 | 8.11 | 3024 | 0 | 0 | 0 | 385 | 387 |  |  | DB Search |
| Q.RLC.N | N | 16.16 | 390.2049 | 3 | 4.43 | 391.213 | 1 | 3.37 | 1421 | 1.01e3 | 1 | 1 | 168 | 170 |  |  | DB Search |
| I.EGS.F | N | 15.77 | 291.1066 | 3 | 7.44 | 292.1154 | 1 | 3.53 | 1538 | 0 | 0 | 0 | 646 | 648 |  |  | DB Search |
| L.KST.L | N | 15.47 | 334.1852 | 3 | 0.84 | 335.192 | 1 | 54.68 | 16215 | 3.66e2 | 1 | 1 | 431 | 433 |  |  | DB Search |
| S.NGV.F | N | 15.37 | 288.1434 | 3 | 8 | 289.1522 | 1 | 5.91 | 2301 | 6.8e3 | 1 | 1 | 438 | 440 |  |  | DB Search |
| total 14 peptides |
| --- |

Best Unique PSM (Scan POS\_R15-1.wiff:10463, m/z=515.2699, z=2, RT=30.46, ppm=-6.28):


C4R2I3|C4R2I3\_KOMPG

back to list

  

| Protein Coverage
| Supporting Peptides
| Best Unique PSM
|

Protein Coverage:

Supporting Peptides:

| Peptide | Uniq | -10lgP | Mass | Length | ppm | m/z | z | RT | Scan | Area POS\_R15-1 | #Feature | #Feature POS\_R15-1 | Start | End | PTM | AScore | Found By |
| --- | --- | --- | --- | --- | --- | --- | --- | --- | --- | --- | --- | --- | --- | --- | --- | --- | --- |
| L.AVGF.I | N | 17.44 | 392.2059 | 4 | -3.97 | 393.2107 | 1 | 15.98 | 6324 | 2.62e3 | 1 | 1 | 64 | 67 |  |  | DB Search |
| S.TAIP.G | N | 16.96 | 400.2322 | 4 | -5.15 | 401.2364 | 1 | 9.48 | 3664 | 0 | 0 | 0 | 3 | 6 |  |  | DB Search |
| M.STAI.P | N | 15.79 | 390.2114 | 4 | -5.32 | 391.2157 | 1 | 6.25 | 2317 | 1.07e4 | 1 | 1 | 2 | 5 |  |  | DB Search |
| A.GGSSST.M | Y | 15.76 | 494.1972 | 6 | 5.46 | 495.206 | 1 | 9.85 | 3816 | 4.44e3 | 1 | 1 | 36 | 41 |  |  | DB Search |
| total 4 peptides |
| --- |

Best Unique PSM (Scan POS\_R15-1.wiff:3816, m/z=495.206, z=1, RT=9.85, ppm=2.97):


C4R875|C4R875\_KOMPG

back to list

  

| Protein Coverage
| Supporting Peptides
| Best Unique PSM
|

Protein Coverage:

Supporting Peptides:

| Peptide | Uniq | -10lgP | Mass | Length | ppm | m/z | z | RT | Scan | Area POS\_R15-1 | #Feature | #Feature POS\_R15-1 | Start | End | PTM | AScore | Found By |
| --- | --- | --- | --- | --- | --- | --- | --- | --- | --- | --- | --- | --- | --- | --- | --- | --- | --- |
| D.TLVI.K | N | 26.01 | 444.2948 | 4 | -4 | 445.2992 | 1 | 28.31 | 9909 | 1.89e3 | 1 | 1 | 862 | 865 |  |  | DB Search |
| K.LLLP.L | N | 24.07 | 454.3155 | 4 | -2.4 | 455.3206 | 1 | 34.52 | 11565 | 2.64e3 | 1 | 1 | 217 | 220 |  |  | DB Search |
| P.SSLF.G | N | 21.63 | 452.2271 | 4 | -3.7 | 453.2316 | 1 | 22.49 | 8262 | 1.33e3 | 1 | 1 | 743 | 746 |  |  | DB Search |
| S.TSG.P | N | 21.44 | 263.1117 | 3 | 2.31 | 264.119 | 1 | 3.71 | 1537 | 5.72e3 | 1 | 1 | 136 | 138 |  |  | DB Search |
| L.DVTI.Q | N | 21.02 | 446.2376 | 4 | -4.47 | 447.2418 | 1 | 16.24 | 6386 | 3.07e3 | 1 | 1 | 465 | 468 |  |  | DB Search |
| T.VSLS.S | N | 20.57 | 404.2271 | 4 | -7.08 | 405.2305 | 1 | 8.17 | 2961 | 7.47e3 | 1 | 1 | 131 | 134 |  |  | DB Search |
| S.LSVP.T | N | 19.74 | 414.2478 | 4 | -0.09 | 415.254 | 1 | 17.14 | 6914 | 3.72e3 | 1 | 1 | 723 | 726 |  |  | DB Search |
| P.KGY.T | N | 19.27 | 366.1903 | 3 | -9.39 | 367.1932 | 1 | 6.66 | 2561 | 3.55e1 | 1 | 1 | 67 | 69 |  |  | DB Search |
| P.SNG.N | N | 19.07 | 276.107 | 3 | 1.61 | 277.114 | 1 | 16.82 | 6604 | 0 | 0 | 0 | 412 | 414 |  |  | DB Search |
| L.QSPL.M | N | 18.64 | 443.238 | 4 | -7.76 | 444.2407 | 1 | 10.47 | 4087 | 4.96e2 | 1 | 1 | 167 | 170 |  |  | DB Search |
| S.GSSI.S | N | 18.21 | 362.1801 | 4 | -9.58 | 363.183 | 1 | 6.30 | 2465 | 1.38e3 | 1 | 1 | 731 | 734 |  |  | DB Search |
| D.SFY.K | N | 18.1 | 415.1743 | 3 | 6.32 | 416.1832 | 1 | 8.32 | 3151 | 0 | 0 | 0 | 262 | 264 |  |  | DB Search |
| Q.SVTVSLSSTSGP.S | Y | 16.97 | 1120.5612 | 12 | 1.03 | 561.287 | 2 | 27.59 | 9711 | 0 | 0 | 0 | 128 | 139 |  |  | DB Search |
| E.SPK.S | N | 16.89 | 330.1903 | 3 | -8.46 | 331.194 | 1 | 5.91 | 2235 | 5.17e3 | 1 | 1 | 363 | 365 |  |  | DB Search |
| V.KST.V | N | 15.47 | 334.1852 | 3 | 0.84 | 335.192 | 1 | 54.68 | 16215 | 3.66e2 | 1 | 1 | 883 | 885 |  |  | DB Search |
| total 15 peptides |
| --- |

Best Unique PSM (Scan POS\_R15-1.wiff:9711, m/z=561.287, z=2, RT=27.59, ppm=-1.45):


C4QZ71|C4QZ71\_KOMPG

back to list

  

| Protein Coverage
| Supporting Peptides
| Best Unique PSM
|

Protein Coverage:

Supporting Peptides:

| Peptide | Uniq | -10lgP | Mass | Length | ppm | m/z | z | RT | Scan | Area POS\_R15-1 | #Feature | #Feature POS\_R15-1 | Start | End | PTM | AScore | Found By |
| --- | --- | --- | --- | --- | --- | --- | --- | --- | --- | --- | --- | --- | --- | --- | --- | --- | --- |
| I.NIL.A | N | 28.22 | 358.2216 | 3 | -4.68 | 359.2263 | 1 | 19.18 | 7326 | 4.19e3 | 1 | 1 | 646 | 648 |  |  | DB Search |
| P.TIPT.A | N | 26.11 | 430.2427 | 4 | -4.36 | 431.2471 | 1 | 9.16 | 3520 | 0 | 0 | 0 | 307 | 310 |  |  | DB Search |
| P.SPT.P | N | 23.16 | 303.143 | 3 | 6.39 | 304.1515 | 1 | 7.14 | 2731 | 5.4e3 | 2 | 2 | 441 | 443 |  |  | DB Search |
| M.EW.Y | N | 22.33 | 333.1325 | 2 | -3.27 | 334.1378 | 1 | 12.76 | 5036 | 2.08e4 | 1 | 1 | 416 | 417 |  |  | DB Search |
| V.LELSG.N | N | 22.23 | 517.2748 | 5 | -2.69 | 518.2794 | 1 | 11.88 | 4696 | 0 | 0 | 0 | 162 | 166 |  |  | DB Search |
| Q.TSG.E | N | 21.44 | 263.1117 | 3 | 2.31 | 264.119 | 1 | 3.71 | 1537 | 5.72e3 | 1 | 1 | 18 | 20 |  |  | DB Search |
| L.PSVP.P | N | 21.16 | 398.2165 | 4 | 4.76 | 399.2247 | 1 | 9.14 | 3512 | 0 | 0 | 0 | 280 | 283 |  |  | DB Search |
| K.DVTI.R | N | 21.02 | 446.2376 | 4 | -4.47 | 447.2418 | 1 | 16.24 | 6386 | 3.07e3 | 1 | 1 | 677 | 680 |  |  | DB Search |
| T.LSVS.T | N | 20.49 | 404.2271 | 4 | -7.08 | 405.2305 | 1 | 8.17 | 3035 | 7.47e3 | 1 | 1 | 729 | 732 |  |  | DB Search |
| N.GTIF.N | N | 20.4 | 436.2322 | 4 | -4.1 | 437.2366 | 1 | 23.18 | 8454 | 1.09e3 | 1 | 1 | 349 | 352 |  |  | DB Search |
| N.SSD.E | N | 20.1 | 307.1016 | 3 | 8.02 | 308.1105 | 1 | 10.67 | 4164 | 0 | 0 | 0 | 421 | 423 |  |  | DB Search |
| S.TPLT.A | N | 18.8 | 430.2427 | 4 | -8.61 | 431.2452 | 1 | 9.60 | 3908 | 8.62e3 | 1 | 1 | 733 | 736 |  |  | DB Search |
| Q.QVPSPTPTSLT.D | Y | 18.75 | 1126.587 | 11 | -1.4 | 564.2986 | 2 | 17.09 | 6721 | 0 | 0 | 0 | 438 | 448 |  |  | DB Search |
| T.HP.T | N | 17.77 | 252.1222 | 2 | -2.49 | 253.1283 | 1 | 5.54 | 2216 | 1.25e4 | 1 | 1 | 746 | 747 |  |  | DB Search |
| L.RSF.L | N | 17.5 | 408.2121 | 3 | -1.76 | 409.2177 | 1 | 9.17 | 3526 | 0 | 0 | 0 | 193 | 195 |  |  | DB Search |
| T.AGE.D | N | 17.5 | 275.1117 | 3 | 5.02 | 276.1197 | 1 | 9.57 | 3694 | 3.1e3 | 1 | 1 | 532 | 534 |  |  | DB Search |
| M.ADL.P | N | 17.27 | 317.1587 | 3 | -4.79 | 318.1636 | 1 | 3.30 | 1336 | 3.11e3 | 1 | 1 | 656 | 658 |  |  | DB Search |
| I.FNH.E | N | 17.22 | 416.1808 | 3 | -5.74 | 417.1847 | 1 | 6.08 | 2390 | 0 | 0 | 0 | 352 | 354 |  |  | DB Search |
| L.SPK.D | N | 16.89 | 330.1903 | 3 | -8.46 | 331.194 | 1 | 5.91 | 2235 | 5.17e3 | 1 | 1 | 321 | 323 |  |  | DB Search |
| S.AAQ.V | N | 16.83 | 288.1434 | 3 | 8 | 289.1522 | 1 | 5.91 | 2315 | 6.8e3 | 1 | 1 | 561 | 563 |  |  | DB Search |
| E.NHI.S | N | 15.79 | 382.1965 | 3 | -0.71 | 383.2025 | 1 | 19.27 | 7368 | 1.28e3 | 1 | 1 | 223 | 225 |  |  | DB Search |
| T.EAIE.M | N | 15.32 | 460.2169 | 4 | -0.24 | 461.2229 | 1 | 13.77 | 5437 | 0 | 0 | 0 | 58 | 61 |  |  | DB Search |
| K.PVE.Q | N | 15.19 | 343.1743 | 3 | -3.43 | 344.1796 | 1 | 12.76 | 5037 | 6.34e2 | 1 | 1 | 253 | 255 |  |  | DB Search |
| total 23 peptides |
| --- |

Best Unique PSM (Scan POS\_R15-1.wiff:6721, m/z=564.2986, z=2, RT=17.09, ppm=-3.88):


C4QXD7|C4QXD7\_KOMPG

back to list

  

| Protein Coverage
| Supporting Peptides
| Best Unique PSM
|

Protein Coverage:

Supporting Peptides:

| Peptide | Uniq | -10lgP | Mass | Length | ppm | m/z | z | RT | Scan | Area POS\_R15-1 | #Feature | #Feature POS\_R15-1 | Start | End | PTM | AScore | Found By |
| --- | --- | --- | --- | --- | --- | --- | --- | --- | --- | --- | --- | --- | --- | --- | --- | --- | --- |
| E.LGGL.Y | N | 27.34 | 358.2216 | 4 | -4.39 | 359.2264 | 1 | 15.45 | 6114 | 7.1e3 | 1 | 1 | 368 | 371 |  |  | DB Search |
| L.FLSLVP.F | N | 20.93 | 674.4003 | 6 | -6.34 | 675.4016 | 1 | 42.02 | 13315 | 6.45e2 | 1 | 1 | 11 | 16 |  |  | DB Search |
| Q.DVVY.A | N | 17.92 | 494.2376 | 4 | -7.07 | 495.2402 | 1 | 12.58 | 4967 | 1.85e3 | 1 | 1 | 165 | 168 |  |  | DB Search |
| H.LDFGP.L | N | 17.23 | 547.2642 | 5 | 0.18 | 548.2702 | 1 | 16.89 | 6622 | 0 | 0 | 0 | 71 | 75 |  |  | DB Search |
| V.LDYF.C | N | 16.91 | 556.2533 | 4 | -2.81 | 557.2576 | 1 | 23.71 | 8633 | 4.27e1 | 1 | 1 | 230 | 233 |  |  | DB Search |
| V.GPQQHW.L | Y | 16.17 | 751.3402 | 6 | -3.4 | 752.343 | 1 | 9.72 | 3807 | 8.53e2 | 1 | 1 | 338 | 343 |  |  | DB Search |
| A.YMR.M | N | 15.84 | 468.2155 | 3 | 0.49 | 469.2218 | 1 | 8.86 | 3455 | 2.39e3 | 1 | 1 | 327 | 329 |  |  | DB Search |
| G.PQ(+0.98)QHW.L | Y | 15.21 | 695.3027 | 5 | -4.62 | 696.3051 | 1 | 4.29 | 1720 | 0 | 0 | 0 | 339 | 343 | Deamidation (NQ) | Q2:Deamidation (NQ):27.96 | DB Search |
| total 8 peptides |
| --- |

Best Unique PSM (Scan POS\_R15-1.wiff:3807, m/z=752.343, z=1, RT=9.72, ppm=-5.88):


C4R567|C4R567\_KOMPG

back to list

  

| Protein Coverage
| Supporting Peptides
| Best Unique PSM
|

Protein Coverage:

Supporting Peptides:

| Peptide | Uniq | -10lgP | Mass | Length | ppm | m/z | z | RT | Scan | Area POS\_R15-1 | #Feature | #Feature POS\_R15-1 | Start | End | PTM | AScore | Found By |
| --- | --- | --- | --- | --- | --- | --- | --- | --- | --- | --- | --- | --- | --- | --- | --- | --- | --- |
| P.LTGL.N | N | 27.55 | 402.2478 | 4 | -1.76 | 403.2534 | 1 | 15.04 | 5964 | 1.56e4 | 2 | 2 | 459 | 462 |  |  | DB Search |
| D.IPTP.L | N | 25.97 | 426.2478 | 4 | -3.74 | 427.2524 | 1 | 12.64 | 4915 | 1.93e3 | 1 | 1 | 626 | 629 |  |  | DB Search |
| K.VIGS.F | N | 24.2 | 374.2165 | 4 | -4.06 | 375.2213 | 1 | 5.20 | 2012 | 6.27e3 | 1 | 1 | 558 | 561 |  |  | DB Search |
| I.TFR.K | N | 20.53 | 422.2278 | 3 | -9.2 | 423.2301 | 1 | 22.38 | 8244 | 2.98e1 | 1 | 1 | 737 | 739 |  |  | DB Search |
| T.AIVE.T | N | 20.45 | 430.2427 | 4 | -5.9 | 431.2464 | 1 | 12.49 | 4901 | 0 | 0 | 0 | 343 | 346 |  |  | DB Search |
| T.NSG.G | N | 20.42 | 276.107 | 3 | 6.48 | 277.1154 | 1 | 16.74 | 6592 | 0 | 0 | 0 | 976 | 978 |  |  | DB Search |
| L.KCA.N | N | 19.71 | 320.1518 | 3 | 2.66 | 321.1591 | 1 | 15.28 | 6053 | 3.59e2 | 1 | 1 | 86 | 88 |  |  | DB Search |
| T.SNG.E | N | 19.07 | 276.107 | 3 | 1.61 | 277.114 | 1 | 16.82 | 6604 | 0 | 0 | 0 | 43 | 45 |  |  | DB Search |
| E.TIGV.V | N | 18.91 | 388.2322 | 4 | -5.15 | 389.2365 | 1 | 17.34 | 6852 | 1.43e3 | 1 | 1 | 386 | 389 |  |  | DB Search |
| K.EQW.K | N | 18.42 | 461.191 | 3 | -3.23 | 462.1957 | 1 | 11.54 | 4588 | 2.45e3 | 1 | 1 | 632 | 634 |  |  | DB Search |
| L.SPSI.N | N | 18.22 | 402.2114 | 4 | -3.42 | 403.2163 | 1 | 12.42 | 4855 | 2.18e3 | 1 | 1 | 864 | 867 |  |  | DB Search |
| K.LDFS.P | N | 18.2 | 480.222 | 4 | -2.08 | 481.2271 | 1 | 16.69 | 6580 | 0 | 0 | 0 | 741 | 744 |  |  | DB Search |
| A.SFY.N | N | 18.1 | 415.1743 | 3 | 6.32 | 416.1832 | 1 | 8.32 | 3151 | 0 | 0 | 0 | 317 | 319 |  |  | DB Search |
| S.KEF.G | N | 17.73 | 422.2165 | 3 | 5.21 | 423.2249 | 1 | 10.75 | 4130 | 2.15e3 | 1 | 1 | 415 | 417 |  |  | DB Search |
| I.IEFP.L | N | 17.54 | 504.2584 | 4 | -6.47 | 505.2611 | 1 | 30.11 | 10334 | 3.53e2 | 2 | 2 | 270 | 273 |  |  | DB Search |
| I.TSN.G | N | 17.54 | 320.1332 | 3 | 3.34 | 321.1407 | 1 | 8.11 | 3024 | 0 | 0 | 0 | 42 | 44 |  |  | DB Search |
| T.ESW.F | N | 17.43 | 420.1645 | 3 | -0.16 | 421.1707 | 1 | 11.21 | 4460 | 0 | 0 | 0 | 170 | 172 |  |  | DB Search |
| S.IVEG.P | N | 17.38 | 416.2271 | 4 | -5.82 | 417.2309 | 1 | 4.62 | 1798 | 0 | 0 | 0 | 152 | 155 |  |  | DB Search |
| F.SSEI.R | N | 17.13 | 434.2013 | 4 | -0.44 | 435.2073 | 1 | 2.87 | 1206 | 7.24e2 | 1 | 1 | 206 | 209 |  |  | DB Search |
| F.SPK.V | N | 16.89 | 330.1903 | 3 | -8.46 | 331.194 | 1 | 5.91 | 2235 | 5.17e3 | 1 | 1 | 744 | 746 |  |  | DB Search |
| K.ELQSE.A | N | 16.66 | 604.2704 | 5 | 2.32 | 605.2776 | 1 | 9.98 | 3912 | 2.56e2 | 1 | 1 | 886 | 890 |  |  | DB Search |
| K.QKRQEQ.A | Y | 16.29 | 815.425 | 6 | 5.8 | 816.4349 | 1 | 16.77 | 6596 | 0 | 0 | 0 | 595 | 600 |  |  | DB Search |
| K.E(-18.01)PH.L | N | 16.21 | 363.1543 | 3 | -0.12 | 364.1606 | 1 | 2.32 | 1021 | 3.39e4 | 1 | 1 | 712 | 714 | Pyro-glu from E | E1:Pyro-glu from E:1000 | DB Search |
| E.QAVE.P | N | 15.8 | 445.2172 | 4 | 7.06 | 446.2266 | 1 | 2.45 | 1067 | 0 | 0 | 0 | 643 | 646 |  |  | DB Search |
| L.FRG.N | N | 15.51 | 378.2015 | 3 | -9.17 | 379.2044 | 1 | 3.37 | 1492 | 1.94e3 | 1 | 1 | 533 | 535 |  |  | DB Search |
| total 25 peptides |
| --- |

Best Unique PSM (Scan POS\_R15-1.wiff:6596, m/z=816.441, z=1, RT=16.81, ppm=3.31):


C4R7W9|C4R7W9\_KOMPG

back to list

  

| Protein Coverage
| Supporting Peptides
| Best Unique PSM
|

Protein Coverage:

Supporting Peptides:

| Peptide | Uniq | -10lgP | Mass | Length | ppm | m/z | z | RT | Scan | Area POS\_R15-1 | #Feature | #Feature POS\_R15-1 | Start | End | PTM | AScore | Found By |
| --- | --- | --- | --- | --- | --- | --- | --- | --- | --- | --- | --- | --- | --- | --- | --- | --- | --- |
| R.IGGL.K | N | 27.34 | 358.2216 | 4 | -4.39 | 359.2264 | 1 | 15.45 | 6114 | 7.1e3 | 1 | 1 | 1021 | 1024 |  |  | DB Search |
| K.FYR.F | N | 26.82 | 484.2434 | 3 | 1.64 | 485.2503 | 1 | 17.34 | 6854 | 0 | 0 | 0 | 612 | 614 |  |  | DB Search |
| S.VGGL.S | N | 24.87 | 344.2059 | 4 | -5.22 | 345.2106 | 1 | 10.74 | 4195 | 1.51e3 | 1 | 1 | 617 | 620 |  |  | DB Search |
| L.SPT.I | N | 23.16 | 303.143 | 3 | 6.39 | 304.1515 | 1 | 7.14 | 2731 | 5.4e3 | 2 | 2 | 1003 | 1005 |  |  | DB Search |
| Q.SAGLQ.D | N | 23.04 | 474.2438 | 5 | -3.9 | 475.248 | 1 | 3.16 | 1372 | 1.15e3 | 1 | 1 | 752 | 756 |  |  | DB Search |
| V.EW.Y | N | 22.33 | 333.1325 | 2 | -3.27 | 334.1378 | 1 | 12.76 | 5036 | 2.08e4 | 1 | 1 | 1066 | 1067 |  |  | DB Search |
| V.IEISG.Y | N | 22.23 | 517.2748 | 5 | -2.69 | 518.2794 | 1 | 11.88 | 4696 | 0 | 0 | 0 | 727 | 731 |  |  | DB Search |
| L.SLAL.D | N | 21.98 | 402.2478 | 4 | -2.21 | 403.2532 | 1 | 20.49 | 7711 | 0 | 0 | 0 | 995 | 998 |  |  | DB Search |
| D.ELF.P | N | 21.7 | 407.2056 | 3 | -8.99 | 408.2082 | 1 | 29.23 | 10157 | 9.42e2 | 1 | 1 | 255 | 257 |  |  | DB Search |
| S.AGVT.M | N | 21.65 | 346.1852 | 4 | -1.45 | 347.1911 | 1 | 2.64 | 1118 | 0 | 0 | 0 | 985 | 988 |  |  | DB Search |
| D.EIA.K | N | 21.09 | 331.1743 | 3 | -5.12 | 332.1791 | 1 | 6.98 | 2655 | 4.41e3 | 1 | 1 | 509 | 511 |  |  | DB Search |
| S.IAR.A | N | 20.74 | 358.2328 | 3 | -3.48 | 359.238 | 1 | 2.08 | 887 | 2.98e3 | 1 | 1 | 604 | 606 |  |  | DB Search |
| P.VPVE.W | N | 20.6 | 442.2427 | 4 | -5.67 | 443.2464 | 1 | 7.79 | 2913 | 1.07e3 | 1 | 1 | 1063 | 1066 |  |  | DB Search |
| Y.PH.K | N | 19.52 | 252.1222 | 2 | -5.45 | 253.1275 | 1 | 5.57 | 2095 | 1.25e4 | 1 | 1 | 247 | 248 |  |  | DB Search |
| D.KGY.P | N | 19.27 | 366.1903 | 3 | -9.39 | 367.1932 | 1 | 6.66 | 2561 | 3.55e1 | 1 | 1 | 190 | 192 |  |  | DB Search |
| K.EM.S | N | 19.13 | 278.0936 | 2 | 0.1 | 279.1003 | 1 | 3.52 | 1504 | 7.14e3 | 1 | 1 | 370 | 371 |  |  | DB Search |
| G.PR.L | N | 18.98 | 271.1644 | 2 | -8.93 | 272.1686 | 1 | 5.03 | 2023 | 0 | 0 | 0 | 30 | 31 |  |  | DB Search |
| H.EIG.V | N | 18.95 | 317.1587 | 3 | -4.31 | 318.1638 | 1 | 5.49 | 2177 | 4.11e3 | 1 | 1 | 218 | 220 |  |  | DB Search |
| G.Q(-17.03)SF.K | N | 18.71 | 363.143 | 3 | 2.8 | 364.1504 | 1 | 14.74 | 5815 | 0 | 0 | 0 | 23 | 25 | Pyro-glu from Q | Q1:Pyro-glu from Q:1000 | DB Search |
| E.FHH.T | N | 18.2 | 439.1968 | 3 | 7.99 | 440.2065 | 1 | 9.67 | 3787 | 2e3 | 1 | 1 | 53 | 55 |  |  | DB Search |
| L.EFIA.V | N | 18.15 | 478.2427 | 4 | -2.38 | 479.2477 | 1 | 20.03 | 7568 | 1.11e3 | 2 | 2 | 571 | 574 |  |  | DB Search |
| E.ITP.D | N | 17.99 | 329.1951 | 3 | 1.36 | 330.202 | 1 | 7.49 | 2815 | 1.47e3 | 1 | 1 | 870 | 872 |  |  | DB Search |
| P.ESF.K | N | 17.93 | 381.1536 | 3 | -4.82 | 382.1581 | 1 | 5.07 | 1981 | 2.63e3 | 1 | 1 | 864 | 866 |  |  | DB Search |
| V.YP.Q | N | 17.92 | 278.1266 | 2 | -4.14 | 279.1321 | 1 | 3.49 | 1522 | 2.89e2 | 1 | 1 | 152 | 153 |  |  | DB Search |
| E.ISGY.I | N | 17.88 | 438.2114 | 4 | -3.08 | 439.2163 | 1 | 7.95 | 2900 | 3.53e4 | 1 | 1 | 729 | 732 |  |  | DB Search |
| I.TSN.V | N | 17.54 | 320.1332 | 3 | 3.34 | 321.1407 | 1 | 8.11 | 3024 | 0 | 0 | 0 | 226 | 228 |  |  | DB Search |
| A.WT.S | N | 17.04 | 305.1375 | 2 | -1.68 | 306.1436 | 1 | 9.15 | 3517 | 5.58e3 | 1 | 1 | 903 | 904 |  |  | DB Search |
| L.ELLD.P | N | 16.42 | 488.2482 | 4 | -0.37 | 489.2541 | 1 | 13.94 | 5546 | 0 | 0 | 0 | 680 | 683 |  |  | DB Search |
| L.E(-18.01)PL.M | N | 16.1 | 339.1794 | 3 | -5.62 | 340.1839 | 1 | 16.05 | 6351 | 1.63e4 | 1 | 1 | 517 | 519 | Pyro-glu from E | E1:Pyro-glu from E:1000 | DB Search |
| R.IDEL.F | N | 16.03 | 488.2482 | 4 | -7.44 | 489.2506 | 1 | 15.57 | 6209 | 9.61e2 | 1 | 1 | 253 | 256 |  |  | DB Search |
| P.KDGP.S | N | 15.69 | 415.2067 | 4 | -1.18 | 416.2124 | 1 | 12.10 | 4891 | 1.77e3 | 1 | 1 | 980 | 983 |  |  | DB Search |
| L.Q(-17.03)PL.Q | N | 15.26 | 339.1794 | 3 | -4.38 | 340.1844 | 1 | 15.14 | 5814 | 1.63e4 | 1 | 1 | 45 | 47 | Pyro-glu from Q | Q1:Pyro-glu from Q:1000 | DB Search |
| V.PVE.W | N | 15.19 | 343.1743 | 3 | -3.43 | 344.1796 | 1 | 12.76 | 5037 | 6.34e2 | 1 | 1 | 1064 | 1066 |  |  | DB Search |
| S.LALDKSLSP.T | Y | 15.17 | 942.5386 | 9 | 2.92 | 472.2768 | 2 | 27.31 | 9630 | 0 | 0 | 0 | 996 | 1004 |  |  | DB Search |
| total 34 peptides |
| --- |

Best Unique PSM (Scan POS\_R15-1.wiff:9630, m/z=472.2762, z=1, RT=27.32, ppm=0.43):


C4R8A6|C4R8A6\_KOMPG

back to list

  

| Protein Coverage
| Supporting Peptides
| Best Unique PSM
|

Protein Coverage:

Supporting Peptides:

| Peptide | Uniq | -10lgP | Mass | Length | ppm | m/z | z | RT | Scan | Area POS\_R15-1 | #Feature | #Feature POS\_R15-1 | Start | End | PTM | AScore | Found By |
| --- | --- | --- | --- | --- | --- | --- | --- | --- | --- | --- | --- | --- | --- | --- | --- | --- | --- |
| V.VGSL.S | N | 21.91 | 374.2165 | 4 | 0.34 | 375.223 | 1 | 13.82 | 5444 | 2.07e3 | 1 | 1 | 150 | 153 |  |  | DB Search |
| I.ISVP.I | N | 19.74 | 414.2478 | 4 | -0.09 | 415.254 | 1 | 17.14 | 6914 | 3.72e3 | 1 | 1 | 191 | 194 |  |  | DB Search |
| L.STIA.L | N | 17.83 | 390.2114 | 4 | -5.32 | 391.2157 | 1 | 6.25 | 2360 | 1.07e4 | 1 | 1 | 217 | 220 |  |  | DB Search |
| K.KHF.G | N | 17.4 | 430.2328 | 3 | 0.78 | 431.2394 | 1 | 20.46 | 7703 | 1.69e2 | 1 | 1 | 6 | 8 |  |  | DB Search |
| I.INGF.G | N | 16.9 | 449.2274 | 4 | 0.62 | 450.2339 | 1 | 15.55 | 6174 | 0 | 0 | 0 | 119 | 122 |  |  | DB Search |
| L.GWGFYL.L | Y | 15.29 | 741.3486 | 6 | -7.19 | 742.3487 | 1 | 33.19 | 11234 | 1.83e2 | 1 | 1 | 434 | 439 |  |  | DB Search |
| total 6 peptides |
| --- |

Best Unique PSM (Scan POS\_R15-1.wiff:11234, m/z=742.3009, z=2, RT=33.28, ppm=-9.67):


C4R6A0|C4R6A0\_KOMPG

back to list

  

| Protein Coverage
| Supporting Peptides
| Best Unique PSM
|

Protein Coverage:

Supporting Peptides:

| Peptide | Uniq | -10lgP | Mass | Length | ppm | m/z | z | RT | Scan | Area POS\_R15-1 | #Feature | #Feature POS\_R15-1 | Start | End | PTM | AScore | Found By |
| --- | --- | --- | --- | --- | --- | --- | --- | --- | --- | --- | --- | --- | --- | --- | --- | --- | --- |
| K.SPIL.T | N | 26.66 | 428.2635 | 4 | -1.63 | 429.269 | 1 | 21.35 | 7905 | 0 | 0 | 0 | 616 | 619 |  |  | DB Search |
| Q.TLVL.N | N | 26.01 | 444.2948 | 4 | -4 | 445.2992 | 1 | 28.31 | 9909 | 1.89e3 | 1 | 1 | 1143 | 1146 |  |  | DB Search |
| V.VLGS.T | N | 24.2 | 374.2165 | 4 | -4.06 | 375.2213 | 1 | 5.20 | 2012 | 6.27e3 | 1 | 1 | 1466 | 1469 |  |  | DB Search |
| T.EIVR.R | N | 23.24 | 515.3067 | 4 | -4.7 | 516.3103 | 1 | 11.12 | 4401 | 1.13e4 | 1 | 1 | 936 | 939 |  |  | DB Search |
| D.RCF.A | N | 21.23 | 424.1893 | 3 | -9.2 | 425.1916 | 1 | 10.20 | 3945 | 1.22e3 | 1 | 1 | 815 | 817 |  |  | DB Search |
| D.GPALF.T | N | 20.53 | 503.2744 | 5 | -8.95 | 504.2759 | 1 | 28.75 | 10039 | 1.84e3 | 1 | 1 | 390 | 394 |  |  | DB Search |
| S.TLAV.A | N | 20.37 | 402.2478 | 4 | -1.76 | 403.2534 | 1 | 15.04 | 5856 | 1.07e4 | 1 | 1 | 958 | 961 |  |  | DB Search |
| R.KCHL.N | N | 19.04 | 499.2577 | 4 | -1.12 | 500.2632 | 1 | 26.46 | 9390 | 0 | 0 | 0 | 1195 | 1198 |  |  | DB Search |
| I.STLA.S | N | 17.83 | 390.2114 | 4 | -5.32 | 391.2157 | 1 | 6.25 | 2360 | 1.07e4 | 1 | 1 | 792 | 795 |  |  | DB Search |
| Q.WPR.V | N | 16.44 | 457.2437 | 3 | 4.14 | 458.2518 | 1 | 16.95 | 6653 | 0 | 0 | 0 | 1961 | 1963 |  |  | DB Search |
| S.RIC.Q | N | 16.16 | 390.2049 | 3 | 4.43 | 391.213 | 1 | 3.37 | 1421 | 1.01e3 | 1 | 1 | 661 | 663 |  |  | DB Search |
| H.VINF.F | N | 16.08 | 491.2744 | 4 | -1.92 | 492.2795 | 1 | 24.95 | 8989 | 2.33e2 | 1 | 1 | 1223 | 1226 |  |  | DB Search |
| Y.EALQ.R | N | 15.76 | 459.2329 | 4 | -3.37 | 460.2375 | 1 | 4.47 | 1741 | 3.16e3 | 2 | 2 | 1707 | 1710 |  |  | DB Search |
| G.GRIIVY.P | Y | 15.76 | 719.433 | 6 | -5.09 | 720.4348 | 1 | 16.14 | 6414 | 0 | 0 | 0 | 1394 | 1399 |  |  | DB Search |
| P.SEIA.F | N | 15.6 | 418.2063 | 4 | -9.49 | 419.2086 | 1 | 4.93 | 1962 | 0 | 0 | 0 | 1511 | 1514 |  |  | DB Search |
| Q.EPEY.T | N | 15.35 | 536.2118 | 4 | -3.41 | 537.2159 | 1 | 4.38 | 1742 | 8.35e2 | 1 | 1 | 4 | 7 |  |  | DB Search |
| total 16 peptides |
| --- |

Best Unique PSM (Scan POS\_R15-1.wiff:6414, m/z=720.4325, z=1, RT=16.60, ppm=-7.58):


C4QY05|C4QY05\_KOMPG

back to list

  

| Protein Coverage
| Supporting Peptides
| Best Unique PSM
|

Protein Coverage:

Supporting Peptides:

| Peptide | Uniq | -10lgP | Mass | Length | ppm | m/z | z | RT | Scan | Area POS\_R15-1 | #Feature | #Feature POS\_R15-1 | Start | End | PTM | AScore | Found By |
| --- | --- | --- | --- | --- | --- | --- | --- | --- | --- | --- | --- | --- | --- | --- | --- | --- | --- |
| G.VLLP.S | N | 22.17 | 440.2998 | 4 | -5.96 | 441.3034 | 1 | 30.99 | 10567 | 1.25e3 | 1 | 1 | 79 | 82 |  |  | DB Search |
| S.TSG.I | N | 21.44 | 263.1117 | 3 | 2.31 | 264.119 | 1 | 3.71 | 1537 | 5.72e3 | 1 | 1 | 71 | 73 |  |  | DB Search |
| S.LAR.S | N | 20.74 | 358.2328 | 3 | -3.48 | 359.238 | 1 | 2.08 | 887 | 2.98e3 | 1 | 1 | 87 | 89 |  |  | DB Search |
| S.VTGV.L | N | 20.36 | 374.2165 | 4 | -4.06 | 375.2213 | 1 | 5.20 | 1917 | 6.27e3 | 1 | 1 | 76 | 79 |  |  | DB Search |
| P.SSD.G | N | 20.1 | 307.1016 | 3 | 8.02 | 308.1105 | 1 | 10.67 | 4164 | 0 | 0 | 0 | 340 | 342 |  |  | DB Search |
| Q.FPS.P | N | 19.27 | 349.1638 | 3 | -4.81 | 350.1685 | 1 | 16.32 | 6461 | 1.41e3 | 1 | 1 | 8 | 10 |  |  | DB Search |
| R.DAVP.F | N | 18.95 | 400.1958 | 4 | -2.37 | 401.2011 | 1 | 8.27 | 3131 | 4.2e3 | 1 | 1 | 97 | 100 |  |  | DB Search |
| S.TW.T | N | 18.09 | 305.1375 | 2 | -1.88 | 306.1435 | 1 | 9.22 | 3551 | 5.58e3 | 1 | 1 | 105 | 106 |  |  | DB Search |
| S.IPVGP.G | Y | 17.97 | 481.29 | 5 | -3.31 | 482.2945 | 1 | 17.05 | 6621 | 1.08e3 | 1 | 1 | 245 | 249 |  |  | DB Search |
| E.YP.L | N | 17.92 | 278.1266 | 2 | -4.14 | 279.1321 | 1 | 3.49 | 1522 | 2.89e2 | 1 | 1 | 146 | 147 |  |  | DB Search |
| T.HP.N | N | 17.77 | 252.1222 | 2 | -2.49 | 253.1283 | 1 | 5.54 | 2216 | 1.25e4 | 1 | 1 | 136 | 137 |  |  | DB Search |
| L.TCR.K | N | 17.37 | 378.1685 | 3 | 5.06 | 379.1768 | 1 | 12.47 | 4890 | 0 | 0 | 0 | 297 | 299 |  |  | DB Search |
| G.YSL.A | N | 17.18 | 381.19 | 3 | 2.91 | 382.1974 | 1 | 6.77 | 2598 | 3.84e2 | 1 | 1 | 265 | 267 |  |  | DB Search |
| T.WT.D | N | 17.04 | 305.1375 | 2 | -1.68 | 306.1436 | 1 | 9.15 | 3517 | 5.58e3 | 1 | 1 | 106 | 107 |  |  | DB Search |
| D.KST.P | N | 15.47 | 334.1852 | 3 | 0.84 | 335.192 | 1 | 54.68 | 16215 | 3.66e2 | 1 | 1 | 109 | 111 |  |  | DB Search |
| D.Q(-17.03)PI.Y | N | 15.26 | 339.1794 | 3 | -4.38 | 340.1844 | 1 | 15.14 | 5814 | 1.63e4 | 1 | 1 | 167 | 169 | Pyro-glu from Q | Q1:Pyro-glu from Q:1000 | DB Search |
| total 16 peptides |
| --- |

Best Unique PSM (Scan POS\_R15-1.wiff:6621, m/z=482.2945, z=1, RT=17.05, ppm=-5.8):


C4QYZ9|C4QYZ9\_KOMPG

back to list

  

| Protein Coverage
| Supporting Peptides
| Best Unique PSM
|

Protein Coverage:

Supporting Peptides:

| Peptide | Uniq | -10lgP | Mass | Length | ppm | m/z | z | RT | Scan | Area POS\_R15-1 | #Feature | #Feature POS\_R15-1 | Start | End | PTM | AScore | Found By |
| --- | --- | --- | --- | --- | --- | --- | --- | --- | --- | --- | --- | --- | --- | --- | --- | --- | --- |
| K.DVTL.T | N | 21.02 | 446.2376 | 4 | -4.47 | 447.2418 | 1 | 16.24 | 6386 | 3.07e3 | 1 | 1 | 45 | 48 |  |  | DB Search |
| T.KPQAPIRGP.R | Y | 16.76 | 962.5661 | 9 | -0.35 | 482.289 | 2 | 16.89 | 6621 | 0 | 0 | 0 | 89 | 97 |  |  | DB Search |
| total 2 peptides |
| --- |

Best Unique PSM (Scan POS\_R15-1.wiff:6621, m/z=482.2945, z=1, RT=17.05, ppm=-2.83):


C4QWG1|C4QWG1\_KOMPG

back to list

  

| Protein Coverage
| Supporting Peptides
| Best Unique PSM
|

Protein Coverage:

Supporting Peptides:

| Peptide | Uniq | -10lgP | Mass | Length | ppm | m/z | z | RT | Scan | Area POS\_R15-1 | #Feature | #Feature POS\_R15-1 | Start | End | PTM | AScore | Found By |
| --- | --- | --- | --- | --- | --- | --- | --- | --- | --- | --- | --- | --- | --- | --- | --- | --- | --- |
| Q.LPQQP.P | N | 25.19 | 581.3173 | 5 | -2.43 | 582.3217 | 1 | 8.89 | 3457 | 0 | 0 | 0 | 218 | 222 |  |  | DB Search |
| M.VSSL.K | N | 20.48 | 404.2271 | 4 | -7.08 | 405.2305 | 1 | 8.17 | 2993 | 7.47e3 | 1 | 1 | 61 | 64 |  |  | DB Search |
| Q.QLPQQP.P | Y | 19.58 | 709.3759 | 6 | -7.55 | 710.376 | 1 | 8.73 | 3397 | 1.32e3 | 1 | 1 | 217 | 222 |  |  | DB Search |
| N.Q(-17.03)PIL.L | N | 17.35 | 452.2635 | 4 | -6.01 | 453.2669 | 1 | 32.30 | 10953 | 1.74e3 | 1 | 1 | 259 | 262 | Pyro-glu from Q | Q1:Pyro-glu from Q:1000 | DB Search |
| K.VLPV.V | N | 17 | 426.2842 | 4 | -1.74 | 427.2897 | 1 | 17.76 | 6936 | 3.8e3 | 1 | 1 | 104 | 107 |  |  | DB Search |
| Q.QQLP.Q | N | 15.07 | 484.2645 | 4 | -3.24 | 485.269 | 1 | 10.16 | 3975 | 0 | 0 | 0 | 216 | 219 |  |  | DB Search |
| total 6 peptides |
| --- |

Best Unique PSM (Scan POS\_R15-1.wiff:3397, m/z=710.3774, z=2, RT=8.76, ppm=-10.03):


C4R0N9|C4R0N9\_KOMPG

back to list

  

| Protein Coverage
| Supporting Peptides
| Best Unique PSM
|

Protein Coverage:

Supporting Peptides:

| Peptide | Uniq | -10lgP | Mass | Length | ppm | m/z | z | RT | Scan | Area POS\_R15-1 | #Feature | #Feature POS\_R15-1 | Start | End | PTM | AScore | Found By |
| --- | --- | --- | --- | --- | --- | --- | --- | --- | --- | --- | --- | --- | --- | --- | --- | --- | --- |
| L.PSIL.M | N | 29.95 | 428.2635 | 4 | -0.56 | 429.2694 | 1 | 21.28 | 7884 | 0 | 0 | 0 | 611 | 614 |  |  | DB Search |
| Y.LSVGVVLQ.G | Y | 26.53 | 813.496 | 8 | -4.82 | 814.4973 | 1 | 28.73 | 10013 | 3.04e3 | 1 | 1 | 438 | 445 |  |  | DB Search |
| L.SVGV.V | N | 26.46 | 360.2009 | 4 | -6.45 | 361.2049 | 1 | 8.68 | 3331 | 1.41e4 | 1 | 1 | 439 | 442 |  |  | DB Search |
| N.ELIR.E | N | 25.05 | 529.3224 | 4 | -4.56 | 530.3259 | 1 | 17.34 | 6855 | 6.68e3 | 1 | 1 | 56 | 59 |  |  | DB Search |
| A.HFT.V | N | 23.64 | 403.1856 | 3 | -0.83 | 404.1915 | 1 | 13.94 | 5543 | 0 | 0 | 0 | 506 | 508 |  |  | DB Search |
| G.GVSL.L | N | 22.74 | 374.2165 | 4 | -2.51 | 375.2219 | 1 | 13.91 | 5529 | 2.07e3 | 1 | 1 | 566 | 569 |  |  | DB Search |
| R.EIF.D | N | 21.7 | 407.2056 | 3 | -8.99 | 408.2082 | 1 | 29.23 | 10157 | 9.42e2 | 1 | 1 | 60 | 62 |  |  | DB Search |
| H.THH.N | N | 21.55 | 393.1761 | 3 | -4.54 | 394.1806 | 1 | 2.11 | 908 | 2.12e3 | 1 | 1 | 52 | 54 |  |  | DB Search |
| K.PGE.A | N | 21.28 | 301.1274 | 3 | 1.88 | 302.1345 | 1 | 2.04 | 869 | 2.57e3 | 1 | 1 | 466 | 468 |  |  | DB Search |
| R.KFH.V | N | 20.21 | 430.2328 | 3 | 9.29 | 431.243 | 1 | 12.31 | 5009 | 5.31e3 | 1 | 1 | 476 | 478 |  |  | DB Search |
| A.NDT.R | N | 19.64 | 348.1281 | 3 | 7.11 | 349.137 | 1 | 7.31 | 2704 | 6.3e4 | 1 | 1 | 620 | 622 |  |  | DB Search |
| I.PH.T | N | 19.52 | 252.1222 | 2 | -5.45 | 253.1275 | 1 | 5.57 | 2095 | 1.25e4 | 1 | 1 | 77 | 78 |  |  | DB Search |
| F.IRV.A | N | 18.9 | 386.2641 | 3 | -1.28 | 387.27 | 1 | 27.77 | 9760 | 0 | 0 | 0 | 161 | 163 |  |  | DB Search |
| Q.KHT.H | N | 18.78 | 384.2121 | 3 | -3.61 | 385.217 | 1 | 6.37 | 2316 | 2.15e3 | 1 | 1 | 50 | 52 |  |  | DB Search |
| A.HP.D | N | 17.77 | 252.1222 | 2 | -2.49 | 253.1283 | 1 | 5.54 | 2216 | 1.25e4 | 1 | 1 | 165 | 166 |  |  | DB Search |
| W.KEF.N | N | 17.73 | 422.2165 | 3 | 5.21 | 423.2249 | 1 | 10.75 | 4130 | 2.15e3 | 1 | 1 | 69 | 71 |  |  | DB Search |
| L.LLTD.F | N | 17.52 | 460.2533 | 4 | -9.09 | 461.2552 | 1 | 10.16 | 3965 | 9.46e2 | 1 | 1 | 222 | 225 |  |  | DB Search |
| E.AAQ.K | N | 16.83 | 288.1434 | 3 | 8 | 289.1522 | 1 | 5.91 | 2315 | 6.8e3 | 1 | 1 | 173 | 175 |  |  | DB Search |
| S.AAKN.L | N | 16.54 | 402.2227 | 4 | -0.92 | 403.2286 | 1 | 56.57 | 16912 | 1.95e3 | 1 | 1 | 120 | 123 |  |  | DB Search |
| N.E(-18.01)PL.P | N | 16.1 | 339.1794 | 3 | -5.62 | 340.1839 | 1 | 16.05 | 6351 | 1.63e4 | 1 | 1 | 636 | 638 | Pyro-glu from E | E1:Pyro-glu from E:1000 | DB Search |
| R.TFT.T | N | 15.92 | 367.1743 | 3 | -7.47 | 368.1779 | 1 | 8.54 | 3263 | 0 | 0 | 0 | 20 | 22 |  |  | DB Search |
| V.EGS.I | N | 15.77 | 291.1066 | 3 | 7.44 | 292.1154 | 1 | 3.53 | 1538 | 0 | 0 | 0 | 93 | 95 |  |  | DB Search |
| H.NGV.R | N | 15.37 | 288.1434 | 3 | 8 | 289.1522 | 1 | 5.91 | 2301 | 6.8e3 | 1 | 1 | 472 | 474 |  |  | DB Search |
| I.Q(-17.03)PL.V | N | 15.26 | 339.1794 | 3 | -4.38 | 340.1844 | 1 | 15.14 | 5814 | 1.63e4 | 1 | 1 | 32 | 34 | Pyro-glu from Q | Q1:Pyro-glu from Q:1000 | DB Search |
| total 24 peptides |
| --- |

Best Unique PSM (Scan POS\_R15-1.wiff:10013, m/z=814.4963, z=1, RT=28.75, ppm=-7.3):


C4QXW1|C4QXW1\_KOMPG

back to list

  

| Protein Coverage
| Supporting Peptides
| Best Unique PSM
|

Protein Coverage:

Supporting Peptides:

| Peptide | Uniq | -10lgP | Mass | Length | ppm | m/z | z | RT | Scan | Area POS\_R15-1 | #Feature | #Feature POS\_R15-1 | Start | End | PTM | AScore | Found By |
| --- | --- | --- | --- | --- | --- | --- | --- | --- | --- | --- | --- | --- | --- | --- | --- | --- | --- |
| R.LGGI.P | N | 27.34 | 358.2216 | 4 | -4.39 | 359.2264 | 1 | 15.45 | 6114 | 7.1e3 | 1 | 1 | 1864 | 1867 |  |  | DB Search |
| E.VAAP.S | N | 23.43 | 356.2059 | 4 | -1.11 | 357.2119 | 1 | 7.38 | 2801 | 2.39e3 | 1 | 1 | 25 | 28 |  |  | DB Search |
| N.VFADLITQ(+0.98).Y | Y | 22.9 | 906.4698 | 8 | -3.43 | 907.4717 | 1 | 25.12 | 9035 | 2.73e2 | 1 | 1 | 889 | 896 | Deamidation (NQ) | Q8:Deamidation (NQ):1000 | DB Search |
| E.DVTI.S | N | 21.02 | 446.2376 | 4 | -4.47 | 447.2418 | 1 | 16.24 | 6386 | 3.07e3 | 1 | 1 | 1327 | 1330 |  |  | DB Search |
| D.TFR.Q | N | 20.53 | 422.2278 | 3 | -9.2 | 423.2301 | 1 | 22.38 | 8244 | 2.98e1 | 1 | 1 | 326 | 328 |  |  | DB Search |
| R.SLGD.K | N | 20.24 | 390.175 | 4 | -7.72 | 391.1783 | 1 | 3.30 | 1421 | 1.05e3 | 1 | 1 | 167 | 170 |  |  | DB Search |
| F.RSSS.S | N | 18.8 | 435.2078 | 4 | -3.98 | 436.2122 | 1 | 11.38 | 4548 | 7.31e2 | 1 | 1 | 466 | 469 |  |  | DB Search |
| R.SVGI.G | N | 18.49 | 374.2165 | 4 | -4.96 | 375.221 | 1 | 14.62 | 5748 | 1.93e2 | 1 | 1 | 1710 | 1713 |  |  | DB Search |
| K.IGQP.Y | N | 17.62 | 413.2274 | 4 | -2.94 | 414.2325 | 1 | 10.81 | 4261 | 5.46e2 | 1 | 1 | 691 | 694 |  |  | DB Search |
| V.TCR.S | N | 17.37 | 378.1685 | 3 | 5.06 | 379.1768 | 1 | 12.47 | 4890 | 0 | 0 | 0 | 1707 | 1709 |  |  | DB Search |
| G.Q(-17.03)PII.L | N | 17.35 | 452.2635 | 4 | -6.01 | 453.2669 | 1 | 32.30 | 10953 | 1.74e3 | 1 | 1 | 1730 | 1733 | Pyro-glu from Q | Q1:Pyro-glu from Q:1000 | DB Search |
| L.E(-18.01)LNP.R | N | 16.86 | 453.2223 | 4 | -6.06 | 454.2257 | 1 | 14.47 | 5683 | 1.68e4 | 1 | 1 | 364 | 367 | Pyro-glu from E | E1:Pyro-glu from E:1000 | DB Search |
| S.ISAL.H | N | 16.63 | 402.2478 | 4 | -8.97 | 403.2505 | 1 | 9.42 | 3655 | 1.65e3 | 1 | 1 | 814 | 817 |  |  | DB Search |
| A.ELRGGSWVVVDP.T | Y | 16.45 | 1312.6775 | 12 | -8.15 | 657.3391 | 2 | 30.19 | 10387 | 0 | 0 | 0 | 1974 | 1985 |  |  | DB Search |
| Y.AEVE.V | N | 16.37 | 446.2013 | 4 | -1.36 | 447.2068 | 1 | 4.90 | 1940 | 0 | 0 | 0 | 696 | 699 |  |  | DB Search |
| V.EYLY.S | N | 16.17 | 586.2639 | 4 | 4.94 | 587.2726 | 1 | 16.20 | 6451 | 2.9e2 | 1 | 1 | 350 | 353 |  |  | DB Search |
| I.VEIA.E | N | 15.81 | 430.2427 | 4 | -4.19 | 431.2471 | 1 | 15.22 | 6032 | 0 | 0 | 0 | 120 | 123 |  |  | DB Search |
| E.APVT.I | N | 15.47 | 386.2165 | 4 | -4.26 | 387.2212 | 1 | 3.33 | 1445 | 2.99e3 | 1 | 1 | 317 | 320 |  |  | DB Search |
| V.SGYV.V | N | 15.39 | 424.1958 | 4 | 2.7 | 425.2032 | 1 | 2.77 | 1161 | 4e2 | 1 | 1 | 1425 | 1428 |  |  | DB Search |
| R.AYSV.R | N | 15.26 | 438.2114 | 4 | -3.08 | 439.2163 | 1 | 7.95 | 2920 | 3.53e4 | 1 | 1 | 1075 | 1078 |  |  | DB Search |
| N.PDPL.F | N | 15.18 | 440.2271 | 4 | 0.52 | 441.2335 | 1 | 16.91 | 6630 | 0 | 0 | 0 | 860 | 863 |  |  | DB Search |
| total 21 peptides |
| --- |

Best Unique PSM (Scan POS\_R15-1.wiff:9035, m/z=907.4739, z=1, RT=25.14, ppm=-5.91):


C4QX93|C4QX93\_KOMPG

back to list

  

| Protein Coverage
| Supporting Peptides
| Best Unique PSM
|

Protein Coverage:

Supporting Peptides:

| Peptide | Uniq | -10lgP | Mass | Length | ppm | m/z | z | RT | Scan | Area POS\_R15-1 | #Feature | #Feature POS\_R15-1 | Start | End | PTM | AScore | Found By |
| --- | --- | --- | --- | --- | --- | --- | --- | --- | --- | --- | --- | --- | --- | --- | --- | --- | --- |
| K.TSLA.H | N | 24.8 | 390.2114 | 4 | -5.32 | 391.2157 | 1 | 6.25 | 2416 | 1.07e4 | 1 | 1 | 632 | 635 |  |  | DB Search |
| N.LLLP.S | N | 24.07 | 454.3155 | 4 | -2.4 | 455.3206 | 1 | 34.52 | 11565 | 2.64e3 | 1 | 1 | 116 | 119 |  |  | DB Search |
| G.TVGV.Y | N | 20.58 | 374.2165 | 4 | -4.06 | 375.2213 | 1 | 5.20 | 1970 | 6.27e3 | 1 | 1 | 779 | 782 |  |  | DB Search |
| T.SITP.F | N | 19.55 | 416.2271 | 4 | 2 | 417.2342 | 1 | 10.25 | 4029 | 3.69e3 | 1 | 1 | 798 | 801 |  |  | DB Search |
| G.PAFV.S | N | 19.17 | 432.2372 | 4 | -3.2 | 433.2421 | 1 | 24.76 | 8853 | 3.98e2 | 1 | 1 | 303 | 306 |  |  | DB Search |
| N.LIQP.K | N | 19.13 | 469.29 | 4 | -8.42 | 470.2922 | 1 | 12.16 | 4756 | 6.43e3 | 1 | 1 | 599 | 602 |  |  | DB Search |
| I.NSAGVPH.C | Y | 19.04 | 680.3242 | 7 | 4.78 | 681.333 | 1 | 20.19 | 7540 | 2.47e2 | 1 | 1 | 97 | 103 |  |  | DB Search |
| D.ESPL.P | N | 18.2 | 444.222 | 4 | -3.28 | 445.2267 | 1 | 12.52 | 4847 | 1.87e3 | 1 | 1 | 283 | 286 |  |  | DB Search |
| F.IYSS.Q | N | 17.99 | 468.222 | 4 | 0.32 | 469.2283 | 1 | 16.06 | 6398 | 1.94e3 | 1 | 1 | 371 | 374 |  |  | DB Search |
| T.TCR.H | N | 17.37 | 378.1685 | 3 | 5.06 | 379.1768 | 1 | 12.47 | 4890 | 0 | 0 | 0 | 470 | 472 |  |  | DB Search |
| G.LGGV.E | N | 15.94 | 344.2059 | 4 | 0.19 | 345.2124 | 1 | 5.48 | 2143 | 3.05e3 | 1 | 1 | 275 | 278 |  |  | DB Search |
| E.FRG.T | N | 15.51 | 378.2015 | 3 | -9.17 | 379.2044 | 1 | 3.37 | 1492 | 1.94e3 | 1 | 1 | 403 | 405 |  |  | DB Search |
| total 12 peptides |
| --- |

Best Unique PSM (Scan POS\_R15-1.wiff:7540, m/z=681.333, z=1, RT=20.19, ppm=2.3):


C4R1F5|C4R1F5\_KOMPG

back to list

  

| Protein Coverage
| Supporting Peptides
| Best Unique PSM
|

Protein Coverage:

Supporting Peptides:

| Peptide | Uniq | -10lgP | Mass | Length | ppm | m/z | z | RT | Scan | Area POS\_R15-1 | #Feature | #Feature POS\_R15-1 | Start | End | PTM | AScore | Found By |
| --- | --- | --- | --- | --- | --- | --- | --- | --- | --- | --- | --- | --- | --- | --- | --- | --- | --- |
| V.ALELSG.G | Y | 28.74 | 588.3119 | 6 | -2.07 | 589.3165 | 1 | 14.07 | 5590 | 1.66e4 | 1 | 1 | 20 | 25 |  |  | DB Search |
| A.YHH.E | N | 24.3 | 455.1917 | 3 | -4.98 | 456.1956 | 1 | 6.94 | 2666 | 0 | 0 | 0 | 710 | 712 |  |  | DB Search |
| A.LELSG.G | N | 22.23 | 517.2748 | 5 | -2.69 | 518.2794 | 1 | 11.88 | 4696 | 0 | 0 | 0 | 21 | 25 |  |  | DB Search |
| R.SLGD.I | N | 20.24 | 390.175 | 4 | -7.72 | 391.1783 | 1 | 3.30 | 1421 | 1.05e3 | 1 | 1 | 598 | 601 |  |  | DB Search |
| F.GSPE.H | N | 16.5 | 388.1594 | 4 | -0.08 | 389.1657 | 1 | 19.06 | 7294 | 2.88e3 | 1 | 1 | 105 | 108 |  |  | DB Search |
| total 5 peptides |
| --- |

Best Unique PSM (Scan POS\_R15-1.wiff:5590, m/z=589.3126, z=1, RT=14.12, ppm=-4.55):


C4R3X1|C4R3X1\_KOMPG

back to list

  

| Protein Coverage
| Supporting Peptides
| Best Unique PSM
|

Protein Coverage:

Supporting Peptides:

| Peptide | Uniq | -10lgP | Mass | Length | ppm | m/z | z | RT | Scan | Area POS\_R15-1 | #Feature | #Feature POS\_R15-1 | Start | End | PTM | AScore | Found By |
| --- | --- | --- | --- | --- | --- | --- | --- | --- | --- | --- | --- | --- | --- | --- | --- | --- | --- |
| S.SPIL.N | N | 26.66 | 428.2635 | 4 | -1.63 | 429.269 | 1 | 21.35 | 7905 | 0 | 0 | 0 | 114 | 117 |  |  | DB Search |
| L.VKEVGGGA.Q | Y | 22.37 | 715.3864 | 8 | -7.18 | 716.3868 | 1 | 8.78 | 3406 | 0 | 0 | 0 | 534 | 541 |  |  | DB Search |
| N.WAN.E | N | 21.03 | 389.1699 | 3 | 5.94 | 390.1785 | 1 | 10.28 | 4047 | 1.16e3 | 1 | 1 | 320 | 322 |  |  | DB Search |
| L.FRC.T | N | 17.7 | 424.1893 | 3 | -9.2 | 425.1916 | 1 | 10.20 | 3929 | 1.22e3 | 1 | 1 | 259 | 261 |  |  | DB Search |
| S.TLNG.R | N | 15.96 | 403.2067 | 4 | -9.11 | 404.2093 | 1 | 5.03 | 2025 | 0 | 0 | 0 | 52 | 55 |  |  | DB Search |
| total 5 peptides |
| --- |

Best Unique PSM (Scan POS\_R15-1.wiff:3406, m/z=716.3868, z=1, RT=8.78, ppm=-9.67):


C4QWS1|C4QWS1\_KOMPG

back to list

  

| Protein Coverage
| Supporting Peptides
| Best Unique PSM
|

Protein Coverage:

Supporting Peptides:

| Peptide | Uniq | -10lgP | Mass | Length | ppm | m/z | z | RT | Scan | Area POS\_R15-1 | #Feature | #Feature POS\_R15-1 | Start | End | PTM | AScore | Found By |
| --- | --- | --- | --- | --- | --- | --- | --- | --- | --- | --- | --- | --- | --- | --- | --- | --- | --- |
| A.TLVI.V | N | 26.01 | 444.2948 | 4 | -4 | 445.2992 | 1 | 28.31 | 9909 | 1.89e3 | 1 | 1 | 429 | 432 |  |  | DB Search |
| T.ASAI.H | N | 22.98 | 360.2009 | 4 | -6.45 | 361.2049 | 1 | 8.68 | 3069 | 1.41e4 | 1 | 1 | 749 | 752 |  |  | DB Search |
| A.SSIF.A | N | 21.63 | 452.2271 | 4 | -3.7 | 453.2316 | 1 | 22.49 | 8262 | 1.33e3 | 1 | 1 | 483 | 486 |  |  | DB Search |
| W.VISG.T | N | 21.25 | 374.2165 | 4 | -4.06 | 375.2213 | 1 | 5.20 | 1991 | 6.27e3 | 1 | 1 | 534 | 537 |  |  | DB Search |
| K.VISLQELC(-1.01).V | Y | 21.07 | 902.4657 | 8 | 1.17 | 903.4718 | 1 | 16.16 | 6425 | 0 | 0 | 0 | 356 | 363 | Half of a disulfide bridge | C8:Half of a disulfide bridge:1000 | DB Search |
| L.RCI.Y | N | 20.6 | 390.2049 | 3 | -4.41 | 391.2095 | 1 | 8.08 | 3003 | 0 | 0 | 0 | 498 | 500 |  |  | DB Search |
| E.TFR.E | N | 20.53 | 422.2278 | 3 | -9.2 | 423.2301 | 1 | 22.38 | 8244 | 2.98e1 | 1 | 1 | 114 | 116 |  |  | DB Search |
| Y.YSPR.D | N | 20.52 | 521.2598 | 4 | 7.22 | 522.2695 | 1 | 8.15 | 2984 | 9.49e2 | 1 | 1 | 722 | 725 |  |  | DB Search |
| I.GTLF.F | N | 20.4 | 436.2322 | 4 | -4.1 | 437.2366 | 1 | 23.18 | 8454 | 1.09e3 | 1 | 1 | 20 | 23 |  |  | DB Search |
| S.WNVPL.V | Y | 19.6 | 627.338 | 5 | 2.34 | 628.3452 | 1 | 26.28 | 9350 | 2.15e2 | 1 | 1 | 1117 | 1121 |  |  | DB Search |
| G.TAY.Y | N | 19.3 | 353.1587 | 3 | -1.32 | 354.1646 | 1 | 12.51 | 4913 | 1.39e3 | 1 | 1 | 719 | 721 |  |  | DB Search |
| L.SLDT.Y | N | 19.03 | 434.2013 | 4 | -3.95 | 435.2057 | 1 | 15.10 | 5999 | 0 | 0 | 0 | 771 | 774 |  |  | DB Search |
| Q.LPTIP.K | N | 18.9 | 539.3319 | 5 | -4.16 | 540.3356 | 1 | 28.83 | 10048 | 0 | 0 | 0 | 339 | 343 |  |  | DB Search |
| L.VIVP.D | N | 18.9 | 426.2842 | 4 | -1.74 | 427.2897 | 1 | 17.76 | 6878 | 3.8e3 | 1 | 1 | 431 | 434 |  |  | DB Search |
| V.IDFS.P | N | 18.2 | 480.222 | 4 | -2.08 | 481.2271 | 1 | 16.69 | 6580 | 0 | 0 | 0 | 143 | 146 |  |  | DB Search |
| F.STLA.P | N | 17.83 | 390.2114 | 4 | -5.32 | 391.2157 | 1 | 6.25 | 2360 | 1.07e4 | 1 | 1 | 34 | 37 |  |  | DB Search |
| D.KHF.S | N | 17.4 | 430.2328 | 3 | 0.78 | 431.2394 | 1 | 20.46 | 7703 | 1.69e2 | 1 | 1 | 447 | 449 |  |  | DB Search |
| Q.Q(-17.03)PIL.S | N | 17.35 | 452.2635 | 4 | -6.01 | 453.2669 | 1 | 32.30 | 10953 | 1.74e3 | 1 | 1 | 207 | 210 | Pyro-glu from Q | Q1:Pyro-glu from Q:1000 | DB Search |
| P.SELA.R | N | 15.6 | 418.2063 | 4 | -9.49 | 419.2086 | 1 | 4.93 | 1962 | 0 | 0 | 0 | 521 | 524 |  |  | DB Search |
| total 19 peptides |
| --- |

Best Unique PSM (Scan POS\_R15-1.wiff:6425, m/z=903.4613, z=1, RT=16.20, ppm=-1.32):


C4R8N7|C4R8N7\_KOMPG

back to list

  

| Protein Coverage
| Supporting Peptides
| Best Unique PSM
|

Protein Coverage:

Supporting Peptides:

| Peptide | Uniq | -10lgP | Mass | Length | ppm | m/z | z | RT | Scan | Area POS\_R15-1 | #Feature | #Feature POS\_R15-1 | Start | End | PTM | AScore | Found By |
| --- | --- | --- | --- | --- | --- | --- | --- | --- | --- | --- | --- | --- | --- | --- | --- | --- | --- |
| D.NGL.L | N | 28.25 | 302.159 | 3 | -3.76 | 303.1644 | 1 | 6.06 | 2374 | 3.14e3 | 1 | 1 | 101 | 103 |  |  | DB Search |
| I.TLPT.F | N | 26.11 | 430.2427 | 4 | -4.36 | 431.2471 | 1 | 9.16 | 3520 | 0 | 0 | 0 | 287 | 290 |  |  | DB Search |
| I.LSLE.I | N | 25.68 | 460.2533 | 4 | -2.19 | 461.2584 | 1 | 13.54 | 5303 | 1.35e4 | 1 | 1 | 455 | 458 |  |  | DB Search |
| F.TSLA.I | N | 24.8 | 390.2114 | 4 | -5.32 | 391.2157 | 1 | 6.25 | 2416 | 1.07e4 | 1 | 1 | 313 | 316 |  |  | DB Search |
| C.ATLF.V | N | 23.38 | 450.2478 | 4 | -9.17 | 451.2498 | 1 | 24.44 | 8854 | 1.68e3 | 1 | 1 | 846 | 849 |  |  | DB Search |
| G.ISGL.I | N | 22.6 | 388.2322 | 4 | -3.81 | 389.237 | 1 | 9.87 | 3804 | 6.08e3 | 1 | 1 | 219 | 222 |  |  | DB Search |
| T.SLAI.A | N | 21.98 | 402.2478 | 4 | -2.21 | 403.2532 | 1 | 20.49 | 7711 | 0 | 0 | 0 | 314 | 317 |  |  | DB Search |
| N.AQIP.L | N | 21.52 | 427.2431 | 4 | -3.04 | 428.248 | 1 | 11.76 | 4668 | 6.31e3 | 1 | 1 | 320 | 323 |  |  | DB Search |
| N.NTS.T | N | 21.44 | 320.1332 | 3 | 8.58 | 321.1424 | 1 | 6.16 | 2421 | 0 | 0 | 0 | 521 | 523 |  |  | DB Search |
| P.PEF.I | N | 20.63 | 391.1743 | 3 | 0.03 | 392.1806 | 1 | 4.06 | 1653 | 5.45e3 | 1 | 1 | 73 | 75 |  |  | DB Search |
| C.RCI.E | N | 20.6 | 390.2049 | 3 | -4.41 | 391.2095 | 1 | 8.08 | 3003 | 0 | 0 | 0 | 388 | 390 |  |  | DB Search |
| F.NDT.L | N | 19.64 | 348.1281 | 3 | 7.11 | 349.137 | 1 | 7.31 | 2704 | 6.3e4 | 1 | 1 | 188 | 190 |  |  | DB Search |
| I.SGLI.N | N | 19.42 | 388.2322 | 4 | -5.15 | 389.2365 | 1 | 17.34 | 6766 | 1.43e3 | 1 | 1 | 220 | 223 |  |  | DB Search |
| H.FPS.H | N | 19.27 | 349.1638 | 3 | -4.81 | 350.1685 | 1 | 16.32 | 6461 | 1.41e3 | 1 | 1 | 61 | 63 |  |  | DB Search |
| N.NITL.P | N | 19.19 | 459.2693 | 4 | -0.54 | 460.2752 | 1 | 17.07 | 6712 | 2.02e3 | 1 | 1 | 285 | 288 |  |  | DB Search |
| S.SNG.V | N | 19.07 | 276.107 | 3 | 1.61 | 277.114 | 1 | 16.82 | 6604 | 0 | 0 | 0 | 210 | 212 |  |  | DB Search |
| W.EIG.V | N | 18.95 | 317.1587 | 3 | -4.31 | 318.1638 | 1 | 5.49 | 2177 | 4.11e3 | 1 | 1 | 639 | 641 |  |  | DB Search |
| N.ESF.L | N | 17.93 | 381.1536 | 3 | -4.82 | 382.1581 | 1 | 5.07 | 1981 | 2.63e3 | 1 | 1 | 490 | 492 |  |  | DB Search |
| C.IEID.I | N | 17.9 | 488.2482 | 4 | 1 | 489.2548 | 1 | 13.83 | 5478 | 0 | 0 | 0 | 390 | 393 |  |  | DB Search |
| I.TSN.K | N | 17.54 | 320.1332 | 3 | 3.34 | 321.1407 | 1 | 8.11 | 3024 | 0 | 0 | 0 | 687 | 689 |  |  | DB Search |
| R.IITD | N | 17.52 | 460.2533 | 4 | -9.09 | 461.2552 | 1 | 10.16 | 3965 | 9.46e2 | 1 | 1 | 859 | 862 |  |  | DB Search |
| G.RSF.T | N | 17.5 | 408.2121 | 3 | -1.76 | 409.2177 | 1 | 9.17 | 3526 | 0 | 0 | 0 | 426 | 428 |  |  | DB Search |
| S.FNH.V | N | 17.22 | 416.1808 | 3 | -5.74 | 417.1847 | 1 | 6.08 | 2390 | 0 | 0 | 0 | 585 | 587 |  |  | DB Search |
| H.ESN.Q | N | 17.14 | 348.1281 | 3 | 8.51 | 349.1375 | 1 | 5.67 | 2161 | 1.07e4 | 1 | 1 | 278 | 280 |  |  | DB Search |
| S.DTLP.S | N | 17.01 | 444.222 | 4 | -3.28 | 445.2267 | 1 | 12.52 | 4902 | 1.87e3 | 1 | 1 | 18 | 21 |  |  | DB Search |
| K.E(-18.01)PI.M | N | 16.1 | 339.1794 | 3 | -5.62 | 340.1839 | 1 | 16.05 | 6351 | 1.63e4 | 1 | 1 | 484 | 486 | Pyro-glu from E | E1:Pyro-glu from E:1000 | DB Search |
| P.TFT.I | N | 15.92 | 367.1743 | 3 | -7.47 | 368.1779 | 1 | 8.54 | 3263 | 0 | 0 | 0 | 290 | 292 |  |  | DB Search |
| V.PMVATN(+0.98).W | Y | 15.91 | 632.284 | 6 | 4.94 | 633.2928 | 1 | 12.02 | 4727 | 3.52e2 | 1 | 1 | 643 | 648 | Deamidation (NQ) | N6:Deamidation (NQ):1000 | DB Search |
| S.NGV.V | N | 15.37 | 288.1434 | 3 | 8 | 289.1522 | 1 | 5.91 | 2301 | 6.8e3 | 1 | 1 | 211 | 213 |  |  | DB Search |
| E.IGVP.M | N | 15.32 | 384.2372 | 4 | -3.68 | 385.2422 | 1 | 16.13 | 6408 | 0 | 0 | 0 | 640 | 643 |  |  | DB Search |
| L.Q(-17.03)PI.K | N | 15.26 | 339.1794 | 3 | -4.38 | 340.1844 | 1 | 15.14 | 5814 | 1.63e4 | 1 | 1 | 337 | 339 | Pyro-glu from Q | Q1:Pyro-glu from Q:1000 | DB Search |
| D.Q(-17.03)PF.N | N | 15.22 | 373.1638 | 3 | -0.75 | 374.1698 | 1 | 21.41 | 7929 | 8.45e4 | 1 | 1 | 665 | 667 | Pyro-glu from Q | Q1:Pyro-glu from Q:1000 | DB Search |
| total 32 peptides |
| --- |

Best Unique PSM (Scan POS\_R15-1.wiff:4727, m/z=633.2928, z=1, RT=12.02, ppm=2.46):


C4R4D4|C4R4D4\_KOMPG

back to list

  

| Protein Coverage
| Supporting Peptides
| Best Unique PSM
|

Protein Coverage:

Supporting Peptides:

| Peptide | Uniq | -10lgP | Mass | Length | ppm | m/z | z | RT | Scan | Area POS\_R15-1 | #Feature | #Feature POS\_R15-1 | Start | End | PTM | AScore | Found By |
| --- | --- | --- | --- | --- | --- | --- | --- | --- | --- | --- | --- | --- | --- | --- | --- | --- | --- |
| I.YSIF.E | N | 26.31 | 528.2584 | 4 | -2.91 | 529.2628 | 1 | 34.97 | 11598 | 3.74e3 | 1 | 1 | 509 | 512 |  |  | DB Search |
| P.IVAP.T | N | 22.5 | 398.2529 | 4 | -6.51 | 399.2566 | 1 | 9.50 | 3744 | 1.49e3 | 1 | 1 | 533 | 536 |  |  | DB Search |
| I.TIGV.D | N | 18.91 | 388.2322 | 4 | -5.15 | 389.2365 | 1 | 17.34 | 6852 | 1.43e3 | 1 | 1 | 340 | 343 |  |  | DB Search |
| R.KHF.I | N | 17.4 | 430.2328 | 3 | 0.78 | 431.2394 | 1 | 20.46 | 7703 | 1.69e2 | 1 | 1 | 59 | 61 |  |  | DB Search |
| F.FFAP.A | N | 16.42 | 480.2372 | 4 | -4.03 | 481.2414 | 1 | 10.17 | 3931 | 9.58e2 | 1 | 1 | 25 | 28 |  |  | DB Search |
| G.IGRIIYSI.F | Y | 16.29 | 933.5647 | 8 | -2.15 | 467.7875 | 2 | 34.97 | 11670 | 1.68e3 | 1 | 1 | 504 | 511 |  |  | DB Search |
| total 6 peptides |
| --- |

Best Unique PSM (Scan POS\_R15-1.wiff:11670, m/z=467.7889, z=2, RT=34.97, ppm=-4.64):


C4R2L2|C4R2L2\_KOMPG

back to list

  

| Protein Coverage
| Supporting Peptides
| Best Unique PSM
|

Protein Coverage:

Supporting Peptides:

| Peptide | Uniq | -10lgP | Mass | Length | ppm | m/z | z | RT | Scan | Area POS\_R15-1 | #Feature | #Feature POS\_R15-1 | Start | End | PTM | AScore | Found By |
| --- | --- | --- | --- | --- | --- | --- | --- | --- | --- | --- | --- | --- | --- | --- | --- | --- | --- |
| F.ALLSL.P | N | 24.46 | 515.3319 | 5 | -6.84 | 516.3344 | 1 | 37.01 | 12185 | 8.65e2 | 1 | 1 | 947 | 951 |  |  | DB Search |
| G.RCL.S | N | 20.6 | 390.2049 | 3 | -4.41 | 391.2095 | 1 | 8.08 | 3003 | 0 | 0 | 0 | 1033 | 1035 |  |  | DB Search |
| H.TFR.L | N | 20.53 | 422.2278 | 3 | -9.2 | 423.2301 | 1 | 22.38 | 8244 | 2.98e1 | 1 | 1 | 923 | 925 |  |  | DB Search |
| F.SLTP.E | N | 19.55 | 416.2271 | 4 | 2 | 417.2342 | 1 | 10.25 | 4029 | 3.69e3 | 1 | 1 | 375 | 378 |  |  | DB Search |
| A.PAFV.E | N | 19.17 | 432.2372 | 4 | -3.2 | 433.2421 | 1 | 24.76 | 8853 | 3.98e2 | 1 | 1 | 960 | 963 |  |  | DB Search |
| N.LIQP.V | N | 19.13 | 469.29 | 4 | -8.42 | 470.2922 | 1 | 12.16 | 4756 | 6.43e3 | 1 | 1 | 308 | 311 |  |  | DB Search |
| K.QIQ(+0.98)QLQI.Q | N | 18.57 | 870.4811 | 7 | -8.47 | 871.4788 | 1 | 30.71 | 10532 | 3.59e2 | 1 | 1 | 394 | 400 | Deamidation (NQ) | Q3:Deamidation (NQ):14.04 | DB Search |
| Q.SPSL.Y | N | 18.22 | 402.2114 | 4 | -3.42 | 403.2163 | 1 | 12.42 | 4855 | 2.18e3 | 1 | 1 | 148 | 151 |  |  | DB Search |
| Q.Q(-17.03)QQPQVIPQ(+0.98)RSQ(+0.98).L | Y | 16.14 | 1420.6947 | 12 | 3.1 | 711.355 | 2 | 28.09 | 9847 | 2.4e4 | 1 | 1 | 480 | 491 | Pyro-glu from Q, Deamidation (NQ), Deamidation (NQ) | Q1:Pyro-glu from Q:1000 Q9:Deamidation (NQ):61.69 Q12:Deamidation (NQ):50.34 | DB Search |
| S.DVGL.D | N | 15.88 | 402.2114 | 4 | -1.5 | 403.2171 | 1 | 9.75 | 3763 | 2.23e3 | 1 | 1 | 624 | 627 |  |  | DB Search |
| total 10 peptides |
| --- |

Best Unique PSM (Scan POS\_R15-1.wiff:9847, m/z=711.3598, z=2, RT=28.26, ppm=0.62):


C4R153|C4R153\_KOMPG

back to list

  

| Protein Coverage
| Supporting Peptides
| Best Unique PSM
|

Protein Coverage:

Supporting Peptides:

| Peptide | Uniq | -10lgP | Mass | Length | ppm | m/z | z | RT | Scan | Area POS\_R15-1 | #Feature | #Feature POS\_R15-1 | Start | End | PTM | AScore | Found By |
| --- | --- | --- | --- | --- | --- | --- | --- | --- | --- | --- | --- | --- | --- | --- | --- | --- | --- |
| R.LPRP.R | N | 21.04 | 481.3012 | 4 | -9.61 | 482.3027 | 1 | 17.02 | 6682 | 0 | 0 | 0 | 84 | 87 |  |  | DB Search |
| K.TFR.S | N | 20.53 | 422.2278 | 3 | -9.2 | 423.2301 | 1 | 22.38 | 8244 | 2.98e1 | 1 | 1 | 447 | 449 |  |  | DB Search |
| R.SLGD.Y | N | 20.24 | 390.175 | 4 | -7.72 | 391.1783 | 1 | 3.30 | 1421 | 1.05e3 | 1 | 1 | 504 | 507 |  |  | DB Search |
| I.NDT.E | N | 19.64 | 348.1281 | 3 | 7.11 | 349.137 | 1 | 7.31 | 2704 | 6.3e4 | 1 | 1 | 340 | 342 |  |  | DB Search |
| K.FPS.N | N | 19.27 | 349.1638 | 3 | -4.81 | 350.1685 | 1 | 16.32 | 6461 | 1.41e3 | 1 | 1 | 51 | 53 |  |  | DB Search |
| S.EIG.F | N | 18.95 | 317.1587 | 3 | -4.31 | 318.1638 | 1 | 5.49 | 2177 | 4.11e3 | 1 | 1 | 432 | 434 |  |  | DB Search |
| F.LELD.S | N | 17.9 | 488.2482 | 4 | 1 | 489.2548 | 1 | 13.83 | 5478 | 0 | 0 | 0 | 173 | 176 |  |  | DB Search |
| E.VLPV.L | N | 17 | 426.2842 | 4 | -1.74 | 427.2897 | 1 | 17.76 | 6936 | 3.8e3 | 1 | 1 | 193 | 196 |  |  | DB Search |
| E.E(-18.01)PI.A | N | 16.1 | 339.1794 | 3 | -5.62 | 340.1839 | 1 | 16.05 | 6351 | 1.63e4 | 1 | 1 | 151 | 153 | Pyro-glu from E | E1:Pyro-glu from E:1000 | DB Search |
| S.SLIE.K | N | 15.23 | 460.2533 | 4 | -2.19 | 461.2584 | 1 | 13.54 | 5437 | 1.35e4 | 1 | 1 | 524 | 527 |  |  | DB Search |
| F.DAPRA.N | Y | 15.16 | 528.2656 | 5 | 5.19 | 529.2743 | 1 | 14.53 | 5735 | 4.7e1 | 1 | 1 | 206 | 210 |  |  | DB Search |
| total 11 peptides |
| --- |

Best Unique PSM (Scan POS\_R15-1.wiff:5735, m/z=530.2267, z=1, RT=14.59, ppm=2.7):


C4QYA1|C4QYA1\_KOMPG

back to list

  

| Protein Coverage
| Supporting Peptides
| Best Unique PSM
|

Protein Coverage:

Supporting Peptides:

| Peptide | Uniq | -10lgP | Mass | Length | ppm | m/z | z | RT | Scan | Area POS\_R15-1 | #Feature | #Feature POS\_R15-1 | Start | End | PTM | AScore | Found By |
| --- | --- | --- | --- | --- | --- | --- | --- | --- | --- | --- | --- | --- | --- | --- | --- | --- | --- |
| E.SPT.Y | N | 23.16 | 303.143 | 3 | 6.39 | 304.1515 | 1 | 7.14 | 2731 | 5.4e3 | 2 | 2 | 186 | 188 |  |  | DB Search |
| E.EIF.S | N | 21.7 | 407.2056 | 3 | -8.99 | 408.2082 | 1 | 29.23 | 10157 | 9.42e2 | 1 | 1 | 379 | 381 |  |  | DB Search |
| T.TFR.P | N | 20.53 | 422.2278 | 3 | -9.2 | 423.2301 | 1 | 22.38 | 8244 | 2.98e1 | 1 | 1 | 252 | 254 |  |  | DB Search |
| S.NSG.S | N | 20.42 | 276.107 | 3 | 6.48 | 277.1154 | 1 | 16.74 | 6592 | 0 | 0 | 0 | 334 | 336 |  |  | DB Search |
| S.PQFPP.Q | Y | 19.84 | 584.2958 | 5 | -6.09 | 585.2981 | 1 | 19.54 | 7437 | 3.9e2 | 1 | 1 | 87 | 91 |  |  | DB Search |
| E.NDT.K | N | 19.64 | 348.1281 | 3 | 7.11 | 349.137 | 1 | 7.31 | 2704 | 6.3e4 | 1 | 1 | 268 | 270 |  |  | DB Search |
| Q.FPPQ.K | N | 18.18 | 487.2431 | 4 | -6.62 | 488.2459 | 1 | 11.19 | 4443 | 2.22e3 | 1 | 1 | 89 | 92 |  |  | DB Search |
| G.FGRL.L | N | 17.51 | 491.2856 | 4 | -7.95 | 492.2878 | 1 | 15.01 | 5965 | 9.54e2 | 1 | 1 | 278 | 281 |  |  | DB Search |
| E.SELA.G | N | 15.6 | 418.2063 | 4 | -9.49 | 419.2086 | 1 | 4.93 | 1962 | 0 | 0 | 0 | 239 | 242 |  |  | DB Search |
| total 9 peptides |
| --- |

Best Unique PSM (Scan POS\_R15-1.wiff:7437, m/z=585.2505, z=2, RT=19.63, ppm=-8.57):


C4R123|C4R123\_KOMPG

back to list

  

| Protein Coverage
| Supporting Peptides
| Best Unique PSM
|

Protein Coverage:

Supporting Peptides:

| Peptide | Uniq | -10lgP | Mass | Length | ppm | m/z | z | RT | Scan | Area POS\_R15-1 | #Feature | #Feature POS\_R15-1 | Start | End | PTM | AScore | Found By |
| --- | --- | --- | --- | --- | --- | --- | --- | --- | --- | --- | --- | --- | --- | --- | --- | --- | --- |
| Y.LGSL.N | N | 25.26 | 388.2322 | 4 | -3.81 | 389.237 | 1 | 9.87 | 3785 | 6.08e3 | 1 | 1 | 708 | 711 |  |  | DB Search |
| K.HLN(+0.98)RSPP.S | Y | 21.65 | 820.4191 | 7 | -7.96 | 821.4178 | 1 | 29.43 | 10202 | 2.6e2 | 1 | 1 | 59 | 65 | Deamidation (NQ) | N3:Deamidation (NQ):1000 | DB Search |
| L.VPVE.S | N | 20.6 | 442.2427 | 4 | -5.67 | 443.2464 | 1 | 7.79 | 2913 | 1.07e3 | 1 | 1 | 390 | 393 |  |  | DB Search |
| T.VSSL.A | N | 20.48 | 404.2271 | 4 | -7.08 | 405.2305 | 1 | 8.17 | 2993 | 7.47e3 | 1 | 1 | 666 | 669 |  |  | DB Search |
| V.SIGD.C | N | 20.24 | 390.175 | 4 | -7.72 | 391.1783 | 1 | 3.30 | 1421 | 1.05e3 | 1 | 1 | 557 | 560 |  |  | DB Search |
| C.IAEV.M | N | 19.27 | 430.2427 | 4 | 1.69 | 431.2497 | 1 | 13.56 | 5318 | 0 | 0 | 0 | 824 | 827 |  |  | DB Search |
| V.IIQP.N | N | 19.13 | 469.29 | 4 | -8.42 | 470.2922 | 1 | 12.16 | 4756 | 6.43e3 | 1 | 1 | 850 | 853 |  |  | DB Search |
| S.RDR.S | N | 18.68 | 445.2397 | 3 | 8.14 | 446.2495 | 1 | 10.60 | 4131 | 1.44e2 | 1 | 1 | 889 | 891 |  |  | DB Search |
| R.ETSW.Q | N | 17.06 | 521.2122 | 4 | 1.03 | 522.2187 | 1 | 13.40 | 5265 | 3.31e2 | 1 | 1 | 1000 | 1003 |  |  | DB Search |
| L.E(-18.01)PR.E | N | 16.87 | 382.1965 | 3 | -0.37 | 383.2026 | 1 | 2.61 | 1082 | 1.62e4 | 1 | 1 | 246 | 248 | Pyro-glu from E | E1:Pyro-glu from E:1000 | DB Search |
| R.AQIE.F | N | 16.19 | 459.2329 | 4 | -1.44 | 460.2384 | 1 | 4.72 | 1830 | 1.97e3 | 1 | 1 | 195 | 198 |  |  | DB Search |
| F.Q(-17.03)PH.P | N | 15.48 | 363.1543 | 3 | -0.14 | 364.1606 | 1 | 2.32 | 992 | 3.39e4 | 1 | 1 | 476 | 478 | Pyro-glu from Q | Q1:Pyro-glu from Q:1000 | DB Search |
| T.LTET.I | N | 15.47 | 462.2326 | 4 | 1.41 | 463.2393 | 1 | 15.37 | 6065 | 2.41e2 | 1 | 1 | 787 | 790 |  |  | DB Search |
| total 13 peptides |
| --- |

Best Unique PSM (Scan POS\_R15-1.wiff:10202, m/z=821.4178, z=1, RT=29.43, ppm=-10.44):


C4QV05|C4QV05\_KOMPG

back to list

  

| Protein Coverage
| Supporting Peptides
| Best Unique PSM
|

Protein Coverage:

Supporting Peptides:

| Peptide | Uniq | -10lgP | Mass | Length | ppm | m/z | z | RT | Scan | Area POS\_R15-1 | #Feature | #Feature POS\_R15-1 | Start | End | PTM | AScore | Found By |
| --- | --- | --- | --- | --- | --- | --- | --- | --- | --- | --- | --- | --- | --- | --- | --- | --- | --- |
| P.AITIG.Y | N | 23.3 | 473.2849 | 5 | -4.41 | 474.2889 | 1 | 17.39 | 6818 | 3.71e3 | 1 | 1 | 171 | 175 |  |  | DB Search |
| L.SPT.D | N | 23.16 | 303.143 | 3 | 6.39 | 304.1515 | 1 | 7.14 | 2731 | 5.4e3 | 2 | 2 | 39 | 41 |  |  | DB Search |
| L.VILP.G | N | 22.17 | 440.2998 | 4 | -5.96 | 441.3034 | 1 | 30.99 | 10567 | 1.25e3 | 1 | 1 | 62 | 65 |  |  | DB Search |
| V.KDLL.R | N | 22.06 | 487.3006 | 4 | -0.76 | 488.3063 | 1 | 10.37 | 4062 | 0 | 0 | 0 | 282 | 285 |  |  | DB Search |
| I.YGR.A | N | 21.6 | 394.1965 | 3 | -4.25 | 395.2011 | 1 | 5.35 | 1961 | 1.67e4 | 1 | 1 | 337 | 339 |  |  | DB Search |
| I.AAVE.D | N | 20.66 | 388.1958 | 4 | -4.49 | 389.2003 | 1 | 2.65 | 1124 | 0 | 0 | 0 | 394 | 397 |  |  | DB Search |
| S.RPD.S | N | 19.79 | 386.1914 | 3 | -3.61 | 387.1963 | 1 | 2.95 | 1225 | 1.33e2 | 1 | 1 | 416 | 418 |  |  | DB Search |
| L.TAY.N | N | 19.3 | 353.1587 | 3 | -1.32 | 354.1646 | 1 | 12.51 | 4913 | 1.39e3 | 1 | 1 | 618 | 620 |  |  | DB Search |
| A.SVGL.G | N | 18.49 | 374.2165 | 4 | -4.96 | 375.221 | 1 | 14.62 | 5748 | 1.93e2 | 1 | 1 | 542 | 545 |  |  | DB Search |
| T.TVVS.D | N | 18.11 | 404.2271 | 4 | -7.08 | 405.2305 | 1 | 8.17 | 3091 | 7.47e3 | 1 | 1 | 513 | 516 |  |  | DB Search |
| I.SFTEIAKWTSLNT.A | Y | 17.42 | 1496.7511 | 13 | 3.86 | 749.3839 | 2 | 37.23 | 12244 | 2.34e2 | 1 | 1 | 562 | 574 |  |  | DB Search |
| V.SGLH.T | N | 16.98 | 412.207 | 4 | -3.29 | 413.2119 | 1 | 1.21 | 286 | 3.65e4 | 1 | 1 | 185 | 188 |  |  | DB Search |
| S.SPK.G | N | 16.89 | 330.1903 | 3 | -8.46 | 331.194 | 1 | 5.91 | 2235 | 5.17e3 | 1 | 1 | 205 | 207 |  |  | DB Search |
| L.E(-18.01)PR.F | N | 16.87 | 382.1965 | 3 | -0.37 | 383.2026 | 1 | 2.61 | 1082 | 1.62e4 | 1 | 1 | 657 | 659 | Pyro-glu from E | E1:Pyro-glu from E:1000 | DB Search |
| A.AAQ.G | N | 16.83 | 288.1434 | 3 | 8 | 289.1522 | 1 | 5.91 | 2315 | 6.8e3 | 1 | 1 | 123 | 125 |  |  | DB Search |
| K.LDLG.M | N | 15.65 | 416.2271 | 4 | -5.75 | 417.2309 | 1 | 13.92 | 5536 | 5.65e3 | 1 | 1 | 265 | 268 |  |  | DB Search |
| Y.NGV.N | N | 15.37 | 288.1434 | 3 | 8 | 289.1522 | 1 | 5.91 | 2301 | 6.8e3 | 1 | 1 | 621 | 623 |  |  | DB Search |
| total 17 peptides |
| --- |

Best Unique PSM (Scan POS\_R15-1.wiff:12244, m/z=749.3839, z=2, RT=37.23, ppm=1.38):


C4QVT8|C4QVT8\_KOMPG

back to list

  

| Protein Coverage
| Supporting Peptides
| Best Unique PSM
|

Protein Coverage:

Supporting Peptides:

| Peptide | Uniq | -10lgP | Mass | Length | ppm | m/z | z | RT | Scan | Area POS\_R15-1 | #Feature | #Feature POS\_R15-1 | Start | End | PTM | AScore | Found By |
| --- | --- | --- | --- | --- | --- | --- | --- | --- | --- | --- | --- | --- | --- | --- | --- | --- | --- |
| F.FYR.G | N | 26.82 | 484.2434 | 3 | 1.64 | 485.2503 | 1 | 17.34 | 6854 | 0 | 0 | 0 | 1425 | 1427 |  |  | DB Search |
| Q.ELIR.T | N | 25.05 | 529.3224 | 4 | -4.56 | 530.3259 | 1 | 17.34 | 6855 | 6.68e3 | 1 | 1 | 205 | 208 |  |  | DB Search |
| Y.AIFG.G | N | 24.47 | 406.2216 | 4 | 1.3 | 407.2284 | 1 | 22.63 | 8312 | 0 | 0 | 0 | 166 | 169 |  |  | DB Search |
| T.ILIP.T | N | 24.07 | 454.3155 | 4 | -2.4 | 455.3206 | 1 | 34.52 | 11565 | 2.64e3 | 1 | 1 | 23 | 26 |  |  | DB Search |
| Q.SPT.Q | N | 23.16 | 303.143 | 3 | 6.39 | 304.1515 | 1 | 7.14 | 2731 | 5.4e3 | 2 | 2 | 1019 | 1021 |  |  | DB Search |
| Y.ANLP.G | N | 22.26 | 413.2274 | 4 | 0.01 | 414.2337 | 1 | 11.08 | 4376 | 0 | 0 | 0 | 1589 | 1592 |  |  | DB Search |
| I.KDIL.E | N | 22.06 | 487.3006 | 4 | -0.76 | 488.3063 | 1 | 10.37 | 4062 | 0 | 0 | 0 | 1081 | 1084 |  |  | DB Search |
| D.YNP.I | N | 21.45 | 392.1696 | 3 | -3.75 | 393.1744 | 1 | 8.45 | 3226 | 1.39e2 | 1 | 1 | 1576 | 1578 |  |  | DB Search |
| P.NTS.L | N | 21.44 | 320.1332 | 3 | 8.58 | 321.1424 | 1 | 6.16 | 2421 | 0 | 0 | 0 | 324 | 326 |  |  | DB Search |
| A.TSG.V | N | 21.44 | 263.1117 | 3 | 2.31 | 264.119 | 1 | 3.71 | 1537 | 5.72e3 | 1 | 1 | 4 | 6 |  |  | DB Search |
| I.DVTL.R | N | 21.02 | 446.2376 | 4 | -4.47 | 447.2418 | 1 | 16.24 | 6386 | 3.07e3 | 1 | 1 | 949 | 952 |  |  | DB Search |
| L.TFR.C | N | 20.53 | 422.2278 | 3 | -9.2 | 423.2301 | 1 | 22.38 | 8244 | 2.98e1 | 1 | 1 | 1478 | 1480 |  |  | DB Search |
| R.ALVE.Q | N | 20.45 | 430.2427 | 4 | -5.9 | 431.2464 | 1 | 12.49 | 4901 | 0 | 0 | 0 | 1607 | 1610 |  |  | DB Search |
| K.SIGD.L | N | 20.24 | 390.175 | 4 | -7.72 | 391.1783 | 1 | 3.30 | 1421 | 1.05e3 | 1 | 1 | 1225 | 1228 |  |  | DB Search |
| G.ISVP.F | N | 19.74 | 414.2478 | 4 | -0.09 | 415.254 | 1 | 17.14 | 6914 | 3.72e3 | 1 | 1 | 1990 | 1993 |  |  | DB Search |
| S.QFF.Y | N | 19.56 | 440.206 | 3 | 3.92 | 441.2139 | 1 | 19.15 | 7313 | 0 | 0 | 0 | 1423 | 1425 |  |  | DB Search |
| L.SNG.Y | N | 19.07 | 276.107 | 3 | 1.61 | 277.114 | 1 | 16.82 | 6604 | 0 | 0 | 0 | 1368 | 1370 |  |  | DB Search |
| V.EQW.A | N | 18.42 | 461.191 | 3 | -3.23 | 462.1957 | 1 | 11.54 | 4588 | 2.45e3 | 1 | 1 | 1610 | 1612 |  |  | DB Search |
| L.LSIP.M | N | 18.29 | 428.2635 | 4 | -7.33 | 429.2665 | 1 | 24.15 | 8767 | 0 | 0 | 0 | 240 | 243 |  |  | DB Search |
| T.FRC.E | N | 17.7 | 424.1893 | 3 | -9.2 | 425.1916 | 1 | 10.20 | 3929 | 1.22e3 | 1 | 1 | 1479 | 1481 |  |  | DB Search |
| T.DGFA.P | N | 17.56 | 408.1645 | 4 | -4.25 | 409.169 | 1 | 8.22 | 3102 | 0 | 0 | 0 | 1258 | 1261 |  |  | DB Search |
| V.TCR.I | N | 17.37 | 378.1685 | 3 | 5.06 | 379.1768 | 1 | 12.47 | 4890 | 0 | 0 | 0 | 260 | 262 |  |  | DB Search |
| H.Q(-17.03)PIL.Q | N | 17.35 | 452.2635 | 4 | -6.01 | 453.2669 | 1 | 32.30 | 10953 | 1.74e3 | 1 | 1 | 757 | 760 | Pyro-glu from Q | Q1:Pyro-glu from Q:1000 | DB Search |
| V.LDFGP.G | N | 17.23 | 547.2642 | 5 | 0.18 | 548.2702 | 1 | 16.89 | 6622 | 0 | 0 | 0 | 510 | 514 |  |  | DB Search |
| P.GTITHG.M | Y | 17 | 584.2918 | 6 | -1.19 | 585.2969 | 1 | 23.90 | 8681 | 0 | 0 | 0 | 1593 | 1598 |  |  | DB Search |
| V.AAQ.F | N | 16.83 | 288.1434 | 3 | 8 | 289.1522 | 1 | 5.91 | 2315 | 6.8e3 | 1 | 1 | 1069 | 1071 |  |  | DB Search |
| V.DVVF.Y | N | 16.74 | 478.2427 | 4 | 2.28 | 479.2499 | 1 | 30.82 | 10557 | 3.05e2 | 1 | 1 | 1847 | 1850 |  |  | DB Search |
| E.TFT.V | N | 15.92 | 367.1743 | 3 | -7.47 | 368.1779 | 1 | 8.54 | 3263 | 0 | 0 | 0 | 1137 | 1139 |  |  | DB Search |
| L.QAVE.F | N | 15.8 | 445.2172 | 4 | 7.06 | 446.2266 | 1 | 2.45 | 1067 | 0 | 0 | 0 | 306 | 309 |  |  | DB Search |
| T.HAVG.N | N | 15.71 | 382.1964 | 4 | -0.36 | 383.2026 | 1 | 2.61 | 1034 | 1.62e4 | 1 | 1 | 1316 | 1319 |  |  | DB Search |
| R.KSLP.Q | N | 15.42 | 443.2744 | 4 | -3.64 | 444.2789 | 1 | 17.16 | 6753 | 0 | 0 | 0 | 42 | 45 |  |  | DB Search |
| V.NPRM(+15.99)LQWGIPM(+15.99)I.K | Y | 15.05 | 1486.7425 | 12 | -0.75 | 744.3761 | 2 | 29.87 | 10312 | 0 | 0 | 0 | 664 | 675 | Oxidation (M), Oxidation (M) | M4:Oxidation (M):1000 M11:Oxidation (M):1000 | DB Search |
| total 32 peptides |
| --- |

Best Unique PSM (Scan POS\_R15-1.wiff:8681, m/z=585.3195, z=1, RT=24.08, ppm=-3.68):


C4R2V7|C4R2V7\_KOMPG

back to list

  

| Protein Coverage
| Supporting Peptides
| Best Unique PSM
|

Protein Coverage:

Supporting Peptides:

| Peptide | Uniq | -10lgP | Mass | Length | ppm | m/z | z | RT | Scan | Area POS\_R15-1 | #Feature | #Feature POS\_R15-1 | Start | End | PTM | AScore | Found By |
| --- | --- | --- | --- | --- | --- | --- | --- | --- | --- | --- | --- | --- | --- | --- | --- | --- | --- |
| C.ITGL.N | N | 27.55 | 402.2478 | 4 | -1.76 | 403.2534 | 1 | 15.04 | 5964 | 1.56e4 | 2 | 2 | 241 | 244 |  |  | DB Search |
| K.PVIV.I | N | 23.27 | 426.2842 | 4 | 4.41 | 427.2923 | 1 | 20.36 | 7664 | 0 | 0 | 0 | 513 | 516 |  |  | DB Search |
| K.FVVP.E | N | 23.15 | 460.2685 | 4 | -7.54 | 461.2712 | 1 | 28.36 | 9923 | 0 | 0 | 0 | 131 | 134 |  |  | DB Search |
| D.VGSL.Q | N | 21.91 | 374.2165 | 4 | 0.34 | 375.223 | 1 | 13.82 | 5444 | 2.07e3 | 1 | 1 | 559 | 562 |  |  | DB Search |
| R.SSLF.K | N | 21.63 | 452.2271 | 4 | -3.7 | 453.2316 | 1 | 22.49 | 8262 | 1.33e3 | 1 | 1 | 315 | 318 |  |  | DB Search |
| V.GYYP.S | N | 20.14 | 498.2114 | 4 | 1.33 | 499.2181 | 1 | 14.02 | 5579 | 4.82e2 | 1 | 1 | 427 | 430 |  |  | DB Search |
| N.KGHNTFGPAE.V | Y | 19.63 | 1056.4988 | 10 | 5.88 | 529.2585 | 2 | 13.90 | 5521 | 0 | 0 | 0 | 657 | 666 |  |  | DB Search |
| G.DTLP.L | N | 17.01 | 444.222 | 4 | -3.28 | 445.2267 | 1 | 12.52 | 4902 | 1.87e3 | 1 | 1 | 495 | 498 |  |  | DB Search |
| I.AHVI.Y | N | 16.82 | 438.259 | 4 | -6.35 | 439.2625 | 1 | 8.70 | 3374 | 6.21e2 | 1 | 1 | 550 | 553 |  |  | DB Search |
| V.DLVT.Q | N | 16.75 | 446.2376 | 4 | -4.95 | 447.2416 | 1 | 23.24 | 8484 | 0 | 0 | 0 | 443 | 446 |  |  | DB Search |
| N.IAHV.I | N | 15.98 | 438.259 | 4 | -6.35 | 439.2625 | 1 | 8.70 | 3417 | 6.21e2 | 1 | 1 | 549 | 552 |  |  | DB Search |
| A.YMR.R | N | 15.84 | 468.2155 | 3 | 0.49 | 469.2218 | 1 | 8.86 | 3455 | 2.39e3 | 1 | 1 | 625 | 627 |  |  | DB Search |
| V.EFEG.P | N | 15.01 | 480.1856 | 4 | -5.04 | 481.1893 | 1 | 10.64 | 4144 | 0 | 0 | 0 | 190 | 193 |  |  | DB Search |
| total 13 peptides |
| --- |

Best Unique PSM (Scan POS\_R15-1.wiff:5521, m/z=529.2585, z=2, RT=13.90, ppm=3.4):


C4R782|C4R782\_KOMPG

back to list

  

| Protein Coverage
| Supporting Peptides
| Best Unique PSM
|

Protein Coverage:

Supporting Peptides:

| Peptide | Uniq | -10lgP | Mass | Length | ppm | m/z | z | RT | Scan | Area POS\_R15-1 | #Feature | #Feature POS\_R15-1 | Start | End | PTM | AScore | Found By |
| --- | --- | --- | --- | --- | --- | --- | --- | --- | --- | --- | --- | --- | --- | --- | --- | --- | --- |
| N.VGSQP.D | Y | 23.82 | 486.2438 | 5 | 2.03 | 487.2509 | 1 | 4.40 | 1725 | 1.26e3 | 2 | 2 | 123 | 127 |  |  | DB Search |
| D.QFF.E | N | 19.56 | 440.206 | 3 | 3.92 | 441.2139 | 1 | 19.15 | 7313 | 0 | 0 | 0 | 62 | 64 |  |  | DB Search |
| K.ESPL.R | N | 18.2 | 444.222 | 4 | -3.28 | 445.2267 | 1 | 12.52 | 4847 | 1.87e3 | 1 | 1 | 507 | 510 |  |  | DB Search |
| T.FNH.L | N | 17.22 | 416.1808 | 3 | -5.74 | 417.1847 | 1 | 6.08 | 2390 | 0 | 0 | 0 | 280 | 282 |  |  | DB Search |
| K.SHAP.T | N | 16.11 | 410.1914 | 4 | 5.6 | 411.1999 | 1 | 13.65 | 5386 | 5.56e2 | 1 | 1 | 326 | 329 |  |  | DB Search |
| K.AMY.S | N | 15.75 | 383.1515 | 3 | -9.49 | 384.1542 | 1 | 17.73 | 6972 | 0 | 0 | 0 | 410 | 412 |  |  | DB Search |
| total 6 peptides |
| --- |

Best Unique PSM (Scan POS\_R15-1.wiff:1725, m/z=487.2509, z=1, RT=4.40, ppm=-0.45):


C4R6X0|C4R6X0\_KOMPG

back to list

  

| Protein Coverage
| Supporting Peptides
| Best Unique PSM
|

Protein Coverage:

Supporting Peptides:

| Peptide | Uniq | -10lgP | Mass | Length | ppm | m/z | z | RT | Scan | Area POS\_R15-1 | #Feature | #Feature POS\_R15-1 | Start | End | PTM | AScore | Found By |
| --- | --- | --- | --- | --- | --- | --- | --- | --- | --- | --- | --- | --- | --- | --- | --- | --- | --- |
| F.PSIL.S | N | 29.95 | 428.2635 | 4 | -0.56 | 429.2694 | 1 | 21.28 | 7884 | 0 | 0 | 0 | 1069 | 1072 |  |  | DB Search |
| T.ATLF.G | N | 23.38 | 450.2478 | 4 | -9.17 | 451.2498 | 1 | 24.44 | 8854 | 1.68e3 | 1 | 1 | 2223 | 2226 |  |  | DB Search |
| P.MLLTT.T | N | 22.82 | 577.3145 | 5 | -6.02 | 578.3169 | 1 | 21.75 | 8004 | 0 | 0 | 0 | 651 | 655 |  |  | DB Search |
| H.PQNAHEI.A | Y | 22.73 | 807.3875 | 7 | 1.12 | 808.3937 | 1 | 23.85 | 8645 | 9.34e2 | 1 | 1 | 1652 | 1658 |  |  | DB Search |
| A.ISGL.K | N | 22.6 | 388.2322 | 4 | -3.81 | 389.237 | 1 | 9.87 | 3804 | 6.08e3 | 1 | 1 | 822 | 825 |  |  | DB Search |
| L.ANLP.S | N | 22.26 | 413.2274 | 4 | 0.01 | 414.2337 | 1 | 11.08 | 4376 | 0 | 0 | 0 | 2051 | 2054 |  |  | DB Search |
| S.YGR.C | N | 21.6 | 394.1965 | 3 | -4.25 | 395.2011 | 1 | 5.35 | 1961 | 1.67e4 | 1 | 1 | 1348 | 1350 |  |  | DB Search |
| S.AQLP.T | N | 21.52 | 427.2431 | 4 | -3.04 | 428.248 | 1 | 11.76 | 4668 | 6.31e3 | 1 | 1 | 86 | 89 |  |  | DB Search |
| M.RCI.C | N | 20.6 | 390.2049 | 3 | -4.41 | 391.2095 | 1 | 8.08 | 3003 | 0 | 0 | 0 | 555 | 557 |  |  | DB Search |
| H.TFR.K | N | 20.53 | 422.2278 | 3 | -9.2 | 423.2301 | 1 | 22.38 | 8244 | 2.98e1 | 1 | 1 | 2397 | 2399 |  |  | DB Search |
| A.VFST.C | N | 19.6 | 452.2271 | 4 | -3.7 | 453.2316 | 1 | 22.49 | 8270 | 1.33e3 | 1 | 1 | 2253 | 2256 |  |  | DB Search |
| L.LLQP.N | N | 19.13 | 469.29 | 4 | -8.42 | 470.2922 | 1 | 12.16 | 4756 | 6.43e3 | 1 | 1 | 585 | 588 |  |  | DB Search |
| N.SIDT.K | N | 19.03 | 434.2013 | 4 | -3.95 | 435.2057 | 1 | 15.10 | 5999 | 0 | 0 | 0 | 2340 | 2343 |  |  | DB Search |
| R.TLGV.K | N | 18.91 | 388.2322 | 4 | -5.15 | 389.2365 | 1 | 17.34 | 6852 | 1.43e3 | 1 | 1 | 960 | 963 |  |  | DB Search |
| Q.LPTLP.P | N | 18.9 | 539.3319 | 5 | -4.16 | 540.3356 | 1 | 28.83 | 10048 | 0 | 0 | 0 | 88 | 92 |  |  | DB Search |
| T.KHT.A | N | 18.78 | 384.2121 | 3 | -3.61 | 385.217 | 1 | 6.37 | 2316 | 2.15e3 | 1 | 1 | 135 | 137 |  |  | DB Search |
| I.SPSI.L | N | 18.22 | 402.2114 | 4 | -3.42 | 403.2163 | 1 | 12.42 | 4855 | 2.18e3 | 1 | 1 | 1761 | 1764 |  |  | DB Search |
| S.FPPQ.N | N | 18.18 | 487.2431 | 4 | -6.62 | 488.2459 | 1 | 11.19 | 4443 | 2.22e3 | 1 | 1 | 11 | 14 |  |  | DB Search |
| D.TVVS.G | N | 18.11 | 404.2271 | 4 | -7.08 | 405.2305 | 1 | 8.17 | 3091 | 7.47e3 | 1 | 1 | 1201 | 1204 |  |  | DB Search |
| L.ESW.L | N | 17.43 | 420.1645 | 3 | -0.16 | 421.1707 | 1 | 11.21 | 4460 | 0 | 0 | 0 | 879 | 881 |  |  | DB Search |
| L.Q(-17.03)PII.M | N | 17.35 | 452.2635 | 4 | -6.01 | 453.2669 | 1 | 32.30 | 10953 | 1.74e3 | 1 | 1 | 2006 | 2009 | Pyro-glu from Q | Q1:Pyro-glu from Q:1000 | DB Search |
| L.WFR.V | N | 17.26 | 507.2594 | 3 | 7.35 | 508.2691 | 1 | 12.65 | 5011 | 0 | 0 | 0 | 1064 | 1066 |  |  | DB Search |
| L.HLCL.D | N | 16.17 | 484.2468 | 4 | 0.35 | 485.253 | 1 | 13.46 | 5287 | 1.1e4 | 1 | 1 | 194 | 197 |  |  | DB Search |
| C.RLC.W | N | 16.16 | 390.2049 | 3 | 4.43 | 391.213 | 1 | 3.37 | 1421 | 1.01e3 | 1 | 1 | 559 | 561 |  |  | DB Search |
| T.SSITT.G | N | 15.84 | 507.254 | 5 | -2.92 | 508.2586 | 1 | 14.59 | 5688 | 3.02e2 | 1 | 1 | 1235 | 1239 |  |  | DB Search |
| L.FRG.S | N | 15.51 | 378.2015 | 3 | -9.17 | 379.2044 | 1 | 3.37 | 1492 | 1.94e3 | 1 | 1 | 2436 | 2438 |  |  | DB Search |
| G.VGADL.I | N | 15.44 | 473.2485 | 5 | -0.97 | 474.2542 | 1 | 13.69 | 5320 | 4.72e3 | 1 | 1 | 2166 | 2170 |  |  | DB Search |
| E.KYPT.G | N | 15.36 | 507.2693 | 4 | -6.99 | 508.2718 | 1 | 16.69 | 6581 | 0 | 0 | 0 | 396 | 399 |  |  | DB Search |
| P.QNND.D | N | 15.34 | 489.1819 | 4 | 5.34 | 490.1906 | 1 | 2.90 | 1227 | 0 | 0 | 0 | 14 | 17 |  |  | DB Search |
| D.AYSV.V | N | 15.26 | 438.2114 | 4 | -3.08 | 439.2163 | 1 | 7.95 | 2920 | 3.53e4 | 1 | 1 | 2294 | 2297 |  |  | DB Search |
| K.QNRFSVNS.G | Y | 15.09 | 950.457 | 8 | 9.26 | 951.4707 | 1 | 10.29 | 4052 | 2.09e2 | 1 | 1 | 2509 | 2516 |  |  | DB Search |
| D.EFEG.N | N | 15.01 | 480.1856 | 4 | -5.04 | 481.1893 | 1 | 10.64 | 4144 | 0 | 0 | 0 | 942 | 945 |  |  | DB Search |
| K.AEVA.L | N | 15.01 | 388.1958 | 4 | 1.72 | 389.2028 | 1 | 3.34 | 1446 | 4.24e3 | 1 | 1 | 249 | 252 |  |  | DB Search |
| total 33 peptides |
| --- |

Best Unique PSM (Scan POS\_R15-1.wiff:8645, m/z=808.3937, z=1, RT=23.85, ppm=-1.36):


C4R150|C4R150\_KOMPG

back to list

  

| Protein Coverage
| Supporting Peptides
| Best Unique PSM
|

Protein Coverage:

Supporting Peptides:

| Peptide | Uniq | -10lgP | Mass | Length | ppm | m/z | z | RT | Scan | Area POS\_R15-1 | #Feature | #Feature POS\_R15-1 | Start | End | PTM | AScore | Found By |
| --- | --- | --- | --- | --- | --- | --- | --- | --- | --- | --- | --- | --- | --- | --- | --- | --- | --- |
| P.Q(-17.03)PQQPQ.P | N | 28.36 | 707.3239 | 6 | -2.97 | 708.3273 | 1 | 6.57 | 2541 | 0 | 0 | 0 | 337 | 342 | Pyro-glu from Q | Q1:Pyro-glu from Q:1000 | DB Search |
| K.ELLR.N | N | 25.05 | 529.3224 | 4 | -4.56 | 530.3259 | 1 | 17.34 | 6855 | 6.68e3 | 1 | 1 | 505 | 508 |  |  | DB Search |
| G.TSIA.N | N | 24.8 | 390.2114 | 4 | -5.32 | 391.2157 | 1 | 6.25 | 2416 | 1.07e4 | 1 | 1 | 169 | 172 |  |  | DB Search |
| P.Q(-17.03)PQQP.Q | N | 22.54 | 579.2653 | 5 | -5.46 | 580.2679 | 1 | 8.21 | 3095 | 8.92e3 | 1 | 1 | 337 | 341 | Pyro-glu from Q | Q1:Pyro-glu from Q:1000 | DB Search |
| A.TAY.Q | N | 19.3 | 353.1587 | 3 | -1.32 | 354.1646 | 1 | 12.51 | 4913 | 1.39e3 | 1 | 1 | 276 | 278 |  |  | DB Search |
| Q.PQPQ(+0.98)Q(+0.98)PQP.A | Y | 18.7 | 920.424 | 8 | 8.1 | 921.4364 | 1 | 26.73 | 9400 | 2.14e4 | 1 | 1 | 336 | 343 | Deamidation (NQ), Deamidation (NQ) | Q4:Deamidation (NQ):13.13 Q5:Deamidation (NQ):22.87 | DB Search |
| T.GSAS.S | N | 17.29 | 320.1332 | 4 | 7.75 | 321.1422 | 1 | 6.30 | 2464 | 0 | 0 | 0 | 305 | 308 |  |  | DB Search |
| L.ADEF.V | N | 16.65 | 480.1856 | 4 | 0.36 | 481.1919 | 1 | 10.67 | 4169 | 0 | 0 | 0 | 533 | 536 |  |  | DB Search |
| Q.Q(-17.03)PHQ.V | N | 16.34 | 491.2128 | 4 | 0.63 | 492.2192 | 1 | 2.44 | 1064 | 0 | 0 | 0 | 155 | 158 | Pyro-glu from Q | Q1:Pyro-glu from Q:1000 | DB Search |
| Q.Q(-17.03)PH.Q | N | 15.48 | 363.1543 | 3 | -0.14 | 364.1606 | 1 | 2.32 | 992 | 3.39e4 | 1 | 1 | 155 | 157 | Pyro-glu from Q | Q1:Pyro-glu from Q:1000 | DB Search |
| G.TPTGTP.V | Y | 15.05 | 572.2806 | 6 | 5.87 | 573.2898 | 1 | 21.48 | 7917 | 4.12e2 | 1 | 1 | 206 | 211 |  |  | DB Search |
| total 11 peptides |
| --- |

Best Unique PSM (Scan POS\_R15-1.wiff:9400, m/z=921.4364, z=1, RT=26.73, ppm=5.62):


C4R801|C4R801\_KOMPG

back to list

  

| Protein Coverage
| Supporting Peptides
| Best Unique PSM
|

Protein Coverage:

Supporting Peptides:

| Peptide | Uniq | -10lgP | Mass | Length | ppm | m/z | z | RT | Scan | Area POS\_R15-1 | #Feature | #Feature POS\_R15-1 | Start | End | PTM | AScore | Found By |
| --- | --- | --- | --- | --- | --- | --- | --- | --- | --- | --- | --- | --- | --- | --- | --- | --- | --- |
| Y.KDIL.K | N | 22.06 | 487.3006 | 4 | -0.76 | 488.3063 | 1 | 10.37 | 4062 | 0 | 0 | 0 | 29 | 32 |  |  | DB Search |
| A.PAFV.Y | N | 19.17 | 432.2372 | 4 | -3.2 | 433.2421 | 1 | 24.76 | 8853 | 3.98e2 | 1 | 1 | 113 | 116 |  |  | DB Search |
| H.SVGI.L | N | 18.49 | 374.2165 | 4 | -4.96 | 375.221 | 1 | 14.62 | 5748 | 1.93e2 | 1 | 1 | 41 | 44 |  |  | DB Search |
| Q.TSN.P | N | 17.54 | 320.1332 | 3 | 3.34 | 321.1407 | 1 | 8.11 | 3024 | 0 | 0 | 0 | 71 | 73 |  |  | DB Search |
| I.SADQQP.P | Y | 15.96 | 644.2766 | 6 | 8.23 | 645.2875 | 1 | 30.75 | 10542 | 0 | 0 | 0 | 119 | 124 |  |  | DB Search |
| E.YHG.P | N | 15.26 | 375.1543 | 3 | -7.53 | 376.1578 | 1 | 4.73 | 1840 | 0 | 0 | 0 | 217 | 219 |  |  | DB Search |
| total 6 peptides |
| --- |

Best Unique PSM (Scan POS\_R15-1.wiff:10542, m/z=645.2875, z=1, RT=30.75, ppm=5.75):


C4R723|C4R723\_KOMPG

back to list

  

| Protein Coverage
| Supporting Peptides
| Best Unique PSM
|

Protein Coverage:

Supporting Peptides:

| Peptide | Uniq | -10lgP | Mass | Length | ppm | m/z | z | RT | Scan | Area POS\_R15-1 | #Feature | #Feature POS\_R15-1 | Start | End | PTM | AScore | Found By |
| --- | --- | --- | --- | --- | --- | --- | --- | --- | --- | --- | --- | --- | --- | --- | --- | --- | --- |
| A.GGGGI.G | N | 28.62 | 359.1805 | 5 | -3.01 | 360.1858 | 1 | 6.13 | 2409 | 0 | 0 | 0 | 786 | 790 |  |  | DB Search |
| G.GGGI.G | N | 27.46 | 302.159 | 4 | -3.72 | 303.1644 | 1 | 6.06 | 2387 | 3.14e3 | 1 | 1 | 787 | 790 |  |  | DB Search |
| F.SPLL.D | N | 26.66 | 428.2635 | 4 | -1.63 | 429.269 | 1 | 21.35 | 7905 | 0 | 0 | 0 | 297 | 300 |  |  | DB Search |
| M.VVNSP.R | Y | 23.94 | 514.2751 | 5 | -0.42 | 515.2809 | 1 | 9.29 | 3594 | 1.21e3 | 1 | 1 | 1802 | 1806 |  |  | DB Search |
| E.PVLV.N | N | 23.27 | 426.2842 | 4 | 4.41 | 427.2923 | 1 | 20.36 | 7664 | 0 | 0 | 0 | 393 | 396 |  |  | DB Search |
| K.YNP.S | N | 21.45 | 392.1696 | 3 | -3.75 | 393.1744 | 1 | 8.45 | 3226 | 1.39e2 | 1 | 1 | 1606 | 1608 |  |  | DB Search |
| T.FISLVP.E | N | 20.93 | 674.4003 | 6 | -6.34 | 675.4016 | 1 | 42.02 | 13315 | 6.45e2 | 1 | 1 | 564 | 569 |  |  | DB Search |
| T.AAVE.S | N | 20.66 | 388.1958 | 4 | -4.49 | 389.2003 | 1 | 2.65 | 1124 | 0 | 0 | 0 | 279 | 282 |  |  | DB Search |
| T.TFR.F | N | 20.53 | 422.2278 | 3 | -9.2 | 423.2301 | 1 | 22.38 | 8244 | 2.98e1 | 1 | 1 | 351 | 353 |  |  | DB Search |
| L.LPLP.K | N | 20.33 | 438.2842 | 4 | -7.82 | 439.287 | 1 | 27.42 | 9635 | 1.51e3 | 1 | 1 | 475 | 478 |  |  | DB Search |
| Y.KCA.G | N | 19.71 | 320.1518 | 3 | 2.66 | 321.1591 | 1 | 15.28 | 6053 | 3.59e2 | 1 | 1 | 893 | 895 |  |  | DB Search |
| K.EQW.K | N | 18.42 | 461.191 | 3 | -3.23 | 462.1957 | 1 | 11.54 | 4588 | 2.45e3 | 1 | 1 | 1283 | 1285 |  |  | DB Search |
| A.LDFS.P | N | 18.2 | 480.222 | 4 | -2.08 | 481.2271 | 1 | 16.69 | 6580 | 0 | 0 | 0 | 294 | 297 |  |  | DB Search |
| L.FHH.D | N | 18.2 | 439.1968 | 3 | 7.99 | 440.2065 | 1 | 9.67 | 3787 | 2e3 | 1 | 1 | 1136 | 1138 |  |  | DB Search |
| G.TVVS.A | N | 18.11 | 404.2271 | 4 | -7.08 | 405.2305 | 1 | 8.17 | 3091 | 7.47e3 | 1 | 1 | 998 | 1001 |  |  | DB Search |
| G.VPLVP.G | N | 17.97 | 523.337 | 5 | -6.37 | 524.3396 | 1 | 27.22 | 9508 | 8.88e3 | 1 | 1 | 753 | 757 |  |  | DB Search |
| F.LGQP.E | N | 17.62 | 413.2274 | 4 | -2.94 | 414.2325 | 1 | 10.81 | 4261 | 5.46e2 | 1 | 1 | 1224 | 1227 |  |  | DB Search |
| F.ATGL.V | N | 17.01 | 360.2009 | 4 | -0.18 | 361.2072 | 1 | 8.07 | 3002 | 1.41e4 | 1 | 1 | 129 | 132 |  |  | DB Search |
| Q.AVVA.Q | N | 16.9 | 358.2216 | 4 | -4.99 | 359.2262 | 1 | 6.95 | 2675 | 0 | 0 | 0 | 1354 | 1357 |  |  | DB Search |
| V.IGVT.G | N | 16.69 | 388.2322 | 4 | -3.81 | 389.237 | 1 | 9.87 | 3731 | 6.08e3 | 1 | 1 | 1142 | 1145 |  |  | DB Search |
| K.ADEF.S | N | 16.65 | 480.1856 | 4 | 0.36 | 481.1919 | 1 | 10.67 | 4169 | 0 | 0 | 0 | 1756 | 1759 |  |  | DB Search |
| L.LSAL.V | N | 16.63 | 402.2478 | 4 | -8.97 | 403.2505 | 1 | 9.42 | 3655 | 1.65e3 | 1 | 1 | 30 | 33 |  |  | DB Search |
| L.DAVT.R | N | 16.27 | 404.1907 | 4 | -1.99 | 405.1962 | 1 | 8.31 | 3091 | 8.02e2 | 1 | 1 | 1534 | 1537 |  |  | DB Search |
| I.TLNG.P | N | 15.96 | 403.2067 | 4 | -9.11 | 404.2093 | 1 | 5.03 | 2025 | 0 | 0 | 0 | 1128 | 1131 |  |  | DB Search |
| R.TFT.P | N | 15.92 | 367.1743 | 3 | -7.47 | 368.1779 | 1 | 8.54 | 3263 | 0 | 0 | 0 | 1611 | 1613 |  |  | DB Search |
| Q.WARP.D | Y | 15.78 | 528.2808 | 4 | 0.68 | 529.2872 | 1 | 26.15 | 9307 | 4.57e1 | 1 | 1 | 1305 | 1308 |  |  | DB Search |
| R.IGVP.P | N | 15.32 | 384.2372 | 4 | -3.68 | 385.2422 | 1 | 16.13 | 6408 | 0 | 0 | 0 | 1096 | 1099 |  |  | DB Search |
| total 27 peptides |
| --- |

Best Unique PSM (Scan POS\_R15-1.wiff:3594, m/z=515.2405, z=1, RT=9.32, ppm=-2.9):


C4R9D6|C4R9D6\_KOMPG

back to list

  

| Protein Coverage
| Supporting Peptides
| Best Unique PSM
|

Protein Coverage:

Supporting Peptides:

| Peptide | Uniq | -10lgP | Mass | Length | ppm | m/z | z | RT | Scan | Area POS\_R15-1 | #Feature | #Feature POS\_R15-1 | Start | End | PTM | AScore | Found By |
| --- | --- | --- | --- | --- | --- | --- | --- | --- | --- | --- | --- | --- | --- | --- | --- | --- | --- |
| T.HFT.K | N | 23.64 | 403.1856 | 3 | -0.83 | 404.1915 | 1 | 13.94 | 5543 | 0 | 0 | 0 | 118 | 120 |  |  | DB Search |
| P.TSG.S | N | 21.44 | 263.1117 | 3 | 2.31 | 264.119 | 1 | 3.71 | 1537 | 5.72e3 | 1 | 1 | 57 | 59 |  |  | DB Search |
| T.GGHF.N | N | 18.9 | 416.1808 | 4 | -1.24 | 417.1865 | 1 | 6.38 | 2309 | 2.04e4 | 1 | 1 | 81 | 84 |  |  | DB Search |
| A.NFPAGGS.Y | Y | 17.8 | 648.2867 | 7 | 5.98 | 649.2963 | 1 | 9.50 | 3509 | 1.35e3 | 1 | 1 | 24 | 30 |  |  | DB Search |
| L.TLNG.L | N | 15.96 | 403.2067 | 4 | -9.11 | 404.2093 | 1 | 5.03 | 2025 | 0 | 0 | 0 | 189 | 192 |  |  | DB Search |
| I.TFT.P | N | 15.92 | 367.1743 | 3 | -7.47 | 368.1779 | 1 | 8.54 | 3263 | 0 | 0 | 0 | 38 | 40 |  |  | DB Search |
| total 6 peptides |
| --- |

Best Unique PSM (Scan POS\_R15-1.wiff:3509, m/z=649.2963, z=1, RT=9.50, ppm=3.5):


C4R6F1|C4R6F1\_KOMPG

back to list

  

| Protein Coverage
| Supporting Peptides
| Best Unique PSM
|

Protein Coverage:

Supporting Peptides:

| Peptide | Uniq | -10lgP | Mass | Length | ppm | m/z | z | RT | Scan | Area POS\_R15-1 | #Feature | #Feature POS\_R15-1 | Start | End | PTM | AScore | Found By |
| --- | --- | --- | --- | --- | --- | --- | --- | --- | --- | --- | --- | --- | --- | --- | --- | --- | --- |
| V.YSIF.S | N | 26.31 | 528.2584 | 4 | -2.91 | 529.2628 | 1 | 34.97 | 11598 | 3.74e3 | 1 | 1 | 9 | 12 |  |  | DB Search |
| F.G(+42.01)KILVYSIF.S | Y | 23.27 | 1080.6219 | 9 | -1.11 | 541.3163 | 2 | 43.88 | 13638 | 1.72e3 | 1 | 1 | 4 | 12 | Acetylation (Protein N-term) | G1:Acetylation (Protein N-term):1000 | DB Search |
| C.SNG.R | N | 19.07 | 276.107 | 3 | 1.61 | 277.114 | 1 | 16.82 | 6604 | 0 | 0 | 0 | 107 | 109 |  |  | DB Search |
| S.HWP.S | N | 16.98 | 438.2015 | 3 | 8.65 | 439.2115 | 1 | 8.79 | 3374 | 2.52e3 | 1 | 1 | 94 | 96 |  |  | DB Search |
| T.LGGV.V | N | 15.94 | 344.2059 | 4 | 0.19 | 345.2124 | 1 | 5.48 | 2143 | 3.05e3 | 1 | 1 | 17 | 20 |  |  | DB Search |
| total 5 peptides |
| --- |

Best Unique PSM (Scan POS\_R15-1.wiff:13638, m/z=541.3163, z=2, RT=43.88, ppm=-3.6):


C4R8B9|C4R8B9\_KOMPG

back to list

  

| Protein Coverage
| Supporting Peptides
| Best Unique PSM
|

Protein Coverage:

Supporting Peptides:

| Peptide | Uniq | -10lgP | Mass | Length | ppm | m/z | z | RT | Scan | Area POS\_R15-1 | #Feature | #Feature POS\_R15-1 | Start | End | PTM | AScore | Found By |
| --- | --- | --- | --- | --- | --- | --- | --- | --- | --- | --- | --- | --- | --- | --- | --- | --- | --- |
| L.AFTP.V | N | 25.4 | 434.2165 | 4 | -5.48 | 435.2203 | 1 | 13.12 | 5200 | 6.05e3 | 1 | 1 | 381 | 384 |  |  | DB Search |
| T.TSIA.L | N | 24.8 | 390.2114 | 4 | -5.32 | 391.2157 | 1 | 6.25 | 2416 | 1.07e4 | 1 | 1 | 209 | 212 |  |  | DB Search |
| F.VAAP.T | N | 23.43 | 356.2059 | 4 | -1.11 | 357.2119 | 1 | 7.38 | 2801 | 2.39e3 | 1 | 1 | 255 | 258 |  |  | DB Search |
| H.EW.N | N | 22.33 | 333.1325 | 2 | -3.27 | 334.1378 | 1 | 12.76 | 5036 | 2.08e4 | 1 | 1 | 45 | 46 |  |  | DB Search |
| G.VTVP.D | N | 22.26 | 414.2478 | 4 | -0.09 | 415.254 | 1 | 17.14 | 6680 | 3.72e3 | 1 | 1 | 439 | 442 |  |  | DB Search |
| T.SIAL.S | N | 21.98 | 402.2478 | 4 | -2.21 | 403.2532 | 1 | 20.49 | 7711 | 0 | 0 | 0 | 210 | 213 |  |  | DB Search |
| R.VGSL.S | N | 21.91 | 374.2165 | 4 | 0.34 | 375.223 | 1 | 13.82 | 5444 | 2.07e3 | 1 | 1 | 182 | 185 |  |  | DB Search |
| N.NTS.R | N | 21.44 | 320.1332 | 3 | 8.58 | 321.1424 | 1 | 6.16 | 2421 | 0 | 0 | 0 | 172 | 174 |  |  | DB Search |
| N.TSG.S | N | 21.44 | 263.1117 | 3 | 2.31 | 264.119 | 1 | 3.71 | 1537 | 5.72e3 | 1 | 1 | 480 | 482 |  |  | DB Search |
| G.LAR.G | N | 20.74 | 358.2328 | 3 | -3.48 | 359.238 | 1 | 2.08 | 887 | 2.98e3 | 1 | 1 | 435 | 437 |  |  | DB Search |
| R.NSG.L | N | 20.42 | 276.107 | 3 | 6.48 | 277.1154 | 1 | 16.74 | 6592 | 0 | 0 | 0 | 377 | 379 |  |  | DB Search |
| G.RPD.L | N | 19.79 | 386.1914 | 3 | -3.61 | 387.1963 | 1 | 2.95 | 1225 | 1.33e2 | 1 | 1 | 430 | 432 |  |  | DB Search |
| L.PH.I | N | 19.52 | 252.1222 | 2 | -5.45 | 253.1275 | 1 | 5.57 | 2095 | 1.25e4 | 1 | 1 | 15 | 16 |  |  | DB Search |
| P.SLDT.V | N | 19.03 | 434.2013 | 4 | -3.95 | 435.2057 | 1 | 15.10 | 5999 | 0 | 0 | 0 | 238 | 241 |  |  | DB Search |
| Q.HAVI.E | N | 18.78 | 438.259 | 4 | -6.35 | 439.2625 | 1 | 8.70 | 3348 | 6.21e2 | 1 | 1 | 28 | 31 |  |  | DB Search |
| H.HP.E | N | 17.77 | 252.1222 | 2 | -2.49 | 253.1283 | 1 | 5.54 | 2216 | 1.25e4 | 1 | 1 | 70 | 71 |  |  | DB Search |
| Q.RSF.S | N | 17.5 | 408.2121 | 3 | -1.76 | 409.2177 | 1 | 9.17 | 3526 | 0 | 0 | 0 | 487 | 489 |  |  | DB Search |
| Q.HAAGPVPES.I | Y | 16 | 863.4137 | 9 | 1.97 | 864.4205 | 1 | 11.04 | 4370 | 1.87e3 | 1 | 1 | 544 | 552 |  |  | DB Search |
| H.AVIE.L | N | 15.33 | 430.2427 | 4 | -8.59 | 431.2452 | 1 | 9.60 | 3778 | 8.62e3 | 1 | 1 | 29 | 32 |  |  | DB Search |
| total 19 peptides |
| --- |

Best Unique PSM (Scan POS\_R15-1.wiff:4370, m/z=864.4197, z=1, RT=11.07, ppm=-0.51):


C4R937|C4R937\_KOMPG

back to list

  

| Protein Coverage
| Supporting Peptides
| Best Unique PSM
|

Protein Coverage:

Supporting Peptides:

| Peptide | Uniq | -10lgP | Mass | Length | ppm | m/z | z | RT | Scan | Area POS\_R15-1 | #Feature | #Feature POS\_R15-1 | Start | End | PTM | AScore | Found By |
| --- | --- | --- | --- | --- | --- | --- | --- | --- | --- | --- | --- | --- | --- | --- | --- | --- | --- |
| E.GFDTP.T | Y | 19.32 | 535.2278 | 5 | -5.91 | 536.2306 | 1 | 13.65 | 5375 | 0 | 0 | 0 | 103 | 107 |  |  | DB Search |
| total 1 peptides |
| --- |

Best Unique PSM (Scan POS\_R15-1.wiff:5375, m/z=536.2306, z=1, RT=13.65, ppm=-8.4):


C4QXQ4|C4QXQ4\_KOMPG

back to list

  

| Protein Coverage
| Supporting Peptides
| Best Unique PSM
|

Protein Coverage:

Supporting Peptides:

| Peptide | Uniq | -10lgP | Mass | Length | ppm | m/z | z | RT | Scan | Area POS\_R15-1 | #Feature | #Feature POS\_R15-1 | Start | End | PTM | AScore | Found By |
| --- | --- | --- | --- | --- | --- | --- | --- | --- | --- | --- | --- | --- | --- | --- | --- | --- | --- |
| I.VGGI.A | N | 24.87 | 344.2059 | 4 | -5.22 | 345.2106 | 1 | 10.74 | 4195 | 1.51e3 | 1 | 1 | 451 | 454 |  |  | DB Search |
| A.RLVP.S | N | 24.65 | 483.3169 | 4 | -4.88 | 484.3206 | 1 | 10.06 | 3922 | 3.47e3 | 1 | 1 | 445 | 448 |  |  | DB Search |
| T.LSGL.F | N | 22.6 | 388.2322 | 4 | -3.81 | 389.237 | 1 | 9.87 | 3804 | 6.08e3 | 1 | 1 | 480 | 483 |  |  | DB Search |
| V.ATAQ(+0.98)DIQT.I | Y | 22.46 | 847.3923 | 8 | -4.5 | 848.3937 | 1 | 25.46 | 9101 | 1.31e2 | 1 | 1 | 219 | 226 | Deamidation (NQ) | Q4:Deamidation (NQ):29.32 | DB Search |
| L.YGR.K | N | 21.6 | 394.1965 | 3 | -4.25 | 395.2011 | 1 | 5.35 | 1961 | 1.67e4 | 1 | 1 | 198 | 200 |  |  | DB Search |
| E.THH.P | N | 21.55 | 393.1761 | 3 | -4.54 | 394.1806 | 1 | 2.11 | 908 | 2.12e3 | 1 | 1 | 314 | 316 |  |  | DB Search |
| F.VSSL.G | N | 20.48 | 404.2271 | 4 | -7.08 | 405.2305 | 1 | 8.17 | 2993 | 7.47e3 | 1 | 1 | 206 | 209 |  |  | DB Search |
| L.IPLP.I | N | 20.33 | 438.2842 | 4 | -7.82 | 439.287 | 1 | 27.42 | 9635 | 1.51e3 | 1 | 1 | 554 | 557 |  |  | DB Search |
| L.TAY.P | N | 19.3 | 353.1587 | 3 | -1.32 | 354.1646 | 1 | 12.51 | 4913 | 1.39e3 | 1 | 1 | 387 | 389 |  |  | DB Search |
| N.KGY.H | N | 19.27 | 366.1903 | 3 | -9.39 | 367.1932 | 1 | 6.66 | 2561 | 3.55e1 | 1 | 1 | 395 | 397 |  |  | DB Search |
| total 10 peptides |
| --- |

Best Unique PSM (Scan POS\_R15-1.wiff:9101, m/z=848.3937, z=1, RT=25.46, ppm=-6.98):


C4R6N4|C4R6N4\_KOMPG

back to list

  

| Protein Coverage
| Supporting Peptides
| Best Unique PSM
|

Protein Coverage:

Supporting Peptides:

| Peptide | Uniq | -10lgP | Mass | Length | ppm | m/z | z | RT | Scan | Area POS\_R15-1 | #Feature | #Feature POS\_R15-1 | Start | End | PTM | AScore | Found By |
| --- | --- | --- | --- | --- | --- | --- | --- | --- | --- | --- | --- | --- | --- | --- | --- | --- | --- |
| I.NLI.D | N | 28.22 | 358.2216 | 3 | -4.68 | 359.2263 | 1 | 19.18 | 7326 | 4.19e3 | 1 | 1 | 88 | 90 |  |  | DB Search |
| Q.VGGP.V | N | 23.36 | 328.1746 | 4 | -1.91 | 329.1805 | 1 | 5.92 | 2159 | 4.71e4 | 1 | 1 | 639 | 642 |  |  | DB Search |
| N.PGE.A | N | 21.28 | 301.1274 | 3 | 1.88 | 302.1345 | 1 | 2.04 | 869 | 2.57e3 | 1 | 1 | 309 | 311 |  |  | DB Search |
| Q.IAR.F | N | 20.74 | 358.2328 | 3 | -3.48 | 359.238 | 1 | 2.08 | 887 | 2.98e3 | 1 | 1 | 5 | 7 |  |  | DB Search |
| N.IPLP.Q | N | 20.33 | 438.2842 | 4 | -7.82 | 439.287 | 1 | 27.42 | 9635 | 1.51e3 | 1 | 1 | 748 | 751 |  |  | DB Search |
| G.VVDI.F | N | 19.9 | 444.2584 | 4 | 0.77 | 445.2649 | 1 | 17.32 | 6776 | 2.15e3 | 1 | 1 | 191 | 194 |  |  | DB Search |
| K.PH.D | N | 19.52 | 252.1222 | 2 | -5.45 | 253.1275 | 1 | 5.57 | 2095 | 1.25e4 | 1 | 1 | 397 | 398 |  |  | DB Search |
| Y.SGIL.K | N | 19.42 | 388.2322 | 4 | -5.15 | 389.2365 | 1 | 17.34 | 6766 | 1.43e3 | 1 | 1 | 366 | 369 |  |  | DB Search |
| A.SLQVGGP.V | Y | 19.05 | 656.3493 | 7 | -6.58 | 657.3506 | 1 | 5.97 | 2321 | 6.84e2 | 1 | 1 | 636 | 642 |  |  | DB Search |
| M.EIG.K | N | 18.95 | 317.1587 | 3 | -4.31 | 318.1638 | 1 | 5.49 | 2177 | 4.11e3 | 1 | 1 | 523 | 525 |  |  | DB Search |
| K.AITP.L | N | 18.91 | 400.2322 | 4 | -8.5 | 401.235 | 1 | 9.38 | 3619 | 0 | 0 | 0 | 774 | 777 |  |  | DB Search |
| S.LRV.L | N | 18.9 | 386.2641 | 3 | -1.28 | 387.27 | 1 | 27.77 | 9760 | 0 | 0 | 0 | 106 | 108 |  |  | DB Search |
| A.ITP.L | N | 17.99 | 329.1951 | 3 | 1.36 | 330.202 | 1 | 7.49 | 2815 | 1.47e3 | 1 | 1 | 775 | 777 |  |  | DB Search |
| T.HP.T | N | 17.77 | 252.1222 | 2 | -2.49 | 253.1283 | 1 | 5.54 | 2216 | 1.25e4 | 1 | 1 | 353 | 354 |  |  | DB Search |
| D.AVAG.V | N | 17.64 | 316.1746 | 4 | -4.4 | 317.1797 | 1 | 5.33 | 2131 | 0 | 0 | 0 | 118 | 121 |  |  | DB Search |
| A.GDR.R | N | 17.49 | 346.1601 | 3 | -0.12 | 347.1664 | 1 | 10.73 | 4173 | 1.09e2 | 1 | 1 | 467 | 469 |  |  | DB Search |
| P.ADL.A | N | 17.27 | 317.1587 | 3 | -4.79 | 318.1636 | 1 | 3.30 | 1336 | 3.11e3 | 1 | 1 | 755 | 757 |  |  | DB Search |
| R.AAQ.V | N | 16.83 | 288.1434 | 3 | 8 | 289.1522 | 1 | 5.91 | 2315 | 6.8e3 | 1 | 1 | 720 | 722 |  |  | DB Search |
| M.E(-18.01)PI.M | N | 16.1 | 339.1794 | 3 | -5.62 | 340.1839 | 1 | 16.05 | 6351 | 1.63e4 | 1 | 1 | 694 | 696 | Pyro-glu from E | E1:Pyro-glu from E:1000 | DB Search |
| I.SINVE.N | N | 15.86 | 560.2806 | 5 | -2.77 | 561.2849 | 1 | 10.15 | 3966 | 0 | 0 | 0 | 412 | 416 |  |  | DB Search |
| S.GM(+15.99)GELHLD.I | Y | 15.54 | 886.3854 | 8 | -3.53 | 887.3874 | 1 | 19.91 | 7502 | 3.41e2 | 1 | 1 | 501 | 508 | Oxidation (M) | M2:Oxidation (M):1000 | DB Search |
| V.Q(-17.03)PL.M | N | 15.26 | 339.1794 | 3 | -4.38 | 340.1844 | 1 | 15.14 | 5814 | 1.63e4 | 1 | 1 | 296 | 298 | Pyro-glu from Q | Q1:Pyro-glu from Q:1000 | DB Search |
| total 22 peptides |
| --- |

Best Unique PSM (Scan POS\_R15-1.wiff:2321, m/z=657.3506, z=1, RT=5.97, ppm=-9.06):


C4R8H2|C4R8H2\_KOMPG

back to list

  

| Protein Coverage
| Supporting Peptides
| Best Unique PSM
|

Protein Coverage:

Supporting Peptides:

| Peptide | Uniq | -10lgP | Mass | Length | ppm | m/z | z | RT | Scan | Area POS\_R15-1 | #Feature | #Feature POS\_R15-1 | Start | End | PTM | AScore | Found By |
| --- | --- | --- | --- | --- | --- | --- | --- | --- | --- | --- | --- | --- | --- | --- | --- | --- | --- |
| R.LFSP.S | N | 26.07 | 462.2478 | 4 | -0.35 | 463.2538 | 1 | 20.06 | 7534 | 6.05e2 | 1 | 1 | 49 | 52 |  |  | DB Search |
| A.LSGL.L | N | 22.6 | 388.2322 | 4 | -3.81 | 389.237 | 1 | 9.87 | 3804 | 6.08e3 | 1 | 1 | 98 | 101 |  |  | DB Search |
| D.YGR.A | N | 21.6 | 394.1965 | 3 | -4.25 | 395.2011 | 1 | 5.35 | 1961 | 1.67e4 | 1 | 1 | 398 | 400 |  |  | DB Search |
| N.ISVGL.S | N | 20.08 | 487.3006 | 5 | -4.27 | 488.3046 | 1 | 21.68 | 7886 | 8.06e3 | 1 | 1 | 300 | 304 |  |  | DB Search |
| I.SVGL.S | N | 18.49 | 374.2165 | 4 | -4.96 | 375.221 | 1 | 14.62 | 5748 | 1.93e2 | 1 | 1 | 301 | 304 |  |  | DB Search |
| Q.LSAL.S | N | 16.63 | 402.2478 | 4 | -8.97 | 403.2505 | 1 | 9.42 | 3655 | 1.65e3 | 1 | 1 | 95 | 98 |  |  | DB Search |
| R.MAPNYTA.Q | Y | 16.39 | 766.332 | 7 | 8.84 | 767.3441 | 1 | 25.11 | 9020 | 5.8e1 | 1 | 1 | 59 | 65 |  |  | DB Search |
| P.AQLE.P | N | 16.19 | 459.2329 | 4 | -1.44 | 460.2384 | 1 | 4.72 | 1830 | 1.97e3 | 1 | 1 | 120 | 123 |  |  | DB Search |
| total 8 peptides |
| --- |

Best Unique PSM (Scan POS\_R15-1.wiff:9020, m/z=767.3563, z=1, RT=25.14, ppm=6.36):


C4R3K9|C4R3K9\_KOMPG

back to list

  

| Protein Coverage
| Supporting Peptides
| Best Unique PSM
|

Protein Coverage:

Supporting Peptides:

| Peptide | Uniq | -10lgP | Mass | Length | ppm | m/z | z | RT | Scan | Area POS\_R15-1 | #Feature | #Feature POS\_R15-1 | Start | End | PTM | AScore | Found By |
| --- | --- | --- | --- | --- | --- | --- | --- | --- | --- | --- | --- | --- | --- | --- | --- | --- | --- |
| I.LLLP.P | N | 24.07 | 454.3155 | 4 | -2.4 | 455.3206 | 1 | 34.52 | 11565 | 2.64e3 | 1 | 1 | 551 | 554 |  |  | DB Search |
| S.VTTL.M | N | 20.12 | 432.2584 | 4 | -4.38 | 433.2627 | 1 | 11.15 | 4417 | 0 | 0 | 0 | 623 | 626 |  |  | DB Search |
| V.LSVP.E | N | 19.74 | 414.2478 | 4 | -0.09 | 415.254 | 1 | 17.14 | 6914 | 3.72e3 | 1 | 1 | 569 | 572 |  |  | DB Search |
| P.PVLSVP.E | Y | 19.43 | 610.369 | 6 | -0.89 | 611.3742 | 1 | 27.21 | 9600 | 1.8e3 | 1 | 1 | 567 | 572 |  |  | DB Search |
| F.KHT.N | N | 18.78 | 384.2121 | 3 | -3.61 | 385.217 | 1 | 6.37 | 2316 | 2.15e3 | 1 | 1 | 79 | 81 |  |  | DB Search |
| S.GSSL.P | N | 18.21 | 362.1801 | 4 | -9.58 | 363.183 | 1 | 6.30 | 2465 | 1.38e3 | 1 | 1 | 598 | 601 |  |  | DB Search |
| P.QPYP.Q | N | 17.73 | 503.238 | 4 | 1.89 | 504.245 | 1 | 15.02 | 5950 | 0 | 0 | 0 | 577 | 580 |  |  | DB Search |
| G.AAQ.V | N | 16.83 | 288.1434 | 3 | 8 | 289.1522 | 1 | 5.91 | 2315 | 6.8e3 | 1 | 1 | 205 | 207 |  |  | DB Search |
| N.NGST.L | N | 16.67 | 377.1547 | 4 | -5.39 | 378.159 | 1 | 16.22 | 6455 | 6.96e2 | 1 | 1 | 109 | 112 |  |  | DB Search |
| N.TFT.P | N | 15.92 | 367.1743 | 3 | -7.47 | 368.1779 | 1 | 8.54 | 3263 | 0 | 0 | 0 | 187 | 189 |  |  | DB Search |
| P.MIQP.V | N | 15.3 | 487.2464 | 4 | 3.36 | 488.2542 | 1 | 15.67 | 6182 | 1.26e3 | 1 | 1 | 413 | 416 |  |  | DB Search |
| Q.Q(-17.03)PF.Q | N | 15.22 | 373.1638 | 3 | -0.75 | 374.1698 | 1 | 21.41 | 7929 | 8.45e4 | 1 | 1 | 442 | 444 | Pyro-glu from Q | Q1:Pyro-glu from Q:1000 | DB Search |
| total 12 peptides |
| --- |

Best Unique PSM (Scan POS\_R15-1.wiff:9600, m/z=611.3707, z=1, RT=27.30, ppm=-3.37):


C4R609|C4R609\_KOMPG

back to list

  

| Protein Coverage
| Supporting Peptides
| Best Unique PSM
|

Protein Coverage:

Supporting Peptides:

| Peptide | Uniq | -10lgP | Mass | Length | ppm | m/z | z | RT | Scan | Area POS\_R15-1 | #Feature | #Feature POS\_R15-1 | Start | End | PTM | AScore | Found By |
| --- | --- | --- | --- | --- | --- | --- | --- | --- | --- | --- | --- | --- | --- | --- | --- | --- | --- |
| P.LPPP.P | N | 27.73 | 422.2529 | 4 | -1.45 | 423.2585 | 1 | 9.57 | 3721 | 1.63e3 | 1 | 1 | 121 | 124 |  |  | DB Search |
| F.VLGS.N | N | 24.2 | 374.2165 | 4 | -4.06 | 375.2213 | 1 | 5.20 | 2012 | 6.27e3 | 1 | 1 | 318 | 321 |  |  | DB Search |
| S.NFSP.V | N | 19.33 | 463.2067 | 4 | -2.34 | 464.2117 | 1 | 8.58 | 3287 | 2.64e3 | 1 | 1 | 322 | 325 |  |  | DB Search |
| D.KGY.Y | N | 19.27 | 366.1903 | 3 | -9.39 | 367.1932 | 1 | 6.66 | 2561 | 3.55e1 | 1 | 1 | 422 | 424 |  |  | DB Search |
| T.AHFG.G | N | 18.82 | 430.1964 | 4 | 8.04 | 431.2061 | 1 | 11.26 | 4416 | 1.31e3 | 1 | 1 | 355 | 358 |  |  | DB Search |
| G.LDNF.A | N | 18.37 | 507.2329 | 4 | -5.34 | 508.2362 | 1 | 14.74 | 5819 | 0 | 0 | 0 | 393 | 396 |  |  | DB Search |
| M.N(+42.01)PNIPP.V | Y | 16.82 | 692.3493 | 6 | -2.24 | 693.3533 | 1 | 17.40 | 6779 | 3.63e3 | 1 | 1 | 2 | 7 | Acetylation (Protein N-term) | N1:Acetylation (Protein N-term):1000 | DB Search |
| G.ASGALLGVMAVNI.L | Y | 15.98 | 1214.6693 | 13 | 4.47 | 1215.679 | 1 | 44.31 | 13727 | 1.85e3 | 1 | 1 | 333 | 345 |  |  | DB Search |
| T.NPSGESQ.Q | Y | 15.58 | 717.2929 | 7 | 8.97 | 718.3049 | 1 | 15.48 | 6132 | 0 | 0 | 0 | 105 | 111 |  |  | DB Search |
| total 9 peptides |
| --- |

Best Unique PSM (Scan POS\_R15-1.wiff:6779, m/z=693.3533, z=1, RT=17.40, ppm=-4.72):


C4R6B2|C4R6B2\_KOMPG

back to list

  

| Protein Coverage
| Supporting Peptides
| Best Unique PSM
|

Protein Coverage:

Supporting Peptides:

| Peptide | Uniq | -10lgP | Mass | Length | ppm | m/z | z | RT | Scan | Area POS\_R15-1 | #Feature | #Feature POS\_R15-1 | Start | End | PTM | AScore | Found By |
| --- | --- | --- | --- | --- | --- | --- | --- | --- | --- | --- | --- | --- | --- | --- | --- | --- | --- |
| L.NGI.G | N | 28.25 | 302.159 | 3 | -3.76 | 303.1644 | 1 | 6.06 | 2374 | 3.14e3 | 1 | 1 | 147 | 149 |  |  | DB Search |
| L.E(-18.01)QPPYP.S | Y | 26.29 | 711.3228 | 6 | -3.74 | 712.3256 | 1 | 17.49 | 6909 | 4.48e3 | 1 | 1 | 837 | 842 | Pyro-glu from E | E1:Pyro-glu from E:1000 | DB Search |
| Q.EIIR.K | N | 25.05 | 529.3224 | 4 | -4.56 | 530.3259 | 1 | 17.34 | 6855 | 6.68e3 | 1 | 1 | 82 | 85 |  |  | DB Search |
| Y.ALLSL.G | N | 24.46 | 515.3319 | 5 | -6.84 | 516.3344 | 1 | 37.01 | 12185 | 8.65e2 | 1 | 1 | 629 | 633 |  |  | DB Search |
| A.LSGL.N | N | 22.6 | 388.2322 | 4 | -3.81 | 389.237 | 1 | 9.87 | 3804 | 6.08e3 | 1 | 1 | 820 | 823 |  |  | DB Search |
| E.EW.N | N | 22.33 | 333.1325 | 2 | -3.27 | 334.1378 | 1 | 12.76 | 5036 | 2.08e4 | 1 | 1 | 924 | 925 |  |  | DB Search |
| L.EIF.K | N | 21.7 | 407.2056 | 3 | -8.99 | 408.2082 | 1 | 29.23 | 10157 | 9.42e2 | 1 | 1 | 698 | 700 |  |  | DB Search |
| R.YGR.K | N | 21.6 | 394.1965 | 3 | -4.25 | 395.2011 | 1 | 5.35 | 1961 | 1.67e4 | 1 | 1 | 600 | 602 |  |  | DB Search |
| W.LAR.V | N | 20.74 | 358.2328 | 3 | -3.48 | 359.238 | 1 | 2.08 | 887 | 2.98e3 | 1 | 1 | 339 | 341 |  |  | DB Search |
| Q.SSD.F | N | 20.1 | 307.1016 | 3 | 8.02 | 308.1105 | 1 | 10.67 | 4164 | 0 | 0 | 0 | 497 | 499 |  |  | DB Search |
| A.RPD.L | N | 19.79 | 386.1914 | 3 | -3.61 | 387.1963 | 1 | 2.95 | 1225 | 1.33e2 | 1 | 1 | 60 | 62 |  |  | DB Search |
| D.SGLI.L | N | 19.42 | 388.2322 | 4 | -5.15 | 389.2365 | 1 | 17.34 | 6766 | 1.43e3 | 1 | 1 | 142 | 145 |  |  | DB Search |
| D.FPS.D | N | 19.27 | 349.1638 | 3 | -4.81 | 350.1685 | 1 | 16.32 | 6461 | 1.41e3 | 1 | 1 | 583 | 585 |  |  | DB Search |
| P.EAY.L | N | 19.24 | 381.1536 | 3 | -5.14 | 382.158 | 1 | 5.09 | 2057 | 2.63e3 | 1 | 1 | 444 | 446 |  |  | DB Search |
| K.SNG.N | N | 19.07 | 276.107 | 3 | 1.61 | 277.114 | 1 | 16.82 | 6604 | 0 | 0 | 0 | 568 | 570 |  |  | DB Search |
| R.SFY.G | N | 18.1 | 415.1743 | 3 | 6.32 | 416.1832 | 1 | 8.32 | 3151 | 0 | 0 | 0 | 591 | 593 |  |  | DB Search |
| V.HP.Q | N | 17.77 | 252.1222 | 2 | -2.49 | 253.1283 | 1 | 5.54 | 2216 | 1.25e4 | 1 | 1 | 244 | 245 |  |  | DB Search |
| S.QALE.L | N | 17.59 | 459.2329 | 4 | -5.43 | 460.2365 | 1 | 4.85 | 1909 | 7.42e3 | 2 | 2 | 197 | 200 |  |  | DB Search |
| Y.TSN.K | N | 17.54 | 320.1332 | 3 | 3.34 | 321.1407 | 1 | 8.11 | 3024 | 0 | 0 | 0 | 554 | 556 |  |  | DB Search |
| V.RSF.Y | N | 17.5 | 408.2121 | 3 | -1.76 | 409.2177 | 1 | 9.17 | 3526 | 0 | 0 | 0 | 590 | 592 |  |  | DB Search |
| R.AAQ.F | N | 16.83 | 288.1434 | 3 | 8 | 289.1522 | 1 | 5.91 | 2315 | 6.8e3 | 1 | 1 | 662 | 664 |  |  | DB Search |
| Y.LSAL.P | N | 16.63 | 402.2478 | 4 | -8.97 | 403.2505 | 1 | 9.42 | 3655 | 1.65e3 | 1 | 1 | 10 | 13 |  |  | DB Search |
| K.KST.N | N | 15.47 | 334.1852 | 3 | 0.84 | 335.192 | 1 | 54.68 | 16215 | 3.66e2 | 1 | 1 | 175 | 177 |  |  | DB Search |
| V.KSLP.N | N | 15.42 | 443.2744 | 4 | -3.64 | 444.2789 | 1 | 17.16 | 6753 | 0 | 0 | 0 | 804 | 807 |  |  | DB Search |
| G.NGV.L | N | 15.37 | 288.1434 | 3 | 8 | 289.1522 | 1 | 5.91 | 2301 | 6.8e3 | 1 | 1 | 151 | 153 |  |  | DB Search |
| total 25 peptides |
| --- |

Best Unique PSM (Scan POS\_R15-1.wiff:6909, m/z=712.3229, z=1, RT=17.54, ppm=-6.22):


C4R0G3|C4R0G3\_KOMPG

back to list

  

| Protein Coverage
| Supporting Peptides
| Best Unique PSM
|

Protein Coverage:

Supporting Peptides:

| Peptide | Uniq | -10lgP | Mass | Length | ppm | m/z | z | RT | Scan | Area POS\_R15-1 | #Feature | #Feature POS\_R15-1 | Start | End | PTM | AScore | Found By |
| --- | --- | --- | --- | --- | --- | --- | --- | --- | --- | --- | --- | --- | --- | --- | --- | --- | --- |
| T.PSLL.R | N | 29.95 | 428.2635 | 4 | -0.56 | 429.2694 | 1 | 21.28 | 7884 | 0 | 0 | 0 | 635 | 638 |  |  | DB Search |
| T.NGGL.F | N | 28.18 | 359.1805 | 4 | -1.08 | 360.1865 | 1 | 6.05 | 2378 | 0 | 0 | 0 | 670 | 673 |  |  | DB Search |
| K.LSLE.D | N | 25.68 | 460.2533 | 4 | -2.19 | 461.2584 | 1 | 13.54 | 5303 | 1.35e4 | 1 | 1 | 531 | 534 |  |  | DB Search |
| G.RLVP.V | N | 24.65 | 483.3169 | 4 | -4.88 | 484.3206 | 1 | 10.06 | 3922 | 3.47e3 | 1 | 1 | 913 | 916 |  |  | DB Search |
| D.AILSI.V | N | 24.46 | 515.3319 | 5 | -6.84 | 516.3344 | 1 | 37.01 | 12185 | 8.65e2 | 1 | 1 | 309 | 313 |  |  | DB Search |
| A.VAAP.K | N | 23.43 | 356.2059 | 4 | -1.11 | 357.2119 | 1 | 7.38 | 2801 | 2.39e3 | 1 | 1 | 526 | 529 |  |  | DB Search |
| N.VSIS.Q | N | 20.57 | 404.2271 | 4 | -7.08 | 405.2305 | 1 | 8.17 | 2961 | 7.47e3 | 1 | 1 | 1117 | 1120 |  |  | DB Search |
| S.TDVL.T | N | 19.75 | 446.2376 | 4 | -5.56 | 447.2413 | 1 | 13.64 | 5362 | 0 | 0 | 0 | 687 | 690 |  |  | DB Search |
| F.QFF.L | N | 19.56 | 440.206 | 3 | 3.92 | 441.2139 | 1 | 19.15 | 7313 | 0 | 0 | 0 | 601 | 603 |  |  | DB Search |
| E.RDR.L | N | 18.68 | 445.2397 | 3 | 8.14 | 446.2495 | 1 | 10.60 | 4131 | 1.44e2 | 1 | 1 | 455 | 457 |  |  | DB Search |
| I.GSSI.S | N | 18.21 | 362.1801 | 4 | -9.58 | 363.183 | 1 | 6.30 | 2465 | 1.38e3 | 1 | 1 | 407 | 410 |  |  | DB Search |
| P.ASFPP.P | Y | 18.06 | 517.2536 | 5 | -0.19 | 518.2595 | 1 | 21.82 | 8012 | 7.16e1 | 1 | 1 | 145 | 149 |  |  | DB Search |
| G.DVVY.I | N | 17.92 | 494.2376 | 4 | -7.07 | 495.2402 | 1 | 12.58 | 4967 | 1.85e3 | 1 | 1 | 361 | 364 |  |  | DB Search |
| G.STLA.S | N | 17.83 | 390.2114 | 4 | -5.32 | 391.2157 | 1 | 6.25 | 2360 | 1.07e4 | 1 | 1 | 401 | 404 |  |  | DB Search |
| D.TFDV.Y | N | 16.44 | 480.222 | 4 | -1.51 | 481.2274 | 1 | 11.18 | 4442 | 1.56e3 | 1 | 1 | 96 | 99 |  |  | DB Search |
| D.AEP.A | N | 15.46 | 315.143 | 3 | -3.02 | 316.1486 | 1 | 2.92 | 1198 | 1.54e3 | 1 | 1 | 165 | 167 |  |  | DB Search |
| T.NGV.K | N | 15.37 | 288.1434 | 3 | 8 | 289.1522 | 1 | 5.91 | 2301 | 6.8e3 | 1 | 1 | 520 | 522 |  |  | DB Search |
| total 17 peptides |
| --- |

Best Unique PSM (Scan POS\_R15-1.wiff:8012, m/z=518.2595, z=1, RT=21.82, ppm=-2.67):


C4R1C2|C4R1C2\_KOMPG

back to list

  

| Protein Coverage
| Supporting Peptides
| Best Unique PSM
|

Protein Coverage:

Supporting Peptides:

| Peptide | Uniq | -10lgP | Mass | Length | ppm | m/z | z | RT | Scan | Area POS\_R15-1 | #Feature | #Feature POS\_R15-1 | Start | End | PTM | AScore | Found By |
| --- | --- | --- | --- | --- | --- | --- | --- | --- | --- | --- | --- | --- | --- | --- | --- | --- | --- |
| A.PSLL.E | N | 29.95 | 428.2635 | 4 | -0.56 | 429.2694 | 1 | 21.28 | 7884 | 0 | 0 | 0 | 455 | 458 |  |  | DB Search |
| W.E(-18.01)VLFAGFG.N | Y | 23.4 | 820.4119 | 8 | -9.26 | 821.4095 | 1 | 47.32 | 14277 | 2.06e2 | 1 | 1 | 1335 | 1342 | Pyro-glu from E | E1:Pyro-glu from E:1000 | DB Search |
| D.LVAP.R | N | 22.5 | 398.2529 | 4 | -6.51 | 399.2566 | 1 | 9.50 | 3744 | 1.49e3 | 1 | 1 | 1263 | 1266 |  |  | DB Search |
| V.VLSG.A | N | 21.25 | 374.2165 | 4 | -4.06 | 375.2213 | 1 | 5.20 | 1991 | 6.27e3 | 1 | 1 | 202 | 205 |  |  | DB Search |
| A.VPVE.Q | N | 20.6 | 442.2427 | 4 | -5.67 | 443.2464 | 1 | 7.79 | 2913 | 1.07e3 | 1 | 1 | 258 | 261 |  |  | DB Search |
| Q.VSSL.T | N | 20.48 | 404.2271 | 4 | -7.08 | 405.2305 | 1 | 8.17 | 2993 | 7.47e3 | 1 | 1 | 559 | 562 |  |  | DB Search |
| A.AGAA.G | N | 20.37 | 288.1433 | 4 | 8.03 | 289.1522 | 1 | 5.91 | 2203 | 6.8e3 | 1 | 1 | 827 | 830 |  |  | DB Search |
| A.VTTI.P | N | 20.12 | 432.2584 | 4 | -4.38 | 433.2627 | 1 | 11.15 | 4417 | 0 | 0 | 0 | 207 | 210 |  |  | DB Search |
| L.SITP.G | N | 19.55 | 416.2271 | 4 | 2 | 417.2342 | 1 | 10.25 | 4029 | 3.69e3 | 1 | 1 | 1075 | 1078 |  |  | DB Search |
| S.APVE.V | N | 19.31 | 414.2114 | 4 | -0.87 | 415.2173 | 1 | 3.61 | 1513 | 3.09e3 | 1 | 1 | 1145 | 1148 |  |  | DB Search |
| E.TIGV.R | N | 18.91 | 388.2322 | 4 | -5.15 | 389.2365 | 1 | 17.34 | 6852 | 1.43e3 | 1 | 1 | 472 | 475 |  |  | DB Search |
| V.EQW.A | N | 18.42 | 461.191 | 3 | -3.23 | 462.1957 | 1 | 11.54 | 4588 | 2.45e3 | 1 | 1 | 261 | 263 |  |  | DB Search |
| T.FGRL.P | N | 17.51 | 491.2856 | 4 | -7.95 | 492.2878 | 1 | 15.01 | 5965 | 9.54e2 | 1 | 1 | 100 | 103 |  |  | DB Search |
| A.EVSQ.V | N | 17.14 | 461.2122 | 4 | -7.43 | 462.2149 | 1 | 2.29 | 955 | 6.81e2 | 1 | 1 | 604 | 607 |  |  | DB Search |
| A.ALDT.S | N | 16.7 | 418.2063 | 4 | -7.95 | 419.2093 | 1 | 4.85 | 1907 | 0 | 0 | 0 | 1223 | 1226 |  |  | DB Search |
| Y.AQIE.A | N | 16.19 | 459.2329 | 4 | -1.44 | 460.2384 | 1 | 4.72 | 1830 | 1.97e3 | 1 | 1 | 651 | 654 |  |  | DB Search |
| D.LGGV.K | N | 15.94 | 344.2059 | 4 | 0.19 | 345.2124 | 1 | 5.48 | 2143 | 3.05e3 | 1 | 1 | 428 | 431 |  |  | DB Search |
| P.Q(-17.03)PH.P | N | 15.48 | 363.1543 | 3 | -0.14 | 364.1606 | 1 | 2.32 | 992 | 3.39e4 | 1 | 1 | 375 | 377 | Pyro-glu from Q | Q1:Pyro-glu from Q:1000 | DB Search |
| total 18 peptides |
| --- |

Best Unique PSM (Scan POS\_R15-1.wiff:14277, m/z=821.4095, z=1, RT=47.32, ppm=-11.74):


C4QVD7|C4QVD7\_KOMPG

back to list

  

| Protein Coverage
| Supporting Peptides
| Best Unique PSM
|

Protein Coverage:

Supporting Peptides:

| Peptide | Uniq | -10lgP | Mass | Length | ppm | m/z | z | RT | Scan | Area POS\_R15-1 | #Feature | #Feature POS\_R15-1 | Start | End | PTM | AScore | Found By |
| --- | --- | --- | --- | --- | --- | --- | --- | --- | --- | --- | --- | --- | --- | --- | --- | --- | --- |
| F.FYR.E | N | 26.82 | 484.2434 | 3 | 1.64 | 485.2503 | 1 | 17.34 | 6854 | 0 | 0 | 0 | 373 | 375 |  |  | DB Search |
| R.SLGD.S | N | 20.24 | 390.175 | 4 | -7.72 | 391.1783 | 1 | 3.30 | 1421 | 1.05e3 | 1 | 1 | 1756 | 1759 |  |  | DB Search |
| Y.KFH.M | N | 20.21 | 430.2328 | 3 | 9.29 | 431.243 | 1 | 12.31 | 5009 | 5.31e3 | 1 | 1 | 666 | 668 |  |  | DB Search |
| H.LLQP.Q | N | 19.13 | 469.29 | 4 | -8.42 | 470.2922 | 1 | 12.16 | 4756 | 6.43e3 | 1 | 1 | 510 | 513 |  |  | DB Search |
| L.FHH.H | N | 18.2 | 439.1968 | 3 | 7.99 | 440.2065 | 1 | 9.67 | 3787 | 2e3 | 1 | 1 | 1656 | 1658 |  |  | DB Search |
| T.DLVT.L | N | 16.75 | 446.2376 | 4 | -4.95 | 447.2416 | 1 | 23.24 | 8484 | 0 | 0 | 0 | 560 | 563 |  |  | DB Search |
| I.VEIA.Q | N | 15.81 | 430.2427 | 4 | -4.19 | 431.2471 | 1 | 15.22 | 6032 | 0 | 0 | 0 | 632 | 635 |  |  | DB Search |
| V.STAI.V | N | 15.79 | 390.2114 | 4 | -5.32 | 391.2157 | 1 | 6.25 | 2317 | 1.07e4 | 1 | 1 | 942 | 945 |  |  | DB Search |
| D.SELA.I | N | 15.6 | 418.2063 | 4 | -9.49 | 419.2086 | 1 | 4.93 | 1962 | 0 | 0 | 0 | 1470 | 1473 |  |  | DB Search |
| S.QIVA.E | N | 15.44 | 429.2587 | 4 | -4.77 | 430.2629 | 1 | 10.63 | 4142 | 1.75e3 | 1 | 1 | 1761 | 1764 |  |  | DB Search |
| MSLSDPE.D | Y | 15.33 | 777.3215 | 7 | 2.01 | 778.3284 | 1 | 20.20 | 7571 | 2.89e1 | 1 | 1 | 1 | 7 |  |  | DB Search |
| total 11 peptides |
| --- |

Best Unique PSM (Scan POS\_R15-1.wiff:7571, m/z=778.3661, z=1, RT=20.28, ppm=-0.47):


C4QVP8|C4QVP8\_KOMPG

back to list

  

| Protein Coverage
| Supporting Peptides
| Best Unique PSM
|

Protein Coverage:

Supporting Peptides:

| Peptide | Uniq | -10lgP | Mass | Length | ppm | m/z | z | RT | Scan | Area POS\_R15-1 | #Feature | #Feature POS\_R15-1 | Start | End | PTM | AScore | Found By |
| --- | --- | --- | --- | --- | --- | --- | --- | --- | --- | --- | --- | --- | --- | --- | --- | --- | --- |
| Q.ILLP.M | N | 24.07 | 454.3155 | 4 | -2.4 | 455.3206 | 1 | 34.52 | 11565 | 2.64e3 | 1 | 1 | 91 | 94 |  |  | DB Search |
| V.SSNK.K | N | 20.83 | 434.2125 | 4 | -0.81 | 435.2184 | 1 | 2.82 | 1206 | 3.52e2 | 1 | 1 | 234 | 237 |  |  | DB Search |
| D.ATVM.R | N | 17.53 | 420.2042 | 4 | 1.23 | 421.211 | 1 | 13.78 | 5446 | 5.11e2 | 1 | 1 | 117 | 120 |  |  | DB Search |
| A.ERFQP.Y | N | 17.3 | 675.334 | 5 | -0.55 | 676.3392 | 1 | 9.74 | 3812 | 2.46e3 | 1 | 1 | 252 | 256 |  |  | DB Search |
| V.AQLE.K | N | 16.19 | 459.2329 | 4 | -1.44 | 460.2384 | 1 | 4.72 | 1830 | 1.97e3 | 1 | 1 | 218 | 221 |  |  | DB Search |
| F.E(-18.01)C(-1.01)SEEE.L | Y | 15.88 | 705.2037 | 6 | -2.49 | 706.2075 | 1 | 11.26 | 4477 | 4.72e3 | 1 | 1 | 153 | 158 | Pyro-glu from E, Half of a disulfide bridge | E1:Pyro-glu from E:1000 C2:Half of a disulfide bridge:1000 | DB Search |
| total 6 peptides |
| --- |

Best Unique PSM (Scan POS\_R15-1.wiff:4477, m/z=706.2075, z=1, RT=11.26, ppm=-4.97):


C4R6R0|C4R6R0\_KOMPG

back to list

  

| Protein Coverage
| Supporting Peptides
| Best Unique PSM
|

Protein Coverage:

Supporting Peptides:

| Peptide | Uniq | -10lgP | Mass | Length | ppm | m/z | z | RT | Scan | Area POS\_R15-1 | #Feature | #Feature POS\_R15-1 | Start | End | PTM | AScore | Found By |
| --- | --- | --- | --- | --- | --- | --- | --- | --- | --- | --- | --- | --- | --- | --- | --- | --- | --- |
| I.YNP.M | N | 21.45 | 392.1696 | 3 | -3.75 | 393.1744 | 1 | 8.45 | 3226 | 1.39e2 | 1 | 1 | 60 | 62 |  |  | DB Search |
| P.KFH.A | N | 20.21 | 430.2328 | 3 | 9.29 | 431.243 | 1 | 12.31 | 5009 | 5.31e3 | 1 | 1 | 45 | 47 |  |  | DB Search |
| M.QPVSVPALPQ(+0.98)GY.N | Y | 18.17 | 1255.6448 | 12 | 5.83 | 628.8318 | 2 | 30.32 | 10412 | 4.39e3 | 1 | 1 | 2 | 13 | Deamidation (NQ) | Q10:Deamidation (NQ):46.29 | DB Search |
| total 3 peptides |
| --- |

Best Unique PSM (Scan POS\_R15-1.wiff:10412, m/z=628.8318, z=2, RT=30.32, ppm=3.34):


C4QZ37|C4QZ37\_KOMPG

back to list

  

| Protein Coverage
| Supporting Peptides
| Best Unique PSM
|

Protein Coverage:

Supporting Peptides:

| Peptide | Uniq | -10lgP | Mass | Length | ppm | m/z | z | RT | Scan | Area POS\_R15-1 | #Feature | #Feature POS\_R15-1 | Start | End | PTM | AScore | Found By |
| --- | --- | --- | --- | --- | --- | --- | --- | --- | --- | --- | --- | --- | --- | --- | --- | --- | --- |
| P.TILMLGGL.Y | Y | 32.75 | 816.4779 | 8 | -9.7 | 817.4752 | 1 | 46.74 | 14179 | 9.87e2 | 1 | 1 | 316 | 323 |  |  | DB Search |
| A.PSII.F | N | 29.95 | 428.2635 | 4 | -0.56 | 429.2694 | 1 | 21.28 | 7884 | 0 | 0 | 0 | 455 | 458 |  |  | DB Search |
| L.GGGI.F | N | 27.46 | 302.159 | 4 | -3.72 | 303.1644 | 1 | 6.06 | 2387 | 3.14e3 | 1 | 1 | 337 | 340 |  |  | DB Search |
| M.LGGL.Y | N | 27.34 | 358.2216 | 4 | -4.39 | 359.2264 | 1 | 15.45 | 6114 | 7.1e3 | 1 | 1 | 320 | 323 |  |  | DB Search |
| S.IGSL.E | N | 25.26 | 388.2322 | 4 | -3.81 | 389.237 | 1 | 9.87 | 3785 | 6.08e3 | 1 | 1 | 271 | 274 |  |  | DB Search |
| L.KHF.E | N | 17.4 | 430.2328 | 3 | 0.78 | 431.2394 | 1 | 20.46 | 7703 | 1.69e2 | 1 | 1 | 598 | 600 |  |  | DB Search |
| Q.FNH.Q | N | 17.22 | 416.1808 | 3 | -5.74 | 417.1847 | 1 | 6.08 | 2390 | 0 | 0 | 0 | 45 | 47 |  |  | DB Search |
| S.AALG.Y | N | 16.78 | 330.1903 | 4 | -8.43 | 331.194 | 1 | 5.91 | 2160 | 5.17e3 | 1 | 1 | 657 | 660 |  |  | DB Search |
| F.FSIGS.L | N | 16.56 | 509.2485 | 5 | -3.68 | 510.2527 | 1 | 11.03 | 4274 | 5.2e2 | 1 | 1 | 269 | 273 |  |  | DB Search |
| L.TPGF.S | N | 15.9 | 420.2009 | 4 | 2.59 | 421.2082 | 1 | 12.63 | 4943 | 3.01e3 | 1 | 1 | 568 | 571 |  |  | DB Search |
| total 10 peptides |
| --- |

Best Unique PSM (Scan POS\_R15-1.wiff:14179, m/z=817.4709, z=1, RT=46.80, ppm=-12.18):


C4R4T4|C4R4T4\_KOMPG

back to list

  

| Protein Coverage
| Supporting Peptides
| Best Unique PSM
|

Protein Coverage:

Supporting Peptides:

| Peptide | Uniq | -10lgP | Mass | Length | ppm | m/z | z | RT | Scan | Area POS\_R15-1 | #Feature | #Feature POS\_R15-1 | Start | End | PTM | AScore | Found By |
| --- | --- | --- | --- | --- | --- | --- | --- | --- | --- | --- | --- | --- | --- | --- | --- | --- | --- |
| S.SPT.M | N | 23.16 | 303.143 | 3 | 6.39 | 304.1515 | 1 | 7.14 | 2731 | 5.4e3 | 2 | 2 | 62 | 64 |  |  | DB Search |
| S.SSSQQP.L | Y | 22.63 | 632.2766 | 6 | -5.1 | 633.2791 | 1 | 9.63 | 3737 | 1.16e3 | 1 | 1 | 92 | 97 |  |  | DB Search |
| E.TFR.C | N | 20.53 | 422.2278 | 3 | -9.2 | 423.2301 | 1 | 22.38 | 8244 | 2.98e1 | 1 | 1 | 235 | 237 |  |  | DB Search |
| F.GSVI.R | N | 20.47 | 374.2165 | 4 | -5.12 | 375.2209 | 1 | 10.69 | 4154 | 1e3 | 1 | 1 | 129 | 132 |  |  | DB Search |
| A.SGLI.S | N | 19.42 | 388.2322 | 4 | -5.15 | 389.2365 | 1 | 17.34 | 6766 | 1.43e3 | 1 | 1 | 38 | 41 |  |  | DB Search |
| R.GSSL.G | N | 18.21 | 362.1801 | 4 | -9.58 | 363.183 | 1 | 6.30 | 2465 | 1.38e3 | 1 | 1 | 31 | 34 |  |  | DB Search |
| T.ESPL.F | N | 18.2 | 444.222 | 4 | -3.28 | 445.2267 | 1 | 12.52 | 4847 | 1.87e3 | 1 | 1 | 210 | 213 |  |  | DB Search |
| R.LTP.F | N | 17.99 | 329.1951 | 3 | 1.36 | 330.202 | 1 | 7.49 | 2815 | 1.47e3 | 1 | 1 | 113 | 115 |  |  | DB Search |
| T.FRC.V | N | 17.7 | 424.1893 | 3 | -9.2 | 425.1916 | 1 | 10.20 | 3929 | 1.22e3 | 1 | 1 | 236 | 238 |  |  | DB Search |
| D.FRG.G | N | 15.51 | 378.2015 | 3 | -9.17 | 379.2044 | 1 | 3.37 | 1492 | 1.94e3 | 1 | 1 | 228 | 230 |  |  | DB Search |
| Q.Q(-17.03)PL.P | N | 15.26 | 339.1794 | 3 | -4.38 | 340.1844 | 1 | 15.14 | 5814 | 1.63e4 | 1 | 1 | 96 | 98 | Pyro-glu from Q | Q1:Pyro-glu from Q:1000 | DB Search |
| total 11 peptides |
| --- |

Best Unique PSM (Scan POS\_R15-1.wiff:3737, m/z=633.2791, z=1, RT=9.63, ppm=-7.58):


C4R8L4|C4R8L4\_KOMPG

back to list

  

| Protein Coverage
| Supporting Peptides
| Best Unique PSM
|

Protein Coverage:

Supporting Peptides:

| Peptide | Uniq | -10lgP | Mass | Length | ppm | m/z | z | RT | Scan | Area POS\_R15-1 | #Feature | #Feature POS\_R15-1 | Start | End | PTM | AScore | Found By |
| --- | --- | --- | --- | --- | --- | --- | --- | --- | --- | --- | --- | --- | --- | --- | --- | --- | --- |
| M.LQEQ.S | N | 18.84 | 516.2544 | 4 | -0.21 | 517.2603 | 1 | 2.57 | 1095 | 1.3e3 | 1 | 1 | 2 | 5 |  |  | DB Search |
| R.KAQAGSNG.G | Y | 15.76 | 731.3562 | 8 | -8.8 | 732.3552 | 1 | 8.83 | 3433 | 2.92e2 | 1 | 1 | 197 | 204 |  |  | DB Search |
| G.QNND.L | N | 15.34 | 489.1819 | 4 | 5.34 | 490.1906 | 1 | 2.90 | 1227 | 0 | 0 | 0 | 294 | 297 |  |  | DB Search |
| total 3 peptides |
| --- |

Best Unique PSM (Scan POS\_R15-1.wiff:3433, m/z=732.3134, z=1, RT=8.84, ppm=-11.28):


C4R2R4|C4R2R4\_KOMPG

back to list

  

| Protein Coverage
| Supporting Peptides
| Best Unique PSM
|

Protein Coverage:

Supporting Peptides:

| Peptide | Uniq | -10lgP | Mass | Length | ppm | m/z | z | RT | Scan | Area POS\_R15-1 | #Feature | #Feature POS\_R15-1 | Start | End | PTM | AScore | Found By |
| --- | --- | --- | --- | --- | --- | --- | --- | --- | --- | --- | --- | --- | --- | --- | --- | --- | --- |
| T.ITGI.A | N | 27.55 | 402.2478 | 4 | -1.76 | 403.2534 | 1 | 15.04 | 5964 | 1.56e4 | 2 | 2 | 196 | 199 |  |  | DB Search |
| N.TGIGLALVG.A | Y | 21.83 | 799.4803 | 9 | -9.03 | 800.4784 | 1 | 32.22 | 10935 | 6.06e2 | 1 | 1 | 85 | 93 |  |  | DB Search |
| A.AIVE.N | N | 20.45 | 430.2427 | 4 | -5.9 | 431.2464 | 1 | 12.49 | 4901 | 0 | 0 | 0 | 472 | 475 |  |  | DB Search |
| V.KGY.R | N | 19.27 | 366.1903 | 3 | -9.39 | 367.1932 | 1 | 6.66 | 2561 | 3.55e1 | 1 | 1 | 96 | 98 |  |  | DB Search |
| K.LNGF.T | N | 16.9 | 449.2274 | 4 | 0.62 | 450.2339 | 1 | 15.55 | 6174 | 0 | 0 | 0 | 329 | 332 |  |  | DB Search |
| total 5 peptides |
| --- |

Best Unique PSM (Scan POS\_R15-1.wiff:10935, m/z=800.4784, z=1, RT=32.22, ppm=-11.51):


C4R8V1|C4R8V1\_KOMPG

back to list

  

| Protein Coverage
| Supporting Peptides
| Best Unique PSM
|

Protein Coverage:

Supporting Peptides:

| Peptide | Uniq | -10lgP | Mass | Length | ppm | m/z | z | RT | Scan | Area POS\_R15-1 | #Feature | #Feature POS\_R15-1 | Start | End | PTM | AScore | Found By |
| --- | --- | --- | --- | --- | --- | --- | --- | --- | --- | --- | --- | --- | --- | --- | --- | --- | --- |
| S.DYNIQ.K | Y | 20.46 | 651.2864 | 5 | -0.94 | 652.2914 | 1 | 10.88 | 4258 | 1.35e3 | 1 | 1 | 58 | 62 |  |  | DB Search |
| total 1 peptides |
| --- |

Best Unique PSM (Scan POS\_R15-1.wiff:4258, m/z=652.2914, z=1, RT=10.88, ppm=-3.43):


C4R646|C4R646\_KOMPG

back to list

  

| Protein Coverage
| Supporting Peptides
| Best Unique PSM
|

Protein Coverage:

Supporting Peptides:

| Peptide | Uniq | -10lgP | Mass | Length | ppm | m/z | z | RT | Scan | Area POS\_R15-1 | #Feature | #Feature POS\_R15-1 | Start | End | PTM | AScore | Found By |
| --- | --- | --- | --- | --- | --- | --- | --- | --- | --- | --- | --- | --- | --- | --- | --- | --- | --- |
| H.GAPNYAHP.A | Y | 24.12 | 825.3769 | 8 | -5.56 | 826.3776 | 1 | 14.20 | 5638 | 3.3e2 | 1 | 1 | 204 | 211 |  |  | DB Search |
| P.VGGP.V | N | 23.36 | 328.1746 | 4 | -1.91 | 329.1805 | 1 | 5.92 | 2159 | 4.71e4 | 1 | 1 | 239 | 242 |  |  | DB Search |
| A.HPAA.A | N | 22.78 | 394.1964 | 4 | 8.24 | 395.206 | 1 | 15.22 | 6038 | 1e3 | 1 | 1 | 210 | 213 |  |  | DB Search |
| Y.TFT.T | N | 15.92 | 367.1743 | 3 | -7.47 | 368.1779 | 1 | 8.54 | 3263 | 0 | 0 | 0 | 125 | 127 |  |  | DB Search |
| G.EGS.A | N | 15.77 | 291.1066 | 3 | 7.44 | 292.1154 | 1 | 3.53 | 1538 | 0 | 0 | 0 | 183 | 185 |  |  | DB Search |
| total 5 peptides |
| --- |

Best Unique PSM (Scan POS\_R15-1.wiff:5638, m/z=826.3638, z=1, RT=14.25, ppm=-8.05):


C4R5W4|C4R5W4\_KOMPG

back to list

  

| Protein Coverage
| Supporting Peptides
| Best Unique PSM
|

Protein Coverage:

Supporting Peptides:

| Peptide | Uniq | -10lgP | Mass | Length | ppm | m/z | z | RT | Scan | Area POS\_R15-1 | #Feature | #Feature POS\_R15-1 | Start | End | PTM | AScore | Found By |
| --- | --- | --- | --- | --- | --- | --- | --- | --- | --- | --- | --- | --- | --- | --- | --- | --- | --- |
| N.PSLL.N | N | 29.95 | 428.2635 | 4 | -0.56 | 429.2694 | 1 | 21.28 | 7884 | 0 | 0 | 0 | 300 | 303 |  |  | DB Search |
| K.PSVP.H | N | 21.16 | 398.2165 | 4 | 4.76 | 399.2247 | 1 | 9.14 | 3512 | 0 | 0 | 0 | 313 | 316 |  |  | DB Search |
| P.KFH.P | N | 20.21 | 430.2328 | 3 | 9.29 | 431.243 | 1 | 12.31 | 5009 | 5.31e3 | 1 | 1 | 217 | 219 |  |  | DB Search |
| N.APVE.T | N | 19.31 | 414.2114 | 4 | -0.87 | 415.2173 | 1 | 3.61 | 1513 | 3.09e3 | 1 | 1 | 331 | 334 |  |  | DB Search |
| N.AITP.D | N | 18.91 | 400.2322 | 4 | -8.5 | 401.235 | 1 | 9.38 | 3619 | 0 | 0 | 0 | 494 | 497 |  |  | DB Search |
| Q.PQPQ(+0.98)QQ(+0.98)QPQPQ.Q | Y | 17.92 | 1304.5997 | 11 | 0.72 | 653.306 | 2 | 21.85 | 8049 | 1.02e3 | 1 | 1 | 231 | 241 | Deamidation (NQ), Deamidation (NQ) | Q4:Deamidation (NQ):0 Q6:Deamidation (NQ):20 | DB Search |
| P.KHF.K | N | 17.4 | 430.2328 | 3 | 0.78 | 431.2394 | 1 | 20.46 | 7703 | 1.69e2 | 1 | 1 | 59 | 61 |  |  | DB Search |
| K.DILP.K | N | 16.51 | 456.2584 | 4 | -3.13 | 457.2631 | 1 | 23.84 | 8659 | 1.35e3 | 1 | 1 | 55 | 58 |  |  | DB Search |
| A.SELA.E | N | 15.6 | 418.2063 | 4 | -9.49 | 419.2086 | 1 | 4.93 | 1962 | 0 | 0 | 0 | 134 | 137 |  |  | DB Search |
| Q.Q(-17.03)PH.H | N | 15.48 | 363.1543 | 3 | -0.14 | 364.1606 | 1 | 2.32 | 992 | 3.39e4 | 1 | 1 | 223 | 225 | Pyro-glu from Q | Q1:Pyro-glu from Q:1000 | DB Search |
| L.EALE.N | N | 15.32 | 460.2169 | 4 | -0.24 | 461.2229 | 1 | 13.77 | 5437 | 0 | 0 | 0 | 114 | 117 |  |  | DB Search |
| total 11 peptides |
| --- |

Best Unique PSM (Scan POS\_R15-1.wiff:8049, m/z=653.306, z=2, RT=21.85, ppm=-1.77):


C4R581|C4R581\_KOMPG

back to list

  

| Protein Coverage
| Supporting Peptides
| Best Unique PSM
|

Protein Coverage:

Supporting Peptides:

| Peptide | Uniq | -10lgP | Mass | Length | ppm | m/z | z | RT | Scan | Area POS\_R15-1 | #Feature | #Feature POS\_R15-1 | Start | End | PTM | AScore | Found By |
| --- | --- | --- | --- | --- | --- | --- | --- | --- | --- | --- | --- | --- | --- | --- | --- | --- | --- |
| L.PFPP.A | N | 23.62 | 456.2372 | 4 | 1.78 | 457.2442 | 1 | 13.81 | 5467 | 0 | 0 | 0 | 157 | 160 |  |  | DB Search |
| Q.SRIPQ(+0.98)YG.H | Y | 22.05 | 820.4079 | 7 | 5.43 | 821.4176 | 1 | 12.64 | 5004 | 4.29e2 | 2 | 2 | 165 | 171 | Deamidation (NQ) | Q5:Deamidation (NQ):1000 | DB Search |
| Y.NTS.Q | N | 21.44 | 320.1332 | 3 | 8.58 | 321.1424 | 1 | 6.16 | 2421 | 0 | 0 | 0 | 199 | 201 |  |  | DB Search |
| N.DVTL.Y | N | 21.02 | 446.2376 | 4 | -4.47 | 447.2418 | 1 | 16.24 | 6386 | 3.07e3 | 1 | 1 | 794 | 797 |  |  | DB Search |
| D.TFR.A | N | 20.53 | 422.2278 | 3 | -9.2 | 423.2301 | 1 | 22.38 | 8244 | 2.98e1 | 1 | 1 | 448 | 450 |  |  | DB Search |
| A.KFH.S | N | 20.21 | 430.2328 | 3 | 9.29 | 431.243 | 1 | 12.31 | 5009 | 5.31e3 | 1 | 1 | 779 | 781 |  |  | DB Search |
| L.RPD.I | N | 19.79 | 386.1914 | 3 | -3.61 | 387.1963 | 1 | 2.95 | 1225 | 1.33e2 | 1 | 1 | 693 | 695 |  |  | DB Search |
| L.QFF.S | N | 19.56 | 440.206 | 3 | 3.92 | 441.2139 | 1 | 19.15 | 7313 | 0 | 0 | 0 | 386 | 388 |  |  | DB Search |
| I.RSSS.Q | N | 18.8 | 435.2078 | 4 | -3.98 | 436.2122 | 1 | 11.38 | 4548 | 7.31e2 | 1 | 1 | 143 | 146 |  |  | DB Search |
| A.WEDL.T | N | 18.39 | 561.2435 | 4 | -3.61 | 562.2473 | 1 | 13.44 | 5311 | 1.98e2 | 1 | 1 | 946 | 949 |  |  | DB Search |
| Y.GHY.P | N | 18.12 | 375.1543 | 3 | -2.57 | 376.1596 | 1 | 4.82 | 1883 | 3.43e3 | 1 | 1 | 171 | 173 |  |  | DB Search |
| K.TKC.D | N | 16.14 | 350.1624 | 3 | 7.67 | 351.1715 | 1 | 4.93 | 1958 | 0 | 0 | 0 | 19 | 21 |  |  | DB Search |
| N.NNKP.E | N | 15.98 | 471.2441 | 4 | -8.81 | 472.2461 | 1 | 7.31 | 2757 | 3.31e2 | 1 | 1 | 115 | 118 |  |  | DB Search |
| K.TFT.I | N | 15.92 | 367.1743 | 3 | -7.47 | 368.1779 | 1 | 8.54 | 3263 | 0 | 0 | 0 | 239 | 241 |  |  | DB Search |
| L.Q(-17.03)PH.D | N | 15.48 | 363.1543 | 3 | -0.14 | 364.1606 | 1 | 2.32 | 992 | 3.39e4 | 1 | 1 | 72 | 74 | Pyro-glu from Q | Q1:Pyro-glu from Q:1000 | DB Search |
| A.SVDP.N | N | 15.45 | 416.1907 | 4 | -1.13 | 417.1965 | 1 | 4.88 | 1929 | 2.13e3 | 1 | 1 | 857 | 860 |  |  | DB Search |
| L.IGVP.H | N | 15.32 | 384.2372 | 4 | -3.68 | 385.2422 | 1 | 16.13 | 6408 | 0 | 0 | 0 | 122 | 125 |  |  | DB Search |
| total 17 peptides |
| --- |

Best Unique PSM (Scan POS\_R15-1.wiff:5004, m/z=821.4055, z=1, RT=12.69, ppm=2.95):


C4QV93|C4QV93\_KOMPG

back to list

  

| Protein Coverage
| Supporting Peptides
| Best Unique PSM
|

Protein Coverage:

Supporting Peptides:

| Peptide | Uniq | -10lgP | Mass | Length | ppm | m/z | z | RT | Scan | Area POS\_R15-1 | #Feature | #Feature POS\_R15-1 | Start | End | PTM | AScore | Found By |
| --- | --- | --- | --- | --- | --- | --- | --- | --- | --- | --- | --- | --- | --- | --- | --- | --- | --- |
| S.LGSL.S | N | 25.26 | 388.2322 | 4 | -3.81 | 389.237 | 1 | 9.87 | 3785 | 6.08e3 | 1 | 1 | 104 | 107 |  |  | DB Search |
| P.FSNTLI.E | Y | 22.94 | 693.3697 | 6 | -4.9 | 694.3719 | 1 | 28.23 | 9887 | 0 | 0 | 0 | 511 | 516 |  |  | DB Search |
| N.SIVGL.D | N | 22.5 | 487.3006 | 5 | -4.27 | 488.3046 | 1 | 21.68 | 8060 | 8.06e3 | 1 | 1 | 204 | 208 |  |  | DB Search |
| K.RSTS.I | N | 21.58 | 449.2234 | 4 | -1.6 | 450.2289 | 1 | 15.62 | 6208 | 1.17e3 | 1 | 1 | 248 | 251 |  |  | DB Search |
| K.LTDL.S | N | 20.88 | 460.2533 | 4 | -0.2 | 461.2593 | 1 | 14.80 | 5851 | 7.36e2 | 1 | 1 | 567 | 570 |  |  | DB Search |
| P.LSPM.E | N | 19.59 | 446.2199 | 4 | -1.9 | 447.2252 | 1 | 15.64 | 6201 | 6.79e2 | 1 | 1 | 211 | 214 |  |  | DB Search |
| D.QSVA.H | N | 19.54 | 403.2067 | 4 | -0.86 | 404.2126 | 1 | 2.86 | 1199 | 5.58e3 | 1 | 1 | 697 | 700 |  |  | DB Search |
| A.STSP.K | N | 19.15 | 390.1751 | 4 | -0.08 | 391.1813 | 1 | 5.92 | 2360 | 3.31e2 | 1 | 1 | 612 | 615 |  |  | DB Search |
| S.SNG.L | N | 19.07 | 276.107 | 3 | 1.61 | 277.114 | 1 | 16.82 | 6604 | 0 | 0 | 0 | 79 | 81 |  |  | DB Search |
| K.HISTA.P | N | 17.56 | 527.2703 | 5 | 2.65 | 528.2777 | 1 | 21.95 | 8111 | 1.51e2 | 1 | 1 | 617 | 621 |  |  | DB Search |
| T.SPK.H | N | 16.89 | 330.1903 | 3 | -8.46 | 331.194 | 1 | 5.91 | 2235 | 5.17e3 | 1 | 1 | 614 | 616 |  |  | DB Search |
| I.E(-18.01)PR.F | N | 16.87 | 382.1965 | 3 | -0.37 | 383.2026 | 1 | 2.61 | 1082 | 1.62e4 | 1 | 1 | 167 | 169 | Pyro-glu from E | E1:Pyro-glu from E:1000 | DB Search |
| A.PACR.I | N | 16.06 | 445.2107 | 4 | 4.57 | 446.2189 | 1 | 4.98 | 1993 | 0 | 0 | 0 | 656 | 659 |  |  | DB Search |
| M.VELA.P | N | 15.81 | 430.2427 | 4 | -4.19 | 431.2471 | 1 | 15.22 | 6032 | 0 | 0 | 0 | 652 | 655 |  |  | DB Search |
| S.RQHSP.V | N | 15.54 | 623.3139 | 5 | -6.52 | 624.3156 | 1 | 19.06 | 7330 | 4.41e2 | 1 | 1 | 266 | 270 |  |  | DB Search |
| total 15 peptides |
| --- |

Best Unique PSM (Scan POS\_R15-1.wiff:9887, m/z=694.3719, z=1, RT=28.23, ppm=-7.38):


C4R6C5|C4R6C5\_KOMPG

back to list

  

| Protein Coverage
| Supporting Peptides
| Best Unique PSM
|

Protein Coverage:

Supporting Peptides:

| Peptide | Uniq | -10lgP | Mass | Length | ppm | m/z | z | RT | Scan | Area POS\_R15-1 | #Feature | #Feature POS\_R15-1 | Start | End | PTM | AScore | Found By |
| --- | --- | --- | --- | --- | --- | --- | --- | --- | --- | --- | --- | --- | --- | --- | --- | --- | --- |
| F.SPII.T | N | 26.66 | 428.2635 | 4 | -1.63 | 429.269 | 1 | 21.35 | 7905 | 0 | 0 | 0 | 888 | 891 |  |  | DB Search |
| E.ALGI.N | N | 25.12 | 372.2372 | 4 | -5.1 | 373.2417 | 1 | 21.84 | 8058 | 0 | 0 | 0 | 446 | 449 |  |  | DB Search |
| Y.ELIR.S | N | 25.05 | 529.3224 | 4 | -4.56 | 530.3259 | 1 | 17.34 | 6855 | 6.68e3 | 1 | 1 | 734 | 737 |  |  | DB Search |
| Y.PVLV.Q | N | 23.27 | 426.2842 | 4 | 4.41 | 427.2923 | 1 | 20.36 | 7664 | 0 | 0 | 0 | 48 | 51 |  |  | DB Search |
| S.SLPT.K | N | 22.87 | 416.2271 | 4 | -6.88 | 417.2305 | 1 | 14.64 | 5767 | 5.65e3 | 1 | 1 | 177 | 180 |  |  | DB Search |
| S.TIDL.Y | N | 22.27 | 460.2533 | 4 | -8.03 | 461.2557 | 1 | 9.41 | 3625 | 2.97e2 | 1 | 1 | 1011 | 1014 |  |  | DB Search |
| T.NTS.V | N | 21.44 | 320.1332 | 3 | 8.58 | 321.1424 | 1 | 6.16 | 2421 | 0 | 0 | 0 | 859 | 861 |  |  | DB Search |
| F.PEF.T | N | 20.63 | 391.1743 | 3 | 0.03 | 392.1806 | 1 | 4.06 | 1653 | 5.45e3 | 1 | 1 | 994 | 996 |  |  | DB Search |
| P.FPS.I | N | 19.27 | 349.1638 | 3 | -4.81 | 350.1685 | 1 | 16.32 | 6461 | 1.41e3 | 1 | 1 | 282 | 284 |  |  | DB Search |
| P.GSSL.P | N | 18.21 | 362.1801 | 4 | -9.58 | 363.183 | 1 | 6.30 | 2465 | 1.38e3 | 1 | 1 | 175 | 178 |  |  | DB Search |
| K.LIVD.N | N | 18.11 | 458.274 | 4 | -9.29 | 459.2759 | 1 | 14.70 | 5798 | 0 | 0 | 0 | 619 | 622 |  |  | DB Search |
| T.TSN.P | N | 17.54 | 320.1332 | 3 | 3.34 | 321.1407 | 1 | 8.11 | 3024 | 0 | 0 | 0 | 265 | 267 |  |  | DB Search |
| P.IITD.Q | N | 17.52 | 460.2533 | 4 | -9.09 | 461.2552 | 1 | 10.16 | 3965 | 9.46e2 | 1 | 1 | 890 | 893 |  |  | DB Search |
| L.KHF.L | N | 17.4 | 430.2328 | 3 | 0.78 | 431.2394 | 1 | 20.46 | 7703 | 1.69e2 | 1 | 1 | 776 | 778 |  |  | DB Search |
| E.E(-18.01)PR.S | N | 16.87 | 382.1965 | 3 | -0.37 | 383.2026 | 1 | 2.61 | 1082 | 1.62e4 | 1 | 1 | 100 | 102 | Pyro-glu from E | E1:Pyro-glu from E:1000 | DB Search |
| L.AALG.I | N | 16.78 | 330.1903 | 4 | -8.43 | 331.194 | 1 | 5.91 | 2160 | 5.17e3 | 1 | 1 | 469 | 472 |  |  | DB Search |
| F.EVQ.P | N | 16.23 | 374.1801 | 3 | 0.92 | 375.1868 | 1 | 2.99 | 1221 | 7.34e3 | 1 | 1 | 315 | 317 |  |  | DB Search |
| D.EALGIN.L | Y | 16.21 | 615.3228 | 6 | -4.11 | 616.326 | 1 | 15.07 | 5966 | 1.47e3 | 1 | 1 | 445 | 450 |  |  | DB Search |
| K.Q(-17.03)QLIG.K | N | 16.12 | 540.2908 | 5 | -4.73 | 541.2941 | 1 | 23.69 | 8617 | 1.76e4 | 1 | 1 | 707 | 711 | Pyro-glu from Q | Q1:Pyro-glu from Q:1000 | DB Search |
| S.IDEI.K | N | 16.03 | 488.2482 | 4 | -7.44 | 489.2506 | 1 | 15.57 | 6209 | 9.61e2 | 1 | 1 | 134 | 137 |  |  | DB Search |
| M.VELA.N | N | 15.81 | 430.2427 | 4 | -4.19 | 431.2471 | 1 | 15.22 | 6032 | 0 | 0 | 0 | 765 | 768 |  |  | DB Search |
| R.NHL.T | N | 15.79 | 382.1965 | 3 | -0.71 | 383.2025 | 1 | 19.27 | 7368 | 1.28e3 | 1 | 1 | 434 | 436 |  |  | DB Search |
| G.KSGN.K | N | 15.78 | 404.2019 | 4 | 4.41 | 405.21 | 1 | 11.16 | 4428 | 0 | 0 | 0 | 614 | 617 |  |  | DB Search |
| R.QVAL.K | N | 15.07 | 429.2587 | 4 | 5.6 | 430.2673 | 1 | 21.95 | 8124 | 0 | 0 | 0 | 927 | 930 |  |  | DB Search |
| total 24 peptides |
| --- |

Best Unique PSM (Scan POS\_R15-1.wiff:5966, m/z=616.326, z=1, RT=15.07, ppm=-6.59):


C4R1R9|C4R1R9\_KOMPG

back to list

  

| Protein Coverage
| Supporting Peptides
| Best Unique PSM
|

Protein Coverage:

Supporting Peptides:

| Peptide | Uniq | -10lgP | Mass | Length | ppm | m/z | z | RT | Scan | Area POS\_R15-1 | #Feature | #Feature POS\_R15-1 | Start | End | PTM | AScore | Found By |
| --- | --- | --- | --- | --- | --- | --- | --- | --- | --- | --- | --- | --- | --- | --- | --- | --- | --- |
| G.LTGI.E | N | 27.55 | 402.2478 | 4 | -1.76 | 403.2534 | 1 | 15.04 | 5964 | 1.56e4 | 2 | 2 | 651 | 654 |  |  | DB Search |
| E.YGR.G | N | 21.6 | 394.1965 | 3 | -4.25 | 395.2011 | 1 | 5.35 | 1961 | 1.67e4 | 1 | 1 | 113 | 115 |  |  | DB Search |
| V.TAY.D | N | 19.3 | 353.1587 | 3 | -1.32 | 354.1646 | 1 | 12.51 | 4913 | 1.39e3 | 1 | 1 | 156 | 158 |  |  | DB Search |
| Q.VNVP.V | N | 18.75 | 427.2431 | 4 | 2.54 | 428.2504 | 1 | 12.03 | 4834 | 6.48e3 | 1 | 1 | 5 | 8 |  |  | DB Search |
| D.AAVL.K | N | 18.4 | 372.2372 | 4 | -5.92 | 373.2414 | 1 | 12.97 | 5130 | 2.29e3 | 1 | 1 | 28 | 31 |  |  | DB Search |
| D.IDNF.E | N | 18.37 | 507.2329 | 4 | -5.34 | 508.2362 | 1 | 14.74 | 5819 | 0 | 0 | 0 | 470 | 473 |  |  | DB Search |
| L.VELG.I | N | 17.8 | 416.2271 | 4 | -4.65 | 417.2314 | 1 | 10.12 | 3944 | 3.69e3 | 1 | 1 | 118 | 121 |  |  | DB Search |
| I.SKVH.Q | N | 16.42 | 469.2649 | 4 | -2.56 | 470.2698 | 1 | 1.09 | 269 | 3.02e4 | 1 | 1 | 636 | 639 |  |  | DB Search |
| K.TLNG.E | N | 15.96 | 403.2067 | 4 | -9.11 | 404.2093 | 1 | 5.03 | 2025 | 0 | 0 | 0 | 561 | 564 |  |  | DB Search |
| R.N(+0.98)VLAGIGGA.S | Y | 15.73 | 771.4126 | 9 | -4.62 | 772.4144 | 1 | 15.01 | 5947 | 5.82e2 | 1 | 1 | 285 | 293 | Deamidation (NQ) | N1:Deamidation (NQ):1000 | DB Search |
| G.LTET.V | N | 15.47 | 462.2326 | 4 | 1.41 | 463.2393 | 1 | 15.37 | 6065 | 2.41e2 | 1 | 1 | 458 | 461 |  |  | DB Search |
| P.QNGFVTSAQ.K | Y | 15.11 | 950.4457 | 9 | -1.27 | 951.4495 | 1 | 10.25 | 4052 | 1.17e3 | 1 | 1 | 670 | 678 |  |  | DB Search |
| A.QVAI.D | N | 15.07 | 429.2587 | 4 | 5.6 | 430.2673 | 1 | 21.95 | 8124 | 0 | 0 | 0 | 223 | 226 |  |  | DB Search |
| total 13 peptides |
| --- |

Best Unique PSM (Scan POS\_R15-1.wiff:5947, m/z=772.4144, z=1, RT=15.01, ppm=-7.11):


C4R155|C4R155\_KOMPG

back to list

  

| Protein Coverage
| Supporting Peptides
| Best Unique PSM
|

Protein Coverage:

Supporting Peptides:

| Peptide | Uniq | -10lgP | Mass | Length | ppm | m/z | z | RT | Scan | Area POS\_R15-1 | #Feature | #Feature POS\_R15-1 | Start | End | PTM | AScore | Found By |
| --- | --- | --- | --- | --- | --- | --- | --- | --- | --- | --- | --- | --- | --- | --- | --- | --- | --- |
| K.LPSNP.Q | N | 34.2 | 526.2751 | 5 | -5.22 | 527.2783 | 1 | 8.79 | 3411 | 0 | 0 | 0 | 1976 | 1980 |  |  | DB Search |
| E.LTGL.K | N | 27.55 | 402.2478 | 4 | -1.76 | 403.2534 | 1 | 15.04 | 5964 | 1.56e4 | 2 | 2 | 1221 | 1224 |  |  | DB Search |
| H.FYR.I | N | 26.82 | 484.2434 | 3 | 1.64 | 485.2503 | 1 | 17.34 | 6854 | 0 | 0 | 0 | 245 | 247 |  |  | DB Search |
| F.VVGI.G | N | 26.8 | 386.2529 | 4 | -6 | 387.2569 | 1 | 17.69 | 6924 | 2.17e3 | 1 | 1 | 553 | 556 |  |  | DB Search |
| S.ELVR.S | N | 23.24 | 515.3067 | 4 | -4.7 | 516.3103 | 1 | 11.12 | 4401 | 1.13e4 | 1 | 1 | 8 | 11 |  |  | DB Search |
| L.IPRP.G | N | 21.04 | 481.3012 | 4 | -9.61 | 482.3027 | 1 | 17.02 | 6682 | 0 | 0 | 0 | 1961 | 1964 |  |  | DB Search |
| C.DVTI.R | N | 21.02 | 446.2376 | 4 | -4.47 | 447.2418 | 1 | 16.24 | 6386 | 3.07e3 | 1 | 1 | 1200 | 1203 |  |  | DB Search |
| Q.RCI.S | N | 20.6 | 390.2049 | 3 | -4.41 | 391.2095 | 1 | 8.08 | 3003 | 0 | 0 | 0 | 89 | 91 |  |  | DB Search |
| V.TVGV.L | N | 20.58 | 374.2165 | 4 | -4.06 | 375.2213 | 1 | 5.20 | 1970 | 6.27e3 | 1 | 1 | 1799 | 1802 |  |  | DB Search |
| P.VTGV.F | N | 20.36 | 374.2165 | 4 | -4.06 | 375.2213 | 1 | 5.20 | 1917 | 6.27e3 | 1 | 1 | 700 | 703 |  |  | DB Search |
| R.EGVP.G | N | 19.01 | 400.1958 | 4 | -2.37 | 401.2011 | 1 | 8.27 | 3077 | 4.2e3 | 1 | 1 | 1366 | 1369 |  |  | DB Search |
| V.ESPI.S | N | 18.2 | 444.222 | 4 | -3.28 | 445.2267 | 1 | 12.52 | 4847 | 1.87e3 | 1 | 1 | 890 | 893 |  |  | DB Search |
| D.STIA.T | N | 17.83 | 390.2114 | 4 | -5.32 | 391.2157 | 1 | 6.25 | 2360 | 1.07e4 | 1 | 1 | 472 | 475 |  |  | DB Search |
| P.EKAP.A | N | 17 | 443.238 | 4 | -7.26 | 444.2409 | 1 | 12.32 | 4807 | 1.66e2 | 1 | 1 | 954 | 957 |  |  | DB Search |
| N.ALDT.L | N | 16.7 | 418.2063 | 4 | -7.95 | 419.2093 | 1 | 4.85 | 1907 | 0 | 0 | 0 | 989 | 992 |  |  | DB Search |
| E.LSAI.I | N | 16.63 | 402.2478 | 4 | -8.97 | 403.2505 | 1 | 9.42 | 3655 | 1.65e3 | 1 | 1 | 1534 | 1537 |  |  | DB Search |
| V.FVVG.I | N | 16.5 | 420.2372 | 4 | -7.94 | 421.2401 | 1 | 13.63 | 5446 | 6.2e2 | 1 | 1 | 552 | 555 |  |  | DB Search |
| E.TPGF.I | N | 15.9 | 420.2009 | 4 | 2.59 | 421.2082 | 1 | 12.63 | 4943 | 3.01e3 | 1 | 1 | 1925 | 1928 |  |  | DB Search |
| R.EGVPGI.S | N | 15.68 | 570.3013 | 6 | -4.01 | 571.3049 | 1 | 22.52 | 8280 | 2.27e3 | 1 | 1 | 1366 | 1371 |  |  | DB Search |
| L.STPSPTS.L | Y | 15.6 | 675.3075 | 7 | 2.69 | 676.3149 | 1 | 13.27 | 5227 | 2.18e2 | 1 | 1 | 1953 | 1959 |  |  | DB Search |
| total 20 peptides |
| --- |

Best Unique PSM (Scan POS\_R15-1.wiff:5227, m/z=676.3149, z=1, RT=13.27, ppm=0.2):


C4R075|C4R075\_KOMPG

back to list

  

| Protein Coverage
| Supporting Peptides
| Best Unique PSM
|

Protein Coverage:

Supporting Peptides:

| Peptide | Uniq | -10lgP | Mass | Length | ppm | m/z | z | RT | Scan | Area POS\_R15-1 | #Feature | #Feature POS\_R15-1 | Start | End | PTM | AScore | Found By |
| --- | --- | --- | --- | --- | --- | --- | --- | --- | --- | --- | --- | --- | --- | --- | --- | --- | --- |
| Q.AIGL.S | N | 25.12 | 372.2372 | 4 | -5.1 | 373.2417 | 1 | 21.84 | 8058 | 0 | 0 | 0 | 609 | 612 |  |  | DB Search |
| F.EIIR.D | N | 25.05 | 529.3224 | 4 | -4.56 | 530.3259 | 1 | 17.34 | 6855 | 6.68e3 | 1 | 1 | 550 | 553 |  |  | DB Search |
| K.VLGS.T | N | 24.2 | 374.2165 | 4 | -4.06 | 375.2213 | 1 | 5.20 | 2012 | 6.27e3 | 1 | 1 | 544 | 547 |  |  | DB Search |
| G.EIVR.G | N | 23.24 | 515.3067 | 4 | -4.7 | 516.3103 | 1 | 11.12 | 4401 | 1.13e4 | 1 | 1 | 787 | 790 |  |  | DB Search |
| W.VLSG.K | N | 21.25 | 374.2165 | 4 | -4.06 | 375.2213 | 1 | 5.20 | 1991 | 6.27e3 | 1 | 1 | 629 | 632 |  |  | DB Search |
| M.ALVE.K | N | 20.45 | 430.2427 | 4 | -5.9 | 431.2464 | 1 | 12.49 | 4901 | 0 | 0 | 0 | 539 | 542 |  |  | DB Search |
| G.VVDI.S | N | 19.9 | 444.2584 | 4 | 0.77 | 445.2649 | 1 | 17.32 | 6776 | 2.15e3 | 1 | 1 | 279 | 282 |  |  | DB Search |
| T.SIDT.T | N | 19.03 | 434.2013 | 4 | -3.95 | 435.2057 | 1 | 15.10 | 5999 | 0 | 0 | 0 | 750 | 753 |  |  | DB Search |
| K.IDNF.Y | N | 18.37 | 507.2329 | 4 | -5.34 | 508.2362 | 1 | 14.74 | 5819 | 0 | 0 | 0 | 794 | 797 |  |  | DB Search |
| D.E(-18.01)STYQI.F | Y | 16.99 | 721.3283 | 6 | 0.24 | 722.3339 | 1 | 23.63 | 8585 | 1.9e3 | 1 | 1 | 236 | 241 | Pyro-glu from E | E1:Pyro-glu from E:1000 | DB Search |
| P.IGVT.Y | N | 16.69 | 388.2322 | 4 | -3.81 | 389.237 | 1 | 9.87 | 3731 | 6.08e3 | 1 | 1 | 227 | 230 |  |  | DB Search |
| F.YSSV.D | N | 15.06 | 454.2064 | 4 | -3.39 | 455.211 | 1 | 23.68 | 8615 | 0 | 0 | 0 | 798 | 801 |  |  | DB Search |
| total 12 peptides |
| --- |

Best Unique PSM (Scan POS\_R15-1.wiff:8585, m/z=722.3339, z=1, RT=23.63, ppm=-2.24):


C4QXM2|C4QXM2\_KOMPG

back to list

  

| Protein Coverage
| Supporting Peptides
| Best Unique PSM
|

Protein Coverage:

Supporting Peptides:

| Peptide | Uniq | -10lgP | Mass | Length | ppm | m/z | z | RT | Scan | Area POS\_R15-1 | #Feature | #Feature POS\_R15-1 | Start | End | PTM | AScore | Found By |
| --- | --- | --- | --- | --- | --- | --- | --- | --- | --- | --- | --- | --- | --- | --- | --- | --- | --- |
| G.HFT.Q | N | 23.64 | 403.1856 | 3 | -0.83 | 404.1915 | 1 | 13.94 | 5543 | 0 | 0 | 0 | 243 | 245 |  |  | DB Search |
| E.STSP.T | N | 19.15 | 390.1751 | 4 | -0.08 | 391.1813 | 1 | 5.92 | 2360 | 3.31e2 | 1 | 1 | 124 | 127 |  |  | DB Search |
| G.VDGL.E | N | 18.71 | 402.2114 | 4 | 1.69 | 403.2184 | 1 | 10.06 | 3921 | 2.85e3 | 1 | 1 | 172 | 175 |  |  | DB Search |
| S.STLA.G | N | 17.83 | 390.2114 | 4 | -5.32 | 391.2157 | 1 | 6.25 | 2360 | 1.07e4 | 1 | 1 | 49 | 52 |  |  | DB Search |
| G.CAFK.Y | Y | 17.09 | 467.2202 | 4 | -1.49 | 468.2256 | 1 | 22.80 | 8358 | 0 | 0 | 0 | 257 | 260 |  |  | DB Search |
| total 5 peptides |
| --- |

Best Unique PSM (Scan POS\_R15-1.wiff:8358, m/z=468.2172, z=1, RT=22.99, ppm=-3.98):


C4R9E3|C4R9E3\_KOMPG

back to list

  

| Protein Coverage
| Supporting Peptides
| Best Unique PSM
|

Protein Coverage:

Supporting Peptides:

| Peptide | Uniq | -10lgP | Mass | Length | ppm | m/z | z | RT | Scan | Area POS\_R15-1 | #Feature | #Feature POS\_R15-1 | Start | End | PTM | AScore | Found By |
| --- | --- | --- | --- | --- | --- | --- | --- | --- | --- | --- | --- | --- | --- | --- | --- | --- | --- |
| R.LSLE.Q | N | 25.68 | 460.2533 | 4 | -2.19 | 461.2584 | 1 | 13.54 | 5303 | 1.35e4 | 1 | 1 | 553 | 556 |  |  | DB Search |
| A.VLGS.P | N | 24.2 | 374.2165 | 4 | -4.06 | 375.2213 | 1 | 5.20 | 2012 | 6.27e3 | 1 | 1 | 168 | 171 |  |  | DB Search |
| T.AIAGSIGIG.G | Y | 23.09 | 757.4334 | 9 | -4.57 | 758.4353 | 1 | 27.03 | 9540 | 2.32e3 | 1 | 1 | 401 | 409 |  |  | DB Search |
| S.VLLP.H | N | 22.17 | 440.2998 | 4 | -5.96 | 441.3034 | 1 | 30.99 | 10567 | 1.25e3 | 1 | 1 | 81 | 84 |  |  | DB Search |
| S.SSIF.G | N | 21.63 | 452.2271 | 4 | -3.7 | 453.2316 | 1 | 22.49 | 8262 | 1.33e3 | 1 | 1 | 4 | 7 |  |  | DB Search |
| T.TSG.G | N | 21.44 | 263.1117 | 3 | 2.31 | 264.119 | 1 | 3.71 | 1537 | 5.72e3 | 1 | 1 | 525 | 527 |  |  | DB Search |
| A.AAVL.G | N | 18.4 | 372.2372 | 4 | -5.92 | 373.2414 | 1 | 12.97 | 5130 | 2.29e3 | 1 | 1 | 166 | 169 |  |  | DB Search |
| G.IGPV.S | N | 18.01 | 384.2372 | 4 | -2.96 | 385.2424 | 1 | 11.56 | 4504 | 2.76e3 | 1 | 1 | 477 | 480 |  |  | DB Search |
| V.RSF.G | N | 17.5 | 408.2121 | 3 | -1.76 | 409.2177 | 1 | 9.17 | 3526 | 0 | 0 | 0 | 494 | 496 |  |  | DB Search |
| I.SPK.T | N | 16.89 | 330.1903 | 3 | -8.46 | 331.194 | 1 | 5.91 | 2235 | 5.17e3 | 1 | 1 | 397 | 399 |  |  | DB Search |
| Q.LDEL.F | N | 16.03 | 488.2482 | 4 | -7.44 | 489.2506 | 1 | 15.57 | 6209 | 9.61e2 | 1 | 1 | 558 | 561 |  |  | DB Search |
| A.AMY.L | N | 15.75 | 383.1515 | 3 | -9.49 | 384.1542 | 1 | 17.73 | 6972 | 0 | 0 | 0 | 465 | 467 |  |  | DB Search |
| S.VVPM.L | N | 15.69 | 444.2406 | 4 | -1.25 | 445.2462 | 1 | 22.52 | 8277 | 0 | 0 | 0 | 227 | 230 |  |  | DB Search |
| total 13 peptides |
| --- |

Best Unique PSM (Scan POS\_R15-1.wiff:9540, m/z=758.4353, z=1, RT=27.03, ppm=-7.05):


C4QZ32|C4QZ32\_KOMPG

back to list

  

| Protein Coverage
| Supporting Peptides
| Best Unique PSM
|

Protein Coverage:

Supporting Peptides:

| Peptide | Uniq | -10lgP | Mass | Length | ppm | m/z | z | RT | Scan | Area POS\_R15-1 | #Feature | #Feature POS\_R15-1 | Start | End | PTM | AScore | Found By |
| --- | --- | --- | --- | --- | --- | --- | --- | --- | --- | --- | --- | --- | --- | --- | --- | --- | --- |
| E.EILR.R | N | 25.05 | 529.3224 | 4 | -4.56 | 530.3259 | 1 | 17.34 | 6855 | 6.68e3 | 1 | 1 | 109 | 112 |  |  | DB Search |
| N.GVSI.M | N | 22.74 | 374.2165 | 4 | -2.51 | 375.2219 | 1 | 13.91 | 5529 | 2.07e3 | 1 | 1 | 166 | 169 |  |  | DB Search |
| M.VTVL.Y | N | 21.25 | 430.2791 | 4 | -6.35 | 431.2826 | 1 | 27.75 | 9755 | 1.56e3 | 1 | 1 | 70 | 73 |  |  | DB Search |
| R.AGYGT.F | Y | 16.64 | 467.2016 | 5 | -4.01 | 468.2058 | 1 | 3.66 | 1562 | 0 | 0 | 0 | 114 | 118 |  |  | DB Search |
| L.VEIA.F | N | 15.81 | 430.2427 | 4 | -4.19 | 431.2471 | 1 | 15.22 | 6032 | 0 | 0 | 0 | 226 | 229 |  |  | DB Search |
| total 5 peptides |
| --- |

Best Unique PSM (Scan POS\_R15-1.wiff:1562, m/z=468.2058, z=1, RT=3.66, ppm=-6.49):


C4QVT9|C4QVT9\_KOMPG

back to list

  

| Protein Coverage
| Supporting Peptides
| Best Unique PSM
|

Protein Coverage:

Supporting Peptides:

| Peptide | Uniq | -10lgP | Mass | Length | ppm | m/z | z | RT | Scan | Area POS\_R15-1 | #Feature | #Feature POS\_R15-1 | Start | End | PTM | AScore | Found By |
| --- | --- | --- | --- | --- | --- | --- | --- | --- | --- | --- | --- | --- | --- | --- | --- | --- | --- |
| D.NGL.P | N | 28.25 | 302.159 | 3 | -3.76 | 303.1644 | 1 | 6.06 | 2374 | 3.14e3 | 1 | 1 | 61 | 63 |  |  | DB Search |
| D.NIL.L | N | 28.22 | 358.2216 | 3 | -4.68 | 359.2263 | 1 | 19.18 | 7326 | 4.19e3 | 1 | 1 | 765 | 767 |  |  | DB Search |
| Q.AIGL.V | N | 25.12 | 372.2372 | 4 | -5.1 | 373.2417 | 1 | 21.84 | 8058 | 0 | 0 | 0 | 1171 | 1174 |  |  | DB Search |
| F.VGGL.Y | N | 24.87 | 344.2059 | 4 | -5.22 | 345.2106 | 1 | 10.74 | 4195 | 1.51e3 | 1 | 1 | 1212 | 1215 |  |  | DB Search |
| D.EF.A | N | 22.86 | 294.1216 | 2 | -6.92 | 295.1261 | 1 | 9.67 | 3652 | 3.06e4 | 1 | 1 | 332 | 333 |  |  | DB Search |
| S.EV.R | N | 22.06 | 246.1216 | 2 | -3.05 | 247.1275 | 1 | 3.37 | 1479 | 1.39e4 | 2 | 2 | 168 | 169 |  |  | DB Search |
| T.YGR.T | N | 21.6 | 394.1965 | 3 | -4.25 | 395.2011 | 1 | 5.35 | 1961 | 1.67e4 | 1 | 1 | 1226 | 1228 |  |  | DB Search |
| Y.YNP.A | N | 21.45 | 392.1696 | 3 | -3.75 | 393.1744 | 1 | 8.45 | 3226 | 1.39e2 | 1 | 1 | 470 | 472 |  |  | DB Search |
| I.ITDL.T | N | 20.88 | 460.2533 | 4 | -0.2 | 461.2593 | 1 | 14.80 | 5851 | 7.36e2 | 1 | 1 | 1243 | 1246 |  |  | DB Search |
| R.LAR.I | N | 20.74 | 358.2328 | 3 | -3.48 | 359.238 | 1 | 2.08 | 887 | 2.98e3 | 1 | 1 | 1163 | 1165 |  |  | DB Search |
| T.ALVE.S | N | 20.45 | 430.2427 | 4 | -5.9 | 431.2464 | 1 | 12.49 | 4901 | 0 | 0 | 0 | 560 | 563 |  |  | DB Search |
| F.SAE.E | N | 20.39 | 305.1223 | 3 | 3.83 | 306.13 | 1 | 8.24 | 3110 | 9.42e3 | 2 | 2 | 547 | 549 |  |  | DB Search |
| V.SGLL.E | N | 19.42 | 388.2322 | 4 | -5.15 | 389.2365 | 1 | 17.34 | 6766 | 1.43e3 | 1 | 1 | 683 | 686 |  |  | DB Search |
| R.FPS.Y | N | 19.27 | 349.1638 | 3 | -4.81 | 350.1685 | 1 | 16.32 | 6461 | 1.41e3 | 1 | 1 | 171 | 173 |  |  | DB Search |
| S.TVVI.V | N | 19.26 | 430.2791 | 4 | -6.78 | 431.2824 | 1 | 27.81 | 9764 | 1.56e3 | 1 | 1 | 126 | 129 |  |  | DB Search |
| F.VTAT.I | N | 19.15 | 390.2114 | 4 | -9 | 391.2142 | 1 | 7.51 | 2842 | 9.19e2 | 1 | 1 | 76 | 79 |  |  | DB Search |
| F.EM.L | N | 19.13 | 278.0936 | 2 | 0.1 | 279.1003 | 1 | 3.52 | 1504 | 7.14e3 | 1 | 1 | 879 | 880 |  |  | DB Search |
| N.RT.S | N | 19.04 | 275.1593 | 2 | 3.6 | 276.1669 | 1 | 5.27 | 2115 | 2.64e3 | 1 | 1 | 298 | 299 |  |  | DB Search |
| S.PR.D | N | 18.98 | 271.1644 | 2 | -8.93 | 272.1686 | 1 | 5.03 | 2023 | 0 | 0 | 0 | 66 | 67 |  |  | DB Search |
| L.IRV.L | N | 18.9 | 386.2641 | 3 | -1.28 | 387.27 | 1 | 27.77 | 9760 | 0 | 0 | 0 | 759 | 761 |  |  | DB Search |
| S.TW.T | N | 18.09 | 305.1375 | 2 | -1.88 | 306.1435 | 1 | 9.22 | 3551 | 5.58e3 | 1 | 1 | 1013 | 1014 |  |  | DB Search |
| P.ESF.R | N | 17.93 | 381.1536 | 3 | -4.82 | 382.1581 | 1 | 5.07 | 1981 | 2.63e3 | 1 | 1 | 353 | 355 |  |  | DB Search |
| K.YP.Q | N | 17.92 | 278.1266 | 2 | -4.14 | 279.1321 | 1 | 3.49 | 1522 | 2.89e2 | 1 | 1 | 362 | 363 |  |  | DB Search |
| H.HP.E | N | 17.77 | 252.1222 | 2 | -2.49 | 253.1283 | 1 | 5.54 | 2216 | 1.25e4 | 1 | 1 | 457 | 458 |  |  | DB Search |
| S.TSN.F | N | 17.54 | 320.1332 | 3 | 3.34 | 321.1407 | 1 | 8.11 | 3024 | 0 | 0 | 0 | 444 | 446 |  |  | DB Search |
| Q.LLTD.A | N | 17.52 | 460.2533 | 4 | -9.09 | 461.2552 | 1 | 10.16 | 3965 | 9.46e2 | 1 | 1 | 266 | 269 |  |  | DB Search |
| F.ADL.E | N | 17.27 | 317.1587 | 3 | -4.79 | 318.1636 | 1 | 3.30 | 1336 | 3.11e3 | 1 | 1 | 993 | 995 |  |  | DB Search |
| D.FMR.L | N | 17.07 | 452.2206 | 3 | -0.76 | 453.2264 | 1 | 15.57 | 6149 | 2.05e2 | 1 | 1 | 1160 | 1162 |  |  | DB Search |
| T.WT.S | N | 17.04 | 305.1375 | 2 | -1.68 | 306.1436 | 1 | 9.15 | 3517 | 5.58e3 | 1 | 1 | 1014 | 1015 |  |  | DB Search |
| F.LSAI.K | N | 16.63 | 402.2478 | 4 | -8.97 | 403.2505 | 1 | 9.42 | 3655 | 1.65e3 | 1 | 1 | 179 | 182 |  |  | DB Search |
| S.TV.V | N | 16.54 | 218.1266 | 2 | -6.32 | 219.132 | 1 | 6.06 | 2461 | 9.87e2 | 1 | 1 | 126 | 127 |  |  | DB Search |
| H.HHVQ.M | Y | 16.38 | 519.2554 | 4 | 7.17 | 520.2651 | 1 | 14.97 | 5921 | 0 | 0 | 0 | 1083 | 1086 |  |  | DB Search |
| V.LSVE.S | N | 16.12 | 446.2376 | 4 | -1.26 | 447.2433 | 1 | 9.47 | 3656 | 0 | 0 | 0 | 27 | 30 |  |  | DB Search |
| V.EAG.S | N | 16.1 | 275.1117 | 3 | 5.02 | 276.1197 | 1 | 9.57 | 3712 | 3.1e3 | 1 | 1 | 1065 | 1067 |  |  | DB Search |
| L.NHI.K | N | 15.79 | 382.1965 | 3 | -0.71 | 383.2025 | 1 | 19.27 | 7368 | 1.28e3 | 1 | 1 | 701 | 703 |  |  | DB Search |
| S.LDVG.D | N | 15.79 | 402.2114 | 4 | -1.2 | 403.2172 | 1 | 9.22 | 3553 | 3.61e3 | 1 | 1 | 615 | 618 |  |  | DB Search |
| S.KST.S | N | 15.47 | 334.1852 | 3 | 0.84 | 335.192 | 1 | 54.68 | 16215 | 3.66e2 | 1 | 1 | 442 | 444 |  |  | DB Search |
| H.DGIID.R | N | 15.37 | 531.254 | 5 | 7.19 | 532.2638 | 1 | 17.21 | 6856 | 7.84e2 | 1 | 1 | 416 | 420 |  |  | DB Search |
| L.PVE.E | N | 15.19 | 343.1743 | 3 | -3.43 | 344.1796 | 1 | 12.76 | 5037 | 6.34e2 | 1 | 1 | 944 | 946 |  |  | DB Search |
| total 39 peptides |
| --- |

Best Unique PSM (Scan POS\_R15-1.wiff:5921, m/z=520.2651, z=1, RT=14.97, ppm=4.68):


C4QYQ0|C4QYQ0\_KOMPG

back to list

  

| Protein Coverage
| Supporting Peptides
| Best Unique PSM
|

Protein Coverage:

Supporting Peptides:

| Peptide | Uniq | -10lgP | Mass | Length | ppm | m/z | z | RT | Scan | Area POS\_R15-1 | #Feature | #Feature POS\_R15-1 | Start | End | PTM | AScore | Found By |
| --- | --- | --- | --- | --- | --- | --- | --- | --- | --- | --- | --- | --- | --- | --- | --- | --- | --- |
| N.AAGQY.P | Y | 32.48 | 508.2281 | 5 | 0.3 | 509.2343 | 1 | 4.42 | 1758 | 1.42e4 | 1 | 1 | 324 | 328 |  |  | DB Search |
| Q.PVIV.S | N | 23.27 | 426.2842 | 4 | 4.41 | 427.2923 | 1 | 20.36 | 7664 | 0 | 0 | 0 | 499 | 502 |  |  | DB Search |
| D.SPT.F | N | 23.16 | 303.143 | 3 | 6.39 | 304.1515 | 1 | 7.14 | 2731 | 5.4e3 | 2 | 2 | 90 | 92 |  |  | DB Search |
| G.EW.F | N | 22.33 | 333.1325 | 2 | -3.27 | 334.1378 | 1 | 12.76 | 5036 | 2.08e4 | 1 | 1 | 186 | 187 |  |  | DB Search |
| K.PGE.W | N | 21.28 | 301.1274 | 3 | 1.88 | 302.1345 | 1 | 2.04 | 869 | 2.57e3 | 1 | 1 | 184 | 186 |  |  | DB Search |
| R.ELA.Y | N | 21.09 | 331.1743 | 3 | -5.12 | 332.1791 | 1 | 6.98 | 2655 | 4.41e3 | 1 | 1 | 227 | 229 |  |  | DB Search |
| P.IAR.D | N | 20.74 | 358.2328 | 3 | -3.48 | 359.238 | 1 | 2.08 | 887 | 2.98e3 | 1 | 1 | 84 | 86 |  |  | DB Search |
| F.SSD.C | N | 20.1 | 307.1016 | 3 | 8.02 | 308.1105 | 1 | 10.67 | 4164 | 0 | 0 | 0 | 214 | 216 |  |  | DB Search |
| D.PH.L | N | 19.52 | 252.1222 | 2 | -5.45 | 253.1275 | 1 | 5.57 | 2095 | 1.25e4 | 1 | 1 | 296 | 297 |  |  | DB Search |
| E.PR.T | N | 18.98 | 271.1644 | 2 | -8.93 | 272.1686 | 1 | 5.03 | 2023 | 0 | 0 | 0 | 473 | 474 |  |  | DB Search |
| N.EIG.F | N | 18.95 | 317.1587 | 3 | -4.31 | 318.1638 | 1 | 5.49 | 2177 | 4.11e3 | 1 | 1 | 107 | 109 |  |  | DB Search |
| G.LRV.Y | N | 18.9 | 386.2641 | 3 | -1.28 | 387.27 | 1 | 27.77 | 9760 | 0 | 0 | 0 | 209 | 211 |  |  | DB Search |
| S.Q(-17.03)SF.I | N | 18.71 | 363.143 | 3 | 2.8 | 364.1504 | 1 | 14.74 | 5815 | 0 | 0 | 0 | 63 | 65 | Pyro-glu from Q | Q1:Pyro-glu from Q:1000 | DB Search |
| Q.SVGI.A | N | 18.49 | 374.2165 | 4 | -4.96 | 375.221 | 1 | 14.62 | 5748 | 1.93e2 | 1 | 1 | 269 | 272 |  |  | DB Search |
| E.YP.P | N | 17.92 | 278.1266 | 2 | -4.14 | 279.1321 | 1 | 3.49 | 1522 | 2.89e2 | 1 | 1 | 204 | 205 |  |  | DB Search |
| T.HP.N | N | 17.77 | 252.1222 | 2 | -2.49 | 253.1283 | 1 | 5.54 | 2216 | 1.25e4 | 1 | 1 | 10 | 11 |  |  | DB Search |
| S.TSN.F | N | 17.54 | 320.1332 | 3 | 3.34 | 321.1407 | 1 | 8.11 | 3024 | 0 | 0 | 0 | 423 | 425 |  |  | DB Search |
| L.E(-18.01)PR.T | N | 16.87 | 382.1965 | 3 | -0.37 | 383.2026 | 1 | 2.61 | 1082 | 1.62e4 | 1 | 1 | 472 | 474 | Pyro-glu from E | E1:Pyro-glu from E:1000 | DB Search |
| V.KSVH.T | N | 16 | 469.2649 | 4 | -2.56 | 470.2698 | 1 | 1.09 | 257 | 3.02e4 | 1 | 1 | 364 | 367 |  |  | DB Search |
| P.TFT.L | N | 15.92 | 367.1743 | 3 | -7.47 | 368.1779 | 1 | 8.54 | 3263 | 0 | 0 | 0 | 92 | 94 |  |  | DB Search |
| H.ITET.K | N | 15.47 | 462.2326 | 4 | 1.41 | 463.2393 | 1 | 15.37 | 6065 | 2.41e2 | 1 | 1 | 414 | 417 |  |  | DB Search |
| K.KST.T | N | 15.47 | 334.1852 | 3 | 0.84 | 335.192 | 1 | 54.68 | 16215 | 3.66e2 | 1 | 1 | 57 | 59 |  |  | DB Search |
| D.NGV.I | N | 15.37 | 288.1434 | 3 | 8 | 289.1522 | 1 | 5.91 | 2301 | 6.8e3 | 1 | 1 | 515 | 517 |  |  | DB Search |
| total 23 peptides |
| --- |

Best Unique PSM (Scan POS\_R15-1.wiff:1758, m/z=509.2319, z=1, RT=4.48, ppm=-2.19):


C4R6L3|C4R6L3\_KOMPG

back to list

  

| Protein Coverage
| Supporting Peptides
| Best Unique PSM
|

Protein Coverage:

Supporting Peptides:

| Peptide | Uniq | -10lgP | Mass | Length | ppm | m/z | z | RT | Scan | Area POS\_R15-1 | #Feature | #Feature POS\_R15-1 | Start | End | PTM | AScore | Found By |
| --- | --- | --- | --- | --- | --- | --- | --- | --- | --- | --- | --- | --- | --- | --- | --- | --- | --- |
| S.TLAV.L | N | 20.37 | 402.2478 | 4 | -1.76 | 403.2534 | 1 | 15.04 | 5856 | 1.07e4 | 1 | 1 | 179 | 182 |  |  | DB Search |
| M.STLA.V | N | 17.83 | 390.2114 | 4 | -5.32 | 391.2157 | 1 | 6.25 | 2360 | 1.07e4 | 1 | 1 | 178 | 181 |  |  | DB Search |
| L.AVVA.N | N | 16.9 | 358.2216 | 4 | -4.99 | 359.2262 | 1 | 6.95 | 2675 | 0 | 0 | 0 | 132 | 135 |  |  | DB Search |
| A.AAQGCL.I | Y | 15.92 | 561.2581 | 6 | 0.37 | 562.2642 | 1 | 13.57 | 5311 | 1.92e2 | 1 | 1 | 49 | 54 |  |  | DB Search |
| total 4 peptides |
| --- |

Best Unique PSM (Scan POS\_R15-1.wiff:5311, m/z=562.2642, z=1, RT=13.57, ppm=-2.11):


C4R6L5|C4R6L5\_KOMPG

back to list

  

| Protein Coverage
| Supporting Peptides
| Best Unique PSM
|

Protein Coverage:

Supporting Peptides:

| Peptide | Uniq | -10lgP | Mass | Length | ppm | m/z | z | RT | Scan | Area POS\_R15-1 | #Feature | #Feature POS\_R15-1 | Start | End | PTM | AScore | Found By |
| --- | --- | --- | --- | --- | --- | --- | --- | --- | --- | --- | --- | --- | --- | --- | --- | --- | --- |
| A.TGALVVG.S | Y | 21.94 | 615.3591 | 7 | -0.84 | 616.3644 | 1 | 13.63 | 5356 | 2.79e3 | 1 | 1 | 113 | 119 |  |  | DB Search |
| L.QSVA.K | N | 19.54 | 403.2067 | 4 | -0.86 | 404.2126 | 1 | 2.86 | 1199 | 5.58e3 | 1 | 1 | 16 | 19 |  |  | DB Search |
| V.TAY.E | N | 19.3 | 353.1587 | 3 | -1.32 | 354.1646 | 1 | 12.51 | 4913 | 1.39e3 | 1 | 1 | 23 | 25 |  |  | DB Search |
| F.LIQP.L | N | 19.13 | 469.29 | 4 | -8.42 | 470.2922 | 1 | 12.16 | 4756 | 6.43e3 | 1 | 1 | 319 | 322 |  |  | DB Search |
| F.ISGY.S | N | 17.88 | 438.2114 | 4 | -3.08 | 439.2163 | 1 | 7.95 | 2900 | 3.53e4 | 1 | 1 | 329 | 332 |  |  | DB Search |
| K.TVTA.Y | N | 17.32 | 390.2114 | 4 | -8.37 | 391.2145 | 1 | 7.97 | 2951 | 0 | 0 | 0 | 21 | 24 |  |  | DB Search |
| M.AMY.N | N | 15.75 | 383.1515 | 3 | -9.49 | 384.1542 | 1 | 17.73 | 6972 | 0 | 0 | 0 | 413 | 415 |  |  | DB Search |
| I.AGISI.V | N | 15.43 | 459.2693 | 5 | -3.77 | 460.2737 | 1 | 22.08 | 8169 | 4.69e2 | 1 | 1 | 235 | 239 |  |  | DB Search |
| total 8 peptides |
| --- |

Best Unique PSM (Scan POS\_R15-1.wiff:5356, m/z=616.3644, z=1, RT=13.63, ppm=-3.32):


C4QWL7|C4QWL7\_KOMPG

back to list

  

| Protein Coverage
| Supporting Peptides
| Best Unique PSM
|

Protein Coverage:

Supporting Peptides:

| Peptide | Uniq | -10lgP | Mass | Length | ppm | m/z | z | RT | Scan | Area POS\_R15-1 | #Feature | #Feature POS\_R15-1 | Start | End | PTM | AScore | Found By |
| --- | --- | --- | --- | --- | --- | --- | --- | --- | --- | --- | --- | --- | --- | --- | --- | --- | --- |
| R.VVGL.S | N | 26.8 | 386.2529 | 4 | -6 | 387.2569 | 1 | 17.69 | 6924 | 2.17e3 | 1 | 1 | 440 | 443 |  |  | DB Search |
| R.ETW.F | N | 26.2 | 434.1801 | 3 | -4.77 | 435.1843 | 1 | 12.45 | 4871 | 1.27e3 | 1 | 1 | 1874 | 1876 |  |  | DB Search |
| F.WNPH.I | N | 24.26 | 552.2445 | 4 | 6.18 | 553.2538 | 1 | 5.42 | 2167 | 2.61e2 | 1 | 1 | 1681 | 1684 |  |  | DB Search |
| M.MGR.A | N | 20.83 | 362.1736 | 3 | 6.82 | 363.1825 | 1 | 3.30 | 1420 | 1.5e3 | 1 | 1 | 1480 | 1482 |  |  | DB Search |
| R.GSVI.E | N | 20.47 | 374.2165 | 4 | -5.12 | 375.2209 | 1 | 10.69 | 4154 | 1e3 | 1 | 1 | 417 | 420 |  |  | DB Search |
| A.QFF.W | N | 19.56 | 440.206 | 3 | 3.92 | 441.2139 | 1 | 19.15 | 7313 | 0 | 0 | 0 | 1013 | 1015 |  |  | DB Search |
| E.AFR.D | N | 19.1 | 392.2172 | 3 | -7.11 | 393.2207 | 1 | 6.33 | 2475 | 0 | 0 | 0 | 1467 | 1469 |  |  | DB Search |
| V.ATSTLA.W | Y | 19.03 | 562.2962 | 6 | -9.74 | 563.2966 | 1 | 8.25 | 3118 | 0 | 0 | 0 | 1438 | 1443 |  |  | DB Search |
| A.AAVI.I | N | 18.4 | 372.2372 | 4 | -5.92 | 373.2414 | 1 | 12.97 | 5130 | 2.29e3 | 1 | 1 | 618 | 621 |  |  | DB Search |
| T.STLA.W | N | 17.83 | 390.2114 | 4 | -5.32 | 391.2157 | 1 | 6.25 | 2360 | 1.07e4 | 1 | 1 | 607 | 610 |  |  | DB Search |
| Y.KHF.L | N | 17.4 | 430.2328 | 3 | 0.78 | 431.2394 | 1 | 20.46 | 7703 | 1.69e2 | 1 | 1 | 1507 | 1509 |  |  | DB Search |
| A.RIC.R | N | 16.16 | 390.2049 | 3 | 4.43 | 391.213 | 1 | 3.37 | 1421 | 1.01e3 | 1 | 1 | 885 | 887 |  |  | DB Search |
| K.Q(-17.03)QLIG.V | N | 16.12 | 540.2908 | 5 | -4.73 | 541.2941 | 1 | 23.69 | 8617 | 1.76e4 | 1 | 1 | 479 | 483 | Pyro-glu from Q | Q1:Pyro-glu from Q:1000 | DB Search |
| K.IEAF.R | N | 15.58 | 478.2427 | 4 | -6.65 | 479.2456 | 1 | 20.19 | 7599 | 4.22e2 | 1 | 1 | 1465 | 1468 |  |  | DB Search |
| K.QGGF.T | N | 15.34 | 407.1805 | 4 | -8.08 | 408.1834 | 1 | 8.67 | 3345 | 0 | 0 | 0 | 632 | 635 |  |  | DB Search |
| Q.LGVP.A | N | 15.32 | 384.2372 | 4 | -3.68 | 385.2422 | 1 | 16.13 | 6408 | 0 | 0 | 0 | 1814 | 1817 |  |  | DB Search |
| K.YHG.N | N | 15.26 | 375.1543 | 3 | -7.53 | 376.1578 | 1 | 4.73 | 1840 | 0 | 0 | 0 | 1008 | 1010 |  |  | DB Search |
| total 17 peptides |
| --- |

Best Unique PSM (Scan POS\_R15-1.wiff:3118, m/z=563.2959, z=1, RT=8.30, ppm=-12.22):


C4QX04|C4QX04\_KOMPG

back to list

  

| Protein Coverage
| Supporting Peptides
| Best Unique PSM
|

Protein Coverage:

Supporting Peptides:

| Peptide | Uniq | -10lgP | Mass | Length | ppm | m/z | z | RT | Scan | Area POS\_R15-1 | #Feature | #Feature POS\_R15-1 | Start | End | PTM | AScore | Found By |
| --- | --- | --- | --- | --- | --- | --- | --- | --- | --- | --- | --- | --- | --- | --- | --- | --- | --- |
| Y.LTGL.L | N | 27.55 | 402.2478 | 4 | -1.76 | 403.2534 | 1 | 15.04 | 5964 | 1.56e4 | 2 | 2 | 528 | 531 |  |  | DB Search |
| A.TIVL.L | N | 26.01 | 444.2948 | 4 | -4 | 445.2992 | 1 | 28.31 | 9909 | 1.89e3 | 1 | 1 | 162 | 165 |  |  | DB Search |
| P.QIEQP.L | N | 19.65 | 613.3071 | 5 | -8.19 | 614.3079 | 1 | 8.07 | 2985 | 2.59e3 | 1 | 1 | 134 | 138 |  |  | DB Search |
| Q.LVKNST.N | Y | 17.53 | 660.3806 | 6 | -5.41 | 661.3827 | 1 | 35.28 | 11760 | 7.58e2 | 1 | 1 | 104 | 109 |  |  | DB Search |
| D.SKVH.A | N | 16.42 | 469.2649 | 4 | -2.56 | 470.2698 | 1 | 1.09 | 269 | 3.02e4 | 1 | 1 | 234 | 237 |  |  | DB Search |
| total 5 peptides |
| --- |

Best Unique PSM (Scan POS\_R15-1.wiff:11760, m/z=661.3838, z=2, RT=35.31, ppm=-7.9):


C4QWA7|C4QWA7\_KOMPG

back to list

  

| Protein Coverage
| Supporting Peptides
| Best Unique PSM
|

Protein Coverage:

Supporting Peptides:

| Peptide | Uniq | -10lgP | Mass | Length | ppm | m/z | z | RT | Scan | Area POS\_R15-1 | #Feature | #Feature POS\_R15-1 | Start | End | PTM | AScore | Found By |
| --- | --- | --- | --- | --- | --- | --- | --- | --- | --- | --- | --- | --- | --- | --- | --- | --- | --- |
| P.LTGL.S | N | 27.55 | 402.2478 | 4 | -1.76 | 403.2534 | 1 | 15.04 | 5964 | 1.56e4 | 2 | 2 | 75 | 78 |  |  | DB Search |
| Q.LPTP.V | N | 25.97 | 426.2478 | 4 | -3.74 | 427.2524 | 1 | 12.64 | 4915 | 1.93e3 | 1 | 1 | 221 | 224 |  |  | DB Search |
| L.SITP.G | N | 19.55 | 416.2271 | 4 | 2 | 417.2342 | 1 | 10.25 | 4029 | 3.69e3 | 1 | 1 | 86 | 89 |  |  | DB Search |
| S.VSPP.N | N | 17.71 | 398.2165 | 4 | -2.14 | 399.222 | 1 | 8.11 | 3026 | 0 | 0 | 0 | 4 | 7 |  |  | DB Search |
| Q.LGGV.E | N | 15.94 | 344.2059 | 4 | 0.19 | 345.2124 | 1 | 5.48 | 2143 | 3.05e3 | 1 | 1 | 348 | 351 |  |  | DB Search |
| L.IDQLGGVE.N | Y | 15.06 | 829.4181 | 8 | -6.03 | 830.4183 | 1 | 14.94 | 5903 | 3.76e3 | 1 | 1 | 345 | 352 |  |  | DB Search |
| total 6 peptides |
| --- |

Best Unique PSM (Scan POS\_R15-1.wiff:5903, m/z=830.4235, z=1, RT=15.30, ppm=-8.51):


C4R9D9|C4R9D9\_KOMPG

back to list

  

| Protein Coverage
| Supporting Peptides
| Best Unique PSM
|

Protein Coverage:

Supporting Peptides:

| Peptide | Uniq | -10lgP | Mass | Length | ppm | m/z | z | RT | Scan | Area POS\_R15-1 | #Feature | #Feature POS\_R15-1 | Start | End | PTM | AScore | Found By |
| --- | --- | --- | --- | --- | --- | --- | --- | --- | --- | --- | --- | --- | --- | --- | --- | --- | --- |
| T.TIVI.A | N | 26.01 | 444.2948 | 4 | -4 | 445.2992 | 1 | 28.31 | 9909 | 1.89e3 | 1 | 1 | 592 | 595 |  |  | DB Search |
| M.VGSI.S | N | 21.91 | 374.2165 | 4 | 0.34 | 375.223 | 1 | 13.82 | 5444 | 2.07e3 | 1 | 1 | 1140 | 1143 |  |  | DB Search |
| Y.RCF.K | N | 21.23 | 424.1893 | 3 | -9.2 | 425.1916 | 1 | 10.20 | 3945 | 1.22e3 | 1 | 1 | 795 | 797 |  |  | DB Search |
| V.TFR.Y | N | 20.53 | 422.2278 | 3 | -9.2 | 423.2301 | 1 | 22.38 | 8244 | 2.98e1 | 1 | 1 | 1048 | 1050 |  |  | DB Search |
| V.AGAA.L | N | 20.37 | 288.1433 | 4 | 8.03 | 289.1522 | 1 | 5.91 | 2203 | 6.8e3 | 1 | 1 | 77 | 80 |  |  | DB Search |
| L.VTTL.A | N | 20.12 | 432.2584 | 4 | -4.38 | 433.2627 | 1 | 11.15 | 4417 | 0 | 0 | 0 | 819 | 822 |  |  | DB Search |
| A.LTVA.F | N | 19.62 | 402.2478 | 4 | -4.79 | 403.2522 | 1 | 10.65 | 4156 | 4.9e3 | 1 | 1 | 205 | 208 |  |  | DB Search |
| S.KGY.G | N | 19.27 | 366.1903 | 3 | -9.39 | 367.1932 | 1 | 6.66 | 2561 | 3.55e1 | 1 | 1 | 286 | 288 |  |  | DB Search |
| A.TVVS.A | N | 18.11 | 404.2271 | 4 | -7.08 | 405.2305 | 1 | 8.17 | 3091 | 7.47e3 | 1 | 1 | 200 | 203 |  |  | DB Search |
| H.QNMAY.L | Y | 17.72 | 625.253 | 5 | 1.38 | 626.2596 | 1 | 13.96 | 5523 | 1.05e3 | 1 | 1 | 159 | 163 |  |  | DB Search |
| S.SGLH.S | N | 16.98 | 412.207 | 4 | -3.29 | 413.2119 | 1 | 1.21 | 286 | 3.65e4 | 1 | 1 | 100 | 103 |  |  | DB Search |
| S.ALDT.R | N | 16.7 | 418.2063 | 4 | -7.95 | 419.2093 | 1 | 4.85 | 1907 | 0 | 0 | 0 | 570 | 573 |  |  | DB Search |
| D.ESPV.K | N | 16.59 | 430.2063 | 4 | 5.53 | 431.2149 | 1 | 12.65 | 5009 | 0 | 0 | 0 | 687 | 690 |  |  | DB Search |
| W.EFISQ.M | N | 16.28 | 622.2962 | 5 | -5.13 | 623.2988 | 1 | 16.19 | 6365 | 4.92e2 | 1 | 1 | 521 | 525 |  |  | DB Search |
| V.IEAF.Q | N | 15.58 | 478.2427 | 4 | -6.65 | 479.2456 | 1 | 20.19 | 7599 | 4.22e2 | 1 | 1 | 738 | 741 |  |  | DB Search |
| E.GLSGGTAA.Q | N | 15.49 | 632.3129 | 8 | 7.29 | 633.3232 | 1 | 27.22 | 9602 | 4.58e2 | 1 | 1 | 831 | 838 |  |  | DB Search |
| total 16 peptides |
| --- |

Best Unique PSM (Scan POS\_R15-1.wiff:5523, m/z=626.2596, z=1, RT=13.96, ppm=-1.11):


C4R8P7|C4R8P7\_KOMPG

back to list

  

| Protein Coverage
| Supporting Peptides
| Best Unique PSM
|

Protein Coverage:

Supporting Peptides:

| Peptide | Uniq | -10lgP | Mass | Length | ppm | m/z | z | RT | Scan | Area POS\_R15-1 | #Feature | #Feature POS\_R15-1 | Start | End | PTM | AScore | Found By |
| --- | --- | --- | --- | --- | --- | --- | --- | --- | --- | --- | --- | --- | --- | --- | --- | --- | --- |
| F.IPQQP.Q | N | 25.19 | 581.3173 | 5 | -2.43 | 582.3217 | 1 | 8.89 | 3457 | 0 | 0 | 0 | 167 | 171 |  |  | DB Search |
| G.NTS.A | N | 21.44 | 320.1332 | 3 | 8.58 | 321.1424 | 1 | 6.16 | 2421 | 0 | 0 | 0 | 651 | 653 |  |  | DB Search |
| Q.QQQQQQKQQPPP.P | Y | 20.99 | 1461.7324 | 12 | -0.8 | 731.8711 | 2 | 41.07 | 13132 | 6.76e3 | 1 | 1 | 460 | 471 |  |  | DB Search |
| S.SFY.S | N | 18.1 | 415.1743 | 3 | 6.32 | 416.1832 | 1 | 8.32 | 3151 | 0 | 0 | 0 | 331 | 333 |  |  | DB Search |
| V.MLPP.Q | N | 18.04 | 456.2406 | 4 | 3.4 | 457.2483 | 1 | 13.75 | 5423 | 0 | 0 | 0 | 620 | 623 |  |  | DB Search |
| Q.Q(-17.03)PHQ.Q | N | 16.34 | 491.2128 | 4 | 0.63 | 492.2192 | 1 | 2.44 | 1064 | 0 | 0 | 0 | 310 | 313 | Pyro-glu from Q | Q1:Pyro-glu from Q:1000 | DB Search |
| Q.Q(-17.03)PIPQQ.N | Y | 16.04 | 692.3493 | 6 | -6.83 | 693.3502 | 1 | 13.65 | 5379 | 1.36e3 | 1 | 1 | 256 | 261 | Pyro-glu from Q | Q1:Pyro-glu from Q:1000 | DB Search |
| Q.QPQQ(+0.98)QAQG.Q | Y | 15.78 | 884.3988 | 8 | -0.78 | 885.4032 | 1 | 28.68 | 9993 | 6.41e1 | 1 | 1 | 170 | 177 | Deamidation (NQ) | Q4:Deamidation (NQ):0 | DB Search |
| Q.Q(-17.03)PH.Q | N | 15.48 | 363.1543 | 3 | -0.14 | 364.1606 | 1 | 2.32 | 992 | 3.39e4 | 1 | 1 | 310 | 312 | Pyro-glu from Q | Q1:Pyro-glu from Q:1000 | DB Search |
| S.KST.K | N | 15.47 | 334.1852 | 3 | 0.84 | 335.192 | 1 | 54.68 | 16215 | 3.66e2 | 1 | 1 | 732 | 734 |  |  | DB Search |
| A.PSVM.T | N | 15.29 | 432.2042 | 4 | 7.34 | 433.2136 | 1 | 17.71 | 6957 | 1.06e2 | 1 | 1 | 294 | 297 |  |  | DB Search |
| total 11 peptides |
| --- |

Best Unique PSM (Scan POS\_R15-1.wiff:13132, m/z=731.8701, z=2, RT=41.10, ppm=-3.29):


C4QZT4|C4QZT4\_KOMPG

back to list

  

| Protein Coverage
| Supporting Peptides
| Best Unique PSM
|

Protein Coverage:

Supporting Peptides:

| Peptide | Uniq | -10lgP | Mass | Length | ppm | m/z | z | RT | Scan | Area POS\_R15-1 | #Feature | #Feature POS\_R15-1 | Start | End | PTM | AScore | Found By |
| --- | --- | --- | --- | --- | --- | --- | --- | --- | --- | --- | --- | --- | --- | --- | --- | --- | --- |
| T.LPPP.I | N | 27.73 | 422.2529 | 4 | -1.45 | 423.2585 | 1 | 9.57 | 3721 | 1.63e3 | 1 | 1 | 522 | 525 |  |  | DB Search |
| S.IPTP.S | N | 25.97 | 426.2478 | 4 | -3.74 | 427.2524 | 1 | 12.64 | 4915 | 1.93e3 | 1 | 1 | 503 | 506 |  |  | DB Search |
| P.SPT.V | N | 23.16 | 303.143 | 3 | 6.39 | 304.1515 | 1 | 7.14 | 2731 | 5.4e3 | 2 | 2 | 119 | 121 |  |  | DB Search |
| P.SIPT.P | N | 22.87 | 416.2271 | 4 | -6.88 | 417.2305 | 1 | 14.64 | 5767 | 5.65e3 | 1 | 1 | 502 | 505 |  |  | DB Search |
| P.PPGGPM.N | Y | 22.39 | 554.2522 | 6 | 5.67 | 555.2613 | 1 | 21.77 | 8025 | 4.01e2 | 1 | 1 | 13 | 18 |  |  | DB Search |
| A.PSVP.G | N | 21.16 | 398.2165 | 4 | 4.76 | 399.2247 | 1 | 9.14 | 3512 | 0 | 0 | 0 | 232 | 235 |  |  | DB Search |
| V.SSNK.T | N | 20.83 | 434.2125 | 4 | -0.81 | 435.2184 | 1 | 2.82 | 1206 | 3.52e2 | 1 | 1 | 131 | 134 |  |  | DB Search |
| S.TAPQ.I | N | 20.69 | 415.2067 | 4 | 3.43 | 416.2144 | 1 | 8.30 | 3151 | 1.09e3 | 1 | 1 | 488 | 491 |  |  | DB Search |
| P.NSG.A | N | 20.42 | 276.107 | 3 | 6.48 | 277.1154 | 1 | 16.74 | 6592 | 0 | 0 | 0 | 312 | 314 |  |  | DB Search |
| V.PH.V | N | 19.52 | 252.1222 | 2 | -5.45 | 253.1275 | 1 | 5.57 | 2095 | 1.25e4 | 1 | 1 | 463 | 464 |  |  | DB Search |
| A.SNG.Q | N | 19.07 | 276.107 | 3 | 1.61 | 277.114 | 1 | 16.82 | 6604 | 0 | 0 | 0 | 642 | 644 |  |  | DB Search |
| T.Q(-17.03)SF.G | N | 18.71 | 363.143 | 3 | 2.8 | 364.1504 | 1 | 14.74 | 5815 | 0 | 0 | 0 | 399 | 401 | Pyro-glu from Q | Q1:Pyro-glu from Q:1000 | DB Search |
| R.IEID.D | N | 17.9 | 488.2482 | 4 | 1 | 489.2548 | 1 | 13.83 | 5478 | 0 | 0 | 0 | 652 | 655 |  |  | DB Search |
| L.ADI.Q | N | 17.27 | 317.1587 | 3 | -4.79 | 318.1636 | 1 | 3.30 | 1336 | 3.11e3 | 1 | 1 | 242 | 244 |  |  | DB Search |
| L.YPSGRG.S | Y | 17.08 | 635.3027 | 6 | 2.7 | 636.3101 | 1 | 13.53 | 5307 | 0 | 0 | 0 | 680 | 685 |  |  | DB Search |
| F.SPK.V | N | 16.89 | 330.1903 | 3 | -8.46 | 331.194 | 1 | 5.91 | 2235 | 5.17e3 | 1 | 1 | 459 | 461 |  |  | DB Search |
| N.DVHV.V | N | 15.6 | 468.2332 | 4 | -2.33 | 469.2383 | 1 | 8.21 | 3094 | 0 | 0 | 0 | 436 | 439 |  |  | DB Search |
| N.SGAPP.V | Y | 15.32 | 427.2067 | 5 | -5.29 | 428.2106 | 1 | 2.46 | 1073 | 2.72e2 | 1 | 1 | 313 | 317 |  |  | DB Search |
| total 18 peptides |
| --- |

Best Unique PSM (Scan POS\_R15-1.wiff:8025, m/z=555.2586, z=1, RT=21.79, ppm=3.19):


C4QV87|C4QV87\_KOMPG

back to list

  

| Protein Coverage
| Supporting Peptides
| Best Unique PSM
|

Protein Coverage:

Supporting Peptides:

| Peptide | Uniq | -10lgP | Mass | Length | ppm | m/z | z | RT | Scan | Area POS\_R15-1 | #Feature | #Feature POS\_R15-1 | Start | End | PTM | AScore | Found By |
| --- | --- | --- | --- | --- | --- | --- | --- | --- | --- | --- | --- | --- | --- | --- | --- | --- | --- |
| Y.LPSNP.N | N | 34.2 | 526.2751 | 5 | -5.22 | 527.2783 | 1 | 8.79 | 3411 | 0 | 0 | 0 | 1526 | 1530 |  |  | DB Search |
| L.IGGI.S | N | 27.34 | 358.2216 | 4 | -4.39 | 359.2264 | 1 | 15.45 | 6114 | 7.1e3 | 1 | 1 | 774 | 777 |  |  | DB Search |
| E.SLPT.K | N | 22.87 | 416.2271 | 4 | -6.88 | 417.2305 | 1 | 14.64 | 5767 | 5.65e3 | 1 | 1 | 1819 | 1822 |  |  | DB Search |
| E.YNP.T | N | 21.45 | 392.1696 | 3 | -3.75 | 393.1744 | 1 | 8.45 | 3226 | 1.39e2 | 1 | 1 | 867 | 869 |  |  | DB Search |
| D.VVDI.H | N | 19.9 | 444.2584 | 4 | 0.77 | 445.2649 | 1 | 17.32 | 6776 | 2.15e3 | 1 | 1 | 1534 | 1537 |  |  | DB Search |
| L.KCA.I | N | 19.71 | 320.1518 | 3 | 2.66 | 321.1591 | 1 | 15.28 | 6053 | 3.59e2 | 1 | 1 | 1031 | 1033 |  |  | DB Search |
| E.SHFL.K | N | 19.3 | 502.254 | 4 | 3.85 | 503.2619 | 1 | 15.62 | 6238 | 4.84e2 | 1 | 1 | 467 | 470 |  |  | DB Search |
| K.VTAT.A | N | 19.15 | 390.2114 | 4 | -9 | 391.2142 | 1 | 7.51 | 2842 | 9.19e2 | 1 | 1 | 1621 | 1624 |  |  | DB Search |
| H.TVVS.L | N | 18.11 | 404.2271 | 4 | -7.08 | 405.2305 | 1 | 8.17 | 3091 | 7.47e3 | 1 | 1 | 367 | 370 |  |  | DB Search |
| M.LGPV.T | N | 18.01 | 384.2372 | 4 | -2.96 | 385.2424 | 1 | 11.56 | 4504 | 2.76e3 | 1 | 1 | 1286 | 1289 |  |  | DB Search |
| V.GRFF.Q | N | 17.52 | 525.2699 | 4 | 8.75 | 526.2805 | 1 | 24.25 | 8792 | 5.9e1 | 1 | 1 | 226 | 229 |  |  | DB Search |
| A.ESW.N | N | 17.43 | 420.1645 | 3 | -0.16 | 421.1707 | 1 | 11.21 | 4460 | 0 | 0 | 0 | 680 | 682 |  |  | DB Search |
| M.KHF.S | N | 17.4 | 430.2328 | 3 | 0.78 | 431.2394 | 1 | 20.46 | 7703 | 1.69e2 | 1 | 1 | 533 | 535 |  |  | DB Search |
| N.EWGYT.T | Y | 16.36 | 654.2649 | 5 | 6.23 | 655.2747 | 1 | 20.67 | 7746 | 5.07e1 | 1 | 1 | 1414 | 1418 |  |  | DB Search |
| N.YMR.G | N | 15.84 | 468.2155 | 3 | 0.49 | 469.2218 | 1 | 8.86 | 3455 | 2.39e3 | 1 | 1 | 1808 | 1810 |  |  | DB Search |
| A.AYSV.I | N | 15.26 | 438.2114 | 4 | -3.08 | 439.2163 | 1 | 7.95 | 2920 | 3.53e4 | 1 | 1 | 1680 | 1683 |  |  | DB Search |
| total 16 peptides |
| --- |

Best Unique PSM (Scan POS\_R15-1.wiff:7746, m/z=655.2747, z=1, RT=20.67, ppm=3.75):


C4R8H1|C4R8H1\_KOMPG

back to list

  

| Protein Coverage
| Supporting Peptides
| Best Unique PSM
|

Protein Coverage:

Supporting Peptides:

| Peptide | Uniq | -10lgP | Mass | Length | ppm | m/z | z | RT | Scan | Area POS\_R15-1 | #Feature | #Feature POS\_R15-1 | Start | End | PTM | AScore | Found By |
| --- | --- | --- | --- | --- | --- | --- | --- | --- | --- | --- | --- | --- | --- | --- | --- | --- | --- |
| D.ELIR.Q | N | 25.05 | 529.3224 | 4 | -4.56 | 530.3259 | 1 | 17.34 | 6855 | 6.68e3 | 1 | 1 | 647 | 650 |  |  | DB Search |
| Y.TSG.K | N | 21.44 | 263.1117 | 3 | 2.31 | 264.119 | 1 | 3.71 | 1537 | 5.72e3 | 1 | 1 | 953 | 955 |  |  | DB Search |
| A.KYT.S | N | 21.18 | 410.2165 | 3 | -4.93 | 411.2208 | 1 | 12.54 | 4954 | 3.83e2 | 1 | 1 | 951 | 953 |  |  | DB Search |
| D.IPRP.T | N | 21.04 | 481.3012 | 4 | -9.61 | 482.3027 | 1 | 17.02 | 6682 | 0 | 0 | 0 | 1170 | 1173 |  |  | DB Search |
| L.RCL.R | N | 20.6 | 390.2049 | 3 | -4.41 | 391.2095 | 1 | 8.08 | 3003 | 0 | 0 | 0 | 292 | 294 |  |  | DB Search |
| S.VSLS.S | N | 20.57 | 404.2271 | 4 | -7.08 | 405.2305 | 1 | 8.17 | 2961 | 7.47e3 | 1 | 1 | 1644 | 1647 |  |  | DB Search |
| S.KFH.P | N | 20.21 | 430.2328 | 3 | 9.29 | 431.243 | 1 | 12.31 | 5009 | 5.31e3 | 1 | 1 | 1488 | 1490 |  |  | DB Search |
| L.NITI.N | N | 19.19 | 459.2693 | 4 | -0.54 | 460.2752 | 1 | 17.07 | 6712 | 2.02e3 | 1 | 1 | 608 | 611 |  |  | DB Search |
| V.IDNF.E | N | 18.37 | 507.2329 | 4 | -5.34 | 508.2362 | 1 | 14.74 | 5819 | 0 | 0 | 0 | 1521 | 1524 |  |  | DB Search |
| N.IDFS.L | N | 18.2 | 480.222 | 4 | -2.08 | 481.2271 | 1 | 16.69 | 6580 | 0 | 0 | 0 | 869 | 872 |  |  | DB Search |
| S.IYSS.T | N | 17.99 | 468.222 | 4 | 0.32 | 469.2283 | 1 | 16.06 | 6398 | 1.94e3 | 1 | 1 | 929 | 932 |  |  | DB Search |
| Y.ISGY.E | N | 17.88 | 438.2114 | 4 | -3.08 | 439.2163 | 1 | 7.95 | 2900 | 3.53e4 | 1 | 1 | 352 | 355 |  |  | DB Search |
| K.QALE.H | N | 17.59 | 459.2329 | 4 | -5.43 | 460.2365 | 1 | 4.85 | 1909 | 7.42e3 | 2 | 2 | 720 | 723 |  |  | DB Search |
| I.SSEL.S | N | 17.13 | 434.2013 | 4 | -0.44 | 435.2073 | 1 | 2.87 | 1206 | 7.24e2 | 1 | 1 | 903 | 906 |  |  | DB Search |
| L.PSN(+0.98)IAGGAAPPP.P | Y | 16.95 | 1048.5189 | 12 | 6.52 | 1049.5304 | 1 | 20.67 | 7708 | 1.52e3 | 1 | 1 | 1111 | 1122 | Deamidation (NQ) | N3:Deamidation (NQ):1000 | DB Search |
| S.PFAD.I | N | 16.94 | 448.1958 | 4 | -4.71 | 449.1998 | 1 | 8.74 | 3383 | 2.68e3 | 1 | 1 | 1166 | 1169 |  |  | DB Search |
| S.TFT.L | N | 15.92 | 367.1743 | 3 | -7.47 | 368.1779 | 1 | 8.54 | 3263 | 0 | 0 | 0 | 1774 | 1776 |  |  | DB Search |
| P.SPVPSPFLPSS.P | Y | 15.77 | 1113.5706 | 11 | 5.38 | 557.7942 | 2 | 26.10 | 9302 | 1.47e1 | 1 | 1 | 1155 | 1165 |  |  | DB Search |
| Q.LTET.P | N | 15.47 | 462.2326 | 4 | 1.41 | 463.2393 | 1 | 15.37 | 6065 | 2.41e2 | 1 | 1 | 1783 | 1786 |  |  | DB Search |
| F.LPSSPFA.D | Y | 15.39 | 717.3697 | 7 | 0.04 | 718.3752 | 1 | 13.68 | 5451 | 1.31e2 | 1 | 1 | 1162 | 1168 |  |  | DB Search |
| total 20 peptides |
| --- |

Best Unique PSM (Scan POS\_R15-1.wiff:7708, m/z=1049.5304, z=1, RT=20.67, ppm=4.04):


C4R0K4|C4R0K4\_KOMPG

back to list

  

| Protein Coverage
| Supporting Peptides
| Best Unique PSM
|

Protein Coverage:

Supporting Peptides:

| Peptide | Uniq | -10lgP | Mass | Length | ppm | m/z | z | RT | Scan | Area POS\_R15-1 | #Feature | #Feature POS\_R15-1 | Start | End | PTM | AScore | Found By |
| --- | --- | --- | --- | --- | --- | --- | --- | --- | --- | --- | --- | --- | --- | --- | --- | --- | --- |
| P.TDVP.E | N | 22.14 | 430.2063 | 4 | -7.31 | 431.2094 | 1 | 8.43 | 3152 | 9.04e3 | 3 | 3 | 407 | 410 |  |  | DB Search |
| D.DYNLQ.L | Y | 20.46 | 651.2864 | 5 | -0.94 | 652.2914 | 1 | 10.88 | 4258 | 1.35e3 | 1 | 1 | 97 | 101 |  |  | DB Search |
| L.WSD.P | N | 19.99 | 406.1488 | 3 | -8.45 | 407.1517 | 1 | 8.75 | 3390 | 0 | 0 | 0 | 420 | 422 |  |  | DB Search |
| G.LSPM.L | N | 19.59 | 446.2199 | 4 | -1.9 | 447.2252 | 1 | 15.64 | 6201 | 6.79e2 | 1 | 1 | 390 | 393 |  |  | DB Search |
| V.RSF.R | N | 17.5 | 408.2121 | 3 | -1.76 | 409.2177 | 1 | 9.17 | 3526 | 0 | 0 | 0 | 29 | 31 |  |  | DB Search |
| R.Q(-17.03)PIL.K | N | 17.35 | 452.2635 | 4 | -6.01 | 453.2669 | 1 | 32.30 | 10953 | 1.74e3 | 1 | 1 | 113 | 116 | Pyro-glu from Q | Q1:Pyro-glu from Q:1000 | DB Search |
| M.E(-18.01)PR.Q | N | 16.87 | 382.1965 | 3 | -0.37 | 383.2026 | 1 | 2.61 | 1082 | 1.62e4 | 1 | 1 | 110 | 112 | Pyro-glu from E | E1:Pyro-glu from E:1000 | DB Search |
| F.TKC.G | N | 16.14 | 350.1624 | 3 | 7.67 | 351.1715 | 1 | 4.93 | 1958 | 0 | 0 | 0 | 291 | 293 |  |  | DB Search |
| T.NGV.S | N | 15.37 | 288.1434 | 3 | 8 | 289.1522 | 1 | 5.91 | 2301 | 6.8e3 | 1 | 1 | 123 | 125 |  |  | DB Search |
| total 9 peptides |
| --- |

Best Unique PSM (Scan POS\_R15-1.wiff:4258, m/z=652.2914, z=1, RT=10.88, ppm=-3.43):


C4QYR1|C4QYR1\_KOMPG

back to list

  

| Protein Coverage
| Supporting Peptides
| Best Unique PSM
|

Protein Coverage:

Supporting Peptides:

| Peptide | Uniq | -10lgP | Mass | Length | ppm | m/z | z | RT | Scan | Area POS\_R15-1 | #Feature | #Feature POS\_R15-1 | Start | End | PTM | AScore | Found By |
| --- | --- | --- | --- | --- | --- | --- | --- | --- | --- | --- | --- | --- | --- | --- | --- | --- | --- |
| K.ETW.A | N | 26.2 | 434.1801 | 3 | -4.77 | 435.1843 | 1 | 12.45 | 4871 | 1.27e3 | 1 | 1 | 23 | 25 |  |  | DB Search |
| Q.HFT.L | N | 23.64 | 403.1856 | 3 | -0.83 | 404.1915 | 1 | 13.94 | 5543 | 0 | 0 | 0 | 238 | 240 |  |  | DB Search |
| K.RSSS.L | N | 18.8 | 435.2078 | 4 | -3.98 | 436.2122 | 1 | 11.38 | 4548 | 7.31e2 | 1 | 1 | 98 | 101 |  |  | DB Search |
| S.LGGE.D | N | 17.74 | 374.1801 | 4 | 0.95 | 375.1868 | 1 | 2.99 | 1264 | 7.34e3 | 1 | 1 | 345 | 348 |  |  | DB Search |
| Q.EKIALG.K | Y | 16.75 | 629.3748 | 6 | -4.88 | 630.3774 | 1 | 28.81 | 10027 | 1.37e3 | 1 | 1 | 408 | 413 |  |  | DB Search |
| G.TLQW.R | N | 15.55 | 546.2802 | 4 | 0.72 | 547.2865 | 1 | 22.01 | 8160 | 8.82e1 | 1 | 1 | 168 | 171 |  |  | DB Search |
| total 6 peptides |
| --- |

Best Unique PSM (Scan POS\_R15-1.wiff:10027, m/z=630.3774, z=1, RT=28.81, ppm=-7.36):


C4R564|C4R564\_KOMPG

back to list

  

| Protein Coverage
| Supporting Peptides
| Best Unique PSM
|

Protein Coverage:

Supporting Peptides:

| Peptide | Uniq | -10lgP | Mass | Length | ppm | m/z | z | RT | Scan | Area POS\_R15-1 | #Feature | #Feature POS\_R15-1 | Start | End | PTM | AScore | Found By |
| --- | --- | --- | --- | --- | --- | --- | --- | --- | --- | --- | --- | --- | --- | --- | --- | --- | --- |
| S.GGGL.I | N | 27.46 | 302.159 | 4 | -3.72 | 303.1644 | 1 | 6.06 | 2387 | 3.14e3 | 1 | 1 | 222 | 225 |  |  | DB Search |
| L.AFTP.Q | N | 25.4 | 434.2165 | 4 | -5.48 | 435.2203 | 1 | 13.12 | 5200 | 6.05e3 | 1 | 1 | 16 | 19 |  |  | DB Search |
| F.VGGL.Q | N | 24.87 | 344.2059 | 4 | -5.22 | 345.2106 | 1 | 10.74 | 4195 | 1.51e3 | 1 | 1 | 849 | 852 |  |  | DB Search |
| C.RLVP.G | N | 24.65 | 483.3169 | 4 | -4.88 | 484.3206 | 1 | 10.06 | 3922 | 3.47e3 | 1 | 1 | 1294 | 1297 |  |  | DB Search |
| I.VGSI.L | N | 21.91 | 374.2165 | 4 | 0.34 | 375.223 | 1 | 13.82 | 5444 | 2.07e3 | 1 | 1 | 1417 | 1420 |  |  | DB Search |
| F.ELW.S | N | 21.42 | 446.2165 | 3 | -5.13 | 447.2204 | 1 | 31.30 | 10711 | 1.59e3 | 1 | 1 | 1226 | 1228 |  |  | DB Search |
| A.PGE.D | N | 21.28 | 301.1274 | 3 | 1.88 | 302.1345 | 1 | 2.04 | 869 | 2.57e3 | 1 | 1 | 744 | 746 |  |  | DB Search |
| Q.GSVI.V | N | 20.47 | 374.2165 | 4 | -5.12 | 375.2209 | 1 | 10.69 | 4154 | 1e3 | 1 | 1 | 1133 | 1136 |  |  | DB Search |
| L.VTGV.A | N | 20.36 | 374.2165 | 4 | -4.06 | 375.2213 | 1 | 5.20 | 1917 | 6.27e3 | 1 | 1 | 1422 | 1425 |  |  | DB Search |
| A.GQCTDGS.E | Y | 19.96 | 666.2279 | 7 | -3.85 | 667.231 | 1 | 3.20 | 1375 | 0 | 0 | 0 | 142 | 148 |  |  | DB Search |
| S.TAY.I | N | 19.3 | 353.1587 | 3 | -1.32 | 354.1646 | 1 | 12.51 | 4913 | 1.39e3 | 1 | 1 | 84 | 86 |  |  | DB Search |
| F.KGY.P | N | 19.27 | 366.1903 | 3 | -9.39 | 367.1932 | 1 | 6.66 | 2561 | 3.55e1 | 1 | 1 | 27 | 29 |  |  | DB Search |
| N.EAY.V | N | 19.24 | 381.1536 | 3 | -5.14 | 382.158 | 1 | 5.09 | 2057 | 2.63e3 | 1 | 1 | 728 | 730 |  |  | DB Search |
| N.SNG.T | N | 19.07 | 276.107 | 3 | 1.61 | 277.114 | 1 | 16.82 | 6604 | 0 | 0 | 0 | 975 | 977 |  |  | DB Search |
| S.RDR.A | N | 18.68 | 445.2397 | 3 | 8.14 | 446.2495 | 1 | 10.60 | 4131 | 1.44e2 | 1 | 1 | 774 | 776 |  |  | DB Search |
| L.HGY.T | N | 18.63 | 375.1543 | 3 | -9 | 376.1572 | 1 | 4.83 | 1959 | 3.43e3 | 1 | 1 | 1068 | 1070 |  |  | DB Search |
| Q.IDFS.H | N | 18.2 | 480.222 | 4 | -2.08 | 481.2271 | 1 | 16.69 | 6580 | 0 | 0 | 0 | 1203 | 1206 |  |  | DB Search |
| V.EPKP.C | N | 17.69 | 469.2536 | 4 | -5.47 | 470.2572 | 1 | 15.43 | 6115 | 1.58e3 | 1 | 1 | 1341 | 1344 |  |  | DB Search |
| V.RSF.I | N | 17.5 | 408.2121 | 3 | -1.76 | 409.2177 | 1 | 9.17 | 3526 | 0 | 0 | 0 | 349 | 351 |  |  | DB Search |
| T.ASVVY.H | N | 16.99 | 537.2798 | 5 | -3.91 | 538.2837 | 1 | 16.27 | 6471 | 8.86e2 | 1 | 1 | 40 | 44 |  |  | DB Search |
| A.YFPP.N | N | 16.74 | 522.2478 | 4 | -3.53 | 523.252 | 1 | 10.13 | 3956 | 0 | 0 | 0 | 930 | 933 |  |  | DB Search |
| Y.ELID.A | N | 16.42 | 488.2482 | 4 | -0.37 | 489.2541 | 1 | 13.94 | 5546 | 0 | 0 | 0 | 329 | 332 |  |  | DB Search |
| E.EYIY.L | N | 16.17 | 586.2639 | 4 | 4.94 | 587.2726 | 1 | 16.20 | 6451 | 2.9e2 | 1 | 1 | 703 | 706 |  |  | DB Search |
| G.RLC.Y | N | 16.16 | 390.2049 | 3 | 4.43 | 391.213 | 1 | 3.37 | 1421 | 1.01e3 | 1 | 1 | 1254 | 1256 |  |  | DB Search |
| K.ICIM.G | N | 16.12 | 478.2284 | 4 | 8.85 | 479.2387 | 1 | 29.77 | 10263 | 1.63e2 | 1 | 1 | 538 | 541 |  |  | DB Search |
| Q.LDLG.G | N | 15.65 | 416.2271 | 4 | -5.75 | 417.2309 | 1 | 13.92 | 5536 | 5.65e3 | 1 | 1 | 65 | 68 |  |  | DB Search |
| N.FRG.V | N | 15.51 | 378.2015 | 3 | -9.17 | 379.2044 | 1 | 3.37 | 1492 | 1.94e3 | 1 | 1 | 512 | 514 |  |  | DB Search |
| T.Q(-17.03)PH.K | N | 15.48 | 363.1543 | 3 | -0.14 | 364.1606 | 1 | 2.32 | 992 | 3.39e4 | 1 | 1 | 1264 | 1266 | Pyro-glu from Q | Q1:Pyro-glu from Q:1000 | DB Search |
| S.VAISA.G | N | 15.29 | 459.2693 | 5 | -9.29 | 460.2711 | 1 | 17.14 | 6749 | 2.02e3 | 1 | 1 | 285 | 289 |  |  | DB Search |
| R.PVE.Y | N | 15.19 | 343.1743 | 3 | -3.43 | 344.1796 | 1 | 12.76 | 5037 | 6.34e2 | 1 | 1 | 1314 | 1316 |  |  | DB Search |
| total 30 peptides |
| --- |

Best Unique PSM (Scan POS\_R15-1.wiff:1375, m/z=667.3011, z=1, RT=3.26, ppm=-6.33):


C4R586|C4R586\_KOMPG

back to list

  

| Protein Coverage
| Supporting Peptides
| Best Unique PSM
|

Protein Coverage:

Supporting Peptides:

| Peptide | Uniq | -10lgP | Mass | Length | ppm | m/z | z | RT | Scan | Area POS\_R15-1 | #Feature | #Feature POS\_R15-1 | Start | End | PTM | AScore | Found By |
| --- | --- | --- | --- | --- | --- | --- | --- | --- | --- | --- | --- | --- | --- | --- | --- | --- | --- |
| T.GVSI.L | N | 22.74 | 374.2165 | 4 | -2.51 | 375.2219 | 1 | 13.91 | 5529 | 2.07e3 | 1 | 1 | 271 | 274 |  |  | DB Search |
| S.RCL.R | N | 20.6 | 390.2049 | 3 | -4.41 | 391.2095 | 1 | 8.08 | 3003 | 0 | 0 | 0 | 61 | 63 |  |  | DB Search |
| H.GSVL.G | N | 20.47 | 374.2165 | 4 | -5.12 | 375.2209 | 1 | 10.69 | 4154 | 1e3 | 1 | 1 | 225 | 228 |  |  | DB Search |
| G.TLGV.I | N | 18.91 | 388.2322 | 4 | -5.15 | 389.2365 | 1 | 17.34 | 6852 | 1.43e3 | 1 | 1 | 265 | 268 |  |  | DB Search |
| V.GGNVAC(-1.01)N.A | Y | 17.82 | 632.2462 | 7 | 4.86 | 633.255 | 1 | 11.36 | 4380 | 3.33e3 | 1 | 1 | 205 | 211 | Half of a disulfide bridge | C6:Half of a disulfide bridge:1000 | DB Search |
| total 5 peptides |
| --- |

Best Unique PSM (Scan POS\_R15-1.wiff:4380, m/z=633.255, z=1, RT=11.36, ppm=2.38):


C4R406|C4R406\_KOMPG

back to list

  

| Protein Coverage
| Supporting Peptides
| Best Unique PSM
|

Protein Coverage:

Supporting Peptides:

| Peptide | Uniq | -10lgP | Mass | Length | ppm | m/z | z | RT | Scan | Area POS\_R15-1 | #Feature | #Feature POS\_R15-1 | Start | End | PTM | AScore | Found By |
| --- | --- | --- | --- | --- | --- | --- | --- | --- | --- | --- | --- | --- | --- | --- | --- | --- | --- |
| F.ALFG.G | N | 24.47 | 406.2216 | 4 | 1.3 | 407.2284 | 1 | 22.63 | 8312 | 0 | 0 | 0 | 190 | 193 |  |  | DB Search |
| L.GSAS.A | N | 17.29 | 320.1332 | 4 | 7.75 | 321.1422 | 1 | 6.30 | 2464 | 0 | 0 | 0 | 20 | 23 |  |  | DB Search |
| I.LGSASAGI.C | Y | 16.47 | 674.3599 | 8 | -2.99 | 675.3635 | 1 | 5.72 | 2269 | 0 | 0 | 0 | 19 | 26 |  |  | DB Search |
| total 3 peptides |
| --- |

Best Unique PSM (Scan POS\_R15-1.wiff:2269, m/z=675.3635, z=1, RT=5.72, ppm=-5.47):


C4R3X3|C4R3X3\_KOMPG

back to list

  

| Protein Coverage
| Supporting Peptides
| Best Unique PSM
|

Protein Coverage:

Supporting Peptides:

| Peptide | Uniq | -10lgP | Mass | Length | ppm | m/z | z | RT | Scan | Area POS\_R15-1 | #Feature | #Feature POS\_R15-1 | Start | End | PTM | AScore | Found By |
| --- | --- | --- | --- | --- | --- | --- | --- | --- | --- | --- | --- | --- | --- | --- | --- | --- | --- |
| L.IDLLGGA.A | Y | 25.17 | 657.3697 | 7 | -6.66 | 658.371 | 1 | 26.90 | 9524 | 7.97e2 | 1 | 1 | 200 | 206 |  |  | DB Search |
| H.PVLV.E | N | 23.27 | 426.2842 | 4 | 4.41 | 427.2923 | 1 | 20.36 | 7664 | 0 | 0 | 0 | 311 | 314 |  |  | DB Search |
| P.LSGL.S | N | 22.6 | 388.2322 | 4 | -3.81 | 389.237 | 1 | 9.87 | 3804 | 6.08e3 | 1 | 1 | 245 | 248 |  |  | DB Search |
| L.IDLLGG.A | N | 17.01 | 586.3326 | 6 | -7.77 | 587.3339 | 1 | 21.28 | 7911 | 6.6e2 | 1 | 1 | 200 | 205 |  |  | DB Search |
| total 4 peptides |
| --- |

Best Unique PSM (Scan POS\_R15-1.wiff:9524, m/z=658.3725, z=2, RT=26.93, ppm=-9.15):


C4QYF1|C4QYF1\_KOMPG

back to list

  

| Protein Coverage
| Supporting Peptides
| Best Unique PSM
|

Protein Coverage:

Supporting Peptides:

| Peptide | Uniq | -10lgP | Mass | Length | ppm | m/z | z | RT | Scan | Area POS\_R15-1 | #Feature | #Feature POS\_R15-1 | Start | End | PTM | AScore | Found By |
| --- | --- | --- | --- | --- | --- | --- | --- | --- | --- | --- | --- | --- | --- | --- | --- | --- | --- |
| P.PSII.E | N | 29.95 | 428.2635 | 4 | -0.56 | 429.2694 | 1 | 21.28 | 7884 | 0 | 0 | 0 | 317 | 320 |  |  | DB Search |
| K.IGGL.F | N | 27.34 | 358.2216 | 4 | -4.39 | 359.2264 | 1 | 15.45 | 6114 | 7.1e3 | 1 | 1 | 736 | 739 |  |  | DB Search |
| S.GSGSGC(-1.01)GSGSG.P | Y | 26.99 | 810.2688 | 11 | -8.16 | 811.2675 | 1 | 1.90 | 778 | 1.27e4 | 1 | 1 | 24 | 34 | Half of a disulfide bridge | C6:Half of a disulfide bridge:1000 | DB Search |
| D.SPII.P | N | 26.66 | 428.2635 | 4 | -1.63 | 429.269 | 1 | 21.35 | 7905 | 0 | 0 | 0 | 150 | 153 |  |  | DB Search |
| S.GSGSGSGC(-1.01)GSG.S | Y | 24.81 | 810.2688 | 11 | -8.24 | 811.2674 | 1 | 1.94 | 825 | 1.27e4 | 1 | 1 | 22 | 32 | Half of a disulfide bridge | C8:Half of a disulfide bridge:1000 | DB Search |
| G.TSLA.D | N | 24.8 | 390.2114 | 4 | -5.32 | 391.2157 | 1 | 6.25 | 2416 | 1.07e4 | 1 | 1 | 815 | 818 |  |  | DB Search |
| G.PGE.N | N | 21.28 | 301.1274 | 3 | 1.88 | 302.1345 | 1 | 2.04 | 869 | 2.57e3 | 1 | 1 | 296 | 298 |  |  | DB Search |
| G.RCL.M | N | 20.6 | 390.2049 | 3 | -4.41 | 391.2095 | 1 | 8.08 | 3003 | 0 | 0 | 0 | 839 | 841 |  |  | DB Search |
| V.YSPR.K | N | 20.52 | 521.2598 | 4 | 7.22 | 522.2695 | 1 | 8.15 | 2984 | 9.49e2 | 1 | 1 | 241 | 244 |  |  | DB Search |
| E.FARP.F | N | 20.36 | 489.2699 | 4 | -4.45 | 490.2738 | 1 | 10.19 | 3954 | 6.32e2 | 1 | 1 | 538 | 541 |  |  | DB Search |
| S.VTTL.L | N | 20.12 | 432.2584 | 4 | -4.38 | 433.2627 | 1 | 11.15 | 4417 | 0 | 0 | 0 | 892 | 895 |  |  | DB Search |
| K.EAY.F | N | 19.24 | 381.1536 | 3 | -5.14 | 382.158 | 1 | 5.09 | 2057 | 2.63e3 | 1 | 1 | 561 | 563 |  |  | DB Search |
| D.SIDT.T | N | 19.03 | 434.2013 | 4 | -3.95 | 435.2057 | 1 | 15.10 | 5999 | 0 | 0 | 0 | 432 | 435 |  |  | DB Search |
| N.RSSS.T | N | 18.8 | 435.2078 | 4 | -3.98 | 436.2122 | 1 | 11.38 | 4548 | 7.31e2 | 1 | 1 | 691 | 694 |  |  | DB Search |
| F.ESPF.L | N | 17.8 | 478.2063 | 4 | 2.35 | 479.2136 | 1 | 13.84 | 5519 | 6.96e2 | 1 | 1 | 6 | 9 |  |  | DB Search |
| I.Q(-17.03)PIL.G | N | 17.35 | 452.2635 | 4 | -6.01 | 453.2669 | 1 | 32.30 | 10953 | 1.74e3 | 1 | 1 | 230 | 233 | Pyro-glu from Q | Q1:Pyro-glu from Q:1000 | DB Search |
| L.E(-18.01)PR.R | N | 16.87 | 382.1965 | 3 | -0.37 | 383.2026 | 1 | 2.61 | 1082 | 1.62e4 | 1 | 1 | 631 | 633 | Pyro-glu from E | E1:Pyro-glu from E:1000 | DB Search |
| C.DLVT.V | N | 16.75 | 446.2376 | 4 | -4.95 | 447.2416 | 1 | 23.24 | 8484 | 0 | 0 | 0 | 945 | 948 |  |  | DB Search |
| Q.TPGF.T | N | 15.9 | 420.2009 | 4 | 2.59 | 421.2082 | 1 | 12.63 | 4943 | 3.01e3 | 1 | 1 | 415 | 418 |  |  | DB Search |
| V.AEP.K | N | 15.46 | 315.143 | 3 | -3.02 | 316.1486 | 1 | 2.92 | 1198 | 1.54e3 | 1 | 1 | 212 | 214 |  |  | DB Search |
| P.Q(-17.03)PF.T | N | 15.22 | 373.1638 | 3 | -0.75 | 374.1698 | 1 | 21.41 | 7929 | 8.45e4 | 1 | 1 | 347 | 349 | Pyro-glu from Q | Q1:Pyro-glu from Q:1000 | DB Search |
| total 21 peptides |
| --- |

Best Unique PSM (Scan POS\_R15-1.wiff:778, m/z=811.2675, z=1, RT=1.90, ppm=-10.64):


C4QXF3|C4QXF3\_KOMPG

back to list

  

| Protein Coverage
| Supporting Peptides
| Best Unique PSM
|

Protein Coverage:

Supporting Peptides:

| Peptide | Uniq | -10lgP | Mass | Length | ppm | m/z | z | RT | Scan | Area POS\_R15-1 | #Feature | #Feature POS\_R15-1 | Start | End | PTM | AScore | Found By |
| --- | --- | --- | --- | --- | --- | --- | --- | --- | --- | --- | --- | --- | --- | --- | --- | --- | --- |
| R.LPPP.A | N | 27.73 | 422.2529 | 4 | -1.45 | 423.2585 | 1 | 9.57 | 3721 | 1.63e3 | 1 | 1 | 75 | 78 |  |  | DB Search |
| M.ITGL.F | N | 27.55 | 402.2478 | 4 | -1.76 | 403.2534 | 1 | 15.04 | 5964 | 1.56e4 | 2 | 2 | 672 | 675 |  |  | DB Search |
| P.GGGI.L | N | 27.46 | 302.159 | 4 | -3.72 | 303.1644 | 1 | 6.06 | 2387 | 3.14e3 | 1 | 1 | 159 | 162 |  |  | DB Search |
| F.HFT.P | N | 23.64 | 403.1856 | 3 | -0.83 | 404.1915 | 1 | 13.94 | 5543 | 0 | 0 | 0 | 480 | 482 |  |  | DB Search |
| N.VTVP.G | N | 22.26 | 414.2478 | 4 | -0.09 | 415.254 | 1 | 17.14 | 6680 | 3.72e3 | 1 | 1 | 680 | 683 |  |  | DB Search |
| D.SSNK.K | N | 20.83 | 434.2125 | 4 | -0.81 | 435.2184 | 1 | 2.82 | 1206 | 3.52e2 | 1 | 1 | 328 | 331 |  |  | DB Search |
| L.GTIF.F | N | 20.4 | 436.2322 | 4 | -4.1 | 437.2366 | 1 | 23.18 | 8454 | 1.09e3 | 1 | 1 | 663 | 666 |  |  | DB Search |
| T.DGFA.P | N | 17.56 | 408.1645 | 4 | -4.25 | 409.169 | 1 | 8.22 | 3102 | 0 | 0 | 0 | 518 | 521 |  |  | DB Search |
| R.YTGGNST.S | Y | 15.6 | 698.2871 | 7 | -6.83 | 699.2879 | 1 | 4.79 | 1865 | 0 | 0 | 0 | 739 | 745 |  |  | DB Search |
| total 9 peptides |
| --- |

Best Unique PSM (Scan POS\_R15-1.wiff:1865, m/z=699.2879, z=1, RT=4.79, ppm=-9.31):


C4R001|C4R001\_KOMPG

back to list

  

| Protein Coverage
| Supporting Peptides
| Best Unique PSM
|

Protein Coverage:

Supporting Peptides:

| Peptide | Uniq | -10lgP | Mass | Length | ppm | m/z | z | RT | Scan | Area POS\_R15-1 | #Feature | #Feature POS\_R15-1 | Start | End | PTM | AScore | Found By |
| --- | --- | --- | --- | --- | --- | --- | --- | --- | --- | --- | --- | --- | --- | --- | --- | --- | --- |
| S.ELVR.D | N | 23.24 | 515.3067 | 4 | -4.7 | 516.3103 | 1 | 11.12 | 4401 | 1.13e4 | 1 | 1 | 747 | 750 |  |  | DB Search |
| S.AQLP.G | N | 21.52 | 427.2431 | 4 | -3.04 | 428.248 | 1 | 11.76 | 4668 | 6.31e3 | 1 | 1 | 984 | 987 |  |  | DB Search |
| L.LPRP.R | N | 21.04 | 481.3012 | 4 | -9.61 | 482.3027 | 1 | 17.02 | 6682 | 0 | 0 | 0 | 498 | 501 |  |  | DB Search |
| K.AGAA.W | N | 20.37 | 288.1433 | 4 | 8.03 | 289.1522 | 1 | 5.91 | 2203 | 6.8e3 | 1 | 1 | 736 | 739 |  |  | DB Search |
| M.ISEPAGEP.Y | Y | 19.41 | 798.3759 | 8 | -6.48 | 799.376 | 1 | 23.32 | 8506 | 4.34e1 | 1 | 1 | 120 | 127 |  |  | DB Search |
| S.NLTL.D | N | 19.19 | 459.2693 | 4 | -0.54 | 460.2752 | 1 | 17.07 | 6712 | 2.02e3 | 1 | 1 | 952 | 955 |  |  | DB Search |
| I.QSPI.W | N | 18.64 | 443.238 | 4 | -7.76 | 444.2407 | 1 | 10.47 | 4087 | 4.96e2 | 1 | 1 | 1309 | 1312 |  |  | DB Search |
| K.VSPP.P | N | 17.71 | 398.2165 | 4 | -2.14 | 399.222 | 1 | 8.11 | 3026 | 0 | 0 | 0 | 465 | 468 |  |  | DB Search |
| Y.DAML.H | N | 17.34 | 448.1992 | 4 | 9.54 | 449.2096 | 1 | 15.07 | 5980 | 0 | 0 | 0 | 26 | 29 |  |  | DB Search |
| W.AHVI.C | N | 16.82 | 438.259 | 4 | -6.35 | 439.2625 | 1 | 8.70 | 3374 | 6.21e2 | 1 | 1 | 1108 | 1111 |  |  | DB Search |
| E.KFEA.A | N | 15.65 | 493.2536 | 4 | 5 | 494.2621 | 1 | 24.41 | 8849 | 7.46e1 | 1 | 1 | 592 | 595 |  |  | DB Search |
| K.SEIA.N | N | 15.6 | 418.2063 | 4 | -9.49 | 419.2086 | 1 | 4.93 | 1962 | 0 | 0 | 0 | 199 | 202 |  |  | DB Search |
| K.NNSV.K | N | 15.01 | 432.1969 | 4 | 8.48 | 433.2067 | 1 | 7.60 | 2865 | 3.02e3 | 1 | 1 | 1295 | 1298 |  |  | DB Search |
| total 13 peptides |
| --- |

Best Unique PSM (Scan POS\_R15-1.wiff:8506, m/z=799.4417, z=1, RT=23.36, ppm=-8.96):


C4R883|C4R883\_KOMPG

back to list

  

| Protein Coverage
| Supporting Peptides
| Best Unique PSM
|

Protein Coverage:

Supporting Peptides:

| Peptide | Uniq | -10lgP | Mass | Length | ppm | m/z | z | RT | Scan | Area POS\_R15-1 | #Feature | #Feature POS\_R15-1 | Start | End | PTM | AScore | Found By |
| --- | --- | --- | --- | --- | --- | --- | --- | --- | --- | --- | --- | --- | --- | --- | --- | --- | --- |
| T.VVDT.P | N | 16.67 | 432.222 | 4 | -1.67 | 433.2275 | 1 | 10.86 | 4240 | 4.88e2 | 1 | 1 | 156 | 159 |  |  | DB Search |
| Q.E(-18.01)PH.A | N | 16.21 | 363.1543 | 3 | -0.12 | 364.1606 | 1 | 2.32 | 1021 | 3.39e4 | 1 | 1 | 459 | 461 | Pyro-glu from E | E1:Pyro-glu from E:1000 | DB Search |
| D.TPGF.G | N | 15.9 | 420.2009 | 4 | 2.59 | 421.2082 | 1 | 12.63 | 4943 | 3.01e3 | 1 | 1 | 159 | 162 |  |  | DB Search |
| H.QEPHAQ.P | Y | 15.76 | 708.3191 | 6 | -1.32 | 709.3237 | 1 | 2.84 | 1189 | 0 | 0 | 0 | 458 | 463 |  |  | DB Search |
| total 4 peptides |
| --- |

Best Unique PSM (Scan POS\_R15-1.wiff:1189, m/z=709.3113, z=1, RT=2.96, ppm=-3.81):


C4QVD8|C4QVD8\_KOMPG

back to list

  

| Protein Coverage
| Supporting Peptides
| Best Unique PSM
|

Protein Coverage:

Supporting Peptides:

| Peptide | Uniq | -10lgP | Mass | Length | ppm | m/z | z | RT | Scan | Area POS\_R15-1 | #Feature | #Feature POS\_R15-1 | Start | End | PTM | AScore | Found By |
| --- | --- | --- | --- | --- | --- | --- | --- | --- | --- | --- | --- | --- | --- | --- | --- | --- | --- |
| K.RIVP.L | N | 24.65 | 483.3169 | 4 | -4.88 | 484.3206 | 1 | 10.06 | 3922 | 3.47e3 | 1 | 1 | 386 | 389 |  |  | DB Search |
| L.PVLV.L | N | 23.27 | 426.2842 | 4 | 4.41 | 427.2923 | 1 | 20.36 | 7664 | 0 | 0 | 0 | 991 | 994 |  |  | DB Search |
| D.TFR.K | N | 20.53 | 422.2278 | 3 | -9.2 | 423.2301 | 1 | 22.38 | 8244 | 2.98e1 | 1 | 1 | 1054 | 1056 |  |  | DB Search |
| N.VSSL.N | N | 20.48 | 404.2271 | 4 | -7.08 | 405.2305 | 1 | 8.17 | 2993 | 7.47e3 | 1 | 1 | 316 | 319 |  |  | DB Search |
| D.LPLP.S | N | 20.33 | 438.2842 | 4 | -7.82 | 439.287 | 1 | 27.42 | 9635 | 1.51e3 | 1 | 1 | 729 | 732 |  |  | DB Search |
| E.KFH.Y | N | 20.21 | 430.2328 | 3 | 9.29 | 431.243 | 1 | 12.31 | 5009 | 5.31e3 | 1 | 1 | 63 | 65 |  |  | DB Search |
| E.NGSS.M | N | 20.12 | 363.139 | 4 | -0.16 | 364.1453 | 1 | 14.82 | 5815 | 1.67e3 | 1 | 1 | 901 | 904 |  |  | DB Search |
| Q.FISH.A | N | 19.74 | 502.254 | 4 | 7.98 | 503.264 | 1 | 15.82 | 6278 | 1.63e2 | 1 | 1 | 414 | 417 |  |  | DB Search |
| F.TAY.L | N | 19.3 | 353.1587 | 3 | -1.32 | 354.1646 | 1 | 12.51 | 4913 | 1.39e3 | 1 | 1 | 881 | 883 |  |  | DB Search |
| N.ESPI.V | N | 18.2 | 444.222 | 4 | -3.28 | 445.2267 | 1 | 12.52 | 4847 | 1.87e3 | 1 | 1 | 835 | 838 |  |  | DB Search |
| E.LYSS.M | N | 17.99 | 468.222 | 4 | 0.32 | 469.2283 | 1 | 16.06 | 6398 | 1.94e3 | 1 | 1 | 239 | 242 |  |  | DB Search |
| I.ESPF.S | N | 17.8 | 478.2063 | 4 | 2.35 | 479.2136 | 1 | 13.84 | 5519 | 6.96e2 | 1 | 1 | 631 | 634 |  |  | DB Search |
| I.SQAF.V | N | 16.62 | 451.2067 | 4 | -3.62 | 452.2112 | 1 | 10.42 | 4095 | 4.08e2 | 1 | 1 | 193 | 196 |  |  | DB Search |
| S.DAVT.R | N | 16.27 | 404.1907 | 4 | -1.99 | 405.1962 | 1 | 8.31 | 3091 | 8.02e2 | 1 | 1 | 9 | 12 |  |  | DB Search |
| F.FEAHPF.F | Y | 15.97 | 746.3387 | 6 | -3.18 | 747.3418 | 1 | 19.00 | 7244 | 4.86e1 | 1 | 1 | 547 | 552 |  |  | DB Search |
| total 15 peptides |
| --- |

Best Unique PSM (Scan POS\_R15-1.wiff:7244, m/z=747.3418, z=1, RT=19.00, ppm=-5.66):


C4QZ09|C4QZ09\_KOMPG

back to list

  

| Protein Coverage
| Supporting Peptides
| Best Unique PSM
|

Protein Coverage:

Supporting Peptides:

| Peptide | Uniq | -10lgP | Mass | Length | ppm | m/z | z | RT | Scan | Area POS\_R15-1 | #Feature | #Feature POS\_R15-1 | Start | End | PTM | AScore | Found By |
| --- | --- | --- | --- | --- | --- | --- | --- | --- | --- | --- | --- | --- | --- | --- | --- | --- | --- |
| Q.NGSS.D | N | 20.12 | 363.139 | 4 | -0.16 | 364.1453 | 1 | 14.82 | 5815 | 1.67e3 | 1 | 1 | 583 | 586 |  |  | DB Search |
| R.EGVP.R | N | 19.01 | 400.1958 | 4 | -2.37 | 401.2011 | 1 | 8.27 | 3077 | 4.2e3 | 1 | 1 | 391 | 394 |  |  | DB Search |
| G.E(-18.01)QQPGAF.I | Y | 15.16 | 757.3395 | 7 | 1.01 | 758.3456 | 1 | 18.47 | 7142 | 0 | 0 | 0 | 492 | 498 | Pyro-glu from E | E1:Pyro-glu from E:1000 | DB Search |
| total 3 peptides |
| --- |

Best Unique PSM (Scan POS\_R15-1.wiff:7142, m/z=758.3456, z=1, RT=18.47, ppm=-1.48):


C4QXT4|C4QXT4\_KOMPG

back to list

  

| Protein Coverage
| Supporting Peptides
| Best Unique PSM
|

Protein Coverage:

Supporting Peptides:

| Peptide | Uniq | -10lgP | Mass | Length | ppm | m/z | z | RT | Scan | Area POS\_R15-1 | #Feature | #Feature POS\_R15-1 | Start | End | PTM | AScore | Found By |
| --- | --- | --- | --- | --- | --- | --- | --- | --- | --- | --- | --- | --- | --- | --- | --- | --- | --- |
| E.NII.Q | N | 28.22 | 358.2216 | 3 | -4.68 | 359.2263 | 1 | 19.18 | 7326 | 4.19e3 | 1 | 1 | 67 | 69 |  |  | DB Search |
| S.LPPP.Q | N | 27.73 | 422.2529 | 4 | -1.45 | 423.2585 | 1 | 9.57 | 3721 | 1.63e3 | 1 | 1 | 98 | 101 |  |  | DB Search |
| T.SPIL.S | N | 26.66 | 428.2635 | 4 | -1.63 | 429.269 | 1 | 21.35 | 7905 | 0 | 0 | 0 | 473 | 476 |  |  | DB Search |
| Q.YSLF.V | N | 26.31 | 528.2584 | 4 | -2.91 | 529.2628 | 1 | 34.97 | 11598 | 3.74e3 | 1 | 1 | 156 | 159 |  |  | DB Search |
| F.VGGL.A | N | 24.87 | 344.2059 | 4 | -5.22 | 345.2106 | 1 | 10.74 | 4195 | 1.51e3 | 1 | 1 | 282 | 285 |  |  | DB Search |
| A.SPT.V | N | 23.16 | 303.143 | 3 | 6.39 | 304.1515 | 1 | 7.14 | 2731 | 5.4e3 | 2 | 2 | 501 | 503 |  |  | DB Search |
| S.RCF.G | N | 21.23 | 424.1893 | 3 | -9.2 | 425.1916 | 1 | 10.20 | 3945 | 1.22e3 | 1 | 1 | 198 | 200 |  |  | DB Search |
| P.SSKSS.P | N | 21.18 | 494.2336 | 5 | -2.91 | 495.2383 | 1 | 12.41 | 4849 | 0 | 0 | 0 | 42 | 46 |  |  | DB Search |
| P.ELA.S | N | 21.09 | 331.1743 | 3 | -5.12 | 332.1791 | 1 | 6.98 | 2655 | 4.41e3 | 1 | 1 | 498 | 500 |  |  | DB Search |
| P.MGR.N | N | 20.83 | 362.1736 | 3 | 6.82 | 363.1825 | 1 | 3.30 | 1420 | 1.5e3 | 1 | 1 | 376 | 378 |  |  | DB Search |
| H.PH.P | N | 19.52 | 252.1222 | 2 | -5.45 | 253.1275 | 1 | 5.57 | 2095 | 1.25e4 | 1 | 1 | 361 | 362 |  |  | DB Search |
| H.EM.S | N | 19.13 | 278.0936 | 2 | 0.1 | 279.1003 | 1 | 3.52 | 1504 | 7.14e3 | 1 | 1 | 217 | 218 |  |  | DB Search |
| S.RT.L | N | 19.04 | 275.1593 | 2 | 3.6 | 276.1669 | 1 | 5.27 | 2115 | 2.64e3 | 1 | 1 | 52 | 53 |  |  | DB Search |
| V.PR.S | N | 18.98 | 271.1644 | 2 | -8.93 | 272.1686 | 1 | 5.03 | 2023 | 0 | 0 | 0 | 129 | 130 |  |  | DB Search |
| P.IRV.A | N | 18.9 | 386.2641 | 3 | -1.28 | 387.27 | 1 | 27.77 | 9760 | 0 | 0 | 0 | 228 | 230 |  |  | DB Search |
| N.AISGMH.G | Y | 18.6 | 614.2846 | 6 | 0.26 | 615.2905 | 1 | 23.61 | 8583 | 2.74e2 | 1 | 1 | 329 | 334 |  |  | DB Search |
| S.SPSL.A | N | 18.22 | 402.2114 | 4 | -3.42 | 403.2163 | 1 | 12.42 | 4855 | 2.18e3 | 1 | 1 | 46 | 49 |  |  | DB Search |
| V.GSSI.G | N | 18.21 | 362.1801 | 4 | -9.58 | 363.183 | 1 | 6.30 | 2465 | 1.38e3 | 1 | 1 | 428 | 431 |  |  | DB Search |
| Y.YP.T | N | 17.92 | 278.1266 | 2 | -4.14 | 279.1321 | 1 | 3.49 | 1522 | 2.89e2 | 1 | 1 | 402 | 403 |  |  | DB Search |
| S.HP.H | N | 17.77 | 252.1222 | 2 | -2.49 | 253.1283 | 1 | 5.54 | 2216 | 1.25e4 | 1 | 1 | 360 | 361 |  |  | DB Search |
| Q.YSL.F | N | 17.18 | 381.19 | 3 | 2.91 | 382.1974 | 1 | 6.77 | 2598 | 3.84e2 | 1 | 1 | 156 | 158 |  |  | DB Search |
| F.ERH.E | N | 16.79 | 440.2132 | 3 | -0.12 | 441.2193 | 1 | 19.25 | 7313 | 6.39e2 | 1 | 1 | 112 | 114 |  |  | DB Search |
| H.LGVT.D | N | 16.69 | 388.2322 | 4 | -3.81 | 389.237 | 1 | 9.87 | 3731 | 6.08e3 | 1 | 1 | 6 | 9 |  |  | DB Search |
| total 23 peptides |
| --- |

Best Unique PSM (Scan POS\_R15-1.wiff:8583, m/z=616.3643, z=1, RT=23.76, ppm=-2.23):


C4QVN9|C4QVN9\_KOMPG

back to list

  

| Protein Coverage
| Supporting Peptides
| Best Unique PSM
|

Protein Coverage:

Supporting Peptides:

| Peptide | Uniq | -10lgP | Mass | Length | ppm | m/z | z | RT | Scan | Area POS\_R15-1 | #Feature | #Feature POS\_R15-1 | Start | End | PTM | AScore | Found By |
| --- | --- | --- | --- | --- | --- | --- | --- | --- | --- | --- | --- | --- | --- | --- | --- | --- | --- |
| K.NGI.V | N | 28.25 | 302.159 | 3 | -3.76 | 303.1644 | 1 | 6.06 | 2374 | 3.14e3 | 1 | 1 | 23 | 25 |  |  | DB Search |
| F.IGGI.A | N | 27.34 | 358.2216 | 4 | -4.39 | 359.2264 | 1 | 15.45 | 6114 | 7.1e3 | 1 | 1 | 168 | 171 |  |  | DB Search |
| F.LGSI.V | N | 25.26 | 388.2322 | 4 | -3.81 | 389.237 | 1 | 9.87 | 3785 | 6.08e3 | 1 | 1 | 207 | 210 |  |  | DB Search |
| G.SIVGL.E | N | 22.5 | 487.3006 | 5 | -4.27 | 488.3046 | 1 | 21.68 | 8060 | 8.06e3 | 1 | 1 | 209 | 213 |  |  | DB Search |
| G.SSIF.D | N | 21.63 | 452.2271 | 4 | -3.7 | 453.2316 | 1 | 22.49 | 8262 | 1.33e3 | 1 | 1 | 224 | 227 |  |  | DB Search |
| I.VTTL.L | N | 20.12 | 432.2584 | 4 | -4.38 | 433.2627 | 1 | 11.15 | 4417 | 0 | 0 | 0 | 9 | 12 |  |  | DB Search |
| L.ISVGI.V | N | 20.08 | 487.3006 | 5 | -4.27 | 488.3046 | 1 | 21.68 | 7886 | 8.06e3 | 1 | 1 | 4 | 8 |  |  | DB Search |
| N.FPS.S | N | 19.27 | 349.1638 | 3 | -4.81 | 350.1685 | 1 | 16.32 | 6461 | 1.41e3 | 1 | 1 | 193 | 195 |  |  | DB Search |
| I.SVGI.V | N | 18.49 | 374.2165 | 4 | -4.96 | 375.221 | 1 | 14.62 | 5748 | 1.93e2 | 1 | 1 | 5 | 8 |  |  | DB Search |
| L.GSSI.F | N | 18.21 | 362.1801 | 4 | -9.58 | 363.183 | 1 | 6.30 | 2465 | 1.38e3 | 1 | 1 | 223 | 226 |  |  | DB Search |
| Q.EFIA.S | N | 18.15 | 478.2427 | 4 | -2.38 | 479.2477 | 1 | 20.03 | 7568 | 1.11e3 | 2 | 2 | 122 | 125 |  |  | DB Search |
| I.TW.T | N | 18.09 | 305.1375 | 2 | -1.88 | 306.1435 | 1 | 9.22 | 3551 | 5.58e3 | 1 | 1 | 181 | 182 |  |  | DB Search |
| T.VPAGL.S | N | 17.32 | 455.2744 | 5 | -4.8 | 456.2783 | 1 | 20.80 | 7635 | 5.46e3 | 1 | 1 | 159 | 163 |  |  | DB Search |
| T.WT.V | N | 17.04 | 305.1375 | 2 | -1.68 | 306.1436 | 1 | 9.15 | 3517 | 5.58e3 | 1 | 1 | 182 | 183 |  |  | DB Search |
| L.FKAS.I | N | 16.8 | 451.2431 | 4 | 8.89 | 452.2532 | 1 | 15.62 | 6149 | 2.82e2 | 1 | 1 | 231 | 234 |  |  | DB Search |
| L.GSSIFDT.L | Y | 16.14 | 725.3232 | 7 | -4.08 | 726.3257 | 1 | 14.90 | 5886 | 9.8e2 | 1 | 1 | 223 | 229 |  |  | DB Search |
| P.AGLSL.I | N | 15.43 | 459.2693 | 5 | -3.77 | 460.2737 | 1 | 22.08 | 8169 | 4.69e2 | 1 | 1 | 161 | 165 |  |  | DB Search |
| T.KSIP.L | N | 15.42 | 443.2744 | 4 | -3.64 | 444.2789 | 1 | 17.16 | 6753 | 0 | 0 | 0 | 38 | 41 |  |  | DB Search |
| S.Q(-17.03)PF.D | N | 15.22 | 373.1638 | 3 | -0.75 | 374.1698 | 1 | 21.41 | 7929 | 8.45e4 | 1 | 1 | 103 | 105 | Pyro-glu from Q | Q1:Pyro-glu from Q:1000 | DB Search |
| Q.QVAL.S | N | 15.07 | 429.2587 | 4 | 5.6 | 430.2673 | 1 | 21.95 | 8124 | 0 | 0 | 0 | 64 | 67 |  |  | DB Search |
| total 20 peptides |
| --- |

Best Unique PSM (Scan POS\_R15-1.wiff:5886, m/z=726.3257, z=1, RT=14.90, ppm=-6.56):


C4QZN7|C4QZN7\_KOMPG

back to list

  

| Protein Coverage
| Supporting Peptides
| Best Unique PSM
|

Protein Coverage:

Supporting Peptides:

| Peptide | Uniq | -10lgP | Mass | Length | ppm | m/z | z | RT | Scan | Area POS\_R15-1 | #Feature | #Feature POS\_R15-1 | Start | End | PTM | AScore | Found By |
| --- | --- | --- | --- | --- | --- | --- | --- | --- | --- | --- | --- | --- | --- | --- | --- | --- | --- |
| L.NLI.R | N | 28.22 | 358.2216 | 3 | -4.68 | 359.2263 | 1 | 19.18 | 7326 | 4.19e3 | 1 | 1 | 97 | 99 |  |  | DB Search |
| K.NLI.Q | N | 28.22 | 358.2216 | 3 | -4.68 | 359.2263 | 1 | 19.18 | 7326 | 4.19e3 | 1 | 1 | 64 | 66 |  |  | DB Search |
| H.SPLL.P | N | 26.66 | 428.2635 | 4 | -1.63 | 429.269 | 1 | 21.35 | 7905 | 0 | 0 | 0 | 348 | 351 |  |  | DB Search |
| Q.LGSI.V | N | 25.26 | 388.2322 | 4 | -3.81 | 389.237 | 1 | 9.87 | 3785 | 6.08e3 | 1 | 1 | 569 | 572 |  |  | DB Search |
| Y.SPT.H | N | 23.16 | 303.143 | 3 | 6.39 | 304.1515 | 1 | 7.14 | 2731 | 5.4e3 | 2 | 2 | 404 | 406 |  |  | DB Search |
| F.ELF.S | N | 21.7 | 407.2056 | 3 | -8.99 | 408.2082 | 1 | 29.23 | 10157 | 9.42e2 | 1 | 1 | 645 | 647 |  |  | DB Search |
| H.FLSIVP.N | N | 20.93 | 674.4003 | 6 | -6.34 | 675.4016 | 1 | 42.02 | 13315 | 6.45e2 | 1 | 1 | 150 | 155 |  |  | DB Search |
| F.RCL.A | N | 20.6 | 390.2049 | 3 | -4.41 | 391.2095 | 1 | 8.08 | 3003 | 0 | 0 | 0 | 228 | 230 |  |  | DB Search |
| K.NSG.A | N | 20.42 | 276.107 | 3 | 6.48 | 277.1154 | 1 | 16.74 | 6592 | 0 | 0 | 0 | 530 | 532 |  |  | DB Search |
| G.PH.Q | N | 19.52 | 252.1222 | 2 | -5.45 | 253.1275 | 1 | 5.57 | 2095 | 1.25e4 | 1 | 1 | 596 | 597 |  |  | DB Search |
| T.RT.Q | N | 19.04 | 275.1593 | 2 | 3.6 | 276.1669 | 1 | 5.27 | 2115 | 2.64e3 | 1 | 1 | 80 | 81 |  |  | DB Search |
| I.LRV.I | N | 18.9 | 386.2641 | 3 | -1.28 | 387.27 | 1 | 27.77 | 9760 | 0 | 0 | 0 | 99 | 101 |  |  | DB Search |
| C.HAVI.Y | N | 18.78 | 438.259 | 4 | -6.35 | 439.2625 | 1 | 8.70 | 3348 | 6.21e2 | 1 | 1 | 162 | 165 |  |  | DB Search |
| L.L(+42.01)DVEPQQ.D | Y | 18.28 | 869.413 | 7 | -0.79 | 870.4175 | 1 | 5.61 | 2245 | 0 | 0 | 0 | 15 | 21 | Acetylation (Protein N-term) | L1:Acetylation (Protein N-term):1000 | DB Search |
| K.LELD.N | N | 17.9 | 488.2482 | 4 | 1 | 489.2548 | 1 | 13.83 | 5478 | 0 | 0 | 0 | 499 | 502 |  |  | DB Search |
| V.FRC.L | N | 17.7 | 424.1893 | 3 | -9.2 | 425.1916 | 1 | 10.20 | 3929 | 1.22e3 | 1 | 1 | 227 | 229 |  |  | DB Search |
| Q.LLTD.Q | N | 17.52 | 460.2533 | 4 | -9.09 | 461.2552 | 1 | 10.16 | 3965 | 9.46e2 | 1 | 1 | 317 | 320 |  |  | DB Search |
| N.FNH.S | N | 17.22 | 416.1808 | 3 | -5.74 | 417.1847 | 1 | 6.08 | 2390 | 0 | 0 | 0 | 345 | 347 |  |  | DB Search |
| C.ESN.M | N | 17.14 | 348.1281 | 3 | 8.51 | 349.1375 | 1 | 5.67 | 2161 | 1.07e4 | 1 | 1 | 339 | 341 |  |  | DB Search |
| L.ELLD.S | N | 16.42 | 488.2482 | 4 | -0.37 | 489.2541 | 1 | 13.94 | 5546 | 0 | 0 | 0 | 174 | 177 |  |  | DB Search |
| A.TKC.Y | N | 16.14 | 350.1624 | 3 | 7.67 | 351.1715 | 1 | 4.93 | 1958 | 0 | 0 | 0 | 275 | 277 |  |  | DB Search |
| total 21 peptides |
| --- |

Best Unique PSM (Scan POS\_R15-1.wiff:2245, m/z=870.4221, z=1, RT=5.69, ppm=-3.27):


C4R5C0|C4R5C0\_KOMPG

back to list

  

| Protein Coverage
| Supporting Peptides
| Best Unique PSM
|

Protein Coverage:

Supporting Peptides:

| Peptide | Uniq | -10lgP | Mass | Length | ppm | m/z | z | RT | Scan | Area POS\_R15-1 | #Feature | #Feature POS\_R15-1 | Start | End | PTM | AScore | Found By |
| --- | --- | --- | --- | --- | --- | --- | --- | --- | --- | --- | --- | --- | --- | --- | --- | --- | --- |
| R.LGSL.K | N | 25.26 | 388.2322 | 4 | -3.81 | 389.237 | 1 | 9.87 | 3785 | 6.08e3 | 1 | 1 | 415 | 418 |  |  | DB Search |
| T.SPT.P | N | 23.16 | 303.143 | 3 | 6.39 | 304.1515 | 1 | 7.14 | 2731 | 5.4e3 | 2 | 2 | 494 | 496 |  |  | DB Search |
| K.TSG.V | N | 21.44 | 263.1117 | 3 | 2.31 | 264.119 | 1 | 3.71 | 1537 | 5.72e3 | 1 | 1 | 400 | 402 |  |  | DB Search |
| V.RCL.K | N | 20.6 | 390.2049 | 3 | -4.41 | 391.2095 | 1 | 8.08 | 3003 | 0 | 0 | 0 | 50 | 52 |  |  | DB Search |
| P.STSP.T | N | 19.15 | 390.1751 | 4 | -0.08 | 391.1813 | 1 | 5.92 | 2360 | 3.31e2 | 1 | 1 | 544 | 547 |  |  | DB Search |
| L.AFR.R | N | 19.1 | 392.2172 | 3 | -7.11 | 393.2207 | 1 | 6.33 | 2475 | 0 | 0 | 0 | 801 | 803 |  |  | DB Search |
| A.SNG.S | N | 19.07 | 276.107 | 3 | 1.61 | 277.114 | 1 | 16.82 | 6604 | 0 | 0 | 0 | 612 | 614 |  |  | DB Search |
| S.RSSS.V | N | 18.8 | 435.2078 | 4 | -3.98 | 436.2122 | 1 | 11.38 | 4548 | 7.31e2 | 1 | 1 | 447 | 450 |  |  | DB Search |
| K.FGRL.F | N | 17.51 | 491.2856 | 4 | -7.95 | 492.2878 | 1 | 15.01 | 5965 | 9.54e2 | 1 | 1 | 848 | 851 |  |  | DB Search |
| D.RSF.N | N | 17.5 | 408.2121 | 3 | -1.76 | 409.2177 | 1 | 9.17 | 3526 | 0 | 0 | 0 | 93 | 95 |  |  | DB Search |
| S.AVVA.S | N | 16.9 | 358.2216 | 4 | -4.99 | 359.2262 | 1 | 6.95 | 2675 | 0 | 0 | 0 | 529 | 532 |  |  | DB Search |
| G.LGGV.R | N | 15.94 | 344.2059 | 4 | 0.19 | 345.2124 | 1 | 5.48 | 2143 | 3.05e3 | 1 | 1 | 784 | 787 |  |  | DB Search |
| V.STAI.E | N | 15.79 | 390.2114 | 4 | -5.32 | 391.2157 | 1 | 6.25 | 2317 | 1.07e4 | 1 | 1 | 344 | 347 |  |  | DB Search |
| S.RAPGLG.G | Y | 15.75 | 569.3285 | 6 | -3.81 | 570.3322 | 1 | 5.74 | 2271 | 0 | 0 | 0 | 780 | 785 |  |  | DB Search |
| total 14 peptides |
| --- |

Best Unique PSM (Scan POS\_R15-1.wiff:2271, m/z=569.2428, z=1, RT=6.01, ppm=-6.29):


C4QXL2|C4QXL2\_KOMPG

back to list

  

| Protein Coverage
| Supporting Peptides
| Best Unique PSM
|

Protein Coverage:

Supporting Peptides:

| Peptide | Uniq | -10lgP | Mass | Length | ppm | m/z | z | RT | Scan | Area POS\_R15-1 | #Feature | #Feature POS\_R15-1 | Start | End | PTM | AScore | Found By |
| --- | --- | --- | --- | --- | --- | --- | --- | --- | --- | --- | --- | --- | --- | --- | --- | --- | --- |
| Q.TLPT.T | N | 26.11 | 430.2427 | 4 | -4.36 | 431.2471 | 1 | 9.16 | 3520 | 0 | 0 | 0 | 397 | 400 |  |  | DB Search |
| M.LPTP.P | N | 25.97 | 426.2478 | 4 | -3.74 | 427.2524 | 1 | 12.64 | 4915 | 1.93e3 | 1 | 1 | 966 | 969 |  |  | DB Search |
| G.LPQQP.W | N | 25.19 | 581.3173 | 5 | -2.43 | 582.3217 | 1 | 8.89 | 3457 | 0 | 0 | 0 | 4 | 8 |  |  | DB Search |
| Q.Q(-17.03)PQQP.P | N | 22.54 | 579.2653 | 5 | -5.46 | 580.2679 | 1 | 8.21 | 3095 | 8.92e3 | 1 | 1 | 279 | 283 | Pyro-glu from Q | Q1:Pyro-glu from Q:1000 | DB Search |
| P.QQPPQQ.S | Y | 20.62 | 724.3504 | 6 | -4.61 | 725.3525 | 1 | 3.38 | 1468 | 0 | 0 | 0 | 281 | 286 |  |  | DB Search |
| K.VSSI.K | N | 20.48 | 404.2271 | 4 | -7.08 | 405.2305 | 1 | 8.17 | 2993 | 7.47e3 | 1 | 1 | 72 | 75 |  |  | DB Search |
| L.E(-18.01)QQVP.Q | N | 19.25 | 581.2809 | 5 | -4.42 | 582.2842 | 1 | 10.75 | 4255 | 1.02e3 | 1 | 1 | 747 | 751 | Pyro-glu from E | E1:Pyro-glu from E:1000 | DB Search |
| S.ATQQP.Q | N | 19.21 | 543.2653 | 5 | 1.21 | 544.2719 | 1 | 6.86 | 2639 | 0 | 0 | 0 | 288 | 292 |  |  | DB Search |
| Q.FPPQ.Q | N | 18.18 | 487.2431 | 4 | -6.62 | 488.2459 | 1 | 11.19 | 4443 | 2.22e3 | 1 | 1 | 690 | 693 |  |  | DB Search |
| S.E(-18.01)LNP.K | N | 16.86 | 453.2223 | 4 | -6.06 | 454.2257 | 1 | 14.47 | 5683 | 1.68e4 | 1 | 1 | 12 | 15 | Pyro-glu from E | E1:Pyro-glu from E:1000 | DB Search |
| A.TLNG.M | N | 15.96 | 403.2067 | 4 | -9.11 | 404.2093 | 1 | 5.03 | 2025 | 0 | 0 | 0 | 96 | 99 |  |  | DB Search |
| A.EALQ.L | N | 15.76 | 459.2329 | 4 | -3.37 | 460.2375 | 1 | 4.47 | 1741 | 3.16e3 | 2 | 2 | 471 | 474 |  |  | DB Search |
| F.IKTP.G | N | 15.64 | 457.29 | 4 | -5.17 | 458.2938 | 1 | 15.66 | 6234 | 0 | 0 | 0 | 917 | 920 |  |  | DB Search |
| N.SLMPNQ.I | Y | 15.07 | 688.3214 | 6 | 5.93 | 689.3311 | 1 | 16.46 | 6532 | 1.45e2 | 1 | 1 | 681 | 686 |  |  | DB Search |
| M.QQLP.N | N | 15.07 | 484.2645 | 4 | -3.24 | 485.269 | 1 | 10.16 | 3975 | 0 | 0 | 0 | 726 | 729 |  |  | DB Search |
| total 15 peptides |
| --- |

Best Unique PSM (Scan POS\_R15-1.wiff:1468, m/z=725.3525, z=1, RT=3.38, ppm=-7.09):


C4QX36|C4QX36\_KOMPG

back to list

  

| Protein Coverage
| Supporting Peptides
| Best Unique PSM
|

Protein Coverage:

Supporting Peptides:

| Peptide | Uniq | -10lgP | Mass | Length | ppm | m/z | z | RT | Scan | Area POS\_R15-1 | #Feature | #Feature POS\_R15-1 | Start | End | PTM | AScore | Found By |
| --- | --- | --- | --- | --- | --- | --- | --- | --- | --- | --- | --- | --- | --- | --- | --- | --- | --- |
| S.ETW.S | N | 26.2 | 434.1801 | 3 | -4.77 | 435.1843 | 1 | 12.45 | 4871 | 1.27e3 | 1 | 1 | 155 | 157 |  |  | DB Search |
| L.EIIR.N | N | 25.05 | 529.3224 | 4 | -4.56 | 530.3259 | 1 | 17.34 | 6855 | 6.68e3 | 1 | 1 | 376 | 379 |  |  | DB Search |
| M.EIW.K | N | 21.42 | 446.2165 | 3 | -5.13 | 447.2204 | 1 | 31.30 | 10711 | 1.59e3 | 1 | 1 | 382 | 384 |  |  | DB Search |
| V.PGE.N | N | 21.28 | 301.1274 | 3 | 1.88 | 302.1345 | 1 | 2.04 | 869 | 2.57e3 | 1 | 1 | 127 | 129 |  |  | DB Search |
| A.TFR.L | N | 20.53 | 422.2278 | 3 | -9.2 | 423.2301 | 1 | 22.38 | 8244 | 2.98e1 | 1 | 1 | 7 | 9 |  |  | DB Search |
| K.NDT.L | N | 19.64 | 348.1281 | 3 | 7.11 | 349.137 | 1 | 7.31 | 2704 | 6.3e4 | 1 | 1 | 80 | 82 |  |  | DB Search |
| A.VAVG.S | N | 18.17 | 344.2059 | 4 | -3.72 | 345.2111 | 1 | 5.51 | 2206 | 3.05e3 | 1 | 1 | 239 | 242 |  |  | DB Search |
| D.AAQ.S | N | 16.83 | 288.1434 | 3 | 8 | 289.1522 | 1 | 5.91 | 2315 | 6.8e3 | 1 | 1 | 151 | 153 |  |  | DB Search |
| D.WPR.E | N | 16.44 | 457.2437 | 3 | 4.14 | 458.2518 | 1 | 16.95 | 6653 | 0 | 0 | 0 | 69 | 71 |  |  | DB Search |
| G.SGYV.Y | N | 15.39 | 424.1958 | 4 | 2.7 | 425.2032 | 1 | 2.77 | 1161 | 4e2 | 1 | 1 | 243 | 246 |  |  | DB Search |
| E.NGHTP.S | Y | 15.34 | 524.2343 | 5 | -9.5 | 525.2353 | 1 | 25.52 | 9077 | 2.89e2 | 1 | 1 | 284 | 288 |  |  | DB Search |
| total 11 peptides |
| --- |

Best Unique PSM (Scan POS\_R15-1.wiff:9077, m/z=525.2353, z=1, RT=25.52, ppm=-11.99):


C4R5L7|C4R5L7\_KOMPG

back to list

  

| Protein Coverage
| Supporting Peptides
| Best Unique PSM
|

Protein Coverage:

Supporting Peptides:

| Peptide | Uniq | -10lgP | Mass | Length | ppm | m/z | z | RT | Scan | Area POS\_R15-1 | #Feature | #Feature POS\_R15-1 | Start | End | PTM | AScore | Found By |
| --- | --- | --- | --- | --- | --- | --- | --- | --- | --- | --- | --- | --- | --- | --- | --- | --- | --- |
| A.LGGL.G | N | 27.34 | 358.2216 | 4 | -4.39 | 359.2264 | 1 | 15.45 | 6114 | 7.1e3 | 1 | 1 | 227 | 230 |  |  | DB Search |
| G.TLVL.G | N | 26.01 | 444.2948 | 4 | -4 | 445.2992 | 1 | 28.31 | 9909 | 1.89e3 | 1 | 1 | 605 | 608 |  |  | DB Search |
| L.VIGS.G | N | 24.2 | 374.2165 | 4 | -4.06 | 375.2213 | 1 | 5.20 | 2012 | 6.27e3 | 1 | 1 | 65 | 68 |  |  | DB Search |
| Y.PVLV.R | N | 23.27 | 426.2842 | 4 | 4.41 | 427.2923 | 1 | 20.36 | 7664 | 0 | 0 | 0 | 754 | 757 |  |  | DB Search |
| L.SIGQAGE.F | N | 22.97 | 660.3078 | 7 | -6.95 | 661.3089 | 1 | 12.24 | 4780 | 1.27e2 | 1 | 1 | 72 | 78 |  |  | DB Search |
| N.YNP.E | N | 21.45 | 392.1696 | 3 | -3.75 | 393.1744 | 1 | 8.45 | 3226 | 1.39e2 | 1 | 1 | 644 | 646 |  |  | DB Search |
| L.SLAP.Q | N | 20.83 | 386.2165 | 4 | -4.65 | 387.221 | 1 | 8.35 | 3276 | 4.13e3 | 1 | 1 | 250 | 253 |  |  | DB Search |
| E.ITGPF.N | Y | 19.12 | 533.2849 | 5 | -3.52 | 534.289 | 1 | 28.06 | 9841 | 0 | 0 | 0 | 865 | 869 |  |  | DB Search |
| Q.PAW.K | N | 18.43 | 372.1797 | 3 | -0.47 | 373.1859 | 1 | 13.13 | 5130 | 2.29e3 | 1 | 1 | 733 | 735 |  |  | DB Search |
| E.LEAIGS.L | Y | 17.77 | 588.3119 | 6 | -4.97 | 589.3148 | 1 | 13.66 | 5377 | 1.66e4 | 1 | 1 | 519 | 524 |  |  | DB Search |
| V.IEFP.K | N | 17.54 | 504.2584 | 4 | -6.47 | 505.2611 | 1 | 30.11 | 10334 | 3.53e2 | 2 | 2 | 1043 | 1046 |  |  | DB Search |
| G.ADPF.L | N | 15.69 | 448.1958 | 4 | -3.76 | 449.2003 | 1 | 15.12 | 5897 | 8.13e2 | 1 | 1 | 952 | 955 |  |  | DB Search |
| A.VSEHVE.N | N | 15.1 | 698.3235 | 6 | -4.9 | 699.3256 | 1 | 15.99 | 6329 | 0 | 0 | 0 | 821 | 826 |  |  | DB Search |
| total 13 peptides |
| --- |

Best Unique PSM (Scan POS\_R15-1.wiff:9841, m/z=534.291, z=1, RT=28.10, ppm=-6.01):


C4QVG3|C4QVG3\_KOMPG

back to list

  

| Protein Coverage
| Supporting Peptides
| Best Unique PSM
|

Protein Coverage:

Supporting Peptides:

| Peptide | Uniq | -10lgP | Mass | Length | ppm | m/z | z | RT | Scan | Area POS\_R15-1 | #Feature | #Feature POS\_R15-1 | Start | End | PTM | AScore | Found By |
| --- | --- | --- | --- | --- | --- | --- | --- | --- | --- | --- | --- | --- | --- | --- | --- | --- | --- |
| G.SIPT.N | N | 22.87 | 416.2271 | 4 | -6.88 | 417.2305 | 1 | 14.64 | 5767 | 5.65e3 | 1 | 1 | 499 | 502 |  |  | DB Search |
| L.ISGL.I | N | 22.6 | 388.2322 | 4 | -3.81 | 389.237 | 1 | 9.87 | 3804 | 6.08e3 | 1 | 1 | 246 | 249 |  |  | DB Search |
| Y.PSVP.L | N | 21.16 | 398.2165 | 4 | 4.76 | 399.2247 | 1 | 9.14 | 3512 | 0 | 0 | 0 | 283 | 286 |  |  | DB Search |
| L.LLQP.I | N | 19.13 | 469.29 | 4 | -8.42 | 470.2922 | 1 | 12.16 | 4756 | 6.43e3 | 1 | 1 | 313 | 316 |  |  | DB Search |
| S.HGY.V | N | 18.63 | 375.1543 | 3 | -9 | 376.1572 | 1 | 4.83 | 1959 | 3.43e3 | 1 | 1 | 516 | 518 |  |  | DB Search |
| H.GHY.Q | N | 18.12 | 375.1543 | 3 | -2.57 | 376.1596 | 1 | 4.82 | 1883 | 3.43e3 | 1 | 1 | 511 | 513 |  |  | DB Search |
| L.Q(-17.03)PIL.L | N | 17.35 | 452.2635 | 4 | -6.01 | 453.2669 | 1 | 32.30 | 10953 | 1.74e3 | 1 | 1 | 315 | 318 | Pyro-glu from Q | Q1:Pyro-glu from Q:1000 | DB Search |
| L.GSAS.A | N | 17.29 | 320.1332 | 4 | 7.75 | 321.1422 | 1 | 6.30 | 2464 | 0 | 0 | 0 | 635 | 638 |  |  | DB Search |
| D.EVVQ.I | N | 17.07 | 473.2485 | 4 | 0.37 | 474.2548 | 1 | 9.22 | 3556 | 4.07e3 | 1 | 1 | 180 | 183 |  |  | DB Search |
| E.AALG.S | N | 16.78 | 330.1903 | 4 | -8.43 | 331.194 | 1 | 5.91 | 2160 | 5.17e3 | 1 | 1 | 609 | 612 |  |  | DB Search |
| F.E(-18.01)QPFP.K | N | 16.73 | 598.2751 | 5 | -6.33 | 599.2771 | 1 | 25.30 | 9006 | 1.93e3 | 1 | 1 | 229 | 233 | Pyro-glu from E | E1:Pyro-glu from E:1000 | DB Search |
| E.RLC.Q | N | 16.16 | 390.2049 | 3 | 4.43 | 391.213 | 1 | 3.37 | 1421 | 1.01e3 | 1 | 1 | 277 | 279 |  |  | DB Search |
| S.KKFGNSRF.T | Y | 15.49 | 982.5348 | 8 | 9.75 | 492.2783 | 2 | 24.96 | 8989 | 0 | 0 | 0 | 46 | 53 |  |  | DB Search |
| Q.Q(-17.03)PH.P | N | 15.48 | 363.1543 | 3 | -0.14 | 364.1606 | 1 | 2.32 | 992 | 3.39e4 | 1 | 1 | 405 | 407 | Pyro-glu from Q | Q1:Pyro-glu from Q:1000 | DB Search |
| total 14 peptides |
| --- |

Best Unique PSM (Scan POS\_R15-1.wiff:8989, m/z=492.2527, z=1, RT=25.18, ppm=7.26):


C4QVH8|C4QVH8\_KOMPG

back to list

  

| Protein Coverage
| Supporting Peptides
| Best Unique PSM
|

Protein Coverage:

Supporting Peptides:

| Peptide | Uniq | -10lgP | Mass | Length | ppm | m/z | z | RT | Scan | Area POS\_R15-1 | #Feature | #Feature POS\_R15-1 | Start | End | PTM | AScore | Found By |
| --- | --- | --- | --- | --- | --- | --- | --- | --- | --- | --- | --- | --- | --- | --- | --- | --- | --- |
| V.SLAP.S | N | 20.83 | 386.2165 | 4 | -4.65 | 387.221 | 1 | 8.35 | 3276 | 4.13e3 | 1 | 1 | 377 | 380 |  |  | DB Search |
| W.DLVT.A | N | 16.75 | 446.2376 | 4 | -4.95 | 447.2416 | 1 | 23.24 | 8484 | 0 | 0 | 0 | 313 | 316 |  |  | DB Search |
| I.SATTIAI.L | Y | 16.29 | 675.3803 | 7 | -1.84 | 676.3846 | 1 | 24.51 | 8862 | 2.41e3 | 1 | 1 | 196 | 202 |  |  | DB Search |
| total 3 peptides |
| --- |

Best Unique PSM (Scan POS\_R15-1.wiff:8862, m/z=676.3514, z=2, RT=24.78, ppm=-4.33):


C4QWZ3|C4QWZ3\_KOMPG

back to list

  

| Protein Coverage
| Supporting Peptides
| Best Unique PSM
|

Protein Coverage:

Supporting Peptides:

| Peptide | Uniq | -10lgP | Mass | Length | ppm | m/z | z | RT | Scan | Area POS\_R15-1 | #Feature | #Feature POS\_R15-1 | Start | End | PTM | AScore | Found By |
| --- | --- | --- | --- | --- | --- | --- | --- | --- | --- | --- | --- | --- | --- | --- | --- | --- | --- |
| L.ELLR.G | N | 25.05 | 529.3224 | 4 | -4.56 | 530.3259 | 1 | 17.34 | 6855 | 6.68e3 | 1 | 1 | 297 | 300 |  |  | DB Search |
| N.LSGI.D | N | 22.6 | 388.2322 | 4 | -3.81 | 389.237 | 1 | 9.87 | 3804 | 6.08e3 | 1 | 1 | 406 | 409 |  |  | DB Search |
| W.SSLF.M | N | 21.63 | 452.2271 | 4 | -3.7 | 453.2316 | 1 | 22.49 | 8262 | 1.33e3 | 1 | 1 | 249 | 252 |  |  | DB Search |
| E.THH.I | N | 21.55 | 393.1761 | 3 | -4.54 | 394.1806 | 1 | 2.11 | 908 | 2.12e3 | 1 | 1 | 390 | 392 |  |  | DB Search |
| G.RCF.G | N | 21.23 | 424.1893 | 3 | -9.2 | 425.1916 | 1 | 10.20 | 3945 | 1.22e3 | 1 | 1 | 305 | 307 |  |  | DB Search |
| S.GRCFG.A | Y | 17.99 | 538.2322 | 5 | 1.87 | 539.2391 | 1 | 4.95 | 1975 | 0 | 0 | 0 | 304 | 308 |  |  | DB Search |
| D.EDIH.T | N | 17.26 | 512.2231 | 4 | -4.88 | 513.2266 | 1 | 2.77 | 1153 | 2.08e2 | 1 | 1 | 88 | 91 |  |  | DB Search |
| R.TATGGTSKTAV.L | Y | 16.32 | 992.5138 | 11 | 0.77 | 497.2633 | 2 | 8.66 | 3335 | 0 | 0 | 0 | 442 | 452 |  |  | DB Search |
| I.KSIP.S | N | 15.42 | 443.2744 | 4 | -3.64 | 444.2789 | 1 | 17.16 | 6753 | 0 | 0 | 0 | 318 | 321 |  |  | DB Search |
| total 9 peptides |
| --- |

Best Unique PSM (Scan POS\_R15-1.wiff:1975, m/z=539.2391, z=1, RT=4.95, ppm=-0.61):


C4QZJ2|C4QZJ2\_KOMPG

back to list

  

| Protein Coverage
| Supporting Peptides
| Best Unique PSM
|

Protein Coverage:

Supporting Peptides:

| Peptide | Uniq | -10lgP | Mass | Length | ppm | m/z | z | RT | Scan | Area POS\_R15-1 | #Feature | #Feature POS\_R15-1 | Start | End | PTM | AScore | Found By |
| --- | --- | --- | --- | --- | --- | --- | --- | --- | --- | --- | --- | --- | --- | --- | --- | --- | --- |
| S.ITGL.M | N | 27.55 | 402.2478 | 4 | -1.76 | 403.2534 | 1 | 15.04 | 5964 | 1.56e4 | 2 | 2 | 801 | 804 |  |  | DB Search |
| P.SPLL.R | N | 26.66 | 428.2635 | 4 | -1.63 | 429.269 | 1 | 21.35 | 7905 | 0 | 0 | 0 | 160 | 163 |  |  | DB Search |
| G.TLVL.P | N | 26.01 | 444.2948 | 4 | -4 | 445.2992 | 1 | 28.31 | 9909 | 1.89e3 | 1 | 1 | 1013 | 1016 |  |  | DB Search |
| S.HPAA.A | N | 22.78 | 394.1964 | 4 | 8.24 | 395.206 | 1 | 15.22 | 6038 | 1e3 | 1 | 1 | 237 | 240 |  |  | DB Search |
| A.RCL.V | N | 20.6 | 390.2049 | 3 | -4.41 | 391.2095 | 1 | 8.08 | 3003 | 0 | 0 | 0 | 409 | 411 |  |  | DB Search |
| V.Q(-17.03)SF.D | N | 18.71 | 363.143 | 3 | 2.8 | 364.1504 | 1 | 14.74 | 5815 | 0 | 0 | 0 | 57 | 59 | Pyro-glu from Q | Q1:Pyro-glu from Q:1000 | DB Search |
| E.RDR.R | N | 18.68 | 445.2397 | 3 | 8.14 | 446.2495 | 1 | 10.60 | 4131 | 1.44e2 | 1 | 1 | 1131 | 1133 |  |  | DB Search |
| S.DVYVNA.Q | Y | 16.99 | 679.3177 | 6 | -8.68 | 680.3174 | 1 | 14.61 | 5772 | 3.06e3 | 1 | 1 | 1169 | 1174 |  |  | DB Search |
| L.AHVI.T | N | 16.82 | 438.259 | 4 | -6.35 | 439.2625 | 1 | 8.70 | 3374 | 6.21e2 | 1 | 1 | 633 | 636 |  |  | DB Search |
| D.FVTP.P | N | 16.21 | 462.2478 | 4 | -6.29 | 463.251 | 1 | 16.04 | 6344 | 0 | 0 | 0 | 696 | 699 |  |  | DB Search |
| P.LAHV.I | N | 15.98 | 438.259 | 4 | -6.35 | 439.2625 | 1 | 8.70 | 3417 | 6.21e2 | 1 | 1 | 632 | 635 |  |  | DB Search |
| R.IKTP.K | N | 15.64 | 457.29 | 4 | -5.17 | 458.2938 | 1 | 15.66 | 6234 | 0 | 0 | 0 | 893 | 896 |  |  | DB Search |
| V.Q(-17.03)PF.Q | N | 15.22 | 373.1638 | 3 | -0.75 | 374.1698 | 1 | 21.41 | 7929 | 8.45e4 | 1 | 1 | 497 | 499 | Pyro-glu from Q | Q1:Pyro-glu from Q:1000 | DB Search |
| P.PVE.Y | N | 15.19 | 343.1743 | 3 | -3.43 | 344.1796 | 1 | 12.76 | 5037 | 6.34e2 | 1 | 1 | 622 | 624 |  |  | DB Search |
| E.NNSV.S | N | 15.01 | 432.1969 | 4 | 8.48 | 433.2067 | 1 | 7.60 | 2865 | 3.02e3 | 1 | 1 | 215 | 218 |  |  | DB Search |
| total 15 peptides |
| --- |

Best Unique PSM (Scan POS\_R15-1.wiff:5772, m/z=680.3184, z=2, RT=14.65, ppm=-11.16):


C4QWD1|C4QWD1\_KOMPG

back to list

  

| Protein Coverage
| Supporting Peptides
| Best Unique PSM
|

Protein Coverage:

Supporting Peptides:

| Peptide | Uniq | -10lgP | Mass | Length | ppm | m/z | z | RT | Scan | Area POS\_R15-1 | #Feature | #Feature POS\_R15-1 | Start | End | PTM | AScore | Found By |
| --- | --- | --- | --- | --- | --- | --- | --- | --- | --- | --- | --- | --- | --- | --- | --- | --- | --- |
| S.PSIL.E | N | 29.95 | 428.2635 | 4 | -0.56 | 429.2694 | 1 | 21.28 | 7884 | 0 | 0 | 0 | 2835 | 2838 |  |  | DB Search |
| A.LTGL.K | N | 27.55 | 402.2478 | 4 | -1.76 | 403.2534 | 1 | 15.04 | 5964 | 1.56e4 | 2 | 2 | 3843 | 3846 |  |  | DB Search |
| T.RGFI.T | N | 25.24 | 491.2856 | 4 | -0.24 | 492.2915 | 1 | 14.92 | 5891 | 9.54e2 | 1 | 1 | 485 | 488 |  |  | DB Search |
| A.ELLR.K | N | 25.05 | 529.3224 | 4 | -4.56 | 530.3259 | 1 | 17.34 | 6855 | 6.68e3 | 1 | 1 | 1378 | 1381 |  |  | DB Search |
| S.VGGI.L | N | 24.87 | 344.2059 | 4 | -5.22 | 345.2106 | 1 | 10.74 | 4195 | 1.51e3 | 1 | 1 | 261 | 264 |  |  | DB Search |
| G.ILIP.Y | N | 24.07 | 454.3155 | 4 | -2.4 | 455.3206 | 1 | 34.52 | 11565 | 2.64e3 | 1 | 1 | 264 | 267 |  |  | DB Search |
| D.EW.V | N | 22.33 | 333.1325 | 2 | -3.27 | 334.1378 | 1 | 12.76 | 5036 | 2.08e4 | 1 | 1 | 916 | 917 |  |  | DB Search |
| I.VLLP.Q | N | 22.17 | 440.2998 | 4 | -5.96 | 441.3034 | 1 | 30.99 | 10567 | 1.25e3 | 1 | 1 | 443 | 446 |  |  | DB Search |
| V.IDGI.N | N | 22.09 | 416.2271 | 4 | -1.79 | 417.2326 | 1 | 13.94 | 5544 | 2.42e3 | 1 | 1 | 4882 | 4885 |  |  | DB Search |
| S.KDII.K | N | 22.06 | 487.3006 | 4 | -0.76 | 488.3063 | 1 | 10.37 | 4062 | 0 | 0 | 0 | 330 | 333 |  |  | DB Search |
| S.SLAL.T | N | 21.98 | 402.2478 | 4 | -2.21 | 403.2532 | 1 | 20.49 | 7711 | 0 | 0 | 0 | 2613 | 2616 |  |  | DB Search |
| T.EIF.S | N | 21.7 | 407.2056 | 3 | -8.99 | 408.2082 | 1 | 29.23 | 10157 | 9.42e2 | 1 | 1 | 480 | 482 |  |  | DB Search |
| A.SSLF.S | N | 21.63 | 452.2271 | 4 | -3.7 | 453.2316 | 1 | 22.49 | 8262 | 1.33e3 | 1 | 1 | 2542 | 2545 |  |  | DB Search |
| F.QGLY.Y | N | 21.52 | 479.238 | 4 | 7.78 | 480.2478 | 1 | 12.12 | 4746 | 5.21e3 | 1 | 1 | 3168 | 3171 |  |  | DB Search |
| E.NTS.S | N | 21.44 | 320.1332 | 3 | 8.58 | 321.1424 | 1 | 6.16 | 2421 | 0 | 0 | 0 | 747 | 749 |  |  | DB Search |
| Y.TSG.T | N | 21.44 | 263.1117 | 3 | 2.31 | 264.119 | 1 | 3.71 | 1537 | 5.72e3 | 1 | 1 | 351 | 353 |  |  | DB Search |
| T.ELW.V | N | 21.42 | 446.2165 | 3 | -5.13 | 447.2204 | 1 | 31.30 | 10711 | 1.59e3 | 1 | 1 | 887 | 889 |  |  | DB Search |
[truncated: 155,935 more chars]
