# Supplementary material for: Mining umami peptides in lager and multidimensional sensory evaluation of the beer body integrating computational biology with modern sensomics
Source: Food Chem X. 2025 Oct 6;31:103132. doi: 10.1016/j.fochx.2025.103132 (PMC12538414; doi:10.1016/j.fochx.2025.103132)
Supplement: Supplementary material 6 — Detailed results of peptide retrieval in beer [file mmc6.zip › db.peptides.html]

peptide list


Peptide List

  

| Peptide | -10lgP | Mass | Length | ppm | m/z | RT | Scan | Area POS\_R15-1 | #Feature | #Feature POS\_R15-1 | Accession | PTM | AScore | Found By |
| --- | --- | --- | --- | --- | --- | --- | --- | --- | --- | --- | --- | --- | --- | --- |
| VPTVDVSVVD | 60.49 | 1028.5389 | 10 | -6.03 | 1029.5375 | 30.36 | 10434 | 0 | 0 | 0 | C4R0P1|C4R0P1\_KOMPG |  |  | DB Search |
| VPTVDVS | 37.21 | 715.3752 | 7 | -2.34 | 716.379 | 15.28 | 6054 | 2.47e3 | 2 | 2 | C4R0P1|C4R0P1\_KOMPG |  |  | DB Search |
| VDVSVVD | 36.35 | 731.3701 | 7 | -3.95 | 732.3727 | 17.66 | 6952 | 2.47e3 | 1 | 1 | C4R0P1|C4R0P1\_KOMPG |  |  | DB Search |
| VKLDVLQTL | 35.97 | 1027.6277 | 9 | -9.38 | 1028.6228 | 42.64 | 13414 | 1.91e2 | 1 | 1 | C4R6W9|C4R6W9\_KOMPG |  |  | DB Search |
| IPSNP | 34.2 | 526.2751 | 5 | -5.22 | 527.2783 | 8.79 | 3411 | 0 | 0 | 0 | C4R905|C4R905\_KOMPG |  |  | DB Search |
| LPSNP | 34.2 | 526.2751 | 5 | -5.22 | 527.2783 | 8.79 | 3411 | 0 | 0 | 0 | C4R901|C4R901\_KOMPG:C4R155|C4R155\_KOMPG:C4QZS0|C4QZS0\_KOMPG:C4QV87|C4QV87\_KOMPG:C4R5R6|C4R5R6\_KOMPG |  |  | DB Search |
| TILMLGGL | 32.75 | 816.4779 | 8 | -9.7 | 817.4752 | 46.74 | 14179 | 9.87e2 | 1 | 1 | C4QZ37|C4QZ37\_KOMPG |  |  | DB Search |
| AAGQY | 32.48 | 508.2281 | 5 | 0.3 | 509.2343 | 4.42 | 1758 | 1.42e4 | 1 | 1 | C4QYQ0|C4QYQ0\_KOMPG |  |  | DB Search |
| Q(+0.98)PQQ(+0.98)PQQP | 30.12 | 951.4297 | 8 | 7.51 | 952.4418 | 22.99 | 8410 | 0 | 0 | 0 | C4QXV6|C4QXV6\_KOMPG | Deamidation (NQ), Deamidation (NQ) | Q1:Deamidation (NQ):11.22 Q4:Deamidation (NQ):14.02 | DB Search |
| PSII | 29.95 | 428.2635 | 4 | -0.56 | 429.2694 | 21.28 | 7884 | 0 | 0 | 0 | C4R5V3|C4R5V3\_KOMPG:C4QVI6|C4QVI6\_KOMPG:C4R232|C4R232\_KOMPG:C4R1G8|C4R1G8\_KOMPG:C4QZ37|C4QZ37\_KOMPG:C4R6N6|C4R6N6\_KOMPG:C4R148|C4R148\_KOMPG:C4R6B6|C4R6B6\_KOMPG:C4QXK7|C4QXK7\_KOMPG:C4QVY9|C4QVY9\_KOMPG:C4R8U8|C4R8U8\_KOMPG:C4R1A8|C4R1A8\_KOMPG:C4QWL6|C4QWL6\_KOMPG:C4R134|C4R134\_KOMPG:C4R7R4|C4R7R4\_KOMPG:C4R051|C4R051\_KOMPG:C4QW83|C4QW83\_KOMPG:C4R7Y5|C4R7Y5\_KOMPG:C4QZX7|C4QZX7\_KOMPG:C4QVK6|C4QVK6\_KOMPG:C4R950|C4R950\_KOMPG:C4R768|C4R768\_KOMPG:C4R3R7|C4R3R7\_KOMPG:C4R1X0|C4R1X0\_KOMPG:C4R1W2|C4R1W2\_KOMPG:C4QVM0|C4QVM0\_KOMPG:C4QVJ6|C4QVJ6\_KOMPG:C4R548|C4R548\_KOMPG:C4R9A3|C4R9A3\_KOMPG:C4R735|C4R735\_KOMPG:C4QVR8|C4QVR8\_KOMPG:C4R4H0|C4R4H0\_KOMPG:C4R7M1|C4R7M1\_KOMPG:C4R314|C4R314\_KOMPG:C4R1I5|C4R1I5\_KOMPG:C4R0J8|C4R0J8\_KOMPG:C4R817|C4R817\_KOMPG:C4QYF1|C4QYF1\_KOMPG:C4R409|C4R409\_KOMPG:C4QUZ0|C4QUZ0\_KOMPG:C4R9A6|C4R9A6\_KOMPG |  |  | DB Search |
| PSIL | 29.95 | 428.2635 | 4 | -0.56 | 429.2694 | 21.28 | 7884 | 0 | 0 | 0 | C4R0N9|C4R0N9\_KOMPG:C4QWS2|C4QWS2\_KOMPG:C4R6X0|C4R6X0\_KOMPG:C4QX06|C4QX06\_KOMPG:C4QWB0|C4QWB0\_KOMPG:C4R8T2|C4R8T2\_KOMPG:C4QWW6|C4QWW6\_KOMPG:C4R399|C4R399\_KOMPG:C4R6M3|C4R6M3\_KOMPG:C4R4A3|C4R4A3\_KOMPG:C4QYY2|C4QYY2\_KOMPG:C4R705|C4R705\_KOMPG:C4QYZ1|C4QYZ1\_KOMPG:C4R4I4|C4R4I4\_KOMPG:C4R5S2|C4R5S2\_KOMPG:C4QYL7|AIM9\_KOMPG:C4QV53|C4QV53\_KOMPG:C4R2Z8|C4R2Z8\_KOMPG:C4QY37|C4QY37\_KOMPG:C4QVX7|C4QVX7\_KOMPG:C4QW45|C4QW45\_KOMPG:C4R2S4|C4R2S4\_KOMPG:C4R6A8|C4R6A8\_KOMPG:C4QZP8|C4QZP8\_KOMPG:C4R908|C4R908\_KOMPG:C4QZ59|C4QZ59\_KOMPG:C4R1C4|C4R1C4\_KOMPG:C4R287|C4R287\_KOMPG:C4R6J6|C4R6J6\_KOMPG:C4R6X5|C4R6X5\_KOMPG:C4QYQ2|C4QYQ2\_KOMPG:C4QVB2|C4QVB2\_KOMPG:C4R1M0|C4R1M0\_KOMPG:C4R4T6|C4R4T6\_KOMPG:C4R8I0|C4R8I0\_KOMPG:C4QXW3|C4QXW3\_KOMPG:C4R1N8|C4R1N8\_KOMPG:C4R0P2|C4R0P2\_KOMPG:C4R2B4|C4R2B4\_KOMPG:C4QZD0|C4QZD0\_KOMPG:C4QWA5|C4QWA5\_KOMPG:C4QYE4|C4QYE4\_KOMPG:C4R5C9|C4R5C9\_KOMPG:C4QWD1|C4QWD1\_KOMPG:C4R832|C4R832\_KOMPG:C4R3F9|C4R3F9\_KOMPG:C4R3E3|C4R3E3\_KOMPG:C4R7M0|C4R7M0\_KOMPG:C4QXZ9|C4QXZ9\_KOMPG:C4R5Y5|C4R5Y5\_KOMPG:C4QVR6|C4QVR6\_KOMPG:C4R207|C4R207\_KOMPG:C4QVM6|C4QVM6\_KOMPG:C4R7R5|C4R7R5\_KOMPG:C4R239|C4R239\_KOMPG:C4R424|C4R424\_KOMPG |  |  | DB Search |
| PSLI | 29.95 | 428.2635 | 4 | -0.56 | 429.2694 | 21.28 | 7884 | 0 | 0 | 0 | C4R086|C4R086\_KOMPG:C4QXE5|C4QXE5\_KOMPG:C4QWG8|C4QWG8\_KOMPG:C4QZI3|C4QZI3\_KOMPG:C4R8D0|C4R8D0\_KOMPG:C4R5D8|COQ4\_KOMPG:C4R003|C4R003\_KOMPG:C4R0F2|C4R0F2\_KOMPG:C4R3T1|C4R3T1\_KOMPG:C4R398|C4R398\_KOMPG:C4QW51|C4QW51\_KOMPG:C4QZK4|C4QZK4\_KOMPG:C4R4D5|C4R4D5\_KOMPG:C4QYC6|C4QYC6\_KOMPG:C4R109|C4R109\_KOMPG:C4R3C5|C4R3C5\_KOMPG:C4R6L8|C4R6L8\_KOMPG:C4R498|C4R498\_KOMPG:C4R244|C4R244\_KOMPG:C4R3L8|C4R3L8\_KOMPG:C4R0G6|C4R0G6\_KOMPG:C4R4J3|C4R4J3\_KOMPG:C4R724|C4R724\_KOMPG:C4QW21|C4QW21\_KOMPG:C4R915|C4R915\_KOMPG:C4R173|C4R173\_KOMPG:C4R5W2|C4R5W2\_KOMPG:C4R7E3|C4R7E3\_KOMPG:C4R4R0|C4R4R0\_KOMPG:C4R245|C4R245\_KOMPG:C4R4N9|C4R4N9\_KOMPG:C4R0X6|C4R0X6\_KOMPG:C4R678|C4R678\_KOMPG:C4R984|C4R984\_KOMPG:C4QXA5|PFKA2\_KOMPG:C4R1Y0|C4R1Y0\_KOMPG:C4R9A7|C4R9A7\_KOMPG:C4QVV8|C4QVV8\_KOMPG:C4QX20|C4QX20\_KOMPG:C4QXE1|C4QXE1\_KOMPG:C4R1H7|C4R1H7\_KOMPG:C4R6S4|C4R6S4\_KOMPG:C4R254|C4R254\_KOMPG:C4R7J2|C4R7J2\_KOMPG:C4QV26|C4QV26\_KOMPG:C4QZ42|C4QZ42\_KOMPG:C4R1I1|C4R1I1\_KOMPG:C4R2X0|C4R2X0\_KOMPG:C4R5X1|C4R5X1\_KOMPG |  |  | DB Search |
| PSLL | 29.95 | 428.2635 | 4 | -0.56 | 429.2694 | 21.28 | 7884 | 0 | 0 | 0 | C4R3D1|C4R3D1\_KOMPG:C4QWF8|C4QWF8\_KOMPG:C4R2V8|C4R2V8\_KOMPG:C4R4C2|C4R4C2\_KOMPG:C4R6C2|PEX1\_KOMPG:C4R8Q4|C4R8Q4\_KOMPG:C4R4L0|C4R4L0\_KOMPG:C4R4G8|C4R4G8\_KOMPG:C4R6J9|C4R6J9\_KOMPG:C4R998|C4R998\_KOMPG:C4QYV4|C4QYV4\_KOMPG:C4R4L7|C4R4L7\_KOMPG:C4R300|C4R300\_KOMPG:C4QX76|C4QX76\_KOMPG:C4R4C6|C4R4C6\_KOMPG:C4QZ89|C4QZ89\_KOMPG:C4QWK2|C4QWK2\_KOMPG:C4QYS8|C4QYS8\_KOMPG:C4R555|C4R555\_KOMPG:C4QWZ4|C4QWZ4\_KOMPG:C4R4M8|C4R4M8\_KOMPG:C4QVX0|C4QVX0\_KOMPG:C4QVH0|C4QVH0\_KOMPG:C4R6Z7|C4R6Z7\_KOMPG:C4QZ97|C4QZ97\_KOMPG:C4R7E8|C4R7E8\_KOMPG:C4QW90|C4QW90\_KOMPG:C4R3C1|C4R3C1\_KOMPG:C4R7Q5|EXO5\_KOMPG:C4QYY0|C4QYY0\_KOMPG:C4R6D9|C4R6D9\_KOMPG:C4R0G3|C4R0G3\_KOMPG:C4QZ36|C4QZ36\_KOMPG:C4R966|C4R966\_KOMPG:C4QYQ3|C4QYQ3\_KOMPG:C4R474|C4R474\_KOMPG:C4R6F6|C4R6F6\_KOMPG:C4QX51|C4QX51\_KOMPG:C4R640|C4R640\_KOMPG:C4R6H4|C4R6H4\_KOMPG:C4R1W9|C4R1W9\_KOMPG:C4R1V2|C4R1V2\_KOMPG:C4QVP0|C4QVP0\_KOMPG:C4QWV1|C4QWV1\_KOMPG:C4QYS1|C4QYS1\_KOMPG:C4R341|C4R341\_KOMPG:C4R6I7|C4R6I7\_KOMPG:C4QZG9|C4QZG9\_KOMPG:C4R2Q9|C4R2Q9\_KOMPG:C4R6Q8|C4R6Q8\_KOMPG:C4QYU4|C4QYU4\_KOMPG:C4R4H4|C4R4H4\_KOMPG:C4R4K8|C4R4K8\_KOMPG:C4QWB1|C4QWB1\_KOMPG:C4R926|C4R926\_KOMPG:C4R3L0|C4R3L0\_KOMPG:C4QVX3|C4QVX3\_KOMPG:C4QZP7|C4QZP7\_KOMPG:C4R8Q0|C4R8Q0\_KOMPG:C4QVD4|C4QVD4\_KOMPG:C4QV66|C4QV66\_KOMPG:C4R8E0|C4R8E0\_KOMPG:C4R5W4|C4R5W4\_KOMPG:C4R4N0|C4R4N0\_KOMPG:C4R136|C4R136\_KOMPG:C4R4K9|C4R4K9\_KOMPG:C4QV27|C4QV27\_KOMPG:C4R2K1|C4R2K1\_KOMPG:C4QVR2|C4QVR2\_KOMPG:C4QXY3|C4QXY3\_KOMPG:C4R5F7|C4R5F7\_KOMPG:C4QW09|C4QW09\_KOMPG:C4QWQ8|C4QWQ8\_KOMPG:C4R465|C4R465\_KOMPG:C4R4K7|C4R4K7\_KOMPG:C4R821|C4R821\_KOMPG:C4R3X7|C4R3X7\_KOMPG:C4R680|C4R680\_KOMPG:C4QXX0|C4QXX0\_KOMPG:C4QYK2|C4QYK2\_KOMPG:C4R7Q2|C4R7Q2\_KOMPG:C4R1C2|C4R1C2\_KOMPG:C4R8Z2|C4R8Z2\_KOMPG:C4R326|C4R326\_KOMPG:C4R6U0|C4R6U0\_KOMPG:C4R4H7|C4R4H7\_KOMPG:C4R7Z4|C4R7Z4\_KOMPG:C4QWS9|C4QWS9\_KOMPG:C4R3T3|C4R3T3\_KOMPG:C4QYC0|C4QYC0\_KOMPG:C4QYR5|C4QYR5\_KOMPG:C4QWG7|C4QWG7\_KOMPG:C4R8V3|C4R8V3\_KOMPG:C4QV23|C4QV23\_KOMPG |  |  | DB Search |
| ITLGM | 29.47 | 533.2883 | 5 | -6.08 | 534.291 | 28.10 | 9841 | 7.28e3 | 1 | 1 | C4R368|C4R368\_KOMPG:C4R2D0|C4R2D0\_KOMPG |  |  | DB Search |
| LTIGM | 29.47 | 533.2883 | 5 | -6.08 | 534.291 | 28.10 | 9841 | 7.28e3 | 1 | 1 | C4R2P8|C4R2P8\_KOMPG:C4R8Q9|C4R8Q9\_KOMPG |  |  | DB Search |
| LTLGM | 29.47 | 533.2883 | 5 | -6.08 | 534.291 | 28.10 | 9841 | 7.28e3 | 1 | 1 | C4R2T7|C4R2T7\_KOMPG:C4R8B3|C4R8B3\_KOMPG |  |  | DB Search |
| ALELSG | 28.74 | 588.3119 | 6 | -2.07 | 589.3165 | 14.07 | 5590 | 1.66e4 | 1 | 1 | C4R1F5|C4R1F5\_KOMPG |  |  | DB Search |
| GGGGI | 28.62 | 359.1805 | 5 | -3.01 | 360.1858 | 6.13 | 2409 | 0 | 0 | 0 | C4QXF2|C4QXF2\_KOMPG:C4R723|C4R723\_KOMPG:C4QWK0|C4QWK0\_KOMPG:C4QWH8|C4QWH8\_KOMPG:C4R524|C4R524\_KOMPG:C4R6N7|C4R6N7\_KOMPG |  |  | DB Search |
| GGGGL | 28.62 | 359.1805 | 5 | -3.01 | 360.1858 | 6.13 | 2409 | 0 | 0 | 0 | C4QZN5|C4QZN5\_KOMPG:C4R1X7|C4R1X7\_KOMPG:C4R922|C4R922\_KOMPG:C4R4Y0|C4R4Y0\_KOMPG:C4QXV5|C4QXV5\_KOMPG |  |  | DB Search |
| Q(-17.03)PQQPQ | 28.36 | 707.3239 | 6 | -2.97 | 708.3273 | 6.57 | 2541 | 0 | 0 | 0 | C4QY77|C4QY77\_KOMPG:C4QXV6|C4QXV6\_KOMPG:C4R150|C4R150\_KOMPG:C4R0K1|C4R0K1\_KOMPG | Pyro-glu from Q | Q1:Pyro-glu from Q:1000 | DB Search |
| NGI | 28.25 | 302.159 | 3 | -3.76 | 303.1644 | 6.06 | 2374 | 3.14e3 | 1 | 1 | C4R8W0|C4R8W0\_KOMPG:C4R6D2|C4R6D2\_KOMPG:C4QVN9|C4QVN9\_KOMPG:C4R0D6|C4R0D6\_KOMPG:C4QW03|C4QW03\_KOMPG:C4QZ72|C4QZ72\_KOMPG:C4R3D0|C4R3D0\_KOMPG:C4QXX5|C4QXX5\_KOMPG:C4QV84|C4QV84\_KOMPG:C4QZZ8|C4QZZ8\_KOMPG:C4R4M1|C4R4M1\_KOMPG:C4R036|C4R036\_KOMPG:C4R902|C4R902\_KOMPG:C4R784|C4R784\_KOMPG:C4R1P8|C4R1P8\_KOMPG:C4QX76|C4QX76\_KOMPG:C4QWE3|C4QWE3\_KOMPG:C4R6B2|C4R6B2\_KOMPG:C4QV07|C4QV07\_KOMPG:C4QVX4|C4QVX4\_KOMPG:C4R463|C4R463\_KOMPG:C4R7Y5|C4R7Y5\_KOMPG:C4R912|C4R912\_KOMPG:C4QWI9|C4QWI9\_KOMPG:C4R2P9|C4R2P9\_KOMPG:C4R6G3|C4R6G3\_KOMPG:C4QY48|C4QY48\_KOMPG:C4R4A5|C4R4A5\_KOMPG:C4R5N8|C4R5N8\_KOMPG:C4R2E1|C4R2E1\_KOMPG:C4QV46|C4QV46\_KOMPG:C4R1G4|C4R1G4\_KOMPG:C4R826|PEX4\_KOMPG:P04842|ALOX1\_KOMPG:C4QXU5|C4QXU5\_KOMPG:C4QYV9|C4QYV9\_KOMPG:C4QY99|C4QY99\_KOMPG:C4QVK0|C4QVK0\_KOMPG:C4QWH2|C4QWH2\_KOMPG:C4R8I1|C4R8I1\_KOMPG:C4R0J8|C4R0J8\_KOMPG:C4R702|ALOX2\_KOMPG:C4QZU4|C4QZU4\_KOMPG:C4QW00|C4QW00\_KOMPG:C4QVT0|C4QVT0\_KOMPG:C4R893|C4R893\_KOMPG:C4QZY8|C4QZY8\_KOMPG:C4R5I2|C4R5I2\_KOMPG:C4QW33|C4QW33\_KOMPG:C4QXZ3|C4QXZ3\_KOMPG:C4QVV6|C4QVV6\_KOMPG:C4QZJ4|C4QZJ4\_KOMPG:C4QYZ3|C4QYZ3\_KOMPG:C4R5U2|C4R5U2\_KOMPG:C4R2L6|C4R2L6\_KOMPG:C4R0X4|C4R0X4\_KOMPG:C4R5S1|C4R5S1\_KOMPG:C4R5G8|C4R5G8\_KOMPG:C4QYH5|C4QYH5\_KOMPG:C4QY22|C4QY22\_KOMPG:C4R8D8|C4R8D8\_KOMPG:C4R709|C4R709\_KOMPG:C4R438|C4R438\_KOMPG:C4R6H9|C4R6H9\_KOMPG:C4QZY7|C4QZY7\_KOMPG:C4R5D6|C4R5D6\_KOMPG:C4R6W3|C4R6W3\_KOMPG:C4R951|C4R951\_KOMPG:C4QYA5|C4QYA5\_KOMPG:C4R4A2|C4R4A2\_KOMPG:C4QW27|C4QW27\_KOMPG:C4R4K5|C4R4K5\_KOMPG:C4R0E3|C4R0E3\_KOMPG:C4QZV2|C4QZV2\_KOMPG:C4R7R8|C4R7R8\_KOMPG:C4R4T5|C4R4T5\_KOMPG:C4R686|C4R686\_KOMPG:C4R0J5|C4R0J5\_KOMPG:C4QZB4|C4QZB4\_KOMPG:C4R8X9|C4R8X9\_KOMPG:C4R7P3|C4R7P3\_KOMPG:C4QXH1|C4QXH1\_KOMPG:C4QWV4|C4QWV4\_KOMPG:C4R6M2|C4R6M2\_KOMPG:C4QY10|C4QY10\_KOMPG:C4R813|C4R813\_KOMPG:C4R3H2|C4R3H2\_KOMPG:C4QVK4|C4QVK4\_KOMPG:C4R462|C4R462\_KOMPG:C4QXE9|CHO2\_KOMPG:C4R4B2|C4R4B2\_KOMPG:C4R387|C4R387\_KOMPG:C4R649|C4R649\_KOMPG |  |  | DB Search |
| NGL | 28.25 | 302.159 | 3 | -3.76 | 303.1644 | 6.06 | 2374 | 3.14e3 | 1 | 1 | C4R8N1|C4R8N1\_KOMPG:C4R1S0|C4R1S0\_KOMPG:C4R3U6|C4R3U6\_KOMPG:C4QYR2|C4QYR2\_KOMPG:C4QV06|C4QV06\_KOMPG:C4R4S7|C4R4S7\_KOMPG:C4R3G3|C4R3G3\_KOMPG:C4QVL2|C4QVL2\_KOMPG:C4QYY7|C4QYY7\_KOMPG:C4R2A4|C4R2A4\_KOMPG:C4R5J4|C4R5J4\_KOMPG:C4R4X8|C4R4X8\_KOMPG:C4R2C5|C4R2C5\_KOMPG:C4R2S5|C4R2S5\_KOMPG:C4R6X6|C4R6X6\_KOMPG:C4R4R8|ARO1\_KOMPG:C4R6H0|C4R6H0\_KOMPG:C4R8D3|C4R8D3\_KOMPG:C4R571|C4R571\_KOMPG:C4R404|C4R404\_KOMPG:C4R8Q7|C4R8Q7\_KOMPG:C4R7S0|C4R7S0\_KOMPG:C4QVP0|C4QVP0\_KOMPG:C4QYE3|C4QYE3\_KOMPG:C4R169|C4R169\_KOMPG:C4R0G4|C4R0G4\_KOMPG:C4R8Y4|C4R8Y4\_KOMPG:C4R7X8|BMT2\_KOMPG:C4QVX9|C4QVX9\_KOMPG:C4R2T7|C4R2T7\_KOMPG:C4QYU4|C4QYU4\_KOMPG:C4R310|C4R310\_KOMPG:C4QWL2|C4QWL2\_KOMPG:C4R7V7|C4R7V7\_KOMPG:C4R8G6|C4R8G6\_KOMPG:C4QV66|C4QV66\_KOMPG:C4QVI9|C4QVI9\_KOMPG:C4QWF6|C4QWF6\_KOMPG:C4R447|C4R447\_KOMPG:C4R517|C4R517\_KOMPG:C4R8Y6|C4R8Y6\_KOMPG:C4QXA9|C4QXA9\_KOMPG:C4QYP7|C4QYP7\_KOMPG:C4R4A6|C4R4A6\_KOMPG:C4R129|C4R129\_KOMPG:C4R4V7|C4R4V7\_KOMPG:C4R446|C4R446\_KOMPG:C4R3W1|C4R3W1\_KOMPG:C4QVT9|C4QVT9\_KOMPG:C4QWA5|C4QWA5\_KOMPG:C4R2A9|C4R2A9\_KOMPG:C4R6C6|C4R6C6\_KOMPG:C4R095|C4R095\_KOMPG:C4R0L5|C4R0L5\_KOMPG:C4R5V0|C4R5V0\_KOMPG:C4R3D1|C4R3D1\_KOMPG:C4QVY3|C4QVY3\_KOMPG:C4R0S7|C4R0S7\_KOMPG:C4R7D0|C4R7D0\_KOMPG:C4R8N7|C4R8N7\_KOMPG:C4R1R3|C4R1R3\_KOMPG:C4R1P4|C4R1P4\_KOMPG:C4R0B0|C4R0B0\_KOMPG:C4QV67|C4QV67\_KOMPG:C4R7C2|C4R7C2\_KOMPG:C4R568|C4R568\_KOMPG:C4R3A3|C4R3A3\_KOMPG:C4QWA3|C4QWA3\_KOMPG:C4QWS8|PEX36\_KOMPG:C4R6L7|C4R6L7\_KOMPG:C4R534|C4R534\_KOMPG:C4R0H7|C4R0H7\_KOMPG:C4R5J0|C4R5J0\_KOMPG:C4R2J3|C4R2J3\_KOMPG:C4R069|C4R069\_KOMPG:C4QYT3|C4QYT3\_KOMPG:C4R3H7|C4R3H7\_KOMPG:C4R8L7|C4R8L7\_KOMPG:C4QY28|C4QY28\_KOMPG:C4R0W3|C4R0W3\_KOMPG:C4R6D7|C4R6D7\_KOMPG:C4R5I3|C4R5I3\_KOMPG:C4QVH4|C4QVH4\_KOMPG:C4R1N3|C4R1N3\_KOMPG:C4R760|C4R760\_KOMPG:C4R923|C4R923\_KOMPG:C4R5C1|C4R5C1\_KOMPG:C4R1G6|C4R1G6\_KOMPG:C4R8M6|C4R8M6\_KOMPG:C4QXG2|C4QXG2\_KOMPG:C4R921|C4R921\_KOMPG:C4QWU3|C4QWU3\_KOMPG:C4R3J3|C4R3J3\_KOMPG:C4R5P8|C4R5P8\_KOMPG:C4R546|C4R546\_KOMPG:C4R8F4|C4R8F4\_KOMPG:C4R803|C4R803\_KOMPG:C4QYQ2|C4QYQ2\_KOMPG:C4R6Q6|C4R6Q6\_KOMPG:C4R8C1|C4R8C1\_KOMPG:C4QVZ3|C4QVZ3\_KOMPG:C4QXM9|C4QXM9\_KOMPG:C4R516|C4R516\_KOMPG:C4R1U7|C4R1U7\_KOMPG:C4R370|C4R370\_KOMPG:C4R764|C4R764\_KOMPG:C4QVP3|C4QVP3\_KOMPG:C4R126|C4R126\_KOMPG:C4R2P7|C4R2P7\_KOMPG:C4R4V1|C4R4V1\_KOMPG:C4QZC0|C4QZC0\_KOMPG:C4R2Q0|C4R2Q0\_KOMPG:C4R2G5|C4R2G5\_KOMPG:C4R5V4|C4R5V4\_KOMPG:C4R2K9|C4R2K9\_KOMPG:C4QXS6|C4QXS6\_KOMPG:C4R3Q9|C4R3Q9\_KOMPG:C4R855|C4R855\_KOMPG:C4QZS0|C4QZS0\_KOMPG:C4QXD5|C4QXD5\_KOMPG:C4R405|C4R405\_KOMPG |  |  | DB Search |
| NII | 28.22 | 358.2216 | 3 | -4.68 | 359.2263 | 19.18 | 7326 | 4.19e3 | 1 | 1 | C4R921|C4R921\_KOMPG:C4QXT4|C4QXT4\_KOMPG:C4R7J7|C4R7J7\_KOMPG:C4QZZ8|C4QZZ8\_KOMPG:C4R2D3|C4R2D3\_KOMPG:C4R5F7|C4R5F7\_KOMPG:C4R0X2|C4R0X2\_KOMPG:C4QZX7|C4QZX7\_KOMPG:C4QYY0|C4QYY0\_KOMPG:C4R4F0|C4R4F0\_KOMPG:C4QX28|C4QX28\_KOMPG:C4R8J5|C4R8J5\_KOMPG:C4QZC6|C4QZC6\_KOMPG:C4R3L8|C4R3L8\_KOMPG:C4R3Y5|C4R3Y5\_KOMPG:C4QVB5|C4QVB5\_KOMPG:C4R639|C4R639\_KOMPG:C4R047|C4R047\_KOMPG:C4QXB6|C4QXB6\_KOMPG:C4QVS5|C4QVS5\_KOMPG:C4R3A8|C4R3A8\_KOMPG:C4R0P1|C4R0P1\_KOMPG:C4QXN2|MIC60\_KOMPG:C4QX95|C4QX95\_KOMPG:C4QYK4|C4QYK4\_KOMPG |  |  | DB Search |
| NIL | 28.22 | 358.2216 | 3 | -4.68 | 359.2263 | 19.18 | 7326 | 4.19e3 | 1 | 1 | C4QXL0|C4QXL0\_KOMPG:C4QX92|C4QX92\_KOMPG:C4R8H4|C4R8H4\_KOMPG:C4R2Y2|C4R2Y2\_KOMPG:C4R6K9|C4R6K9\_KOMPG:C4R142|C4R142\_KOMPG:C4QZ28|C4QZ28\_KOMPG:C4R4C2|C4R4C2\_KOMPG:C4R0S9|C4R0S9\_KOMPG:C4R7W8|C4R7W8\_KOMPG:C4QVX8|C4QVX8\_KOMPG:C4R2W1|C4R2W1\_KOMPG:C4R6N1|C4R6N1\_KOMPG:C4R2D2|C4R2D2\_KOMPG:C4R1V5|C4R1V5\_KOMPG:C4R5D6|C4R5D6\_KOMPG:C4R0Q0|C4R0Q0\_KOMPG:C4R4X8|C4R4X8\_KOMPG:C4QZ71|C4QZ71\_KOMPG:C4QVT9|C4QVT9\_KOMPG:C4R126|C4R126\_KOMPG:C4QZV2|C4QZV2\_KOMPG:C4QY28|C4QY28\_KOMPG:C4R5J2|C4R5J2\_KOMPG:C4R0Z6|C4R0Z6\_KOMPG:C4R2G2|C4R2G2\_KOMPG:C4R2Q0|C4R2Q0\_KOMPG:C4QYI8|C4QYI8\_KOMPG:C4R749|C4R749\_KOMPG:C4QVP0|C4QVP0\_KOMPG:C4R033|C4R033\_KOMPG:C4QZN7|C4QZN7\_KOMPG:C4QW33|C4QW33\_KOMPG:C4QVK4|C4QVK4\_KOMPG:C4QXD2|C4QXD2\_KOMPG:C4R4M3|C4R4M3\_KOMPG |  |  | DB Search |
| NLI | 28.22 | 358.2216 | 3 | -4.68 | 359.2263 | 19.18 | 7326 | 4.19e3 | 1 | 1 | C4QVZ8|C4QVZ8\_KOMPG:C4R5U0|C4R5U0\_KOMPG:C4R2Q7|C4R2Q7\_KOMPG:C4QVX9|C4QVX9\_KOMPG:C4R6Q8|C4R6Q8\_KOMPG:C4R0X4|C4R0X4\_KOMPG:C4QWJ0|C4QWJ0\_KOMPG:C4QV73|C4QV73\_KOMPG:C4R2V3|C4R2V3\_KOMPG:C4R5P8|C4R5P8\_KOMPG:C4R4F7|C4R4F7\_KOMPG:C4R8C1|C4R8C1\_KOMPG:C4QWA3|C4QWA3\_KOMPG:C4QVM1|C4QVM1\_KOMPG:C4R093|C4R093\_KOMPG:C4QZX0|C4QZX0\_KOMPG:C4R5X0|C4R5X0\_KOMPG:C4R1G9|C4R1G9\_KOMPG:C4R7C8|C4R7C8\_KOMPG:C4R6E1|C4R6E1\_KOMPG:C4R987|C4R987\_KOMPG:C4R8T8|C4R8T8\_KOMPG:C4QZN7|C4QZN7\_KOMPG:C4R6N4|C4R6N4\_KOMPG:C4QWR3|C4QWR3\_KOMPG |  |  | DB Search |
| NLL | 28.22 | 358.2216 | 3 | -4.68 | 359.2263 | 19.18 | 7326 | 4.19e3 | 1 | 1 | C4R8N1|C4R8N1\_KOMPG:C4R554|C4R554\_KOMPG:C4QYH7|C4QYH7\_KOMPG:C4R972|C4R972\_KOMPG:C4R1N5|C4R1N5\_KOMPG:C4QWD7|C4QWD7\_KOMPG:C4R1P8|C4R1P8\_KOMPG:C4R2A4|C4R2A4\_KOMPG:C4QWA3|C4QWA3\_KOMPG:C4QYG5|C4QYG5\_KOMPG:C4QWS8|PEX36\_KOMPG:C4QWD4|C4QWD4\_KOMPG:C4R912|C4R912\_KOMPG:C4R490|C4R490\_KOMPG:C4QXM1|C4QXM1\_KOMPG:C4QXU3|C4QXU3\_KOMPG:C4QVM0|C4QVM0\_KOMPG:C4QXU1|C4QXU1\_KOMPG:C4R4C7|C4R4C7\_KOMPG:C4R717|C4R717\_KOMPG:C4R849|C4R849\_KOMPG:C4R4L4|C4R4L4\_KOMPG:C4R111|C4R111\_KOMPG:C4R965|C4R965\_KOMPG:C4R5C1|C4R5C1\_KOMPG:C4QY36|C4QY36\_KOMPG:C4QX78|C4QX78\_KOMPG:C4R0M2|C4R0M2\_KOMPG:C4R5C7|C4R5C7\_KOMPG:C4R148|C4R148\_KOMPG:C4R0V8|C4R0V8\_KOMPG:C4R1H3|C4R1H3\_KOMPG:C4QYQ2|C4QYQ2\_KOMPG:C4R276|C4R276\_KOMPG:C4R7S9|GET3\_KOMPG:C4R129|C4R129\_KOMPG:C4R1W8|C4R1W8\_KOMPG:C4R692|C4R692\_KOMPG:C4R847|C4R847\_KOMPG:C4R332|C4R332\_KOMPG:C4R565|C4R565\_KOMPG:C4R5V4|C4R5V4\_KOMPG:C4R5H2|C4R5H2\_KOMPG:C4QXZ9|C4QXZ9\_KOMPG:C4R855|C4R855\_KOMPG:C4R3H2|C4R3H2\_KOMPG:C4R0L5|C4R0L5\_KOMPG:C4R8D9|C4R8D9\_KOMPG:C4R3A9|C4R3A9\_KOMPG:C4R800|C4R800\_KOMPG |  |  | DB Search |
| NGGI | 28.18 | 359.1805 | 4 | -1.08 | 360.1865 | 6.05 | 2378 | 0 | 0 | 0 | C4QWQ5|C4QWQ5\_KOMPG:C4QY54|C4QY54\_KOMPG:C4QWG8|C4QWG8\_KOMPG:C4QZW2|C4QZW2\_KOMPG:C4R4A1|C4R4A1\_KOMPG:C4R3J4|C4R3J4\_KOMPG:C4QXV9|C4QXV9\_KOMPG:C4QWT6|C4QWT6\_KOMPG:C4R695|C4R695\_KOMPG:C4QXC1|C4QXC1\_KOMPG:C4R0N5|C4R0N5\_KOMPG:C4R2J4|C4R2J4\_KOMPG:C4R570|C4R570\_KOMPG:C4R102|C4R102\_KOMPG:C4R900|C4R900\_KOMPG:C4R5H8|C4R5H8\_KOMPG:C4R243|C4R243\_KOMPG:C4R143|C4R143\_KOMPG:C4R6K5|C4R6K5\_KOMPG:C4R178|C4R178\_KOMPG:C4QZA7|C4QZA7\_KOMPG:C4QZK6|C4QZK6\_KOMPG:C4R2I7|C4R2I7\_KOMPG:C4QZM0|C4QZM0\_KOMPG:C4R3A0|C4R3A0\_KOMPG:C4R8P6|C4R8P6\_KOMPG:C4QYK4|C4QYK4\_KOMPG:C4QYC0|C4QYC0\_KOMPG:C4R0E0|C4R0E0\_KOMPG |  |  | DB Search |
| NGGL | 28.18 | 359.1805 | 4 | -1.08 | 360.1865 | 6.05 | 2378 | 0 | 0 | 0 | C4R4E3|C4R4E3\_KOMPG:C4R811|C4R811\_KOMPG:C4R4H2|C4R4H2\_KOMPG:C4QWF7|C4QWF7\_KOMPG:C4QVA9|C4QVA9\_KOMPG:C4R3U6|C4R3U6\_KOMPG:C4R589|C4R589\_KOMPG:C4QZV6|C4QZV6\_KOMPG:C4QXV4|C4QXV4\_KOMPG:C4R6C2|PEX1\_KOMPG:C4QYB1|C4QYB1\_KOMPG:C4R0X5|C4R0X5\_KOMPG:C4QW62|C4QW62\_KOMPG:C4R4D6|C4R4D6\_KOMPG:C4QYP6|C4QYP6\_KOMPG:C4R5A0|C4R5A0\_KOMPG:C4R4D5|C4R4D5\_KOMPG:C4QYC6|C4QYC6\_KOMPG:C4R549|C4R549\_KOMPG:C4R8Y5|C4R8Y5\_KOMPG:C4QVI7|C4QVI7\_KOMPG:C4R131|C4R131\_KOMPG:C4QY42|C4QY42\_KOMPG:C4R451|C4R451\_KOMPG:C4R0G3|C4R0G3\_KOMPG:C4QYT3|C4QYT3\_KOMPG:C4QWN9|C4QWN9\_KOMPG:C4R3Y2|C4R3Y2\_KOMPG:C4QZS4|C4QZS4\_KOMPG:C4QXA7|C4QXA7\_KOMPG:C4QYY1|C4QYY1\_KOMPG:C4QYN7|C4QYN7\_KOMPG:C4R6H4|C4R6H4\_KOMPG:C4QY10|C4QY10\_KOMPG:C4R3S8|C4R3S8\_KOMPG |  |  | DB Search |
| GAPGGAAPG | 28.1 | 653.3132 | 9 | -3.45 | 654.3167 | 21.83 | 7995 | 4.68e2 | 1 | 1 | C4R887|C4R887\_KOMPG:C4R3X8|C4R3X8\_KOMPG |  |  | DB Search |
| Q(-17.03)QQPYP | 28 | 742.3286 | 6 | -0.48 | 743.3337 | 13.68 | 5404 | 1.42e3 | 1 | 1 | C4QVB3|C4QVB3\_KOMPG | Pyro-glu from Q | Q1:Pyro-glu from Q:1000 | DB Search |
| HCQSLQ | 27.97 | 714.3119 | 6 | -5.59 | 715.3134 | 9.61 | 3750 | 1.07e3 | 1 | 1 | C4R5H2|C4R5H2\_KOMPG |  |  | DB Search |
| IPPP | 27.73 | 422.2529 | 4 | -1.45 | 423.2585 | 9.57 | 3721 | 1.63e3 | 1 | 1 | C4QVV6|C4QVV6\_KOMPG:C4R339|C4R339\_KOMPG:C4R6S8|C4R6S8\_KOMPG:C4QVN4|C4QVN4\_KOMPG:C4QZ48|C4QZ48\_KOMPG:C4R289|C4R289\_KOMPG:C4R0G5|C4R0G5\_KOMPG:C4R5P4|C4R5P4\_KOMPG:C4R234|C4R234\_KOMPG:C4R8F3|C4R8F3\_KOMPG:C4R2B3|C4R2B3\_KOMPG:C4QWY2|C4QWY2\_KOMPG:C4QXJ0|C4QXJ0\_KOMPG:C4R569|C4R569\_KOMPG:C4R656|C4R656\_KOMPG:C4R888|C4R888\_KOMPG:C4QWA0|C4QWA0\_KOMPG:C4R2D0|C4R2D0\_KOMPG:C4R5W3|C4R5W3\_KOMPG:C4QYX9|C4QYX9\_KOMPG:C4R3M7|C4R3M7\_KOMPG:C4R2B5|C4R2B5\_KOMPG:C4R3T8|C4R3T8\_KOMPG:C4R370|C4R370\_KOMPG:C4QXU3|C4QXU3\_KOMPG:C4R048|C4R048\_KOMPG:C4QVX5|C4QVX5\_KOMPG:C4R8L3|C4R8L3\_KOMPG:C4QVI3|C4QVI3\_KOMPG:C4R7Y9|C4R7Y9\_KOMPG:C4QZW1|C4QZW1\_KOMPG:C4R0J8|C4R0J8\_KOMPG:C4QXR0|C4QXR0\_KOMPG:C4R3Z3|C4R3Z3\_KOMPG:C4R701|C4R701\_KOMPG:C4QXP4|C4QXP4\_KOMPG:C4QXZ9|C4QXZ9\_KOMPG:C4R666|C4R666\_KOMPG:C4R2Z9|C4R2Z9\_KOMPG:C4QW70|C4QW70\_KOMPG:C4QVG6|C4QVG6\_KOMPG:C4R3P1|C4R3P1\_KOMPG:C4R3G7|C4R3G7\_KOMPG |  |  | DB Search |
| LPPP | 27.73 | 422.2529 | 4 | -1.45 | 423.2585 | 9.57 | 3721 | 1.63e3 | 1 | 1 | C4QXF3|C4QXF3\_KOMPG:C4R811|C4R811\_KOMPG:C4R0V4|C4R0V4\_KOMPG:C4QY74|C4QY74\_KOMPG:C4QZL0|C4QZL0\_KOMPG:C4R271|C4R271\_KOMPG:C4R998|C4R998\_KOMPG:C4R609|C4R609\_KOMPG:C4R5M9|C4R5M9\_KOMPG:C4R476|C4R476\_KOMPG:C4R3M2|C4R3M2\_KOMPG:C4R8U8|C4R8U8\_KOMPG:C4QZ55|C4QZ55\_KOMPG:C4R6U7|C4R6U7\_KOMPG:C4R712|C4R712\_KOMPG:C4QWK2|C4QWK2\_KOMPG:C4R7P6|C4R7P6\_KOMPG:C4QXU4|C4QXU4\_KOMPG:C4R2P9|C4R2P9\_KOMPG:C4R1P2|C4R1P2\_KOMPG:C4R0T5|C4R0T5\_KOMPG:C4R7Q5|EXO5\_KOMPG:C4R5B6|C4R5B6\_KOMPG:C4R2X4|C4R2X4\_KOMPG:C4R2V0|C4R2V0\_KOMPG:C4QZT4|C4QZT4\_KOMPG:C4R5S2|C4R5S2\_KOMPG:C4R4Q2|C4R4Q2\_KOMPG:C4QVK5|C4QVK5\_KOMPG:C4R3D9|C4R3D9\_KOMPG:C4R6Y5|C4R6Y5\_KOMPG:C4R7G6|C4R7G6\_KOMPG:C4QUZ1|C4QUZ1\_KOMPG:C4R525|C4R525\_KOMPG:C4QY00|C4QY00\_KOMPG:C4QW72|C4QW72\_KOMPG:C4QW95|C4QW95\_KOMPG:C4R6D0|C4R6D0\_KOMPG:C4R2F3|C4R2F3\_KOMPG:C4R475|C4R475\_KOMPG:C4QXL0|C4QXL0\_KOMPG:C4R1G6|C4R1G6\_KOMPG:C4R9E8|C4R9E8\_KOMPG:C4QXT4|C4QXT4\_KOMPG:C4R1S9|C4R1S9\_KOMPG:C4QW84|C4QW84\_KOMPG:C4R714|C4R714\_KOMPG:C4R0M3|C4R0M3\_KOMPG:C4QZ88|C4QZ88\_KOMPG:C4R5Z1|C4R5Z1\_KOMPG:C4R808|C4R808\_KOMPG:C4R7J3|C4R7J3\_KOMPG:C4R928|C4R928\_KOMPG:C4R0H6|C4R0H6\_KOMPG:C4QVJ4|C4QVJ4\_KOMPG:C4QZY3|C4QZY3\_KOMPG:C4R3Z6|C4R3Z6\_KOMPG:C4QVK6|C4QVK6\_KOMPG:C4QZB6|C4QZB6\_KOMPG:C4QXJ7|C4QXJ7\_KOMPG:C4QXY8|C4QXY8\_KOMPG:C4QX25|C4QX25\_KOMPG:C4R3Y6|C4R3Y6\_KOMPG:C4R677|C4R677\_KOMPG:C4R8N5|C4R8N5\_KOMPG:C4R387|C4R387\_KOMPG |  |  | DB Search |
| ITGI | 27.55 | 402.2478 | 4 | -1.76 | 403.2534 | 15.04 | 5964 | 1.56e4 | 2 | 2 | C4QYU1|C4QYU1\_KOMPG:C4QYT7|C4QYT7\_KOMPG:C4R8G4|C4R8G4\_KOMPG:C4QXT1|C4QXT1\_KOMPG:C4R894|C4R894\_KOMPG:C4R4R3|C4R4R3\_KOMPG:C4QX96|C4QX96\_KOMPG:C4R2L3|C4R2L3\_KOMPG:C4QZG0|C4QZG0\_KOMPG:C4R3D3|C4R3D3\_KOMPG:C4R2R4|C4R2R4\_KOMPG:C4QW18|C4QW18\_KOMPG:C4R0N6|C4R0N6\_KOMPG:C4QYM8|C4QYM8\_KOMPG:C4QYT1|C4QYT1\_KOMPG:C4QW30|C4QW30\_KOMPG:C4QYZ1|C4QYZ1\_KOMPG:C4R664|C4R664\_KOMPG:C4QVL4|C4QVL4\_KOMPG:C4R4T2|C4R4T2\_KOMPG:C4R991|C4R991\_KOMPG:C4R3U1|C4R3U1\_KOMPG:C4QXU6|C4QXU6\_KOMPG:C4R8D3|C4R8D3\_KOMPG:C4QVV9|C4QVV9\_KOMPG:C4R7Z8|BMT4\_KOMPG:C4R3V3|C4R3V3\_KOMPG |  |  | DB Search |
| ITGL | 27.55 | 402.2478 | 4 | -1.76 | 403.2534 | 15.04 | 5964 | 1.56e4 | 2 | 2 | C4QXF3|C4QXF3\_KOMPG:C4R357|C4R357\_KOMPG:C4R5P0|C4R5P0\_KOMPG:C4R5D8|COQ4\_KOMPG:C4QWZ9|C4QWZ9\_KOMPG:C4R665|C4R665\_KOMPG:C4R614|C4R614\_KOMPG:C4QYP0|C4QYP0\_KOMPG:C4R737|C4R737\_KOMPG:C4R7P0|C4R7P0\_KOMPG:C4QW48|C4QW48\_KOMPG:C4R5M4|C4R5M4\_KOMPG:C4QW86|C4QW86\_KOMPG:C4QYT4|C4QYT4\_KOMPG:C4QVR8|C4QVR8\_KOMPG:C4R016|C4R016\_KOMPG:C4R057|C4R057\_KOMPG:C4QYD4|C4QYD4\_KOMPG:C4R169|C4R169\_KOMPG:C4R8Y4|C4R8Y4\_KOMPG:C4QZ47|C4QZ47\_KOMPG:C4R5F3|C4R5F3\_KOMPG:C4R877|C4R877\_KOMPG:C4QX78|C4QX78\_KOMPG:C4QWF7|C4QWF7\_KOMPG:C4R091|C4R091\_KOMPG:C4QZ93|C4QZ93\_KOMPG:C4R2A1|C4R2A1\_KOMPG:C4R008|C4R008\_KOMPG:C4R1K8|ERT1\_KOMPG:C4R8I2|C4R8I2\_KOMPG:C4R546|C4R546\_KOMPG:C4R7A7|C4R7A7\_KOMPG:C4R738|C4R738\_KOMPG:C4QZJ2|C4QZJ2\_KOMPG:C4R824|C4R824\_KOMPG:C4QV70|C4QV70\_KOMPG:C4R093|C4R093\_KOMPG:C4R703|C4R703\_KOMPG:C4QWP7|C4QWP7\_KOMPG:C4QXV3|C4QXV3\_KOMPG:C4R446|C4R446\_KOMPG:C4R6Z8|C4R6Z8\_KOMPG:C4QYF4|C4QYF4\_KOMPG:C4R165|C4R165\_KOMPG:C4QWW8|C4QWW8\_KOMPG:C4R3I1|C4R3I1\_KOMPG:C4R2V7|C4R2V7\_KOMPG:C4R565|C4R565\_KOMPG:C4R6W0|C4R6W0\_KOMPG:C4QYD5|C4QYD5\_KOMPG:C4R8K5|C4R8K5\_KOMPG:C4R3M1|C4R3M1\_KOMPG:C4R5W9|C4R5W9\_KOMPG |  |  | DB Search |
| LTGI | 27.55 | 402.2478 | 4 | -1.76 | 403.2534 | 15.04 | 5964 | 1.56e4 | 2 | 2 | C4R9E5|C4R9E5\_KOMPG:C4R3R4|C4R3R4\_KOMPG:C4R163|C4R163\_KOMPG:C4QXQ1|C4QXQ1\_KOMPG:C4R4U2|C4R4U2\_KOMPG:C4R1R9|C4R1R9\_KOMPG:C4QZS3|C4QZS3\_KOMPG:C4QZ80|C4QZ80\_KOMPG:C4QZF6|C4QZF6\_KOMPG:C4R4Q7|C4R4Q7\_KOMPG:C4R286|C4R286\_KOMPG:C4QWU6|C4QWU6\_KOMPG:C4QX60|C4QX60\_KOMPG:C4QVW1|C4QVW1\_KOMPG:C4QXK9|C4QXK9\_KOMPG:C4R8V7|C4R8V7\_KOMPG:C4QZW3|C4QZW3\_KOMPG:C4QYN2|C4QYN2\_KOMPG:C4QX47|C4QX47\_KOMPG:C4R7J5|C4R7J5\_KOMPG:C4R6F6|C4R6F6\_KOMPG:C4R2L0|PEX5\_KOMPG:Q92448|PFKA1\_KOMPG:C4R4F2|C4R4F2\_KOMPG:C4R3A0|C4R3A0\_KOMPG:C4R7X7|C4R7X7\_KOMPG:C4QVC9|C4QVC9\_KOMPG:C4R4E5|C4R4E5\_KOMPG:C4QYG9|C4QYG9\_KOMPG:C4R044|C4R044\_KOMPG:C4QZI1|C4QZI1\_KOMPG:C4R6V9|C4R6V9\_KOMPG:C4R3C2|C4R3C2\_KOMPG:C4R5C2|C4R5C2\_KOMPG:C4QV89|C4QV89\_KOMPG:C4QVE1|C4QVE1\_KOMPG:C4R2M2|C4R2M2\_KOMPG:C4QVX3|C4QVX3\_KOMPG:C4R328|C4R328\_KOMPG:C4R4P0|C4R4P0\_KOMPG:C4QW62|C4QW62\_KOMPG:C4R647|C4R647\_KOMPG:C4QVV7|C4QVV7\_KOMPG:C4R957|C4R957\_KOMPG:C4R4Y1|C4R4Y1\_KOMPG:C4R175|C4R175\_KOMPG:C4R6W1|C4R6W1\_KOMPG:C4R038|C4R038\_KOMPG:C4R9E1|C4R9E1\_KOMPG:C4QYA3|C4QYA3\_KOMPG:C4R3B6|C4R3B6\_KOMPG |  |  | DB Search |
| LTGL | 27.55 | 402.2478 | 4 | -1.76 | 403.2534 | 15.04 | 5964 | 1.56e4 | 2 | 2 | C4QWA7|C4QWA7\_KOMPG:P52710|CBPY\_KOMPG:C4QZF7|C4QZF7\_KOMPG:C4QV09|C4QV09\_KOMPG:C4R4B0|C4R4B0\_KOMPG:C4QZL5|C4QZL5\_KOMPG:C4R535|C4R535\_KOMPG:C4R195|C4R195\_KOMPG:C4R6R6|C4R6R6\_KOMPG:C4R0U6|C4R0U6\_KOMPG:C4QVJ7|C4QVJ7\_KOMPG:C4R6S9|C4R6S9\_KOMPG:C4QWK1|C4QWK1\_KOMPG:C4R0L6|C4R0L6\_KOMPG:C4R559|C4R559\_KOMPG:C4QW80|C4QW80\_KOMPG:C4R155|C4R155\_KOMPG:C4R1E5|C4R1E5\_KOMPG:C4QY28|C4QY28\_KOMPG:C4R8W7|C4R8W7\_KOMPG:C4QZM3|C4QZM3\_KOMPG:C4R6P0|C4R6P0\_KOMPG:C4R578|C4R578\_KOMPG:C4R0P1|C4R0P1\_KOMPG:C4R3P6|C4R3P6\_KOMPG:C4QY92|C4QY92\_KOMPG:C4R1S2|C4R1S2\_KOMPG:C4R8A9|C4R8A9\_KOMPG:C4QZ00|C4QZ00\_KOMPG:C4R1U0|C4R1U0\_KOMPG:C4R0C2|C4R0C2\_KOMPG:C4R2I4|C4R2I4\_KOMPG:C4R610|C4R610\_KOMPG:C4R4I3|C4R4I3\_KOMPG:C4QVL8|C4QVL8\_KOMPG:C4R921|C4R921\_KOMPG:C4QZH6|C4QZH6\_KOMPG:C4R3I8|C4R3I8\_KOMPG:C4QXI7|C4QXI7\_KOMPG:C4R2L6|C4R2L6\_KOMPG:C4R704|C4R704\_KOMPG:C4R714|C4R714\_KOMPG:C4R3G4|C4R3G4\_KOMPG:C4R432|SEY1\_KOMPG:C4QZ07|C4QZ07\_KOMPG:C4R4D2|C4R4D2\_KOMPG:C4QXH8|C4QXH8\_KOMPG:C4R2V3|C4R2V3\_KOMPG:C4R5P8|C4R5P8\_KOMPG:C4R5Q0|C4R5Q0\_KOMPG:C4R3C0|C4R3C0\_KOMPG:C4R867|C4R867\_KOMPG:C4QW46|C4QW46\_KOMPG:C4QXW4|C4QXW4\_KOMPG:C4R2N5|C4R2N5\_KOMPG:C4R6R4|C4R6R4\_KOMPG:C4R2Y5|C4R2Y5\_KOMPG:C4R172|C4R172\_KOMPG:C4R468|C4R468\_KOMPG:C4R2D4|C4R2D4\_KOMPG:C4R7M6|C4R7M6\_KOMPG:C4QXA3|C4QXA3\_KOMPG:C4R906|C4R906\_KOMPG:C4R247|C4R247\_KOMPG:C4R3I0|C4R3I0\_KOMPG:C4R062|C4R062\_KOMPG:C4QZ08|C4QZ08\_KOMPG:C4QX04|C4QX04\_KOMPG:C4R604|C4R604\_KOMPG:C4R3Q8|C4R3Q8\_KOMPG:C4R030|C4R030\_KOMPG:C4R7L0|C4R7L0\_KOMPG:C4R8E8|PURA\_KOMPG:C4QWD1|C4QWD1\_KOMPG:C4QXB9|C4QXB9\_KOMPG:C4R2G2|C4R2G2\_KOMPG:C4R361|C4R361\_KOMPG:C4R567|C4R567\_KOMPG:C4QVD6|SHO1\_KOMPG:C4R1Z8|C4R1Z8\_KOMPG:C4R2W8|C4R2W8\_KOMPG:C4R0W6|C4R0W6\_KOMPG:C4R7S6|C4R7S6\_KOMPG:C4QZB1|C4QZB1\_KOMPG:C4R697|C4R697\_KOMPG:C4QXS2|C4QXS2\_KOMPG |  |  | DB Search |
| T(+42.01)RRGGQ | 27.51 | 715.3725 | 6 | 8.91 | 716.3844 | 8.73 | 3378 | 3.25e3 | 1 | 1 | C4R2A4|C4R2A4\_KOMPG | Acetylation (Protein N-term) | T1:Acetylation (Protein N-term):1000 | DB Search |
| GGGI | 27.46 | 302.159 | 4 | -3.72 | 303.1644 | 6.06 | 2387 | 3.14e3 | 1 | 1 | C4R3D1|C4R3D1\_KOMPG:C4R5A9|C4R5A9\_KOMPG:C4R3B3|C4R3B3\_KOMPG:C4QXF3|C4QXF3\_KOMPG:C4QZ37|C4QZ37\_KOMPG:C4R6N7|C4R6N7\_KOMPG:C4QW17|C4QW17\_KOMPG:C4QYZ6|C4QYZ6\_KOMPG:C4R221|C4R221\_KOMPG:C4R1L3|C4R1L3\_KOMPG:C4R5S4|C4R5S4\_KOMPG:C4QWE3|C4QWE3\_KOMPG:C4R8P0|C4R8P0\_KOMPG:C4QWG3|C4QWG3\_KOMPG:C4QX73|C4QX73\_KOMPG:C4R4R6|C4R4R6\_KOMPG:C4QZI2|C4QZI2\_KOMPG:C4R4R8|ARO1\_KOMPG:C4R3L7|C4R3L7\_KOMPG:C4R8U4|C4R8U4\_KOMPG:C4R3S6|C4R3S6\_KOMPG:C4R2X6|C4R2X6\_KOMPG:C4R255|C4R255\_KOMPG:C4R312|C4R312\_KOMPG:C4QWH8|C4QWH8\_KOMPG:C4QUZ4|C4QUZ4\_KOMPG:C4QWK0|C4QWK0\_KOMPG:C4QZV5|C4QZV5\_KOMPG:C4R310|C4R310\_KOMPG:C4R3J3|C4R3J3\_KOMPG:C4R3K5|RTC5\_KOMPG:C4R7F9|C4R7F9\_KOMPG:C4R3R0|C4R3R0\_KOMPG:C4R7D9|MTNB\_KOMPG:C4QZU0|C4QZU0\_KOMPG:C4R3B7|C4R3B7\_KOMPG:C4R6W2|C4R6W2\_KOMPG:C4R723|C4R723\_KOMPG:C4R950|C4R950\_KOMPG:C4R0E7|C4R0E7\_KOMPG:C4R7L1|C4R7L1\_KOMPG:C4R524|C4R524\_KOMPG:C4R4A4|C4R4A4\_KOMPG:C4QZJ7|C4QZJ7\_KOMPG:C4QXF2|C4QXF2\_KOMPG:C4R5X7|C4R5X7\_KOMPG:C4R715|C4R715\_KOMPG:C4R096|C4R096\_KOMPG:C4QZ42|C4QZ42\_KOMPG:C4QWE7|C4QWE7\_KOMPG |  |  | DB Search |
| GGGL | 27.46 | 302.159 | 4 | -3.72 | 303.1644 | 6.06 | 2387 | 3.14e3 | 1 | 1 | C4R3X2|C4R3X2\_KOMPG:C4QVG7|C4QVG7\_KOMPG:C4R2Q4|C4R2Q4\_KOMPG:C4QZZ1|C4QZZ1\_KOMPG:C4R922|C4R922\_KOMPG:C4R3U0|GET2\_KOMPG:C4R925|C4R925\_KOMPG:C4R0I9|C4R0I9\_KOMPG:C4QYZ3|C4QYZ3\_KOMPG:C4QZ72|C4QZ72\_KOMPG:C4R3L0|C4R3L0\_KOMPG:C4R564|C4R564\_KOMPG:C4R7Q1|C4R7Q1\_KOMPG:C4R0S8|C4R0S8\_KOMPG:C4QXZ1|C4QXZ1\_KOMPG:C4R1J7|C4R1J7\_KOMPG:C4R7B4|C4R7B4\_KOMPG:C4R517|C4R517\_KOMPG:C4R8H7|C4R8H7\_KOMPG:C4R5Q4|C4R5Q4\_KOMPG:C4R7N9|C4R7N9\_KOMPG:C4QZU6|C4QZU6\_KOMPG:C4R4J5|C4R4J5\_KOMPG:C4R335|C4R335\_KOMPG:C4QYX1|C4QYX1\_KOMPG:C4QZN5|C4QZN5\_KOMPG:C4R058|C4R058\_KOMPG:C4R1X7|C4R1X7\_KOMPG:C4R2M6|C4R2M6\_KOMPG:C4R103|C4R103\_KOMPG:C4R4Y0|C4R4Y0\_KOMPG:C4R5H8|C4R5H8\_KOMPG:C4QYT0|C4QYT0\_KOMPG:C4R1Q7|C4R1Q7\_KOMPG:C4R5A8|C4R5A8\_KOMPG:C4QXV5|C4QXV5\_KOMPG:C4R3Q7|C4R3Q7\_KOMPG:C4R7I8|C4R7I8\_KOMPG:C4R1L1|C4R1L1\_KOMPG:C4QVK2|C4QVK2\_KOMPG:C4R6N8|C4R6N8\_KOMPG:C4R0S3|C4R0S3\_KOMPG:C4R1Z6|C4R1Z6\_KOMPG |  |  | DB Search |
| IGGI | 27.34 | 358.2216 | 4 | -4.39 | 359.2264 | 15.45 | 6114 | 7.1e3 | 1 | 1 | C4R418|C4R418\_KOMPG:C4QZQ5|C4QZQ5\_KOMPG:C4R8H4|C4R8H4\_KOMPG:C4R6R3|C4R6R3\_KOMPG:C4QVN9|C4QVN9\_KOMPG:C4R1S9|C4R1S9\_KOMPG:C4QZ23|C4QZ23\_KOMPG:C4R9D2|C4R9D2\_KOMPG:C4QWH6|C4QWH6\_KOMPG:C4QZD1|C4QZD1\_KOMPG:C4QV87|C4QV87\_KOMPG:C4QXP8|C4QXP8\_KOMPG:C4QVI9|C4QVI9\_KOMPG:C4QYA0|C4QYA0\_KOMPG:C4R8F4|C4R8F4\_KOMPG:C4R789|C4R789\_KOMPG:C4R265|C4R265\_KOMPG:C4R617|C4R617\_KOMPG:C4R885|C4R885\_KOMPG:C4R6Q2|C4R6Q2\_KOMPG:C4R051|C4R051\_KOMPG:C4R0Y6|C4R0Y6\_KOMPG:C4R172|C4R172\_KOMPG:C4R1U3|C4R1U3\_KOMPG:C4R8S0|C4R8S0\_KOMPG:C4R0Z7|C4R0Z7\_KOMPG:C4QW10|C4QW10\_KOMPG:C4R5M2|C4R5M2\_KOMPG:C4R0M6|C4R0M6\_KOMPG:C4R1W1|C4R1W1\_KOMPG:C4QW75|C4QW75\_KOMPG:C4R0F0|C4R0F0\_KOMPG:C4QX03|C4QX03\_KOMPG:C4R8G1|C4R8G1\_KOMPG:C4QZB0|C4QZB0\_KOMPG:C4R5U7|C4R5U7\_KOMPG:C4R713|C4R713\_KOMPG |  |  | DB Search |
| IGGL | 27.34 | 358.2216 | 4 | -4.39 | 359.2264 | 15.45 | 6114 | 7.1e3 | 1 | 1 | C4R2U7|C4R2U7\_KOMPG:C4R0R3|C4R0R3\_KOMPG:C4QZZ7|GEP3\_KOMPG:C4QVL1|C4QVL1\_KOMPG:C4R0V7|C4R0V7\_KOMPG:C4R809|C4R809\_KOMPG:C4QWG8|C4QWG8\_KOMPG:C4QY74|C4QY74\_KOMPG:C4R0V3|C4R0V3\_KOMPG:C4R6C2|PEX1\_KOMPG:C4R972|C4R972\_KOMPG:C4QVE2|C4QVE2\_KOMPG:C4R5V2|C4R5V2\_KOMPG:C4QV06|C4QV06\_KOMPG:C4R7C2|C4R7C2\_KOMPG:C4R4L7|C4R4L7\_KOMPG:C4R6I8|C4R6I8\_KOMPG:C4QX76|C4QX76\_KOMPG:C4R2E8|C4R2E8\_KOMPG:C4R8R8|C4R8R8\_KOMPG:C4QVJ7|C4QVJ7\_KOMPG:C4R745|C4R745\_KOMPG:C4R124|C4R124\_KOMPG:C4QXD1|C4QXD1\_KOMPG:C4R742|C4R742\_KOMPG:C4R7W9|C4R7W9\_KOMPG:C4R076|C4R076\_KOMPG:C4QX73|C4QX73\_KOMPG:C4R841|C4R841\_KOMPG:C4QWB9|C4QWB9\_KOMPG:C4R6Z3|C4R6Z3\_KOMPG:C4QYB0|C4QYB0\_KOMPG:C4QZQ0|C4QZQ0\_KOMPG:C4R2I9|C4R2I9\_KOMPG:C4R7G6|C4R7G6\_KOMPG:C4QYZ8|C4QYZ8\_KOMPG:C4QW88|C4QW88\_KOMPG:C4QVX7|C4QVX7\_KOMPG:C4R0B4|C4R0B4\_KOMPG:C4R1K4|C4R1K4\_KOMPG:C4QWL4|C4QWL4\_KOMPG:C4R2J1|C4R2J1\_KOMPG:C4QUZ4|C4QUZ4\_KOMPG:C4QX78|C4QX78\_KOMPG:C4QWF7|C4QWF7\_KOMPG:C4R7Z5|C4R7Z5\_KOMPG:C4QXI7|C4QXI7\_KOMPG:C4QVU3|SLD1\_KOMPG:C4QY14|C4QY14\_KOMPG:C4R9C8|C4R9C8\_KOMPG:C4R7G3|C4R7G3\_KOMPG:C4R262|C4R262\_KOMPG:C4R7C9|C4R7C9\_KOMPG:C4QWW0|C4QWW0\_KOMPG:C4R5L2|C4R5L2\_KOMPG:C4QW62|C4QW62\_KOMPG:C4QW55|C4QW55\_KOMPG:C4R4S9|C4R4S9\_KOMPG:C4R1H3|C4R1H3\_KOMPG:C4R293|C4R293\_KOMPG:C4R0H6|C4R0H6\_KOMPG:C4R1F7|C4R1F7\_KOMPG:C4R3B7|C4R3B7\_KOMPG:C4R7S3|C4R7S3\_KOMPG:C4R2K1|C4R2K1\_KOMPG:C4R647|C4R647\_KOMPG:C4R495|C4R495\_KOMPG:C4R807|C4R807\_KOMPG:C4QXE6|C4QXE6\_KOMPG:C4R1W8|C4R1W8\_KOMPG:C4QXQ0|C4QXQ0\_KOMPG:C4R1Y9|C4R1Y9\_KOMPG:C4R7C8|C4R7C8\_KOMPG:C4R4T5|C4R4T5\_KOMPG:C4R8B0|C4R8B0\_KOMPG:C4R1I5|C4R1I5\_KOMPG:C4QV24|C4QV24\_KOMPG:C4QYF1|C4QYF1\_KOMPG:C4R9A6|C4R9A6\_KOMPG:C4R8I9|C4R8I9\_KOMPG |  |  | DB Search |
| LGGI | 27.34 | 358.2216 | 4 | -4.39 | 359.2264 | 15.45 | 6114 | 7.1e3 | 1 | 1 | C4R8W0|C4R8W0\_KOMPG:C4R278|C4R278\_KOMPG:C4QXW1|C4QXW1\_KOMPG:C4R811|C4R811\_KOMPG:C4R1E9|C4R1E9\_KOMPG:C4R593|C4R593\_KOMPG:C4R558|C4R558\_KOMPG:C4R1P3|C4R1P3\_KOMPG:C4R2R8|C4R2R8\_KOMPG:C4R784|C4R784\_KOMPG:C4R350|C4R350\_KOMPG:C4R5S4|C4R5S4\_KOMPG:C4R952|C4R952\_KOMPG:C4R5A3|C4R5A3\_KOMPG:C4QZ86|C4QZ86\_KOMPG:C4R3Z8|C4R3Z8\_KOMPG:C4R2C5|C4R2C5\_KOMPG:C4QWQ7|C4QWQ7\_KOMPG:C4QVI3|C4QVI3\_KOMPG:C4R127|COA3\_KOMPG:C4QZF0|C4QZF0\_KOMPG:C4R4L4|C4R4L4\_KOMPG:C4R8A5|C4R8A5\_KOMPG:C4R1B2|C4R1B2\_KOMPG:C4QVC9|C4QVC9\_KOMPG:C4R0K8|C4R0K8\_KOMPG:C4QVU6|C4QVU6\_KOMPG:C4R189|C4R189\_KOMPG:C4QW73|C4QW73\_KOMPG:C4R7Q7|C4R7Q7\_KOMPG:C4R7X8|BMT2\_KOMPG:C4QZK9|C4QZK9\_KOMPG:C4R0D4|C4R0D4\_KOMPG:C4QZW2|C4QZW2\_KOMPG:C4R595|C4R595\_KOMPG:C4R0M3|C4R0M3\_KOMPG:C4R8D8|C4R8D8\_KOMPG:C4QW46|C4QW46\_KOMPG:C4R0A2|C4R0A2\_KOMPG:C4QZC3|C4QZC3\_KOMPG:C4R5N7|C4R5N7\_KOMPG:C4R6W7|C4R6W7\_KOMPG:C4QZB6|C4QZB6\_KOMPG:C4QYU7|C4QYU7\_KOMPG:C4QYL9|C4QYL9\_KOMPG:C4QXS7|C4QXS7\_KOMPG:C4R524|C4R524\_KOMPG:C4R8Z4|C4R8Z4\_KOMPG:C4R814|C4R814\_KOMPG:C4R855|C4R855\_KOMPG:C4QWG7|C4QWG7\_KOMPG:C4R7G5|C4R7G5\_KOMPG |  |  | DB Search |
| LGGL | 27.34 | 358.2216 | 4 | -4.39 | 359.2264 | 15.45 | 6114 | 7.1e3 | 1 | 1 | C4R5W6|C4R5W6\_KOMPG:C4R641|C4R641\_KOMPG:C4QYE1|C4QYE1\_KOMPG:C4R0J0|C4R0J0\_KOMPG:C4R1D2|C4R1D2\_KOMPG:C4R006|C4R006\_KOMPG:C4R4N4|C4R4N4\_KOMPG:C4R2B7|C4R2B7\_KOMPG:C4QZ37|C4QZ37\_KOMPG:C4QZJ9|C4QZJ9\_KOMPG:C4R1W0|C4R1W0\_KOMPG:C4QYL3|C4QYL3\_KOMPG:C4R511|C4R511\_KOMPG:C4R8T0|C4R8T0\_KOMPG:C4R5C6|C4R5C6\_KOMPG:C4R7D8|C4R7D8\_KOMPG:C4R0H8|C4R0H8\_KOMPG:C4R4Q8|C4R4Q8\_KOMPG:C4R3R6|C4R3R6\_KOMPG:C4R1X1|C4R1X1\_KOMPG:C4QWD4|C4QWD4\_KOMPG:C4QV07|C4QV07\_KOMPG:C4QVA7|C4QVA7\_KOMPG:C4R2K3|C4R2K3\_KOMPG:C4R6Z7|C4R6Z7\_KOMPG:C4QVE0|C4QVE0\_KOMPG:C4R7Q5|EXO5\_KOMPG:C4QYY0|C4QYY0\_KOMPG:C4R983|C4R983\_KOMPG:C4R2F5|C4R2F5\_KOMPG:C4R6E1|C4R6E1\_KOMPG:C4QV53|C4QV53\_KOMPG:C4R6F6|C4R6F6\_KOMPG:C4R263|C4R263\_KOMPG:C4QXM8|C4QXM8\_KOMPG:C4QVF7|C4QVF7\_KOMPG:C4R701|C4R701\_KOMPG:C4R1K5|C4R1K5\_KOMPG:C4QWV6|C4QWV6\_KOMPG:C4R0P0|C4R0P0\_KOMPG:C4QZI1|C4QZI1\_KOMPG:C4QY54|C4QY54\_KOMPG:C4R2Q9|C4R2Q9\_KOMPG:C4R5W2|C4R5W2\_KOMPG:C4QYB3|C4QYB3\_KOMPG:C4R289|C4R289\_KOMPG:C4R4B1|C4R4B1\_KOMPG:C4R645|C4R645\_KOMPG:C4R8Y7|C4R8Y7\_KOMPG:C4R580|C4R580\_KOMPG:C4R9G4|C4R9G4\_KOMPG:C4QZN1|C4QZN1\_KOMPG:C4R5H4|C4R5H4\_KOMPG:C4QWU4|C4QWU4\_KOMPG:C4QXB5|C4QXB5\_KOMPG:C4R3C3|C4R3C3\_KOMPG:C4R703|C4R703\_KOMPG:C4R194|C4R194\_KOMPG:C4R3A2|C4R3A2\_KOMPG:C4R886|C4R886\_KOMPG:C4R5L1|C4R5L1\_KOMPG:C4R0V2|C4R0V2\_KOMPG:C4QWR7|C4QWR7\_KOMPG:C4R950|C4R950\_KOMPG:C4R5L7|C4R5L7\_KOMPG:C4QWB6|C4QWB6\_KOMPG:C4QVP3|C4QVP3\_KOMPG:C4QX81|C4QX81\_KOMPG:C4R7A2|C4R7A2\_KOMPG:C4R9A9|C4R9A9\_KOMPG:C4R4T2|C4R4T2\_KOMPG:C4R7U3|C4R7U3\_KOMPG:C4QX77|C4QX77\_KOMPG:C4QYA2|C4QYA2\_KOMPG:C4R0Z6|C4R0Z6\_KOMPG:C4QXD7|C4QXD7\_KOMPG:C4R5E1|C4R5E1\_KOMPG:C4QUZ0|C4QUZ0\_KOMPG:C4R2W5|C4R2W5\_KOMPG:C4QYE7|C4QYE7\_KOMPG:C4R5Z8|C4R5Z8\_KOMPG:C4R6N8|C4R6N8\_KOMPG |  |  | DB Search |
| GSGSGC(-1.01)GSGSG | 26.99 | 810.2688 | 11 | -8.16 | 811.2675 | 1.90 | 778 | 1.27e4 | 1 | 1 | C4QYF1|C4QYF1\_KOMPG | Half of a disulfide bridge | C6:Half of a disulfide bridge:1000 | DB Search |
| FYR | 26.82 | 484.2434 | 3 | 1.64 | 485.2503 | 17.34 | 6854 | 0 | 0 | 0 | C4R7K5|C4R7K5\_KOMPG:C4R6I4|C4R6I4\_KOMPG:C4QVT8|C4QVT8\_KOMPG:C4R6R3|C4R6R3\_KOMPG:C4R603|C4R603\_KOMPG:C4QWB0|C4QWB0\_KOMPG:C4QY40|C4QY40\_KOMPG:C4QXH4|C4QXH4\_KOMPG:C4R558|C4R558\_KOMPG:C4R5E8|C4R5E8\_KOMPG:C4QYK0|RSSA\_KOMPG:C4R3M2|C4R3M2\_KOMPG:C4R542|C4R542\_KOMPG:C4QY88|C4QY88\_KOMPG:C4QYX9|C4QYX9\_KOMPG:C4R1B9|C4R1B9\_KOMPG:C4QWK2|C4QWK2\_KOMPG:C4QZA0|C4QZA0\_KOMPG:C4R7P6|C4R7P6\_KOMPG:C4QWZ4|C4QWZ4\_KOMPG:C4R628|PFF1\_KOMPG:C4QY93|C4QY93\_KOMPG:C4R0N2|C4R0N2\_KOMPG:C4QVF3|C4QVF3\_KOMPG:C4R484|C4R484\_KOMPG:C4QVK3|C4QVK3\_KOMPG:C4R155|C4R155\_KOMPG:C4R7E6|C4R7E6\_KOMPG:C4R2V0|C4R2V0\_KOMPG:C4QVD7|C4QVD7\_KOMPG:C4R968|C4R968\_KOMPG:C4R5K0|C4R5K0\_KOMPG:C4QVR8|C4QVR8\_KOMPG:C4R4L4|C4R4L4\_KOMPG:C4R362|C4R362\_KOMPG:C4R144|C4R144\_KOMPG:C4QYN9|C4QYN9\_KOMPG:C4QYV2|C4QYV2\_KOMPG:C4R8P9|C4R8P9\_KOMPG:C4R3C4|C4R3C4\_KOMPG:C4R3U4|C4R3U4\_KOMPG:C4QYW3|C4QYW3\_KOMPG:C4R667|C4R667\_KOMPG:C4R6I6|C4R6I6\_KOMPG:C4QVJ2|C4QVJ2\_KOMPG:C4R7C0|C4R7C0\_KOMPG:C4R0A8|C4R0A8\_KOMPG:C4R2Y2|C4R2Y2\_KOMPG:C4R7N8|C4R7N8\_KOMPG:C4QX78|C4QX78\_KOMPG:C4QWF7|C4QWF7\_KOMPG:C4R744|C4R744\_KOMPG:C4QVH3|C4QVH3\_KOMPG:C4R5G8|C4R5G8\_KOMPG:C4QVL3|C4QVL3\_KOMPG:C4QXN3|C4QXN3\_KOMPG:C4QXP2|C4QXP2\_KOMPG:C4R2L3|C4R2L3\_KOMPG:C4R289|C4R289\_KOMPG:C4R201|C4R201\_KOMPG:C4R447|C4R447\_KOMPG:C4R517|C4R517\_KOMPG:C4R4D6|C4R4D6\_KOMPG:C4QXA9|C4QXA9\_KOMPG:C4QVP1|C4QVP1\_KOMPG:C4QYQ8|C4QYQ8\_KOMPG:C4R838|C4R838\_KOMPG:C4QWQ8|C4QWQ8\_KOMPG:C4R325|C4R325\_KOMPG:C4R821|C4R821\_KOMPG:C4QYW2|C4QYW2\_KOMPG:C4R129|C4R129\_KOMPG:C4R7K4|C4R7K4\_KOMPG:C4R7Q2|C4R7Q2\_KOMPG:C4QWI0|C4QWI0\_KOMPG:C4R1S3|C4R1S3\_KOMPG:C4R120|C4R120\_KOMPG:C4QWD8|C4QWD8\_KOMPG:C4R219|C4R219\_KOMPG:C4R9A8|C4R9A8\_KOMPG:C4R207|C4R207\_KOMPG:C4R3V3|C4R3V3\_KOMPG:C4R625|C4R625\_KOMPG:C4R5R3|C4R5R3\_KOMPG:C4R3D1|C4R3D1\_KOMPG:C4R8W0|C4R8W0\_KOMPG:C4R3A1|C4R3A1\_KOMPG:C4QWA9|C4QWA9\_KOMPG:C4QVY3|C4QVY3\_KOMPG:C4QZ58|C4QZ58\_KOMPG:C4R895|C4R895\_KOMPG:C4R594|C4R594\_KOMPG:C4R183|C4R183\_KOMPG:C4R436|C4R436\_KOMPG:C4QVS9|C4QVS9\_KOMPG:C4R5C6|C4R5C6\_KOMPG:C4QXQ2|C4QXQ2\_KOMPG:C4QYU9|C4QYU9\_KOMPG:C4R7D8|C4R7D8\_KOMPG:C4R3N6|C4R3N6\_KOMPG:C4R8I4|C4R8I4\_KOMPG:C4R308|C4R308\_KOMPG:C4QXW6|C4QXW6\_KOMPG:C4R964|C4R964\_KOMPG:C4R7Z9|C4R7Z9\_KOMPG:C4R6X4|C4R6X4\_KOMPG:C4R8P0|C4R8P0\_KOMPG:C4R1T5|C4R1T5\_KOMPG:C4QZH5|C4QZH5\_KOMPG:C4R1J3|C4R1J3\_KOMPG:C4QX97|C4QX97\_KOMPG:C4R663|C4R663\_KOMPG:C4QWW1|C4QWW1\_KOMPG:C4QYS5|C4QYS5\_KOMPG:C4R7U9|C4R7U9\_KOMPG:C4QWS3|C4QWS3\_KOMPG:C4R8S0|C4R8S0\_KOMPG:C4R6V5|C4R6V5\_KOMPG:C4R7W9|C4R7W9\_KOMPG:C4QWT2|C4QWT2\_KOMPG:C4R090|C4R090\_KOMPG:C4QY42|C4QY42\_KOMPG:C4R994|C4R994\_KOMPG:C4QY52|C4QY52\_KOMPG:C4QYM6|C4QYM6\_KOMPG:C4R3H7|C4R3H7\_KOMPG:C4QYL7|AIM9\_KOMPG:C4QZE4|C4QZE4\_KOMPG:C4R0G2|C4R0G2\_KOMPG:C4R0W3|C4R0W3\_KOMPG:C4QZF5|C4QZF5\_KOMPG:C4QYY1|C4QYY1\_KOMPG:C4R255|C4R255\_KOMPG:C4QYZ8|C4QYZ8\_KOMPG:C4R965|C4R965\_KOMPG:C4QZN8|C4QZN8\_KOMPG:C4QYG9|C4QYG9\_KOMPG:C4QXR9|C4QXR9\_KOMPG:C4R892|MMM1\_KOMPG:C4R7E9|C4R7E9\_KOMPG:C4R029|C4R029\_KOMPG:C4QWX7|C4QWX7\_KOMPG:C4QZA5|C4QZA5\_KOMPG:C4R4W7|C4R4W7\_KOMPG:C4QVU1|C4QVU1\_KOMPG:C4R827|C4R827\_KOMPG:C4R5C2|C4R5C2\_KOMPG:C4R5T9|C4R5T9\_KOMPG:C4R2A1|C4R2A1\_KOMPG:C4R3N5|C4R3N5\_KOMPG:C4QXH8|C4QXH8\_KOMPG:C4R222|C4R222\_KOMPG:C4QZT5|C4QZT5\_KOMPG:C4R097|C4R097\_KOMPG:C4R7Q4|AIM11\_KOMPG:C4R5R2|C4R5R2\_KOMPG:C4QVQ2|C4QVQ2\_KOMPG:C4R0R5|C4R0R5\_KOMPG:C4R159|ATG28\_KOMPG:C4R766|C4R766\_KOMPG:C4R7Y8|C4R7Y8\_KOMPG:C4QYT1|C4QYT1\_KOMPG:C4QZ21|C4QZ21\_KOMPG:C4R0D7|C4R0D7\_KOMPG:C4QZX1|C4QZX1\_KOMPG:C4QW77|C4QW77\_KOMPG:C4R516|C4R516\_KOMPG:C4R5S5|C4R5S5\_KOMPG:C4R0S6|C4R0S6\_KOMPG:C4QXL1|C4QXL1\_KOMPG:C4QW06|C4QW06\_KOMPG:C4QZS9|C4QZS9\_KOMPG:C4R5G5|C4R5G5\_KOMPG:C4QX80|PSD1\_KOMPG:C4QY02|C4QY02\_KOMPG:C4QZ20|FEN1\_KOMPG:C4R7S4|C4R7S4\_KOMPG:C4R700|C4R700\_KOMPG:C4QXB9|C4QXB9\_KOMPG:C4R0F0|C4R0F0\_KOMPG:C4R5B7|C4R5B7\_KOMPG:C4R4J4|C4R4J4\_KOMPG:C4QVA1|C4QVA1\_KOMPG:C4R3F9|C4R3F9\_KOMPG:C4QVH1|C4QVH1\_KOMPG:C4R360|PSD2\_KOMPG:C4R7S6|C4R7S6\_KOMPG:C4R4P4|C4R4P4\_KOMPG:C4R6I0|C4R6I0\_KOMPG:C4QY68|PEX8\_KOMPG |  |  | DB Search |
| VVGI | 26.8 | 386.2529 | 4 | -6 | 387.2569 | 17.69 | 6924 | 2.17e3 | 1 | 1 | C4R8Y4|C4R8Y4\_KOMPG:C4R6I7|C4R6I7\_KOMPG:C4R080|C4R080\_KOMPG:C4R5S6|C4R5S6\_KOMPG:C4R6V9|C4R6V9\_KOMPG:C4QZ28|C4QZ28\_KOMPG:C4QZ23|C4QZ23\_KOMPG:C4R595|C4R595\_KOMPG:C4QZH7|C4QZH7\_KOMPG:C4R0V8|C4R0V8\_KOMPG:C4QV06|C4QV06\_KOMPG:C4QWW0|C4QWW0\_KOMPG:C4R9B4|C4R9B4\_KOMPG:C4R794|C4R794\_KOMPG:C4R8Y7|C4R8Y7\_KOMPG:C4R195|C4R195\_KOMPG:C4QWA3|C4QWA3\_KOMPG:C4R137|C4R137\_KOMPG:C4R4N3|C4R4N3\_KOMPG:C4R960|C4R960\_KOMPG:C4QV99|C4QV99\_KOMPG:C4R939|C4R939\_KOMPG:C4QXV0|C4QXV0\_KOMPG:C4QYT3|C4QYT3\_KOMPG:C4R155|C4R155\_KOMPG:C4QZP0|C4QZP0\_KOMPG:C4QYJ2|C4QYJ2\_KOMPG:C4R043|C4R043\_KOMPG:C4R3G5|C4R3G5\_KOMPG:C4R7S8|C4R7S8\_KOMPG:C4QZM9|C4QZM9\_KOMPG:C4R1I5|C4R1I5\_KOMPG:C4R3A0|C4R3A0\_KOMPG:C4R785|C4R785\_KOMPG:C4R8C8|C4R8C8\_KOMPG:C4R760|C4R760\_KOMPG:C4QV23|C4QV23\_KOMPG |  |  | DB Search |
| VVGL | 26.8 | 386.2529 | 4 | -6 | 387.2569 | 17.69 | 6924 | 2.17e3 | 1 | 1 | C4R8S8|C4R8S8\_KOMPG:C4R1E4|C4R1E4\_KOMPG:C4QXG2|C4QXG2\_KOMPG:C4QV61|C4QV61\_KOMPG:C4QX99|C4QX99\_KOMPG:C4QWU3|C4QWU3\_KOMPG:C4R4S8|C4R4S8\_KOMPG:C4QY22|C4QY22\_KOMPG:C4QV09|C4QV09\_KOMPG:C4QXW4|C4QXW4\_KOMPG:C4QW62|C4QW62\_KOMPG:C4R5U9|C4R5U9\_KOMPG:C4R159|ATG28\_KOMPG:C4R8C1|C4R8C1\_KOMPG:C4QYG3|C4QYG3\_KOMPG:C4QWL7|C4QWL7\_KOMPG:C4R1C0|C4R1C0\_KOMPG:C4QXB5|C4QXB5\_KOMPG:C4R320|C4R320\_KOMPG:C4R9C2|C4R9C2\_KOMPG:C4QYX9|C4QYX9\_KOMPG:C4QV45|C4QV45\_KOMPG:C4R869|C4R869\_KOMPG:C4R7Q5|EXO5\_KOMPG:C4QYX6|C4QYX6\_KOMPG:C4QW96|C4QW96\_KOMPG:C4R4T2|C4R4T2\_KOMPG:C4R373|C4R373\_KOMPG:C4R8J5|C4R8J5\_KOMPG:C4QYM6|C4QYM6\_KOMPG:C4R7P7|C4R7P7\_KOMPG:C4R7I4|C4R7I4\_KOMPG:C4R2D8|C4R2D8\_KOMPG:C4QYK8|C4QYK8\_KOMPG:C4R686|C4R686\_KOMPG:C4R0F0|C4R0F0\_KOMPG:C4R7X1|C4R7X1\_KOMPG:C4R1C7|C4R1C7\_KOMPG:C4QYI8|C4QYI8\_KOMPG:C4QXD8|C4QXD8\_KOMPG:C4QZL8|C4QZL8\_KOMPG:C4R971|C4R971\_KOMPG:C4R403|C4R403\_KOMPG:C4R4C9|C4R4C9\_KOMPG:C4R8S3|C4R8S3\_KOMPG:C4QWN2|C4QWN2\_KOMPG:C4R6K4|C4R6K4\_KOMPG:C4R2S4|C4R2S4\_KOMPG |  |  | DB Search |
| SPII | 26.66 | 428.2635 | 4 | -1.63 | 429.269 | 21.35 | 7905 | 0 | 0 | 0 | C4R5A9|C4R5A9\_KOMPG:C4R3B8|C4R3B8\_KOMPG:C4R5S6|C4R5S6\_KOMPG:C4QYB2|C4QYB2\_KOMPG:C4QXI7|C4QXI7\_KOMPG:C4R287|C4R287\_KOMPG:C4QZI3|C4QZI3\_KOMPG:C4R4F1|C4R4F1\_KOMPG:C4QWD9|C4QWD9\_KOMPG:C4R8D0|C4R8D0\_KOMPG:C4R6C5|C4R6C5\_KOMPG:C4R0V8|C4R0V8\_KOMPG:C4QXI5|C4QXI5\_KOMPG:C4R3R5|C4R3R5\_KOMPG:C4R9C6|C4R9C6\_KOMPG:C4R6P8|C4R6P8\_KOMPG:C4R2V6|C4R2V6\_KOMPG:C4QYH0|C4QYH0\_KOMPG:C4QXC1|C4QXC1\_KOMPG:C4R112|C4R112\_KOMPG:C4R3H9|C4R3H9\_KOMPG:C4QW09|C4QW09\_KOMPG:C4QZN0|C4QZN0\_KOMPG:C4QZA0|C4QZA0\_KOMPG:C4R881|C4R881\_KOMPG:C4R175|C4R175\_KOMPG:C4R252|C4R252\_KOMPG:C4R1U7|C4R1U7\_KOMPG:C4R2E9|C4R2E9\_KOMPG:C4R553|C4R553\_KOMPG:C4R8Q9|C4R8Q9\_KOMPG:C4R4J6|C4R4J6\_KOMPG:C4R2W3|C4R2W3\_KOMPG:C4R1W2|C4R1W2\_KOMPG:C4R6E1|C4R6E1\_KOMPG:C4QWZ1|C4QWZ1\_KOMPG:C4QYF1|C4QYF1\_KOMPG:C4R0X8|C4R0X8\_KOMPG:C4QXC2|C4QXC2\_KOMPG:C4QVR6|C4QVR6\_KOMPG:C4QYR5|C4QYR5\_KOMPG:C4QZJ0|C4QZJ0\_KOMPG |  |  | DB Search |
| SPIL | 26.66 | 428.2635 | 4 | -1.63 | 429.269 | 21.35 | 7905 | 0 | 0 | 0 | C4QVM2|C4QVM2\_KOMPG:C4QW34|C4QW34\_KOMPG:C4R368|C4R368\_KOMPG:C4QXQ1|C4QXQ1\_KOMPG:C4QY67|C4QY67\_KOMPG:C4R3X1|C4R3X1\_KOMPG:C4R0D8|C4R0D8\_KOMPG:C4QZF6|C4QZF6\_KOMPG:C4R1B1|C4R1B1\_KOMPG:C4R6X4|C4R6X4\_KOMPG:C4R726|C4R726\_KOMPG:C4R5G6|C4R5G6\_KOMPG:C4R3Z8|C4R3Z8\_KOMPG:C4QYS8|C4QYS8\_KOMPG:C4R0L6|C4R0L6\_KOMPG:C4R6F4|C4R6F4\_KOMPG:C4QZ27|C4QZ27\_KOMPG:C4R4R6|C4R4R6\_KOMPG:C4QZ46|C4QZ46\_KOMPG:C4R2Z8|C4R2Z8\_KOMPG:C4QXI9|C4QXI9\_KOMPG:C4QXD4|C4QXD4\_KOMPG:C4R2I9|C4R2I9\_KOMPG:C4R2F9|C4R2F9\_KOMPG:C4QV59|C4QV59\_KOMPG:C4R267|C4R267\_KOMPG:C4QY59|C4QY59\_KOMPG:C4R3P0|C4R3P0\_KOMPG:C4R0M9|C4R0M9\_KOMPG:C4R376|C4R376\_KOMPG:C4QX83|C4QX83\_KOMPG:C4QX89|C4QX89\_KOMPG:C4QVB3|C4QVB3\_KOMPG:C4R1J5|C4R1J5\_KOMPG:C4QXT4|C4QXT4\_KOMPG:C4R091|C4R091\_KOMPG:C4R706|C4R706\_KOMPG:C4R4A7|C4R4A7\_KOMPG:C4QZP7|C4QZP7\_KOMPG:C4R6H9|C4R6H9\_KOMPG:C4R4S5|C4R4S5\_KOMPG:C4R8L0|C4R8L0\_KOMPG:C4R340|C4R340\_KOMPG:C4R2M3|C4R2M3\_KOMPG:C4R8C1|C4R8C1\_KOMPG:C4R2D2|C4R2D2\_KOMPG:C4QZN1|C4QZN1\_KOMPG:C4R6X7|C4R6X7\_KOMPG:C4QYT1|C4QYT1\_KOMPG:C4QZ21|C4QZ21\_KOMPG:C4R957|C4R957\_KOMPG:C4QXJ3|C4QXJ3\_KOMPG:C4R6N0|C4R6N0\_KOMPG:C4R103|C4R103\_KOMPG:C4R692|C4R692\_KOMPG:C4QX81|C4QX81\_KOMPG:C4R604|C4R604\_KOMPG:C4R4R1|C4R4R1\_KOMPG:C4QXZ9|C4QXZ9\_KOMPG:C4R818|C4R818\_KOMPG:C4R6A0|C4R6A0\_KOMPG:C4QZ99|C4QZ99\_KOMPG |  |  | DB Search |
| SPLI | 26.66 | 428.2635 | 4 | -1.63 | 429.269 | 21.35 | 7905 | 0 | 0 | 0 | C4R6S8|C4R6S8\_KOMPG:C4QZJ3|C4QZJ3\_KOMPG:C4R895|C4R895\_KOMPG:C4R0M4|C4R0M4\_KOMPG:C4R049|C4R049\_KOMPG:C4R6M7|C4R6M7\_KOMPG:C4QV09|C4QV09\_KOMPG:C4QZZ6|C4QZZ6\_KOMPG:C4QWN7|C4QWN7\_KOMPG:C4R4G5|C4R4G5\_KOMPG:C4QV51|C4QV51\_KOMPG:C4R0U6|C4R0U6\_KOMPG:C4QZY5|C4QZY5\_KOMPG:C4R5M0|C4R5M0\_KOMPG:C4R0Q6|C4R0Q6\_KOMPG:C4R2V0|C4R2V0\_KOMPG:C4R3N2|C4R3N2\_KOMPG:C4R4K6|C4R4K6\_KOMPG:C4R283|C4R283\_KOMPG:C4R281|C4R281\_KOMPG:C4R5J3|C4R5J3\_KOMPG:C4R7M1|C4R7M1\_KOMPG:C4R1H0|C4R1H0\_KOMPG:C4R7G2|C4R7G2\_KOMPG:C4QVT3|C4QVT3\_KOMPG:C4R012|C4R012\_KOMPG:C4R6T2|C4R6T2\_KOMPG:C4R341|C4R341\_KOMPG:C4R3D7|C4R3D7\_KOMPG:C4R9F1|C4R9F1\_KOMPG:C4QZI1|C4QZI1\_KOMPG:C4QZV5|C4QZV5\_KOMPG:C4R2Q9|C4R2Q9\_KOMPG:C4R4A1|C4R4A1\_KOMPG:C4QYU4|C4QYU4\_KOMPG:C4R4K8|C4R4K8\_KOMPG:C4QVD4|C4QVD4\_KOMPG:C4R4V6|C4R4V6\_KOMPG:C4R6F5|C4R6F5\_KOMPG:C4QZ24|C4QZ24\_KOMPG:C4QXY0|C4QXY0\_KOMPG:C4QV36|C4QV36\_KOMPG:C4R0V2|C4R0V2\_KOMPG:C4QYG7|C4QYG7\_KOMPG:C4R1L5|C4R1L5\_KOMPG:C4R7K4|C4R7K4\_KOMPG:C4R872|C4R872\_KOMPG:C4R6M8|C4R6M8\_KOMPG:C4R995|C4R995\_KOMPG:C4QZB4|C4QZB4\_KOMPG:C4R2C0|C4R2C0\_KOMPG:C4QZX4|C4QZX4\_KOMPG |  |  | DB Search |
| SPLL | 26.66 | 428.2635 | 4 | -1.63 | 429.269 | 21.35 | 7905 | 0 | 0 | 0 | C4R1B7|C4R1B7\_KOMPG:C4QZT2|C4QZT2\_KOMPG:C4QWA4|C4QWA4\_KOMPG:C4R8H4|C4R8H4\_KOMPG:C4R4V0|C4R4V0\_KOMPG:C4QYS3|C4QYS3\_KOMPG:C4QWN8|C4QWN8\_KOMPG:C4R0D2|C4R0D2\_KOMPG:C4QZG8|C4QZG8\_KOMPG:C4R6C2|PEX1\_KOMPG:C4R7L5|C4R7L5\_KOMPG:C4R3L9|C4R3L9\_KOMPG:C4QYP0|C4QYP0\_KOMPG:C4R656|C4R656\_KOMPG:C4R1A8|C4R1A8\_KOMPG:C4QW48|C4QW48\_KOMPG:C4R5B8|C4R5B8\_KOMPG:C4QWQ7|C4QWQ7\_KOMPG:C4QVS8|C4QVS8\_KOMPG:C4QZI2|C4QZI2\_KOMPG:C4R7L6|C4R7L6\_KOMPG:C4R0E9|C4R0E9\_KOMPG:C4R263|C4R263\_KOMPG:C4R670|C4R670\_KOMPG:C4QXX7|C4QXX7\_KOMPG:C4QW67|C4QW67\_KOMPG:C4QVC9|C4QVC9\_KOMPG:C4QZN7|C4QZN7\_KOMPG:C4R7W5|C4R7W5\_KOMPG:C4R3W4|C4R3W4\_KOMPG:C4QWX4|C4QWX4\_KOMPG:C4R233|C4R233\_KOMPG:C4R166|C4R166\_KOMPG:C4R5I0|C4R5I0\_KOMPG:C4R078|C4R078\_KOMPG:C4R541|C4R541\_KOMPG:C4QZA5|C4QZA5\_KOMPG:C4R5N9|C4R5N9\_KOMPG:C4QXT1|C4QXT1\_KOMPG:C4QWV3|C4QWV3\_KOMPG:C4R8Z7|C4R8Z7\_KOMPG:C4R5W2|C4R5W2\_KOMPG:C4R879|C4R879\_KOMPG:C4R7Q4|AIM11\_KOMPG:C4R328|C4R328\_KOMPG:C4QYA7|C4QYA7\_KOMPG:C4R2B8|C4R2B8\_KOMPG:C4QW37|C4QW37\_KOMPG:C4QZJ2|C4QZJ2\_KOMPG:C4QZQ9|C4QZQ9\_KOMPG:C4R066|C4R066\_KOMPG:C4QZF3|C4QZF3\_KOMPG:C4R7H2|C4R7H2\_KOMPG:C4R4R4|C4R4R4\_KOMPG:C4R6L0|C4R6L0\_KOMPG:C4R194|C4R194\_KOMPG:C4R5L1|C4R5L1\_KOMPG:C4R723|C4R723\_KOMPG:C4QYK2|C4QYK2\_KOMPG:C4R3R1|C4R3R1\_KOMPG:C4QZ64|C4QZ64\_KOMPG:C4R4P7|C4R4P7\_KOMPG:C4R4X5|C4R4X5\_KOMPG:C4R038|C4R038\_KOMPG:C4QZW1|C4QZW1\_KOMPG:C4R3W0|C4R3W0\_KOMPG:C4R832|C4R832\_KOMPG:C4R2H9|C4R2H9\_KOMPG:C4R1V8|C4R1V8\_KOMPG:C4R3Z3|C4R3Z3\_KOMPG:C4R483|C4R483\_KOMPG:C4R6Z4|C4R6Z4\_KOMPG:C4R5V0|C4R5V0\_KOMPG:C4R0Z4|C4R0Z4\_KOMPG:C4QXM5|C4QXM5\_KOMPG:C4R713|C4R713\_KOMPG:C4R3P1|C4R3P1\_KOMPG |  |  | DB Search |
| SAGLVNS | 26.66 | 646.3286 | 7 | -5.62 | 647.3306 | 8.58 | 3269 | 1.55e4 | 1 | 1 | C4R1H4|C4R1H4\_KOMPG |  |  | DB Search |
| LSVGVVLQ | 26.53 | 813.496 | 8 | -4.82 | 814.4973 | 28.73 | 10013 | 3.04e3 | 1 | 1 | C4R0N9|C4R0N9\_KOMPG |  |  | DB Search |
| SVGV | 26.46 | 360.2009 | 4 | -6.45 | 361.2049 | 8.68 | 3331 | 1.41e4 | 1 | 1 | C4R3B8|C4R3B8\_KOMPG:C4QXJ1|C4QXJ1\_KOMPG:C4QYT9|C4QYT9\_KOMPG:C4R0N9|C4R0N9\_KOMPG:C4R842|C4R842\_KOMPG:C4R8D6|C4R8D6\_KOMPG:C4QZ69|C4QZ69\_KOMPG:C4QVI1|C4QVI1\_KOMPG:C4QZC7|C4QZC7\_KOMPG:C4R245|C4R245\_KOMPG:C4R7X2|C4R7X2\_KOMPG:C4R737|C4R737\_KOMPG:C4R1L3|C4R1L3\_KOMPG:C4R2A4|C4R2A4\_KOMPG:C4R454|C4R454\_KOMPG:C4R9C1|C4R9C1\_KOMPG:C4R961|C4R961\_KOMPG:C4R6E2|C4R6E2\_KOMPG:C4QV36|C4QV36\_KOMPG:C4QWV9|C4QWV9\_KOMPG:C4R0E7|C4R0E7\_KOMPG:C4QX98|C4QX98\_KOMPG:C4R1C3|C4R1C3\_KOMPG:C4QXU3|C4QXU3\_KOMPG:C4QVW8|C4QVW8\_KOMPG:C4R804|C4R804\_KOMPG:C4R7W6|C4R7W6\_KOMPG:C4R0S6|C4R0S6\_KOMPG:C4R2G7|C4R2G7\_KOMPG:C4R050|C4R050\_KOMPG:C4R778|C4R778\_KOMPG:C4QWH2|C4QWH2\_KOMPG:C4QYG1|C4QYG1\_KOMPG:C4QYV2|C4QYV2\_KOMPG:C4QVR5|C4QVR5\_KOMPG:C4QVN6|C4QVN6\_KOMPG |  |  | DB Search |
| YSIF | 26.31 | 528.2584 | 4 | -2.91 | 529.2628 | 34.97 | 11598 | 3.74e3 | 1 | 1 | C4QZ47|C4QZ47\_KOMPG:C4QY63|C4QY63\_KOMPG:C4QWG8|C4QWG8\_KOMPG:C4R2R1|C4R2R1\_KOMPG:C4R6F1|C4R6F1\_KOMPG:C4R083|C4R083\_KOMPG:C4R4D4|C4R4D4\_KOMPG:C4QZZ6|C4QZZ6\_KOMPG:C4QW62|C4QW62\_KOMPG:C4R3Y9|C4R3Y9\_KOMPG:C4R803|C4R803\_KOMPG:C4QVC7|C4QVC7\_KOMPG:C4R5Q6|C4R5Q6\_KOMPG:C4R6L8|C4R6L8\_KOMPG:C4R5P5|C4R5P5\_KOMPG:C4QZ94|C4QZ94\_KOMPG:C4R498|C4R498\_KOMPG:C4R983|C4R983\_KOMPG:C4R050|C4R050\_KOMPG:C4QZR8|C4QZR8\_KOMPG:C4R8Z2|C4R8Z2\_KOMPG:C4R6H0|C4R6H0\_KOMPG:C4R1S3|C4R1S3\_KOMPG:C4QYZ8|C4QYZ8\_KOMPG:C4QVT0|C4QVT0\_KOMPG:C4R3L1|C4R3L1\_KOMPG:C4QYA3|C4QYA3\_KOMPG |  |  | DB Search |
| YSLF | 26.31 | 528.2584 | 4 | -2.91 | 529.2628 | 34.97 | 11598 | 3.74e3 | 1 | 1 | C4R672|C4R672\_KOMPG:C4QWV6|C4QWV6\_KOMPG:C4QYW7|C4QYW7\_KOMPG:C4R6V9|C4R6V9\_KOMPG:C4R2Q9|C4R2Q9\_KOMPG:C4QXT4|C4QXT4\_KOMPG:C4R658|C4R658\_KOMPG:C4R669|C4R669\_KOMPG:C4QXI5|C4QXI5\_KOMPG:C4R381|C4R381\_KOMPG:C4R3X7|C4R3X7\_KOMPG:C4QWB8|C4QWB8\_KOMPG:C4R5X0|C4R5X0\_KOMPG:C4R4T2|C4R4T2\_KOMPG:C4QYT3|C4QYT3\_KOMPG:C4QXA2|C4QXA2\_KOMPG:C4R3A8|C4R3A8\_KOMPG:C4QV24|C4QV24\_KOMPG:C4R3U7|C4R3U7\_KOMPG:C4R0N3|C4R0N3\_KOMPG:C4R1G0|C4R1G0\_KOMPG:C4R818|C4R818\_KOMPG:C4R6A3|C4R6A3\_KOMPG:C4QYK4|C4QYK4\_KOMPG:C4QV23|C4QV23\_KOMPG:C4R0G4|C4R0G4\_KOMPG:C4QXZ3|C4QXZ3\_KOMPG |  |  | DB Search |
| E(-18.01)QPPYP | 26.29 | 711.3228 | 6 | -3.74 | 712.3256 | 17.49 | 6909 | 4.48e3 | 1 | 1 | C4R6B2|C4R6B2\_KOMPG | Pyro-glu from E | E1:Pyro-glu from E:1000 | DB Search |
| ETW | 26.2 | 434.1801 | 3 | -4.77 | 435.1843 | 12.45 | 4871 | 1.27e3 | 1 | 1 | C4R5V3|C4R5V3\_KOMPG:C4R6N9|C4R6N9\_KOMPG:C4R2V8|C4R2V8\_KOMPG:C4R3K3|C4R3K3\_KOMPG:C4QX07|C4QX07\_KOMPG:C4R603|C4R603\_KOMPG:C4R2C9|C4R2C9\_KOMPG:C4R4R3|C4R4R3\_KOMPG:C4QYR2|C4QYR2\_KOMPG:C4QY67|C4QY67\_KOMPG:C4QZZ8|C4QZZ8\_KOMPG:C4R747|C4R747\_KOMPG:C4R617|C4R617\_KOMPG:C4R1A8|C4R1A8\_KOMPG:C4R540|C4R540\_KOMPG:C4R5Z4|C4R5Z4\_KOMPG:C4QY66|C4QY66\_KOMPG:C4R1T4|C4R1T4\_KOMPG:C4QWS3|C4QWS3\_KOMPG:C4R8S0|C4R8S0\_KOMPG:C4R1P2|C4R1P2\_KOMPG:C4QXK9|C4QXK9\_KOMPG:C4R0U3|C4R0U3\_KOMPG:C4R192|VPS10\_KOMPG:C4R6W6|C4R6W6\_KOMPG:C4R4C7|C4R4C7\_KOMPG:C4R338|C4R338\_KOMPG:C4R2F8|C4R2F8\_KOMPG:C4R7S0|C4R7S0\_KOMPG:C4R4I8|C4R4I8\_KOMPG:C4QVC9|C4QVC9\_KOMPG:C4R9D7|C4R9D7\_KOMPG:C4QY50|C4QY50\_KOMPG:C4QVN0|C4QVN0\_KOMPG:C4R117|C4R117\_KOMPG:C4QYU1|C4QYU1\_KOMPG:C4QZ18|C4QZ18\_KOMPG:C4QZ88|C4QZ88\_KOMPG:C4R477|C4R477\_KOMPG:C4R4Z9|C4R4Z9\_KOMPG:C4R4D1|C4R4D1\_KOMPG:C4R438|C4R438\_KOMPG:C4QZG5|C4QZG5\_KOMPG:C4R215|C4R215\_KOMPG:C4QWL3|C4QWL3\_KOMPG:C4QWL7|C4QWL7\_KOMPG:C4QZC3|C4QZC3\_KOMPG:C4R434|C4R434\_KOMPG:C4R957|C4R957\_KOMPG:C4QV28|C4QV28\_KOMPG:C4QX36|C4QX36\_KOMPG:C4R7K8|C4R7K8\_KOMPG:C4QX00|C4QX00\_KOMPG:C4QYR1|C4QYR1\_KOMPG:C4R0G9|C4R0G9\_KOMPG:C4R8K2|C4R8K2\_KOMPG:C4QWB6|C4QWB6\_KOMPG:C4QY72|C4QY72\_KOMPG:C4R0I1|C4R0I1\_KOMPG:C4R250|C4R250\_KOMPG:C4R407|C4R407\_KOMPG:C4QVA1|C4QVA1\_KOMPG:C4R332|C4R332\_KOMPG:C4QZX6|C4QZX6\_KOMPG:C4R6P7|C4R6P7\_KOMPG:C4R6W0|C4R6W0\_KOMPG:C4QZ42|C4QZ42\_KOMPG:C4R2F4|C4R2F4\_KOMPG:C4R818|C4R818\_KOMPG:C4QYK4|C4QYK4\_KOMPG:C4QYY9|C4QYY9\_KOMPG:C4R0C8|C4R0C8\_KOMPG |  |  | DB Search |
| TIPT | 26.11 | 430.2427 | 4 | -4.36 | 431.2471 | 9.16 | 3520 | 0 | 0 | 0 | C4QV17|GSM1\_KOMPG:C4R0Z3|C4R0Z3\_KOMPG:C4QZV5|C4QZV5\_KOMPG:C4R029|C4R029\_KOMPG:C4QXK2|C4QXK2\_KOMPG:C4QYZ3|C4QYZ3\_KOMPG:C4R4C3|C4R4C3\_KOMPG:C4QVL3|C4QVL3\_KOMPG:C4R5G8|C4R5G8\_KOMPG:C4QXZ4|C4QXZ4\_KOMPG:C4R7V7|C4R7V7\_KOMPG:C4R2A2|C4R2A2\_KOMPG:C4R0S8|C4R0S8\_KOMPG:C4R887|C4R887\_KOMPG:C4R1N5|C4R1N5\_KOMPG:C4R3W9|C4R3W9\_KOMPG:C4QWL3|C4QWL3\_KOMPG:C4R2M3|C4R2M3\_KOMPG:C4R251|C4R251\_KOMPG:C4QXQ9|C4QXQ9\_KOMPG:C4R2U8|C4R2U8\_KOMPG:C4QV45|C4QV45\_KOMPG:C4R492|OCA5\_KOMPG:C4QZ71|C4QZ71\_KOMPG:C4R1N9|C4R1N9\_KOMPG:C4R3V8|C4R3V8\_KOMPG:C4R081|C4R081\_KOMPG:C4R2W7|C4R2W7\_KOMPG:C4R062|C4R062\_KOMPG:C4QVM0|C4QVM0\_KOMPG:C4R4T7|C4R4T7\_KOMPG:C4R0G7|C4R0G7\_KOMPG:C4R8D3|C4R8D3\_KOMPG:C4R5N1|C4R5N1\_KOMPG:C4R8I1|C4R8I1\_KOMPG:C4R701|C4R701\_KOMPG:C4R3G7|C4R3G7\_KOMPG |  |  | DB Search |
| TLPT | 26.11 | 430.2427 | 4 | -4.36 | 431.2471 | 9.16 | 3520 | 0 | 0 | 0 | C4QZX5|C4QZX5\_KOMPG:C4QZ62|C4QZ62\_KOMPG:C4R811|C4R811\_KOMPG:C4R1M9|C4R1M9\_KOMPG:C4R4Y6|C4R4Y6\_KOMPG:C4R6A1|C4R6A1\_KOMPG:C4R5E2|C4R5E2\_KOMPG:C4R025|C4R025\_KOMPG:C4QYB1|C4QYB1\_KOMPG:C4R8N7|C4R8N7\_KOMPG:C4R505|C4R505\_KOMPG:C4R7H6|C4R7H6\_KOMPG:C4R027|C4R027\_KOMPG:C4R2B3|C4R2B3\_KOMPG:C4R6S7|C4R6S7\_KOMPG:C4R4Q7|C4R4Q7\_KOMPG:C4R8F1|C4R8F1\_KOMPG:C4R1U8|C4R1U8\_KOMPG:C4QXL7|C4QXL7\_KOMPG:C4QYY2|C4QYY2\_KOMPG:C4R540|C4R540\_KOMPG:C4QW94|C4QW94\_KOMPG:C4QZH5|C4QZH5\_KOMPG:C4R0X6|C4R0X6\_KOMPG:C4R5B0|C4R5B0\_KOMPG:C4R2I0|C4R2I0\_KOMPG:C4QXL2|C4QXL2\_KOMPG:C4R7M6|C4R7M6\_KOMPG:C4R8C9|AIM23\_KOMPG:C4R2G6|C4R2G6\_KOMPG:C4QWT0|C4QWT0\_KOMPG:C4QZ34|C4QZ34\_KOMPG:C4R7B3|C4R7B3\_KOMPG:C4R5H2|C4R5H2\_KOMPG:C4QVX7|C4QVX7\_KOMPG:C4R1A9|C4R1A9\_KOMPG:C4R5V0|C4R5V0\_KOMPG:C4R0L5|C4R0L5\_KOMPG:C4R6I0|C4R6I0\_KOMPG:C4R556|C4R556\_KOMPG |  |  | DB Search |
| IFSP | 26.07 | 462.2478 | 4 | -0.35 | 463.2538 | 20.06 | 7534 | 6.05e2 | 1 | 1 | C4R4V2|C4R4V2\_KOMPG:C4R2Q9|C4R2Q9\_KOMPG:C4R5B2|C4R5B2\_KOMPG:C4QWF7|C4QWF7\_KOMPG:C4R2H0|C4R2H0\_KOMPG:C4R204|C4R204\_KOMPG:C4R0Z0|C4R0Z0\_KOMPG:C4QWL1|C4QWL1\_KOMPG:C4R837|C4R837\_KOMPG:C4R4G5|C4R4G5\_KOMPG:C4R228|C4R228\_KOMPG:C4QVT4|C4QVT4\_KOMPG:C4QZ60|C4QZ60\_KOMPG:C4R4L1|C4R4L1\_KOMPG:C4QYY0|C4QYY0\_KOMPG:C4R6R7|C4R6R7\_KOMPG:C4R4A5|C4R4A5\_KOMPG:C4R1Y9|C4R1Y9\_KOMPG:C4QYF8|C4QYF8\_KOMPG:C4R2Z8|C4R2Z8\_KOMPG:C4R2T3|C4R2T3\_KOMPG:C4R349|C4R349\_KOMPG:C4R7R0|C4R7R0\_KOMPG:C4R144|C4R144\_KOMPG:C4R5Y2|C4R5Y2\_KOMPG:C4QVT3|C4QVT3\_KOMPG:C4R1S6|C4R1S6\_KOMPG:C4QYS2|C4QYS2\_KOMPG:C4R6W5|C4R6W5\_KOMPG |  |  | DB Search |
| LFSP | 26.07 | 462.2478 | 4 | -0.35 | 463.2538 | 20.06 | 7534 | 6.05e2 | 1 | 1 | C4R908|C4R908\_KOMPG:C4QXB3|C4QXB3\_KOMPG:C4R337|C4R337\_KOMPG:C4QWN3|C4QWN3\_KOMPG:C4R6K9|C4R6K9\_KOMPG:C4R7Q0|C4R7Q0\_KOMPG:C4R5Z3|C4R5Z3\_KOMPG:C4R8J1|C4R8J1\_KOMPG:C4R1S9|C4R1S9\_KOMPG:C4R6S8|C4R6S8\_KOMPG:C4R8D6|C4R8D6\_KOMPG:C4R0K6|C4R0K6\_KOMPG:C4R0V4|C4R0V4\_KOMPG:C4R3K7|C4R3K7\_KOMPG:C4QV22|C4QV22\_KOMPG:C4R5T1|ATG30\_KOMPG:C4R8U6|C4R8U6\_KOMPG:C4R5X3|C4R5X3\_KOMPG:C4R202|C4R202\_KOMPG:C4R7Z1|C4R7Z1\_KOMPG:C4QXB0|C4QXB0\_KOMPG:C4R2U0|C4R2U0\_KOMPG:C4R3U5|C4R3U5\_KOMPG:C4R737|C4R737\_KOMPG:C4R1P8|C4R1P8\_KOMPG:C4QW51|C4QW51\_KOMPG:C4QYX9|C4QYX9\_KOMPG:C4QXJ3|C4QXJ3\_KOMPG:C4R1J3|C4R1J3\_KOMPG:C4R5D1|C4R5D1\_KOMPG:C4QVX5|C4QVX5\_KOMPG:C4QZ64|C4QZ64\_KOMPG:C4R7R8|C4R7R8\_KOMPG:C4R574|C4R574\_KOMPG:C4R210|C4R210\_KOMPG:C4R6F6|C4R6F6\_KOMPG:C4R8V2|C4R8V2\_KOMPG:C4QV64|C4QV64\_KOMPG:C4R0Z6|C4R0Z6\_KOMPG:C4R1F1|C4R1F1\_KOMPG:C4R901|C4R901\_KOMPG:C4R317|C4R317\_KOMPG:C4R1T6|C4R1T6\_KOMPG:C4R0S4|C4R0S4\_KOMPG:C4R8H2|C4R8H2\_KOMPG:C4R176|C4R176\_KOMPG:C4R267|C4R267\_KOMPG |  |  | DB Search |
| TIVI | 26.01 | 444.2948 | 4 | -4 | 445.2992 | 28.31 | 9909 | 1.89e3 | 1 | 1 | C4R9D9|C4R9D9\_KOMPG:C4R418|C4R418\_KOMPG:C4QXA5|PFKA2\_KOMPG:C4QY27|C4QY27\_KOMPG:C4QZR9|C4QZR9\_KOMPG:C4R6H3|LIS1\_KOMPG:C4QYB2|C4QYB2\_KOMPG:C4R455|C4R455\_KOMPG:C4R2J5|C4R2J5\_KOMPG:C4R094|C4R094\_KOMPG:C4R9C9|C4R9C9\_KOMPG:C4QWZ9|C4QWZ9\_KOMPG:C4R8I2|C4R8I2\_KOMPG:C4R630|C4R630\_KOMPG:C4QZ24|C4QZ24\_KOMPG:C4R3A8|C4R3A8\_KOMPG:C4QXA9|C4QXA9\_KOMPG:C4R508|C4R508\_KOMPG:C4QYP6|C4QYP6\_KOMPG:C4R137|C4R137\_KOMPG:C4QWV8|C4QWV8\_KOMPG:C4QZ04|C4QZ04\_KOMPG:C4R3S5|C4R3S5\_KOMPG:C4R8A9|C4R8A9\_KOMPG |  |  | DB Search |
| TIVL | 26.01 | 444.2948 | 4 | -4 | 445.2992 | 28.31 | 9909 | 1.89e3 | 1 | 1 | C4R315|C4R315\_KOMPG:C4R8D7|ATG37\_KOMPG:C4QZD7|C4QZD7\_KOMPG:C4R3M8|C4R3M8\_KOMPG:C4QZ59|C4QZ59\_KOMPG:C4R091|C4R091\_KOMPG:C4R3G4|C4R3G4\_KOMPG:C4R141|C4R141\_KOMPG:C4R183|C4R183\_KOMPG:C4QVU5|C4QVU5\_KOMPG:C4R5N2|C4R5N2\_KOMPG:C4R4P0|C4R4P0\_KOMPG:C4QYZ7|C4QYZ7\_KOMPG:C4QZC3|C4QZC3\_KOMPG:C4R8I0|C4R8I0\_KOMPG:C4QX60|C4QX60\_KOMPG:C4R172|C4R172\_KOMPG:C4R468|C4R468\_KOMPG:C4R2D4|C4R2D4\_KOMPG:C4QX04|C4QX04\_KOMPG:C4R250|C4R250\_KOMPG:C4R7R8|C4R7R8\_KOMPG:C4QZ46|C4QZ46\_KOMPG:C4R4L5|C4R4L5\_KOMPG:C4QXJ6|C4QXJ6\_KOMPG:C4R0W3|C4R0W3\_KOMPG:C4R3P6|C4R3P6\_KOMPG:C4R5E1|C4R5E1\_KOMPG:C4R6T6|C4R6T6\_KOMPG:C4R6W0|C4R6W0\_KOMPG:C4R8T6|C4R8T6\_KOMPG:C4R2F8|C4R2F8\_KOMPG:C4QVH1|C4QVH1\_KOMPG:C4QVC9|C4QVC9\_KOMPG:C4R0C3|C4R0C3\_KOMPG:C4QVT3|C4QVT3\_KOMPG:C4R6H2|C4R6H2\_KOMPG |  |  | DB Search |
| TLVI | 26.01 | 444.2948 | 4 | -4 | 445.2992 | 28.31 | 9909 | 1.89e3 | 1 | 1 | C4R3W3|C4R3W3\_KOMPG:C4R851|C4R851\_KOMPG:C4QZ38|C4QZ38\_KOMPG:C4R4X9|C4R4X9\_KOMPG:C4R554|C4R554\_KOMPG:C4QWK5|C4QWK5\_KOMPG:C4QZH7|C4QZH7\_KOMPG:C4R2H2|C4R2H2\_KOMPG:C4QZB3|C4QZB3\_KOMPG:C4R8J6|C4R8J6\_KOMPG:C4R313|C4R313\_KOMPG:C4R0Y6|C4R0Y6\_KOMPG:C4R8P3|C4R8P3\_KOMPG:C4R3R7|C4R3R7\_KOMPG:C4QZW3|C4QZW3\_KOMPG:C4R8A2|C4R8A2\_KOMPG:C4R2W3|C4R2W3\_KOMPG:C4QWS1|C4QWS1\_KOMPG:C4R875|C4R875\_KOMPG:C4R524|C4R524\_KOMPG:C4QW50|C4QW50\_KOMPG:C4R573|C4R573\_KOMPG:C4QXU5|C4QXU5\_KOMPG:C4R6U9|C4R6U9\_KOMPG:C4QVA1|C4QVA1\_KOMPG:C4R8X8|C4R8X8\_KOMPG:C4R6G0|C4R6G0\_KOMPG:C4R312|C4R312\_KOMPG:C4R8P5|C4R8P5\_KOMPG:C4R7K2|C4R7K2\_KOMPG:C4QY68|PEX8\_KOMPG |  |  | DB Search |
| TLVL | 26.01 | 444.2948 | 4 | -4 | 445.2992 | 28.31 | 9909 | 1.89e3 | 1 | 1 | C4R4V2|C4R4V2\_KOMPG:C4R8G4|C4R8G4\_KOMPG:C4R696|C4R696\_KOMPG:C4QWJ5|C4QWJ5\_KOMPG:C4QVC0|C4QVC0\_KOMPG:C4R856|C4R856\_KOMPG:C4R6Y0|C4R6Y0\_KOMPG:C4R3M2|C4R3M2\_KOMPG:C4QZU6|C4QZU6\_KOMPG:C4R857|C4R857\_KOMPG:C4QWI5|C4QWI5\_KOMPG:C4R4K2|C4R4K2\_KOMPG:C4R870|C4R870\_KOMPG:C4R963|C4R963\_KOMPG:C4R7N4|C4R7N4\_KOMPG:C4R2X4|C4R2X4\_KOMPG:C4R2G7|C4R2G7\_KOMPG:C4QV53|C4QV53\_KOMPG:C4R7L6|C4R7L6\_KOMPG:C4QVR8|C4QVR8\_KOMPG:Q6DNA2|ALG3\_KOMPG:C4QVA3|C4QVA3\_KOMPG:C4R0B4|C4R0B4\_KOMPG:C4R1A9|C4R1A9\_KOMPG:C4QZN2|C4QZN2\_KOMPG:C4R7W4|C4R7W4\_KOMPG:C4QYU4|C4QYU4\_KOMPG:C4QZ50|C4QZ50\_KOMPG:C4R4F1|C4R4F1\_KOMPG:C4QZ35|C4QZ35\_KOMPG:C4R8X5|C4R8X5\_KOMPG:C4QZE1|C4QZE1\_KOMPG:C4QUZ9|C4QUZ9\_KOMPG:C4QVQ2|C4QVQ2\_KOMPG:C4QZJ2|C4QZJ2\_KOMPG:C4QW18|C4QW18\_KOMPG:C4R112|C4R112\_KOMPG:C4R3Z7|C4R3Z7\_KOMPG:C4R4A6|C4R4A6\_KOMPG:C4R4J1|C4R4J1\_KOMPG:C4R306|C4R306\_KOMPG:C4R4U0|C4R4U0\_KOMPG:C4QW27|C4QW27\_KOMPG:C4R5L7|C4R5L7\_KOMPG:C4R288|C4R288\_KOMPG:C4QZT7|C4QZT7\_KOMPG:C4QVR3|C4QVR3\_KOMPG:C4QZB7|C4QZB7\_KOMPG:C4R4Z5|C4R4Z5\_KOMPG:C4QZA6|C4QZA6\_KOMPG:C4R254|C4R254\_KOMPG:C4R120|C4R120\_KOMPG:C4R7X9|BMT1\_KOMPG:C4R668|C4R668\_KOMPG:C4R1D9|C4R1D9\_KOMPG:C4QZS0|C4QZS0\_KOMPG:C4R6A0|C4R6A0\_KOMPG:C4R2C0|C4R2C0\_KOMPG:C4QXE9|CHO2\_KOMPG:C4R5Z8|C4R5Z8\_KOMPG:C4R6C8|C4R6C8\_KOMPG:C4QZR2|C4QZR2\_KOMPG:C4R8Y1|C4R8Y1\_KOMPG |  |  | DB Search |
| IGGISS | 26 | 532.2856 | 6 | -3.42 | 533.2898 | 10.97 | 4322 | 4.75e3 | 1 | 1 | C4R6R3|C4R6R3\_KOMPG |  |  | DB Search |
| IPTP | 25.97 | 426.2478 | 4 | -3.74 | 427.2524 | 12.64 | 4915 | 1.93e3 | 1 | 1 | C4R6M4|C4R6M4\_KOMPG:C4QXF7|C4QXF7\_KOMPG:C4QYV4|C4QYV4\_KOMPG:C4QYE2|C4QYE2\_KOMPG:C4R7J9|C4R7J9\_KOMPG:C4R2M3|C4R2M3\_KOMPG:C4QXQ9|C4QXQ9\_KOMPG:C4QX02|C4QX02\_KOMPG:C4R3B1|C4R3B1\_KOMPG:C4QZC5|C4QZC5\_KOMPG:C4R6Z8|C4R6Z8\_KOMPG:C4R6V1|C4R6V1\_KOMPG:C4R4X4|C4R4X4\_KOMPG:C4R3K6|C4R3K6\_KOMPG:C4QZT4|C4QZT4\_KOMPG:C4R3B0|C4R3B0\_KOMPG:C4R9C9|C4R9C9\_KOMPG:C4R735|C4R735\_KOMPG:C4R0Z5|C4R0Z5\_KOMPG:C4R364|C4R364\_KOMPG:C4R4L4|C4R4L4\_KOMPG:C4R2E4|C4R2E4\_KOMPG:C4R5R5|C4R5R5\_KOMPG:C4R3P6|C4R3P6\_KOMPG:C4R144|C4R144\_KOMPG:C4R420|C4R420\_KOMPG:C4R567|C4R567\_KOMPG:C4QXS6|C4QXS6\_KOMPG:C4R6A2|C4R6A2\_KOMPG:C4QZL6|C4QZL6\_KOMPG:C4R1Q2|C4R1Q2\_KOMPG:C4QZ06|BMT3\_KOMPG:C4R430|C4R430\_KOMPG:C4QXZ3|C4QXZ3\_KOMPG |  |  | DB Search |
| LPTP | 25.97 | 426.2478 | 4 | -3.74 | 427.2524 | 12.64 | 4915 | 1.93e3 | 1 | 1 | C4R233|C4R233\_KOMPG:C4R6A7|C4R6A7\_KOMPG:C4QWA7|C4QWA7\_KOMPG:C4QXT2|C4QXT2\_KOMPG:C4R3G6|C4R3G6\_KOMPG:C4R602|C4R602\_KOMPG:C4QYI2|C4QYI2\_KOMPG:C4QY76|C4QY76\_KOMPG:C4R811|C4R811\_KOMPG:C4QWE6|C4QWE6\_KOMPG:C4QZX9|C4QZX9\_KOMPG:C4QXB4|C4QXB4\_KOMPG:C4R202|C4R202\_KOMPG:C4QZM1|C4QZM1\_KOMPG:C4R1R3|C4R1R3\_KOMPG:C4R3I7|C4R3I7\_KOMPG:C4R510|C4R510\_KOMPG:C4R3R6|C4R3R6\_KOMPG:C4R3A3|C4R3A3\_KOMPG:C4R1A8|C4R1A8\_KOMPG:C4R898|C4R898\_KOMPG:C4R543|C4R543\_KOMPG:C4QW07|C4QW07\_KOMPG:C4R712|C4R712\_KOMPG:C4QXL2|C4QXL2\_KOMPG:C4R7N6|C4R7N6\_KOMPG:C4QUZ8|C4QUZ8\_KOMPG:C4QYE4|C4QYE4\_KOMPG:C4R613|DEGS\_KOMPG:C4R3T0|C4R3T0\_KOMPG:C4R6H0|C4R6H0\_KOMPG:C4R861|C4R861\_KOMPG:C4R6Y5|C4R6Y5\_KOMPG:C4QZW4|C4QZW4\_KOMPG:C4R724|C4R724\_KOMPG:C4R8S7|C4R8S7\_KOMPG:C4QZM4|C4QZM4\_KOMPG:C4R0L5|C4R0L5\_KOMPG:C4R6X2|C4R6X2\_KOMPG:C4QWV1|C4QWV1\_KOMPG:C4R556|C4R556\_KOMPG |  |  | DB Search |
| FPVG | 25.77 | 418.2216 | 4 | -7.2 | 419.2248 | 16.08 | 6373 | 0 | 0 | 0 | C4QV45|C4QV45\_KOMPG:C4R611|C4R611\_KOMPG:C4QW61|C4QW61\_KOMPG:C4R7K8|C4R7K8\_KOMPG:C4R2G0|C4R2G0\_KOMPG:C4R9F1|C4R9F1\_KOMPG:C4QZH8|C4QZH8\_KOMPG:C4R707|C4R707\_KOMPG:C4QYM6|C4QYM6\_KOMPG:C4QYF8|C4QYF8\_KOMPG:C4R7L0|C4R7L0\_KOMPG:C4R1T3|C4R1T3\_KOMPG:C4QYK8|C4QYK8\_KOMPG:C4R0M8|C4R0M8\_KOMPG:C4R738|C4R738\_KOMPG |  |  | DB Search |
| VKLDVLQT | 25.73 | 914.5436 | 8 | -8.3 | 915.5411 | 31.10 | 10647 | 0 | 0 | 0 | C4R6W9|C4R6W9\_KOMPG |  |  | DB Search |
| ISIE | 25.68 | 460.2533 | 4 | -2.19 | 461.2584 | 13.54 | 5303 | 1.35e4 | 1 | 1 | C4QWI9|C4QWI9\_KOMPG:C4QYG7|C4QYG7\_KOMPG:C4R7L8|C4R7L8\_KOMPG:C4R010|C4R010\_KOMPG:C4R4Z8|C4R4Z8\_KOMPG:C4R4A1|C4R4A1\_KOMPG:C4R2T0|C4R2T0\_KOMPG:C4R993|C4R993\_KOMPG:C4R3R9|C4R3R9\_KOMPG:C4R5S1|C4R5S1\_KOMPG:C4QWW6|C4QWW6\_KOMPG:C4QYN8|C4QYN8\_KOMPG:C4R1N5|C4R1N5\_KOMPG:C4QZE1|C4QZE1\_KOMPG:C4R0K3|C4R0K3\_KOMPG:C4QVP0|C4QVP0\_KOMPG:C4R453|C4R453\_KOMPG |  |  | DB Search |
| ISLE | 25.68 | 460.2533 | 4 | -2.19 | 461.2584 | 13.54 | 5303 | 1.35e4 | 1 | 1 | C4R8Y4|C4R8Y4\_KOMPG:C4QXL0|C4QXL0\_KOMPG:C4QVY3|C4QVY3\_KOMPG:C4R925|C4R925\_KOMPG:C4QZ90|C4QZ90\_KOMPG:C4R3P2|C4R3P2\_KOMPG:C4R1U2|C4R1U2\_KOMPG:C4QYR2|C4QYR2\_KOMPG:C4R5G8|C4R5G8\_KOMPG:C4R6C2|PEX1\_KOMPG:C4R5C4|C4R5C4\_KOMPG:C4R2H6|C4R2H6\_KOMPG:C4QY13|C4QY13\_KOMPG:C4QW93|C4QW93\_KOMPG:C4QXW6|C4QXW6\_KOMPG:C4R5P2|C4R5P2\_KOMPG:C4R129|C4R129\_KOMPG:C4R3N3|C4R3N3\_KOMPG:C4R1W8|C4R1W8\_KOMPG:C4R692|C4R692\_KOMPG:C4QYY0|C4QYY0\_KOMPG:C4QWP8|C4QWP8\_KOMPG:C4QXK3|C4QXK3\_KOMPG:C4R0D1|C4R0D1\_KOMPG:C4QWA5|C4QWA5\_KOMPG:C4R8K4|C4R8K4\_KOMPG:C4R1E3|C4R1E3\_KOMPG:C4R7A4|C4R7A4\_KOMPG:C4QYX7|C4QYX7\_KOMPG:C4QXF2|C4QXF2\_KOMPG:C4QZM0|C4QZM0\_KOMPG:C4R3G2|C4R3G2\_KOMPG:C4QYA3|C4QYA3\_KOMPG:C4R417|C4R417\_KOMPG |  |  | DB Search |
| LSIE | 25.68 | 460.2533 | 4 | -2.19 | 461.2584 | 13.54 | 5303 | 1.35e4 | 1 | 1 | C4QW02|C4QW02\_KOMPG:C4QVI7|C4QVI7\_KOMPG:C4R4N2|C4R4N2\_KOMPG:C4R446|C4R446\_KOMPG:C4R109|C4R109\_KOMPG:C4QZ28|C4QZ28\_KOMPG:Q9Y751|ATG26\_KOMPG:C4R396|C4R396\_KOMPG:C4R8Z7|C4R8Z7\_KOMPG:C4R5P0|C4R5P0\_KOMPG:C4QV74|C4QV74\_KOMPG:C4QZS3|C4QZS3\_KOMPG:C4R887|C4R887\_KOMPG:C4R120|C4R120\_KOMPG:C4QX55|C4QX55\_KOMPG:C4R5U6|C4R5U6\_KOMPG:C4R695|C4R695\_KOMPG:C4R7H2|C4R7H2\_KOMPG:C4R677|C4R677\_KOMPG:C4QVT3|C4QVT3\_KOMPG:C4QVH4|C4QVH4\_KOMPG |  |  | DB Search |
| LSLE | 25.68 | 460.2533 | 4 | -2.19 | 461.2584 | 13.54 | 5303 | 1.35e4 | 1 | 1 | C4R8I3|C4R8I3\_KOMPG:C4QV81|C4QV81\_KOMPG:C4QX82|C4QX82\_KOMPG:C4QWE1|C4QWE1\_KOMPG:C4R2V8|C4R2V8\_KOMPG:C4R324|C4R324\_KOMPG:C4R6G4|C4R6G4\_KOMPG:C4R0R9|C4R0R9\_KOMPG:C4R8N7|C4R8N7\_KOMPG:C4R0B0|C4R0B0\_KOMPG:C4R698|C4R698\_KOMPG:C4R1L3|C4R1L3\_KOMPG:C4R5J4|C4R5J4\_KOMPG:C4R061|C4R061\_KOMPG:C4R5W3|C4R5W3\_KOMPG:C4R0Q3|C4R0Q3\_KOMPG:C4QZV0|C4QZV0\_KOMPG:C4R4I4|C4R4I4\_KOMPG:C4R7J8|C4R7J8\_KOMPG:C4R2C5|C4R2C5\_KOMPG:C4R0G3|C4R0G3\_KOMPG:C4R804|C4R804\_KOMPG:C4QYT3|C4QYT3\_KOMPG:C4R6H0|C4R6H0\_KOMPG:C4R1F1|C4R1F1\_KOMPG:C4R6D7|C4R6D7\_KOMPG:C4R785|C4R785\_KOMPG:C4QYI8|C4QYI8\_KOMPG:C4QW41|C4QW41\_KOMPG:C4QYV2|C4QYV2\_KOMPG:C4QVM3|C4QVM3\_KOMPG:C4QVN0|C4QVN0\_KOMPG:C4R189|C4R189\_KOMPG:C4R341|C4R341\_KOMPG:C4QZ47|C4QZ47\_KOMPG:C4R2I4|C4R2I4\_KOMPG:C4QY61|C4QY61\_KOMPG:C4R9E3|C4R9E3\_KOMPG:C4R0M2|C4R0M2\_KOMPG:C4QZU3|C4QZU3\_KOMPG:C4R032|C4R032\_KOMPG:C4R6J0|C4R6J0\_KOMPG:C4QXG1|C4QXG1\_KOMPG:C4QWF2|C4QWF2\_KOMPG:C4R5H1|C4R5H1\_KOMPG:C4R3N9|C4R3N9\_KOMPG:C4R162|C4R162\_KOMPG:C4R517|C4R517\_KOMPG:C4R1F7|C4R1F7\_KOMPG:C4QZF3|C4QZF3\_KOMPG:C4R885|C4R885\_KOMPG:C4R869|C4R869\_KOMPG:C4QYA5|C4QYA5\_KOMPG:C4R6G5|C4R6G5\_KOMPG:C4R1I3|C4R1I3\_KOMPG:C4R126|C4R126\_KOMPG:C4R196|C4R196\_KOMPG:C4QX70|C4QX70\_KOMPG:C4QVB5|C4QVB5\_KOMPG:C4R240|C4R240\_KOMPG:C4QV64|C4QV64\_KOMPG:C4QXX1|C4QXX1\_KOMPG:C4QXB9|C4QXB9\_KOMPG:C4R385|C4R385\_KOMPG:C4R7Z4|C4R7Z4\_KOMPG:C4R0N3|C4R0N3\_KOMPG:C4R2W5|C4R2W5\_KOMPG:C4R5W9|C4R5W9\_KOMPG:C4R2C0|C4R2C0\_KOMPG:C4R176|C4R176\_KOMPG |  |  | DB Search |
| AFTP | 25.4 | 434.2165 | 4 | -5.48 | 435.2203 | 13.12 | 5200 | 6.05e3 | 1 | 1 | C4R6W4|C4R6W4\_KOMPG:C4R903|C4R903\_KOMPG:C4R6G8|C4R6G8\_KOMPG:C4QXD0|C4QXD0\_KOMPG:C4R5F5|C4R5F5\_KOMPG:C4R091|C4R091\_KOMPG:C4QXN4|C4QXN4\_KOMPG:C4R5U1|C4R5U1\_KOMPG:C4QXZ4|C4QXZ4\_KOMPG:C4R564|C4R564\_KOMPG:C4R6F6|C4R6F6\_KOMPG:C4R4U3|C4R4U3\_KOMPG:C4R1F1|C4R1F1\_KOMPG:C4R5E4|C4R5E4\_KOMPG:C4R901|C4R901\_KOMPG:C4QVQ8|C4QVQ8\_KOMPG:C4R8B9|C4R8B9\_KOMPG:C4R4G5|C4R4G5\_KOMPG:C4QZV1|C4QZV1\_KOMPG:C4R3S5|C4R3S5\_KOMPG |  |  | DB Search |
| SSSSG | 25.38 | 423.1601 | 5 | -3.71 | 424.1648 | 9.84 | 3798 | 5.82e2 | 1 | 1 | C4R4G0|C4R4G0\_KOMPG:C4R7Q7|C4R7Q7\_KOMPG:C4QZU8|C4QZU8\_KOMPG:C4R8D6|C4R8D6\_KOMPG:C4R8L5|C4R8L5\_KOMPG:C4QYA8|C4QYA8\_KOMPG:C4QVX2|C4QVX2\_KOMPG:C4R948|C4R948\_KOMPG:C4R222|C4R222\_KOMPG:C4QYA0|C4QYA0\_KOMPG:C4R4A3|C4R4A3\_KOMPG:C4R8R8|C4R8R8\_KOMPG:C4R726|C4R726\_KOMPG:C4R1X5|C4R1X5\_KOMPG:C4R108|C4R108\_KOMPG:C4R5G4|C4R5G4\_KOMPG:C4R4F5|C4R4F5\_KOMPG:C4R3Z2|C4R3Z2\_KOMPG:C4R3B1|C4R3B1\_KOMPG:C4R275|C4R275\_KOMPG:C4R7K1|C4R7K1\_KOMPG:C4R604|C4R604\_KOMPG:C4QZ96|C4QZ96\_KOMPG:C4R2Z5|C4R2Z5\_KOMPG:C4R4J9|C4R4J9\_KOMPG |  |  | DB Search |
| IGSI | 25.26 | 388.2322 | 4 | -3.81 | 389.237 | 9.87 | 3785 | 6.08e3 | 1 | 1 | C4R3B8|C4R3B8\_KOMPG:C4R086|C4R086\_KOMPG:C4R8N1|C4R8N1\_KOMPG:C4QZI1|C4QZI1\_KOMPG:C4R895|C4R895\_KOMPG:Q9Y751|ATG26\_KOMPG:C4R3N8|C4R3N8\_KOMPG:C4R4V3|C4R4V3\_KOMPG:C4QVW3|C4QVW3\_KOMPG:C4R0V3|C4R0V3\_KOMPG:C4R0S9|C4R0S9\_KOMPG:C4QZM1|C4QZM1\_KOMPG:C4QZT8|C4QZT8\_KOMPG:C4QW81|C4QW81\_KOMPG:C4QYJ9|C4QYJ9\_KOMPG:C4QXQ6|C4QXQ6\_KOMPG:C4QWS0|C4QWS0\_KOMPG:C4R6L7|C4R6L7\_KOMPG:C4R956|C4R956\_KOMPG:C4R726|C4R726\_KOMPG:C4R647|C4R647\_KOMPG:C4QWB6|C4QWB6\_KOMPG:C4QWP8|C4QWP8\_KOMPG:C4R4T2|C4R4T2\_KOMPG:C4R5M2|C4R5M2\_KOMPG:C4R3S3|C4R3S3\_KOMPG:C4QYA2|C4QYA2\_KOMPG:C4R8G1|C4R8G1\_KOMPG:C4R785|C4R785\_KOMPG:C4QYZ8|C4QYZ8\_KOMPG:C4R8P6|C4R8P6\_KOMPG:C4R8T3|C4R8T3\_KOMPG:C4QXZ9|C4QXZ9\_KOMPG:C4R666|C4R666\_KOMPG:C4R813|C4R813\_KOMPG:C4R5X9|C4R5X9\_KOMPG:C4QYE3|C4QYE3\_KOMPG:C4R8P5|C4R8P5\_KOMPG |  |  | DB Search |
| IGSL | 25.26 | 388.2322 | 4 | -3.81 | 389.237 | 9.87 | 3785 | 6.08e3 | 1 | 1 | C4R0I7|C4R0I7\_KOMPG:C4R5S6|C4R5S6\_KOMPG:C4QWC8|C4QWC8\_KOMPG:C4R5B3|C4R5B3\_KOMPG:C4QZ37|C4QZ37\_KOMPG:C4R515|C4R515\_KOMPG:C4QWM0|C4QWM0\_KOMPG:C4QZK1|C4QZK1\_KOMPG:C4R4G5|C4R4G5\_KOMPG:C4R7R3|C4R7R3\_KOMPG:C4QZK7|C4QZK7\_KOMPG:C4R663|C4R663\_KOMPG:C4QVP5|C4QVP5\_KOMPG:C4R5M0|C4R5M0\_KOMPG:C4R6F4|C4R6F4\_KOMPG:C4QX47|C4QX47\_KOMPG:C4QZF5|C4QZF5\_KOMPG:C4R354|C4R354\_KOMPG:C4QY80|C4QY80\_KOMPG:C4QVM3|C4QVM3\_KOMPG:C4QW00|C4QW00\_KOMPG:C4R366|C4R366\_KOMPG:C4QV97|C4QV97\_KOMPG:C4R5A7|C4R5A7\_KOMPG:C4QYK7|C4QYK7\_KOMPG:C4QZW5|C4QZW5\_KOMPG:C4R5F3|C4R5F3\_KOMPG:C4QX50|C4QX50\_KOMPG:C4R3F4|C4R3F4\_KOMPG:C4R6F2|C4R6F2\_KOMPG:C4R1L9|C4R1L9\_KOMPG:C4QZW9|C4QZW9\_KOMPG:C4QWH1|C4QWH1\_KOMPG:C4R0U5|C4R0U5\_KOMPG:C4QYQ8|C4QYQ8\_KOMPG:C4R6Q6|C4R6Q6\_KOMPG:C4R5H4|C4R5H4\_KOMPG:C4R1M0|C4R1M0\_KOMPG:C4R1U8|C4R1U8\_KOMPG:C4QXC1|C4QXC1\_KOMPG:C4R068|C4R068\_KOMPG:C4R320|C4R320\_KOMPG:C4R2K1|C4R2K1\_KOMPG:C4R8Y5|C4R8Y5\_KOMPG:C4R1A0|C4R1A0\_KOMPG:C4R2V2|C4R2V2\_KOMPG:C4R2S6|C4R2S6\_KOMPG:C4QYC4|C4QYC4\_KOMPG:C4R4E7|C4R4E7\_KOMPG:C4QXF2|C4QXF2\_KOMPG:C4QXB9|C4QXB9\_KOMPG:C4QXE2|C4QXE2\_KOMPG:C4R4Q5|C4R4Q5\_KOMPG:C4R697|C4R697\_KOMPG |  |  | DB Search |
| LGSI | 25.26 | 388.2322 | 4 | -3.81 | 389.237 | 9.87 | 3785 | 6.08e3 | 1 | 1 | C4QZH4|C4QZH4\_KOMPG:C4QVN9|C4QVN9\_KOMPG:C4QYB2|C4QYB2\_KOMPG:C4QWG8|C4QWG8\_KOMPG:C4R0V4|C4R0V4\_KOMPG:C4QZD1|C4QZD1\_KOMPG:C4QWF9|C4QWF9\_KOMPG:C4QYY4|C4QYY4\_KOMPG:C4R4G8|C4R4G8\_KOMPG:C4QVH6|C4QVH6\_KOMPG:C4R0U1|C4R0U1\_KOMPG:C4R7Y5|C4R7Y5\_KOMPG:C4R1U9|C4R1U9\_KOMPG:C4QWX5|C4QWX5\_KOMPG:C4R5S9|C4R5S9\_KOMPG:C4R804|C4R804\_KOMPG:C4QYT3|C4QYT3\_KOMPG:C4QYM6|C4QYM6\_KOMPG:C4R4J8|C4R4J8\_KOMPG:C4R2L4|C4R2L4\_KOMPG:C4R113|C4R113\_KOMPG:C4QXQ5|C4QXQ5\_KOMPG:C4QYU6|C4QYU6\_KOMPG:C4QZN7|C4QZN7\_KOMPG:C4R104|C4R104\_KOMPG:C4QYM5|C4QYM5\_KOMPG:C4R5I0|C4R5I0\_KOMPG:C4R675|C4R675\_KOMPG:C4QWX7|C4QWX7\_KOMPG:C4R3N1|C4R3N1\_KOMPG:C4QZ03|C4QZ03\_KOMPG:C4R2M2|C4R2M2\_KOMPG:C4QZA9|C4QZA9\_KOMPG:C4QZH7|C4QZH7\_KOMPG:C4QZ69|C4QZ69\_KOMPG:C4R238|C4R238\_KOMPG:C4R1J6|C4R1J6\_KOMPG:C4R1R4|C4R1R4\_KOMPG:C4R162|C4R162\_KOMPG:C4R4A2|C4R4A2\_KOMPG:C4QXV3|C4QXV3\_KOMPG:C4R8Y8|C4R8Y8\_KOMPG:C4QZY1|C4QZY1\_KOMPG:C4R4Y0|C4R4Y0\_KOMPG:C4R6S5|C4R6S5\_KOMPG:C4R9A9|C4R9A9\_KOMPG:C4R6M8|C4R6M8\_KOMPG:C4R4A4|C4R4A4\_KOMPG:C4R2A9|C4R2A9\_KOMPG:C4QV24|C4QV24\_KOMPG:C4R855|C4R855\_KOMPG:C4QXX3|C4QXX3\_KOMPG:C4QZX4|C4QZX4\_KOMPG:C4QV23|C4QV23\_KOMPG |  |  | DB Search |
| LGSL | 25.26 | 388.2322 | 4 | -3.81 | 389.237 | 9.87 | 3785 | 6.08e3 | 1 | 1 | C4R5N4|C4R5N4\_KOMPG:C4QWE1|C4QWE1\_KOMPG:C4R5R7|C4R5R7\_KOMPG:C4R8L6|C4R8L6\_KOMPG:C4QV93|C4QV93\_KOMPG:C4R7F2|C4R7F2\_KOMPG:C4QWI8|C4QWI8\_KOMPG:C4R5C0|C4R5C0\_KOMPG:C4R253|C4R253\_KOMPG:C4R343|C4R343\_KOMPG:C4R9F0|C4R9F0\_KOMPG:C4R2R7|C4R2R7\_KOMPG:C4QVE2|C4QVE2\_KOMPG:C4R0S8|C4R0S8\_KOMPG:C4R5N2|C4R5N2\_KOMPG:C4QZZ6|C4QZZ6\_KOMPG:C4R568|C4R568\_KOMPG:C4R6M3|C4R6M3\_KOMPG:C4R4L7|C4R4L7\_KOMPG:C4R1D0|C4R1D0\_KOMPG:C4R888|C4R888\_KOMPG:C4R110|C4R110\_KOMPG:C4R383|C4R383\_KOMPG:C4QWA3|C4QWA3\_KOMPG:C4R3N4|C4R3N4\_KOMPG:C4R251|C4R251\_KOMPG:C4R676|C4R676\_KOMPG:C4R9C5|C4R9C5\_KOMPG:C4QVX4|C4QVX4\_KOMPG:C4R0B7|C4R0B7\_KOMPG:C4QZN5|C4QZN5\_KOMPG:C4R124|C4R124\_KOMPG:C4QZI9|C4QZI9\_KOMPG:C4QV32|C4QV32\_KOMPG:C4R2G6|C4R2G6\_KOMPG:C4R2E9|C4R2E9\_KOMPG:C4R2C5|C4R2C5\_KOMPG:C4QXU2|C4QXU2\_KOMPG:C4QWS6|C4QWS6\_KOMPG:C4R7B7|C4R7B7\_KOMPG:C4R3Q3|C4R3Q3\_KOMPG:C4R4C7|C4R4C7\_KOMPG:C4QWS7|C4QWS7\_KOMPG:C4R7J5|C4R7J5\_KOMPG:C4R6F6|C4R6F6\_KOMPG:C4QWU0|C4QWU0\_KOMPG:C4R571|C4R571\_KOMPG:C4R608|C4R608\_KOMPG:C4QV52|C4QV52\_KOMPG:C4R770|C4R770\_KOMPG:C4R144|C4R144\_KOMPG:C4R7S0|C4R7S0\_KOMPG:C4R1K4|C4R1K4\_KOMPG:C4QZ95|C4QZ95\_KOMPG:C4R6T2|C4R6T2\_KOMPG:C4R154|C4R154\_KOMPG:C4QWL4|C4QWL4\_KOMPG:C4R3Y4|C4R3Y4\_KOMPG:C4QUZ4|C4QUZ4\_KOMPG:C4R3C2|C4R3C2\_KOMPG:C4R1J8|C4R1J8\_KOMPG:C4R792|C4R792\_KOMPG:C4R5U2|C4R5U2\_KOMPG:C4R7T1|C4R7T1\_KOMPG:C4QZ07|C4QZ07\_KOMPG:C4QVD4|C4QVD4\_KOMPG:C4QV66|C4QV66\_KOMPG:C4R2N7|C4R2N7\_KOMPG:C4R2Z7|C4R2Z7\_KOMPG:C4QYP8|C4QYP8\_KOMPG:C4QXB8|C4QXB8\_KOMPG:C4R2D1|C4R2D1\_KOMPG:C4QZX0|C4QZX0\_KOMPG:C4R927|C4R927\_KOMPG:C4R123|C4R123\_KOMPG:C4QXA5|PFKA2\_KOMPG:C4R1N8|C4R1N8\_KOMPG:C4QZB6|C4QZB6\_KOMPG:C4R6W1|C4R6W1\_KOMPG:C4R524|C4R524\_KOMPG:C4R5L0|C4R5L0\_KOMPG:C4R2S2|C4R2S2\_KOMPG:C4R3V2|C4R3V2\_KOMPG:C4QZ04|C4QZ04\_KOMPG:C4R3B6|C4R3B6\_KOMPG |  |  | DB Search |
| RGFI | 25.24 | 491.2856 | 4 | -0.24 | 492.2915 | 14.92 | 5891 | 9.54e2 | 1 | 1 | C4QX83|C4QX83\_KOMPG:C4QY61|C4QY61\_KOMPG:C4R7J0|C4R7J0\_KOMPG:C4R5P0|C4R5P0\_KOMPG:C4R2M8|C4R2M8\_KOMPG:C4R4T5|C4R4T5\_KOMPG:C4R1W7|C4R1W7\_KOMPG:C4QWD1|C4QWD1\_KOMPG:C4R901|C4R901\_KOMPG:C4R3F9|C4R3F9\_KOMPG:C4QY37|C4QY37\_KOMPG:C4R8U8|C4R8U8\_KOMPG:C4R1L3|C4R1L3\_KOMPG:C4R1D9|C4R1D9\_KOMPG:C4R6E2|C4R6E2\_KOMPG |  |  | DB Search |
| RGFL | 25.24 | 491.2856 | 4 | -0.24 | 492.2915 | 14.92 | 5891 | 9.54e2 | 1 | 1 | C4R1I6|C4R1I6\_KOMPG:C4R979|C4R979\_KOMPG:C4R4Q0|C4R4Q0\_KOMPG:C4QYY8|C4QYY8\_KOMPG:C4QZ17|C4QZ17\_KOMPG:C4QWQ7|C4QWQ7\_KOMPG:C4R373|C4R373\_KOMPG:C4QY28|C4QY28\_KOMPG:C4QWC7|C4QWC7\_KOMPG:C4R7B3|C4R7B3\_KOMPG:C4QZ69|C4QZ69\_KOMPG:C4R8L2|C4R8L2\_KOMPG:C4R5I5|C4R5I5\_KOMPG:C4QZF1|C4QZF1\_KOMPG:C4QUZ0|C4QUZ0\_KOMPG:C4R885|C4R885\_KOMPG:C4R760|C4R760\_KOMPG:C4R697|C4R697\_KOMPG:C4R2F3|C4R2F3\_KOMPG |  |  | DB Search |
| IPQQP | 25.19 | 581.3173 | 5 | -2.43 | 582.3217 | 8.89 | 3457 | 0 | 0 | 0 | C4R8P7|C4R8P7\_KOMPG |  |  | DB Search |
| LPQQP | 25.19 | 581.3173 | 5 | -2.43 | 582.3217 | 8.89 | 3457 | 0 | 0 | 0 | C4QXL2|C4QXL2\_KOMPG:C4QW44|C4QW44\_KOMPG:C4QWG1|C4QWG1\_KOMPG:C4R0J2|C4R0J2\_KOMPG |  |  | DB Search |
| IDLLGGA | 25.17 | 657.3697 | 7 | -6.66 | 658.371 | 26.90 | 9524 | 7.97e2 | 1 | 1 | C4R3X3|C4R3X3\_KOMPG |  |  | DB Search |
| AIGI | 25.12 | 372.2372 | 4 | -5.1 | 373.2417 | 21.84 | 8058 | 0 | 0 | 0 | C4QWN3|C4QWN3\_KOMPG:C4R3Z4|C4R3Z4\_KOMPG:C4QWF0|C4QWF0\_KOMPG:C4QWX8|C4QWX8\_KOMPG:C4R6B7|C4R6B7\_KOMPG:C4QW29|C4QW29\_KOMPG:C4R2Q3|C4R2Q3\_KOMPG:C4R5Q0|C4R5Q0\_KOMPG:C4R5P8|C4R5P8\_KOMPG:C4QZI4|C4QZI4\_KOMPG:C4R5P1|C4R5P1\_KOMPG:C4R6Z6|C4R6Z6\_KOMPG:C4R5E4|C4R5E4\_KOMPG:C4R4S9|C4R4S9\_KOMPG:C4R880|C4R880\_KOMPG:C4R1C8|C4R1C8\_KOMPG:C4R2A7|C4R2A7\_KOMPG:C4R8E1|C4R8E1\_KOMPG:C4R199|C4R199\_KOMPG:C4R1L5|C4R1L5\_KOMPG:C4QXB2|C4QXB2\_KOMPG:C4QZP6|C4QZP6\_KOMPG:C4R7H0|C4R7H0\_KOMPG:C4QXF6|C4QXF6\_KOMPG:C4R5J2|C4R5J2\_KOMPG:C4R362|C4R362\_KOMPG:C4QYT8|C4QYT8\_KOMPG:C4R7M1|C4R7M1\_KOMPG:C4QV24|C4QV24\_KOMPG:C4QYA3|C4QYA3\_KOMPG:C4R1G0|C4R1G0\_KOMPG:C4R117|C4R117\_KOMPG:C4R9A6|C4R9A6\_KOMPG:C4R424|C4R424\_KOMPG |  |  | DB Search |
| AIGL | 25.12 | 372.2372 | 4 | -5.1 | 373.2417 | 21.84 | 8058 | 0 | 0 | 0 | C4QZL2|C4QZL2\_KOMPG:C4R4H2|C4R4H2\_KOMPG:C4R5V6|C4R5V6\_KOMPG:P52710|CBPY\_KOMPG:C4QVZ4|C4QVZ4\_KOMPG:C4R4I5|C4R4I5\_KOMPG:C4R7C2|C4R7C2\_KOMPG:C4R4L7|C4R4L7\_KOMPG:C4R846|C4R846\_KOMPG:C4R221|C4R221\_KOMPG:C4R588|C4R588\_KOMPG:C4QVW1|C4QVW1\_KOMPG:C4QW14|C4QW14\_KOMPG:C4R8M0|C4R8M0\_KOMPG:C4R8L3|C4R8L3\_KOMPG:C4R1F4|C4R1F4\_KOMPG:C4QXM8|C4QXM8\_KOMPG:C4R075|C4R075\_KOMPG:C4R8A5|C4R8A5\_KOMPG:C4QXX7|C4QXX7\_KOMPG:C4R0E2|C4R0E2\_KOMPG:C4R312|C4R312\_KOMPG:C4R539|C4R539\_KOMPG:C4R6G8|C4R6G8\_KOMPG:C4R166|C4R166\_KOMPG:C4QYT7|C4QYT7\_KOMPG:C4QX89|C4QX89\_KOMPG:C4QYU4|C4QYU4\_KOMPG:C4QZ07|C4QZ07\_KOMPG:C4R099|C4R099\_KOMPG:C4R7D2|C4R7D2\_KOMPG:C4R4K0|C4R4K0\_KOMPG:C4R6D1|C4R6D1\_KOMPG:C4R738|C4R738\_KOMPG:C4R518|C4R518\_KOMPG:C4R806|C4R806\_KOMPG:C4R8Q5|C4R8Q5\_KOMPG:C4R1S1|C4R1S1\_KOMPG:C4R5Q6|C4R5Q6\_KOMPG:C4R0E7|C4R0E7\_KOMPG:C4QVT9|C4QVT9\_KOMPG:C4R720|C4R720\_KOMPG:C4R931|C4R931\_KOMPG:C4R6Y4|C4R6Y4\_KOMPG:C4R494|C4R494\_KOMPG:C4R3G2|C4R3G2\_KOMPG:C4QVV9|C4QVV9\_KOMPG:C4R1V8|C4R1V8\_KOMPG:C4R7X3|C4R7X3\_KOMPG:C4R1H4|C4R1H4\_KOMPG:C4R1Z4|C4R1Z4\_KOMPG:C4R8E3|C4R8E3\_KOMPG:C4QWN2|C4QWN2\_KOMPG:C4R6Y8|C4R6Y8\_KOMPG |  |  | DB Search |
| ALGI | 25.12 | 372.2372 | 4 | -5.1 | 373.2417 | 21.84 | 8058 | 0 | 0 | 0 | C4R0R3|C4R0R3\_KOMPG:C4R3W3|C4R3W3\_KOMPG:C4R211|C4R211\_KOMPG:C4R675|C4R675\_KOMPG:C4R7N8|C4R7N8\_KOMPG:Q9Y751|ATG26\_KOMPG:C4QZZ3|C4QZZ3\_KOMPG:C4R6C5|C4R6C5\_KOMPG:C4R6G9|C4R6G9\_KOMPG:C4R026|C4R026\_KOMPG:C4R614|C4R614\_KOMPG:C4R747|C4R747\_KOMPG:C4R0Q8|C4R0Q8\_KOMPG:C4R0U5|C4R0U5\_KOMPG:C4R382|C4R382\_KOMPG:C4QVE8|C4QVE8\_KOMPG:C4R0U6|C4R0U6\_KOMPG:C4R431|C4R431\_KOMPG:C4R2K7|C4R2K7\_KOMPG:C4R7Y5|C4R7Y5\_KOMPG:C4QWU5|C4QWU5\_KOMPG:C4QZA0|C4QZA0\_KOMPG:C4R5S0|C4R5S0\_KOMPG:C4R0L6|C4R0L6\_KOMPG:C4R1Y0|C4R1Y0\_KOMPG:C4R3B1|C4R3B1\_KOMPG:C4R6M0|C4R6M0\_KOMPG:C4QYE4|C4QYE4\_KOMPG:C4R4Q2|C4R4Q2\_KOMPG:C4R6F6|C4R6F6\_KOMPG:C4R2D8|C4R2D8\_KOMPG:C4R1F0|C4R1F0\_KOMPG:C4R4A4|C4R4A4\_KOMPG:C4QYS6|MTNA\_KOMPG:C4QX51|C4QX51\_KOMPG:C4QXS1|C4QXS1\_KOMPG:C4QZQ7|C4QZQ7\_KOMPG:C4R5N3|C4R5N3\_KOMPG:C4R1C7|C4R1C7\_KOMPG:C4R7X7|C4R7X7\_KOMPG:C4QVH1|C4QVH1\_KOMPG:C4QW98|C4QW98\_KOMPG:C4QZ04|C4QZ04\_KOMPG:C4QWL9|C4QWL9\_KOMPG |  |  | DB Search |
| ALGL | 25.12 | 372.2372 | 4 | -5.1 | 373.2417 | 21.84 | 8058 | 0 | 0 | 0 | C4R295|C4R295\_KOMPG:C4QZU8|C4QZU8\_KOMPG:C4R8R9|C4R8R9\_KOMPG:C4QXS5|C4QXS5\_KOMPG:C4R4V3|C4R4V3\_KOMPG:C4R5T4|C4R5T4\_KOMPG:C4QZD1|C4QZD1\_KOMPG:C4R436|C4R436\_KOMPG:C4QVH6|C4QVH6\_KOMPG:C4R8R3|C4R8R3\_KOMPG:C4R0J1|C4R0J1\_KOMPG:C4R0S8|C4R0S8\_KOMPG:C4R5X2|C4R5X2\_KOMPG:C4QX22|C4QX22\_KOMPG:C4QUZ3|C4QUZ3\_KOMPG:C4R461|C4R461\_KOMPG:C4R929|C4R929\_KOMPG:C4R912|C4R912\_KOMPG:C4R007|C4R007\_KOMPG:C4R767|C4R767\_KOMPG:C4QW86|C4QW86\_KOMPG:C4QYS8|C4QYS8\_KOMPG:C4QV33|C4QV33\_KOMPG:C4R500|C4R500\_KOMPG:C4R4A5|C4R4A5\_KOMPG:C4QX47|C4QX47\_KOMPG:C4QV53|C4QV53\_KOMPG:C4QYW1|C4QYW1\_KOMPG:C4R778|C4R778\_KOMPG:C4R4W5|C4R4W5\_KOMPG:C4QZS4|C4QZS4\_KOMPG:C4QXR0|C4QXR0\_KOMPG:C4R0I3|C4R0I3\_KOMPG:C4R701|C4R701\_KOMPG:C4QVT0|C4QVT0\_KOMPG:C4R2F8|C4R2F8\_KOMPG:C4R4E5|C4R4E5\_KOMPG:C4R6A8|C4R6A8\_KOMPG:C4QZK9|C4QZK9\_KOMPG:C4R044|C4R044\_KOMPG:C4QZV5|C4QZV5\_KOMPG:C4QZR7|C4QZR7\_KOMPG:C4R0I8|C4R0I8\_KOMPG:C4R1K2|C4R1K2\_KOMPG:Q9P4C8|SAR1\_KOMPG:C4R7H6|C4R7H6\_KOMPG:C4R7G9|C4R7G9\_KOMPG:C4QW55|C4QW55\_KOMPG:C4R7X0|C4R7X0\_KOMPG:C4R703|C4R703\_KOMPG:C4R325|C4R325\_KOMPG:C4R8W5|C4R8W5\_KOMPG:C4QYY3|C4QYY3\_KOMPG:C4R224|C4R224\_KOMPG:C4QXA2|C4QXA2\_KOMPG:C4QYX5|C4QYX5\_KOMPG:C4R1D5|C4R1D5\_KOMPG:C4R668|C4R668\_KOMPG:C4R329|C4R329\_KOMPG:C4QVW6|C4QVW6\_KOMPG:C4QWY3|C4QWY3\_KOMPG |  |  | DB Search |
| EIIR | 25.05 | 529.3224 | 4 | -4.56 | 530.3259 | 17.34 | 6855 | 6.68e3 | 1 | 1 | C4R6M4|C4R6M4\_KOMPG:C4QZP8|C4QZP8\_KOMPG:C4QX36|C4QX36\_KOMPG:C4R2I4|C4R2I4\_KOMPG:C4R3J0|C4R3J0\_KOMPG:C4QWF8|C4QWF8\_KOMPG:C4R2G6|C4R2G6\_KOMPG:C4R1D2|C4R1D2\_KOMPG:C4R4W9|C4R4W9\_KOMPG:C4R324|C4R324\_KOMPG:C4QVL7|C4QVL7\_KOMPG:C4R2H0|C4R2H0\_KOMPG:C4R730|C4R730\_KOMPG:C4R2E1|C4R2E1\_KOMPG:C4R9F0|C4R9F0\_KOMPG:C4R404|C4R404\_KOMPG:C4R075|C4R075\_KOMPG:C4R4Q7|C4R4Q7\_KOMPG:C4QW93|C4QW93\_KOMPG:C4R6B2|C4R6B2\_KOMPG:C4QVC2|C4QVC2\_KOMPG:C4R5A0|C4R5A0\_KOMPG |  |  | DB Search |
| EILR | 25.05 | 529.3224 | 4 | -4.56 | 530.3259 | 17.34 | 6855 | 6.68e3 | 1 | 1 | C4R217|C4R217\_KOMPG:C4R392|C4R392\_KOMPG:C4R413|C4R413\_KOMPG:C4R214|C4R214\_KOMPG:C4R0V4|C4R0V4\_KOMPG:C4R1G8|C4R1G8\_KOMPG:C4R7T8|C4R7T8\_KOMPG:C4R0B5|SDHF2\_KOMPG:C4R6C2|PEX1\_KOMPG:C4QZZ8|C4QZZ8\_KOMPG:C4R9B4|C4R9B4\_KOMPG:C4R4Q8|C4R4Q8\_KOMPG:C4R4D9|C4R4D9\_KOMPG:C4R5M5|C4R5M5\_KOMPG:C4R2J4|C4R2J4\_KOMPG:C4QVD3|C4QVD3\_KOMPG:C4R499|C4R499\_KOMPG:C4R4B6|C4R4B6\_KOMPG:C4R6F6|C4R6F6\_KOMPG:C4R6H0|C4R6H0\_KOMPG:C4R1Z5|C4R1Z5\_KOMPG:C4R046|C4R046\_KOMPG:C4R9A0|C4R9A0\_KOMPG:C4QYF9|C4QYF9\_KOMPG:C4R0L0|C4R0L0\_KOMPG:C4R154|C4R154\_KOMPG:C4R908|C4R908\_KOMPG:C4QX83|C4QX83\_KOMPG:C4QZ59|C4QZ59\_KOMPG:C4QWV3|C4QWV3\_KOMPG:C4QX32|C4QX32\_KOMPG:C4R3R5|C4R3R5\_KOMPG:C4QXA9|C4QXA9\_KOMPG:C4R7I3|C4R7I3\_KOMPG:C4R0L7|C4R0L7\_KOMPG:C4R0X6|C4R0X6\_KOMPG:C4QZ32|C4QZ32\_KOMPG:C4R492|OCA5\_KOMPG:C4R516|C4R516\_KOMPG:C4R1I3|C4R1I3\_KOMPG:C4QZY1|C4QZY1\_KOMPG:C4R0D0|C4R0D0\_KOMPG:C4QYX7|C4QYX7\_KOMPG:C4R8F9|C4R8F9\_KOMPG:C4R7V9|C4R7V9\_KOMPG:C4R493|C4R493\_KOMPG:C4QXS6|C4QXS6\_KOMPG:C4R7Z4|C4R7Z4\_KOMPG:C4R677|C4R677\_KOMPG:C4R462|C4R462\_KOMPG:C4R417|C4R417\_KOMPG:C4R1W5|C4R1W5\_KOMPG |  |  | DB Search |
| ELIR | 25.05 | 529.3224 | 4 | -4.56 | 530.3259 | 17.34 | 6855 | 6.68e3 | 1 | 1 | C4R0N9|C4R0N9\_KOMPG:C4R3D7|C4R3D7\_KOMPG:C4QVT8|C4QVT8\_KOMPG:C4R5E3|C4R5E3\_KOMPG:C4R4V0|C4R4V0\_KOMPG:C4R8K0|C4R8K0\_KOMPG:C4R6C5|C4R6C5\_KOMPG:C4R8H1|C4R8H1\_KOMPG:C4R1R3|C4R1R3\_KOMPG:C4QWF2|C4QWF2\_KOMPG:C4R5V2|C4R5V2\_KOMPG:C4QV72|C4QV72\_KOMPG:C4R7F9|C4R7F9\_KOMPG:C4R8E0|C4R8E0\_KOMPG:C4QZ92|C4QZ92\_KOMPG:C4QXN8|C4QXN8\_KOMPG:C4QWY2|C4QWY2\_KOMPG:C4R0R1|C4R0R1\_KOMPG:C4R569|C4R569\_KOMPG:C4R4G1|C4R4G1\_KOMPG:C4R0T1|C4R0T1\_KOMPG:C4QXX4|C4QXX4\_KOMPG:C4R542|C4R542\_KOMPG:C4R572|C4R572\_KOMPG:C4QVX4|C4QVX4\_KOMPG:C4R0I5|C4R0I5\_KOMPG:C4R7Y5|C4R7Y5\_KOMPG:C4R5G4|C4R5G4\_KOMPG:C4QYU3|C4QYU3\_KOMPG:C4QZB5|C4QZB5\_KOMPG:C4QZP0|C4QZP0\_KOMPG:C4QYC4|C4QYC4\_KOMPG:C4R0S0|C4R0S0\_KOMPG:C4R6U9|C4R6U9\_KOMPG:C4QVM6|C4QVM6\_KOMPG:C4R5W9|C4R5W9\_KOMPG:C4QXE9|CHO2\_KOMPG:C4R4X6|C4R4X6\_KOMPG |  |  | DB Search |
| ELLR | 25.05 | 529.3224 | 4 | -4.56 | 530.3259 | 17.34 | 6855 | 6.68e3 | 1 | 1 | C4R8T7|C4R8T7\_KOMPG:C4R006|C4R006\_KOMPG:C4R4G6|C4R4G6\_KOMPG:C4R5P0|C4R5P0\_KOMPG:C4R0P7|C4R0P7\_KOMPG:C4QWJ3|C4QWJ3\_KOMPG:C4R1E7|C4R1E7\_KOMPG:C4QZX7|C4QZX7\_KOMPG:C4R4E9|C4R4E9\_KOMPG:C4QZU5|C4QZU5\_KOMPG:C4R750|C4R750\_KOMPG:C4R363|C4R363\_KOMPG:C4R150|C4R150\_KOMPG:C4R3S3|C4R3S3\_KOMPG:C4R849|C4R849\_KOMPG:C4QXU5|C4QXU5\_KOMPG:C4QYK8|C4QYK8\_KOMPG:C4R364|C4R364\_KOMPG:C4R2E4|C4R2E4\_KOMPG:C4QZS4|C4QZS4\_KOMPG:C4R810|C4R810\_KOMPG:C4R785|C4R785\_KOMPG:C4QYN9|C4QYN9\_KOMPG:C4R8S7|C4R8S7\_KOMPG:C4QX95|C4QX95\_KOMPG:C4QW33|C4QW33\_KOMPG:C4R7Z7|C4R7Z7\_KOMPG:C4R5W5|C4R5W5\_KOMPG:C4QWZ3|C4QWZ3\_KOMPG:C4QY17|DRE2\_KOMPG:C4QZ65|C4QZ65\_KOMPG:C4QVX9|C4QVX9\_KOMPG:C4R7V2|C4R7V2\_KOMPG:C4QVN1|C4QVN1\_KOMPG:C4R5C7|C4R5C7\_KOMPG:C4R7J7|C4R7J7\_KOMPG:C4QV73|C4QV73\_KOMPG:C4R7G3|C4R7G3\_KOMPG:C4R4S3|C4R4S3\_KOMPG:C4R328|C4R328\_KOMPG:C4QY53|C4QY53\_KOMPG:C4R276|C4R276\_KOMPG:C4R0H6|C4R0H6\_KOMPG:C4R7E7|C4R7E7\_KOMPG:C4QZE7|C4QZE7\_KOMPG:C4R0Q0|C4R0Q0\_KOMPG:C4R951|C4R951\_KOMPG:C4R325|C4R325\_KOMPG:C4R8G9|C4R8G9\_KOMPG:C4R4M0|C4R4M0\_KOMPG:C4R4J6|C4R4J6\_KOMPG:C4R8K4|C4R8K4\_KOMPG:C4QWU8|C4QWU8\_KOMPG:C4R6I2|C4R6I2\_KOMPG:C4QXV5|C4QXV5\_KOMPG:C4QXU6|C4QXU6\_KOMPG:C4QWD1|C4QWD1\_KOMPG:C4QW16|C4QW16\_KOMPG:C4R6X3|C4R6X3\_KOMPG:C4QXI1|C4QXI1\_KOMPG:C4R5Z8|C4R5Z8\_KOMPG |  |  | DB Search |
| IGGGA | 25.05 | 373.1961 | 5 | 1.54 | 374.203 | 3.00 | 1292 | 0 | 0 | 0 | C4QYI6|C4QYI6\_KOMPG:C4R4Z1|C4R4Z1\_KOMPG:C4QWB0|C4QWB0\_KOMPG |  |  | DB Search |
| LGGGA | 25.05 | 373.1961 | 5 | 1.54 | 374.203 | 3.00 | 1292 | 0 | 0 | 0 | C4R5H7|C4R5H7\_KOMPG:C4QYT3|C4QYT3\_KOMPG:C4R2E8|C4R2E8\_KOMPG |  |  | DB Search |
| PVVP | 25.03 | 410.2529 | 4 | 0.6 | 411.2594 | 13.91 | 5530 | 1.28e4 | 1 | 1 | C4R7Q7|C4R7Q7\_KOMPG:C4R7H9|C4R7H9\_KOMPG:C4QVL1|C4QVL1\_KOMPG:C4R2Q9|C4R2Q9\_KOMPG:C4R1E2|C4R1E2\_KOMPG:C4QXG4|C4QXG4\_KOMPG:C4R2B7|C4R2B7\_KOMPG:C4QWR4|C4QWR4\_KOMPG:C4R262|C4R262\_KOMPG:C4R5X8|C4R5X8\_KOMPG:C4R6P0|C4R6P0\_KOMPG:C4R3G3|C4R3G3\_KOMPG:C4QY58|C4QY58\_KOMPG:C4R4Q1|C4R4Q1\_KOMPG:C4R5E4|C4R5E4\_KOMPG:C4R2C7|C4R2C7\_KOMPG:C4QYN9|C4QYN9\_KOMPG:C4R6Q7|C4R6Q7\_KOMPG:C4R0N3|C4R0N3\_KOMPG:C4QWI5|C4QWI5\_KOMPG |  |  | DB Search |
| VGGI | 24.87 | 344.206 | 4 | -5.22 | 345.2106 | 10.74 | 4195 | 1.51e3 | 1 | 1 | C4R107|C4R107\_KOMPG:C4QXJ4|C4QXJ4\_KOMPG:C4QY74|C4QY74\_KOMPG:C4R3D0|C4R3D0\_KOMPG:C4R3N5|C4R3N5\_KOMPG:C4QWU9|C4QWU9\_KOMPG:C4R3L9|C4R3L9\_KOMPG:C4QW42|C4QW42\_KOMPG:C4R352|C4R352\_KOMPG:C4R2N5|C4R2N5\_KOMPG:C4R3N4|C4R3N4\_KOMPG:C4R5J9|C4R5J9\_KOMPG:C4R4Y1|C4R4Y1\_KOMPG:C4R4K2|C4R4K2\_KOMPG:C4R198|C4R198\_KOMPG:C4R549|C4R549\_KOMPG:C4R767|C4R767\_KOMPG:C4R753|C4R753\_KOMPG:C4R935|C4R935\_KOMPG:C4R0F4|C4R0F4\_KOMPG:C4R993|C4R993\_KOMPG:C4R373|C4R373\_KOMPG:C4R653|C4R653\_KOMPG:C4R8Q2|C4R8Q2\_KOMPG:C4R094|C4R094\_KOMPG:C4R5L6|C4R5L6\_KOMPG:C4QWD1|C4QWD1\_KOMPG:C4QXE2|C4QXE2\_KOMPG:C4QXQ4|C4QXQ4\_KOMPG:C4R361|C4R361\_KOMPG:C4R6U8|C4R6U8\_KOMPG:C4QZ04|C4QZ04\_KOMPG:C4R239|C4R239\_KOMPG:C4R6I0|C4R6I0\_KOMPG |  |  | DB Search |
| VGGL | 24.87 | 344.206 | 4 | -5.22 | 345.2106 | 10.74 | 4195 | 1.51e3 | 1 | 1 | C4R5B3|C4R5B3\_KOMPG:C4R564|C4R564\_KOMPG:C4R1L8|C4R1L8\_KOMPG:C4R9B4|C4R9B4\_KOMPG:C4QVY9|C4QVY9\_KOMPG:C4QZL5|C4QZL5\_KOMPG:C4QWN5|C4QWN5\_KOMPG:C4QYZ7|C4QYZ7\_KOMPG:C4R308|C4R308\_KOMPG:C4R3A3|C4R3A3\_KOMPG:C4R2A7|C4R2A7\_KOMPG:C4R7U2|C4R7U2\_KOMPG:C4R7W9|C4R7W9\_KOMPG:C4QXU8|C4QXU8\_KOMPG:C4R076|C4R076\_KOMPG:C4R6F4|C4R6F4\_KOMPG:C4R0F6|C4R0F6\_KOMPG:C4QZI2|C4QZI2\_KOMPG:C4R2Z8|C4R2Z8\_KOMPG:C4QZF0|C4QZF0\_KOMPG:C4R7R0|C4R7R0\_KOMPG:C4R0J8|C4R0J8\_KOMPG:C4R7X7|C4R7X7\_KOMPG:C4QVT0|C4QVT0\_KOMPG:C4QYG4|C4QYG4\_KOMPG:C4QZD7|C4QZD7\_KOMPG:C4R3D7|C4R3D7\_KOMPG:C4R0Z3|C4R0Z3\_KOMPG:C4QYI2|C4QYI2\_KOMPG:C4QXT4|C4QXT4\_KOMPG:C4QZ23|C4QZ23\_KOMPG:C4R411|C4R411\_KOMPG:C4R2Y3|C4R2Y3\_KOMPG:C4QXI8|PEX6\_KOMPG:C4R3J3|C4R3J3\_KOMPG:C4QVI1|C4QVI1\_KOMPG:C4R6Q4|C4R6Q4\_KOMPG:C4QW62|C4QW62\_KOMPG:C4R7J3|C4R7J3\_KOMPG:C4R8Y6|C4R8Y6\_KOMPG:C4QZ30|C4QZ30\_KOMPG:C4R335|C4R335\_KOMPG:C4R5F2|C4R5F2\_KOMPG:C4R468|C4R468\_KOMPG:C4R7M8|C4R7M8\_KOMPG:C4QX02|C4QX02\_KOMPG:C4R4K5|C4R4K5\_KOMPG:C4QYC3|C4QYC3\_KOMPG:C4QVT9|C4QVT9\_KOMPG:C4QWC1|C4QWC1\_KOMPG:C4QWS5|C4QWS5\_KOMPG:C4QZD0|C4QZD0\_KOMPG:C4QY35|C4QY35\_KOMPG:C4R6R5|C4R6R5\_KOMPG:C4R4V5|C4R4V5\_KOMPG:C4R5L0|C4R5L0\_KOMPG:C4R845|C4R845\_KOMPG:C4R326|C4R326\_KOMPG:C4R2Z5|C4R2Z5\_KOMPG:C4R642|C4R642\_KOMPG:C4R4X3|C4R4X3\_KOMPG:C4R6P9|C4R6P9\_KOMPG:C4R8I5|C4R8I5\_KOMPG:C4R1J0|C4R1J0\_KOMPG:C4QV23|C4QV23\_KOMPG |  |  | DB Search |
| GSGSGSGC(-1.01)GSG | 24.81 | 810.2688 | 11 | -8.24 | 811.2674 | 1.94 | 825 | 1.27e4 | 1 | 1 | C4QYF1|C4QYF1\_KOMPG | Half of a disulfide bridge | C8:Half of a disulfide bridge:1000 | DB Search |
| TSIA | 24.8 | 390.2114 | 4 | -5.32 | 391.2157 | 6.25 | 2416 | 1.07e4 | 1 | 1 | C4QWE5|C4QWE5\_KOMPG:C4R0H9|C4R0H9\_KOMPG:C4R5T4|C4R5T4\_KOMPG:C4R920|C4R920\_KOMPG:C4R0Z0|C4R0Z0\_KOMPG:C4R3T4|C4R3T4\_KOMPG:C4R7W7|C4R7W7\_KOMPG:C4R8B9|C4R8B9\_KOMPG:C4R195|C4R195\_KOMPG:C4R952|C4R952\_KOMPG:C4R9D8|C4R9D8\_KOMPG:C4R7U9|C4R7U9\_KOMPG:C4R3U2|C4R3U2\_KOMPG:C4QY29|C4QY29\_KOMPG:C4R268|C4R268\_KOMPG:C4R5M2|C4R5M2\_KOMPG:C4R150|C4R150\_KOMPG:C4QZS4|C4QZS4\_KOMPG:C4QZU4|C4QZU4\_KOMPG:C4QZK5|C4QZK5\_KOMPG:C4QV97|C4QV97\_KOMPG:C4R280|C4R280\_KOMPG:C4R0L0|C4R0L0\_KOMPG:C4QWR3|C4QWR3\_KOMPG:C4R2E5|C4R2E5\_KOMPG:C4R915|C4R915\_KOMPG:C4QWW5|C4QWW5\_KOMPG:C4QWF7|C4QWF7\_KOMPG:C4QVA9|C4QVA9\_KOMPG:C4QVX1|C4QVX1\_KOMPG:C4R0K1|C4R0K1\_KOMPG:C4R156|C4R156\_KOMPG:C4R0U5|C4R0U5\_KOMPG:C4R8H7|C4R8H7\_KOMPG:C4R8V0|C4R8V0\_KOMPG:C4R6R4|C4R6R4\_KOMPG:C4QY43|C4QY43\_KOMPG:C4R495|C4R495\_KOMPG:C4R3T6|C4R3T6\_KOMPG:C4QX02|C4QX02\_KOMPG:C4R5I6|C4R5I6\_KOMPG:C4R644|C4R644\_KOMPG:C4R1G1|C4R1G1\_KOMPG:C4R0D0|C4R0D0\_KOMPG:C4QVZ0|C4QVZ0\_KOMPG:C4R055|C4R055\_KOMPG:C4R096|C4R096\_KOMPG:C4R971|C4R971\_KOMPG:C4R814|C4R814\_KOMPG:C4R0F7|C4R0F7\_KOMPG:C4R713|C4R713\_KOMPG |  |  | DB Search |
| TSLA | 24.8 | 390.2114 | 4 | -5.32 | 391.2157 | 6.25 | 2416 | 1.07e4 | 1 | 1 | C4QYY5|C4QYY5\_KOMPG:C4R5F5|C4R5F5\_KOMPG:C4QXG3|C4QXG3\_KOMPG:C4R8N7|C4R8N7\_KOMPG:C4QWY8|AIM36\_KOMPG:C4R3D3|C4R3D3\_KOMPG:C4R7P2|C4R7P2\_KOMPG:C4QVC4|C4QVC4\_KOMPG:C4QV51|C4QV51\_KOMPG:C4QX93|C4QX93\_KOMPG:C4R5W3|C4R5W3\_KOMPG:C4QXC0|C4QXC0\_KOMPG:C4R0U1|C4R0U1\_KOMPG:C4R8W8|C4R8W8\_KOMPG:C4QXC5|C4QXC5\_KOMPG:C4R5M0|C4R5M0\_KOMPG:C4R5N8|C4R5N8\_KOMPG:C4R7Q8|C4R7Q8\_KOMPG:C4R7Y0|C4R7Y0\_KOMPG:C4R236|C4R236\_KOMPG:C4QWE2|C4QWE2\_KOMPG:C4R4H3|C4R4H3\_KOMPG:C4QVK5|C4QVK5\_KOMPG:C4QVM3|C4QVM3\_KOMPG:C4QW38|C4QW38\_KOMPG:C4QYF9|C4QYF9\_KOMPG:C4QVT0|C4QVT0\_KOMPG:C4QZV1|C4QZV1\_KOMPG:C4R403|C4R403\_KOMPG:C4R117|C4R117\_KOMPG:C4R475|C4R475\_KOMPG:C4R0N4|C4R0N4\_KOMPG:C4R3X4|C4R3X4\_KOMPG:C4R925|C4R925\_KOMPG:C4R429|C4R429\_KOMPG:C4QVL3|C4QVL3\_KOMPG:C4QV74|C4QV74\_KOMPG:C4R441|C4R441\_KOMPG:C4R7Q4|AIM11\_KOMPG:C4R299|C4R299\_KOMPG:C4R803|C4R803\_KOMPG:C4R4Z1|C4R4Z1\_KOMPG:C4R6U1|C4R6U1\_KOMPG:C4R7H2|C4R7H2\_KOMPG:C4R824|C4R824\_KOMPG:C4R8D5|C4R8D5\_KOMPG:C4R4F5|C4R4F5\_KOMPG:C4R4B3|CEGT\_KOMPG:C4QXV3|C4QXV3\_KOMPG:C4R1X7|C4R1X7\_KOMPG:C4R3W1|C4R3W1\_KOMPG:C4QXR1|C4QXR1\_KOMPG:C4R7B3|C4R7B3\_KOMPG:C4QY02|C4QY02\_KOMPG:C4R1W7|C4R1W7\_KOMPG:C4QW05|C4QW05\_KOMPG:C4R2I7|C4R2I7\_KOMPG:C4R317|C4R317\_KOMPG:C4QZX6|C4QZX6\_KOMPG:C4R6P7|C4R6P7\_KOMPG:C4QV26|C4QV26\_KOMPG:C4QYF1|C4QYF1\_KOMPG:C4R6D5|C4R6D5\_KOMPG:C4QWE7|C4QWE7\_KOMPG |  |  | DB Search |
| TPVSF | 24.78 | 549.2798 | 5 | -5.66 | 550.2827 | 22.56 | 8279 | 3.9e3 | 1 | 1 | C4QZM2|C4QZM2\_KOMPG:C4R0E0|C4R0E0\_KOMPG:C4R2Q8|C4R2Q8\_KOMPG |  |  | DB Search |
| RIVP | 24.65 | 483.3169 | 4 | -4.88 | 484.3206 | 10.06 | 3922 | 3.47e3 | 1 | 1 | C4R2U7|C4R2U7\_KOMPG:C4R0R3|C4R0R3\_KOMPG:C4R237|C4R237\_KOMPG:C4QZA8|C4QZA8\_KOMPG:C4R0V7|C4R0V7\_KOMPG:C4R584|C4R584\_KOMPG:C4R7B5|C4R7B5\_KOMPG:C4R9B7|C4R9B7\_KOMPG:C4QWF7|C4QWF7\_KOMPG:C4QXX5|C4QXX5\_KOMPG:C4QXV9|C4QXV9\_KOMPG:C4R0G8|C4R0G8\_KOMPG:C4QZ24|C4QZ24\_KOMPG:C4R6N1|C4R6N1\_KOMPG:C4R997|C4R997\_KOMPG:C4QZA0|C4QZA0\_KOMPG:C4R4Z6|C4R4Z6\_KOMPG:C4QVD8|C4QVD8\_KOMPG:C4R7N4|C4R7N4\_KOMPG:C4R993|C4R993\_KOMPG:C4R6H8|C4R6H8\_KOMPG:C4R5S2|C4R5S2\_KOMPG:C4R7D6|C4R7D6\_KOMPG:C4QZK2|C4QZK2\_KOMPG:C4R2J9|C4R2J9\_KOMPG:C4R8B3|C4R8B3\_KOMPG:C4R873|C4R873\_KOMPG |  |  | DB Search |
| RLVP | 24.65 | 483.3169 | 4 | -4.88 | 484.3206 | 10.06 | 3922 | 3.47e3 | 1 | 1 | C4QZQ4|C4QZQ4\_KOMPG:C4R2Y2|C4R2Y2\_KOMPG:C4QVP6|C4QVP6\_KOMPG:C4R7R7|C4R7R7\_KOMPG:C4QYB1|C4QYB1\_KOMPG:C4R564|C4R564\_KOMPG:C4QVH6|C4QVH6\_KOMPG:C4R1V9|C4R1V9\_KOMPG:C4QZ19|C4QZ19\_KOMPG:C4QV20|C4QV20\_KOMPG:C4R7N9|C4R7N9\_KOMPG:C4R537|C4R537\_KOMPG:C4QX52|C4QX52\_KOMPG:C4R1J3|C4R1J3\_KOMPG:C4QV32|C4QV32\_KOMPG:C4R5J0|C4R5J0\_KOMPG:C4R0G3|C4R0G3\_KOMPG:C4R1W2|C4R1W2\_KOMPG:C4R968|C4R968\_KOMPG:C4R773|C4R773\_KOMPG:C4QWM6|C4QWM6\_KOMPG:C4R6D4|C4R6D4\_KOMPG:C4QZM0|C4QZM0\_KOMPG:C4QXQ4|C4QXQ4\_KOMPG:C4QV97|C4QV97\_KOMPG:C4R8N0|C4R8N0\_KOMPG:C4R8R5|C4R8R5\_KOMPG:C4R627|C4R627\_KOMPG:C4R087|C4R087\_KOMPG |  |  | DB Search |
| AIFG | 24.47 | 406.2216 | 4 | 1.3 | 407.2284 | 22.63 | 8312 | 0 | 0 | 0 | C4R0J0|C4R0J0\_KOMPG:C4R3D7|C4R3D7\_KOMPG:C4QVT8|C4QVT8\_KOMPG:C4QZ90|C4QZ90\_KOMPG:C4QXA8|C4QXA8\_KOMPG:C4QWB0|C4QWB0\_KOMPG:C4R436|C4R436\_KOMPG:C4R831|C4R831\_KOMPG:C4QV41|C4QV41\_KOMPG:C4QWH1|C4QWH1\_KOMPG:C4QY82|C4QY82\_KOMPG:C4QZH5|C4QZH5\_KOMPG:C4QXU4|C4QXU4\_KOMPG:C4QVE0|C4QVE0\_KOMPG:C4R5Q7|C4R5Q7\_KOMPG:C4QW65|C4QW65\_KOMPG:C4R4P7|C4R4P7\_KOMPG:C4R4T7|C4R4T7\_KOMPG:C4R845|C4R845\_KOMPG:C4R8F9|C4R8F9\_KOMPG:C4R043|C4R043\_KOMPG:C4R464|C4R464\_KOMPG:C4R3A8|C4R3A8\_KOMPG:C4R668|C4R668\_KOMPG:C4QXD4|C4QXD4\_KOMPG:C4QVH1|C4QVH1\_KOMPG:C4QWY3|C4QWY3\_KOMPG:C4R813|C4R813\_KOMPG:C4R2B2|C4R2B2\_KOMPG:C4QZ49|C4QZ49\_KOMPG:C4R0P5|C4R0P5\_KOMPG:C4QZ00|C4QZ00\_KOMPG:C4R3B6|C4R3B6\_KOMPG |  |  | DB Search |
| ALFG | 24.47 | 406.2216 | 4 | 1.3 | 407.2284 | 22.63 | 8312 | 0 | 0 | 0 | C4QZN8|C4QZN8\_KOMPG:C4QYS7|C4QYS7\_KOMPG:C4QYG4|C4QYG4\_KOMPG:C4QYB2|C4QYB2\_KOMPG:C4QWJ4|MDM10\_KOMPG:C4QWW5|C4QWW5\_KOMPG:C4R8R2|C4R8R2\_KOMPG:C4QX99|C4QX99\_KOMPG:C4R9D2|C4R9D2\_KOMPG:C4QWN8|C4QWN8\_KOMPG:C4R6E3|C4R6E3\_KOMPG:C4QZJ9|C4QZJ9\_KOMPG:C4R1Z3|C4R1Z3\_KOMPG:C4R099|C4R099\_KOMPG:C4R7Q1|C4R7Q1\_KOMPG:C4R4U5|C4R4U5\_KOMPG:C4R289|C4R289\_KOMPG:C4R0Z0|C4R0Z0\_KOMPG:C4QW62|C4QW62\_KOMPG:C4R8R4|C4R8R4\_KOMPG:C4R406|C4R406\_KOMPG:C4QWA3|C4QWA3\_KOMPG:C4R3K2|C4R3K2\_KOMPG:C4R4T6|C4R4T6\_KOMPG:C4R051|C4R051\_KOMPG:C4R5M8|C4R5M8\_KOMPG:C4R7K4|C4R7K4\_KOMPG:C4R2S5|C4R2S5\_KOMPG:C4QZV2|C4QZV2\_KOMPG:C4R8J5|C4R8J5\_KOMPG:C4QVI3|C4QVI3\_KOMPG:C4R7Y1|C4R7Y1\_KOMPG:C4R5J2|C4R5J2\_KOMPG:C4R0J4|C4R0J4\_KOMPG:C4R174|C4R174\_KOMPG:C4R2N2|C4R2N2\_KOMPG:C4R0M6|C4R0M6\_KOMPG:C4QV75|C4QV75\_KOMPG:C4R0I3|C4R0I3\_KOMPG:C4QUZ0|C4QUZ0\_KOMPG:C4QYE3|C4QYE3\_KOMPG:C4R2H7|C4R2H7\_KOMPG |  |  | DB Search |
| AIISI | 24.46 | 515.3319 | 5 | -6.84 | 516.3344 | 37.01 | 12185 | 8.65e2 | 1 | 1 | C4QWG8|C4QWG8\_KOMPG:C4R6Q6|C4R6Q6\_KOMPG |  |  | DB Search |
| AIISL | 24.46 | 515.3319 | 5 | -6.84 | 516.3344 | 37.01 | 12185 | 8.65e2 | 1 | 1 | C4R1J1|C4R1J1\_KOMPG:C4QZM3|C4QZM3\_KOMPG |  |  | DB Search |
| AILSI | 24.46 | 515.3319 | 5 | -6.84 | 516.3344 | 37.01 | 12185 | 8.65e2 | 1 | 1 | C4R4V4|C4R4V4\_KOMPG:C4R1M9|C4R1M9\_KOMPG:C4R0G3|C4R0G3\_KOMPG:C4R0D0|C4R0D0\_KOMPG |  |  | DB Search |
| AILSL | 24.46 | 515.3319 | 5 | -6.84 | 516.3344 | 37.01 | 12185 | 8.65e2 | 1 | 1 | C4R2V2|C4R2V2\_KOMPG:C4R924|C4R924\_KOMPG:C4R4S1|C4R4S1\_KOMPG:C4R7I2|C4R7I2\_KOMPG:C4R6Q1|C4R6Q1\_KOMPG:C4R524|C4R524\_KOMPG:C4QW99|C4QW99\_KOMPG:C4QYH5|C4QYH5\_KOMPG:C4QWF2|C4QWF2\_KOMPG:C4R4M7|C4R4M7\_KOMPG:C4R4D0|C4R4D0\_KOMPG:C4R2D0|C4R2D0\_KOMPG:C4QXZ5|C4QXZ5\_KOMPG:C4R7G1|C4R7G1\_KOMPG |  |  | DB Search |
| ALISI | 24.46 | 515.3319 | 5 | -6.84 | 516.3344 | 37.01 | 12185 | 8.65e2 | 1 | 1 | C4QV42|C4QV42\_KOMPG:C4R4F0|C4R4F0\_KOMPG:C4R2C0|C4R2C0\_KOMPG |  |  | DB Search |
| ALISL | 24.46 | 515.3319 | 5 | -6.84 | 516.3344 | 37.01 | 12185 | 8.65e2 | 1 | 1 | C4R325|C4R325\_KOMPG:C4R7J8|C4R7J8\_KOMPG:C4R2Q9|C4R2Q9\_KOMPG:C4R1L9|C4R1L9\_KOMPG:C4QXG8|C4QXG8\_KOMPG:C4QWW6|C4QWW6\_KOMPG:C4R6F6|C4R6F6\_KOMPG:C4R2H6|C4R2H6\_KOMPG:C4R5V4|C4R5V4\_KOMPG:C4R2N6|C4R2N6\_KOMPG:C4R1H3|C4R1H3\_KOMPG:C4R642|C4R642\_KOMPG:C4R207|C4R207\_KOMPG |  |  | DB Search |
| ALLSI | 24.46 | 515.3319 | 5 | -6.84 | 516.3344 | 37.01 | 12185 | 8.65e2 | 1 | 1 | C4R2Z8|C4R2Z8\_KOMPG:C4R109|C4R109\_KOMPG:C4R793|C4R793\_KOMPG:C4QY37|C4QY37\_KOMPG:C4R1W9|C4R1W9\_KOMPG |  |  | DB Search |
| ALLSL | 24.46 | 515.3319 | 5 | -6.84 | 516.3344 | 37.01 | 12185 | 8.65e2 | 1 | 1 | C4R7X4|C4R7X4\_KOMPG:C4R5R3|C4R5R3\_KOMPG:C4QXE2|C4QXE2\_KOMPG:C4QZB6|C4QZB6\_KOMPG:C4R1D2|C4R1D2\_KOMPG:C4R724|C4R724\_KOMPG:C4R2L2|C4R2L2\_KOMPG:C4QYN7|C4QYN7\_KOMPG:C4R091|C4R091\_KOMPG:C4R6B2|C4R6B2\_KOMPG:C4R0X4|C4R0X4\_KOMPG:C4QZY0|C4QZY0\_KOMPG |  |  | DB Search |
| PFLTSE | 24.41 | 692.3381 | 6 | -7.52 | 693.3384 | 20.10 | 7523 | 8.77e2 | 1 | 1 | C4QYT3|C4QYT3\_KOMPG |  |  | DB Search |
| GVGP | 24.4 | 328.1747 | 4 | -1.91 | 329.1805 | 5.92 | 2228 | 4.71e4 | 1 | 1 | C4QVB7|C4QVB7\_KOMPG:C4R0E4|C4R0E4\_KOMPG:C4QZU8|C4QZU8\_KOMPG:C4R0U7|C4R0U7\_KOMPG:C4QWB0|C4QWB0\_KOMPG:C4R2C2|C4R2C2\_KOMPG:C4R193|C4R193\_KOMPG:C4QWZ9|C4QWZ9\_KOMPG:C4R558|C4R558\_KOMPG:C4QZ19|C4QZ19\_KOMPG:C4R110|C4R110\_KOMPG:C4R2A8|C4R2A8\_KOMPG:C4R8S1|C4R8S1\_KOMPG:C4R226|C4R226\_KOMPG:C4R1F2|C4R1F2\_KOMPG:C4QWX5|C4QWX5\_KOMPG:C4R3C7|C4R3C7\_KOMPG:C4R7B9|C4R7B9\_KOMPG:C4QZ20|FEN1\_KOMPG:C4QZF0|C4QZF0\_KOMPG:C4QXD4|C4QXD4\_KOMPG:C4R544|C4R544\_KOMPG:C4R741|C4R741\_KOMPG:C4QZB1|C4QZB1\_KOMPG:C4QW72|C4QW72\_KOMPG:C4R3P1|C4R3P1\_KOMPG:C4R822|C4R822\_KOMPG |  |  | DB Search |
| PQ(+0.98)QPQ(+0.98)QPQ | 24.38 | 951.4297 | 8 | 1.16 | 952.4358 | 34.78 | 11631 | 1.08e2 | 1 | 1 | C4QXV6|C4QXV6\_KOMPG | Deamidation (NQ), Deamidation (NQ) | Q2:Deamidation (NQ):0 Q5:Deamidation (NQ):11.22 | DB Search |
| CGKHA | 24.37 | 514.2322 | 5 | 1.96 | 515.2392 | 12.62 | 4989 | 0 | 0 | 0 | C4R048|C4R048\_KOMPG |  |  | DB Search |
| YHH | 24.3 | 455.1917 | 3 | -4.98 | 456.1956 | 6.94 | 2666 | 0 | 0 | 0 | C4R2C1|C4R2C1\_KOMPG:C4QX82|C4QX82\_KOMPG:C4R7K5|C4R7K5\_KOMPG:C4R6I4|C4R6I4\_KOMPG:C4R864|C4R864\_KOMPG:C4QVN4|C4QVN4\_KOMPG:C4QVW3|C4QVW3\_KOMPG:C4QW59|C4QW59\_KOMPG:C4QV43|C4QV43\_KOMPG:C4R5W8|C4R5W8\_KOMPG:C4R0G0|C4R0G0\_KOMPG:C4R1X4|C4R1X4\_KOMPG:C4R383|C4R383\_KOMPG:C4QXQ9|C4QXQ9\_KOMPG:C4R7I1|C4R7I1\_KOMPG:C4QVT4|C4QVT4\_KOMPG:C4R663|C4R663\_KOMPG:C4QW61|C4QW61\_KOMPG:C4QYW8|C4QYW8\_KOMPG:C4QXB2|C4QXB2\_KOMPG:C4QXU2|C4QXU2\_KOMPG:C4R048|C4R048\_KOMPG:C4R725|C4R725\_KOMPG:C4R6F3|C4R6F3\_KOMPG:C4QV53|C4QV53\_KOMPG:C4R4R8|ARO1\_KOMPG:C4QXJ6|C4QXJ6\_KOMPG:C4QVR7|C4QVR7\_KOMPG:C4R590|C4R590\_KOMPG:C4QZF5|C4QZF5\_KOMPG:C4QZF1|C4QZF1\_KOMPG:C4R7C5|C4R7C5\_KOMPG:C4R2P6|C4R2P6\_KOMPG:C4R739|C4R739\_KOMPG:C4QZ41|C4QZ41\_KOMPG:C4R513|C4R513\_KOMPG:C4R169|C4R169\_KOMPG:C4R475|C4R475\_KOMPG:C4R8B6|C4R8B6\_KOMPG:C4R3F4|C4R3F4\_KOMPG:C4R955|C4R955\_KOMPG:C4R5S3|C4R5S3\_KOMPG:C4R091|C4R091\_KOMPG:C4R3E8|C4R3E8\_KOMPG:C4R5F8|C4R5F8\_KOMPG:C4QYP5|C4QYP5\_KOMPG:C4QZ69|C4QZ69\_KOMPG:C4QXV7|C4QXV7\_KOMPG:C4QWT6|C4QWT6\_KOMPG:C4QZW9|C4QZW9\_KOMPG:C4QX86|C4QX86\_KOMPG:C4R4N9|C4R4N9\_KOMPG:C4R8D5|C4R8D5\_KOMPG:C4R9F6|C4R9F6\_KOMPG:C4QZM8|C4QZM8\_KOMPG:C4QX00|C4QX00\_KOMPG:C4R720|C4R720\_KOMPG:C4QVT5|C4QVT5\_KOMPG:C4R0S6|C4R0S6\_KOMPG:C4QYC1|C4QYC1\_KOMPG:C4R1Q5|C4R1Q5\_KOMPG:C4R3Y7|C4R3Y7\_KOMPG:C4R1F5|C4R1F5\_KOMPG:C4R7Q9|C4R7Q9\_KOMPG:C4R7J2|C4R7J2\_KOMPG:C4R4R1|C4R4R1\_KOMPG:C4R4X3|C4R4X3\_KOMPG:C4R1D9|C4R1D9\_KOMPG:C4R207|C4R207\_KOMPG:C4QVK4|C4QVK4\_KOMPG:C4R6R1|C4R6R1\_KOMPG:C4R4M5|C4R4M5\_KOMPG:C4R8V3|C4R8V3\_KOMPG |  |  | DB Search |
| DAKDAN | 24.27 | 632.2766 | 6 | -5.06 | 633.2791 | 27.73 | 9717 | 3.83e3 | 1 | 1 | C4R5R1|C4R5R1\_KOMPG |  |  | DB Search |
| WNPH | 24.26 | 552.2444 | 4 | 6.18 | 553.2538 | 5.42 | 2167 | 2.61e2 | 1 | 1 | C4R9B5|C4R9B5\_KOMPG:C4R295|C4R295\_KOMPG:C4QZV5|C4QZV5\_KOMPG:C4QWL7|C4QWL7\_KOMPG:C4QWE2|C4QWE2\_KOMPG:C4R4L8|C4R4L8\_KOMPG:C4R2S4|C4R2S4\_KOMPG |  |  | DB Search |
| IPGGIL | 24.26 | 568.3584 | 6 | -1.66 | 569.3633 | 38.32 | 12498 | 0 | 0 | 0 | C4R713|C4R713\_KOMPG |  |  | DB Search |
| IPGGLL | 24.26 | 568.3584 | 6 | -1.66 | 569.3633 | 38.32 | 12498 | 0 | 0 | 0 | C4R700|C4R700\_KOMPG |  |  | DB Search |
| YDAF | 24.25 | 514.2064 | 4 | 0.11 | 515.2124 | 17.26 | 6784 | 9.51e2 | 1 | 1 | C4R6B9|C4R6B9\_KOMPG:C4QX99|C4QX99\_KOMPG:C4R704|C4R704\_KOMPG:C4R3B0|C4R3B0\_KOMPG:C4R7R8|C4R7R8\_KOMPG:C4R3H4|C4R3H4\_KOMPG:C4R1H0|C4R1H0\_KOMPG:C4QX53|C4QX53\_KOMPG:C4QX88|C4QX88\_KOMPG:C4R3V2|C4R3V2\_KOMPG:C4QVC2|C4QVC2\_KOMPG:C4R207|C4R207\_KOMPG:C4R4P4|C4R4P4\_KOMPG:C4R1E7|C4R1E7\_KOMPG:C4R5Z4|C4R5Z4\_KOMPG:C4QWR3|C4QWR3\_KOMPG |  |  | DB Search |
| LGATTLEL | 24.2 | 816.4593 | 8 | -2.06 | 817.4628 | 42.05 | 13313 | 0 | 0 | 0 | C4R6D7|C4R6D7\_KOMPG |  |  | DB Search |
| VIGS | 24.2 | 374.2165 | 4 | -4.06 | 375.2213 | 5.20 | 2012 | 6.27e3 | 1 | 1 | C4R4U6|C4R4U6\_KOMPG:C4QYE1|C4QYE1\_KOMPG:C4R074|C4R074\_KOMPG:C4QXK0|C4QXK0\_KOMPG:C4R940|C4R940\_KOMPG:C4R4V3|C4R4V3\_KOMPG:C4R2U1|C4R2U1\_KOMPG:C4QYZ6|C4QYZ6\_KOMPG:C4QZL5|C4QZL5\_KOMPG:C4R6Y2|C4R6Y2\_KOMPG:C4QW53|C4QW53\_KOMPG:C4R7V4|C4R7V4\_KOMPG:C4R4E0|C4R4E0\_KOMPG:C4R382|C4R382\_KOMPG:C4QX76|C4QX76\_KOMPG:C4QZ74|C4QZ74\_KOMPG:C4R6L7|C4R6L7\_KOMPG:C4R116|C4R116\_KOMPG:C4R9C2|C4R9C2\_KOMPG:C4R108|C4R108\_KOMPG:C4R0L6|C4R0L6\_KOMPG:C4QYR3|C4QYR3\_KOMPG:C4R6F4|C4R6F4\_KOMPG:C4R7Y0|C4R7Y0\_KOMPG:C4R7F5|C4R7F5\_KOMPG:C4QXR7|C4QXR7\_KOMPG:C4QZ87|C4QZ87\_KOMPG:C4R0W7|C4R0W7\_KOMPG:C4QY37|C4QY37\_KOMPG:C4R8S3|C4R8S3\_KOMPG:C4QZZ1|C4QZZ1\_KOMPG:C4R825|C4R825\_KOMPG:C4R610|C4R610\_KOMPG:C4QZ59|C4QZ59\_KOMPG:C4QXH2|AIM24\_KOMPG:C4QZV6|C4QZV6\_KOMPG:C4R482|C4R482\_KOMPG:C4QVX2|C4QVX2\_KOMPG:C4R2V3|C4R2V3\_KOMPG:C4R928|C4R928\_KOMPG:C4QZW9|C4QZW9\_KOMPG:C4R942|C4R942\_KOMPG:C4QWE8|C4QWE8\_KOMPG:C4R1F2|C4R1F2\_KOMPG:C4R8Y5|C4R8Y5\_KOMPG:C4R5L7|C4R5L7\_KOMPG:C4QXA3|C4QXA3\_KOMPG:C4R615|C4R615\_KOMPG:C4R7X9|BMT1\_KOMPG:C4R668|C4R668\_KOMPG:C4QVA8|C4QVA8\_KOMPG:C4R4V4|C4R4V4\_KOMPG:C4R567|C4R567\_KOMPG:C4R8N0|C4R8N0\_KOMPG:C4R2H7|C4R2H7\_KOMPG:C4R697|C4R697\_KOMPG:C4R649|C4R649\_KOMPG |  |  | DB Search |
| VLGS | 24.2 | 374.2165 | 4 | -4.06 | 375.2213 | 5.20 | 2012 | 6.27e3 | 1 | 1 | C4R585|C4R585\_KOMPG:C4QWE1|C4QWE1\_KOMPG:C4R5S6|C4R5S6\_KOMPG:C4R324|C4R324\_KOMPG:C4R0K6|C4R0K6\_KOMPG:C4R609|C4R609\_KOMPG:C4R561|C4R561\_KOMPG:C4R1X4|C4R1X4\_KOMPG:C4R789|C4R789\_KOMPG:C4R1D0|C4R1D0\_KOMPG:C4R6Q7|C4R6Q7\_KOMPG:C4R061|C4R061\_KOMPG:C4R9C5|C4R9C5\_KOMPG:C4QVA7|C4QVA7\_KOMPG:C4QVX4|C4QVX4\_KOMPG:C4R549|C4R549\_KOMPG:C4QXD1|C4QXD1\_KOMPG:C4R2E9|C4R2E9\_KOMPG:C4R268|C4R268\_KOMPG:C4R6R7|C4R6R7\_KOMPG:C4R2Z3|C4R2Z3\_KOMPG:C4R451|C4R451\_KOMPG:C4R2R9|C4R2R9\_KOMPG:C4QYM6|C4QYM6\_KOMPG:C4QZZ4|C4QZZ4\_KOMPG:C4R359|C4R359\_KOMPG:C4R235|C4R235\_KOMPG:C4R404|C4R404\_KOMPG:C4QV52|C4QV52\_KOMPG:C4R7E1|C4R7E1\_KOMPG:C4R075|C4R075\_KOMPG:C4R770|C4R770\_KOMPG:C4R1H0|C4R1H0\_KOMPG:C4QYY1|C4QYY1\_KOMPG:C4R0I3|C4R0I3\_KOMPG:C4QYU6|C4QYU6\_KOMPG:C4R3D2|C4R3D2\_KOMPG:C4R117|C4R117\_KOMPG:C4R169|C4R169\_KOMPG:C4QYC9|C4QYC9\_KOMPG:C4R1Y5|C4R1Y5\_KOMPG:C4R5I0|C4R5I0\_KOMPG:C4QY76|C4QY76\_KOMPG:C4R9F1|C4R9F1\_KOMPG:C4QVX9|C4QVX9\_KOMPG:C4R9E3|C4R9E3\_KOMPG:C4R2Q9|C4R2Q9\_KOMPG:C4R0U9|C4R0U9\_KOMPG:C4R2T8|C4R2T8\_KOMPG:C4R5U1|C4R5U1\_KOMPG:C4QV22|C4QV22\_KOMPG:C4R7I2|C4R7I2\_KOMPG:C4QZA9|C4QZA9\_KOMPG:C4R867|C4R867\_KOMPG:C4R9C6|C4R9C6\_KOMPG:C4QVD1|C4QVD1\_KOMPG:C4QXY0|C4QXY0\_KOMPG:C4R8V0|C4R8V0\_KOMPG:C4R537|C4R537\_KOMPG:C4QXJ3|C4QXJ3\_KOMPG:C4R821|C4R821\_KOMPG:C4QWC3|C4QWC3\_KOMPG:C4R7Q2|C4R7Q2\_KOMPG:C4R5K1|C4R5K1\_KOMPG:C4R8U7|C4R8U7\_KOMPG:C4R1P7|C4R1P7\_KOMPG:C4R8E8|PURA\_KOMPG:C4R0E5|C4R0E5\_KOMPG:C4R6S4|C4R6S4\_KOMPG:C4R514|C4R514\_KOMPG:C4QWK4|C4QWK4\_KOMPG:C4R379|C4R379\_KOMPG:C4R6A0|C4R6A0\_KOMPG:C4R1Z6|C4R1Z6\_KOMPG |  |  | DB Search |
| DLSVAS | 24.18 | 590.2911 | 6 | -4.57 | 591.2943 | 12.99 | 5122 | 8.42e3 | 1 | 1 | C4R7W6|C4R7W6\_KOMPG |  |  | DB Search |
| DRF | 24.14 | 436.207 | 3 | -6.05 | 437.2106 | 17.42 | 6833 | 5.05e2 | 1 | 1 | C4R529|C4R529\_KOMPG:C4QV81|C4QV81\_KOMPG:C4R5U0|C4R5U0\_KOMPG:C4R8U9|C4R8U9\_KOMPG:C4R1D2|C4R1D2\_KOMPG:C4QW28|C4QW28\_KOMPG:C4R006|C4R006\_KOMPG:C4R3Q5|C4R3Q5\_KOMPG:C4R1G7|C4R1G7\_KOMPG:C4R4G8|C4R4G8\_KOMPG:C4R003|C4R003\_KOMPG:C4QVC0|C4QVC0\_KOMPG:C4R510|C4R510\_KOMPG:C4R0L8|C4R0L8\_KOMPG:C4QVX4|C4QVX4\_KOMPG:C4R4D9|C4R4D9\_KOMPG:C4R1B9|C4R1B9\_KOMPG:C4QWI9|C4QWI9\_KOMPG:C4R1T4|C4R1T4\_KOMPG:C4R014|C4R014\_KOMPG:C4R6G3|C4R6G3\_KOMPG:C4QZ97|C4QZ97\_KOMPG:C4R0N2|C4R0N2\_KOMPG:C4QXU2|C4QXU2\_KOMPG:C4R3H5|C4R3H5\_KOMPG:C4QVM0|C4QVM0\_KOMPG:C4R0I6|C4R0I6\_KOMPG:C4R5S2|C4R5S2\_KOMPG:C4QWU0|C4QWU0\_KOMPG:C4R571|C4R571\_KOMPG:C4R4W5|C4R4W5\_KOMPG:C4R8Q7|C4R8Q7\_KOMPG:C4QYN9|C4QYN9\_KOMPG:C4R2R6|C4R2R6\_KOMPG:C4R666|C4R666\_KOMPG:C4R1A9|C4R1A9\_KOMPG:C4R1S8|C4R1S8\_KOMPG:C4R6A6|C4R6A6\_KOMPG:C4QWJ2|C4QWJ2\_KOMPG:C4QXI3|C4QXI3\_KOMPG:C4QYX0|C4QYX0\_KOMPG:C4R189|C4R189\_KOMPG:C4R2J1|C4R2J1\_KOMPG:C4QXZ3|C4QXZ3\_KOMPG:C4R6I6|C4R6I6\_KOMPG:C4QXM3|C4QXM3\_KOMPG:C4R779|C4R779\_KOMPG:C4R635|C4R635\_KOMPG:C4R925|C4R925\_KOMPG:C4QW64|C4QW64\_KOMPG:C4QVL3|C4QVL3\_KOMPG:C4R2K0|UTP25\_KOMPG:C4QYQ4|C4QYQ4\_KOMPG:C4R1S7|C4R1S7\_KOMPG:C4R438|C4R438\_KOMPG:C4R808|C4R808\_KOMPG:C4QZ19|C4QZ19\_KOMPG:C4QYP8|C4QYP8\_KOMPG:C4R1H3|C4R1H3\_KOMPG:C4QY65|C4QY65\_KOMPG:C4R8D5|C4R8D5\_KOMPG:C4R0L7|C4R0L7\_KOMPG:C4QV44|C4QV44\_KOMPG:C4R996|C4R996\_KOMPG:C4QWQ8|C4QWQ8\_KOMPG:C4QXX8|AIM41\_KOMPG:C4QZN0|C4QZN0\_KOMPG:C4QVM7|C4QVM7\_KOMPG:C4R6B9|C4R6B9\_KOMPG:C4R4N2|C4R4N2\_KOMPG:C4QYG7|C4QYG7\_KOMPG:C4QYK2|C4QYK2\_KOMPG:C4R7K4|C4R7K4\_KOMPG:C4QZB6|C4QZB6\_KOMPG:C4QYX6|C4QYX6\_KOMPG:C4R407|C4R407\_KOMPG:C4QZB4|C4QZB4\_KOMPG:C4R3U7|C4R3U7\_KOMPG:C4QW15|C4QW15\_KOMPG:C4R8D9|C4R8D9\_KOMPG:C4R0J3|C4R0J3\_KOMPG:C4R0W0|C4R0W0\_KOMPG:C4R322|C4R322\_KOMPG:C4QVK4|C4QVK4\_KOMPG:C4QVY3|C4QVY3\_KOMPG:C4R7I9|C4R7I9\_KOMPG:C4R1Q8|C4R1Q8\_KOMPG:C4R467|C4R467\_KOMPG:C4R554|C4R554\_KOMPG:C4R291|C4R291\_KOMPG:C4QZ79|C4QZ79\_KOMPG:C4R6N7|C4R6N7\_KOMPG:C4R7I6|C4R7I6\_KOMPG:C4R1V4|C4R1V4\_KOMPG:C4R5U6|C4R5U6\_KOMPG:C4QX55|C4QX55\_KOMPG:C4R534|C4R534\_KOMPG:C4QXC5|C4QXC5\_KOMPG:C4R8B7|C4R8B7\_KOMPG:C4R297|C4R297\_KOMPG:C4R983|C4R983\_KOMPG:C4R657|C4R657\_KOMPG:C4R0K3|C4R0K3\_KOMPG:C4QWR8|C4QWR8\_KOMPG:C4QX95|C4QX95\_KOMPG:C4R1N3|C4R1N3\_KOMPG:C4QYT7|C4QYT7\_KOMPG:C4R374|C4R374\_KOMPG:C4R5P8|C4R5P8\_KOMPG:C4R148|C4R148\_KOMPG:C4R1T2|C4R1T2\_KOMPG:C4R0G8|C4R0G8\_KOMPG:C4QY13|C4QY13\_KOMPG:C4R2W1|C4R2W1\_KOMPG:C4R276|C4R276\_KOMPG:C4QW18|C4QW18\_KOMPG:C4R8C1|C4R8C1\_KOMPG:C4QXM9|C4QXM9\_KOMPG:C4R0E6|C4R0E6\_KOMPG:C4QXA5|PFKA2\_KOMPG:C4R644|C4R644\_KOMPG:C4R1B8|C4R1B8\_KOMPG:C4R370|C4R370\_KOMPG:C4QW68|C4QW68\_KOMPG:C4QVP3|C4QVP3\_KOMPG:C4R167|C4R167\_KOMPG:C4R4V1|C4R4V1\_KOMPG:C4R1W1|C4R1W1\_KOMPG:C4R6G2|C4R6G2\_KOMPG:C4QVA1|C4QVA1\_KOMPG:C4R4H7|C4R4H7\_KOMPG:C4QXS6|C4QXS6\_KOMPG:C4QW87|C4QW87\_KOMPG:C4R387|C4R387\_KOMPG |  |  | DB Search |
| GAPNYAHP | 24.12 | 825.377 | 8 | -5.56 | 826.3776 | 14.20 | 5638 | 3.3e2 | 1 | 1 | C4R646|C4R646\_KOMPG |  |  | DB Search |
| IIIP | 24.07 | 454.3155 | 4 | -2.4 | 455.3206 | 34.52 | 11565 | 2.64e3 | 1 | 1 | C4R951|C4R951\_KOMPG:C4R7S8|C4R7S8\_KOMPG:C4R278|C4R278\_KOMPG:C4R4U4|C4R4U4\_KOMPG:C4R773|C4R773\_KOMPG:C4R8D7|ATG37\_KOMPG:C4QVX5|C4QVX5\_KOMPG:C4R6W1|C4R6W1\_KOMPG:C4R5I2|C4R5I2\_KOMPG:C4R6Q9|C4R6Q9\_KOMPG:C4R230|C4R230\_KOMPG |  |  | DB Search |
| IILP | 24.07 | 454.3155 | 4 | -2.4 | 455.3206 | 34.52 | 11565 | 2.64e3 | 1 | 1 | C4R4N2|C4R4N2\_KOMPG:C4R3W6|C4R3W6\_KOMPG:C4QZ58|C4QZ58\_KOMPG:C4R3C5|C4R3C5\_KOMPG:C4R4V3|C4R4V3\_KOMPG:C4QWN8|C4QWN8\_KOMPG:C4R4C7|C4R4C7\_KOMPG:C4R7J5|C4R7J5\_KOMPG:C4R349|C4R349\_KOMPG:C4R565|C4R565\_KOMPG:C4R754|C4R754\_KOMPG:C4QYQ8|C4QYQ8\_KOMPG:C4QY10|C4QY10\_KOMPG:C4QX95|C4QX95\_KOMPG:C4QYI9|C4QYI9\_KOMPG:C4R534|C4R534\_KOMPG:C4R5Z8|C4R5Z8\_KOMPG:C4R0L0|C4R0L0\_KOMPG:C4R424|C4R424\_KOMPG |  |  | DB Search |
| ILIP | 24.07 | 454.3155 | 4 | -2.4 | 455.3206 | 34.52 | 11565 | 2.64e3 | 1 | 1 | C4R3P0|C4R3P0\_KOMPG:C4QW86|C4QW86\_KOMPG:C4QXA5|PFKA2\_KOMPG:C4QVT8|C4QVT8\_KOMPG:C4R5Z7|C4R5Z7\_KOMPG:C4R601|C4R601\_KOMPG:C4R253|C4R253\_KOMPG:C4QWP8|C4QWP8\_KOMPG:C4R5F5|C4R5F5\_KOMPG:C4R6F9|C4R6F9\_KOMPG:C4R659|C4R659\_KOMPG:C4QVB5|C4QVB5\_KOMPG:C4R2D6|C4R2D6\_KOMPG:C4R1T0|C4R1T0\_KOMPG:C4R6F6|C4R6F6\_KOMPG:C4QWD1|C4QWD1\_KOMPG:C4R5Y7|C4R5Y7\_KOMPG:C4QYJ5|C4QYJ5\_KOMPG:C4R2D2|C4R2D2\_KOMPG:C4R3B7|C4R3B7\_KOMPG:C4QY32|C4QY32\_KOMPG:C4R6K0|C4R6K0\_KOMPG |  |  | DB Search |
| ILLP | 24.07 | 454.3155 | 4 | -2.4 | 455.3206 | 34.52 | 11565 | 2.64e3 | 1 | 1 | C4R1G2|C4R1G2\_KOMPG:C4QVP8|C4QVP8\_KOMPG:C4R5U2|C4R5U2\_KOMPG:C4QWB0|C4QWB0\_KOMPG:C4QXK0|C4QXK0\_KOMPG:C4QY44|C4QY44\_KOMPG:C4R972|C4R972\_KOMPG:C4R2K0|UTP25\_KOMPG:C4QX30|C4QX30\_KOMPG:C4QV66|C4QV66\_KOMPG:C4R6M3|C4R6M3\_KOMPG:C4R839|C4R839\_KOMPG:C4R350|C4R350\_KOMPG:C4QY81|C4QY81\_KOMPG:C4QWK2|C4QWK2\_KOMPG:C4QVR4|C4QVR4\_KOMPG:C4R0P3|C4R0P3\_KOMPG:C4R4T2|C4R4T2\_KOMPG:C4R8Z1|C4R8Z1\_KOMPG:C4R7B7|C4R7B7\_KOMPG:C4R849|C4R849\_KOMPG:C4QZM0|C4QZM0\_KOMPG:C4QXD4|C4QXD4\_KOMPG:C4R5D9|C4R5D9\_KOMPG:C4R8E9|C4R8E9\_KOMPG:C4QZX4|C4QZX4\_KOMPG:C4R713|C4R713\_KOMPG:C4QWR3|C4QWR3\_KOMPG |  |  | DB Search |
| LIIP | 24.07 | 454.3155 | 4 | -2.4 | 455.3206 | 34.52 | 11565 | 2.64e3 | 1 | 1 | C4R172|C4R172\_KOMPG:C4QXS8|C4QXS8\_KOMPG:C4R490|C4R490\_KOMPG:C4R5S6|C4R5S6\_KOMPG:C4R7K4|C4R7K4\_KOMPG:C4R1I3|C4R1I3\_KOMPG:C4R692|C4R692\_KOMPG:C4QYY0|C4QYY0\_KOMPG:C4QVL3|C4QVL3\_KOMPG:C4QV84|C4QV84\_KOMPG:C4R8D1|C4R8D1\_KOMPG:C4R524|C4R524\_KOMPG:C4R2J0|C4R2J0\_KOMPG:C4QZH7|C4QZH7\_KOMPG:C4R8R4|C4R8R4\_KOMPG:C4R0Z9|C4R0Z9\_KOMPG:C4QYP0|C4QYP0\_KOMPG:C4R220|C4R220\_KOMPG:C4R647|C4R647\_KOMPG:C4QVM6|C4QVM6\_KOMPG:C4R189|C4R189\_KOMPG |  |  | DB Search |
| LILP | 24.07 | 454.3155 | 4 | -2.4 | 455.3206 | 34.52 | 11565 | 2.64e3 | 1 | 1 | C4QWF8|C4QWF8\_KOMPG:C4R142|C4R142\_KOMPG:C4QWG8|C4QWG8\_KOMPG:C4R904|C4R904\_KOMPG:C4R2S7|C4R2S7\_KOMPG:C4R0M7|C4R0M7\_KOMPG:C4R1E9|C4R1E9\_KOMPG:C4R658|C4R658\_KOMPG:C4R9B4|C4R9B4\_KOMPG:C4R7C4|C4R7C4\_KOMPG:C4R4S7|C4R4S7\_KOMPG:C4QXN0|C4QXN0\_KOMPG:C4R957|C4R957\_KOMPG:C4R732|C4R732\_KOMPG:C4QW27|C4QW27\_KOMPG:C4QXM1|C4QXM1\_KOMPG:C4R275|C4R275\_KOMPG:C4QYD3|C4QYD3\_KOMPG:C4R5N8|C4R5N8\_KOMPG:C4QWB9|C4QWB9\_KOMPG:C4R1Q5|C4R1Q5\_KOMPG:C4R657|C4R657\_KOMPG:C4R1D3|C4R1D3\_KOMPG:C4R0M0|C4R0M0\_KOMPG:C4QYM4|C4QYM4\_KOMPG:C4R6C6|C4R6C6\_KOMPG:C4R4X3|C4R4X3\_KOMPG:C4R2B2|C4R2B2\_KOMPG:C4R1Q0|C4R1Q0\_KOMPG:C4R5C8|C4R5C8\_KOMPG |  |  | DB Search |
| LLIP | 24.07 | 454.3155 | 4 | -2.4 | 455.3206 | 34.52 | 11565 | 2.64e3 | 1 | 1 | C4R3B8|C4R3B8\_KOMPG:C4QXL0|C4QXL0\_KOMPG:C4QYH1|C4QYH1\_KOMPG:C4QXQ8|C4QXQ8\_KOMPG:C4R0V4|C4R0V4\_KOMPG:C4QZM1|C4QZM1\_KOMPG:C4R4Q7|C4R4Q7\_KOMPG:C4R6D1|C4R6D1\_KOMPG:C4QXA9|C4QXA9\_KOMPG:C4R0P7|C4R0P7\_KOMPG:C4R2C5|C4R2C5\_KOMPG:C4R370|C4R370\_KOMPG:C4R9B2|C4R9B2\_KOMPG:C4R804|C4R804\_KOMPG:C4QYT0|C4QYT0\_KOMPG:C4R3W8|C4R3W8\_KOMPG:C4R5J2|C4R5J2\_KOMPG:C4R1F0|C4R1F0\_KOMPG:C4R1T3|C4R1T3\_KOMPG:C4QW05|C4QW05\_KOMPG:C4QYS6|MTNA\_KOMPG:C4QWH2|C4QWH2\_KOMPG:C4QW41|C4QW41\_KOMPG:C4R3H2|C4R3H2\_KOMPG:C4R721|C4R721\_KOMPG |  |  | DB Search |
| LLLP | 24.07 | 454.3155 | 4 | -2.4 | 455.3206 | 34.52 | 11565 | 2.64e3 | 1 | 1 | C4QV04|C4QV04\_KOMPG:C4QZQ4|C4QZQ4\_KOMPG:C4QZI1|C4QZI1\_KOMPG:C4R3P2|C4R3P2\_KOMPG:C4R7M9|C4R7M9\_KOMPG:C4R852|C4R852\_KOMPG:C4QZ83|C4QZ83\_KOMPG:C4QVC0|C4QVC0\_KOMPG:C4R7P2|C4R7P2\_KOMPG:C4R398|C4R398\_KOMPG:C4R518|C4R518\_KOMPG:C4R3A3|C4R3A3\_KOMPG:C4R1A8|C4R1A8\_KOMPG:C4R824|C4R824\_KOMPG:C4R5D6|C4R5D6\_KOMPG:C4R2K1|C4R2K1\_KOMPG:C4QX93|C4QX93\_KOMPG:C4QY88|C4QY88\_KOMPG:C4R318|C4R318\_KOMPG:C4R8I0|C4R8I0\_KOMPG:C4R929|C4R929\_KOMPG:C4R927|C4R927\_KOMPG:C4R2D4|C4R2D4\_KOMPG:C4R325|C4R325\_KOMPG:C4R1M6|C4R1M6\_KOMPG:C4R109|C4R109\_KOMPG:C4R446|C4R446\_KOMPG:C4QV42|C4QV42\_KOMPG:C4R7E8|C4R7E8\_KOMPG:C4R720|C4R720\_KOMPG:C4R9A9|C4R9A9\_KOMPG:C4R3K9|C4R3K9\_KOMPG:C4R875|C4R875\_KOMPG:C4R218|C4R218\_KOMPG:C4R6H6|C4R6H6\_KOMPG:C4R178|C4R178\_KOMPG:C4R1X9|C4R1X9\_KOMPG:C4R5R4|C4R5R4\_KOMPG:C4QZJ7|C4QZJ7\_KOMPG:C4R810|C4R810\_KOMPG:C4QWZ8|C4QWZ8\_KOMPG:C4R813|C4R813\_KOMPG:C4R5V0|C4R5V0\_KOMPG:C4R0W0|C4R0W0\_KOMPG:C4R239|C4R239\_KOMPG:C4R5W9|C4R5W9\_KOMPG:C4QW72|C4QW72\_KOMPG |  |  | DB Search |
| VGVT | 24.06 | 374.2165 | 4 | -2.35 | 375.222 | 5.91 | 2327 | 7.9e3 | 1 | 1 | C4QZQ4|C4QZQ4\_KOMPG:C4R6V9|C4R6V9\_KOMPG:C4R2T7|C4R2T7\_KOMPG:C4R1Q4|C4R1Q4\_KOMPG:C4R8D6|C4R8D6\_KOMPG:C4QXI7|C4QXI7\_KOMPG:C4QWB0|C4QWB0\_KOMPG:C4R2F1|C4R2F1\_KOMPG:C4R0I4|C4R0I4\_KOMPG:C4QW62|C4QW62\_KOMPG:C4R7D8|C4R7D8\_KOMPG:C4R8F3|C4R8F3\_KOMPG:C4R4S9|C4R4S9\_KOMPG:C4R6M5|C4R6M5\_KOMPG:C4QY56|C4QY56\_KOMPG:C4QWA3|C4QWA3\_KOMPG:C4R3A3|C4R3A3\_KOMPG:C4R2A4|C4R2A4\_KOMPG:C4R226|C4R226\_KOMPG:C4QYW8|C4QYW8\_KOMPG:C4R014|C4R014\_KOMPG:C4R0E7|C4R0E7\_KOMPG:C4R5X0|C4R5X0\_KOMPG:C4R143|C4R143\_KOMPG:C4R4C7|C4R4C7\_KOMPG:C4R2R6|C4R2R6\_KOMPG:C4R618|C4R618\_KOMPG:C4R1V1|C4R1V1\_KOMPG:C4QWG7|C4QWG7\_KOMPG:C4R513|C4R513\_KOMPG |  |  | DB Search |
| TTFTT | 23.99 | 569.2697 | 5 | 1.65 | 570.2765 | 3.50 | 1524 | 0 | 0 | 0 | C4R2T4|C4R2T4\_KOMPG:C4QYW7|C4QYW7\_KOMPG:C4R3A0|C4R3A0\_KOMPG:C4R2E9|C4R2E9\_KOMPG |  |  | DB Search |
| VVNSP | 23.94 | 514.2751 | 5 | -0.42 | 515.2809 | 9.29 | 3594 | 1.21e3 | 1 | 1 | C4R723|C4R723\_KOMPG |  |  | DB Search |
| S(+42.01)SPNAFH | 23.88 | 800.3453 | 7 | -0.93 | 801.3499 | 11.93 | 4708 | 1.97e3 | 1 | 1 | C4QY80|C4QY80\_KOMPG | Acetylation (Protein N-term) | S1:Acetylation (Protein N-term):1000 | DB Search |
| THGP | 23.87 | 410.1914 | 4 | 5.6 | 411.1999 | 13.65 | 5373 | 5.56e2 | 1 | 1 | C4R6W3|C4R6W3\_KOMPG:C4QV06|C4QV06\_KOMPG:C4R4S4|C4R4S4\_KOMPG:C4QZH1|C4QZH1\_KOMPG:C4R1F7|C4R1F7\_KOMPG:C4R4N7|C4R4N7\_KOMPG:C4R3C3|C4R3C3\_KOMPG:C4R5W3|C4R5W3\_KOMPG:C4R627|C4R627\_KOMPG:C4QZL1|C4QZL1\_KOMPG:C4R147|C4R147\_KOMPG |  |  | DB Search |
| VIIGS | 23.87 | 487.3006 | 5 | 1.5 | 488.3074 | 15.69 | 6182 | 1.57e3 | 1 | 1 | C4R558|C4R558\_KOMPG:C4R3B5|C4R3B5\_KOMPG |  |  | DB Search |
| VILGS | 23.87 | 487.3006 | 5 | 1.5 | 488.3074 | 15.69 | 6182 | 1.57e3 | 1 | 1 | C4R641|C4R641\_KOMPG:C4R785|C4R785\_KOMPG:C4R7F2|C4R7F2\_KOMPG:C4R8Z6|C4R8Z6\_KOMPG:C4R7B6|C4R7B6\_KOMPG:C4R5L0|C4R5L0\_KOMPG:C4QY16|C4QY16\_KOMPG |  |  | DB Search |
| VLIGS | 23.87 | 487.3006 | 5 | 1.5 | 488.3074 | 15.69 | 6182 | 1.57e3 | 1 | 1 | C4QWL1|C4QWL1\_KOMPG:C4QW97|C4QW97\_KOMPG:C4R4D0|C4R4D0\_KOMPG:C4R7R8|C4R7R8\_KOMPG |  |  | DB Search |
| VLLGS | 23.87 | 487.3006 | 5 | 1.5 | 488.3074 | 15.69 | 6182 | 1.57e3 | 1 | 1 | C4R4Y9|C4R4Y9\_KOMPG:C4QYK9|C4QYK9\_KOMPG:C4R655|C4R655\_KOMPG:C4R110|C4R110\_KOMPG:C4QXJ4|C4QXJ4\_KOMPG:C4R3V7|C4R3V7\_KOMPG:C4QXI3|C4QXI3\_KOMPG |  |  | DB Search |
| TVGVAGI | 23.87 | 615.3591 | 7 | -0.94 | 616.3643 | 23.76 | 8599 | 2.96e3 | 1 | 1 | C4R701|C4R701\_KOMPG |  |  | DB Search |
| VGSQP | 23.82 | 486.2438 | 5 | 2.03 | 487.2509 | 4.40 | 1725 | 1.26e3 | 2 | 2 | C4R782|C4R782\_KOMPG |  |  | DB Search |
| KFHT | 23.81 | 531.2805 | 4 | 4.98 | 532.2891 | 16.61 | 6548 | 3.34e2 | 1 | 1 | C4R4F8|C4R4F8\_KOMPG:C4R5R1|C4R5R1\_KOMPG:C4R8R4|C4R8R4\_KOMPG:C4R1Q9|C4R1Q9\_KOMPG:C4R553|C4R553\_KOMPG:C4R6C8|C4R6C8\_KOMPG |  |  | DB Search |
| LASGAI | 23.69 | 530.3064 | 6 | 0.98 | 531.3129 | 13.72 | 5408 | 0 | 0 | 0 | C4R643|C4R643\_KOMPG |  |  | DB Search |
| HFT | 23.64 | 403.1855 | 3 | -0.83 | 404.1915 | 13.94 | 5543 | 0 | 0 | 0 | C4R9D6|C4R9D6\_KOMPG:C4QYG6|C4QYG6\_KOMPG:C4R3H3|C4R3H3\_KOMPG:C4R0Z1|C4R0Z1\_KOMPG:C4R214|C4R214\_KOMPG:C4R368|C4R368\_KOMPG:C4R7R7|C4R7R7\_KOMPG:C4R9F0|C4R9F0\_KOMPG:C4QWW6|C4QWW6\_KOMPG:C4R003|C4R003\_KOMPG:C4QVE8|C4QVE8\_KOMPG:C4QY84|C4QY84\_KOMPG:C4R1X5|C4R1X5\_KOMPG:C4QVF2|C4QVF2\_KOMPG:C4QV50|C4QV50\_KOMPG:C4R1X0|C4R1X0\_KOMPG:C4R5S9|C4R5S9\_KOMPG:C4QZB9|C4QZB9\_KOMPG:C4R6E1|C4R6E1\_KOMPG:C4QV53|C4QV53\_KOMPG:C4R7S8|C4R7S8\_KOMPG:C4R2E4|C4R2E4\_KOMPG:C4R1F1|C4R1F1\_KOMPG:C4R4L4|C4R4L4\_KOMPG:C4QW38|C4QW38\_KOMPG:C4R893|C4R893\_KOMPG:C4R5U7|C4R5U7\_KOMPG:C4QW70|C4QW70\_KOMPG:C4QWH8|C4QWH8\_KOMPG:C4QXM2|C4QXM2\_KOMPG:C4R0L0|C4R0L0\_KOMPG:C4QZ41|C4QZ41\_KOMPG:C4QZD7|C4QZD7\_KOMPG:C4R7Q7|C4R7Q7\_KOMPG:C4R395|C4R395\_KOMPG:C4R8M7|C4R8M7\_KOMPG:C4R1S7|C4R1S7\_KOMPG:C4R3I9|C4R3I9\_KOMPG:C4R7I0|C4R7I0\_KOMPG:C4R381|C4R381\_KOMPG:C4QZZ5|C4QZZ5\_KOMPG:C4R1U8|C4R1U8\_KOMPG:C4R1M0|C4R1M0\_KOMPG:C4R468|C4R468\_KOMPG:C4QXW3|C4QXW3\_KOMPG:C4R6B9|C4R6B9\_KOMPG:C4QVW2|C4QVW2\_KOMPG:C4QZ68|C4QZ68\_KOMPG:C4R6M0|C4R6M0\_KOMPG:C4QYC4|C4QYC4\_KOMPG:C4R7S5|C4R7S5\_KOMPG:C4R270|C4R270\_KOMPG:C4QWI4|C4QWI4\_KOMPG:C4R6M2|C4R6M2\_KOMPG:C4R814|C4R814\_KOMPG:C4R207|C4R207\_KOMPG:C4R3V3|C4R3V3\_KOMPG:C4R3X2|C4R3X2\_KOMPG:C4R292|C4R292\_KOMPG:C4QZQ4|C4QZQ4\_KOMPG:C4R0N9|C4R0N9\_KOMPG:C4QXF3|C4QXF3\_KOMPG:C4QVW4|C4QVW4\_KOMPG:C4QZJ3|C4QZJ3\_KOMPG:C4R0V4|C4R0V4\_KOMPG:C4QXG3|C4QXG3\_KOMPG:C4R2B7|C4R2B7\_KOMPG:C4R7D4|C4R7D4\_KOMPG:C4QVE2|C4QVE2\_KOMPG:C4QVS9|C4QVS9\_KOMPG:C4QZI7|C4QZI7\_KOMPG:C4QX08|C4QX08\_KOMPG:C4R1J4|C4R1J4\_KOMPG:C4QYJ6|C4QYJ6\_KOMPG:C4QV51|C4QV51\_KOMPG:C4R534|C4R534\_KOMPG:C4R540|C4R540\_KOMPG:C4QYZ1|C4QYZ1\_KOMPG:C4R912|C4R912\_KOMPG:C4R5M5|C4R5M5\_KOMPG:C4QWW1|C4QWW1\_KOMPG:C4R679|C4R679\_KOMPG:C4QZ36|C4QZ36\_KOMPG:C4R6D7|C4R6D7\_KOMPG:C4R145|C4R145\_KOMPG:C4R3P0|C4R3P0\_KOMPG:C4R851|C4R851\_KOMPG:C4QZ59|C4QZ59\_KOMPG:C4R1H2|C4R1H2\_KOMPG:C4R0C4|C4R0C4\_KOMPG:C4R7W8|C4R7W8\_KOMPG:C4R4C8|C4R4C8\_KOMPG:C4R4Z9|C4R4Z9\_KOMPG:C4R2H6|C4R2H6\_KOMPG:C4R1T2|C4R1T2\_KOMPG:C4QZY7|C4QZY7\_KOMPG:C4R8I0|C4R8I0\_KOMPG:C4QX00|C4QX00\_KOMPG:C4QYR1|C4QYR1\_KOMPG:C4QVR9|IRC19\_KOMPG:C4R4Y0|C4R4Y0\_KOMPG:C4R743|C4R743\_KOMPG:C4R9A9|C4R9A9\_KOMPG:C4R7W0|C4R7W0\_KOMPG:C4QX80|PSD1\_KOMPG:C4R0T3|C4R0T3\_KOMPG:C4R1R8|C4R1R8\_KOMPG:C4R4R1|C4R4R1\_KOMPG:C4R5H2|C4R5H2\_KOMPG:C4QXZ9|C4QXZ9\_KOMPG:C4R556|C4R556\_KOMPG |  |  | DB Search |
| PFPP | 23.62 | 456.2372 | 4 | 1.78 | 457.2442 | 13.81 | 5467 | 0 | 0 | 0 | C4QWR5|C4QWR5\_KOMPG:C4R184|C4R184\_KOMPG:C4R581|C4R581\_KOMPG:C4R074|C4R074\_KOMPG:C4QWT5|C4QWT5\_KOMPG:C4QY74|C4QY74\_KOMPG:C4R6F6|C4R6F6\_KOMPG:C4R4U5|C4R4U5\_KOMPG:C4R476|C4R476\_KOMPG:C4R810|C4R810\_KOMPG:C4QZG4|C4QZG4\_KOMPG:C4R0Z4|C4R0Z4\_KOMPG:C4R318|C4R318\_KOMPG |  |  | DB Search |
| PNDQPP | 23.54 | 666.2973 | 6 | 1.2 | 667.3037 | 13.01 | 5077 | 7.33e3 | 1 | 1 | C4R692|C4R692\_KOMPG |  |  | DB Search |
| VATGF | 23.43 | 493.2536 | 5 | -5.76 | 494.2568 | 16.27 | 6468 | 1.51e4 | 1 | 1 | C4R360|PSD2\_KOMPG |  |  | DB Search |
| VAAP | 23.43 | 356.206 | 4 | -1.11 | 357.2119 | 7.38 | 2801 | 2.39e3 | 1 | 1 | C4QYS9|C4QYS9\_KOMPG:C4R864|C4R864\_KOMPG:C4R044|C4R044\_KOMPG:C4QXW1|C4QXW1\_KOMPG:C4R0Q2|C4R0Q2\_KOMPG:C4R4C2|C4R4C2\_KOMPG:C4R9B4|C4R9B4\_KOMPG:C4R5T3|C4R5T3\_KOMPG:C4R4Q7|C4R4Q7\_KOMPG:C4R3R6|C4R3R6\_KOMPG:C4R8B9|C4R8B9\_KOMPG:C4R8V0|C4R8V0\_KOMPG:C4R4C6|C4R4C6\_KOMPG:C4QYX1|C4QYX1\_KOMPG:C4R4D9|C4R4D9\_KOMPG:C4R5H5|C4R5H5\_KOMPG:C4R2I0|C4R2I0\_KOMPG:C4R5Q6|C4R5Q6\_KOMPG:C4R9G2|C4R9G2\_KOMPG:C4QWI6|C4QWI6\_KOMPG:C4R1Y0|C4R1Y0\_KOMPG:C4QXU8|C4QXU8\_KOMPG:C4QZU5|C4QZU5\_KOMPG:C4QV50|C4QV50\_KOMPG:C4R048|C4R048\_KOMPG:C4R0G3|C4R0G3\_KOMPG:C4R3H7|C4R3H7\_KOMPG:C4R1X9|C4R1X9\_KOMPG:C4R4K6|C4R4K6\_KOMPG:C4R3H4|C4R3H4\_KOMPG:C4QV64|C4QV64\_KOMPG:C4R0Z6|C4R0Z6\_KOMPG:C4R5R5|C4R5R5\_KOMPG:C4QYT8|C4QYT8\_KOMPG:C4QW22|C4QW22\_KOMPG:C4QX05|C4QX05\_KOMPG:C4R5Y6|C4R5Y6\_KOMPG:C4QZB1|C4QZB1\_KOMPG:C4R045|C4R045\_KOMPG:C4QV59|C4QV59\_KOMPG:C4R649|C4R649\_KOMPG:C4QVB4|C4QVB4\_KOMPG |  |  | DB Search |
| E(-18.01)VLFAGFG | 23.4 | 820.4119 | 8 | -9.26 | 821.4095 | 47.32 | 14277 | 2.06e2 | 1 | 1 | C4R1C2|C4R1C2\_KOMPG | Pyro-glu from E | E1:Pyro-glu from E:1000 | DB Search |
| PNPGPTH | 23.38 | 718.3398 | 7 | 1.31 | 719.3463 | 19.08 | 7299 | 1.22e3 | 1 | 1 | C4QXJ4|C4QXJ4\_KOMPG |  |  | DB Search |
| ATIF | 23.38 | 450.2478 | 4 | -9.17 | 451.2498 | 24.44 | 8854 | 1.68e3 | 1 | 1 | C4R851|C4R851\_KOMPG:C4R2G0|C4R2G0\_KOMPG:C4R252|C4R252\_KOMPG:C4QZU8|C4QZU8\_KOMPG:C4R7Q2|C4R7Q2\_KOMPG:C4R148|C4R148\_KOMPG:C4R3L2|C4R3L2\_KOMPG:C4R914|C4R914\_KOMPG:C4QXX6|C4QXX6\_KOMPG:C4R4I8|C4R4I8\_KOMPG:C4R6Q6|C4R6Q6\_KOMPG:C4R526|C4R526\_KOMPG:C4R3H2|C4R3H2\_KOMPG:C4R033|C4R033\_KOMPG:C4QV00|C4QV00\_KOMPG:C4R4R9|C4R4R9\_KOMPG:C4QYD4|C4QYD4\_KOMPG |  |  | DB Search |
| ATLF | 23.38 | 450.2478 | 4 | -9.17 | 451.2498 | 24.44 | 8854 | 1.68e3 | 1 | 1 | C4QYY6|C4QYY6\_KOMPG:C4QWE1|C4QWE1\_KOMPG:C4R6X0|C4R6X0\_KOMPG:C4QXA8|C4QXA8\_KOMPG:C4R070|C4R070\_KOMPG:C4R926|C4R926\_KOMPG:C4R0S7|C4R0S7\_KOMPG:C4R1M3|C4R1M3\_KOMPG:C4R8N7|C4R8N7\_KOMPG:C4R481|C4R481\_KOMPG:C4R774|C4R774\_KOMPG:C4R4N0|C4R4N0\_KOMPG:C4R7A9|C4R7A9\_KOMPG:C4R6N0|C4R6N0\_KOMPG:C4R8Y5|C4R8Y5\_KOMPG:C4QX02|C4QX02\_KOMPG:C4R490|C4R490\_KOMPG:C4R499|C4R499\_KOMPG:C4R1X9|C4R1X9\_KOMPG:C4R6D4|C4R6D4\_KOMPG:C4R8U4|C4R8U4\_KOMPG:C4R6L9|C4R6L9\_KOMPG:C4QWM4|C4QWM4\_KOMPG:C4R1H0|C4R1H0\_KOMPG:C4R2X6|C4R2X6\_KOMPG:C4R5E5|C4R5E5\_KOMPG:C4QV24|C4QV24\_KOMPG:C4R439|C4R439\_KOMPG:C4R5C8|C4R5C8\_KOMPG:C4R9C7|C4R9C7\_KOMPG |  |  | DB Search |
| VGGP | 23.36 | 328.1747 | 4 | -1.91 | 329.1805 | 5.92 | 2159 | 4.71e4 | 1 | 1 | C4QYY5|C4QYY5\_KOMPG:C4R680|C4R680\_KOMPG:C4QXB2|C4QXB2\_KOMPG:C4R877|C4R877\_KOMPG:C4R646|C4R646\_KOMPG:C4QV60|C4QV60\_KOMPG:C4R3D0|C4R3D0\_KOMPG:C4R115|C4R115\_KOMPG:C4R271|C4R271\_KOMPG:C4QWC7|C4QWC7\_KOMPG:C4QV65|C4QV65\_KOMPG:C4QVS2|C4QVS2\_KOMPG:C4R8L0|C4R8L0\_KOMPG:C4R580|C4R580\_KOMPG:C4R443|C4R443\_KOMPG:C4QY21|C4QY21\_KOMPG:C4R781|C4R781\_KOMPG:C4R4C9|C4R4C9\_KOMPG:C4R6P6|C4R6P6\_KOMPG:C4R6N4|C4R6N4\_KOMPG:C4R957|C4R957\_KOMPG |  |  | DB Search |
| AITIG | 23.3 | 473.2849 | 5 | -4.41 | 474.2889 | 17.39 | 6818 | 3.71e3 | 1 | 1 | C4QV04|C4QV04\_KOMPG:C4QV05|C4QV05\_KOMPG:C4R049|C4R049\_KOMPG |  |  | DB Search |
| AITLG | 23.3 | 473.2849 | 5 | -4.41 | 474.2889 | 17.39 | 6818 | 3.71e3 | 1 | 1 | C4R9B4|C4R9B4\_KOMPG:C4R6R4|C4R6R4\_KOMPG |  |  | DB Search |
| ALTIG | 23.3 | 473.2849 | 5 | -4.41 | 474.2889 | 17.39 | 6818 | 3.71e3 | 1 | 1 | C4R4D0|C4R4D0\_KOMPG:C4R3A5|C4R3A5\_KOMPG |  |  | DB Search |
| ALTLG | 23.3 | 473.2849 | 5 | -4.41 | 474.2889 | 17.39 | 6818 | 3.71e3 | 1 | 1 | C4R697|C4R697\_KOMPG |  |  | DB Search |
| PVIV | 23.27 | 426.2842 | 4 | 4.41 | 427.2923 | 20.36 | 7664 | 0 | 0 | 0 | C4R3D7|C4R3D7\_KOMPG:C4R3B3|C4R3B3\_KOMPG:C4R2Q9|C4R2Q9\_KOMPG:C4QUZ7|C4QUZ7\_KOMPG:C4QW84|C4QW84\_KOMPG:C4R1G8|C4R1G8\_KOMPG:C4R6F7|C4R6F7\_KOMPG:C4R8D0|C4R8D0\_KOMPG:C4R4V6|C4R4V6\_KOMPG:C4R1K1|C4R1K1\_KOMPG:C4R846|C4R846\_KOMPG:C4R136|C4R136\_KOMPG:C4R4T6|C4R4T6\_KOMPG:C4R453|C4R453\_KOMPG:C4R198|C4R198\_KOMPG:C4R7N6|C4R7N6\_KOMPG:C4QX98|C4QX98\_KOMPG:C4R2E9|C4R2E9\_KOMPG:C4QWC1|C4QWC1\_KOMPG:C4QY97|C4QY97\_KOMPG:C4R6H8|C4R6H8\_KOMPG:C4R2V7|C4R2V7\_KOMPG:C4QYQ0|C4QYQ0\_KOMPG:C4R817|C4R817\_KOMPG:C4R6R8|C4R6R8\_KOMPG:C4QYM3|C4QYM3\_KOMPG:C4R938|C4R938\_KOMPG |  |  | DB Search |
| PVLV | 23.27 | 426.2842 | 4 | 4.41 | 427.2923 | 20.36 | 7664 | 0 | 0 | 0 | C4R783|C4R783\_KOMPG:C4QZ56|C4QZ56\_KOMPG:C4R7I9|C4R7I9\_KOMPG:C4R9D3|C4R9D3\_KOMPG:C4R6C5|C4R6C5\_KOMPG:C4R1J1|C4R1J1\_KOMPG:C4QY98|C4QY98\_KOMPG:C4R669|C4R669\_KOMPG:C4R2R3|C4R2R3\_KOMPG:C4QVD8|C4QVD8\_KOMPG:C4R829|C4R829\_KOMPG:C4R5Q7|C4R5Q7\_KOMPG:C4R7N4|C4R7N4\_KOMPG:C4QWJ7|C4QWJ7\_KOMPG:C4R2E4|C4R2E4\_KOMPG:C4QWR2|C4QWR2\_KOMPG:Q6DNA2|ALG3\_KOMPG:C4R7S0|C4R7S0\_KOMPG:C4R4I8|C4R4I8\_KOMPG:C4R022|C4R022\_KOMPG:C4R1V1|C4R1V1\_KOMPG:C4R342|C4R342\_KOMPG:C4R822|C4R822\_KOMPG:C4R0L9|C4R0L9\_KOMPG:C4R3Z4|C4R3Z4\_KOMPG:C4QY76|C4QY76\_KOMPG:C4QV61|C4QV61\_KOMPG:C4R1Q4|C4R1Q4\_KOMPG:C4R7N8|C4R7N8\_KOMPG:C4R5E3|C4R5E3\_KOMPG:Q9P4C8|SAR1\_KOMPG:C4QYD0|C4QYD0\_KOMPG:C4QZT8|C4QZT8\_KOMPG:C4R2U0|C4R2U0\_KOMPG:C4R508|C4R508\_KOMPG:C4R335|C4R335\_KOMPG:C4R0Y6|C4R0Y6\_KOMPG:C4R723|C4R723\_KOMPG:C4R5L7|C4R5L7\_KOMPG:C4R2B4|C4R2B4\_KOMPG:C4R6I9|C4R6I9\_KOMPG:C4R3X3|C4R3X3\_KOMPG:C4QZ94|C4QZ94\_KOMPG:C4QZM7|C4QZM7\_KOMPG:C4R6M8|C4R6M8\_KOMPG:C4R317|C4R317\_KOMPG:C4R4Y3|C4R4Y3\_KOMPG:C4QW79|C4QW79\_KOMPG:C4QVW6|C4QVW6\_KOMPG:C4QX91|C4QX91\_KOMPG:C4R1W5|C4R1W5\_KOMPG:C4R3P1|C4R3P1\_KOMPG |  |  | DB Search |
| G(+42.01)KILVYSIF | 23.27 | 1080.6219 | 9 | -1.11 | 541.3163 | 43.88 | 13638 | 1.72e3 | 1 | 1 | C4R6F1|C4R6F1\_KOMPG | Acetylation (Protein N-term) | G1:Acetylation (Protein N-term):1000 | DB Search |
| EIVR | 23.24 | 515.3067 | 4 | -4.7 | 516.3103 | 11.12 | 4401 | 1.13e4 | 1 | 1 | C4R6V6|C4R6V6\_KOMPG:C4QY01|C4QY01\_KOMPG:C4R584|C4R584\_KOMPG:C4QXS5|C4QXS5\_KOMPG:C4QY20|C4QY20\_KOMPG:C4QVB0|RRP36\_KOMPG:C4R6N7|C4R6N7\_KOMPG:C4R5P8|C4R5P8\_KOMPG:C4R5Q0|C4R5Q0\_KOMPG:C4QYV4|C4QYV4\_KOMPG:C4R3V6|C4R3V6\_KOMPG:C4QXF8|C4QXF8\_KOMPG:C4QWL1|C4QWL1\_KOMPG:C4QVN3|C4QVN3\_KOMPG:C4R2E8|C4R2E8\_KOMPG:C4QXL7|C4QXL7\_KOMPG:C4R9E4|C4R9E4\_KOMPG:C4QVQ5|C4QVQ5\_KOMPG:C4R1B0|C4R1B0\_KOMPG:C4R7K4|C4R7K4\_KOMPG:C4QY25|C4QY25\_KOMPG:C4QXH5|C4QXH5\_KOMPG:C4QYY0|C4QYY0\_KOMPG:C4QWQ7|C4QWQ7\_KOMPG:C4R5A8|C4R5A8\_KOMPG:C4R167|C4R167\_KOMPG:C4QYU0|C4QYU0\_KOMPG:C4R7J5|C4R7J5\_KOMPG:C4R571|C4R571\_KOMPG:C4R075|C4R075\_KOMPG:C4QY10|C4QY10\_KOMPG:C4R6A0|C4R6A0\_KOMPG:Q9P4D1|ACT\_KOMPG |  |  | DB Search |
| ELVR | 23.24 | 515.3067 | 4 | -4.7 | 516.3103 | 11.12 | 4401 | 1.13e4 | 1 | 1 | C4R2Y7|C4R2Y7\_KOMPG:C4QZZ7|GEP3\_KOMPG:C4R467|C4R467\_KOMPG:C4R072|C4R072\_KOMPG:C4QX43|C4QX43\_KOMPG:C4R730|C4R730\_KOMPG:C4R4C3|C4R4C3\_KOMPG:C4R001|C4R001\_KOMPG:C4QYU9|C4QYU9\_KOMPG:C4R747|C4R747\_KOMPG:C4R8A8|C4R8A8\_KOMPG:C4QVS7|C4QVS7\_KOMPG:C4QZ31|C4QZ31\_KOMPG:C4QZK7|C4QZK7\_KOMPG:C4R2J4|C4R2J4\_KOMPG:C4R4I4|C4R4I4\_KOMPG:C4R2P8|C4R2P8\_KOMPG:C4R155|C4R155\_KOMPG:C4R6W9|C4R6W9\_KOMPG:C4QVS8|C4QVS8\_KOMPG:C4R8Y0|C4R8Y0\_KOMPG:C4R1E5|C4R1E5\_KOMPG:C4R3V5|C4R3V5\_KOMPG:C4QYZ5|C4QYZ5\_KOMPG:C4QYJ2|C4QYJ2\_KOMPG:C4QYV3|C4QYV3\_KOMPG:C4R3M4|C4R3M4\_KOMPG:C4R8T8|C4R8T8\_KOMPG:C4R893|C4R893\_KOMPG:C4QW32|C4QW32\_KOMPG:C4R0X8|C4R0X8\_KOMPG:C4QVP0|C4QVP0\_KOMPG:C4R0P5|C4R0P5\_KOMPG:C4QVV6|C4QVV6\_KOMPG:C4QYV1|C4QYV1\_KOMPG:C4R6C9|C4R6C9\_KOMPG:C4QZ59|C4QZ59\_KOMPG:C4R1J5|C4R1J5\_KOMPG:C4QZW2|C4QZW2\_KOMPG:C4R455|C4R455\_KOMPG:C4QZ18|C4QZ18\_KOMPG:C4R412|C4R412\_KOMPG:C4QWF2|C4QWF2\_KOMPG:C4R5Z1|C4R5Z1\_KOMPG:C4QZ19|C4QZ19\_KOMPG:C4R4F7|C4R4F7\_KOMPG:C4R6X7|C4R6X7\_KOMPG:C4R4Z1|C4R4Z1\_KOMPG:C4R3S5|C4R3S5\_KOMPG:C4R2W2|C4R2W2\_KOMPG:C4QWR7|C4QWR7\_KOMPG:C4QXA3|C4QXA3\_KOMPG:C4R0F4|C4R0F4\_KOMPG:C4QZB5|C4QZB5\_KOMPG:C4R9A5|C4R9A5\_KOMPG:C4R8J4|C4R8J4\_KOMPG:C4R7L1|C4R7L1\_KOMPG:C4R862|C4R862\_KOMPG:C4R250|C4R250\_KOMPG:C4QXH9|C4QXH9\_KOMPG:C4R7R8|C4R7R8\_KOMPG:C4R3Q8|C4R3Q8\_KOMPG:C4QXJ7|C4QXJ7\_KOMPG:C4R4C4|C4R4C4\_KOMPG:C4R3X5|C4R3X5\_KOMPG:C4QW16|C4QW16\_KOMPG:C4QYR4|C4QYR4\_KOMPG:C4QZS6|C4QZS6\_KOMPG:C4QZ02|C4QZ02\_KOMPG:C4R5Z8|C4R5Z8\_KOMPG |  |  | DB Search |
| SPT | 23.16 | 303.143 | 3 | 6.39 | 304.1515 | 7.14 | 2731 | 5.4e3 | 2 | 2 | C4R529|C4R529\_KOMPG:C4R296|C4R296\_KOMPG:C4QW28|C4QW28\_KOMPG:C4R7C4|C4R7C4\_KOMPG:C4QYT2|C4QYT2\_KOMPG:C4R8Q8|C4R8Q8\_KOMPG:C4R7V4|C4R7V4\_KOMPG:C4R110|C4R110\_KOMPG:C4R8R8|C4R8R8\_KOMPG:C4QYX9|C4QYX9\_KOMPG:C4R7Y5|C4R7Y5\_KOMPG:C4R3V0|C4R3V0\_KOMPG:C4QWE0|C4QWE0\_KOMPG:C4QVT4|C4QVT4\_KOMPG:C4QY81|C4QY81\_KOMPG:C4QZ71|C4QZ71\_KOMPG:C4QXU2|C4QXU2\_KOMPG:C4R4A5|C4R4A5\_KOMPG:C4R6X6|C4R6X6\_KOMPG:C4QV46|C4QV46\_KOMPG:C4R5J2|C4R5J2\_KOMPG:C4R6H1|C4R6H1\_KOMPG:C4QY11|C4QY11\_KOMPG:C4R8Q7|C4R8Q7\_KOMPG:C4R4M7|C4R4M7\_KOMPG:C4R7S0|C4R7S0\_KOMPG:C4R666|C4R666\_KOMPG:C4QVX7|C4QVX7\_KOMPG:C4R4H9|C4R4H9\_KOMPG:C4R681|C4R681\_KOMPG:C4R9E2|C4R9E2\_KOMPG:C4QXZ3|C4QXZ3\_KOMPG:C4R6A7|C4R6A7\_KOMPG:C4QZ47|C4QZ47\_KOMPG:C4QY85|C4QY85\_KOMPG:C4QW89|C4QW89\_KOMPG:C4R6J7|C4R6J7\_KOMPG:C4QX56|C4QX56\_KOMPG:C4QV98|C4QV98\_KOMPG:C4R6D1|C4R6D1\_KOMPG:C4R839|C4R839\_KOMPG:C4R1G3|C4R1G3\_KOMPG:C4QYA5|C4QYA5\_KOMPG:C4R950|C4R950\_KOMPG:C4R275|C4R275\_KOMPG:C4R1H8|C4R1H8\_KOMPG:C4R240|C4R240\_KOMPG:C4R693|C4R693\_KOMPG:C4QYI9|C4QYI9\_KOMPG:C4R322|C4R322\_KOMPG:C4R417|C4R417\_KOMPG:C4R7I9|C4R7I9\_KOMPG:C4QV86|C4QV86\_KOMPG:Q9Y751|ATG26\_KOMPG:C4R7D0|C4R7D0\_KOMPG:C4R8D0|C4R8D0\_KOMPG:C4QX46|C4QX46\_KOMPG:C4R998|C4R998\_KOMPG:C4QWP6|C4QWP6\_KOMPG:C4R0U8|C4R0U8\_KOMPG:C4R6L7|C4R6L7\_KOMPG:C4R5H5|C4R5H5\_KOMPG:C4R7W9|C4R7W9\_KOMPG:C4R6B0|OXDD\_KOMPG:C4QVY5|C4QVY5\_KOMPG:C4QZC6|C4QZC6\_KOMPG:C4R178|C4R178\_KOMPG:C4R2N2|C4R2N2\_KOMPG:C4R2W4|C4R2W4\_KOMPG:C4QXB6|C4QXB6\_KOMPG:C4R5R5|C4R5R5\_KOMPG:C4QYQ0|C4QYQ0\_KOMPG:C4QW41|C4QW41\_KOMPG:C4R9D7|C4R9D7\_KOMPG:C4R5I2|C4R5I2\_KOMPG:C4QW95|C4QW95\_KOMPG:C4R4T4|C4R4T4\_KOMPG:C4R0V9|C4R0V9\_KOMPG:C4QVY7|C4QVY7\_KOMPG:C4R1G6|C4R1G6\_KOMPG:C4R3U3|C4R3U3\_KOMPG:C4R1S9|C4R1S9\_KOMPG:C4QXT4|C4QXT4\_KOMPG:C4R1H2|C4R1H2\_KOMPG:C4R5C2|C4R5C2\_KOMPG:C4R374|C4R374\_KOMPG:C4R148|C4R148\_KOMPG:C4QZS7|C4QZS7\_KOMPG:C4R0Q9|C4R0Q9\_KOMPG:C4R2Z7|C4R2Z7\_KOMPG:C4R299|C4R299\_KOMPG:C4QYA1|C4QYA1\_KOMPG:C4R9G2|C4R9G2\_KOMPG:C4QV05|C4QV05\_KOMPG:C4QXX0|C4QXX0\_KOMPG:C4QZQ2|C4QZQ2\_KOMPG:C4R3R7|C4R3R7\_KOMPG:C4QVH2|C4QVH2\_KOMPG:C4R2T0|C4R2T0\_KOMPG:C4R4P7|C4R4P7\_KOMPG:C4QX70|C4QX70\_KOMPG:C4R3W8|C4R3W8\_KOMPG:C4R5V4|C4R5V4\_KOMPG:C4R7Z8|BMT4\_KOMPG:C4QY10|C4QY10\_KOMPG:C4R5H2|C4R5H2\_KOMPG:C4QVM6|C4QVM6\_KOMPG:C4R6N8|C4R6N8\_KOMPG:C4QYG6|C4QYG6\_KOMPG:C4QYE1|C4QYE1\_KOMPG:C4R8L6|C4R8L6\_KOMPG:C4QVT8|C4QVT8\_KOMPG:C4R4N1|C4R4N1\_KOMPG:C4R1D2|C4R1D2\_KOMPG:C4R7Z0|C4R7Z0\_KOMPG:C4QVM4|C4QVM4\_KOMPG:C4QY44|C4QY44\_KOMPG:C4R0S8|C4R0S8\_KOMPG:C4R1L3|C4R1L3\_KOMPG:C4R442|C4R442\_KOMPG:C4R0X2|C4R0X2\_KOMPG:C4R7M7|C4R7M7\_KOMPG:C4R8A4|C4R8A4\_KOMPG:C4R7N4|C4R7N4\_KOMPG:C4QVM0|C4QVM0\_KOMPG:C4R2R9|C4R2R9\_KOMPG:C4QVZ1|C4QVZ1\_KOMPG:C4R8V2|C4R8V2\_KOMPG:C4R2E4|C4R2E4\_KOMPG:C4QZQ6|C4QZQ6\_KOMPG:C4QZH9|C4QZH9\_KOMPG:C4R4Q3|C4R4Q3\_KOMPG:C4QZN7|C4QZN7\_KOMPG:C4QXI3|C4QXI3\_KOMPG:C4QZW5|C4QZW5\_KOMPG:C4R5W5|C4R5W5\_KOMPG:C4R3D7|C4R3D7\_KOMPG:C4R925|C4R925\_KOMPG:C4QX96|C4QX96\_KOMPG:C4QVX3|C4QVX3\_KOMPG:C4QYQ4|C4QYQ4\_KOMPG:C4R6D6|C4R6D6\_KOMPG:C4R4B1|C4R4B1\_KOMPG:C4QWF6|C4QWF6\_KOMPG:C4R4F5|C4R4F5\_KOMPG:C4R821|C4R821\_KOMPG:C4R4N2|C4R4N2\_KOMPG:C4R7K4|C4R7K4\_KOMPG:C4QYU7|C4QYU7\_KOMPG:C4R0E3|C4R0E3\_KOMPG:C4QZB5|C4QZB5\_KOMPG:C4QZY1|C4QZY1\_KOMPG:C4R7Q2|C4R7Q2\_KOMPG:C4QZT7|C4QZT7\_KOMPG:C4R407|C4R407\_KOMPG:C4R1R6|C4R1R6\_KOMPG:C4R1D5|C4R1D5\_KOMPG:C4R5B1|C4R5B1\_KOMPG:C4QZB4|C4QZB4\_KOMPG:C4R332|C4R332\_KOMPG:C4R1R5|C4R1R5\_KOMPG:C4R4X6|C4R4X6\_KOMPG:C4QWI8|C4QWI8\_KOMPG:C4R5Z3|C4R5Z3\_KOMPG:C4R583|C4R583\_KOMPG:C4R467|C4R467\_KOMPG:C4R5C0|C4R5C0\_KOMPG:C4R4V3|C4R4V3\_KOMPG:C4QYS3|C4QYS3\_KOMPG:C4QXY5|C4QXY5\_KOMPG:C4R8J3|C4R8J3\_KOMPG:C4R787|C4R787\_KOMPG:C4R7D8|C4R7D8\_KOMPG:C4R751|C4R751\_KOMPG:C4QXW6|C4QXW6\_KOMPG:C4QWE3|C4QWE3\_KOMPG:C4QVA0|C4QVA0\_KOMPG:C4R534|C4R534\_KOMPG:C4QZ39|C4QZ39\_KOMPG:C4R5W3|C4R5W3\_KOMPG:C4R9C2|C4R9C2\_KOMPG:C4R622|C4R622\_KOMPG:C4R6V5|C4R6V5\_KOMPG:C4R6T8|C4R6T8\_KOMPG:C4R7Y2|C4R7Y2\_KOMPG:C4R2E9|C4R2E9\_KOMPG:C4QXU3|C4QXU3\_KOMPG:C4QVD3|C4QVD3\_KOMPG:C4R7K1|C4R7K1\_KOMPG:C4QZT4|C4QZT4\_KOMPG:C4R717|C4R717\_KOMPG:C4R657|C4R657\_KOMPG:C4R5R4|C4R5R4\_KOMPG:C4R0W3|C4R0W3\_KOMPG:C4R5I5|C4R5I5\_KOMPG:C4QZF0|C4QZF0\_KOMPG:C4R3A0|C4R3A0\_KOMPG:C4QW32|C4QW32\_KOMPG:C4QVT3|C4QVT3\_KOMPG:C4R1Q0|C4R1Q0\_KOMPG:C4R3B8|C4R3B8\_KOMPG:C4R921|C4R921\_KOMPG:C4QZI1|C4QZI1\_KOMPG:C4R070|C4R070\_KOMPG:C4R6L2|C4R6L2\_KOMPG:C4QZ48|C4QZ48\_KOMPG:C4R4S3|C4R4S3\_KOMPG:C4QWF2|C4QWF2\_KOMPG:C4R5L2|C4R5L2\_KOMPG:C4R460|C4R460\_KOMPG:C4R8H7|C4R8H7\_KOMPG:C4R0D7|C4R0D7\_KOMPG:C4QX81|C4QX81\_KOMPG:C4R3W5|C4R3W5\_KOMPG:C4QY03|C4QY03\_KOMPG:C4R8H0|C4R8H0\_KOMPG:C4R981|C4R981\_KOMPG:C4R2Z1|C4R2Z1\_KOMPG:C4QXW5|C4QXW5\_KOMPG:C4QW16|C4QW16\_KOMPG:C4R677|C4R677\_KOMPG:C4QXD5|C4QXD5\_KOMPG:C4R721|C4R721\_KOMPG:C4R3M1|C4R3M1\_KOMPG:C4R5W9|C4R5W9\_KOMPG:C4R8W9|C4R8W9\_KOMPG:C4R458|C4R458\_KOMPG |  |  | DB Search |
| FVVP | 23.15 | 460.2686 | 4 | -7.54 | 461.2712 | 28.36 | 9923 | 0 | 0 | 0 | C4R6W3|C4R6W3\_KOMPG:C4R2M0|C4R2M0\_KOMPG:C4R034|C4R034\_KOMPG:C4R0Z7|C4R0Z7\_KOMPG:C4QZQ8|C4QZQ8\_KOMPG:Q9Y751|ATG26\_KOMPG:C4QYR9|C4QYR9\_KOMPG:C4R5T4|C4R5T4\_KOMPG:C4R187|C4R187\_KOMPG:C4R0V8|C4R0V8\_KOMPG:C4QXK7|C4QXK7\_KOMPG:C4R7D8|C4R7D8\_KOMPG:C4R2V7|C4R2V7\_KOMPG:C4R814|C4R814\_KOMPG:C4QWL0|C4QWL0\_KOMPG:C4R176|C4R176\_KOMPG:C4R4J1|C4R4J1\_KOMPG |  |  | DB Search |
| LTPMT | 23.09 | 561.2832 | 5 | -6.07 | 562.2857 | 20.23 | 7638 | 4.29e3 | 1 | 1 | C4R1W6|C4R1W6\_KOMPG |  |  | DB Search |
| AIAGSIGIG | 23.09 | 757.4333 | 9 | -4.57 | 758.4353 | 27.03 | 9540 | 2.32e3 | 1 | 1 | C4R9E3|C4R9E3\_KOMPG |  |  | DB Search |
| DAIP | 23.06 | 414.2114 | 4 | 0.68 | 415.218 | 12.91 | 5068 | 2.99e2 | 1 | 1 | C4R8T0|C4R8T0\_KOMPG:C4R8W0|C4R8W0\_KOMPG:C4R6N9|C4R6N9\_KOMPG:C4R2W9|C4R2W9\_KOMPG:C4QVL2|C4QVL2\_KOMPG:C4QYR3|C4QYR3\_KOMPG:C4QZW1|C4QZW1\_KOMPG:C4QVA1|C4QVA1\_KOMPG:C4R5L9|C4R5L9\_KOMPG:C4R0V4|C4R0V4\_KOMPG:C4R6Q5|C4R6Q5\_KOMPG:C4R093|C4R093\_KOMPG |  |  | DB Search |
| DALP | 23.06 | 414.2114 | 4 | 0.68 | 415.218 | 12.91 | 5068 | 2.99e2 | 1 | 1 | C4R284|C4R284\_KOMPG:C4R295|C4R295\_KOMPG:C4R2M6|C4R2M6\_KOMPG:C4R152|C4R152\_KOMPG:C4QV42|C4QV42\_KOMPG:C4R8C0|C4R8C0\_KOMPG:C4QZ59|C4QZ59\_KOMPG:C4R2Q9|C4R2Q9\_KOMPG:C4R7K9|C4R7K9\_KOMPG:C4R734|C4R734\_KOMPG:C4QVZ1|C4QVZ1\_KOMPG:C4R748|C4R748\_KOMPG:C4R0G5|C4R0G5\_KOMPG:C4QVX8|C4QVX8\_KOMPG:C4R867|C4R867\_KOMPG:C4QX72|C4QX72\_KOMPG:C4R5N2|C4R5N2\_KOMPG:C4R0S4|C4R0S4\_KOMPG:C4R2F9|C4R2F9\_KOMPG:C4R0A2|C4R0A2\_KOMPG:C4R7W4|C4R7W4\_KOMPG:C4QX33|C4QX33\_KOMPG:C4R4M3|C4R4M3\_KOMPG |  |  | DB Search |
| SAGIQ | 23.04 | 474.2438 | 5 | -3.9 | 475.248 | 3.16 | 1372 | 1.15e3 | 1 | 1 | C4R0P1|C4R0P1\_KOMPG |  |  | DB Search |
| SAGLQ | 23.04 | 474.2438 | 5 | -3.9 | 475.248 | 3.16 | 1372 | 1.15e3 | 1 | 1 | C4R7W9|C4R7W9\_KOMPG:C4R816|C4R816\_KOMPG:C4QYX0|C4QYX0\_KOMPG |  |  | DB Search |
| ASAI | 22.98 | 360.2009 | 4 | -6.45 | 361.2049 | 8.68 | 3069 | 1.41e4 | 1 | 1 | C4R6K9|C4R6K9\_KOMPG:C4R9F1|C4R9F1\_KOMPG:C4R2X5|C4R2X5\_KOMPG:C4R7F2|C4R7F2\_KOMPG:C4R4H2|C4R4H2\_KOMPG:C4R5C7|C4R5C7\_KOMPG:C4R0X4|C4R0X4\_KOMPG:C4R6C2|PEX1\_KOMPG:C4QX96|C4QX96\_KOMPG:C4QXN3|C4QXN3\_KOMPG:C4QVH6|C4QVH6\_KOMPG:C4QXW7|C4QXW7\_KOMPG:C4R517|C4R517\_KOMPG:C4R1P3|C4R1P3\_KOMPG:C4R803|C4R803\_KOMPG:C4R3J7|C4R3J7\_KOMPG:C4R705|C4R705\_KOMPG:C4R1X5|C4R1X5\_KOMPG:C4R3M7|C4R3M7\_KOMPG:C4R0L7|C4R0L7\_KOMPG:C4QXB2|C4QXB2\_KOMPG:C4R3I3|C4R3I3\_KOMPG:C4R109|C4R109\_KOMPG:C4R6Z7|C4R6Z7\_KOMPG:C4R6B8|C4R6B8\_KOMPG:C4R2S6|C4R2S6\_KOMPG:C4QYZ0|C4QYZ0\_KOMPG:C4QWS1|C4QWS1\_KOMPG:C4QXG8|C4QXG8\_KOMPG:C4QY39|C4QY39\_KOMPG:C4QYX5|C4QYX5\_KOMPG:C4QXF2|C4QXF2\_KOMPG:C4QZF5|C4QZF5\_KOMPG:C4QZM9|C4QZM9\_KOMPG:C4R7R0|C4R7R0\_KOMPG:C4R5N3|C4R5N3\_KOMPG:C4QY80|C4QY80\_KOMPG:C4QV16|C4QV16\_KOMPG:C4R3V3|C4R3V3\_KOMPG:C4R0L2|C4R0L2\_KOMPG:C4R7T5|C4R7T5\_KOMPG |  |  | DB Search |
| ASAL | 22.98 | 360.2009 | 4 | -6.45 | 361.2049 | 8.68 | 3069 | 1.41e4 | 1 | 1 | C4R2T2|C4R2T2\_KOMPG:C4R8W0|C4R8W0\_KOMPG:C4R418|C4R418\_KOMPG:C4QWB0|C4QWB0\_KOMPG:C4R0S7|C4R0S7\_KOMPG:C4R1U2|C4R1U2\_KOMPG:C4QZ79|C4QZ79\_KOMPG:C4QY67|C4QY67\_KOMPG:C4R1B1|C4R1B1\_KOMPG:C4QYP0|C4QYP0\_KOMPG:C4R0U8|C4R0U8\_KOMPG:Q9P4D0|SEC17\_KOMPG:C4R313|C4R313\_KOMPG:C4R2A7|C4R2A7\_KOMPG:C4R7M7|C4R7M7\_KOMPG:C4R108|C4R108\_KOMPG:C4R5H5|C4R5H5\_KOMPG:C4R9D8|C4R9D8\_KOMPG:C4R9G1|C4R9G1\_KOMPG:C4QYQ7|C4QYQ7\_KOMPG:C4QZK7|C4QZK7\_KOMPG:C4QWK2|C4QWK2\_KOMPG:C4QWZ4|C4QWZ4\_KOMPG:C4R0L6|C4R0L6\_KOMPG:C4QYR3|C4QYR3\_KOMPG:C4QWB9|C4QWB9\_KOMPG:C4R3B0|C4R3B0\_KOMPG:C4R0I6|C4R0I6\_KOMPG:C4R7J5|C4R7J5\_KOMPG:C4R354|C4R354\_KOMPG:C4QX05|C4QX05\_KOMPG:C4R526|C4R526\_KOMPG:C4R366|C4R366\_KOMPG:C4QXC2|C4QXC2\_KOMPG:C4R1Q2|C4R1Q2\_KOMPG:C4QYU4|C4QYU4\_KOMPG:C4R1S9|C4R1S9\_KOMPG:C4QYN3|C4QYN3\_KOMPG:C4R7N3|C4R7N3\_KOMPG:C4QZ23|C4QZ23\_KOMPG:C4QY22|C4QY22\_KOMPG:C4R162|C4R162\_KOMPG:C4R2Z7|C4R2Z7\_KOMPG:C4QXA9|C4QXA9\_KOMPG:C4R7Y4|C4R7Y4\_KOMPG:C4R654|C4R654\_KOMPG:C4R5P2|C4R5P2\_KOMPG:C4R7F6|C4R7F6\_KOMPG:C4R5M8|C4R5M8\_KOMPG:C4R4A2|C4R4A2\_KOMPG:C4QXE6|C4QXE6\_KOMPG:C4QY04|C4QY04\_KOMPG:C4QV96|C4QV96\_KOMPG:C4R3R1|C4R3R1\_KOMPG:C4R931|C4R931\_KOMPG:C4R1Y9|C4R1Y9\_KOMPG:C4R604|C4R604\_KOMPG:C4QWW8|C4QWW8\_KOMPG:C4QXA6|C4QXA6\_KOMPG:C4R5J6|C4R5J6\_KOMPG:C4QV80|C4QV80\_KOMPG:C4R4H7|C4R4H7\_KOMPG:C4R1D9|C4R1D9\_KOMPG:C4QZP4|C4QZP4\_KOMPG:C4R721|C4R721\_KOMPG:C4R2C0|C4R2C0\_KOMPG:C4R7G1|C4R7G1\_KOMPG |  |  | DB Search |
| PIGGI | 22.98 | 455.2744 | 5 | -4.8 | 456.2783 | 20.80 | 7682 | 5.46e3 | 1 | 1 | C4R789|C4R789\_KOMPG:C4R713|C4R713\_KOMPG |  |  | DB Search |
| PIGGL | 22.98 | 455.2744 | 5 | -4.8 | 456.2783 | 20.80 | 7682 | 5.46e3 | 1 | 1 | C4R2U7|C4R2U7\_KOMPG:C4R2I9|C4R2I9\_KOMPG:C4QVX7|C4QVX7\_KOMPG:C4R0B4|C4R0B4\_KOMPG:C4QY14|C4QY14\_KOMPG |  |  | DB Search |
| PLGGI | 22.98 | 455.2744 | 5 | -4.8 | 456.2783 | 20.80 | 7682 | 5.46e3 | 1 | 1 | C4QXS7|C4QXS7\_KOMPG |  |  | DB Search |
| PLGGL | 22.98 | 455.2744 | 5 | -4.8 | 456.2783 | 20.80 | 7682 | 5.46e3 | 1 | 1 | C4QWR7|C4QWR7\_KOMPG:C4R1D2|C4R1D2\_KOMPG:C4QYE7|C4QYE7\_KOMPG |  |  | DB Search |
| SIGQAGE | 22.97 | 660.3079 | 7 | -6.95 | 661.3089 | 12.24 | 4780 | 1.27e2 | 1 | 1 | C4R5L7|C4R5L7\_KOMPG:C4R2Q9|C4R2Q9\_KOMPG |  |  | DB Search |
| FSNTLI | 22.94 | 693.3698 | 6 | -4.9 | 694.3719 | 28.23 | 9887 | 0 | 0 | 0 | C4QV93|C4QV93\_KOMPG |  |  | DB Search |
| VDPSGSY | 22.91 | 723.3075 | 7 | 6.4 | 724.3176 | 8.57 | 3261 | 7.31e3 | 1 | 1 | C4QZX2|C4QZX2\_KOMPG |  |  | DB Search |
| VFADLITQ(+0.98) | 22.9 | 906.4698 | 8 | -3.43 | 907.4717 | 25.12 | 9035 | 2.73e2 | 1 | 1 | C4QXW1|C4QXW1\_KOMPG | Deamidation (NQ) | Q8:Deamidation (NQ):1000 | DB Search |
| LC(-1.01)LVGGS | 22.88 | 646.3234 | 7 | -2.23 | 647.3276 | 8.48 | 3231 | 0 | 0 | 0 | C4QXE6|C4QXE6\_KOMPG | Half of a disulfide bridge | C2:Half of a disulfide bridge:1000 | DB Search |
| SIPT | 22.87 | 416.2271 | 4 | -6.88 | 417.2305 | 14.64 | 5767 | 5.65e3 | 1 | 1 | C4R6M4|C4R6M4\_KOMPG:C4QZD7|C4QZD7\_KOMPG:C4QXF7|C4QXF7\_KOMPG:C4R3I8|C4R3I8\_KOMPG:C4R1J5|C4R1J5\_KOMPG:C4R3H3|C4R3H3\_KOMPG:C4R744|C4R744\_KOMPG:C4QZN9|C4QZN9\_KOMPG:C4R310|C4R310\_KOMPG:C4R4D8|C4R4D8\_KOMPG:C4R289|C4R289\_KOMPG:C4R980|C4R980\_KOMPG:C4QXI0|C4QXI0\_KOMPG:C4R1V3|C4R1V3\_KOMPG:C4R5U9|C4R5U9\_KOMPG:C4QYP0|C4QYP0\_KOMPG:C4R8H7|C4R8H7\_KOMPG:C4R7T4|C4R7T4\_KOMPG:C4R286|C4R286\_KOMPG:C4QW71|C4QW71\_KOMPG:C4R468|C4R468\_KOMPG:C4R4X8|C4R4X8\_KOMPG:C4R0G9|C4R0G9\_KOMPG:C4R7E8|C4R7E8\_KOMPG:C4QWT2|C4QWT2\_KOMPG:C4QVG3|C4QVG3\_KOMPG:C4R7H0|C4R7H0\_KOMPG:C4R3L4|C4R3L4\_KOMPG:C4R7K1|C4R7K1\_KOMPG:C4QZT4|C4QZT4\_KOMPG:C4R735|C4R735\_KOMPG:C4R1S3|C4R1S3\_KOMPG:C4R0Y7|C4R0Y7\_KOMPG:C4R901|C4R901\_KOMPG:C4QXS6|C4QXS6\_KOMPG:C4QW33|C4QW33\_KOMPG:C4R087|C4R087\_KOMPG |  |  | DB Search |
| SLPT | 22.87 | 416.2271 | 4 | -6.88 | 417.2305 | 14.64 | 5767 | 5.65e3 | 1 | 1 | C4R3X2|C4R3X2\_KOMPG:C4QXT2|C4QXT2\_KOMPG:C4R8G4|C4R8G4\_KOMPG:C4R532|C4R532\_KOMPG:C4R8N1|C4R8N1\_KOMPG:C4R368|C4R368\_KOMPG:C4R1U1|C4R1U1\_KOMPG:C4R291|C4R291\_KOMPG:C4QWI1|C4QWI1\_KOMPG:C4QYC7|C4QYC7\_KOMPG:C4R6C5|C4R6C5\_KOMPG:C4QYT2|C4QYT2\_KOMPG:C4R3D5|C4R3D5\_KOMPG:C4R705|C4R705\_KOMPG:C4QVW1|C4QVW1\_KOMPG:C4R4Q0|C4R4Q0\_KOMPG:C4QXU4|C4QXU4\_KOMPG:C4R6Z7|C4R6Z7\_KOMPG:C4QV03|C4QV03\_KOMPG:C4R5R6|C4R5R6\_KOMPG:C4QYF5|C4QYF5\_KOMPG:C4QXI4|C4QXI4\_KOMPG:C4R6F6|C4R6F6\_KOMPG:C4QW54|C4QW54\_KOMPG:C4R2E4|C4R2E4\_KOMPG:C4R3P6|C4R3P6\_KOMPG:C4QYZ4|C4QYZ4\_KOMPG:C4R5N1|C4R5N1\_KOMPG:C4R724|C4R724\_KOMPG:C4R377|C4R377\_KOMPG:C4R0J8|C4R0J8\_KOMPG:C4R6A7|C4R6A7\_KOMPG:C4QZ59|C4QZ59\_KOMPG:C4QVB3|C4QVB3\_KOMPG:C4R7N8|C4R7N8\_KOMPG:C4QZX9|C4QZX9\_KOMPG:C4R919|C4R919\_KOMPG:C4R2H0|C4R2H0\_KOMPG:C4QVH3|C4QVH3\_KOMPG:C4QV87|C4QV87\_KOMPG:C4QYP4|C4QYP4\_KOMPG:C4QXB4|C4QXB4\_KOMPG:C4QVS6|C4QVS6\_KOMPG:C4R4Z7|C4R4Z7\_KOMPG:C4R1N6|C4R1N6\_KOMPG:C4QY65|C4QY65\_KOMPG:C4R6Y6|C4R6Y6\_KOMPG:C4QYT1|C4QYT1\_KOMPG:C4R220|C4R220\_KOMPG:C4R527|C4R527\_KOMPG:C4QVK6|C4QVK6\_KOMPG:C4QY04|C4QY04\_KOMPG:C4QWV7|C4QWV7\_KOMPG:C4R7S4|C4R7S4\_KOMPG:C4R254|C4R254\_KOMPG:C4R332|C4R332\_KOMPG:C4R861|C4R861\_KOMPG:C4QYG1|C4QYG1\_KOMPG:C4R5H2|C4R5H2\_KOMPG:C4R2W8|C4R2W8\_KOMPG:C4QYA3|C4QYA3\_KOMPG:C4QZP4|C4QZP4\_KOMPG:C4QVM6|C4QVM6\_KOMPG:C4R545|C4R545\_KOMPG:C4R556|C4R556\_KOMPG |  |  | DB Search |
| EF | 22.86 | 294.1216 | 2 | -6.92 | 295.1261 | 9.67 | 3652 | 3.06e4 | 1 | 1 | C4QX82|C4QX82\_KOMPG:C4QZ38|C4QZ38\_KOMPG:C4R8H4|C4R8H4\_KOMPG:C4R9E5|C4R9E5\_KOMPG:C4R142|C4R142\_KOMPG:C4R6G4|C4R6G4\_KOMPG:C4R554|C4R554\_KOMPG:C4R7Q3|C4R7Q3\_KOMPG:C4QV84|C4QV84\_KOMPG:C4QZZ8|C4QZZ8\_KOMPG:C4R1N5|C4R1N5\_KOMPG:C4R2Z6|C4R2Z6\_KOMPG:C4QW53|C4QW53\_KOMPG:C4R2A4|C4R2A4\_KOMPG:C4QYG5|C4QYG5\_KOMPG:C4R4G9|OXDA\_KOMPG:C4R912|C4R912\_KOMPG:C4R4X8|C4R4X8\_KOMPG:C4QYE9|C4QYE9\_KOMPG:C4QYJ8|C4QYJ8\_KOMPG:C4R042|C4R042\_KOMPG:C4R4C7|C4R4C7\_KOMPG:C4R5J2|C4R5J2\_KOMPG:C4R6E1|C4R6E1\_KOMPG:C4R3T0|C4R3T0\_KOMPG:C4R4A0|C4R4A0\_KOMPG:C4R0G2|C4R0G2\_KOMPG:C4R349|C4R349\_KOMPG:C4R8I1|C4R8I1\_KOMPG:C4QYI8|C4QYI8\_KOMPG:C4R8T8|C4R8T8\_KOMPG:C4R9E2|C4R9E2\_KOMPG:C4QWR3|C4QWR3\_KOMPG:C4QXL0|C4QXL0\_KOMPG:C4R0B6|C4R0B6\_KOMPG:C4R5C1|C4R5C1\_KOMPG:C4R450|C4R450\_KOMPG:C4R877|C4R877\_KOMPG:C4R688|C4R688\_KOMPG:C4QXG2|C4QXG2\_KOMPG:C4R8S4|C4R8S4\_KOMPG:C4R7N8|C4R7N8\_KOMPG:C4QW84|C4QW84\_KOMPG:C4R0V8|C4R0V8\_KOMPG:C4QWF6|C4QWF6\_KOMPG:C4R959|C4R959\_KOMPG:C4R2W1|C4R2W1\_KOMPG:C4R8C1|C4R8C1\_KOMPG:C4R5F7|C4R5F7\_KOMPG:C4R093|C4R093\_KOMPG:C4R6N0|C4R6N0\_KOMPG:C4QZX0|C4QZX0\_KOMPG:C4R0Q0|C4R0Q0\_KOMPG:C4R7S9|GET3\_KOMPG:C4R129|C4R129\_KOMPG:C4QY04|C4QY04\_KOMPG:C4R692|C4R692\_KOMPG:C4QVT9|C4QVT9\_KOMPG:C4R4T5|C4R4T5\_KOMPG:C4QZF2|C4QZF2\_KOMPG:C4R3L2|C4R3L2\_KOMPG:C4R0J5|C4R0J5\_KOMPG:C4R565|C4R565\_KOMPG:C4QWS9|C4QWS9\_KOMPG:C4QZT6|C4QZT6\_KOMPG:C4R503|C4R503\_KOMPG:C4QYK4|C4QYK4\_KOMPG:C4R8D9|C4R8D9\_KOMPG:C4R625|C4R625\_KOMPG:C4R2W0|C4R2W0\_KOMPG |  |  | DB Search |
| E(-18.01)VCC(-1.01)AGS | 22.83 | 648.2122 | 7 | -3.74 | 649.2154 | 1.76 | 727 | 1.1e4 | 1 | 1 | C4R664|C4R664\_KOMPG:C4R0W4|C4R0W4\_KOMPG:C4R6P6|C4R6P6\_KOMPG | Pyro-glu from E, Half of a disulfide bridge | E1:Pyro-glu from E:1000 C4:Half of a disulfide bridge:0 | DB Search |
| MLLTT | 22.82 | 577.3145 | 5 | -6.02 | 578.3169 | 21.75 | 8004 | 0 | 0 | 0 | C4R6X0|C4R6X0\_KOMPG:C4R1V8|C4R1V8\_KOMPG |  |  | DB Search |
| HPAA | 22.78 | 394.1964 | 4 | 8.24 | 395.206 | 15.22 | 6038 | 1e3 | 1 | 1 | C4R5M5|C4R5M5\_KOMPG:C4R418|C4R418\_KOMPG:C4QZ23|C4QZ23\_KOMPG:C4R646|C4R646\_KOMPG:C4R778|C4R778\_KOMPG:C4QZK6|C4QZK6\_KOMPG:C4R4Y3|C4R4Y3\_KOMPG:C4R510|C4R510\_KOMPG:C4QZJ2|C4QZJ2\_KOMPG:C4R1Y3|C4R1Y3\_KOMPG:C4QXV8|C4QXV8\_KOMPG:C4R7S3|C4R7S3\_KOMPG:C4R2A7|C4R2A7\_KOMPG:C4R9D8|C4R9D8\_KOMPG |  |  | DB Search |
| GVSI | 22.74 | 374.2165 | 4 | -2.51 | 375.2219 | 13.91 | 5529 | 2.07e3 | 1 | 1 | C4R8W0|C4R8W0\_KOMPG:C4R586|C4R586\_KOMPG:C4R5S6|C4R5S6\_KOMPG:C4R6P4|C4R6P4\_KOMPG:C4QVB3|C4QVB3\_KOMPG:C4R0Y9|C4R0Y9\_KOMPG:C4R6L2|C4R6L2\_KOMPG:C4R4V3|C4R4V3\_KOMPG:C4R2K6|C4R2K6\_KOMPG:C4QZ79|C4QZ79\_KOMPG:C4R8D0|C4R8D0\_KOMPG:C4R6N6|C4R6N6\_KOMPG:C4R4S8|C4R4S8\_KOMPG:C4R561|C4R561\_KOMPG:C4R4Q8|C4R4Q8\_KOMPG:C4R1Q3|C4R1Q3\_KOMPG:C4R6U1|C4R6U1\_KOMPG:C4R912|C4R912\_KOMPG:C4QV45|C4QV45\_KOMPG:C4QV56|C4QV56\_KOMPG:C4QZR1|C4QZR1\_KOMPG:C4QZ32|C4QZ32\_KOMPG:C4R386|C4R386\_KOMPG:C4QZB5|C4QZB5\_KOMPG:C4R1C3|C4R1C3\_KOMPG:C4R7K1|C4R7K1\_KOMPG:C4R2R9|C4R2R9\_KOMPG:C4R043|C4R043\_KOMPG:C4QZF0|C4QZF0\_KOMPG:C4QXB6|C4QXB6\_KOMPG:C4R4X3|C4R4X3\_KOMPG:C4QXN5|C4QXN5\_KOMPG:C4QVU6|C4QVU6\_KOMPG:C4R6N8|C4R6N8\_KOMPG:C4QX33|C4QX33\_KOMPG:C4QWL0|C4QWL0\_KOMPG |  |  | DB Search |
| GVSL | 22.74 | 374.2165 | 4 | -2.51 | 375.2219 | 13.91 | 5529 | 2.07e3 | 1 | 1 | C4R2V8|C4R2V8\_KOMPG:C4R0N9|C4R0N9\_KOMPG:C4QZ58|C4QZ58\_KOMPG:C4R5Z3|C4R5Z3\_KOMPG:C4QWN8|C4QWN8\_KOMPG:C4QYV4|C4QYV4\_KOMPG:C4QWU9|C4QWU9\_KOMPG:C4QZZ6|C4QZZ6\_KOMPG:C4R5L3|C4R5L3\_KOMPG:C4R4Q7|C4R4Q7\_KOMPG:C4R463|C4R463\_KOMPG:C4R5D1|C4R5D1\_KOMPG:C4R1T4|C4R1T4\_KOMPG:C4R687|C4R687\_KOMPG:C4R8X0|C4R8X0\_KOMPG:C4R6R7|C4R6R7\_KOMPG:C4QXJ8|C4QXJ8\_KOMPG:C4QXD3|C4QXD3\_KOMPG:C4QZX2|C4QZX2\_KOMPG:C4R5S2|C4R5S2\_KOMPG:C4QZR8|C4QZR8\_KOMPG:C4R4B6|C4R4B6\_KOMPG:C4R7J5|C4R7J5\_KOMPG:C4R2Z8|C4R2Z8\_KOMPG:C4R0G7|C4R0G7\_KOMPG:C4R1Z2|C4R1Z2\_KOMPG:C4R354|C4R354\_KOMPG:C4R6T2|C4R6T2\_KOMPG:C4R2Q9|C4R2Q9\_KOMPG:C4QYU4|C4QYU4\_KOMPG:C4R4F1|C4R4F1\_KOMPG:C4R032|C4R032\_KOMPG:C4QZM1|C4QZM1\_KOMPG:C4QWY4|C4QWY4\_KOMPG:C4R0V2|C4R0V2\_KOMPG:C4R1N8|C4R1N8\_KOMPG:C4R7N6|C4R7N6\_KOMPG:C4R7H1|C4R7H1\_KOMPG:C4R553|C4R553\_KOMPG:C4R3I2|C4R3I2\_KOMPG:C4QZT7|C4QZT7\_KOMPG:C4R524|C4R524\_KOMPG:C4R2S2|C4R2S2\_KOMPG:C4R7L7|C4R7L7\_KOMPG:C4QYX5|C4QYX5\_KOMPG:C4R5H0|C4R5H0\_KOMPG:C4QUZ0|C4QUZ0\_KOMPG:C4R045|C4R045\_KOMPG:C4QWE7|C4QWE7\_KOMPG |  |  | DB Search |
| PQNAHEI | 22.73 | 807.3875 | 7 | 1.12 | 808.3937 | 23.85 | 8645 | 9.34e2 | 1 | 1 | C4R6X0|C4R6X0\_KOMPG |  |  | DB Search |
| SVIVLP | 22.7 | 626.4003 | 6 | -7.22 | 627.4015 | 39.05 | 12647 | 2.6e3 | 1 | 1 | C4R3K7|C4R3K7\_KOMPG |  |  | DB Search |
| VPTVD | 22.67 | 529.2748 | 5 | -8.92 | 530.276 | 9.43 | 3636 | 0 | 0 | 0 | C4R0P1|C4R0P1\_KOMPG |  |  | DB Search |
| SSSQQP | 22.63 | 632.2766 | 6 | -5.1 | 633.2791 | 9.63 | 3737 | 1.16e3 | 1 | 1 | C4R4T4|C4R4T4\_KOMPG |  |  | DB Search |
| ISGI | 22.6 | 388.2322 | 4 | -3.81 | 389.237 | 9.87 | 3804 | 6.08e3 | 1 | 1 | C4QYG9|C4QYG9\_KOMPG:C4QZD7|C4QZD7\_KOMPG:C4QUZ4|C4QUZ4\_KOMPG:C4R8H4|C4R8H4\_KOMPG:C4R0Z3|C4R0Z3\_KOMPG:C4R8X6|C4R8X6\_KOMPG:C4R357|C4R357\_KOMPG:C4R5B3|C4R5B3\_KOMPG:C4R8T2|C4R8T2\_KOMPG:C4R008|C4R008\_KOMPG:C4R8Y2|C4R8Y2\_KOMPG:C4QYV4|C4QYV4\_KOMPG:C4R5H1|C4R5H1\_KOMPG:C4QV09|C4QV09\_KOMPG:C4R4P0|C4R4P0\_KOMPG:C4R1Z1|C4R1Z1\_KOMPG:C4R663|C4R663\_KOMPG:C4R5Q6|C4R5Q6\_KOMPG:C4R8W8|C4R8W8\_KOMPG:C4R8W5|C4R8W5\_KOMPG:C4R555|C4R555\_KOMPG:C4QXU4|C4QXU4\_KOMPG:C4R6L8|C4R6L8\_KOMPG:C4R7Q2|C4R7Q2\_KOMPG:C4R6F4|C4R6F4\_KOMPG:C4R243|C4R243\_KOMPG:C4R021|C4R021\_KOMPG:C4QZE5|C4QZE5\_KOMPG:C4R599|C4R599\_KOMPG:C4R5R4|C4R5R4\_KOMPG:C4R5A6|C4R5A6\_KOMPG:C4R349|C4R349\_KOMPG:C4R4L3|C4R4L3\_KOMPG:C4R918|C4R918\_KOMPG:C4R5Y6|C4R5Y6\_KOMPG:C4R4X3|C4R4X3\_KOMPG:C4QV23|C4QV23\_KOMPG:C4R2J1|C4R2J1\_KOMPG |  |  | DB Search |
| ISGL | 22.6 | 388.2322 | 4 | -3.81 | 389.237 | 9.87 | 3804 | 6.08e3 | 1 | 1 | C4R5R3|C4R5R3\_KOMPG:C4R3U8|C4R3U8\_KOMPG:C4R479|C4R479\_KOMPG:C4R6Q0|C4R6Q0\_KOMPG:C4R5S6|C4R5S6\_KOMPG:C4QZH4|C4QZH4\_KOMPG:C4R6X0|C4R6X0\_KOMPG:C4R8C2|C4R8C2\_KOMPG:C4QVD5|C4QVD5\_KOMPG:C4QWJ0|C4QWJ0\_KOMPG:C4QVW3|C4QVW3\_KOMPG:C4R6C2|PEX1\_KOMPG:C4R8N7|C4R8N7\_KOMPG:C4R1Z3|C4R1Z3\_KOMPG:C4R1J4|C4R1J4\_KOMPG:C4R6M3|C4R6M3\_KOMPG:C4R6Q7|C4R6Q7\_KOMPG:C4QWA3|C4QWA3\_KOMPG:C4QYP6|C4QYP6\_KOMPG:C4R4G9|OXDA\_KOMPG:C4R6P1|C4R6P1\_KOMPG:C4R2D0|C4R2D0\_KOMPG:C4R463|C4R463\_KOMPG:C4R2T9|C4R2T9\_KOMPG:C4QV12|C4QV12\_KOMPG:C4QY25|C4QY25\_KOMPG:C4R874|C4R874\_KOMPG:C4R0Q6|C4R0Q6\_KOMPG:C4QV25|C4QV25\_KOMPG:C4QWQ4|C4QWQ4\_KOMPG:C4QWU0|C4QWU0\_KOMPG:C4QYK8|C4QYK8\_KOMPG:C4QVK5|C4QVK5\_KOMPG:C4R8M8|C4R8M8\_KOMPG:C4R953|C4R953\_KOMPG:C4R3D7|C4R3D7\_KOMPG:C4R6I5|C4R6I5\_KOMPG:C4R7E9|C4R7E9\_KOMPG:C4R5U8|C4R5U8\_KOMPG:C4R4Z9|C4R4Z9\_KOMPG:C4R839|C4R839\_KOMPG:C4QWE8|C4QWE8\_KOMPG:C4QXS3|C4QXS3\_KOMPG:C4R1M0|C4R1M0\_KOMPG:C4R1R1|C4R1R1\_KOMPG:C4R3B7|C4R3B7\_KOMPG:C4QYB6|C4QYB6\_KOMPG:C4QV45|C4QV45\_KOMPG:C4R4F5|C4R4F5\_KOMPG:C4QYA5|C4QYA5\_KOMPG:C4QVG3|C4QVG3\_KOMPG:C4R8E4|C4R8E4\_KOMPG:C4R931|C4R931\_KOMPG:C4R1G1|C4R1G1\_KOMPG:C4R4J4|C4R4J4\_KOMPG:C4R6E8|C4R6E8\_KOMPG:C4R545|C4R545\_KOMPG |  |  | DB Search |
| LSGI | 22.6 | 388.2322 | 4 | -3.81 | 389.237 | 9.87 | 3804 | 6.08e3 | 1 | 1 | C4R023|C4R023\_KOMPG:C4QZ58|C4QZ58\_KOMPG:C4QWB0|C4QWB0\_KOMPG:C4R0S7|C4R0S7\_KOMPG:C4R3D3|C4R3D3\_KOMPG:C4QX30|C4QX30\_KOMPG:C4R7P2|C4R7P2\_KOMPG:C4QY32|C4QY32\_KOMPG:C4R0X2|C4R0X2\_KOMPG:C4R7R3|C4R7R3\_KOMPG:C4QYI5|C4QYI5\_KOMPG:C4R284|C4R284\_KOMPG:C4R1X3|C4R1X3\_KOMPG:C4QYY0|C4QYY0\_KOMPG:C4QYU8|C4QYU8\_KOMPG:C4R548|C4R548\_KOMPG:C4QYR8|C4QYR8\_KOMPG:C4R6H0|C4R6H0\_KOMPG:C4QZQ7|C4QZQ7\_KOMPG:C4QWR8|C4QWR8\_KOMPG:C4QWZ8|C4QWZ8\_KOMPG:C4R3X8|C4R3X8\_KOMPG:C4R1K4|C4R1K4\_KOMPG:C4QZA2|C4QZA2\_KOMPG:C4R1S2|C4R1S2\_KOMPG:C4R6S3|C4R6S3\_KOMPG:C4R341|C4R341\_KOMPG:C4QXL0|C4QXL0\_KOMPG:C4QX27|C4QX27\_KOMPG:C4R3G8|C4R3G8\_KOMPG:C4QZW5|C4QZW5\_KOMPG:C4QWZ3|C4QWZ3\_KOMPG:C4R3F4|C4R3F4\_KOMPG:C4QV82|C4QV82\_KOMPG:C4R380|C4R380\_KOMPG:C4QVN1|C4QVN1\_KOMPG:C4QV92|C4QV92\_KOMPG:C4R0K1|C4R0K1\_KOMPG:C4QW40|C4QW40\_KOMPG:C4QZ83|C4QZ83\_KOMPG:C4R5E0|C4R5E0\_KOMPG:C4R887|C4R887\_KOMPG:C4QW81|C4QW81\_KOMPG:C4R808|C4R808\_KOMPG:C4R2M3|C4R2M3\_KOMPG:C4R0N6|C4R0N6\_KOMPG:C4R0S2|C4R0S2\_KOMPG:C4QZ10|C4QZ10\_KOMPG:C4R8C7|C4R8C7\_KOMPG:C4R7J4|C4R7J4\_KOMPG:C4R317|C4R317\_KOMPG:C4R637|C4R637\_KOMPG:C4R1Z8|C4R1Z8\_KOMPG:C4R713|C4R713\_KOMPG:C4R797|C4R797\_KOMPG |  |  | DB Search |
| LSGL | 22.6 | 388.2322 | 4 | -3.81 | 389.237 | 9.87 | 3804 | 6.08e3 | 1 | 1 | C4QZA8|C4QZA8\_KOMPG:C4QVP4|C4QVP4\_KOMPG:C4R466|C4R466\_KOMPG:C4QZ15|C4QZ15\_KOMPG:C4R5Q1|C4R5Q1\_KOMPG:C4R669|C4R669\_KOMPG:C4QWT1|C4QWT1\_KOMPG:C4R3T1|C4R3T1\_KOMPG:C4R4M2|C4R4M2\_KOMPG:C4R1P8|C4R1P8\_KOMPG:C4R3N4|C4R3N4\_KOMPG:C4R6B2|C4R6B2\_KOMPG:C4R0U6|C4R0U6\_KOMPG:C4R313|C4R313\_KOMPG:C4QWK2|C4QWK2\_KOMPG:C4R3U2|C4R3U2\_KOMPG:C4QVX0|C4QVX0\_KOMPG:C4R5M0|C4R5M0\_KOMPG:C4QV46|C4QV46\_KOMPG:C4R6X6|C4R6X6\_KOMPG:C4R4C7|C4R4C7\_KOMPG:C4R3L8|C4R3L8\_KOMPG:C4R5R1|C4R5R1\_KOMPG:C4QVY8|C4QVY8\_KOMPG:C4QVF7|C4QVF7\_KOMPG:C4QYF9|C4QYF9\_KOMPG:C4QWY1|C4QWY1\_KOMPG:C4R0G4|C4R0G4\_KOMPG:C4QY85|C4QY85\_KOMPG:C4R2T7|C4R2T7\_KOMPG:C4R7N8|C4R7N8\_KOMPG:C4R714|C4R714\_KOMPG:C4R580|C4R580\_KOMPG:C4R0U5|C4R0U5\_KOMPG:C4R2D2|C4R2D2\_KOMPG:C4QY65|C4QY65\_KOMPG:C4R4Y2|C4R4Y2\_KOMPG:C4R8Z3|C4R8Z3\_KOMPG:C4R7M8|C4R7M8\_KOMPG:C4R1U3|C4R1U3\_KOMPG:C4R321|C4R321\_KOMPG:C4R4N2|C4R4N2\_KOMPG:C4R2V2|C4R2V2\_KOMPG:C4R3X3|C4R3X3\_KOMPG:C4QZT7|C4QZT7\_KOMPG:C4R872|C4R872\_KOMPG:C4R043|C4R043\_KOMPG:C4R3G5|C4R3G5\_KOMPG:C4R8Q1|GATF\_KOMPG:C4QXQ4|C4QXQ4\_KOMPG:C4QX35|C4QX35\_KOMPG:C4R092|C4R092\_KOMPG:C4QW98|C4QW98\_KOMPG:C4R2F4|C4R2F4\_KOMPG:C4R0L5|C4R0L5\_KOMPG:C4R2C0|C4R2C0\_KOMPG:C4R2V8|C4R2V8\_KOMPG:C4R5I7|C4R5I7\_KOMPG:C4R6B7|C4R6B7\_KOMPG:C4QWG8|C4QWG8\_KOMPG:C4R852|C4R852\_KOMPG:C4R5T5|C4R5T5\_KOMPG:C4QVX8|C4QVX8\_KOMPG:C4R4U4|C4R4U4\_KOMPG:C4QXN8|C4QXN8\_KOMPG:C4R286|C4R286\_KOMPG:C4QYJ6|C4QYJ6\_KOMPG:C4R7N0|C4R7N0\_KOMPG:C4R1A8|C4R1A8\_KOMPG:C4R6P6|C4R6P6\_KOMPG:C4R1T5|C4R1T5\_KOMPG:C4R0H7|C4R0H7\_KOMPG:C4QVW1|C4QVW1\_KOMPG:C4R4F0|C4R4F0\_KOMPG:C4QWQ7|C4QWQ7\_KOMPG:C4R7K1|C4R7K1\_KOMPG:C4R8Y9|C4R8Y9\_KOMPG:C4QY28|C4QY28\_KOMPG:C4QX47|C4QX47\_KOMPG:C4R778|C4R778\_KOMPG:C4QXA7|C4QXA7\_KOMPG:C4R8H2|C4R8H2\_KOMPG:C4R7V3|C4R7V3\_KOMPG:C4QZ59|C4QZ59\_KOMPG:C4QW84|C4QW84\_KOMPG:C4R3L3|C4R3L3\_KOMPG:C4R4D1|C4R4D1\_KOMPG:C4R0X5|C4R0X5\_KOMPG:C4R8S6|C4R8S6\_KOMPG:C4R2U0|C4R2U0\_KOMPG:C4R6Q6|C4R6Q6\_KOMPG:C4R5P2|C4R5P2\_KOMPG:C4QZE7|C4QZE7\_KOMPG:C4R8R6|C4R8R6\_KOMPG:C4R4P7|C4R4P7\_KOMPG:C4R4T2|C4R4T2\_KOMPG:C4R8X1|C4R8X1\_KOMPG:C4R3W8|C4R3W8\_KOMPG:C4QYX5|C4QYX5\_KOMPG:C4R2G8|C4R2G8\_KOMPG:C4R7U8|C4R7U8\_KOMPG:C4QYN7|C4QYN7\_KOMPG:C4R6U0|C4R6U0\_KOMPG |  |  | DB Search |
| EI | 22.54 | 260.1372 | 2 | -4.37 | 261.1427 | 8.17 | 3066 | 4.03e4 | 1 | 1 | C4QY19|C4QY19\_KOMPG:C4R129|C4R129\_KOMPG:C4R0P1|C4R0P1\_KOMPG:C4R0S5|C4R0S5\_KOMPG:C4R688|C4R688\_KOMPG:C4QZV4|C4QZV4\_KOMPG:C4QW84|C4QW84\_KOMPG:C4R0V8|C4R0V8\_KOMPG:C4R4T5|C4R4T5\_KOMPG |  |  | DB Search |
| EL | 22.54 | 260.1372 | 2 | -4.37 | 261.1427 | 8.17 | 3066 | 4.03e4 | 1 | 1 | C4R912|C4R912\_KOMPG:C4QX82|C4QX82\_KOMPG:C4R0B6|C4R0B6\_KOMPG:C4R8H4|C4R8H4\_KOMPG:C4QV73|C4QV73\_KOMPG:C4R3T0|C4R3T0\_KOMPG:C4R3L2|C4R3L2\_KOMPG:C4R1N5|C4R1N5\_KOMPG:C4R215|C4R215\_KOMPG:C4R2Z6|C4R2Z6\_KOMPG:C4QW47|C4QW47\_KOMPG:C4R2A4|C4R2A4\_KOMPG:C4QYG5|C4QYG5\_KOMPG:C4R507|C4R507\_KOMPG:C4R1C0|C4R1C0\_KOMPG:C4R6N0|C4R6N0\_KOMPG |  |  | DB Search |
| Q(-17.03)PQQP | 22.54 | 579.2653 | 5 | -5.46 | 580.2679 | 8.21 | 3095 | 8.92e3 | 1 | 1 | C4QXL2|C4QXL2\_KOMPG:C4QY77|C4QY77\_KOMPG:C4QVX0|C4QVX0\_KOMPG:C4R4Q5|C4R4Q5\_KOMPG:C4QXV6|C4QXV6\_KOMPG:C4R150|C4R150\_KOMPG:C4R0K1|C4R0K1\_KOMPG:C4R5M4|C4R5M4\_KOMPG | Pyro-glu from Q | Q1:Pyro-glu from Q:1000 | DB Search |
| SIVGI | 22.5 | 487.3006 | 5 | -4.27 | 488.3046 | 21.68 | 8060 | 8.06e3 | 1 | 1 | C4R3S9|C4R3S9\_KOMPG:C4R4V2|C4R4V2\_KOMPG:C4R2G9|C4R2G9\_KOMPG:C4R4R8|ARO1\_KOMPG |  |  | DB Search |
| SIVGL | 22.5 | 487.3006 | 5 | -4.27 | 488.3046 | 21.68 | 8060 | 8.06e3 | 1 | 1 | C4QV93|C4QV93\_KOMPG:C4QVN9|C4QVN9\_KOMPG:C4R2N7|C4R2N7\_KOMPG:C4QXF6|C4QXF6\_KOMPG:C4R7U3|C4R7U3\_KOMPG |  |  | DB Search |
| SLVGI | 22.5 | 487.3006 | 5 | -4.27 | 488.3046 | 21.68 | 8060 | 8.06e3 | 1 | 1 | C4R8Q0|C4R8Q0\_KOMPG:C4QV83|C4QV83\_KOMPG:C4R700|C4R700\_KOMPG:C4R1A0|C4R1A0\_KOMPG:C4R6V9|C4R6V9\_KOMPG:C4R1I8|C4R1I8\_KOMPG |  |  | DB Search |
| SLVGL | 22.5 | 487.3006 | 5 | -4.27 | 488.3046 | 21.68 | 8060 | 8.06e3 | 1 | 1 | C4R2E5|C4R2E5\_KOMPG:C4R3B1|C4R3B1\_KOMPG:C4QVH0|C4QVH0\_KOMPG:C4QWQ7|C4QWQ7\_KOMPG:C4R0X3|C4R0X3\_KOMPG:C4R4W8|C4R4W8\_KOMPG:C4R706|C4R706\_KOMPG:C4R816|C4R816\_KOMPG:C4R8M2|C4R8M2\_KOMPG:C4R7Z1|C4R7Z1\_KOMPG:C4QW62|C4QW62\_KOMPG:C4R5H2|C4R5H2\_KOMPG:C4R6G7|C4R6G7\_KOMPG |  |  | DB Search |
| QIEQIQ | 22.5 | 757.397 | 6 | -8.46 | 758.396 | 10.38 | 4065 | 0 | 0 | 0 | C4QY50|C4QY50\_KOMPG |  |  | DB Search |
| QIEQLQ | 22.5 | 757.397 | 6 | -8.46 | 758.396 | 10.38 | 4065 | 0 | 0 | 0 | C4R2R7|C4R2R7\_KOMPG |  |  | DB Search |
| IVAP | 22.5 | 398.2529 | 4 | -6.51 | 399.2566 | 9.50 | 3744 | 1.49e3 | 1 | 1 | C4R570|C4R570\_KOMPG:C4R6I7|C4R6I7\_KOMPG:C4R1Y5|C4R1Y5\_KOMPG:C4R7N6|C4R7N6\_KOMPG:C4R0E7|C4R0E7\_KOMPG:C4QZP6|C4QZP6\_KOMPG:C4R3B3|C4R3B3\_KOMPG:C4QZ34|C4QZ34\_KOMPG:C4R8Z7|C4R8Z7\_KOMPG:C4QZ75|C4QZ75\_KOMPG:C4R8Z2|C4R8Z2\_KOMPG:C4R1E3|C4R1E3\_KOMPG:C4QWT8|C4QWT8\_KOMPG:C4R4D4|C4R4D4\_KOMPG:C4QXW5|C4QXW5\_KOMPG:C4QZU7|C4QZU7\_KOMPG:C4R149|C4R149\_KOMPG:C4R6V4|C4R6V4\_KOMPG:C4R4T6|C4R4T6\_KOMPG:C4QY82|C4QY82\_KOMPG |  |  | DB Search |
| LVAP | 22.5 | 398.2529 | 4 | -6.51 | 399.2566 | 9.50 | 3744 | 1.49e3 | 1 | 1 | C4QWN3|C4QWN3\_KOMPG:C4R662|C4R662\_KOMPG:C4QZ58|C4QZ58\_KOMPG:C4R2Q9|C4R2Q9\_KOMPG:C4R4C1|C4R4C1\_KOMPG:C4QXJ4|C4QXJ4\_KOMPG:C4R4N4|C4R4N4\_KOMPG:C4QVU5|C4QVU5\_KOMPG:C4R193|C4R193\_KOMPG:C4R5Q9|C4R5Q9\_KOMPG:C4R1K0|C4R1K0\_KOMPG:C4QXW7|C4QXW7\_KOMPG:C4R5J4|C4R5J4\_KOMPG:C4QZ55|C4QZ55\_KOMPG:C4R0U6|C4R0U6\_KOMPG:C4R1F2|C4R1F2\_KOMPG:C4R3N0|C4R3N0\_KOMPG:C4QV45|C4QV45\_KOMPG:C4R5B0|C4R5B0\_KOMPG:C4R1F8|C4R1F8\_KOMPG:C4QWG3|C4QWG3\_KOMPG:C4R644|C4R644\_KOMPG:C4R7Q2|C4R7Q2\_KOMPG:C4QWC1|C4QWC1\_KOMPG:C4R8E4|C4R8E4\_KOMPG:C4QZ27|C4QZ27\_KOMPG:C4R1C2|C4R1C2\_KOMPG:C4QX63|C4QX63\_KOMPG:C4R2F5|C4R2F5\_KOMPG:C4R8P4|C4R8P4\_KOMPG:C4R1T0|C4R1T0\_KOMPG:C4R4B8|C4R4B8\_KOMPG:C4QX72|C4QX72\_KOMPG:C4R1F1|C4R1F1\_KOMPG:C4R0L0|C4R0L0\_KOMPG:C4R4M5|C4R4M5\_KOMPG:C4R0R2|C4R0R2\_KOMPG |  |  | DB Search |
| ATAQ(+0.98)DIQT | 22.46 | 847.3923 | 8 | -4.5 | 848.3937 | 25.46 | 9101 | 1.31e2 | 1 | 1 | C4QXQ4|C4QXQ4\_KOMPG | Deamidation (NQ) | Q4:Deamidation (NQ):29.32 | DB Search |
| TATSIA | 22.4 | 562.2962 | 6 | -9.2 | 563.2969 | 8.27 | 3134 | 0 | 0 | 0 | C4R0F7|C4R0F7\_KOMPG |  |  | DB Search |
| IGVND | 22.39 | 516.2543 | 5 | -3.74 | 517.2584 | 5.18 | 2080 | 0 | 0 | 0 | C4QXI3|C4QXI3\_KOMPG:C4R0R2|C4R0R2\_KOMPG |  |  | DB Search |
| LGVND | 22.39 | 516.2543 | 5 | -3.74 | 517.2584 | 5.18 | 2080 | 0 | 0 | 0 | C4R7K8|C4R7K8\_KOMPG:C4R6R3|C4R6R3\_KOMPG:C4R070|C4R070\_KOMPG:C4QZN1|C4QZN1\_KOMPG:C4R7M7|C4R7M7\_KOMPG:C4R774|C4R774\_KOMPG |  |  | DB Search |
| PPGGPM | 22.39 | 554.2523 | 6 | 5.67 | 555.2613 | 21.77 | 8025 | 4.01e2 | 1 | 1 | C4QZT4|C4QZT4\_KOMPG |  |  | DB Search |
| LDQSPATV | 22.38 | 829.4182 | 8 | 0.14 | 830.4235 | 15.30 | 6034 | 3.76e3 | 1 | 1 | C4QW22|C4QW22\_KOMPG |  |  | DB Search |
| NIQVD | 22.38 | 587.2915 | 5 | 1.98 | 588.2985 | 9.98 | 3878 | 1.51e3 | 1 | 1 | C4QWY0|C4QWY0\_KOMPG |  |  | DB Search |
| NLQVD | 22.38 | 587.2915 | 5 | 1.98 | 588.2985 | 9.98 | 3878 | 1.51e3 | 1 | 1 | C4R7R9|C4R7R9\_KOMPG:C4R2N7|C4R2N7\_KOMPG |  |  | DB Search |
| VKEVGGGA | 22.37 | 715.3864 | 8 | -7.18 | 716.3868 | 8.78 | 3406 | 0 | 0 | 0 | C4R3X1|C4R3X1\_KOMPG |  |  | DB Search |
| EW | 22.33 | 333.1324 | 2 | -3.27 | 334.1378 | 12.76 | 5036 | 2.08e4 | 1 | 1 | C4QYE6|C4QYE6\_KOMPG:C4QVY1|C4QVY1\_KOMPG:C4QVZ8|C4QVZ8\_KOMPG:C4R3J0|C4R3J0\_KOMPG:C4QWE1|C4QWE1\_KOMPG:C4R529|C4R529\_KOMPG:C4R8U9|C4R8U9\_KOMPG:C4QV10|C4QV10\_KOMPG:C4R8N1|C4R8N1\_KOMPG:C4R6K9|C4R6K9\_KOMPG:C4QZ28|C4QZ28\_KOMPG:C4R006|C4R006\_KOMPG:C4R948|C4R948\_KOMPG:C4QZZ8|C4QZZ8\_KOMPG:C4R3G3|C4R3G3\_KOMPG:C4R181|C4R181\_KOMPG:C4R2Z6|C4R2Z6\_KOMPG:C4R1P8|C4R1P8\_KOMPG:C4R8B9|C4R8B9\_KOMPG:C4R110|C4R110\_KOMPG:C4R6B2|C4R6B2\_KOMPG:C4QVX4|C4QVX4\_KOMPG:C4QYX9|C4QYX9\_KOMPG:C4R0X2|C4R0X2\_KOMPG:C4QZ71|C4QZ71\_KOMPG:C4QXM1|C4QXM1\_KOMPG:C4QV42|C4QV42\_KOMPG:C4R4A5|C4R4A5\_KOMPG:C4QYY0|C4QYY0\_KOMPG:C4R042|C4R042\_KOMPG:C4QVM0|C4QVM0\_KOMPG:C4QXU1|C4QXU1\_KOMPG:C4R5J2|C4R5J2\_KOMPG:C4R5K0|C4R5K0\_KOMPG:C4R5N1|C4R5N1\_KOMPG:C4R8T8|C4R8T8\_KOMPG:C4QWJ2|C4QWJ2\_KOMPG:C4R233|C4R233\_KOMPG:C4QZ47|C4QZ47\_KOMPG:C4R2Y2|C4R2Y2\_KOMPG:C4QZJ4|C4QZJ4\_KOMPG:C4QVX9|C4QVX9\_KOMPG:C4R4V9|C4R4V9\_KOMPG:C4R7N8|C4R7N8\_KOMPG:C4R6Q8|C4R6Q8\_KOMPG:C4QX78|C4QX78\_KOMPG:C4R0X4|C4R0X4\_KOMPG:C4R5S1|C4R5S1\_KOMPG:C4R1S7|C4R1S7\_KOMPG:C4R162|C4R162\_KOMPG:C4QZV3|C4QZV3\_KOMPG:C4QVQ8|C4QVQ8\_KOMPG:C4R093|C4R093\_KOMPG:C4R4F5|C4R4F5\_KOMPG:C4R0E3|C4R0E3\_KOMPG:C4R4Z8|C4R4Z8\_KOMPG:C4R8J5|C4R8J5\_KOMPG:C4R6W1|C4R6W1\_KOMPG:C4R7R8|C4R7R8\_KOMPG:C4R240|C4R240\_KOMPG:C4QWD1|C4QWD1\_KOMPG:C4R3L2|C4R3L2\_KOMPG:C4R773|C4R773\_KOMPG:C4R3A8|C4R3A8\_KOMPG:C4R095|C4R095\_KOMPG:C4R3H2|C4R3H2\_KOMPG:C4R0L5|C4R0L5\_KOMPG:C4R1I1|C4R1I1\_KOMPG:C4R8D9|C4R8D9\_KOMPG:C4R4M3|C4R4M3\_KOMPG:C4R3D1|C4R3D1\_KOMPG:C4QXT2|C4QXT2\_KOMPG:C4QX82|C4QX82\_KOMPG:C4R8J1|C4R8J1\_KOMPG:C4R413|C4R413\_KOMPG:C4R554|C4R554\_KOMPG:C4R0H0|C4R0H0\_KOMPG:C4QVZ4|C4QVZ4\_KOMPG:C4R1N5|C4R1N5\_KOMPG:C4R751|C4R751\_KOMPG:Q9P4D0|SEC17\_KOMPG:C4QWA3|C4QWA3\_KOMPG:C4R6L7|C4R6L7\_KOMPG:C4R4I2|C4R4I2\_KOMPG:C4R912|C4R912\_KOMPG:C4R0S5|C4R0S5\_KOMPG:C4R7W9|C4R7W9\_KOMPG:C4QYT3|C4QYT3\_KOMPG:C4QY28|C4QY28\_KOMPG:C4R6H6|C4R6H6\_KOMPG:C4R3T0|C4R3T0\_KOMPG:C4R2T3|C4R2T3\_KOMPG:C4QYZ4|C4QYZ4\_KOMPG:C4QYQ0|C4QYQ0\_KOMPG:C4QW41|C4QW41\_KOMPG:C4QW00|C4QW00\_KOMPG:C4QY92|C4QY92\_KOMPG:C4R033|C4R033\_KOMPG:C4R513|C4R513\_KOMPG:C4QVV3|C4QVV3\_KOMPG:C4QXL0|C4QXL0\_KOMPG:C4R7V3|C4R7V3\_KOMPG:C4R877|C4R877\_KOMPG:C4R921|C4R921\_KOMPG:C4R1H2|C4R1H2\_KOMPG:C4R0M2|C4R0M2\_KOMPG:C4QW84|C4QW84\_KOMPG:C4QZ69|C4QZ69\_KOMPG:C4R6F8|C4R6F8\_KOMPG:C4R848|C4R848\_KOMPG:C4QYQ2|C4QYQ2\_KOMPG:C4R5D6|C4R5D6\_KOMPG:C4R3C3|C4R3C3\_KOMPG:C4QXT3|C4QXT3\_KOMPG:C4QV45|C4QV45\_KOMPG:C4QWC1|C4QWC1\_KOMPG:C4R126|C4R126\_KOMPG:C4R3Y7|C4R3Y7\_KOMPG:C4R4T5|C4R4T5\_KOMPG:C4QZF2|C4QZF2\_KOMPG:C4R065|C4R065\_KOMPG:C4QVA1|C4QVA1\_KOMPG:C4R2Q0|C4R2Q0\_KOMPG:C4QY10|C4QY10\_KOMPG:C4QXS6|C4QXS6\_KOMPG:C4R5H2|C4R5H2\_KOMPG:C4QXZ9|C4QXZ9\_KOMPG:C4R749|C4R749\_KOMPG:C4R855|C4R855\_KOMPG:C4R721|C4R721\_KOMPG:C4R800|C4R800\_KOMPG:C4R2W0|C4R2W0\_KOMPG:C4R3A9|C4R3A9\_KOMPG |  |  | DB Search |
| ETLISE | 22.28 | 690.3436 | 6 | -5.58 | 691.3453 | 17.12 | 6821 | 2.46e2 | 1 | 1 | C4R7T5|C4R7T5\_KOMPG |  |  | DB Search |
| TIDI | 22.27 | 460.2533 | 4 | -8.03 | 461.2557 | 9.41 | 3625 | 2.97e2 | 1 | 1 | C4QYI7|C4QYI7\_KOMPG:C4QVV6|C4QVV6\_KOMPG:C4QVI7|C4QVI7\_KOMPG:C4R6R3|C4R6R3\_KOMPG:C4R3C5|C4R3C5\_KOMPG:C4R217|C4R217\_KOMPG:C4R3F6|C4R3F6\_KOMPG:C4QXQ1|C4QXQ1\_KOMPG:C4R571|C4R571\_KOMPG:C4R5B1|C4R5B1\_KOMPG:C4QW82|C4QW82\_KOMPG:C4R3U7|C4R3U7\_KOMPG:C4R6T4|C4R6T4\_KOMPG:C4R3F5|C4R3F5\_KOMPG:C4R8X4|C4R8X4\_KOMPG:C4R3P1|C4R3P1\_KOMPG:C4QXZ6|C4QXZ6\_KOMPG |  |  | DB Search |
| TIDL | 22.27 | 460.2533 | 4 | -8.03 | 461.2557 | 9.41 | 3625 | 2.97e2 | 1 | 1 | C4R3S7|C4R3S7\_KOMPG:C4QXL0|C4QXL0\_KOMPG:C4R922|C4R922\_KOMPG:C4R339|C4R339\_KOMPG:C4R2J5|C4R2J5\_KOMPG:C4R368|C4R368\_KOMPG:C4QWJ0|C4QWJ0\_KOMPG:C4QXX5|C4QXX5\_KOMPG:C4R6C5|C4R6C5\_KOMPG:C4QZT5|C4QZT5\_KOMPG:C4R0G5|C4R0G5\_KOMPG:C4QVW5|C4QVW5\_KOMPG:C4QYP8|C4QYP8\_KOMPG:C4QZY7|C4QZY7\_KOMPG:C4QXT5|C4QXT5\_KOMPG:C4R320|C4R320\_KOMPG:C4QZ21|C4QZ21\_KOMPG:C4QZV8|C4QZV8\_KOMPG:C4R7E0|C4R7E0\_KOMPG:C4QW27|C4QW27\_KOMPG:C4QXD1|C4QXD1\_KOMPG:C4R4M0|C4R4M0\_KOMPG:C4R5M0|C4R5M0\_KOMPG:C4QY86|C4QY86\_KOMPG:C4R370|C4R370\_KOMPG:C4R0Y0|C4R0Y0\_KOMPG:C4R6W9|C4R6W9\_KOMPG:C4R604|C4R604\_KOMPG:C4R3H7|C4R3H7\_KOMPG:C4R1E3|C4R1E3\_KOMPG:C4QY02|C4QY02\_KOMPG:C4R281|C4R281\_KOMPG:C4QXF0|C4QXF0\_KOMPG:C4R7J4|C4R7J4\_KOMPG:C4QYR7|C4QYR7\_KOMPG:C4R970|ASA1\_KOMPG:C4R1Z4|C4R1Z4\_KOMPG:C4R154|C4R154\_KOMPG:C4R2G3|C4R2G3\_KOMPG |  |  | DB Search |
| TLDI | 22.27 | 460.2533 | 4 | -8.03 | 461.2557 | 9.41 | 3625 | 2.97e2 | 1 | 1 | C4QYG6|C4QYG6\_KOMPG:C4R0A8|C4R0A8\_KOMPG:C4QZL2|C4QZL2\_KOMPG:C4QVX1|C4QVX1\_KOMPG:C4QWE6|C4QWE6\_KOMPG:C4R3K1|C4R3K1\_KOMPG:C4R6F9|C4R6F9\_KOMPG:C4QZW7|C4QZW7\_KOMPG:C4R2K0|UTP25\_KOMPG:C4R1T2|C4R1T2\_KOMPG:C4R5E0|C4R5E0\_KOMPG:C4R4D1|C4R4D1\_KOMPG:C4R0G8|C4R0G8\_KOMPG:C4QZE1|C4QZE1\_KOMPG:C4QX26|C4QX26\_KOMPG:C4R8N3|C4R8N3\_KOMPG:C4QZB6|C4QZB6\_KOMPG:C4R4J6|C4R4J6\_KOMPG:C4QY28|C4QY28\_KOMPG:C4R7L0|C4R7L0\_KOMPG:C4QWU0|C4QWU0\_KOMPG:C4R882|C4R882\_KOMPG:C4QWR8|C4QWR8\_KOMPG:C4R420|C4R420\_KOMPG:C4QYF9|C4QYF9\_KOMPG:C4QUZ0|C4QUZ0\_KOMPG:C4R813|C4R813\_KOMPG:C4QZS0|C4QZS0\_KOMPG:C4R7W4|C4R7W4\_KOMPG |  |  | DB Search |
| TLDL | 22.27 | 460.2533 | 4 | -8.03 | 461.2557 | 9.41 | 3625 | 2.97e2 | 1 | 1 | C4R3A1|C4R3A1\_KOMPG:C4QWF8|C4QWF8\_KOMPG:C4QZJ3|C4QZJ3\_KOMPG:C4R7D0|C4R7D0\_KOMPG:C4R998|C4R998\_KOMPG:C4QYV4|C4QYV4\_KOMPG:C4R036|C4R036\_KOMPG:C4R5U6|C4R5U6\_KOMPG:C4QYT5|C4QYT5\_KOMPG:C4R542|C4R542\_KOMPG:C4QYG5|C4QYG5\_KOMPG:C4R1W6|C4R1W6\_KOMPG:C4R0X2|C4R0X2\_KOMPG:C4R543|C4R543\_KOMPG:C4R3C1|C4R3C1\_KOMPG:C4R9G3|C4R9G3\_KOMPG:C4QXW2|C4QXW2\_KOMPG:C4R6E1|C4R6E1\_KOMPG:C4R2L4|C4R2L4\_KOMPG:C4QWM4|C4QWM4\_KOMPG:C4R666|C4R666\_KOMPG:C4R169|C4R169\_KOMPG:C4R3D7|C4R3D7\_KOMPG:C4QZK9|C4QZK9\_KOMPG:C4QZA5|C4QZA5\_KOMPG:C4QYU4|C4QYU4\_KOMPG:C4R659|C4R659\_KOMPG:C4R432|SEY1\_KOMPG:C4R8V6|C4R8V6\_KOMPG:C4R162|C4R162\_KOMPG:C4R5X4|C4R5X4\_KOMPG:C4R517|C4R517\_KOMPG:C4R6N1|C4R6N1\_KOMPG:C4QXY0|C4QXY0\_KOMPG:C4QYV6|C4QYV6\_KOMPG:C4QV56|C4QV56\_KOMPG:C4QYA5|C4QYA5\_KOMPG:C4R2D4|C4R2D4\_KOMPG:C4R0E3|C4R0E3\_KOMPG:C4R7D7|C4R7D7\_KOMPG:C4R8H5|C4R8H5\_KOMPG:C4R4T5|C4R4T5\_KOMPG:C4R2A9|C4R2A9\_KOMPG:C4R096|C4R096\_KOMPG:C4R749|C4R749\_KOMPG:C4R360|PSD2\_KOMPG:C4R6Z4|C4R6Z4\_KOMPG:C4R6N8|C4R6N8\_KOMPG:C4R8V3|C4R8V3\_KOMPG |  |  | DB Search |
| VTVP | 22.26 | 414.2478 | 4 | -0.09 | 415.254 | 17.14 | 6680 | 3.72e3 | 1 | 1 | C4R0V9|C4R0V9\_KOMPG:C4R584|C4R584\_KOMPG:C4QX89|C4QX89\_KOMPG:C4QXF3|C4QXF3\_KOMPG:C4R214|C4R214\_KOMPG:C4R3G4|C4R3G4\_KOMPG:C4R4C3|C4R4C3\_KOMPG:C4R0N1|C4R0N1\_KOMPG:C4R2V3|C4R2V3\_KOMPG:C4QZS3|C4QZS3\_KOMPG:C4R887|C4R887\_KOMPG:C4R5E4|C4R5E4\_KOMPG:C4QYK5|C4QYK5\_KOMPG:C4QWD7|C4QWD7\_KOMPG:C4R8B9|C4R8B9\_KOMPG:C4QW37|C4QW37\_KOMPG:C4QWA0|C4QWA0\_KOMPG:C4R4G5|C4R4G5\_KOMPG:C4QZY7|C4QZY7\_KOMPG:C4R5Z4|C4R5Z4\_KOMPG:C4R527|C4R527\_KOMPG:C4R4P9|C4R4P9\_KOMPG:C4QVS8|C4QVS8\_KOMPG:C4QZZ4|C4QZZ4\_KOMPG:C4QX31|C4QX31\_KOMPG:C4QVR8|C4QVR8\_KOMPG:C4R3A8|C4R3A8\_KOMPG:C4R2Q6|C4R2Q6\_KOMPG:C4R092|C4R092\_KOMPG:C4R918|C4R918\_KOMPG:C4R3X8|C4R3X8\_KOMPG:C4R113|C4R113\_KOMPG:C4R8N5|C4R8N5\_KOMPG:C4QV59|C4QV59\_KOMPG:C4R3G7|C4R3G7\_KOMPG |  |  | DB Search |
| ANIP | 22.26 | 413.2274 | 4 | 0.01 | 414.2337 | 11.08 | 4376 | 0 | 0 | 0 | C4R680|C4R680\_KOMPG:C4R0S5|C4R0S5\_KOMPG:C4QZV2|C4QZV2\_KOMPG:C4R5U1|C4R5U1\_KOMPG:C4R791|C4R791\_KOMPG:C4R7Q6|C4R7Q6\_KOMPG:C4R6F6|C4R6F6\_KOMPG:C4R5H3|C4R5H3\_KOMPG:C4QX25|C4QX25\_KOMPG:C4R7V9|C4R7V9\_KOMPG:C4R808|C4R808\_KOMPG:C4R0S4|C4R0S4\_KOMPG:C4QZU6|C4QZU6\_KOMPG:C4R497|C4R497\_KOMPG:C4R5F1|C4R5F1\_KOMPG:C4R0Q3|C4R0Q3\_KOMPG:C4R0E0|C4R0E0\_KOMPG:C4R938|C4R938\_KOMPG:C4R8Z5|C4R8Z5\_KOMPG:C4QYX4|C4QYX4\_KOMPG |  |  | DB Search |
| ANLP | 22.26 | 413.2274 | 4 | 0.01 | 414.2337 | 11.08 | 4376 | 0 | 0 | 0 | C4QVM2|C4QVM2\_KOMPG:C4R2C1|C4R2C1\_KOMPG:C4R915|C4R915\_KOMPG:C4R660|C4R660\_KOMPG:C4QVT8|C4QVT8\_KOMPG:C4R6C9|C4R6C9\_KOMPG:C4R6X0|C4R6X0\_KOMPG:C4R069|C4R069\_KOMPG:C4QW58|C4QW58\_KOMPG:C4R9C8|C4R9C8\_KOMPG:C4QWN6|C4QWN6\_KOMPG:C4QV43|C4QV43\_KOMPG:C4R6F5|C4R6F5\_KOMPG:C4QV49|C4QV49\_KOMPG:C4QZU7|C4QZU7\_KOMPG:C4R4Q8|C4R4Q8\_KOMPG:C4R403|C4R403\_KOMPG:C4QXW0|C4QXW0\_KOMPG:C4R503|C4R503\_KOMPG:C4R1E7|C4R1E7\_KOMPG |  |  | DB Search |
| VDYN | 22.24 | 509.2122 | 4 | 1.18 | 510.2188 | 5.69 | 2263 | 7.88e3 | 1 | 1 | C4R912|C4R912\_KOMPG:C4QX00|C4QX00\_KOMPG:C4R8P2|GATB\_KOMPG:C4R743|C4R743\_KOMPG:C4R013|C4R013\_KOMPG:C4QXG8|C4QXG8\_KOMPG:C4QZE6|C4QZE6\_KOMPG:C4R7L0|C4R7L0\_KOMPG:C4QX30|C4QX30\_KOMPG:C4R340|C4R340\_KOMPG:C4QVH1|C4QVH1\_KOMPG:C4R454|C4R454\_KOMPG:C4R087|C4R087\_KOMPG |  |  | DB Search |
| VENGDVI | 22.24 | 744.3654 | 7 | -6.72 | 745.3658 | 15.15 | 6014 | 0 | 0 | 0 | C4QYI7|C4QYI7\_KOMPG |  |  | DB Search |
| IEISG | 22.23 | 517.2748 | 5 | -2.69 | 518.2794 | 11.88 | 4696 | 0 | 0 | 0 | C4QVJ2|C4QVJ2\_KOMPG:C4R7W9|C4R7W9\_KOMPG:C4R042|C4R042\_KOMPG |  |  | DB Search |
| IELSG | 22.23 | 517.2748 | 5 | -2.69 | 518.2794 | 11.88 | 4696 | 0 | 0 | 0 | C4R286|C4R286\_KOMPG:C4R429|C4R429\_KOMPG |  |  | DB Search |
| LEISG | 22.23 | 517.2748 | 5 | -2.69 | 518.2794 | 11.88 | 4696 | 0 | 0 | 0 | C4QYR3|C4QYR3\_KOMPG:C4R901|C4R901\_KOMPG:C4R789|C4R789\_KOMPG:C4R1W3|C4R1W3\_KOMPG:C4R3M7|C4R3M7\_KOMPG |  |  | DB Search |
| LELSG | 22.23 | 517.2748 | 5 | -2.69 | 518.2794 | 11.88 | 4696 | 0 | 0 | 0 | C4QZ60|C4QZ60\_KOMPG:C4R1F5|C4R1F5\_KOMPG:C4QZ71|C4QZ71\_KOMPG:C4R6D1|C4R6D1\_KOMPG:C4R4R6|C4R4R6\_KOMPG:C4R4W0|C4R4W0\_KOMPG |  |  | DB Search |
| WLRNQ | 22.22 | 715.3765 | 5 | 3.29 | 716.3844 | 8.73 | 3406 | 3.25e3 | 1 | 1 | C4QXA9|C4QXA9\_KOMPG |  |  | DB Search |
| VIIP | 22.17 | 440.2998 | 4 | -5.96 | 441.3034 | 30.99 | 10567 | 1.25e3 | 1 | 1 | C4R6B9|C4R6B9\_KOMPG:C4R1M6|C4R1M6\_KOMPG:C4R675|C4R675\_KOMPG:C4R9A9|C4R9A9\_KOMPG:C4QY31|C4QY31\_KOMPG:C4R1E5|C4R1E5\_KOMPG:C4R3V5|C4R3V5\_KOMPG:C4QXM7|C4QXM7\_KOMPG:C4R911|C4R911\_KOMPG:C4QYN8|C4QYN8\_KOMPG:C4R8I2|C4R8I2\_KOMPG:C4R1D5|C4R1D5\_KOMPG:C4R780|C4R780\_KOMPG:C4R1H0|C4R1H0\_KOMPG:C4R1Q3|C4R1Q3\_KOMPG:C4R694|C4R694\_KOMPG:C4QVU0|C4QVU0\_KOMPG:C4R5X9|C4R5X9\_KOMPG:C4R1M0|C4R1M0\_KOMPG:C4R7M7|C4R7M7\_KOMPG |  |  | DB Search |
| VILP | 22.17 | 440.2998 | 4 | -5.96 | 441.3034 | 30.99 | 10567 | 1.25e3 | 1 | 1 | C4R6N9|C4R6N9\_KOMPG:C4R1B3|C4R1B3\_KOMPG:C4R7N8|C4R7N8\_KOMPG:C4QVB8|C4QVB8\_KOMPG:C4R2K0|UTP25\_KOMPG:C4R0B9|C4R0B9\_KOMPG:C4R502|C4R502\_KOMPG:C4QYV4|C4QYV4\_KOMPG:C4R399|C4R399\_KOMPG:C4R0Q9|C4R0Q9\_KOMPG:C4R4T9|C4R4T9\_KOMPG:C4R0N6|C4R0N6\_KOMPG:C4R286|C4R286\_KOMPG:C4R134|C4R134\_KOMPG:C4R6U7|C4R6U7\_KOMPG:C4R6W4|C4R6W4\_KOMPG:C4QV05|C4QV05\_KOMPG:C4R180|C4R180\_KOMPG:C4QW27|C4QW27\_KOMPG:C4R175|C4R175\_KOMPG:C4R3X6|C4R3X6\_KOMPG:C4R285|C4R285\_KOMPG:C4QV35|C4QV35\_KOMPG:C4QWB6|C4QWB6\_KOMPG:C4QX81|C4QX81\_KOMPG:C4R3S4|C4R3S4\_KOMPG:C4QWQ7|C4QWQ7\_KOMPG:C4R8Z1|C4R8Z1\_KOMPG:C4QYF5|C4QYF5\_KOMPG:C4R263|C4R263\_KOMPG:C4R0G6|C4R0G6\_KOMPG:C4R1E0|C4R1E0\_KOMPG:C4R754|C4R754\_KOMPG:C4R8X8|C4R8X8\_KOMPG:C4R4Q3|C4R4Q3\_KOMPG:C4R8X4|C4R8X4\_KOMPG |  |  | DB Search |
| VLIP | 22.17 | 440.2998 | 4 | -5.96 | 441.3034 | 30.99 | 10567 | 1.25e3 | 1 | 1 | C4R3J0|C4R3J0\_KOMPG:C4R7X8|BMT2\_KOMPG:C4R006|C4R006\_KOMPG:C4R2S7|C4R2S7\_KOMPG:C4QVU5|C4QVU5\_KOMPG:C4QVS6|C4QVS6\_KOMPG:C4R4X2|C4R4X2\_KOMPG:C4QZJ5|C4QZJ5\_KOMPG:C4R1D0|C4R1D0\_KOMPG:C4QXM0|C4QXM0\_KOMPG:C4QYP6|C4QYP6\_KOMPG:C4QYY2|C4QYY2\_KOMPG:C4R051|C4R051\_KOMPG:C4R927|C4R927\_KOMPG:C4R325|C4R325\_KOMPG:C4R1T4|C4R1T4\_KOMPG:C4QXA5|PFKA2\_KOMPG:C4R628|PFF1\_KOMPG:C4R5I6|C4R5I6\_KOMPG:C4QWV9|C4QWV9\_KOMPG:C4R963|C4R963\_KOMPG:C4R3L8|C4R3L8\_KOMPG:C4QXN2|MIC60\_KOMPG:C4R5Y6|C4R5Y6\_KOMPG:C4QZS0|C4QZS0\_KOMPG:C4R3M1|C4R3M1\_KOMPG:C4QZ06|BMT3\_KOMPG:C4R4X6|C4R4X6\_KOMPG:C4R6K4|C4R6K4\_KOMPG |  |  | DB Search |
| VLLP | 22.17 | 440.2998 | 4 | -5.96 | 441.3034 | 30.99 | 10567 | 1.25e3 | 1 | 1 | C4R0N4|C4R0N4\_KOMPG:C4QVY3|C4QVY3\_KOMPG:C4R9E3|C4R9E3\_KOMPG:C4QYU4|C4QYU4\_KOMPG:C4R0D6|C4R0D6\_KOMPG:C4R3D0|C4R3D0\_KOMPG:C4QVH6|C4QVH6\_KOMPG:C4R221|C4R221\_KOMPG:C4QY05|C4QY05\_KOMPG:C4QYJ5|C4QYJ5\_KOMPG:C4QWE8|C4QWE8\_KOMPG:C4QWH3|C4QWH3\_KOMPG:C4R647|C4R647\_KOMPG:C4QWB3|C4QWB3\_KOMPG:C4R865|C4R865\_KOMPG:C4QVK6|C4QVK6\_KOMPG:C4QX48|C4QX48\_KOMPG:C4QYR3|C4QYR3\_KOMPG:C4QX75|C4QX75\_KOMPG:C4R109|C4R109\_KOMPG:C4R8N8|C4R8N8\_KOMPG:C4R8B7|C4R8B7\_KOMPG:C4R4S1|C4R4S1\_KOMPG:C4R6E1|C4R6E1\_KOMPG:C4QWD1|C4QWD1\_KOMPG:C4R941|GLG\_KOMPG:C4R7Q9|C4R7Q9\_KOMPG:C4QYZ4|C4QYZ4\_KOMPG:C4QYN9|C4QYN9\_KOMPG:C4QW41|C4QW41\_KOMPG:C4R5H2|C4R5H2\_KOMPG:C4R111|C4R111\_KOMPG:C4R1S8|C4R1S8\_KOMPG:C4R8P5|C4R8P5\_KOMPG:C4R5A7|C4R5A7\_KOMPG:C4R8E3|C4R8E3\_KOMPG:C4R0L0|C4R0L0\_KOMPG |  |  | DB Search |
| TDVP | 22.14 | 430.2064 | 4 | -7.31 | 431.2094 | 8.43 | 3152 | 9.04e3 | 3 | 3 | C4R3U0|GET2\_KOMPG:C4QVA9|C4QVA9\_KOMPG:C4R5L9|C4R5L9\_KOMPG:C4QVZ4|C4QVZ4\_KOMPG:C4R9B4|C4R9B4\_KOMPG:C4QZI7|C4QZI7\_KOMPG:C4QW46|C4QW46\_KOMPG:C4QVK1|C4QVK1\_KOMPG:C4R2V9|C4R2V9\_KOMPG:C4R228|C4R228\_KOMPG:C4QW94|C4QW94\_KOMPG:C4R5Z4|C4R5Z4\_KOMPG:C4R0K4|C4R0K4\_KOMPG:C4R527|C4R527\_KOMPG:C4QZN0|C4QZN0\_KOMPG:C4QZC5|C4QZC5\_KOMPG:C4QW65|C4QW65\_KOMPG:C4QYX6|C4QYX6\_KOMPG:C4R608|C4R608\_KOMPG:C4QVV4|C4QVV4\_KOMPG:C4QWI4|C4QWI4\_KOMPG:C4R1H4|C4R1H4\_KOMPG:C4QVJ9|C4QVJ9\_KOMPG:C4QWZ7|C4QWZ7\_KOMPG:C4QZS0|C4QZS0\_KOMPG:C4QYW9|C4QYW9\_KOMPG |  |  | DB Search |
| IIAGP | 22.13 | 469.29 | 5 | -3.4 | 470.2945 | 12.41 | 4848 | 6.43e3 | 1 | 1 | C4R1D2|C4R1D2\_KOMPG:C4R5Z6|C4R5Z6\_KOMPG:C4R3W4|C4R3W4\_KOMPG:C4R961|C4R961\_KOMPG |  |  | DB Search |
| ILAGP | 22.13 | 469.29 | 5 | -3.4 | 470.2945 | 12.41 | 4848 | 6.43e3 | 1 | 1 | C4R3Y3|C4R3Y3\_KOMPG |  |  | DB Search |
| LIAGP | 22.13 | 469.29 | 5 | -3.4 | 470.2945 | 12.41 | 4848 | 6.43e3 | 1 | 1 | C4R1G1|C4R1G1\_KOMPG |  |  | DB Search |
| LLAGP | 22.13 | 469.29 | 5 | -3.4 | 470.2945 | 12.41 | 4848 | 6.43e3 | 1 | 1 | C4QVF2|C4QVF2\_KOMPG:C4R7J2|C4R7J2\_KOMPG:C4R2M2|C4R2M2\_KOMPG |  |  | DB Search |
| SLQLSG | 22.13 | 603.3228 | 6 | 3.43 | 604.3306 | 12.84 | 5062 | 1.54e3 | 1 | 1 | C4R6V5|C4R6V5\_KOMPG |  |  | DB Search |
| TIGM | 22.09 | 420.2043 | 4 | -5.45 | 421.2082 | 12.63 | 5019 | 3.01e3 | 1 | 1 | C4R2P8|C4R2P8\_KOMPG:C4QY42|C4QY42\_KOMPG:C4R8Q9|C4R8Q9\_KOMPG:C4QXV4|C4QXV4\_KOMPG |  |  | DB Search |
| TLGM | 22.09 | 420.2043 | 4 | -5.45 | 421.2082 | 12.63 | 5019 | 3.01e3 | 1 | 1 | C4R3F8|C4R3F8\_KOMPG:C4QV32|C4QV32\_KOMPG:C4R3D7|C4R3D7\_KOMPG:C4R7B5|C4R7B5\_KOMPG:C4R2T7|C4R2T7\_KOMPG:C4R6R7|C4R6R7\_KOMPG:C4R895|C4R895\_KOMPG:C4R368|C4R368\_KOMPG:C4QXM7|C4QXM7\_KOMPG:C4R867|C4R867\_KOMPG:C4R907|C4R907\_KOMPG:C4QUZ9|C4QUZ9\_KOMPG:C4R398|C4R398\_KOMPG:C4R2D0|C4R2D0\_KOMPG:C4R8B3|C4R8B3\_KOMPG |  |  | DB Search |
| IDGI | 22.09 | 416.2271 | 4 | -1.79 | 417.2326 | 13.94 | 5544 | 2.42e3 | 1 | 1 | C4QVV6|C4QVV6\_KOMPG:C4R8B8|C4R8B8\_KOMPG:C4R2Y2|C4R2Y2\_KOMPG:C4R687|C4R687\_KOMPG:C4QYU4|C4QYU4\_KOMPG:C4R4J6|C4R4J6\_KOMPG:C4R5E3|C4R5E3\_KOMPG:C4QZK8|C4QZK8\_KOMPG:C4R3B4|C4R3B4\_KOMPG:C4R1W3|C4R1W3\_KOMPG:C4QVU5|C4QVU5\_KOMPG:C4QYX7|C4QYX7\_KOMPG:C4QWD1|C4QWD1\_KOMPG:C4R6F8|C4R6F8\_KOMPG:C4QYB0|C4QYB0\_KOMPG:C4QVA1|C4QVA1\_KOMPG:C4R4Q8|C4R4Q8\_KOMPG:C4QY80|C4QY80\_KOMPG:C4R1E6|C4R1E6\_KOMPG:C4R9A8|C4R9A8\_KOMPG:C4R1Z8|C4R1Z8\_KOMPG:C4QZK2|C4QZK2\_KOMPG:C4R797|C4R797\_KOMPG |  |  | DB Search |
| IDGL | 22.09 | 416.2271 | 4 | -1.79 | 417.2326 | 13.94 | 5544 | 2.42e3 | 1 | 1 | C4QV04|C4QV04\_KOMPG:C4QYT7|C4QYT7\_KOMPG:C4QYL1|C4QYL1\_KOMPG:C4QZ59|C4QZ59\_KOMPG:Q9Y751|ATG26\_KOMPG:C4R032|C4R032\_KOMPG:C4R0I8|C4R0I8\_KOMPG:C4R6J0|C4R6J0\_KOMPG:C4R8D0|C4R8D0\_KOMPG:C4QWW6|C4QWW6\_KOMPG:C4QVL2|C4QVL2\_KOMPG:C4R8B1|C4R8B1\_KOMPG:C4R902|C4R902\_KOMPG:C4QYA6|C4QYA6\_KOMPG:C4R588|C4R588\_KOMPG:C4R228|C4R228\_KOMPG:C4R2C6|C4R2C6\_KOMPG:C4R1F8|C4R1F8\_KOMPG:C4R100|C4R100\_KOMPG:C4QXF0|C4QXF0\_KOMPG:C4QVK5|C4QVK5\_KOMPG:C4R332|C4R332\_KOMPG:C4R121|C4R121\_KOMPG:C4R0J8|C4R0J8\_KOMPG:C4R092|C4R092\_KOMPG:C4R425|C4R425\_KOMPG:C4R2J1|C4R2J1\_KOMPG |  |  | DB Search |
| LDGI | 22.09 | 416.2271 | 4 | -1.79 | 417.2326 | 13.94 | 5544 | 2.42e3 | 1 | 1 | C4R2Q4|C4R2Q4\_KOMPG:C4R779|C4R779\_KOMPG:C4R142|C4R142\_KOMPG:C4R2I2|C4R2I2\_KOMPG:C4R118|C4R118\_KOMPG:C4QZA1|C4QZA1\_KOMPG:C4QZZ8|C4QZZ8\_KOMPG:C4QZ83|C4QZ83\_KOMPG:C4QX32|C4QX32\_KOMPG:C4R4S8|C4R4S8\_KOMPG:C4QZT8|C4QZT8\_KOMPG:C4QXQ2|C4QXQ2\_KOMPG:C4QW55|C4QW55\_KOMPG:C4QW18|C4QW18\_KOMPG:C4R8A8|C4R8A8\_KOMPG:C4QVN3|C4QVN3\_KOMPG:C4QXM9|C4QXM9\_KOMPG:C4QV70|C4QV70\_KOMPG:C4QVG5|C4QVG5\_KOMPG:C4QVT4|C4QVT4\_KOMPG:C4R712|C4R712\_KOMPG:C4R3E1|C4R3E1\_KOMPG:C4QUZ2|C4QUZ2\_KOMPG:C4QV96|C4QV96\_KOMPG:C4QZB6|C4QZB6\_KOMPG:C4R7E8|C4R7E8\_KOMPG:C4R8X0|C4R8X0\_KOMPG:C4QWY6|C4QWY6\_KOMPG:C4R8D2|C4R8D2\_KOMPG:C4QXV5|C4QXV5\_KOMPG:C4R1W1|C4R1W1\_KOMPG:C4R8I1|C4R8I1\_KOMPG:C4R5V4|C4R5V4\_KOMPG:C4R7C5|C4R7C5\_KOMPG:C4R329|C4R329\_KOMPG:C4R625|C4R625\_KOMPG |  |  | DB Search |
| LDGL | 22.09 | 416.2271 | 4 | -1.79 | 417.2326 | 13.94 | 5544 | 2.42e3 | 1 | 1 | C4R428|C4R428\_KOMPG:C4R877|C4R877\_KOMPG:C4R3F4|C4R3F4\_KOMPG:C4R6Q8|C4R6Q8\_KOMPG:C4QUZ7|C4QUZ7\_KOMPG:C4R895|C4R895\_KOMPG:C4R1U2|C4R1U2\_KOMPG:C4R0M3|C4R0M3\_KOMPG:C4R5P0|C4R5P0\_KOMPG:C4QWT6|C4QWT6\_KOMPG:C4R4B0|C4R4B0\_KOMPG:C4R1N5|C4R1N5\_KOMPG:C4R2U0|C4R2U0\_KOMPG:C4R3R5|C4R3R5\_KOMPG:C4R1H3|C4R1H3\_KOMPG:C4R766|C4R766\_KOMPG:C4QXX4|C4QXX4\_KOMPG:C4QVZ3|C4QVZ3\_KOMPG:C4R093|C4R093\_KOMPG:C4R6W7|C4R6W7\_KOMPG:C4R191|C4R191\_KOMPG:C4R034|C4R034\_KOMPG:C4QX75|C4QX75\_KOMPG:C4R516|C4R516\_KOMPG:C4R6I1|C4R6I1\_KOMPG:C4R165|C4R165\_KOMPG:C4QZG6|C4QZG6\_KOMPG:C4R6J2|C4R6J2\_KOMPG:C4R5C9|C4R5C9\_KOMPG:C4R7C8|C4R7C8\_KOMPG:C4R7U3|C4R7U3\_KOMPG:C4R3Q7|C4R3Q7\_KOMPG:C4R7Q9|C4R7Q9\_KOMPG:C4QWZ8|C4QWZ8\_KOMPG:C4R754|C4R754\_KOMPG:C4QVX7|C4QVX7\_KOMPG:C4QY10|C4QY10\_KOMPG:C4R9D7|C4R9D7\_KOMPG:C4R2W5|C4R2W5\_KOMPG:C4R3H2|C4R3H2\_KOMPG:C4R5A7|C4R5A7\_KOMPG:C4R1J0|C4R1J0\_KOMPG:C4R8B3|C4R8B3\_KOMPG |  |  | DB Search |
| SHKDWR | 22.06 | 827.4038 | 6 | 9.61 | 828.417 | 14.99 | 5914 | 7.04e2 | 1 | 1 | C4R0P1|C4R0P1\_KOMPG |  |  | DB Search |
| EV | 22.06 | 246.1216 | 2 | -3.05 | 247.1275 | 3.37 | 1479 | 1.39e4 | 2 | 2 | C4QZ38|C4QZ38\_KOMPG:C4R8H4|C4R8H4\_KOMPG:C4R6G4|C4R6G4\_KOMPG:C4R554|C4R554\_KOMPG:C4R7Q3|C4R7Q3\_KOMPG:C4QV84|C4QV84\_KOMPG:C4QZZ8|C4QZZ8\_KOMPG:C4R5F6|C4R5F6\_KOMPG:C4QY19|C4QY19\_KOMPG:C4R1N5|C4R1N5\_KOMPG:C4R1J4|C4R1J4\_KOMPG:C4QW53|C4QW53\_KOMPG:C4R2A4|C4R2A4\_KOMPG:C4QYG5|C4QYG5\_KOMPG:C4QW31|C4QW31\_KOMPG:C4R4G9|OXDA\_KOMPG:C4R912|C4R912\_KOMPG:C4R4X8|C4R4X8\_KOMPG:C4R0S5|C4R0S5\_KOMPG:C4R042|C4R042\_KOMPG:C4R4C7|C4R4C7\_KOMPG:C4R5J2|C4R5J2\_KOMPG:C4R6E1|C4R6E1\_KOMPG:C4R3T0|C4R3T0\_KOMPG:C4R0G2|C4R0G2\_KOMPG:C4R0P1|C4R0P1\_KOMPG:C4R8I1|C4R8I1\_KOMPG:C4QYI8|C4QYI8\_KOMPG:C4QYV2|C4QYV2\_KOMPG:C4R4N6|C4R4N6\_KOMPG:C4QVP0|C4QVP0\_KOMPG:C4QX17|C4QX17\_KOMPG:C4QXL0|C4QXL0\_KOMPG:C4R0B6|C4R0B6\_KOMPG:C4R877|C4R877\_KOMPG:C4R8S4|C4R8S4\_KOMPG:C4R7N8|C4R7N8\_KOMPG:C4QW84|C4QW84\_KOMPG:C4QV98|C4QV98\_KOMPG:C4R0V8|C4R0V8\_KOMPG:C4R0X5|C4R0X5\_KOMPG:C4R959|C4R959\_KOMPG:C4R2W1|C4R2W1\_KOMPG:C4R5D6|C4R5D6\_KOMPG:C4R093|C4R093\_KOMPG:C4QZX0|C4QZX0\_KOMPG:C4R7S9|GET3\_KOMPG:C4R129|C4R129\_KOMPG:C4QY04|C4QY04\_KOMPG:C4R692|C4R692\_KOMPG:C4QVT9|C4QVT9\_KOMPG:C4R4T5|C4R4T5\_KOMPG:C4R3L2|C4R3L2\_KOMPG:C4QWS9|C4QWS9\_KOMPG:C4QZT6|C4QZT6\_KOMPG:C4R503|C4R503\_KOMPG:C4QYK4|C4QYK4\_KOMPG:C4R2W0|C4R2W0\_KOMPG |  |  | DB Search |
| KDII | 22.06 | 487.3006 | 4 | -0.76 | 488.3063 | 10.37 | 4062 | 0 | 0 | 0 | C4R492|OCA5\_KOMPG:C4QZ97|C4QZ97\_KOMPG:C4QZU5|C4QZU5\_KOMPG:C4R919|C4R919\_KOMPG:C4R895|C4R895\_KOMPG:C4R0S7|C4R0S7\_KOMPG:C4R3Q5|C4R3Q5\_KOMPG:C4QWU8|C4QWU8\_KOMPG:C4R271|C4R271\_KOMPG:C4QXA2|C4QXA2\_KOMPG:C4QWF2|C4QWF2\_KOMPG:C4R330|C4R330\_KOMPG:C4QWU0|C4QWU0\_KOMPG:C4QWD1|C4QWD1\_KOMPG:C4QX57|C4QX57\_KOMPG:C4QZU0|C4QZU0\_KOMPG:C4R0J2|C4R0J2\_KOMPG:C4QYT1|C4QYT1\_KOMPG:C4R824|C4R824\_KOMPG:C4R3V3|C4R3V3\_KOMPG:C4R8P5|C4R8P5\_KOMPG |  |  | DB Search |
| KDIL | 22.06 | 487.3006 | 4 | -0.76 | 488.3063 | 10.37 | 4062 | 0 | 0 | 0 | C4QVT8|C4QVT8\_KOMPG:C4R3Z4|C4R3Z4\_KOMPG:C4R5G1|C4R5G1\_KOMPG:C4QW03|C4QW03\_KOMPG:C4QZU3|C4QZU3\_KOMPG:C4R5C7|C4R5C7\_KOMPG:C4QZ69|C4QZ69\_KOMPG:C4R8T0|C4R8T0\_KOMPG:C4R3W9|C4R3W9\_KOMPG:C4R8Y6|C4R8Y6\_KOMPG:C4R4G1|C4R4G1\_KOMPG:C4QYP0|C4QYP0\_KOMPG:C4R340|C4R340\_KOMPG:C4R801|C4R801\_KOMPG:C4R1Y3|C4R1Y3\_KOMPG:C4R0N6|C4R0N6\_KOMPG:C4R956|C4R956\_KOMPG:C4R1B9|C4R1B9\_KOMPG:C4R7L8|C4R7L8\_KOMPG:C4R8Y8|C4R8Y8\_KOMPG:C4QV63|C4QV63\_KOMPG:C4R8Y0|C4R8Y0\_KOMPG:C4R7C8|C4R7C8\_KOMPG:C4QVA1|C4QVA1\_KOMPG:C4QZQ6|C4QZQ6\_KOMPG:C4R855|C4R855\_KOMPG:C4R5C8|C4R5C8\_KOMPG |  |  | DB Search |
| KDLI | 22.06 | 487.3006 | 4 | -0.76 | 488.3063 | 10.37 | 4062 | 0 | 0 | 0 | C4QXT7|C4QXT7\_KOMPG:C4R6Q8|C4R6Q8\_KOMPG:C4QZN9|C4QZN9\_KOMPG:C4QYR2|C4QYR2\_KOMPG:C4QXH8|C4QXH8\_KOMPG:C4R4B1|C4R4B1\_KOMPG:C4QV66|C4QV66\_KOMPG:C4QZ24|C4QZ24\_KOMPG:C4R162|C4R162\_KOMPG:C4R848|C4R848\_KOMPG:C4R568|C4R568\_KOMPG:C4R6V0|C4R6V0\_KOMPG:C4R463|C4R463\_KOMPG:C4R5G4|C4R5G4\_KOMPG:C4QXC5|C4QXC5\_KOMPG:C4R7Q2|C4R7Q2\_KOMPG:C4R5X5|C4R5X5\_KOMPG:C4QXK3|C4QXK3\_KOMPG:C4R579|C4R579\_KOMPG:C4R8K4|C4R8K4\_KOMPG:C4QWQ4|C4QWQ4\_KOMPG:C4R7R8|C4R7R8\_KOMPG:C4R4A0|C4R4A0\_KOMPG:C4R366|C4R366\_KOMPG:C4QXI3|C4QXI3\_KOMPG:C4R6E8|C4R6E8\_KOMPG |  |  | DB Search |
| KDLL | 22.06 | 487.3006 | 4 | -0.76 | 488.3063 | 10.37 | 4062 | 0 | 0 | 0 | C4R3S7|C4R3S7\_KOMPG:C4QXL0|C4QXL0\_KOMPG:C4R3J0|C4R3J0\_KOMPG:C4R5W5|C4R5W5\_KOMPG:C4QYT7|C4QYT7\_KOMPG:C4R211|C4R211\_KOMPG:C4QX89|C4QX89\_KOMPG:C4QXQ8|C4QXQ8\_KOMPG:C4R032|C4R032\_KOMPG:C4R4V3|C4R4V3\_KOMPG:C4QV84|C4QV84\_KOMPG:C4R0S9|C4R0S9\_KOMPG:C4R083|C4R083\_KOMPG:C4QX18|C4QX18\_KOMPG:C4R7F1|C4R7F1\_KOMPG:C4QYQ2|C4QYQ2\_KOMPG:C4R350|C4R350\_KOMPG:C4R286|C4R286\_KOMPG:C4R5Y3|C4R5Y3\_KOMPG:C4R068|C4R068\_KOMPG:C4QZV0|C4QZV0\_KOMPG:C4R5H5|C4R5H5\_KOMPG:C4R2K7|C4R2K7\_KOMPG:C4R0Q0|C4R0Q0\_KOMPG:C4R678|C4R678\_KOMPG:C4R3M5|C4R3M5\_KOMPG:C4QV05|C4QV05\_KOMPG:C4R0D7|C4R0D7\_KOMPG:C4QW27|C4QW27\_KOMPG:C4R3N3|C4R3N3\_KOMPG:C4R2C5|C4R2C5\_KOMPG:C4R370|C4R370\_KOMPG:C4QYY0|C4QYY0\_KOMPG:C4R924|C4R924\_KOMPG:C4R8Z1|C4R8Z1\_KOMPG:C4QZ08|C4QZ08\_KOMPG:C4R5J2|C4R5J2\_KOMPG:P04842|ALOX1\_KOMPG:C4R6C4|C4R6C4\_KOMPG:C4R3L2|C4R3L2\_KOMPG:C4R702|ALOX2\_KOMPG:C4QXZ9|C4QXZ9\_KOMPG:C4R6Z4|C4R6Z4\_KOMPG:C4R2C0|C4R2C0\_KOMPG |  |  | DB Search |
| SRIPQ(+0.98)YG | 22.05 | 820.4079 | 7 | 5.43 | 821.4176 | 12.64 | 5004 | 4.29e2 | 2 | 2 | C4R581|C4R581\_KOMPG | Deamidation (NQ) | Q5:Deamidation (NQ):1000 | DB Search |
| ERPLDSVVD | 22.01 | 1028.5138 | 9 | -3.3 | 1029.5151 | 30.42 | 10438 | 1.01e2 | 1 | 1 | C4R3M1|C4R3M1\_KOMPG |  |  | DB Search |
| SIAI | 21.98 | 402.2478 | 4 | -2.21 | 403.2532 | 20.49 | 7711 | 0 | 0 | 0 | C4R1B9|C4R1B9\_KOMPG:C4QZ47|C4QZ47\_KOMPG:C4R3U2|C4R3U2\_KOMPG:C4R7N8|C4R7N8\_KOMPG:C4R7Y0|C4R7Y0\_KOMPG:C4R8Y2|C4R8Y2\_KOMPG:C4R1N5|C4R1N5\_KOMPG:C4QVC0|C4QVC0\_KOMPG:C4R1H3|C4R1H3\_KOMPG:C4R6T2|C4R6T2\_KOMPG:C4QYJ7|C4QYJ7\_KOMPG:C4R6V0|C4R6V0\_KOMPG:C4R713|C4R713\_KOMPG |  |  | DB Search |
| SIAL | 21.98 | 402.2478 | 4 | -2.21 | 403.2532 | 20.49 | 7711 | 0 | 0 | 0 | C4R675|C4R675\_KOMPG:C4QY85|C4QY85\_KOMPG:C4R8R2|C4R8R2\_KOMPG:C4R644|C4R644\_KOMPG:C4R4K8|C4R4K8\_KOMPG:C4R1C5|C4R1C5\_KOMPG:C4QXU1|C4QXU1\_KOMPG:C4R8D0|C4R8D0\_KOMPG:C4R1E5|C4R1E5\_KOMPG:C4R3L8|C4R3L8\_KOMPG:C4R9B4|C4R9B4\_KOMPG:C4R3Q7|C4R3Q7\_KOMPG:C4R2Q0|C4R2Q0\_KOMPG:C4R565|C4R565\_KOMPG:C4R902|C4R902\_KOMPG:C4R3R5|C4R3R5\_KOMPG:C4R219|C4R219\_KOMPG:C4R8B9|C4R8B9\_KOMPG:C4QVW7|C4QVW7\_KOMPG:C4R6Q6|C4R6Q6\_KOMPG:C4R1N6|C4R1N6\_KOMPG:C4QV97|C4QV97\_KOMPG:C4R721|C4R721\_KOMPG:C4QVT3|C4QVT3\_KOMPG |  |  | DB Search |
| SLAI | 21.98 | 402.2478 | 4 | -2.21 | 403.2532 | 20.49 | 7711 | 0 | 0 | 0 | C4R3M3|C4R3M3\_KOMPG:C4R4I3|C4R4I3\_KOMPG:C4QYU4|C4QYU4\_KOMPG:C4R2S7|C4R2S7\_KOMPG:C4QVX3|C4QVX3\_KOMPG:C4R8N7|C4R8N7\_KOMPG:C4R7V7|C4R7V7\_KOMPG:C4QVS5|C4QVS5\_KOMPG:C4R698|C4R698\_KOMPG:C4R162|C4R162\_KOMPG:C4R8J6|C4R8J6\_KOMPG:C4R1X4|C4R1X4\_KOMPG:C4R016|C4R016\_KOMPG:C4R110|C4R110\_KOMPG:C4R385|C4R385\_KOMPG |  |  | DB Search |
| SLAL | 21.98 | 402.2478 | 4 | -2.21 | 403.2532 | 20.49 | 7711 | 0 | 0 | 0 | C4R6M4|C4R6M4\_KOMPG:C4R5F3|C4R5F3\_KOMPG:C4R3D7|C4R3D7\_KOMPG:C4QXK0|C4QXK0\_KOMPG:C4R119|C4R119\_KOMPG:C4QXX5|C4QXX5\_KOMPG:C4QXI8|PEX6\_KOMPG:C4QXB1|C4QXB1\_KOMPG:C4R441|C4R441\_KOMPG:C4R0V8|C4R0V8\_KOMPG:C4QZ69|C4QZ69\_KOMPG:C4QVQ0|C4QVQ0\_KOMPG:C4QYQ2|C4QYQ2\_KOMPG:C4QYH3|C4QYH3\_KOMPG:C4R0U8|C4R0U8\_KOMPG:C4R2D2|C4R2D2\_KOMPG:C4QVB2|C4QVB2\_KOMPG:C4R8E5|C4R8E5\_KOMPG:C4R4K2|C4R4K2\_KOMPG:C4R4F5|C4R4F5\_KOMPG:C4QUZ2|C4QUZ2\_KOMPG:C4R2P9|C4R2P9\_KOMPG:C4R241|C4R241\_KOMPG:C4R1W8|C4R1W8\_KOMPG:C4R288|C4R288\_KOMPG:C4R692|C4R692\_KOMPG:C4R7W9|C4R7W9\_KOMPG:C4QW65|C4QW65\_KOMPG:C4R924|C4R924\_KOMPG:C4R0X3|C4R0X3\_KOMPG:C4R2E1|C4R2E1\_KOMPG:C4R8J5|C4R8J5\_KOMPG:C4R7C8|C4R7C8\_KOMPG:C4R6H0|C4R6H0\_KOMPG:C4QWD1|C4QWD1\_KOMPG:C4R5H3|C4R5H3\_KOMPG:C4QVT0|C4QVT0\_KOMPG:C4QXZ9|C4QXZ9\_KOMPG:C4R280|C4R280\_KOMPG:C4R749|C4R749\_KOMPG:C4QWK9|C4QWK9\_KOMPG:C4R3T3|C4R3T3\_KOMPG:C4R176|C4R176\_KOMPG:C4R8A0|C4R8A0\_KOMPG |  |  | DB Search |
| TGALVVG | 21.94 | 615.3591 | 7 | -0.84 | 616.3644 | 13.63 | 5356 | 2.79e3 | 1 | 1 | C4R6L5|C4R6L5\_KOMPG |  |  | DB Search |
| DVYGG | 21.94 | 509.2122 | 5 | -4.25 | 510.216 | 5.77 | 2278 | 7.88e3 | 1 | 1 | C4QXB5|C4QXB5\_KOMPG:C4QYL9|C4QYL9\_KOMPG |  |  | DB Search |
| SGVI | 21.93 | 374.2165 | 4 | -4.88 | 375.221 | 11.77 | 4666 | 3.28e3 | 1 | 1 | C4QVY1|C4QVY1\_KOMPG:C4R278|C4R278\_KOMPG:C4R5V1|C4R5V1\_KOMPG:C4QYH1|C4QYH1\_KOMPG:C4QWS2|C4QWS2\_KOMPG:C4R2Q9|C4R2Q9\_KOMPG:C4R1S9|C4R1S9\_KOMPG:C4QWE6|C4QWE6\_KOMPG:C4QVE4|C4QVE4\_KOMPG:C4R6J9|C4R6J9\_KOMPG:C4QVU5|C4QVU5\_KOMPG:C4R2V3|C4R2V3\_KOMPG:C4QVE2|C4QVE2\_KOMPG:C4R517|C4R517\_KOMPG:C4R846|C4R846\_KOMPG:C4QWE8|C4QWE8\_KOMPG:C4R0A2|C4R0A2\_KOMPG:C4R1U8|C4R1U8\_KOMPG:C4QYH0|C4QYH0\_KOMPG:C4R8D5|C4R8D5\_KOMPG:C4R768|C4R768\_KOMPG:C4R6X9|C4R6X9\_KOMPG:C4R323|C4R323\_KOMPG:C4R1C3|C4R1C3\_KOMPG:C4R983|C4R983\_KOMPG:C4R2M9|C4R2M9\_KOMPG:C4R604|C4R604\_KOMPG:C4R0D0|C4R0D0\_KOMPG:C4R573|C4R573\_KOMPG:C4QXY8|C4QXY8\_KOMPG:C4QW16|C4QW16\_KOMPG:C4R5B1|C4R5B1\_KOMPG:C4R0R6|C4R0R6\_KOMPG:C4QZQ7|C4QZQ7\_KOMPG:C4R7Z8|BMT4\_KOMPG:C4QZ00|C4QZ00\_KOMPG:C4R965|C4R965\_KOMPG |  |  | DB Search |
| SGVL | 21.93 | 374.2165 | 4 | -4.88 | 375.221 | 11.77 | 4666 | 3.28e3 | 1 | 1 | C4QXY4|C4QXY4\_KOMPG:C4R1B3|C4R1B3\_KOMPG:C4QZA8|C4QZA8\_KOMPG:C4QW60|C4QW60\_KOMPG:C4R1D2|C4R1D2\_KOMPG:C4R3F6|C4R3F6\_KOMPG:C4R6K3|C4R6K3\_KOMPG:C4QXN4|C4QXN4\_KOMPG:C4R936|C4R936\_KOMPG:C4R4N4|C4R4N4\_KOMPG:C4R852|C4R852\_KOMPG:C4QVL3|C4QVL3\_KOMPG:C4QW59|C4QW59\_KOMPG:C4R2B6|C4R2B6\_KOMPG:C4R566|C4R566\_KOMPG:C4QY33|C4QY33\_KOMPG:C4QV09|C4QV09\_KOMPG:C4R7M4|C4R7M4\_KOMPG:C4R7T4|C4R7T4\_KOMPG:C4R454|C4R454\_KOMPG:C4QYY2|C4QYY2\_KOMPG:C4QZL1|C4QZL1\_KOMPG:C4QWP7|C4QWP7\_KOMPG:C4R7F0|C4R7F0\_KOMPG:C4R799|C4R799\_KOMPG:C4R0L6|C4R0L6\_KOMPG:C4QYC3|C4QYC3\_KOMPG:C4R4M0|C4R4M0\_KOMPG:C4R0F4|C4R0F4\_KOMPG:C4QWC1|C4QWC1\_KOMPG:C4QZ27|C4QZ27\_KOMPG:C4R069|C4R069\_KOMPG:C4QX47|C4QX47\_KOMPG:C4QXR7|C4QXR7\_KOMPG:C4R6H0|C4R6H0\_KOMPG:C4R3V3|C4R3V3\_KOMPG:C4R8V4|C4R8V4\_KOMPG |  |  | DB Search |
| VGSI | 21.91 | 374.2165 | 4 | 0.34 | 375.223 | 13.82 | 5444 | 2.07e3 | 1 | 1 | C4R9D9|C4R9D9\_KOMPG:C4QX01|C4QX01\_KOMPG:C4QZA8|C4QZA8\_KOMPG:C4R208|C4R208\_KOMPG:C4R6R3|C4R6R3\_KOMPG:C4R8R2|C4R8R2\_KOMPG:C4R583|C4R583\_KOMPG:C4R006|C4R006\_KOMPG:C4R564|C4R564\_KOMPG:C4QXW7|C4QXW7\_KOMPG:C4R1G5|C4R1G5\_KOMPG:C4QVN3|C4QVN3\_KOMPG:C4R3M7|C4R3M7\_KOMPG:C4R8J7|C4R8J7\_KOMPG:C4R021|C4R021\_KOMPG:C4R2M9|C4R2M9\_KOMPG:C4QYQ3|C4QYQ3\_KOMPG:Q92448|PFKA1\_KOMPG:C4QZQ7|C4QZQ7\_KOMPG:C4QX23|C4QX23\_KOMPG:C4QZN2|C4QZN2\_KOMPG:C4R486|C4R486\_KOMPG:C4R1S6|C4R1S6\_KOMPG:C4R087|C4R087\_KOMPG:C4R2Q4|C4R2Q4\_KOMPG:C4QZI1|C4QZI1\_KOMPG:C4QX89|C4QX89\_KOMPG:C4R3C2|C4R3C2\_KOMPG:C4QVC1|C4QVC1\_KOMPG:C4R0M3|C4R0M3\_KOMPG:C4R774|C4R774\_KOMPG:C4QWT6|C4QWT6\_KOMPG:C4R3D6|C4R3D6\_KOMPG:C4R7G9|C4R7G9\_KOMPG:C4R517|C4R517\_KOMPG:C4R5T3|C4R5T3\_KOMPG:C4R7I3|C4R7I3\_KOMPG:C4R8I0|C4R8I0\_KOMPG:C4R898|C4R898\_KOMPG:C4R2C6|C4R2C6\_KOMPG:C4QXA5|PFKA2\_KOMPG:C4R365|C4R365\_KOMPG:C4R9A9|C4R9A9\_KOMPG:C4QVT5|C4QVT5\_KOMPG:C4R0D0|C4R0D0\_KOMPG:C4QX80|PSD1\_KOMPG:C4R4K3|C4R4K3\_KOMPG:C4QUZ0|C4QUZ0\_KOMPG:C4R360|PSD2\_KOMPG:C4R946|C4R946\_KOMPG:C4QX33|C4QX33\_KOMPG |  |  | DB Search |
| VGSL | 21.91 | 374.2165 | 4 | 0.34 | 375.223 | 13.82 | 5444 | 2.07e3 | 1 | 1 | C4QWI8|C4QWI8\_KOMPG:C4R4L0|C4R4L0\_KOMPG:C4QY98|C4QY98\_KOMPG:C4R003|C4R003\_KOMPG:C4QZZ6|C4QZZ6\_KOMPG:C4R3L9|C4R3L9\_KOMPG:C4R751|C4R751\_KOMPG:C4QX22|C4QX22\_KOMPG:C4R8B9|C4R8B9\_KOMPG:C4R8Z0|C4R8Z0\_KOMPG:C4R2V9|C4R2V9\_KOMPG:C4R6R6|C4R6R6\_KOMPG:C4R0U6|C4R0U6\_KOMPG:C4R6E2|C4R6E2\_KOMPG:C4R2C5|C4R2C5\_KOMPG:C4QVF4|C4QVF4\_KOMPG:C4R8A6|C4R8A6\_KOMPG:C4R804|C4R804\_KOMPG:C4R5R6|C4R5R6\_KOMPG:C4R2N2|C4R2N2\_KOMPG:C4R7J5|C4R7J5\_KOMPG:C4R2N4|C4R2N4\_KOMPG:C4R3H6|C4R3H6\_KOMPG:C4QWC5|C4QWC5\_KOMPG:C4QVF7|C4QVF7\_KOMPG:C4R1K4|C4R1K4\_KOMPG:C4QW13|C4QW13\_KOMPG:C4R4W7|C4R4W7\_KOMPG:C4R6V9|C4R6V9\_KOMPG:C4R070|C4R070\_KOMPG:C4R6L2|C4R6L2\_KOMPG:C4QW84|C4QW84\_KOMPG:C4QZH7|C4QZH7\_KOMPG:C4R3V9|C4R3V9\_KOMPG:C4R0Q9|C4R0Q9\_KOMPG:C4R7A7|C4R7A7\_KOMPG:C4QWE4|C4QWE4\_KOMPG:C4QW37|C4QW37\_KOMPG:C4R4K9|C4R4K9\_KOMPG:C4QZL9|C4QZL9\_KOMPG:C4R685|C4R685\_KOMPG:C4R3T2|C4R3T2\_KOMPG:C4R5D6|C4R5D6\_KOMPG:C4R3B7|C4R3B7\_KOMPG:C4R7B6|C4R7B6\_KOMPG:C4R4F5|C4R4F5\_KOMPG:C4R4K7|C4R4K7\_KOMPG:C4R4A2|C4R4A2\_KOMPG:C4R0D7|C4R0D7\_KOMPG:C4R2B4|C4R2B4\_KOMPG:C4R4T2|C4R4T2\_KOMPG:C4R6M8|C4R6M8\_KOMPG:C4R5J6|C4R5J6\_KOMPG:C4QYC4|C4QYC4\_KOMPG:C4QX54|C4QX54\_KOMPG:C4R6S4|C4R6S4\_KOMPG:C4R047|C4R047\_KOMPG:C4R0B8|C4R0B8\_KOMPG:C4R0B3|C4R0B3\_KOMPG:C4R317|C4R317\_KOMPG:C4R2V7|C4R2V7\_KOMPG:C4R4Q5|C4R4Q5\_KOMPG:C4R5E1|C4R5E1\_KOMPG:C4R5E5|C4R5E5\_KOMPG:C4QZS6|C4QZS6\_KOMPG:C4QXX3|C4QXX3\_KOMPG:C4QYM3|C4QYM3\_KOMPG:C4R7R5|C4R7R5\_KOMPG:C4R8W9|C4R8W9\_KOMPG:C4QWG7|C4QWG7\_KOMPG:C4R018|C4R018\_KOMPG:C4QXS2|C4QXS2\_KOMPG |  |  | DB Search |
| TFET | 21.91 | 496.2169 | 4 | -4.8 | 497.2206 | 10.16 | 3900 | 3.34e2 | 1 | 1 | C4R8H9|C4R8H9\_KOMPG:C4QVG7|C4QVG7\_KOMPG:C4R325|C4R325\_KOMPG:C4R5B8|C4R5B8\_KOMPG:C4R660|C4R660\_KOMPG:C4R4E3|C4R4E3\_KOMPG:C4R687|C4R687\_KOMPG:C4R8X0|C4R8X0\_KOMPG:C4QXU3|C4QXU3\_KOMPG:C4R9A9|C4R9A9\_KOMPG:C4QV84|C4QV84\_KOMPG:C4R6C4|C4R6C4\_KOMPG:C4QY33|C4QY33\_KOMPG:C4QVD4|C4QVD4\_KOMPG:C4R3A8|C4R3A8\_KOMPG:C4R3S2|C4R3S2\_KOMPG:C4R5M9|C4R5M9\_KOMPG:C4QVH7|C4QVH7\_KOMPG:C4R454|C4R454\_KOMPG:C4R2C0|C4R2C0\_KOMPG |  |  | DB Search |
| Q(+0.98)PQ(+0.98)QPQQPQ | 21.83 | 1079.4884 | 9 | -0.25 | 1080.4927 | 31.88 | 10869 | 1.58e2 | 2 | 2 | C4QXV6|C4QXV6\_KOMPG | Deamidation (NQ), Deamidation (NQ) | Q1:Deamidation (NQ):0 Q3:Deamidation (NQ):0 | DB Search |
| TGIGLALVG | 21.83 | 799.4803 | 9 | -9.03 | 800.4784 | 32.22 | 10935 | 6.06e2 | 1 | 1 | C4R2R4|C4R2R4\_KOMPG |  |  | DB Search |
| VSVVD | 21.8 | 517.2748 | 5 | -5.76 | 518.2778 | 9.99 | 3883 | 0 | 0 | 0 | C4R3D7|C4R3D7\_KOMPG:C4R0P1|C4R0P1\_KOMPG:C4QV61|C4QV61\_KOMPG |  |  | DB Search |
| QAIP | 21.78 | 427.2431 | 4 | -3.04 | 428.248 | 11.76 | 4638 | 6.31e3 | 1 | 1 | C4QY30|C4QY30\_KOMPG:C4R7X8|BMT2\_KOMPG:C4QZC5|C4QZC5\_KOMPG:C4R750|C4R750\_KOMPG:C4R2V0|C4R2V0\_KOMPG:C4R5P0|C4R5P0\_KOMPG:C4QYV4|C4QYV4\_KOMPG:C4QXU5|C4QXU5\_KOMPG:C4R4J8|C4R4J8\_KOMPG:C4R5R1|C4R5R1\_KOMPG:C4QY37|C4QY37\_KOMPG:C4QYD9|C4QYD9\_KOMPG:C4R206|C4R206\_KOMPG:C4QZ06|BMT3\_KOMPG |  |  | DB Search |
| QALP | 21.78 | 427.2431 | 4 | -3.04 | 428.248 | 11.76 | 4638 | 6.31e3 | 1 | 1 | C4QZP3|C4QZP3\_KOMPG:C4R5M5|C4R5M5\_KOMPG:C4QYA5|C4QYA5\_KOMPG:C4R8V7|C4R8V7\_KOMPG:C4R0F6|C4R0F6\_KOMPG:C4QXG6|C4QXG6\_KOMPG:C4R7G0|C4R7G0\_KOMPG:C4R0B8|C4R0B8\_KOMPG:C4QZ19|C4QZ19\_KOMPG:C4R8V8|C4R8V8\_KOMPG:C4QVI5|C4QVI5\_KOMPG:C4R286|C4R286\_KOMPG:C4QVJ4|C4QVJ4\_KOMPG:C4QXM9|C4QXM9\_KOMPG:C4QVC2|C4QVC2\_KOMPG:C4R104|C4R104\_KOMPG:C4R5H5|C4R5H5\_KOMPG |  |  | DB Search |
| IGTGF | 21.73 | 493.2536 | 5 | -5.76 | 494.2568 | 16.27 | 6433 | 1.51e4 | 1 | 1 | C4R6J3|C4R6J3\_KOMPG |  |  | DB Search |
| LGTGF | 21.73 | 493.2536 | 5 | -5.76 | 494.2568 | 16.27 | 6433 | 1.51e4 | 1 | 1 | C4QZI6|C4QZI6\_KOMPG:C4R4I0|C4R4I0\_KOMPG:C4R931|C4R931\_KOMPG:C4R1Z3|C4R1Z3\_KOMPG:C4QWT8|C4QWT8\_KOMPG |  |  | DB Search |
| EIF | 21.7 | 407.2056 | 3 | -8.99 | 408.2082 | 29.23 | 10157 | 9.42e2 | 1 | 1 | C4R8Y4|C4R8Y4\_KOMPG:C4QWE1|C4QWE1\_KOMPG:C4R0N9|C4R0N9\_KOMPG:C4R2Y2|C4R2Y2\_KOMPG:C4R8S4|C4R8S4\_KOMPG:C4R1H2|C4R1H2\_KOMPG:C4R0I8|C4R0I8\_KOMPG:C4R310|C4R310\_KOMPG:C4R7V7|C4R7V7\_KOMPG:C4QZZ8|C4QZZ8\_KOMPG:C4R0V8|C4R0V8\_KOMPG:C4R5W8|C4R5W8\_KOMPG:C4QYP0|C4QYP0\_KOMPG:C4R588|C4R588\_KOMPG:C4R6B2|C4R6B2\_KOMPG:C4R7H2|C4R7H2\_KOMPG:C4QVA0|C4QVA0\_KOMPG:C4QYA1|C4QYA1\_KOMPG:C4QWH3|C4QWH3\_KOMPG:C4R622|C4R622\_KOMPG:C4QVT4|C4QVT4\_KOMPG:C4R7I5|C4R7I5\_KOMPG:C4R3W1|C4R3W1\_KOMPG:C4QYY0|C4QYY0\_KOMPG:C4R5X0|C4R5X0\_KOMPG:C4QXT6|C4QXT6\_KOMPG:C4R2X7|C4R2X7\_KOMPG:C4QYJ8|C4QYJ8\_KOMPG:C4R6X6|C4R6X6\_KOMPG:C4QXU1|C4QXU1\_KOMPG:C4R7R8|C4R7R8\_KOMPG:C4R6I2|C4R6I2\_KOMPG:C4R6E1|C4R6E1\_KOMPG:C4QWD1|C4QWD1\_KOMPG:C4QXU5|C4QXU5\_KOMPG:C4R2G2|C4R2G2\_KOMPG:C4R332|C4R332\_KOMPG:C4R095|C4R095\_KOMPG:C4R2K9|C4R2K9\_KOMPG:C4QXZ9|C4QXZ9\_KOMPG:C4R0L5|C4R0L5\_KOMPG:C4QYK4|C4QYK4\_KOMPG:C4R322|C4R322\_KOMPG |  |  | DB Search |
| ELF | 21.7 | 407.2056 | 3 | -8.99 | 408.2082 | 29.23 | 10157 | 9.42e2 | 1 | 1 | C4QX92|C4QX92\_KOMPG:C4R8N1|C4R8N1\_KOMPG:C4QZW7|C4QZW7\_KOMPG:C4R0H0|C4R0H0\_KOMPG:C4QYR2|C4QYR2\_KOMPG:C4QY19|C4QY19\_KOMPG:C4R4S7|C4R4S7\_KOMPG:C4R3G3|C4R3G3\_KOMPG:C4R7C2|C4R7C2\_KOMPG:C4R185|C4R185\_KOMPG:C4QW93|C4QW93\_KOMPG:C4R964|C4R964\_KOMPG:C4QX76|C4QX76\_KOMPG:C4QVQ7|C4QVQ7\_KOMPG:C4R0X2|C4R0X2\_KOMPG:C4R661|C4R661\_KOMPG:C4R5H5|C4R5H5\_KOMPG:C4R3Z8|C4R3Z8\_KOMPG:C4R7W9|C4R7W9\_KOMPG:C4R9B2|C4R9B2\_KOMPG:C4R924|C4R924\_KOMPG:C4R8B5|C4R8B5\_KOMPG:C4QYT3|C4QYT3\_KOMPG:C4QX63|C4QX63\_KOMPG:C4R1G9|C4R1G9\_KOMPG:C4QY28|C4QY28\_KOMPG:C4R4C7|C4R4C7\_KOMPG:C4R5J2|C4R5J2\_KOMPG:C4R1E3|C4R1E3\_KOMPG:C4R3L8|C4R3L8\_KOMPG:C4R639|C4R639\_KOMPG:C4QWR1|C4QWR1\_KOMPG:C4R6D7|C4R6D7\_KOMPG:C4R349|C4R349\_KOMPG:C4QYM4|C4QYM4\_KOMPG:C4R0J8|C4R0J8\_KOMPG:C4QYV2|C4QYV2\_KOMPG:C4R0E2|C4R0E2\_KOMPG:C4R4Q3|C4R4Q3\_KOMPG:C4QZN7|C4QZN7\_KOMPG:C4R5I2|C4R5I2\_KOMPG:C4R154|C4R154\_KOMPG:C4R9E2|C4R9E2\_KOMPG:C4QWR3|C4QWR3\_KOMPG:C4R052|C4R052\_KOMPG:C4R233|C4R233\_KOMPG:C4QXP3|C4QXP3\_KOMPG:C4R0B6|C4R0B6\_KOMPG:C4R877|C4R877\_KOMPG:C4R688|C4R688\_KOMPG:C4R921|C4R921\_KOMPG:C4R7N8|C4R7N8\_KOMPG:C4QYZ3|C4QYZ3\_KOMPG:C4R0X4|C4R0X4\_KOMPG:C4QVX3|C4QVX3\_KOMPG:C4R7W8|C4R7W8\_KOMPG:C4R162|C4R162\_KOMPG:C4R5X4|C4R5X4\_KOMPG:C4R1Q3|C4R1Q3\_KOMPG:C4R1H3|C4R1H3\_KOMPG:C4R2D2|C4R2D2\_KOMPG:C4R179|C4R179\_KOMPG:C4QV45|C4QV45\_KOMPG:C4R129|C4R129\_KOMPG:C4QV13|C4QV13\_KOMPG:C4QW68|C4QW68\_KOMPG:C4QXC8|C4QXC8\_KOMPG:C4R4T5|C4R4T5\_KOMPG:C4R047|C4R047\_KOMPG:C4R0Z6|C4R0Z6\_KOMPG:C4QZ54|C4QZ54\_KOMPG:C4QVA1|C4QVA1\_KOMPG:C4QW82|C4QW82\_KOMPG:C4R5V4|C4R5V4\_KOMPG:C4R3U7|C4R3U7\_KOMPG:C4QY10|C4QY10\_KOMPG:C4R855|C4R855\_KOMPG:C4R2F4|C4R2F4\_KOMPG:C4R0K2|C4R0K2\_KOMPG:C4R800|C4R800\_KOMPG |  |  | DB Search |
| WHP | 21.69 | 438.2015 | 3 | 8.65 | 439.2115 | 8.79 | 3333 | 2.52e3 | 1 | 1 | C4QZB2|C4QZB2\_KOMPG:C4R0F9|C4R0F9\_KOMPG:C4R2S7|C4R2S7\_KOMPG:C4R368|C4R368\_KOMPG:Q9P4C8|SAR1\_KOMPG:C4R5C4|C4R5C4\_KOMPG:C4QW55|C4QW55\_KOMPG:C4QXX4|C4QXX4\_KOMPG:C4R422|C4R422\_KOMPG:C4R0U4|C4R0U4\_KOMPG:C4QYH0|C4QYH0\_KOMPG:C4QZY9|C4QZY9\_KOMPG:C4R982|C4R982\_KOMPG:C4R8W8|C4R8W8\_KOMPG:C4R4U0|C4R4U0\_KOMPG:C4R6Z0|C4R6Z0\_KOMPG:C4QXU3|C4QXU3\_KOMPG:C4R0X3|C4R0X3\_KOMPG:C4QZC0|C4QZC0\_KOMPG:C4R254|C4R254\_KOMPG:C4R5E5|C4R5E5\_KOMPG:C4R8X8|C4R8X8\_KOMPG:C4QWS9|C4QWS9\_KOMPG:C4QXC2|C4QXC2\_KOMPG:C4R3U4|C4R3U4\_KOMPG:C4R6R9|C4R6R9\_KOMPG:C4R4B2|C4R4B2\_KOMPG:C4R6Y8|C4R6Y8\_KOMPG:C4R4B7|C4R4B7\_KOMPG |  |  | DB Search |
| AGVT | 21.65 | 346.1852 | 4 | -1.45 | 347.1911 | 2.64 | 1118 | 0 | 0 | 0 | C4R3X2|C4R3X2\_KOMPG:C4R5U0|C4R5U0\_KOMPG:C4R450|C4R450\_KOMPG:C4R793|C4R793\_KOMPG:C4R5G1|C4R5G1\_KOMPG:C4QXR2|C4QXR2\_KOMPG:C4R9D2|C4R9D2\_KOMPG:C4QZ07|C4QZ07\_KOMPG:C4QY57|C4QY57\_KOMPG:C4R156|C4R156\_KOMPG:C4R6M7|C4R6M7\_KOMPG:C4R3T1|C4R3T1\_KOMPG:C4R4Z7|C4R4Z7\_KOMPG:C4R5P6|C4R5P6\_KOMPG:C4R162|C4R162\_KOMPG:C4R5T8|C4R5T8\_KOMPG:C4R0Q9|C4R0Q9\_KOMPG:C4QW55|C4QW55\_KOMPG:C4R0P8|C4R0P8\_KOMPG:C4QVZ3|C4QVZ3\_KOMPG:C4R182|C4R182\_KOMPG:C4QX87|C4QX87\_KOMPG:C4R7W9|C4R7W9\_KOMPG:C4R850|C4R850\_KOMPG:C4R122|C4R122\_KOMPG:C4R4Q2|C4R4Q2\_KOMPG:C4R8M2|C4R8M2\_KOMPG:C4R6W0|C4R6W0\_KOMPG:C4QZB0|C4QZB0\_KOMPG:C4R5H2|C4R5H2\_KOMPG:C4QUZ0|C4QUZ0\_KOMPG:C4R9D7|C4R9D7\_KOMPG:C4QV23|C4QV23\_KOMPG:C4QWR3|C4QWR3\_KOMPG |  |  | DB Search |
| HLN(+0.98)RSPP | 21.65 | 820.4191 | 7 | -7.96 | 821.4178 | 29.43 | 10202 | 2.6e2 | 1 | 1 | C4R123|C4R123\_KOMPG | Deamidation (NQ) | N3:Deamidation (NQ):1000 | DB Search |
| SSIF | 21.63 | 452.2271 | 4 | -3.7 | 453.2316 | 22.49 | 8262 | 1.33e3 | 1 | 1 | C4R1B7|C4R1B7\_KOMPG:C4QVZ8|C4QVZ8\_KOMPG:C4R2V8|C4R2V8\_KOMPG:C4R641|C4R641\_KOMPG:C4QVN9|C4QVN9\_KOMPG:C4QYB2|C4QYB2\_KOMPG:C4R7Z0|C4R7Z0\_KOMPG:C4QX06|C4QX06\_KOMPG:C4QZL0|C4QZL0\_KOMPG:C4R0V3|C4R0V3\_KOMPG:C4R4G8|C4R4G8\_KOMPG:C4R5Q1|C4R5Q1\_KOMPG:C4R669|C4R669\_KOMPG:C4R5C6|C4R5C6\_KOMPG:C4R561|C4R561\_KOMPG:C4QWA3|C4QWA3\_KOMPG:C4R0P7|C4R0P7\_KOMPG:C4R534|C4R534\_KOMPG:C4R5L4|C4R5L4\_KOMPG:C4QW91|C4QW91\_KOMPG:C4R7R3|C4R7R3\_KOMPG:C4QVW1|C4QVW1\_KOMPG:C4QYS5|C4QYS5\_KOMPG:C4QVR4|C4QVR4\_KOMPG:C4QWS1|C4QWS1\_KOMPG:C4R804|C4R804\_KOMPG:C4QVS8|C4QVS8\_KOMPG:C4QYF5|C4QYF5\_KOMPG:C4QXT9|C4QXT9\_KOMPG:C4QZI2|C4QZI2\_KOMPG:C4QWV0|C4QWV0\_KOMPG:C4QYK8|C4QYK8\_KOMPG:C4QX72|C4QX72\_KOMPG:C4R0P1|C4R0P1\_KOMPG:C4R3D9|C4R3D9\_KOMPG:C4R8V8|C4R8V8\_KOMPG:C4QZQ6|C4QZQ6\_KOMPG:C4R893|C4R893\_KOMPG:C4R666|C4R666\_KOMPG:C4R796|C4R796\_KOMPG:C4QY51|C4QY51\_KOMPG:C4QZW5|C4QZW5\_KOMPG:C4R3X4|C4R3X4\_KOMPG:C4QXH0|C4QXH0\_KOMPG:C4R922|C4R922\_KOMPG:C4QZJ4|C4QZJ4\_KOMPG:C4R9E3|C4R9E3\_KOMPG:C4R1J5|C4R1J5\_KOMPG:C4QZR7|C4QZR7\_KOMPG:C4R5C7|C4R5C7\_KOMPG:C4R3E8|C4R3E8\_KOMPG:C4R203|C4R203\_KOMPG:C4QZA9|C4QZA9\_KOMPG:C4R0K1|C4R0K1\_KOMPG:C4R7B0|C4R7B0\_KOMPG:C4R4F4|C4R4F4\_KOMPG:C4R7J3|C4R7J3\_KOMPG:C4R159|ATG28\_KOMPG:C4R1M0|C4R1M0\_KOMPG:C4R220|C4R220\_KOMPG:C4R732|C4R732\_KOMPG:C4R8Q5|C4R8Q5\_KOMPG:C4QZ60|C4QZ60\_KOMPG:C4QX81|C4QX81\_KOMPG:C4QVR3|C4QVR3\_KOMPG:C4R3L2|C4R3L2\_KOMPG:C4R5Y9|C4R5Y9\_KOMPG:C4R5I8|C4R5I8\_KOMPG:C4R2G2|C4R2G2\_KOMPG:C4R5E5|C4R5E5\_KOMPG:C4R6U0|C4R6U0\_KOMPG:C4QVM6|C4QVM6\_KOMPG:C4R2D7|FLO11\_KOMPG:C4R462|C4R462\_KOMPG:C4R176|C4R176\_KOMPG |  |  | DB Search |
| SSLF | 21.63 | 452.2271 | 4 | -3.7 | 453.2316 | 22.49 | 8262 | 1.33e3 | 1 | 1 | C4R8H4|C4R8H4\_KOMPG:C4QZZ7|GEP3\_KOMPG:C4R184|C4R184\_KOMPG:C4R8F2|C4R8F2\_KOMPG:C4QXH4|C4QXH4\_KOMPG:C4R5P3|C4R5P3\_KOMPG:C4QW51|C4QW51\_KOMPG:C4QVJ7|C4QVJ7\_KOMPG:C4R8W8|C4R8W8\_KOMPG:C4QW86|C4QW86\_KOMPG:C4R628|PFF1\_KOMPG:C4QX73|C4QX73\_KOMPG:C4QVE9|C4QVE9\_KOMPG:C4R5J2|C4R5J2\_KOMPG:C4R7S8|C4R7S8\_KOMPG:C4R5X8|C4R5X8\_KOMPG:C4R6P0|C4R6P0\_KOMPG:C4R571|C4R571\_KOMPG:C4QWH8|C4QWH8\_KOMPG:C4R087|C4R087\_KOMPG:C4R052|C4R052\_KOMPG:C4QZX5|C4QZX5\_KOMPG:C4QWZ3|C4QWZ3\_KOMPG:C4R3D7|C4R3D7\_KOMPG:C4R044|C4R044\_KOMPG:C4R1M9|C4R1M9\_KOMPG:C4R0G1|C4R0G1\_KOMPG:C4QWF7|C4QWF7\_KOMPG:C4QZ73|C4QZ73\_KOMPG:C4R714|C4R714\_KOMPG:C4R3J4|C4R3J4\_KOMPG:C4R7V7|C4R7V7\_KOMPG:C4QYC8|C4QYC8\_KOMPG:C4R6N1|C4R6N1\_KOMPG:C4R2D2|C4R2D2\_KOMPG:C4R269|C4R269\_KOMPG:C4QZT7|C4QZT7\_KOMPG:C4QXQ0|C4QXQ0\_KOMPG:C4R480|C4R480\_KOMPG:C4QWD1|C4QWD1\_KOMPG:C4R2V7|C4R2V7\_KOMPG:C4R1E0|C4R1E0\_KOMPG:C4R2Q6|C4R2Q6\_KOMPG:C4R5V0|C4R5V0\_KOMPG:C4QYM3|C4QYM3\_KOMPG:C4R8I9|C4R8I9\_KOMPG:C4R5K8|C4R5K8\_KOMPG:C4QZ44|C4QZ44\_KOMPG:C4QYH7|C4QYH7\_KOMPG:C4R8D0|C4R8D0\_KOMPG:C4QW17|C4QW17\_KOMPG:C4R998|C4R998\_KOMPG:C4QXZ1|C4QXZ1\_KOMPG:C4R3N6|C4R3N6\_KOMPG:C4R258|C4R258\_KOMPG:C4R286|C4R286\_KOMPG:C4QV51|C4QV51\_KOMPG:C4R206|C4R206\_KOMPG:C4R463|C4R463\_KOMPG:C4QWU5|C4QWU5\_KOMPG:C4QXL6|C4QXL6\_KOMPG:C4R944|C4R944\_KOMPG:C4QVD3|C4QVD3\_KOMPG:C4QXA7|C4QXA7\_KOMPG:C4R301|C4R301\_KOMPG:C4QW41|C4QW41\_KOMPG:C4QW67|C4QW67\_KOMPG:C4R1N3|C4R1N3\_KOMPG:C4R225|C4R225\_KOMPG:C4R166|C4R166\_KOMPG:C4QY75|C4QY75\_KOMPG:C4QZ59|C4QZ59\_KOMPG:C4QZY6|C4QZY6\_KOMPG:C4QVE1|C4QVE1\_KOMPG:C4QVU3|SLD1\_KOMPG:C4QV22|C4QV22\_KOMPG:C4QXI8|PEX6\_KOMPG:C4R5Q0|C4R5Q0\_KOMPG:C4R5P8|C4R5P8\_KOMPG:C4R709|C4R709\_KOMPG:C4R4I6|C4R4I6\_KOMPG:C4R7X2|C4R7X2\_KOMPG:C4QWB3|C4QWB3\_KOMPG:C4R703|C4R703\_KOMPG:C4QYX4|C4QYX4\_KOMPG:C4R2Y1|C4R2Y1\_KOMPG:C4QX02|C4QX02\_KOMPG:C4QX00|C4QX00\_KOMPG:C4QYB8|C4QYB8\_KOMPG:C4QX70|C4QX70\_KOMPG:C4R875|C4R875\_KOMPG:C4R3W8|C4R3W8\_KOMPG:C4QX25|C4QX25\_KOMPG:C4R1D3|C4R1D3\_KOMPG:C4R1Y8|C4R1Y8\_KOMPG:C4QZ04|C4QZ04\_KOMPG:C4R4L9|C4R4L9\_KOMPG:C4R6E8|C4R6E8\_KOMPG:C4R800|C4R800\_KOMPG |  |  | DB Search |
| RRSY | 21.61 | 580.3082 | 4 | 4.49 | 291.1619 | 14.00 | 5567 | 9.56e4 | 1 | 1 | C4R7F7|C4R7F7\_KOMPG:C4R2G0|C4R2G0\_KOMPG:C4R8B7|C4R8B7\_KOMPG:C4QV46|C4QV46\_KOMPG:C4QZ24|C4QZ24\_KOMPG:C4R0B3|C4R0B3\_KOMPG:C4R785|C4R785\_KOMPG:C4QYH0|C4QYH0\_KOMPG:C4QXC1|C4QXC1\_KOMPG:C4R4R4|C4R4R4\_KOMPG:C4R8R5|C4R8R5\_KOMPG:C4QZV0|C4QZV0\_KOMPG:C4R587|C4R587\_KOMPG |  |  | DB Search |
| YGR | 21.6 | 394.1965 | 3 | -4.25 | 395.2011 | 5.35 | 1961 | 1.67e4 | 1 | 1 | C4QXJ1|C4QXJ1\_KOMPG:C4R512|C4R512\_KOMPG:C4QWJ4|MDM10\_KOMPG:C4R3Z9|C4R3Z9\_KOMPG:C4QXK0|C4QXK0\_KOMPG:C4R4G6|C4R4G6\_KOMPG:C4QVW3|C4QVW3\_KOMPG:C4QV84|C4QV84\_KOMPG:C4R1R9|C4R1R9\_KOMPG:C4R3B2|C4R3B2\_KOMPG:C4QZI4|C4QZI4\_KOMPG:C4R9B5|C4R9B5\_KOMPG:C4R558|C4R558\_KOMPG:C4R561|C4R561\_KOMPG:C4R0W9|C4R0W9\_KOMPG:C4R0L8|C4R0L8\_KOMPG:C4R7R9|C4R7R9\_KOMPG:C4R6C1|C4R6C1\_KOMPG:C4R4M2|C4R4M2\_KOMPG:C4R6B2|C4R6B2\_KOMPG:C4R2V9|C4R2V9\_KOMPG:C4QVX4|C4QVX4\_KOMPG:P53024|SEC13\_KOMPG:C4R124|C4R124\_KOMPG:C4R753|C4R753\_KOMPG:C4R3K4|C4R3K4\_KOMPG:C4QXM1|C4QXM1\_KOMPG:C4R1C6|C4R1C6\_KOMPG:C4R394|C4R394\_KOMPG:C4R451|C4R451\_KOMPG:C4R0K5|C4R0K5\_KOMPG:C4R0Y0|C4R0Y0\_KOMPG:C4R1Q7|C4R1Q7\_KOMPG:C4QXR7|C4QXR7\_KOMPG:C4R0Y7|C4R0Y7\_KOMPG:C4R5R1|C4R5R1\_KOMPG:C4R7M1|C4R7M1\_KOMPG:C4R8H6|C4R8H6\_KOMPG:C4R9A4|C4R9A4\_KOMPG:C4R3D2|C4R3D2\_KOMPG:C4R953|C4R953\_KOMPG:C4R341|C4R341\_KOMPG:C4QVV6|C4QVV6\_KOMPG:C4R1E4|C4R1E4\_KOMPG:C4R3U0|GET2\_KOMPG:C4R5N9|C4R5N9\_KOMPG:C4R1M9|C4R1M9\_KOMPG:C4R744|C4R744\_KOMPG:C4R2K0|UTP25\_KOMPG:C4QZP7|C4QZP7\_KOMPG:C4R607|C4R607\_KOMPG:C4R7Z6|C4R7Z6\_KOMPG:C4R7A0|C4R7A0\_KOMPG:C4R5J9|C4R5J9\_KOMPG:C4R3K2|C4R3K2\_KOMPG:C4R8G8|C4R8G8\_KOMPG:C4R8U0|C4R8U0\_KOMPG:C4R2X1|C4R2X1\_KOMPG:C4R821|C4R821\_KOMPG:C4R0J7|C4R0J7\_KOMPG:C4QVI7|C4QVI7\_KOMPG:C4R772|C4R772\_KOMPG:C4QVT9|C4QVT9\_KOMPG:C4QXQ0|C4QXQ0\_KOMPG:C4R6I2|C4R6I2\_KOMPG:C4QZ34|C4QZ34\_KOMPG:C4R7M5|C4R7M5\_KOMPG:C4QWY7|C4QWY7\_KOMPG:C4R7V9|C4R7V9\_KOMPG:C4R6U5|C4R6U5\_KOMPG:C4R1D5|C4R1D5\_KOMPG:C4R975|C4R975\_KOMPG:C4QXQ4|C4QXQ4\_KOMPG:C4QXH1|C4QXH1\_KOMPG:C4R092|C4R092\_KOMPG:C4R3J2|C4R3J2\_KOMPG:C4R1H4|C4R1H4\_KOMPG:C4R3H2|C4R3H2\_KOMPG:C4R3K0|C4R3K0\_KOMPG:C4R8P5|C4R8P5\_KOMPG:C4R2X0|C4R2X0\_KOMPG:C4QZQ4|C4QZQ4\_KOMPG:C4R6X0|C4R6X0\_KOMPG:C4R217|C4R217\_KOMPG:C4QXA8|C4QXA8\_KOMPG:C4R2R1|C4R2R1\_KOMPG:C4QVD5|C4QVD5\_KOMPG:C4QXX5|C4QXX5\_KOMPG:C4R1R3|C4R1R3\_KOMPG:C4R0X7|C4R0X7\_KOMPG:C4R1P4|C4R1P4\_KOMPG:C4R4H8|C4R4H8\_KOMPG:C4R914|C4R914\_KOMPG:C4R2L1|C4R2L1\_KOMPG:C4R1Z1|C4R1Z1\_KOMPG:C4R0T4|C4R0T4\_KOMPG:C4QUZ3|C4QUZ3\_KOMPG:C4QY32|C4QY32\_KOMPG:C4QZK7|C4QZK7\_KOMPG:C4QWG0|C4QWG0\_KOMPG:C4R7U9|C4R7U9\_KOMPG:C4QXD1|C4QXD1\_KOMPG:C4R5B8|C4R5B8\_KOMPG:C4R6T8|C4R6T8\_KOMPG:C4QX24|C4QX24\_KOMPG:C4QXK3|C4QXK3\_KOMPG:C4QWS6|C4QWS6\_KOMPG:C4R5H8|C4R5H8\_KOMPG:C4R8L3|C4R8L3\_KOMPG:C4QWQ4|C4QWQ4\_KOMPG:C4R4A0|C4R4A0\_KOMPG:C4R2Z8|C4R2Z8\_KOMPG:C4R1M4|C4R1M4\_KOMPG:C4R0G6|C4R0G6\_KOMPG:C4R338|C4R338\_KOMPG:C4QZQ7|C4QZQ7\_KOMPG:C4R6G7|C4R6G7\_KOMPG:C4QXQ5|C4QXQ5\_KOMPG:C4R3L1|C4R3L1\_KOMPG:C4QYD4|C4QYD4\_KOMPG:C4QW95|C4QW95\_KOMPG:C4R8H2|C4R8H2\_KOMPG:C4R6X1|C4R6X1\_KOMPG:C4QZP8|C4QZP8\_KOMPG:C4R0P0|C4R0P0\_KOMPG:C4R830|C4R830\_KOMPG:C4R602|C4R602\_KOMPG:C4R2Q9|C4R2Q9\_KOMPG:C4R1S9|C4R1S9\_KOMPG:C4QVV1|C4QVV1\_KOMPG:C4R8F6|C4R8F6\_KOMPG:C4QZ48|C4QZ48\_KOMPG:C4R2M8|C4R2M8\_KOMPG:C4QW40|C4QW40\_KOMPG:C4R6S2|C4R6S2\_KOMPG:C4R460|C4R460\_KOMPG:C4QWX2|C4QWX2\_KOMPG:C4R7Y8|C4R7Y8\_KOMPG:C4R2W1|C4R2W1\_KOMPG:C4R4Q9|C4R4Q9\_KOMPG:C4R8S1|C4R8S1\_KOMPG:C4QXS3|C4QXS3\_KOMPG:C4R0S2|C4R0S2\_KOMPG:C4QV05|C4QV05\_KOMPG:C4R8C0|C4R8C0\_KOMPG:C4R1U7|C4R1U7\_KOMPG:C4R370|C4R370\_KOMPG:C4R4P7|C4R4P7\_KOMPG:C4QXF5|C4QXF5\_KOMPG:C4QWC7|C4QWC7\_KOMPG:C4R9A3|C4R9A3\_KOMPG:C4QVB5|C4QVB5\_KOMPG:C4R5G5|C4R5G5\_KOMPG:C4R7S4|C4R7S4\_KOMPG:C4QXF2|C4QXF2\_KOMPG:C4R0F0|C4R0F0\_KOMPG:C4R7L2|C4R7L2\_KOMPG:C4R063|C4R063\_KOMPG:C4R439|C4R439\_KOMPG:C4R1Z8|C4R1Z8\_KOMPG:C4QVH1|C4QVH1\_KOMPG:C4R6Z1|C4R6Z1\_KOMPG:C4QWG7|C4QWG7\_KOMPG:C4R697|C4R697\_KOMPG:C4R1W5|C4R1W5\_KOMPG:C4R6Y8|C4R6Y8\_KOMPG |  |  | DB Search |
| RSTS | 21.58 | 449.2234 | 4 | -1.6 | 450.2289 | 15.62 | 6208 | 1.17e3 | 1 | 1 | C4R5A9|C4R5A9\_KOMPG:C4QZT2|C4QZT2\_KOMPG:C4R0R3|C4R0R3\_KOMPG:C4R7K5|C4R7K5\_KOMPG:C4R6I4|C4R6I4\_KOMPG:C4R8G4|C4R8G4\_KOMPG:C4QV93|C4QV93\_KOMPG:C4R324|C4R324\_KOMPG:C4QWJ5|C4QWJ5\_KOMPG:C4QY67|C4QY67\_KOMPG:C4R6J9|C4R6J9\_KOMPG:C4R1J1|C4R1J1\_KOMPG:C4QWU9|C4QWU9\_KOMPG:C4R5K5|C4R5K5\_KOMPG:C4QXC3|C4QXC3\_KOMPG:C4QZA4|C4QZA4\_KOMPG:C4QWX5|C4QWX5\_KOMPG:C4QWU5|C4QWU5\_KOMPG:C4QY81|C4QY81\_KOMPG:C4QWW9|C4QWW9\_KOMPG:C4R6Z2|C4R6Z2\_KOMPG:C4R2E1|C4R2E1\_KOMPG:C4QWJ7|C4QWJ7\_KOMPG:C4R3Q7|C4R3Q7\_KOMPG:C4QXF9|C4QXF9\_KOMPG:C4R4I8|C4R4I8\_KOMPG:C4QW70|C4QW70\_KOMPG:C4QZ95|C4QZ95\_KOMPG:C4R8M8|C4R8M8\_KOMPG:C4R3W4|C4R3W4\_KOMPG:C4R990|C4R990\_KOMPG:C4R0R2|C4R0R2\_KOMPG:C4R2F3|C4R2F3\_KOMPG:C4QXZ3|C4QXZ3\_KOMPG:C4QZ47|C4QZ47\_KOMPG:C4R0G1|C4R0G1\_KOMPG:C4R3G4|C4R3G4\_KOMPG:C4R5G8|C4R5G8\_KOMPG:C4R0K1|C4R0K1\_KOMPG:C4R027|C4R027\_KOMPG:C4R569|C4R569\_KOMPG:C4R0P8|C4R0P8\_KOMPG:C4R6B5|C4R6B5\_KOMPG:C4QWP2|C4QWP2\_KOMPG:C4R5P2|C4R5P2\_KOMPG:C4QW71|C4QW71\_KOMPG:C4QVH9|C4QVH9\_KOMPG:C4QYB8|C4QYB8\_KOMPG:C4R6B9|C4R6B9\_KOMPG:C4R0P2|C4R0P2\_KOMPG:C4QV96|C4QV96\_KOMPG:C4QZ64|C4QZ64\_KOMPG:C4R7Y9|C4R7Y9\_KOMPG:C4R043|C4R043\_KOMPG:C4R1R8|C4R1R8\_KOMPG:C4R1B6|C4R1B6\_KOMPG:C4R2G2|C4R2G2\_KOMPG:C4QYR4|C4QYR4\_KOMPG:C4R5Z8|C4R5Z8\_KOMPG |  |  | DB Search |
| THH | 21.55 | 393.1761 | 3 | -4.54 | 394.1806 | 2.11 | 908 | 2.12e3 | 1 | 1 | C4R9G5|C4R9G5\_KOMPG:C4R3S9|C4R3S9\_KOMPG:C4QWG2|C4QWG2\_KOMPG:C4R0N9|C4R0N9\_KOMPG:C4R2H1|C4R2H1\_KOMPG:C4QXW8|C4QXW8\_KOMPG:C4QYM7|C4QYM7\_KOMPG:C4QWN0|C4QWN0\_KOMPG:C4R3W7|C4R3W7\_KOMPG:C4R948|C4R948\_KOMPG:C4R1J1|C4R1J1\_KOMPG:C4R0J1|C4R0J1\_KOMPG:C4R9B5|C4R9B5\_KOMPG:C4R4B0|C4R4B0\_KOMPG:C4QXC3|C4QXC3\_KOMPG:C4R3P8|C4R3P8\_KOMPG:C4R7P2|C4R7P2\_KOMPG:C4QY56|C4QY56\_KOMPG:C4R350|C4R350\_KOMPG:C4R0C6|C4R0C6\_KOMPG:C4R9B6|C4R9B6\_KOMPG:C4QWK2|C4QWK2\_KOMPG:C4R7Y6|C4R7Y6\_KOMPG:C4R860|C4R860\_KOMPG:C4QWC6|C4QWC6\_KOMPG:C4R6V2|C4R6V2\_KOMPG:C4R5M2|C4R5M2\_KOMPG:C4R624|C4R624\_KOMPG:C4R5J2|C4R5J2\_KOMPG:C4QXI4|C4QXI4\_KOMPG:C4R7V0|C4R7V0\_KOMPG:C4R4W4|C4R4W4\_KOMPG:Q92448|PFKA1\_KOMPG:C4QZF1|C4QZF1\_KOMPG:C4R6T4|C4R6T4\_KOMPG:C4QW32|C4QW32\_KOMPG:C4R3S8|C4R3S8\_KOMPG:C4QXI3|C4QXI3\_KOMPG:C4QWV1|C4QWV1\_KOMPG:C4R169|C4R169\_KOMPG:C4R5K2|C4R5K2\_KOMPG:C4QW13|C4QW13\_KOMPG:C4QYU1|C4QYU1\_KOMPG:C4QWZ3|C4QWZ3\_KOMPG:C4R6A1|C4R6A1\_KOMPG:C4QVA9|C4QVA9\_KOMPG:C4R6J7|C4R6J7\_KOMPG:C4R4F1|C4R4F1\_KOMPG:C4R0L4|C4R0L4\_KOMPG:C4R505|C4R505\_KOMPG:C4R5Q5|C4R5Q5\_KOMPG:C4R2N1|C4R2N1\_KOMPG:C4QVI1|C4QVI1\_KOMPG:C4R3I9|C4R3I9\_KOMPG:C4QW62|C4QW62\_KOMPG:C4R7J3|C4R7J3\_KOMPG:C4R7D2|C4R7D2\_KOMPG:C4QYT1|C4QYT1\_KOMPG:C4R824|C4R824\_KOMPG:C4QWP2|C4QWP2\_KOMPG:C4R3P9|C4R3P9\_KOMPG:C4R9A7|C4R9A7\_KOMPG:C4QYK2|C4QYK2\_KOMPG:C4R504|C4R504\_KOMPG:C4QW74|C4QW74\_KOMPG:C4QZA7|C4QZA7\_KOMPG:C4R700|C4R700\_KOMPG:C4R254|C4R254\_KOMPG:C4R6U5|C4R6U5\_KOMPG:C4R4J4|C4R4J4\_KOMPG:C4R1B6|C4R1B6\_KOMPG:C4R332|C4R332\_KOMPG:C4QXQ4|C4QXQ4\_KOMPG:C4R3F9|C4R3F9\_KOMPG:C4QWK4|C4QWK4\_KOMPG:C4R6C6|C4R6C6\_KOMPG:C4R3H2|C4R3H2\_KOMPG:C4QXD5|C4QXD5\_KOMPG:C4R3M1|C4R3M1\_KOMPG:C4R322|C4R322\_KOMPG |  |  | DB Search |
| QGIY | 21.52 | 479.238 | 4 | 7.78 | 480.2478 | 12.12 | 4746 | 5.21e3 | 1 | 1 | C4R302|C4R302\_KOMPG:C4QZN8|C4QZN8\_KOMPG:C4R235|C4R235\_KOMPG:C4QYA2|C4QYA2\_KOMPG:C4QVL1|C4QVL1\_KOMPG:C4R3I8|C4R3I8\_KOMPG:C4QYI9|C4QYI9\_KOMPG:C4R5C9|C4R5C9\_KOMPG:C4QXJ3|C4QXJ3\_KOMPG |  |  | DB Search |
| QGLY | 21.52 | 479.238 | 4 | 7.78 | 480.2478 | 12.12 | 4746 | 5.21e3 | 1 | 1 | C4QYS5|C4QYS5\_KOMPG:C4R753|C4R753\_KOMPG:C4R8V7|C4R8V7\_KOMPG:C4R013|C4R013\_KOMPG:C4QWD1|C4QWD1\_KOMPG:C4QVA1|C4QVA1\_KOMPG:C4R2Q1|C4R2Q1\_KOMPG:C4R6M5|C4R6M5\_KOMPG:C4QVX7|C4QVX7\_KOMPG:C4QZZ5|C4QZZ5\_KOMPG:C4R4L9|C4R4L9\_KOMPG:C4R6L1|C4R6L1\_KOMPG:C4R2W0|C4R2W0\_KOMPG |  |  | DB Search |
| AQIP | 21.52 | 427.2431 | 4 | -3.04 | 428.248 | 11.76 | 4668 | 6.31e3 | 1 | 1 | C4R783|C4R783\_KOMPG:C4R6K6|C4R6K6\_KOMPG:C4QW61|C4QW61\_KOMPG:C4R3D7|C4R3D7\_KOMPG:C4R4N0|C4R4N0\_KOMPG:C4QVX1|C4QVX1\_KOMPG:C4R0A7|C4R0A7\_KOMPG:C4R551|C4R551\_KOMPG:C4QVC7|C4QVC7\_KOMPG:C4R8N7|C4R8N7\_KOMPG |  |  | DB Search |
| AQLP | 21.52 | 427.2431 | 4 | -3.04 | 428.248 | 11.76 | 4668 | 6.31e3 | 1 | 1 | C4QY77|C4QY77\_KOMPG:C4R6X0|C4R6X0\_KOMPG:C4QW10|C4QW10\_KOMPG:C4QVW8|C4QVW8\_KOMPG:C4R6U4|C4R6U4\_KOMPG:C4QVZ9|C4QVZ9\_KOMPG:C4R5U1|C4R5U1\_KOMPG:C4R4B9|C4R4B9\_KOMPG:C4R001|C4R001\_KOMPG:C4R879|C4R879\_KOMPG:C4QXY8|C4QXY8\_KOMPG:C4QYE2|C4QYE2\_KOMPG:C4QWU9|C4QWU9\_KOMPG:C4R8I8|C4R8I8\_KOMPG:C4R4M2|C4R4M2\_KOMPG:C4R361|C4R361\_KOMPG:C4QZU0|C4QZU0\_KOMPG:C4R5H4|C4R5H4\_KOMPG:C4R4Z1|C4R4Z1\_KOMPG:C4R483|C4R483\_KOMPG:C4R0B4|C4R0B4\_KOMPG:C4R5V8|C4R5V8\_KOMPG |  |  | DB Search |
| YNP | 21.45 | 392.1696 | 3 | -3.75 | 393.1744 | 8.45 | 3226 | 1.39e2 | 1 | 1 | C4R2H1|C4R2H1\_KOMPG:C4R023|C4R023\_KOMPG:C4QVP4|C4QVP4\_KOMPG:C4QX99|C4QX99\_KOMPG:C4QVA5|C4QVA5\_KOMPG:C4R4X1|C4R4X1\_KOMPG:C4R7D5|C4R7D5\_KOMPG:C4R4Q8|C4R4Q8\_KOMPG:C4QZK1|C4QZK1\_KOMPG:C4R5J4|C4R5J4\_KOMPG:C4QVC2|C4QVC2\_KOMPG:C4R4C6|C4R4C6\_KOMPG:C4R0U1|C4R0U1\_KOMPG:C4R3U2|C4R3U2\_KOMPG:C4R0L6|C4R0L6\_KOMPG:C4R2C5|C4R2C5\_KOMPG:C4R348|C4R348\_KOMPG:C4QYU8|C4QYU8\_KOMPG:C4QVK5|C4QVK5\_KOMPG:C4R1Z5|C4R1Z5\_KOMPG:C4QZL7|C4QZL7\_KOMPG:C4R1A9|C4R1A9\_KOMPG:C4QZ41|C4QZ41\_KOMPG:C4QXZ3|C4QXZ3\_KOMPG:C4R926|C4R926\_KOMPG:C4R6D8|C4R6D8\_KOMPG:C4R5G8|C4R5G8\_KOMPG:C4R7V7|C4R7V7\_KOMPG:C4R3D6|C4R3D6\_KOMPG:C4QX57|C4QX57\_KOMPG:C4QY65|C4QY65\_KOMPG:C4QWQ8|C4QWQ8\_KOMPG:C4QWP7|C4QWP7\_KOMPG:C4R0J7|C4R0J7\_KOMPG:C4R7B9|C4R7B9\_KOMPG:C4QXH5|C4QXH5\_KOMPG:C4R772|C4R772\_KOMPG:C4QVT9|C4QVT9\_KOMPG:C4R6R0|C4R6R0\_KOMPG:C4QXQ0|C4QXQ0\_KOMPG:C4QY09|C4QY09\_KOMPG:C4QWI0|C4QWI0\_KOMPG:C4QZA7|C4QZA7\_KOMPG:C4R1I5|C4R1I5\_KOMPG:C4R5T6|C4R5T6\_KOMPG:C4R4E8|C4R4E8\_KOMPG:C4R0E1|C4R0E1\_KOMPG:C4R009|C4R009\_KOMPG:C4QY38|C4QY38\_KOMPG:C4R9G5|C4R9G5\_KOMPG:C4QWX8|C4QWX8\_KOMPG:C4QZ11|C4QZ11\_KOMPG:C4QVN4|C4QVN4\_KOMPG:C4R554|C4R554\_KOMPG:C4R8T2|C4R8T2\_KOMPG:C4QYB1|C4QYB1\_KOMPG:C4R515|C4R515\_KOMPG:C4QZ80|C4QZ80\_KOMPG:C4QVW5|C4QVW5\_KOMPG:C4QYP0|C4QYP0\_KOMPG:C4R6L7|C4R6L7\_KOMPG:C4R5Y3|C4R5Y3\_KOMPG:C4R956|C4R956\_KOMPG:C4QWG0|C4QWG0\_KOMPG:C4R2R3|C4R2R3\_KOMPG:C4R8R7|C4R8R7\_KOMPG:C4R5B8|C4R5B8\_KOMPG:C4R6X8|C4R6X8\_KOMPG:C4QXC5|C4QXC5\_KOMPG:C4R8L3|C4R8L3\_KOMPG:C4R178|C4R178\_KOMPG:C4R0J4|C4R0J4\_KOMPG:C4QZP1|C4QZP1\_KOMPG:C4R9A1|C4R9A1\_KOMPG:C4R655|C4R655\_KOMPG:C4R6G7|C4R6G7\_KOMPG:C4R3L1|C4R3L1\_KOMPG:C4QXC2|C4QXC2\_KOMPG:C4QXP3|C4QXP3\_KOMPG:C4R8D7|ATG37\_KOMPG:C4R5Y4|C4R5Y4\_KOMPG:C4QZA5|C4QZA5\_KOMPG:C4R827|C4R827\_KOMPG:C4R287|C4R287\_KOMPG:C4R1E2|C4R1E2\_KOMPG:C4R7W8|C4R7W8\_KOMPG:C4R441|C4R441\_KOMPG:C4R8Z9|C4R8Z9\_KOMPG:C4R8I2|C4R8I2\_KOMPG:C4R299|C4R299\_KOMPG:C4QXX4|C4QXX4\_KOMPG:C4R179|C4R179\_KOMPG:C4R8I0|C4R8I0\_KOMPG:C4QYQ9|C4QYQ9\_KOMPG:C4R1P9|C4R1P9\_KOMPG:C4QW97|C4QW97\_KOMPG:C4QWC1|C4QWC1\_KOMPG:C4QZB7|C4QZB7\_KOMPG:C4QVP2|C4QVP2\_KOMPG:C4R024|C4R024\_KOMPG:C4QYX7|C4QYX7\_KOMPG:C4R615|C4R615\_KOMPG:C4QY10|C4QY10\_KOMPG:C4R6A3|C4R6A3\_KOMPG:C4R6X2|C4R6X2\_KOMPG:C4QXN9|C4QXN9\_KOMPG:C4QWQ5|C4QWQ5\_KOMPG:C4QW04|LCL3\_KOMPG:C4R6I4|C4R6I4\_KOMPG:C4QVT8|C4QVT8\_KOMPG:C4QYB2|C4QYB2\_KOMPG:C4QXP6|C4QXP6\_KOMPG:C4QWE5|C4QWE5\_KOMPG:C4QY74|C4QY74\_KOMPG:C4QV84|C4QV84\_KOMPG:C4R1G7|C4R1G7\_KOMPG:C4R0D8|C4R0D8\_KOMPG:C4R658|C4R658\_KOMPG:C4R5K5|C4R5K5\_KOMPG:C4R6R2|C4R6R2\_KOMPG:C4R784|C4R784\_KOMPG:C4R1D6|C4R1D6\_KOMPG:C4R108|C4R108\_KOMPG:C4R058|C4R058\_KOMPG:C4QVX0|C4QVX0\_KOMPG:C4R742|C4R742\_KOMPG:C4QXU8|C4QXU8\_KOMPG:C4R1C3|C4R1C3\_KOMPG:C4QZB9|C4QZB9\_KOMPG:C4R7Q5|EXO5\_KOMPG:C4R4S1|C4R4S1\_KOMPG:C4R0Y0|C4R0Y0\_KOMPG:C4R5A8|C4R5A8\_KOMPG:C4R6E1|C4R6E1\_KOMPG:C4QZF1|C4QZF1\_KOMPG:C4QVJ1|C4QVJ1\_KOMPG:C4QVY8|C4QVY8\_KOMPG:C4QZB0|C4QZB0\_KOMPG:C4QZ95|C4QZ95\_KOMPG:C4QZW5|C4QZW5\_KOMPG:C4QX78|C4QX78\_KOMPG:C4R6N6|C4R6N6\_KOMPG:C4R1K8|ERT1\_KOMPG:C4R017|C4R017\_KOMPG:C4QV66|C4QV66\_KOMPG:C4R3G0|C4R3G0\_KOMPG:C4QWP1|C4QWP1\_KOMPG:C4R0A2|C4R0A2\_KOMPG:C4R1U8|C4R1U8\_KOMPG:C4R572|C4R572\_KOMPG:C4R9E4|C4R9E4\_KOMPG:C4R6G5|C4R6G5\_KOMPG:C4QWI6|C4QWI6\_KOMPG:C4QYX6|C4QYX6\_KOMPG:C4R7C8|C4R7C8\_KOMPG:C4QYT8|C4QYT8\_KOMPG:C4QZW1|C4QZW1\_KOMPG:C4R6E7|C4R6E7\_KOMPG:C4QZQ4|C4QZQ4\_KOMPG:C4R324|C4R324\_KOMPG:C4R522|C4R522\_KOMPG:C4R0H0|C4R0H0\_KOMPG:C4QVU5|C4QVU5\_KOMPG:C4R2E3|C4R2E3\_KOMPG:C4QY56|C4QY56\_KOMPG:C4R040|C4R040\_KOMPG:C4QVW1|C4QVW1\_KOMPG:C4R3I3|C4R3I3\_KOMPG:C4R5Z7|C4R5Z7\_KOMPG:C4R5X5|C4R5X5\_KOMPG:C4R6D9|C4R6D9\_KOMPG:C4R548|C4R548\_KOMPG:C4R3S3|C4R3S3\_KOMPG:C4QW67|C4QW67\_KOMPG:C4QXQ5|C4QXQ5\_KOMPG:C4R229|C4R229\_KOMPG:C4QVT3|C4QVT3\_KOMPG:C4R877|C4R877\_KOMPG:C4R8M6|C4R8M6\_KOMPG:C4R2Q9|C4R2Q9\_KOMPG:C4R070|C4R070\_KOMPG:C4R776|C4R776\_KOMPG:C4R173|C4R173\_KOMPG:C4QZ48|C4QZ48\_KOMPG:C4QV87|C4QV87\_KOMPG:C4R3L3|C4R3L3\_KOMPG:C4R0C4|C4R0C4\_KOMPG:C4QWF2|C4QWF2\_KOMPG:C4R3S5|C4R3S5\_KOMPG:C4R1F2|C4R1F2\_KOMPG:C4R703|C4R703\_KOMPG:C4R723|C4R723\_KOMPG:C4R5L7|C4R5L7\_KOMPG:C4QZD0|C4QZD0\_KOMPG:C4R1W2|C4R1W2\_KOMPG:C4R0S6|C4R0S6\_KOMPG:C4R673|C4R673\_KOMPG:C4QW06|C4QW06\_KOMPG:C4QY02|C4QY02\_KOMPG:C4QV91|C4QV91\_KOMPG:C4R677|C4R677\_KOMPG:C4QXX3|C4QXX3\_KOMPG:C4R4K4|C4R4K4\_KOMPG:C4QYJ3|C4QYJ3\_KOMPG |  |  | DB Search |
| NTS | 21.44 | 320.1332 | 3 | 8.58 | 321.1424 | 6.16 | 2421 | 0 | 0 | 0 | C4R529|C4R529\_KOMPG:C4R368|C4R368\_KOMPG:C4R6C5|C4R6C5\_KOMPG:C4R6M3|C4R6M3\_KOMPG:C4R8B9|C4R8B9\_KOMPG:C4R110|C4R110\_KOMPG:C4QVE8|C4QVE8\_KOMPG:C4QYX9|C4QYX9\_KOMPG:C4R7Y5|C4R7Y5\_KOMPG:C4R3V0|C4R3V0\_KOMPG:C4QWX5|C4QWX5\_KOMPG:C4QY81|C4QY81\_KOMPG:C4R2J4|C4R2J4\_KOMPG:C4R4X8|C4R4X8\_KOMPG:C4R3U2|C4R3U2\_KOMPG:C4R2C5|C4R2C5\_KOMPG:C4QX28|C4QX28\_KOMPG:C4QWB9|C4QWB9\_KOMPG:C4R6X6|C4R6X6\_KOMPG:C4QV46|C4QV46\_KOMPG:C4R7L6|C4R7L6\_KOMPG:C4QWH2|C4QWH2\_KOMPG:C4QY11|C4QY11\_KOMPG:C4R8Q7|C4R8Q7\_KOMPG:C4R2F8|C4R2F8\_KOMPG:C4R1A9|C4R1A9\_KOMPG:C4QW45|C4QW45\_KOMPG:C4R681|C4R681\_KOMPG:C4QWR3|C4QWR3\_KOMPG:C4R1U0|C4R1U0\_KOMPG:C4R684|C4R684\_KOMPG:C4R5F3|C4R5F3\_KOMPG:C4QVX9|C4QVX9\_KOMPG:C4R118|C4R118\_KOMPG:C4QVN1|C4QVN1\_KOMPG:C4QYA8|C4QYA8\_KOMPG:C4QZA3|C4QZA3\_KOMPG:C4R8G6|C4R8G6\_KOMPG:C4QX32|C4QX32\_KOMPG:C4R8Y6|C4R8Y6\_KOMPG:C4QYP7|C4QYP7\_KOMPG:C4R340|C4R340\_KOMPG:C4QY65|C4QY65\_KOMPG:C4QWQ8|C4QWQ8\_KOMPG:C4R288|C4R288\_KOMPG:C4QYK2|C4QYK2\_KOMPG:C4QWD1|C4QWD1\_KOMPG:C4R3L2|C4R3L2\_KOMPG:C4R5I8|C4R5I8\_KOMPG:C4R3A8|C4R3A8\_KOMPG:C4R795|C4R795\_KOMPG:C4R095|C4R095\_KOMPG:C4QYA3|C4QYA3\_KOMPG:C4R0L5|C4R0L5\_KOMPG:C4R503|C4R503\_KOMPG:C4R3T3|C4R3T3\_KOMPG:C4R322|C4R322\_KOMPG:C4R417|C4R417\_KOMPG:C4QZV4|C4QZV4\_KOMPG:C4R895|C4R895\_KOMPG:C4R6G4|C4R6G4\_KOMPG:C4R8N7|C4R8N7\_KOMPG:C4R7A8|C4R7A8\_KOMPG:C4R8R4|C4R8R4\_KOMPG:C4R454|C4R454\_KOMPG:C4QWS8|PEX36\_KOMPG:C4R5Y3|C4R5Y3\_KOMPG:C4QV07|C4QV07\_KOMPG:C4QXB6|C4QXB6\_KOMPG:C4R5R5|C4R5R5\_KOMPG:C4QZU4|C4QZU4\_KOMPG:C4QXL0|C4QXL0\_KOMPG:C4R675|C4R675\_KOMPG:C4QZ23|C4QZ23\_KOMPG:C4QVB8|C4QVB8\_KOMPG:C4QXB1|C4QXB1\_KOMPG:C4R0M3|C4R0M3\_KOMPG:C4R3C0|C4R3C0\_KOMPG:C4QZ69|C4QZ69\_KOMPG:C4R0A9|C4R0A9\_KOMPG:C4QVD4|C4QVD4\_KOMPG:C4QV72|C4QV72\_KOMPG:C4R276|C4R276\_KOMPG:C4R7T4|C4R7T4\_KOMPG:C4R3W8|C4R3W8\_KOMPG:C4QXM7|C4QXM7\_KOMPG:C4R8H5|C4R8H5\_KOMPG:C4R7L2|C4R7L2\_KOMPG:C4R5V4|C4R5V4\_KOMPG:C4R7R5|C4R7R5\_KOMPG:C4R2W0|C4R2W0\_KOMPG:C4R176|C4R176\_KOMPG:C4R5A9|C4R5A9\_KOMPG:C4QXJ1|C4QXJ1\_KOMPG:C4R278|C4R278\_KOMPG:C4R8L6|C4R8L6\_KOMPG:C4QVT8|C4QVT8\_KOMPG:C4R1D2|C4R1D2\_KOMPG:C4R214|C4R214\_KOMPG:C4R0F2|C4R0F2\_KOMPG:C4R0T1|C4R0T1\_KOMPG:C4QVX4|C4QVX4\_KOMPG:C4R0X2|C4R0X2\_KOMPG:C4QZX7|C4QZX7\_KOMPG:C4R753|C4R753\_KOMPG:C4QXM1|C4QXM1\_KOMPG:C4R6G3|C4R6G3\_KOMPG:C4R7Y0|C4R7Y0\_KOMPG:C4R8Y0|C4R8Y0\_KOMPG:C4R4J8|C4R4J8\_KOMPG:C4R2E4|C4R2E4\_KOMPG:C4R4L4|C4R4L4\_KOMPG:C4QZQ6|C4QZQ6\_KOMPG:C4QW69|C4QW69\_KOMPG:C4R0E2|C4R0E2\_KOMPG:C4R4Q3|C4R4Q3\_KOMPG:C4QX17|C4QX17\_KOMPG:C4R0L0|C4R0L0\_KOMPG:C4R2J1|C4R2J1\_KOMPG:C4R341|C4R341\_KOMPG:C4QZ65|C4QZ65\_KOMPG:C4QZW2|C4QZW2\_KOMPG:C4R4Y5|C4R4Y5\_KOMPG:C4QZP7|C4QZP7\_KOMPG:C4R5C4|C4R5C4\_KOMPG:C4QYE2|C4QYE2\_KOMPG:C4R438|C4R438\_KOMPG:C4R1K1|C4R1K1\_KOMPG:C4R517|C4R517\_KOMPG:C4R2D2|C4R2D2\_KOMPG:C4R7I3|C4R7I3\_KOMPG:C4R951|C4R951\_KOMPG:C4R5K3|C4R5K3\_KOMPG:C4R527|C4R527\_KOMPG:C4R7F0|C4R7F0\_KOMPG:C4R8G9|C4R8G9\_KOMPG:C4QYU7|C4QYU7\_KOMPG:C4R7N1|C4R7N1\_KOMPG:C4R504|C4R504\_KOMPG:C4R243|C4R243\_KOMPG:C4R7R8|C4R7R8\_KOMPG:C4QYC4|C4QYC4\_KOMPG:C4R2C0|C4R2C0\_KOMPG:C4R4B2|C4R4B2\_KOMPG:C4R8I9|C4R8I9\_KOMPG:C4QV23|C4QV23\_KOMPG:C4QZQ4|C4QZQ4\_KOMPG:C4R581|C4R581\_KOMPG:C4QVD5|C4QVD5\_KOMPG:C4QWA3|C4QWA3\_KOMPG:C4R1A8|C4R1A8\_KOMPG:C4R534|C4R534\_KOMPG:C4R5Z4|C4R5Z4\_KOMPG:C4R8P7|C4R8P7\_KOMPG:C4QZK7|C4QZK7\_KOMPG:C4QY12|C4QY12\_KOMPG:C4R034|C4R034\_KOMPG:C4QXU3|C4QXU3\_KOMPG:C4QXF9|C4QXF9\_KOMPG:C4R1H0|C4R1H0\_KOMPG:C4QXA7|C4QXA7\_KOMPG:C4R7G6|C4R7G6\_KOMPG:C4QWR8|C4QWR8\_KOMPG:C4R7X7|C4R7X7\_KOMPG:C4QW32|C4QW32\_KOMPG:C4QX95|C4QX95\_KOMPG:C4QVT3|C4QVT3\_KOMPG:C4R6S3|C4R6S3\_KOMPG:C4QZP8|C4QZP8\_KOMPG:C4R916|C4R916\_KOMPG:C4R3K1|C4R3K1\_KOMPG:C4R032|C4R032\_KOMPG:C4QWU3|C4QWU3\_KOMPG:C4R4S3|C4R4S3\_KOMPG:C4QY13|C4QY13\_KOMPG:C4R8H7|C4R8H7\_KOMPG:C4QWH1|C4QWH1\_KOMPG:C4R2V6|C4R2V6\_KOMPG:C4QXS3|C4QXS3\_KOMPG:C4R8G2|C4R8G2\_KOMPG:C4R5F7|C4R5F7\_KOMPG:C4R5P2|C4R5P2\_KOMPG:C4R0Q0|C4R0Q0\_KOMPG:C4QVK6|C4QVK6\_KOMPG:C4QX02|C4QX02\_KOMPG:C4QYY8|C4QYY8\_KOMPG:C4QX81|C4QX81\_KOMPG:C4QVP3|C4QVP3\_KOMPG:C4R3I0|C4R3I0\_KOMPG:C4R0N8|C4R0N8\_KOMPG:C4R167|C4R167\_KOMPG:C4QXC8|C4QXC8\_KOMPG:C4R1R8|C4R1R8\_KOMPG:C4R6G2|C4R6G2\_KOMPG:C4QW16|C4QW16\_KOMPG:C4QVS5|C4QVS5\_KOMPG:C4QXS6|C4QXS6\_KOMPG:C4R721|C4R721\_KOMPG |  |  | DB Search |
| TSG | 21.44 | 263.1117 | 3 | 2.31 | 264.119 | 3.71 | 1537 | 5.72e3 | 1 | 1 | C4R9D6|C4R9D6\_KOMPG:C4R3J0|C4R3J0\_KOMPG:C4R3H3|C4R3H3\_KOMPG:C4R368|C4R368\_KOMPG:C4R5T1|ATG30\_KOMPG:C4R7Q3|C4R7Q3\_KOMPG:C4R8D1|C4R8D1\_KOMPG:C4QWW6|C4QWW6\_KOMPG:C4R5F6|C4R5F6\_KOMPG:C4R1X4|C4R1X4\_KOMPG:C4R1D0|C4R1D0\_KOMPG:C4R8B9|C4R8B9\_KOMPG:C4R2A4|C4R2A4\_KOMPG:C4R116|C4R116\_KOMPG:C4R5J4|C4R5J4\_KOMPG:C4R8R8|C4R8R8\_KOMPG:C4R4D9|C4R4D9\_KOMPG:C4R7Y5|C4R7Y5\_KOMPG:C4R2J4|C4R2J4\_KOMPG:C4QZ71|C4QZ71\_KOMPG:C4R5Q7|C4R5Q7\_KOMPG:C4R7L6|C4R7L6\_KOMPG:C4R6H0|C4R6H0\_KOMPG:C4R8D3|C4R8D3\_KOMPG:C4QY58|C4QY58\_KOMPG:C4R5N1|C4R5N1\_KOMPG:C4QVX7|C4QVX7\_KOMPG:C4QW45|C4QW45\_KOMPG:C4QVB4|C4QVB4\_KOMPG:C4R6A7|C4R6A7\_KOMPG:C4R1U0|C4R1U0\_KOMPG:C4R5K2|C4R5K2\_KOMPG:C4R044|C4R044\_KOMPG:C4QY85|C4QY85\_KOMPG:C4R9E3|C4R9E3\_KOMPG:C4R3L0|C4R3L0\_KOMPG:C4R310|C4R310\_KOMPG:C4R8X5|C4R8X5\_KOMPG:C4R5S1|C4R5S1\_KOMPG:C4QYA8|C4QYA8\_KOMPG:C4QZ88|C4QZ88\_KOMPG:C4QV54|C4QV54\_KOMPG:C4R481|C4R481\_KOMPG:C4QVL5|C4QVL5\_KOMPG:C4R7F1|C4R7F1\_KOMPG:C4QXB8|C4QXB8\_KOMPG:C4R6B5|C4R6B5\_KOMPG:C4R434|C4R434\_KOMPG:C4QZF9|C4QZF9\_KOMPG:C4R950|C4R950\_KOMPG:C4R8Y8|C4R8Y8\_KOMPG:C4R692|C4R692\_KOMPG:C4R275|C4R275\_KOMPG:C4QWD1|C4QWD1\_KOMPG:C4QXE2|C4QXE2\_KOMPG:C4R1E0|C4R1E0\_KOMPG:C4R4L3|C4R4L3\_KOMPG:C4R0L5|C4R0L5\_KOMPG:C4R1I1|C4R1I1\_KOMPG:C4QVR5|C4QVR5\_KOMPG:C4QY08|C4QY08\_KOMPG:C4R5N4|C4R5N4\_KOMPG:C4QV86|C4QV86\_KOMPG:Q9Y751|ATG26\_KOMPG:C4QXX5|C4QXX5\_KOMPG:C4R3I7|C4R3I7\_KOMPG:C4QWP6|C4QWP6\_KOMPG:C4QYP0|C4QYP0\_KOMPG:C4R454|C4R454\_KOMPG:C4R061|C4R061\_KOMPG:C4QYC6|C4QYC6\_KOMPG:C4R1X3|C4R1X3\_KOMPG:C4R8B7|C4R8B7\_KOMPG:C4QX63|C4QX63\_KOMPG:C4QY99|C4QY99\_KOMPG:C4R6U9|C4R6U9\_KOMPG:C4R3E4|C4R3E4\_KOMPG:C4R2U9|C4R2U9\_KOMPG:C4R618|C4R618\_KOMPG:C4QZU4|C4QZU4\_KOMPG:C4R9D7|C4R9D7\_KOMPG:C4QZY8|C4QZY8\_KOMPG:C4R5A7|C4R5A7\_KOMPG:C4QX29|C4QX29\_KOMPG:C4R760|C4R760\_KOMPG:C4QXL0|C4QXL0\_KOMPG:C4R6P4|C4R6P4\_KOMPG:C4R1S9|C4R1S9\_KOMPG:C4R0M2|C4R0M2\_KOMPG:C4QZ23|C4QZ23\_KOMPG:C4R148|C4R148\_KOMPG:C4QXI5|C4QXI5\_KOMPG:C4QZE1|C4QZE1\_KOMPG:C4R6H9|C4R6H9\_KOMPG:C4QWX6|C4QWX6\_KOMPG:C4R5T3|C4R5T3\_KOMPG:C4QZW9|C4QZW9\_KOMPG:C4QXX4|C4QXX4\_KOMPG:C4R1N6|C4R1N6\_KOMPG:C4R134|C4R134\_KOMPG:C4QYQ9|C4QYQ9\_KOMPG:C4R7M6|C4R7M6\_KOMPG:C4QZQ2|C4QZQ2\_KOMPG:C4R102|C4R102\_KOMPG:C4R931|C4R931\_KOMPG:C4R2I6|C4R2I6\_KOMPG:C4QXJ7|C4QXJ7\_KOMPG:C4R7B3|C4R7B3\_KOMPG:C4QYX7|C4QYX7\_KOMPG:C4QVA1|C4QVA1\_KOMPG:C4R565|C4R565\_KOMPG:C4QY10|C4QY10\_KOMPG:C4R5H2|C4R5H2\_KOMPG:C4R813|C4R813\_KOMPG:C4QVM6|C4QVM6\_KOMPG:C4R5A9|C4R5A9\_KOMPG:C4R8L6|C4R8L6\_KOMPG:C4QVT8|C4QVT8\_KOMPG:C4R1D2|C4R1D2\_KOMPG:C4R6A4|C4R6A4\_KOMPG:C4R396|C4R396\_KOMPG:C4R4U2|C4R4U2\_KOMPG:C4R6C2|PEX1\_KOMPG:C4QY05|C4QY05\_KOMPG:C4R8D4|C4R8D4\_KOMPG:C4R812|C4R812\_KOMPG:C4QWD4|C4QWD4\_KOMPG:C4R753|C4R753\_KOMPG:C4R8A4|C4R8A4\_KOMPG:C4R0Y0|C4R0Y0\_KOMPG:C4R1Q7|C4R1Q7\_KOMPG:C4QVZ1|C4QVZ1\_KOMPG:C4QWM2|C4QWM2\_KOMPG:C4R6E1|C4R6E1\_KOMPG:C4R3Q7|C4R3Q7\_KOMPG:C4QXN2|MIC60\_KOMPG:C4R8I1|C4R8I1\_KOMPG:C4R3S8|C4R3S8\_KOMPG:C4R012|C4R012\_KOMPG:C4R169|C4R169\_KOMPG:C4R2J1|C4R2J1\_KOMPG:C4R1T1|C4R1T1\_KOMPG:C4QX83|C4QX83\_KOMPG:C4R3F4|C4R3F4\_KOMPG:C4R808|C4R808\_KOMPG:C4R517|C4R517\_KOMPG:C4QZV3|C4QZV3\_KOMPG:C4R7I3|C4R7I3\_KOMPG:C4R4F5|C4R4F5\_KOMPG:C4R6G5|C4R6G5\_KOMPG:C4R7F0|C4R7F0\_KOMPG:C4R241|C4R241\_KOMPG:C4R8G9|C4R8G9\_KOMPG:C4QZB5|C4QZB5\_KOMPG:C4R664|C4R664\_KOMPG:C4R243|C4R243\_KOMPG:C4R378|C4R378\_KOMPG:C4R254|C4R254\_KOMPG:C4QZB4|C4QZB4\_KOMPG:C4R3U7|C4R3U7\_KOMPG:C4QZ77|C4QZ77\_KOMPG:C4R2F4|C4R2F4\_KOMPG:C4R2C0|C4R2C0\_KOMPG:C4R4X6|C4R4X6\_KOMPG:C4R8I9|C4R8I9\_KOMPG:C4R3A1|C4R3A1\_KOMPG:C4R5C0|C4R5C0\_KOMPG:C4R594|C4R594\_KOMPG:C4QVB9|C4QVB9\_KOMPG:C4R1Y2|C4R1Y2\_KOMPG:C4R1V4|C4R1V4\_KOMPG:C4R1J4|C4R1J4\_KOMPG:C4R846|C4R846\_KOMPG:C4QX76|C4QX76\_KOMPG:C4QV21|C4QV21\_KOMPG:C4R6V5|C4R6V5\_KOMPG:C4R5X5|C4R5X5\_KOMPG:C4QXU3|C4QXU3\_KOMPG:C4QZE9|C4QZE9\_KOMPG:C4R1Y4|C4R1Y4\_KOMPG:C4QXU5|C4QXU5\_KOMPG:C4QY80|C4QY80\_KOMPG:C4QVT0|C4QVT0\_KOMPG:C4R057|C4R057\_KOMPG:C4R111|C4R111\_KOMPG:C4QVT3|C4QVT3\_KOMPG:C4QVU6|C4QVU6\_KOMPG:C4R1Q0|C4R1Q0\_KOMPG:C4R8S4|C4R8S4\_KOMPG:C4R070|C4R070\_KOMPG:C4R032|C4R032\_KOMPG:C4QZ18|C4QZ18\_KOMPG:C4R8H1|C4R8H1\_KOMPG:C4R1L4|C4R1L4\_KOMPG:C4R0X5|C4R0X5\_KOMPG:C4QY13|C4QY13\_KOMPG:C4QYQ2|C4QYQ2\_KOMPG:C4QYJ7|C4QYJ7\_KOMPG:C4QWK8|C4QWK8\_KOMPG:C4R984|C4R984\_KOMPG:C4QX02|C4QX02\_KOMPG:C4QXA5|PFKA2\_KOMPG:C4R875|C4R875\_KOMPG:C4QWC7|C4QWC7\_KOMPG:C4QZF2|C4QZF2\_KOMPG:C4QZC0|C4QZC0\_KOMPG:C4R2Z1|C4R2Z1\_KOMPG:C4QW16|C4QW16\_KOMPG:C4QXE9|CHO2\_KOMPG:C4R3B6|C4R3B6\_KOMPG |  |  | DB Search |
| EIW | 21.42 | 446.2165 | 3 | -5.13 | 447.2204 | 31.30 | 10711 | 1.59e3 | 1 | 1 | C4R278|C4R278\_KOMPG:C4QV81|C4QV81\_KOMPG:C4R5I4|C4R5I4\_KOMPG:C4R083|C4R083\_KOMPG:C4QZZ8|C4QZZ8\_KOMPG:C4QX59|C4QX59\_KOMPG:C4QZZ6|C4QZZ6\_KOMPG:C4R1Y2|C4R1Y2\_KOMPG:C4R8R4|C4R8R4\_KOMPG:C4R6C1|C4R6C1\_KOMPG:C4R258|C4R258\_KOMPG:C4R422|C4R422\_KOMPG:C4R812|C4R812\_KOMPG:C4QVQ7|C4QVQ7\_KOMPG:C4QV07|C4QV07\_KOMPG:C4QWU6|C4QWU6\_KOMPG:C4QV42|C4QV42\_KOMPG:C4R3S4|C4R3S4\_KOMPG:C4QYY0|C4QYY0\_KOMPG:C4QYT3|C4QYT3\_KOMPG:C4R6K5|C4R6K5\_KOMPG:C4R5A8|C4R5A8\_KOMPG:C4R3H7|C4R3H7\_KOMPG:C4R7J5|C4R7J5\_KOMPG:C4QXU5|C4QXU5\_KOMPG:C4R571|C4R571\_KOMPG:Q92448|PFKA1\_KOMPG:C4QX12|C4QX12\_KOMPG:C4R0S4|C4R0S4\_KOMPG:C4QZU4|C4QZU4\_KOMPG:C4QZP8|C4QZP8\_KOMPG:C4R7X8|BMT2\_KOMPG:C4R0Z3|C4R0Z3\_KOMPG:C4QZY6|C4QZY6\_KOMPG:C4QZ13|C4QZ13\_KOMPG:C4QWD9|C4QWD9\_KOMPG:C4QV54|C4QV54\_KOMPG:C4R1N0|C4R1N0\_KOMPG:C4QZ88|C4QZ88\_KOMPG:C4R8V6|C4R8V6\_KOMPG:C4R517|C4R517\_KOMPG:C4R3R5|C4R3R5\_KOMPG:C4R2V6|C4R2V6\_KOMPG:C4QYT1|C4QYT1\_KOMPG:C4R6G5|C4R6G5\_KOMPG:C4QX36|C4QX36\_KOMPG:C4R516|C4R516\_KOMPG:C4QXL9|C4QXL9\_KOMPG:C4R122|C4R122\_KOMPG:C4R1H7|C4R1H7\_KOMPG:C4R0S0|C4R0S0\_KOMPG:C4R056|C4R056\_KOMPG:C4QVV5|C4QVV5\_KOMPG:C4R470|C4R470\_KOMPG:C4QUZ0|C4QUZ0\_KOMPG:C4QW15|C4QW15\_KOMPG:C4QVR5|C4QVR5\_KOMPG:C4QZ06|BMT3\_KOMPG:C4R8B3|C4R8B3\_KOMPG:C4R4M5|C4R4M5\_KOMPG:C4R0P9|C4R0P9\_KOMPG |  |  | DB Search |
| ELW | 21.42 | 446.2165 | 3 | -5.13 | 447.2204 | 31.30 | 10711 | 1.59e3 | 1 | 1 | C4R6R3|C4R6R3\_KOMPG:C4QWJ4|MDM10\_KOMPG:C4QZJ3|C4QZJ3\_KOMPG:C4R006|C4R006\_KOMPG:C4R3C6|C4R3C6\_KOMPG:C4R1U1|C4R1U1\_KOMPG:C4R4G6|C4R4G6\_KOMPG:C4QXQ1|C4QXQ1\_KOMPG:C4R564|C4R564\_KOMPG:C4QVZ4|C4QVZ4\_KOMPG:C4QVH6|C4QVH6\_KOMPG:C4R897|C4R897\_KOMPG:C4R9B5|C4R9B5\_KOMPG:C4R4B0|C4R4B0\_KOMPG:C4R888|C4R888\_KOMPG:C4R5Z4|C4R5Z4\_KOMPG:C4R650|C4R650\_KOMPG:C4QWK2|C4QWK2\_KOMPG:C4R4I4|C4R4I4\_KOMPG:C4QY73|C4QY73\_KOMPG:C4R768|C4R768\_KOMPG:C4QY29|C4QY29\_KOMPG:C4R7E8|C4R7E8\_KOMPG:C4R5X5|C4R5X5\_KOMPG:C4QXU3|C4QXU3\_KOMPG:C4R8D2|C4R8D2\_KOMPG:C4R6Z2|C4R6Z2\_KOMPG:C4R069|C4R069\_KOMPG:C4R548|C4R548\_KOMPG:C4R8V2|C4R8V2\_KOMPG:C4R2Z8|C4R2Z8\_KOMPG:C4QWT7|C4QWT7\_KOMPG:C4R2W4|C4R2W4\_KOMPG:C4R6U9|C4R6U9\_KOMPG:C4R8G1|C4R8G1\_KOMPG:C4R666|C4R666\_KOMPG:C4QW21|C4QW21\_KOMPG:C4R0B4|C4R0B4\_KOMPG:C4QVH4|C4QVH4\_KOMPG:C4QYD4|C4QYD4\_KOMPG:C4QWP0|C4QWP0\_KOMPG:C4R513|C4R513\_KOMPG:C4R2S4|C4R2S4\_KOMPG:C4QVT2|C4QVT2\_KOMPG:C4R5W5|C4R5W5\_KOMPG:C4R1L0|C4R1L0\_KOMPG:C4R916|C4R916\_KOMPG:C4R7N8|C4R7N8\_KOMPG:C4QVY2|C4QVY2\_KOMPG:C4R0M3|C4R0M3\_KOMPG:C4QZ07|C4QZ07\_KOMPG:C4QYH5|C4QYH5\_KOMPG:C4QZ69|C4QZ69\_KOMPG:C4QWT6|C4QWT6\_KOMPG:C4R794|C4R794\_KOMPG:C4R0A9|C4R0A9\_KOMPG:C4R7J3|C4R7J3\_KOMPG:C4QY13|C4QY13\_KOMPG:C4R803|C4R803\_KOMPG:C4R6D1|C4R6D1\_KOMPG:C4R265|C4R265\_KOMPG:C4R6Q6|C4R6Q6\_KOMPG:C4R0A2|C4R0A2\_KOMPG:C4R0Y8|C4R0Y8\_KOMPG:C4R0Q0|C4R0Q0\_KOMPG:C4R2C6|C4R2C6\_KOMPG:C4R492|OCA5\_KOMPG:C4QYP1|C4QYP1\_KOMPG:C4QVR9|IRC19\_KOMPG:C4R4M0|C4R4M0\_KOMPG:C4R4V7|C4R4V7\_KOMPG:C4R4Y0|C4R4Y0\_KOMPG:C4R6S5|C4R6S5\_KOMPG:C4QV63|C4QV63\_KOMPG:C4R4P7|C4R4P7\_KOMPG:C4R9C9|C4R9C9\_KOMPG:C4QZF2|C4QZF2\_KOMPG:C4QWD1|C4QWD1\_KOMPG:C4R5B1|C4R5B1\_KOMPG:C4R493|C4R493\_KOMPG:C4R565|C4R565\_KOMPG:C4R1Z8|C4R1Z8\_KOMPG:C4R1I8|C4R1I8\_KOMPG:C4R2F4|C4R2F4\_KOMPG:C4R6Z4|C4R6Z4\_KOMPG:C4R0L5|C4R0L5\_KOMPG:C4R4B2|C4R4B2\_KOMPG:C4R697|C4R697\_KOMPG:C4R713|C4R713\_KOMPG:C4R545|C4R545\_KOMPG |  |  | DB Search |
| GVSIA | 21.38 | 445.2536 | 5 | -8.02 | 446.2562 | 11.62 | 4611 | 3.39e3 | 1 | 1 | C4R7I0|C4R7I0\_KOMPG:C4QWF7|C4QWF7\_KOMPG |  |  | DB Search |
| GVSLA | 21.38 | 445.2536 | 5 | -8.02 | 446.2562 | 11.62 | 4611 | 3.39e3 | 1 | 1 | C4R7J5|C4R7J5\_KOMPG:C4R6U4|C4R6U4\_KOMPG:C4QXJ8|C4QXJ8\_KOMPG:C4R463|C4R463\_KOMPG:C4QVJ7|C4QVJ7\_KOMPG:C4R3H8|C4R3H8\_KOMPG |  |  | DB Search |
| ALGSPSV | 21.29 | 629.3384 | 7 | 1.91 | 630.3453 | 10.19 | 3968 | 3.85e3 | 1 | 1 | C4R558|C4R558\_KOMPG |  |  | DB Search |
| PGE | 21.28 | 301.1274 | 3 | 1.88 | 302.1345 | 2.04 | 869 | 2.57e3 | 1 | 1 | C4QV10|C4QV10\_KOMPG:C4QW04|LCL3\_KOMPG:C4R3H3|C4R3H3\_KOMPG:C4R4X1|C4R4X1\_KOMPG:C4R2C9|C4R2C9\_KOMPG:C4R3R9|C4R3R9\_KOMPG:C4QZA1|C4QZA1\_KOMPG:C4QXY2|C4QXY2\_KOMPG:C4R564|C4R564\_KOMPG:C4R0M7|C4R0M7\_KOMPG:C4R6M3|C4R6M3\_KOMPG:C4R2A4|C4R2A4\_KOMPG:C4R350|C4R350\_KOMPG:C4R0H4|C4R0H4\_KOMPG:C4QYY2|C4QYY2\_KOMPG:C4QY88|C4QY88\_KOMPG:C4QYX9|C4QYX9\_KOMPG:C4R7M7|C4R7M7\_KOMPG:C4R6W4|C4R6W4\_KOMPG:C4QXA0|C4QXA0\_KOMPG:C4R8A4|C4R8A4\_KOMPG:C4R8Q9|C4R8Q9\_KOMPG:C4R1Q7|C4R1Q7\_KOMPG:C4R5A8|C4R5A8\_KOMPG:C4R4H3|C4R4H3\_KOMPG:C4R4Q2|C4R4Q2\_KOMPG:C4R4R8|ARO1\_KOMPG:C4R7A4|C4R7A4\_KOMPG:C4R571|C4R571\_KOMPG:C4QWR1|C4QWR1\_KOMPG:C4R420|C4R420\_KOMPG:C4R7S0|C4R7S0\_KOMPG:C4R012|C4R012\_KOMPG:C4QW45|C4QW45\_KOMPG:C4R0B6|C4R0B6\_KOMPG:C4R5F3|C4R5F3\_KOMPG:C4R384|C4R384\_KOMPG:C4R002|C4R002\_KOMPG:C4R4Y5|C4R4Y5\_KOMPG:C4R2C2|C4R2C2\_KOMPG:C4QV66|C4QV66\_KOMPG:C4R162|C4R162\_KOMPG:C4R6D1|C4R6D1\_KOMPG:C4R2D2|C4R2D2\_KOMPG:C4R8G8|C4R8G8\_KOMPG:C4R6B9|C4R6B9\_KOMPG:C4QWY9|C4QWY9\_KOMPG:C4R0D1|C4R0D1\_KOMPG:C4QVW8|C4QVW8\_KOMPG:C4R243|C4R243\_KOMPG:C4R7R8|C4R7R8\_KOMPG:C4R1T0|C4R1T0\_KOMPG:C4QWD1|C4QWD1\_KOMPG:C4QYF1|C4QYF1\_KOMPG:C4R6M2|C4R6M2\_KOMPG:C4R3H2|C4R3H2\_KOMPG:C4R6N4|C4R6N4\_KOMPG:C4R6C8|C4R6C8\_KOMPG:C4QZQ4|C4QZQ4\_KOMPG:C4R0N9|C4R0N9\_KOMPG:C4QWX8|C4QWX8\_KOMPG:C4QZ11|C4QZ11\_KOMPG:C4R522|C4R522\_KOMPG:C4R554|C4R554\_KOMPG:C4R6T5|C4R6T5\_KOMPG:C4R2L9|C4R2L9\_KOMPG:C4QX46|C4QX46\_KOMPG:C4R0J1|C4R0J1\_KOMPG:C4R7D8|C4R7D8\_KOMPG:C4R9B6|C4R9B6\_KOMPG:C4R7U9|C4R7U9\_KOMPG:C4R4V8|C4R4V8\_KOMPG:C4R3C5|C4R3C5\_KOMPG:C4QYT3|C4QYT3\_KOMPG:C4QVY5|C4QVY5\_KOMPG:C4R3V7|C4R3V7\_KOMPG:C4R8B2|C4R8B2\_KOMPG:C4R3T0|C4R3T0\_KOMPG:C4QYW0|C4QYW0\_KOMPG:C4QXB6|C4QXB6\_KOMPG:C4QX58|C4QX58\_KOMPG:C4QYQ0|C4QYQ0\_KOMPG:C4QW32|C4QW32\_KOMPG:C4R6G7|C4R6G7\_KOMPG:C4R0B4|C4R0B4\_KOMPG:C4R1N3|C4R1N3\_KOMPG:C4R8M6|C4R8M6\_KOMPG:C4R5E6|C4R5E6\_KOMPG:C4R2Q9|C4R2Q9\_KOMPG:C4R070|C4R070\_KOMPG:C4R1S9|C4R1S9\_KOMPG:C4R4Z2|C4R4Z2\_KOMPG:C4QZ48|C4QZ48\_KOMPG:C4R8U5|C4R8U5\_KOMPG:C4R3F0|C4R3F0\_KOMPG:C4R148|C4R148\_KOMPG:C4R6H9|C4R6H9\_KOMPG:C4QY13|C4QY13\_KOMPG:C4R2H4|C4R2H4\_KOMPG:C4R6Q6|C4R6Q6\_KOMPG:C4R8I0|C4R8I0\_KOMPG:C4QYX4|C4QYX4\_KOMPG:C4QXT3|C4QXT3\_KOMPG:C4R7X4|C4R7X4\_KOMPG:C4R0Q0|C4R0Q0\_KOMPG:C4R1S1|C4R1S1\_KOMPG:C4QX36|C4QX36\_KOMPG:C4QY27|C4QY27\_KOMPG:C4QW27|C4QW27\_KOMPG:C4R1W8|C4R1W8\_KOMPG:C4R1I3|C4R1I3\_KOMPG:C4R7W0|C4R7W0\_KOMPG:C4QZF2|C4QZF2\_KOMPG:C4R0F0|C4R0F0\_KOMPG:C4R7L2|C4R7L2\_KOMPG:C4QZK2|C4QZK2\_KOMPG |  |  | DB Search |
| ITFPL | 21.27 | 589.3475 | 5 | -4.36 | 590.3508 | 43.54 | 13581 | 0 | 0 | 0 | C4R922|C4R922\_KOMPG:C4R7I2|C4R7I2\_KOMPG |  |  | DB Search |
| LTFPI | 21.27 | 589.3475 | 5 | -4.36 | 590.3508 | 43.54 | 13581 | 0 | 0 | 0 | C4QX37|C4QX37\_KOMPG |  |  | DB Search |
| LTFPL | 21.27 | 589.3475 | 5 | -4.36 | 590.3508 | 43.54 | 13581 | 0 | 0 | 0 | C4QZF6|C4QZF6\_KOMPG |  |  | DB Search |
| T(+42.01)TR | 21.26 | 418.2176 | 3 | -1.72 | 419.2231 | 16.24 | 6420 | 6.81e3 | 1 | 1 | C4R2I5|C4R2I5\_KOMPG:C4R5S0|C4R5S0\_KOMPG:C4R166|C4R166\_KOMPG:C4R870|C4R870\_KOMPG:C4QV03|C4QV03\_KOMPG:C4QZ28|C4QZ28\_KOMPG:C4R764|C4R764\_KOMPG:C4R524|C4R524\_KOMPG:C4QV30|C4QV30\_KOMPG:C4QZF7|C4QZF7\_KOMPG:C4R2Z1|C4R2Z1\_KOMPG:C4QYG8|C4QYG8\_KOMPG:C4R0Q9|C4R0Q9\_KOMPG:C4R322|C4R322\_KOMPG:C4QZ41|C4QZ41\_KOMPG | Acetylation (Protein N-term) | T1:Acetylation (Protein N-term):1000 | DB Search |
| VTVI | 21.25 | 430.2791 | 4 | -6.35 | 431.2826 | 27.75 | 9755 | 1.56e3 | 1 | 1 | C4QZN8|C4QZN8\_KOMPG:C4R0R3|C4R0R3\_KOMPG:C4R152|C4R152\_KOMPG:C4QYP9|C4QYP9\_KOMPG:C4QZV5|C4QZV5\_KOMPG:C4QZ17|C4QZ17\_KOMPG:C4R4A1|C4R4A1\_KOMPG:C4R603|C4R603\_KOMPG:C4R6K2|C4R6K2\_KOMPG:C4QWF9|C4QWF9\_KOMPG:C4QW29|C4QW29\_KOMPG:C4R008|C4R008\_KOMPG:C4QVX3|C4QVX3\_KOMPG:C4R8R3|C4R8R3\_KOMPG:C4R6L6|C4R6L6\_KOMPG:C4R3G3|C4R3G3\_KOMPG:C4R614|C4R614\_KOMPG:C4R6S7|C4R6S7\_KOMPG:C4R6S0|C4R6S0\_KOMPG:C4R8Y6|C4R8Y6\_KOMPG:C4R2E8|C4R2E8\_KOMPG:C4R6I9|C4R6I9\_KOMPG:C4R243|C4R243\_KOMPG:C4R8Q6|C4R8Q6\_KOMPG:C4R0D0|C4R0D0\_KOMPG:C4QYK1|C4QYK1\_KOMPG:C4QZB0|C4QZB0\_KOMPG:C4R3C4|C4R3C4\_KOMPG:C4QX95|C4QX95\_KOMPG:C4QV16|C4QV16\_KOMPG:C4R117|C4R117\_KOMPG:C4R267|C4R267\_KOMPG |  |  | DB Search |
| VTVL | 21.25 | 430.2791 | 4 | -6.35 | 431.2826 | 27.75 | 9755 | 1.56e3 | 1 | 1 | C4R5U0|C4R5U0\_KOMPG:C4QVM9|C4QVM9\_KOMPG:C4R044|C4R044\_KOMPG:C4R3I8|C4R3I8\_KOMPG:C4R1Q4|C4R1Q4\_KOMPG:C4QXQ8|C4QXQ8\_KOMPG:C4R9F9|C4R9F9\_KOMPG:C4QWB0|C4QWB0\_KOMPG:C4R091|C4R091\_KOMPG:C4QYV5|C4QYV5\_KOMPG:C4R7V7|C4R7V7\_KOMPG:C4R193|C4R193\_KOMPG:C4R1K1|C4R1K1\_KOMPG:C4R1V4|C4R1V4\_KOMPG:C4QYP0|C4QYP0\_KOMPG:C4R9C1|C4R9C1\_KOMPG:C4QYX4|C4QYX4\_KOMPG:C4QV45|C4QV45\_KOMPG:C4R3Z6|C4R3Z6\_KOMPG:C4R4Q0|C4R4Q0\_KOMPG:C4QZ32|C4QZ32\_KOMPG:C4R224|C4R224\_KOMPG:C4R7N6|C4R7N6\_KOMPG:C4R8C0|C4R8C0\_KOMPG:C4QY86|C4QY86\_KOMPG:C4QYT3|C4QYT3\_KOMPG:C4R498|C4R498\_KOMPG:C4R7W6|C4R7W6\_KOMPG:C4R5M2|C4R5M2\_KOMPG:C4R050|C4R050\_KOMPG:C4R9C9|C4R9C9\_KOMPG:C4R0U0|C4R0U0\_KOMPG:C4QWD1|C4QWD1\_KOMPG:C4R6X3|C4R6X3\_KOMPG:C4R377|C4R377\_KOMPG:C4R9A1|C4R9A1\_KOMPG:C4R754|C4R754\_KOMPG:C4R3S8|C4R3S8\_KOMPG:C4R264|C4R264\_KOMPG:C4R0K2|C4R0K2\_KOMPG:C4R3D2|C4R3D2\_KOMPG:C4QZL6|C4QZL6\_KOMPG:C4R1I2|C4R1I2\_KOMPG:C4R8I5|C4R8I5\_KOMPG:C4R6K4|C4R6K4\_KOMPG |  |  | DB Search |
| VISG | 21.25 | 374.2165 | 4 | -4.06 | 375.2213 | 5.20 | 1991 | 6.27e3 | 1 | 1 | C4R7H8|C4R7H8\_KOMPG:C4QWQ0|C4QWQ0\_KOMPG:C4R1B3|C4R1B3\_KOMPG:C4R010|C4R010\_KOMPG:C4R5S6|C4R5S6\_KOMPG:C4R6T3|C4R6T3\_KOMPG:C4R339|C4R339\_KOMPG:C4R2F1|C4R2F1\_KOMPG:C4QXK0|C4QXK0\_KOMPG:C4R343|C4R343\_KOMPG:C4QWJ0|C4QWJ0\_KOMPG:C4QW17|C4QW17\_KOMPG:C4QYL8|C4QYL8\_KOMPG:C4R8Y2|C4R8Y2\_KOMPG:C4R2H4|C4R2H4\_KOMPG:C4R7T4|C4R7T4\_KOMPG:C4R8C1|C4R8C1\_KOMPG:C4R8W5|C4R8W5\_KOMPG:C4QYF2|LIPA\_KOMPG:C4QY04|C4QY04\_KOMPG:C4R4Y0|C4R4Y0\_KOMPG:C4R6V1|C4R6V1\_KOMPG:C4QWS1|C4QWS1\_KOMPG:C4QYC4|C4QYC4\_KOMPG:C4QWC7|C4QWC7\_KOMPG:C4QXY8|C4QXY8\_KOMPG:C4R5R4|C4R5R4\_KOMPG:C4R5B1|C4R5B1\_KOMPG:C4R5Y8|C4R5Y8\_KOMPG:C4R6U9|C4R6U9\_KOMPG:C4R1Y8|C4R1Y8\_KOMPG:C4R332|C4R332\_KOMPG:C4R5M1|C4R5M1\_KOMPG:C4R1B5|C4R1B5\_KOMPG:C4R255|C4R255\_KOMPG:C4R6W0|C4R6W0\_KOMPG:C4R985|C4R985\_KOMPG:C4QVN0|C4QVN0\_KOMPG:C4QY08|C4QY08\_KOMPG:C4R5Z8|C4R5Z8\_KOMPG:C4QWN2|C4QWN2\_KOMPG |  |  | DB Search |
| VLSG | 21.25 | 374.2165 | 4 | -4.06 | 375.2213 | 5.20 | 1991 | 6.27e3 | 1 | 1 | C4R0V7|C4R0V7\_KOMPG:C4QXW8|C4QXW8\_KOMPG:C4R4M1|C4R4M1\_KOMPG:C4QZS3|C4QZS3\_KOMPG:C4R5N0|SLX4\_KOMPG:C4R1D1|C4R1D1\_KOMPG:C4R3N4|C4R3N4\_KOMPG:C4R4G5|C4R4G5\_KOMPG:C4R116|C4R116\_KOMPG:C4R676|C4R676\_KOMPG:C4R6W4|C4R6W4\_KOMPG:C4R0C0|C4R0C0\_KOMPG:C4R8A4|C4R8A4\_KOMPG:C4R5M0|C4R5M0\_KOMPG:C4R4F0|C4R4F0\_KOMPG:C4R5S9|C4R5S9\_KOMPG:C4QZG6|C4QZG6\_KOMPG:C4QYT0|C4QYT0\_KOMPG:C4R069|C4R069\_KOMPG:C4R983|C4R983\_KOMPG:C4QVZ9|C4QVZ9\_KOMPG:C4QYR8|C4QYR8\_KOMPG:C4R1M4|C4R1M4\_KOMPG:C4R075|C4R075\_KOMPG:C4QXP7|SEC11\_KOMPG:C4QVF7|C4QVF7\_KOMPG:C4R098|C4R098\_KOMPG:C4R1N3|C4R1N3\_KOMPG:C4R0C2|C4R0C2\_KOMPG:C4QXG2|C4QXG2\_KOMPG:C4R3U3|C4R3U3\_KOMPG:C4QZV5|C4QZV5\_KOMPG:C4QYI6|C4QYI6\_KOMPG:C4R2Q9|C4R2Q9\_KOMPG:C4R5V9|C4R5V9\_KOMPG:C4R2T8|C4R2T8\_KOMPG:C4QZ69|C4QZ69\_KOMPG:C4QY53|C4QY53\_KOMPG:C4R4K0|C4R4K0\_KOMPG:C4R942|C4R942\_KOMPG:C4R1N6|C4R1N6\_KOMPG:C4QZC3|C4QZC3\_KOMPG:C4QY43|C4QY43\_KOMPG:C4R951|C4R951\_KOMPG:C4R4N2|C4R4N2\_KOMPG:C4R1Z7|C4R1Z7\_KOMPG:C4QY86|C4QY86\_KOMPG:C4R536|C4R536\_KOMPG:C4R1C2|C4R1C2\_KOMPG:C4R8J5|C4R8J5\_KOMPG:C4QXQ0|C4QXQ0\_KOMPG:C4QZ10|C4QZ10\_KOMPG:C4QZC0|C4QZC0\_KOMPG:C4R0S0|C4R0S0\_KOMPG:C4R055|C4R055\_KOMPG:C4R6D4|C4R6D4\_KOMPG:C4QYG8|C4QYG8\_KOMPG:C4R1C7|C4R1C7\_KOMPG:C4R2K5|C4R2K5\_KOMPG:C4R6E7|C4R6E7\_KOMPG:C4R3S1|C4R3S1\_KOMPG:C4R713|C4R713\_KOMPG:C4R6Y8|C4R6Y8\_KOMPG:C4R3A9|C4R3A9\_KOMPG |  |  | DB Search |
| RCF | 21.23 | 424.1893 | 3 | -9.2 | 425.1916 | 10.20 | 3945 | 1.22e3 | 1 | 1 | C4R3A1|C4R3A1\_KOMPG:C4R3J0|C4R3J0\_KOMPG:C4QWA9|C4QWA9\_KOMPG:C4R9D9|C4R9D9\_KOMPG:C4R6I4|C4R6I4\_KOMPG:C4R2M0|C4R2M0\_KOMPG:C4QZZ7|GEP3\_KOMPG:C4QYB2|C4QYB2\_KOMPG:C4QWI8|C4QWI8\_KOMPG:C4QYA9|C4QYA9\_KOMPG:C4QXW8|C4QXW8\_KOMPG:C4R1U2|C4R1U2\_KOMPG:C4QVD5|C4QVD5\_KOMPG:C4R5F0|C4R5F0\_KOMPG:C4QWM0|C4QWM0\_KOMPG:C4QV01|C4QV01\_KOMPG:C4QVL2|C4QVL2\_KOMPG:C4R5P6|C4R5P6\_KOMPG:C4R780|C4R780\_KOMPG:C4R568|C4R568\_KOMPG:C4R5D2|C4R5D2\_KOMPG:C4R8I4|C4R8I4\_KOMPG:C4QY82|C4QY82\_KOMPG:C4QVC7|C4QVC7\_KOMPG:C4R4I2|C4R4I2\_KOMPG:C4R6S9|C4R6S9\_KOMPG:C4R5D1|C4R5D1\_KOMPG:C4R124|C4R124\_KOMPG:C4R8V7|C4R8V7\_KOMPG:C4R6V2|C4R6V2\_KOMPG:C4QV46|C4QV46\_KOMPG:C4R3S3|C4R3S3\_KOMPG:C4R1M4|C4R1M4\_KOMPG:C4R6P0|C4R6P0\_KOMPG:C4QV18|C4QV18\_KOMPG:C4R3P6|C4R3P6\_KOMPG:C4QYJ1|C4QYJ1\_KOMPG:C4QXD8|C4QXD8\_KOMPG:C4R1V2|C4R1V2\_KOMPG:C4QVP0|C4QVP0\_KOMPG:C4R3W4|C4R3W4\_KOMPG:C4QYS7|C4QYS7\_KOMPG:C4R7V3|C4R7V3\_KOMPG:C4R892|MMM1\_KOMPG:C4R166|C4R166\_KOMPG:C4QWZ3|C4QWZ3\_KOMPG:C4R2G0|C4R2G0\_KOMPG:C4R3Z4|C4R3Z4\_KOMPG:C4R0Z3|C4R0Z3\_KOMPG:C4R955|C4R955\_KOMPG:C4R2Q9|C4R2Q9\_KOMPG:C4R4A1|C4R4A1\_KOMPG:C4QXT4|C4QXT4\_KOMPG:C4QZH7|C4QZH7\_KOMPG:C4R4D8|C4R4D8\_KOMPG:C4QYP5|C4QYP5\_KOMPG:C4R7Z6|C4R7Z6\_KOMPG:C4QZV3|C4QZV3\_KOMPG:C4R0P8|C4R0P8\_KOMPG:C4QYG2|C4QYG2\_KOMPG:C4R4F7|C4R4F7\_KOMPG:C4R0H6|C4R0H6\_KOMPG:C4R1F7|C4R1F7\_KOMPG:C4R6U1|C4R6U1\_KOMPG:C4R685|C4R685\_KOMPG:C4R1U8|C4R1U8\_KOMPG:C4R068|C4R068\_KOMPG:C4R320|C4R320\_KOMPG:C4R4Y4|C4R4Y4\_KOMPG:C4R703|C4R703\_KOMPG:C4R2X1|C4R2X1\_KOMPG:C4R2Q8|C4R2Q8\_KOMPG:C4R6N0|C4R6N0\_KOMPG:C4QXD6|C4QXD6\_KOMPG:C4R678|C4R678\_KOMPG:C4QW02|C4QW02\_KOMPG:C4R8C9|AIM23\_KOMPG:C4R1L5|C4R1L5\_KOMPG:C4R0I1|C4R0I1\_KOMPG:C4QVR3|C4QVR3\_KOMPG:C4R4W8|C4R4W8\_KOMPG:C4QZE6|C4QZE6\_KOMPG:C4R8I6|C4R8I6\_KOMPG:C4R2Q0|C4R2Q0\_KOMPG:C4R814|C4R814\_KOMPG:C4QZT6|C4QZT6\_KOMPG:C4R6A0|C4R6A0\_KOMPG:C4QYQ1|C4QYQ1\_KOMPG:C4R009|C4R009\_KOMPG |  |  | DB Search |
| DIGF | 21.22 | 450.2114 | 4 | 2.45 | 451.2187 | 25.55 | 9169 | 5.5e2 | 1 | 1 | C4R5N2|C4R5N2\_KOMPG:C4QZQ2|C4QZQ2\_KOMPG:C4R0B3|C4R0B3\_KOMPG:C4QYN4|C4QYN4\_KOMPG:C4QY53|C4QY53\_KOMPG:C4QXH0|C4QXH0\_KOMPG:C4R3D9|C4R3D9\_KOMPG:C4R772|C4R772\_KOMPG:C4R8S1|C4R8S1\_KOMPG:C4QV60|C4QV60\_KOMPG:C4R093|C4R093\_KOMPG |  |  | DB Search |
| DLGF | 21.22 | 450.2114 | 4 | 2.45 | 451.2187 | 25.55 | 9169 | 5.5e2 | 1 | 1 | C4R166|C4R166\_KOMPG:C4R5V5|C4R5V5\_KOMPG:C4R916|C4R916\_KOMPG:C4R1D2|C4R1D2\_KOMPG:C4R1U2|C4R1U2\_KOMPG:C4QV22|C4QV22\_KOMPG:C4QVL3|C4QVL3\_KOMPG:C4QWC4|C4QWC4\_KOMPG:C4R248|C4R248\_KOMPG:C4R5H1|C4R5H1\_KOMPG:C4QWU9|C4QWU9\_KOMPG:C4R4J2|C4R4J2\_KOMPG:C4R136|C4R136\_KOMPG:C4R6X7|C4R6X7\_KOMPG:C4R051|C4R051\_KOMPG:C4R7Y5|C4R7Y5\_KOMPG:C4QVR4|C4QVR4\_KOMPG:C4R192|VPS10\_KOMPG:C4R7Q2|C4R7Q2\_KOMPG:C4R4F3|C4R4F3\_KOMPG:C4R3Y7|C4R3Y7\_KOMPG:C4R7I4|C4R7I4\_KOMPG:C4R0T3|C4R0T3\_KOMPG:C4QXB9|C4QXB9\_KOMPG:C4R578|C4R578\_KOMPG:C4QYN7|C4QYN7\_KOMPG:C4R4U1|C4R4U1\_KOMPG:C4R322|C4R322\_KOMPG:C4QWG7|C4QWG7\_KOMPG:C4R513|C4R513\_KOMPG:C4R3S1|C4R3S1\_KOMPG |  |  | DB Search |
| ALVFCIF | 21.21 | 811.4302 | 7 | 6.11 | 812.4404 | 44.09 | 13690 | 2.35e3 | 1 | 1 | C4QX22|C4QX22\_KOMPG |  |  | DB Search |
| AIVTAFVP | 21.21 | 816.4745 | 8 | -4.06 | 817.4764 | 40.62 | 13039 | 1.2e3 | 1 | 1 | C4QZI1|C4QZI1\_KOMPG |  |  | DB Search |
| LAIAVVASGI | 21.18 | 912.5644 | 10 | -8.55 | 913.5616 | 43.28 | 13541 | 5.51e2 | 1 | 1 | C4R610|C4R610\_KOMPG |  |  | DB Search |
| SSKSS | 21.18 | 494.2336 | 5 | -2.91 | 495.2383 | 12.41 | 4849 | 0 | 0 | 0 | C4QXM3|C4QXM3\_KOMPG:C4R6P4|C4R6P4\_KOMPG:C4QXT4|C4QXT4\_KOMPG:C4QV84|C4QV84\_KOMPG:C4QVJ0|C4QVJ0\_KOMPG:C4QYA0|C4QYA0\_KOMPG:C4QVQ2|C4QVQ2\_KOMPG:C4QX55|C4QX55\_KOMPG:C4R8I4|C4R8I4\_KOMPG:C4R4I1|C4R4I1\_KOMPG:C4R382|C4R382\_KOMPG:C4R7H2|C4R7H2\_KOMPG:C4QVC7|C4QVC7\_KOMPG:C4R8C9|AIM23\_KOMPG:C4QVL4|C4QVL4\_KOMPG:C4QW96|C4QW96\_KOMPG:C4R7K1|C4R7K1\_KOMPG:C4R2V0|C4R2V0\_KOMPG:C4QXL1|C4QXL1\_KOMPG:C4R1S5|C4R1S5\_KOMPG:C4QW41|C4QW41\_KOMPG:C4R5E5|C4R5E5\_KOMPG:C4QZQ6|C4QZQ6\_KOMPG:C4R8T3|C4R8T3\_KOMPG:C4QY10|C4QY10\_KOMPG:C4QY26|C4QY26\_KOMPG |  |  | DB Search |
| KYT | 21.18 | 410.2165 | 3 | -4.93 | 411.2208 | 12.54 | 4954 | 3.83e2 | 1 | 1 | C4R278|C4R278\_KOMPG:C4R8L6|C4R8L6\_KOMPG:C4QYA9|C4QYA9\_KOMPG:C4R894|C4R894\_KOMPG:C4R006|C4R006\_KOMPG:C4QX16|C4QX16\_KOMPG:C4R0M7|C4R0M7\_KOMPG:C4R0D8|C4R0D8\_KOMPG:C4QYT2|C4QYT2\_KOMPG:C4R5W8|C4R5W8\_KOMPG:C4QVL2|C4QVL2\_KOMPG:C4R8J6|C4R8J6\_KOMPG:C4R1X4|C4R1X4\_KOMPG:C4R1P8|C4R1P8\_KOMPG:C4R0U4|C4R0U4\_KOMPG:C4QVS7|C4QVS7\_KOMPG:C4R1J2|C4R1J2\_KOMPG:C4QZX7|C4QZX7\_KOMPG:C4QW86|C4QW86\_KOMPG:C4R1T4|C4R1T4\_KOMPG:C4R076|C4R076\_KOMPG:C4QZU5|C4QZU5\_KOMPG:C4R2S5|C4R2S5\_KOMPG:C4R5N8|C4R5N8\_KOMPG:C4R573|C4R573\_KOMPG:C4QWU0|C4QWU0\_KOMPG:C4R3C4|C4R3C4\_KOMPG:C4R6A6|C4R6A6\_KOMPG:C4R0L0|C4R0L0\_KOMPG:C4QWR3|C4QWR3\_KOMPG:C4R233|C4R233\_KOMPG:C4QZX5|C4QZX5\_KOMPG:C4R1G2|C4R1G2\_KOMPG:C4QY36|C4QY36\_KOMPG:C4R955|C4R955\_KOMPG:C4R384|C4R384\_KOMPG:C4R1M9|C4R1M9\_KOMPG:C4QZA3|C4QZA3\_KOMPG:C4R8Y2|C4R8Y2\_KOMPG:C4R0V8|C4R0V8\_KOMPG:C4R8Q0|C4R8Q0\_KOMPG:C4QY23|C4QY23\_KOMPG:C4R951|C4R951\_KOMPG:C4R5K3|C4R5K3\_KOMPG:C4R6G5|C4R6G5\_KOMPG:C4R0J7|C4R0J7\_KOMPG:C4R1M6|C4R1M6\_KOMPG:C4QWA5|C4QWA5\_KOMPG:C4R8J5|C4R8J5\_KOMPG:C4QWA8|C4QWA8\_KOMPG:C4R6I2|C4R6I2\_KOMPG:C4QWD1|C4QWD1\_KOMPG:C4R378|C4R378\_KOMPG:C4QZW1|C4QZW1\_KOMPG:C4R1I1|C4R1I1\_KOMPG:C4R8P5|C4R8P5\_KOMPG:C4R424|C4R424\_KOMPG:C4QZL4|C4QZL4\_KOMPG:C4QZQ4|C4QZQ4\_KOMPG:C4R8J1|C4R8J1\_KOMPG:C4QW03|C4QW03\_KOMPG:C4QYH7|C4QYH7\_KOMPG:C4R2B7|C4R2B7\_KOMPG:C4QZ79|C4QZ79\_KOMPG:C4R6N7|C4R6N7\_KOMPG:C4R1N5|C4R1N5\_KOMPG:C4QV67|C4QV67\_KOMPG:C4R7P5|C4R7P5\_KOMPG:C4QYJ6|C4QYJ6\_KOMPG:C4R6P6|C4R6P6\_KOMPG:C4R6D3|C4R6D3\_KOMPG:C4QV12|C4QV12\_KOMPG:C4R3C5|C4R3C5\_KOMPG:C4R601|C4R601\_KOMPG:C4R069|C4R069\_KOMPG:C4QYT3|C4QYT3\_KOMPG:C4QVJ6|C4QVJ6\_KOMPG:C4R4A0|C4R4A0\_KOMPG:C4R0P1|C4R0P1\_KOMPG:C4R6U9|C4R6U9\_KOMPG:C4QXD4|C4QXD4\_KOMPG:C4QW41|C4QW41\_KOMPG:C4QX95|C4QX95\_KOMPG:C4R8G0|C4R8G0\_KOMPG:C4R3M3|C4R3M3\_KOMPG:C4QZE2|C4QZE2\_KOMPG:C4R070|C4R070\_KOMPG:C4R3K1|C4R3K1\_KOMPG:C4R0M3|C4R0M3\_KOMPG:C4R8H1|C4R8H1\_KOMPG:C4R0A9|C4R0A9\_KOMPG:C4QYQ2|C4QYQ2\_KOMPG:C4QZV9|C4QZV9\_KOMPG:C4QW18|C4QW18\_KOMPG:C4R508|C4R508\_KOMPG:C4R316|C4R316\_KOMPG:C4R134|C4R134\_KOMPG:C4R7X4|C4R7X4\_KOMPG:C4R3B1|C4R3B1\_KOMPG:C4R3R7|C4R3R7\_KOMPG:C4QVR9|IRC19\_KOMPG:C4QV13|C4QV13\_KOMPG:C4QWC1|C4QWC1\_KOMPG:C4QWU8|C4QWU8\_KOMPG:C4R7P7|C4R7P7\_KOMPG:C4QWC7|C4QWC7\_KOMPG:C4QW06|C4QW06\_KOMPG:C4QZB8|C4QZB8\_KOMPG:C4R0J5|C4R0J5\_KOMPG:C4R5D7|C4R5D7\_KOMPG:C4R0N3|C4R0N3\_KOMPG:C4QZS0|C4QZS0\_KOMPG:C4QYR5|C4QYR5\_KOMPG |  |  | DB Search |
| GVSPGGV | 21.17 | 571.2966 | 7 | -8.51 | 572.2975 | 10.15 | 3967 | 0 | 0 | 0 | C4R7H9|C4R7H9\_KOMPG |  |  | DB Search |
| VPVP | 21.17 | 410.2529 | 4 | -4.84 | 411.2572 | 13.89 | 5373 | 1.28e4 | 1 | 1 | C4R0R3|C4R0R3\_KOMPG:C4R8N9|C4R8N9\_KOMPG:C4QZE2|C4QZE2\_KOMPG:C4QWF7|C4QWF7\_KOMPG:C4QVY0|C4QVY0\_KOMPG:C4R709|C4R709\_KOMPG:C4R3V6|C4R3V6\_KOMPG:C4R8F3|C4R8F3\_KOMPG:C4R4S9|C4R4S9\_KOMPG:C4QYG2|C4QYG2\_KOMPG:C4R308|C4R308\_KOMPG:C4QW37|C4QW37\_KOMPG:C4R108|C4R108\_KOMPG:C4QX52|C4QX52\_KOMPG:C4R570|C4R570\_KOMPG:C4R762|C4R762\_KOMPG:C4QYR3|C4QYR3\_KOMPG:C4R3T8|C4R3T8\_KOMPG:C4QWC1|C4QWC1\_KOMPG:C4QY42|C4QY42\_KOMPG:C4R4J6|C4R4J6\_KOMPG:C4R931|C4R931\_KOMPG:C4R858|C4R858\_KOMPG:C4R0W5|C4R0W5\_KOMPG:C4QVK2|C4QVK2\_KOMPG:C4QVC9|C4QVC9\_KOMPG:C4R6Z4|C4R6Z4\_KOMPG:C4R1X8|C4R1X8\_KOMPG |  |  | DB Search |
| PSVP | 21.16 | 398.2165 | 4 | 4.76 | 399.2247 | 9.14 | 3512 | 0 | 0 | 0 | C4QVV6|C4QVV6\_KOMPG:C4QVY4|C4QVY4\_KOMPG:C4R0Z3|C4R0Z3\_KOMPG:C4R744|C4R744\_KOMPG:C4QV60|C4QV60\_KOMPG:C4QV22|C4QV22\_KOMPG:C4QYP4|C4QYP4\_KOMPG:C4R0V8|C4R0V8\_KOMPG:C4QV43|C4QV43\_KOMPG:C4R3I7|C4R3I7\_KOMPG:C4QZ76|C4QZ76\_KOMPG:C4R234|C4R234\_KOMPG:C4R476|C4R476\_KOMPG:C4R5W4|C4R5W4\_KOMPG:C4R4C6|C4R4C6\_KOMPG:C4QWI6|C4QWI6\_KOMPG:C4QZ71|C4QZ71\_KOMPG:C4R288|C4R288\_KOMPG:C4QVG3|C4QVG3\_KOMPG:C4QZT4|C4QZT4\_KOMPG:C4R8Y0|C4R8Y0\_KOMPG:C4QY16|C4QY16\_KOMPG:C4R7S7|GET1\_KOMPG:C4R100|C4R100\_KOMPG:C4R735|C4R735\_KOMPG:C4QV65|C4QV65\_KOMPG:C4R5R5|C4R5R5\_KOMPG:C4R795|C4R795\_KOMPG:C4QZU4|C4QZU4\_KOMPG:C4R2K9|C4R2K9\_KOMPG:C4R057|C4R057\_KOMPG:C4R430|C4R430\_KOMPG |  |  | DB Search |
| EIA | 21.09 | 331.1743 | 3 | -5.12 | 332.1791 | 6.98 | 2655 | 4.41e3 | 1 | 1 | C4R107|C4R107\_KOMPG:C4QZ38|C4QZ38\_KOMPG:C4R8H4|C4R8H4\_KOMPG:C4R8S4|C4R8S4\_KOMPG:C4QYZ3|C4QYZ3\_KOMPG:C4QZK8|C4QZK8\_KOMPG:C4R291|C4R291\_KOMPG:C4QYR2|C4QYR2\_KOMPG:C4R1R3|C4R1R3\_KOMPG:C4QY19|C4QY19\_KOMPG:C4R3G3|C4R3G3\_KOMPG:C4R2W1|C4R2W1\_KOMPG:C4R6L7|C4R6L7\_KOMPG:C4QVQ7|C4QVQ7\_KOMPG:C4R912|C4R912\_KOMPG:C4R3C7|C4R3C7\_KOMPG:C4R7W9|C4R7W9\_KOMPG:C4QXU3|C4QXU3\_KOMPG:C4R9B2|C4R9B2\_KOMPG:C4R7Y1|C4R7Y1\_KOMPG:C4QY28|C4QY28\_KOMPG:C4R849|C4R849\_KOMPG:C4QXU5|C4QXU5\_KOMPG:C4R2G2|C4R2G2\_KOMPG:C4R0K3|C4R0K3\_KOMPG:C4R332|C4R332\_KOMPG:C4R565|C4R565\_KOMPG:C4R095|C4R095\_KOMPG:C4R855|C4R855\_KOMPG:C4R033|C4R033\_KOMPG:C4QYE3|C4QYE3\_KOMPG:C4R9E2|C4R9E2\_KOMPG |  |  | DB Search |
| ELA | 21.09 | 331.1743 | 3 | -5.12 | 332.1791 | 6.98 | 2655 | 4.41e3 | 1 | 1 | C4R6G4|C4R6G4\_KOMPG:C4R554|C4R554\_KOMPG:C4R0S9|C4R0S9\_KOMPG:C4QZZ8|C4QZZ8\_KOMPG:C4R3I7|C4R3I7\_KOMPG:C4R1N5|C4R1N5\_KOMPG:C4R751|C4R751\_KOMPG:Q9P4D0|SEC17\_KOMPG:C4QYG5|C4QYG5\_KOMPG:C4R5J4|C4R5J4\_KOMPG:C4R1C0|C4R1C0\_KOMPG:C4QZX7|C4QZX7\_KOMPG:C4R1B9|C4R1B9\_KOMPG:C4R765|C4R765\_KOMPG:C4R3N3|C4R3N3\_KOMPG:C4QXM1|C4QXM1\_KOMPG:C4QYY0|C4QYY0\_KOMPG:C4R5N8|C4R5N8\_KOMPG:C4R707|C4R707\_KOMPG:C4QWS7|C4QWS7\_KOMPG:C4R349|C4R349\_KOMPG:C4QWH2|C4QWH2\_KOMPG:C4QXN2|MIC60\_KOMPG:C4QYQ0|C4QYQ0\_KOMPG:C4R2U9|C4R2U9\_KOMPG:C4QVT0|C4QVT0\_KOMPG:C4R893|C4R893\_KOMPG:C4QV97|C4QV97\_KOMPG:C4R233|C4R233\_KOMPG:C4R341|C4R341\_KOMPG:C4QXL0|C4QXL0\_KOMPG:C4QXM3|C4QXM3\_KOMPG:C4QVV6|C4QVV6\_KOMPG:C4R5C1|C4R5C1\_KOMPG:C4R2Y2|C4R2Y2\_KOMPG:C4QVL8|C4QVL8\_KOMPG:C4QVX9|C4QVX9\_KOMPG:C4QXT4|C4QXT4\_KOMPG:C4R7N8|C4R7N8\_KOMPG:C4QW84|C4QW84\_KOMPG:C4R0X4|C4R0X4\_KOMPG:C4R0I8|C4R0I8\_KOMPG:C4R7J7|C4R7J7\_KOMPG:C4R310|C4R310\_KOMPG:C4R5S1|C4R5S1\_KOMPG:C4QVX3|C4QVX3\_KOMPG:C4R2V3|C4R2V3\_KOMPG:C4R148|C4R148\_KOMPG:C4R0V8|C4R0V8\_KOMPG:C4QZ69|C4QZ69\_KOMPG:C4QX18|C4QX18\_KOMPG:C4R6F8|C4R6F8\_KOMPG:C4R1Q3|C4R1Q3\_KOMPG:C4QZV3|C4QZV3\_KOMPG:C4QVQ8|C4QVQ8\_KOMPG:C4R1H3|C4R1H3\_KOMPG:C4R276|C4R276\_KOMPG:C4R4L6|C4R4L6\_KOMPG:C4R6N1|C4R6N1\_KOMPG:C4R5F7|C4R5F7\_KOMPG:C4R4Y4|C4R4Y4\_KOMPG:C4R093|C4R093\_KOMPG:C4R5K3|C4R5K3\_KOMPG:C4QZX0|C4QZX0\_KOMPG:C4R0Q0|C4R0Q0\_KOMPG:C4R269|C4R269\_KOMPG:C4R129|C4R129\_KOMPG:C4R4K5|C4R4K5\_KOMPG:C4R393|C4R393\_KOMPG:C4R4V7|C4R4V7\_KOMPG:C4R1U7|C4R1U7\_KOMPG:C4R370|C4R370\_KOMPG:C4QV13|C4QV13\_KOMPG:C4R7R8|C4R7R8\_KOMPG:C4QWU8|C4QWU8\_KOMPG:C4R7C8|C4R7C8\_KOMPG:C4R1H7|C4R1H7\_KOMPG:C4QXA2|C4QXA2\_KOMPG:C4QWD1|C4QWD1\_KOMPG:C4R5H2|C4R5H2\_KOMPG:C4QXZ9|C4QXZ9\_KOMPG:C4QY10|C4QY10\_KOMPG:C4QWS9|C4QWS9\_KOMPG:C4R749|C4R749\_KOMPG:C4R3H2|C4R3H2\_KOMPG:C4R503|C4R503\_KOMPG:C4QYK4|C4QYK4\_KOMPG:C4QVR5|C4QVR5\_KOMPG:C4R405|C4R405\_KOMPG |  |  | DB Search |
| VISLQELC(-1.01) | 21.07 | 902.4657 | 8 | 1.17 | 903.4718 | 16.16 | 6425 | 0 | 0 | 0 | C4QWS1|C4QWS1\_KOMPG | Half of a disulfide bridge | C8:Half of a disulfide bridge:1000 | DB Search |
| IPRP | 21.04 | 481.3012 | 4 | -9.61 | 482.3027 | 17.02 | 6682 | 0 | 0 | 0 | C4QZE2|C4QZE2\_KOMPG:C4R5E7|C4R5E7\_KOMPG:C4R1C3|C4R1C3\_KOMPG:C4QWS5|C4QWS5\_KOMPG:C4R6B0|OXDD\_KOMPG:C4R155|C4R155\_KOMPG:C4R5T4|C4R5T4\_KOMPG:C4R407|C4R407\_KOMPG:C4R8H1|C4R8H1\_KOMPG:C4QX80|PSD1\_KOMPG:C4R4Z7|C4R4Z7\_KOMPG:C4R3L9|C4R3L9\_KOMPG:C4R293|C4R293\_KOMPG:C4R6R8|C4R6R8\_KOMPG:C4QVJ9|C4QVJ9\_KOMPG:C4R4G9|OXDA\_KOMPG:C4QYP6|C4QYP6\_KOMPG:C4R0X8|C4R0X8\_KOMPG:C4QXM9|C4QXM9\_KOMPG:C4R6W7|C4R6W7\_KOMPG |  |  | DB Search |
| LPRP | 21.04 | 481.3012 | 4 | -9.61 | 482.3027 | 17.02 | 6682 | 0 | 0 | 0 | C4R5W6|C4R5W6\_KOMPG:C4QY66|C4QY66\_KOMPG:C4R8D7|ATG37\_KOMPG:C4R999|C4R999\_KOMPG:C4QZI9|C4QZI9\_KOMPG:C4R8Q9|C4R8Q9\_KOMPG:C4R4N7|C4R4N7\_KOMPG:C4R8L3|C4R8L3\_KOMPG:C4R001|C4R001\_KOMPG:C4R153|C4R153\_KOMPG:C4R828|C4R828\_KOMPG:C4R0W3|C4R0W3\_KOMPG:C4R246|C4R246\_KOMPG:C4QYG8|C4QYG8\_KOMPG:C4R508|C4R508\_KOMPG:C4R7W7|C4R7W7\_KOMPG:C4R6Z9|C4R6Z9\_KOMPG:C4QZY8|C4QZY8\_KOMPG:C4R4Y2|C4R4Y2\_KOMPG:C4QYY9|C4QYY9\_KOMPG:C4R6X2|C4R6X2\_KOMPG |  |  | DB Search |
| WAN | 21.03 | 389.1699 | 3 | 5.94 | 390.1785 | 10.28 | 4047 | 1.16e3 | 1 | 1 | C4R6R3|C4R6R3\_KOMPG:C4QX06|C4QX06\_KOMPG:C4R006|C4R006\_KOMPG:C4QV60|C4QV60\_KOMPG:C4R3X1|C4R3X1\_KOMPG:C4QV06|C4QV06\_KOMPG:C4QXZ1|C4QXZ1\_KOMPG:C4R7P2|C4R7P2\_KOMPG:C4R181|C4R181\_KOMPG:C4R6Y0|C4R6Y0\_KOMPG:C4R5F1|C4R5F1\_KOMPG:C4QVX4|C4QVX4\_KOMPG:C4R5Y8|C4R5Y8\_KOMPG:C4QW41|C4QW41\_KOMPG:C4QYF9|C4QYF9\_KOMPG:C4R0R2|C4R0R2\_KOMPG:C4R2Q4|C4R2Q4\_KOMPG:C4QXL0|C4QXL0\_KOMPG:C4QZQ5|C4QZQ5\_KOMPG:C4R921|C4R921\_KOMPG:C4QZ59|C4QZ59\_KOMPG:C4R5T9|C4R5T9\_KOMPG:C4R4P5|C4R4P5\_KOMPG:C4R3V1|C4R3V1\_KOMPG:C4R0N1|C4R0N1\_KOMPG:C4QXI8|PEX6\_KOMPG:C4R8K7|C4R8K7\_KOMPG:C4QZM1|C4QZM1\_KOMPG:C4QZ51|C4QZ51\_KOMPG:C4R0A6|C4R0A6\_KOMPG:C4R5R2|C4R5R2\_KOMPG:C4R162|C4R162\_KOMPG:C4R4I6|C4R4I6\_KOMPG:C4R8S6|C4R8S6\_KOMPG:C4R7D2|C4R7D2\_KOMPG:C4R8F3|C4R8F3\_KOMPG:C4QWE4|C4QWE4\_KOMPG:C4QYP7|C4QYP7\_KOMPG:C4R7Y7|C4R7Y7\_KOMPG:C4R4A6|C4R4A6\_KOMPG:C4R2Y1|C4R2Y1\_KOMPG:C4R1A0|C4R1A0\_KOMPG:C4R2K8|C4R2K8\_KOMPG:C4QVW2|C4QVW2\_KOMPG:C4R692|C4R692\_KOMPG:C4QY86|C4QY86\_KOMPG:C4R5X0|C4R5X0\_KOMPG:C4QYX6|C4QYX6\_KOMPG:C4R862|C4R862\_KOMPG:C4QVW8|C4QVW8\_KOMPG:C4R5C9|C4R5C9\_KOMPG:C4R1D3|C4R1D3\_KOMPG:C4QYK1|C4QYK1\_KOMPG:C4R813|C4R813\_KOMPG:C4R2B2|C4R2B2\_KOMPG:C4R2F4|C4R2F4\_KOMPG:C4R0W0|C4R0W0\_KOMPG:C4QVM6|C4QVM6\_KOMPG:C4R176|C4R176\_KOMPG |  |  | DB Search |
| DVTI | 21.02 | 446.2376 | 4 | -4.47 | 447.2418 | 16.24 | 6386 | 3.07e3 | 1 | 1 | C4R953|C4R953\_KOMPG:C4R6A8|C4R6A8\_KOMPG:C4QYT7|C4QYT7\_KOMPG:C4QXW1|C4QXW1\_KOMPG:C4R809|C4R809\_KOMPG:C4QZR7|C4QZR7\_KOMPG:C4QW03|C4QW03\_KOMPG:C4R413|C4R413\_KOMPG:C4R5A4|C4R5A4\_KOMPG:C4R7I2|C4R7I2\_KOMPG:C4QX68|C4QX68\_KOMPG:C4R7F9|C4R7F9\_KOMPG:C4R6P1|C4R6P1\_KOMPG:C4R705|C4R705\_KOMPG:C4R4D7|C4R4D7\_KOMPG:C4R198|C4R198\_KOMPG:C4QVI7|C4QVI7\_KOMPG:C4QZ71|C4QZ71\_KOMPG:C4QVX5|C4QVX5\_KOMPG:C4R155|C4R155\_KOMPG:C4R875|C4R875\_KOMPG:C4QXF0|C4QXF0\_KOMPG:C4QZS4|C4QZS4\_KOMPG:C4QZH9|C4QZH9\_KOMPG:C4R970|ASA1\_KOMPG:C4QYD4|C4QYD4\_KOMPG |  |  | DB Search |
| DVTL | 21.02 | 446.2376 | 4 | -4.47 | 447.2418 | 16.24 | 6386 | 3.07e3 | 1 | 1 | C4QXE7|C4QXE7\_KOMPG:C4QZQ5|C4QZQ5\_KOMPG:C4R2G0|C4R2G0\_KOMPG:C4QVT8|C4QVT8\_KOMPG:C4R581|C4R581\_KOMPG:C4QYU4|C4QYU4\_KOMPG:C4R4H4|C4R4H4\_KOMPG:C4R8F2|C4R8F2\_KOMPG:C4R1U2|C4R1U2\_KOMPG:C4QYZ9|C4QYZ9\_KOMPG:C4R025|C4R025\_KOMPG:C4R0N7|C4R0N7\_KOMPG:C4R447|C4R447\_KOMPG:C4QYP0|C4QYP0\_KOMPG:C4R656|C4R656\_KOMPG:C4QYP7|C4QYP7\_KOMPG:C4R221|C4R221\_KOMPG:C4R806|C4R806\_KOMPG:C4R1A8|C4R1A8\_KOMPG:C4R5J9|C4R5J9\_KOMPG:C4QVT7|C4QVT7\_KOMPG:C4R6P6|C4R6P6\_KOMPG:C4R1T5|C4R1T5\_KOMPG:C4R1T4|C4R1T4\_KOMPG:C4QWR5|C4QWR5\_KOMPG:C4QZC5|C4QZC5\_KOMPG:C4R4L1|C4R4L1\_KOMPG:C4R4V5|C4R4V5\_KOMPG:C4R9A3|C4R9A3\_KOMPG:C4R7L0|C4R7L0\_KOMPG:C4QYX7|C4QYX7\_KOMPG:C4QYX5|C4QYX5\_KOMPG:C4QZB8|C4QZB8\_KOMPG:C4R7Q9|C4R7Q9\_KOMPG:C4QZC9|C4QZC9\_KOMPG:C4R0H5|C4R0H5\_KOMPG:C4QVK2|C4QVK2\_KOMPG:C4R360|PSD2\_KOMPG:C4R4E8|C4R4E8\_KOMPG:C4R2D7|FLO11\_KOMPG:C4R4M5|C4R4M5\_KOMPG |  |  | DB Search |
| QQQQQQKQQPPP | 20.99 | 1461.7324 | 12 | -0.8 | 731.8711 | 41.07 | 13132 | 6.76e3 | 1 | 1 | C4R8P7|C4R8P7\_KOMPG |  |  | DB Search |
| ISAGI | 20.97 | 459.2693 | 5 | -3.77 | 460.2737 | 22.08 | 8183 | 4.69e2 | 1 | 1 | C4R713|C4R713\_KOMPG |  |  | DB Search |
| ISAGL | 20.97 | 459.2693 | 5 | -3.77 | 460.2737 | 22.08 | 8183 | 4.69e2 | 1 | 1 | C4R7F2|C4R7F2\_KOMPG:C4R0U5|C4R0U5\_KOMPG |  |  | DB Search |
| LSAGI | 20.97 | 459.2693 | 5 | -3.77 | 460.2737 | 22.08 | 8183 | 4.69e2 | 1 | 1 | C4R3D7|C4R3D7\_KOMPG:C4QX81|C4QX81\_KOMPG:C4QY10|C4QY10\_KOMPG:C4R154|C4R154\_KOMPG |  |  | DB Search |
| LSAGL | 20.97 | 459.2693 | 5 | -3.77 | 460.2737 | 22.08 | 8183 | 4.69e2 | 1 | 1 | C4R7U2|C4R7U2\_KOMPG:C4QZF9|C4QZF9\_KOMPG:C4QW53|C4QW53\_KOMPG:C4R814|C4R814\_KOMPG:C4R3B0|C4R3B0\_KOMPG:P04842|ALOX1\_KOMPG |  |  | DB Search |
| ASGVE | 20.96 | 461.2122 | 5 | -3.23 | 462.2168 | 4.82 | 1775 | 2.59e3 | 1 | 1 | C4QZ19|C4QZ19\_KOMPG:C4R5G2|C4R5G2\_KOMPG:C4R0K5|C4R0K5\_KOMPG:C4R115|C4R115\_KOMPG |  |  | DB Search |
| FISLVP | 20.93 | 674.4003 | 6 | -6.34 | 675.4016 | 42.02 | 13315 | 6.45e2 | 1 | 1 | C4R723|C4R723\_KOMPG |  |  | DB Search |
| FLSIVP | 20.93 | 674.4003 | 6 | -6.34 | 675.4016 | 42.02 | 13315 | 6.45e2 | 1 | 1 | C4QZN7|C4QZN7\_KOMPG |  |  | DB Search |
| FLSLVP | 20.93 | 674.4003 | 6 | -6.34 | 675.4016 | 42.02 | 13315 | 6.45e2 | 1 | 1 | C4QXD7|C4QXD7\_KOMPG |  |  | DB Search |
| VASSI | 20.92 | 475.2642 | 5 | -5.07 | 476.2679 | 17.73 | 6973 | 0 | 0 | 0 | C4R675|C4R675\_KOMPG:C4R2E9|C4R2E9\_KOMPG:C4R7S0|C4R7S0\_KOMPG:C4R5M2|C4R5M2\_KOMPG:C4R239|C4R239\_KOMPG:C4R3R8|ARGJ\_KOMPG:C4R849|C4R849\_KOMPG |  |  | DB Search |
| VASSL | 20.92 | 475.2642 | 5 | -5.07 | 476.2679 | 17.73 | 6973 | 0 | 0 | 0 | C4R3B8|C4R3B8\_KOMPG:C4R571|C4R571\_KOMPG:C4R6I8|C4R6I8\_KOMPG:C4QWZ7|C4QWZ7\_KOMPG |  |  | DB Search |
| Q(-17.03)PVTGSRQPP | 20.91 | 1048.5302 | 10 | 4.05 | 1049.5391 | 20.46 | 7708 | 3.73e2 | 1 | 1 | C4R317|C4R317\_KOMPG | Pyro-glu from Q | Q1:Pyro-glu from Q:1000 | DB Search |
| ITDI | 20.88 | 460.2533 | 4 | -0.2 | 461.2593 | 14.80 | 5851 | 7.36e2 | 1 | 1 | C4R830|C4R830\_KOMPG:C4QXR3|C4QXR3\_KOMPG:C4R3D7|C4R3D7\_KOMPG:C4R2T7|C4R2T7\_KOMPG:C4R1D2|C4R1D2\_KOMPG:C4R2H0|C4R2H0\_KOMPG:C4R0M3|C4R0M3\_KOMPG:C4R2V3|C4R2V3\_KOMPG:C4QYT2|C4QYT2\_KOMPG:C4QVL2|C4QVL2\_KOMPG:C4QV01|C4QV01\_KOMPG:C4R4Q8|C4R4Q8\_KOMPG:C4R381|C4R381\_KOMPG:C4R051|C4R051\_KOMPG:C4QWE0|C4QWE0\_KOMPG:C4QWQ3|C4QWQ3\_KOMPG:C4R7D7|C4R7D7\_KOMPG:C4R3C5|C4R3C5\_KOMPG:C4R6L8|C4R6L8\_KOMPG:C4R772|C4R772\_KOMPG:C4R5Q7|C4R5Q7\_KOMPG:C4R243|C4R243\_KOMPG:C4R5J2|C4R5J2\_KOMPG:C4QZA7|C4QZA7\_KOMPG:C4R1S3|C4R1S3\_KOMPG:C4R8P5|C4R8P5\_KOMPG:C4QYD7|C4QYD7\_KOMPG:C4R189|C4R189\_KOMPG:C4R4M3|C4R4M3\_KOMPG |  |  | DB Search |
| ITDL | 20.88 | 460.2533 | 4 | -0.2 | 461.2593 | 14.80 | 5851 | 7.36e2 | 1 | 1 | C4QZW5|C4QZW5\_KOMPG:C4QWQ0|C4QWQ0\_KOMPG:C4R467|C4R467\_KOMPG:C4QVG1|C4QVG1\_KOMPG:C4R8U5|C4R8U5\_KOMPG:C4QZ83|C4QZ83\_KOMPG:C4QV66|C4QV66\_KOMPG:C4R1K1|C4R1K1\_KOMPG:C4R398|C4R398\_KOMPG:C4QWN5|C4QWN5\_KOMPG:C4QW83|C4QW83\_KOMPG:C4R325|C4R325\_KOMPG:C4QW86|C4QW86\_KOMPG:C4R492|OCA5\_KOMPG:C4R393|C4R393\_KOMPG:C4R516|C4R516\_KOMPG:C4QZU5|C4QZU5\_KOMPG:C4QVT9|C4QVT9\_KOMPG:C4R3Q3|C4R3Q3\_KOMPG:C4QWM2|C4QWM2\_KOMPG:C4R6N3|C4R6N3\_KOMPG:C4R548|C4R548\_KOMPG:C4R174|C4R174\_KOMPG:C4R7Q9|C4R7Q9\_KOMPG:C4R3W0|C4R3W0\_KOMPG:C4R3C4|C4R3C4\_KOMPG:C4R7U4|C4R7U4\_KOMPG |  |  | DB Search |
| LTDI | 20.88 | 460.2533 | 4 | -0.2 | 461.2593 | 14.80 | 5851 | 7.36e2 | 1 | 1 | C4R2U7|C4R2U7\_KOMPG:C4R821|C4R821\_KOMPG:C4R9D1|C4R9D1\_KOMPG:C4R2P3|MAP2\_KOMPG:C4QX89|C4QX89\_KOMPG:C4R8J1|C4R8J1\_KOMPG:C4R5A2|C4R5A2\_KOMPG:C4R0X3|C4R0X3\_KOMPG:C4R143|C4R143\_KOMPG:C4R4V3|C4R4V3\_KOMPG:C4QYL9|C4QYL9\_KOMPG:C4R2U1|C4R2U1\_KOMPG:C4R6H6|C4R6H6\_KOMPG:C4R2H6|C4R2H6\_KOMPG:C4R1M4|C4R1M4\_KOMPG:C4R9B4|C4R9B4\_KOMPG:C4R941|GLG\_KOMPG:C4R6U9|C4R6U9\_KOMPG:C4R747|C4R747\_KOMPG:C4QVM1|C4QVM1\_KOMPG:C4R2D0|C4R2D0\_KOMPG:C4QVT3|C4QVT3\_KOMPG |  |  | DB Search |
| LTDL | 20.88 | 460.2533 | 4 | -0.2 | 461.2593 | 14.80 | 5851 | 7.36e2 | 1 | 1 | C4QXE5|C4QXE5\_KOMPG:C4R2V8|C4R2V8\_KOMPG:C4R315|C4R315\_KOMPG:C4QYT7|C4QYT7\_KOMPG:C4R584|C4R584\_KOMPG:C4QV93|C4QV93\_KOMPG:C4QX67|C4QX67\_KOMPG:C4R6H5|C4R6H5\_KOMPG:C4R214|C4R214\_KOMPG:C4R1U2|C4R1U2\_KOMPG:C4QVL3|C4QVL3\_KOMPG:C4QZ07|C4QZ07\_KOMPG:C4QZZ8|C4QZZ8\_KOMPG:C4R7Z6|C4R7Z6\_KOMPG:C4R5F6|C4R5F6\_KOMPG:C4R7G4|C4R7G4\_KOMPG:C4QY53|C4QY53\_KOMPG:C4R0Q9|C4R0Q9\_KOMPG:C4QX55|C4QX55\_KOMPG:C4R888|C4R888\_KOMPG:C4R7I3|C4R7I3\_KOMPG:C4QW91|C4QW91\_KOMPG:C4R0X2|C4R0X2\_KOMPG:C4R7R3|C4R7R3\_KOMPG:C4R869|C4R869\_KOMPG:C4R191|C4R191\_KOMPG:C4R6W4|C4R6W4\_KOMPG:C4QWI9|C4QWI9\_KOMPG:C4QW27|C4QW27\_KOMPG:C4R664|C4R664\_KOMPG:C4R0D1|C4R0D1\_KOMPG:C4R9B2|C4R9B2\_KOMPG:C4R2G7|C4R2G7\_KOMPG:C4QXA2|C4QXA2\_KOMPG:C4R7J5|C4R7J5\_KOMPG:C4QYL4|C4QYL4\_KOMPG:C4R2Z8|C4R2Z8\_KOMPG:C4QWD1|C4QWD1\_KOMPG:C4R778|C4R778\_KOMPG:C4QW16|C4QW16\_KOMPG:C4R975|C4R975\_KOMPG:C4R7U8|C4R7U8\_KOMPG:C4QYI8|C4QYI8\_KOMPG:C4QVM3|C4QVM3\_KOMPG:C4R2F4|C4R2F4\_KOMPG:C4QZP4|C4QZP4\_KOMPG |  |  | DB Search |
| MGR | 20.83 | 362.1736 | 3 | 6.82 | 363.1825 | 3.30 | 1420 | 1.5e3 | 1 | 1 | C4R8U2|C4R8U2\_KOMPG:C4R237|C4R237\_KOMPG:C4QZL2|C4QZL2\_KOMPG:C4R3R9|C4R3R9\_KOMPG:C4QVU5|C4QVU5\_KOMPG:C4R202|C4R202\_KOMPG:C4QVY0|C4QVY0\_KOMPG:C4R1J1|C4R1J1\_KOMPG:C4QVZ4|C4QVZ4\_KOMPG:C4QVH6|C4QVH6\_KOMPG:C4QWW6|C4QWW6\_KOMPG:C4R4S7|C4R4S7\_KOMPG:C4R1M5|C4R1M5\_KOMPG:C4R300|C4R300\_KOMPG:C4R588|C4R588\_KOMPG:C4QX76|C4QX76\_KOMPG:C4QVI5|C4QVI5\_KOMPG:C4QXT5|C4QXT5\_KOMPG:C4R4C6|C4R4C6\_KOMPG:C4QV14|C4QV14\_KOMPG:C4QVK9|C4QVK9\_KOMPG:C4QWH0|C4QWH0\_KOMPG:C4R1R2|C4R1R2\_KOMPG:C4R6L8|C4R6L8\_KOMPG:C4QWH4|C4QWH4\_KOMPG:C4R6V1|C4R6V1\_KOMPG:C4R0Q1|C4R0Q1\_KOMPG:C4R874|C4R874\_KOMPG:C4R8B7|C4R8B7\_KOMPG:C4R8L3|C4R8L3\_KOMPG:C4R3F7|C4R3F7\_KOMPG:C4QWQ4|C4QWQ4\_KOMPG:C4R115|C4R115\_KOMPG:C4QYM6|C4QYM6\_KOMPG:C4R571|C4R571\_KOMPG:C4R2L0|PEX5\_KOMPG:C4R4H5|C4R4H5\_KOMPG:Q92448|PFKA1\_KOMPG:C4R145|C4R145\_KOMPG:C4QZU2|C4QZU2\_KOMPG:C4R8V8|C4R8V8\_KOMPG:C4QYB9|C4QYB9\_KOMPG:C4QX11|RGI1\_KOMPG:C4R0B4|C4R0B4\_KOMPG:C4R304|C4R304\_KOMPG:C4R315|C4R315\_KOMPG:C4QVY7|C4QVY7\_KOMPG:C4QZQ5|C4QZQ5\_KOMPG:C4QW23|C4QW23\_KOMPG:C4R1G6|C4R1G6\_KOMPG:C4R922|C4R922\_KOMPG:C4QZK9|C4QZK9\_KOMPG:C4QXT1|C4QXT1\_KOMPG:C4QXT4|C4QXT4\_KOMPG:C4R827|C4R827\_KOMPG:C4QWG6|C4QWG6\_KOMPG:C4R6F9|C4R6F9\_KOMPG:C4QZV6|C4QZV6\_KOMPG:C4R2M8|C4R2M8\_KOMPG:C4QX34|C4QX34\_KOMPG:C4R438|C4R438\_KOMPG:C4R7J3|C4R7J3\_KOMPG:C4R1Q3|C4R1Q3\_KOMPG:C4QY13|C4QY13\_KOMPG:C4QW55|C4QW55\_KOMPG:C4R580|C4R580\_KOMPG:C4R7Y4|C4R7Y4\_KOMPG:C4QYN5|C4QYN5\_KOMPG:C4QWL7|C4QWL7\_KOMPG:C4QYT1|C4QYT1\_KOMPG:C4QV48|C4QV48\_KOMPG:C4QX02|C4QX02\_KOMPG:C4QXA5|PFKA2\_KOMPG:C4R2K8|C4R2K8\_KOMPG:C4R469|C4R469\_KOMPG:C4QXY7|C4QXY7\_KOMPG:C4QYR9|C4QYR9\_KOMPG:C4R3W5|C4R3W5\_KOMPG:C4R673|C4R673\_KOMPG:C4R448|C4R448\_KOMPG:C4QZZ2|C4QZZ2\_KOMPG:C4R1W1|C4R1W1\_KOMPG:C4R615|C4R615\_KOMPG:C4QWD8|C4QWD8\_KOMPG:C4QZC0|C4QZC0\_KOMPG:C4R6U5|C4R6U5\_KOMPG:C4R7X3|C4R7X3\_KOMPG:C4QYR7|C4QYR7\_KOMPG:C4R8K3|C4R8K3\_KOMPG:C4R6Z1|C4R6Z1\_KOMPG:C4QX91|C4QX91\_KOMPG:C4QYM3|C4QYM3\_KOMPG |  |  | DB Search |
| SSNK | 20.83 | 434.2125 | 4 | -0.81 | 435.2184 | 2.82 | 1206 | 3.52e2 | 1 | 1 | C4R5A9|C4R5A9\_KOMPG:C4R840|C4R840\_KOMPG:C4R3A1|C4R3A1\_KOMPG:C4QXF3|C4QXF3\_KOMPG:C4R440|C4R440\_KOMPG:C4R106|C4R106\_KOMPG:C4QYL8|C4QYL8\_KOMPG:C4R6J4|C4R6J4\_KOMPG:C4QWY8|AIM36\_KOMPG:C4R8R3|C4R8R3\_KOMPG:C4QYV4|C4QYV4\_KOMPG:C4QXH4|C4QXH4\_KOMPG:C4R5W8|C4R5W8\_KOMPG:C4R902|C4R902\_KOMPG:C4R6Y0|C4R6Y0\_KOMPG:C4R422|C4R422\_KOMPG:C4QVI5|C4QVI5\_KOMPG:C4R344|C4R344\_KOMPG:C4R4C6|C4R4C6\_KOMPG:C4R1T5|C4R1T5\_KOMPG:C4QYC6|C4QYC6\_KOMPG:C4R1U9|C4R1U9\_KOMPG:C4QZ27|C4QZ27\_KOMPG:C4R048|C4R048\_KOMPG:C4R8L3|C4R8L3\_KOMPG:C4QZT4|C4QZT4\_KOMPG:C4QY15|C4QY15\_KOMPG:C4R283|C4R283\_KOMPG:C4QXI9|C4QXI9\_KOMPG:C4QW92|C4QW92\_KOMPG:C4QXD4|C4QXD4\_KOMPG:C4R301|C4R301\_KOMPG:C4QZR4|C4QZR4\_KOMPG:C4QZK5|C4QZK5\_KOMPG:C4R117|C4R117\_KOMPG:C4QV59|C4QV59\_KOMPG:C4R990|C4R990\_KOMPG:C4R667|C4R667\_KOMPG:C4R873|C4R873\_KOMPG:C4R3J1|C4R3J1\_KOMPG:C4QVP8|C4QVP8\_KOMPG:C4QZJ4|C4QZJ4\_KOMPG:C4R925|C4R925\_KOMPG:C4R402|C4R402\_KOMPG:C4R5U2|C4R5U2\_KOMPG:C4R4Y5|C4R4Y5\_KOMPG:C4QYA8|C4QYA8\_KOMPG:C4R0K1|C4R0K1\_KOMPG:C4R2Y0|C4R2Y0\_KOMPG:C4R6F5|C4R6F5\_KOMPG:C4R808|C4R808\_KOMPG:C4R5X4|C4R5X4\_KOMPG:C4QWL3|C4QWL3\_KOMPG:C4QV41|C4QV41\_KOMPG:C4QX57|C4QX57\_KOMPG:C4R0J2|C4R0J2\_KOMPG:C4R228|C4R228\_KOMPG:C4R7A6|C4R7A6\_KOMPG:C4QXE6|C4QXE6\_KOMPG:C4R886|C4R886\_KOMPG:C4R771|C4R771\_KOMPG:C4R446|C4R446\_KOMPG:C4R275|C4R275\_KOMPG:C4R579|C4R579\_KOMPG:C4R8Z1|C4R8Z1\_KOMPG:C4QX20|C4QX20\_KOMPG:C4R240|C4R240\_KOMPG:C4R448|C4R448\_KOMPG:C4R4U3|C4R4U3\_KOMPG:C4R1W7|C4R1W7\_KOMPG:C4R1D5|C4R1D5\_KOMPG:C4R3G2|C4R3G2\_KOMPG:C4R140|C4R140\_KOMPG:C4R1Y8|C4R1Y8\_KOMPG:C4R7U8|C4R7U8\_KOMPG:C4R5N3|C4R5N3\_KOMPG:C4R5D7|C4R5D7\_KOMPG:C4R361|C4R361\_KOMPG:C4R8T3|C4R8T3\_KOMPG:C4QYD5|C4QYD5\_KOMPG:C4R721|C4R721\_KOMPG:C4R3V3|C4R3V3\_KOMPG:C4QUY9|C4QUY9\_KOMPG:C4R3B6|C4R3B6\_KOMPG:C4QWE7|C4QWE7\_KOMPG |  |  | DB Search |
| SIAP | 20.83 | 386.2165 | 4 | -4.65 | 387.221 | 8.35 | 3276 | 4.13e3 | 1 | 1 | C4QW89|C4QW89\_KOMPG:C4QZV5|C4QZV5\_KOMPG:C4R1D2|C4R1D2\_KOMPG:C4R554|C4R554\_KOMPG:C4QVD5|C4QVD5\_KOMPG:C4R6C2|PEX1\_KOMPG:C4QVD4|C4QVD4\_KOMPG:C4R878|C4R878\_KOMPG:C4QW61|C4QW61\_KOMPG:C4R3C5|C4R3C5\_KOMPG:C4R1C6|C4R1C6\_KOMPG:C4R3V4|C4R3V4\_KOMPG:C4R986|C4R986\_KOMPG:C4R2Z3|C4R2Z3\_KOMPG:C4R243|C4R243\_KOMPG:C4QVT5|C4QVT5\_KOMPG:C4R3V5|C4R3V5\_KOMPG:C4QXJ6|C4QXJ6\_KOMPG:C4R5H3|C4R5H3\_KOMPG:C4QX72|C4QX72\_KOMPG:C4R349|C4R349\_KOMPG:C4QVY8|C4QVY8\_KOMPG:C4QY80|C4QY80\_KOMPG:C4R971|C4R971\_KOMPG:C4R101|C4R101\_KOMPG:C4R5X9|C4R5X9\_KOMPG:C4R9A6|C4R9A6\_KOMPG |  |  | DB Search |
| SLAP | 20.83 | 386.2165 | 4 | -4.65 | 387.221 | 8.35 | 3276 | 4.13e3 | 1 | 1 | C4QZT2|C4QZT2\_KOMPG:C4R292|C4R292\_KOMPG:C4R296|C4R296\_KOMPG:C4QW28|C4QW28\_KOMPG:C4R0Z1|C4R0Z1\_KOMPG:C4QVH6|C4QVH6\_KOMPG:C4R3G3|C4R3G3\_KOMPG:C4R5K5|C4R5K5\_KOMPG:C4R0W9|C4R0W9\_KOMPG:C4R6Q7|C4R6Q7\_KOMPG:C4QW51|C4QW51\_KOMPG:C4QYX1|C4QYX1\_KOMPG:C4R303|C4R303\_KOMPG:C4QWT2|C4QWT2\_KOMPG:C4R268|C4R268\_KOMPG:C4QZ12|C4QZ12\_KOMPG:C4QVK5|C4QVK5\_KOMPG:C4R6Y5|C4R6Y5\_KOMPG:C4R301|C4R301\_KOMPG:C4R544|C4R544\_KOMPG:C4QVH8|C4QVH8\_KOMPG:C4R634|C4R634\_KOMPG:C4QZD7|C4QZD7\_KOMPG:C4R3D7|C4R3D7\_KOMPG:C4R4Z2|C4R4Z2\_KOMPG:C4R5C2|C4R5C2\_KOMPG:C4R1L9|C4R1L9\_KOMPG:C4R328|C4R328\_KOMPG:C4R438|C4R438\_KOMPG:C4QXA9|C4QXA9\_KOMPG:C4QW37|C4QW37\_KOMPG:C4R179|C4R179\_KOMPG:C4R8G8|C4R8G8\_KOMPG:C4R7Y7|C4R7Y7\_KOMPG:C4R732|C4R732\_KOMPG:C4QYI7|C4QYI7\_KOMPG:C4R468|C4R468\_KOMPG:C4R5W0|C4R5W0\_KOMPG:C4R6V7|C4R6V7\_KOMPG:C4R5L7|C4R5L7\_KOMPG:C4R771|C4R771\_KOMPG:C4R7Q2|C4R7Q2\_KOMPG:C4QXL9|C4QXL9\_KOMPG:C4R791|C4R791\_KOMPG:C4QXJ7|C4QXJ7\_KOMPG:C4QXA2|C4QXA2\_KOMPG:C4R3W0|C4R3W0\_KOMPG:C4QV91|C4QV91\_KOMPG:C4QZP5|C4QZP5\_KOMPG:C4QWV4|C4QWV4\_KOMPG:C4R4B2|C4R4B2\_KOMPG:C4R0C8|C4R0C8\_KOMPG:C4R0E1|C4R0E1\_KOMPG |  |  | DB Search |
| LDVNPLT | 20.82 | 770.4174 | 7 | -3.05 | 771.4204 | 23.58 | 8592 | 5.92e3 | 1 | 1 | C4QZS3|C4QZS3\_KOMPG |  |  | DB Search |
| SVGVMGDQRT | 20.74 | 1048.4971 | 10 | 2.17 | 525.2557 | 28.12 | 9854 | 0 | 0 | 0 | C4QVI1|C4QVI1\_KOMPG |  |  | DB Search |
| IAR | 20.74 | 358.2328 | 3 | -3.48 | 359.238 | 2.08 | 887 | 2.98e3 | 1 | 1 | C4QYE6|C4QYE6\_KOMPG:C4R217|C4R217\_KOMPG:C4QZ17|C4QZ17\_KOMPG:C4QZ72|C4QZ72\_KOMPG:C4R0S7|C4R0S7\_KOMPG:C4R396|C4R396\_KOMPG:C4R2U1|C4R2U1\_KOMPG:C4QV84|C4QV84\_KOMPG:C4QVZ4|C4QVZ4\_KOMPG:C4R0D8|C4R0D8\_KOMPG:C4R4S7|C4R4S7\_KOMPG:C4R3I7|C4R3I7\_KOMPG:C4R3W9|C4R3W9\_KOMPG:C4QYP0|C4QYP0\_KOMPG:C4R784|C4R784\_KOMPG:C4QZE8|C4QZE8\_KOMPG:C4QX76|C4QX76\_KOMPG:C4R6L7|C4R6L7\_KOMPG:C4R6Z9|C4R6Z9\_KOMPG:C4R4I2|C4R4I2\_KOMPG:C4QVT4|C4QVT4\_KOMPG:C4R912|C4R912\_KOMPG:C4R3Z8|C4R3Z8\_KOMPG:C4R7W9|C4R7W9\_KOMPG:C4R2E9|C4R2E9\_KOMPG:C4R5Q7|C4R5Q7\_KOMPG:C4R679|C4R679\_KOMPG:C4R042|C4R042\_KOMPG:C4QX63|C4QX63\_KOMPG:C4QWM2|C4QWM2\_KOMPG:C4R6E1|C4R6E1\_KOMPG:C4R5K0|C4R5K0\_KOMPG:C4R263|C4R263\_KOMPG:C4QXU5|C4QXU5\_KOMPG:C4QWR1|C4QWR1\_KOMPG:C4R2T3|C4R2T3\_KOMPG:C4R4L4|C4R4L4\_KOMPG:C4QXF9|C4QXF9\_KOMPG:C4QX12|C4QX12\_KOMPG:C4QYQ0|C4QYQ0\_KOMPG:C4QYV2|C4QYV2\_KOMPG:C4QZU4|C4QZU4\_KOMPG:C4R7X7|C4R7X7\_KOMPG:C4QVT0|C4QVT0\_KOMPG:C4R3S8|C4R3S8\_KOMPG:C4R1L2|C4R1L2\_KOMPG:C4R6T7|C4R6T7\_KOMPG:C4QVH4|C4QVH4\_KOMPG:C4R0B6|C4R0B6\_KOMPG:C4QY76|C4QY76\_KOMPG:C4R8S4|C4R8S4\_KOMPG:C4R0F3|C4R0F3\_KOMPG:C4QYU4|C4QYU4\_KOMPG:C4R070|C4R070\_KOMPG:C4R6C7|C4R6C7\_KOMPG:C4QVB8|C4QVB8\_KOMPG:C4R5S1|C4R5S1\_KOMPG:C4QYA8|C4QYA8\_KOMPG:C4QZP7|C4QZP7\_KOMPG:C4R162|C4R162\_KOMPG:C4R3R5|C4R3R5\_KOMPG:C4QWX6|C4QWX6\_KOMPG:C4QYP8|C4QYP8\_KOMPG:C4R8C1|C4R8C1\_KOMPG:C4QVZ3|C4QVZ3\_KOMPG:C4R4A6|C4R4A6\_KOMPG:C4QZY0|C4QZY0\_KOMPG:C4R6W3|C4R6W3\_KOMPG:C4QV45|C4QV45\_KOMPG:C4QYA5|C4QYA5\_KOMPG:C4R4A2|C4R4A2\_KOMPG:C4R1A0|C4R1A0\_KOMPG:C4R516|C4R516\_KOMPG:C4QWC1|C4QWC1\_KOMPG:C4QYX6|C4QYX6\_KOMPG:C4QX40|C4QX40\_KOMPG:C4R243|C4R243\_KOMPG:C4R981|C4R981\_KOMPG:C4QVA1|C4QVA1\_KOMPG:C4QXZ9|C4QXZ9\_KOMPG:C4R0L5|C4R0L5\_KOMPG:C4R6N4|C4R6N4\_KOMPG:C4R405|C4R405\_KOMPG |  |  | DB Search |
| LAR | 20.74 | 358.2328 | 3 | -3.48 | 359.238 | 2.08 | 887 | 2.98e3 | 1 | 1 | C4QYY5|C4QYY5\_KOMPG:C4R8H4|C4R8H4\_KOMPG:C4R6K9|C4R6K9\_KOMPG:C4R142|C4R142\_KOMPG:C4QX06|C4QX06\_KOMPG:C4QWX3|C4QWX3\_KOMPG:C4R7Q3|C4R7Q3\_KOMPG:C4R3B2|C4R3B2\_KOMPG:C4R0F2|C4R0F2\_KOMPG:C4R6M3|C4R6M3\_KOMPG:C4R181|C4R181\_KOMPG:C4R1D0|C4R1D0\_KOMPG:C4R1P8|C4R1P8\_KOMPG:C4R8B9|C4R8B9\_KOMPG:C4R110|C4R110\_KOMPG:C4QY05|C4QY05\_KOMPG:C4R6B2|C4R6B2\_KOMPG:C4R0I5|C4R0I5\_KOMPG:C4R4D9|C4R4D9\_KOMPG:C4QWI9|C4QWI9\_KOMPG:C4R490|C4R490\_KOMPG:C4QXM1|C4QXM1\_KOMPG:C4QX98|C4QX98\_KOMPG:C4QV42|C4QV42\_KOMPG:C4QY48|C4QY48\_KOMPG:C4R5N8|C4R5N8\_KOMPG:C4R7E6|C4R7E6\_KOMPG:C4R5J2|C4R5J2\_KOMPG:C4R3L8|C4R3L8\_KOMPG:C4R7A4|C4R7A4\_KOMPG:C4QWZ5|C4QWZ5\_KOMPG:C4R571|C4R571\_KOMPG:C4R4J8|C4R4J8\_KOMPG:C4QWH2|C4QWH2\_KOMPG:C4QXN2|MIC60\_KOMPG:C4R161|C4R161\_KOMPG:C4R1A9|C4R1A9\_KOMPG:C4R1S8|C4R1S8\_KOMPG:C4R341|C4R341\_KOMPG:C4R8B6|C4R8B6\_KOMPG:C4R2R5|C4R2R5\_KOMPG:C4QV89|C4QV89\_KOMPG:C4R310|C4R310\_KOMPG:C4R7V7|C4R7V7\_KOMPG:C4QX18|C4QX18\_KOMPG:C4QV66|C4QV66\_KOMPG:C4R438|C4R438\_KOMPG:C4R1K1|C4R1K1\_KOMPG:C4QXA9|C4QXA9\_KOMPG:C4R4Z1|C4R4Z1\_KOMPG:C4QVM1|C4QVM1\_KOMPG:C4QZC8|C4QZC8\_KOMPG:C4R093|C4R093\_KOMPG:C4R996|C4R996\_KOMPG:C4R172|C4R172\_KOMPG:C4R0J7|C4R0J7\_KOMPG:C4QYW2|C4QYW2\_KOMPG:C4R4V7|C4R4V7\_KOMPG:C4R3W1|C4R3W1\_KOMPG:C4QYU7|C4QYU7\_KOMPG:C4R275|C4R275\_KOMPG:C4R4Z8|C4R4Z8\_KOMPG:C4QVT9|C4QVT9\_KOMPG:C4R7R8|C4R7R8\_KOMPG:C4R7C8|C4R7C8\_KOMPG:C4QZA7|C4QZA7\_KOMPG:C4R1R6|C4R1R6\_KOMPG:C4R0Z6|C4R0Z6\_KOMPG:C4R5I8|C4R5I8\_KOMPG:C4R8Z4|C4R8Z4\_KOMPG:C4QZB4|C4QZB4\_KOMPG:C4R095|C4R095\_KOMPG:C4QVN7|C4QVN7\_KOMPG:C4R8D9|C4R8D9\_KOMPG:C4R3V3|C4R3V3\_KOMPG:C4R462|C4R462\_KOMPG:C4R7G1|C4R7G1\_KOMPG:C4R4M3|C4R4M3\_KOMPG:C4R8W0|C4R8W0\_KOMPG:C4QY38|C4QY38\_KOMPG:C4QWI8|C4QWI8\_KOMPG:C4R5F5|C4R5F5\_KOMPG:C4QXX5|C4QXX5\_KOMPG:C4R972|C4R972\_KOMPG:C4R787|C4R787\_KOMPG:C4QYU9|C4QYU9\_KOMPG:C4R7A8|C4R7A8\_KOMPG:C4R751|C4R751\_KOMPG:C4R695|C4R695\_KOMPG:C4R0U8|C4R0U8\_KOMPG:C4QWA3|C4QWA3\_KOMPG:C4R1C0|C4R1C0\_KOMPG:C4R463|C4R463\_KOMPG:C4QZK4|C4QZK4\_KOMPG:C4R5H5|C4R5H5\_KOMPG:C4R5M5|C4R5M5\_KOMPG:C4R7U9|C4R7U9\_KOMPG:C4R0S5|C4R0S5\_KOMPG:C4R5J0|C4R5J0\_KOMPG:C4R069|C4R069\_KOMPG:C4QYT0|C4QYT0\_KOMPG:C4R717|C4R717\_KOMPG:P04842|ALOX1\_KOMPG:C4R3T0|C4R3T0\_KOMPG:C4R349|C4R349\_KOMPG:C4R702|ALOX2\_KOMPG:C4QW00|C4QW00\_KOMPG:C4R4N6|C4R4N6\_KOMPG:C4R8G0|C4R8G0\_KOMPG:C4QVT3|C4QVT3\_KOMPG:C4R6S3|C4R6S3\_KOMPG:C4QVZ2|C4QVZ2\_KOMPG:C4R904|C4R904\_KOMPG:C4QW84|C4QW84\_KOMPG:C4R2V3|C4R2V3\_KOMPG:C4QZ69|C4QZ69\_KOMPG:C4R8D8|C4R8D8\_KOMPG:C4QVD4|C4QVD4\_KOMPG:C4R5L2|C4R5L2\_KOMPG:C4R5X4|C4R5X4\_KOMPG:C4R1Q3|C4R1Q3\_KOMPG:C4R5T3|C4R5T3\_KOMPG:C4R508|C4R508\_KOMPG:C4QVJ4|C4QVJ4\_KOMPG:C4R7H2|C4R7H2\_KOMPG:C4R5F7|C4R5F7\_KOMPG:C4R0R8|C4R0R8\_KOMPG:C4R1F2|C4R1F2\_KOMPG:C4R984|C4R984\_KOMPG:C4QX02|C4QX02\_KOMPG:C4QW27|C4QW27\_KOMPG:C4R370|C4R370\_KOMPG:C4R2I6|C4R2I6\_KOMPG:C4R4N7|C4R4N7\_KOMPG:C4QVB5|C4QVB5\_KOMPG:C4QV64|C4QV64\_KOMPG:C4QZF2|C4QZF2\_KOMPG:C4QVS5|C4QVS5\_KOMPG:C4QY10|C4QY10\_KOMPG:C4QWS9|C4QWS9\_KOMPG:C4QXK5|C4QXK5\_KOMPG:C4QXS6|C4QXS6\_KOMPG:C4R5H2|C4R5H2\_KOMPG:C4R749|C4R749\_KOMPG:C4R855|C4R855\_KOMPG:C4R4G3|C4R4G3\_KOMPG:C4QV16|C4QV16\_KOMPG:C4R176|C4R176\_KOMPG |  |  | DB Search |
| TAEP | 20.73 | 416.1907 | 4 | -0.83 | 417.1966 | 2.96 | 1139 | 5.35e3 | 1 | 1 | C4QZN4|C4QZN4\_KOMPG:C4QXW3|C4QXW3\_KOMPG:C4R6X8|C4R6X8\_KOMPG:C4R394|C4R394\_KOMPG:C4QVY0|C4QVY0\_KOMPG:C4QZ96|C4QZ96\_KOMPG:C4R281|C4R281\_KOMPG:C4R4H0|C4R4H0\_KOMPG:C4R5U9|C4R5U9\_KOMPG:C4QV80|C4QV80\_KOMPG:C4R770|C4R770\_KOMPG:C4R0J8|C4R0J8\_KOMPG:C4QZ19|C4QZ19\_KOMPG:C4R3T4|C4R3T4\_KOMPG:C4QZW9|C4QZW9\_KOMPG:C4QX05|C4QX05\_KOMPG:C4R3A3|C4R3A3\_KOMPG:C4QZH9|C4QZH9\_KOMPG:C4QVA7|C4QVA7\_KOMPG:C4R5H5|C4R5H5\_KOMPG |  |  | DB Search |
| QEVP | 20.72 | 471.2329 | 4 | -6.59 | 472.2359 | 8.35 | 3123 | 1.8e4 | 1 | 1 | C4QYF4|C4QYF4\_KOMPG:C4QVF4|C4QVF4\_KOMPG:C4R5L9|C4R5L9\_KOMPG:C4QV25|C4QV25\_KOMPG:C4QZ43|C4QZ43\_KOMPG:C4QW84|C4QW84\_KOMPG:C4R0N1|C4R0N1\_KOMPG:C4QVZ0|C4QVZ0\_KOMPG:C4R0T3|C4R0T3\_KOMPG:C4QXV7|C4QXV7\_KOMPG:C4R828|C4R828\_KOMPG:C4QWR1|C4QWR1\_KOMPG:C4QZZ6|C4QZZ6\_KOMPG:C4R3W2|C4R3W2\_KOMPG:C4R1V4|C4R1V4\_KOMPG:C4R3M2|C4R3M2\_KOMPG:C4R6Z1|C4R6Z1\_KOMPG:C4QXS3|C4QXS3\_KOMPG:C4R1M0|C4R1M0\_KOMPG:C4QVH4|C4QVH4\_KOMPG |  |  | DB Search |
| TAPQ | 20.69 | 415.2067 | 4 | 3.43 | 416.2144 | 8.30 | 3151 | 1.09e3 | 1 | 1 | C4R198|C4R198\_KOMPG:C4R3C5|C4R3C5\_KOMPG:C4R370|C4R370\_KOMPG:C4R4C2|C4R4C2\_KOMPG:C4QVX5|C4QVX5\_KOMPG:C4R6K2|C4R6K2\_KOMPG:C4QZT4|C4QZT4\_KOMPG:C4R3G4|C4R3G4\_KOMPG:C4R2N1|C4R2N1\_KOMPG:C4QY95|C4QY95\_KOMPG:C4QV65|C4QV65\_KOMPG:C4R4F9|C4R4F9\_KOMPG:C4QXM9|C4QXM9\_KOMPG:C4R8N0|C4R8N0\_KOMPG:C4R898|C4R898\_KOMPG |  |  | DB Search |
| YTNP | 20.69 | 493.2173 | 4 | 5.11 | 494.2258 | 6.94 | 2648 | 5.65e2 | 1 | 1 | C4R6W3|C4R6W3\_KOMPG:C4R5K5|C4R5K5\_KOMPG:C4QWA9|C4QWA9\_KOMPG:C4R7J9|C4R7J9\_KOMPG:C4R832|C4R832\_KOMPG:C4QWD7|C4QWD7\_KOMPG:C4R6D1|C4R6D1\_KOMPG:C4R1H3|C4R1H3\_KOMPG:C4QX13|C4QX13\_KOMPG |  |  | DB Search |
| YADP | 20.67 | 464.1907 | 4 | 1.09 | 465.1973 | 6.04 | 2345 | 3.62e3 | 1 | 1 | C4R036|C4R036\_KOMPG:C4QY53|C4QY53\_KOMPG:C4R8X6|C4R8X6\_KOMPG:C4QXK3|C4QXK3\_KOMPG:C4QW84|C4QW84\_KOMPG:C4QXL7|C4QXL7\_KOMPG:C4R521|C4R521\_KOMPG:C4R991|C4R991\_KOMPG:C4R8E3|C4R8E3\_KOMPG:C4QYU5|C4QYU5\_KOMPG |  |  | DB Search |
| AAVE | 20.66 | 388.1958 | 4 | -4.49 | 389.2003 | 2.65 | 1124 | 0 | 0 | 0 | C4R6B4|C4R6B4\_KOMPG:C4R6K9|C4R6K9\_KOMPG:C4R8X6|C4R8X6\_KOMPG:C4R339|C4R339\_KOMPG:C4QXV4|C4QXV4\_KOMPG:C4R7V7|C4R7V7\_KOMPG:C4R0J1|C4R0J1\_KOMPG:C4R748|C4R748\_KOMPG:C4QVI5|C4QVI5\_KOMPG:C4R2A4|C4R2A4\_KOMPG:C4R708|C4R708\_KOMPG:C4R7I3|C4R7I3\_KOMPG:C4QXL7|C4QXL7\_KOMPG:C4R2X1|C4R2X1\_KOMPG:C4QV05|C4QV05\_KOMPG:C4R723|C4R723\_KOMPG:C4QYR3|C4QYR3\_KOMPG:C4QVP5|C4QVP5\_KOMPG:C4R3C5|C4R3C5\_KOMPG:C4R7H0|C4R7H0\_KOMPG:C4QVH5|C4QVH5\_KOMPG:C4QWC1|C4QWC1\_KOMPG:C4QX13|C4QX13\_KOMPG:C4QY03|C4QY03\_KOMPG:C4QWD1|C4QWD1\_KOMPG:C4R608|C4R608\_KOMPG:C4R2A0|C4R2A0\_KOMPG:C4R2R0|C4R2R0\_KOMPG:C4QYY1|C4QYY1\_KOMPG:C4QW19|C4QW19\_KOMPG:C4QZL7|C4QZL7\_KOMPG:C4R5W1|C4R5W1\_KOMPG:C4R8N0|C4R8N0\_KOMPG |  |  | DB Search |
| VGEIQ | 20.66 | 544.2856 | 5 | -2.5 | 545.2902 | 11.22 | 4464 | 3.37e3 | 1 | 1 | C4R0J1|C4R0J1\_KOMPG:C4R9D4|C4R9D4\_KOMPG:C4R3T5|C4R3T5\_KOMPG:C4R4V3|C4R4V3\_KOMPG:C4R710|C4R710\_KOMPG |  |  | DB Search |
| VGELQ | 20.66 | 544.2856 | 5 | -2.5 | 545.2902 | 11.22 | 4464 | 3.37e3 | 1 | 1 | C4R5B4|C4R5B4\_KOMPG:C4R2B0|C4R2B0\_KOMPG:C4R8S7|C4R8S7\_KOMPG |  |  | DB Search |
| PEF | 20.63 | 391.1743 | 3 | 0.03 | 392.1806 | 4.06 | 1653 | 5.45e3 | 1 | 1 | C4R8S8|C4R8S8\_KOMPG:C4R2H1|C4R2H1\_KOMPG:C4QX07|C4QX07\_KOMPG:C4QXP6|C4QXP6\_KOMPG:C4R1D2|C4R1D2\_KOMPG:C4R7Z0|C4R7Z0\_KOMPG:C4R1S0|C4R1S0\_KOMPG:C4QZA1|C4QZA1\_KOMPG:C4R4N4|C4R4N4\_KOMPG:C4R6C5|C4R6C5\_KOMPG:C4QWW6|C4QWW6\_KOMPG:C4R7C4|C4R7C4\_KOMPG:C4QZE8|C4QZE8\_KOMPG:C4R588|C4R588\_KOMPG:C4R1P8|C4R1P8\_KOMPG:C4R344|C4R344\_KOMPG:C4QVE8|C4QVE8\_KOMPG:C4QW91|C4QW91\_KOMPG:C4QXZ6|C4QXZ6\_KOMPG:C4QWI9|C4QWI9\_KOMPG:C4R768|C4R768\_KOMPG:C4R042|C4R042\_KOMPG:C4QXW2|C4QXW2\_KOMPG:C4R1Q7|C4R1Q7\_KOMPG:C4R4Q2|C4R4Q2\_KOMPG:C4R5X8|C4R5X8\_KOMPG:C4QYN9|C4QYN9\_KOMPG:C4R8V8|C4R8V8\_KOMPG:C4QZQ6|C4QZQ6\_KOMPG:C4R8P9|C4R8P9\_KOMPG:C4QW21|C4QW21\_KOMPG:C4QXV8|C4QXV8\_KOMPG:C4R012|C4R012\_KOMPG:C4R9E2|C4R9E2\_KOMPG:C4QWR3|C4QWR3\_KOMPG:C4QXM3|C4QXM3\_KOMPG:C4R6Y7|C4R6Y7\_KOMPG:C4R0A5|C4R0A5\_KOMPG:C4QYZ3|C4QYZ3\_KOMPG:C4QVY2|C4QVY2\_KOMPG:C4QV89|C4QV89\_KOMPG:C4R4Y5|C4R4Y5\_KOMPG:C4R5G8|C4R5G8\_KOMPG:C4QVL3|C4QVL3\_KOMPG:C4QW64|C4QW64\_KOMPG:C4QWT6|C4QWT6\_KOMPG:C4QZC1|C4QZC1\_KOMPG:C4QVI9|C4QVI9\_KOMPG:C4R162|C4R162\_KOMPG:C4R517|C4R517\_KOMPG:C4R7J3|C4R7J3\_KOMPG:C4QZ19|C4QZ19\_KOMPG:C4QXA9|C4QXA9\_KOMPG:C4R340|C4R340\_KOMPG:C4R8G8|C4R8G8\_KOMPG:C4R4A6|C4R4A6\_KOMPG:C4R6G5|C4R6G5\_KOMPG:C4R821|C4R821\_KOMPG:C4QZF9|C4QZF9\_KOMPG:C4R446|C4R446\_KOMPG:C4R3W1|C4R3W1\_KOMPG:C4QYX6|C4QYX6\_KOMPG:C4R621|C4R621\_KOMPG:C4QZ34|C4QZ34\_KOMPG:C4R356|C4R356\_KOMPG:C4R719|C4R719\_KOMPG:C4R1F0|C4R1F0\_KOMPG:C4QWD1|C4QWD1\_KOMPG:C4R773|C4R773\_KOMPG:C4R1S3|C4R1S3\_KOMPG:C4R3U7|C4R3U7\_KOMPG:C4R385|C4R385\_KOMPG:C4R2F4|C4R2F4\_KOMPG:C4R3H2|C4R3H2\_KOMPG:C4R0L5|C4R0L5\_KOMPG:C4QZT6|C4QZT6\_KOMPG:C4R8P5|C4R8P5\_KOMPG:C4R2V8|C4R2V8\_KOMPG:C4R0V4|C4R0V4\_KOMPG:C4QVD5|C4QVD5\_KOMPG:C4R4V3|C4R4V3\_KOMPG:C4R8N7|C4R8N7\_KOMPG:C4QYL3|C4QYL3\_KOMPG:C4QZF8|C4QZF8\_KOMPG:C4R568|C4R568\_KOMPG:C4R535|C4R535\_KOMPG:C4R3N6|C4R3N6\_KOMPG:C4R8I4|C4R8I4\_KOMPG:C4R534|C4R534\_KOMPG:C4R5W3|C4R5W3\_KOMPG:C4QXC0|C4QXC0\_KOMPG:C4R9C2|C4R9C2\_KOMPG:C4QYC6|C4QYC6\_KOMPG:C4QWH0|C4QWH0\_KOMPG:C4R2P9|C4R2P9\_KOMPG:C4R034|C4R034\_KOMPG:C4R6T8|C4R6T8\_KOMPG:C4R5Z7|C4R5Z7\_KOMPG:C4QYD3|C4QYD3\_KOMPG:C4R4A0|C4R4A0\_KOMPG:C4R5A5|C4R5A5\_KOMPG:C4QYY1|C4QYY1\_KOMPG:C4R7G6|C4R7G6\_KOMPG:C4R2U9|C4R2U9\_KOMPG:C4R7X7|C4R7X7\_KOMPG:C4R9D7|C4R9D7\_KOMPG:C4R5I2|C4R5I2\_KOMPG:C4R6T2|C4R6T2\_KOMPG:C4R8M6|C4R8M6\_KOMPG:C4R8K6|C4R8K6\_KOMPG:C4R7W8|C4R7W8\_KOMPG:C4QZ69|C4QZ69\_KOMPG:C4R5L2|C4R5L2\_KOMPG:C4R4D1|C4R4D1\_KOMPG:C4R2K2|C4R2K2\_KOMPG:C4R0Q9|C4R0Q9\_KOMPG:C4R1Q3|C4R1Q3\_KOMPG:C4R3R5|C4R3R5\_KOMPG:C4R6Q6|C4R6Q6\_KOMPG:C4QYN5|C4QYN5\_KOMPG:C4R0R8|C4R0R8\_KOMPG:C4R1F2|C4R1F2\_KOMPG:C4R0Q0|C4R0Q0\_KOMPG:C4R0X6|C4R0X6\_KOMPG:C4R4A2|C4R4A2\_KOMPG:C4QY27|C4QY27\_KOMPG:C4QWC1|C4QWC1\_KOMPG:C4QX81|C4QX81\_KOMPG:C4R7D3|C4R7D3\_KOMPG:C4QYX7|C4QYX7\_KOMPG:C4QVM5|C4QVM5\_KOMPG:C4QV64|C4QV64\_KOMPG:C4R0S0|C4R0S0\_KOMPG:C4QVS5|C4QVS5\_KOMPG:C4R065|C4R065\_KOMPG:C4QVA1|C4QVA1\_KOMPG:C4QWS9|C4QWS9\_KOMPG:C4QYR5|C4QYR5\_KOMPG:C4R5W9|C4R5W9\_KOMPG:C4R6E8|C4R6E8\_KOMPG |  |  | DB Search |
| MVHA | 20.62 | 456.2155 | 4 | 1.6 | 457.2224 | 10.81 | 4262 | 0 | 0 | 0 | C4QZ69|C4QZ69\_KOMPG:C4R8M1|C4R8M1\_KOMPG |  |  | DB Search |
| QQPPQQ | 20.62 | 724.3504 | 6 | -4.61 | 725.3525 | 3.38 | 1468 | 0 | 0 | 0 | C4QXL2|C4QXL2\_KOMPG |  |  | DB Search |
| VPVE | 20.6 | 442.2427 | 4 | -5.67 | 443.2464 | 7.79 | 2913 | 1.07e3 | 1 | 1 | C4R2M0|C4R2M0\_KOMPG:C4QYW7|C4QYW7\_KOMPG:C4QZ59|C4QZ59\_KOMPG:C4QW03|C4QW03\_KOMPG:C4R1G8|C4R1G8\_KOMPG:C4R4C8|C4R4C8\_KOMPG:C4QXI5|C4QXI5\_KOMPG:C4R0Q8|C4R0Q8\_KOMPG:C4QWE8|C4QWE8\_KOMPG:C4R1T5|C4R1T5\_KOMPG:C4R471|C4R471\_KOMPG:C4R4A2|C4R4A2\_KOMPG:C4R123|C4R123\_KOMPG:C4R2B5|C4R2B5\_KOMPG:C4R3R7|C4R3R7\_KOMPG:C4R7W9|C4R7W9\_KOMPG:C4R3C5|C4R3C5\_KOMPG:C4R579|C4R579\_KOMPG:C4R1C2|C4R1C2\_KOMPG:C4QXS7|C4QXS7\_KOMPG:C4QYX5|C4QYX5\_KOMPG:C4QX51|C4QX51\_KOMPG:C4R941|GLG\_KOMPG:C4QWM6|C4QWM6\_KOMPG:C4R701|C4R701\_KOMPG:C4R2Z9|C4R2Z9\_KOMPG:C4QW70|C4QW70\_KOMPG:C4R4M5|C4R4M5\_KOMPG |  |  | DB Search |
| HVFT | 20.6 | 502.254 | 4 | 6.47 | 503.2632 | 14.60 | 5950 | 1.04e3 | 1 | 1 | C4R0D8|C4R0D8\_KOMPG:C4R0G8|C4R0G8\_KOMPG:C4R4S5|C4R4S5\_KOMPG:C4QX73|C4QX73\_KOMPG:C4R7T4|C4R7T4\_KOMPG:C4QXX3|C4QXX3\_KOMPG:C4QVW3|C4QVW3\_KOMPG:C4R543|C4R543\_KOMPG |  |  | DB Search |
| RCI | 20.6 | 390.2049 | 3 | -4.41 | 391.2095 | 8.08 | 3003 | 0 | 0 | 0 | C4R2Q7|C4R2Q7\_KOMPG:C4R1B3|C4R1B3\_KOMPG:C4R208|C4R208\_KOMPG:C4R0Z1|C4R0Z1\_KOMPG:C4R214|C4R214\_KOMPG:C4R8F2|C4R8F2\_KOMPG:C4R0M4|C4R0M4\_KOMPG:C4QZZ8|C4QZZ8\_KOMPG:C4R2Q3|C4R2Q3\_KOMPG:C4R003|C4R003\_KOMPG:C4R5Q8|C4R5Q8\_KOMPG:C4R930|C4R930\_KOMPG:C4R4I1|C4R4I1\_KOMPG:C4QVL9|C4QVL9\_KOMPG:C4R0I5|C4R0I5\_KOMPG:C4QYX9|C4QYX9\_KOMPG:C4R6E2|C4R6E2\_KOMPG:C4R2I0|C4R2I0\_KOMPG:C4QYW8|C4QYW8\_KOMPG:C4R5D1|C4R5D1\_KOMPG:C4R829|C4R829\_KOMPG:C4R1X0|C4R1X0\_KOMPG:C4R394|C4R394\_KOMPG:C4QWS1|C4QWS1\_KOMPG:C4QZP0|C4QZP0\_KOMPG:C4R155|C4R155\_KOMPG:C4R7J5|C4R7J5\_KOMPG:C4QW92|C4QW92\_KOMPG:C4R5Z5|C4R5Z5\_KOMPG:C4QY37|C4QY37\_KOMPG:C4R689|C4R689\_KOMPG:C4R0E2|C4R0E2\_KOMPG:C4R9A4|C4R9A4\_KOMPG:C4R6T9|C4R6T9\_KOMPG:C4R4H9|C4R4H9\_KOMPG:C4R0A8|C4R0A8\_KOMPG:C4R232|C4R232\_KOMPG:C4QVL3|C4QVL3\_KOMPG:C4R1K8|ERT1\_KOMPG:C4R4I7|C4R4I7\_KOMPG:C4R194|C4R194\_KOMPG:C4R7K8|C4R7K8\_KOMPG:C4R7B8|C4R7B8\_KOMPG:C4R4N2|C4R4N2\_KOMPG:C4QWD1|C4QWD1\_KOMPG:C4R7X3|C4R7X3\_KOMPG:C4QV37|C4QV37\_KOMPG:C4R0W6|C4R0W6\_KOMPG:C4R818|C4R818\_KOMPG:C4R0J3|C4R0J3\_KOMPG:C4R6E7|C4R6E7\_KOMPG:C4QWN2|C4QWN2\_KOMPG:C4QVM2|C4QVM2\_KOMPG:C4R5S6|C4R5S6\_KOMPG:C4R7Q0|C4R7Q0\_KOMPG:C4QVJ3|C4QVJ3\_KOMPG:C4R6X0|C4R6X0\_KOMPG:C4R5A2|C4R5A2\_KOMPG:Q9Y751|ATG26\_KOMPG:C4R5P0|C4R5P0\_KOMPG:C4R8N7|C4R8N7\_KOMPG:C4QVR0|C4QVR0\_KOMPG:C4R3I7|C4R3I7\_KOMPG:C4R011|C4R011\_KOMPG:C4QXW6|C4QXW6\_KOMPG:C4R8A8|C4R8A8\_KOMPG:C4QUZ3|C4QUZ3\_KOMPG:C4R2K7|C4R2K7\_KOMPG:C4R1U9|C4R1U9\_KOMPG:C4R1X3|C4R1X3\_KOMPG:C4QWB8|C4QWB8\_KOMPG:C4R0T5|C4R0T5\_KOMPG:C4R3C1|C4R3C1\_KOMPG:C4R986|C4R986\_KOMPG:C4R530|C4R530\_KOMPG:C4QXF6|C4QXF6\_KOMPG:C4QZF5|C4QZF5\_KOMPG:C4QZQ0|C4QZQ0\_KOMPG:C4QZQ7|C4QZQ7\_KOMPG:C4R8G0|C4R8G0\_KOMPG:C4QW72|C4QW72\_KOMPG:C4R1Q0|C4R1Q0\_KOMPG:C4R6V6|C4R6V6\_KOMPG:C4R6M4|C4R6M4\_KOMPG:C4QXH0|C4QXH0\_KOMPG:C4QY61|C4QY61\_KOMPG:C4QZ18|C4QZ18\_KOMPG:C4R7R1|C4R7R1\_KOMPG:C4QZV6|C4QZV6\_KOMPG:C4R6F2|C4R6F2\_KOMPG:C4R0C4|C4R0C4\_KOMPG:C4QZM1|C4QZM1\_KOMPG:C4R222|C4R222\_KOMPG:C4QZ69|C4QZ69\_KOMPG:C4R8L2|C4R8L2\_KOMPG:C4R0Q9|C4R0Q9\_KOMPG:C4R3Y3|C4R3Y3\_KOMPG:C4R4Q9|C4R4Q9\_KOMPG:C4R068|C4R068\_KOMPG:C4R2U8|C4R2U8\_KOMPG:C4QV63|C4QV63\_KOMPG:C4R7Y9|C4R7Y9\_KOMPG:C4R164|C4R164\_KOMPG:C4R1W1|C4R1W1\_KOMPG:C4R7L2|C4R7L2\_KOMPG:C4QZM9|C4QZM9\_KOMPG:C4R2G5|C4R2G5\_KOMPG:C4QW79|C4QW79\_KOMPG:C4QVR1|C4QVR1\_KOMPG:C4R1W5|C4R1W5\_KOMPG:C4R0S3|C4R0S3\_KOMPG |  |  | DB Search |
| RCL | 20.6 | 390.2049 | 3 | -4.41 | 391.2095 | 8.08 | 3003 | 0 | 0 | 0 | C4R5A9|C4R5A9\_KOMPG:C4QVW3|C4QVW3\_KOMPG:C4QWW6|C4QWW6\_KOMPG:C4QWQ1|C4QWQ1\_KOMPG:C4R0L8|C4R0L8\_KOMPG:C4QWP3|C4QWP3\_KOMPG:C4QY84|C4QY84\_KOMPG:C4R5L4|C4R5L4\_KOMPG:C4QW94|C4QW94\_KOMPG:C4R084|C4R084\_KOMPG:C4R1T4|C4R1T4\_KOMPG:C4R555|C4R555\_KOMPG:C4R2D9|C4R2D9\_KOMPG:C4R1C3|C4R1C3\_KOMPG:C4R2Z3|C4R2Z3\_KOMPG:C4R5N8|C4R5N8\_KOMPG:C4QZD4|C4QZD4\_KOMPG:C4QVM0|C4QVM0\_KOMPG:C4QZ75|C4QZ75\_KOMPG:C4QV53|C4QV53\_KOMPG:C4R834|C4R834\_KOMPG:C4R5X8|C4R5X8\_KOMPG:C4R4J8|C4R4J8\_KOMPG:C4R3E6|C4R3E6\_KOMPG:C4R4H5|C4R4H5\_KOMPG:C4R0W7|C4R0W7\_KOMPG:C4R4R7|C4R4R7\_KOMPG:C4R1Z5|C4R1Z5\_KOMPG:C4QX71|C4QX71\_KOMPG:C4R366|C4R366\_KOMPG:C4R893|C4R893\_KOMPG:C4R2F9|C4R2F9\_KOMPG:C4QVC9|C4QVC9\_KOMPG:C4R5U7|C4R5U7\_KOMPG:C4QZN7|C4QZN7\_KOMPG:C4R6Y7|C4R6Y7\_KOMPG:C4QVV6|C4QVV6\_KOMPG:C4R586|C4R586\_KOMPG:C4QYI2|C4QYI2\_KOMPG:C4R955|C4R955\_KOMPG:C4QY20|C4QY20\_KOMPG:C4R1M9|C4R1M9\_KOMPG:C4QZ03|C4QZ03\_KOMPG:C4R002|C4R002\_KOMPG:C4R777|C4R777\_KOMPG:C4R3G4|C4R3G4\_KOMPG:C4R223|C4R223\_KOMPG:C4R432|SEY1\_KOMPG:C4R2K0|UTP25\_KOMPG:C4QX32|C4QX32\_KOMPG:C4R7E3|C4R7E3\_KOMPG:C4R4B9|C4R4B9\_KOMPG:C4R3D8|C4R3D8\_KOMPG:C4R6S6|C4R6S6\_KOMPG:C4R808|C4R808\_KOMPG:C4R162|C4R162\_KOMPG:C4R3H1|C4R3H1\_KOMPG:C4QZJ2|C4QZJ2\_KOMPG:C4R8L1|C4R8L1\_KOMPG:C4R6E5|C4R6E5\_KOMPG:C4R4A6|C4R4A6\_KOMPG:C4R1D7|C4R1D7\_KOMPG:C4R325|C4R325\_KOMPG:C4QYA5|C4QYA5\_KOMPG:C4R8G9|C4R8G9\_KOMPG:C4R252|C4R252\_KOMPG:C4R393|C4R393\_KOMPG:C4R900|C4R900\_KOMPG:C4R6M9|C4R6M9\_KOMPG:C4QW35|C4QW35\_KOMPG:C4R3U1|C4R3U1\_KOMPG:C4R4Q4|C4R4Q4\_KOMPG:C4R332|C4R332\_KOMPG:C4R270|C4R270\_KOMPG:C4QYF1|C4QYF1\_KOMPG:C4R2Q6|C4R2Q6\_KOMPG:C4R8T3|C4R8T3\_KOMPG:C4R2W5|C4R2W5\_KOMPG:C4R018|C4R018\_KOMPG:C4R1X8|C4R1X8\_KOMPG:C4R430|C4R430\_KOMPG:C4QWF8|C4QWF8\_KOMPG:C4QW34|C4QW34\_KOMPG:C4R641|C4R641\_KOMPG:C4R5C0|C4R5C0\_KOMPG:C4R2R1|C4R2R1\_KOMPG:C4QZJ3|C4QZJ3\_KOMPG:C4QVV2|C4QVV2\_KOMPG:C4R7D0|C4R7D0\_KOMPG:C4R1U1|C4R1U1\_KOMPG:C4R8D0|C4R8D0\_KOMPG:C4R0B0|C4R0B0\_KOMPG:C4R3D3|C4R3D3\_KOMPG:C4R2L2|C4R2L2\_KOMPG:Q9P4D0|SEC17\_KOMPG:C4QX76|C4QX76\_KOMPG:C4QX61|C4QX61\_KOMPG:C4QZY9|C4QZY9\_KOMPG:C4QZA4|C4QZA4\_KOMPG:C4R5M4|C4R5M4\_KOMPG:C4R4V8|C4R4V8\_KOMPG:C4R6T8|C4R6T8\_KOMPG:C4R2M6|C4R2M6\_KOMPG:C4QV33|C4QV33\_KOMPG:C4R6L8|C4R6L8\_KOMPG:C4QVD3|C4QVD3\_KOMPG:C4R021|C4R021\_KOMPG:C4R3S3|C4R3S3\_KOMPG:C4R866|C4R866\_KOMPG:C4R2N4|C4R2N4\_KOMPG:C4R778|C4R778\_KOMPG:C4R608|C4R608\_KOMPG:C4R0R6|C4R0R6\_KOMPG:C4R4F2|C4R4F2\_KOMPG:C4R6L9|C4R6L9\_KOMPG:C4R1H0|C4R1H0\_KOMPG:C4R7G6|C4R7G6\_KOMPG:C4QWR8|C4QWR8\_KOMPG:C4QW88|C4QW88\_KOMPG:C4R0H5|C4R0H5\_KOMPG:C4QZU4|C4QZU4\_KOMPG:C4R486|C4R486\_KOMPG:C4QXR9|C4QXR9\_KOMPG:C4R851|C4R851\_KOMPG:C4R0Z3|C4R0Z3\_KOMPG:C4QZB2|C4QZB2\_KOMPG:C4R9E8|C4R9E8\_KOMPG:C4R6V9|C4R6V9\_KOMPG:C4R904|C4R904\_KOMPG:C4R2A1|C4R2A1\_KOMPG:C4R659|C4R659\_KOMPG:C4R8H1|C4R8H1\_KOMPG:C4QYP5|C4QYP5\_KOMPG:C4R7J9|C4R7J9\_KOMPG:C4R3R5|C4R3R5\_KOMPG:C4QWX2|C4QWX2\_KOMPG:C4QZV9|C4QZV9\_KOMPG:C4QZN6|C4QZN6\_KOMPG:C4R807|C4R807\_KOMPG:C4QV36|C4QV36\_KOMPG:C4R3P9|C4R3P9\_KOMPG:C4R2Y1|C4R2Y1\_KOMPG:C4QV48|C4QV48\_KOMPG:C4R1U7|C4R1U7\_KOMPG:C4R0F8|C4R0F8\_KOMPG:C4QW97|C4QW97\_KOMPG:C4R388|C4R388\_KOMPG:C4R3I0|C4R3I0\_KOMPG:C4QX81|C4QX81\_KOMPG:C4R8N4|C4R8N4\_KOMPG:C4R1W2|C4R1W2\_KOMPG:C4R0S6|C4R0S6\_KOMPG:C4R353|C4R353\_KOMPG:C4R9D0|C4R9D0\_KOMPG:C4QY39|C4QY39\_KOMPG:C4QXW5|C4QXW5\_KOMPG:C4R4Y3|C4R4Y3\_KOMPG:C4QYK1|C4QYK1\_KOMPG:C4R4J9|C4R4J9\_KOMPG:C4QYN7|C4QYN7\_KOMPG:C4QY64|C4QY64\_KOMPG:C4R360|PSD2\_KOMPG:C4QVR6|C4QVR6\_KOMPG:C4R4P4|C4R4P4\_KOMPG:C4R8N5|C4R8N5\_KOMPG:C4QUY9|C4QUY9\_KOMPG:C4QY68|PEX8\_KOMPG |  |  | DB Search |
| TVGV | 20.58 | 374.2165 | 4 | -4.06 | 375.2213 | 5.20 | 1970 | 6.27e3 | 1 | 1 | C4R1E4|C4R1E4\_KOMPG:Q9Y751|ATG26\_KOMPG:C4R2C2|C4R2C2\_KOMPG:C4R5T1|ATG30\_KOMPG:C4R5S1|C4R5S1\_KOMPG:C4QW59|C4QW59\_KOMPG:C4R5X3|C4R5X3\_KOMPG:C4QXI2|C4QXI2\_KOMPG:C4R5D2|C4R5D2\_KOMPG:C4QWA3|C4QWA3\_KOMPG:C4QX93|C4QX93\_KOMPG:C4R3R3|C4R3R3\_KOMPG:C4QVC7|C4QVC7\_KOMPG:C4R5G3|C4R5G3\_KOMPG:C4QYI7|C4QYI7\_KOMPG:C4R0P2|C4R0P2\_KOMPG:C4R7N6|C4R7N6\_KOMPG:C4R7H0|C4R7H0\_KOMPG:C4QV13|C4QV13\_KOMPG:C4R5X0|C4R5X0\_KOMPG:C4R155|C4R155\_KOMPG:C4R2F5|C4R2F5\_KOMPG:C4R4V5|C4R4V5\_KOMPG:C4R5H0|C4R5H0\_KOMPG:C4QZM9|C4QZM9\_KOMPG:C4R618|C4R618\_KOMPG:C4QZU4|C4QZU4\_KOMPG:C4R701|C4R701\_KOMPG:C4QX91|C4QX91\_KOMPG:C4QYS2|C4QYS2\_KOMPG:C4R4M3|C4R4M3\_KOMPG |  |  | DB Search |
| VSIS | 20.57 | 404.2271 | 4 | -7.08 | 405.2305 | 8.17 | 2961 | 7.47e3 | 1 | 1 | C4R8W0|C4R8W0\_KOMPG:C4QZ28|C4QZ28\_KOMPG:C4R895|C4R895\_KOMPG:C4R9B5|C4R9B5\_KOMPG:C4QWN5|C4QWN5\_KOMPG:C4R5D2|C4R5D2\_KOMPG:C4R7P5|C4R7P5\_KOMPG:C4QWA3|C4QWA3\_KOMPG:C4QWH3|C4QWH3\_KOMPG:C4R788|C4R788\_KOMPG:C4R3M7|C4R3M7\_KOMPG:C4QZX7|C4QZX7\_KOMPG:C4QZR1|C4QZR1\_KOMPG:C4QYR3|C4QYR3\_KOMPG:C4QZ25|ATP25\_KOMPG:C4R0G3|C4R0G3\_KOMPG:C4QYT3|C4QYT3\_KOMPG:C4QW58|C4QW58\_KOMPG:C4R3Y5|C4R3Y5\_KOMPG:C4R8P4|C4R8P4\_KOMPG:C4R4W4|C4R4W4\_KOMPG:C4R016|C4R016\_KOMPG:C4QVU6|C4QVU6\_KOMPG:C4R8Y4|C4R8Y4\_KOMPG:C4R315|C4R315\_KOMPG:C4QZW5|C4QZW5\_KOMPG:C4R955|C4R955\_KOMPG:C4QZ59|C4QZ59\_KOMPG:C4R4K8|C4R4K8\_KOMPG:C4R0V6|C4R0V6\_KOMPG:C4R1T2|C4R1T2\_KOMPG:C4R5X6|C4R5X6\_KOMPG:C4R3R5|C4R3R5\_KOMPG:C4R766|C4R766\_KOMPG:C4QXA9|C4QXA9\_KOMPG:C4QZW9|C4QZW9\_KOMPG:C4R276|C4R276\_KOMPG:C4R8C1|C4R8C1\_KOMPG:C4QV45|C4QV45\_KOMPG:C4QV56|C4QV56\_KOMPG:C4R4K7|C4R4K7\_KOMPG:C4QX00|C4QX00\_KOMPG:C4R288|C4R288\_KOMPG:C4QVL4|C4QVL4\_KOMPG:C4R9A9|C4R9A9\_KOMPG:C4R1Q5|C4R1Q5\_KOMPG:C4R7L0|C4R7L0\_KOMPG:C4R4T5|C4R4T5\_KOMPG:C4R941|GLG\_KOMPG:C4QY10|C4QY10\_KOMPG:C4QZX4|C4QZX4\_KOMPG |  |  | DB Search |
| VSLS | 20.57 | 404.2271 | 4 | -7.08 | 405.2305 | 8.17 | 2961 | 7.47e3 | 1 | 1 | C4R2B1|C4R2B1\_KOMPG:C4QXB3|C4QXB3\_KOMPG:C4R184|C4R184\_KOMPG:C4QWG8|C4QWG8\_KOMPG:C4R357|C4R357\_KOMPG:C4QWH6|C4QWH6\_KOMPG:C4QW59|C4QW59\_KOMPG:C4R8D0|C4R8D0\_KOMPG:C4R003|C4R003\_KOMPG:C4R1D1|C4R1D1\_KOMPG:C4R7D8|C4R7D8\_KOMPG:C4R1P3|C4R1P3\_KOMPG:C4R286|C4R286\_KOMPG:C4R2K7|C4R2K7\_KOMPG:C4QY66|C4QY66\_KOMPG:C4R1T4|C4R1T4\_KOMPG:C4R090|C4R090\_KOMPG:C4R8D2|C4R8D2\_KOMPG:C4QW80|C4QW80\_KOMPG:C4R9B2|C4R9B2\_KOMPG:C4QVZ1|C4QVZ1\_KOMPG:C4R866|C4R866\_KOMPG:C4R1Z2|C4R1Z2\_KOMPG:C4R2E4|C4R2E4\_KOMPG:C4R670|C4R670\_KOMPG:C4QXN2|MIC60\_KOMPG:C4R377|C4R377\_KOMPG:C4R5U4|C4R5U4\_KOMPG:C4R057|C4R057\_KOMPG:C4R4Q3|C4R4Q3\_KOMPG:C4R9C7|C4R9C7\_KOMPG:C4QYU4|C4QYU4\_KOMPG:C4QVV1|C4QVV1\_KOMPG:C4QZ18|C4QZ18\_KOMPG:C4R7J6|C4R7J6\_KOMPG:C4R008|C4R008\_KOMPG:C4R8H1|C4R8H1\_KOMPG:C4R1S7|C4R1S7\_KOMPG:C4R808|C4R808\_KOMPG:C4QZV3|C4QZV3\_KOMPG:C4QZU0|C4QZU0\_KOMPG:C4R6Q6|C4R6Q6\_KOMPG:C4R885|C4R885\_KOMPG:C4QWY4|C4QWY4\_KOMPG:C4R7S3|C4R7S3\_KOMPG:C4R5F7|C4R5F7\_KOMPG:C4R9C1|C4R9C1\_KOMPG:C4QYN6|C4QYN6\_KOMPG:C4R1X6|C4R1X6\_KOMPG:C4R446|C4R446\_KOMPG:C4R1I3|C4R1I3\_KOMPG:C4QZT7|C4QZT7\_KOMPG:C4QY97|C4QY97\_KOMPG:C4R875|C4R875\_KOMPG:C4R3T9|C4R3T9\_KOMPG:C4QZJ7|C4QZJ7\_KOMPG:C4R5H0|C4R5H0\_KOMPG:C4R668|C4R668\_KOMPG:C4R3U7|C4R3U7\_KOMPG:C4R439|C4R439\_KOMPG:C4R2W5|C4R2W5\_KOMPG:C4R045|C4R045\_KOMPG:C4R8N5|C4R8N5\_KOMPG |  |  | DB Search |
| TFR | 20.53 | 422.2278 | 3 | -9.2 | 423.2301 | 22.38 | 8244 | 2.98e1 | 1 | 1 | C4R7F7|C4R7F7\_KOMPG:C4R8N1|C4R8N1\_KOMPG:C4R0Z1|C4R0Z1\_KOMPG:C4QWE6|C4QWE6\_KOMPG:C4R5L8|C4R5L8\_KOMPG:C4R897|C4R897\_KOMPG:C4R4I5|C4R4I5\_KOMPG:C4QWW6|C4QWW6\_KOMPG:C4QWW7|C4QWW7\_KOMPG:C4QX22|C4QX22\_KOMPG:C4R737|C4R737\_KOMPG:C4R350|C4R350\_KOMPG:C4R182|C4R182\_KOMPG:C4QW51|C4QW51\_KOMPG:C4R2A7|C4R2A7\_KOMPG:C4R9C5|C4R9C5\_KOMPG:C4QVF6|C4QVF6\_KOMPG:C4R461|C4R461\_KOMPG:C4QV42|C4QV42\_KOMPG:C4R4P2|C4R4P2\_KOMPG:C4R2S5|C4R2S5\_KOMPG:C4R6F4|C4R6F4\_KOMPG:C4QWS1|C4QWS1\_KOMPG:C4QZI2|C4QZI2\_KOMPG:C4R281|C4R281\_KOMPG:C4R571|C4R571\_KOMPG:C4R7S0|C4R7S0\_KOMPG:C4R2U4|C4R2U4\_KOMPG:C4R189|C4R189\_KOMPG:C4QXD2|C4QXD2\_KOMPG:C4R8Y4|C4R8Y4\_KOMPG:C4R6A7|C4R6A7\_KOMPG:C4QY01|C4QY01\_KOMPG:C4R2W9|C4R2W9\_KOMPG:C4R315|C4R315\_KOMPG:C4R0A8|C4R0A8\_KOMPG:C4QY69|C4QY69\_KOMPG:C4R1N4|C4R1N4\_KOMPG:C4QY85|C4QY85\_KOMPG:C4R232|C4R232\_KOMPG:C4R3G4|C4R3G4\_KOMPG:C4R7V7|C4R7V7\_KOMPG:C4R6D1|C4R6D1\_KOMPG:C4R311|C4R311\_KOMPG:C4R8D5|C4R8D5\_KOMPG:C4QX26|C4QX26\_KOMPG:C4QV28|C4QV28\_KOMPG:C4QYA5|C4QYA5\_KOMPG:C4R393|C4R393\_KOMPG:C4R358|C4R358\_KOMPG:C4R0E5|C4R0E5\_KOMPG:C4R047|C4R047\_KOMPG:C4QX35|C4QX35\_KOMPG:C4R7X3|C4R7X3\_KOMPG:C4R567|C4R567\_KOMPG:C4QZM4|C4QZM4\_KOMPG:C4QZB1|C4QZB1\_KOMPG:C4R462|C4R462\_KOMPG:C4QVQ9|C4QVQ9\_KOMPG:C4R5V1|C4R5V1\_KOMPG:C4QZH4|C4QZH4\_KOMPG:C4R4H1|C4R4H1\_KOMPG:C4R895|C4R895\_KOMPG:C4R5V6|C4R5V6\_KOMPG:C4R4B5|C4R4B5\_KOMPG:C4QYB1|C4QYB1\_KOMPG:C4R566|C4R566\_KOMPG:C4QXZ1|C4QXZ1\_KOMPG:C4R011|C4R011\_KOMPG:C4R2L2|C4R2L2\_KOMPG:C4R8F1|C4R8F1\_KOMPG:C4QYG5|C4QYG5\_KOMPG:C4R3F8|C4R3F8\_KOMPG:C4R4V8|C4R4V8\_KOMPG:C4QVD8|C4QVD8\_KOMPG:C4R924|C4R924\_KOMPG:C4R8P4|C4R8P4\_KOMPG:C4R153|C4R153\_KOMPG:C4R6U9|C4R6U9\_KOMPG:C4R2Q1|C4R2Q1\_KOMPG:C4R257|C4R257\_KOMPG:C4R3E4|C4R3E4\_KOMPG:C4R6G7|C4R6G7\_KOMPG:C4R544|C4R544\_KOMPG:C4R5I2|C4R5I2\_KOMPG:C4R1S2|C4R1S2\_KOMPG:C4R4T4|C4R4T4\_KOMPG:C4R825|C4R825\_KOMPG:C4R851|C4R851\_KOMPG:C4QYX8|C4QYX8\_KOMPG:C4QX89|C4QX89\_KOMPG:C4R1S9|C4R1S9\_KOMPG:C4R704|C4R704\_KOMPG:C4R374|C4R374\_KOMPG:C4R5P8|C4R5P8\_KOMPG:C4QYP5|C4QYP5\_KOMPG:C4R4U5|C4R4U5\_KOMPG:C4R3K5|RTC5\_KOMPG:C4R276|C4R276\_KOMPG:C4R066|C4R066\_KOMPG:C4R2Y5|C4R2Y5\_KOMPG:C4QYA1|C4QYA1\_KOMPG:C4R495|C4R495\_KOMPG:C4QXD6|C4QXD6\_KOMPG:C4R4Y0|C4R4Y0\_KOMPG:C4R370|C4R370\_KOMPG:C4R9A3|C4R9A3\_KOMPG:C4QX90|C4QX90\_KOMPG:C4R472|C4R472\_KOMPG:C4QVA1|C4QVA1\_KOMPG:C4QYK4|C4QYK4\_KOMPG:C4QVM6|C4QVM6\_KOMPG:C4R9D9|C4R9D9\_KOMPG:C4R7J1|C4R7J1\_KOMPG:C4R8N9|C4R8N9\_KOMPG:C4QVT8|C4QVT8\_KOMPG:C4R6R3|C4R6R3\_KOMPG:C4QYB2|C4QYB2\_KOMPG:C4R603|C4R603\_KOMPG:C4QVM4|C4QVM4\_KOMPG:C4QZL0|C4QZL0\_KOMPG:C4QY40|C4QY40\_KOMPG:C4QX65|C4QX65\_KOMPG:C4QVL2|C4QVL2\_KOMPG:C4R3W9|C4R3W9\_KOMPG:C4R3U9|C4R3U9\_KOMPG:C4QVX4|C4QVX4\_KOMPG:C4R3M7|C4R3M7\_KOMPG:C4QYE5|C4QYE5\_KOMPG:C4R8L9|C4R8L9\_KOMPG:C4R742|C4R742\_KOMPG:C4R0U3|C4R0U3\_KOMPG:C4R1C3|C4R1C3\_KOMPG:C4R2Z3|C4R2Z3\_KOMPG:C4QXT6|C4QXT6\_KOMPG:C4QVK3|C4QVK3\_KOMPG:C4R048|C4R048\_KOMPG:C4QZ46|C4QZ46\_KOMPG:C4R2G7|C4R2G7\_KOMPG:C4R4J8|C4R4J8\_KOMPG:C4R3H6|C4R3H6\_KOMPG:C4QY79|C4QY79\_KOMPG:C4R4H5|C4R4H5\_KOMPG:C4QZD9|C4QZD9\_KOMPG:C4R7M1|C4R7M1\_KOMPG:C4QXN2|MIC60\_KOMPG:C4R6V4|C4R6V4\_KOMPG:C4QY00|C4QY00\_KOMPG:C4R8S7|C4R8S7\_KOMPG:C4QZQ6|C4QZQ6\_KOMPG:C4R4Q3|C4R4Q3\_KOMPG:C4QYG4|C4QYG4\_KOMPG:C4R4I3|C4R4I3\_KOMPG:C4QY76|C4QY76\_KOMPG:C4QZW2|C4QZW2\_KOMPG:C4R6B3|C4R6B3\_KOMPG:C4QX96|C4QX96\_KOMPG:C4R505|C4R505\_KOMPG:C4R517|C4R517\_KOMPG:C4R569|C4R569\_KOMPG:C4QYQ8|C4QYQ8\_KOMPG:C4R1M0|C4R1M0\_KOMPG:C4R298|C4R298\_KOMPG:C4R1X6|C4R1X6\_KOMPG:C4R0E3|C4R0E3\_KOMPG:C4R0F4|C4R0F4\_KOMPG:C4QZB5|C4QZB5\_KOMPG:C4R8P2|GATB\_KOMPG:C4QXV5|C4QXV5\_KOMPG:C4QXY8|C4QXY8\_KOMPG:C4R5Y9|C4R5Y9\_KOMPG:C4R5X7|C4R5X7\_KOMPG:C4R3G2|C4R3G2\_KOMPG:C4R861|C4R861\_KOMPG:C4QV37|C4QV37\_KOMPG:C4R3H2|C4R3H2\_KOMPG:C4R3V3|C4R3V3\_KOMPG:C4R797|C4R797\_KOMPG:C4R5R7|C4R5R7\_KOMPG:C4QZ58|C4QZ58\_KOMPG:C4R581|C4R581\_KOMPG:C4R6X0|C4R6X0\_KOMPG:C4QXW1|C4QXW1\_KOMPG:C4R5F5|C4R5F5\_KOMPG:C4R0H0|C4R0H0\_KOMPG:C4R8R3|C4R8R3\_KOMPG:C4R593|C4R593\_KOMPG:C4R8G5|C4R8G5\_KOMPG:C4R7D8|C4R7D8\_KOMPG:C4R1J4|C4R1J4\_KOMPG:C4R846|C4R846\_KOMPG:C4R7N0|C4R7N0\_KOMPG:C4R0H7|C4R0H7\_KOMPG:C4QWS3|C4QWS3\_KOMPG:C4R3V8|C4R3V8\_KOMPG:C4R5Z7|C4R5Z7\_KOMPG:C4QWH4|C4QWH4\_KOMPG:C4R7H0|C4R7H0\_KOMPG:C4QY42|C4QY42\_KOMPG:C4R1G4|C4R1G4\_KOMPG:C4QX47|C4QX47\_KOMPG:C4R2Z8|C4R2Z8\_KOMPG:C4R1M4|C4R1M4\_KOMPG:C4QXP0|C4QXP0\_KOMPG:C4QYB0|C4QYB0\_KOMPG:C4R7G6|C4R7G6\_KOMPG:C4QX23|C4QX23\_KOMPG:C4R0H5|C4R0H5\_KOMPG:C4R057|C4R057\_KOMPG:C4R3B8|C4R3B8\_KOMPG:C4R2Q4|C4R2Q4\_KOMPG:C4R6L2|C4R6L2\_KOMPG:C4QV62|ETT1\_KOMPG:C4R7I2|C4R7I2\_KOMPG:C4R3L3|C4R3L3\_KOMPG:C4R2M8|C4R2M8\_KOMPG:C4QZH7|C4QZH7\_KOMPG:C4R5Q0|C4R5Q0\_KOMPG:C4R4S3|C4R4S3\_KOMPG:C4QXN6|C4QXN6\_KOMPG:C4R8L2|C4R8L2\_KOMPG:C4QZC3|C4QZC3\_KOMPG:C4QX36|C4QX36\_KOMPG:C4R723|C4R723\_KOMPG:C4R7J0|C4R7J0\_KOMPG:C4QVP3|C4QVP3\_KOMPG:C4R3I0|C4R3I0\_KOMPG:C4R6J2|C4R6J2\_KOMPG:C4R3W5|C4R3W5\_KOMPG:C4R4T1|C4R4T1\_KOMPG:C4R7P7|C4R7P7\_KOMPG:C4R1R8|C4R1R8\_KOMPG:C4R735|C4R735\_KOMPG:C4R1D3|C4R1D3\_KOMPG:C4QZT9|C4QZT9\_KOMPG:C4R721|C4R721\_KOMPG:C4QXE9|CHO2\_KOMPG:C4R387|C4R387\_KOMPG:C4QYJ3|C4QYJ3\_KOMPG |  |  | DB Search |
| GPALF | 20.53 | 503.2744 | 5 | -8.95 | 504.2759 | 28.75 | 10039 | 1.84e3 | 1 | 1 | C4R314|C4R314\_KOMPG:C4R6A0|C4R6A0\_KOMPG |  |  | DB Search |
| FAEP | 20.52 | 462.2114 | 4 | -4.02 | 463.2157 | 13.12 | 5209 | 7e3 | 1 | 1 | C4R4B3|CEGT\_KOMPG:C4QYN6|C4QYN6\_KOMPG:C4R4W4|C4R4W4\_KOMPG:C4R3N3|C4R3N3\_KOMPG:C4R8I4|C4R8I4\_KOMPG:C4R9D3|C4R9D3\_KOMPG:C4QV92|C4QV92\_KOMPG |  |  | DB Search |
| YSPR | 20.52 | 521.2598 | 4 | 7.22 | 522.2695 | 8.15 | 2984 | 9.49e2 | 1 | 1 | C4QX27|C4QX27\_KOMPG:C4R000|C4R000\_KOMPG:C4QWS1|C4QWS1\_KOMPG:C4R8D0|C4R8D0\_KOMPG:C4QZ96|C4QZ96\_KOMPG:C4R7Z3|C9MT\_KOMPG:C4R140|C4R140\_KOMPG:C4QZP5|C4QZP5\_KOMPG:C4R6D1|C4R6D1\_KOMPG:C4QZR4|C4QZR4\_KOMPG:C4QYF1|C4QYF1\_KOMPG:C4R1M0|C4R1M0\_KOMPG:C4R5V0|C4R5V0\_KOMPG:C4R3T3|C4R3T3\_KOMPG |  |  | DB Search |
| ISVS | 20.49 | 404.2271 | 4 | -7.08 | 405.2305 | 8.17 | 3035 | 7.47e3 | 1 | 1 | C4R3S7|C4R3S7\_KOMPG:C4R3B8|C4R3B8\_KOMPG:C4R3D7|C4R3D7\_KOMPG:C4R0Z3|C4R0Z3\_KOMPG:C4QXN3|C4QXN3\_KOMPG:C4R9B4|C4R9B4\_KOMPG:C4R5E0|C4R5E0\_KOMPG:C4R6M6|C4R6M6\_KOMPG:C4QVL2|C4QVL2\_KOMPG:C4R568|C4R568\_KOMPG:C4QW55|C4QW55\_KOMPG:C4R6D1|C4R6D1\_KOMPG:C4QY43|C4QY43\_KOMPG:C4R1T5|C4R1T5\_KOMPG:C4QZV8|C4QZV8\_KOMPG:C4R1D7|C4R1D7\_KOMPG:C4QYS5|C4QYS5\_KOMPG:C4R6Z7|C4R6Z7\_KOMPG:C4R1G1|C4R1G1\_KOMPG:C4R8Z1|C4R8Z1\_KOMPG:C4QVY5|C4QVY5\_KOMPG:C4QYL9|C4QYL9\_KOMPG:C4R6F6|C4R6F6\_KOMPG:C4R281|C4R281\_KOMPG:C4R4W5|C4R4W5\_KOMPG:C4R677|C4R677\_KOMPG |  |  | DB Search |
| LSVS | 20.49 | 404.2271 | 4 | -7.08 | 405.2305 | 8.17 | 3035 | 7.47e3 | 1 | 1 | C4R341|C4R341\_KOMPG:C4QZW5|C4QZW5\_KOMPG:C4R479|C4R479\_KOMPG:C4R8S4|C4R8S4\_KOMPG:C4QZ59|C4QZ59\_KOMPG:C4QWB0|C4QWB0\_KOMPG:C4QVN1|C4QVN1\_KOMPG:C4QY57|C4QY57\_KOMPG:C4R5P8|C4R5P8\_KOMPG:C4R5Q0|C4R5Q0\_KOMPG:C4R1S7|C4R1S7\_KOMPG:C4R763|C4R763\_KOMPG:C4R447|C4R447\_KOMPG:C4R510|C4R510\_KOMPG:C4QV41|C4QV41\_KOMPG:C4QX64|C4QX64\_KOMPG:C4QWA3|C4QWA3\_KOMPG:C4R5G4|C4R5G4\_KOMPG:C4QY81|C4QY81\_KOMPG:C4QYY3|C4QYY3\_KOMPG:C4QZ71|C4QZ71\_KOMPG:C4QY04|C4QY04\_KOMPG:C4QVX0|C4QVX0\_KOMPG:C4R288|C4R288\_KOMPG:C4R829|C4R829\_KOMPG:C4R8D2|C4R8D2\_KOMPG:C4QYY0|C4QYY0\_KOMPG:C4R3S4|C4R3S4\_KOMPG:C4R4R6|C4R4R6\_KOMPG:C4R993|C4R993\_KOMPG:C4QWB9|C4QWB9\_KOMPG:C4R3Q3|C4R3Q3\_KOMPG:C4R4C7|C4R4C7\_KOMPG:C4R1X9|C4R1X9\_KOMPG:C4R773|C4R773\_KOMPG:C4QXF9|C4QXF9\_KOMPG:C4R4Q3|C4R4Q3\_KOMPG:C4R1A9|C4R1A9\_KOMPG:C4QZ04|C4QZ04\_KOMPG:C4R3M1|C4R3M1\_KOMPG:C4R2X0|C4R2X0\_KOMPG:C4R8B3|C4R8B3\_KOMPG:C4QZR2|C4QZR2\_KOMPG |  |  | DB Search |
| VSSI | 20.48 | 404.2271 | 4 | -7.08 | 405.2305 | 8.17 | 2993 | 7.47e3 | 1 | 1 | C4R4Y8|C4R4Y8\_KOMPG:C4QZA8|C4QZA8\_KOMPG:C4R8G4|C4R8G4\_KOMPG:C4QVL1|C4QVL1\_KOMPG:C4QZH4|C4QZH4\_KOMPG:C4R2X5|C4R2X5\_KOMPG:C4QXW8|C4QXW8\_KOMPG:C4R5V6|C4R5V6\_KOMPG:C4QVA5|C4QVA5\_KOMPG:C4R4B5|C4R4B5\_KOMPG:C4R8D0|C4R8D0\_KOMPG:C4R6J9|C4R6J9\_KOMPG:C4QV43|C4QV43\_KOMPG:C4R1D1|C4R1D1\_KOMPG:C4QXJ0|C4QXJ0\_KOMPG:C4R3D4|PEX2\_KOMPG:C4R0T1|C4R0T1\_KOMPG:C4QZZ0|C4QZZ0\_KOMPG:C4R382|C4R382\_KOMPG:C4QVC2|C4QVC2\_KOMPG:C4QYH0|C4QYH0\_KOMPG:C4R952|C4R952\_KOMPG:C4QYZ1|C4QYZ1\_KOMPG:C4R767|C4R767\_KOMPG:C4QVX0|C4QVX0\_KOMPG:C4R2L7|C4R2L7\_KOMPG:C4QV42|C4QV42\_KOMPG:C4R7W6|C4R7W6\_KOMPG:C4QVI3|C4QVI3\_KOMPG:C4R5M2|C4R5M2\_KOMPG:C4R094|C4R094\_KOMPG:C4QZI2|C4QZI2\_KOMPG:C4QX47|C4QX47\_KOMPG:C4R474|C4R474\_KOMPG:C4R4J8|C4R4J8\_KOMPG:C4QVR8|C4QVR8\_KOMPG:C4QZF0|C4QZF0\_KOMPG:C4R149|C4R149\_KOMPG:C4R3P6|C4R3P6\_KOMPG:C4QZQ6|C4QZQ6\_KOMPG:C4QZH9|C4QZH9\_KOMPG:C4R3B8|C4R3B8\_KOMPG:C4QVZ2|C4QVZ2\_KOMPG:C4R3G8|C4R3G8\_KOMPG:C4R166|C4R166\_KOMPG:C4QXF7|C4QXF7\_KOMPG:C4R0Z3|C4R0Z3\_KOMPG:C4R8S4|C4R8S4\_KOMPG:C4QZV5|C4QZV5\_KOMPG:C4R1J5|C4R1J5\_KOMPG:C4R1J8|C4R1J8\_KOMPG:C4QZR7|C4QZR7\_KOMPG:C4R0A5|C4R0A5\_KOMPG:C4QVH3|C4QVH3\_KOMPG:C4R141|C4R141\_KOMPG:C4R1L9|C4R1L9\_KOMPG:C4QXB4|C4QXB4\_KOMPG:C4R7Q1|C4R7Q1\_KOMPG:C4R8G6|C4R8G6\_KOMPG:C4QWF2|C4QWF2\_KOMPG:C4R4S3|C4R4S3\_KOMPG:C4R0G8|C4R0G8\_KOMPG:C4R1R4|C4R1R4\_KOMPG:C4R5E4|C4R5E4\_KOMPG:C4R7A9|C4R7A9\_KOMPG:C4R0R5|C4R0R5\_KOMPG:C4R2D2|C4R2D2\_KOMPG:C4QWW2|C4QWW2\_KOMPG:C4R1M0|C4R1M0\_KOMPG:C4R6U1|C4R6U1\_KOMPG:C4QWY4|C4QWY4\_KOMPG:C4R1V5|C4R1V5\_KOMPG:C4R4T6|C4R4T6\_KOMPG:C4QVG5|C4QVG5\_KOMPG:C4QW83|C4QW83\_KOMPG:C4QXL2|C4QXL2\_KOMPG:C4R8W5|C4R8W5\_KOMPG:C4R0F1|C4R0F1\_KOMPG:C4R1U3|C4R1U3\_KOMPG:C4R199|C4R199\_KOMPG:C4R9A7|C4R9A7\_KOMPG:C4R1W8|C4R1W8\_KOMPG:C4R7K4|C4R7K4\_KOMPG:C4R407|C4R407\_KOMPG:C4QYX5|C4QYX5\_KOMPG:C4R1T3|C4R1T3\_KOMPG:C4QWD1|C4QWD1\_KOMPG:C4QZN3|C4QZN3\_KOMPG:C4QZM9|C4QZM9\_KOMPG:C4R7K6|C4R7K6\_KOMPG:C4R668|C4R668\_KOMPG:C4R3U7|C4R3U7\_KOMPG:C4R092|C4R092\_KOMPG:C4R3Q9|C4R3Q9\_KOMPG:C4R8E3|C4R8E3\_KOMPG:C4R8V3|C4R8V3\_KOMPG |  |  | DB Search |
| VSSL | 20.48 | 404.2271 | 4 | -7.08 | 405.2305 | 8.17 | 2993 | 7.47e3 | 1 | 1 | C4R1B7|C4R1B7\_KOMPG:C4QXE5|C4QXE5\_KOMPG:C4QYG6|C4QYG6\_KOMPG:C4R4V2|C4R4V2\_KOMPG:C4R6I4|C4R6I4\_KOMPG:C4R3M8|C4R3M8\_KOMPG:C4R3P2|C4R3P2\_KOMPG:P52710|CBPY\_KOMPG:C4QX16|C4QX16\_KOMPG:C4R816|C4R816\_KOMPG:C4R5C3|C4R5C3\_KOMPG:C4R110|C4R110\_KOMPG:C4R350|C4R350\_KOMPG:C4R0C6|C4R0C6\_KOMPG:C4QY84|C4QY84\_KOMPG:C4QY88|C4QY88\_KOMPG:C4R1W6|C4R1W6\_KOMPG:C4R5B0|C4R5B0\_KOMPG:C4R2J4|C4R2J4\_KOMPG:C4R4E9|C4R4E9\_KOMPG:C4R5M0|C4R5M0\_KOMPG:C4QX73|C4QX73\_KOMPG:C4QYY0|C4QYY0\_KOMPG:C4QZB9|C4QZB9\_KOMPG:C4QXW2|C4QXW2\_KOMPG:C4QYN2|C4QYN2\_KOMPG:C4R6F6|C4R6F6\_KOMPG:C4R7J5|C4R7J5\_KOMPG:C4QWZ5|C4QWZ5\_KOMPG:C4R362|C4R362\_KOMPG:C4QXN2|MIC60\_KOMPG:C4R8N6|C4R8N6\_KOMPG:C4QYN9|C4QYN9\_KOMPG:C4QW38|C4QW38\_KOMPG:C4QZW5|C4QZW5\_KOMPG:C4QW13|C4QW13\_KOMPG:C4QY76|C4QY76\_KOMPG:C4R2T7|C4R2T7\_KOMPG:C4R1M9|C4R1M9\_KOMPG:C4R6Q8|C4R6Q8\_KOMPG:C4R0G1|C4R0G1\_KOMPG:C4QWL2|C4QWL2\_KOMPG:C4R7E3|C4R7E3\_KOMPG:C4R3D8|C4R3D8\_KOMPG:C4R1S7|C4R1S7\_KOMPG:C4R3G0|C4R3G0\_KOMPG:C4R7A7|C4R7A7\_KOMPG:C4QXA9|C4QXA9\_KOMPG:C4R3U5|C4R3U5\_KOMPG:C4R381|C4R381\_KOMPG:C4R6N1|C4R6N1\_KOMPG:C4R7I3|C4R7I3\_KOMPG:C4R4Q6|C4R4Q6\_KOMPG:C4R3K2|C4R3K2\_KOMPG:C4QYF3|C4QYF3\_KOMPG:C4R8G8|C4R8G8\_KOMPG:C4R982|C4R982\_KOMPG:C4R269|C4R269\_KOMPG:C4QYA5|C4QYA5\_KOMPG:C4R191|C4R191\_KOMPG:C4R4I0|C4R4I0\_KOMPG:C4R692|C4R692\_KOMPG:C4R275|C4R275\_KOMPG:C4R8A2|C4R8A2\_KOMPG:C4QWG1|C4QWG1\_KOMPG:C4QXY8|C4QXY8\_KOMPG:C4R0B8|C4R0B8\_KOMPG:C4R2G2|C4R2G2\_KOMPG:C4R317|C4R317\_KOMPG:C4QXQ4|C4QXQ4\_KOMPG:C4QWI4|C4QWI4\_KOMPG:C4R814|C4R814\_KOMPG:C4R6Z4|C4R6Z4\_KOMPG:C4R818|C4R818\_KOMPG:C4R8E9|C4R8E9\_KOMPG:C4QYM3|C4QYM3\_KOMPG:C4R8I9|C4R8I9\_KOMPG:C4QZR2|C4QZR2\_KOMPG:C4R5S6|C4R5S6\_KOMPG:C4R5Z3|C4R5Z3\_KOMPG:C4R1Q8|C4R1Q8\_KOMPG:C4R5I7|C4R5I7\_KOMPG:C4QYH7|C4QYH7\_KOMPG:C4QYV4|C4QYV4\_KOMPG:C4R1Q9|C4R1Q9\_KOMPG:C4QYS4|C4QYS4\_KOMPG:C4R5M9|C4R5M9\_KOMPG:C4R7W7|C4R7W7\_KOMPG:C4R286|C4R286\_KOMPG:C4R5S4|C4R5S4\_KOMPG:C4QYX1|C4QYX1\_KOMPG:C4R9D8|C4R9D8\_KOMPG:C4R929|C4R929\_KOMPG:C4QX97|C4QX97\_KOMPG:C4QZI9|C4QZI9\_KOMPG:C4QVD8|C4QVD8\_KOMPG:C4QXU3|C4QXU3\_KOMPG:C4R433|C4R433\_KOMPG:C4R804|C4R804\_KOMPG:C4QX13|C4QX13\_KOMPG:C4R0R6|C4R0R6\_KOMPG:C4QXA7|C4QXA7\_KOMPG:C4QY34|C4QY34\_KOMPG:C4R111|C4R111\_KOMPG:C4R4E5|C4R4E5\_KOMPG:C4QVY7|C4QVY7\_KOMPG:C4QYU1|C4QYU1\_KOMPG:C4R5I0|C4R5I0\_KOMPG:C4R602|C4R602\_KOMPG:C4QYH1|C4QYH1\_KOMPG:C4R877|C4R877\_KOMPG:C4QZ59|C4QZ59\_KOMPG:C4R444|C4R444\_KOMPG:C4R1S9|C4R1S9\_KOMPG:C4R5V9|C4R5V9\_KOMPG:C4R0F9|C4R0F9\_KOMPG:C4QV22|C4QV22\_KOMPG:C4QZH7|C4QZH7\_KOMPG:C4QZ69|C4QZ69\_KOMPG:C4R703|C4R703\_KOMPG:C4R123|C4R123\_KOMPG:C4R212|C4R212\_KOMPG:C4R5S0|C4R5S0\_KOMPG:C4QVH2|C4QVH2\_KOMPG:C4QVP3|C4QVP3\_KOMPG:C4R1C2|C4R1C2\_KOMPG:C4QVT5|C4QVT5\_KOMPG:C4QXG4|C4QXG4\_KOMPG:C4QX70|C4QX70\_KOMPG:C4R4T7|C4R4T7\_KOMPG:C4R448|C4R448\_KOMPG:C4R473|C4R473\_KOMPG:C4R8V9|C4R8V9\_KOMPG:C4R3B5|C4R3B5\_KOMPG:C4R483|C4R483\_KOMPG:C4R5W9|C4R5W9\_KOMPG:C4R8W9|C4R8W9\_KOMPG:C4R8N0|C4R8N0\_KOMPG |  |  | DB Search |
| GSVI | 20.47 | 374.2165 | 4 | -5.12 | 375.2209 | 10.69 | 4154 | 1e3 | 1 | 1 | C4R491|C4R491\_KOMPG:C4QWA4|C4QWA4\_KOMPG:C4QYE1|C4QYE1\_KOMPG:C4QVA5|C4QVA5\_KOMPG:C4R895|C4R895\_KOMPG:C4QZD1|C4QZD1\_KOMPG:C4R3R9|C4R3R9\_KOMPG:C4R2K6|C4R2K6\_KOMPG:C4R564|C4R564\_KOMPG:C4R083|C4R083\_KOMPG:C4QYZ6|C4QYZ6\_KOMPG:C4R1L8|C4R1L8\_KOMPG:C4R5L3|C4R5L3\_KOMPG:C4R7Y5|C4R7Y5\_KOMPG:C4R058|C4R058\_KOMPG:C4R9F2|C4R9F2\_KOMPG:C4R0L6|C4R0L6\_KOMPG:C4R386|C4R386\_KOMPG:C4QX98|C4QX98\_KOMPG:C4R3C5|C4R3C5\_KOMPG:C4R7L4|C4R7L4\_KOMPG:C4QVM0|C4QVM0\_KOMPG:C4R3B0|C4R3B0\_KOMPG:C4R421|C4R421\_KOMPG:C4R8Q6|C4R8Q6\_KOMPG:C4QX13|C4QX13\_KOMPG:C4R7J5|C4R7J5\_KOMPG:C4R3A0|C4R3A0\_KOMPG:C4QYU6|C4QYU6\_KOMPG:C4R4T4|C4R4T4\_KOMPG:C4R6M4|C4R6M4\_KOMPG:C4R634|C4R634\_KOMPG:C4R925|C4R925\_KOMPG:C4R728|C4R728\_KOMPG:C4R4Z9|C4R4Z9\_KOMPG:C4R0G5|C4R0G5\_KOMPG:C4R690|C4R690\_KOMPG:C4R1Q3|C4R1Q3\_KOMPG:C4R8F4|C4R8F4\_KOMPG:C4R0U5|C4R0U5\_KOMPG:C4QYQ8|C4QYQ8\_KOMPG:C4QW18|C4QW18\_KOMPG:C4QWL7|C4QWL7\_KOMPG:C4R0E6|C4R0E6\_KOMPG:C4QYY3|C4QYY3\_KOMPG:C4R3B1|C4R3B1\_KOMPG:C4QVT5|C4QVT5\_KOMPG:C4R9C9|C4R9C9\_KOMPG:C4R971|C4R971\_KOMPG:C4R092|C4R092\_KOMPG:C4R2B2|C4R2B2\_KOMPG:C4QZ04|C4QZ04\_KOMPG |  |  | DB Search |
| GSVL | 20.47 | 374.2165 | 4 | -5.12 | 375.2209 | 10.69 | 4154 | 1e3 | 1 | 1 | C4QVM2|C4QVM2\_KOMPG:C4QWF8|C4QWF8\_KOMPG:C4R080|C4R080\_KOMPG:C4R662|C4R662\_KOMPG:C4QW60|C4QW60\_KOMPG:C4R6T3|C4R6T3\_KOMPG:C4R4C2|C4R4C2\_KOMPG:C4R6A4|C4R6A4\_KOMPG:C4R343|C4R343\_KOMPG:C4QWK5|C4QWK5\_KOMPG:C4R3D3|C4R3D3\_KOMPG:C4QV06|C4QV06\_KOMPG:C4R5C6|C4R5C6\_KOMPG:C4R234|C4R234\_KOMPG:C4QXC3|C4QXC3\_KOMPG:C4R286|C4R286\_KOMPG:C4R1L3|C4R1L3\_KOMPG:C4R084|C4R084\_KOMPG:C4R6S9|C4R6S9\_KOMPG:C4R929|C4R929\_KOMPG:C4QV90|C4QV90\_KOMPG:C4QX48|C4QX48\_KOMPG:C4R4Y9|C4R4Y9\_KOMPG:C4R6X8|C4R6X8\_KOMPG:C4R2T9|C4R2T9\_KOMPG:C4QV42|C4QV42\_KOMPG:C4R7J8|C4R7J8\_KOMPG:C4QWC6|C4QWC6\_KOMPG:C4R323|C4R323\_KOMPG:C4R451|C4R451\_KOMPG:C4R5R6|C4R5R6\_KOMPG:C4R0I6|C4R0I6\_KOMPG:C4R437|C4R437\_KOMPG:C4R573|C4R573\_KOMPG:C4QXF0|C4QXF0\_KOMPG:C4R2E4|C4R2E4\_KOMPG:C4R0R6|C4R0R6\_KOMPG:C4R810|C4R810\_KOMPG:C4QY11|C4QY11\_KOMPG:C4R6U8|C4R6U8\_KOMPG:C4QW21|C4QW21\_KOMPG:C4R0E2|C4R0E2\_KOMPG:C4QYG9|C4QYG9\_KOMPG:C4R586|C4R586\_KOMPG:C4QZD7|C4QZD7\_KOMPG:C4R1T1|C4R1T1\_KOMPG:C4QX83|C4QX83\_KOMPG:C4R6C0|C4R6C0\_KOMPG:C4QZ59|C4QZ59\_KOMPG:C4QZ03|C4QZ03\_KOMPG:C4R0U9|C4R0U9\_KOMPG:C4R441|C4R441\_KOMPG:C4QVS6|C4QVS6\_KOMPG:C4QYH5|C4QYH5\_KOMPG:C4QZ76|C4QZ76\_KOMPG:C4R2U0|C4R2U0\_KOMPG:C4R2Z7|C4R2Z7\_KOMPG:C4R6D1|C4R6D1\_KOMPG:C4R6R4|C4R6R4\_KOMPG:C4R0Y6|C4R0Y6\_KOMPG:C4R325|C4R325\_KOMPG:C4R6W2|C4R6W2\_KOMPG:C4QY86|C4QY86\_KOMPG:C4R5X0|C4R5X0\_KOMPG:C4R122|C4R122\_KOMPG:C4R4T2|C4R4T2\_KOMPG:C4R6W1|C4R6W1\_KOMPG:C4R7R8|C4R7R8\_KOMPG:C4R6I2|C4R6I2\_KOMPG:C4QZA7|C4QZA7\_KOMPG:C4QZB8|C4QZB8\_KOMPG:C4QVV9|C4QVV9\_KOMPG:C4R1Y8|C4R1Y8\_KOMPG:C4R3F9|C4R3F9\_KOMPG:C4R077|C4R077\_KOMPG:C4QWY3|C4QWY3\_KOMPG:C4R627|C4R627\_KOMPG:C4R0C8|C4R0C8\_KOMPG:C4R8I9|C4R8I9\_KOMPG:C4QYX3|C4QYX3\_KOMPG |  |  | DB Search |
| DYNIQ | 20.46 | 651.2864 | 5 | -0.94 | 652.2914 | 10.88 | 4258 | 1.35e3 | 1 | 1 | C4R5D4|C4R5D4\_KOMPG:C4R8V1|C4R8V1\_KOMPG:C4R0U2|C4R0U2\_KOMPG |  |  | DB Search |
| DYNLQ | 20.46 | 651.2864 | 5 | -0.94 | 652.2914 | 10.88 | 4258 | 1.35e3 | 1 | 1 | C4R0K4|C4R0K4\_KOMPG |  |  | DB Search |
| AIVE | 20.45 | 430.2427 | 4 | -5.9 | 431.2464 | 12.49 | 4901 | 0 | 0 | 0 | C4R166|C4R166\_KOMPG:C4QYU1|C4QYU1\_KOMPG:C4R943|C4R943\_KOMPG:C4R0Z3|C4R0Z3\_KOMPG:C4QV61|C4QV61\_KOMPG:C4QYX8|C4QYX8\_KOMPG:C4R1P6|C4R1P6\_KOMPG:C4R4V3|C4R4V3\_KOMPG:C4QZV6|C4QZV6\_KOMPG:C4R3W7|C4R3W7\_KOMPG:C4QVX8|C4QVX8\_KOMPG:C4R2R4|C4R2R4\_KOMPG:C4R7G4|C4R7G4\_KOMPG:C4QVQ2|C4QVQ2\_KOMPG:C4QY13|C4QY13\_KOMPG:C4R1Z1|C4R1Z1\_KOMPG:C4R580|C4R580\_KOMPG:C4R0U5|C4R0U5\_KOMPG:C4R3T5|C4R3T5\_KOMPG:C4R0T6|C4R0T6\_KOMPG:C4R6Y6|C4R6Y6\_KOMPG:C4R4T6|C4R4T6\_KOMPG:C4R1X5|C4R1X5\_KOMPG:C4QV90|C4QV90\_KOMPG:C4R6G5|C4R6G5\_KOMPG:C4R3M5|C4R3M5\_KOMPG:C4QWS3|C4QWS3\_KOMPG:C4R559|C4R559\_KOMPG:C4R3R1|C4R3R1\_KOMPG:C4QV25|C4QV25\_KOMPG:C4R530|C4R530\_KOMPG:C4R297|C4R297\_KOMPG:C4R3V5|C4R3V5\_KOMPG:C4QVI8|C4QVI8\_KOMPG:C4QX77|C4QX77\_KOMPG:C4QYS6|MTNA\_KOMPG:C4R404|C4R404\_KOMPG:C4R349|C4R349\_KOMPG:C4R901|C4R901\_KOMPG:C4R3S6|C4R3S6\_KOMPG:C4R567|C4R567\_KOMPG:C4QYF9|C4QYF9\_KOMPG:C4QVT0|C4QVT0\_KOMPG:C4QYE3|C4QYE3\_KOMPG |  |  | DB Search |
| ALVE | 20.45 | 430.2427 | 4 | -5.9 | 431.2464 | 12.49 | 4901 | 0 | 0 | 0 | C4QVT8|C4QVT8\_KOMPG:C4QZ44|C4QZ44\_KOMPG:C4QXK4|C4QXK4\_KOMPG:C4QWE6|C4QWE6\_KOMPG:C4R5E2|C4R5E2\_KOMPG:C4QVG9|C4QVG9\_KOMPG:C4QV01|C4QV01\_KOMPG:C4R6P2|C4R6P2\_KOMPG:C4R568|C4R568\_KOMPG:C4R7Z9|C4R7Z9\_KOMPG:C4R5P3|C4R5P3\_KOMPG:C4R1C8|C4R1C8\_KOMPG:C4QZU6|C4QZU6\_KOMPG:C4QWV8|C4QWV8\_KOMPG:C4R8E1|C4R8E1\_KOMPG:C4R563|C4R563\_KOMPG:C4R8E7|C4R8E7\_KOMPG:C4QWI9|C4QWI9\_KOMPG:C4R9F2|C4R9F2\_KOMPG:C4R1T4|C4R1T4\_KOMPG:C4R331|RRG9\_KOMPG:C4QWW9|C4QWW9\_KOMPG:C4R5J0|C4R5J0\_KOMPG:C4QYR3|C4QYR3\_KOMPG:C4QY42|C4QY42\_KOMPG:C4R5M2|C4R5M2\_KOMPG:C4R968|C4R968\_KOMPG:C4R1M4|C4R1M4\_KOMPG:C4QVK5|C4QVK5\_KOMPG:C4QWM9|C4QWM9\_KOMPG:C4R2L0|PEX5\_KOMPG:C4R075|C4R075\_KOMPG:C4QYN9|C4QYN9\_KOMPG:C4QZR4|C4QZR4\_KOMPG:C4R4H9|C4R4H9\_KOMPG:C4QXT1|C4QXT1\_KOMPG:C4R339|C4R339\_KOMPG:C4R7N8|C4R7N8\_KOMPG:C4R904|C4R904\_KOMPG:C4R6C7|C4R6C7\_KOMPG:C4R310|C4R310\_KOMPG:C4QXN3|C4QXN3\_KOMPG:C4R7Q1|C4R7Q1\_KOMPG:C4R4V6|C4R4V6\_KOMPG:C4R1M0|C4R1M0\_KOMPG:C4R8L1|C4R8L1\_KOMPG:C4QYI7|C4QYI7\_KOMPG:C4R7K8|C4R7K8\_KOMPG:C4R1F8|C4R1F8\_KOMPG:C4QYW2|C4QYW2\_KOMPG:C4R129|C4R129\_KOMPG:C4QYG7|C4QYG7\_KOMPG:C4QXA3|C4QXA3\_KOMPG:C4R8Q3|C4R8Q3\_KOMPG:C4QVT9|C4QVT9\_KOMPG:C4R4J0|C4R4J0\_KOMPG:C4R6Y4|C4R6Y4\_KOMPG:C4QWW8|C4QWW8\_KOMPG:C4QXL1|C4QXL1\_KOMPG:C4R8I6|C4R8I6\_KOMPG:C4R4G7|C4R4G7\_KOMPG:C4R2G2|C4R2G2\_KOMPG:C4QYR7|C4QYR7\_KOMPG:C4QXX3|C4QXX3\_KOMPG:C4R970|ASA1\_KOMPG:C4R4P4|C4R4P4\_KOMPG:C4R6E7|C4R6E7\_KOMPG:C4R8N5|C4R8N5\_KOMPG:C4R649|C4R649\_KOMPG |  |  | DB Search |
| NSG | 20.42 | 276.107 | 3 | 6.48 | 277.1154 | 16.74 | 6592 | 0 | 0 | 0 | C4QYE6|C4QYE6\_KOMPG:C4R585|C4R585\_KOMPG:C4R1S0|C4R1S0\_KOMPG:C4R7R7|C4R7R7\_KOMPG:C4R4G8|C4R4G8\_KOMPG:C4R897|C4R897\_KOMPG:C4QWW6|C4QWW6\_KOMPG:C4R5N2|C4R5N2\_KOMPG:C4R1X4|C4R1X4\_KOMPG:C4R8B9|C4R8B9\_KOMPG:C4QW93|C4QW93\_KOMPG:C4R4J5|C4R4J5\_KOMPG:C4QYX9|C4QYX9\_KOMPG:C4QVS7|C4QVS7\_KOMPG:C4R4D9|C4R4D9\_KOMPG:C4QY81|C4QY81\_KOMPG:C4R1T4|C4R1T4\_KOMPG:C4QXK9|C4QXK9\_KOMPG:C4QYQ5|C4QYQ5\_KOMPG:C4QZ97|C4QZ97\_KOMPG:C4R571|C4R571\_KOMPG:C4R0Y7|C4R0Y7\_KOMPG:C4R8Q7|C4R8Q7\_KOMPG:C4R3S6|C4R3S6\_KOMPG:C4R016|C4R016\_KOMPG:C4R4M7|C4R4M7\_KOMPG:C4R666|C4R666\_KOMPG:C4R739|C4R739\_KOMPG:C4QXY6|C4QXY6\_KOMPG:C4R6A7|C4R6A7\_KOMPG:C4R684|C4R684\_KOMPG:C4R2Y2|C4R2Y2\_KOMPG:C4R044|C4R044\_KOMPG:C4QW89|C4QW89\_KOMPG:C4QYU4|C4QYU4\_KOMPG:C4QUZ7|C4QUZ7\_KOMPG:C4QVY2|C4QVY2\_KOMPG:C4QXN4|C4QXN4\_KOMPG:C4QYA8|C4QYA8\_KOMPG:C4QV54|C4QV54\_KOMPG:C4R0V8|C4R0V8\_KOMPG:C4R8C4|C4R8C4\_KOMPG:C4QVI9|C4QVI9\_KOMPG:C4R447|C4R447\_KOMPG:C4R0Q7|C4R0Q7\_KOMPG:C4QXA9|C4QXA9\_KOMPG:C4R6D1|C4R6D1\_KOMPG:C4R340|C4R340\_KOMPG:C4R7B9|C4R7B9\_KOMPG:C4QYK2|C4QYK2\_KOMPG:C4R0D1|C4R0D1\_KOMPG:C4R579|C4R579\_KOMPG:C4QZA7|C4QZA7\_KOMPG:C4QWD1|C4QWD1\_KOMPG:C4R941|GLG\_KOMPG:C4R567|C4R567\_KOMPG:C4R7I9|C4R7I9\_KOMPG:C4QV86|C4QV86\_KOMPG:Q9Y751|ATG26\_KOMPG:C4R0S7|C4R0S7\_KOMPG:C4R1U2|C4R1U2\_KOMPG:C4R7D0|C4R7D0\_KOMPG:C4R2L9|C4R2L9\_KOMPG:C4R1N5|C4R1N5\_KOMPG:C4R0U8|C4R0U8\_KOMPG:C4R6L7|C4R6L7\_KOMPG:C4R912|C4R912\_KOMPG:C4QXC5|C4QXC5\_KOMPG:C4R371|C4R371\_KOMPG:C4QW88|C4QW88\_KOMPG:C4R5A7|C4R5A7\_KOMPG:C4QX89|C4QX89\_KOMPG:C4R148|C4R148\_KOMPG:C4QZ69|C4QZ69\_KOMPG:C4QVD4|C4QVD4\_KOMPG:C4QXX4|C4QXX4\_KOMPG:C4QYA1|C4QYA1\_KOMPG:C4R6W3|C4R6W3\_KOMPG:C4R4A2|C4R4A2\_KOMPG:C4R1I3|C4R1I3\_KOMPG:C4R126|C4R126\_KOMPG:C4R2P7|C4R2P7\_KOMPG:C4R0F0|C4R0F0\_KOMPG:C4R5H2|C4R5H2\_KOMPG:C4R0N3|C4R0N3\_KOMPG:C4R176|C4R176\_KOMPG:C4R5A9|C4R5A9\_KOMPG:C4QYE1|C4QYE1\_KOMPG:C4R1B3|C4R1B3\_KOMPG:C4R4N1|C4R4N1\_KOMPG:C4R1D2|C4R1D2\_KOMPG:C4R6A4|C4R6A4\_KOMPG:C4QV84|C4QV84\_KOMPG:C4QY40|C4QY40\_KOMPG:C4R5Q1|C4R5Q1\_KOMPG:C4R7A3|C4R7A3\_KOMPG:C4QXW7|C4QXW7\_KOMPG:C4R1X1|C4R1X1\_KOMPG:C4R8D4|C4R8D4\_KOMPG:C4QWU6|C4QWU6\_KOMPG:C4R7M7|C4R7M7\_KOMPG:C4QZR1|C4QZR1\_KOMPG:C4R490|C4R490\_KOMPG:C4QWV0|C4QWV0\_KOMPG:C4QWU0|C4QWU0\_KOMPG:C4QZN7|C4QZN7\_KOMPG:C4R0G4|C4R0G4\_KOMPG:C4R3C9|C4R3C9\_KOMPG:C4QZW5|C4QZW5\_KOMPG:C4QWQ0|C4QWQ0\_KOMPG:C4R3D7|C4R3D7\_KOMPG:C4QX83|C4QX83\_KOMPG:C4R078|C4R078\_KOMPG:C4QY76|C4QY76\_KOMPG:C4R3F4|C4R3F4\_KOMPG:C4R925|C4R925\_KOMPG:C4R955|C4R955\_KOMPG:C4QZW2|C4QZW2\_KOMPG:C4QX78|C4QX78\_KOMPG:C4R455|C4R455\_KOMPG:C4QVL3|C4QVL3\_KOMPG:C4QWL2|C4QWL2\_KOMPG:C4R1N0|C4R1N0\_KOMPG:C4QZ83|C4QZ83\_KOMPG:C4QV66|C4QV66\_KOMPG:C4R517|C4R517\_KOMPG:C4R0W4|C4R0W4\_KOMPG:C4R572|C4R572\_KOMPG:C4QYB6|C4QYB6\_KOMPG:C4R6F0|PEX3\_KOMPG:C4R6G5|C4R6G5\_KOMPG:C4R8G9|C4R8G9\_KOMPG:C4R446|C4R446\_KOMPG:C4QYU7|C4QYU7\_KOMPG:C4R6X9|C4R6X9\_KOMPG:C4R7Q2|C4R7Q2\_KOMPG:C4QWM7|C4QWM7\_KOMPG:C4R165|C4R165\_KOMPG:C4QWA5|C4QWA5\_KOMPG:C4QX40|C4QX40\_KOMPG:C4R3T9|C4R3T9\_KOMPG:C4R254|C4R254\_KOMPG:C4R0Z6|C4R0Z6\_KOMPG:C4R5B1|C4R5B1\_KOMPG:C4R5X7|C4R5X7\_KOMPG:C4R0M0|C4R0M0\_KOMPG:C4QZB4|C4QZB4\_KOMPG:C4R2G2|C4R2G2\_KOMPG:C4R332|C4R332\_KOMPG:C4QW82|C4QW82\_KOMPG:C4QZR5|C4QZR5\_KOMPG:C4R6Z4|C4R6Z4\_KOMPG:C4R8D9|C4R8D9\_KOMPG:C4R4X6|C4R4X6\_KOMPG:C4R5V5|C4R5V5\_KOMPG:C4R6Q0|C4R6Q0\_KOMPG:C4QWG8|C4QWG8\_KOMPG:C4R6J1|C4R6J1\_KOMPG:C4R5F5|C4R5F5\_KOMPG:C4R0V4|C4R0V4\_KOMPG:C4QZD1|C4QZD1\_KOMPG:C4QZ79|C4QZ79\_KOMPG:C4R2B6|C4R2B6\_KOMPG:C4R330|C4R330\_KOMPG:C4R534|C4R534\_KOMPG:C4R6P6|C4R6P6\_KOMPG:C4QYT3|C4QYT3\_KOMPG:C4QYT0|C4QYT0\_KOMPG:C4QZT4|C4QZT4\_KOMPG:C4QWB5|C4QWB5\_KOMPG:C4R1E3|C4R1E3\_KOMPG:C4R717|C4R717\_KOMPG:C4R1M4|C4R1M4\_KOMPG:C4QZF0|C4QZF0\_KOMPG:C4R349|C4R349\_KOMPG:C4QY80|C4QY80\_KOMPG:C4R754|C4R754\_KOMPG:C4QVT0|C4QVT0\_KOMPG:C4R409|C4R409\_KOMPG:C4R7U4|C4R7U4\_KOMPG:C4R6M4|C4R6M4\_KOMPG:C4R877|C4R877\_KOMPG:C4R032|C4R032\_KOMPG:C4QZ48|C4QZ48\_KOMPG:C4QZH7|C4QZH7\_KOMPG:C4R709|C4R709\_KOMPG:C4R3R5|C4R3R5\_KOMPG:C4QY13|C4QY13\_KOMPG:C4R803|C4R803\_KOMPG:C4QYQ2|C4QYQ2\_KOMPG:C4R7H2|C4R7H2\_KOMPG:C4R4D7|C4R4D7\_KOMPG:C4R898|C4R898\_KOMPG:C4R5S0|C4R5S0\_KOMPG:C4QVK6|C4QVK6\_KOMPG:C4QW27|C4QW27\_KOMPG:C4QXA5|PFKA2\_KOMPG:C4R4M0|C4R4M0\_KOMPG:C4R516|C4R516\_KOMPG:C4R2S8|C4R2S8\_KOMPG:C4R743|C4R743\_KOMPG:C4QVP3|C4QVP3\_KOMPG:C4QX81|C4QX81\_KOMPG:C4R1T3|C4R1T3\_KOMPG:C4QZC0|C4QZC0\_KOMPG:C4QYF7|C4QYF7\_KOMPG:C4R1V0|C4R1V0\_KOMPG:C4R7U8|C4R7U8\_KOMPG:C4QWS9|C4QWS9\_KOMPG:C4R855|C4R855\_KOMPG:C4R721|C4R721\_KOMPG |  |  | DB Search |
| GTIF | 20.4 | 436.2322 | 4 | -4.1 | 437.2366 | 23.18 | 8454 | 1.09e3 | 1 | 1 | C4R2D4|C4R2D4\_KOMPG:C4QXL0|C4QXL0\_KOMPG:C4QZ71|C4QZ71\_KOMPG:C4QZ59|C4QZ59\_KOMPG:C4QXF3|C4QXF3\_KOMPG:C4QYU4|C4QYU4\_KOMPG:C4R357|C4R357\_KOMPG:C4R035|C4R035\_KOMPG:C4QZY4|C4QZY4\_KOMPG:C4R0W8|C4R0W8\_KOMPG:C4R6U3|C4R6U3\_KOMPG:C4R7P2|C4R7P2\_KOMPG:C4QWK4|C4QWK4\_KOMPG:C4QZE8|C4QZE8\_KOMPG:C4QWA3|C4QWA3\_KOMPG:C4QVM1|C4QVM1\_KOMPG:C4R379|C4R379\_KOMPG:C4R8X4|C4R8X4\_KOMPG:C4R318|C4R318\_KOMPG:C4R649|C4R649\_KOMPG:C4R1Y7|C4R1Y7\_KOMPG |  |  | DB Search |
| GTLF | 20.4 | 436.2322 | 4 | -4.1 | 437.2366 | 23.18 | 8454 | 1.09e3 | 1 | 1 | C4R479|C4R479\_KOMPG:C4QY30|C4QY30\_KOMPG:C4R1D2|C4R1D2\_KOMPG:C4R6Q8|C4R6Q8\_KOMPG:C4QVL3|C4QVL3\_KOMPG:C4R4B9|C4R4B9\_KOMPG:C4QVH6|C4QVH6\_KOMPG:C4QZM1|C4QZM1\_KOMPG:C4R8U8|C4R8U8\_KOMPG:C4QYQ8|C4QYQ8\_KOMPG:C4R7B2|C4R7B2\_KOMPG:C4R6V3|C4R6V3\_KOMPG:C4R284|C4R284\_KOMPG:C4R3Z8|C4R3Z8\_KOMPG:C4QWH7|C4QWH7\_KOMPG:C4R7N1|C4R7N1\_KOMPG:C4QWS1|C4QWS1\_KOMPG:C4QYF8|C4QYF8\_KOMPG:C4R7P7|C4R7P7\_KOMPG:C4R187|C4R187\_KOMPG:C4R8E2|C4R8E2\_KOMPG:C4QYV3|C4QYV3\_KOMPG:C4R5Z5|C4R5Z5\_KOMPG:C4R8Q7|C4R8Q7\_KOMPG:C4QZL3|C4QZL3\_KOMPG:C4QVN5|C4QVN5\_KOMPG:C4R1K5|C4R1K5\_KOMPG:C4QWE7|C4QWE7\_KOMPG |  |  | DB Search |
| SAE | 20.39 | 305.1223 | 3 | 3.83 | 306.13 | 8.24 | 3110 | 9.42e3 | 2 | 2 | C4QXJ1|C4QXJ1\_KOMPG:C4QWE1|C4QWE1\_KOMPG:C4QYE1|C4QYE1\_KOMPG:C4R6K9|C4R6K9\_KOMPG:C4R214|C4R214\_KOMPG:C4R4U2|C4R4U2\_KOMPG:C4R1G7|C4R1G7\_KOMPG:C4QX59|C4QX59\_KOMPG:C4R5W8|C4R5W8\_KOMPG:C4R3W9|C4R3W9\_KOMPG:C4QWD7|C4QWD7\_KOMPG:C4R2A4|C4R2A4\_KOMPG:C4R8D4|C4R8D4\_KOMPG:C4R0U4|C4R0U4\_KOMPG:C4R5J4|C4R5J4\_KOMPG:C4R3V0|C4R3V0\_KOMPG:C4R6G3|C4R6G3\_KOMPG:C4QV42|C4QV42\_KOMPG:C4R5Q7|C4R5Q7\_KOMPG:C4R7E6|C4R7E6\_KOMPG:C4QYW1|C4QYW1\_KOMPG:C4R571|C4R571\_KOMPG:C4QYV9|C4QYV9\_KOMPG:C4R4M7|C4R4M7\_KOMPG:C4R7S0|C4R7S0\_KOMPG:C4R233|C4R233\_KOMPG:C4R684|C4R684\_KOMPG:C4QVV6|C4QVV6\_KOMPG:C4R6H7|C4R6H7\_KOMPG:C4R7X8|BMT2\_KOMPG:C4QVX9|C4QVX9\_KOMPG:C4QZW2|C4QZW2\_KOMPG:C4R5U2|C4R5U2\_KOMPG:C4R4K8|C4R4K8\_KOMPG:C4R3L0|C4R3L0\_KOMPG:C4R0X4|C4R0X4\_KOMPG:C4QV66|C4QV66\_KOMPG:C4QWF6|C4QWF6\_KOMPG:C4R438|C4R438\_KOMPG:C4R4D6|C4R4D6\_KOMPG:C4QYP8|C4QYP8\_KOMPG:C4R6D1|C4R6D1\_KOMPG:C4R4K9|C4R4K9\_KOMPG:C4R4Z1|C4R4Z1\_KOMPG:C4R4F5|C4R4F5\_KOMPG:C4R4B3|CEGT\_KOMPG:C4R129|C4R129\_KOMPG:C4R1X6|C4R1X6\_KOMPG:C4QYU7|C4QYU7\_KOMPG:C4QVT9|C4QVT9\_KOMPG:C4QYX6|C4QYX6\_KOMPG:C4R8J5|C4R8J5\_KOMPG:C4R243|C4R243\_KOMPG:C4R1D4|C4R1D4\_KOMPG:C4R7C8|C4R7C8\_KOMPG:C4QYC4|C4QYC4\_KOMPG:C4QZA7|C4QZA7\_KOMPG:C4QWD1|C4QWD1\_KOMPG:C4R3L2|C4R3L2\_KOMPG:C4R686|C4R686\_KOMPG:C4R8Z4|C4R8Z4\_KOMPG:C4R6M2|C4R6M2\_KOMPG:C4R8D9|C4R8D9\_KOMPG:C4R7G1|C4R7G1\_KOMPG:C4R1U5|C4R1U5\_KOMPG:C4R3A1|C4R3A1\_KOMPG:C4R759|C4R759\_KOMPG:C4QXT2|C4QXT2\_KOMPG:C4R0D6|C4R0D6\_KOMPG:C4R4C2|C4R4C2\_KOMPG:C4QZN9|C4QZN9\_KOMPG:C4R2U1|C4R2U1\_KOMPG:C4R972|C4R972\_KOMPG:C4R1N5|C4R1N5\_KOMPG:C4R011|C4R011\_KOMPG:C4QYP0|C4QYP0\_KOMPG:Q9P4D0|SEC17\_KOMPG:C4QYH3|C4QYH3\_KOMPG:C4R8F1|C4R8F1\_KOMPG:C4QWA3|C4QWA3\_KOMPG:C4QY32|C4QY32\_KOMPG:C4R912|C4R912\_KOMPG:C4R3M5|C4R3M5\_KOMPG:C4QYT3|C4QYT3\_KOMPG:C4R826|PEX4\_KOMPG:C4R3T0|C4R3T0\_KOMPG:C4QX72|C4QX72\_KOMPG:C4R349|C4R349\_KOMPG:C4R5K6|C4R5K6\_KOMPG:C4R0B4|C4R0B4\_KOMPG:C4QVH4|C4QVH4\_KOMPG:C4R154|C4R154\_KOMPG:C4QVV3|C4QVV3\_KOMPG:C4QXL0|C4QXL0\_KOMPG:C4QVL8|C4QVL8\_KOMPG:C4QZE2|C4QZE2\_KOMPG:C4QYX8|C4QYX8\_KOMPG:C4QZ69|C4QZ69\_KOMPG:C4QZ51|C4QZ51\_KOMPG:C4QVD4|C4QVD4\_KOMPG:C4R6Q4|C4R6Q4\_KOMPG:C4QWX6|C4QWX6\_KOMPG:C4R5T3|C4R5T3\_KOMPG:C4R7H2|C4R7H2\_KOMPG:C4R179|C4R179\_KOMPG:C4R5F7|C4R5F7\_KOMPG:C4QV48|C4QV48\_KOMPG:C4QX02|C4QX02\_KOMPG:C4QW27|C4QW27\_KOMPG:C4R1W8|C4R1W8\_KOMPG:C4R1I3|C4R1I3\_KOMPG:C4QXM7|C4QXM7\_KOMPG:C4QYX7|C4QYX7\_KOMPG:C4R7U8|C4R7U8\_KOMPG:C4R565|C4R565\_KOMPG:C4R5V4|C4R5V4\_KOMPG:C4QY10|C4QY10\_KOMPG:C4QV16|C4QV16\_KOMPG:C4QYR5|C4QYR5\_KOMPG:C4QXE9|CHO2\_KOMPG:C4R405|C4R405\_KOMPG:C4R3A9|C4R3A9\_KOMPG |  |  | DB Search |
| TIAV | 20.37 | 402.2478 | 4 | -1.76 | 403.2534 | 15.04 | 5856 | 1.07e4 | 1 | 1 | C4R5C1|C4R5C1\_KOMPG:C4QYW7|C4QYW7\_KOMPG:C4R296|C4R296\_KOMPG:C4R4Y5|C4R4Y5\_KOMPG:C4R6C2|PEX1\_KOMPG:C4QW59|C4QW59\_KOMPG:C4R7H6|C4R7H6\_KOMPG:C4QV66|C4QV66\_KOMPG:C4QX08|C4QX08\_KOMPG:C4R2U0|C4R2U0\_KOMPG:C4QYZ7|C4QYZ7\_KOMPG:C4R125|C4R125\_KOMPG:C4R863|C4R863\_KOMPG:C4R3A3|C4R3A3\_KOMPG:C4QXE3|C4QXE3\_KOMPG:C4QWV8|C4QWV8\_KOMPG:C4QXZ6|C4QXZ6\_KOMPG:C4R6Z0|C4R6Z0\_KOMPG:C4R601|C4R601\_KOMPG:C4R8Q3|C4R8Q3\_KOMPG:C4R3A4|C4R3A4\_KOMPG:C4QVQ3|C4QVQ3\_KOMPG:C4R3Q7|C4R3Q7\_KOMPG:C4R3A0|C4R3A0\_KOMPG:C4R668|C4R668\_KOMPG:C4QVR5|C4QVR5\_KOMPG:C4QZX4|C4QZX4\_KOMPG:C4QVM6|C4QVM6\_KOMPG:C4R713|C4R713\_KOMPG:C4QYW4|C4QYW4\_KOMPG:C4R5C8|C4R5C8\_KOMPG |  |  | DB Search |
| TLAV | 20.37 | 402.2478 | 4 | -1.76 | 403.2534 | 15.04 | 5856 | 1.07e4 | 1 | 1 | C4QZT2|C4QZT2\_KOMPG:C4R037|C4R037\_KOMPG:P52710|CBPY\_KOMPG:C4R1G8|C4R1G8\_KOMPG:C4R5P0|C4R5P0\_KOMPG:C4QVE2|C4QVE2\_KOMPG:C4QVH6|C4QVH6\_KOMPG:C4R5Q8|C4R5Q8\_KOMPG:C4QYM8|C4QYM8\_KOMPG:C4R2E8|C4R2E8\_KOMPG:C4R6P6|C4R6P6\_KOMPG:C4R0B7|C4R0B7\_KOMPG:C4R227|C4R227\_KOMPG:C4R628|PFF1\_KOMPG:C4R7H0|C4R7H0\_KOMPG:C4R4F6|C4R4F6\_KOMPG:C4R986|C4R986\_KOMPG:C4R866|C4R866\_KOMPG:C4R144|C4R144\_KOMPG:C4QZU4|C4QZU4\_KOMPG:C4R5Z2|C4R5Z2\_KOMPG:C4R0P5|C4R0P5\_KOMPG:C4QYD4|C4QYD4\_KOMPG:C4QWV6|C4QWV6\_KOMPG:C4R315|C4R315\_KOMPG:C4QXT7|C4QXT7\_KOMPG:C4R6L3|C4R6L3\_KOMPG:C4R070|C4R070\_KOMPG:C4R5U2|C4R5U2\_KOMPG:C4QVH3|C4QVH3\_KOMPG:C4QXI8|PEX6\_KOMPG:C4R1N0|C4R1N0\_KOMPG:C4QX57|C4QX57\_KOMPG:C4R6N1|C4R6N1\_KOMPG:C4R9C1|C4R9C1\_KOMPG:C4R495|C4R495\_KOMPG:C4QZP3|C4QZP3\_KOMPG:C4R7I5|C4R7I5\_KOMPG:C4QYC3|C4QYC3\_KOMPG:C4R6I9|C4R6I9\_KOMPG:C4R4V5|C4R4V5\_KOMPG:C4R415|MDM12\_KOMPG:C4R0F0|C4R0F0\_KOMPG:C4R5X7|C4R5X7\_KOMPG:C4R7Z4|C4R7Z4\_KOMPG:C4R6A0|C4R6A0\_KOMPG:C4QZB1|C4QZB1\_KOMPG:C4R2C0|C4R2C0\_KOMPG:C4R4M5|C4R4M5\_KOMPG |  |  | DB Search |
| AGAA | 20.37 | 288.1433 | 4 | 8.03 | 289.1522 | 5.91 | 2203 | 6.8e3 | 1 | 1 | C4QW56|C4QW56\_KOMPG:C4R9D9|C4R9D9\_KOMPG:C4QYF0|C4QYF0\_KOMPG:C4R8L6|C4R8L6\_KOMPG:C4QXV4|C4QXV4\_KOMPG:C4R2U1|C4R2U1\_KOMPG:C4R001|C4R001\_KOMPG:C4R1D1|C4R1D1\_KOMPG:C4R422|C4R422\_KOMPG:C4R2V9|C4R2V9\_KOMPG:C4R1C8|C4R1C8\_KOMPG:C4R6P6|C4R6P6\_KOMPG:C4R4D9|C4R4D9\_KOMPG:C4QY12|C4QY12\_KOMPG:C4R4P9|C4R4P9\_KOMPG:C4R3C5|C4R3C5\_KOMPG:C4R048|C4R048\_KOMPG:C4R178|C4R178\_KOMPG:C4QX51|C4QX51\_KOMPG:C4R4J8|C4R4J8\_KOMPG:C4QW88|C4QW88\_KOMPG:C4R3X8|C4R3X8\_KOMPG:C4R3H8|C4R3H8\_KOMPG:C4R367|C4R367\_KOMPG:C4QY78|C4QY78\_KOMPG:C4R0D4|C4R0D4\_KOMPG:C4R395|C4R395\_KOMPG:C4R3T7|C4R3T7\_KOMPG:C4R002|C4R002\_KOMPG:C4R651|C4R651\_KOMPG:C4QZH7|C4QZH7\_KOMPG:C4R774|C4R774\_KOMPG:C4R5H7|C4R5H7\_KOMPG:C4QWF6|C4QWF6\_KOMPG:C4QW62|C4QW62\_KOMPG:C4R891|C4R891\_KOMPG:C4R0H6|C4R0H6\_KOMPG:C4R4Z1|C4R4Z1\_KOMPG:C4R5H4|C4R5H4\_KOMPG:C4R7B6|C4R7B6\_KOMPG:C4R6N0|C4R6N0\_KOMPG:C4QZP6|C4QZP6\_KOMPG:C4R4Y0|C4R4Y0\_KOMPG:C4R1C2|C4R1C2\_KOMPG:C4R0N8|C4R0N8\_KOMPG:C4QVV8|C4QVV8\_KOMPG:C4QZK6|C4QZK6\_KOMPG:C4R2G2|C4R2G2\_KOMPG:C4QV91|C4QV91\_KOMPG:C4R7I8|C4R7I8\_KOMPG:C4QZ42|C4QZ42\_KOMPG:C4R1G0|C4R1G0\_KOMPG |  |  | DB Search |
| FSKSPV | 20.37 | 663.3591 | 6 | 2.51 | 664.3665 | 39.33 | 12741 | 2.69e2 | 1 | 1 | C4R2E9|C4R2E9\_KOMPG |  |  | DB Search |
| FARP | 20.36 | 489.27 | 4 | -4.45 | 490.2738 | 10.19 | 3954 | 6.32e2 | 1 | 1 | C4R8W8|C4R8W8\_KOMPG:C4R5E6|C4R5E6\_KOMPG:C4QVM3|C4QVM3\_KOMPG:C4QYF1|C4QYF1\_KOMPG:C4R343|C4R343\_KOMPG:C4R3Y6|C4R3Y6\_KOMPG:C4QVC2|C4QVC2\_KOMPG:C4QXL7|C4QXL7\_KOMPG:C4QX34|C4QX34\_KOMPG |  |  | DB Search |
| VTGV | 20.36 | 374.2165 | 4 | -4.06 | 375.2213 | 5.20 | 1917 | 6.27e3 | 1 | 1 | C4QWF8|C4QWF8\_KOMPG:C4R2Q9|C4R2Q9\_KOMPG:C4R776|C4R776\_KOMPG:C4R603|C4R603\_KOMPG:C4R564|C4R564\_KOMPG:C4R690|C4R690\_KOMPG:C4R8W4|C4R8W4\_KOMPG:C4QV09|C4QV09\_KOMPG:C4R2A6|C4R2A6\_KOMPG:C4R747|C4R747\_KOMPG:C4QY05|C4QY05\_KOMPG:C4QVT4|C4QVT4\_KOMPG:C4R1L5|C4R1L5\_KOMPG:C4QZU5|C4QZU5\_KOMPG:C4R750|C4R750\_KOMPG:C4R155|C4R155\_KOMPG:C4R621|C4R621\_KOMPG:C4R042|C4R042\_KOMPG:C4R4Q2|C4R4Q2\_KOMPG:C4R901|C4R901\_KOMPG:C4R3F9|C4R3F9\_KOMPG:C4R6H4|C4R6H4\_KOMPG:C4QYW5|C4QYW5\_KOMPG:C4R2D7|FLO11\_KOMPG:C4R169|C4R169\_KOMPG |  |  | DB Search |
| DVLRCLPVD | 20.34 | 1028.5325 | 9 | -3.8 | 515.2703 | 30.44 | 10463 | 9.03e2 | 1 | 1 | C4R0H5|C4R0H5\_KOMPG |  |  | DB Search |
| IPIP | 20.33 | 438.2842 | 4 | -7.82 | 439.287 | 27.42 | 9635 | 1.51e3 | 1 | 1 | C4R184|C4R184\_KOMPG:C4QV86|C4QV86\_KOMPG:C4QWE6|C4QWE6\_KOMPG:C4QYZ3|C4QYZ3\_KOMPG:C4QXN4|C4QXN4\_KOMPG:C4R368|C4R368\_KOMPG:C4QXI8|PEX6\_KOMPG:C4R8Z7|C4R8Z7\_KOMPG:C4R5K5|C4R5K5\_KOMPG:C4R438|C4R438\_KOMPG:C4QXA9|C4QXA9\_KOMPG:C4R4T6|C4R4T6\_KOMPG:C4R4C6|C4R4C6\_KOMPG:C4R1J3|C4R1J3\_KOMPG:C4QZ86|C4QZ86\_KOMPG:C4R1T4|C4R1T4\_KOMPG:C4R0U3|C4R0U3\_KOMPG:C4R9E0|C4R9E0\_KOMPG:C4R3P4|C4R3P4\_KOMPG:C4R370|C4R370\_KOMPG:C4R720|C4R720\_KOMPG:C4R4A5|C4R4A5\_KOMPG:C4R0D0|C4R0D0\_KOMPG:C4R127|COA3\_KOMPG:C4QYR8|C4QYR8\_KOMPG:C4QVI8|C4QVI8\_KOMPG:C4R5R4|C4R5R4\_KOMPG:C4R281|C4R281\_KOMPG:C4R047|C4R047\_KOMPG:C4R135|C4R135\_KOMPG:C4QYA3|C4QYA3\_KOMPG:C4R4P4|C4R4P4\_KOMPG |  |  | DB Search |
| IPLP | 20.33 | 438.2842 | 4 | -7.82 | 439.287 | 27.42 | 9635 | 1.51e3 | 1 | 1 | C4R2U7|C4R2U7\_KOMPG:C4R278|C4R278\_KOMPG:C4R5Z3|C4R5Z3\_KOMPG:C4R1J5|C4R1J5\_KOMPG:C4R9F5|C4R9F5\_KOMPG:C4R8D6|C4R8D6\_KOMPG:C4R926|C4R926\_KOMPG:C4QVH3|C4QVH3\_KOMPG:C4QVW3|C4QVW3\_KOMPG:C4R008|C4R008\_KOMPG:C4R4D8|C4R4D8\_KOMPG:C4QVH6|C4QVH6\_KOMPG:C4R561|C4R561\_KOMPG:C4QXC3|C4QXC3\_KOMPG:C4R011|C4R011\_KOMPG:C4R221|C4R221\_KOMPG:C4R4M4|C4R4M4\_KOMPG:C4R8F1|C4R8F1\_KOMPG:C4R9B6|C4R9B6\_KOMPG:C4R5Z9|C4R5Z9\_KOMPG:C4QXJ3|C4QXJ3\_KOMPG:C4R8J9|C4R8J9\_KOMPG:C4QWU7|C4QWU7\_KOMPG:C4QZB5|C4QZB5\_KOMPG:C4R1Q7|C4R1Q7\_KOMPG:C4R3W5|C4R3W5\_KOMPG:C4R6H6|C4R6H6\_KOMPG:C4QYC4|C4QYC4\_KOMPG:C4QV64|C4QV64\_KOMPG:C4QVQ3|C4QVQ3\_KOMPG:C4R1D5|C4R1D5\_KOMPG:C4QZF5|C4QZF5\_KOMPG:C4R7R0|C4R7R0\_KOMPG:C4QXQ4|C4QXQ4\_KOMPG:C4R785|C4R785\_KOMPG:C4R795|C4R795\_KOMPG:C4R814|C4R814\_KOMPG:C4R6N4|C4R6N4\_KOMPG:C4R6D0|C4R6D0\_KOMPG |  |  | DB Search |
| LPIP | 20.33 | 438.2842 | 4 | -7.82 | 439.287 | 27.42 | 9635 | 1.51e3 | 1 | 1 | C4QVM2|C4QVM2\_KOMPG:C4QZ56|C4QZ56\_KOMPG:C4R232|C4R232\_KOMPG:C4R7D0|C4R7D0\_KOMPG:C4QYP5|C4QYP5\_KOMPG:C4QWU9|C4QWU9\_KOMPG:C4R8J3|C4R8J3\_KOMPG:C4QVC5|C4QVC5\_KOMPG:C4QVQ2|C4QVQ2\_KOMPG:C4R4T9|C4R4T9\_KOMPG:C4QYP0|C4QYP0\_KOMPG:C4R508|C4R508\_KOMPG:C4QYM8|C4QYM8\_KOMPG:C4R885|C4R885\_KOMPG:C4R182|C4R182\_KOMPG:C4R534|C4R534\_KOMPG:C4R1E7|C4R1E7\_KOMPG:C4R318|C4R318\_KOMPG:C4QXZ6|C4QXZ6\_KOMPG:C4R0G9|C4R0G9\_KOMPG:C4QVX0|C4QVX0\_KOMPG:C4R5G9|C4R5G9\_KOMPG:C4R8D2|C4R8D2\_KOMPG:C4QVX5|C4QVX5\_KOMPG:C4R1M4|C4R1M4\_KOMPG:C4QX25|C4QX25\_KOMPG:C4R5J3|C4R5J3\_KOMPG:C4QV49|C4QV49\_KOMPG:C4QXC9|C4QXC9\_KOMPG:C4R668|C4R668\_KOMPG:C4R8V8|C4R8V8\_KOMPG:C4QYN7|C4QYN7\_KOMPG:C4QZ42|C4QZ42\_KOMPG:C4R3M1|C4R3M1\_KOMPG:C4R8N0|C4R8N0\_KOMPG:C4QXZ3|C4QXZ3\_KOMPG |  |  | DB Search |
| LPLP | 20.33 | 438.2842 | 4 | -7.82 | 439.287 | 27.42 | 9635 | 1.51e3 | 1 | 1 | C4R2B1|C4R2B1\_KOMPG:C4R324|C4R324\_KOMPG:C4R2I2|C4R2I2\_KOMPG:C4QXW8|C4QXW8\_KOMPG:C4R357|C4R357\_KOMPG:C4R554|C4R554\_KOMPG:C4QWK5|C4QWK5\_KOMPG:C4QX46|C4QX46\_KOMPG:C4R5F4|C4R5F4\_KOMPG:C4QVC4|C4QVC4\_KOMPG:C4QZA0|C4QZA0\_KOMPG:C4QVD8|C4QVD8\_KOMPG:C4R6H3|LIS1\_KOMPG:C4R5Z7|C4R5Z7\_KOMPG:C4QVF3|C4QVF3\_KOMPG:C4QWK6|C4QWK6\_KOMPG:C4R4L4|C4R4L4\_KOMPG:C4QZS4|C4QZS4\_KOMPG:C4R4W5|C4R4W5\_KOMPG:C4R7M1|C4R7M1\_KOMPG:C4QX58|C4QX58\_KOMPG:C4R8T8|C4R8T8\_KOMPG:C4QVC9|C4QVC9\_KOMPG:C4QVX7|C4QVX7\_KOMPG:C4R280|C4R280\_KOMPG:C4R8S3|C4R8S3\_KOMPG:C4QYD4|C4QYD4\_KOMPG:C4R915|C4R915\_KOMPG:C4R5U2|C4R5U2\_KOMPG:C4R756|C4R756\_KOMPG:C4R0V8|C4R0V8\_KOMPG:C4R289|C4R289\_KOMPG:C4QZ19|C4QZ19\_KOMPG:C4R959|C4R959\_KOMPG:C4R0P8|C4R0P8\_KOMPG:C4R1N6|C4R1N6\_KOMPG:C4QX26|C4QX26\_KOMPG:C4QW71|C4QW71\_KOMPG:C4R1D7|C4R1D7\_KOMPG:C4QZ60|C4QZ60\_KOMPG:C4R723|C4R723\_KOMPG:C4R516|C4R516\_KOMPG:C4QVH2|C4QVH2\_KOMPG:C4QWS5|C4QWS5\_KOMPG:C4R3I0|C4R3I0\_KOMPG:C4R4X0|C4R4X0\_KOMPG:C4R8R6|C4R8R6\_KOMPG:C4R8Z1|C4R8Z1\_KOMPG:C4QXA2|C4QXA2\_KOMPG:C4R0T3|C4R0T3\_KOMPG:C4QWD1|C4QWD1\_KOMPG:C4R4X5|C4R4X5\_KOMPG:C4R7J4|C4R7J4\_KOMPG:C4R8H3|C4R8H3\_KOMPG:C4R5Y2|C4R5Y2\_KOMPG:C4QV16|C4QV16\_KOMPG:C4R207|C4R207\_KOMPG:C4R6E7|C4R6E7\_KOMPG |  |  | DB Search |
| GEGVI | 20.32 | 473.2485 | 5 | -0.97 | 474.2542 | 13.69 | 5382 | 4.72e3 | 1 | 1 | C4R3C7|C4R3C7\_KOMPG:C4R632|C4R632\_KOMPG |  |  | DB Search |
| DLTRGV | 20.27 | 659.3602 | 6 | -4.19 | 660.3631 | 8.19 | 3083 | 0 | 0 | 0 | C4R2U0|C4R2U0\_KOMPG |  |  | DB Search |
| DTVP | 20.25 | 430.2064 | 4 | -7.17 | 431.2095 | 8.44 | 3205 | 7.7e3 | 1 | 1 | C4R3A2|C4R3A2\_KOMPG:C4R3X4|C4R3X4\_KOMPG:C4QV76|C4QV76\_KOMPG:C4R6C9|C4R6C9\_KOMPG:C4R186|C4R186\_KOMPG:C4R444|C4R444\_KOMPG:C4QW65|C4QW65\_KOMPG:C4QW80|C4QW80\_KOMPG:C4R926|C4R926\_KOMPG:C4R5U1|C4R5U1\_KOMPG:C4QY57|C4QY57\_KOMPG:C4QZ83|C4QZ83\_KOMPG:C4QZF7|C4QZF7\_KOMPG:C4QZF2|C4QZF2\_KOMPG:C4QV09|C4QV09\_KOMPG:C4R6P7|C4R6P7\_KOMPG:C4QW55|C4QW55\_KOMPG:C4R9G4|C4R9G4\_KOMPG:C4R456|C4R456\_KOMPG:C4R2F9|C4R2F9\_KOMPG:C4R6U0|C4R6U0\_KOMPG:C4R1F2|C4R1F2\_KOMPG |  |  | DB Search |
| TPRHT | 20.25 | 610.3187 | 5 | -7.39 | 611.3199 | 19.60 | 7426 | 8.02e2 | 1 | 1 | C4R125|C4R125\_KOMPG:C4R8D2|C4R8D2\_KOMPG |  |  | DB Search |
| SIGD | 20.24 | 390.175 | 4 | -7.72 | 391.1783 | 3.30 | 1421 | 1.05e3 | 1 | 1 | C4R634|C4R634\_KOMPG:C4QVT8|C4QVT8\_KOMPG:C4R7Q0|C4R7Q0\_KOMPG:C4R8Z7|C4R8Z7\_KOMPG:C4R1G7|C4R1G7\_KOMPG:C4R6B6|C4R6B6\_KOMPG:C4R027|C4R027\_KOMPG:C4R6S7|C4R6S7\_KOMPG:C4QZJ5|C4QZJ5\_KOMPG:C4R580|C4R580\_KOMPG:C4R0N6|C4R0N6\_KOMPG:C4R1T5|C4R1T5\_KOMPG:C4QYA5|C4QYA5\_KOMPG:C4R4A2|C4R4A2\_KOMPG:C4R123|C4R123\_KOMPG:C4QYS8|C4QYS8\_KOMPG:C4QVE0|C4QVE0\_KOMPG:C4R0N2|C4R0N2\_KOMPG:C4R0A4|C4R0A4\_KOMPG:C4QYT0|C4QYT0\_KOMPG:C4R7W6|C4R7W6\_KOMPG:C4R3W8|C4R3W8\_KOMPG:C4QY15|C4QY15\_KOMPG:C4R178|C4R178\_KOMPG:C4R0J4|C4R0J4\_KOMPG:C4R0T3|C4R0T3\_KOMPG:C4R371|C4R371\_KOMPG:C4QW54|C4QW54\_KOMPG:C4R8G1|C4R8G1\_KOMPG:C4R605|C4R605\_KOMPG:C4R3U7|C4R3U7\_KOMPG:C4R693|C4R693\_KOMPG:C4QVK2|C4QVK2\_KOMPG:C4R814|C4R814\_KOMPG:C4QVT3|C4QVT3\_KOMPG:C4QZX4|C4QZX4\_KOMPG:C4R835|C4R835\_KOMPG:C4R267|C4R267\_KOMPG |  |  | DB Search |
| SLGD | 20.24 | 390.175 | 4 | -7.72 | 391.1783 | 3.30 | 1421 | 1.05e3 | 1 | 1 | C4QX19|C4QX19\_KOMPG:C4R9C4|C4R9C4\_KOMPG:C4R8H4|C4R8H4\_KOMPG:C4R8R9|C4R8R9\_KOMPG:C4QXW1|C4QXW1\_KOMPG:C4R4L8|C4R4L8\_KOMPG:C4R1B1|C4R1B1\_KOMPG:C4R7C2|C4R7C2\_KOMPG:C4QWN5|C4QWN5\_KOMPG:C4QYH4|C4QYH4\_KOMPG:C4QZD6|C4QZD6\_KOMPG:C4R1L3|C4R1L3\_KOMPG:C4R5J4|C4R5J4\_KOMPG:C4QYH0|C4QYH0\_KOMPG:C4R956|C4R956\_KOMPG:C4R284|C4R284\_KOMPG:C4QXM1|C4QXM1\_KOMPG:C4QYT3|C4QYT3\_KOMPG:C4QVD7|C4QVD7\_KOMPG:C4QWB5|C4QWB5\_KOMPG:C4R4B6|C4R4B6\_KOMPG:C4R153|C4R153\_KOMPG:C4R144|C4R144\_KOMPG:C4R722|C4R722\_KOMPG:C4QW21|C4QW21\_KOMPG:C4R1K9|C4R1K9\_KOMPG:C4QXT7|C4QXT7\_KOMPG:C4R3I8|C4R3I8\_KOMPG:C4R842|C4R842\_KOMPG:C4R7T1|C4R7T1\_KOMPG:C4QWV3|C4QWV3\_KOMPG:C4R0N1|C4R0N1\_KOMPG:C4R6N6|C4R6N6\_KOMPG:C4R505|C4R505\_KOMPG:C4QXN3|C4QXN3\_KOMPG:C4R620|C4R620\_KOMPG:C4R201|C4R201\_KOMPG:C4QV72|C4QV72\_KOMPG:C4R2X9|C4R2X9\_KOMPG:C4R899|C4R899\_KOMPG:C4R0Y6|C4R0Y6\_KOMPG:C4QVH9|C4QVH9\_KOMPG:C4R175|C4R175\_KOMPG:C4R4Y0|C4R4Y0\_KOMPG:C4R1G1|C4R1G1\_KOMPG:C4R243|C4R243\_KOMPG:C4R1Y9|C4R1Y9\_KOMPG:C4R1P7|C4R1P7\_KOMPG:C4R1F5|C4R1F5\_KOMPG:C4R686|C4R686\_KOMPG:C4R1V0|C4R1V0\_KOMPG:C4R5V4|C4R5V4\_KOMPG:C4QVB6|C4QVB6\_KOMPG:C4R4D0|C4R4D0\_KOMPG:C4R9A6|C4R9A6\_KOMPG:C4R6E8|C4R6E8\_KOMPG |  |  | DB Search |
| KFH | 20.21 | 430.2328 | 3 | 9.29 | 431.243 | 12.31 | 5009 | 5.31e3 | 1 | 1 | C4QYB2|C4QYB2\_KOMPG:C4R7Z0|C4R7Z0\_KOMPG:C4R2I2|C4R2I2\_KOMPG:C4QW28|C4QW28\_KOMPG:C4R414|C4R414\_KOMPG:C4R3B4|C4R3B4\_KOMPG:C4R6F7|C4R6F7\_KOMPG:C4QZG0|C4QZG0\_KOMPG:C4R669|C4R669\_KOMPG:C4R1P3|C4R1P3\_KOMPG:C4R4Q8|C4R4Q8\_KOMPG:C4R5Q8|C4R5Q8\_KOMPG:C4QWD7|C4QWD7\_KOMPG:C4R110|C4R110\_KOMPG:C4R737|C4R737\_KOMPG:C4R2L8|C4R2L8\_KOMPG:C4R874|C4R874\_KOMPG:C4QZ27|C4QZ27\_KOMPG:C4R0Q6|C4R0Q6\_KOMPG:C4QZ46|C4QZ46\_KOMPG:C4QVD7|C4QVD7\_KOMPG:C4QXJ6|C4QXJ6\_KOMPG:C4R571|C4R571\_KOMPG:C4R5J3|C4R5J3\_KOMPG:C4R4F8|C4R4F8\_KOMPG:C4R5R1|C4R5R1\_KOMPG:C4R8U4|C4R8U4\_KOMPG:C4QW26|C4QW26\_KOMPG:C4QYN9|C4QYN9\_KOMPG:C4R1B2|C4R1B2\_KOMPG:C4R4I8|C4R4I8\_KOMPG:C4R7S0|C4R7S0\_KOMPG:C4R2S1|C4R2S1\_KOMPG:C4QW21|C4QW21\_KOMPG:C4QW69|C4QW69\_KOMPG:C4R1A9|C4R1A9\_KOMPG:C4R0R2|C4R0R2\_KOMPG:C4QWV6|C4QWV6\_KOMPG:C4QX83|C4QX83\_KOMPG:C4R384|C4R384\_KOMPG:C4R2T7|C4R2T7\_KOMPG:C4R410|C4R410\_KOMPG:C4R0U9|C4R0U9\_KOMPG:C4R3V1|C4R3V1\_KOMPG:C4R141|C4R141\_KOMPG:C4QVL3|C4QVL3\_KOMPG:C4R5C4|C4R5C4\_KOMPG:C4QY33|C4QY33\_KOMPG:C4R630|C4R630\_KOMPG:C4QZT8|C4QZT8\_KOMPG:C4R1R4|C4R1R4\_KOMPG:C4R805|C4R805\_KOMPG:C4QV85|C4QV85\_KOMPG:C4R3V9|C4R3V9\_KOMPG:C4R3H1|C4R3H1\_KOMPG:C4R5W4|C4R5W4\_KOMPG:C4R1H3|C4R1H3\_KOMPG:C4QYG3|C4QYG3\_KOMPG:C4R7A6|C4R7A6\_KOMPG:C4R151|C4R151\_KOMPG:C4R194|C4R194\_KOMPG:C4QVG4|C4QVG4\_KOMPG:C4QWP7|C4QWP7\_KOMPG:C4R6G5|C4R6G5\_KOMPG:C4R7K8|C4R7K8\_KOMPG:C4QVW2|C4QVW2\_KOMPG:C4R553|C4R553\_KOMPG:C4R5E7|C4R5E7\_KOMPG:C4R6R0|C4R6R0\_KOMPG:C4QWL5|C4QWL5\_KOMPG:C4R270|C4R270\_KOMPG:C4R3Q1|C4R3Q1\_KOMPG:C4R6C8|C4R6C8\_KOMPG:C4QV83|C4QV83\_KOMPG:C4R2C1|C4R2C1\_KOMPG:C4QWP5|C4QWP5\_KOMPG:C4R0N9|C4R0N9\_KOMPG:C4QZH4|C4QZH4\_KOMPG:C4R7C7|C4R7C7\_KOMPG:C4R581|C4R581\_KOMPG:C4R4V0|C4R4V0\_KOMPG:C4QXQ1|C4QXQ1\_KOMPG:C4R2B7|C4R2B7\_KOMPG:C4R945|C4R945\_KOMPG:C4QZ81|C4QZ81\_KOMPG:C4R1R3|C4R1R3\_KOMPG:C4QVH6|C4QVH6\_KOMPG:C4R330|C4R330\_KOMPG:C4R844|C4R844\_KOMPG:C4R1K0|C4R1K0\_KOMPG:C4R419|C4R419\_KOMPG:C4R8R4|C4R8R4\_KOMPG:C4R1Q9|C4R1Q9\_KOMPG:C4QYS4|C4QYS4\_KOMPG:C4R535|C4R535\_KOMPG:C4R0U8|C4R0U8\_KOMPG:C4R6Q7|C4R6Q7\_KOMPG:C4R7K0|C4R7K0\_KOMPG:C4QYG5|C4QYG5\_KOMPG:C4QV51|C4QV51\_KOMPG:C4R4I9|C4R4I9\_KOMPG:C4QZY9|C4QZY9\_KOMPG:C4R259|C4R259\_KOMPG:C4QYZ1|C4QYZ1\_KOMPG:C4R2K7|C4R2K7\_KOMPG:C4R7U9|C4R7U9\_KOMPG:C4QVD8|C4QVD8\_KOMPG:C4R6X8|C4R6X8\_KOMPG:C4R8M0|C4R8M0\_KOMPG:C4R0T5|C4R0T5\_KOMPG:C4QVH5|C4QVH5\_KOMPG:C4R1C5|C4R1C5\_KOMPG:C4R624|C4R624\_KOMPG:C4QWZ6|C4QWZ6\_KOMPG:C4R7Z3|C9MT\_KOMPG:C4R4Q1|C4R4Q1\_KOMPG:C4R6U8|C4R6U8\_KOMPG:C4R409|C4R409\_KOMPG:C4QZY8|C4QZY8\_KOMPG:C4R3W4|C4R3W4\_KOMPG:C4QYG9|C4QYG9\_KOMPG:C4R166|C4R166\_KOMPG:C4R2G0|C4R2G0\_KOMPG:C4R877|C4R877\_KOMPG:C4R610|C4R610\_KOMPG:C4QXH0|C4QXH0\_KOMPG:C4QX89|C4QX89\_KOMPG:C4QXT1|C4QXT1\_KOMPG:C4R591|STS1\_KOMPG:C4R411|C4R411\_KOMPG:C4QVB8|C4QVB8\_KOMPG:C4R8U6|C4R8U6\_KOMPG:C4QXI8|PEX6\_KOMPG:C4R0H1|C4R0H1\_KOMPG:C4R8H1|C4R8H1\_KOMPG:C4R5Q5|C4R5Q5\_KOMPG:C4QYN1|C4QYN1\_KOMPG:C4R0X5|C4R0X5\_KOMPG:C4R0G8|C4R0G8\_KOMPG:C4R0A6|C4R0A6\_KOMPG:C4QZE1|C4QZE1\_KOMPG:C4R2U0|C4R2U0\_KOMPG:C4R2A6|C4R2A6\_KOMPG:C4QXX4|C4QXX4\_KOMPG:C4QW18|C4QW18\_KOMPG:C4R2M3|C4R2M3\_KOMPG:C4R6Q6|C4R6Q6\_KOMPG:C4QXL7|C4QXL7\_KOMPG:C4QUZ6|C4QUZ6\_KOMPG:C4QVK6|C4QVK6\_KOMPG:C4R5W7|C4R5W7\_KOMPG:C4R3R1|C4R3R1\_KOMPG:C4QVE3|C4QVE3\_KOMPG:C4QW65|C4QW65\_KOMPG:C4R8R6|C4R8R6\_KOMPG:C4QWC7|C4QWC7\_KOMPG:C4R2Z4|C4R2Z4\_KOMPG:C4R995|C4R995\_KOMPG:C4R7H4|PFKA3\_KOMPG:C4R2T4|C4R2T4\_KOMPG:C4R1D3|C4R1D3\_KOMPG:C4R140|C4R140\_KOMPG:C4R5E1|C4R5E1\_KOMPG:C4R1I8|C4R1I8\_KOMPG:C4QVH1|C4QVH1\_KOMPG:C4R360|PSD2\_KOMPG:C4R721|C4R721\_KOMPG:C4QYJ3|C4QYJ3\_KOMPG:C4R1Z6|C4R1Z6\_KOMPG |  |  | DB Search |
| PVIVP | 20.2 | 523.337 | 5 | -6.37 | 524.3396 | 27.22 | 9490 | 8.88e3 | 1 | 1 | C4R4V6|C4R4V6\_KOMPG |  |  | DB Search |
| PVLVP | 20.2 | 523.337 | 5 | -6.37 | 524.3396 | 27.22 | 9490 | 8.88e3 | 1 | 1 | C4R317|C4R317\_KOMPG:C4R1Q4|C4R1Q4\_KOMPG:C4R7N8|C4R7N8\_KOMPG:C4QYD0|C4QYD0\_KOMPG |  |  | DB Search |
| FAR | 20.16 | 392.2172 | 3 | -6.48 | 393.2209 | 7.32 | 2792 | 7.59e2 | 1 | 1 | C4R2U7|C4R2U7\_KOMPG:C4QZK7|C4QZK7\_KOMPG:C4R712|C4R712\_KOMPG:C4QWK2|C4QWK2\_KOMPG:C4QWF0|C4QWF0\_KOMPG:C4R1S0|C4R1S0\_KOMPG:C4QZB7|C4QZB7\_KOMPG:C4QVN1|C4QVN1\_KOMPG:C4R042|C4R042\_KOMPG:C4QWD1|C4QWD1\_KOMPG:C4R0G8|C4R0G8\_KOMPG:C4R2G2|C4R2G2\_KOMPG:C4R0H3|C4R0H3\_KOMPG |  |  | DB Search |
| GYYP | 20.14 | 498.2114 | 4 | 1.33 | 499.2181 | 14.02 | 5579 | 4.82e2 | 1 | 1 | C4QY66|C4QY66\_KOMPG:C4QVY7|C4QVY7\_KOMPG:C4QZM5|C4QZM5\_KOMPG:C4R919|C4R919\_KOMPG:C4R4G2|C4R4G2\_KOMPG:C4R511|C4R511\_KOMPG:C4R4X2|C4R4X2\_KOMPG:C4R2V7|C4R2V7\_KOMPG:C4R4N3|C4R4N3\_KOMPG:C4R431|C4R431\_KOMPG:C4R0J6|C4R0J6\_KOMPG:C4R040|C4R040\_KOMPG:C4R960|C4R960\_KOMPG |  |  | DB Search |
| NGSS | 20.12 | 363.139 | 4 | -0.16 | 364.1453 | 14.82 | 5815 | 1.67e3 | 1 | 1 | C4QVM2|C4QVM2\_KOMPG:C4R7F7|C4R7F7\_KOMPG:C4R0V7|C4R0V7\_KOMPG:C4QXP6|C4QXP6\_KOMPG:C4R3R4|C4R3R4\_KOMPG:C4QW03|C4QW03\_KOMPG:C4QV31|C4QV31\_KOMPG:C4R214|C4R214\_KOMPG:C4R5L8|C4R5L8\_KOMPG:C4QY40|C4QY40\_KOMPG:C4R4G8|C4R4G8\_KOMPG:C4R511|C4R511\_KOMPG:C4R1E9|C4R1E9\_KOMPG:C4QXH4|C4QXH4\_KOMPG:C4QZ15|C4QZ15\_KOMPG:C4R3G3|C4R3G3\_KOMPG:C4R0N7|C4R0N7\_KOMPG:C4QXN8|C4QXN8\_KOMPG:C4R3R6|C4R3R6\_KOMPG:C4QXQ6|C4QXQ6\_KOMPG:C4QWA3|C4QWA3\_KOMPG:C4QY84|C4QY84\_KOMPG:C4QYZ1|C4QYZ1\_KOMPG:C4QX39|C4QX39\_KOMPG:C4QVK9|C4QVK9\_KOMPG:C4QVD8|C4QVD8\_KOMPG:C4R0B1|C4R0B1\_KOMPG:C4QXU3|C4QXU3\_KOMPG:C4QW58|C4QW58\_KOMPG:C4R2R9|C4R2R9\_KOMPG:C4R041|C4R041\_KOMPG:C4QZ09|C4QZ09\_KOMPG:C4QZF1|C4QZF1\_KOMPG:C4QYA4|C4QYA4\_KOMPG:C4QXR0|C4QXR0\_KOMPG:C4R132|C4R132\_KOMPG:C4R1W9|C4R1W9\_KOMPG:C4QXP4|C4QXP4\_KOMPG:C4R5A7|C4R5A7\_KOMPG:C4R2F3|C4R2F3\_KOMPG:C4R6G8|C4R6G8\_KOMPG:C4R3U0|GET2\_KOMPG:C4QZE2|C4QZE2\_KOMPG:C4R339|C4R339\_KOMPG:C4R744|C4R744\_KOMPG:C4R380|C4R380\_KOMPG:C4QWD9|C4QWD9\_KOMPG:C4R485|C4R485\_KOMPG:C4R928|C4R928\_KOMPG:C4R4K0|C4R4K0\_KOMPG:C4R0P8|C4R0P8\_KOMPG:C4QXA9|C4QXA9\_KOMPG:C4QW18|C4QW18\_KOMPG:C4R1V5|C4R1V5\_KOMPG:C4QWP2|C4QWP2\_KOMPG:C4R703|C4R703\_KOMPG:C4R7P8|C4R7P8\_KOMPG:C4R8Y8|C4R8Y8\_KOMPG:C4R1I3|C4R1I3\_KOMPG:C4QX81|C4QX81\_KOMPG:C4R0D1|C4R0D1\_KOMPG:C4QW96|C4QW96\_KOMPG:C4R8C5|C4R8C5\_KOMPG:C4R0D0|C4R0D0\_KOMPG:C4R8Z2|C4R8Z2\_KOMPG:C4R981|C4R981\_KOMPG:C4QZF2|C4QZF2\_KOMPG:C4R1D3|C4R1D3\_KOMPG:C4R3W0|C4R3W0\_KOMPG:C4R4R1|C4R4R1\_KOMPG:C4QV24|C4QV24\_KOMPG:C4QWV4|C4QWV4\_KOMPG:C4R6M2|C4R6M2\_KOMPG:C4R2Z9|C4R2Z9\_KOMPG:C4QYE7|C4QYE7\_KOMPG:C4R3H2|C4R3H2\_KOMPG:C4R6Z4|C4R6Z4\_KOMPG:C4R5M3|C4R5M3\_KOMPG:C4R458|C4R458\_KOMPG |  |  | DB Search |
| VTTI | 20.12 | 432.2584 | 4 | -4.38 | 433.2627 | 11.15 | 4417 | 0 | 0 | 0 | C4QVJ2|C4QVJ2\_KOMPG:C4R450|C4R450\_KOMPG:C4R7Q7|C4R7Q7\_KOMPG:C4QZW7|C4QZW7\_KOMPG:C4QX41|C4QX41\_KOMPG:C4R0X4|C4R0X4\_KOMPG:C4R310|C4R310\_KOMPG:C4R3K7|C4R3K7\_KOMPG:C4QYR2|C4QYR2\_KOMPG:C4R7J9|C4R7J9\_KOMPG:C4QZT8|C4QZT8\_KOMPG:C4QXR4|C4QXR4\_KOMPG:C4QW81|C4QW81\_KOMPG:C4QZL5|C4QZL5\_KOMPG:C4R5Q8|C4R5Q8\_KOMPG:C4R588|C4R588\_KOMPG:C4R9C5|C4R9C5\_KOMPG:C4R5H5|C4R5H5\_KOMPG:C4R3W6|C4R3W6\_KOMPG:C4QXV0|C4QXV0\_KOMPG:C4R1C2|C4R1C2\_KOMPG:C4QZA7|C4QZA7\_KOMPG:C4R7V1|C4R7V1\_KOMPG:C4R615|C4R615\_KOMPG:C4QXI6|C4QXI6\_KOMPG:C4R439|C4R439\_KOMPG:C4QVX7|C4QVX7\_KOMPG:C4R5T6|C4R5T6\_KOMPG:C4R666|C4R666\_KOMPG:C4QZM6|C4QZM6\_KOMPG:C4QWG7|C4QWG7\_KOMPG |  |  | DB Search |
| VTTL | 20.12 | 432.2584 | 4 | -4.38 | 433.2627 | 11.15 | 4417 | 0 | 0 | 0 | C4QVM2|C4QVM2\_KOMPG:C4R9D9|C4R9D9\_KOMPG:C4QVY3|C4QVY3\_KOMPG:C4QVN9|C4QVN9\_KOMPG:C4QZ44|C4QZ44\_KOMPG:C4QWW6|C4QWW6\_KOMPG:C4R3D3|C4R3D3\_KOMPG:C4QV09|C4QV09\_KOMPG:C4R6I8|C4R6I8\_KOMPG:C4QVJ7|C4QVJ7\_KOMPG:C4R622|C4R622\_KOMPG:C4R7R3|C4R7R3\_KOMPG:C4R8W8|C4R8W8\_KOMPG:C4R2T5|C4R2T5\_KOMPG:C4QX98|C4QX98\_KOMPG:C4R4A5|C4R4A5\_KOMPG:C4R484|C4R484\_KOMPG:C4QWL8|C4QWL8\_KOMPG:C4QXU5|C4QXU5\_KOMPG:C4QYW0|C4QYW0\_KOMPG:C4QZS4|C4QZS4\_KOMPG:C4QZU4|C4QZU4\_KOMPG:C4QYS1|C4QYS1\_KOMPG:C4R6P4|C4R6P4\_KOMPG:C4R044|C4R044\_KOMPG:C4R091|C4R091\_KOMPG:C4R6Q1|C4R6Q1\_KOMPG:C4R867|C4R867\_KOMPG:C4R6X5|C4R6X5\_KOMPG:C4R839|C4R839\_KOMPG:C4R2U6|C4R2U6\_KOMPG:C4R8V0|C4R8V0\_KOMPG:C4R8D5|C4R8D5\_KOMPG:C4R597|C4R597\_KOMPG:C4R869|C4R869\_KOMPG:C4QWQ3|C4QWQ3\_KOMPG:C4R0V2|C4R0V2\_KOMPG:C4QXA5|PFKA2\_KOMPG:C4QZB6|C4QZB6\_KOMPG:C4R3K9|C4R3K9\_KOMPG:C4R4T1|C4R4T1\_KOMPG:C4R024|C4R024\_KOMPG:C4R911|C4R911\_KOMPG:C4QX80|PSD1\_KOMPG:C4R6S4|C4R6S4\_KOMPG:C4R1C7|C4R1C7\_KOMPG:C4QYF1|C4QYF1\_KOMPG:C4R6U0|C4R6U0\_KOMPG:C4QXE9|CHO2\_KOMPG |  |  | DB Search |
| SSD | 20.1 | 307.1016 | 3 | 8.02 | 308.1105 | 10.67 | 4164 | 0 | 0 | 0 | C4QXT2|C4QXT2\_KOMPG:C4R8U9|C4R8U9\_KOMPG:C4QYE1|C4QYE1\_KOMPG:C4R291|C4R291\_KOMPG:C4R0H0|C4R0H0\_KOMPG:C4QYR2|C4QYR2\_KOMPG:C4QZZ8|C4QZZ8\_KOMPG:C4R1R3|C4R1R3\_KOMPG:C4R003|C4R003\_KOMPG:C4R3I7|C4R3I7\_KOMPG:C4R1N5|C4R1N5\_KOMPG:C4R011|C4R011\_KOMPG:C4R1P8|C4R1P8\_KOMPG:C4QY05|C4QY05\_KOMPG:C4QWA3|C4QWA3\_KOMPG:C4QVQ7|C4QVQ7\_KOMPG:C4QWS8|PEX36\_KOMPG:C4R6B2|C4R6B2\_KOMPG:C4QVT4|C4QVT4\_KOMPG:P53024|SEC13\_KOMPG:C4QZ71|C4QZ71\_KOMPG:C4R6G3|C4R6G3\_KOMPG:C4R7J8|C4R7J8\_KOMPG:C4R2C5|C4R2C5\_KOMPG:C4QXU3|C4QXU3\_KOMPG:C4R4F0|C4R4F0\_KOMPG:C4QVD3|C4QVD3\_KOMPG:C4QZH8|C4QZH8\_KOMPG:C4QYT3|C4QYT3\_KOMPG:C4R042|C4R042\_KOMPG:C4QVM0|C4QVM0\_KOMPG:C4R5J2|C4R5J2\_KOMPG:C4R349|C4R349\_KOMPG:C4R4L4|C4R4L4\_KOMPG:C4R0P1|C4R0P1\_KOMPG:C4QWH2|C4QWH2\_KOMPG:C4QYQ0|C4QYQ0\_KOMPG:C4R8I1|C4R8I1\_KOMPG:C4R2U9|C4R2U9\_KOMPG:C4QVT0|C4QVT0\_KOMPG:C4R366|C4R366\_KOMPG:C4QV97|C4QV97\_KOMPG:C4R033|C4R033\_KOMPG:C4QVP0|C4QVP0\_KOMPG:C4R5I2|C4R5I2\_KOMPG:C4R990|C4R990\_KOMPG:C4R684|C4R684\_KOMPG:C4QZ47|C4QZ47\_KOMPG:C4QXL0|C4QXL0\_KOMPG:C4R877|C4R877\_KOMPG:C4R8S4|C4R8S4\_KOMPG:C4R7N8|C4R7N8\_KOMPG:C4R5C7|C4R5C7\_KOMPG:C4R0X4|C4R0X4\_KOMPG:C4R5P8|C4R5P8\_KOMPG:C4R148|C4R148\_KOMPG:C4R0V8|C4R0V8\_KOMPG:C4QZ69|C4QZ69\_KOMPG:C4R848|C4R848\_KOMPG:C4R215|C4R215\_KOMPG:C4R4F7|C4R4F7\_KOMPG:C4R3C3|C4R3C3\_KOMPG:C4QV45|C4QV45\_KOMPG:C4R7I5|C4R7I5\_KOMPG:C4R4K5|C4R4K5\_KOMPG:C4QVF1|C4QVF1\_KOMPG:C4R370|C4R370\_KOMPG:C4R165|C4R165\_KOMPG:C4QY03|C4QY03\_KOMPG:C4R7C8|C4R7C8\_KOMPG:C4R4T5|C4R4T5\_KOMPG:C4R240|C4R240\_KOMPG:C4QYN8|C4QYN8\_KOMPG:C4QWD1|C4QWD1\_KOMPG:C4R4U3|C4R4U3\_KOMPG:C4R3L2|C4R3L2\_KOMPG:C4R047|C4R047\_KOMPG:C4R5V4|C4R5V4\_KOMPG:C4QXS6|C4QXS6\_KOMPG:C4R5H2|C4R5H2\_KOMPG:C4QY10|C4QY10\_KOMPG:C4R3H2|C4R3H2\_KOMPG:C4R721|C4R721\_KOMPG:C4R322|C4R322\_KOMPG |  |  | DB Search |
| ISVGI | 20.08 | 487.3006 | 5 | -4.27 | 488.3046 | 21.68 | 7886 | 8.06e3 | 1 | 1 | C4QYA5|C4QYA5\_KOMPG:C4R6G9|C4R6G9\_KOMPG:C4QXD4|C4QXD4\_KOMPG:C4QVN9|C4QVN9\_KOMPG:C4QZ78|C4QZ78\_KOMPG |  |  | DB Search |
| ISVGL | 20.08 | 487.3006 | 5 | -4.27 | 488.3046 | 21.68 | 7886 | 8.06e3 | 1 | 1 | C4R8B0|C4R8B0\_KOMPG:C4QV24|C4QV24\_KOMPG:C4R4S1|C4R4S1\_KOMPG:C4R1Y9|C4R1Y9\_KOMPG:C4R2C4|C4R2C4\_KOMPG:C4R8H2|C4R8H2\_KOMPG |  |  | DB Search |
| LSVGI | 20.08 | 487.3006 | 5 | -4.27 | 488.3046 | 21.68 | 7886 | 8.06e3 | 1 | 1 | C4R5R3|C4R5R3\_KOMPG:C4R8B1|C4R8B1\_KOMPG:C4QZU2|C4QZU2\_KOMPG:C4R2Q9|C4R2Q9\_KOMPG:C4R368|C4R368\_KOMPG:C4R8B5|C4R8B5\_KOMPG:C4QYL9|C4QYL9\_KOMPG |  |  | DB Search |
| LSVGL | 20.08 | 487.3006 | 5 | -4.27 | 488.3046 | 21.68 | 7886 | 8.06e3 | 1 | 1 | C4R1S7|C4R1S7\_KOMPG:C4R832|C4R832\_KOMPG:C4R6B7|C4R6B7\_KOMPG |  |  | DB Search |
| WSD | 19.99 | 406.1488 | 3 | -8.45 | 407.1517 | 8.75 | 3390 | 0 | 0 | 0 | C4R5V1|C4R5V1\_KOMPG:C4R0Z1|C4R0Z1\_KOMPG:Q9Y751|ATG26\_KOMPG:C4R5E2|C4R5E2\_KOMPG:C4R0K6|C4R0K6\_KOMPG:C4R5F0|C4R5F0\_KOMPG:C4R2B7|C4R2B7\_KOMPG:C4QWW6|C4QWW6\_KOMPG:C4QVW5|C4QVW5\_KOMPG:C4R9B4|C4R9B4\_KOMPG:C4R0W9|C4R0W9\_KOMPG:C4R4Q8|C4R4Q8\_KOMPG:C4QYZ7|C4QYZ7\_KOMPG:C4R747|C4R747\_KOMPG:C4R138|C4R138\_KOMPG:C4R422|C4R422\_KOMPG:C4QX76|C4QX76\_KOMPG:C4QV51|C4QV51\_KOMPG:C4R726|C4R726\_KOMPG:C4QV14|C4QV14\_KOMPG:C4QW94|C4QW94\_KOMPG:C4R347|C4R347\_KOMPG:C4QZH5|C4QZH5\_KOMPG:C4R650|C4R650\_KOMPG:C4QZA0|C4QZA0\_KOMPG:C4R9F2|C4R9F2\_KOMPG:C4QYS8|C4QYS8\_KOMPG:C4R628|PFF1\_KOMPG:C4R0U3|C4R0U3\_KOMPG:C4QZ27|C4QZ27\_KOMPG:C4R4R6|C4R4R6\_KOMPG:C4R048|C4R048\_KOMPG:C4QY15|C4QY15\_KOMPG:C4QVZ1|C4QVZ1\_KOMPG:C4QZR8|C4QZR8\_KOMPG:C4QWR1|C4QWR1\_KOMPG:C4R5X8|C4R5X8\_KOMPG:C4R354|C4R354\_KOMPG:C4R3W2|C4R3W2\_KOMPG:C4QVJ1|C4QVJ1\_KOMPG:C4QZ33|C4QZ33\_KOMPG:C4R0E0|C4R0E0\_KOMPG:C4R7T5|C4R7T5\_KOMPG:C4QYG4|C4QYG4\_KOMPG:C4QVV6|C4QVV6\_KOMPG:C4QZW5|C4QZW5\_KOMPG:C4QXZ2|C4QXZ2\_KOMPG:C4QZ03|C4QZ03\_KOMPG:C4R6L2|C4R6L2\_KOMPG:C4R8I7|C4R8I7\_KOMPG:C4R9C8|C4R9C8\_KOMPG:C4QZ83|C4QZ83\_KOMPG:C4R3D8|C4R3D8\_KOMPG:C4QV72|C4QV72\_KOMPG:C4R1K1|C4R1K1\_KOMPG:C4QWX2|C4QWX2\_KOMPG:C4R352|C4R352\_KOMPG:C4R1Y3|C4R1Y3\_KOMPG:C4R4R4|C4R4R4\_KOMPG:C4R134|C4R134\_KOMPG:C4R4Y4|C4R4Y4\_KOMPG:C4R960|C4R960\_KOMPG:C4R6W3|C4R6W3\_KOMPG:C4R0K4|C4R0K4\_KOMPG:C4QYI7|C4QYI7\_KOMPG:C4R0F1|C4R0F1\_KOMPG:C4R7L1|C4R7L1\_KOMPG:C4R126|C4R126\_KOMPG:C4QZ94|C4QZ94\_KOMPG:C4R1A4|C4R1A4\_KOMPG:C4R4V5|C4R4V5\_KOMPG:C4R7B3|C4R7B3\_KOMPG:C4QZB8|C4QZB8\_KOMPG:C4R795|C4R795\_KOMPG:C4R2Q6|C4R2Q6\_KOMPG:C4QXW0|C4QXW0\_KOMPG:C4QV00|C4QV00\_KOMPG:C4QXX3|C4QXX3\_KOMPG:C4R8E3|C4R8E3\_KOMPG:C4QY08|C4QY08\_KOMPG:C4QWN1|C4QWN1\_KOMPG:C4R682|C4R682\_KOMPG:C4R8Y1|C4R8Y1\_KOMPG |  |  | DB Search |
| GQCTDGS | 19.96 | 666.2279 | 7 | -3.85 | 667.231 | 3.20 | 1375 | 0 | 0 | 0 | C4R564|C4R564\_KOMPG |  |  | DB Search |
| GRSH | 19.96 | 455.2241 | 4 | 7.47 | 456.2336 | 3.17 | 1412 | 2.02e3 | 1 | 1 | C4R6S6|C4R6S6\_KOMPG:C4QXC3|C4QXC3\_KOMPG:C4R5R1|C4R5R1\_KOMPG:C4QXM8|C4QXM8\_KOMPG:C4R668|C4R668\_KOMPG:C4QZ77|C4QZ77\_KOMPG:C4QZ08|C4QZ08\_KOMPG:C4R1Q7|C4R1Q7\_KOMPG |  |  | DB Search |
| HHY | 19.96 | 455.1917 | 3 | -1.96 | 456.197 | 6.88 | 2643 | 0 | 0 | 0 | C4R7K5|C4R7K5\_KOMPG:C4R6I4|C4R6I4\_KOMPG:C4R864|C4R864\_KOMPG:C4QWN8|C4QWN8\_KOMPG:C4QWJ0|C4QWJ0\_KOMPG:C4QVL0|C4QVL0\_KOMPG:C4R049|C4R049\_KOMPG:C4R8T0|C4R8T0\_KOMPG:C4QZZ6|C4QZZ6\_KOMPG:C4R5Q8|C4R5Q8\_KOMPG:C4R989|C4R989\_KOMPG:C4R8D4|C4R8D4\_KOMPG:C4QXH3|C4QXH3\_KOMPG:C4QYY2|C4QYY2\_KOMPG:C4QVX4|C4QVX4\_KOMPG:C4QV14|C4QV14\_KOMPG:C4QXZ6|C4QXZ6\_KOMPG:C4QVH0|C4QVH0\_KOMPG:C4QV42|C4QV42\_KOMPG:C4R348|C4R348\_KOMPG:C4QYL7|AIM9\_KOMPG:C4R8W7|C4R8W7\_KOMPG:C4R2D5|C4R2D5\_KOMPG:C4R3E6|C4R3E6\_KOMPG:C4R2C7|C4R2C7\_KOMPG:C4R810|C4R810\_KOMPG:C4QZF1|C4QZF1\_KOMPG:C4R2P6|C4R2P6\_KOMPG:C4QZQ6|C4QZQ6\_KOMPG:C4R098|C4R098\_KOMPG:C4R8M8|C4R8M8\_KOMPG:C4R5K2|C4R5K2\_KOMPG:C4QVZ2|C4QVZ2\_KOMPG:C4R078|C4R078\_KOMPG:C4R7B5|C4R7B5\_KOMPG:C4R6I5|C4R6I5\_KOMPG:C4R3F4|C4R3F4\_KOMPG:C4R044|C4R044\_KOMPG:C4QVH3|C4QVH3\_KOMPG:C4R6Q5|C4R6Q5\_KOMPG:C4R7D2|C4R7D2\_KOMPG:C4R0Q7|C4R0Q7\_KOMPG:C4R2A6|C4R2A6\_KOMPG:C4R4U9|C4R4U9\_KOMPG:C4R1H3|C4R1H3\_KOMPG:C4R5A1|C4R5A1\_KOMPG:C4R4R4|C4R4R4\_KOMPG:C4R6L0|C4R6L0\_KOMPG:C4R6G5|C4R6G5\_KOMPG:C4QVK6|C4QVK6\_KOMPG:C4QXQ7|C4QXQ7\_KOMPG:C4QXX0|C4QXX0\_KOMPG:C4R252|C4R252\_KOMPG:C4R735|C4R735\_KOMPG:C4QYA2|C4QYA2\_KOMPG:C4R140|C4R140\_KOMPG:C4R7X3|C4R7X3\_KOMPG:C4R3J8|C4R3J8\_KOMPG:C4R6H2|C4R6H2\_KOMPG |  |  | DB Search |
| PGSAQSY | 19.96 | 708.3079 | 7 | -3.22 | 709.3111 | 6.91 | 2679 | 2.49e3 | 1 | 1 | C4R614|C4R614\_KOMPG |  |  | DB Search |
| RAMY | 19.94 | 539.2526 | 4 | 0.36 | 540.2587 | 13.80 | 5440 | 1.04e3 | 1 | 1 | C4R4V8|C4R4V8\_KOMPG:C4R7Q0|C4R7Q0\_KOMPG:C4R7Z8|BMT4\_KOMPG |  |  | DB Search |
| LCSLYSSQF | 19.92 | 1046.4742 | 9 | -2.99 | 1047.4758 | 35.32 | 11768 | 1.02e2 | 2 | 2 | C4QZJ3|C4QZJ3\_KOMPG |  |  | DB Search |
| VVDI | 19.9 | 444.2584 | 4 | 0.77 | 445.2649 | 17.32 | 6776 | 2.15e3 | 1 | 1 | C4R7J1|C4R7J1\_KOMPG:C4R5U8|C4R5U8\_KOMPG:C4QZX9|C4QZX9\_KOMPG:C4R214|C4R214\_KOMPG:C4QVH3|C4QVH3\_KOMPG:C4R3D0|C4R3D0\_KOMPG:C4QV87|C4QV87\_KOMPG:C4QXB1|C4QXB1\_KOMPG:C4QWY8|AIM36\_KOMPG:C4QVS9|C4QVS9\_KOMPG:C4QWF2|C4QWF2\_KOMPG:C4QZC1|C4QZC1\_KOMPG:C4R848|C4R848\_KOMPG:C4QW55|C4QW55\_KOMPG:C4QXQ6|C4QXQ6\_KOMPG:C4R8V0|C4R8V0\_KOMPG:C4R0J2|C4R0J2\_KOMPG:C4R1C8|C4R1C8\_KOMPG:C4R3C5|C4R3C5\_KOMPG:C4R126|C4R126\_KOMPG:C4R621|C4R621\_KOMPG:C4QVM0|C4QVM0\_KOMPG:C4R3H7|C4R3H7\_KOMPG:C4R653|C4R653\_KOMPG:C4R094|C4R094\_KOMPG:C4R7M5|C4R7M5\_KOMPG:C4R3T0|C4R3T0\_KOMPG:C4R5H3|C4R5H3\_KOMPG:C4R075|C4R075\_KOMPG:C4R1V8|C4R1V8\_KOMPG:C4R2P6|C4R2P6\_KOMPG:C4R6H4|C4R6H4\_KOMPG:C4R7Z4|C4R7Z4\_KOMPG:C4R4F9|C4R4F9\_KOMPG:C4QVT3|C4QVT3\_KOMPG:C4R8E3|C4R8E3\_KOMPG:C4R2H7|C4R2H7\_KOMPG:C4R6N4|C4R6N4\_KOMPG:C4QZ41|C4QZ41\_KOMPG:C4R0L0|C4R0L0\_KOMPG:C4R0R2|C4R0R2\_KOMPG |  |  | DB Search |
| VVDL | 19.9 | 444.2584 | 4 | 0.77 | 445.2649 | 17.32 | 6776 | 2.15e3 | 1 | 1 | C4QXE5|C4QXE5\_KOMPG:C4R7K7|C4R7K7\_KOMPG:C4QVP4|C4QVP4\_KOMPG:C4R1D2|C4R1D2\_KOMPG:C4R2I1|C4R2I1\_KOMPG:C4R343|C4R343\_KOMPG:C4QWC4|C4QWC4\_KOMPG:C4R8R3|C4R8R3\_KOMPG:C4QWY5|C4QWY5\_KOMPG:C4R3T4|C4R3T4\_KOMPG:C4R3M2|C4R3M2\_KOMPG:C4R5F4|C4R5F4\_KOMPG:C4R4H6|C4R4H6\_KOMPG:C4QY84|C4QY84\_KOMPG:C4R622|C4R622\_KOMPG:C4QW07|C4QW07\_KOMPG:C4QXD1|C4QXD1\_KOMPG:C4R5Z7|C4R5Z7\_KOMPG:C4R5Q7|C4R5Q7\_KOMPG:C4QY48|C4QY48\_KOMPG:C4R6B0|OXDD\_KOMPG:C4QVS8|C4QVS8\_KOMPG:C4R1T9|ENOPH\_KOMPG:C4R4R8|ARO1\_KOMPG:C4R100|C4R100\_KOMPG:C4R778|C4R778\_KOMPG:C4R0P1|C4R0P1\_KOMPG:C4R5K6|C4R5K6\_KOMPG:C4R3D9|C4R3D9\_KOMPG:C4R8T8|C4R8T8\_KOMPG:C4QXQ5|C4QXQ5\_KOMPG:C4R098|C4R098\_KOMPG:C4QY26|C4QY26\_KOMPG:C4QW69|C4QW69\_KOMPG:C4R5Z2|C4R5Z2\_KOMPG:C4R8W2|C4R8W2\_KOMPG:C4R154|C4R154\_KOMPG:C4R667|C4R667\_KOMPG:C4R3D7|C4R3D7\_KOMPG:C4QXF7|C4QXF7\_KOMPG:C4R044|C4R044\_KOMPG:C4R842|C4R842\_KOMPG:C4R2A1|C4R2A1\_KOMPG:C4R8T1|C4R8T1\_KOMPG:C4R201|C4R201\_KOMPG:C4R2N7|C4R2N7\_KOMPG:C4R2N5|C4R2N5\_KOMPG:C4R0N6|C4R0N6\_KOMPG:C4QXS3|C4QXS3\_KOMPG:C4R0W4|C4R0W4\_KOMPG:C4R1U8|C4R1U8\_KOMPG:C4QXM9|C4QXM9\_KOMPG:C4R8D5|C4R8D5\_KOMPG:C4R4T6|C4R4T6\_KOMPG:C4QW71|C4QW71\_KOMPG:C4QYA5|C4QYA5\_KOMPG:C4R2N3|C4R2N3\_KOMPG:C4R1N8|C4R1N8\_KOMPG:C4R393|C4R393\_KOMPG:C4R7L1|C4R7L1\_KOMPG:C4R5S5|C4R5S5\_KOMPG:C4R947|C4R947\_KOMPG:C4R8Z2|C4R8Z2\_KOMPG:C4R700|C4R700\_KOMPG:C4R6P7|C4R6P7\_KOMPG:C4R7I8|C4R7I8\_KOMPG:C4QW79|C4QW79\_KOMPG:C4QXX3|C4QXX3\_KOMPG:C4QZ22|C4QZ22\_KOMPG:C4R890|C4R890\_KOMPG:C4R8V3|C4R8V3\_KOMPG |  |  | DB Search |
| PQQQ(+0.98)PQ(+0.98)QQPP | 19.9 | 1176.5411 | 10 | 4.97 | 589.2793 | 24.08 | 8743 | 3.75e3 | 1 | 1 | C4QYQ4|C4QYQ4\_KOMPG | Deamidation (NQ), Deamidation (NQ) | Q4:Deamidation (NQ):0 Q6:Deamidation (NQ):30.46 | DB Search |
| PSTIQ | 19.87 | 544.2856 | 5 | -2.52 | 545.2902 | 11.22 | 4444 | 3.37e3 | 1 | 1 | C4R6C9|C4R6C9\_KOMPG:C4R9F5|C4R9F5\_KOMPG:C4R0Z1|C4R0Z1\_KOMPG:C4QVP9|C4QVP9\_KOMPG:C4QXI3|C4QXI3\_KOMPG |  |  | DB Search |
| PSTLQ | 19.87 | 544.2856 | 5 | -2.52 | 545.2902 | 11.22 | 4444 | 3.37e3 | 1 | 1 | C4R908|C4R908\_KOMPG:C4R4R1|C4R4R1\_KOMPG |  |  | DB Search |
| LAGSP | 19.85 | 443.238 | 5 | -4.02 | 444.2424 | 5.55 | 2243 | 5.62e3 | 1 | 1 | C4R5I1|C4R5I1\_KOMPG:C4R836|C4R836\_KOMPG |  |  | DB Search |
| IVPSVAPA | 19.84 | 752.4432 | 8 | -6.42 | 753.4438 | 25.52 | 9111 | 4.27e2 | 1 | 1 | C4R7A9|C4R7A9\_KOMPG |  |  | DB Search |
| PQFPP | 19.84 | 584.2958 | 5 | -6.09 | 585.2981 | 19.54 | 7437 | 3.9e2 | 1 | 1 | C4QYA1|C4QYA1\_KOMPG |  |  | DB Search |
| RPD | 19.79 | 386.1914 | 3 | -3.61 | 387.1963 | 2.95 | 1225 | 1.33e2 | 1 | 1 | C4R672|C4R672\_KOMPG:C4R3J0|C4R3J0\_KOMPG:C4R6R3|C4R6R3\_KOMPG:C4QX06|C4QX06\_KOMPG:C4R3N8|C4R3N8\_KOMPG:C4R214|C4R214\_KOMPG:C4R6E3|C4R6E3\_KOMPG:C4R6C2|PEX1\_KOMPG:C4R4A8|C4R4A8\_KOMPG:C4R3W9|C4R3W9\_KOMPG:C4R399|C4R399\_KOMPG:C4R510|C4R510\_KOMPG:C4QYK0|RSSA\_KOMPG:C4R3T4|C4R3T4\_KOMPG:C4R8B9|C4R8B9\_KOMPG:C4R4G9|OXDA\_KOMPG:C4R6B2|C4R6B2\_KOMPG:C4R0H4|C4R0H4\_KOMPG:C4QY88|C4QY88\_KOMPG:C4R1I0|C4R1I0\_KOMPG:C4R014|C4R014\_KOMPG:C4R0Y0|C4R0Y0\_KOMPG:C4R1Q7|C4R1Q7\_KOMPG:C4R2R9|C4R2R9\_KOMPG:C4R4H3|C4R4H3\_KOMPG:C4R1E5|C4R1E5\_KOMPG:C4QXR7|C4QXR7\_KOMPG:C4R7A4|C4R7A4\_KOMPG:C4QWT9|C4QWT9\_KOMPG:C4R3D9|C4R3D9\_KOMPG:C4R420|C4R420\_KOMPG:C4R4I8|C4R4I8\_KOMPG:C4R189|C4R189\_KOMPG:C4R4U7|C4R4U7\_KOMPG:C4R688|C4R688\_KOMPG:C4QW89|C4QW89\_KOMPG:C4R7N8|C4R7N8\_KOMPG:C4R792|C4R792\_KOMPG:C4R6B3|C4R6B3\_KOMPG:C4R794|C4R794\_KOMPG:C4QY53|C4QY53\_KOMPG:C4R517|C4R517\_KOMPG:C4QZ19|C4QZ19\_KOMPG:C4QYQ8|C4QYQ8\_KOMPG:C4R2C6|C4R2C6\_KOMPG:C4R821|C4R821\_KOMPG:C4QYW2|C4QYW2\_KOMPG:C4R2N3|C4R2N3\_KOMPG:C4R950|C4R950\_KOMPG:C4R7K4|C4R7K4\_KOMPG:C4QZB6|C4QZB6\_KOMPG:C4R7Q2|C4R7Q2\_KOMPG:C4R0D0|C4R0D0\_KOMPG:C4R7U3|C4R7U3\_KOMPG:C4R773|C4R773\_KOMPG:C4R941|GLG\_KOMPG:C4R5I8|C4R5I8\_KOMPG:C4QZB4|C4QZB4\_KOMPG:C4R1B6|C4R1B6\_KOMPG:C4R795|C4R795\_KOMPG:C4R3U7|C4R3U7\_KOMPG:C4R2X0|C4R2X0\_KOMPG:C4R6T1|C4R6T1\_KOMPG:C4R6C8|C4R6C8\_KOMPG:C4R7G1|C4R7G1\_KOMPG:C4QWF8|C4QWF8\_KOMPG:C4R581|C4R581\_KOMPG:C4QXA8|C4QXA8\_KOMPG:C4QV86|C4QV86\_KOMPG:C4R522|C4R522\_KOMPG:C4R2J5|C4R2J5\_KOMPG:C4R7D0|C4R7D0\_KOMPG:C4R0H0|C4R0H0\_KOMPG:C4R0S9|C4R0S9\_KOMPG:C4QVU5|C4QVU5\_KOMPG:C4R998|C4R998\_KOMPG:C4R1J4|C4R1J4\_KOMPG:C4R751|C4R751\_KOMPG:C4QVQ7|C4QVQ7\_KOMPG:C4R6L7|C4R6L7\_KOMPG:C4R534|C4R534\_KOMPG:C4R956|C4R956\_KOMPG:C4R259|C4R259\_KOMPG:C4QV21|C4QV21\_KOMPG:C4R4I4|C4R4I4\_KOMPG:C4R1X3|C4R1X3\_KOMPG:C4R6I1|C4R6I1\_KOMPG:C4QWF3|C4QWF3\_KOMPG:C4R1H5|C4R1H5\_KOMPG:C4QY42|C4QY42\_KOMPG:C4QYT3|C4QYT3\_KOMPG:C4QYT0|C4QYT0\_KOMPG:C4QYF5|C4QYF5\_KOMPG:C4R6U9|C4R6U9\_KOMPG:C4R0J8|C4R0J8\_KOMPG:C4R724|C4R724\_KOMPG:C4QUZ1|C4QUZ1\_KOMPG:C4R6M4|C4R6M4\_KOMPG:C4QUZ4|C4QUZ4\_KOMPG:C4R877|C4R877\_KOMPG:C4R6P4|C4R6P4\_KOMPG:C4QVL8|C4QVL8\_KOMPG:C4R2Q9|C4R2Q9\_KOMPG:C4QWB1|C4QWB1\_KOMPG:C4R591|STS1\_KOMPG:C4QY14|C4QY14\_KOMPG:C4QXI8|PEX6\_KOMPG:C4QZH7|C4QZH7\_KOMPG:C4R099|C4R099\_KOMPG:C4R4P0|C4R4P0\_KOMPG:C4QWH1|C4QWH1\_KOMPG:C4R1N6|C4R1N6\_KOMPG:C4QXM9|C4QXM9\_KOMPG:C4QXL7|C4QXL7\_KOMPG:C4R3B7|C4R3B7\_KOMPG:C4R068|C4R068\_KOMPG:C4R3C3|C4R3C3\_KOMPG:C4R836|C4R836\_KOMPG:C4R6W7|C4R6W7\_KOMPG:C4QV05|C4QV05\_KOMPG:C4R0V2|C4R0V2\_KOMPG:C4R126|C4R126\_KOMPG:C4QXJ7|C4QXJ7\_KOMPG:C4QYX7|C4QYX7\_KOMPG:C4R7B3|C4R7B3\_KOMPG:C4QXS9|C4QXS9\_KOMPG:C4R0W5|C4R0W5\_KOMPG:C4R1Z4|C4R1Z4\_KOMPG:C4R9A6|C4R9A6\_KOMPG |  |  | DB Search |
| TDVI | 19.75 | 446.2376 | 4 | -5.56 | 447.2413 | 13.64 | 5362 | 0 | 0 | 0 | C4R821|C4R821\_KOMPG:C4R1M6|C4R1M6\_KOMPG:C4R602|C4R602\_KOMPG:C4QVB3|C4QVB3\_KOMPG:C4R0A4|C4R0A4\_KOMPG:C4R7E6|C4R7E6\_KOMPG:C4R0M4|C4R0M4\_KOMPG:C4R1W2|C4R1W2\_KOMPG:C4R2L9|C4R2L9\_KOMPG:C4R7C8|C4R7C8\_KOMPG:C4R6F6|C4R6F6\_KOMPG:C4R1M4|C4R1M4\_KOMPG:C4R1D3|C4R1D3\_KOMPG:C4R8G1|C4R8G1\_KOMPG:C4R0Q9|C4R0Q9\_KOMPG:C4R8V8|C4R8V8\_KOMPG:C4R746|C4R746\_KOMPG:C4R8A8|C4R8A8\_KOMPG:C4QVM1|C4QVM1\_KOMPG:C4R2Y5|C4R2Y5\_KOMPG:C4QXM5|C4QXM5\_KOMPG:C4QZ31|C4QZ31\_KOMPG |  |  | DB Search |
| TDVL | 19.75 | 446.2376 | 4 | -5.56 | 447.2413 | 13.64 | 5362 | 0 | 0 | 0 | C4R3D7|C4R3D7\_KOMPG:C4R7K3|C4R7K3\_KOMPG:C4R6V9|C4R6V9\_KOMPG:C4R8D6|C4R8D6\_KOMPG:P52710|CBPY\_KOMPG:C4QZH2|C4QZH2\_KOMPG:C4QW64|C4QW64\_KOMPG:C4QXH4|C4QXH4\_KOMPG:C4R4B1|C4R4B1\_KOMPG:C4R1V9|C4R1V9\_KOMPG:C4R546|C4R546\_KOMPG:C4R6M3|C4R6M3\_KOMPG:C4QVU9|C4QVU9\_KOMPG:C4R7D2|C4R7D2\_KOMPG:C4R6Z9|C4R6Z9\_KOMPG:C4R4X7|C4R4X7\_KOMPG:C4R488|C4R488\_KOMPG:C4R843|C4R843\_KOMPG:C4R0G3|C4R0G3\_KOMPG:C4R7L0|C4R7L0\_KOMPG:C4QWD1|C4QWD1\_KOMPG:C4R7S8|C4R7S8\_KOMPG:C4R0F0|C4R0F0\_KOMPG:C4R7I8|C4R7I8\_KOMPG:C4R7M3|C4R7M3\_KOMPG:C4R6G7|C4R6G7\_KOMPG:C4R470|C4R470\_KOMPG:C4QZX4|C4QZX4\_KOMPG:C4R5Z8|C4R5Z8\_KOMPG:C4QVG6|C4QVG6\_KOMPG:C4R7G5|C4R7G5\_KOMPG |  |  | DB Search |
| FISH | 19.74 | 502.254 | 4 | 7.98 | 503.264 | 15.82 | 6278 | 1.63e2 | 1 | 1 | C4R1L5|C4R1L5\_KOMPG:C4QVD8|C4QVD8\_KOMPG:C4R6Z8|C4R6Z8\_KOMPG:C4QY42|C4QY42\_KOMPG:C4R6E1|C4R6E1\_KOMPG:C4R1R8|C4R1R8\_KOMPG:C4R669|C4R669\_KOMPG:C4R5R0|C4R5R0\_KOMPG:C4QYM4|C4QYM4\_KOMPG:C4R7L2|C4R7L2\_KOMPG:C4R517|C4R517\_KOMPG:C4QW53|C4QW53\_KOMPG:C4R5T6|C4R5T6\_KOMPG:C4R9A6|C4R9A6\_KOMPG |  |  | DB Search |
| FLSH | 19.74 | 502.254 | 4 | 7.98 | 503.264 | 15.82 | 6278 | 1.63e2 | 1 | 1 | C4QX92|C4QX92\_KOMPG:C4R799|C4R799\_KOMPG:C4R0L9|C4R0L9\_KOMPG:C4QX89|C4QX89\_KOMPG:C4R0Z1|C4R0Z1\_KOMPG:C4QVS8|C4QVS8\_KOMPG:C4R1U1|C4R1U1\_KOMPG:C4QY40|C4QY40\_KOMPG:C4R719|C4R719\_KOMPG:C4R1X9|C4R1X9\_KOMPG:C4R665|C4R665\_KOMPG:C4QYV9|C4QYV9\_KOMPG:C4R773|C4R773\_KOMPG:C4R780|C4R780\_KOMPG:C4R1U8|C4R1U8\_KOMPG:C4QZ55|C4QZ55\_KOMPG:C4R462|C4R462\_KOMPG:C4R7P1|C4R7P1\_KOMPG:C4QVK7|C4QVK7\_KOMPG:C4R151|C4R151\_KOMPG |  |  | DB Search |
| ISVP | 19.74 | 414.2478 | 4 | -0.09 | 415.254 | 17.14 | 6914 | 3.72e3 | 1 | 1 | C4R7U7|C4R7U7\_KOMPG:C4R0N4|C4R0N4\_KOMPG:C4QY30|C4QY30\_KOMPG:C4QVT8|C4QVT8\_KOMPG:C4QY85|C4QY85\_KOMPG:C4R402|C4R402\_KOMPG:C4R842|C4R842\_KOMPG:C4R5G8|C4R5G8\_KOMPG:C4QXB4|C4QXB4\_KOMPG:C4R6J4|C4R6J4\_KOMPG:C4R568|C4R568\_KOMPG:C4R569|C4R569\_KOMPG:C4R2N5|C4R2N5\_KOMPG:C4R3Y3|C4R3Y3\_KOMPG:C4R249|C4R249\_KOMPG:C4R2D2|C4R2D2\_KOMPG:C4R0H4|C4R0H4\_KOMPG:C4QZY2|C4QZY2\_KOMPG:C4R540|C4R540\_KOMPG:C4R5G3|C4R5G3\_KOMPG:C4QVK6|C4QVK6\_KOMPG:C4R446|C4R446\_KOMPG:C4R963|C4R963\_KOMPG:C4R028|C4R028\_KOMPG:C4R0F4|C4R0F4\_KOMPG:C4R8A6|C4R8A6\_KOMPG:C4QWM2|C4QWM2\_KOMPG:C4R717|C4R717\_KOMPG:C4R5K0|C4R5K0\_KOMPG:C4QXV5|C4QXV5\_KOMPG:C4R1R8|C4R1R8\_KOMPG:C4QYX5|C4QYX5\_KOMPG:C4R2E4|C4R2E4\_KOMPG:C4QV75|C4QV75\_KOMPG:C4R2R0|C4R2R0\_KOMPG:C4R1B6|C4R1B6\_KOMPG:C4R1Y8|C4R1Y8\_KOMPG:C4R5N3|C4R5N3\_KOMPG:C4QVB6|C4QVB6\_KOMPG:C4QXX6|C4QXX6\_KOMPG:C4QXS6|C4QXS6\_KOMPG:C4QZV7|KEX1\_KOMPG:C4R0W2|C4R0W2\_KOMPG:C4R0J3|C4R0J3\_KOMPG:C4R3M1|C4R3M1\_KOMPG:C4R556|C4R556\_KOMPG |  |  | DB Search |
| LSVP | 19.74 | 414.2478 | 4 | -0.09 | 415.254 | 17.14 | 6914 | 3.72e3 | 1 | 1 | C4R5U0|C4R5U0\_KOMPG:C4QWN8|C4QWN8\_KOMPG:C4R1G8|C4R1G8\_KOMPG:C4QYR2|C4QYR2\_KOMPG:C4R658|C4R658\_KOMPG:C4R5N2|C4R5N2\_KOMPG:C4R3D4|PEX2\_KOMPG:C4R6L7|C4R6L7\_KOMPG:C4R007|C4R007\_KOMPG:C4QVK8|C4QVK8\_KOMPG:C4R9E0|C4R9E0\_KOMPG:C4R6L8|C4R6L8\_KOMPG:C4R9B2|C4R9B2\_KOMPG:C4R4M6|C4R4M6\_KOMPG:C4R5J2|C4R5J2\_KOMPG:C4R548|C4R548\_KOMPG:C4R7J5|C4R7J5\_KOMPG:C4QXU5|C4QXU5\_KOMPG:C4R2L0|PEX5\_KOMPG:C4R724|C4R724\_KOMPG:C4R1A9|C4R1A9\_KOMPG:C4QVH4|C4QVH4\_KOMPG:C4R1Y7|C4R1Y7\_KOMPG:C4QXR9|C4QXR9\_KOMPG:C4QXF7|C4QXF7\_KOMPG:C4QZ59|C4QZ59\_KOMPG:C4QYU4|C4QYU4\_KOMPG:C4R070|C4R070\_KOMPG:C4R5U2|C4R5U2\_KOMPG:C4QW84|C4QW84\_KOMPG:C4QVB8|C4QVB8\_KOMPG:C4QV22|C4QV22\_KOMPG:C4QVX3|C4QVX3\_KOMPG:C4QVL5|C4QVL5\_KOMPG:C4QZ05|C4QZ05\_KOMPG:C4R803|C4R803\_KOMPG:C4R1N6|C4R1N6\_KOMPG:C4R5J9|C4R5J9\_KOMPG:C4R7M8|C4R7M8\_KOMPG:C4QWC3|C4QWC3\_KOMPG:C4R2S6|C4R2S6\_KOMPG:C4QYF4|C4QYF4\_KOMPG:C4R3K9|C4R3K9\_KOMPG:C4R875|C4R875\_KOMPG:C4QYX7|C4QYX7\_KOMPG:C4QY39|C4QY39\_KOMPG:C4R7S4|C4R7S4\_KOMPG:C4QYA2|C4QYA2\_KOMPG:C4R493|C4R493\_KOMPG:C4R063|C4R063\_KOMPG:C4QYA3|C4QYA3\_KOMPG:C4QV00|C4QV00\_KOMPG:C4R5V0|C4R5V0\_KOMPG |  |  | DB Search |
| KCA | 19.71 | 320.1518 | 3 | 2.66 | 321.1591 | 15.28 | 6053 | 3.59e2 | 1 | 1 | C4R2H1|C4R2H1\_KOMPG:C4QVL1|C4QVL1\_KOMPG:C4R1D2|C4R1D2\_KOMPG:C4R142|C4R142\_KOMPG:C4R6A4|C4R6A4\_KOMPG:C4QWE6|C4QWE6\_KOMPG:C4QWB0|C4QWB0\_KOMPG:C4QVX8|C4QVX8\_KOMPG:C4QV43|C4QV43\_KOMPG:C4R5Q9|C4R5Q9\_KOMPG:C4QXQ2|C4QXQ2\_KOMPG:C4R234|C4R234\_KOMPG:C4QVK1|C4QVK1\_KOMPG:C4QY56|C4QY56\_KOMPG:Q9P4D0|SEC17\_KOMPG:C4R989|C4R989\_KOMPG:C4R286|C4R286\_KOMPG:C4QYP6|C4QYP6\_KOMPG:C4QZK4|C4QZK4\_KOMPG:C4R961|C4R961\_KOMPG:C4R198|C4R198\_KOMPG:C4R2I0|C4R2I0\_KOMPG:C4R007|C4R007\_KOMPG:C4R124|C4R124\_KOMPG:C4R2B5|C4R2B5\_KOMPG:C4R6V5|C4R6V5\_KOMPG:C4R725|C4R725\_KOMPG:C4QVM0|C4QVM0\_KOMPG:C4R8B2|C4R8B2\_KOMPG:C4R2G7|C4R2G7\_KOMPG:C4QZU9|C4QZU9\_KOMPG:C4R2F8|C4R2F8\_KOMPG:C4QVF9|C4QVF9\_KOMPG:C4QZH9|C4QZH9\_KOMPG:C4QXP4|C4QXP4\_KOMPG:C4QWH9|C4QWH9\_KOMPG:C4R3P0|C4R3P0\_KOMPG:C4QV04|C4QV04\_KOMPG:C4R1Y5|C4R1Y5\_KOMPG:C4QY17|DRE2\_KOMPG:C4R2Q9|C4R2Q9\_KOMPG:C4QZ48|C4QZ48\_KOMPG:C4QV87|C4QV87\_KOMPG:C4QYP4|C4QYP4\_KOMPG:C4R7I2|C4R7I2\_KOMPG:C4R8Z7|C4R8Z7\_KOMPG:C4R114|C4R114\_KOMPG:C4QXN3|C4QXN3\_KOMPG:C4QY22|C4QY22\_KOMPG:C4R4I6|C4R4I6\_KOMPG:C4R4F4|C4R4F4\_KOMPG:C4R1U8|C4R1U8\_KOMPG:C4R899|C4R899\_KOMPG:C4QV45|C4QV45\_KOMPG:C4R723|C4R723\_KOMPG:C4R3X6|C4R3X6\_KOMPG:C4QZC5|C4QZC5\_KOMPG:C4R4E1|C4R4E1\_KOMPG:C4R8G3|C4R8G3\_KOMPG:C4R7R8|C4R7R8\_KOMPG:C4R8Z2|C4R8Z2\_KOMPG:C4R735|C4R735\_KOMPG:C4R055|C4R055\_KOMPG:C4R1B6|C4R1B6\_KOMPG:C4R3K8|C4R3K8\_KOMPG:C4R077|C4R077\_KOMPG:C4R567|C4R567\_KOMPG:C4R2W8|C4R2W8\_KOMPG:C4R7Z4|C4R7Z4\_KOMPG:C4QVH1|C4QVH1\_KOMPG:C4R264|C4R264\_KOMPG:C4R2N9|C4R2N9\_KOMPG:C4R405|C4R405\_KOMPG |  |  | DB Search |
| IQEQP | 19.7 | 613.3071 | 5 | -8.19 | 614.3079 | 8.07 | 3008 | 2.59e3 | 1 | 1 | C4QY97|C4QY97\_KOMPG |  |  | DB Search |
| LQEQP | 19.7 | 613.3071 | 5 | -8.19 | 614.3079 | 8.07 | 3008 | 2.59e3 | 1 | 1 | C4QXB2|C4QXB2\_KOMPG |  |  | DB Search |
| QIEQP | 19.65 | 613.3071 | 5 | -8.19 | 614.3079 | 8.07 | 2985 | 2.59e3 | 1 | 1 | C4R446|C4R446\_KOMPG:C4QX04|C4QX04\_KOMPG |  |  | DB Search |
| QLEQP | 19.65 | 613.3071 | 5 | -8.19 | 614.3079 | 8.07 | 2985 | 2.59e3 | 1 | 1 | C4R669|C4R669\_KOMPG |  |  | DB Search |
| NDT | 19.64 | 348.1281 | 3 | 7.11 | 349.137 | 7.31 | 2704 | 6.3e4 | 1 | 1 | C4QVY1|C4QVY1\_KOMPG:C4QV81|C4QV81\_KOMPG:C4R7J1|C4R7J1\_KOMPG:C4R4N1|C4R4N1\_KOMPG:C4R1D2|C4R1D2\_KOMPG:C4R894|C4R894\_KOMPG:C4R1A5|C4R1A5\_KOMPG:C4R3U6|C4R3U6\_KOMPG:C4QYR2|C4QYR2\_KOMPG:C4R6C2|PEX1\_KOMPG:C4QWI1|C4QWI1\_KOMPG:C4R1G7|C4R1G7\_KOMPG:C4QV06|C4QV06\_KOMPG:C4R036|C4R036\_KOMPG:C4R003|C4R003\_KOMPG:C4QWD7|C4QWD7\_KOMPG:C4R1P8|C4R1P8\_KOMPG:C4R2A4|C4R2A4\_KOMPG:C4QVX4|C4QVX4\_KOMPG:C4QVT4|C4QVT4\_KOMPG:C4R3U2|C4R3U2\_KOMPG:C4QV46|C4QV46\_KOMPG:C4R019|C4R019\_KOMPG:C4R6E1|C4R6E1\_KOMPG:C4R4R8|ARO1\_KOMPG:C4R8D3|C4R8D3\_KOMPG:C4R990|C4R990\_KOMPG:C4R052|C4R052\_KOMPG:C4R341|C4R341\_KOMPG:C4R8Y4|C4R8Y4\_KOMPG:C4QVV6|C4QVV6\_KOMPG:C4QZW5|C4QZW5\_KOMPG:C4QWQ0|C4QWQ0\_KOMPG:C4QY85|C4QY85\_KOMPG:C4QZ73|C4QZ73\_KOMPG:C4QVL3|C4QVL3\_KOMPG:C4R9E6|C4R9E6\_KOMPG:C4QYH5|C4QYH5\_KOMPG:C4QYE2|C4QYE2\_KOMPG:C4R1S7|C4R1S7\_KOMPG:C4QYH6|C4QYH6\_KOMPG:C4QV66|C4QV66\_KOMPG:C4QYP7|C4QYP7\_KOMPG:C4R6D1|C4R6D1\_KOMPG:C4R6N1|C4R6N1\_KOMPG:C4R5H4|C4R5H4\_KOMPG:C4R8D5|C4R8D5\_KOMPG:C4R6V0|C4R6V0\_KOMPG:C4QVI7|C4QVI7\_KOMPG:C4R393|C4R393\_KOMPG:C4R275|C4R275\_KOMPG:C4R165|C4R165\_KOMPG:C4R1F0|C4R1F0\_KOMPG:C4QWD1|C4QWD1\_KOMPG:C4R3L2|C4R3L2\_KOMPG:C4R5H3|C4R5H3\_KOMPG:C4R773|C4R773\_KOMPG:C4R120|C4R120\_KOMPG:C4R2G2|C4R2G2\_KOMPG:C4R332|C4R332\_KOMPG:C4QZW1|C4QZW1\_KOMPG:C4R0K2|C4R0K2\_KOMPG:C4R3T3|C4R3T3\_KOMPG:C4QVK4|C4QVK4\_KOMPG:C4QV23|C4QV23\_KOMPG:C4R3A1|C4R3A1\_KOMPG:C4R0N9|C4R0N9\_KOMPG:C4QYH7|C4QYH7\_KOMPG:C4R7D0|C4R7D0\_KOMPG:C4QXX5|C4QXX5\_KOMPG:C4R8N7|C4R8N7\_KOMPG:C4R4M1|C4R4M1\_KOMPG:C4QVW5|C4QVW5\_KOMPG:C4QWA3|C4QWA3\_KOMPG:C4R454|C4R454\_KOMPG:C4QVA7|C4QVA7\_KOMPG:C4QYC6|C4QYC6\_KOMPG:C4R912|C4R912\_KOMPG:C4QV21|C4QV21\_KOMPG:C4R4I4|C4R4I4\_KOMPG:C4R7J8|C4R7J8\_KOMPG:C4QXU3|C4QXU3\_KOMPG:C4QXT9|C4QXT9\_KOMPG:C4QVJ6|C4QVJ6\_KOMPG:C4R1E3|C4R1E3\_KOMPG:C4R153|C4R153\_KOMPG:C4R0T7|C4R0T7\_KOMPG:C4R3Y2|C4R3Y2\_KOMPG:C4QX72|C4QX72\_KOMPG:C4R349|C4R349\_KOMPG:C4R0P1|C4R0P1\_KOMPG:C4QYZ4|C4QYZ4\_KOMPG:C4R724|C4R724\_KOMPG:C4R0H5|C4R0H5\_KOMPG:C4R5I2|C4R5I2\_KOMPG:C4R1N3|C4R1N3\_KOMPG:C4R6S3|C4R6S3\_KOMPG:C4R450|C4R450\_KOMPG:C4R1G6|C4R1G6\_KOMPG:C4R6Q5|C4R6Q5\_KOMPG:C4QV73|C4QV73\_KOMPG:C4R803|C4R803\_KOMPG:C4R6Q6|C4R6Q6\_KOMPG:C4QYA1|C4QYA1\_KOMPG:C4R1F2|C4R1F2\_KOMPG:C4QX36|C4QX36\_KOMPG:C4QW27|C4QW27\_KOMPG:C4QY04|C4QY04\_KOMPG:C4R743|C4R743\_KOMPG:C4QX81|C4QX81\_KOMPG:C4R0J5|C4R0J5\_KOMPG:C4QVA1|C4QVA1\_KOMPG:C4R5V4|C4R5V4\_KOMPG:C4R0W5|C4R0W5\_KOMPG:C4QY10|C4QY10\_KOMPG:C4QXZ9|C4QXZ9\_KOMPG:C4R855|C4R855\_KOMPG:C4QYQ1|C4QYQ1\_KOMPG:C4R697|C4R697\_KOMPG:C4R713|C4R713\_KOMPG |  |  | DB Search |
| VTAPLSL | 19.64 | 699.4167 | 7 | -9.2 | 700.4158 | 26.75 | 9472 | 8.73e2 | 1 | 1 | C4QYT3|C4QYT3\_KOMPG |  |  | DB Search |
| WAHI | 19.63 | 525.27 | 4 | 8.75 | 526.2805 | 15.65 | 6227 | 1.52e2 | 1 | 1 | C4QVK8|C4QVK8\_KOMPG |  |  | DB Search |
| WAHL | 19.63 | 525.27 | 4 | 8.75 | 526.2805 | 15.65 | 6227 | 1.52e2 | 1 | 1 | C4R325|C4R325\_KOMPG:C4R1I3|C4R1I3\_KOMPG:C4QZJ5|C4QZJ5\_KOMPG:C4R4Q5|C4R4Q5\_KOMPG |  |  | DB Search |
| KGHNTFGPAE | 19.63 | 1056.4988 | 10 | 5.88 | 529.2585 | 13.90 | 5521 | 0 | 0 | 0 | C4R2V7|C4R2V7\_KOMPG |  |  | DB Search |
| ITVA | 19.62 | 402.2478 | 4 | -4.79 | 403.2522 | 10.65 | 4156 | 4.9e3 | 1 | 1 | C4QY47|C4QY47\_KOMPG:C4R4C1|C4R4C1\_KOMPG:C4R5F5|C4R5F5\_KOMPG:C4QYM7|C4QYM7\_KOMPG:C4R4C3|C4R4C3\_KOMPG:C4R5S1|C4R5S1\_KOMPG:C4R7V7|C4R7V7\_KOMPG:C4R8K7|C4R8K7\_KOMPG:C4QXP8|C4QXP8\_KOMPG:C4R248|C4R248\_KOMPG:C4R234|C4R234\_KOMPG:C4R0H8|C4R0H8\_KOMPG:C4R8Y7|C4R8Y7\_KOMPG:C4R510|C4R510\_KOMPG:C4QZ19|C4QZ19\_KOMPG:C4R520|C4R520\_KOMPG:C4R1L3|C4R1L3\_KOMPG:C4R4Y2|C4R4Y2\_KOMPG:C4QWY4|C4QWY4\_KOMPG:C4R4A6|C4R4A6\_KOMPG:C4R6V3|C4R6V3\_KOMPG:C4R2C6|C4R2C6\_KOMPG:C4R1S1|C4R1S1\_KOMPG:C4QXU8|C4QXU8\_KOMPG:C4R7Y0|C4R7Y0\_KOMPG:C4R3H5|C4R3H5\_KOMPG:C4QZK0|C4QZK0\_KOMPG:C4QVR8|C4QVR8\_KOMPG:C4R6D7|C4R6D7\_KOMPG:C4R8P5|C4R8P5\_KOMPG:C4QWR3|C4QWR3\_KOMPG |  |  | DB Search |
| LTVA | 19.62 | 402.2478 | 4 | -4.79 | 403.2522 | 10.65 | 4156 | 4.9e3 | 1 | 1 | C4QYG9|C4QYG9\_KOMPG:C4R9D9|C4R9D9\_KOMPG:C4QYU1|C4QYU1\_KOMPG:C4R2G0|C4R2G0\_KOMPG:C4R8G4|C4R8G4\_KOMPG:C4QY85|C4QY85\_KOMPG:C4R7K3|C4R7K3\_KOMPG:C4R0F3|C4R0F3\_KOMPG:C4R8J1|C4R8J1\_KOMPG:C4QX99|C4QX99\_KOMPG:C4QZJ3|C4QZJ3\_KOMPG:C4R648|C4R648\_KOMPG:C4R2A1|C4R2A1\_KOMPG:C4R232|C4R232\_KOMPG:C4QYA8|C4QYA8\_KOMPG:C4QYV4|C4QYV4\_KOMPG:C4QXN6|C4QXN6\_KOMPG:C4R5P1|C4R5P1\_KOMPG:C4R1D1|C4R1D1\_KOMPG:C4R162|C4R162\_KOMPG:C4R6M3|C4R6M3\_KOMPG:C4R5Q8|C4R5Q8\_KOMPG:C4QY13|C4QY13\_KOMPG:C4QW42|C4QW42\_KOMPG:C4R738|C4R738\_KOMPG:C4QYQ2|C4QYQ2\_KOMPG:C4R611|C4R611\_KOMPG:C4R6G5|C4R6G5\_KOMPG:C4R1T4|C4R1T4\_KOMPG:C4QV48|C4QV48\_KOMPG:C4QXD1|C4QXD1\_KOMPG:C4R7H0|C4R7H0\_KOMPG:C4QX81|C4QX81\_KOMPG:C4R8Z6|C4R8Z6\_KOMPG:C4QVT5|C4QVT5\_KOMPG:C4R889|C4R889\_KOMPG:C4R1G4|C4R1G4\_KOMPG:C4QYF8|C4QYF8\_KOMPG:C4QZP1|C4QZP1\_KOMPG:C4R5G5|C4R5G5\_KOMPG:C4R7S4|C4R7S4\_KOMPG:C4R6S4|C4R6S4\_KOMPG:C4R6D4|C4R6D4\_KOMPG:C4QX58|C4QX58\_KOMPG:C4QXV8|C4QXV8\_KOMPG:C4R5W9|C4R5W9\_KOMPG:C4QYT6|C4QYT6\_KOMPG:C4R868|C4R868\_KOMPG |  |  | DB Search |
| VFST | 19.6 | 452.2271 | 4 | -3.7 | 453.2316 | 22.49 | 8270 | 1.33e3 | 1 | 1 | C4QZP8|C4QZP8\_KOMPG:C4R0Z3|C4R0Z3\_KOMPG:C4R6X0|C4R6X0\_KOMPG:C4R842|C4R842\_KOMPG:C4R070|C4R070\_KOMPG:C4R4H2|C4R4H2\_KOMPG:C4R6H5|C4R6H5\_KOMPG:C4R7U5|C4R7U5\_KOMPG:C4QVU3|SLD1\_KOMPG:C4R4C3|C4R4C3\_KOMPG:C4QYJ4|C4QYJ4\_KOMPG:C4R8I2|C4R8I2\_KOMPG:C4QWD3|C4QWD3\_KOMPG:C4R0W9|C4R0W9\_KOMPG:C4R1H3|C4R1H3\_KOMPG:C4R7K0|C4R7K0\_KOMPG:C4R4E0|C4R4E0\_KOMPG:C4QZ21|C4QZ21\_KOMPG:C4R869|C4R869\_KOMPG:C4R7H1|C4R7H1\_KOMPG:C4QYY8|C4QYY8\_KOMPG:C4R691|C4R691\_KOMPG:C4R1C3|C4R1C3\_KOMPG:C4QY09|C4QY09\_KOMPG:C4R4Q2|C4R4Q2\_KOMPG:C4QZI2|C4QZI2\_KOMPG:C4R254|C4R254\_KOMPG:C4QV15|C4QV15\_KOMPG:C4R5H0|C4R5H0\_KOMPG:C4QVV9|C4QVV9\_KOMPG:C4R2G5|C4R2G5\_KOMPG:C4QVW7|C4QVW7\_KOMPG:C4R3C4|C4R3C4\_KOMPG:C4R8E9|C4R8E9\_KOMPG:C4R7U0|C4R7U0\_KOMPG |  |  | DB Search |
| WNVPL | 19.6 | 627.338 | 5 | 2.34 | 628.3452 | 26.28 | 9350 | 2.15e2 | 1 | 1 | C4QWS1|C4QWS1\_KOMPG |  |  | DB Search |
| ISPM | 19.59 | 446.2199 | 4 | -1.9 | 447.2252 | 15.64 | 6201 | 6.79e2 | 1 | 1 | C4QW82|C4QW82\_KOMPG:C4R1B8|C4R1B8\_KOMPG:C4QWE8|C4QWE8\_KOMPG:C4QY26|C4QY26\_KOMPG:C4R0X4|C4R0X4\_KOMPG:C4R5J2|C4R5J2\_KOMPG:C4R543|C4R543\_KOMPG:C4R3E0|C4R3E0\_KOMPG:C4QZ96|C4QZ96\_KOMPG |  |  | DB Search |
| LSPM | 19.59 | 446.2199 | 4 | -1.9 | 447.2252 | 15.64 | 6201 | 6.79e2 | 1 | 1 | C4R0K4|C4R0K4\_KOMPG:C4QWK2|C4QWK2\_KOMPG:C4R166|C4R166\_KOMPG:C4QZB6|C4QZB6\_KOMPG:C4QV93|C4QV93\_KOMPG:C4R1D2|C4R1D2\_KOMPG:C4R2Z8|C4R2Z8\_KOMPG:C4R5Q1|C4R5Q1\_KOMPG:C4QWR1|C4QWR1\_KOMPG:C4R4R1|C4R4R1\_KOMPG:C4QWL3|C4QWL3\_KOMPG:C4R1W4|C4R1W4\_KOMPG:C4R6T6|C4R6T6\_KOMPG:C4R2K5|C4R2K5\_KOMPG:C4QYV2|C4QYV2\_KOMPG:C4R7G1|C4R7G1\_KOMPG |  |  | DB Search |
| QLPQQP | 19.58 | 709.3759 | 6 | -7.55 | 710.376 | 8.73 | 3397 | 1.32e3 | 1 | 1 | C4QWG1|C4QWG1\_KOMPG |  |  | DB Search |
| SISGI | 19.58 | 475.2642 | 5 | -6.87 | 476.267 | 11.14 | 4400 | 2.28e3 | 1 | 1 | C4R357|C4R357\_KOMPG:C4R5Y6|C4R5Y6\_KOMPG |  |  | DB Search |
| SISGL | 19.58 | 475.2642 | 5 | -6.87 | 476.267 | 11.14 | 4400 | 2.28e3 | 1 | 1 | C4QWU0|C4QWU0\_KOMPG:C4QYK8|C4QYK8\_KOMPG:C4QVK5|C4QVK5\_KOMPG:C4R6Q0|C4R6Q0\_KOMPG:C4QZR7|C4QZR7\_KOMPG:C4R2D0|C4R2D0\_KOMPG:C4R4Z9|C4R4Z9\_KOMPG |  |  | DB Search |
| SLSGI | 19.58 | 475.2642 | 5 | -6.87 | 476.267 | 11.14 | 4400 | 2.28e3 | 1 | 1 | C4R3D3|C4R3D3\_KOMPG:C4R7J4|C4R7J4\_KOMPG:C4R2M3|C4R2M3\_KOMPG |  |  | DB Search |
| SLSGL | 19.58 | 475.2642 | 5 | -6.87 | 476.267 | 11.14 | 4400 | 2.28e3 | 1 | 1 | C4QWK2|C4QWK2\_KOMPG:C4R2V8|C4R2V8\_KOMPG:C4R7K1|C4R7K1\_KOMPG:C4R466|C4R466\_KOMPG:C4R4C7|C4R4C7\_KOMPG:C4QYX5|C4QYX5\_KOMPG:C4R3T1|C4R3T1\_KOMPG:C4R8S6|C4R8S6\_KOMPG:C4QXN8|C4QXN8\_KOMPG:C4R4M2|C4R4M2\_KOMPG:C4QVY8|C4QVY8\_KOMPG:C4R580|C4R580\_KOMPG:C4QWY1|C4QWY1\_KOMPG:C4R1A8|C4R1A8\_KOMPG:C4R0L5|C4R0L5\_KOMPG |  |  | DB Search |
| IYSG | 19.57 | 438.2114 | 4 | -3.08 | 439.2163 | 7.95 | 2941 | 3.53e4 | 1 | 1 | C4R5K3|C4R5K3\_KOMPG:C4QZ47|C4QZ47\_KOMPG:C4R570|C4R570\_KOMPG:C4QV12|C4QV12\_KOMPG:C4R4A1|C4R4A1\_KOMPG:C4QV92|C4QV92\_KOMPG:C4QXJ4|C4QXJ4\_KOMPG:C4R0N8|C4R0N8\_KOMPG:C4R4T1|C4R4T1\_KOMPG:C4QWL2|C4QWL2\_KOMPG:C4R3C0|C4R3C0\_KOMPG:C4R1S5|C4R1S5\_KOMPG:C4QXY8|C4QXY8\_KOMPG:C4QWD1|C4QWD1\_KOMPG:C4R834|C4R834\_KOMPG:C4R6X3|C4R6X3\_KOMPG:C4R4V4|C4R4V4\_KOMPG:C4QVP1|C4QVP1\_KOMPG:C4R454|C4R454\_KOMPG:C4QX17|C4QX17\_KOMPG:C4R4T6|C4R4T6\_KOMPG |  |  | DB Search |
| LYSG | 19.57 | 438.2114 | 4 | -3.08 | 439.2163 | 7.95 | 2941 | 3.53e4 | 1 | 1 | C4QX82|C4QX82\_KOMPG:C4R2Y2|C4R2Y2\_KOMPG:C4R793|C4R793\_KOMPG:C4R5V9|C4R5V9\_KOMPG:C4R0Y9|C4R0Y9\_KOMPG:C4QZ79|C4QZ79\_KOMPG:C4QVL3|C4QVL3\_KOMPG:C4QW17|C4QW17\_KOMPG:C4R1Z3|C4R1Z3\_KOMPG:C4R5F6|C4R5F6\_KOMPG:C4R1P4|C4R1P4\_KOMPG:C4R4S0|C4R4S0\_KOMPG:C4R438|C4R438\_KOMPG:C4R546|C4R546\_KOMPG:C4R3Q6|C4R3Q6\_KOMPG:C4QXB5|C4QXB5\_KOMPG:C4QYB6|C4QYB6\_KOMPG:C4R8E5|C4R8E5\_KOMPG:C4QYS5|C4QYS5\_KOMPG:C4R0F1|C4R0F1\_KOMPG:C4QW86|C4QW86\_KOMPG:C4R4K5|C4R4K5\_KOMPG:C4QWC3|C4QWC3\_KOMPG:C4QW10|C4QW10\_KOMPG:C4R8L3|C4R8L3\_KOMPG:C4R778|C4R778\_KOMPG:C4R364|C4R364\_KOMPG:C4R1F1|C4R1F1\_KOMPG:C4QYK1|C4QYK1\_KOMPG:C4R6A2|C4R6A2\_KOMPG:C4R3U4|C4R3U4\_KOMPG:C4R8P1|C4R8P1\_KOMPG:C4R322|C4R322\_KOMPG |  |  | DB Search |
| QFF | 19.56 | 440.206 | 3 | 3.92 | 441.2139 | 19.15 | 7313 | 0 | 0 | 0 | C4R7X6|C4R7X6\_KOMPG:C4R3J0|C4R3J0\_KOMPG:C4R6K9|C4R6K9\_KOMPG:C4R0Z1|C4R0Z1\_KOMPG:C4QXK0|C4QXK0\_KOMPG:C4QWB0|C4QWB0\_KOMPG:C4QWN8|C4QWN8\_KOMPG:C4R669|C4R669\_KOMPG:C4R1X4|C4R1X4\_KOMPG:C4R0H4|C4R0H4\_KOMPG:C4QW51|C4QW51\_KOMPG:C4QWB2|C4QWB2\_KOMPG:C4QYY2|C4QYY2\_KOMPG:C4R555|C4R555\_KOMPG:C4R1T4|C4R1T4\_KOMPG:C4QWH7|C4QWH7\_KOMPG:C4R768|C4R768\_KOMPG:C4QYY0|C4QYY0\_KOMPG:C4R499|C4R499\_KOMPG:C4R5J2|C4R5J2\_KOMPG:C4QZI2|C4QZI2\_KOMPG:C4R2D5|C4R2D5\_KOMPG:C4R404|C4R404\_KOMPG:C4R0Y7|C4R0Y7\_KOMPG:C4R121|C4R121\_KOMPG:C4QYN9|C4QYN9\_KOMPG:C4QY37|C4QY37\_KOMPG:C4R161|C4R161\_KOMPG:C4QVC9|C4QVC9\_KOMPG:C4R8Y4|C4R8Y4\_KOMPG:C4QW13|C4QW13\_KOMPG:C4QXZ2|C4QXZ2\_KOMPG:C4QY69|C4QY69\_KOMPG:C4QW36|C4QW36\_KOMPG:C4QYN3|C4QYN3\_KOMPG:C4QYU4|C4QYU4\_KOMPG:C4QWF7|C4QWF7\_KOMPG:C4R6U4|C4R6U4\_KOMPG:C4R232|C4R232\_KOMPG:C4R3G4|C4R3G4\_KOMPG:C4R7V7|C4R7V7\_KOMPG:C4R1R0|C4R1R0\_KOMPG:C4R7J3|C4R7J3\_KOMPG:C4R3Q6|C4R3Q6\_KOMPG:C4R6D1|C4R6D1\_KOMPG:C4R4F7|C4R4F7\_KOMPG:C4R8V0|C4R8V0\_KOMPG:C4QY65|C4QY65\_KOMPG:C4R7Y7|C4R7Y7\_KOMPG:C4QWQ8|C4QWQ8\_KOMPG:C4R4P1|C4R4P1\_KOMPG:C4R252|C4R252\_KOMPG:C4R393|C4R393\_KOMPG:C4R900|C4R900\_KOMPG:C4QYF4|C4QYF4\_KOMPG:C4QXQ0|C4QXQ0\_KOMPG:C4QYL9|C4QYL9\_KOMPG:C4QZM2|C4QZM2\_KOMPG:C4QWD1|C4QWD1\_KOMPG:C4QWL5|C4QWL5\_KOMPG:C4R8E9|C4R8E9\_KOMPG:C4QVK4|C4QVK4\_KOMPG:C4QZR2|C4QZR2\_KOMPG:C4R5V1|C4R5V1\_KOMPG:C4QV86|C4QV86\_KOMPG:C4R2R1|C4R2R1\_KOMPG:Q9Y751|ATG26\_KOMPG:C4QVN4|C4QVN4\_KOMPG:C4R1U2|C4R1U2\_KOMPG:C4R1U1|C4R1U1\_KOMPG:C4R5P0|C4R5P0\_KOMPG:C4R5T5|C4R5T5\_KOMPG:C4R5C6|C4R5C6\_KOMPG:C4R880|C4R880\_KOMPG:C4R454|C4R454\_KOMPG:C4R4I9|C4R4I9\_KOMPG:C4R540|C4R540\_KOMPG:C4QZ31|C4QZ31\_KOMPG:C4R5A3|C4R5A3\_KOMPG:C4R5B6|C4R5B6\_KOMPG:C4QXF6|C4QXF6\_KOMPG:C4R810|C4R810\_KOMPG:C4R3L1|C4R3L1\_KOMPG:C4R5I2|C4R5I2\_KOMPG:C4R7T5|C4R7T5\_KOMPG:C4QZN8|C4QZN8\_KOMPG:C4R2G0|C4R2G0\_KOMPG:C4QY75|C4QY75\_KOMPG:C4R8I7|C4R8I7\_KOMPG:C4R3N5|C4R3N5\_KOMPG:C4R2P5|LCL2\_KOMPG:C4R441|C4R441\_KOMPG:C4R148|C4R148\_KOMPG:C4R7X2|C4R7X2\_KOMPG:C4R1N6|C4R1N6\_KOMPG:C4QWL7|C4QWL7\_KOMPG:C4R612|C4R612\_KOMPG:C4QZV8|C4QZV8\_KOMPG:C4QZL1|C4QZL1\_KOMPG:C4QZ40|C4QZ40\_KOMPG:C4QYQ9|C4QYQ9\_KOMPG:C4R5K1|C4R5K1\_KOMPG:C4R6M8|C4R6M8\_KOMPG:C4QWV2|C4QWV2\_KOMPG:C4R0B3|C4R0B3\_KOMPG:C4R1T6|C4R1T6\_KOMPG:C4QYK1|C4QYK1\_KOMPG:C4R7M0|C4R7M0\_KOMPG:C4QXZ9|C4QXZ9\_KOMPG:C4R4H7|C4R4H7\_KOMPG:C4R2J9|C4R2J9\_KOMPG:C4R1Z6|C4R1Z6\_KOMPG:C4R2Z2|C4R2Z2\_KOMPG:C4QVL1|C4QVL1\_KOMPG:C4QVT8|C4QVT8\_KOMPG:C4R4X9|C4R4X9\_KOMPG:C4R006|C4R006\_KOMPG:C4R214|C4R214\_KOMPG:C4R4G6|C4R4G6\_KOMPG:C4R3W7|C4R3W7\_KOMPG:C4R5K5|C4R5K5\_KOMPG:C4R558|C4R558\_KOMPG:C4R7L5|C4R7L5\_KOMPG:C4QZJ5|C4QZJ5\_KOMPG:C4R753|C4R753\_KOMPG:C4R1C6|C4R1C6\_KOMPG:C4QX73|C4QX73\_KOMPG:C4R5S9|C4R5S9\_KOMPG:C4R0Y4|C4R0Y4\_KOMPG:C4R0G3|C4R0G3\_KOMPG:C4R5S2|C4R5S2\_KOMPG:C4QZF1|C4QZF1\_KOMPG:C4R7X1|C4R7X1\_KOMPG:C4QZH9|C4QZH9\_KOMPG:C4R1Z0|C4R1Z0\_KOMPG:C4R6I6|C4R6I6\_KOMPG:C4R6L4|C4R6L4\_KOMPG:C4R925|C4R925\_KOMPG:C4QXQ8|C4QXQ8\_KOMPG:C4QVL3|C4QVL3\_KOMPG:C4R6Q1|C4R6Q1\_KOMPG:C4QVX3|C4QVX3\_KOMPG:C4QV66|C4QV66\_KOMPG:C4QW55|C4QW55\_KOMPG:C4QVG4|C4QVG4\_KOMPG:C4R680|C4R680\_KOMPG:C4R7K8|C4R7K8\_KOMPG:C4R7B8|C4R7B8\_KOMPG:C4QZT7|C4QZT7\_KOMPG:C4R6M9|C4R6M9\_KOMPG:C4QZ08|C4QZ08\_KOMPG:C4R8C5|C4R8C5\_KOMPG:C4QVQ4|C4QVQ4\_KOMPG:C4R1R6|C4R1R6\_KOMPG:C4R378|C4R378\_KOMPG:C4R334|C4R334\_KOMPG:C4R3G2|C4R3G2\_KOMPG:C4QZP5|C4QZP5\_KOMPG:C4R270|C4R270\_KOMPG:C4QV37|C4QV37\_KOMPG:C4R3V3|C4R3V3\_KOMPG:C4R782|C4R782\_KOMPG:C4R4B2|C4R4B2\_KOMPG:C4QX33|C4QX33\_KOMPG:C4R295|C4R295\_KOMPG:C4R292|C4R292\_KOMPG:C4QWA9|C4QWA9\_KOMPG:C4R9E5|C4R9E5\_KOMPG:C4R581|C4R581\_KOMPG:C4QWG8|C4QWG8\_KOMPG:C4R4V3|C4R4V3\_KOMPG:C4R1G8|C4R1G8\_KOMPG:C4QVE2|C4QVE2\_KOMPG:C4QYV4|C4QYV4\_KOMPG:C4R6K6|C4R6K6\_KOMPG:C4QWD3|C4QWD3\_KOMPG:C4R6Y0|C4R6Y0\_KOMPG:C4R6X4|C4R6X4\_KOMPG:C4QWH3|C4QWH3\_KOMPG:C4R347|C4R347\_KOMPG:C4R0T2|C4R0T2\_KOMPG:C4R7J8|C4R7J8\_KOMPG:C4R986|C4R986\_KOMPG:C4R750|C4R750\_KOMPG:C4QWQ4|C4QWQ4\_KOMPG:C4R5M2|C4R5M2\_KOMPG:C4QY28|C4QY28\_KOMPG:C4R1E3|C4R1E3\_KOMPG:C4QX47|C4QX47\_KOMPG:C4QXU5|C4QXU5\_KOMPG:C4QXD4|C4QXD4\_KOMPG:C4R301|C4R301\_KOMPG:C4QXQ5|C4QXQ5\_KOMPG:C4R7W5|C4R7W5\_KOMPG:C4R0P5|C4R0P5\_KOMPG:C4R4Z4|C4R4Z4\_KOMPG:C4R915|C4R915\_KOMPG:C4R610|C4R610\_KOMPG:C4R070|C4R070\_KOMPG:C4R2M8|C4R2M8\_KOMPG:C4QW40|C4QW40\_KOMPG:C4R2H6|C4R2H6\_KOMPG:C4R0G8|C4R0G8\_KOMPG:C4R460|C4R460\_KOMPG:C4R738|C4R738\_KOMPG:C4QZY2|C4QZY2\_KOMPG:C4R7H2|C4R7H2\_KOMPG:C4R4D7|C4R4D7\_KOMPG:C4R703|C4R703\_KOMPG:C4R0V2|C4R0V2\_KOMPG:C4QXL3|C4QXL3\_KOMPG:C4R2S8|C4R2S8\_KOMPG:C4QV13|C4QV13\_KOMPG:C4R2Z1|C4R2Z1\_KOMPG:C4R1I8|C4R1I8\_KOMPG:C4R8T9|C4R8T9\_KOMPG:C4QXD5|C4QXD5\_KOMPG:C4QZ04|C4QZ04\_KOMPG:C4R3M1|C4R3M1\_KOMPG:C4R2N9|C4R2N9\_KOMPG:C4R7G5|C4R7G5\_KOMPG |  |  | DB Search |
| SITP | 19.55 | 416.2271 | 4 | 2 | 417.2342 | 10.25 | 4029 | 3.69e3 | 1 | 1 | C4QWA7|C4QWA7\_KOMPG:C4R0R3|C4R0R3\_KOMPG:C4R6N9|C4R6N9\_KOMPG:C4R641|C4R641\_KOMPG:C4QZ65|C4QZ65\_KOMPG:C4R925|C4R925\_KOMPG:C4QZ59|C4QZ59\_KOMPG:C4R402|C4R402\_KOMPG:C4R728|C4R728\_KOMPG:C4R5X3|C4R5X3\_KOMPG:C4R7V7|C4R7V7\_KOMPG:C4QXG1|C4QXG1\_KOMPG:C4QYV4|C4QYV4\_KOMPG:C4QYT2|C4QYT2\_KOMPG:C4R7G9|C4R7G9\_KOMPG:C4QYA0|C4QYA0\_KOMPG:C4QXN8|C4QXN8\_KOMPG:C4R2U5|C4R2U5\_KOMPG:C4R569|C4R569\_KOMPG:C4R228|C4R228\_KOMPG:C4QZY5|C4QZY5\_KOMPG:C4QX93|C4QX93\_KOMPG:C4R3R7|C4R3R7\_KOMPG:C4QYU7|C4QYU7\_KOMPG:C4QZ26|C4QZ26\_KOMPG:C4R1C2|C4R1C2\_KOMPG:C4R841|C4R841\_KOMPG:C4QWW8|C4QWW8\_KOMPG:C4R2G7|C4R2G7\_KOMPG:C4R791|C4R791\_KOMPG:C4QZM2|C4QZM2\_KOMPG:C4R757|C4R757\_KOMPG:C4QWD1|C4QWD1\_KOMPG:C4R4L4|C4R4L4\_KOMPG:C4R2Q0|C4R2Q0\_KOMPG:C4R6T6|C4R6T6\_KOMPG:C4R655|C4R655\_KOMPG:C4R618|C4R618\_KOMPG:C4QYR7|C4QYR7\_KOMPG:C4QX95|C4QX95\_KOMPG:C4QVR1|C4QVR1\_KOMPG:C4R2C0|C4R2C0\_KOMPG:C4R342|C4R342\_KOMPG |  |  | DB Search |
| SLTP | 19.55 | 416.2271 | 4 | 2 | 417.2342 | 10.25 | 4029 | 3.69e3 | 1 | 1 | C4R0X1|C4R0X1\_KOMPG:C4R0R7|C4R0R7\_KOMPG:C4R811|C4R811\_KOMPG:C4QZD1|C4QZD1\_KOMPG:C4R025|C4R025\_KOMPG:C4R4G8|C4R4G8\_KOMPG:C4R2L2|C4R2L2\_KOMPG:C4R0B2|C4R0B2\_KOMPG:C4R1T5|C4R1T5\_KOMPG:C4QWU5|C4QWU5\_KOMPG:C4R0C0|C4R0C0\_KOMPG:C4QW80|C4QW80\_KOMPG:C4QYT0|C4QYT0\_KOMPG:C4QVJ6|C4QVJ6\_KOMPG:C4R5I9|C4R5I9\_KOMPG:C4R4J8|C4R4J8\_KOMPG:C4R3H6|C4R3H6\_KOMPG:C4R6D7|C4R6D7\_KOMPG:C4QYB0|C4QYB0\_KOMPG:C4R4N8|C4R4N8\_KOMPG:C4R8U4|C4R8U4\_KOMPG:C4R144|C4R144\_KOMPG:C4QVM3|C4QVM3\_KOMPG:C4R280|C4R280\_KOMPG:C4R7W4|C4R7W4\_KOMPG:C4QZ00|C4QZ00\_KOMPG:C4R2R5|C4R2R5\_KOMPG:C4QZZ1|C4QZZ1\_KOMPG:C4QV08|C4QV08\_KOMPG:C4R444|C4R444\_KOMPG:C4QZ73|C4QZ73\_KOMPG:C4R6N6|C4R6N6\_KOMPG:C4R7B0|C4R7B0\_KOMPG:C4R5P8|C4R5P8\_KOMPG:C4R5Q0|C4R5Q0\_KOMPG:C4QYP5|C4QYP5\_KOMPG:C4QV85|C4QV85\_KOMPG:C4QZ19|C4QZ19\_KOMPG:C4R1U8|C4R1U8\_KOMPG:C4R8D5|C4R8D5\_KOMPG:C4QVG5|C4QVG5\_KOMPG:C4R393|C4R393\_KOMPG:C4QZB6|C4QZB6\_KOMPG:C4R692|C4R692\_KOMPG:C4QWV2|C4QWV2\_KOMPG:C4R7M5|C4R7M5\_KOMPG:C4R9D0|C4R9D0\_KOMPG:C4R615|C4R615\_KOMPG:C4R317|C4R317\_KOMPG:C4R1W4|C4R1W4\_KOMPG:C4R2A5|C4R2A5\_KOMPG:C4R385|C4R385\_KOMPG:C4R3B5|C4R3B5\_KOMPG:C4R2Z9|C4R2Z9\_KOMPG:C4R2W5|C4R2W5\_KOMPG:C4R1D9|C4R1D9\_KOMPG:C4R2J9|C4R2J9\_KOMPG:C4R3P1|C4R3P1\_KOMPG |  |  | DB Search |
| QSVA | 19.54 | 403.2067 | 4 | -0.86 | 404.2126 | 2.86 | 1199 | 5.58e3 | 1 | 1 | C4QZL2|C4QZL2\_KOMPG:C4QWJ4|MDM10\_KOMPG:C4QV93|C4QV93\_KOMPG:C4QZH7|C4QZH7\_KOMPG:C4R1J6|C4R1J6\_KOMPG:C4R656|C4R656\_KOMPG:C4R5X2|C4R5X2\_KOMPG:C4R5Z0|C4R5Z0\_KOMPG:C4R6L5|C4R6L5\_KOMPG:C4QV51|C4QV51\_KOMPG:C4R1F2|C4R1F2\_KOMPG:C4R0Y6|C4R0Y6\_KOMPG:C4R172|C4R172\_KOMPG:C4R6W2|C4R6W2\_KOMPG:C4QYS8|C4QYS8\_KOMPG:C4QXA3|C4QXA3\_KOMPG:C4R2S6|C4R2S6\_KOMPG:C4R5M0|C4R5M0\_KOMPG:C4QYB7|C4QYB7\_KOMPG:C4R2V0|C4R2V0\_KOMPG:C4R3E9|C4R3E9\_KOMPG:C4QXV5|C4QXV5\_KOMPG:C4R2Z8|C4R2Z8\_KOMPG:C4QXU5|C4QXU5\_KOMPG:C4R571|C4R571\_KOMPG:C4QY10|C4QY10\_KOMPG:C4QV00|C4QV00\_KOMPG:C4R710|C4R710\_KOMPG:C4QWH8|C4QWH8\_KOMPG |  |  | DB Search |
| AAYH | 19.53 | 460.207 | 4 | 5.58 | 231.1115 | 13.04 | 5166 | 0 | 0 | 0 | C4QV61|C4QV61\_KOMPG:C4QW87|C4QW87\_KOMPG:C4R7W6|C4R7W6\_KOMPG:C4QYU8|C4QYU8\_KOMPG:C4R320|C4R320\_KOMPG:C4QWU8|C4QWU8\_KOMPG:C4R533|C4R533\_KOMPG |  |  | DB Search |
| E(-18.01)SRGAHA | 19.52 | 708.3303 | 7 | 8.42 | 709.3418 | 2.95 | 1189 | 4.44e2 | 1 | 1 | C4R8S1|C4R8S1\_KOMPG | Pyro-glu from E | E1:Pyro-glu from E:1000 | DB Search |
| PH | 19.52 | 252.1222 | 2 | -5.45 | 253.1275 | 5.57 | 2095 | 1.25e4 | 1 | 1 | C4QVY1|C4QVY1\_KOMPG:C4R3J0|C4R3J0\_KOMPG:C4R5U0|C4R5U0\_KOMPG:C4QV10|C4QV10\_KOMPG:C4R8H4|C4R8H4\_KOMPG:C4R452|C4R452\_KOMPG:C4R6K9|C4R6K9\_KOMPG:C4QZ17|C4QZ17\_KOMPG:C4R006|C4R006\_KOMPG:C4R214|C4R214\_KOMPG:C4QV84|C4QV84\_KOMPG:C4QZZ8|C4QZZ8\_KOMPG:C4R0D8|C4R0D8\_KOMPG:C4QWD7|C4QWD7\_KOMPG:C4R1P8|C4R1P8\_KOMPG:C4R8B9|C4R8B9\_KOMPG:C4R110|C4R110\_KOMPG:C4R2A4|C4R2A4\_KOMPG:C4R4G9|OXDA\_KOMPG:C4QVX4|C4QVX4\_KOMPG:C4QZ01|C4QZ01\_KOMPG:C4R661|C4R661\_KOMPG:C4R4K2|C4R4K2\_KOMPG:P53024|SEC13\_KOMPG:C4QVT4|C4QVT4\_KOMPG:C4R4X8|C4R4X8\_KOMPG:C4QX49|C4QX49\_KOMPG:C4R2C5|C4R2C5\_KOMPG:C4QYY0|C4QYY0\_KOMPG:C4QZJ6|C4QZJ6\_KOMPG:C4R5N8|C4R5N8\_KOMPG:C4QZH8|C4QZH8\_KOMPG:C4QVM0|C4QVM0\_KOMPG:C4R4C7|C4R4C7\_KOMPG:C4R5J2|C4R5J2\_KOMPG:C4R5K0|C4R5K0\_KOMPG:C4R263|C4R263\_KOMPG:C4QWR1|C4QWR1\_KOMPG:C4R8D3|C4R8D3\_KOMPG:C4QYV3|C4QYV3\_KOMPG:C4R3F2|C4R3F2\_KOMPG:C4QWH2|C4QWH2\_KOMPG:C4R366|C4R366\_KOMPG:C4R0E2|C4R0E2\_KOMPG:C4QZN7|C4QZN7\_KOMPG:C4QVP0|C4QVP0\_KOMPG:C4QX17|C4QX17\_KOMPG:C4R739|C4R739\_KOMPG:C4R990|C4R990\_KOMPG:C4R8Y4|C4R8Y4\_KOMPG:C4QZ47|C4QZ47\_KOMPG:C4QXM3|C4QXM3\_KOMPG:C4R2Y2|C4R2Y2\_KOMPG:C4QZJ4|C4QZJ4\_KOMPG:C4R7N8|C4R7N8\_KOMPG:C4QX78|C4QX78\_KOMPG:C4R5U2|C4R5U2\_KOMPG:C4QZK8|C4QZK8\_KOMPG:C4R0X4|C4R0X4\_KOMPG:C4R310|C4R310\_KOMPG:C4R7V7|C4R7V7\_KOMPG:C4R0V8|C4R0V8\_KOMPG:C4R447|C4R447\_KOMPG:C4QZV3|C4QZV3\_KOMPG:C4R4D6|C4R4D6\_KOMPG:C4R1H3|C4R1H3\_KOMPG:C4R443|C4R443\_KOMPG:C4R4F7|C4R4F7\_KOMPG:C4QVM1|C4QVM1\_KOMPG:C4R093|C4R093\_KOMPG:C4R6N0|C4R6N0\_KOMPG:C4R4B3|CEGT\_KOMPG:C4R0J7|C4R0J7\_KOMPG:C4R7B8|C4R7B8\_KOMPG:C4R4V7|C4R4V7\_KOMPG:C4R692|C4R692\_KOMPG:C4R8Y8|C4R8Y8\_KOMPG:C4R165|C4R165\_KOMPG:C4R6W1|C4R6W1\_KOMPG:C4R7R8|C4R7R8\_KOMPG:C4R6I2|C4R6I2\_KOMPG:C4R7C8|C4R7C8\_KOMPG:C4R1H7|C4R1H7\_KOMPG:C4R240|C4R240\_KOMPG:C4QWD1|C4QWD1\_KOMPG:C4R0Z6|C4R0Z6\_KOMPG:C4R2G2|C4R2G2\_KOMPG:C4R1B6|C4R1B6\_KOMPG:C4R332|C4R332\_KOMPG:C4R3U7|C4R3U7\_KOMPG:C4R095|C4R095\_KOMPG:C4R3H2|C4R3H2\_KOMPG:C4R8D9|C4R8D9\_KOMPG:C4QVK4|C4QVK4\_KOMPG:C4R625|C4R625\_KOMPG:C4R322|C4R322\_KOMPG:C4R6N4|C4R6N4\_KOMPG:C4R5C8|C4R5C8\_KOMPG:C4QX92|C4QX92\_KOMPG:C4R0N9|C4R0N9\_KOMPG:C4QZ38|C4QZ38\_KOMPG:C4R0S7|C4R0S7\_KOMPG:C4R0H0|C4R0H0\_KOMPG:C4R972|C4R972\_KOMPG:C4R2U1|C4R2U1\_KOMPG:C4R3I7|C4R3I7\_KOMPG:C4R1J4|C4R1J4\_KOMPG:C4R185|C4R185\_KOMPG:C4QWA3|C4QWA3\_KOMPG:C4QVA0|C4QVA0\_KOMPG:C4QXC0|C4QXC0\_KOMPG:C4R912|C4R912\_KOMPG:C4R5M5|C4R5M5\_KOMPG:C4R7W9|C4R7W9\_KOMPG:C4QYE9|C4QYE9\_KOMPG:C4R601|C4R601\_KOMPG:C4QXU3|C4QXU3\_KOMPG:C4R9G3|C4R9G3\_KOMPG:C4R9B2|C4R9B2\_KOMPG:C4QYT3|C4QYT3\_KOMPG:C4QZT4|C4QZT4\_KOMPG:C4R1G4|C4R1G4\_KOMPG:C4R707|C4R707\_KOMPG:C4R8L7|C4R8L7\_KOMPG:C4R717|C4R717\_KOMPG:C4R3T0|C4R3T0\_KOMPG:C4QXU5|C4QXU5\_KOMPG:C4R3Y2|C4R3Y2\_KOMPG:C4R2T3|C4R2T3\_KOMPG:C4QXB6|C4QXB6\_KOMPG:C4R5K6|C4R5K6\_KOMPG:C4R0K3|C4R0K3\_KOMPG:C4QYQ0|C4QYQ0\_KOMPG:C4R702|ALOX2\_KOMPG:C4QVT0|C4QVT0\_KOMPG:C4R450|C4R450\_KOMPG:C4R877|C4R877\_KOMPG:C4R921|C4R921\_KOMPG:C4R8S4|C4R8S4\_KOMPG:C4QXT4|C4QXT4\_KOMPG:C4R919|C4R919\_KOMPG:C4R1H2|C4R1H2\_KOMPG:C4R904|C4R904\_KOMPG:C4QW84|C4QW84\_KOMPG:C4R6Q5|C4R6Q5\_KOMPG:C4R7J7|C4R7J7\_KOMPG:C4R0I8|C4R0I8\_KOMPG:C4R039|C4R039\_KOMPG:C4R2V3|C4R2V3\_KOMPG:C4QZU1|C4QZU1\_KOMPG:C4R148|C4R148\_KOMPG:C4QZ69|C4QZ69\_KOMPG:C4R0X5|C4R0X5\_KOMPG:C4R6H9|C4R6H9\_KOMPG:C4R1Q3|C4R1Q3\_KOMPG:C4QYQ2|C4QYQ2\_KOMPG:C4R179|C4R179\_KOMPG:C4R3C3|C4R3C3\_KOMPG:C4QYY3|C4QYY3\_KOMPG:C4QV13|C4QV13\_KOMPG:C4R3I0|C4R3I0\_KOMPG:C4QWU8|C4QWU8\_KOMPG:C4R133|C4R133\_KOMPG:C4QZF2|C4QZF2\_KOMPG:C4R4U3|C4R4U3\_KOMPG:C4QXS9|C4QXS9\_KOMPG:C4QVS5|C4QVS5\_KOMPG:C4R5V4|C4R5V4\_KOMPG:C4R2K9|C4R2K9\_KOMPG:C4R5H2|C4R5H2\_KOMPG:C4QY10|C4QY10\_KOMPG:C4QXS6|C4QXS6\_KOMPG:C4QXD5|C4QXD5\_KOMPG:C4QXN9|C4QXN9\_KOMPG:C4R387|C4R387\_KOMPG:C4R649|C4R649\_KOMPG |  |  | DB Search |
| YVSALIM(+15.99) | 19.51 | 811.415 | 7 | 0.38 | 812.4205 | 44.03 | 13690 | 3.93e2 | 1 | 1 | C4QWV6|C4QWV6\_KOMPG | Oxidation (M) | M7:Oxidation (M):1000 | DB Search |
| LIGAP | 19.51 | 469.29 | 5 | -9.18 | 470.2918 | 12.51 | 4816 | 6.43e3 | 1 | 1 | C4R0B7|C4R0B7\_KOMPG:C4R1X5|C4R1X5\_KOMPG |  |  | DB Search |
| LLGAP | 19.51 | 469.29 | 5 | -9.18 | 470.2918 | 12.51 | 4816 | 6.43e3 | 1 | 1 | C4QXF2|C4QXF2\_KOMPG:C4R2X4|C4R2X4\_KOMPG:C4R717|C4R717\_KOMPG:C4QZP1|C4QZP1\_KOMPG |  |  | DB Search |
| HAIF | 19.48 | 486.2591 | 4 | -6.17 | 487.2621 | 15.47 | 6161 | 8e2 | 1 | 1 | C4QXW8|C4QXW8\_KOMPG:C4R035|C4R035\_KOMPG |  |  | DB Search |
| HALF | 19.48 | 486.2591 | 4 | -6.17 | 487.2621 | 15.47 | 6161 | 8e2 | 1 | 1 | C4R1B9|C4R1B9\_KOMPG:C4R1T4|C4R1T4\_KOMPG:C4R224|C4R224\_KOMPG:C4R2Q9|C4R2Q9\_KOMPG:C4QZG6|C4QZG6\_KOMPG:C4R174|C4R174\_KOMPG:C4R0G2|C4R0G2\_KOMPG:C4QWD1|C4QWD1\_KOMPG:C4QZB8|C4QZB8\_KOMPG:C4R1H0|C4R1H0\_KOMPG:C4R4H6|C4R4H6\_KOMPG:C4R1U8|C4R1U8\_KOMPG:C4QZC3|C4QZC3\_KOMPG:C4QW45|C4QW45\_KOMPG:C4QWY0|C4QWY0\_KOMPG |  |  | DB Search |
| AHSVHQ | 19.46 | 677.3245 | 6 | 4.29 | 678.333 | 15.22 | 6028 | 1.41e2 | 1 | 1 | C4QWP7|C4QWP7\_KOMPG |  |  | DB Search |
| E(-18.01)ISESE | 19.46 | 674.2759 | 6 | -6.05 | 675.2774 | 11.31 | 4525 | 0 | 0 | 0 | C4QZX1|C4QZX1\_KOMPG | Pyro-glu from E | E1:Pyro-glu from E:1000 | DB Search |
| AHVF | 19.44 | 472.2434 | 4 | -1.46 | 473.2488 | 10.18 | 3988 | 0 | 0 | 0 | C4QWD1|C4QWD1\_KOMPG:C4QV09|C4QV09\_KOMPG:C4R5C6|C4R5C6\_KOMPG:C4QY27|C4QY27\_KOMPG:C4QZ04|C4QZ04\_KOMPG |  |  | DB Search |
| PVLSVP | 19.43 | 610.369 | 6 | -0.89 | 611.3742 | 27.21 | 9600 | 1.8e3 | 1 | 1 | C4R3K9|C4R3K9\_KOMPG |  |  | DB Search |
| SGII | 19.42 | 388.2322 | 4 | -5.15 | 389.2365 | 17.34 | 6766 | 1.43e3 | 1 | 1 | C4R2C6|C4R2C6\_KOMPG:C4R4K5|C4R4K5\_KOMPG:C4R3B1|C4R3B1\_KOMPG:C4R5B2|C4R5B2\_KOMPG:C4R0D6|C4R0D6\_KOMPG:C4R6L2|C4R6L2\_KOMPG:Q9Y751|ATG26\_KOMPG:C4R4Q2|C4R4Q2\_KOMPG:C4QXG1|C4QXG1\_KOMPG:C4R5R5|C4R5R5\_KOMPG:C4R6H9|C4R6H9\_KOMPG:C4R4D6|C4R4D6\_KOMPG:C4R4L3|C4R4L3\_KOMPG:C4R3Q4|C4R3Q4\_KOMPG:C4QVU8|C4QVU8\_KOMPG:C4R7B2|C4R7B2\_KOMPG:C4R405|C4R405\_KOMPG:C4R6F0|PEX3\_KOMPG |  |  | DB Search |
| SGIL | 19.42 | 388.2322 | 4 | -5.15 | 389.2365 | 17.34 | 6766 | 1.43e3 | 1 | 1 | C4R341|C4R341\_KOMPG:C4QYG6|C4QYG6\_KOMPG:C4R5F3|C4R5F3\_KOMPG:C4R8L6|C4R8L6\_KOMPG:C4R3D7|C4R3D7\_KOMPG:C4R925|C4R925\_KOMPG:C4R2T7|C4R2T7\_KOMPG:C4R904|C4R904\_KOMPG:C4QVB9|C4QVB9\_KOMPG:C4QXI8|PEX6\_KOMPG:C4R6C2|PEX1\_KOMPG:C4R8Y2|C4R8Y2\_KOMPG:C4R333|C4R333\_KOMPG:C4R7D8|C4R7D8\_KOMPG:C4QXX4|C4QXX4\_KOMPG:C4R0N6|C4R0N6\_KOMPG:C4R3A3|C4R3A3\_KOMPG:C4R534|C4R534\_KOMPG:C4R134|C4R134\_KOMPG:C4R0Q0|C4R0Q0\_KOMPG:C4QW86|C4QW86\_KOMPG:C4R446|C4R446\_KOMPG:C4R6B0|OXDD\_KOMPG:C4R1Z8|C4R1Z8\_KOMPG:C4QZY8|C4QZY8\_KOMPG:C4QZS2|C4QZS2\_KOMPG:C4R012|C4R012\_KOMPG:C4R6N4|C4R6N4\_KOMPG |  |  | DB Search |
| SGLI | 19.42 | 388.2322 | 4 | -5.15 | 389.2365 | 17.34 | 6766 | 1.43e3 | 1 | 1 | C4R4T4|C4R4T4\_KOMPG:C4QYY6|C4QYY6\_KOMPG:C4QZ23|C4QZ23\_KOMPG:C4QZ18|C4QZ18\_KOMPG:C4R1U2|C4R1U2\_KOMPG:C4R039|C4R039\_KOMPG:C4R8N7|C4R8N7\_KOMPG:C4R3J3|C4R3J3\_KOMPG:C4R8R4|C4R8R4\_KOMPG:C4R1X4|C4R1X4\_KOMPG:C4QYJ6|C4QYJ6\_KOMPG:C4R6B2|C4R6B2\_KOMPG:C4R313|C4R313\_KOMPG:C4R8E5|C4R8E5\_KOMPG:C4R5K3|C4R5K3\_KOMPG:C4R6W3|C4R6W3\_KOMPG:C4R172|C4R172\_KOMPG:C4QYA5|C4QYA5\_KOMPG:C4R7M6|C4R7M6\_KOMPG:C4QX81|C4QX81\_KOMPG:C4R4R8|ARO1\_KOMPG:C4R371|C4R371\_KOMPG:C4R420|C4R420\_KOMPG:C4R0B4|C4R0B4\_KOMPG:C4QY08|C4QY08\_KOMPG |  |  | DB Search |
| SGLL | 19.42 | 388.2322 | 4 | -5.15 | 389.2365 | 17.34 | 6766 | 1.43e3 | 1 | 1 | C4R4U6|C4R4U6\_KOMPG:C4QZQ4|C4QZQ4\_KOMPG:C4R7I9|C4R7I9\_KOMPG:C4R3F4|C4R3F4\_KOMPG:C4R044|C4R044\_KOMPG:C4QYU4|C4QYU4\_KOMPG:C4R3C2|C4R3C2\_KOMPG:C4R4C2|C4R4C2\_KOMPG:C4QUZ7|C4QUZ7\_KOMPG:C4R895|C4R895\_KOMPG:C4R006|C4R006\_KOMPG:C4R3L0|C4R3L0\_KOMPG:C4QYH7|C4QYH7\_KOMPG:C4R0S9|C4R0S9\_KOMPG:C4R3C0|C4R3C0\_KOMPG:C4R4U4|C4R4U4\_KOMPG:C4R508|C4R508\_KOMPG:C4R2D2|C4R2D2\_KOMPG:C4R5P2|C4R5P2\_KOMPG:C4R0L7|C4R0L7\_KOMPG:C4R7I5|C4R7I5\_KOMPG:C4R7J8|C4R7J8\_KOMPG:C4QVT9|C4QVT9\_KOMPG:C4R8R6|C4R8R6\_KOMPG:C4R069|C4R069\_KOMPG:C4R4C7|C4R4C7\_KOMPG:C4R1Z9|C4R1Z9\_KOMPG:C4R3L8|C4R3L8\_KOMPG:C4R4J8|C4R4J8\_KOMPG:C4R5V4|C4R5V4\_KOMPG:C4QXI1|C4QXI1\_KOMPG:C4R092|C4R092\_KOMPG:C4R6C6|C4R6C6\_KOMPG:C4QVT0|C4QVT0\_KOMPG:C4R7Z4|C4R7Z4\_KOMPG:C4QXZ9|C4QXZ9\_KOMPG:C4QWS9|C4QWS9\_KOMPG:C4R0E2|C4R0E2\_KOMPG:C4QVP0|C4QVP0\_KOMPG:C4QYD4|C4QYD4\_KOMPG:C4QW72|C4QW72\_KOMPG:C4R8I9|C4R8I9\_KOMPG |  |  | DB Search |
| ISEPAGEP | 19.41 | 798.3759 | 8 | -6.48 | 799.376 | 23.32 | 8506 | 4.34e1 | 1 | 1 | C4R001|C4R001\_KOMPG |  |  | DB Search |
| ITGIAVIQ | 19.38 | 813.496 | 8 | -6.1 | 814.4963 | 28.75 | 10005 | 3.04e3 | 1 | 1 | C4R3D3|C4R3D3\_KOMPG |  |  | DB Search |
| NFSP | 19.33 | 463.2067 | 4 | -2.34 | 464.2117 | 8.58 | 3287 | 2.64e3 | 1 | 1 | C4R315|C4R315\_KOMPG:C4R124|C4R124\_KOMPG:C4R0L9|C4R0L9\_KOMPG:C4R8V7|C4R8V7\_KOMPG:C4QVH0|C4QVH0\_KOMPG:C4R1Q4|C4R1Q4\_KOMPG:C4QZB5|C4QZB5\_KOMPG:C4R7Z0|C4R7Z0\_KOMPG:C4R895|C4R895\_KOMPG:C4R119|C4R119\_KOMPG:C4QXT9|C4QXT9\_KOMPG:C4R9F0|C4R9F0\_KOMPG:C4R609|C4R609\_KOMPG:C4QYW0|C4QYW0\_KOMPG:C4R5E8|C4R5E8\_KOMPG:C4R8B1|C4R8B1\_KOMPG:C4QXD4|C4QXD4\_KOMPG:C4QWL3|C4QWL3\_KOMPG:C4R3M2|C4R3M2\_KOMPG:C4QXZ9|C4QXZ9\_KOMPG:C4R2J9|C4R2J9\_KOMPG |  |  | DB Search |
| GFDTP | 19.32 | 535.2278 | 5 | -5.91 | 536.2306 | 13.65 | 5375 | 0 | 0 | 0 | C4R937|C4R937\_KOMPG |  |  | DB Search |
| APVE | 19.31 | 414.2114 | 4 | -0.87 | 415.2173 | 3.61 | 1513 | 3.09e3 | 1 | 1 | C4QYE6|C4QYE6\_KOMPG:C4R1N1|C4R1N1\_KOMPG:C4R0V7|C4R0V7\_KOMPG:C4QY78|C4QY78\_KOMPG:C4R6R3|C4R6R3\_KOMPG:C4QYX8|C4QYX8\_KOMPG:C4R4X1|C4R4X1\_KOMPG:C4R232|C4R232\_KOMPG:C4QXZ8|C4QXZ8\_KOMPG:C4R7D8|C4R7D8\_KOMPG:C4R5W4|C4R5W4\_KOMPG:C4R7I3|C4R7I3\_KOMPG:C4R3S5|C4R3S5\_KOMPG:C4R8E1|C4R8E1\_KOMPG:C4R5M4|C4R5M4\_KOMPG:C4R198|C4R198\_KOMPG:C4R4U0|C4R4U0\_KOMPG:C4QWK1|C4QWK1\_KOMPG:C4QW80|C4QW80\_KOMPG:C4R1C2|C4R1C2\_KOMPG:C4R6R5|C4R6R5\_KOMPG:C4R5H9|C4R5H9\_KOMPG:C4QZC9|C4QZC9\_KOMPG:C4R6T4|C4R6T4\_KOMPG:C4QWZ8|C4QWZ8\_KOMPG:C4QX05|C4QX05\_KOMPG:C4QYD6|C4QYD6\_KOMPG:C4R5Z2|C4R5Z2\_KOMPG:C4R1A9|C4R1A9\_KOMPG:C4R8S3|C4R8S3\_KOMPG:C4R5Z8|C4R5Z8\_KOMPG:C4R587|C4R587\_KOMPG:C4R2G3|C4R2G3\_KOMPG |  |  | DB Search |
| IPGGI | 19.31 | 455.2744 | 5 | -4.8 | 456.2783 | 20.80 | 7704 | 5.46e3 | 1 | 1 | C4R380|C4R380\_KOMPG:C4R5F7|C4R5F7\_KOMPG:C4R713|C4R713\_KOMPG |  |  | DB Search |
| IPGGL | 19.31 | 455.2744 | 5 | -4.8 | 456.2783 | 20.80 | 7704 | 5.46e3 | 1 | 1 | C4R4U3|C4R4U3\_KOMPG:C4R700|C4R700\_KOMPG:C4QZ23|C4QZ23\_KOMPG:C4R313|C4R313\_KOMPG:C4R2A7|C4R2A7\_KOMPG:C4R9D8|C4R9D8\_KOMPG |  |  | DB Search |
| LPGGI | 19.31 | 455.2744 | 5 | -4.8 | 456.2783 | 20.80 | 7704 | 5.46e3 | 1 | 1 | C4QZX7|C4QZX7\_KOMPG:C4QW04|LCL3\_KOMPG:C4QYJ9|C4QYJ9\_KOMPG:C4R6H5|C4R6H5\_KOMPG:C4R264|C4R264\_KOMPG |  |  | DB Search |
| LPGGL | 19.31 | 455.2744 | 5 | -4.8 | 456.2783 | 20.80 | 7704 | 5.46e3 | 1 | 1 | C4R1I3|C4R1I3\_KOMPG |  |  | DB Search |
| SHFI | 19.3 | 502.254 | 4 | 3.85 | 503.2619 | 15.62 | 6238 | 4.84e2 | 1 | 1 | C4R3P0|C4R3P0\_KOMPG:C4R2U7|C4R2U7\_KOMPG:C4R696|C4R696\_KOMPG:C4QV60|C4QV60\_KOMPG:C4R5C7|C4R5C7\_KOMPG:C4R243|C4R243\_KOMPG:C4R2K6|C4R2K6\_KOMPG:C4R4R8|ARO1\_KOMPG:C4R7I6|C4R7I6\_KOMPG:C4QXW4|C4QXW4\_KOMPG:C4QXC3|C4QXC3\_KOMPG:C4R1R5|C4R1R5\_KOMPG:C4R377|C4R377\_KOMPG:C4QW24|C4QW24\_KOMPG:C4R1U6|C4R1U6\_KOMPG:C4R3I4|C4R3I4\_KOMPG |  |  | DB Search |
| SHFL | 19.3 | 502.254 | 4 | 3.85 | 503.2619 | 15.62 | 6238 | 4.84e2 | 1 | 1 | C4QZT2|C4QZT2\_KOMPG:C4R6G3|C4R6G3\_KOMPG:C4R3I8|C4R3I8\_KOMPG:C4QZU5|C4QZU5\_KOMPG:C4QYI6|C4QYI6\_KOMPG:C4R410|C4R410\_KOMPG:C4QXQ1|C4QXQ1\_KOMPG:C4QVM0|C4QVM0\_KOMPG:C4QV87|C4QV87\_KOMPG:C4QX47|C4QX47\_KOMPG:C4R049|C4R049\_KOMPG:C4R3E6|C4R3E6\_KOMPG:C4R1C7|C4R1C7\_KOMPG:C4R942|C4R942\_KOMPG:C4R893|C4R893\_KOMPG:C4R6Z1|C4R6Z1\_KOMPG:C4R1M0|C4R1M0\_KOMPG:C4QWP9|C4QWP9\_KOMPG:C4QV07|C4QV07\_KOMPG |  |  | DB Search |
| TAY | 19.3 | 353.1587 | 3 | -1.32 | 354.1646 | 12.51 | 4913 | 1.39e3 | 1 | 1 | C4QZA8|C4QZA8\_KOMPG:C4R6K9|C4R6K9\_KOMPG:C4R6H5|C4R6H5\_KOMPG:C4QVW3|C4QVW3\_KOMPG:C4R5L8|C4R5L8\_KOMPG:C4R0R0|C4R0R0\_KOMPG:C4QYL8|C4QYL8\_KOMPG:C4R564|C4R564\_KOMPG:C4QY98|C4QY98\_KOMPG:C4R1R9|C4R1R9\_KOMPG:C4R3V6|C4R3V6\_KOMPG:C4R3L9|C4R3L9\_KOMPG:C4QXR4|C4QXR4\_KOMPG:C4R6M3|C4R6M3\_KOMPG:C4R0H4|C4R0H4\_KOMPG:C4QYY2|C4QYY2\_KOMPG:C4R8N3|C4R8N3\_KOMPG:C4R1X5|C4R1X5\_KOMPG:C4QX39|C4QX39\_KOMPG:C4R1I0|C4R1I0\_KOMPG:C4R0C0|C4R0C0\_KOMPG:C4QVP5|C4QVP5\_KOMPG:C4QWS1|C4QWS1\_KOMPG:C4QYN2|C4QYN2\_KOMPG:C4QY41|C4QY41\_KOMPG:C4QYN9|C4QYN9\_KOMPG:C4R1Z5|C4R1Z5\_KOMPG:C4QY37|C4QY37\_KOMPG:C4R8H6|C4R8H6\_KOMPG:C4QW38|C4QW38\_KOMPG:C4R3C4|C4R3C4\_KOMPG:C4R9A4|C4R9A4\_KOMPG:C4R053|C4R053\_KOMPG:C4R315|C4R315\_KOMPG:C4QZK9|C4QZK9\_KOMPG:C4R3C2|C4R3C2\_KOMPG:C4R6Q8|C4R6Q8\_KOMPG:C4R091|C4R091\_KOMPG:C4R926|C4R926\_KOMPG:C4R8X5|C4R8X5\_KOMPG:C4R7V7|C4R7V7\_KOMPG:C4QY87|C4QY87\_KOMPG:C4R3N7|C4R3N7\_KOMPG:C4R162|C4R162\_KOMPG:C4R2U5|C4R2U5\_KOMPG:C4R6D1|C4R6D1\_KOMPG:C4R5J9|C4R5J9\_KOMPG:C4R306|C4R306\_KOMPG:C4R0Y6|C4R0Y6\_KOMPG:C4R2C6|C4R2C6\_KOMPG:C4QV28|C4QV28\_KOMPG:C4R1M6|C4R1M6\_KOMPG:C4R6D4|C4R6D4\_KOMPG:C4R3A8|C4R3A8\_KOMPG:C4QXQ4|C4QXQ4\_KOMPG:C4R9E1|C4R9E1\_KOMPG:C4R3V2|C4R3V2\_KOMPG:C4QZ99|C4QZ99\_KOMPG:C4R2X0|C4R2X0\_KOMPG:C4R3P1|C4R3P1\_KOMPG:C4R6T3|C4R6T3\_KOMPG:C4R3B3|C4R3B3\_KOMPG:C4R5A2|C4R5A2\_KOMPG:C4R554|C4R554\_KOMPG:C4QW29|C4QW29\_KOMPG:C4R1P4|C4R1P4\_KOMPG:C4QXK7|C4QXK7\_KOMPG:C4R5C6|C4R5C6\_KOMPG:C4R5M9|C4R5M9\_KOMPG:C4R5D2|C4R5D2\_KOMPG:C4R6L7|C4R6L7\_KOMPG:C4QY32|C4QY32\_KOMPG:C4R4V8|C4R4V8\_KOMPG:C4QVD8|C4QVD8\_KOMPG:C4R8P4|C4R8P4\_KOMPG:C4QX31|C4QX31\_KOMPG:C4R3Y2|C4R3Y2\_KOMPG:C4QX72|C4QX72\_KOMPG:C4R525|C4R525\_KOMPG:C4R6G7|C4R6G7\_KOMPG:C4R5I2|C4R5I2\_KOMPG:C4R710|C4R710\_KOMPG:C4R3P0|C4R3P0\_KOMPG:C4R079|C4R079\_KOMPG:C4QYY6|C4QYY6\_KOMPG:C4R2G0|C4R2G0\_KOMPG:C4QYW7|C4QYW7\_KOMPG:C4R0F3|C4R0F3\_KOMPG:C4QV22|C4QV22\_KOMPG:C4R8K7|C4R8K7\_KOMPG:C4R4Z9|C4R4Z9\_KOMPG:C4R4D1|C4R4D1\_KOMPG:C4QXI0|C4QXI0\_KOMPG:C4R6F5|C4R6F5\_KOMPG:C4R2U0|C4R2U0\_KOMPG:C4R6H9|C4R6H9\_KOMPG:C4R6L5|C4R6L5\_KOMPG:C4QYN5|C4QYN5\_KOMPG:C4QYT1|C4QYT1\_KOMPG:C4R8I0|C4R8I0\_KOMPG:C4QV05|C4QV05\_KOMPG:C4QYN6|C4QYN6\_KOMPG:C4R7M6|C4R7M6\_KOMPG:C4QWC3|C4QWC3\_KOMPG:C4R3W8|C4R3W8\_KOMPG:C4QXJ7|C4QXJ7\_KOMPG:C4R024|C4R024\_KOMPG:C4R4P3|C4R4P3\_KOMPG:C4R7L2|C4R7L2\_KOMPG:C4R5P7|CCM1\_KOMPG:C4R749|C4R749\_KOMPG:C4QXW0|C4QXW0\_KOMPG:C4QYK4|C4QYK4\_KOMPG:C4R176|C4R176\_KOMPG:C4R1D2|C4R1D2\_KOMPG:C4R128|C4R128\_KOMPG:C4R8T0|C4R8T0\_KOMPG:C4R9B5|C4R9B5\_KOMPG:C4R003|C4R003\_KOMPG:C4R8J6|C4R8J6\_KOMPG:C4R4A3|C4R4A3\_KOMPG:C4R4I1|C4R4I1\_KOMPG:C4R8D4|C4R8D4\_KOMPG:C4R471|C4R471\_KOMPG:C4R0X2|C4R0X2\_KOMPG:C4QWU6|C4QWU6\_KOMPG:C4QV32|C4QV32\_KOMPG:C4R1P2|C4R1P2\_KOMPG:C4QXM1|C4QXM1\_KOMPG:C4R829|C4R829\_KOMPG:C4R323|C4R323\_KOMPG:C4QZB9|C4QZB9\_KOMPG:C4R7Q5|EXO5\_KOMPG:C4QVM0|C4QVM0\_KOMPG:C4QXZ0|C4QXZ0\_KOMPG:C4QWE9|C4QWE9\_KOMPG:C4QV95|C4QV95\_KOMPG:C4R701|C4R701\_KOMPG:C4QYE3|C4QYE3\_KOMPG:C4QX78|C4QX78\_KOMPG:C4QZW2|C4QZW2\_KOMPG:C4R455|C4R455\_KOMPG:C4QZ03|C4QZ03\_KOMPG:C4QVX1|C4QVX1\_KOMPG:C4R6B3|C4R6B3\_KOMPG:C4QX96|C4QX96\_KOMPG:C4QXN3|C4QXN3\_KOMPG:C4R7E3|C4R7E3\_KOMPG:C4R4B1|C4R4B1\_KOMPG:C4R4L6|C4R4L6\_KOMPG:C4QZN1|C4QZN1\_KOMPG:C4R335|C4R335\_KOMPG:C4QXE0|C4QXE0\_KOMPG:C4R6G5|C4R6G5\_KOMPG:C4QVM7|C4QVM7\_KOMPG:C4R4K5|C4R4K5\_KOMPG:C4R7B8|C4R7B8\_KOMPG:C4R9A7|C4R9A7\_KOMPG:C4QVW2|C4QVW2\_KOMPG:C4QYE4|C4QYE4\_KOMPG:C4R3P5|C4R3P5\_KOMPG:C4QZ96|C4QZ96\_KOMPG:C4R8C7|C4R8C7\_KOMPG:C4R773|C4R773\_KOMPG:C4R135|C4R135\_KOMPG:C4R3J2|C4R3J2\_KOMPG:C4QZR5|C4QZR5\_KOMPG:C4QVY3|C4QVY3\_KOMPG:C4R5R7|C4R5R7\_KOMPG:C4R583|C4R583\_KOMPG:C4QW03|C4QW03\_KOMPG:C4QY67|C4QY67\_KOMPG:C4QVU5|C4QVU5\_KOMPG:C4QVS9|C4QVS9\_KOMPG:C4QYV4|C4QYV4\_KOMPG:C4QXP8|C4QXP8\_KOMPG:C4R6X4|C4R6X4\_KOMPG:C4QV90|C4QV90\_KOMPG:C4QX48|C4QX48\_KOMPG:C4R6T8|C4R6T8\_KOMPG:C4QW90|C4QW90\_KOMPG:C4R3C1|C4R3C1\_KOMPG:C4R090|C4R090\_KOMPG:C4R7K1|C4R7K1\_KOMPG:C4QZ36|C4QZ36\_KOMPG:C4QYT0|C4QYT0\_KOMPG:C4R150|C4R150\_KOMPG:C4R657|C4R657\_KOMPG:C4QWC0|C4QWC0\_KOMPG:C4QZU2|C4QZU2\_KOMPG:C4R314|C4R314\_KOMPG:C4QXQ5|C4QXQ5\_KOMPG:C4R0X8|C4R0X8\_KOMPG:C4R3B8|C4R3B8\_KOMPG:C4R547|C4R547\_KOMPG:C4QYG9|C4QYG9\_KOMPG:C4R8W1|C4R8W1\_KOMPG:C4R3W3|C4R3W3\_KOMPG:C4R776|C4R776\_KOMPG:C4QZ18|C4QZ18\_KOMPG:C4QZ48|C4QZ48\_KOMPG:C4R7I2|C4R7I2\_KOMPG:C4R659|C4R659\_KOMPG:C4R774|C4R774\_KOMPG:C4R2H6|C4R2H6\_KOMPG:C4R7G9|C4R7G9\_KOMPG:C4R5L2|C4R5L2\_KOMPG:C4R5K9|C4R5K9\_KOMPG:C4R3R5|C4R3R5\_KOMPG:C4QWW4|C4QWW4\_KOMPG:C4R984|C4R984\_KOMPG:C4QYB8|C4QYB8\_KOMPG:C4R7P4|C4R7P4\_KOMPG:C4R516|C4R516\_KOMPG:C4R2S8|C4R2S8\_KOMPG:C4R9A9|C4R9A9\_KOMPG:C4R3Q8|C4R3Q8\_KOMPG:C4QY02|C4QY02\_KOMPG:C4QVZ6|C4QVZ6\_KOMPG:C4R2Z1|C4R2Z1\_KOMPG:C4QZM9|C4QZM9\_KOMPG:C4R4M5|C4R4M5\_KOMPG |  |  | DB Search |
| FPS | 19.27 | 349.1638 | 3 | -4.81 | 350.1685 | 16.32 | 6461 | 1.41e3 | 1 | 1 | C4QVZ8|C4QVZ8\_KOMPG:C4QYG6|C4QYG6\_KOMPG:C4R4L2|C4R4L2\_KOMPG:C4QX07|C4QX07\_KOMPG:C4QVN9|C4QVN9\_KOMPG:C4R214|C4R214\_KOMPG:C4R1A5|C4R1A5\_KOMPG:C4R3Q5|C4R3Q5\_KOMPG:C4QYR2|C4QYR2\_KOMPG:C4R948|C4R948\_KOMPG:C4R6C5|C4R6C5\_KOMPG:C4R5Q9|C4R5Q9\_KOMPG:C4QWY2|C4QWY2\_KOMPG:C4QW53|C4QW53\_KOMPG:C4R7V4|C4R7V4\_KOMPG:C4QY05|C4QY05\_KOMPG:C4R1L3|C4R1L3\_KOMPG:C4R812|C4R812\_KOMPG:C4R6B2|C4R6B2\_KOMPG:C4R0U6|C4R0U6\_KOMPG:C4QYX9|C4QYX9\_KOMPG:C4QZX7|C4QZX7\_KOMPG:C4R768|C4R768\_KOMPG:C4QV42|C4QV42\_KOMPG:C4R7E6|C4R7E6\_KOMPG:C4QYQ3|C4QYQ3\_KOMPG:C4R4R8|ARO1\_KOMPG:C4R571|C4R571\_KOMPG:C4QYV9|C4QYV9\_KOMPG:C4R4M7|C4R4M7\_KOMPG:C4R1S8|C4R1S8\_KOMPG:C4QYE3|C4QYE3\_KOMPG:C4QW45|C4QW45\_KOMPG:C4QVB4|C4QVB4\_KOMPG:C4QWR3|C4QWR3\_KOMPG:C4R052|C4R052\_KOMPG:C4R6H7|C4R6H7\_KOMPG:C4R635|C4R635\_KOMPG:C4QY85|C4QY85\_KOMPG:C4QY76|C4QY76\_KOMPG:C4R7N8|C4R7N8\_KOMPG:C4QX78|C4QX78\_KOMPG:C4QYZ3|C4QYZ3\_KOMPG:C4QZ13|C4QZ13\_KOMPG:C4R3L0|C4R3L0\_KOMPG:C4R310|C4R310\_KOMPG:C4QYA8|C4QYA8\_KOMPG:C4R833|C4R833\_KOMPG:C4QV54|C4QV54\_KOMPG:C4R7F9|C4R7F9\_KOMPG:C4R1H3|C4R1H3\_KOMPG:C4QYP7|C4QYP7\_KOMPG:C4R340|C4R340\_KOMPG:C4R4Z1|C4R4Z1\_KOMPG:C4QY65|C4QY65\_KOMPG:C4R685|C4R685\_KOMPG:C4QYV6|C4QYV6\_KOMPG:C4R172|C4R172\_KOMPG:C4R527|C4R527\_KOMPG:C4R252|C4R252\_KOMPG:C4R950|C4R950\_KOMPG:C4R393|C4R393\_KOMPG:C4R692|C4R692\_KOMPG:C4QVT9|C4QVT9\_KOMPG:C4QYX6|C4QYX6\_KOMPG:C4R165|C4R165\_KOMPG:C4QZ26|C4QZ26\_KOMPG:C4QWP8|C4QWP8\_KOMPG:C4R243|C4R243\_KOMPG:C4QZA7|C4QZA7\_KOMPG:C4R1T0|C4R1T0\_KOMPG:C4R1R6|C4R1R6\_KOMPG:C4R2G2|C4R2G2\_KOMPG:C4R6M2|C4R6M2\_KOMPG:C4QW98|C4QW98\_KOMPG:C4R2F4|C4R2F4\_KOMPG:C4R3H2|C4R3H2\_KOMPG:C4R322|C4R322\_KOMPG:C4QZQ4|C4QZQ4\_KOMPG:Q9Y751|ATG26\_KOMPG:C4R291|C4R291\_KOMPG:C4R8D0|C4R8D0\_KOMPG:C4R972|C4R972\_KOMPG:C4R8N7|C4R8N7\_KOMPG:C4R4M1|C4R4M1\_KOMPG:C4R0B0|C4R0B0\_KOMPG:C4QZF8|C4QZF8\_KOMPG:C4R568|C4R568\_KOMPG:C4R751|C4R751\_KOMPG:C4R0U8|C4R0U8\_KOMPG:C4R4I2|C4R4I2\_KOMPG:C4QV21|C4QV21\_KOMPG:C4R2P9|C4R2P9\_KOMPG:C4QXU3|C4QXU3\_KOMPG:C4QY28|C4QY28\_KOMPG:C4R153|C4R153\_KOMPG:C4QXF9|C4QXF9\_KOMPG:C4R539|C4R539\_KOMPG:C4R2Q4|C4R2Q4\_KOMPG:C4R8S4|C4R8S4\_KOMPG:C4R919|C4R919\_KOMPG:C4QWU3|C4QWU3\_KOMPG:C4QW40|C4QW40\_KOMPG:C4R7W8|C4R7W8\_KOMPG:C4QZ69|C4QZ69\_KOMPG:C4R4R0|C4R4R0\_KOMPG:C4R848|C4R848\_KOMPG:C4QW18|C4QW18\_KOMPG:C4R2H4|C4R2H4\_KOMPG:C4QVZ3|C4QVZ3\_KOMPG:C4R5P2|C4R5P2\_KOMPG:C4R3R7|C4R3R7\_KOMPG:C4R1I3|C4R1I3\_KOMPG:C4R3I0|C4R3I0\_KOMPG:C4R8K4|C4R8K4\_KOMPG:C4R4T5|C4R4T5\_KOMPG:C4R2Z1|C4R2Z1\_KOMPG:C4R065|C4R065\_KOMPG:C4R4H7|C4R4H7\_KOMPG:C4R387|C4R387\_KOMPG:C4R3A9|C4R3A9\_KOMPG |  |  | DB Search |
| KGY | 19.27 | 366.1903 | 3 | -9.39 | 367.1932 | 6.66 | 2561 | 3.55e1 | 1 | 1 | C4R3F1|C4R3F1\_KOMPG:C4QX99|C4QX99\_KOMPG:C4QWE6|C4QWE6\_KOMPG:C4R948|C4R948\_KOMPG:C4R564|C4R564\_KOMPG:C4R816|C4R816\_KOMPG:C4R049|C4R049\_KOMPG:C4R036|C4R036\_KOMPG:C4QWM8|C4QWM8\_KOMPG:C4R6M3|C4R6M3\_KOMPG:C4R8Q8|C4R8Q8\_KOMPG:C4R383|C4R383\_KOMPG:C4R7Z2|C4R7Z2\_KOMPG:C4QW51|C4QW51\_KOMPG:C4R0U6|C4R0U6\_KOMPG:C4R1I0|C4R1I0\_KOMPG:C4QVK9|C4QVK9\_KOMPG:C4R1T4|C4R1T4\_KOMPG:C4QY73|C4QY73\_KOMPG:C4QW52|C4QW52\_KOMPG:C4R6V1|C4R6V1\_KOMPG:C4QXU2|C4QXU2\_KOMPG:C4R0Q6|C4R0Q6\_KOMPG:C4QZJ6|C4QZJ6\_KOMPG:C4R1T9|ENOPH\_KOMPG:C4R019|C4R019\_KOMPG:C4R968|C4R968\_KOMPG:C4QZG3|C4QZG3\_KOMPG:C4QZI2|C4QZI2\_KOMPG:C4QYJ2|C4QYJ2\_KOMPG:C4R1X9|C4R1X9\_KOMPG:C4R571|C4R571\_KOMPG:C4QYK8|C4QYK8\_KOMPG:C4R6H1|C4R6H1\_KOMPG:C4QYM4|C4QYM4\_KOMPG:C4QWH2|C4QWH2\_KOMPG:C4R3D9|C4R3D9\_KOMPG:C4R144|C4R144\_KOMPG:C4QYN9|C4QYN9\_KOMPG:C4R2F8|C4R2F8\_KOMPG:C4R954|C4R954\_KOMPG:C4R8A9|C4R8A9\_KOMPG:C4QY01|C4QY01\_KOMPG:C4R315|C4R315\_KOMPG:C4R3G8|C4R3G8\_KOMPG:C4R7X8|BMT2\_KOMPG:C4QYU4|C4QYU4\_KOMPG:C4R926|C4R926\_KOMPG:C4R3L0|C4R3L0\_KOMPG:C4QYA8|C4QYA8\_KOMPG:C4R9E6|C4R9E6\_KOMPG:C4R481|C4R481\_KOMPG:C4QY53|C4QY53\_KOMPG:C4R162|C4R162\_KOMPG:C4R7J3|C4R7J3\_KOMPG:C4R3V9|C4R3V9\_KOMPG:C4R6D1|C4R6D1\_KOMPG:C4QVP1|C4QVP1\_KOMPG:C4QY65|C4QY65\_KOMPG:C4QWI2|C4QWI2\_KOMPG:C4QX26|C4QX26\_KOMPG:C4QXJ3|C4QXJ3\_KOMPG:C4R453|C4R453\_KOMPG:C4R4B3|CEGT\_KOMPG:C4QWP7|C4QWP7\_KOMPG:C4QVI7|C4QVI7\_KOMPG:C4R288|C4R288\_KOMPG:C4QZB6|C4QZB6\_KOMPG:C4R0L3|C4R0L3\_KOMPG:C4QWY6|C4QWY6\_KOMPG:C4R947|C4R947\_KOMPG:C4QWD1|C4QWD1\_KOMPG:C4R3A8|C4R3A8\_KOMPG:C4QXQ4|C4QXQ4\_KOMPG:C4QZ42|C4QZ42\_KOMPG:C4QWK9|C4QWK9\_KOMPG:C4QVK4|C4QVK4\_KOMPG:C4R0E1|C4R0E1\_KOMPG:C4R2P2|C4R2P2\_KOMPG:C4R852|C4R852\_KOMPG:C4R5X3|C4R5X3\_KOMPG:C4R1P4|C4R1P4\_KOMPG:C4QXQ2|C4QXQ2\_KOMPG:C4R5C6|C4R5C6\_KOMPG:C4R4H8|C4R4H8\_KOMPG:C4R011|C4R011\_KOMPG:C4R5U6|C4R5U6\_KOMPG:C4R1M2|C4R1M2\_KOMPG:C4R5L5|C4R5L5\_KOMPG:C4QY32|C4QY32\_KOMPG:C4R956|C4R956\_KOMPG:C4QVA7|C4QVA7\_KOMPG:C4QXW9|C4QXW9\_KOMPG:C4R5M5|C4R5M5\_KOMPG:C4R3Z8|C4R3Z8\_KOMPG:C4R5B8|C4R5B8\_KOMPG:C4R3N3|C4R3N3\_KOMPG:C4R7W9|C4R7W9\_KOMPG:C4R8M0|C4R8M0\_KOMPG:C4QVF4|C4QVF4\_KOMPG:C4R0Y3|C4R0Y3\_KOMPG:C4QVX5|C4QVX5\_KOMPG:C4R2F2|C4R2F2\_KOMPG:C4R7D6|C4R7D6\_KOMPG:C4R5R5|C4R5R5\_KOMPG:C4QW41|C4QW41\_KOMPG:C4QVH4|C4QVH4\_KOMPG:C4R3P0|C4R3P0\_KOMPG:C4R0C2|C4R0C2\_KOMPG:C4QVZ2|C4QVZ2\_KOMPG:C4QV69|C4QV69\_KOMPG:C4R851|C4R851\_KOMPG:C4QY61|C4QY61\_KOMPG:C4R0F3|C4R0F3\_KOMPG:C4R1J5|C4R1J5\_KOMPG:C4R8A3|C4R8A3\_KOMPG:C4QXG1|C4QXG1\_KOMPG:C4R4U5|C4R4U5\_KOMPG:C4R645|C4R645\_KOMPG:C4R299|C4R299\_KOMPG:C4R0P8|C4R0P8\_KOMPG:C4R066|C4R066\_KOMPG:C4R0X6|C4R0X6\_KOMPG:C4R4A2|C4R4A2\_KOMPG:C4QX00|C4QX00\_KOMPG:C4QWB6|C4QWB6\_KOMPG:C4QVE3|C4QVE3\_KOMPG:C4R0X3|C4R0X3\_KOMPG:C4QYX7|C4QYX7\_KOMPG:C4QX90|C4QX90\_KOMPG:C4QYA2|C4QYA2\_KOMPG:C4QWA1|C4QWA1\_KOMPG:C4R5D7|C4R5D7\_KOMPG:C4R439|C4R439\_KOMPG:C4QZH3|C4QZH3\_KOMPG:C4R9D9|C4R9D9\_KOMPG:C4R2Q7|C4R2Q7\_KOMPG:C4R4X9|C4R4X9\_KOMPG:C4R5G1|C4R5G1\_KOMPG:C4QWC8|C4QWC8\_KOMPG:C4R936|C4R936\_KOMPG:C4QXP5|C4QXP5\_KOMPG:C4R2R7|C4R2R7\_KOMPG:C4R609|C4R609\_KOMPG:C4R2R4|C4R2R4\_KOMPG:C4QVZ7|C4QVZ7\_KOMPG:C4R3W9|C4R3W9\_KOMPG:C4R4X2|C4R4X2\_KOMPG:C4R1B1|C4R1B1\_KOMPG:C4QXW7|C4QXW7\_KOMPG:C4R0U4|C4R0U4\_KOMPG:C4R6Z9|C4R6Z9\_KOMPG:C4QXZ6|C4QXZ6\_KOMPG:C4R716|C4R716\_KOMPG:C4R5D1|C4R5D1\_KOMPG:C4QW86|C4QW86\_KOMPG:C4R742|C4R742\_KOMPG:C4QXM1|C4QXM1\_KOMPG:C4R7E8|C4R7E8\_KOMPG:C4R5A8|C4R5A8\_KOMPG:C4QWM2|C4QWM2\_KOMPG:C4QXR7|C4QXR7\_KOMPG:C4R6E1|C4R6E1\_KOMPG:C4R8V2|C4R8V2\_KOMPG:C4R263|C4R263\_KOMPG:C4R5R1|C4R5R1\_KOMPG:C4R4L4|C4R4L4\_KOMPG:C4R420|C4R420\_KOMPG:C4R4Q3|C4R4Q3\_KOMPG:C4R0X0|C4R0X0\_KOMPG:C4QXI3|C4QXI3\_KOMPG:C4R0L0|C4R0L0\_KOMPG:C4R2R5|C4R2R5\_KOMPG:C4QY76|C4QY76\_KOMPG:C4QVX3|C4QVX3\_KOMPG:C4QY33|C4QY33\_KOMPG:C4R7F9|C4R7F9\_KOMPG:C4QXW4|C4QXW4\_KOMPG:C4R1R4|C4R1R4\_KOMPG:C4R517|C4R517\_KOMPG:C4R4G1|C4R4G1\_KOMPG:C4QYA6|C4QYA6\_KOMPG:C4R9B9|C4R9B9\_KOMPG:C4R2D2|C4R2D2\_KOMPG:C4QWE8|C4QWE8\_KOMPG:C4R0A2|C4R0A2\_KOMPG:C4R1U8|C4R1U8\_KOMPG:C4R1M0|C4R1M0\_KOMPG:C4R0W4|C4R0W4\_KOMPG:C4R4T6|C4R4T6\_KOMPG:C4R335|C4R335\_KOMPG:C4R678|C4R678\_KOMPG:C4R5L1|C4R5L1\_KOMPG:C4R7Q2|C4R7Q2\_KOMPG:C4R664|C4R664\_KOMPG:C4QWT5|C4QWT5\_KOMPG:C4QWM1|C4QWM1\_KOMPG:C4R6G1|C4R6G1\_KOMPG:C4QVQ4|C4QVQ4\_KOMPG:C4R2D8|C4R2D8\_KOMPG:C4R1W7|C4R1W7\_KOMPG:C4QV37|C4QV37\_KOMPG:C4QW15|C4QW15\_KOMPG:C4R4U1|C4R4U1\_KOMPG:C4QZX4|C4QZX4\_KOMPG:C4QV23|C4QV23\_KOMPG:C4QZ58|C4QZ58\_KOMPG:C4QWI8|C4QWI8\_KOMPG:C4R217|C4R217\_KOMPG:C4R0X7|C4R0X7\_KOMPG:C4R907|C4R907\_KOMPG:C4R4L7|C4R4L7\_KOMPG:C4R708|C4R708\_KOMPG:C4R3H9|C4R3H9\_KOMPG:C4R0B7|C4R0B7\_KOMPG:C4R1U9|C4R1U9\_KOMPG:C4R663|C4R663\_KOMPG:C4QVW1|C4QVW1\_KOMPG:C4QV12|C4QV12\_KOMPG:C4QYR3|C4QYR3\_KOMPG:C4R1H5|C4R1H5\_KOMPG:C4R6L8|C4R6L8\_KOMPG:C4QXB7|C4QXB7\_KOMPG:C4R1E3|C4R1E3\_KOMPG:C4R2N4|C4R2N4\_KOMPG:C4R0J8|C4R0J8\_KOMPG:C4QY34|C4QY34\_KOMPG:C4R0H5|C4R0H5\_KOMPG:C4QX95|C4QX95\_KOMPG:C4R4B7|C4R4B7\_KOMPG:C4R8W1|C4R8W1\_KOMPG:C4R9C0|C4R9C0\_KOMPG:C4R877|C4R877\_KOMPG:C4R2Q9|C4R2Q9\_KOMPG:C4QZ18|C4QZ18\_KOMPG:C4R2A1|C4R2A1\_KOMPG:C4QXH2|AIM24\_KOMPG:C4R5U1|C4R5U1\_KOMPG:C4R5W2|C4R5W2\_KOMPG:C4R2H6|C4R2H6\_KOMPG:C4R0G8|C4R0G8\_KOMPG:C4QYG2|C4QYG2\_KOMPG:C4QWW2|C4QWW2\_KOMPG:C4R316|C4R316\_KOMPG:C4QVF0|C4QVF0\_KOMPG:C4R4Y1|C4R4Y1\_KOMPG:C4R3E0|C4R3E0\_KOMPG:C4QYY3|C4QYY3\_KOMPG:C4QVL4|C4QVL4\_KOMPG:C4R875|C4R875\_KOMPG:C4R5C9|C4R5C9\_KOMPG:C4QXA6|C4QXA6\_KOMPG:C4QWU8|C4QWU8\_KOMPG:C4R1C9|C4R1C9\_KOMPG:C4QXS9|C4QXS9\_KOMPG:C4R3H4|C4R3H4\_KOMPG:C4QWS9|C4QWS9\_KOMPG:C4QXX3|C4QXX3\_KOMPG:C4R721|C4R721\_KOMPG:C4R6U6|C4R6U6\_KOMPG:C4QXE9|CHO2\_KOMPG |  |  | DB Search |
| IAEV | 19.27 | 430.2427 | 4 | 1.69 | 431.2497 | 13.56 | 5318 | 0 | 0 | 0 | C4QYG4|C4QYG4\_KOMPG:C4QY36|C4QY36\_KOMPG:C4QYI6|C4QYI6\_KOMPG:C4R002|C4R002\_KOMPG:C4QWB0|C4QWB0\_KOMPG:C4R8F2|C4R8F2\_KOMPG:C4R223|C4R223\_KOMPG:C4R436|C4R436\_KOMPG:C4R5T3|C4R5T3\_KOMPG:C4R3M2|C4R3M2\_KOMPG:C4R286|C4R286\_KOMPG:C4R0A2|C4R0A2\_KOMPG:C4R1M0|C4R1M0\_KOMPG:C4R226|C4R226\_KOMPG:C4R431|C4R431\_KOMPG:C4R869|C4R869\_KOMPG:C4R123|C4R123\_KOMPG:C4QX02|C4QX02\_KOMPG:C4QVP3|C4QVP3\_KOMPG:C4R6W9|C4R6W9\_KOMPG:C4QVY5|C4QVY5\_KOMPG:C4R0N8|C4R0N8\_KOMPG:C4QWZ5|C4QWZ5\_KOMPG:C4QV64|C4QV64\_KOMPG:C4QZK6|C4QZK6\_KOMPG:C4R493|C4R493\_KOMPG:C4QX91|C4QX91\_KOMPG:C4R0F7|C4R0F7\_KOMPG:C4R3P1|C4R3P1\_KOMPG |  |  | DB Search |
| LAEV | 19.27 | 430.2427 | 4 | 1.69 | 431.2497 | 13.56 | 5318 | 0 | 0 | 0 | C4QX21|C4QX21\_KOMPG:C4R915|C4R915\_KOMPG:C4R7Q0|C4R7Q0\_KOMPG:C4QV61|C4QV61\_KOMPG:C4R6C0|C4R6C0\_KOMPG:C4R1S9|C4R1S9\_KOMPG:C4R232|C4R232\_KOMPG:C4R6T5|C4R6T5\_KOMPG:C4QZ84|C4QZ84\_KOMPG:C4QWD6|C4QWD6\_KOMPG:C4R0S8|C4R0S8\_KOMPG:C4R669|C4R669\_KOMPG:C4QWX2|C4QWX2\_KOMPG:C4R2Z7|C4R2Z7\_KOMPG:C4R1L6|C4R1L6\_KOMPG:C4R0P7|C4R0P7\_KOMPG:C4R7I3|C4R7I3\_KOMPG:C4R4N9|C4R4N9\_KOMPG:C4QW51|C4QW51\_KOMPG:C4QZ21|C4QZ21\_KOMPG:C4R6G5|C4R6G5\_KOMPG:C4R1T4|C4R1T4\_KOMPG:C4R4Y0|C4R4Y0\_KOMPG:C4R7N1|C4R7N1\_KOMPG:C4R8X0|C4R8X0\_KOMPG:C4R5J2|C4R5J2\_KOMPG:C4R3T0|C4R3T0\_KOMPG:C4R7J5|C4R7J5\_KOMPG:C4R615|C4R615\_KOMPG:C4R7L2|C4R7L2\_KOMPG:C4R6Y3|C4R6Y3\_KOMPG:C4R1Z5|C4R1Z5\_KOMPG:C4R4X3|C4R4X3\_KOMPG:C4QWU2|C4QWU2\_KOMPG:C4QXV8|C4QXV8\_KOMPG:C4R8E9|C4R8E9\_KOMPG:C4R649|C4R649\_KOMPG:C4R5Z8|C4R5Z8\_KOMPG:C4R5X1|C4R5X1\_KOMPG:C4R475|C4R475\_KOMPG:C4R5C8|C4R5C8\_KOMPG |  |  | DB Search |
| TVVI | 19.26 | 430.2791 | 4 | -6.78 | 431.2824 | 27.81 | 9764 | 1.56e3 | 1 | 1 | C4R3A8|C4R3A8\_KOMPG:C4R4I6|C4R4I6\_KOMPG:C4QVT9|C4QVT9\_KOMPG:C4QZU4|C4QZU4\_KOMPG:C4R2V6|C4R2V6\_KOMPG:C4R0S7|C4R0S7\_KOMPG:C4QXN4|C4QXN4\_KOMPG:C4QYG5|C4QYG5\_KOMPG:C4QZD1|C4QZD1\_KOMPG:C4R6H8|C4R6H8\_KOMPG |  |  | DB Search |
| TVVL | 19.26 | 430.2791 | 4 | -6.78 | 431.2824 | 27.81 | 9764 | 1.56e3 | 1 | 1 | C4QX02|C4QX02\_KOMPG:C4QYH7|C4QYH7\_KOMPG:C4R9E6|C4R9E6\_KOMPG:C4QWD1|C4QWD1\_KOMPG:C4QZI6|C4QZI6\_KOMPG:C4QZH9|C4QZH9\_KOMPG:C4QY10|C4QY10\_KOMPG:C4R1A8|C4R1A8\_KOMPG:C4R0W4|C4R0W4\_KOMPG:C4R6P6|C4R6P6\_KOMPG:C4QXE9|CHO2\_KOMPG:C4R7U4|C4R7U4\_KOMPG:C4R697|C4R697\_KOMPG:C4R1Q0|C4R1Q0\_KOMPG |  |  | DB Search |
| E(-18.01)QQVP | 19.25 | 581.2809 | 5 | -4.42 | 582.2842 | 10.75 | 4255 | 1.02e3 | 1 | 1 | C4QXL2|C4QXL2\_KOMPG:C4R892|MMM1\_KOMPG | Pyro-glu from E | E1:Pyro-glu from E:1000 | DB Search |
| EAY | 19.24 | 381.1536 | 3 | -5.14 | 382.158 | 5.09 | 2057 | 2.63e3 | 1 | 1 | C4QVY1|C4QVY1\_KOMPG:C4R585|C4R585\_KOMPG:C4R1B3|C4R1B3\_KOMPG:C4QXP5|C4QXP5\_KOMPG:C4R564|C4R564\_KOMPG:C4R5N2|C4R5N2\_KOMPG:C4R656|C4R656\_KOMPG:C4R5X2|C4R5X2\_KOMPG:C4R588|C4R588\_KOMPG:C4R784|C4R784\_KOMPG:C4R5C3|C4R5C3\_KOMPG:C4R350|C4R350\_KOMPG:C4QVE8|C4QVE8\_KOMPG:C4R6B2|C4R6B2\_KOMPG:C4R6R6|C4R6R6\_KOMPG:C4R2J4|C4R2J4\_KOMPG:C4R5J5|C4R5J5\_KOMPG:C4R768|C4R768\_KOMPG:C4R6G3|C4R6G3\_KOMPG:C4QV50|C4QV50\_KOMPG:C4QV46|C4QV46\_KOMPG:C4R6X6|C4R6X6\_KOMPG:C4R4M6|C4R4M6\_KOMPG:C4R573|C4R573\_KOMPG:C4R1Z5|C4R1Z5\_KOMPG:C4R366|C4R366\_KOMPG:C4QW21|C4QW21\_KOMPG:C4QVP0|C4QVP0\_KOMPG:C4R6W5|C4R6W5\_KOMPG:C4QWJ2|C4QWJ2\_KOMPG:C4R2W9|C4R2W9\_KOMPG:C4R2I4|C4R2I4\_KOMPG:C4QZW5|C4QZW5\_KOMPG:C4QWQ0|C4QWQ0\_KOMPG:C4R688|C4R688\_KOMPG:C4R7N8|C4R7N8\_KOMPG:C4QZ03|C4QZ03\_KOMPG:C4QZ13|C4QZ13\_KOMPG:C4R7V7|C4R7V7\_KOMPG:C4QV54|C4QV54\_KOMPG:C4R6D6|C4R6D6\_KOMPG:C4R162|C4R162\_KOMPG:C4R4F7|C4R4F7\_KOMPG:C4R5H4|C4R5H4\_KOMPG:C4R8G8|C4R8G8\_KOMPG:C4QYV6|C4QYV6\_KOMPG:C4R899|C4R899\_KOMPG:C4R172|C4R172\_KOMPG:C4R0J7|C4R0J7\_KOMPG:C4R1A0|C4R1A0\_KOMPG:C4QVM7|C4QVM7\_KOMPG:C4R8G9|C4R8G9\_KOMPG:C4R7B8|C4R7B8\_KOMPG:C4R446|C4R446\_KOMPG:C4R6X9|C4R6X9\_KOMPG:C4QYE4|C4QYE4\_KOMPG:C4R1Y9|C4R1Y9\_KOMPG:C4QWD1|C4QWD1\_KOMPG:C4R941|GLG\_KOMPG:C4R0Z6|C4R0Z6\_KOMPG:C4R135|C4R135\_KOMPG:C4QZ54|C4QZ54\_KOMPG:C4QZW1|C4QZW1\_KOMPG:C4QXH1|C4QXH1\_KOMPG:C4R3U7|C4R3U7\_KOMPG:C4QYF1|C4QYF1\_KOMPG:C4R101|C4R101\_KOMPG:C4QZM4|C4QZM4\_KOMPG:C4QZ06|BMT3\_KOMPG:C4R430|C4R430\_KOMPG:C4QZT2|C4QZT2\_KOMPG:C4QXA8|C4QXA8\_KOMPG:C4QYS3|C4QYS3\_KOMPG:C4QXX5|C4QXX5\_KOMPG:C4R5P0|C4R5P0\_KOMPG:C4QVS9|C4QVS9\_KOMPG:C4R3N6|C4R3N6\_KOMPG:Q9P4D0|SEC17\_KOMPG:C4QXW6|C4QXW6\_KOMPG:C4R168|C4R168\_KOMPG:C4QX76|C4QX76\_KOMPG:C4R5Z4|C4R5Z4\_KOMPG:C4R1U9|C4R1U9\_KOMPG:C4R2P9|C4R2P9\_KOMPG:C4R6T8|C4R6T8\_KOMPG:C4R5Z7|C4R5Z7\_KOMPG:C4R601|C4R601\_KOMPG:C4R2J3|C4R2J3\_KOMPG:C4QWQ7|C4QWQ7\_KOMPG:C4R069|C4R069\_KOMPG:C4QYT3|C4QYT3\_KOMPG:C4QXJ8|C4QXJ8\_KOMPG:C4R2M9|C4R2M9\_KOMPG:C4QVK0|C4QVK0\_KOMPG:C4R7G6|C4R7G6\_KOMPG:C4QYA4|C4QYA4\_KOMPG:C4QY80|C4QY80\_KOMPG:C4R7X7|C4R7X7\_KOMPG:C4QYV8|C4QYV8\_KOMPG:C4R9D7|C4R9D7\_KOMPG:C4QW72|C4QW72\_KOMPG:C4R1K5|C4R1K5\_KOMPG:C4R2S4|C4R2S4\_KOMPG:C4R802|C4R802\_KOMPG:C4R5C1|C4R5C1\_KOMPG:C4QXG2|C4QXG2\_KOMPG:C4R922|C4R922\_KOMPG:C4R2Q9|C4R2Q9\_KOMPG:C4QWU3|C4QWU3\_KOMPG:C4R099|C4R099\_KOMPG:C4R5P8|C4R5P8\_KOMPG:C4QZ69|C4QZ69\_KOMPG:C4R8D8|C4R8D8\_KOMPG:C4R8I2|C4R8I2\_KOMPG:C4R460|C4R460\_KOMPG:C4R3R5|C4R3R5\_KOMPG:C4R1F7|C4R1F7\_KOMPG:C4R0H6|C4R0H6\_KOMPG:C4QXS3|C4QXS3\_KOMPG:C4QVJ4|C4QVJ4\_KOMPG:C4R5D6|C4R5D6\_KOMPG:C4R3C3|C4R3C3\_KOMPG:C4R134|C4R134\_KOMPG:C4R0X6|C4R0X6\_KOMPG:C4R7I5|C4R7I5\_KOMPG:C4R1S1|C4R1S1\_KOMPG:C4QX02|C4QX02\_KOMPG:C4R5I6|C4R5I6\_KOMPG:C4R4Y0|C4R4Y0\_KOMPG:C4QW68|C4QW68\_KOMPG:C4QWC1|C4QWC1\_KOMPG:C4R931|C4R931\_KOMPG:C4QXV0|C4QXV0\_KOMPG:C4QZB7|C4QZB7\_KOMPG:C4QVQ6|C4QVQ6\_KOMPG:C4QWC7|C4QWC7\_KOMPG:C4QYX7|C4QYX7\_KOMPG:C4R4V1|C4R4V1\_KOMPG:C4R448|C4R448\_KOMPG:C4R7Q9|C4R7Q9\_KOMPG:C4R439|C4R439\_KOMPG:C4R2N6|C4R2N6\_KOMPG:C4R813|C4R813\_KOMPG:C4R0C8|C4R0C8\_KOMPG:C4R9A6|C4R9A6\_KOMPG |  |  | DB Search |
| VDVIA | 19.24 | 515.2955 | 5 | -4.44 | 516.2992 | 15.57 | 6202 | 1.1e3 | 1 | 1 | C4QZU2|C4QZU2\_KOMPG:C4QVX1|C4QVX1\_KOMPG:C4R603|C4R603\_KOMPG |  |  | DB Search |
| Q(-17.03)PLQPQ | 19.23 | 692.3493 | 6 | 2.51 | 693.3566 | 13.59 | 5339 | 1.36e3 | 1 | 1 | C4QYQ4|C4QYQ4\_KOMPG | Pyro-glu from Q | Q1:Pyro-glu from Q:1000 | DB Search |
| ATQQP | 19.21 | 543.2653 | 5 | 1.21 | 544.2719 | 6.86 | 2639 | 0 | 0 | 0 | C4QXL2|C4QXL2\_KOMPG:C4R8C0|C4R8C0\_KOMPG:C4R048|C4R048\_KOMPG |  |  | DB Search |
| NITI | 19.19 | 459.2693 | 4 | -0.54 | 460.2752 | 17.07 | 6712 | 2.02e3 | 1 | 1 | C4R060|C4R060\_KOMPG:C4QYS7|C4QYS7\_KOMPG:C4QX99|C4QX99\_KOMPG:C4R895|C4R895\_KOMPG:C4R0V4|C4R0V4\_KOMPG:C4QVH3|C4QVH3\_KOMPG:C4R523|C4R523\_KOMPG:C4R8H1|C4R8H1\_KOMPG:C4R0Z0|C4R0Z0\_KOMPG:C4QVZ6|C4QVZ6\_KOMPG:C4QZM0|C4QZM0\_KOMPG:C4QYA0|C4QYA0\_KOMPG:C4R5E4|C4R5E4\_KOMPG:C4R5Z5|C4R5Z5\_KOMPG:C4QXD4|C4QXD4\_KOMPG:C4QY13|C4QY13\_KOMPG:C4R350|C4R350\_KOMPG:C4QUZ3|C4QUZ3\_KOMPG:C4R5Q4|C4R5Q4\_KOMPG:C4R6P5|C4R6P5\_KOMPG:C4R2Z9|C4R2Z9\_KOMPG:C4R264|C4R264\_KOMPG:C4R710|C4R710\_KOMPG:C4R3C3|C4R3C3\_KOMPG |  |  | DB Search |
| NITL | 19.19 | 459.2693 | 4 | -0.54 | 460.2752 | 17.07 | 6712 | 2.02e3 | 1 | 1 | C4R1G2|C4R1G2\_KOMPG:C4R2W9|C4R2W9\_KOMPG:C4R3Y4|C4R3Y4\_KOMPG:C4QX19|C4QX19\_KOMPG:C4R5S6|C4R5S6\_KOMPG:C4R626|C4R626\_KOMPG:C4R554|C4R554\_KOMPG:C4QW59|C4QW59\_KOMPG:C4R8N7|C4R8N7\_KOMPG:C4R8Y2|C4R8Y2\_KOMPG:C4R6M7|C4R6M7\_KOMPG:C4QYV4|C4QYV4\_KOMPG:C4QV72|C4QV72\_KOMPG:C4QVC4|C4QVC4\_KOMPG:C4R726|C4R726\_KOMPG:C4R318|C4R318\_KOMPG:C4QYZ1|C4QYZ1\_KOMPG:C4R527|C4R527\_KOMPG:C4R2G6|C4R2G6\_KOMPG:C4QW97|C4QW97\_KOMPG:C4R8X0|C4R8X0\_KOMPG:C4R5M2|C4R5M2\_KOMPG:C4QZS1|C4QZS1\_KOMPG:C4R735|C4R735\_KOMPG:C4R5I5|C4R5I5\_KOMPG:C4QW16|C4QW16\_KOMPG:C4R668|C4R668\_KOMPG:C4R2X6|C4R2X6\_KOMPG:C4QW32|C4QW32\_KOMPG:C4R4X3|C4R4X3\_KOMPG:C4R4E8|C4R4E8\_KOMPG:C4R0L0|C4R0L0\_KOMPG:C4R052|C4R052\_KOMPG:C4R351|C4R351\_KOMPG:C4R0G4|C4R0G4\_KOMPG |  |  | DB Search |
| NLTI | 19.19 | 459.2693 | 4 | -0.54 | 460.2752 | 17.07 | 6712 | 2.02e3 | 1 | 1 | C4R2Y2|C4R2Y2\_KOMPG:C4QZ59|C4QZ59\_KOMPG:C4QVA9|C4QVA9\_KOMPG:C4R4F1|C4R4F1\_KOMPG:C4R440|C4R440\_KOMPG:C4QXH2|AIM24\_KOMPG:C4QYA8|C4QYA8\_KOMPG:C4QZH7|C4QZH7\_KOMPG:C4R3D3|C4R3D3\_KOMPG:C4QZ51|C4QZ51\_KOMPG:C4QZF4|C4QZF4\_KOMPG:C4QV66|C4QV66\_KOMPG:C4QW81|C4QW81\_KOMPG:C4R517|C4R517\_KOMPG:C4R580|C4R580\_KOMPG:C4R293|C4R293\_KOMPG:C4R454|C4R454\_KOMPG:C4QYX1|C4QYX1\_KOMPG:C4QZC2|C4QZC2\_KOMPG:C4R543|C4R543\_KOMPG:C4QWU5|C4QWU5\_KOMPG:C4R0F1|C4R0F1\_KOMPG:C4QV48|C4QV48\_KOMPG:C4QWS3|C4QWS3\_KOMPG:C4R1C3|C4R1C3\_KOMPG:C4R6W9|C4R6W9\_KOMPG:C4R3W8|C4R3W8\_KOMPG:C4QV64|C4QV64\_KOMPG:C4QZ87|C4QZ87\_KOMPG:C4R5Y9|C4R5Y9\_KOMPG:C4R326|C4R326\_KOMPG:C4R6D7|C4R6D7\_KOMPG:C4QVS5|C4QVS5\_KOMPG:Q92448|PFKA1\_KOMPG:C4QYR4|C4QYR4\_KOMPG:C4R1W9|C4R1W9\_KOMPG:C4QYD5|C4QYD5\_KOMPG:C4R8W6|C4R8W6\_KOMPG:C4R818|C4R818\_KOMPG:C4R8E3|C4R8E3\_KOMPG:C4R4T0|C4R4T0\_KOMPG:C4R5Z8|C4R5Z8\_KOMPG |  |  | DB Search |
| NLTL | 19.19 | 459.2693 | 4 | -0.54 | 460.2752 | 17.07 | 6712 | 2.02e3 | 1 | 1 | C4QVG7|C4QVG7\_KOMPG:C4R641|C4R641\_KOMPG:C4QYB2|C4QYB2\_KOMPG:C4QWE6|C4QWE6\_KOMPG:C4QVN4|C4QVN4\_KOMPG:C4R396|C4R396\_KOMPG:C4R4R3|C4R4R3\_KOMPG:C4R0V3|C4R0V3\_KOMPG:C4R083|C4R083\_KOMPG:C4R001|C4R001\_KOMPG:C4R1N5|C4R1N5\_KOMPG:C4QXN0|C4QXN0\_KOMPG:C4R880|C4R880\_KOMPG:C4QWA3|C4QWA3\_KOMPG:C4R540|C4R540\_KOMPG:C4QYD1|C4QYD1\_KOMPG:C4QXZ5|C4QXZ5\_KOMPG:C4R7Y5|C4R7Y5\_KOMPG:C4R5A3|C4R5A3\_KOMPG:C4R244|C4R244\_KOMPG:C4R2E4|C4R2E4\_KOMPG:C4QZQ0|C4QZQ0\_KOMPG:C4R8Q7|C4R8Q7\_KOMPG:C4QVF7|C4QVF7\_KOMPG:C4R456|C4R456\_KOMPG:C4QVC8|C4QVC8\_KOMPG:C4QXC2|C4QXC2\_KOMPG:C4QZ33|C4QZ33\_KOMPG:C4R117|C4R117\_KOMPG:C4QXI3|C4QXI3\_KOMPG:C4QZN8|C4QZN8\_KOMPG:C4R3D7|C4R3D7\_KOMPG:C4R925|C4R925\_KOMPG:C4QVV1|C4QVV1\_KOMPG:C4QUZ7|C4QUZ7\_KOMPG:C4R2H0|C4R2H0\_KOMPG:C4R7Q4|AIM11\_KOMPG:C4R0G8|C4R0G8\_KOMPG:C4R8S6|C4R8S6\_KOMPG:C4R245|C4R245\_KOMPG:C4R7X2|C4R7X2\_KOMPG:C4R1N6|C4R1N6\_KOMPG:C4R0A2|C4R0A2\_KOMPG:C4QZ61|C4QZ61\_KOMPG:C4R2K1|C4R2K1\_KOMPG:C4R2C6|C4R2C6\_KOMPG:C4QXQ7|C4QXQ7\_KOMPG:C4R3C7|C4R3C7\_KOMPG:C4R8C0|C4R8C0\_KOMPG:C4R218|C4R218\_KOMPG:C4R7I4|C4R7I4\_KOMPG:C4QXV5|C4QXV5\_KOMPG:C4QZN3|C4QZN3\_KOMPG:C4R047|C4R047\_KOMPG:C4QYA2|C4QYA2\_KOMPG:C4R8V9|C4R8V9\_KOMPG:C4R5P7|CCM1\_KOMPG:C4R876|C4R876\_KOMPG:C4QVR1|C4QVR1\_KOMPG:C4R8Y1|C4R8Y1\_KOMPG |  |  | DB Search |
| PAFV | 19.17 | 432.2372 | 4 | -3.2 | 433.2421 | 24.76 | 8853 | 3.98e2 | 1 | 1 | C4QYG4|C4QYG4\_KOMPG:C4R932|C4R932\_KOMPG:C4QWN3|C4QWN3\_KOMPG:C4QVK8|C4QVK8\_KOMPG:C4R034|C4R034\_KOMPG:C4QWC3|C4QWC3\_KOMPG:C4R0Z3|C4R0Z3\_KOMPG:C4QWN9|C4QWN9\_KOMPG:C4R1L9|C4R1L9\_KOMPG:C4QZF2|C4QZF2\_KOMPG:C4R2L2|C4R2L2\_KOMPG:C4R801|C4R801\_KOMPG:C4QY90|C4QY90\_KOMPG:C4QX93|C4QX93\_KOMPG:C4QZX4|C4QZX4\_KOMPG:C4R3S1|C4R3S1\_KOMPG:C4R6V3|C4R6V3\_KOMPG |  |  | DB Search |
| STSP | 19.15 | 390.175 | 4 | -0.08 | 391.1813 | 5.92 | 2360 | 3.31e2 | 1 | 1 | C4QYG6|C4QYG6\_KOMPG:C4QV93|C4QV93\_KOMPG:C4QXS5|C4QXS5\_KOMPG:C4R5Z3|C4R5Z3\_KOMPG:C4QZ17|C4QZ17\_KOMPG:C4R5C0|C4R5C0\_KOMPG:C4R811|C4R811\_KOMPG:C4QWB0|C4QWB0\_KOMPG:C4QWU9|C4QWU9\_KOMPG:C4QX30|C4QX30\_KOMPG:C4R5K5|C4R5K5\_KOMPG:C4QXF8|C4QXF8\_KOMPG:C4R3R6|C4R3R6\_KOMPG:C4R888|C4R888\_KOMPG:C4R1C8|C4R1C8\_KOMPG:C4R251|C4R251\_KOMPG:C4R8E1|C4R8E1\_KOMPG:C4QY81|C4QY81\_KOMPG:C4QV21|C4QV21\_KOMPG:C4QWS3|C4QWS3\_KOMPG:C4QWK1|C4QWK1\_KOMPG:C4R8M0|C4R8M0\_KOMPG:C4QYB7|C4QYB7\_KOMPG:C4R4R6|C4R4R6\_KOMPG:C4R679|C4R679\_KOMPG:C4QV25|C4QV25\_KOMPG:C4R6W9|C4R6W9\_KOMPG:C4R5R4|C4R5R4\_KOMPG:C4R5R5|C4R5R5\_KOMPG:C4R8V8|C4R8V8\_KOMPG:C4QXM2|C4QXM2\_KOMPG:C4QXZ3|C4QXZ3\_KOMPG:C4QZP8|C4QZP8\_KOMPG:C4QWL2|C4QWL2\_KOMPG:C4R238|C4R238\_KOMPG:C4R1J7|C4R1J7\_KOMPG:C4R159|ATG28\_KOMPG:C4QWH1|C4QWH1\_KOMPG:C4R4Q9|C4R4Q9\_KOMPG:C4R898|C4R898\_KOMPG:C4R4A2|C4R4A2\_KOMPG:C4QZX1|C4QZX1\_KOMPG:C4R0D7|C4R0D7\_KOMPG:C4R3T6|C4R3T6\_KOMPG:C4R1L5|C4R1L5\_KOMPG:C4R275|C4R275\_KOMPG:C4QZB5|C4QZB5\_KOMPG:C4R062|C4R062\_KOMPG:C4QVW8|C4QVW8\_KOMPG:C4R3W5|C4R3W5\_KOMPG:C4QZM2|C4QZM2\_KOMPG:C4QWT0|C4QWT0\_KOMPG:C4QYE8|C4QYE8\_KOMPG:C4R7P7|C4R7P7\_KOMPG:C4R981|C4R981\_KOMPG:C4R3G5|C4R3G5\_KOMPG:C4QZB4|C4QZB4\_KOMPG:C4QYR4|C4QYR4\_KOMPG:C4QZP4|C4QZP4\_KOMPG |  |  | DB Search |
| VTAT | 19.15 | 390.2114 | 4 | -9 | 391.2142 | 7.51 | 2842 | 9.19e2 | 1 | 1 | C4R802|C4R802\_KOMPG:C4QXJ1|C4QXJ1\_KOMPG:C4R9E7|C4R9E7\_KOMPG:C4R4H2|C4R4H2\_KOMPG:C4QV87|C4QV87\_KOMPG:C4R1N0|C4R1N0\_KOMPG:C4QVS9|C4QVS9\_KOMPG:C4QXH4|C4QXH4\_KOMPG:C4QY71|C4QY71\_KOMPG:C4QYG3|C4QYG3\_KOMPG:C4R9F6|C4R9F6\_KOMPG:C4R2C6|C4R2C6\_KOMPG:C4R691|C4R691\_KOMPG:C4QVE0|C4QVE0\_KOMPG:C4QVT9|C4QVT9\_KOMPG:C4R4J6|C4R4J6\_KOMPG:C4R043|C4R043\_KOMPG:C4R3H4|C4R3H4\_KOMPG:C4QYT8|C4QYT8\_KOMPG:C4R701|C4R701\_KOMPG:C4R409|C4R409\_KOMPG:C4R3C4|C4R3C4\_KOMPG:C4R8K5|C4R8K5\_KOMPG:C4R8E9|C4R8E9\_KOMPG:C4QYY9|C4QYY9\_KOMPG |  |  | DB Search |
| IIQP | 19.13 | 469.29 | 4 | -8.42 | 470.2922 | 12.16 | 4756 | 6.43e3 | 1 | 1 | C4QWJ1|C4QWJ1\_KOMPG:C4R488|C4R488\_KOMPG:C4R2C1|C4R2C1\_KOMPG:C4QZF4|C4QZF4\_KOMPG:C4R123|C4R123\_KOMPG:C4R8Q7|C4R8Q7\_KOMPG:C4R4Q7|C4R4Q7\_KOMPG:C4R955|C4R955\_KOMPG:C4R1J5|C4R1J5\_KOMPG:C4R772|C4R772\_KOMPG:C4R8I0|C4R8I0\_KOMPG |  |  | DB Search |
| ILQP | 19.13 | 469.29 | 4 | -8.42 | 470.2922 | 12.16 | 4756 | 6.43e3 | 1 | 1 | C4R5P9|C4R5P9\_KOMPG:C4QVK8|C4QVK8\_KOMPG:C4QXD1|C4QXD1\_KOMPG:C4R6K9|C4R6K9\_KOMPG:C4QZ90|C4QZ90\_KOMPG:C4R6J6|C4R6J6\_KOMPG:C4R7Q8|C4R7Q8\_KOMPG:C4R2G7|C4R2G7\_KOMPG:C4R6F6|C4R6F6\_KOMPG:C4R630|C4R630\_KOMPG:C4QYZ4|C4QYZ4\_KOMPG:C4QWH1|C4QWH1\_KOMPG:C4QY56|C4QY56\_KOMPG:C4R3L1|C4R3L1\_KOMPG:C4QYE7|C4QYE7\_KOMPG:C4R5V0|C4R5V0\_KOMPG:C4R2K1|C4R2K1\_KOMPG:C4R726|C4R726\_KOMPG:C4R322|C4R322\_KOMPG:C4R453|C4R453\_KOMPG:C4QXZ3|C4QXZ3\_KOMPG |  |  | DB Search |
| LIQP | 19.13 | 469.29 | 4 | -8.42 | 470.2922 | 12.16 | 4756 | 6.43e3 | 1 | 1 | C4R2M0|C4R2M0\_KOMPG:C4R1D2|C4R1D2\_KOMPG:C4R5C7|C4R5C7\_KOMPG:C4QVH3|C4QVH3\_KOMPG:C4R141|C4R141\_KOMPG:C4R059|C4R059\_KOMPG:C4QY40|C4QY40\_KOMPG:C4R5Z1|C4R5Z1\_KOMPG:C4R0P8|C4R0P8\_KOMPG:C4R2L2|C4R2L2\_KOMPG:C4R6L5|C4R6L5\_KOMPG:C4QVQ7|C4QVQ7\_KOMPG:C4QVC2|C4QVC2\_KOMPG:C4QX93|C4QX93\_KOMPG:C4QVH9|C4QVH9\_KOMPG:C4R881|C4R881\_KOMPG:C4QX02|C4QX02\_KOMPG:C4R6V2|C4R6V2\_KOMPG:C4R9B2|C4R9B2\_KOMPG:C4R1Y9|C4R1Y9\_KOMPG:C4QVZ1|C4QVZ1\_KOMPG:C4R5B1|C4R5B1\_KOMPG:C4QXX3|C4QXX3\_KOMPG:C4R0W2|C4R0W2\_KOMPG:C4R8V4|C4R8V4\_KOMPG:C4QYX0|C4QYX0\_KOMPG |  |  | DB Search |
| LLQP | 19.13 | 469.29 | 4 | -8.42 | 470.2922 | 12.16 | 4756 | 6.43e3 | 1 | 1 | C4R2F0|C4R2F0\_KOMPG:C4R2H1|C4R2H1\_KOMPG:C4R0L9|C4R0L9\_KOMPG:C4R925|C4R925\_KOMPG:C4R6X0|C4R6X0\_KOMPG:C4QX99|C4QX99\_KOMPG:C4R919|C4R919\_KOMPG:C4R5U2|C4R5U2\_KOMPG:C4R214|C4R214\_KOMPG:C4R554|C4R554\_KOMPG:C4R232|C4R232\_KOMPG:C4R9C8|C4R9C8\_KOMPG:C4R432|SEY1\_KOMPG:C4R271|C4R271\_KOMPG:C4R5Q5|C4R5Q5\_KOMPG:C4R4P6|C4R4P6\_KOMPG:C4QWN5|C4QWN5\_KOMPG:C4R846|C4R846\_KOMPG:C4R1M0|C4R1M0\_KOMPG:C4QYH0|C4QYH0\_KOMPG:C4R051|C4R051\_KOMPG:C4QZ60|C4QZ60\_KOMPG:C4R0F1|C4R0F1\_KOMPG:C4R0G9|C4R0G9\_KOMPG:C4R6H3|LIS1\_KOMPG:C4QVG3|C4QVG3\_KOMPG:C4R3C1|C4R3C1\_KOMPG:C4R9A9|C4R9A9\_KOMPG:C4R734|C4R734\_KOMPG:C4QVD7|C4QVD7\_KOMPG:C4R2P4|C4R2P4\_KOMPG:C4QZW1|C4QZW1\_KOMPG:C4R1I5|C4R1I5\_KOMPG:C4R1C7|C4R1C7\_KOMPG:C4R0J8|C4R0J8\_KOMPG:C4R7I8|C4R7I8\_KOMPG:C4QYF9|C4QYF9\_KOMPG:C4R111|C4R111\_KOMPG:C4QZS0|C4QZS0\_KOMPG:C4R0J3|C4R0J3\_KOMPG:C4R0E1|C4R0E1\_KOMPG:C4R4K4|C4R4K4\_KOMPG:C4R475|C4R475\_KOMPG |  |  | DB Search |
| EM | 19.13 | 278.0936 | 2 | 0.1 | 279.1003 | 3.52 | 1504 | 7.14e3 | 1 | 1 | C4QVY1|C4QVY1\_KOMPG:C4R8H4|C4R8H4\_KOMPG:C4R8N1|C4R8N1\_KOMPG:C4R142|C4R142\_KOMPG:C4QZ28|C4QZ28\_KOMPG:C4QV84|C4QV84\_KOMPG:C4QZZ8|C4QZZ8\_KOMPG:C4R2Z6|C4R2Z6\_KOMPG:C4R2A4|C4R2A4\_KOMPG:C4QW93|C4QW93\_KOMPG:C4R4G9|OXDA\_KOMPG:C4QVX4|C4QVX4\_KOMPG:C4QZX7|C4QZX7\_KOMPG:C4R870|C4R870\_KOMPG:C4R5Q7|C4R5Q7\_KOMPG:C4QX28|C4QX28\_KOMPG:C4R042|C4R042\_KOMPG:C4R7Y1|C4R7Y1\_KOMPG:C4QWM2|C4QWM2\_KOMPG:C4R6E1|C4R6E1\_KOMPG:C4QWS7|C4QWS7\_KOMPG:C4R849|C4R849\_KOMPG:C4R8D3|C4R8D3\_KOMPG:C4R8I1|C4R8I1\_KOMPG:C4R8T8|C4R8T8\_KOMPG:C4QYV2|C4QYV2\_KOMPG:C4QV97|C4QV97\_KOMPG:C4QWJ2|C4QWJ2\_KOMPG:C4QWR3|C4QWR3\_KOMPG:C4QXD2|C4QXD2\_KOMPG:C4QZ47|C4QZ47\_KOMPG:C4QVV6|C4QVV6\_KOMPG:C4R0B6|C4R0B6\_KOMPG:C4R2Y2|C4R2Y2\_KOMPG:C4R688|C4R688\_KOMPG:C4R7N8|C4R7N8\_KOMPG:C4QX78|C4QX78\_KOMPG:C4R0X4|C4R0X4\_KOMPG:C4R1H3|C4R1H3\_KOMPG:C4R4F7|C4R4F7\_KOMPG:C4QZL9|C4QZL9\_KOMPG:C4QVM1|C4QVM1\_KOMPG:C4R2D3|C4R2D3\_KOMPG:C4R093|C4R093\_KOMPG:C4R4V7|C4R4V7\_KOMPG:C4QVT9|C4QVT9\_KOMPG:C4R8J5|C4R8J5\_KOMPG:C4R7R8|C4R7R8\_KOMPG:C4R7C8|C4R7C8\_KOMPG:C4R2G2|C4R2G2\_KOMPG:C4R332|C4R332\_KOMPG:C4QWU2|C4QWU2\_KOMPG:C4R3H2|C4R3H2\_KOMPG:C4QZT6|C4QZT6\_KOMPG:C4R8D9|C4R8D9\_KOMPG:C4QXT2|C4QXT2\_KOMPG:C4R413|C4R413\_KOMPG:C4QVX8|C4QVX8\_KOMPG:C4R1N5|C4R1N5\_KOMPG:C4QWA3|C4QWA3\_KOMPG:C4QWS8|PEX36\_KOMPG:C4R6L7|C4R6L7\_KOMPG:C4R1C0|C4R1C0\_KOMPG:C4QVA0|C4QVA0\_KOMPG:C4QZ39|C4QZ39\_KOMPG:C4R7W9|C4R7W9\_KOMPG:C4R3T0|C4R3T0\_KOMPG:C4R639|C4R639\_KOMPG:C4R349|C4R349\_KOMPG:C4QXB6|C4QXB6\_KOMPG:C4R5K6|C4R5K6\_KOMPG:C4QW00|C4QW00\_KOMPG:C4R033|C4R033\_KOMPG:C4R5C1|C4R5C1\_KOMPG:C4R9E7|C4R9E7\_KOMPG:C4QXG2|C4QXG2\_KOMPG:C4QXT4|C4QXT4\_KOMPG:C4R904|C4R904\_KOMPG:C4QW84|C4QW84\_KOMPG:C4QV73|C4QV73\_KOMPG:C4R5P8|C4R5P8\_KOMPG:C4R6F8|C4R6F8\_KOMPG:C4R848|C4R848\_KOMPG:C4R6H9|C4R6H9\_KOMPG:C4R5D6|C4R5D6\_KOMPG:C4R0R8|C4R0R8\_KOMPG:C4R0Q0|C4R0Q0\_KOMPG:C4R1W8|C4R1W8\_KOMPG:C4QWC1|C4QWC1\_KOMPG:C4QVB5|C4QVB5\_KOMPG:C4R8B0|C4R8B0\_KOMPG:C4R0J5|C4R0J5\_KOMPG:C4R2Q0|C4R2Q0\_KOMPG:C4R565|C4R565\_KOMPG:C4R5V4|C4R5V4\_KOMPG:C4R5H2|C4R5H2\_KOMPG:C4QXZ9|C4QXZ9\_KOMPG |  |  | DB Search |
| QGMVTQ | 19.12 | 662.3058 | 6 | 7.94 | 663.3167 | 8.42 | 3243 | 2.47e2 | 1 | 1 | C4R783|C4R783\_KOMPG |  |  | DB Search |
| ITGPF | 19.12 | 533.2849 | 5 | -3.52 | 534.289 | 28.06 | 9841 | 0 | 0 | 0 | C4R5L7|C4R5L7\_KOMPG |  |  | DB Search |
| AFR | 19.1 | 392.2172 | 3 | -7.11 | 393.2207 | 6.33 | 2475 | 0 | 0 | 0 | C4QXH6|C4QXH6\_KOMPG:C4R208|C4R208\_KOMPG:C4R5Y0|C4R5Y0\_KOMPG:C4R0Z1|C4R0Z1\_KOMPG:C4QYR2|C4QYR2\_KOMPG:C4R5L8|C4R5L8\_KOMPG:C4R920|C4R920\_KOMPG:C4R3G3|C4R3G3\_KOMPG:C4R7R9|C4R7R9\_KOMPG:C4R676|C4R676\_KOMPG:C4R8N3|C4R8N3\_KOMPG:C4QVK9|C4QVK9\_KOMPG:C4QXU4|C4QXU4\_KOMPG:C4QY73|C4QY73\_KOMPG:C4R416|C4R416\_KOMPG:C4R348|C4R348\_KOMPG:C4R968|C4R968\_KOMPG:C4R499|C4R499\_KOMPG:C4R2M1|C4R2M1\_KOMPG:C4R5J3|C4R5J3\_KOMPG:C4R3S6|C4R3S6\_KOMPG:C4R974|C4R974\_KOMPG:C4QY37|C4QY37\_KOMPG:C4QVF9|C4QVF9\_KOMPG:C4R8T6|C4R8T6\_KOMPG:C4QVF7|C4QVF7\_KOMPG:C4R4C9|C4R4C9\_KOMPG:C4QZS2|C4QZS2\_KOMPG:C4R342|C4R342\_KOMPG:C4QW45|C4QW45\_KOMPG:C4QUZ7|C4QUZ7\_KOMPG:C4R8D6|C4R8D6\_KOMPG:C4R5U8|C4R5U8\_KOMPG:C4R091|C4R091\_KOMPG:C4QWD9|C4QWD9\_KOMPG:C4QVH3|C4QVH3\_KOMPG:C4R706|C4R706\_KOMPG:C4R2C2|C4R2C2\_KOMPG:C4R1L9|C4R1L9\_KOMPG:C4QW64|C4QW64\_KOMPG:C4QYB3|C4QYB3\_KOMPG:C4R2L3|C4R2L3\_KOMPG:C4R6L6|C4R6L6\_KOMPG:C4R506|C4R506\_KOMPG:C4R7A7|C4R7A7\_KOMPG:C4R6D1|C4R6D1\_KOMPG:C4R685|C4R685\_KOMPG:C4R8G8|C4R8G8\_KOMPG:C4R2C6|C4R2C6\_KOMPG:C4R939|C4R939\_KOMPG:C4R7V6|C4R7V6\_KOMPG:C4R8Z2|C4R8Z2\_KOMPG:C4QWD1|C4QWD1\_KOMPG:C4R3L2|C4R3L2\_KOMPG:C4R5E5|C4R5E5\_KOMPG:C4R1X2|C4R1X2\_KOMPG:C4R5R3|C4R5R3\_KOMPG:C4QYT9|C4QYT9\_KOMPG:C4R662|C4R662\_KOMPG:C4R5H6|C4R5H6\_KOMPG:C4R3I7|C4R3I7\_KOMPG:C4R1V3|C4R1V3\_KOMPG:C4R2A8|C4R2A8\_KOMPG:C4R9D8|C4R9D8\_KOMPG:C4QWK1|C4QWK1\_KOMPG:C4R3N3|C4R3N3\_KOMPG:C4R192|VPS10\_KOMPG:C4R2X4|C4R2X4\_KOMPG:C4R244|C4R244\_KOMPG:C4QZR8|C4QZR8\_KOMPG:C4QVF5|C4QVF5\_KOMPG:C4R0P1|C4R0P1\_KOMPG:C4R6U9|C4R6U9\_KOMPG:C4R525|C4R525\_KOMPG:C4QWZ8|C4QWZ8\_KOMPG:C4R758|C4R758\_KOMPG:C4QVH4|C4QVH4\_KOMPG:C4R760|C4R760\_KOMPG:C4R802|C4R802\_KOMPG:C4R1Y5|C4R1Y5\_KOMPG:C4R166|C4R166\_KOMPG:C4QUZ4|C4QUZ4\_KOMPG:C4R704|C4R704\_KOMPG:C4QV92|C4QV92\_KOMPG:C4R411|C4R411\_KOMPG:C4R099|C4R099\_KOMPG:C4QV30|C4QV30\_KOMPG:C4R0Q9|C4R0Q9\_KOMPG:C4R2U0|C4R2U0\_KOMPG:C4R245|C4R245\_KOMPG:C4QWL7|C4QWL7\_KOMPG:C4R6R4|C4R6R4\_KOMPG:C4R3B7|C4R3B7\_KOMPG:C4R7V8|C4R7V8\_KOMPG:C4R613|DEGS\_KOMPG:C4R8H5|C4R8H5\_KOMPG:C4R7I4|C4R7I4\_KOMPG:C4QYX5|C4QYX5\_KOMPG:C4R7H4|PFKA3\_KOMPG:C4QV64|C4QV64\_KOMPG:C4QZ20|FEN1\_KOMPG:C4R565|C4R565\_KOMPG:C4R3F9|C4R3F9\_KOMPG:C4R813|C4R813\_KOMPG:C4R2B2|C4R2B2\_KOMPG:C4QYR5|C4QYR5\_KOMPG:C4QVM6|C4QVM6\_KOMPG:C4R1N7|C4R1N7\_KOMPG:C4QWG7|C4QWG7\_KOMPG:C4R5A9|C4R5A9\_KOMPG:C4R4L2|C4R4L2\_KOMPG:C4R3K3|C4R3K3\_KOMPG:C4R072|C4R072\_KOMPG:C4R9B5|C4R9B5\_KOMPG:C4QX30|C4QX30\_KOMPG:C4R5J1|C4R5J1\_KOMPG:C4R558|C4R558\_KOMPG:C4R769|C4R769\_KOMPG:C4R399|C4R399\_KOMPG:C4R2Z6|C4R2Z6\_KOMPG:C4QVJ7|C4QVJ7\_KOMPG:C4R6Z7|C4R6Z7\_KOMPG:C4QWY1|C4QWY1\_KOMPG:C4R2J1|C4R2J1\_KOMPG:C4R267|C4R267\_KOMPG:C4R6K4|C4R6K4\_KOMPG:C4R1N1|C4R1N1\_KOMPG:C4R337|C4R337\_KOMPG:C4R842|C4R842\_KOMPG:C4R6B3|C4R6B3\_KOMPG:C4QZA9|C4QZA9\_KOMPG:C4QWW3|C4QWW3\_KOMPG:C4QZP7|C4QZP7\_KOMPG:C4R867|C4R867\_KOMPG:C4R794|C4R794\_KOMPG:C4R0R1|C4R0R1\_KOMPG:C4QW55|C4QW55\_KOMPG:C4R4G1|C4R4G1\_KOMPG:C4R1U8|C4R1U8\_KOMPG:C4R0L7|C4R0L7\_KOMPG:C4R680|C4R680\_KOMPG:C4R241|C4R241\_KOMPG:C4R8K2|C4R8K2\_KOMPG:C4R165|C4R165\_KOMPG:C4QZ26|C4QZ26\_KOMPG:C4R243|C4R243\_KOMPG:C4R7M5|C4R7M5\_KOMPG:C4R1H7|C4R1H7\_KOMPG:C4R1F0|C4R1F0\_KOMPG:C4R6X3|C4R6X3\_KOMPG:C4R2Q6|C4R2Q6\_KOMPG:C4R092|C4R092\_KOMPG:C4R1X8|C4R1X8\_KOMPG:C4R1A3|C4R1A3\_KOMPG:C4QV23|C4QV23\_KOMPG:C4R071|C4R071\_KOMPG:C4QWI8|C4QWI8\_KOMPG:C4R5C0|C4R5C0\_KOMPG:C4R6J1|C4R6J1\_KOMPG:C4R522|C4R522\_KOMPG:C4R7R2|C4R7R2\_KOMPG:C4R2K6|C4R2K6\_KOMPG:C4R594|C4R594\_KOMPG:C4R5F0|C4R5F0\_KOMPG:C4R6J9|C4R6J9\_KOMPG:C4QXP8|C4QXP8\_KOMPG:C4R7I6|C4R7I6\_KOMPG:C4R787|C4R787\_KOMPG:C4R1V4|C4R1V4\_KOMPG:C4QX55|C4QX55\_KOMPG:C4R751|C4R751\_KOMPG:C4R146|C4R146\_KOMPG:C4QVN2|C4QVN2\_KOMPG:C4R5J0|C4R5J0\_KOMPG:C4R3C5|C4R3C5\_KOMPG:C4R115|C4R115\_KOMPG:C4R6H6|C4R6H6\_KOMPG:C4R3M6|C4R3M6\_KOMPG:C4QX47|C4QX47\_KOMPG:C4QZQ7|C4QZQ7\_KOMPG:C4QZU2|C4QZU2\_KOMPG:C4R3A0|C4R3A0\_KOMPG:C4R526|C4R526\_KOMPG:C4QWN4|C4QWN4\_KOMPG:C4R5N5|C4R5N5\_KOMPG:C4QY17|DRE2\_KOMPG:C4QYH1|C4QYH1\_KOMPG:C4R8S4|C4R8S4\_KOMPG:C4QZV5|C4QZV5\_KOMPG:C4QXI7|C4QXI7\_KOMPG:C4R904|C4R904\_KOMPG:C4R774|C4R774\_KOMPG:C4R027|C4R027\_KOMPG:C4QZU0|C4QZU0\_KOMPG:C4R1F2|C4R1F2\_KOMPG:C4R5I6|C4R5I6\_KOMPG:C4R1W8|C4R1W8\_KOMPG:C4R1T7|C4R1T7\_KOMPG:C4R1G1|C4R1G1\_KOMPG:C4R3L4|C4R3L4\_KOMPG:C4QY03|C4QY03\_KOMPG:C4QVT1|C4QVT1\_KOMPG:C4QY16|C4QY16\_KOMPG:C4R981|C4R981\_KOMPG:C4QVA8|C4QVA8\_KOMPG:C4QY64|C4QY64\_KOMPG:C4R483|C4R483\_KOMPG:C4R369|C4R369\_KOMPG:C4QZ82|C4QZ82\_KOMPG:C4QYY9|C4QYY9\_KOMPG:C4R387|C4R387\_KOMPG |  |  | DB Search |
| NWH | 19.1 | 455.1917 | 3 | -2.83 | 456.1966 | 3.35 | 1412 | 5.2e3 | 1 | 1 | C4QVG7|C4QVG7\_KOMPG:C4R8U2|C4R8U2\_KOMPG:C4R7K5|C4R7K5\_KOMPG:C4R584|C4R584\_KOMPG:C4R0Q2|C4R0Q2\_KOMPG:C4QZ79|C4QZ79\_KOMPG:C4QZJ9|C4QZJ9\_KOMPG:C4R8D0|C4R8D0\_KOMPG:C4R1R3|C4R1R3\_KOMPG:C4R7B4|C4R7B4\_KOMPG:C4R4G1|C4R4G1\_KOMPG:C4QV29|C4QV29\_KOMPG:C4R5Z0|C4R5Z0\_KOMPG:C4R8L1|C4R8L1\_KOMPG:C4R7P0|C4R7P0\_KOMPG:C4QVC7|C4QVC7\_KOMPG:C4R8W8|C4R8W8\_KOMPG:C4QWK2|C4QWK2\_KOMPG:C4QXK9|C4QXK9\_KOMPG:C4R6V2|C4R6V2\_KOMPG:C4R720|C4R720\_KOMPG:C4R3R2|C4R3R2\_KOMPG:C4R1Y9|C4R1Y9\_KOMPG:C4QXC8|C4QXC8\_KOMPG:C4QWJ8|C4QWJ8\_KOMPG:C4R0T3|C4R0T3\_KOMPG:C4R7J2|C4R7J2\_KOMPG:C4QZF1|C4QZF1\_KOMPG:C4R449|C4R449\_KOMPG:C4R219|C4R219\_KOMPG:C4R0S4|C4R0S4\_KOMPG:C4QWY1|C4QWY1\_KOMPG:C4R462|C4R462\_KOMPG:C4R6Y8|C4R6Y8\_KOMPG:C4R052|C4R052\_KOMPG |  |  | DB Search |
| VSGID | 19.1 | 489.2435 | 5 | 1.86 | 490.2504 | 9.58 | 3726 | 0 | 0 | 0 | C4R2Z1|C4R2Z1\_KOMPG:C4QXV9|C4QXV9\_KOMPG |  |  | DB Search |
| VSGLD | 19.1 | 489.2435 | 5 | 1.86 | 490.2504 | 9.58 | 3726 | 0 | 0 | 0 | C4R7I5|C4R7I5\_KOMPG:C4R4V7|C4R4V7\_KOMPG:C4QV88|C4QV88\_KOMPG |  |  | DB Search |
[truncated: 466,123 more chars]
